# ChatGPT in the diagnosis and management of complex polyneuropathies: comparative analysis with neurologists using real-world cases

**DOI:** 10.1038/s41746-026-02815-y

**Published:** 2026-06-05

**Authors:** Alberto De Lorenzo, Giulia Sofia Moretti, Alessandro Bertini, Alice De Lorenzo, Chiara Pisciotta, Roger Collet-Vidiella, Filip Eftimov, Yuri Matteo Falzone, Alessandra Fasolino, Etienne Fortanier, Thierry Gendre, Francesco Gentile, Robert D. Hadden, Badrul Islam, Kleopas Kleopa, Luciana Leon Cejas, Luca Leonardi, Daniele Mandia, Marion Masingue, Valentin Mira, Stojan Peric, Luis Querol, Yusuf A. Rajabally, Teresa Sevilla, Pedro José Tomaselli, Stefano Tozza, Maria Bosca, Matthijs Brouwer, Antoniangela Cocco, Louis Cousyn, Tiziana De Santis, Fabio Gonzalez, Milica Jecmenica Lukic, Niraj Mistry, Alessio Maranzano, Emanuele Morena, Frederico Nakane Nakano, Gonzalo Olmedo-Saura, Elia Sechi, Nicola Ticozzi, Serena Troisi, Tasos Tsokkos, Aymeric Wittwer, Elisa Bianchi, Eduardo Nobile-Orazio, Davide Pareyson, Pietro Emiliano Doneddu

**Affiliations:** 1 Neuromuscular and Neuroimmunology Unit, IRCCS Humanitas Research Hospital, Milan, Rozzano Italy; 2 University of Milan, Milan, Italy; 3 Unit of Rare Neurological Diseases, Department of Clinical Neurosciences, Fondazione IRCCS Istituto Neurologico Carlo Besta, Milan, Italy; 4 Neuromuscular Diseases Unit, Department of Neurology, IR SANT PAU, Hospital de la Santa Creu i Sant Pau, Barcelona, Spain; 5 Department of Neurology, Amsterdam UMC, University of Amsterdam, Amsterdam, the Netherlands; 6 Division of Neuroscience, Department of Neurology, Institute of Experimental Neurology, San Raffaele Scientific Institute, Milan, Italy; 7 UOC Neurophysiopathology, AORN Cardarelli, Naples, Italy; 8 Referral Centre for Neuromuscular Diseases and ALS, Hospital La Timone, Aix-Marseille University, Marseille, France; 9 Neurology Department, Henri Mondor University Hospital APHP, Créteil, France; 10 AP-HP, Reference Center for Neuromuscular Diseases Nord/Est/Ile-de-France, Paris, France; 11 Biology of Myelin Unit, Division of Genetics and Cell Biology, IRCCS San Raffaele Scientific Institute, Milan, Italy; 12 Neurology Department, King’s College Hospital, London, UK; 13 Department of Basic & Clinical Neuroscience, Institute of Psychiatry, Psychology & Neuroscience, King’s College London, London, UK; 14 Department of Neurology & Neurophysiology, KPJ Specialized Hospital, Dhaka, Bangladesh; 15 Neuroscience Department, The Cyprus Institute of Neurology and Genetics, Nicosia, Cyprus; 16 Center for Neuromuscular Disorders, The Cyprus Institute of Neurology and Genetics, Nicosia, Cyprus; 17 Department of Neurology, Hospital Británico de Buenos Aires, Ciudad Autónoma de Buenos Aires, Argentina; 18 Neuromuscular and Rare Disease Centre, Neurology Unit, Sant’Andrea Hospital, Rome, Italy; 19 Neuro-Metabolism Unit, Reference Center for Lysosomal Diseases, Neurology Department, Hôpital Pitié-Salpêtrière, Paris, France; 20 Reference Center for Neuromuscular Disorders (Nord/Est/Ile de France), Pitié Salpêtrière Hospital, Assistance Publique des Hôpitaux de Paris, Paris, France; 21 Department of Neurology and Pathology of Movement, Timone Hospital, AP-HM, Marseille, France; 22 University Clinical Centre of Serbia – Neurology Clinic, University of Belgrade – Faculty of Medicine, Belgrade, Serbia; 23 Neuromuscular Diseases Unit, Department of Neurology, IR SANT PAU, Hospital de la Santa Creu i Sant Pau, CIBERER, Barcelona, Spain; 24 Aston Medical School, Aston University, Birmingham, UK; 25 Neurology Department, Hospital Universitari i Politècnic La Fe & IIS La Fe, Valencia, Spain; 26 Centro de Investigación Biomédica en Red de Enfermedades Raras (CIBERER), Madrid, Spain; 27 Neurology Department, Universitat de València, Valencia, Spain; 28 Department of Neurosciences and Behavioral Sciences, Ribeirão Preto Medical School, University of São Paulo, Ribeirão Preto, Brazil; 29 Department of Neuroscience and Reproductive and Odontostomatological Sciences, University Federico II of Naples, Naples, Italy; 30 Hospital Clínico Universitario de Valencia, Valencia, Spain; 31 Department of Neurology, IRCCS Humanitas Research Hospital, Milan, Italy; 32 Epilepsy Unit, Pitié-Salpêtrière Hospital and Paris Brain Institute, Assistance Publique Hopitaux de Paris, Sorbonne University, Paris, France; 33 Department of Neurology, Hospital Británico de Buenos Aires, Buenos Aires, Argentina; 34 Department of Clinical Neurosciences, University Hospitals Birmingham NHS Foundation Trust, Birmingham, UK; 35 Department of Neurology and Laboratory of Neuroscience, IRCCS Istituto Auxologico Italiano, Milano, Italy; 36 Unità Di Trattamento Neurovascolare, Ospedale dei Castelli, Rome, Italy; 37 Epilepsy Surgery Center (CIREP), Department of Neurosciences and Behavioral Sciences, University Hospital of the Ribeirão Preto Medical School (HCFMRP), University of São Paulo, Ribeirão Preto, Brazil; 38 Movement disorders Unit, Department of Neurology, Hospital de La Santa Creu I Sant Pau, Universitat Autonoma de Barcelona; Biomedical Research Institute Sant Paul, Barcelona, Spain; 39 Neurology Unit, Department of Medical, Surgical and Experimental Sciences, University Hospital of Sassari, Sassari, Italy; 40 Department of Pathophysiology and Transplantation, “Dino Ferrari” Center, Università degli Studi di Milano, Milano, Italy; 41 Pediatric Neurology, Department of Neuroscience, Santobono-Pausilipon Children’s Hospital, Naples, Italy; 42 Département de Neurologie, AP-HP, Hôpital Henri Mondor, Créteil, France; 43 Stroke Unit, AP-HP, Milano, Italy; 44 Department of Neurosciences, Istituto Di Ricerche Farmacologiche Mario Negri IRCCS, Milan, Italy; 45 Department of Medical Biotechnology and Translational Medicine, Milan University, Milano, Italy; 46 Department of Biomedical Sciences, Humanitas University, Milan, Italy

**Keywords:** Peripheral neuropathies, Medical research

## Abstract

Polyneuropathies are common and often require specialist expertise for accurate diagnosis. This study evaluated the diagnostic performance of ChatGPT-4o on real-world polyneuropathy cases, comparing it to peripheral neuropathy specialists and non-specialist neurologists. One hundred cases were selected from two tertiary centers in Milan, Italy. Standardized summaries included clinical, laboratory, and electrophysiological data. ChatGPT-4o was prompted to provide a leading diagnosis, two differentials, and a confirmatory test. Neurologists reviewed the same cases and generated comparable outputs, then could revise their responses after viewing ChatGPT-4o’s suggestions. ChatGPT-4o achieved 65.5% leading diagnosis accuracy, comparable to non-specialists (63.0%) but lower than specialists (74.0%, *p* = 0.002). For differential diagnoses, it outperformed non-specialists (82.0% vs. 77.5%, *p* = 0.043) and recommended more appropriate tests (68.0% vs. 53.0%, *p* < 0.001). After reviewing ChatGPT-4o outputs, non-specialists revised their assessments in 21.8% of cases, improving accuracy. ChatGPT-4o shows potential as a diagnostic aid, particularly in non-specialist or resource-limited settings.

## Introduction

Polyneuropathies constitute a heterogeneous group of disorders affecting approximately 1% of the general population, with prevalence rates in Western countries increasing to nearly 4% among middle-aged individuals and up to 13% among the elderly^1,2^. General practitioners, neurologists, and physicians from other specialties, including hematology and internal medicine, as well as residents and fellows, frequently encounter patients with diverse types of peripheral neuropathies throughout their clinical careers^3^. In the United States, peripheral nervous system disorders, predominantly polyneuropathy, account for more than 10% of annual neurology consultations^4^. Moreover, polyneuropathy significantly contributes to healthcare resource utilization, and future healthcare expenditures are projected to rise due to the increasing aging population^5^.

Identifying the underlying etiology of polyneuropathy can be challenging due to the wide array of potential etiologies and the fact that, even with comprehensive evaluations, a significant proportion of cases remain idiopathic^1–3^. Despite advancements in diagnostic techniques, establishing an etiological diagnosis of polyneuropathy still heavily relies on detailed patient history and meticulous physical examination, which guide clinicians toward formulating diagnostic hypotheses and selecting appropriate laboratory and instrumental investigations^6^. Indeed, neurologists can ascertain the cause of polyneuropathy in nearly two-thirds of cases based on clinical assessment alone, before initiating diagnostic tests^6^. Inexpensive blood tests further assist neurologists in identifying an additional 15% of polyneuropathy etiologies, whereas more costly investigations, such as magnetic resonance imaging, rarely alter patient management significantly^6^.

Nevertheless, etiologic diagnosis of polyneuropathy often requires substantial clinical expertise. Although many patients are managed in primary care settings, partly due to resource constraints and cost considerations, complex or atypical presentations frequently warrant specialist neurological assessment^7^. Population-based U.S. data indicate that approximately 30% of patients with peripheral neuropathy are evaluated by a neurologist, whereas referral rates from primary care to neurology in Dutch cohorts have been reported to reach 70–80%, depending on symptom characteristics^8,9^. Access to neurologists, particularly those with subspecialty expertise in polyneuropathy, nevertheless remains uneven and is frequently limited in underserved areas^10–12^.

As a consequence of these challenges, numerous studies have highlighted the prevalence of misdiagnoses and significant diagnostic delays in polyneuropathy patients, particularly those initially evaluated at primary or secondary care centers^13–15^. Therefore, there is an urgent need to develop innovative strategies to mitigate diagnostic difficulties and promote earlier and more accurate diagnosis of polyneuropathy.

Artificial intelligence (AI), particularly large language models (LLMs), has advanced significantly and is increasingly recognized as a versatile tool in clinical medicine. Recent studies suggest that LLMs may perform at or exceed the diagnostic capabilities of medical students and physicians in both routine and complex clinical scenarios^16–18^. ChatGPT, a conversational AI developed by OpenAI based on the Generative Pre-trained Transformer architecture, has demonstrated the capacity to generate complex, contextually appropriate diagnostic and medical reasoning without requiring domain-specific training^19^. Its accessibility, coupled with its versatility, low cost/inexpensive nature, and widespread use, positions it as a promising adjunct in clinical practice.

This study aimed to evaluate the diagnostic performance of ChatGPT-4o in real-world, complex polyneuropathy cases typically encountered in tertiary referral centers, where the differential diagnosis is particularly challenging. Its outputs were compared with those of expert and non-expert neurologists specialized in peripheral nervous system disorders.

## Results

### ChatGPT-4o performance metrics and error analysis

Chat-GPT-4o demonstrated substantial agreement across outputs (*κ* = 0.78, *p* < 0.001). Diagnostic accuracy for the leading diagnosis was 65.5%, increasing to 82.0% when all differential diagnoses were considered. The leading diagnosis has a specificity of 98.6%, a sensitivity of 57.3%, and a precision of 72.7%, yielding an F1-score of 58.3%. The accuracy of the confirmatory test was 68.0%. Errors were documented and categorized based on a standardized framework (Table 1). The error prevalence shown in Table 1 is based on the total number of distinct errors identified (*n* = 69) rather than the number of cases. This approach allows for a clearer representation of the distribution of error types. For instance, a single response might contain multiple distinct errors (e.g., misinformation and flawed reasoning).

**Table 1:** Categorization of Chat-GPT-4o errors across two trials (200 cases in total)

Error type and subcategory	Description	Prevalence
**Factual errors**		
Hallucination	Generation of entirely fabricated information.	5/69 (7.2%)
Misinformation	Accurate facts presented incorrectly or misapplied	19/69 (27.5%)
**Query misinterpretation**		0/69 (0%)
**Logical errors**		
Inconsistencies	Conflicting information within the response	3/69 (4.3%)
Flawed deductions		
- *Over-reliance on laboratory findings or historical data*	Excessive focus on lab results or history, ignoring other clinical aspects	16/69 (23.2%)
- *Overlooking clinical information*	Failure to incorporate relevant clinical details into reasoning	25/69 (36.2%)
- *Providing vague conclusions*	Offering ambiguous or unclear final statements	13/69 (18.8%)
**Domain-specific errors**		
Misuse of medical terms	Incorrect application or understanding of medical terminology	1/69 (1.4%)
Incomplete knowledge	Lack of a comprehensive understanding of specific medical conditions	11/69 (15.9%)
Reliance on outdated information	Use of obsolete or superseded medical knowledge	0/69 (0%)

Errors are categorized by type and subcategory, with descriptions and prevalence shown. The denominator (*n* = 69) represents the total number of distinct errors identified across both trials (100 polyneuropathy cases each, totaling 200 cases). The percentages represent the proportion of each error type relative to the total number of errors, not the number of cases.

In the error analysis of missed diagnoses, the most common error type was flawed deductions, which included overlooking provided clinical information, over-reliance on laboratory findings or historical data, and providing vague conclusions (Table 1). Additionally, the model frequently produced factual errors in the form of misinformation or hallucinations. Other error sources encompassed inconsistencies, incomplete internal knowledge, and misuse of medical terms (Table 1). The complete set of Chat-GPT-4o outputs is provided in Supplementary Tables 1–4.

### Comparison of Chat-GPT-4o and neurologists’ performance metrics

**Fig. 1:**
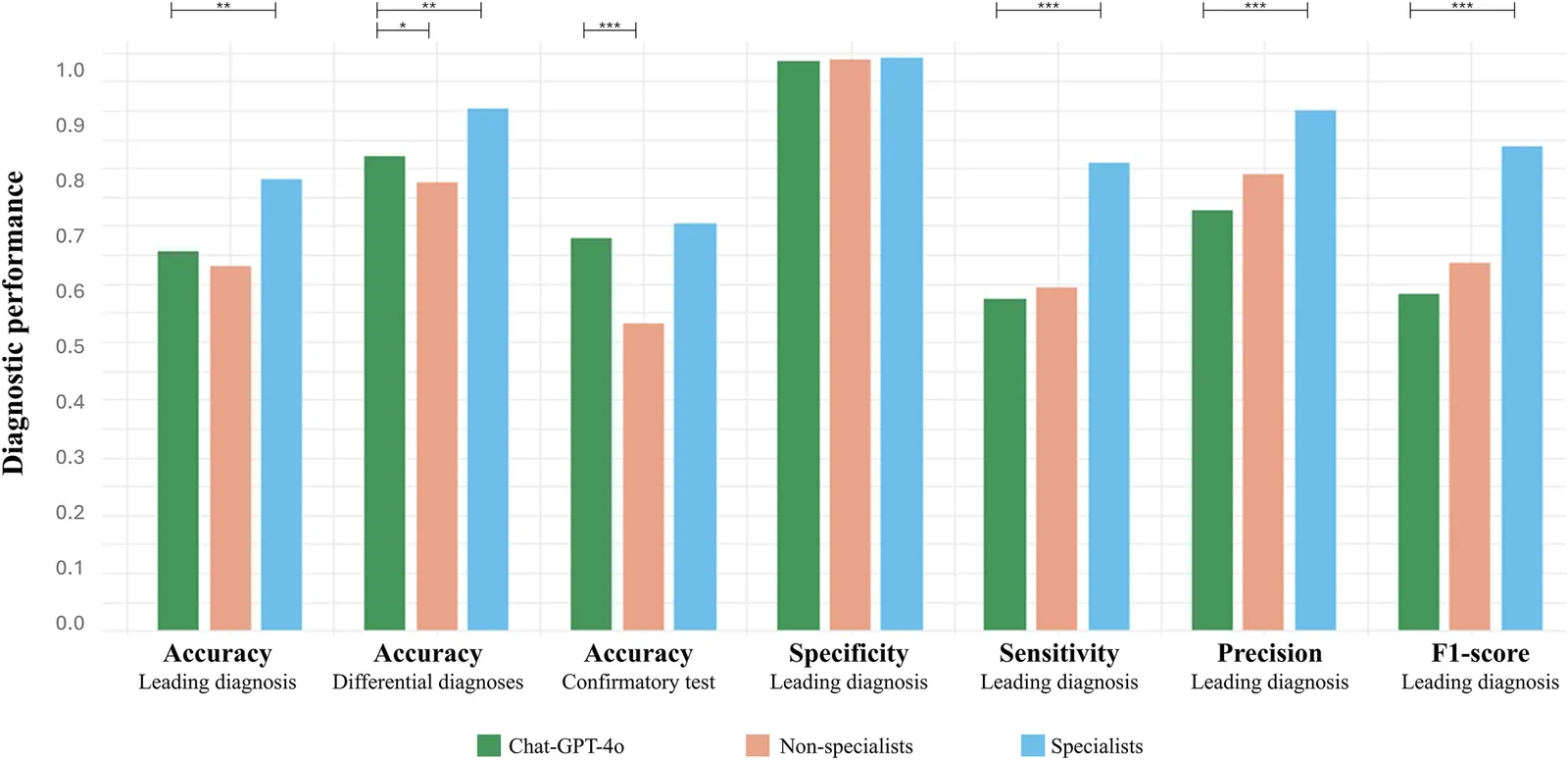
Performance metrics of ChatGPT-4o vs. specialist neurologists in peripheral neuropathies and non-specialist neurologists. Graphical comparison of diagnostic performance metrics across three groups: ChatGPT-4o (green, *n* = 2 trials), peripheral nerve disease specialists (light blue, *n* = 19), and non-specialist neurologists (light orange, *n* = 17), all evaluated on the same set of 100 real-world polyneuropathy cases. Metrics shown include accuracy for the leading diagnosis, accuracy for differential diagnoses, appropriateness of recommended confirmatory tests, sensitivity, specificity, precision, and F1-score. ChatGPT-4o performed comparably to non-specialists on leading diagnosis accuracy and outperformed them in differential diagnosis accuracy and confirmatory test selection, while remaining inferior to specialists across most metrics. *p < 0.05, **p < 0.01, ***p < 0.001.

As summarized in Table 2 and Fig. 1, for the leading diagnosis, Chat-GPT-4o did not differ significantly from non-specialists in accuracy (65.5% vs 63.0%, *p* = 0.379), specificity (98.6% vs 98.8%, *p* = 0.474), sensitivity (57.3% vs 59.4%, *p* = 0.419), precision (72.7% vs 79.0%, *p* = 0.070), or F1-score (58.3% vs 63.7%, *p* = 0.067).

**Table 2:** Performance metrics of ChatGPT-4o vs. specialist neurologists in peripheral neuropathies and non-specialist neurologists

Metrics (mean ± SD)	ChatGPT-4o	Specialist neurologists	*p*-value	Non-specialist neurologists	*p*-value
**Accuracy**					
Leading diagnosis	0.655 ± (0.453)	0.781 ± (0.200)	0.002	0.630 ± (0.290)	0.379
Differentials	0.820 ± (0.366)	0.902 ± (0.123)	0.010	0.775 ± (0.276)	0.043
Confirmatory test	0.680 ± (0.399)	0.705 ± (0.203)	0.517	0.530 ± (0.255)	<0.001
**Specificity**	0.986 ± (0.002)	0.991 ± (0.004)	0.084	0.988 ± (0.005)	0.474
**Sensitivity**	0.573 ± (0.007)	0.810 ± (0.124)	<0.001	0.594 ± (0.093)	0.419
**Precision**	0.727 ± (0.011)	0.901 ± (0.071)	<0.001	0.790 ± (0.121)	0.070
**F1-score**	0.583 ± (0.010)	0.837 ± (0.114)	<0.001	0.637 ± (0.101)	0.067

*Accuracy* refers to the proportion of correct diagnoses across all cases. *Precision* is the fraction of true positives among all predicted positives. *Sensitivity* is the fraction of actual positives correctly identified. *F1-score* is the harmonic mean of Precision and Recall. *Specificity* is the fraction of true negatives correctly identified.

*SD* standard deviation.

In contrast, Chat-GPT-4o did not achieve the performance level of specialists on accuracy (65.5% vs 74%, *p* = 0.018), sensitivity (57.3% vs 81.0%, *p* < 0.001), precision (72.7% vs. 90.1%, *p* < 0.001), and F1-score (58.3% vs. 83.7%, *p* < 0.001) while it had non-significantly inferior specificity (98.6% vs 99.1%, *p* = 0.084).

When differential diagnoses were considered, Chat-GPT-4o had superior accuracy than non-specialists (82% vs 77.5%, *p* = 0.043) but remained inferior to specialists (82% vs. 90.2%, *p* = 0.010). With respect to the accuracy of recommending diagnostic tests, Chat-GPT-4o surpassed non-specialists (68.0% vs 53.0%, *p* < 0.001) and performed similarly to specialists (68.0% vs 70.5%, *p* = 0.517).

No significant differences in performance metrics were observed between the first and second halves of cases, suggesting no measurable learning effect in both ChatGPT-4o and the participating neurologists (Supplementary Table 5).

### Comparison of neurologists’ performance metrics after reviewing Chat-GPT-4o output

**Fig. 2:**
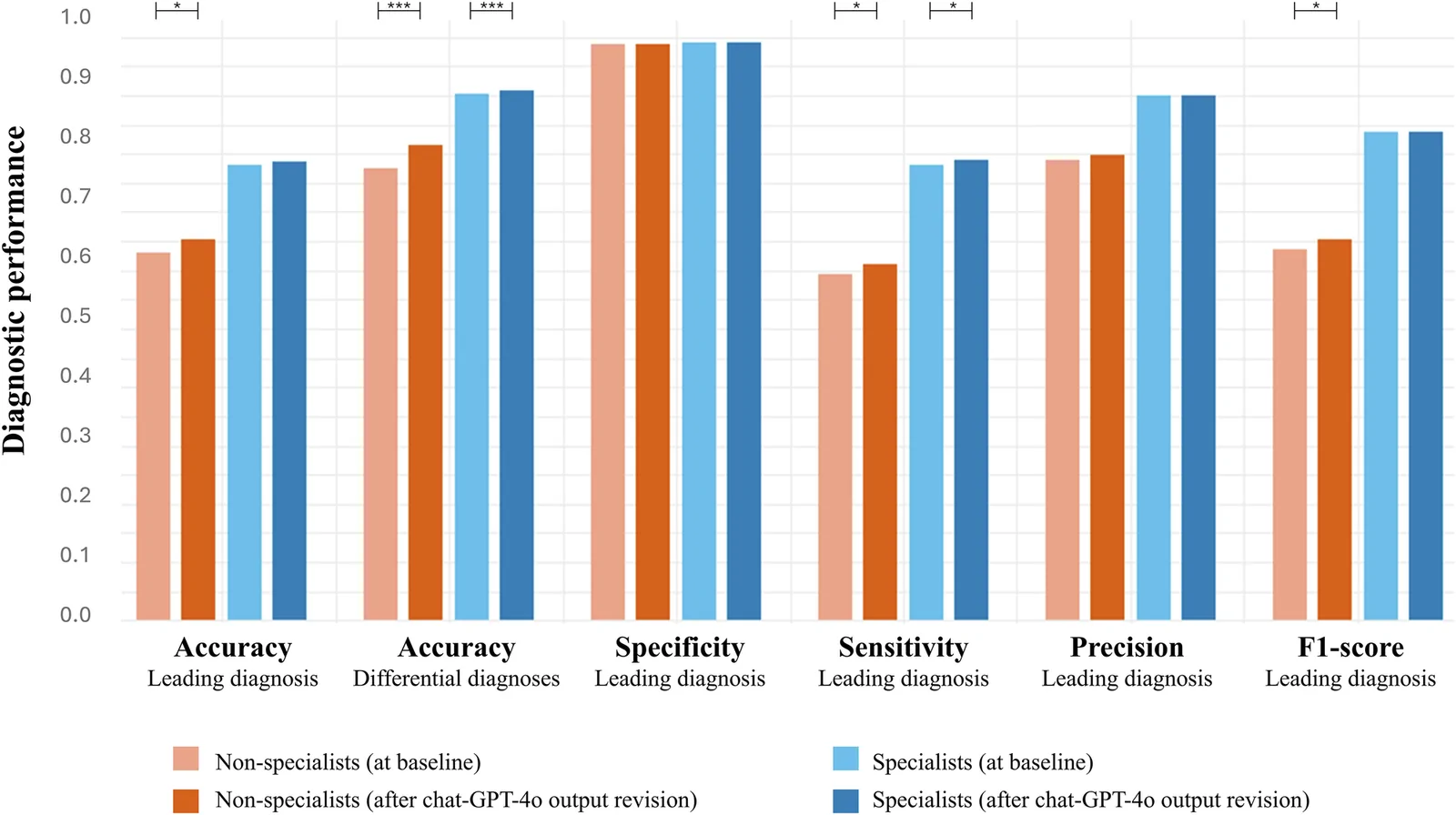
Performance metrics of specialist neurologists in peripheral neuropathies and non-specialist neurologists before and after reviewing Chat-GPT-4o output. Effect of ChatGPT-4o output review on neurologist diagnostic performance. The figure compares performance metrics before and after neurologists were shown ChatGPT-4o diagnostic suggestions, presented separately for non-specialist neurologists (light orange: before; orange: after) and specialist neurologists (light blue: before; blue: after). Metrics displayed include accuracy for the leading diagnosis, accuracy for differential diagnoses, sensitivity, specificity, precision, and F1-score. Non-specialist neurologists showed significant improvements across multiple metrics following AI review, whereas changes among specialists were minimal and largely non-significant, reflecting the differential impact of AI-assisted decision support according to level of experience in peripheral neuropathy. *p < 0.05, **p < 0.01, ***p < 0.001.

After reviewing ChatGPT-4o’s output, non-specialists were significantly more likely to modify their responses compared to specialists (21.8% vs. 9.1%, *p* < 0.001). As summarized in Table 3 and Fig. 2, non-specialists showed slight but significant improvements in their metrics: accuracy increased from 63% to 65.4% (*p* = 0.013), sensitivity from 59.4% to 61.0% (*p* = 0.050), and F1-score from 63.7% to 65.4% (*p* = 0.038). Among specialists, improvements were not significant in leading diagnosis metrics. Similarly, when differential diagnoses were considered, non-specialists’ accuracy improved from 77.5% to 81.5% (*p* < 0.001), whereas specialists’ accuracy increased only marginally from 90.2% to 90.8% (*p* < 0.001) Table 4.

**Table 3:** Performance metrics of specialist neurologists in peripheral neuropathies and non-specialist neurologists before and after reviewing Chat-GPT-4o output

Metrics (mean ± SD)	Specialists (Basic)	Specialists (after GPT-4o)	*p*-value	Non-specialists (Basic)	Non-specialists (after GPT-4o)	*p*-value
Accuracy						
Leading diagnosis	0.781 ± (0.200)	0.787 ± (0.202)	0.174	0.630 ± (0.290)	0.654 ± (0.308)	0.013
Differentials	0.902 ± (0.123)	0.908 ± (0.202)	<0.001	0.775 ± (0.276)	0.815 ± (0.308)	<0.001
**Specificity**	0.991 ± (0.004)	0.991 ± (0.004)	0.594	0.988 ± (0.005)	0.989 ± (0.005)	0.100
**Sensitivity**	0.781 ± (0.117)	0.789 ± (0.117)	0.016	0.594 ± (0.093)	0.610 ± (0.83)	0.050
**Precision**	0.901 ± (0.071)	0.900 ± (0.072)	0.623	0.790 ± (0.121)	0.799 ± (0.123)	0.240
**F1-score**	0.837 ± (0.114)	0.836 ± (0.107)	0.817	0.637 ± (0.101)	0.654 ± (0.092)	0.038

*Accuracy* refers to the proportion of correct diagnoses across all cases. *Precision* is the fraction of true positives among all predicted positives. *Recall* is the fraction of actual positives correctly identified. *F1-score* is the harmonic mean of Precision and Recall. *Specificity* is the fraction of true negatives correctly identified.

*SD* standard deviation.

## Discussion

This study evaluated the diagnostic performance of ChatGPT-4o in real-world polyneuropathy cases, comparing its outputs with those of specialist and non-specialist neurologists. ChatGPT-4o achieved a leading diagnosis accuracy of 65.5%, which increased to 82.0% when considering all differential diagnoses. Although it did not match specialist performance, its accuracy was comparable to that of non-specialists. Notably, ChatGPT-4o outperformed non-specialists in accuracy of proposed differential diagnoses (82% vs 77.5%) and accuracy in recommending diagnostic tests (68.0% vs. 53.0%, *p* < 0.001). These findings are consistent with recent studies showing that LLMs perform comparably to neurologists and residents in clinical scenarios, such as clinical vignettes, clinical score calculation, brain tumor diagnosis, neuroophthalmological reasoning, neurologic localization, and prehospital stroke triage^16,20–26^. Our findings further support the potential of LLMs in complex diagnostic areas such as polyneuropathy, where the wide range of potential causes and diagnostic complexities make such a tool potentially useful in aiding diagnostic decision-making.

A key finding of this study was ChatGPT-4o’s higher accuracy in recommending diagnostic tests compared to non-specialist neurologists (68.0% vs. 53.0%, *p* < 0.001). This suggests that the model could be a valuable tool for clinicians, particularly non-specialist neurologists, by offering evidence-based diagnostic recommendations in settings where specialists are not readily available. This could lead to better rationalization of diagnostic tests and specialist referrals, ultimately reducing healthcare costs and the time to diagnosis. As healthcare systems continue to face challenges such as neurologist shortages and rising costs in both Europe and the US^11,12^, LLMs could help bridge the gap between specialist and non-specialist neurologists. In the future, integrating LLMs into clinical workflows could enable more accurate and timely diagnoses, improving patient outcomes.

Additionally, non-specialist neurologists were significantly more likely to revise their initial diagnoses after reviewing ChatGPT-4o’s outputs (21.8% vs. 9.1% among specialists, *p* < 0.001), which led to an improvement in their performance metrics. This finding suggests that AI-assisted decision support may enhance diagnostic performance in the context of complex polyneuropathy cases, particularly among clinicians with less experience in polyneuropathies. While specialists experienced less improvement, AI may still provide value as a second opinion or as a means of cross-checking diagnostic reasoning. It should be noted, however, that non-specialist neurologists typically manage a case mix dominated by common polyneuropathies, which are underrepresented in this cohort. The present study instead focuses on diagnostically complex cases that are more likely to be missed or substantially delayed before referral and may therefore represent the clinical scenarios in which structured decision support is most relevant.

Given the widespread availability and low cost of ChatGPT, it is plausible that clinicians are already using it in real-world clinical settings when confronted with complex or diagnostically challenging polyneuropathy cases (https://www.ama-assn.org/press-center/press-releases/ama-physician-enthusiasm-grows-health-care-ai)^27^. Understanding its reliability and limitations in such contexts is therefore essential. Careful consideration should be given to prompt phrasing, as it can influence the reliability of the model’s output. In this study, a Chain-of-Thought prompting approach was employed, given its superior performance in previous research^20^, suggesting that similar structured input methods may be beneficial in real-world applications. Despite encouraging results, ChatGPT-4o did not reach specialist-level performance. The observed 34.5% misdiagnosis rate remains substantial, even under conditions of carefully curated and standardized case presentation. A considerable proportion of errors were classified as “flawed deductions,” including over-reliance on laboratory findings (23.2%) and overlooking relevant clinical information (36.2%). Several of these errors were clinically meaningful. Over-reliance on laboratory abnormalities occasionally led to overinterpretation of monoclonal gammopathy in chronic inflammatory demyelinating polyradiculoneuropathy (CIDP) cases, with potential implications for inappropriate or delayed immunotherapy. Conversely, failure to recognize characteristic clinical patterns resulted in missed identification of POEMS (polyneuropathy, organomegaly, endocrinopathy, M-protein, skin changes) syndrome, potentially delaying diagnosis of an underlying hematologic disorder requiring timely treatment. These findings indicate that, although LLMs may support diagnostic reasoning, their errors may carry non-trivial clinical consequences, particularly in complex neuroimmunological conditions. Similar limitations have been noted in other AI diagnostic studies, especially in cases involving multimorbidity or atypical presentations that demand nuanced clinical judgment or managing complex therapeutic decisions.^28–30^. Future updates to the Chat-GPT model or other LLMs may further improve performance, but current systems cannot replace expert clinical reasoning, particularly in complex or ambiguous scenarios.

Another critical consideration is the potential impact of sustained AI exposure on clinician performance. Emerging evidence suggests that continuous reliance on AI systems may attenuate independent diagnostic vigilance and contribute to clinician deskilling^31^. In our study design, GPT-generated differentials were disclosed only after neurologists had formulated their own diagnostic hypotheses. Incorporating cognitive-forcing strategies may help preserve autonomous reasoning and reduce overreliance on AI-generated suggestions, underscoring the importance of carefully structured integration of AI tools into clinical workflows.

This study has several limitations. First, the sample size of 100 cases may not be sufficient to generalize ChatGPT-4o’s performance across all types of polyneuropathies. In addition, cases were selected from tertiary referral centers and therefore may not represent the full spectrum of neuropathies encountered in primary care or community settings. Further research with larger and more diverse datasets, including cases from non-specialist environments, is needed to assess generalizability. A major limitation is the use of standardized, expert-curated case summaries. This likely represents an optimized scenario and may overestimate ChatGPT-4o’s diagnostic performance compared with routine clinical practice, where clinical information is often incomplete, less structured, or inconsistently documented. Data abstraction required recognition of relevant historical details, comorbidities, and subtle neurological signs that may not always be elicited or reported by non-specialist clinicians. Similarly, nerve conduction study results were provided as interpreted summaries rather than raw electrophysiological data. Although these summaries were not independently re-analyzed by experts, their use may still bypass an important real-world diagnostic bottleneck, as misinterpretation of electrophysiological findings is a recognized contributor to misdiagnosis, particularly in CIDP. Nevertheless, the standardized format allowed ChatGPT-4o, specialists, and non-specialists to be compared using the same clinical information, providing an estimate of the model’s potential under controlled conditions. Accordingly, our results should be interpreted as a best-case estimate of ChatGPT-4o’s diagnostic ability, rather than as evidence of effectiveness in routine clinical practice. The specific secure, privacy-compliant version of ChatGPT-4o Enterprise used in this study is not currently intended, validated, or available for routine diagnostic use; therefore, routine use of ChatGPT or similar LLMs for the diagnosis and management of polyneuropathies cannot be recommended at this time. Future prospective studies should evaluate LLM performance in true real-world settings, including unstructured clinical notes, variable-quality examinations, and raw or non-expert-interpreted diagnostic data at the point of care. Finally, case summaries used expert-standardized terminology, which may favor both specialists and LLMs trained on specialist-authored biomedical literature compared with non-specialists less familiar with this language. All cases were translated from Italian into English. Given the structured and codified nature of the data, translation-related bias is unlikely. Although LLMs are predominantly trained on English-language data, and input language could theoretically influence performance, preliminary evidence has not demonstrated a significant effect of source language on LLM diagnostic accuracy^32^; however, these findings require confirmation in real-world settings.

In conclusion, this study provides preliminary evidence that ChatGPT-4o demonstrates moderate diagnostic accuracy in complex polyneuropathy cases, with performance comparable to non-specialist neurologists but inferior to peripheral neuropathy specialists. Notably, the model showed superior performance in certain domains, including differential diagnosis and selection of confirmatory tests, and was associated with measurable improvements in diagnostic accuracy among non-specialists after output review. Although it did not reach specialist-level accuracy, ChatGPT-4o could potentially serve as an adjunct support tool in resource-limited environments at critical decision points in the referral pathway for complex or atypical polyneuropathies. However, these findings were obtained under optimized conditions using standardized, expert-generated case summaries. They should therefore be interpreted as an estimate of the model’s potential under equal-information conditions, not as evidence supporting routine clinical use. Because real-world practice involves incomplete clinical information, variable examination quality, and possible diagnostic test misinterpretation, routine use of ChatGPT or similar LLMs for the diagnosis and management of polyneuropathies is not appropriate or recommended at this time. Future prospective studies should assess LLM performance in real-world settings and evaluate its impact on diagnostic processes and patient outcomes.

## Methods

### Study population and case presentation

Between October and December 2023, demographic, clinical, and diagnostic data were collected from patients with a confirmed diagnosis of polyneuropathy, consecutively selected from those attending the polyneuropathy outpatient clinics of the Humanitas Research Institute and the Fondazione IRCCS Istituto Neurologico Carlo Besta, both located in Milan, Italy. Both institutions serve as tertiary referral centers for peripheral neuropathies, following patients with various types of polyneuropathies, including metabolic, inflammatory, and genetic forms. A total of 409 cases were identified, from which 100 were randomly selected using a computer-generated random sampling method (random number generator), ensuring that each case had an equal probability of selection (Table 4). Data were extracted from medical records at two key time points: the initial neurological consultation and the first follow-up visit, which occurred after the patient had undergone a screening battery of diagnostic tests for polyneuropathy, including laboratory tests and nerve conduction studies.

**Table 4:** Polyneuropathy cases in the study cohort

Cases	*n* (%)
Chronic inflammatory demyelinating polyradiculoneuropathy	13
POEMS syndrome	12
Chemotherapy-induced peripheral neuropathy	11
Charcot-Marie-Tooth disease	9
Anti-myelin-associated glycoprotein (MAG) neuropathy	8
Diabetic polyneuropathy	6
Vitamin B12-deficiency neuropathy	6
Guillain-Barré syndrome	4
Axonal neuropathy associated with MGUS	4
Vasculitic neuropathy	4
Multifocal motor neuropathy	3
Small fiber neuropathy	3
ATTRv neuropathy	3
CANVAS	2
Hepatitis C virus-associated neuropathy	2
Alcohol-associated neuropathy	1
Copper-deficiency neuropathy	1
Folate-deficiency neuropathy	1
Hepatitis B virus-associated neuropathy	1
HNPP	1
Sarcoidosis-associated neuropathy	1
Sjögren syndrome-associated neuropathy	1

*ATTRv* hereditary transthyretin amyloidosis, *CANVAS* cerebellar ataxia, neuropathy, and vestibular areflexia syndrome, *HNPP* hereditary neuropathy with liability to pressure palsies, *MAG* myelin-associated glycoprotein, *MGUS* monoclonal gammopathy of undetermined significance, *MMN* multifocal motor neuropathy, *POEMS syndrome* polyneuropathy, organomegaly, endocrinopathy, monoclonal protein, and skin changes.

The following clinical and demographic data were collected: gender, age at neuropathy symptom onset, type of onset (acute, subacute, chronic-insidious), clinical course (monophasic, relapsing, chronic, stepwise), symptoms distribution (length-dependent or non-length-dependent; symmetric, asymmetric, multineuropathic), type of symptoms (pure motor, pure sensory, mixed), and presence of additional clinical features (e.g., cranial nerve involvement, tremor, pain, ataxia, autonomic symptoms, cramps, fasciculations, foot deformities, chronic cough, skin change, and spasticity). Additional data were gathered on the location of symptom manifestation (community, hospital, intensive care unit), family history of polyneuropathy with potential inheritance pattern (autosomal dominant, recessive, X-linked), and relevant comorbidities (e.g., cardiac abnormalities, monoclonal gammopathy, hematological diseases, diabetes, rheumatological conditions, cancer, chemotherapy exposure, hepatic or renal insufficiency, and alcohol abuse).

Nerve conduction study results were recorded exactly as reported in the original test documentation from the patient’s initial evaluation, most often performed at primary or secondary care centers, using the interpretative summaries provided (e.g., “demyelinating polyneuropathy,” “sensorimotor involvement,” “four-limb involvement”), without access to raw waveform data or independent expert re-analysis of electrophysiological parameters. This approach was used to simulate a real-world scenario in which non-specialist neurologists typically rely on the electrophysiological study reports and to evaluate ChatGPT’s diagnostic capabilities when provided with this type of clinical information. The presence of conditions such as carpal tunnel syndrome, conduction blocks, or other abnormalities was recorded if mentioned in the report. Screening blood test results for polyneuropathy were collected^33,34^, including tests requested by the general practitioner prior to the initial neurological visit or by the neurologist during the first consultation. These tests included vitamin B12, B9, B6, E, and copper levels, erythrocyte sedimentation rate, C-reactive protein, glycemia, HbA1c, cryoglobulinemia, serum protein electrophoresis with immunofixation, HBV or HCV infection markers, ANA (antinuclear antibodies), ENA (extractable nuclear antigens), and ANCA (anti-neutrophil cytoplasmic antibody) autoantibodies. Results from second-level laboratory tests (e.g., vascular endothelial growth factor or VEGF)^33,34^, if requested by the neurologist during the initial visit, were excluded to assess ChatGPT’s diagnostic capabilities based solely on medical history, clinical information, and screening lab tests for polyneuropathy. This approach simulates a first or second neurological consultation, ensuring that the evaluation reflects the diagnostic process typically available at those stages.

Cases with insufficient clinical or diagnostic information were excluded. Only cases with a stable final diagnosis, confirmed after at least 12 months of follow-up, were included (complete selection process data available at Supplementary Fig. 1). Additionally, cases without a confirmed etiological diagnosis were excluded, as the retrospective nature of the analysis would not allow for an accurate assessment of ChatGPT’s diagnostic utility in such cases. All patient data were anonymized in accordance with institutional ethics protocols. This study was conducted in accordance with the principles of the World Medical Association Declaration of Helsinki. The study protocol was approved by the Ethics Committee of Humanitas Research Hospital (approval number: 485/24). In accordance with regional regulations of the Lombardy Region, this approval also applies to studies conducted within the regional clinical network, including IRCCS Istituto Neurologico Carlo Besta. Due to the retrospective design of the study, participants were contacted to obtain informed consent whenever possible, and written informed consent was obtained from those who responded. For individuals who could not be contacted despite reasonable efforts, the requirement for informed consent was waived by the Ethics Committee. All clinical data were fully anonymized prior to analysis. Table 1 displays the final study cohort.

### Study design

**Fig. 3:**
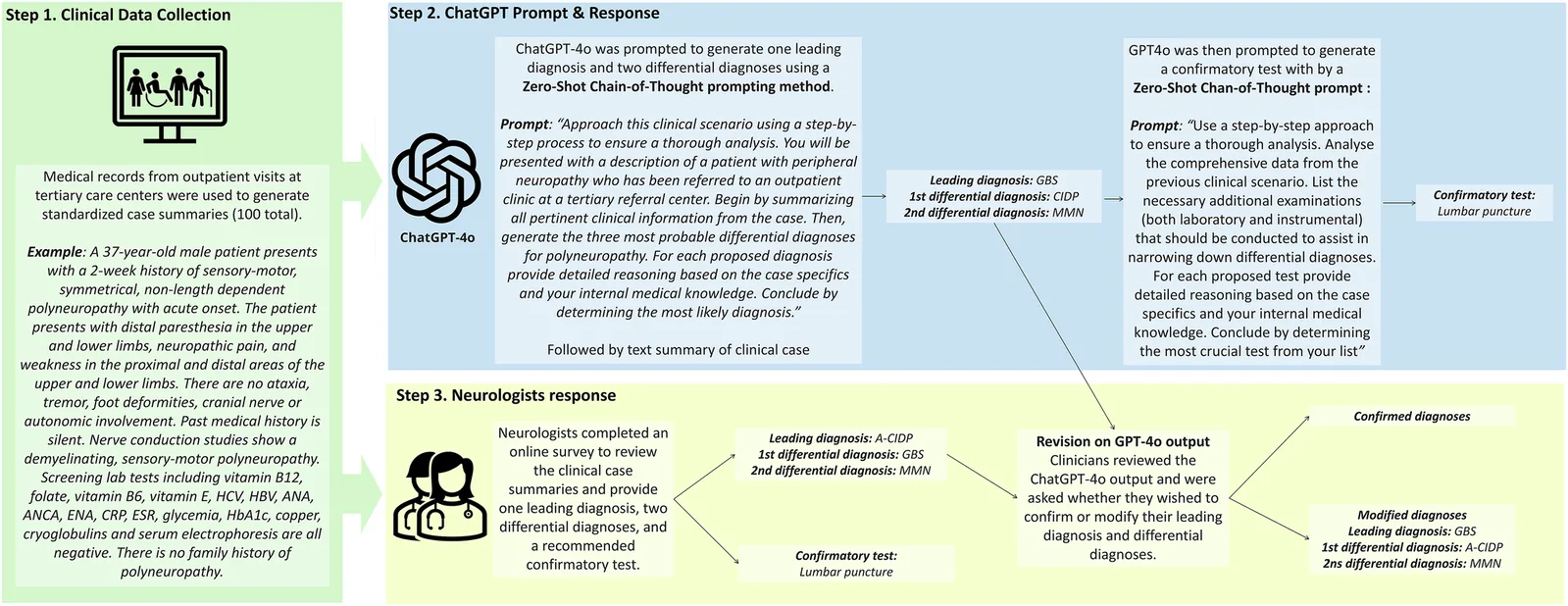
Summary of methods. Overview of study design and methodology. The figure illustrates the end-to-end workflow of the study across three sequential steps. First, 100 anonymized polyneuropathy cases were selected from two tertiary referral centers in Milan, Italy, and synthesized into standardized written summaries presenting clinical, electrophysiological, and laboratory information in a fixed, consistent order (an illustrative example of a complete case summary is reproduced within the figure). Second, each case summary was submitted to ChatGPT-4o using a structured zero-shot chain-of-thought prompt (reproduced in full within the figure) instructing the model to provide one leading diagnosis, two alternative differential diagnoses, and one recommended confirmatory diagnostic test; the entire process was repeated in two fully independent trials to assess the consistency and reproducibility of model outputs. Third, the same 100 cases were presented to 36 neurologists from 24 centers across 10 countries via a dedicated web-based interface, following a two-phase sequential protocol: in Phase 1, neurologists independently formulated their diagnostic responses; in Phase 2, each case was presented again alongside ChatGPT-4o’s response, and neurologists were given the opportunity to confirm or revise their previous answers. This design enabled simultaneous assessment of ChatGPT-4o’s standalone diagnostic performance relative to human raters, and quantification of the influence of AI-generated suggestions on neurologist diagnostic reasoning.

The selected cases were synthesized into standardized case summaries by ADL, AB, and GSM. For each case, clinical information was provided in a fixed order, including patient demographics, detailed symptom description, neurological examination findings, medical history, nerve conduction study results, screening laboratory tests, and family history. An illustrative example is shown in Fig. 3, while the complete dataset is available in Supplementary Tables 1–4.

All cases were presented in English and in randomized order to both ChatGPT and a group of neurologists. The neurologists accessed the study via a web-based interface, providing free-text responses. In the first phase, both ChatGPT and the neurologists were requested to provide one leading diagnosis and two less likely alternative diagnoses for each case (Fig. 3). They were also asked to recommend a laboratory or instrumental test capable of confirming the leading diagnosis while excluding the other two hypotheses (Fig. 3).

In the subsequent phase, each case was shown again to the neurologists, this time accompanied by ChatGPT's answer (Fig. 3). The neurologists were then given the option to confirm or modify their previously stated diagnoses, whether the leading diagnosis, the alternative diagnoses, or both. Participating neurologists were blinded to the correct diagnosis at all stages. Outcome assessors and data analysts were blinded to the identity and category of the participating neurologists by assigning random numbers to each neurologist. The primary endpoint of this study was to evaluate the diagnostic performance of ChatGPT in formulating a leading diagnosis for peripheral neuropathies, compared to expert and non-expert neurologists. The appropriateness of the leading diagnosis was assessed by comparing it with the patient’s final confirmed diagnosis. Secondary endpoints included assessing the accuracy of alternative diagnoses and the appropriateness of the laboratory or instrumental examinations requested. These secondary outcomes were evaluated at the end of the study by three neurologists (ENO, DP, PED) with extensive experience in managing polyneuropathy, who served as expert adjudicators. Acting as a gold standard reference, they independently assessed the alternative diagnoses and requested investigations using binary judgments (correct/incorrect for alternative diagnoses; appropriate/inappropriate for examinations). In cases of disagreement, consensus was reached through discussion.

### Prompt engineering, ChatGPT-4o settings, and error classification

This study utilized ChatGPT-4o Enterprise, developed by OpenAI for professional use and enhanced security. The model runs with default settings that safeguard confidentiality, ensuring no input data is retained or used for future training. Access was via the default ChatGPT interface, where temperature and top-p parameters are managed by the system to produce coherent, contextually appropriate outputs, and are not adjustable by the user.

Clinical cases were presented to ChatGPT-4o from September 16th to September 18th, 2024, using standardized prompts developed through a zero-shot chain-of-thought (CoT) approach, which includes the phrase “Approach this clinical scenario using a step-by-step process” to enhance logical reasoning without requiring exemplars. The prompt was refined iteratively with the model’s assistance (Fig. 3).

To minimize bias, the clinical cases were presented in randomized order, each in a separate chat session with conversation history cleared prior to input. Model outputs were collected in two independent trials to assess response stability and then evaluated for consistency. The first trial’s responses were summarized for review by neurologists. Outputs with incorrect leading diagnoses were analyzed using an error categorization framework based on prior literature and preliminary review of ChatGPT-4o responses^35^. See Table 1 for the detailed description of the error categories.

### Neurologists survey

A total of 36 neurologists from 24 centers across 10 countries participated: Italy (*n* = 12), France (*n* = 8), Spain (*n* = 3), the United Kingdom (*n* = 3), Argentina (*n* = 2), Brazil (*n* = 2), Cyprus (*n* = 2), Serbia (*n* = 2), Bangladesh (*n* = 1), and the Netherlands (*n* = 1). Participants were classified as either peripheral nerve disease specialists (*n* = 19)—neurologists routinely practicing in tertiary care centers dedicated to polyneuropathy management—or non-specialists (*n* = 17), including general neurologists or those sub-specialized in other fields.

### Statistical analysis

*Accuracy* (the proportion of correct diagnoses) was calculated for each classifier (2 GPT trials, 19 Specialists, 17 Non-specialists) across three dimensions: correctness of leading diagnosis, inclusion of the correct diagnosis within the differential diagnosis list, and appropriateness of the suggested confirmatory test. These accuracy measures were then averaged within each group of classifiers (GPT, Specialists, Non-specialists) to obtain aggregate performance metrics for each group of classifiers. Comparisons between ChatGPT-4o and human classifiers (specialists and non-specialists) were conducted using paired *t*-tests, with each case treated as a paired observation across groups.

In line with recommendations from the literature^36^, multiple evaluation metrics were computed to assess leading diagnosis performance. A one-vs-all approach was used to construct binary 2 × 2 contingency tables for each diagnosis represented by more than six cases (CIDP, POEMS, Chemotherapy-induced neuropathy, CMT, anti-MAG neuropathy, diabetic neuropathy, vitamin B12-deficiency neuropathy). For each diagnosis and classifier the following metrics were calculated: *Specificity* (the proportion of true negatives correctly identified, indicating the ability to rule out cases that are not the target condition), *Sensitivity* (the proportion of actual positive cases correctly identified, reflecting how comprehensively the model detects the target condition), *Precision* (the proportion of predicted positives that are actually positive, capturing precision of the model in assigning a positive label), and *F1-score* (the harmonic mean of Precision and Sensitivity, which balances both measures into a single metric). Weighted average metrics over diagnoses were computed for each classifier and subsequently averaged within each group of classifiers (GPT, Specialists, Non-specialists). Group comparisons were conducted using Welch’s *t*-test for between-group analyses and paired *t*-tests to assess changes before and after reviewing ChatGPT output. For all metrics, cases with missing data were excluded from the calculations; no imputation was performed, and analyses were based solely on available data for each variable.

Categorical variables are presented as frequencies and percentages, while continuous variables are reported as means and standard deviations (or medians and interquartile ranges, where appropriate). Consistency across different ChatGPT-4o trials was evaluated using Cohen’s kappa. Statistical significance was set at an α-level of 0.05. All analyses were conducted using IBM SPSS Statistics for Windows, Version 29.0 (Armonk, NY: IBM Corp, USA).

## Supplementary information

Supplementary Information

Description of Additional Supplementary Files

## Data Availability

The complete dataset of Chat-GPT4o outputs generated and analyzed during the current study is available in Supplementary Tables (1–4). The datasets of neurologists' outputs generated and analyzed during the current study are not publicly available due to confidentiality agreements that prevent public sharing, but are available from the corresponding author on reasonable request.
