# Supplementary material for: ChatGPT in the diagnosis and management of complex polyneuropathies: comparative analysis with neurologists using real-world cases
Source: NPJ Digit Med. 2026 Jun 5;9:685. doi: 10.1038/s41746-026-02815-y (PMC13554241; doi:10.1038/s41746-026-02815-y)
Supplement: Supplementary file 1 [file 41746_2026_2815_MOESM1_ESM.pdf]

Supplementary Figure 1. Case selection flowchart

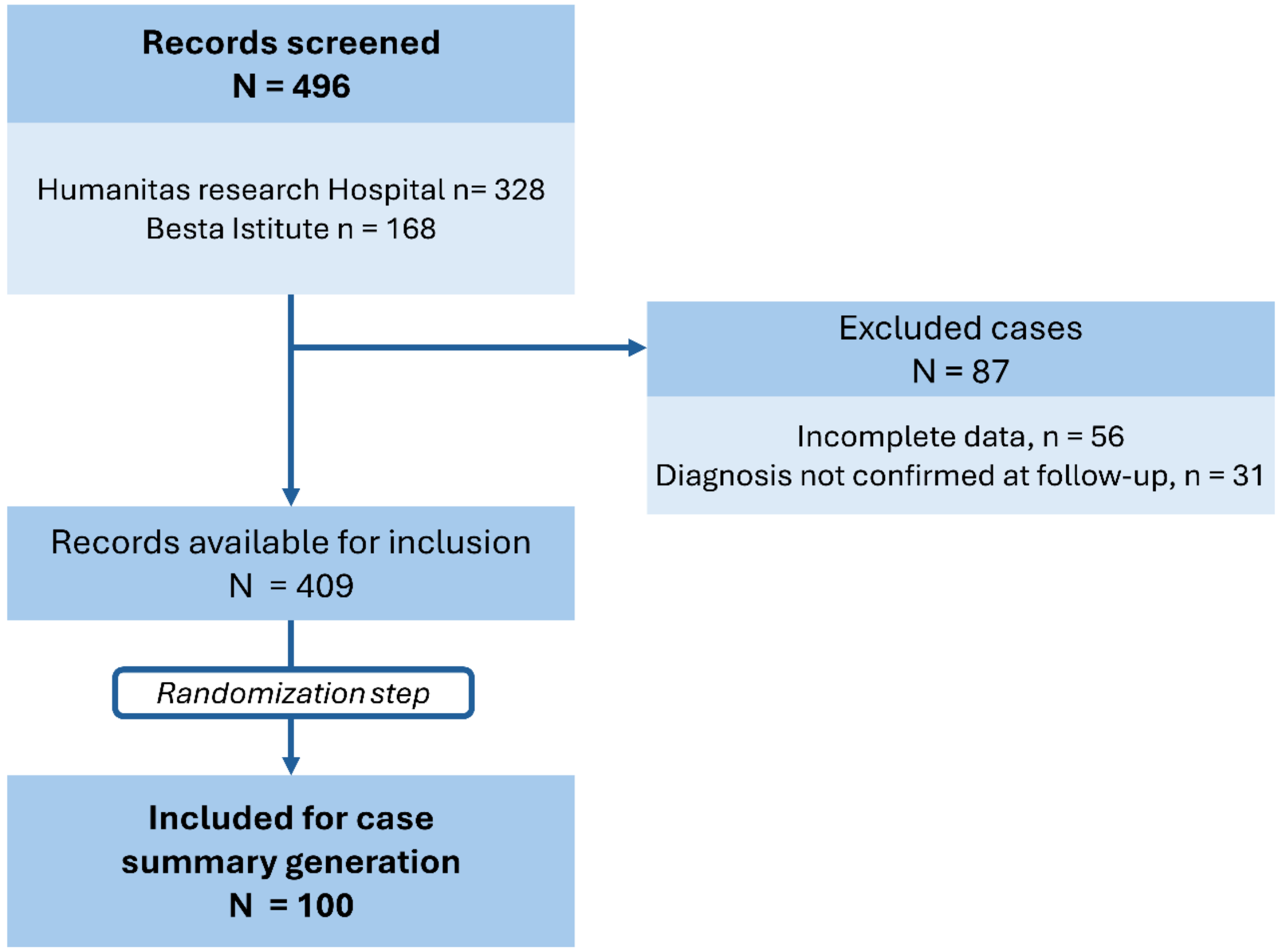

<u>Supplementary Table 1.</u> Trial 1 – Case presentations and GPT-4o output to 1st prompt (diagnosis)

LEGEND: Anti-MAG neuropathy, anti-myelin-associated glycoprotein neuropathy; CANVAS, cerebellar ataxia, neuropathy, and vestibular areflexia syndrome; CIDP, chronic inflammatory demyelinating polyneuropathy; CMT, Charcot-Marie-Tooth disease; GBS, Guillain-Barré syndrome; HBV, hepatitis B virus; HCV, hepatitis C virus; HNPP, hereditary neuropathy with liability to pressure palsies; MGUS, monoclonal gammopathy of undetermined significance; MMN, multifocal motor neuropathy; POEMS, polyneuropathy, organomegaly, endocrinopathy, monoclonal gammopathy, and skin changes syndrome; TTR-Amyloidosis, transthyretin amyloidosis-associated neuropathy;

N°	Case presentation and Diagnosis	Diagnosis	Prompt 1 output
1	**Case presentation:** A 32-year old male patient presents with a 26-year history of sensory-motor, symmetrical, length dependent polyneuropathy with insidious onset and slow progression. The patient presents with scoliosis, neuropathic pain, sensory ataxia, pes cavus, distal weakness in the four limbs, severe calf atrophy with no cranial nerve or autonomic involvement. Past medical history is silent. Nerve conduction studies show an axonal, length dependent, symmetric, sensory-motor polyneuropathy. Screening lab tests including vitamin B12, folate, vitamin B6, vitamin E, HBV, HCV, glycemia, HbA1c, ANA, ANCA, ENA, CRP, ESR, copper, cryoglobulins, and serum electrophoresis are all negative. Both her father and grandfather have foot deformities and walking difficulties.	CMT2	**Summary of Clinical Information**<br>● **Patient**: 32-year-old male<br>● **History**: 26-year history of sensory-motor, symmetrical, length-dependent polyneuropathy<br>○ **Onset**: Insidious<br>○ **Progression**: Slow<br>● **Symptoms**:<br>○ Scoliosis<br>○ Neuropathic pain<br>○ Sensory ataxia<br>○ Pes cavus (high-arched feet)<br>○ Distal weakness in all four limbs<br>○ Severe calf atrophy<br>○ No cranial nerve or autonomic involvement<br>● **Past Medical History**: Unremarkable<br>● **Nerve Conduction Studies**: Axonal, length-dependent, symmetric, sensory-motor polyneuropathy<br>● **Screening Lab Tests**: All negative (including vitamins, viral markers, autoimmune markers, and others)<br>● **Family History**: Father and grandfather have foot deformities and walking difficulties<br>**Differential Diagnoses for Polyneuropathy**<br>1. **Charcot-Marie-Tooth Disease (CMT)**<br>○ **Reasoning**:<br>■ **Genetic Pattern**: The patient's family history of similar symptoms (foot deformities and walking difficulties in father and grandfather) suggests a hereditary neuropathy.<br>■ **Clinical Presentation**: Symptoms such as pes cavus, distal weakness, severe calf atrophy, and length-dependent neuropathy are characteristic of CMT.<br>■ **Progression**: Slow progression over years aligns with the natural history of CMT.<br>■ **Nerve Conduction Studies**: Axonal polyneuropathy is consistent with certain subtypes of CMT, particularly CMT2 which is typically axonal.<br>2. **Hereditary Sensory and Motor Neuropathy (HSMN)**<br>○ **Reasoning**:<br>■ **Genetic Pattern**: Similar to CMT, HSMN encompasses a variety of hereditary neuropathies with similar inheritance patterns.<br>■ **Clinical Presentation**: Sensory ataxia, length-dependent neuropathy, and distal weakness are also seen in HSMN.<br>■ **Nerve Conduction Studies**: Axonal involvement is consistent with many HSMN subtypes.<br>■ **Family History**: Supports a hereditary component.<br>3. **Friedreich's Ataxia**<br>○ **Reasoning**:<br>■ **Clinical Presentation**: Although less common, Friedreich's Ataxia can present with sensory ataxia, scoliosis, and foot deformities (pes cavus).<br>■ **Progression**: Progressive nature over many years fits the disease profile.<br>■ **Family History**: Inherited pattern could align, though usually autosomal recessive.<br>■ **Nerve Conduction Studies**: Can show axonal neuropathy.<br>■ **Additional Features**: Lack of cardiac and endocrine involvement (e.g., diabetes) makes this less likely but still a consideration due to overlapping features.<br>**Most Likely Diagnosis**<br>Based on the clinical presentation, family history, and nerve conduction studies, the most likely diagnosis is **Charcot-Marie-Tooth Disease (CMT)**. The combination of hereditary pattern, typical foot deformities, sensory-motor polyneuropathy, and axonal findings strongly supports this diagnosis. Genetic testing would be the next step to confirm the specific subtype of CMT and further differentiate it from other hereditary neuropathies
2	**Case presentation:** An 88-year-old male patient presents with a 19-month history of pure sensory, symmetrical, length dependent polyneuropathy with insidious onset and slow progressive course. The patient presents with hypoesthesia, paresthesia, and cramps in the lower limbs in a stocking distribution. Clinical examination does not reveal weakness or sensory ataxia, neuropathic pain, tremor, foot deformities, cranial nerve or autonomic involvement. Past medical history is positive for colorectal cancer, for which the patient underwent chemotherapy 5 years ago, and chronic	HCV-associated neuropathy	**Summary of Pertinent Clinical Information**<br>**Patient Details:**<br>● **Age/Gender:** 88-year-old male<br>● **Presentation:** 19-month history of pure sensory, symmetrical, length-dependent polyneuropathy<br>○ **Symptoms:** Hypoesthesia, paresthesia, and cramps in the lower limbs (stocking distribution)<br>○ **Course:** Insidious onset and slow progressive course<br>● **Clinical Examination:**<br>○ **Absence of:** Weakness, sensory ataxia, neuropathic pain, tremor, foot deformities, cranial nerve involvement, and autonomic involvement<br>● **Past Medical History:**<br>○ **Colorectal Cancer:** Treated with chemotherapy 5 years ago<br>○ **Chronic HCV Infection**<br>● **Nerve Conduction Studies:** Pure sensory, symmetric, length-dependent axonal polyneuropathy with unilateral carpal tunnel syndrome<br>● **Laboratory Findings:**<br>○ Normal: Vitamin B12, folate, vitamin B6, vitamin E, HBV, glycemia, HbA1c, ANA, ANCA, ENA, CRP, ESR, copper, cryoglobulins, and serum electrophoresis<br>○ Positive: HCV IgG

	HCV infection. Nerve conduction studies show a pure sensory, symmetric, length-dependent axonal polyneuropathy with unilateral carpal tunnel syndrome. Screening lab tests including vitamin B12, folate, vitamin B6, vitamin E, HBV, glycemia, HbA1c, ANA, ANCA, ENA, CRP, ESR, copper, cryoglobulins, and serum electrophoresis are normal, with positivity for HCV IgG. There is no family history of polyneuropathy.		● **Family History:** No family history of polyneuropathy<br>**Differential Diagnoses for Polyneuropathy**<br>1. **Hepatitis C Virus (HCV)-Related Neuropathy**<br>○ **Reasoning:**<br>■ **Chronic HCV Infection:** The patient has a documented history of chronic HCV infection.<br>■ **Sensory Polyneuropathy:** HCV infection can cause a sensory, length-dependent polyneuropathy, often due to cryoglobulinemia or direct viral effects.<br>■ **Cryoglobulins Normal:** Despite normal cryoglobulins, HCV can still contribute to neuropathy through other mechanisms, such as immune-mediated processes.<br>■ **Slow Progressive Course:** Consistent with the patient's presentation.<br>■ **Positivity for HCV IgG:** Supports the ongoing chronic infection.<br>2. **Chemotherapy-Induced Neuropathy**<br>○ **Reasoning:**<br>■ **History of Chemotherapy:** The patient underwent chemotherapy for colorectal cancer 5 years ago.<br>■ **Delayed Onset:** Chemotherapy-induced neuropathy typically presents shortly after treatment, but there can be a delayed progressive course.<br>■ **Pure Sensory Neuropathy:** Certain chemotherapeutic agents can cause pure sensory neuropathy.<br>■ **Symmetry and Length Dependence:** Characteristic of chemotherapy-induced neuropathy.<br>3. **Idiopathic Sensory Polyneuropathy**<br>○ **Reasoning:**<br>■ **Common in Elderly:** Idiopathic polyneuropathy is a frequent diagnosis in older adults when no specific cause is found.<br>■ **Slow Progressive Course:** Fits the description of the patient's symptoms.<br>■ **Negative Workup:** Extensive negative workup supports idiopathic nature after excluding other causes.<br>■ **No Family History:** Supports non-genetic origin.<br>**Most Likely Diagnosis**<br>The most likely diagnosis in this patient is **Hepatitis C Virus (HCV)-Related Neuropathy**. This conclusion is based on the chronic HCV infection, which is a known cause of sensory polyneuropathy, and the positive HCV IgG. Although chemotherapy-induced neuropathy and idiopathic polyneuropathy are also possible, the presence of chronic HCV infection with the clinical and electrodiagnostic findings strongly supports HCV-related neuropathy as the primary etiology.
3	**Case presentation:** A 75-year-old female patient presents with a 1-year history of sensory-motor, symmetrical, length dependent polyneuropathy with subacute onset. The patient presents with paresthesia and hypoesthesia in the lower limbs and sensory ataxia, without tremor, foot deformities, neuropathic pain, cranial nerve or autonomic involvement. Past medical history shows breast cancer, for which the patient has undergone chemotherapy. Neuropathy symptoms started during chemotherapy. Nerve conduction studies show a symmetric, sensory-motor, axonal polyneuropathy. Screening lab tests including vitamin B12, folate, vitamin B6, vitamin E, HCV, HBV, ANA, ANCA, ENA, CRP, ESR, glycemia, HbA1c, copper, cryoglobulins and serum electrophoresis are all negative. There is no family history of polyneuropathy.	Chemotherapy induced polyneuropathy	**Summary of Pertinent Clinical Information:**<br>● **Patient Profile**: 75-year-old female<br>● **Symptoms**:<br>○ 1-year history of sensory-motor, symmetrical, length-dependent polyneuropathy with subacute onset<br>○ Paresthesia and hypoesthesia in the lower limbs<br>○ Sensory ataxia<br>● **Absent Symptoms**:<br>○ No tremor<br>○ No foot deformities<br>○ No neuropathic pain<br>○ No cranial nerve or autonomic involvement<br>● **Past Medical History**:<br>○ Breast cancer treated with chemotherapy<br>○ Onset of neuropathy symptoms during chemotherapy<br>● **Diagnostic Findings**:<br>○ Nerve conduction studies: symmetric, sensory-motor, axonal polyneuropathy<br>○ Screening lab tests (vitamin B12, folate, vitamin B6, vitamin E, HCV, HBV, ANA, ANCA, ENA, CRP, ESR, glycemia, HbA1c, copper, cryoglobulins, and serum electrophoresis) all negative<br>● **Family History**: No family history of polyneuropathy<br>**Differential Diagnoses for Polyneuropathy:**<br>1. **Chemotherapy-Induced Peripheral Neuropathy (CIPN)**:<br>○ **Reasoning**: The patient's neuropathy symptoms began during chemotherapy treatment for breast cancer. Chemotherapy drugs, particularly those used in breast cancer treatment such as taxanes (e.g., paclitaxel) and platinum compounds (e.g., carboplatin), are well-known for causing peripheral neuropathy. The symmetrical, sensory-motor, axonal nature of the neuropathy is consistent with CIPN. The absence of neuropathic pain and other systemic involvements further supports this diagnosis as CIPN commonly presents with paresthesia, hypoesthesia, and sensory ataxia without affecting the cranial nerves or autonomic functions.<br>2. **Paraneoplastic Neuropathy**:<br>○ **Reasoning**: Although less likely given the timing of symptoms with chemotherapy, paraneoplastic neuropathy should still be considered. This condition can occur in association with malignancies like breast cancer. It is caused by an autoimmune response directed against neural tissues, triggered by the presence of the tumor. However, paraneoplastic neuropathy often presents with more varied symptoms, including autonomic involvement and sometimes cranial nerve involvement, which are absent in this case. Additionally, negative autoimmune markers (ANA, ANCA, ENA) make this diagnosis less probable.<br>3. **Idiopathic Axonal Polyneuropathy**:<br>○ **Reasoning**: In the absence of a clear secondary cause, idiopathic axonal polyneuropathy (IAP) is a diagnosis of exclusion. The patient's comprehensive screening labs were negative for common causes of neuropathy, including vitamin deficiencies, infections, autoimmune diseases, diabetes, and metabolic disorders. While IAP typically presents in older adults and can have a similar clinical presentation, the onset of symptoms during chemotherapy makes this less likely compared to CIPN.<br>**Most Likely Diagnosis:**<br>**Chemotherapy-Induced Peripheral Neuropathy (CIPN)** is the most likely diagnosis for this patient. The temporal correlation between the onset of neuropathy symptoms and the administration of chemotherapy, combined with the typical clinical presentation and negative results for other common causes of polyneuropathy, strongly supports CIPN as the primary cause of her symptoms.
4	**Case presentation:** An 81-year-old male patient presents with a 9-week history of sensory-motor, asymmetrical multineuropathy with subacute onset and	Vasculitic neuropathy	**Summary of Clinical Information**<br>**Patient:** 81-year-old male<br>**History of Present Illness:**<br>● Duration: 9 weeks

	progressive course. The patient presents with paresthesia, hypoesthesia, neuropathic pain, and weakness with a multifocal distribution. Clinical examination does not reveal sensory ataxia nor tremor, foot deformities, cranial nerve or autonomic involvement. Past medical history is positive for giant cell arteritis. Nerve conduction studies show a sensory-motor, axonal multineuropathy. Screening lab tests including vitamin B12, folate, vitamin B6, vitamin E, HBV, HCV, glycemia, HbA1c, ANA, ENA, copper, cryoglobulins, and serum electrophoresis are all negative, with evidence of elevated CRP and ESR. There is no family history of polyneuropathy.		● Onset: Subacute<br>● Course: Progressive<br>● Symptoms: Sensory-motor, asymmetrical multineuropathy<br>○ Paresthesia<br>○ Hypoesthesia<br>○ Neuropathic pain<br>○ Weakness<br>○ Multifocal distribution<br>**Clinical Examination:**<br>● No sensory ataxia<br>● No tremor<br>● No foot deformities<br>● No cranial nerve involvement<br>● No autonomic involvement<br>**Past Medical History:**<br>● Positive for giant cell arteritis<br>**Investigations:**<br>● **Nerve Conduction Studies:** Sensory-motor, axonal multineuropathy<br>● **Laboratory Tests:**<br>○ Negative for vitamin B12, folate, vitamin B6, vitamin E, HBV, HCV, glycemia, HbA1c, ANA, ENA, copper, cryoglobulins, and serum electrophoresis<br>○ Elevated CRP and ESR<br>**Family History:**<br>● No family history of polyneuropathy<br>**Differential Diagnoses for Polyneuropathy**<br>**1. Vasculitic Neuropathy**<br>**Reasoning:**<br>● The patient's history of giant cell arteritis, a type of vasculitis, is highly relevant. Vasculitis can cause inflammation of blood vessels supplying nerves, leading to ischemic damage and neuropathy.<br>● The subacute and progressive nature of the neuropathy aligns with vasculitic processes.<br>● Elevated CRP and ESR support ongoing inflammatory activity, which is consistent with vasculitis.<br>● The multifocal and asymmetrical distribution of neuropathy is typical for vasculitic neuropathy.<br>**2. Paraneoplastic Neuropathy**<br>**Reasoning:**<br>● Paraneoplastic syndromes can present with various neuropathic symptoms, often with a subacute onset and progressive course, similar to this patient's presentation.<br>● Although there is no direct evidence of malignancy in this case, the patient's age and lack of other specific findings make paraneoplastic neuropathy a consideration.<br>● Elevated inflammatory markers (CRP, ESR) can be seen in paraneoplastic syndromes.<br>● The multifocal nature without other specific neurological deficits might point towards a paraneoplastic etiology.<br>**3. Chronic Inflammatory Demyelinating Polyneuropathy (CIDP)**<br>**Reasoning:**<br>● CIDP typically presents with a more chronic course, but subacute variants exist, especially in older patients.<br>● The sensory-motor involvement and progressive nature are consistent with CIDP.<br>● Elevated inflammatory markers can be associated with CIDP, although they are not specific.<br>● The absence of sensory ataxia and tremor does not rule out CIDP, as presentations can vary.<br>● Multifocal distribution, although less common, can occur in CIDP.<br>**Most Likely Diagnosis**<br>**Vasculitic Neuropathy Reasoning:**<br>● The patient's history of giant cell arteritis is a significant risk factor for vasculitic neuropathy.<br>● The clinical presentation (subacute, progressive, asymmetrical, multifocal neuropathy) and elevated inflammatory markers (CRP, ESR) are highly suggestive of a vasculitic process.<br>● The lack of response to initial laboratory tests and the specific pattern seen in nerve conduction studies further support this diagnosis.<br>Given the alignment of clinical presentation, past medical history, and diagnostic findings, vasculitic neuropathy stands out as the most likely diagnosis. Further investigation, such as nerve biopsy and additional immunological studies, may be warranted to confirm the diagnosis and guide treatment.
5	A 61-year-old male patient presents with a 1-year history of sensory-motor, asymmetric, non-length dependent polyneuropathy with subacute onset and progressive course. The patient presents with asymmetric distal paresthesia and neuropathic pain distributed in the four limbs, associated with weakness in the four limbs both proximally and distally. Clinical examination also shows the presence of mild sensory ataxia, peripheral edema in the feet, and mild cutaneous hyperpigmentation in the lower limbs.	POEMS	**Step-by-Step Clinical Analysis:**<br>**1. Summarize Pertinent Clinical Information:**<br>**Patient Profile:**<br>● **Age:** 61 years old<br>● **Gender:** Male<br>● **History:** Psoriatic arthritis<br>**Neurological Symptoms:**<br>● **Duration:** 1 year<br>● **Type:** Sensory-motor, asymmetric, non-length dependent polyneuropathy<br>● **Onset:** Subacute, progressive<br>● **Symptoms:**<br>○ Asymmetric distal paresthesia<br>○ Neuropathic pain in all four limbs

	There are no autonomic symptoms or foot deformities. Past medical history is positive for psoriatic arthritis. Nerve conduction studies show a demyelinating sensory-motor polyneuropathy. Screening lab tests show presence of IgG lambda paraprotein and elevated CRP. Remaining exams, including vitamin B12, folate, vitamin B6, vitamin E, HBV, HCV, ANA, ENA, ANCA, ESR, glycemia, HbA1c, copper, and cryoglobulins are negative. There is no family history of polyneuropathy.		○ Weakness in both proximal and distal limbs<br>○ Mild sensory ataxia<br>○ Peripheral edema in feet<br>○ Mild cutaneous hyperpigmentation in lower limbs<br>● **No autonomic symptoms or foot deformities**<br>**Clinical Findings:**<br>● **Nerve conduction studies:** Demyelinating sensory-motor polyneuropathy<br>**Lab Findings:**<br>● **Positive:** IgG lambda paraprotein, elevated CRP<br>● **Negative:** Vitamin B12, folate, vitamin B6, vitamin E, HBV, HCV, ANA, ENA, ANCA, ESR, glycemia, HbA1c, copper, cryoglobulins<br>● **Family History:** No family history of polyneuropathy<br>**2. Differential Diagnoses for Polyneuropathy:**<br>**a) Monoclonal Gammopathy of Undetermined Significance (MGUS) / POEMS Syndrome:**<br>● **Reasoning:**<br>○ Presence of IgG lambda paraprotein suggests a monoclonal gammopathy.<br>○ Demyelinating pattern on nerve conduction studies aligns with neuropathy seen in MGUS and POEMS.<br>○ Peripheral edema and cutaneous hyperpigmentation are characteristic features of POEMS syndrome.<br>○ Elevated CRP could be associated with inflammatory processes seen in these conditions.<br>○ Psoriatic arthritis may predispose to systemic inflammatory states that could contribute to paraproteinemia and neuropathy.<br>**b) Chronic Inflammatory Demyelinating Polyneuropathy (CIDP):**<br>● **Reasoning:**<br>○ Progressive, asymmetric sensory-motor polyneuropathy is consistent with CIDP.<br>○ Demyelinating nature on nerve conduction studies supports this diagnosis.<br>○ Presence of elevated CRP may indicate ongoing inflammation.<br>○ CIDP can present with peripheral edema and, occasionally, with mild skin changes due to chronic inflammation.<br>**c) Multifocal Motor Neuropathy (MMN):**<br>● **Reasoning:**<br>○ Although less common in the absence of conduction block on nerve studies, MMN can present with asymmetric motor involvement.<br>○ Elevated CRP suggests an inflammatory process which is sometimes seen in MMN.<br>○ The clinical course and nerve conduction findings are more indicative of a demyelinating process rather than purely motor, but MMN remains a consideration.<br>**3. Most Likely Diagnosis:**<br>**Most Likely Diagnosis: POEMS Syndrome**<br>● **Reasoning:**<br>○ The presence of IgG lambda paraprotein points towards a monoclonal gammopathy.<br>○ The combination of demyelinating polyneuropathy, peripheral edema, and cutaneous hyperpigmentation strongly suggest POEMS syndrome.<br>○ Elevated CRP aligns with the inflammatory state often seen in POEMS.<br>○ The lack of autonomic symptoms and other systemic features typically seen in other diagnoses make POEMS more likely.<br>**Conclusion:**<br>Based on the provided clinical scenario, the most likely diagnosis for the patient is POEMS syndrome, considering the constellation of clinical, electrophysiological, and laboratory findings, specifically the presence of IgG lambda paraprotein, demyelinating polyneuropathy, peripheral edema, and cutaneous changes. Further specific diagnostic investigations, including imaging and biopsy, might be necessary to confirm this diagnosis and guide appropriate management.
6	A 73-year-old female patient presents with an 8-month history of pure sensory, symmetrical, length dependent polyneuropathy with subacute onset and progressive course. The patient presents with distal paresthesia in the upper and lower limbs without weakness or neuropathic pain. Clinical examination reveals reduced tactile sensation in the feet. There are no tremor or ataxia, foot deformities, cranial nerve or autonomic involvement. Past medical history is positive for episodic diarrhea. Nerve conduction studies show a pure sensory, symmetric, length dependent, axonal polyneuropathy. Screening lab tests reveal the presence of vitamin B12 deficiency, hyperglycaemia, and elevated HbA1c. Remaining exams including folate, vitamin B6, vitamin E, HCV, HBV, ANA, ENA, ANCA, CRP, ESR, copper, cryoglobulins and, serum electrophoresis are negative. There is no family history of polyneuropathy.	Vit B12 deficiency neuropathy	**Clinical Summary**<br>A 73-year-old female patient presents with:<br>● **Symptoms**: 8-month history of pure sensory, symmetrical, length-dependent polyneuropathy with subacute onset and progressive course. Distal paresthesia in upper and lower limbs without weakness or neuropathic pain.<br>● **Examination Findings**: Reduced tactile sensation in the feet. No tremor, ataxia, foot deformities, cranial nerve, or autonomic involvement.<br>● **Past Medical History**: Episodic diarrhea.<br>● **Nerve Conduction Studies**: Pure sensory, symmetric, length-dependent, axonal polyneuropathy.<br>● **Screening Lab Tests**:<br>○ Positive: Vitamin B12 deficiency, hyperglycemia, elevated HbA1c.<br>○ Negative: Folate, vitamin B6, vitamin E, HCV, HBV, ANA, ENA, ANCA, CRP, ESR, copper, cryoglobulins, serum electrophoresis.<br>● **Family History**: No family history of polyneuropathy.<br>**Differential Diagnoses**<br>1. **Diabetic Polyneuropathy**<br>2. **Vitamin B12 Deficiency Neuropathy**<br>3. **Celiac Disease-Associated Neuropathy**<br>**Detailed Reasoning for Differential Diagnoses**<br>1. **Diabetic Polyneuropathy**<br>○ **Reasoning**:<br>■ **Hyperglycemia and Elevated HbA1c**: These findings indicate poor glycemic control, which is a well-known risk factor for developing diabetic polyneuropathy.<br>■ **Symmetrical, Length-Dependent Polyneuropathy**: This pattern is characteristic of diabetic neuropathy, which often presents with distal sensory loss.<br>■ **Absence of Weakness or Neuropathic Pain**: Diabetic polyneuropathy can present as a pure sensory neuropathy without motor involvement or pain, especially in early stages.<br>○ **Supporting Evidence**: Presence of hyperglycemia and elevated HbA1c, absence of other systemic involvement or alternative causes.<br>2. **Vitamin B12 Deficiency Neuropathy**<br>○ **Reasoning**:

			■ **Vitamin B12 Deficiency**: The patient's lab results show a deficiency in vitamin B12, which is known to cause subacute combined degeneration of the spinal cord and peripheral neuropathy.<br>■ **Symmetrical, Sensory Neuropathy**: Vitamin B12 deficiency typically affects the dorsal columns, leading to sensory deficits, often in a symmetrical pattern.<br>■ **Absence of Motor Symptoms**: While B12 deficiency can cause motor symptoms, it can also present with isolated sensory deficits.<br>○ **Supporting Evidence**: Identified vitamin B12 deficiency, compatible clinical presentation, and lack of other potential causes.<br>3. **Celiac Disease-Associated Neuropathy**<br>○ **Reasoning**:<br>■ **Episodic Diarrhea**: This history raises suspicion for celiac disease, which is associated with malabsorption and secondary neuropathy.<br>■ **Vitamin Deficiency**: Although other vitamin deficiencies (like folate or vitamin E) were not found, celiac disease can cause selective malabsorption.<br>■ **Pure Sensory Neuropathy**: Celiac disease-associated neuropathy often presents as a sensory neuropathy without motor involvement.<br>○ **Supporting Evidence**: Episodic diarrhea suggesting potential malabsorption, compatible neuropathic pattern, absence of other causes in the context of unexplained gastrointestinal symptoms.<br>**Most Likely Diagnosis**<br>**Diabetic Polyneuropathy**<br>● **Primary Reason**: The presence of hyperglycemia and elevated HbA1c strongly suggests diabetes as an underlying cause of the polyneuropathy.<br>● **Consistency with Clinical Presentation**: The pure sensory, symmetrical, length-dependent pattern without motor involvement is typical for diabetic neuropathy.<br>● **Supporting Lab Findings**: Elevated glucose levels and HbA1c directly correlate with the development of diabetic neuropathy.<br>● **Absence of Compelling Evidence for Other Causes**: While vitamin B12 deficiency and celiac disease are possible, the clinical and lab findings align more directly with diabetic polyneuropathy, making it the most probable diagnosis given the provided information.
7	A 70-year-old male patient presents with a 5-week history of pure sensory, symmetrical, length dependent polyneuropathy with subacute onset and progressive course. The patient presents with distal paresthesia and hypoesthesia in the four limbs and sensory ataxia. Clinical examination does not show weakness, tremor, foot deformities, autonomic or cranial nerve involvement. Past medical history is positive for obesity treated with bariatric surgery, anemia, hypercholesterolemia, and type 2 diabetes mellitus. Nerve conduction studies show a pure sensory, symmetric, axonal polyneuropathy. Screening lab tests shows slightly elevated glycemia and HbA1c levels, and severe folate deficiency. Remaining exams including vitamin B12, E, HBV, HCV, ANA, ANCA, ENA, CRP, ESR, copper, cryoglobulins, and serum electrophoresis are normal. There is no family history of polyneuropathy.	Folate deficiency neuropathy	**Summary of Clinical Information**<br>● **Patient**: 70-year-old male<br>● **History**:<br>○ 5-week history of pure sensory, symmetrical, length-dependent polyneuropathy with subacute onset and progressive course<br>○ Distal paresthesia and hypoesthesia in all four limbs<br>○ Sensory ataxia<br>● **Clinical Examination**:<br>○ No weakness, tremor, foot deformities<br>○ No autonomic or cranial nerve involvement<br>● **Past Medical History**:<br>○ Obesity (treated with bariatric surgery)<br>○ Anemia<br>○ Hypercholesterolemia<br>○ Type 2 diabetes mellitus<br>● **Nerve Conduction Studies**: Pure sensory, symmetric, axonal polyneuropathy<br>● **Lab Results**:<br>○ Slightly elevated glycemia and HbA1c levels<br>○ Severe folate deficiency<br>○ Normal levels of vitamin B12, vitamin E, HBV, HCV, ANA, ANCA, ENA, CRP, ESR, copper, cryoglobulins, and serum electrophoresis<br>● **Family History**: No family history of polyneuropathy<br>**Differential Diagnoses for Polyneuropathy**<br>**1. Diabetic Neuropathy**<br>**Reasoning**:<br>● **Clinical Features**: Diabetic neuropathy typically presents as a length-dependent, symmetrical polyneuropathy affecting the distal extremities with sensory symptoms such as paresthesia and hypoesthesia, consistent with the patient's presentation.<br>● **Past Medical History**: The patient has a history of type 2 diabetes mellitus, which is a common cause of peripheral neuropathy.<br>● **Nerve Conduction Studies**: The pure sensory, symmetric, axonal pattern aligns with typical findings in diabetic neuropathy.<br>● **Lab Findings**: Slightly elevated glycemia and HbA1c levels indicate suboptimal glucose control, which can exacerbate diabetic neuropathy.<br>**2. Folate Deficiency Neuropathy**<br>**Reasoning**:<br>● **Clinical Features**: Folate deficiency can cause a subacute onset of peripheral neuropathy with sensory symptoms, which matches the patient's profile.<br>● **Lab Findings**: The patient has severe folate deficiency, which is a known cause of axonal polyneuropathy.<br>● **Past Medical History**: History of anemia and bariatric surgery (which can lead to malabsorption and deficiencies) supports the possibility of a nutritional deficiency.<br>**3. Chronic Idiopathic Axonal Polyneuropathy (CIAP)**<br>**Reasoning**:<br>● **Clinical Features**: CIAP often presents in elderly patients with a subacute or chronic course of sensory symptoms affecting the distal limbs symmetrically, similar to this patient's symptoms.<br>● **Nerve Conduction Studies**: The findings of a pure sensory, symmetric, axonal polyneuropathy are consistent with CIAP.<br>● **Exclusion**: The normal results for various other tests (vitamin B12, HBV, HCV, ANA, etc.) make idiopathic causes more plausible after excluding other common etiologies.<br>**Most Likely Diagnosis**<br>The most likely diagnosis for this patient is **Folate Deficiency Neuropathy**. This conclusion is based on the severe folate deficiency identified in the lab results, the history of bariatric surgery (which can lead to malabsorption and nutritional deficiencies), and the clinical presentation that fits well with neuropathy caused by folate deficiency. While diabetic neuropathy is also a strong contender given the patient's history of diabetes, the severity of the folate deficiency makes it the more immediate and treatable cause of the patient's symptoms.
8	A 74-year-old male patient presents with a 9-month history of pure sensory, symmetrical, length dependent	Chemotherapy-induced neuropathy	**Clinical Summary**<br>● **Patient**: 74-year-old male<br>● **History**:

	polyneuropathy with subacute onset while hospitalised for lymphoma. The patient developed neuropathic pain in the feet with no sensory deficits or weakness. Clinical examination reveals absence of ataxia, tremor, foot deformities, cranial nerve or autonomic involvement. Past medical history is positive for diabetes since the age of 43, and lymphoma for which the patient has undergone chemotherapy. Neuropathy symptoms started during chemotherapy. Nerve conduction studies show a sensory-motor, symmetric, axonal polyneuropathy. Screening lab tests including vitamin B12, folate, vitamin B6, vitamin E, HCV, ANA, ANCA, ENA, CRP, ESR, copper, cryoglobulins and serum electrophoresis are all negative. HbA1c and glycemia levels are elevated and there is evidence of chronic HBV infection. There is no family history of polyneuropathy.		○ 9-month history of pure sensory, symmetrical, length-dependent polyneuropathy with subacute onset during hospitalization for lymphoma.<br>○ Neuropathic pain in feet, no sensory deficits or weakness.<br>● **Clinical Findings**:<br>○ Absence of ataxia, tremor, foot deformities, cranial nerve or autonomic involvement.<br>● **Past Medical History**:<br>○ Diabetes since age 43.<br>○ Lymphoma, treated with chemotherapy.<br>● **Nerve Conduction Studies**:<br>○ Sensory-motor, symmetric, axonal polyneuropathy.<br>● **Laboratory Tests**:<br>○ Vitamin B12, folate, vitamin B6, vitamin E, HCV, ANA, ANCA, ENA, CRP, ESR, copper, cryoglobulins, serum electrophoresis: all negative.<br>○ Elevated HbA1c and glycemia levels.<br>○ Chronic HBV infection.<br>● **Family History**:<br>○ No family history of polyneuropathy.<br>**Differential Diagnoses for Polyneuropathy**<br>1. **Chemotherapy-Induced Peripheral Neuropathy (CIPN)**<br>○ **Reasoning**:<br>■ The onset of neuropathy symptoms coincided with the period of chemotherapy for lymphoma.<br>■ Chemotherapeutic agents are known to cause peripheral neuropathy, especially those used in lymphoma treatment like platinum-based drugs, taxanes, and vinca alkaloids.<br>■ Sensory symptoms without motor involvement initially are common in CIPN.<br>■ CIPN often presents with pain and can have a symmetrical, length-dependent pattern.<br>2. **Diabetic Polyneuropathy**<br>○ **Reasoning**:<br>■ Long-standing history of diabetes with poor glycemic control as indicated by elevated HbA1c and glycemia levels.<br>■ Diabetic neuropathy typically presents as a symmetric, length-dependent polyneuropathy, primarily affecting sensory nerves first.<br>■ The chronic hyperglycemia associated with diabetes can lead to microvascular damage affecting peripheral nerves.<br>■ The absence of autonomic involvement does not exclude diabetic neuropathy, as it can vary in presentation.<br>3. **Chronic Inflammatory Demyelinating Polyneuropathy (CIDP)**<br>○ **Reasoning**:<br>■ CIDP can present with a sensory-motor polyneuropathy.<br>■ Subacute onset aligns with CIDP, though it usually has a more insidious onset compared to acute inflammatory demyelinating polyneuropathy (AIDP).<br>■ Although nerve conduction studies show axonal rather than demyelinating changes, some CIDP variants can present with axonal features.<br>■ Negative screening tests do not rule out CIDP, as it is primarily diagnosed based on clinical presentation and electrophysiological findings.<br>**Most Likely Diagnosis**<br>**Chemotherapy-Induced Peripheral Neuropathy (CIPN)**<br>● **Rationale**:<br>○ The temporal relationship between the onset of neuropathy and chemotherapy initiation strongly suggests CIPN.<br>○ The pattern of pure sensory, symmetrical, length-dependent polyneuropathy without motor involvement fits well with CIPN.<br>○ Although diabetic neuropathy is also a possibility given the patient's history of diabetes, the recent onset during chemotherapy and the nature of symptoms point more convincingly towards CIPN.<br>○ CIDP is less likely given the lack of characteristic demyelinating features on nerve conduction studies and the timing of symptoms relative to chemotherapy.<br>**Conclusion**<br>The most likely diagnosis for this 74-year-old male with the described clinical scenario is **Chemotherapy-Induced Peripheral Neuropathy (CIPN)**. This conclusion is based on the subacute onset of symptoms during chemotherapy, the typical presentation of CIPN, and the supportive nerve conduction study findings. While diabetic neuropathy remains a consideration, the clinical context and timing make CIPN the more probable diagnosis.
9	A 77-year-old female patient presents with a 3-week history of pure sensory, symmetrical, length dependent polyneuropathy with subacute onset and progressive course. The patient presents with hypoesthesia and mild neuropathic pain in the feet. Clinical examination reveals also mild sensory ataxia. There are no tremor, foot deformities, cranial nerve or autonomic involvement. Past medical history is negative. Nerve conduction studies show a pure sensory, length dependent, symmetric, axonal polyneuropathy. Screening lab tests reveal decreased serum level of vitamin B6 and a slight increase in transaminase levels (aspartate aminotransferase/alanine aminotransferase). Remaining exams including vitamin B12, folate, vitamin E,	B6 deficiency neuropathy	**Summary of Clinical Information**<br>**Patient Demographics:**<br>● Age: 77 years old<br>● Gender: Female<br>**Presenting Symptoms:**<br>● 3-week history of pure sensory, symmetrical, length-dependent polyneuropathy<br>● Subacute onset and progressive course<br>● Hypoesthesia and mild neuropathic pain in the feet<br>● Mild sensory ataxia<br>● No tremor, foot deformities, cranial nerve or autonomic involvement<br>**Clinical Findings:**<br>● Nerve conduction studies: Pure sensory, length-dependent, symmetric, axonal polyneuropathy<br>**Laboratory Findings:**<br>● Decreased serum level of vitamin B6<br>● Slight increase in transaminase levels (aspartate aminotransferase/alanine aminotransferase)<br>● Negative for: vitamin B12, folate, vitamin E, HBV, HCV, glycemia, HbA1c, ANA, ANCA, ENA, CRP, ESR, copper, cryoglobulins, serum electrophoresis<br>**Past Medical History:**<br>● Negative<br>**Family History:**

	HBV, HCV, glycemia, HbA1c, ANA, ANCA, ENA, CRP, ESR, copper, cryoglobulins, and serum electrophoresis are negative. There is no family history of polyneuropathy.		● No family history of polyneuropathy<br>**Differential Diagnoses for Polyneuropathy**<br>1. **Vitamin B6 Deficiency Neuropathy**<br>○ **Reasoning:** The patient has a documented deficiency in vitamin B6, which is known to cause sensory neuropathy. Vitamin B6 is essential for nerve health, and its deficiency can lead to nerve damage presenting as sensory neuropathy. The absence of other significant findings that could explain the neuropathy supports this diagnosis.<br>○ **Supporting Evidence:** Pure sensory, length-dependent, symmetric, axonal polyneuropathy on nerve conduction studies and the absence of other abnormalities that could account for the neuropathy.<br>2. **Chronic Liver Disease-Related Neuropathy**<br>○ **Reasoning:** Slightly elevated transaminase levels might suggest liver dysfunction, which can be associated with peripheral neuropathy. Chronic liver disease can cause metabolic disturbances leading to nerve damage.<br>○ **Supporting Evidence:** Elevated liver enzymes and the absence of other common causes of neuropathy. However, the mild increase in transaminases without other signs of liver disease makes this less likely but still a consideration.<br>3. **Idiopathic Sensory Neuropathy**<br>○ **Reasoning:** In elderly patients, it is not uncommon to find cases of idiopathic sensory neuropathy where no definitive cause is identified despite extensive workup. The pure sensory nature of the neuropathy, negative past medical and family history, and negative extensive laboratory workup point towards this possibility.<br>○ **Supporting Evidence:** Negative screening tests for common causes of neuropathy and the clinical presentation of a pure sensory, symmetric, length-dependent polyneuropathy.<br>**Most Likely Diagnosis**<br>**Vitamin B6 Deficiency Neuropathy**<br>**Reasoning:** The presence of a documented vitamin B6 deficiency in the context of a pure sensory, length-dependent, symmetric, axonal polyneuropathy strongly supports vitamin B6 deficiency neuropathy as the most likely diagnosis. The clinical presentation aligns with what is expected in vitamin B6 deficiency neuropathy, and correction of the deficiency should be considered as part of the management plan to see if there is an improvement in symptoms.<br>**Conclusion:** Based on the clinical presentation, nerve conduction studies, and laboratory findings, vitamin B6 deficiency neuropathy is the most likely diagnosis. Further management should include vitamin B6 supplementation and monitoring of neuropathic symptoms for improvement.
10	A 76-year-old female patient presents with a 2-year history of sensory-motor, symmetrical, length dependent polyneuropathy with subacute onset. The patient presents with paresthesia, neuropathic pain, cramps, orthostatic hypotension, and weakness of toe dorsiflexion. There are no foot deformities, cranial nerve involvement, ataxia, or tremor. Past medical history is positive for chronic renal insufficiency and multiple myeloma, for which the patient has undergone chemotherapy. Neuropathy symptoms started at the end of the chemotherapy treatment cycles. Nerve conduction studies show a symmetric, pure sensory, axonal polyneuropathy and unilateral carpal tunnel syndrome. Screening lab tests including vitamin B12, folate, vitamin B6, vitamin E, HBV, HCV, ANA, ANCA, ENA, CRP, ESR, glycemia, HbA1c, copper and cryoglobulins are all negative, with positivity for IgG kappa paraprotein. There is no family history of polyneuropathy.	Chemotherapy-induced neuropathy	**Summary of Pertinent Clinical Information:**<br>● **Patient Demographics**: 76-year-old female<br>● **History**: 2-year history of sensory-motor, symmetrical, length-dependent polyneuropathy with subacute onset.<br>● **Symptoms**:<br>○ Paresthesia<br>○ Neuropathic pain<br>○ Cramps<br>○ Orthostatic hypotension<br>○ Weakness of toe dorsiflexion<br>● **Absent Symptoms**: No foot deformities, cranial nerve involvement, ataxia, or tremor.<br>● **Past Medical History**:<br>○ Chronic renal insufficiency<br>○ Multiple myeloma (treated with chemotherapy)<br>● **Onset of Neuropathy Symptoms**: Post-chemotherapy<br>● **Nerve Conduction Studies**: Symmetric, pure sensory, axonal polyneuropathy and unilateral carpal tunnel syndrome.<br>● **Lab Tests**:<br>○ Negative: Vitamin B12, folate, vitamin B6, vitamin E, HBV, HCV, ANA, ANCA, ENA, CRP, ESR, glycemia, HbA1c, copper, and cryoglobulins<br>○ Positive: IgG kappa paraprotein<br>● **Family History**: No family history of polyneuropathy<br>**Differential Diagnoses for Polyneuropathy:**<br>**1. Chemotherapy-Induced Peripheral Neuropathy (CIPN)**<br>● **Reasoning**:<br>○ The onset of neuropathy symptoms coincided with the end of chemotherapy cycles, suggesting a temporal relationship.<br>○ Chemotherapy agents, especially those used to treat multiple myeloma (such as thalidomide, bortezomib, or vincristine), are well-known causes of peripheral neuropathy.<br>○ Symptoms like paresthesia, neuropathic pain, and motor involvement (weakness of toe dorsiflexion) are typical in CIPN.<br>○ Axonal polyneuropathy is commonly seen in CIPN.<br>**2. Monoclonal Gammopathy of Undetermined Significance (MGUS) with Associated Neuropathy**<br>● **Reasoning**:<br>○ The presence of IgG kappa paraprotein indicates a monoclonal gammopathy.<br>○ MGUS can be associated with peripheral neuropathy, especially axonal types.<br>○ Chronic renal insufficiency and multiple myeloma increase the likelihood of MGUS.<br>○ Neuropathy in MGUS can present as symmetric, sensory-motor, and length-dependent, fitting the patient's profile.<br>**3. Chronic Renal Insufficiency-Associated Neuropathy (Uremic Neuropathy)**<br>● **Reasoning**:<br>○ Chronic renal insufficiency is a significant risk factor for peripheral neuropathy.<br>○ Uremic neuropathy often presents as a symmetric, length-dependent, sensory-motor polyneuropathy.<br>○ Symptoms such as cramps and orthostatic hypotension can be seen in uremic neuropathy.<br>○ However, the pure sensory axonal findings on nerve conduction studies are less typical for uremic neuropathy, which often has mixed sensory-motor involvement.<br>**Most Likely Diagnosis:**<br>Based on the detailed clinical information and the temporal relationship between the onset of symptoms and chemotherapy treatment, **Chemotherapy-Induced Peripheral Neuropathy (CIPN)** is the most likely diagnosis. The pure sensory axonal polyneuropathy seen on nerve conduction studies, combined with the absence of other common causes and the patient's history of chemotherapy for multiple myeloma, strongly supports CIPN as the primary diagnosis. The presence of IgG kappa paraprotein suggests possible MGUS, which can also contribute to the neuropathy, but the direct correlation with chemotherapy timing makes CIPN the more likely primary cause.

11	A 76-year-old female patient presents with a 1-year history of sensory-motor multineuropathy with subacute onset. The patient presents with paresthesia and weakness in a multifocal distribution, without ataxia, neuropathic pain, tremor, foot deformities, cranial nerve or autonomic involvement. Past medical history shows diabetes, anaemia, chronic renal insufficiency, undifferentiated connective tissue disease, osteoarthritis and ANCA vasculitis. Nerve conduction studies show a sensory-motor, axonal multineuropathy. Screening lab tests including vitamin B12, folate, vitamin B6, vitamin E, HCV, HBV, ENA, CRP, ESR, glycemia, HbA1c, copper, cryoglobulins and serum electrophoresis are all negative, with positivity for ANA and ANCA autoantibodies. There is no family history of polyneuropathy.	Vasculitic neuropathy	**Summary of Pertinent Clinical Information:**<br>● **Patient:** 76-year-old female<br>● **Symptoms:**<br>○ 1-year history of sensory-motor multineuropathy with subacute onset<br>○ Paresthesia and weakness in a multifocal distribution<br>○ No ataxia, neuropathic pain, tremor, foot deformities, cranial nerve, or autonomic involvement<br>● **Medical History:**<br>○ Diabetes<br>○ Anaemia<br>○ Chronic renal insufficiency<br>○ Undifferentiated connective tissue disease<br>○ Osteoarthritis<br>○ ANCA vasculitis<br>● **Nerve Conduction Studies:** Sensory-motor, axonal multineuropathy<br>● **Lab Tests:**<br>○ Negative: Vitamin B12, folate, vitamin B6, vitamin E, HCV, HBV, ENA, CRP, ESR, glycemia, HbA1c, copper, cryoglobulins, serum electrophoresis<br>○ Positive: ANA and ANCA autoantibodies<br>● **Family History:** No family history of polyneuropathy<br>**Differential Diagnoses for Polyneuropathy:**<br>**1. Diabetic Neuropathy**<br>**Reasoning:**<br>● **Medical History:** The patient has a history of diabetes, which is a common cause of peripheral neuropathy.<br>● **Symptoms:** Diabetic neuropathy often presents with sensory and motor deficits and can affect multiple nerve areas.<br>● **Nerve Conduction Studies:** Sensory-motor, axonal involvement is consistent with diabetic neuropathy.<br>● **Glycemia and HbA1c:** Despite normal current glycemia and HbA1c, long-term diabetes can still result in neuropathy.<br>**2. Vasculitic Neuropathy (due to ANCA Vasculitis)**<br>**Reasoning:**<br>● **Medical History:** ANCA vasculitis can lead to nerve damage due to inflammation of the blood vessels supplying the nerves.<br>● **Symptoms:** Multineuropathy with sensory and motor involvement fits the pattern of vasculitic neuropathy.<br>● **Lab Findings:** Positivity for ANCA autoantibodies supports this diagnosis.<br>● **Onset:** Subacute onset is common in vasculitic neuropathy.<br>● **Nerve Conduction Studies:** Axonal damage is characteristic of vasculitic neuropathy.<br>**3. Connective Tissue Disease-Related Neuropathy**<br>**Reasoning:**<br>● **Medical History:** The patient has undifferentiated connective tissue disease, which can be associated with peripheral neuropathy.<br>● **Symptoms:** Multineuropathy can be seen in connective tissue diseases due to immune-mediated nerve damage.<br>● **Lab Findings:** Positive ANA autoantibodies are consistent with connective tissue diseases.<br>● **Onset and Nerve Conduction Studies:** The subacute onset and axonal damage align with neuropathy seen in connective tissue diseases.<br>**Most Likely Diagnosis:**<br>Given the patient's medical history, lab findings, and clinical presentation, the most likely diagnosis is **Vasculitic Neuropathy due to ANCA Vasculitis**. This is supported by the presence of ANCA autoantibodies, the subacute onset of symptoms, and the multifocal distribution of sensory-motor deficits without other common diabetic complications such as retinopathy or nephropathy exacerbations. The history of ANCA vasculitis further strengthens this diagnosis, making it the most plausible cause of the patient's polyneuropathy.
12	An 18-year old male patient presents with a 16-year history of sensory-motor, symmetrical, length dependent polyneuropathy with insidious onset and slow progression. The patient presents with sensory ataxia, distal weakness in the lower limbs, severe calf atrophy, pes planus, and hammertoes with no cranial nerve or autonomic involvement. Past medical history is silent. Nerve conduction studies show an axonal, sensory-motor polyneuropathy. Screening lab tests including vitamin B12, folate, vitamin B6, vitamin E, HBV, HCV, glycemia, HbA1c, ANA, ANCA, ENA, CRP, ESR, copper, cryoglobulins, and serum electrophoresis are all negative. Her mother and grandmother have walking difficulties. Her 5-year-old sister has pes cavus.	CMT2	**Summary of Clinical Information:**<br>● **Patient:** 18-year-old male<br>● **History:** 16-year history of sensory-motor, symmetrical, length-dependent polyneuropathy with insidious onset and slow progression.<br>● **Symptoms:**<br>○ Sensory ataxia<br>○ Distal weakness in the lower limbs<br>○ Severe calf atrophy<br>○ Pes planus (flat feet)<br>○ Hammertoes<br>● **Physical Examination:** No cranial nerve or autonomic involvement.<br>● **Nerve Conduction Studies:** Axonal, sensory-motor polyneuropathy.<br>● **Screening Lab Tests:** All negative (including vitamins B12, B6, folate, vitamin E, HBV, HCV, glycemia, HbA1c, ANA, ANCA, ENA, CRP, ESR, copper, cryoglobulins, and serum electrophoresis).<br>● **Family History:**<br>○ Mother and grandmother have walking difficulties.<br>○ 5-year-old sister has pes cavus (high arched feet).<br>**Differential Diagnoses for Polyneuropathy:**<br>**1. Charcot-Marie-Tooth Disease (CMT)**<br>● **Reasoning:**<br>○ **Clinical Presentation:** CMT typically presents with a chronic, progressive, length-dependent sensory-motor polyneuropathy, similar to the patient's symptoms of distal weakness, sensory ataxia, calf atrophy, pes planus, and hammertoes.

			○ **Family History:** The hereditary nature of CMT fits the family history of walking difficulties in the mother and grandmother and pes cavus in the sister.<br>○ **Nerve Conduction Studies:** The axonal type seen in the patient's studies can be consistent with certain types of CMT, particularly CMT2, which is predominantly axonal.<br>**2. Hereditary Sensory and Autonomic Neuropathy (HSAN)**<br>● **Reasoning:**<br>○ **Clinical Presentation:** Although HSAN primarily affects sensory nerves, it can present with sensory ataxia and distal weakness. However, the absence of autonomic involvement and the predominantly motor symptoms make this less likely.<br>○ **Family History:** HSAN can be inherited, which aligns with the familial pattern seen in this patient's family.<br>○ **Nerve Conduction Studies:** HSAN can show an axonal sensory neuropathy, but the involvement of motor nerves makes it less typical.<br>**3. Friedreich's Ataxia**<br>● **Reasoning:**<br>○ **Clinical Presentation:** Friedreich's Ataxia can present with sensory ataxia, distal weakness, and lower limb atrophy. Pes cavus is also a common finding. However, it typically presents with other systemic features such as cardiomyopathy, scoliosis, and diabetes, which are not mentioned in this case.<br>○ **Family History:** This disease is inherited in an autosomal recessive manner, which could fit the family history, though not as directly as CMT.<br>○ **Nerve Conduction Studies:** Typically show sensory neuropathy, and although motor involvement can occur, it is less characteristic.<br>**Most Likely Diagnosis:**<br>**Charcot-Marie-Tooth Disease (CMT)**<br>● **Reasoning:** CMT is the most likely diagnosis due to the combination of the patient's chronic, progressive sensory-motor polyneuropathy, the specific physical manifestations (pes planus, hammertoes, calf atrophy), and the strong family history of similar symptoms. The axonal type of neuropathy seen in nerve conduction studies is also consistent with CMT2. The absence of cranial nerve or autonomic involvement further supports this diagnosis, as CMT typically does not involve these systems.
13	An 82-year-old female patient presents with a 1-year history of sensory-motor, symmetrical, length dependent polyneuropathy with subacute onset and progressive course. The patient presents with paraesthesia and neuropathic pain in the hands and feet and bilateral foot drop. Clinical examination excludes sensory ataxia, cranial nerve or autonomic involvement, tremor or foot deformities. Past medical history is negative. Nerve conduction studies show a demyelinating, sensory-motor, symmetric polyneuropathy. Screening lab tests reveal presence of polycythaemia, IgG lambda paraprotein, positivity for ANA, hyperglycemia, and elevated HbA1c. Remaining exams including vitamin B12, folate, vitamin B6, vitamin E, ANCA, CRP, ESR, copper, and cryoglobulins are negative. There is no family history of polyneuropathy.	POEMS	**Summary of Pertinent Clinical Information**<br>**Patient Profile:**<br>● **Age/Sex:** 82-year-old female<br>● **Presentation:** 1-year history of sensory-motor, symmetrical, length-dependent polyneuropathy<br>● **Onset:** Subacute<br>● **Course:** Progressive<br>**Symptoms:**<br>● Paraesthesia and neuropathic pain in hands and feet<br>● Bilateral foot drop<br>**Clinical Examination:**<br>● Excludes sensory ataxia<br>● No cranial nerve involvement<br>● No autonomic involvement<br>● No tremor or foot deformities<br>**Past Medical History:** Negative<br>**Nerve Conduction Studies:**<br>● Demyelinating, sensory-motor, symmetric polyneuropathy<br>**Lab Findings:**<br>● Presence of polycythaemia<br>● IgG lambda paraprotein<br>● Positive ANA (antinuclear antibodies)<br>● Hyperglycemia<br>● Elevated HbA1c<br>**Negative Tests:**<br>● Vitamin B12, folate, vitamin B6, vitamin E<br>● ANCA, CRP, ESR<br>● Copper, cryoglobulins<br>**Family History:** No family history of polyneuropathy<br>**Differential Diagnoses**<br>1. **Chronic Inflammatory Demyelinating Polyneuropathy (CIDP)**<br>2. **Monoclonal Gammopathy of Undetermined Significance (MGUS) associated Polyneuropathy**<br>3. **Diabetic Polyneuropathy**<br>**Detailed Reasoning for Each Diagnosis**<br>**1. Chronic Inflammatory Demyelinating Polyneuropathy (CIDP)**<br>**Reasoning:**<br>● **Symptoms and Course:** CIDP often presents with a subacute to chronic course of progressive sensory-motor polyneuropathy, which fits the patient's presentation.<br>● **Nerve Conduction Studies:** The demyelinating nature observed in the nerve conduction studies is a key characteristic of CIDP.<br>● **ANA Positivity:** While ANA is not specific to CIDP, autoimmune markers can sometimes be seen in CIDP patients.<br>● **Exclusion of Other Causes:** The negative family history, absence of vitamin deficiencies, and normal copper levels further support the diagnosis of CIDP.<br>**2. Monoclonal Gammopathy of Undetermined Significance (MGUS) associated Polyneuropathy**<br>**Reasoning:**<br>● **IgG Lambda Paraprotein:** The presence of an IgG lambda paraprotein suggests a potential underlying MGUS, which is a known cause of polyneuropathy.<br>● **Demyelinating Polyneuropathy:** MGUS can lead to a demyelinating type of polyneuropathy, consistent with the nerve conduction study findings.<br>● **Polycythaemia:** While not directly linked, polycythaemia could be secondary to underlying plasma cell dyscrasia or other hematologic conditions associated with MGUS.

			● **Absence of Other Causes:** The lack of other common causes such as diabetes or vitamin deficiencies further suggests MGUS as a likely cause.<br>**3. Diabetic Polyneuropathy**<br>**Reasoning:**<br>● **Hyperglycemia and Elevated HbA1c:** These findings strongly indicate poor glycemic control, which is a primary risk factor for diabetic neuropathy.<br>● **Symmetrical, Length-Dependent Polyneuropathy:** The presentation is characteristic of diabetic polyneuropathy, which typically affects the distal extremities first.<br>● **Sensory-Motor Involvement:** Diabetic neuropathy can present with both sensory and motor symptoms, including neuropathic pain and motor deficits like foot drop.<br>● **Age:** Older age is a risk factor for developing diabetic neuropathy, especially in the context of long-standing hyperglycemia.<br>**Most Likely Diagnosis**<br>The most likely diagnosis for this patient is **Diabetic Polyneuropathy**. The presence of significant hyperglycemia and elevated HbA1c are key indicators of diabetes mellitus, a well-known cause of peripheral neuropathy. Despite the presence of an IgG lambda paraprotein, which suggests MGUS, diabetic polyneuropathy is a more straightforward and common explanation for the patient's presentation given the strong correlation between diabetes and peripheral neuropathy. The demyelinating nature of the neuropathy does not rule out diabetes, as it can present with various types of nerve damage. The polycythaemia may require further investigation but does not immediately point to an alternative diagnosis over diabetic polyneuropathy.
14	A 22-year old male patient presents with a 20-year history of chronic, sensory-motor, symmetrical, length dependent polyneuropathy with insidious onset and slow progression. The patient presents with severe distal weakness and atrophy in the four limbs, hypoesthesia up to calves, pes planus, and scoliosis with no cranial nerve or autonomic involvement. He uses a rollator for walking. Past medical history is silent. Nerve conduction studies show demyelinating, symmetric, sensory-motor polyneuropathy. Screening lab tests including vitamin B12, folate, vitamin B6, vitamin E, HBV, HCV, glycemia, HbA1c, ANA, ANCA, ENA, CRP, ESR, copper, cryoglobulins, and serum electrophoresis are all negative. There is no family history of polyneuropathy.	CMT4	**Summary of Clinical Information**<br>**Patient Demographics:**<br>● 22-year-old male<br>**History and Symptoms:**<br>● 20-year history of chronic, sensory-motor, symmetrical, length-dependent polyneuropathy<br>● Insidious onset and slow progression<br>● Severe distal weakness and atrophy in all four limbs<br>● Hypoesthesia up to the calves<br>● Pes planus (flat feet)<br>● Scoliosis<br>● No cranial nerve or autonomic involvement<br>**Functional Impact:**<br>● Requires a rollator for walking<br>**Diagnostic Findings:**<br>● Nerve conduction studies indicate demyelinating, symmetric, sensory-motor polyneuropathy<br>**Lab Results:**<br>● Screening tests for vitamin B12, folate, vitamin B6, vitamin E, HBV, HCV, glycemia, HbA1c, ANA, ANCA, ENA, CRP, ESR, copper, cryoglobulins, and serum electrophoresis are all negative<br>**Family History:**<br>● No family history of polyneuropathy<br>**Differential Diagnoses for Polyneuropathy**<br>1. **Charcot-Marie-Tooth Disease (CMT) Type 1**<br>○ **Reasoning:**<br>■ **Chronic and Progressive:** The patient's long history (20 years) of slowly progressive, length-dependent polyneuropathy is characteristic of CMT.<br>■ **Demyelinating Nature:** Nerve conduction studies show demyelinating polyneuropathy, consistent with CMT type 1, which is a demyelinating form.<br>■ **Physical Findings:** Severe distal weakness, atrophy, pes planus, and scoliosis are all consistent with CMT manifestations.<br>■ **Absence of Systemic Involvement:** No cranial nerve or autonomic involvement aligns with CMT, which typically does not involve these systems.<br>■ **Young Age of Onset:** Symptoms starting at a young age are typical for hereditary neuropathies like CMT.<br>■ **Negative Family History:** While CMT is usually inherited, sporadic mutations can occur, explaining the lack of family history.<br>2. **Hereditary Sensory and Motor Neuropathy (HSMN)**<br>○ **Reasoning:**<br>■ **Presentation:** Chronic, symmetrical, sensory-motor polyneuropathy fits HSMN characteristics.<br>■ **Demyelination:** The demyelinating nature of the neuropathy seen in nerve conduction studies is compatible with certain forms of HSMN.<br>■ **Physical Symptoms:** Severe distal weakness, atrophy, and hypoesthesia are typical for HSMN.<br>■ **Scoliosis and Pes Planus:** These are common skeletal abnormalities seen in HSMN.<br>■ **Age and Slow Progression:** Early onset and gradual progression support a hereditary neuropathy.<br>3. **Chronic Inflammatory Demyelinating Polyneuropathy (CIDP)**<br>○ **Reasoning:**<br>■ **Demyelinating Neuropathy:** Nerve conduction studies show demyelination, a hallmark of CIDP.<br>■ **Chronic Course:** The chronic, slow progression over 20 years could be seen in a chronic form of CIDP.<br>■ **Symmetry:** Symmetric involvement is common in CIDP.<br>■ **Exclusion of Other Causes:** Negative extensive lab work rules out many other causes, supporting an immune-mediated process like CIDP.<br>■ **Lack of Family History:** CIDP is not typically hereditary, aligning with the patient's family history.<br>**Most Likely Diagnosis**<br>Based on the presented clinical information, the most likely diagnosis is **Charcot-Marie-Tooth Disease (CMT) Type 1**. This conclusion is supported by the combination of chronicity, symmetrical and length-dependent nature of the neuropathy, demyelinating features on nerve conduction studies, and associated physical findings (pes planus, scoliosis). The absence of cranial nerve or autonomic involvement and the early onset also favor CMT over other potential diagnoses. Despite the negative family history, sporadic cases can occur, making this diagnosis plausible.
15	A 72-year-old female patient presents with a 2-year history of pure sensory, symmetrical, length dependent polyneuropathy with insidious onset. The patient presents with distal paresthesia	Chemotherapy-induced neuropathy	**Summary of Pertinent Clinical Information:**<br>1. **History and Symptoms**:<br>○ 2-year history of pure sensory, symmetrical, length-dependent polyneuropathy with insidious onset.<br>○ Symptoms: Distal paresthesia and neuropathic pain in the upper and lower limbs in a stocking and glove distribution.<br>○ No ataxia, tremor, foot deformities, cranial nerve, or autonomic involvement.

	and neuropathic pain in the upper and lower limbs in a stocking and glove distribution, without ataxia or tremor, foot deformities, cranial nerve or autonomic involvement. Past medical history shows multiple myeloma, for which the patient has undergone chemotherapy. Neuropathy symptoms started 2 months after chemotherapy. Nerve conduction studies show pure sensory, symmetric, axonal polyneuropathy. Screening lab tests including vitamin B12, folate, vitamin B6, vitamin E, HBV, HCV, ANA, ANCA, ENA, CRP, ESR, glycemia, HbA1c, copper and cryoglobulins are all negative, with positivity for IgG kappa paraprotein. There is no family history of polyneuropathy.		○ Neuropathy symptoms started 2 months after chemotherapy for multiple myeloma.<br>2. **Past Medical History**:<br>○ Multiple myeloma.<br>○ Chemotherapy treatment.<br>3. **Investigations**:<br>○ Nerve conduction studies: Pure sensory, symmetric, axonal polyneuropathy.<br>○ Lab tests: Negative for vitamin B12, folate, vitamin B6, vitamin E, HBV, HCV, ANA, ANCA, ENA, CRP, ESR, glycemia, HbA1c, copper, and cryoglobulins.<br>○ Positive for IgG kappa paraprotein.<br>○ No family history of polyneuropathy.<br>**Differential Diagnoses:**<br>1. **Chemotherapy-Induced Peripheral Neuropathy (CIPN)**:<br>○ **Reasoning**: The onset of neuropathy symptoms occurred 2 months after the initiation of chemotherapy for multiple myeloma, which strongly suggests a temporal relationship. Chemotherapy agents, particularly those used in the treatment of multiple myeloma, such as bortezomib, thalidomide, or vincristine, are well-known to cause peripheral neuropathy.<br>○ **Supporting Evidence**: The pure sensory and symmetric nature of the neuropathy aligns with the typical presentation of CIPN. The stocking and glove distribution is also characteristic of CIPN.<br>2. **Paraneoplastic Neuropathy**:<br>○ **Reasoning**: The presence of multiple myeloma and the positive finding of IgG kappa paraprotein suggest a possible paraneoplastic syndrome. Paraneoplastic syndromes are remote effects of cancer, mediated by immune mechanisms, which can lead to peripheral neuropathy.<br>○ **Supporting Evidence**: The chronic and insidious nature of the neuropathy, as well as the pure sensory involvement without motor or autonomic symptoms, can be seen in paraneoplastic neuropathies. The presence of IgG kappa paraprotein might indicate an immune-mediated mechanism linked to the underlying malignancy.<br>3. **Monoclonal Gammopathy of Undetermined Significance (MGUS)-Related Neuropathy**:<br>○ **Reasoning**: MGUS can be associated with peripheral neuropathy, and the detection of IgG kappa paraprotein might indicate an MGUS-related process. Patients with MGUS can develop a chronic, symmetrical, sensory neuropathy.<br>○ **Supporting Evidence**: The chronic nature of the neuropathy and the presence of a paraprotein support this diagnosis. Although MGUS typically has a less aggressive clinical course compared to multiple myeloma, the overlap between the two conditions (especially if multiple myeloma is in remission) makes this a consideration.<br>**Most Likely Diagnosis:**<br>**Chemotherapy-Induced Peripheral Neuropathy (CIPN)** is the most likely diagnosis. The temporal relationship between the onset of neuropathy and the administration of chemotherapy, combined with the characteristic clinical presentation and the exclusion of other common causes through comprehensive screening, strongly supports this diagnosis.<br>The positive IgG kappa paraprotein could be related to the underlying multiple myeloma or could indicate a coexisting MGUS. However, the timing and clinical course are most consistent with CIPN.
16	A 59-year-old female patient presents with a 1-year history of pure sensory, symmetrical, non-length dependent polyneuropathy with insidious onset. The patient presents with paresthesia distributed proximally and distally in the upper and lower limbs in an asymmetric fashion, and sensory ataxia. There are no foot deformities, tremor, neuropathic pain, cranial nerve or autonomic involvement. Past medical history is positive for IgG lambda monoclonal gammopathy and lung cancer, for which she underwent chemotherapy 4 years ago. Nerve conduction studies show a demyelinating, non-length dependent, sensory-motor polyneuropathy with conduction blocks. Screening lab tests including vitamin B12, folate, vitamin B6, vitamin E, HCV, ANA, ANCA, ENA, CRP, ESR, glycemia, HbA1c, copper and cryoglobulins are all negative, with positivity for IgG lambda paraprotein and evidence of chronic HBV infection. There is no family history of polyneuropathy.	CIDP	**Summary of Pertinent Clinical Information**<br>● **Patient**: 59-year-old female<br>● **History**:<br>○ 1-year history of pure sensory, symmetrical, non-length dependent polyneuropathy<br>○ Insidious onset of paresthesia in upper and lower limbs, asymmetrically distributed both proximally and distally<br>○ Sensory ataxia<br>○ No foot deformities, tremor, neuropathic pain, cranial nerve involvement, or autonomic involvement<br>○ Positive past medical history for IgG lambda monoclonal gammopathy and lung cancer treated with chemotherapy 4 years ago<br>● **Nerve Conduction Studies**: Demyelinating, non-length dependent, sensory-motor polyneuropathy with conduction blocks<br>● **Screening Lab Tests**:<br>○ Negative for vitamin B12, folate, vitamin B6, vitamin E, HCV, ANA, ANCA, ENA, CRP, ESR, glycemia, HbA1c, copper, cryoglobulins<br>○ Positive for IgG lambda paraprotein<br>○ Chronic HBV infection<br>● **Family History**: No history of polyneuropathy<br>**Differential Diagnoses for Polyneuropathy**<br>1. **Monoclonal Gammopathy of Undetermined Significance (MGUS)-associated Polyneuropathy**<br>○ **Reasoning**:<br>■ Presence of IgG lambda monoclonal gammopathy<br>■ Typical association with demyelinating neuropathy<br>■ MGUS is a common cause of polyneuropathy, especially when associated with conduction blocks and no significant autonomic involvement<br>■ Lack of other evident causes in lab tests<br>○ **Supporting Evidence**:<br>■ Polyneuropathy in MGUS is usually sensory or sensory-motor, and can present as non-length dependent<br>■ Nerve conduction studies showing demyelination are consistent with MGUS-related neuropathy<br>2. **Paraneoplastic Polyneuropathy**<br>○ **Reasoning**:<br>■ History of lung cancer, which can be associated with paraneoplastic syndromes affecting the nervous system<br>■ Paraneoplastic neuropathies can present with varied symptoms, including non-length dependent sensory involvement<br>■ Insidious onset and lack of significant improvement post-cancer treatment may suggest a paraneoplastic etiology<br>○ **Supporting Evidence**:<br>■ Paraneoplastic syndromes often present with asymmetric and non-length dependent distribution<br>■ Sensory ataxia is a common feature in paraneoplastic neuropathies<br>3. **Chronic Inflammatory Demyelinating Polyradiculoneuropathy (CIDP)**<br>○ **Reasoning**:

			■ CIDP presents with demyelinating polyneuropathy, which can be symmetric and non-length dependent<br>■ The chronic progressive course and lack of autonomic involvement fit the CIDP profile<br>■ CIDP can occur secondary to other conditions such as MGUS or chronic infections<br>○ **Supporting Evidence**:<br>■ Conduction blocks in nerve conduction studies are characteristic of CIDP<br>■ Symmetrical sensory and motor involvement without length dependency<br>**Most Likely Diagnosis**<br>Based on the clinical presentation and supporting diagnostic evidence, **Monoclonal Gammopathy of Undetermined Significance (MGUS)-associated Polyneuropathy** is the most likely diagnosis. The presence of IgG lambda paraprotein, demyelinating nature of the polyneuropathy, and the lack of other identifiable causes strongly suggest this condition. Additionally, MGUS is a recognized cause of neuropathy in patients with similar presentations, particularly when the nerve conduction studies indicate demyelination and conduction blocks. The chronicity and sensory-predominant features without autonomic involvement further support MGUS-associated neuropathy over the other differentials.
17	A 73-year-old female patient presents with a 1-year history of sensory-motor, symmetrical, polyneuropathy with subacute onset. The patient presents with distal paresthesia and neuropathic pain in the four limbs, sensory ataxia, cramps, orthostatic hypotension and diarrhoea but with no cranial nerve involvement or foot deformities. Clinical examination shows cutaneous angiomas and hyperpigmentation. Laboratory tests reveal presence of thrombocytosis and IgG lambda monoclonal gammopathy. Nerve conduction studies show a symmetric, sensory-motor, demyelinating-axonal polyneuropathy. Screening lab tests including vitamin B12, folate, vitamin B6, vitamin E, HCV, HBV, ANA, ANCA, ENA, CRP, ESR, glycemia, HbA1c, copper and cryoglobulins are negative, with positivity for IgG lambda paraprotein. There is no family history of polyneuropathy.	POEMS	**Summary of Pertinent Clinical Information**<br>● **Patient Demographics:** 73-year-old female<br>● **Clinical History:** 1-year history of sensory-motor, symmetrical, polyneuropathy with subacute onset<br>● **Symptoms:**<br>○ Distal paresthesia<br>○ Neuropathic pain in all four limbs<br>○ Sensory ataxia<br>○ Cramps<br>○ Orthostatic hypotension<br>○ Diarrhoea<br>● **Physical Examination:**<br>○ Cutaneous angiomas<br>○ Hyperpigmentation<br>● **Lab Findings:**<br>○ Thrombocytosis<br>○ IgG lambda monoclonal gammopathy (IgG lambda paraprotein positive)<br>○ Negative for vitamin B12, folate, vitamin B6, vitamin E, HCV, HBV, ANA, ANCA, ENA, CRP, ESR, glycemia, HbA1c, copper, and cryoglobulins<br>● **Nerve Conduction Studies:** Symmetric, sensory-motor, demyelinating-axonal polyneuropathy<br>● **Other:** No cranial nerve involvement or foot deformities; no family history of polyneuropathy<br>**Differential Diagnoses for Polyneuropathy**<br>**1. Monoclonal Gammopathy of Undetermined Significance (MGUS) Related Neuropathy**<br>● **Reasoning:**<br>○ The presence of IgG lambda monoclonal gammopathy strongly suggests a paraneoplastic or MGUS-related neuropathy.<br>○ MGUS can lead to neuropathy through the deposition of paraproteins or immune-mediated mechanisms, which can cause both demyelination and axonal damage as seen in this patient's nerve conduction studies.<br>○ Symptoms like distal paresthesia, neuropathic pain, and sensory ataxia are consistent with MGUS-related neuropathy.<br>○ The lack of other systemic involvements and specific findings (like absence of organomegaly or bone lesions) supports MGUS rather than more severe plasma cell disorders.<br>**2. Primary Amyloidosis (AL Amyloidosis)**<br>● **Reasoning:**<br>○ The cutaneous angiomas and hyperpigmentation can be related to amyloid deposition.<br>○ IgG lambda monoclonal gammopathy could be associated with AL amyloidosis.<br>○ Polyneuropathy in AL amyloidosis is typically symmetric and sensory-motor, often with autonomic features such as orthostatic hypotension and gastrointestinal symptoms (e.g., diarrhea).<br>○ Nerve conduction studies showing demyelinating and axonal features are compatible with amyloid neuropathy.<br>○ AL amyloidosis often presents with multi-organ involvement, but the absence of family history and foot deformities makes this a less common presentation.<br>**3. Chronic Inflammatory Demyelinating Polyneuropathy (CIDP) Associated with Paraproteinemia**<br>● **Reasoning:**<br>○ CIDP is a chronic progressive disease that presents with symmetric, sensory, and motor neuropathy.<br>○ It can be associated with paraproteinemias, particularly monoclonal gammopathies.<br>○ The presence of demyelinating features on nerve conduction studies is typical for CIDP.<br>○ Autonomic symptoms such as orthostatic hypotension and gastrointestinal issues can occur in CIDP, although less commonly than in amyloidosis.<br>○ The subacute onset and the presence of an IgG lambda monoclonal component further suggest a possible link between CIDP and the paraproteinemia.<br>**Most Likely Diagnosis**<br>Given the clinical presentation, laboratory findings, and nerve conduction studies, the most likely diagnosis is **Monoclonal Gammopathy of Undetermined Significance (MGUS) Related Neuropathy**.<br>● The presence of an IgG lambda monoclonal protein in the absence of other specific systemic involvements strongly supports MGUS-related neuropathy.<br>● The clinical features, including distal paresthesia, neuropathic pain, sensory ataxia, and autonomic symptoms, are consistent with this diagnosis.<br>● While primary amyloidosis and CIDP are possible, the lack of additional supportive findings such as systemic amyloid deposits (for amyloidosis) or a purely demyelinating pattern (for CIDP) makes MGUS-related neuropathy the most plausible diagnosis in this scenario.
18	A 55-year-old male patient presents with a 5-month history of pure sensory, asymmetric, non-length dependent polyneuropathy with subacute onset and progressive course. The patient presents	Sjogren-associated ganglionopathy	**Summary of Clinical Information:**<br>● **Patient**: 55-year-old male<br>● **History**:<br>○ 5-month history of sensory, asymmetric, non-length dependent polyneuropathy<br>○ Subacute onset, progressive course

	with paresthesia and hypoesthesia in the distal areas of the four limbs, severe sensory ataxia, orthostatic hypotension, and delayed gastric and intestinal emptying. The patient also reports xerostomia and xerophthalmia. Clinical examination reveals reduced pupillary reflex and anhidrosis. There are no neuropathic pain, tremor, foot deformities or cranial nerve involvement. Past medical history is positive for Hashimoto's thyroiditis. Nerve conduction studies show a pure sensory, non-length dependent, axonal polyneuropathy. Screening lab tests reveal folate deficiency and low-titre ENA positivity. Remaining exams including vitamin B12, vitamin B6, vitamin E, HBV, HCV, ANA, ANCA, CRP, ESR, copper, glycemia, HbA1c, cryoglobulins, and serum electrophoresis are normal. There is no family history of polyneuropathy.		○ Symptoms: paresthesia, hypoesthesia in distal limbs, severe sensory ataxia, orthostatic hypotension, delayed gastric and intestinal emptying, xerostomia, xerophthalmia<br>○ Clinical findings: reduced pupillary reflex, anhidrosis<br>○ No neuropathic pain, tremor, foot deformities, or cranial nerve involvement<br>○ Past medical history: Hashimoto's thyroiditis<br>● **Investigations**:<br>○ Nerve conduction studies: pure sensory, non-length dependent, axonal polyneuropathy<br>○ Lab tests: folate deficiency, low-titre ENA positivity<br>○ Other lab tests (vitamin B12, vitamin B6, vitamin E, HBV, HCV, ANA, ANCA, CRP, ESR, copper, glycemia, HbA1c, cryoglobulins, serum electrophoresis): normal<br>● **Family History**: No family history of polyneuropathy<br>**Differential Diagnoses for Polyneuropathy:**<br>**1. Autoimmune Autonomic Ganglionopathy (AAG)**<br>● **Reasoning**:<br>○ The presence of subacute onset and progressive course with significant autonomic features (orthostatic hypotension, delayed gastric and intestinal emptying, xerostomia, xerophthalmia, reduced pupillary reflex, and anhidrosis) strongly suggests autonomic dysfunction.<br>○ Hashimoto's thyroiditis indicates an underlying autoimmune disorder, which could predispose the patient to other autoimmune conditions.<br>○ Low-titre ENA positivity supports an autoimmune etiology.<br>○ AAG typically presents with sensory neuropathy along with autonomic dysfunction, aligning with the patient's clinical picture.<br>**2. Sjogren's Syndrome-associated Neuropathy**<br>● **Reasoning**:<br>○ Sjogren's syndrome is characterized by xerostomia and xerophthalmia, which are present in this patient.<br>○ The presence of an autoimmune condition (Hashimoto's thyroiditis) increases the likelihood of another autoimmune disorder like Sjogren's syndrome.<br>○ ENA positivity is common in Sjogren's syndrome.<br>○ Sensory neuropathy with autonomic involvement can occur in Sjogren's syndrome, matching the patient's clinical features.<br>**3. Paraneoplastic Neuropathy**<br>● **Reasoning**:<br>○ Paraneoplastic syndromes can present with a wide range of neurological manifestations, including sensory neuropathy and autonomic dysfunction.<br>○ The progressive course and autonomic symptoms (orthostatic hypotension, delayed gastric and intestinal emptying, reduced pupillary reflex, anhidrosis) are consistent with paraneoplastic autonomic neuropathy.<br>○ Although no malignancy has been identified yet, the presence of neuropathy with autonomic features warrants investigation for an occult malignancy.<br>○ Paraneoplastic syndromes can be associated with various autoantibodies, including those against neuronal antigens, which might explain the low-titre ENA positivity.<br>**Most Likely Diagnosis:**<br>Given the clinical presentation of sensory neuropathy with significant autonomic dysfunction, autoimmune history, and supporting laboratory findings, **Autoimmune Autonomic Ganglionopathy (AAG)** is the most likely diagnosis. The combination of severe autonomic symptoms, sensory involvement, and autoimmune markers (low-titre ENA positivity) strongly points towards AAG as the underlying cause. Further diagnostic workup, including testing for ganglionic AChR antibodies, would be beneficial to confirm this diagnosis.
19	A 70-year-old male patient presents with a 5-year history of sensory-motor, symmetrical, length dependent polyneuropathy with insidious onset and fast disease progression. The patient presents with moderate-to-severe distal weakness and hypoesthesia in the four limbs in a distal and symmetric distribution, sensory ataxia, tongue fasciculations and autonomic involvement with no foot deformities or tremor. Past medical history is positive for cardiac insufficiency. Nerve conduction studies show an axonal, sensory-motor polyneuropathy with bilateral carpal tunnel syndrome. Screening lab tests including vitamin B12, vitamin B9, folate, vitamin E, HBV, HCV, ANA, ANCA, ENA, glycemia, HbA1c, CRP, ESR, copper, cryoglobulins, and serum electrophoresis are all negative. His father, who was wheelchair bound, died from cardiac insufficiency.	TTR-Amyloidosis	**Summary of Pertinent Clinical Information**<br>● **Patient**: 70-year-old male<br>● **History**: 5-year history of sensory-motor, symmetrical, length-dependent polyneuropathy<br>○ **Onset**: Insidious<br>○ **Progression**: Fast<br>● **Symptoms**:<br>○ Moderate-to-severe distal weakness and hypoesthesia in all four limbs, distal and symmetric distribution<br>○ Sensory ataxia<br>○ Tongue fasciculations<br>○ Autonomic involvement<br>● **Absent Symptoms**: No foot deformities, no tremor<br>● **Past Medical History**: Cardiac insufficiency<br>● **Nerve Conduction Studies**: Axonal, sensory-motor polyneuropathy with bilateral carpal tunnel syndrome<br>● **Lab Tests**: Negative for vitamin B12, vitamin B9, folate, vitamin E, HBV, HCV, ANA, ANCA, ENA, glycemia, HbA1c, CRP, ESR, copper, cryoglobulins, and serum electrophoresis<br>● **Family History**: Father was wheelchair-bound and died from cardiac insufficiency<br>**Differential Diagnoses for Polyneuropathy**<br>1. **Familial Amyloid Polyneuropathy (FAP)**<br>○ **Reasoning**:<br>■ **Clinical Presentation**: FAP often presents with a similar pattern of sensory-motor, length-dependent polyneuropathy. The autonomic involvement and cardiac issues align well with amyloidosis affecting multiple systems.<br>■ **Family History**: The patient's father's history of being wheelchair-bound and dying from cardiac insufficiency suggests a possible hereditary condition.<br>■ **Tongue Fasciculations**: This symptom can be associated with amyloidosis.<br>■ **Negative Lab Tests**: The extensive negative screening tests further point towards a hereditary condition like FAP, which wouldn't typically show abnormalities in these tests.<br>2. **Hereditary Neuropathy with Liability to Pressure Palsies (HNPP)**<br>○ **Reasoning**:<br>■ **Clinical Presentation**: While HNPP typically involves recurrent pressure palsies, the bilateral carpal tunnel syndrome seen in nerve conduction studies could indicate a hereditary neuropathy.<br>■ **Family History**: The hereditary nature of HNPP fits with the patient's family history of similar symptoms.<br>■ **Negative Lab Tests**: HNPP would not be detected in the usual screening tests performed.<br>■ **Autonomic and Motor Symptoms**: While HNPP usually presents with sensory symptoms and pressure palsies, severe cases can involve motor and autonomic symptoms.

			3. **Multifocal Motor Neuropathy (MMN)**<br>○ **Reasoning**:<br>■ **Clinical Presentation**: MMN typically presents with asymmetric motor weakness, which does not perfectly align with this patient's symmetrical symptoms, but it can sometimes present with distal weakness.<br>■ **Nerve Conduction Studies**: The presence of bilateral carpal tunnel syndrome might be considered in MMN, although axonal polyneuropathy is less typical.<br>■ **Negative Lab Tests**: MMN is often associated with anti-GM1 antibodies, which were not tested, but the negativity of other tests does not exclude MMN.<br>■ **Autonomic Involvement**: Autonomic symptoms are not typical for MMN, making this less likely.<br>**Most Likely Diagnosis**<br>Based on the clinical presentation, family history, and negative lab findings, **Familial Amyloid Polyneuropathy (FAP)**emerges as the most probable diagnosis. The combination of sensory-motor polyneuropathy, autonomic involvement, and family history of similar symptoms strongly suggests a hereditary neuropathy like FAP. Furthermore, the lack of abnormalities in extensive lab tests supports the possibility of a condition not typically detected in standard screenings, such as a genetic disorder like FAP.
20	A 64-year-old male patient presents with an 18-month history of pure motor, asymmetric, non-length dependent polyneuropathy with insidious onset. The patient presents with weakness and atrophy in a multifocal distribution in the upper limbs. He also reports the presence of subjective paresthesia in the hands but at clinical examination objective tactile sensation is normal. There are no neuropathic pain, ataxia, tremor, foot deformities, cranial nerve or autonomic involvement. Past medical history is silent. Nerve conduction studies show pure motor, demyelinating multineuropathy in the upper limbs. Screening lab tests including vitamin B12, folate, vitamin B6, vitamin E, HCV, HBV, ANA, ANCA, ENA, CRP, ESR, glycemia, HbA1c, copper, cryoglobulins and serum electrophoresis are negative. Cervical spine MRI is normal. There is no family history of polyneuropathy.	MMN	**Clinical Summary**<br>**Patient:** 64-year-old male<br>**History:**<br>● 18-month history of pure motor, asymmetric, non-length dependent polyneuropathy with insidious onset<br>● Weakness and atrophy in a multifocal distribution in the upper limbs<br>● Subjective paresthesia in the hands, but objective tactile sensation is normal<br>● No neuropathic pain, ataxia, tremor, foot deformities, cranial nerve, or autonomic involvement<br>● Past medical history is silent<br>● No family history of polyneuropathy<br>**Investigations:**<br>● Nerve conduction studies: Pure motor, demyelinating multineuropathy in the upper limbs<br>● Screening lab tests: Negative (including vitamin B12, folate, vitamin B6, vitamin E, HCV, HBV, ANA, ANCA, ENA, CRP, ESR, glycemia, HbA1c, copper, cryoglobulins, serum electrophoresis)<br>● Cervical spine MRI: Normal<br>**Differential Diagnoses**<br>1. **Multifocal Motor Neuropathy (MMN)**<br>○ **Reasoning:**<br>■ MMN is characterized by asymmetric, predominantly motor neuropathy, which aligns with the patient's presentation of weakness and atrophy in a multifocal distribution.<br>■ The presence of demyelinating features on nerve conduction studies is typical for MMN.<br>■ Absence of sensory involvement (despite subjective paresthesia without objective findings) supports MMN.<br>■ Normal cervical spine MRI and negative broad spectrum of screening lab tests further align with MMN, as it does not usually show systemic involvement or typical lab abnormalities.<br>2. **Chronic Inflammatory Demyelinating Polyneuropathy (CIDP) - Pure Motor Variant**<br>○ **Reasoning:**<br>■ CIDP can present as a pure motor neuropathy, particularly in its atypical forms.<br>■ The chronic, progressive course over 18 months and demyelinating features on nerve conduction studies are consistent with CIDP.<br>■ The absence of sensory loss, which is less common but possible in CIDP, matches the patient's clinical picture.<br>■ However, the lack of response to initial typical CIDP treatments and the asymmetric presentation may make this diagnosis slightly less likely compared to MMN.<br>3. **Lewis-Sumner Syndrome (MADSAM - Multifocal Acquired Demyelinating Sensory and Motor Neuropathy)**<br>○ **Reasoning:**<br>■ This condition presents with asymmetric, multifocal neuropathy involving both motor and sensory nerves.<br>■ The patient's presentation of asymmetric, multifocal weakness and atrophy fits well, although the sensory involvement in this case is more subjective rather than objective.<br>■ The demyelinating nature seen on nerve conduction studies supports this diagnosis.<br>■ Normal lab results and absence of systemic disease also fit, but the predominant motor involvement with only subjective sensory symptoms is less typical for MADSAM, which usually has clear sensory deficits.<br>**Most Likely Diagnosis**<br>**Multifocal Motor Neuropathy (MMN)**<br>Given the patient's presentation of asymmetric, purely motor involvement with demyelinating features on nerve conduction studies and the absence of sensory deficits on examination, MMN is the most likely diagnosis. The chronic and insidious nature of the disease, along with normal systemic investigations and the pattern of nerve involvement, further support this diagnosis. MMN typically does not involve sensory nerves objectively, and the absence of systemic findings or family history is characteristic.
21	A 74-year-old male patient presents with a 3-month history of sensory-motor, asymmetric, length dependent polyneuropathy with subacute onset and progressive course. The patient presents with sensory ataxia associated with neuropathic pain, paraesthesia, and hypoesthesia in the feet, and weakness with distal, asymmetric distribution. Clinical examination does not show presence of tremor, foot deformities, cranial nerve or autonomic involvement. Past medical history is positive for multiple myeloma, in remission for the past three	CIDP	**Summary of Pertinent Clinical Information**<br>**Patient Details:**<br>● **Age/Gender:** 74-year-old male<br>● **Presentation:** 3-month history of sensory-motor, asymmetric, length-dependent polyneuropathy with a subacute onset and progressive course<br>● **Symptoms:** Sensory ataxia, neuropathic pain, paraesthesia, hypoesthesia in feet, and distal asymmetric weakness<br>● **Examination Findings:**<br>○ Sensory ataxia<br>○ No tremor<br>○ No foot deformities<br>○ No cranial nerve or autonomic involvement<br>● **Past Medical History:**<br>○ Multiple myeloma, in remission for 3 years, no maintenance therapy required<br>● **Nerve Conduction Studies:** Demyelinating, sensory-motor polyneuropathy<br>● **Screening Lab Tests:**

	years with no need for maintenance therapy (last chemotherapy cycle performed three years ago). Nerve conduction studies show a demyelinating, sensory-motor polyneuropathy. Screening lab tests show presence of IgG lambda paraprotein. Remaining exams including vitamin B12, folate, vitamin B6, vitamin E, HBV, HCV, ANA, ANCA, ENA, CRP, ESR, glycemia, HbA1c, copper, and cryoglobulins are negative. There is no family history of polyneuropathy.		○ Positive for IgG lambda paraprotein<br>○ Negative for: Vitamin B12, folate, vitamin B6, vitamin E, HBV, HCV, ANA, ANCA, ENA, CRP, ESR, glycemia, HbA1c, copper, cryoglobulins<br>● **Family History:** No family history of polyneuropathy<br>**Differential Diagnoses**<br>1. **Monoclonal Gammopathy of Undetermined Significance (MGUS) - related Polyneuropathy**<br>○ **Reasoning:**<br>■ The presence of IgG lambda paraprotein suggests a monoclonal gammopathy.<br>■ MGUS can be associated with a demyelinating polyneuropathy.<br>■ The patient's history of multiple myeloma in remission raises the possibility of a related monoclonal gammopathy.<br>■ The clinical features of sensory ataxia, neuropathic pain, and asymmetric distribution align with MGUS-related neuropathy.<br>2. **Chronic Inflammatory Demyelinating Polyneuropathy (CIDP)**<br>○ **Reasoning:**<br>■ CIDP often presents with a progressive, sensory-motor polyneuropathy.<br>■ Demyelinating features on nerve conduction studies are consistent with CIDP.<br>■ The lack of cranial nerve and autonomic involvement, along with sensory ataxia and pain, are compatible with CIDP.<br>■ CIDP can sometimes be associated with monoclonal gammopathy, linking it with the patient's history of multiple myeloma.<br>3. **Paraneoplastic Neuropathy**<br>○ **Reasoning:**<br>■ Given the patient's history of multiple myeloma, a paraneoplastic syndrome is a possibility.<br>■ Paraneoplastic neuropathies can present with asymmetric, sensory-motor polyneuropathy.<br>■ The presence of IgG lambda paraprotein could be indicative of an underlying malignancy-related condition.<br>**Most Likely Diagnosis**<br>**Monoclonal Gammopathy of Undetermined Significance (MGUS) - related Polyneuropathy**<br>● The most likely diagnosis is MGUS-related polyneuropathy. The presence of IgG lambda paraprotein, the patient's history of multiple myeloma (which can transform into MGUS when in remission), and the clinical and electrophysiological findings strongly suggest this diagnosis. The pattern of sensory-motor, asymmetric, length-dependent polyneuropathy with demyelinating features on nerve conduction studies aligns well with MGUS-related neuropathy. While CIDP is also a strong contender given the demyelinating nature of the neuropathy, the presence of a monoclonal protein makes MGUS-related neuropathy more likely in this context.
22	An 82-year-old female patient presents with a 2-year history of pure sensory, symmetrical, length dependent polyneuropathy with insidious onset and slow progressive course. The patient presents with paresthesia, hypoesthesia, and mild neuropathic pain in the feet, associated with diffused cramps in the lower limbs. Clinical examination does not reveal sensory ataxia, tremor, foot deformities, cranial nerve or autonomic involvement. Past medical history is positive for chronic HCV infection, psoriatic arthritis, and fibromyalgia. Nerve conduction studies show presence of a pure sensory, length dependent, symmetric, axonal polyneuropathy with unilateral carpal tunnel syndrome. Screening lab tests including vitamin B12, folate, vitamin B6, vitamin E, HBV, ANCA, ENA, CRP, ESR, copper, cryoglobulins and serum electrophoresis are all negative, with positivity for ANA autoantibodies and evidence of chronic HCV infection. There is no family history of polyneuropathy.	HCV-associated neuropathy	**Summary of Pertinent Clinical Information:**<br>● **Patient Demographics**: 82-year-old female<br>● **Clinical History**:<br>○ 2-year history of pure sensory, symmetrical, length-dependent polyneuropathy with an insidious onset and slow progressive course.<br>○ Symptoms: paresthesia, hypoesthesia, mild neuropathic pain in the feet, and diffused cramps in the lower limbs.<br>● **Clinical Examination**:<br>○ No sensory ataxia, tremor, foot deformities, cranial nerve, or autonomic involvement.<br>● **Past Medical History**:<br>○ Chronic HCV infection<br>○ Psoriatic arthritis<br>○ Fibromyalgia<br>● **Nerve Conduction Studies**:<br>○ Pure sensory, length-dependent, symmetric, axonal polyneuropathy<br>○ Unilateral carpal tunnel syndrome<br>● **Laboratory Findings**:<br>○ Negative: vitamin B12, folate, vitamin B6, vitamin E, HBV, ANCA, ENA, CRP, ESR, copper, cryoglobulins, and serum electrophoresis<br>○ Positive: ANA autoantibodies, evidence of chronic HCV infection<br>● **Family History**: No family history of polyneuropathy<br>**Differential Diagnoses for Polyneuropathy:**<br>1. **Hepatitis C Virus (HCV)-Associated Neuropathy**:<br>○ **Reasoning**:<br>■ Chronic HCV infection is present, which is a known cause of peripheral neuropathy.<br>■ HCV can lead to cryoglobulinemia, which was tested and found negative, but HCV itself can cause neuropathy without cryoglobulinemia.<br>■ The slow progressive course and length-dependent nature of the neuropathy align with HCV-associated neuropathy.<br>■ Other causes like vitamin deficiencies, metabolic disorders, and other infections have been ruled out.<br>2. **ANA-Positive Connective Tissue Disease-Associated Neuropathy**:<br>○ **Reasoning**:<br>■ Positive ANA autoantibodies suggest the possibility of an autoimmune process.<br>■ Psoriatic arthritis, an autoimmune condition, is part of her medical history, which might be associated with other autoimmune conditions.<br>■ Sensory polyneuropathy can occur in connective tissue diseases such as lupus or Sjögren's syndrome, which might be ANA-positive.<br>■ The absence of systemic symptoms or signs of other organ involvement makes this less likely but still a consideration given the ANA positivity.<br>3. **Idiopathic Sensory Neuropathy**:<br>○ **Reasoning**:<br>■ Despite extensive workup, no definitive cause has been identified.<br>■ The patient's age is a risk factor for idiopathic neuropathy, which is more common in older adults.<br>■ The negative results for multiple potential etiologies, including vitamin deficiencies, autoimmune markers, and metabolic causes, suggest an idiopathic nature.<br>■ While less specific, this diagnosis remains plausible given the lack of clear alternative causes.

			**Most Likely Diagnosis:**<br>Given the combination of the patient's chronic HCV infection and the pattern of neuropathy, **HCV-associated neuropathy** is the most likely diagnosis. This aligns well with her clinical presentation and the negative findings for other common causes of polyneuropathy. The presence of ANA autoantibodies might be incidental or part of her autoimmune profile related to psoriatic arthritis, but the direct link between HCV and neuropathy makes it the most probable primary cause.
23	A 59-year-old male patient presents with a 1-year history of sensory-motor, symmetrical, non-length dependent polyneuropathy with subacute onset and progressive course. The patient presents with paresthesia, neuropathic pain and weakness distributed proximally and distally in the lower limbs. The patient also reports that in the last months he has noticed the appearance of white nails. Clinical examination does not reveal ataxia, tremor, foot deformities, cranial nerve or autonomic involvement. Past medical history is positive for alcohol abuse. Screening lab tests show the presence of thrombocytosis and IgG lambda monoclonal gammopathy. Vitamin B12, folate, vitamin B6, E, HCV, HBV, ANA, ANCA, ENA, CRP, ESR, glycemia, HbA1c, copper and cryoglobulins are normal. Nerve conduction studies show a mixed demyelinating-axonal, sensory-motor polyneuropathy. There is no family history of polyneuropathy.	POEMS	**Summary of Pertinent Clinical Information**<br>**Patient Profile:**<br>● **Age:** 59 years old<br>● **Sex:** Male<br>**Clinical Presentation:**<br>● **History:** 1-year duration of sensory-motor, symmetrical, non-length dependent polyneuropathy<br>● **Onset:** Subacute onset with progressive course<br>● **Symptoms:**<br>○ Paresthesia<br>○ Neuropathic pain<br>○ Weakness (proximal and distal in lower limbs)<br>○ Appearance of white nails over the last few months<br>**Physical Examination:**<br>● **Findings:**<br>○ No ataxia<br>○ No tremor<br>○ No foot deformities<br>○ No cranial nerve involvement<br>○ No autonomic involvement<br>**Medical History:**<br>● Alcohol abuse<br>**Laboratory Findings:**<br>● **Abnormal:** Thrombocytosis, IgG lambda monoclonal gammopathy<br>● **Normal:**<br>○ Vitamin B12<br>○ Folate<br>○ Vitamin B6<br>○ Vitamin E<br>○ Hepatitis C Virus (HCV)<br>○ Hepatitis B Virus (HBV)<br>○ Antinuclear Antibody (ANA)<br>○ Antineutrophil Cytoplasmic Antibodies (ANCA)<br>○ Extractable Nuclear Antigen (ENA)<br>○ C-Reactive Protein (CRP)<br>○ Erythrocyte Sedimentation Rate (ESR)<br>○ Glycemia<br>○ Hemoglobin A1c (HbA1c)<br>○ Copper<br>○ Cryoglobulins<br>**Nerve Conduction Studies:**<br>● Mixed demyelinating-axonal, sensory-motor polyneuropathy<br>**Family History:**<br>● No family history of polyneuropathy<br>**Differential Diagnoses for Polyneuropathy**<br>1. **Chronic Inflammatory Demyelinating Polyneuropathy (CIDP):**<br>○ **Reasoning:**<br>■ **Subacute onset and progressive course:** Fits well with CIDP, which typically presents with these characteristics.<br>■ **Symmetrical, sensory-motor involvement:** Common in CIDP.<br>■ **Nerve conduction studies:** Showing mixed demyelinating-axonal polyneuropathy is consistent with CIDP.<br>■ **Absence of ataxia, tremor, cranial nerve, and autonomic involvement:** Can be seen in CIDP.<br>■ **IgG lambda monoclonal gammopathy:** Could be related to CIDP as monoclonal gammopathies are sometimes associated with this condition.<br>2. **Monoclonal Gammopathy of Undetermined Significance (MGUS) Associated Neuropathy:**<br>○ **Reasoning:**<br>■ **IgG lambda monoclonal gammopathy:** Highly suggestive of MGUS.<br>■ **Polyneuropathy:** MGUS can lead to a neuropathy that is often similar to CIDP.<br>■ **Subacute onset and progressive course:** Compatible with the presentation of MGUS-associated neuropathy.<br>■ **Nerve conduction studies:** Mixed demyelinating-axonal polyneuropathy can occur in MGUS-related neuropathy.<br>■ **Thrombocytosis:** Can be seen in patients with MGUS.<br>3. **Alcoholic Neuropathy:**<br>○ **Reasoning:**

			■ **History of alcohol abuse:** Strongly predisposes to alcoholic neuropathy.<br>■ **Symmetrical, sensory-motor polyneuropathy:** Commonly seen in alcoholic neuropathy.<br>■ **Subacute onset and progressive course:** Fits the pattern of alcoholic neuropathy, which can develop over months to years.<br>■ **White nails:** Could be indicative of malnutrition, which is common in chronic alcohol abusers.<br>■ **Nerve conduction studies:** Can show mixed demyelinating-axonal features.<br>■ **Absence of ataxia, cranial nerve, and autonomic involvement:** Consistent with alcoholic neuropathy.<br>**Most Likely Diagnosis**<br>Based on the clinical presentation, history, and laboratory findings, the most likely diagnosis is **Monoclonal Gammopathy of Undetermined Significance (MGUS) Associated Neuropathy**. This is supported by the presence of IgG lambda monoclonal gammopathy and the clinical features that align well with MGUS-related neuropathy. Although Chronic Inflammatory Demyelinating Polyneuropathy (CIDP) and alcoholic neuropathy are also strong considerations, the presence of a monoclonal gammopathy tips the balance towards MGUS-associated neuropathy. The thrombocytosis could also be related to the underlying gammopathy rather than being a primary feature of CIDP or alcoholic neuropathy.
24	A 77-year-old male patient presents with a 1-year history of sensory-motor, symmetrical, length dependent polyneuropathy with subacute onset. The patient presents with distal leg paresthesia and neuropathic pain, distal weakness in the upper and lower limbs, without tremor, ataxia, foot deformities, cranial nerve or autonomic involvement. Past medical history shows cutaneous angiomas and IgG lambda monoclonal gammopathy. Nerve conduction studies show a demyelinating, symmetric, sensory-motor polyneuropathy. Screening lab tests including vitamin B12, folate, vitamin B6, vitamin E, HCV, HBV, ANA, ANCA, ENA, CRP, ESR, glycemia, HbA1c, copper and cryoglobulins are all negative, with positivity for IgG lambda paraprotein. There is no family history of polyneuropathy.	POEMS	**Summary of Pertinent Clinical Information:**<br>**Patient Demographics:**<br>● Age: 77 years<br>● Gender: Male<br>**Clinical Presentation:**<br>● Duration: 1 year<br>● Symptoms: Sensory-motor, symmetrical, length-dependent polyneuropathy<br>○ Distal leg paresthesia<br>○ Neuropathic pain<br>○ Distal weakness in upper and lower limbs<br>● Onset: Subacute<br>● Additional Symptoms:<br>○ No tremor<br>○ No ataxia<br>○ No foot deformities<br>○ No cranial nerve involvement<br>○ No autonomic involvement<br>**Past Medical History:**<br>● Cutaneous angiomas<br>● IgG lambda monoclonal gammopathy<br>**Diagnostic Findings:**<br>● Nerve Conduction Studies: Demyelinating, symmetric, sensory-motor polyneuropathy<br>● Screening Lab Tests:<br>○ Negative: Vitamin B12, folate, vitamin B6, vitamin E, HCV, HBV, ANA, ANCA, ENA, CRP, ESR, glycemia, HbA1c, copper, cryoglobulins<br>○ Positive: IgG lambda paraprotein<br>● Family History: No history of polyneuropathy<br>**Differential Diagnoses for Polyneuropathy:**<br>1. **Monoclonal Gammopathy of Undetermined Significance (MGUS) Neuropathy:**<br>○ **Reasoning:**<br>■ The presence of IgG lambda monoclonal gammopathy aligns with MGUS, a common cause of neuropathy in the elderly.<br>■ MGUS can cause a demyelinating, symmetrical, sensory-motor polyneuropathy.<br>■ The absence of systemic features (such as organomegaly, lymphadenopathy) and negative screening tests for other etiologies support this diagnosis.<br>2. **Chronic Inflammatory Demyelinating Polyneuropathy (CIDP):**<br>○ **Reasoning:**<br>■ CIDP often presents with a progressive, symmetric, sensory-motor polyneuropathy and can mimic MGUS neuropathy.<br>■ Demyelination observed in nerve conduction studies is characteristic of CIDP.<br>■ However, CIDP often presents with ataxia and other signs, which are absent in this case. The lack of inflammatory markers (negative CRP, ESR) also argues against this.<br>3. **POEMS Syndrome:**<br>○ **Reasoning:**<br>■ POEMS syndrome (Polyneuropathy, Organomegaly, Endocrinopathy, Monoclonal protein, Skin changes) could be considered given the presence of cutaneous angiomas and monoclonal gammopathy.<br>■ Polyneuropathy in POEMS is usually demyelinating and symmetrical.<br>■ The absence of other POEMS features (organomegaly, endocrinopathy, etc.) makes this less likely but still a consideration.<br>**Most Likely Diagnosis:**<br>**Monoclonal Gammopathy of Undetermined Significance (MGUS) Neuropathy:**<br>● This is the most probable diagnosis due to the strong association between the presence of IgG lambda paraprotein and polyneuropathy.<br>● The clinical presentation, demyelinating nerve conduction study findings, and the absence of systemic symptoms or positive tests for other conditions support MGUS neuropathy over CIDP and POEMS syndrome.<br>● MGUS is a common cause of neuropathy in the elderly, fitting well with the patient's profile and the diagnostic findings.
25	A 78-year-old male patient presents with an 8-month history of sensory-motor, symmetrical, length dependent polyneuropathy with insidious onset and progressive course. The patient presents	anti-MAG polyneuropathy	**Step-by-Step Analysis of the Clinical Scenario**<br>**Clinical Information Summary**<br>● **Patient Profile:**<br>○ Age: 78-year-old male<br>○ Presenting Symptoms:

	with paresthesia, neuropathic pain and weakness distributed distally in the lower limbs. Clinical examination also reveals the presence sensory ataxia. There are no foot deformities, tremor, cranial nerve or autonomic involvement. Past medical history is positive for IgM monoclonal gammopathy. Nerve conduction studies show a demyelinating, symmetric, sensory-motor polyneuropathy. Screening lab tests including vitamin B12, folate, vitamin B6, vitamin E, HCV, HBV, ANA, ANCA, ENA, CRP, ESR, glycemia, HbA1c, copper and cryoglobulins are all negative, with positivity for IgM paraprotein. There is no family history of polyneuropathy.		■ 8-month history of sensory-motor, symmetrical, length-dependent polyneuropathy<br>■ Paresthesia, neuropathic pain, and distal weakness in lower limbs<br>■ Sensory ataxia<br>○ Examination Findings:<br>■ No foot deformities, tremor, cranial nerve, or autonomic involvement<br>○ Past Medical History:<br>■ IgM monoclonal gammopathy<br>● **Diagnostic Findings:**<br>○ Nerve Conduction Studies:<br>■ Demyelinating, symmetric, sensory-motor polyneuropathy<br>○ Screening Lab Tests:<br>■ Negative for vitamin B12, folate, vitamin B6, vitamin E, HCV, HBV, ANA, ANCA, ENA, CRP, ESR, glycemia, HbA1c, copper, and cryoglobulins<br>■ Positive for IgM paraprotein<br>○ No family history of polyneuropathy<br>**Differential Diagnoses**<br>1. **Waldenström Macroglobulinemia (WM):**<br>○ **Reasoning:**<br>■ The presence of IgM monoclonal gammopathy is a significant clue as WM is characterized by an IgM monoclonal gammopathy.<br>■ Patients with WM can develop a demyelinating polyneuropathy.<br>■ Symmetrical, length-dependent sensory-motor polyneuropathy fits with the neuropathic presentation seen in WM.<br>■ Sensory ataxia and the absence of cranial nerve or autonomic involvement further align with neuropathy secondary to WM.<br>2. **Chronic Inflammatory Demyelinating Polyneuropathy (CIDP) with Paraproteinemia:**<br>○ **Reasoning:**<br>■ CIDP often presents with a progressive course of sensory and motor deficits.<br>■ Nerve conduction studies showing demyelination are consistent with CIDP.<br>■ While CIDP typically involves motor and sensory symptoms, the association with IgM monoclonal gammopathy can be seen in a variant known as paraproteinemic demyelinating polyneuropathy.<br>■ The lack of systemic involvement (no cranial nerve or autonomic involvement) is consistent with CIDP.<br>3. **Monoclonal Gammopathy of Undetermined Significance (MGUS)-Associated Neuropathy:**<br>○ **Reasoning:**<br>■ MGUS is commonly associated with IgM paraprotein and can lead to polyneuropathy.<br>■ The neuropathy associated with MGUS can be demyelinating and presents with sensory-motor symptoms, consistent with the patient's presentation.<br>■ This condition can cause sensory ataxia and progressive distal weakness.<br>■ The absence of significant other systemic findings aligns with MGUS, which is often a more benign condition compared to malignancies like WM.<br>**Most Likely Diagnosis**<br>Based on the provided clinical details, the most likely diagnosis is **Waldenström Macroglobulinemia (WM)**. This conclusion is drawn from:<br>● The presence of IgM monoclonal gammopathy.<br>● The demyelinating nature of the polyneuropathy observed in nerve conduction studies.<br>● The insidious onset and progressive course of the sensory-motor symptoms, including sensory ataxia.<br>● The lack of family history, systemic, or autonomic involvement, which are more characteristic of WM-associated neuropathy rather than primary CIDP or MGUS alone.<br>This analysis suggests that further investigations focusing on confirming Waldenström Macroglobulinemia, such as serum viscosity, bone marrow biopsy, and additional hematologic workup, would be appropriate to solidify the diagnosis and guide treatment.
26	An 80-year-old male patient presents with a 2-year history of sensory-motor, asymmetrical, non-length dependent polyneuropathy with subacute onset and a rapid progressive course. The patient presents with paraesthesia, sensory ataxia, neuropathic pain, proximal and distal weakness in the four limbs, and tremor. Clinical examination shows symmetric proximal and distal weakness in the upper and lower limbs, hypoesthesia in a stocking and glove distribution, sensory ataxia, mild intrinsic hand atrophy, and cutaneous angiomas. Clinical examination does not show the presence of foot deformities, cranial nerve, or autonomic involvement. Past medical history is positive for chronic HCV infection. Nerve conduction studies show a mixed demyelinating-axonal, symmetric, sensory-motor polyneuropathy. Screening lab tests reveal the presence of	POEMS	**Step-by-Step Analysis of the Clinical Scenario**<br>**Summary of Pertinent Clinical Information:**<br>1. **Patient Demographics:** 80-year-old male<br>2. **History:**<br>○ Duration: 2 years<br>○ Type: Sensory-motor, asymmetrical, non-length dependent polyneuropathy<br>○ Onset: Subacute with rapid progression<br>3. **Symptoms:**<br>○ Paraesthesia<br>○ Sensory ataxia<br>○ Neuropathic pain<br>○ Proximal and distal weakness in four limbs<br>○ Tremor<br>4. **Clinical Examination:**<br>○ Symmetric proximal and distal weakness in upper and lower limbs<br>○ Hypoesthesia in a stocking and glove distribution<br>○ Sensory ataxia<br>○ Mild intrinsic hand atrophy<br>○ Cutaneous angiomas<br>○ No foot deformities, cranial nerve involvement, or autonomic involvement<br>5. **Past Medical History:**

	polycythaemia, IgM paraprotein, and chronic HCV infection. Remaining lab tests, including vitamin B12, folate, vitamin B6, vitamin E, HBV, ANA, ANCA, ENA, CRP, ESR, copper and cryoglobulins are all negative. There is no family history of polyneuropathy.		○ Chronic Hepatitis C Virus (HCV) infection<br>6. **Investigations:**<br>○ Nerve conduction studies: Mixed demyelinating-axonal, symmetric, sensory-motor polyneuropathy<br>○ Screening lab tests:<br>■ Polycythaemia<br>■ IgM paraprotein<br>■ Chronic HCV infection<br>○ Negative tests: Vitamin B12, folate, vitamin B6, vitamin E, HBV, ANA, ANCA, ENA, CRP, ESR, copper, cryoglobulins<br>○ No family history of polyneuropathy<br>**Differential Diagnoses for Polyneuropathy**<br>1. **Waldenström's Macroglobulinemia (WM):**<br>○ **Reasoning:** The presence of IgM paraprotein suggests a possible monoclonal gammopathy. WM is associated with IgM monoclonal gammopathy and can present with a mixed demyelinating-axonal polyneuropathy. The clinical presentation of symmetric proximal and distal weakness, sensory ataxia, and hypoesthesia fits the profile of neuropathy associated with WM. Additionally, the presence of polycythaemia can be related to the hematologic abnormalities seen in WM.<br>2. **Chronic Inflammatory Demyelinating Polyneuropathy (CIDP):**<br>○ **Reasoning:** CIDP typically presents with a chronic progressive course and can show mixed demyelinating-axonal features on nerve conduction studies. The symmetric proximal and distal weakness, sensory ataxia, and hypoesthesia in a stocking and glove distribution are characteristic of CIDP. However, the presence of IgM paraprotein might suggest a paraproteinemic neuropathy, which is a variant of CIDP.<br>3. **Neuropathy Associated with Hepatitis C Virus (HCV):**<br>○ **Reasoning:** Chronic HCV infection can lead to mixed sensory-motor polyneuropathy. HCV-associated neuropathy often presents with a similar clinical picture, including sensory disturbances, ataxia, and motor weakness. The presence of chronic HCV infection in the patient's history supports this diagnosis. However, the absence of cryoglobulins is a notable point, as cryoglobulinemia is often associated with HCV-related neuropathy.<br>**Most Likely Diagnosis**<br>**Waldenström's Macroglobulinemia (WM)**<br>● **Reasoning:** The presence of IgM paraprotein, along with the clinical presentation of mixed demyelinating-axonal polyneuropathy, symmetric weakness, sensory ataxia, and neuropathic pain, strongly points towards WM. The cutaneous angiomas and polycythaemia further support this diagnosis as WM can present with such hematologic and skin manifestations. Although CIDP and HCV-associated neuropathy are also considerations, the paraproteinemia (IgM) and associated clinical findings make WM the most probable diagnosis.<br>**Conclusion**<br>Waldenström's Macroglobulinemia is the most likely diagnosis given the patient's clinical presentation, nerve conduction study findings, presence of IgM paraprotein, and associated systemic features. Further hematologic evaluation and possibly a bone marrow biopsy would be warranted to confirm the diagnosis and initiate appropriate treatment.
27	A 76-year-old female patient presents with a three-year history of pure sensory, symmetrical, length-dependent polyneuropathy with insidious onset and progressive course. The patient reports paraesthesia, severe neuropathic pain, and cramps in the feet in a symmetric distribution, which began approximately three years ago but significantly worsened after the third cycle of chemotherapy. Clinical examination does not reveal ataxia, tremor, foot deformities, cranial nerve or autonomic dysfunction. Past medical history is positive for type 2 diabetes, non-Hodgkin lymphoma, and breast cancer, for which she has undergone chemotherapy. Nerve conduction studies show a sensorimotor, length-dependent, symmetric, axonal polyneuropathy. Screening lab tests including vitamin B12, folate, vitamin B6, vitamin E, HCV, ANA, ANCA, ENA, CRP, ESR, copper, cryoglobulins, and serum electrophoresis are all negative. However, there is evidence of hyperglycemia, elevated HbA1c, and chronic HBV infection. There is no family history of polyneuropathy.	Superimposed chemotherapy-induced neuropathy on diabetic polyneuropathy	**Step-by-Step Analysis of the Clinical Scenario**<br>**Summary of Pertinent Clinical Information**<br>● **Patient Profile:** 76-year-old female<br>● **Duration of Symptoms:** Three years<br>● **Symptom Type:** Pure sensory, symmetrical, length-dependent polyneuropathy<br>● **Symptom Onset and Course:** Insidious onset and progressive course, worsened post-chemotherapy<br>● **Symptoms Reported:** Paraesthesia, severe neuropathic pain, cramps in the feet<br>● **Distribution of Symptoms:** Symmetric<br>● **Clinical Examination Findings:**<br>○ No ataxia, tremor, foot deformities, cranial nerve, or autonomic dysfunction<br>● **Past Medical History:**<br>○ Type 2 diabetes<br>○ Non-Hodgkin lymphoma<br>○ Breast cancer treated with chemotherapy<br>● **Nerve Conduction Studies:** Sensorimotor, length-dependent, symmetric, axonal polyneuropathy<br>● **Screening Lab Tests:** Negative for various potential causes (e.g., vitamin deficiencies, autoimmune markers)<br>● **Notable Lab Findings:**<br>○ Hyperglycemia<br>○ Elevated HbA1c<br>○ Chronic HBV infection<br>● **Family History:** No family history of polyneuropathy<br>**Differential Diagnoses for Polyneuropathy**<br>1. **Diabetic Polyneuropathy**<br>○ **Reasoning:**<br>■ **Diabetes Mellitus:** The patient has a history of type 2 diabetes, which is a common cause of length-dependent, symmetrical polyneuropathy.<br>■ **Hyperglycemia and Elevated HbA1c:** Lab results show hyperglycemia and elevated HbA1c, indicating poor glycemic control, which is a risk factor for developing diabetic neuropathy.<br>■ **Symptomatology:** The gradual onset and progression of sensory symptoms such as paraesthesia, neuropathic pain, and cramps are typical for diabetic polyneuropathy.<br>○ **Supporting Features:** Chronic hyperglycemia, lack of other definitive causes found in screening tests.<br>2. **Chemotherapy-Induced Peripheral Neuropathy (CIPN)**<br>○ **Reasoning:**<br>■ **Chemotherapy History:** The patient has a history of chemotherapy for breast cancer, which is a known risk factor for developing CIPN.<br>■ **Temporal Relationship:** Symptoms significantly worsened after the third cycle of chemotherapy, suggesting a correlation between chemotherapy and neuropathy.<br>■ **Typical Presentation:** CIPN often presents as a length-dependent, symmetric, sensory polyneuropathy, aligning with the patient's symptoms.<br>○ **Supporting Features:** Clear temporal association with chemotherapy, typical clinical presentation.<br>3. **HBV-Associated Peripheral Neuropathy**

			○ **Reasoning:**<br>■ **Chronic HBV Infection:** The patient has chronic HBV infection, which can sometimes lead to neuropathy, though less common.<br>■ **Pathophysiology:** HBV can lead to neuropathy through immune-mediated mechanisms or direct viral damage to nerves.<br>■ **Clinical Features:** Though less common, HBV-associated neuropathy can present similarly to the patient's symptomatology.<br>○ **Supporting Features:** Presence of chronic HBV infection, negative results for other potential causes.<br>**Determining the Most Likely Diagnosis**<br>Based on the provided clinical scenario and the differential diagnoses discussed, the most likely diagnosis is **Diabetic Polyneuropathy**. This conclusion is supported by the following points:<br>● **Prevalence and Likelihood:** Diabetic neuropathy is a common complication of diabetes, especially with poor glycemic control, as indicated by the patient's hyperglycemia and elevated HbA1c.<br>● **Symptom Consistency:** The patient's symptoms are typical for diabetic polyneuropathy, including the gradual onset, progression, and symmetric distribution of sensory disturbances.<br>● **Exclusion of Other Causes:** Other potential causes such as vitamin deficiencies, autoimmune conditions, and paraproteinemias have been ruled out through screening lab tests.<br>Therefore, the clinical presentation, combined with the patient's history of diabetes and laboratory findings, strongly suggests that diabetic polyneuropathy is the most likely diagnosis.
28	A 37-year-old male patient presents with a 2-week history of sensory-motor, symmetrical, non-length dependent polyneuropathy with acute onset. The patient presents with distal paresthesia in the upper and lower limbs, neuropathic pain, and weakness in the proximal and distal areas of the upper and lower limbs. There are no ataxia, tremor, foot deformities, cranial nerve or autonomic involvement. Past medical history is silent. Nerve conduction studies show a demyelinating, sensory-motor polyneuropathy. Screening lab tests including vitamin B12, folate, vitamin B6, vitamin E, HCV, HBV, ANA, ANCA, ENA, CRP, ESR, glycemia, HbA1c, copper, cryoglobulins and serum electrophoresis are all negative. There is no family history of polyneuropathy.	GBS	**Summary of Pertinent Clinical Information**<br>● **Patient**: 37-year-old male<br>● **Presenting Symptoms**:<br>○ 2-week history of sensory-motor, symmetrical, non-length dependent polyneuropathy<br>○ Acute onset<br>○ Distal paresthesia in upper and lower limbs<br>○ Neuropathic pain<br>○ Weakness in proximal and distal areas of upper and lower limbs<br>● **Negative Symptoms**:<br>○ No ataxia, tremor, foot deformities, cranial nerve involvement, or autonomic involvement<br>● **Past Medical History**: Unremarkable<br>● **Nerve Conduction Studies**: Demyelinating, sensory-motor polyneuropathy<br>● **Screening Lab Tests**: Negative (vitamin B12, folate, vitamin B6, vitamin E, HCV, HBV, ANA, ANCA, ENA, CRP, ESR, glycemia, HbA1c, copper, cryoglobulins, serum electrophoresis)<br>● **Family History**: No family history of polyneuropathy<br>**Differential Diagnoses for Polyneuropathy**<br>**1. Acute Inflammatory Demyelinating Polyradiculoneuropathy (AIDP) - Guillain-Barré Syndrome (GBS)**<br>● **Reasoning**:<br>○ **Acute Onset**: The 2-week acute onset of symptoms fits the typical presentation of GBS.<br>○ **Symmetry**: Symmetrical distribution is characteristic of GBS.<br>○ **Sensory-Motor Involvement**: Both sensory and motor nerves are affected in GBS, which is consistent with the patient's presentation.<br>○ **Demyelinating Pattern**: Nerve conduction studies showing demyelination align with GBS.<br>○ **Absence of Cranial Nerve Involvement**: While cranial nerve involvement can occur, its absence does not exclude GBS.<br>○ **Lack of Autonomic Involvement**: Not all GBS patients present with autonomic dysfunction, especially early in the course.<br>○ **Negative Labs**: Screening tests being negative is consistent as GBS is not diagnosed through these tests but through clinical presentation and electrodiagnostic studies.<br>○ **No Ataxia/Tremor**: Fits typical GBS presentation.<br>**2. Chronic Inflammatory Demyelinating Polyradiculoneuropathy (CIDP)**<br>● **Reasoning**:<br>○ **Sensory-Motor Involvement**: Similar to GBS, CIDP affects both sensory and motor nerves.<br>○ **Demyelinating Pattern**: Nerve conduction studies consistent with demyelination fit CIDP.<br>○ **Symmetrical Distribution**: The symmetry is also characteristic of CIDP.<br>○ **Absence of Systemic Signs**: Negative lab results and lack of systemic involvement (no ataxia, tremor) are consistent with CIDP.<br>○ **Acute Onset**: CIDP can present acutely, though it typically has a more chronic, progressive course. The acute presentation might lean more towards AIDP but does not exclude CIDP entirely.<br>**3. Multifocal Motor Neuropathy (MMN)**<br>● **Reasoning**:<br>○ **Motor Involvement**: Predominantly motor involvement, though typically not sensory, MMN could still present similarly.<br>○ **Demyelinating Pattern**: Demyelination seen in nerve conduction studies can be seen in MMN.<br>○ **Symmetry**: MMN often presents asymmetrically, which is a point against it, but the demyelinating nature and motor involvement can still keep it in consideration.<br>○ **Lack of Systemic Involvement**: The lack of autonomic and cranial nerve involvement aligns with MMN, though the presence of sensory symptoms is less typical.<br>○ **Negative Labs**: Similar to the other conditions, MMN would not necessarily be identified through the lab tests performed.<br>**Most Likely Diagnosis**<br>The most likely diagnosis, considering the acute onset, symmetrical sensory-motor involvement, and demyelinating nature of the polyneuropathy, is **Acute Inflammatory Demyelinating Polyradiculoneuropathy (AIDP) - Guillain-Barré Syndrome (GBS)**.<br>GBS is the most consistent with the rapid progression and the electrodiagnostic findings. The absence of systemic and cranial nerve involvement, as well as the negative laboratory workup, supports this diagnosis, as these are often typical in early GBS. The absence of ataxia and tremor also fits the GBS profile more than CIDP, which tends to have a more insidious onset and chronic progression.
29	A 75-year-old male patient presents with a 4-year history of pure sensory, symmetrical, length dependent polyneuropathy with insidious onset and slow progressive course. The patient presents with paresthesia and neuropathic pain in the feet. Clinical examination does not showtremor or sensory ataxia. There	Diabetic neuropathy	**Summary of Pertinent Clinical Information:**<br>● **Patient Profile**: 75-year-old male<br>● **History of Present Illness**:<br>○ 4-year history of pure sensory, symmetrical, length-dependent polyneuropathy<br>○ Insidious onset with a slow progressive course<br>○ Symptoms: Paresthesia and neuropathic pain in the feet<br>● **Clinical Examination**:<br>○ No tremor or sensory ataxia

	are no signs of foot deformities, cranial nerve or autonomic involvement. Past medical history is positive for type 2 diabetes and arterial hypertension. Nerve conduction studies show a mixed sensory-motor, length dependent, axonal polyneuropathy. Screening lab tests reveal hyperglycaemia and elevated HbA1c. Remaining exams including vitamin B12, vitamin B6, vitamin E, HBV, HCV, ANA, ANCA, ENA, CRP, ESR, copper, cryoglobulin, and serum electrophoresis are normal. There is no family history of polyneuropathy.		○ No foot deformities, cranial nerve, or autonomic involvement<br>● **Past Medical History**:<br>○ Type 2 diabetes mellitus<br>○ Arterial hypertension<br>● **Nerve Conduction Studies**:<br>○ Mixed sensory-motor, length-dependent, axonal polyneuropathy<br>● **Laboratory Findings**:<br>○ Hyperglycaemia<br>○ Elevated HbA1c<br>○ Normal levels of vitamin B12, B6, E, HBV, HCV, ANA, ANCA, ENA, CRP, ESR, copper, cryoglobulin, and serum electrophoresis<br>● **Family History**:<br>○ No family history of polyneuropathy<br>**Differential Diagnoses for Polyneuropathy:**<br>1. **Diabetic Polyneuropathy (DPN)**:<br>○ **Reasoning**:<br>■ The patient has a history of type 2 diabetes, which is the most common cause of peripheral neuropathy in developed countries.<br>■ Hyperglycaemia and elevated HbA1c levels indicate poorly controlled diabetes, which is a known risk factor for developing diabetic neuropathy.<br>■ The pattern of symmetrical, length-dependent, axonal polyneuropathy is characteristic of diabetic neuropathy.<br>■ The absence of autonomic involvement and the presence of purely sensory symptoms with slow progression further support this diagnosis.<br>2. **Chronic Idiopathic Axonal Polyneuropathy (CIAP)**:<br>○ **Reasoning**:<br>■ CIAP typically presents in older adults with a slowly progressive, symmetrical, sensory-predominant neuropathy.<br>■ It is a diagnosis of exclusion when other causes of polyneuropathy are ruled out.<br>■ Normal results of extensive screening tests, including vitamins, infections, autoimmune markers, and serum electrophoresis, could suggest CIAP as a possible diagnosis, especially if no secondary causes are identified.<br>■ However, the presence of diabetes makes this less likely the primary cause.<br>3. **Alcohol-Related Neuropathy**:<br>○ **Reasoning**:<br>■ Chronic alcohol consumption can lead to a similar pattern of neuropathy, typically presenting as a symmetrical, length-dependent, sensory-predominant polyneuropathy.<br>■ However, there is no mention of alcohol use in the patient's history. If there were a history of significant alcohol consumption, it could be considered.<br>■ The absence of other indicative factors in the patient's history makes this diagnosis less probable compared to the other differentials.<br>**Most Likely Diagnosis:**<br>**Diabetic Polyneuropathy (DPN)** is the most likely diagnosis. The patient's history of type 2 diabetes, combined with the clinical presentation of a length-dependent, symmetrical sensory polyneuropathy, supports this diagnosis. The elevated HbA1c indicates poor glycemic control, which is a significant risk factor for the development and progression of diabetic neuropathy. The exclusion of other potential causes through normal laboratory results further strengthens the likelihood of diabetic polyneuropathy as the primary diagnosis.
30	A 60-year-old male patient presents with a 17-month history of sensory, symmetrical, length dependent polyneuropathy with an insidious onset and a slow progressive course. The first occurred symptom was neuropathic pain in the feet, followed by erectile dysfunction, hyperhidrosis and mild distal paraesthesia. Clinical examination does not show presence of sensory ataxia, tremor or foot deformities. Past medical history is silent. Nerve conduction studies show a mixed sensory-motor, symmetric, axonal polyneuropathy and bilateral carpal tunnel syndrome. Screening lab tests including vitamin B12, folate, vitamin B6, vitamin E, HBV, HCV, ANA, ANCA, ENA, CRP, ESR, glycemia, HbA1c, copper, and cryoglobulins are normal. Serum electrophoresis shows presence of IgG lambda paraprotein. There is no family history of polyneuropathy.	Amyloidosis associated polyneuropathy	**Summary of Pertinent Clinical Information**<br>● **Patient Demographics**: 60-year-old male<br>● **Chief Complaint**: 17-month history of sensory, symmetrical, length-dependent polyneuropathy<br>● **Symptom Onset and Course**: Insidious onset with a slow progressive course<br>● **Initial Symptoms**: Neuropathic pain in the feet<br>● **Subsequent Symptoms**: Erectile dysfunction, hyperhidrosis, mild distal paraesthesia<br>● **Clinical Examination Findings**: No sensory ataxia, tremor, or foot deformities<br>● **Past Medical History**: Unremarkable<br>● **Nerve Conduction Studies**: Mixed sensory-motor, symmetric, axonal polyneuropathy; bilateral carpal tunnel syndrome<br>● **Laboratory Results**:<br>○ Normal: Vitamin B12, folate, vitamin B6, vitamin E, HBV, HCV, ANA, ANCA, ENA, CRP, ESR, glycemia, HbA1c, copper, and cryoglobulins<br>○ Abnormal: Serum electrophoresis shows IgG lambda paraprotein<br>● **Family History**: No family history of polyneuropathy<br>**Differential Diagnoses for Polyneuropathy**<br>**1. Monoclonal Gammopathy of Undetermined Significance (MGUS)**<br>● **Reasoning**:<br>○ Presence of IgG lambda paraprotein suggests a monoclonal gammopathy.<br>○ MGUS is a known cause of axonal polyneuropathy, often presenting with similar sensory and motor symptoms.<br>○ The progression and lack of other significant systemic symptoms align with typical MGUS-associated neuropathy.<br>○ Bilateral carpal tunnel syndrome can be associated with MGUS.<br>**2. Light Chain (AL) Amyloidosis**<br>● **Reasoning**:<br>○ Amyloidosis can present with neuropathy and autonomic dysfunction, which fits the patient's symptoms of erectile dysfunction and hyperhidrosis.<br>○ The presence of a paraprotein (IgG lambda) raises suspicion for a possible plasma cell dyscrasia like AL amyloidosis.<br>○ Neuropathic pain, mild distal paraesthesia, and autonomic symptoms are common in AL amyloidosis.<br>○ Axonal type neuropathy is typical in AL amyloidosis, and the slow progression fits the clinical picture.<br>**3. Chronic Inflammatory Demyelinating Polyneuropathy (CIDP)**

			● **Reasoning**:<br>○ Although less likely due to the predominantly axonal findings on nerve conduction studies, CIDP can present with a mixed sensory-motor neuropathy.<br>○ CIDP can sometimes be associated with paraproteinemia, particularly IgG types.<br>○ The chronic progressive nature and the mixed neuropathic pain and motor symptoms are consistent with CIDP.<br>○ The absence of response to initial anti-inflammatory treatments (not mentioned but inferred from progression) could steer away from this diagnosis.<br>**Most Likely Diagnosis**<br>Given the clinical presentation, laboratory findings, and the progression of symptoms, the most likely diagnosis is **Monoclonal Gammopathy of Undetermined Significance (MGUS) with associated polyneuropathy**.<br>**Reasoning:**<br>● The detection of IgG lambda paraprotein strongly supports a diagnosis related to monoclonal gammopathy.<br>● The clinical presentation of a slowly progressive, symmetric, sensory-motor, axonal polyneuropathy aligns well with MGUS-associated neuropathy.<br>● The absence of significant systemic symptoms or more severe manifestations that would be expected in conditions like AL amyloidosis makes MGUS more likely.<br>● The negative results for other common causes of neuropathy (e.g., vitamin deficiencies, infections, autoimmune markers) further support this diagnosis.<br>Further evaluation, including bone marrow biopsy and more specific tests for amyloid, may be warranted to rule out other plasma cell dyscrasias or amyloidosis conclusively.
31	A 54-year-old male patient presents with a 2-year history of chronic, pure sensory, symmetrical, length dependent polyneuropathy with insidious onset. The patient presents with distal paresthesia and neuropathic pain in the lower limbs, without ataxia. Past medical history reveals high alcohol consumption. Clinical examination reveals absence of foot deformities, tremor, cranial nerve or autonomic involvement. Nerve conduction studies show a length-dependent polyneuropathy with symmetric, pure sensory, axonal involvement. Screening lab tests including vitamin B12, folate, vitamin B6, vitamin E, HCV, HBV, ANA, ANCA, ENA, CRP, ESR, glycemia, HbA1c, copper, cryoglobulins and serum electrophoresis are all negative. Serum gamma-glutamyl transferase (GGT) levels are elevated. There is no family history of polyneuropathy.	Alcohol-associated neuropathy	**Clinical Information Summary:**<br>● **Patient Profile:** 54-year-old male<br>● **Duration of Symptoms:** 2 years<br>● **Symptom Description:** Chronic, pure sensory, symmetrical, length-dependent polyneuropathy with distal paresthesia and neuropathic pain in lower limbs.<br>● **Onset:** Insidious<br>● **Past Medical History:** High alcohol consumption<br>● **Clinical Examination:**<br>○ No foot deformities<br>○ No tremor<br>○ No cranial nerve involvement<br>○ No autonomic involvement<br>● **Nerve Conduction Studies:** Symmetric, pure sensory, axonal involvement in a length-dependent pattern.<br>● **Screening Lab Tests:**<br>○ Negative for vitamin B12, folate, vitamin B6, vitamin E, HCV, HBV, ANA, ANCA, ENA, CRP, ESR, glycemia, HbA1c, copper, cryoglobulins, and serum electrophoresis.<br>○ Elevated serum gamma-glutamyl transferase (GGT) levels.<br>● **Family History:** No family history of polyneuropathy.<br>**Differential Diagnoses:**<br>1. **Alcoholic Neuropathy:**<br>○ **Reasoning:**<br>■ The patient's history of high alcohol consumption is a significant risk factor for the development of alcoholic neuropathy.<br>■ Alcoholic neuropathy typically presents as a length-dependent, symmetric polyneuropathy, often with sensory symptoms predominating, which matches the clinical presentation.<br>■ Elevated GGT levels are consistent with chronic alcohol consumption and associated liver enzyme changes.<br>■ The absence of other systemic findings and the exclusion of vitamin deficiencies (B12, folate, B6, E) support this diagnosis.<br>2. **Idiopathic Sensory Polyneuropathy:**<br>○ **Reasoning:**<br>■ When no clear cause is identified, idiopathic sensory polyneuropathy is a common diagnosis.<br>■ This condition often presents similarly with insidious onset, length-dependent sensory symptoms, and absence of motor involvement.<br>■ Comprehensive screening tests being negative further support this diagnosis, suggesting no underlying metabolic, infectious, or inflammatory etiology.<br>■ However, given the patient's significant alcohol use, this diagnosis is less likely compared to alcoholic neuropathy but remains a consideration if no other cause is identified.<br>3. **Chronic Inflammatory Demyelinating Polyneuropathy (CIDP) - Sensory Variant:**<br>○ **Reasoning:**<br>■ CIDP can present as a pure sensory neuropathy, although this is less common.<br>■ The chronic nature and length-dependent pattern could fit, but CIDP often involves some degree of motor involvement, which is not present in this patient.<br>■ The absence of inflammatory markers and the negative autoantibody tests (ANA, ANCA, ENA) make this less likely.<br>■ Despite nerve conduction studies indicating axonal involvement, a sensory variant of CIDP remains a differential due to its chronic progressive nature, although it is lower on the list.<br>**Most Likely Diagnosis:**<br>**Alcoholic Neuropathy**<br>● **Reasoning:** The patient's history of high alcohol consumption is a strong risk factor and fits the clinical presentation perfectly. The absence of other contributory factors, normal screening tests for alternative causes, and elevated GGT levels support this diagnosis. Alcoholic neuropathy typically manifests as a chronic, length-dependent sensory polyneuropathy, aligning with the patient's symptoms and examination findings.<br>**Conclusion:**<br>The most likely diagnosis for this patient with a chronic, pure sensory, symmetrical, length-dependent polyneuropathy and a significant history of alcohol consumption is alcoholic neuropathy. The clinical findings and supportive lab results align well with this condition, making it the most probable diagnosis.
32	An 80-year-old male patient presents with a 3-year history of neuropathic pain and skin redness in the hands and feet. Symptoms started insidiously but progressed over the years. Clinical examination reveals hyperalgesia and allodynia in the hands and feet, where the skin is warm and red. There are no	Small fibre neuropathy	**Summary of Pertinent Clinical Information:**<br>● **Patient:** 80-year-old male<br>● **Symptoms:**<br>○ Neuropathic pain and skin redness in hands and feet for 3 years<br>○ Symptoms progressed insidiously<br>● **Clinical Findings:**<br>○ Hyperalgesia and allodynia in hands and feet<br>○ Warm and red skin in affected areas

	weakness, deficit in tactile sensation, sensory ataxia, tremor, foot deformities, cranial nerve or autonomic involvement. Past medical history is positive for prostate cancer and chronic renal insufficiency. Nerve conduction studies are normal. Screening lab tests including vitamin B12, folate, vitamin B6, vitamin E, HBV, HCV, ANA, ANCA, ENA, CRP, ESR, glycemia, HbA1c, copper, cryoglobulins and serum electrophoresis are negative. There is no family history of polyneuropathy.		○ No weakness, tactile sensation deficit, sensory ataxia, tremor, foot deformities, cranial nerve involvement, or autonomic involvement<br>● **Medical History:**<br>○ Prostate cancer<br>○ Chronic renal insufficiency<br>● **Investigations:**<br>○ Normal nerve conduction studies<br>○ Negative screening lab tests (including B12, folate, B6, vitamin E, HBV, HCV, ANA, ANCA, ENA, CRP, ESR, glycemia, HbA1c, copper, cryoglobulins, and serum electrophoresis)<br>● **Family History:** Negative for polyneuropathy<br>**Differential Diagnoses:**<br>1. **Erythromelalgia:**<br>○ **Reasoning:**<br>■ Erythromelalgia is characterized by episodic burning pain, redness, and warmth in the extremities, often triggered by heat or exercise. The patient's description of neuropathic pain and skin redness, along with the absence of sensory or motor deficits, aligns well with erythromelalgia.<br>■ The progression of symptoms over years and the finding of hyperalgesia and allodynia further support this diagnosis. Chronic renal insufficiency can sometimes be associated with erythromelalgia.<br>2. **Small Fiber Neuropathy (SFN):**<br>○ **Reasoning:**<br>■ SFN typically presents with pain, burning sensations, and autonomic symptoms due to damage to small Aδ and C fibers. The patient's neuropathic pain and hyperalgesia/allodynia without large fiber involvement (normal nerve conduction studies) fit the presentation of SFN.<br>■ Despite the absence of autonomic symptoms, the warmth and redness of the skin could be due to the involvement of small autonomic fibers. Chronic renal insufficiency is a recognized cause of SFN, further supporting this possibility.<br>3. **Paraneoplastic Neuropathy:**<br>○ **Reasoning:**<br>■ Given the patient's history of prostate cancer, paraneoplastic neuropathy must be considered. This condition can present with various neurological symptoms, including neuropathic pain.<br>■ The absence of motor deficits and normal nerve conduction studies do not rule out this diagnosis, as paraneoplastic syndromes can affect small fibers primarily.<br>■ However, the absence of autonomic involvement and the specific presentation of warmth and redness are less typical but still within the realm of paraneoplastic phenomena.<br>**Most Likely Diagnosis:**<br>**Erythromelalgia** is the most likely diagnosis for this patient. The key features aligning with this condition include the chronic, progressive nature of neuropathic pain, the redness, warmth in hands and feet, and the absence of sensory or motor deficits. The patient's chronic renal insufficiency might be a contributing factor. Additionally, erythromelalgia often goes undiagnosed or misdiagnosed due to its rarity and nonspecific symptoms, which could explain the lack of findings in extensive lab tests and normal nerve conduction studies.
33	A 24-year old male patient presents with a 4-year history of sensory-motor, symmetrical, length dependent polyneuropathy with insidious onset and slow progression. The patient presents with severe walking difficulties, bilateral foot-drop, severe distal atrophy in the lower limbs, and hypoestesia up to calves. There is no cranial nerve or autonomic involvement. Past medical history is silent. Nerve conduction studies show a mixed axonal-demyelinating, symmetric, sensory-motor polyneuropathy. Screening lab tests including vitamin B12, folate, vitamin B6, vitamin E, HBV, HCV, glycemia, HbA1c, ANA, ANCA, ENA, CRP, ESR, copper, cryoglobulins, and serum electrophoresis are all negative. His mother and sister have mild walking difficulties.	X-linked CMT	**Summary of Pertinent Clinical Information:**<br>● **Duration and Onset:** 4-year history<br>● **Type:** Sensory-motor, symmetrical, length-dependent polyneuropathy<br>● **Onset:** Insidious<br>● **Progression:** Slow<br>● **Symptoms:**<br>○ Severe walking difficulties<br>○ Bilateral foot-drop<br>○ Severe distal atrophy in lower limbs<br>○ Hypoesthesia up to calves<br>● **Exclusions:** No cranial nerve or autonomic involvement<br>● **Past Medical History:** Silent<br>● **Nerve Conduction Studies:** Mixed axonal-demyelinating, symmetric, sensory-motor polyneuropathy<br>● **Screening Lab Tests:** Negative (including vitamin B12, folate, vitamin B6, vitamin E, HBV, HCV, glycemia, HbA1c, ANA, ANCA, ENA, CRP, ESR, copper, cryoglobulins, and serum electrophoresis)<br>● **Family History:** Mother and sister have mild walking difficulties<br>**Differential Diagnoses for Polyneuropathy:**<br>1. **Hereditary Neuropathy (Charcot-Marie-Tooth Disease - CMT):**<br>○ **Reasoning:** The patient's clinical presentation is highly suggestive of a hereditary neuropathy. The insidious onset, slow progression, and family history of similar symptoms in the mother and sister point towards a genetic etiology. CMT is the most common inherited peripheral neuropathy and can present with a mixed axonal and demyelinating pattern, as seen in this patient.<br>○ **Features Supporting Diagnosis:**<br>■ Symmetrical, length-dependent polyneuropathy<br>■ Severe walking difficulties and foot-drop<br>■ Distal muscle atrophy<br>■ Family history of walking difficulties<br>■ Mixed axonal-demyelinating pattern on nerve conduction studies<br>2. **Chronic Inflammatory Demyelinating Polyneuropathy (CIDP):**<br>○ **Reasoning:** CIDP is an acquired immune-mediated disorder that could present similarly, with a chronic, progressive course and both motor and sensory involvement. However, the family history is less suggestive of CIDP. The mixed axonal-demyelinating pattern could fit with CIDP, but the absence of cranial nerve or autonomic involvement is less typical.<br>○ **Features Supporting Diagnosis:**<br>■ Chronic, progressive nature<br>■ Symmetric sensory-motor involvement<br>■ Mixed axonal-demyelinating features on nerve conduction studies<br>○ **Features Against Diagnosis:**<br>■ Lack of typical inflammatory markers or evidence in lab tests

			■ Negative extensive screening tests<br>■ Family history pointing more towards hereditary etiology<br>3. **Idiopathic Axonal Polyneuropathy:**<br>○ **Reasoning:** While less likely given the family history, idiopathic axonal polyneuropathy could be considered due to the chronic, progressive nature of the symptoms and the mixed axonal-demyelinating pattern on nerve conduction studies. However, the absence of any underlying systemic or metabolic disorder makes this diagnosis less probable.<br>○ **Features Supporting Diagnosis:**<br>■ Chronic, progressive symptoms<br>■ Mixed axonal-demyelinating features on nerve conduction studies<br>○ **Features Against Diagnosis:**<br>■ Strong family history of similar symptoms<br>■ Negative lab tests for common causes of polyneuropathy<br>**Most Likely Diagnosis:**<br>The most likely diagnosis is **Hereditary Neuropathy, specifically Charcot-Marie-Tooth Disease (CMT)**. The combination of the clinical presentation, nerve conduction study findings, and strong family history strongly supports a hereditary neuropathy. The mixed axonal-demyelinating pattern is consistent with some forms of CMT, and the absence of positive findings in the extensive screening for other causes of polyneuropathy further points towards this diagnosis. Genetic testing would be the next step to confirm this diagnosis and identify the specific subtype of CMT.
34	An 83-year-old male patient presents with a 1-year history of pure sensory, symmetrical, length dependent polyneuropathy with insidious onset and progressive course. The patient presents with distal paresthesia and hypoesthesia in the four limbs, mild hand tremor and cramps but no neuropathic pain. He also reports the appearance of gait instability in the past few months. Clinical examination confirms the presence of sensory ataxia with difficulty walking without support. There are no foot deformities, cranial nerve or autonomic involvement. Past medical history shows IgG kappa monoclonal gammopathy, thrombocytopenia, and prostatic cancer. Nerve conduction studies show a pure sensory, symmetric, length-dependent, axonal polyneuropathy. Screening lab tests show the presence of IgG kappa paraprotein and evidence of vitamin B12 deficiency. Remaining exams including folate, vitamin B6, vitamin E, HCV, HBV, ANA, ENA, ANCA, CRP, ESR, glycemia, HbA1c, copper and cryoglobulins are all negative. There is no family history of polyneuropathy.	Vit B12 deficiency neuropathy	**Summary of Pertinent Clinical Information**<br>**Patient Profile:**<br>● **Age:** 83-year-old male<br>● **Chief Complaint:** 1-year history of pure sensory, symmetrical, length-dependent polyneuropathy with insidious onset and progressive course<br>● **Symptoms:**<br>○ Distal paresthesia and hypoesthesia in the four limbs<br>○ Mild hand tremor and cramps<br>○ Gait instability (recent onset)<br>● **Clinical Examination:**<br>○ Sensory ataxia<br>○ Difficulty walking without support<br>○ No foot deformities<br>○ No cranial nerve involvement<br>○ No autonomic involvement<br>● **Medical History:**<br>○ IgG kappa monoclonal gammopathy<br>○ Thrombocytopenia<br>○ Prostatic cancer<br>● **Nerve Conduction Studies:** Pure sensory, symmetric, length-dependent, axonal polyneuropathy<br>● **Lab Tests:**<br>○ Positive: IgG kappa paraprotein, Vitamin B12 deficiency<br>○ Negative: Folate, Vitamin B6, Vitamin E, HCV, HBV, ANA, ENA, ANCA, CRP, ESR, glycemia, HbA1c, copper, cryoglobulins<br>● **Family History:** No family history of polyneuropathy<br>**Differential Diagnoses for Polyneuropathy**<br>1. **Monoclonal Gammopathy of Undetermined Significance (MGUS)-related Polyneuropathy:**<br>○ **Reasoning:**<br>■ The presence of IgG kappa monoclonal gammopathy in the patient's history and screening lab tests is a significant clue. MGUS can lead to a paraneoplastic syndrome affecting peripheral nerves.<br>■ Sensory, symmetric, length-dependent, axonal polyneuropathy is consistent with neuropathies seen in MGUS.<br>■ The absence of autonomic and motor symptoms aligns with the typical presentation of MGUS-related neuropathy, which is predominantly sensory.<br>■ No other systemic features that might suggest an alternative etiology, like multiple myeloma or Waldenström's macroglobulinemia, have been presented.<br>2. **Vitamin B12 Deficiency-related Polyneuropathy:**<br>○ **Reasoning:**<br>■ Vitamin B12 deficiency is confirmed by lab tests and is a known cause of polyneuropathy.<br>■ This condition typically presents with symmetrical, length-dependent sensory deficits, often with additional symptoms like gait instability and sensory ataxia.<br>■ The patient's sensory ataxia and difficulty walking without support are hallmark symptoms of B12 deficiency-related neuropathy.<br>■ However, Vitamin B12 deficiency often presents with more pronounced neurologic deficits and possibly with macrocytic anemia, which is not mentioned in the case.<br>3. **Paraneoplastic Neuropathy:**<br>○ **Reasoning:**<br>■ The patient's history of prostatic cancer raises the possibility of a paraneoplastic syndrome, which can cause peripheral neuropathy.<br>■ Paraneoplastic neuropathies can present as sensory polyneuropathy with a progressive course.<br>■ Although less common, prostate cancer can be associated with paraneoplastic syndromes that might cause polyneuropathy.<br>■ The absence of other paraneoplastic symptoms or markers makes this less likely but still plausible considering the patient's cancer history.<br>**Most Likely Diagnosis**<br>Given the patient's history, clinical presentation, and laboratory findings, the most likely diagnosis is **Monoclonal Gammopathy of Undetermined Significance (MGUS)-related Polyneuropathy**.<br>**Justification:**<br>● The presence of IgG kappa monoclonal gammopathy aligns with MGUS.<br>● The pattern of neuropathy (sensory, symmetric, length-dependent, and axonal) fits well with MGUS-related neuropathy.<br>● The progression of symptoms and the lack of autonomic involvement are typical for MGUS-associated neuropathy.

			● Vitamin B12 deficiency is a significant consideration but is less likely the primary cause given the context of MGUS and the specificity of the neuropathy pattern observed.<br>● Paraneoplastic syndrome remains a differential but is less probable due to the lack of supporting evidence for a broader paraneoplastic syndrome.<br>The next steps would involve management focused on monitoring MGUS, treating the B12 deficiency, and symptomatic management of the neuropathy.
35	An 82-year-old male patient presents with a 3-year history of pure sensory, symmetrical, length dependent polyneuropathy with insidious onset and slow progressive course. The patient presents with paraesthesia and hypoesthesia in the feet. Clinical examination reveals hyperalgesia during pinprick testing and tactile hypoesthesia in the feet, while there are no weakness, sensory ataxia, tremor, foot deformities, cranial nerve or autonomic dysfunction. Past medical history is positive for IgG kappa MGUS, prostate and bladder cancer, for which he underwent chemotherapy five years ago. Nerve conduction studies show a sensory-motor, symmetric, length dependent, axonal polyneuropathy. Screening lab tests reveal presence of IgG kappa paraprotein. Remaining exams including vitamin B12, folate, vitamin B6, vitamin E, HBV, HCV, glycaemia, HbA1c, ANA, ANCA, ENA, CRP, ESR, copper ,and cryoglobulins are normal. There is no family history of polyneuropathy.	Paraproteinemic neuropathy	**Summary of Pertinent Clinical Information**<br>● **Patient Profile:**<br>○ 82-year-old male<br>● **Chief Complaint:**<br>○ 3-year history of pure sensory, symmetrical, length-dependent polyneuropathy with insidious onset and slow progressive course.<br>○ Symptoms: Paraesthesia and hypoesthesia in the feet.<br>● **Clinical Examination:**<br>○ Hyperalgesia during pinprick testing.<br>○ Tactile hypoesthesia in the feet.<br>○ No weakness, sensory ataxia, tremor, foot deformities, cranial nerve involvement, or autonomic dysfunction.<br>● **Medical History:**<br>○ IgG kappa MGUS.<br>○ Prostate and bladder cancer treated with chemotherapy five years ago.<br>● **Diagnostic Tests:**<br>○ Nerve conduction studies: Sensory-motor, symmetric, length-dependent, axonal polyneuropathy.<br>○ Screening lab tests: Presence of IgG kappa paraprotein.<br>○ Normal levels of: Vitamin B12, folate, vitamin B6, vitamin E, HBV, HCV, glycaemia, HbA1c, ANA, ANCA, ENA, CRP, ESR, copper, and cryoglobulins.<br>○ No family history of polyneuropathy.<br>**Differential Diagnoses for Polyneuropathy**<br>1. **Monoclonal Gammopathy of Undetermined Significance (MGUS) Related Neuropathy:**<br>○ **Reasoning:**<br>■ The patient has a known history of IgG kappa MGUS, which is associated with neuropathy in about 10-20% of cases. The presence of IgG kappa paraprotein supports this diagnosis.<br>■ The polyneuropathy in MGUS typically presents as a slowly progressive, sensory-predominant, length-dependent neuropathy, which matches the patient's symptoms and clinical findings.<br>2. **Chemotherapy-Induced Peripheral Neuropathy (CIPN):**<br>○ **Reasoning:**<br>■ The patient underwent chemotherapy for prostate and bladder cancer five years ago. Many chemotherapeutic agents can cause neurotoxicity, leading to axonal polyneuropathy, often with a delayed onset.<br>■ The slowly progressive nature and sensory dominance of the neuropathy fit the profile of CIPN, which can persist and evolve long after treatment cessation.<br>3. **Chronic Idiopathic Axonal Polyneuropathy (CIAP):**<br>○ **Reasoning:**<br>■ CIAP is common in elderly patients and typically presents with a length-dependent, slowly progressive sensory neuropathy.<br>■ The absence of a family history, normal laboratory tests excluding secondary causes, and the clinical presentation align with CIAP.<br>**Most Likely Diagnosis**<br>**Monoclonal Gammopathy of Undetermined Significance (MGUS) Related Neuropathy** is the most likely diagnosis given the patient's medical history, clinical presentation, and the presence of IgG kappa paraprotein. MGUS-related neuropathy typically manifests as a sensory-predominant, length-dependent neuropathy, consistent with the patient's symptoms and examination findings. The presence of an associated paraprotein further supports this diagnosis. Although chemotherapy-induced neuropathy is a strong consideration, the temporal relationship and clinical features are more suggestive of MGUS neuropathy. Chronic idiopathic axonal polyneuropathy remains a differential but is less likely given the specific findings related to MGUS.
36	A 75-year-old male patient presents with a four-year history of pure sensory, symmetrical, length-dependent polyneuropathy with insidious onset and slow progression. The patient reports sensory ataxia, hypoesthesia in the distal lower limbs up to the calves, mild dysphagia and dysarthria, with no distal weakness. Past medical history is positive for chronic cough. Nerve conduction studies show an axonal, symmetric, length-dependent, pure sensory polyneuropathy. Screening lab tests, including vitamin B12, folate, vitamin B6, vitamin E, HBV, HCV, glycemia, HbA1c, ANA, ANCA, CRP, ESR, copper, cryoglobulins, and serum electrophoresis are all negative, with positivity for ENA autoantibodies. There is no family history of polyneuropathy.	CANVAS	**Summary of Pertinent Clinical Information:**<br>● **Patient Demographics:** 75-year-old male<br>● **Clinical Presentation:**<br>○ Four-year history of pure sensory, symmetrical, length-dependent polyneuropathy<br>○ Insidious onset and slow progression<br>○ Symptoms include sensory ataxia, hypoesthesia in distal lower limbs up to the calves, mild dysphagia, and dysarthria<br>○ No distal weakness reported<br>● **Past Medical History:** Chronic cough<br>● **Nerve Conduction Studies:** Axonal, symmetric, length-dependent, pure sensory polyneuropathy<br>● **Laboratory Tests:**<br>○ Negative for vitamin B12, folate, vitamin B6, vitamin E, HBV, HCV, glycemia, HbA1c, ANA, ANCA, CRP, ESR, copper, cryoglobulins, and serum electrophoresis<br>○ Positive for ENA autoantibodies<br>● **Family History:** No family history of polyneuropathy<br>**Three Most Probable Differential Diagnoses:**<br>1. **Paraneoplastic Syndrome:**<br>○ **Reasoning:**<br>■ The presence of a chronic cough in an elderly patient raises suspicion for an underlying malignancy, which could be related to the development of a paraneoplastic syndrome.<br>■ Paraneoplastic syndromes can present with pure sensory neuropathy and are often associated with autoantibodies, such as ENA autoantibodies, which were positive in this patient.<br>■ The insidious onset and slow progression of the symptoms align with paraneoplastic sensory neuropathy.<br>■ Dysphagia and dysarthria could also be linked to a paraneoplastic process affecting the cranial nerves.<br>2. **Sjogren's Syndrome:**<br>○ **Reasoning:**<br>■ Positive ENA autoantibodies suggest an autoimmune etiology.

			■ Sjogren's syndrome can present with sensory neuropathy and has been known to cause pure sensory, length-dependent neuropathy.<br>■ The chronicity and progression of symptoms without weakness are consistent with neuropathy associated with Sjogren's syndrome.<br>■ Dysphagia and dysarthria can occur in Sjogren's syndrome due to the involvement of the autonomic nervous system and cranial neuropathies.<br>3. **Idiopathic Sensory Neuropathy:**<br>○ **Reasoning:**<br>■ Despite extensive screening, many cases of sensory neuropathy remain idiopathic, especially in elderly patients.<br>■ The slow progression and pure sensory nature of the neuropathy without significant laboratory abnormalities other than ENA positivity could point towards an idiopathic origin.<br>■ However, the presence of ENA autoantibodies and mild systemic symptoms (dysphagia and dysarthria) makes this less likely compared to a more definitive autoimmune or paraneoplastic etiology.<br>**Most Likely Diagnosis:**<br>Based on the clinical presentation, the positive ENA autoantibodies, and the systemic symptoms such as chronic cough, dysphagia, and dysarthria, **paraneoplastic syndrome** is the most likely diagnosis. This diagnosis fits well with the patient's age, the chronicity of the cough, and the profile of the sensory neuropathy. Further investigations, including imaging studies (e.g., CT scan of the chest) and tumor markers, should be conducted to identify any underlying malignancy, which could be driving the paraneoplastic process.
37	A 58-year-old female patient presents with a 13-month history of sensory-motor, symmetrical, length dependent polyneuropathy with subacute onset and progressive course. The patient presents with distal weakness, neuropathic pain, and paresthesia in the four limbs. Clinical examination shows the presence of mild intrinsic hand atrophy and a peripheral edema. There are no sensory ataxia, tremor, foot deformities, cranial nerve or autonomic involvement. Past medical history is positive for occasional alcohol use. Nerve conduction studies show a mixed demyelinating-axonal, length dependent, sensory-motor polyneuropathy. Screening lab tests including vitamin B12, folate, vitamin B6, vitamin E, HCV, HBV, ANA, ANCA, ENA, CRP, ESR, glycemia, HbA1c, copper, and cryoglobulins are negative, while serum electrophoresis shows positivity for IgG lambda paraprotein. A cardiac ultrasound, performed to investigate edema in the lower limbs, revealed the presence of pericardial effusion. There is no family history of polyneuropathy.	POEMS	**Summary of Pertinent Clinical Information**<br>● **Patient Demographics:** 58-year-old female<br>● **History:** 13-month history of sensory-motor, symmetrical, length-dependent polyneuropathy with subacute onset and progressive course.<br>● **Symptoms:** Distal weakness, neuropathic pain, paresthesia in four limbs, mild intrinsic hand atrophy, peripheral edema.<br>● **Clinical Findings:**<br>○ No sensory ataxia, tremor, foot deformities, cranial nerve or autonomic involvement.<br>○ Mild intrinsic hand atrophy.<br>○ Peripheral edema.<br>● **Past Medical History:** Occasional alcohol use.<br>● **Nerve Conduction Studies:** Mixed demyelinating-axonal, length-dependent, sensory-motor polyneuropathy.<br>● **Screening Lab Tests:** Negative for vitamin B12, folate, vitamin B6, vitamin E, HCV, HBV, ANA, ANCA, ENA, CRP, ESR, glycemia, HbA1c, copper, and cryoglobulins.<br>● **Serum Electrophoresis:** Positive for IgG lambda paraprotein.<br>● **Cardiac Ultrasound:** Revealed pericardial effusion.<br>● **Family History:** No family history of polyneuropathy.<br>**Differential Diagnoses for Polyneuropathy**<br>**1. Monoclonal Gammopathy of Undetermined Significance (MGUS)**<br>**Reasoning:**<br>● The presence of IgG lambda paraprotein in serum electrophoresis is indicative of a monoclonal gammopathy.<br>● MGUS can be associated with neuropathy, typically presenting as a chronic, progressive, sensory-motor, and length-dependent polyneuropathy.<br>● The absence of systemic symptoms like weight loss, fever, or organomegaly makes multiple myeloma less likely, thus leaning towards MGUS.<br>**2. POEMS Syndrome (Polyneuropathy, Organomegaly, Endocrinopathy, Monoclonal protein, and Skin changes)**<br>**Reasoning:**<br>● The combination of peripheral neuropathy, the presence of IgG lambda paraprotein, and pericardial effusion aligns with POEMS syndrome.<br>● POEMS syndrome often presents with a progressive polyneuropathy and can include features like edema and organomegaly.<br>● However, there is no mention of other hallmark signs such as organomegaly, endocrinopathy, or skin changes in this patient, making this less likely but still possible.<br>**3. Chronic Inflammatory Demyelinating Polyradiculoneuropathy (CIDP)**<br>**Reasoning:**<br>● The mixed demyelinating-axonal features on nerve conduction studies can be seen in CIDP.<br>● CIDP typically presents as a progressive, length-dependent, sensory-motor polyneuropathy.<br>● The presence of mild intrinsic hand atrophy and peripheral edema can be seen in CIDP.<br>● Although IgG lambda paraprotein is not specific to CIDP, paraproteinemic neuropathies can sometimes present similarly to CIDP.<br>**Most Likely Diagnosis**<br>Considering the clinical presentation, the progressive course, and the findings of IgG lambda paraprotein along with the mixed demyelinating-axonal features on nerve conduction studies, the most likely diagnosis is **Monoclonal Gammopathy of Undetermined Significance (MGUS)-associated polyneuropathy**.<br>This diagnosis fits well with the subacute and progressive nature of the neuropathy, the electrophysiological findings, and the presence of monoclonal protein without other significant systemic findings that would suggest multiple myeloma or POEMS syndrome. CIDP remains a differential but is less likely given the specific presence of the IgG lambda paraprotein.
38	A 30-year-old female patient presents with a six-month history of right peroneal mononeuropathy (foot drop) with acute onset. She also reports the presence of mild paresthesia in the fourth and fifth fingers of the left hand over the past two years, and frequent hand paresthesia after using scissors at work. Nerve conduction studies show a mild demyelinating, sensory-motor polyneuropathy, with right carpal tunnel syndrome, left ulnar entrapment at the elbow, and right peroneal nerve entrapment at the fibular head. Screening lab tests including vitamin B12, folate, vitamin B6, vitamin E, HBV,	HNPP	**Step-by-Step Analysis of the Clinical Scenario**<br>**Patient Summary:**<br>● **Age/Gender:** 30-year-old female<br>● **History:**<br>○ **Six-month history:** Right peroneal mononeuropathy (foot drop), acute onset<br>○ **Two-year history:** Mild paresthesia in the fourth and fifth fingers of the left hand<br>○ **Occupational symptom:** Frequent hand paresthesia after using scissors<br>● **Nerve Conduction Studies:**<br>○ Mild demyelinating, sensory-motor polyneuropathy<br>○ Right carpal tunnel syndrome<br>○ Left ulnar entrapment at the elbow<br>○ Right peroneal nerve entrapment at the fibular head<br>● **Screening Lab Tests:**<br>○ Negative results for vitamin B12, folate, vitamin B6, vitamin E, HBV, HCV, glycemia, HbA1c, ANA, ANCA, ENA, CRP, ESR, copper, cryoglobulins, and serum electrophoresis<br>● **Family History:** No family history of polyneuropathy

	HCV, glycemia, HbA1c, ANA, ANCA, ENA, CRP, ESR, copper, cryoglobulins, and serum electrophoresis are all negative. There is no family history of polyneuropathy.		**Differential Diagnosis for Polyneuropathy**<br>1. **Hereditary Neuropathy with Liability to Pressure Palsies (HNPP)**<br>○ **Reasoning:** HNPP is a hereditary condition characterized by recurrent focal pressure neuropathies. This patient has multiple entrapment neuropathies (right peroneal, right carpal tunnel, left ulnar) which are hallmark features of HNPP. The mild demyelinating, sensory-motor polyneuropathy pattern seen in nerve conduction studies also fits well with HNPP. The absence of family history does not rule out this diagnosis as sporadic cases due to new mutations can occur.<br>2. **Multifocal Motor Neuropathy (MMN)**<br>○ **Reasoning:** MMN often presents with asymmetric, slowly progressive motor neuropathy. The presence of multiple focal neuropathies (peroneal, carpal tunnel, ulnar) and the demyelinating nature of the polyneuropathy seen in nerve conduction studies suggest MMN. However, MMN typically does not have significant sensory involvement, making this less likely but still possible given the motor dominance in her symptoms.<br>3. **Chronic Inflammatory Demyelinating Polyneuropathy (CIDP)**<br>○ **Reasoning:** CIDP can present with both sensory and motor involvement and is a chronic condition with progressive symptoms. The demyelinating pattern on nerve conduction studies is consistent with CIDP. However, CIDP usually presents with more generalized weakness and sensory loss, rather than focal entrapments, though variations do exist.<br>**Most Likely Diagnosis**<br>**Hereditary Neuropathy with Liability to Pressure Palsies (HNPP)** is the most likely diagnosis. The multiple entrapment neuropathies (right peroneal, right carpal tunnel, left ulnar) and the demyelinating nature of the polyneuropathy on nerve conduction studies strongly suggest HNPP. While the absence of family history is noted, sporadic mutations can lead to HNPP, making it a plausible diagnosis for this patient.
39	A 76-year-old male patient presents with an 8-year history of chronic, pure sensory, symmetrical, length dependent polyneuropathy with insidious onset and slow progressive course. The patient presents with severe neuropathic pain and heat intolerance in the feet. He does not report weakness, ataxia, paresthesia, tremor, cranial nerve or autonomic involvement. Clinical examination reveals the presence of allodynia and hyperalgesia in the feet. There are no foot deformities. Past medical history is positive for prostatic cancer. Nerve conduction studies are normal. Screening lab tests including vitamin B12, folate, vitamin B6, vitamin E, HBV, HCV, ANA, ANCA, ENA, CRP, ESR, glycemia, HbA1c, copper, cryoglobulins, and serum electrophoresis are negative. There is no family history of polyneuropathy.	Small fibre neuropathy	**Summary of Pertinent Clinical Information:**<br>● **Patient**: 76-year-old male<br>● **History**: 8-year history of chronic, pure sensory, symmetrical, length-dependent polyneuropathy with insidious onset and slow progressive course<br>● **Symptoms**:<br>○ Severe neuropathic pain and heat intolerance in the feet<br>○ No weakness, ataxia, paresthesia, tremor, cranial nerve, or autonomic involvement<br>● **Clinical Findings**:<br>○ Presence of allodynia and hyperalgesia in the feet<br>○ No foot deformities<br>● **Past Medical History**: Prostatic cancer<br>● **Nerve Conduction Studies**: Normal<br>● **Screening Lab Tests**: Negative for vitamin B12, folate, vitamin B6, vitamin E, HBV, HCV, ANA, ANCA, ENA, CRP, ESR, glycemia, HbA1c, copper, cryoglobulins, and serum electrophoresis<br>● **Family History**: No family history of polyneuropathy<br>**Differential Diagnoses for Polyneuropathy:**<br>1. **Paraneoplastic Neuropathy**:<br>○ **Reasoning**: The patient has a history of prostatic cancer, which raises the possibility of a paraneoplastic syndrome. Paraneoplastic neuropathy is typically associated with cancer and may present as a pure sensory neuropathy. The chronic and progressive nature of the symptoms along with normal nerve conduction studies can be consistent with this diagnosis.<br>○ **Supporting Features**:<br>■ History of prostatic cancer<br>■ Normal nerve conduction studies<br>○ **Against**: Lack of other paraneoplastic symptoms or involvement of other systems.<br>2. **Idiopathic Small Fiber Neuropathy (SFN)**:<br>○ **Reasoning**: The presentation of pure sensory symptoms, normal nerve conduction studies, and the presence of allodynia and hyperalgesia are characteristic of small fiber neuropathy. SFN often presents with pain and sensory disturbances without motor involvement or autonomic symptoms.<br>○ **Supporting Features**:<br>■ Normal nerve conduction studies (indicating involvement of small fibers rather than large fibers)<br>■ Allodynia and hyperalgesia<br>○ **Against**: The long duration (8 years) and slow progression could suggest another underlying cause, but idiopathic SFN remains a possibility given the clinical picture.<br>3. **Chemotherapy-Induced Neuropathy**:<br>○ **Reasoning**: Although not specified, it is important to consider the possibility of chemotherapy treatment for prostatic cancer in the patient's past medical history. Chemotherapy drugs, particularly platinum-based agents, taxanes, and vinca alkaloids, can cause a sensory neuropathy.<br>○ **Supporting Features**:<br>■ History of prostatic cancer (potential treatment with neurotoxic chemotherapy)<br>■ Pure sensory neuropathy<br>○ **Against**: The case does not mention specific treatments, and normal nerve conduction studies are less common in chemotherapy-induced neuropathy unless it predominantly affects small fibers.<br>**Most Likely Diagnosis:**<br>Given the patient's clinical presentation, the most likely diagnosis is **Idiopathic Small Fiber Neuropathy (SFN)**. This diagnosis fits well with the described symptoms of severe neuropathic pain, heat intolerance, allodynia, and hyperalgesia, combined with normal nerve conduction studies, which typically assess large fiber function and might not detect small fiber involvement.<br>**Conclusion:**<br>● **Most Likely Diagnosis**: Idiopathic Small Fiber Neuropathy (SFN)<br>● **Rationale**: The combination of pure sensory symptoms, normal nerve conduction studies, and typical small fiber symptoms such as allodynia and hyperalgesia point towards SFN. Despite the patient's history of prostatic cancer, the lack of other systemic or paraneoplastic features and the absence of chemotherapy-related treatment details make SFN the most probable diagnosis.
40	A 79-year-old male patient presents with a 2-year history of pure sensory, symmetrical, length dependent polyneuropathy with insidious onset. The patient presents with paresthesia, hypoesthesia and neuropathic pain in the distal areas of the lower limbs. There are	HBV-associated neuropathy	**Summary of Pertinent Clinical Information**<br>● **Patient Profile:** 79-year-old male<br>● **Symptoms:**<br>○ 2-year history of pure sensory, symmetrical, length-dependent polyneuropathy<br>○ Paresthesia, hypoesthesia, and neuropathic pain in distal lower limbs<br>○ No ataxia, tremor, foot deformities, cranial nerve, or autonomic involvement<br>● **Nerve Conduction Studies:** Pure sensory, axonal polyneuropathy

<table>
<tr><td></td><td>no ataxia, tremor, foot deformities, cranial nerve or autonomic involvement. Past medical history is silent. Nerve conduction studies show pure sensory, axonal polyneuropathy. Screening lab tests including vitamin B12, folate, vitamin B6, vitamin E, HCV, ANA, ANCA, ENA, CRP, ESR, glycemia, HbA1c, copper, cryoglobulins and serum electrophoresis are all negative, with evidence of chronic HBV infection. There is no family history of polyneuropathy.</td><td></td><td>

Laboratory Results:

Negative for vitamin B12, folate, vitamin B6, vitamin E, HCV, ANA, ANCA, ENA, CRP, ESR, glycemia, HbA1c, copper, cryoglobulins, and serum electrophoresis
Positive for chronic HBV infection

Family History: No family history of polyneuropathy

Differential Diagnoses for Polyneuropathy

Chronic Hepatitis B Virus (HBV) Infection-Associated Polyneuropathy
Reasoning:

Chronic HBV infection is present and can be associated with polyneuropathy.
HBV-related neuropathy typically manifests as sensory neuropathy, which fits the patient’s symptoms.
Though less common, HBV can lead to axonal polyneuropathy through immune-mediated mechanisms or direct viral effects.

Idiopathic Sensory Polyneuropathy
Reasoning:

A significant number of cases of sensory polyneuropathy in elderly patients remain idiopathic, meaning no clear cause can be identified after thorough investigation.
The patient's normal screening lab tests for common causes of neuropathy, along with the absence of family history and negative screening for other conditions, suggest the possibility of idiopathic origins.
The typical presentation of idiopathic sensory polyneuropathy aligns with the patient’s symptoms of distal paresthesia, hypoesthesia, and neuropathic pain without motor involvement.

Paraneoplastic Sensory Neuropathy
Reasoning:

Paraneoplastic syndromes can cause sensory neuropathy and often present in older adults.
While the patient’s laboratory workup did not include specific cancer markers or imaging, the absence of more common causes and the patient’s age make this a plausible consideration.
Symptoms like the ones described can be associated with malignancies, particularly small cell lung cancer and other tumors, though the diagnosis requires further investigation through imaging and tumor markers.

Most Likely Diagnosis
Given the clinical presentation and the detailed workup that ruled out many common causes, the most likely diagnosis is:
Chronic Hepatitis B Virus (HBV) Infection-Associated Polyneuropathy
Justification:

The presence of chronic HBV infection, which can be linked to polyneuropathy through immune-mediated mechanisms.
The pure sensory nature and axonal type of neuropathy identified in nerve conduction studies are consistent with HBV-associated neuropathy.
Although idiopathic sensory polyneuropathy remains a strong possibility, the known chronic HBV infection in this patient tilts the balance towards HBV-associated neuropathy as the most probable cause. Further evaluation and management of the HBV infection and associated neuropathy would be the recommended course of action.

</td></tr>
<tr><td>41</td><td>An 82-year-old female patient presents with a 4-year history of pure sensory, length dependent, symmetric polyneuropathy with insidious onset and slow progressive course. The patient presents with paresthesia and mild neuropathic pain in the feet. Clinical examination does not show weakness or sensory ataxia, tremor, foot deformities, cranial nerve or autonomic involvement. Past medical history is positive for IgA kappa monoclonal gammopathy, arterial hypertension, gout, hypercholesterolemia, and cataract. Nerve conduction studies show presence of a pure sensory, length dependent, symmetric, axonal polyneuropathy. Screening lab tests including vitamin B12, folate, vitamin B6, vitamin E, HBV, HCV, glycemia, HbA1c, ANA, ANCA, ENA, CRP, ESR, copper, and cryoglobulins are negative, with positivity to IgA kappa paraprotein. There is no family history of polyneuropathy.</td><td>MGUS-associated neuropathy</td><td>
Summary of Pertinent Clinical Information

Patient Profile: 82-year-old female
Symptoms: 4-year history of pure sensory, length-dependent, symmetric polyneuropathy with insidious onset and slow progression

Paresthesia and mild neuropathic pain in the feet
No weakness, sensory ataxia, tremor, foot deformities, cranial nerve, or autonomic involvement

Past Medical History:

IgA kappa monoclonal gammopathy
Arterial hypertension
Gout
Hypercholesterolemia
Cataract

Clinical Examination:

Pure sensory neuropathy
Nerve conduction studies confirm pure sensory, length-dependent, symmetric, axonal polyneuropathy

Laboratory Findings:

Negative: Vitamin B12, folate, vitamin B6, vitamin E, HBV, HCV, glycemia, HbA1c, ANA, ANCA, ENA, CRP, ESR, copper, and cryoglobulins
Positive: IgA kappa paraprotein

Family History: No family history of polyneuropathy

Differential Diagnoses for Polyneuropathy

Monoclonal Gammopathy of Undetermined Significance (MGUS)-related Polyneuropathy
Reasoning: The presence of IgA kappa monoclonal gammopathy in the patient is a significant clue. MGUS is known to be associated with peripheral neuropathy, especially when other causes are ruled out. The polyneuropathy in MGUS is typically slowly progressive and primarily sensory, matching the patient's clinical presentation. Additionally, the absence of other systemic symptoms or laboratory abnormalities supports this diagnosis.
Chronic Inflammatory Demyelinating Polyneuropathy (CIDP)
Reasoning: Although CIDP typically presents with both sensory and motor involvement, there are variants that are predominantly sensory. The chronic, progressive nature of the symptoms aligns with CIDP. However, the nerve conduction study indicating purely axonal polyneuropathy is less typical for CIDP, which usually shows demyelination. Despite this, the possibility of CIDP should be considered due to its varied presentations.
Diabetic Polyneuropathy
Reasoning: Even though the patient’s glycemia and HbA1c are within normal ranges, diabetes and prediabetes can sometimes be present despite normal blood glucose levels. The length-dependent, symmetric nature of the neuropathy is characteristic of diabetic neuropathy. Additionally, the patient's age and associated comorbidities (like hypercholesterolemia and hypertension) increase the risk of diabetes. However, the absence of hyperglycemia in screening tests makes this less likely but still possible if there are fluctuations in glucose levels not captured by the tests.

Most Likely Diagnosis
Monoclonal Gammopathy of Undetermined Significance (MGUS)-related Polyneuropathy
The most likely diagnosis for this patient is MGUS-related polyneuropathy. The presence of IgA kappa monoclonal gammopathy and the clinical features of a pure sensory, length-dependent, symmetric, and slowly progressive polyneuropathy fit well with this diagnosis. The absence of other significant findings in the lab tests further supports MGUS as the primary underlying cause. MGUS-related neuropathy is known to occur even in the absence of systemic disease or malignancy, and the clinical and electrophysiological findings in this patient are consistent with such a presentation.
</td></tr>
</table>

42	A 47-year-old male patient presents with a 1-year history of pure motor, asymmetrical multineuropathy with subacute onset. Clinical examination reveals the presence of weakness and atrophy in a multifocal distribution in the upper limbs. There are no sensory deficits or subjective numbness, tingling or pain, and physical examination does not show the presence of sensory ataxia, tremor, foot deformities, cranial nerve or autonomic involvement. Past medical history is silent. Nerve conduction studies show pure motor, demyelinating multineuropathy with nerve conduction blocks. Screening lab tests including vitamin B12, folate, vitamin B6, vitamin E, HCV, HBV, ANA, ANCA, ENA, CRP, ESR, glycemia, HbA1c, copper, cryoglobulins and serum electrophoresis are all negative. There is no family history of polyneuropathy.	MMN	**Summary of Pertinent Clinical Information**<br>● **Patient:** 47-year-old male<br>● **History:** 1-year history of pure motor, asymmetrical multineuropathy with subacute onset<br>● **Clinical Examination:**<br>○ Weakness and atrophy in a multifocal distribution in the upper limbs<br>○ No sensory deficits (no numbness, tingling, or pain)<br>○ No sensory ataxia, tremor, foot deformities, cranial nerve, or autonomic involvement<br>● **Past Medical History:** Silent<br>● **Nerve Conduction Studies:** Pure motor, demyelinating multineuropathy with nerve conduction blocks<br>● **Screening Lab Tests:** Negative for vitamin B12, folate, vitamin B6, vitamin E, HCV, HBV, ANA, ANCA, ENA, CRP, ESR, glycemia, HbA1c, copper, cryoglobulins, and serum electrophoresis<br>● **Family History:** No family history of polyneuropathy<br>**Differential Diagnoses for Polyneuropathy**<br>**1. Multifocal Motor Neuropathy (MMN)**<br>**Reasoning:**<br>● **Clinical Presentation:** MMN typically presents as an asymmetric, purely motor neuropathy with a multifocal distribution. The patient's symptoms of subacute onset, multifocal weakness, and atrophy are consistent with MMN.<br>● **Nerve Conduction Studies:** The presence of demyelinating features and conduction blocks strongly suggests MMN, as these are characteristic findings in this condition.<br>● **Lack of Sensory Involvement:** MMN usually does not affect sensory nerves, aligning with the absence of sensory deficits in this patient.<br>● **Negative Laboratory Results:** The negative screening for autoimmune markers and other potential causes is typical as MMN does not typically present with systemic involvement detectable by these tests.<br>**2. Chronic Inflammatory Demyelinating Polyradiculoneuropathy (CIDP) - Motor Variant**<br>**Reasoning:**<br>● **Clinical Presentation:** CIDP can present with a purely motor variant, though it is more commonly a mixed sensory and motor neuropathy. The patient's purely motor presentation with multifocal weakness could fit this variant.<br>● **Nerve Conduction Studies:** Demyelinating neuropathy with conduction blocks can be seen in CIDP.<br>● **Lack of Sensory Involvement:** While rare, CIDP can present without sensory involvement.<br>● **Negative Laboratory Results:** CIDP can have normal routine blood tests. The absence of other systemic symptoms also does not rule out CIDP.<br>**3. Paraneoplastic Motor Neuropathy**<br>**Reasoning:**<br>● **Clinical Presentation:** Paraneoplastic syndromes can present with various neuropathic features, including asymmetric motor neuropathy. The subacute onset and multifocal distribution in this patient could suggest a paraneoplastic cause.<br>● **Nerve Conduction Studies:** Demyelinating features with conduction blocks can also be seen in paraneoplastic neuropathies.<br>● **Lack of Sensory Involvement:** Paraneoplastic motor neuropathy may present without sensory involvement, although it is less common.<br>● **Negative Laboratory Results:** Paraneoplastic syndromes often require specific testing beyond routine labs, such as onconeural antibodies, which were not specified in the case.<br>**Most Likely Diagnosis**<br>The most likely diagnosis for this patient is **Multifocal Motor Neuropathy (MMN)**. This conclusion is based on the asymmetric, purely motor presentation, multifocal distribution, and the characteristic findings of demyelination and conduction blocks on nerve conduction studies. MMN also aligns well with the lack of sensory involvement and the negative results from extensive screening for other common causes of neuropathy.<br>CIDP (motor variant) and paraneoplastic motor neuropathy remain possibilities, but MMN is more consistent with the patient's clinical and electrophysiological profile. Further testing, such as anti-GM1 antibody titers and possibly a trial of immunomodulatory therapy, could help confirm the diagnosis of MMN.
43	An 88-year-old male patient presents with a 4-year history of sensory-motor, symmetrical, length dependent polyneuropathy with insidious onset and slow progressive course. The patient presents with paresthesia and neuropathic pain distally in the lower limbs, with no ataxia, tremor, foot deformities, cranial nerve or autonomic involvement. Past medical history is positive for type 2 diabetes, cardiac insufficiency and lung cancer, for which he underwent chemotherapy 15 years ago. Nerve conduction studies show a sensory-motor, symmetric, axonal polyneuropathy. Screening lab tests including vitamin B12, folate, vitamin B6, vitamin E, HBV, HCV, ANA, ANCA, ENA, CRP, ESR, copper, cryoglobulins and serum electrophoresis are all negative, with evidence of hyperglycaemia and elevated HbA1c. There is no family history of polyneuropathy.	Diabetic neuropathy	**Step-by-Step Analysis of the Clinical Scenario**<br>**Clinical Summary:**<br>● **Patient Profile:** 88-year-old male<br>● **Chief Complaint:** Sensory-motor, symmetrical, length-dependent polyneuropathy<br>● **Duration:** 4 years<br>● **Symptoms:** Paresthesia and neuropathic pain distally in the lower limbs<br>● **Onset and Course:** Insidious onset with a slow progressive course<br>● **No Involvement:** Ataxia, tremor, foot deformities, cranial nerve, or autonomic involvement<br>● **Past Medical History:**<br>○ Type 2 diabetes mellitus<br>○ Cardiac insufficiency<br>○ Lung cancer (treated with chemotherapy 15 years ago)<br>● **Nerve Conduction Studies:** Sensory-motor, symmetric, axonal polyneuropathy<br>● **Lab Results:**<br>○ Negative for vitamin deficiencies (B12, B6, E), HBV, HCV, ANA, ANCA, ENA, CRP, ESR, copper, cryoglobulins, serum electrophoresis<br>○ Hyperglycemia and elevated HbA1c<br>● **Family History:** No family history of polyneuropathy<br>**Differential Diagnoses for Polyneuropathy**<br>1. **Diabetic Polyneuropathy**<br>○ **Reasoning:**<br>■ **Prevalence:** The patient has a history of type 2 diabetes mellitus, a common cause of peripheral neuropathy.<br>■ **Clinical Presentation:** The sensory-motor, symmetrical, length-dependent nature of the neuropathy fits the typical presentation of diabetic neuropathy.<br>■ **Nerve Conduction Studies:** Axonal polyneuropathy is consistent with diabetic neuropathy.<br>■ **Lab Findings:** Hyperglycemia and elevated HbA1c support poorly controlled diabetes, contributing to the development of neuropathy.<br>■ **Absence of Other Causes:** Negative screening tests for other common causes of polyneuropathy.<br>2. **Chemotherapy-Induced Polyneuropathy**<br>○ **Reasoning:**

			■ **History of Chemotherapy:** The patient underwent chemotherapy for lung cancer 15 years ago, which can lead to long-term peripheral neuropathy.<br>■ **Clinical Presentation:** Chemotherapy-induced neuropathy can present as a sensory-motor, symmetrical polyneuropathy, often with a delayed onset after treatment.<br>■ **Type of Neuropathy:** Axonal damage is typical of chemotherapy-induced neuropathy.<br>■ **Duration:** Although the neuropathy appeared years after chemotherapy, some agents can have prolonged effects.<br>3. **Chronic Idiopathic Axonal Polyneuropathy (CIAP)**<br>○ **Reasoning:**<br>■ **Age:** CIAP is more common in elderly patients and typically presents with a slow, progressive course.<br>■ **Clinical Presentation:** The length-dependent, sensory-motor, symmetrical nature of the neuropathy fits CIAP.<br>■ **Negative Screening Tests:** The extensive negative screening tests for other causes of neuropathy suggest idiopathic etiology.<br>■ **No Family History:** The absence of a family history supports the idiopathic nature of the condition.<br>**Most Likely Diagnosis**<br>**Diabetic Polyneuropathy**<br>● **Rationale:**<br>○ The patient's long-standing history of type 2 diabetes mellitus with evidence of hyperglycemia and elevated HbA1c is a strong indicator of diabetic polyneuropathy. The clinical presentation, including the sensory-motor and symmetrical nature of the neuropathy, aligns with typical diabetic neuropathy.<br>○ Additionally, the exclusion of other common causes through negative lab results supports diabetes as the underlying cause.<br>○ While chemotherapy-induced neuropathy and CIAP are potential considerations, the clear correlation with poorly controlled diabetes and the typical presentation make diabetic polyneuropathy the most likely diagnosis.
44	An 81-year-old male patient presents with a six-month history of sensory-motor, symmetrical, length-dependent polyneuropathy with subacute onset and slow progression. The patient reports neuropathic pain in the lower limbs in a distal, symmetric distribution, along with sensory ataxia. Clinical examination does not reveal weakness, tremor, foot deformities, cranial nerve or autonomic involvement. Past medical history is positive for IgM kappa monoclonal gammopathy, Waldenström macroglobulinemia, and colorectal cancer, for which he underwent chemotherapy five years ago. Nerve conduction studies show a length-dependent, sensorimotor, symmetric, demyelinating polyneuropathy. Screening lab tests, including vitamin B12, folate, vitamin B6, vitamin E, HCV, HBV, ANA, ANCA, ENA, CRP, ESR, glycemia, HbA1c, copper, and cryoglobulins are all normal, with evidence of IgM kappa paraprotein. There is no family history of polyneuropathy.	Anti-MAG polyneuropathy	**Clinical Summary**<br>● **Patient Profile**: 81-year-old male<br>● **Symptoms Duration**: 6 months<br>● **Symptoms Description**:<br>○ Sensory-motor, symmetrical, length-dependent polyneuropathy<br>○ Subacute onset with slow progression<br>○ Neuropathic pain in the lower limbs, distal and symmetric distribution<br>○ Sensory ataxia<br>● **Clinical Examination**:<br>○ No weakness or tremor<br>○ No foot deformities<br>○ No cranial nerve or autonomic involvement<br>● **Past Medical History**:<br>○ IgM kappa monoclonal gammopathy<br>○ Waldenström macroglobulinemia<br>○ Colorectal cancer (chemotherapy completed five years ago)<br>● **Nerve Conduction Studies**:<br>○ Length-dependent, sensorimotor, symmetric, demyelinating polyneuropathy<br>● **Lab Tests**:<br>○ Normal: Vitamin B12, folate, vitamin B6, vitamin E, HCV, HBV, ANA, ANCA, ENA, CRP, ESR, glycemia, HbA1c, copper, cryoglobulins<br>○ Evidence of IgM kappa paraprotein<br>● **Family History**: No history of polyneuropathy<br>**Differential Diagnoses**<br>1. **Waldenström Macroglobulinemia-associated Neuropathy**<br>○ **Reasoning**: Waldenström macroglobulinemia is known to be associated with peripheral neuropathy, particularly demyelinating types. The presence of IgM kappa monoclonal gammopathy and Waldenström macroglobulinemia in the patient's history strongly suggests this as a potential cause. The demyelinating nature observed in nerve conduction studies aligns with neuropathies linked to monoclonal gammopathies.<br>○ **Supporting Factors**: IgM kappa paraprotein, history of Waldenström macroglobulinemia.<br>○ **Against**: Lack of cranial nerve or autonomic involvement does not rule out this diagnosis but is less common in this condition.<br>2. **Chemotherapy-induced Neuropathy**<br>○ **Reasoning**: The patient has a history of colorectal cancer treated with chemotherapy, a known risk factor for developing neuropathy. Chemotherapy agents, particularly those used in colorectal cancer like oxaliplatin, can cause neuropathy. However, the onset of symptoms five years post-treatment is atypical, as chemotherapy-induced neuropathy generally presents sooner after treatment.<br>○ **Supporting Factors**: History of chemotherapy.<br>○ **Against**: The long interval (five years) since chemotherapy reduces the likelihood but doesn't completely rule it out.<br>3. **Chronic Inflammatory Demyelinating Polyneuropathy (CIDP)**<br>○ **Reasoning**: CIDP presents with a slowly progressive, symmetrical, sensorimotor neuropathy that can mimic length-dependent neuropathy. The demyelinating pattern on nerve conduction studies supports CIDP. However, CIDP often involves both motor and sensory nerves more prominently, and the lack of motor symptoms (weakness) makes this less likely but still possible.<br>○ **Supporting Factors**: Symmetrical sensorimotor neuropathy, demyelinating pattern.<br>○ **Against**: Absence of significant motor involvement.<br>**Most Likely Diagnosis**<br>The most likely diagnosis is **Waldenström macroglobulinemia-associated neuropathy**. This conclusion is based on the patient's history of Waldenström macroglobulinemia, the presence of IgM kappa paraprotein, and the demyelinating nature of the neuropathy observed in nerve conduction studies. Although other causes are possible, the strong association between IgM monoclonal gammopathy and neuropathy makes this the most probable diagnosis in this case.
45	An 83-year-old male patient presents with a 3-year history of sensory, symmetrical, length dependent polyneuropathy with insidious onset. The patient presents with paresthesia and hypoesthesia in the lower	MGUS-associated neuropathy	**Summary of Pertinent Clinical Information**<br>**Patient Demographics:**<br>● **Age:** 83 years<br>● **Sex:** Male<br>**History and Symptoms:**

	limbs without weakness or sensory ataxia. Clinical examination does not show the presence of tremor, neuropathic pain, foot deformities, cranial nerve or autonomic involvement. Past medical history is positive for IgG lambda monoclonal gammopathy. Nerve conduction studies show axonal, length dependent, sensory-motor polyneuropathy and unilateral carpal tunnel syndrome. Screening lab tests including vitamin B12, folate, vitamin B6, vitamin E, HCV, HBV, ANA, ANCA, ENA, CRP, ESR, glycemia, HbA1c, copper, and cryoglobulins are all negative, with positivity for IgG lambda paraprotein. There is no family history of polyneuropathy.		● **Duration:** 3 years<br>● **Type of Neuropathy:** Sensory, symmetrical, length-dependent polyneuropathy<br>● **Onset:** Insidious<br>● **Symptoms:** Paresthesia and hypoesthesia in the lower limbs<br>● **Absence of:** Weakness, sensory ataxia, tremor, neuropathic pain, foot deformities, cranial nerve or autonomic involvement<br>**Past Medical History:**<br>● **IgG lambda monoclonal gammopathy**<br>**Clinical Examination:**<br>● **Findings:** No tremor, neuropathic pain, foot deformities, cranial nerve or autonomic involvement<br>**Nerve Conduction Studies:**<br>● **Findings:** Axonal, length-dependent, sensory-motor polyneuropathy; unilateral carpal tunnel syndrome<br>**Laboratory Tests:**<br>● **Negative for:** Vitamin B12, folate, vitamin B6, vitamin E, HCV, HBV, ANA, ANCA, ENA, CRP, ESR, glycemia, HbA1c, copper, cryoglobulins<br>● **Positive for:** IgG lambda paraprotein<br>**Family History:**<br>● **Negative for:** Polyneuropathy<br>**Differential Diagnoses for Polyneuropathy**<br>1. **Monoclonal Gammopathy of Undetermined Significance (MGUS)-Related Neuropathy:**<br>○ **Reasoning:** The patient has a known history of IgG lambda monoclonal gammopathy, which is a common cause of peripheral neuropathy, particularly in the elderly. MGUS can lead to axonal sensory-motor polyneuropathy, which aligns with the patient's clinical presentation and nerve conduction study findings. The absence of systemic involvement such as cranial nerve or autonomic dysfunction also supports this diagnosis as MGUS-related neuropathy typically does not involve these systems.<br>2. **Diabetic Polyneuropathy:**<br>○ **Reasoning:** Despite negative screening lab tests for diabetes (glycemia and HbA1c), diabetic neuropathy is a common cause of length-dependent polyneuropathy, especially in elderly patients. The patient might have a pre-diabetic state or intermittent hyperglycemia that was not captured at the time of testing. However, the lack of hyperglycemia in screening labs makes this less likely than MGUS-related neuropathy.<br>3. **Chronic Idiopathic Axonal Polyneuropathy (CIAP):**<br>○ **Reasoning:** CIAP is a diagnosis of exclusion for elderly patients presenting with length-dependent sensory-motor polyneuropathy when no other cause is identified. Given the negative extensive lab workup and the patient's age, CIAP remains a consideration. However, the presence of IgG lambda paraprotein makes MGUS-related neuropathy a more likely specific diagnosis.<br>**Most Likely Diagnosis**<br>**Monoclonal Gammopathy of Undetermined Significance (MGUS)-Related Neuropathy**<br>**Reasoning:**<br>● The patient's history of IgG lambda monoclonal gammopathy strongly points towards MGUS-related neuropathy as the primary cause of the sensory, length-dependent polyneuropathy.<br>● The clinical presentation and nerve conduction study findings are consistent with neuropathy associated with MGUS.<br>● The negative screening lab tests for other common causes of polyneuropathy (e.g., diabetes, vitamin deficiencies, infections) further support MGUS as the underlying etiology.<br>● Although CIAP is a consideration, the identified monoclonal gammopathy provides a specific and likely cause for the patient's symptoms, making MGUS-related neuropathy the most probable diagnosis.
46	A 71-year-old female patient presents with a 2-year history of sensory-motor, asymmetrical multineuropathy with relatively slow onset. The patient presents with weakness, numbness and tingling in a multifocal distribution in the four limbs, with hand tremor and mild sensory ataxia without pain. Clinical examination reveals absence of foot deformities, cranial nerve or autonomic involvement. Past medical history is positive for breast and kidney cancer, for which the patient underwent chemotherapy five years ago, and which were considered cured. Nerve conduction studies show a multifocal, demyelinating, sensory-motor neuropathy with motor nerve conduction blocks. Screening lab tests including vitamin B12, folate, vitamin B6, vitamin E, HCV, HBV, ANA, ANCA, ENA, CRP, ESR, glycemia, HbA1c, copper, cryoglobulins and serum electrophoresis are all negative. There is no family history of polyneuropathy.	Multifocal CIDP	**Summary of Pertinent Clinical Information**<br>● **Patient:** 71-year-old female<br>● **History:**<br>○ 2-year history of sensory-motor, asymmetrical multineuropathy<br>○ Relatively slow onset<br>● **Symptoms:**<br>○ Weakness, numbness, and tingling in a multifocal distribution in the four limbs<br>○ Hand tremor<br>○ Mild sensory ataxia<br>○ No pain<br>● **Clinical Examination:**<br>○ Absence of foot deformities<br>○ No cranial nerve involvement<br>○ No autonomic involvement<br>● **Past Medical History:**<br>○ Breast and kidney cancer (chemotherapy 5 years ago, considered cured)<br>● **Nerve Conduction Studies:**<br>○ Multifocal, demyelinating, sensory-motor neuropathy<br>○ Motor nerve conduction blocks<br>● **Lab Tests:**<br>○ Screening labs (vitamin B12, folate, vitamin B6, vitamin E, HCV, HBV, ANA, ANCA, ENA, CRP, ESR, glycemia, HbA1c, copper, cryoglobulins, serum electrophoresis) all negative<br>● **Family History:** No family history of polyneuropathy<br>**Differential Diagnoses**<br>1. **Chronic Inflammatory Demyelinating Polyneuropathy (CIDP)**<br>○ **Reasoning:**<br>■ The presentation of a sensory-motor neuropathy with multifocal distribution and demyelination is characteristic of CIDP.<br>■ Motor nerve conduction blocks are a key finding in CIDP.<br>■ The slow progression over two years aligns with the typical course of CIDP.

			■ The lack of autonomic or cranial nerve involvement fits well with CIDP.<br>■ Negative lab results for inflammatory markers and autoantibodies do not rule out CIDP, as the diagnosis is primarily clinical and electrophysiological.<br>2. **Multifocal Motor Neuropathy (MMN)**<br>○ **Reasoning:**<br>■ Although MMN typically presents with motor symptoms, the sensory involvement can occasionally be minimal or absent, and motor nerve conduction blocks are a hallmark.<br>■ The asymmetrical nature and multifocal distribution fit MMN.<br>■ The absence of pain and slow progression are also consistent with MMN.<br>■ Negative lab tests, especially for cryoglobulins and other potential inflammatory causes, do not exclude MMN.<br>3. **Paraneoplastic Neuropathy**<br>○ **Reasoning:**<br>■ Given the patient's history of cancer (breast and kidney), a paraneoplastic syndrome could be considered, especially with sensory-motor neuropathy and multifocal demyelination.<br>■ Paraneoplastic syndromes can present with varied and asymmetric neuropathies.<br>■ Although the cancers are considered cured, paraneoplastic symptoms can sometimes emerge or persist long after the primary malignancy is treated.<br>■ However, the negative screening labs, particularly for common paraneoplastic antibodies, and the lack of other systemic symptoms might make this less likely.<br>**Most Likely Diagnosis**<br>**Chronic Inflammatory Demyelinating Polyneuropathy (CIDP)**<br>● **Justification:**<br>○ The clinical presentation of multifocal, demyelinating, sensory-motor neuropathy with conduction blocks strongly supports CIDP.<br>○ The slow progression and absence of cranial nerve and autonomic involvement are typical of CIDP.<br>○ CIDP is a more common cause of these symptoms compared to MMN and paraneoplastic neuropathy, particularly in the absence of other supporting findings for the latter two diagnoses.<br>○ While CIDP is often a diagnosis of exclusion, the detailed nerve conduction study findings are particularly indicative of this condition.
47	An 80-year-old male patient presents with a 4-year history of pure sensory, symmetrical, length dependent polyneuropathy with insidious onset. The patient presents with distal paresthesia, neuropathic pain and cramps in the lower limbs with no ataxia, tremor, foot deformities, cranial nerve or autonomic involvement. Past medical history shows diabetes, psoriatic arthritis, polymyalgia rheumatica and chemotherapy for lung cancer. Neuropathy symptoms started four years after chemotherapy. Nerve conduction studies show mixed sensory-motor, symmetric, axonal polyneuropathy. Screening lab tests including vitamin B12, folate, vitamin B6, vitamin E, HCV, ANA, ANCA, ENA, CRP, ESR, glycemia, copper, cryoglobulins and serum electrophoresis are all negative, with evidence of chronic HBV infection. HbA1c levels are elevated. There is no family history of polyneuropathy.	Diabetic neuropathy	**Summary of Pertinent Clinical Information**<br>● **Patient Profile**: 80-year-old male<br>● **Symptoms**:<br>○ 4-year history of pure sensory, symmetrical, length-dependent polyneuropathy<br>○ Distal paresthesia, neuropathic pain, and cramps in the lower limbs<br>○ No ataxia, tremor, foot deformities, cranial nerve, or autonomic involvement<br>● **Medical History**:<br>○ Diabetes<br>○ Psoriatic arthritis<br>○ Polymyalgia rheumatica<br>○ Chemotherapy for lung cancer (neuropathy started four years post-chemotherapy)<br>● **Nerve Conduction Studies**:<br>○ Mixed sensory-motor, symmetric, axonal polyneuropathy<br>● **Screening Lab Tests**:<br>○ All negative (including vitamin B12, folate, vitamin B6, vitamin E, HCV, ANA, ANCA, ENA, CRP, ESR, glycemia, copper, cryoglobulins, serum electrophoresis)<br>○ Chronic HBV infection<br>○ Elevated HbA1c levels<br>● **Family History**:<br>○ No family history of polyneuropathy<br>**Differential Diagnoses for Polyneuropathy**<br>1. **Diabetic Neuropathy**<br>○ **Reasoning**:<br>■ The patient has a history of diabetes with elevated HbA1c levels, indicating poorly controlled blood glucose levels. Diabetic neuropathy is a common complication of diabetes, characterized by length-dependent polyneuropathy which aligns with the patient's symptoms of distal paresthesia, neuropathic pain, and cramps in the lower limbs.<br>■ The symmetrical, sensory-motor, and axonal nature of the neuropathy seen on nerve conduction studies is typical for diabetic neuropathy.<br>■ Diabetes is a common cause of polyneuropathy in elderly patients, making it a highly probable diagnosis in this context.<br>2. **Chemotherapy-Induced Peripheral Neuropathy (CIPN)**<br>○ **Reasoning**:<br>■ The patient underwent chemotherapy for lung cancer, and symptoms of neuropathy began four years post-chemotherapy. Chemotherapy, especially agents like platinum compounds, taxanes, and vinca alkaloids, is well-known to cause peripheral neuropathy, which can be sensory and axonal.<br>■ The chronicity and persistence of symptoms align with CIPN, which can develop insidiously and progress even after the cessation of chemotherapy.<br>■ The mixed sensory-motor nature of the neuropathy could also be indicative of damage caused by chemotherapy.<br>3. **HBV-Related Neuropathy**<br>○ **Reasoning**:<br>■ Chronic hepatitis B virus (HBV) infection can be associated with neuropathies, although it is less common compared to diabetic neuropathy and CIPN.<br>■ The absence of other significant findings in the extensive screening lab tests might suggest the consideration of a more rare cause, like HBV-related neuropathy.<br>■ The chronic nature of HBV infection and its potential to cause systemic complications, including neuropathy, warrants its inclusion in the differential diagnoses.<br>**Most Likely Diagnosis**<br>Considering the patient's clinical history, laboratory findings, and the characteristics of the neuropathy, **diabetic neuropathy** emerges as the most likely diagnosis. This is supported by the presence of diabetes, the elevated HbA1c levels indicating poor glycemic control, and the typical presentation of a length-dependent, sensory-motor, axonal polyneuropathy commonly seen in diabetic patients. The onset of symptoms and their progression also fit well with the natural history of diabetic neuropathy.

48	A 45-year-old female patient presents with a 1-year history of pure sensory, symmetrical, length dependent polyneuropathy with subacute onset. The patient presents with distal leg paresthesia and neuropathic pain, with no ataxia, tremor, foot deformities, cranial nerve or autonomic involvement. Clinical examination shows hyperalgesia at pinprick test with normal tactile sensation. Past medical history is positive for multiple myeloma, for which the patient has undergone chemotherapy. Neuropathy symptoms started after the first chemotherapy cycle and remained stable ever since. Nerve conduction studies show pure a sensory, symmetric, axonal polyneuropathy. Screening lab tests including vitamin B12, folate, vitamin B6, vitamin E, HBV, HCV, ANA, ANCA, ENA, CRP, ESR, glycemia, HbA1c, copper and cryoglobulins are all negative, with positivity for IgG lambda paraprotein. There is no family history of polyneuropathy.	Chemotherapy-induced neuropathy	**Summary of Pertinent Clinical Information**<br>● **Patient Profile:** 45-year-old female<br>● **Primary Symptoms:** 1-year history of pure sensory, symmetrical, length-dependent polyneuropathy with subacute onset.<br>● **Specific Symptoms:** Distal leg paresthesia and neuropathic pain<br>● **Exclusions:** No ataxia, tremor, foot deformities, cranial nerve, or autonomic involvement<br>● **Clinical Examination Findings:** Hyperalgesia at pinprick test, normal tactile sensation<br>● **Medical History:** Multiple myeloma, chemotherapy<br>● **Symptom Onset:** After the first chemotherapy cycle, stable since then<br>● **Nerve Conduction Studies:** Pure sensory, symmetric, axonal polyneuropathy<br>● **Laboratory Tests:**<br>○ Negative: Vitamin B12, folate, vitamin B6, vitamin E, HBV, HCV, ANA, ANCA, ENA, CRP, ESR, glycemia, HbA1c, copper, cryoglobulins<br>○ Positive: IgG lambda paraprotein<br>● **Family History:** No family history of polyneuropathy<br>**Differential Diagnoses for Polyneuropathy**<br>1. **Chemotherapy-Induced Peripheral Neuropathy (CIPN)**<br>○ **Reasoning:**<br>■ The onset of neuropathy symptoms closely followed the initiation of chemotherapy.<br>■ The stability of symptoms since the onset is characteristic of CIPN.<br>■ Chemotherapy, particularly drugs such as platinum-based compounds, taxanes, and vinca alkaloids, is known to cause sensory polyneuropathy.<br>■ The patient's multiple myeloma treatment likely included such neurotoxic agents.<br>2. **Paraneoplastic Neuropathy**<br>○ **Reasoning:**<br>■ Multiple myeloma can be associated with paraneoplastic syndromes, which can include neuropathy.<br>■ The presence of IgG lambda paraprotein suggests an underlying plasma cell dyscrasia, which is linked to paraneoplastic neuropathies.<br>■ Paraneoplastic neuropathy typically presents as a subacute sensory neuropathy, which matches the patient's symptom profile.<br>■ However, the absence of systemic autoimmune markers and the timing of symptoms with chemotherapy initiation weigh against this being the primary cause.<br>3. **Monoclonal Gammopathy of Undetermined Significance (MGUS)-Related Neuropathy**<br>○ **Reasoning:**<br>■ MGUS is commonly associated with neuropathy, particularly in patients with multiple myeloma.<br>■ The presence of IgG lambda paraprotein aligns with the diagnosis of MGUS, which can cause a chronic, sensory, length-dependent polyneuropathy.<br>■ However, the temporal relationship with chemotherapy initiation suggests that MGUS may not be the primary cause, though it could contribute to the overall neuropathic process.<br>**Most Likely Diagnosis**<br>**Chemotherapy-Induced Peripheral Neuropathy (CIPN)**<br>● **Detailed Reasoning:**<br>○ The temporal association between the initiation of chemotherapy and the onset of neuropathy is strong evidence pointing towards CIPN.<br>○ Chemotherapy agents are well-documented causes of pure sensory, symmetric, axonal polyneuropathy.<br>○ The stability of symptoms after the initial onset is characteristic of CIPN, as the damage tends to plateau rather than progress if chemotherapy is continued or stopped.<br>○ Although the presence of IgG lambda paraprotein and multiple myeloma history could suggest paraneoplastic or MGUS-related neuropathy, the clear timeline correlating neuropathy with chemotherapy makes CIPN the most probable diagnosis.<br>In conclusion, the patient's neuropathy is most likely induced by chemotherapy used to treat her multiple myeloma, aligning with the onset, stability, and clinical findings.
49	A 79-year-old female patient presents with a 1-year history of pure sensory, symmetrical, length dependent polyneuropathy with insidious onset and progressive course. The patient presents with distal leg paresthesia, severe ataxia and postural tremor, which she reported to appear along with the other symptoms. Clinical examination does not show presence of foot deformities, cranial nerve or autonomic involvement. Past medical history is positive for diabetes. Nerve conduction studies show demyelinating, sensory-motor, symmetric polyneuropathy. Screening lab tests show the presence of IgM kappa monoclonal gammopathy, while other exams including vitamin B12, folate, vitamin B6, vitamin E, HCV, HBV, ANA, ANCA, ENA, CRP, ESR, glycemia, HbA1c, copper and cryoglobulins are all negative. There is no family history of polyneuropathy.	Anti-MAG polyneuropathy	**Summary of Pertinent Clinical Information**<br>● **Patient**: 79-year-old female<br>● **History**: 1-year history of pure sensory, symmetrical, length-dependent polyneuropathy<br>○ Onset: Insidious<br>○ Course: Progressive<br>● **Symptoms**:<br>○ Distal leg paresthesia<br>○ Severe ataxia<br>○ Postural tremor<br>● **Clinical Examination**:<br>○ No foot deformities<br>○ No cranial nerve involvement<br>○ No autonomic involvement<br>● **Past Medical History**: Diabetes<br>● **Nerve Conduction Studies**: Demyelinating, sensory-motor, symmetric polyneuropathy<br>● **Lab Tests**:<br>○ Positive: IgM kappa monoclonal gammopathy<br>○ Negative: Vitamin B12, folate, vitamin B6, vitamin E, HCV, HBV, ANA, ANCA, ENA, CRP, ESR, glycemia, HbA1c, copper, cryoglobulins<br>● **Family History**: No family history of polyneuropathy<br>**Differential Diagnoses for Polyneuropathy**<br>1. **Chronic Inflammatory Demyelinating Polyneuropathy (CIDP)**<br>○ **Reasoning**:<br>■ The patient's presentation of demyelinating, sensory-motor, symmetric polyneuropathy is characteristic of CIDP.

			■ CIDP often presents with progressive symptoms over time, similar to the patient's history.<br>■ Severe ataxia and postural tremor can be associated with CIDP.<br>■ The presence of an IgM kappa monoclonal gammopathy can be seen in CIDP, as there is an overlap with monoclonal gammopathy of undetermined significance (MGUS) and CIDP.<br>■ Diabetes is a known risk factor for CIDP.<br>2. **Monoclonal Gammopathy of Undetermined Significance (MGUS)-related Polyneuropathy**<br>○ **Reasoning**:<br>■ The presence of IgM kappa monoclonal gammopathy in the patient is significant and suggests a possible association with the neuropathy.<br>■ MGUS can lead to a demyelinating polyneuropathy, which fits the nerve conduction study findings.<br>■ This condition can present with sensory symptoms and progressive course without motor or autonomic involvement initially.<br>■ The length-dependent nature of the polyneuropathy and the absence of other systemic involvement align with MGUS-related neuropathy.<br>3. **Diabetic Neuropathy**<br>○ **Reasoning**:<br>■ The patient has a history of diabetes, which is a common cause of polyneuropathy.<br>■ Diabetic neuropathy often presents with a distal, symmetrical, sensory polyneuropathy.<br>■ However, diabetic neuropathy is typically axonal rather than demyelinating, which makes it less likely in this case given the nerve conduction study findings.<br>■ The progressive and insidious nature of the symptoms can be seen in diabetic neuropathy.<br>■ Severe ataxia and postural tremor are less common in typical diabetic neuropathy but can be seen in severe cases.<br>**Most Likely Diagnosis**<br>**Chronic Inflammatory Demyelinating Polyneuropathy (CIDP)**<br>● **Justification**:<br>○ The demyelinating nature of the polyneuropathy seen in nerve conduction studies is a hallmark of CIDP.<br>○ The presence of IgM kappa monoclonal gammopathy, while it could suggest MGUS, can also be associated with CIDP, and there is known overlap between the two conditions.<br>○ The clinical presentation, including severe ataxia and postural tremor, aligns well with CIDP.<br>○ Although the patient has diabetes, the demyelinating pattern is more suggestive of CIDP than diabetic neuropathy, which is typically axonal.<br>Therefore, based on the clinical presentation, nerve conduction study findings, and the presence of monoclonal gammopathy, CIDP is the most likely diagnosis for this patient.
50	A 57-year-old female patient presents with a five-year history of chronic, sensory-motor, symmetrical, length-dependent polyneuropathy with insidious onset and slow progression. The patient reports neuropathic pain in the feet, mild distal weakness in the lower limbs, and pes cavus, with no cranial nerve or autonomic involvement. Past medical history is unremarkable. Nerve conduction studies show a mixed axonal-demyelinating, sensory-motor polyneuropathy. Screening lab tests including vitamin B12, folate, vitamin B6, vitamin E, HBV, HCV, glycemia, HbA1c, ANA, ANCA, ENA, CRP, ESR, copper, cryoglobulins, and serum electrophoresis are all negative. Her mother has similar symptoms, while her brother has severe walking difficulties.	X-linked CMT	**Summary of Clinical Information**<br>● **Patient Profile**: 57-year-old female<br>● **History**: Five-year history of chronic, sensory-motor, symmetrical, length-dependent polyneuropathy with insidious onset and slow progression<br>● **Symptoms**:<br>○ Neuropathic pain in the feet<br>○ Mild distal weakness in lower limbs<br>○ Pes cavus (high-arched foot)<br>● **Physical Exam**:<br>○ No cranial nerve involvement<br>○ No autonomic involvement<br>● **Past Medical History**: Unremarkable<br>● **Nerve Conduction Studies**: Mixed axonal-demyelinating, sensory-motor polyneuropathy<br>● **Screening Lab Tests**: All negative (including vitamin B12, folate, vitamin B6, vitamin E, HBV, HCV, glycemia, HbA1c, ANA, ANCA, ENA, CRP, ESR, copper, cryoglobulins, serum electrophoresis)<br>● **Family History**:<br>○ Mother has similar symptoms<br>○ Brother has severe walking difficulties<br>**Differential Diagnoses for Polyneuropathy**<br>**1. Charcot-Marie-Tooth Disease (CMT)**<br>**Reasoning**:<br>● **Clinical Presentation**: The patient's chronic, sensory-motor, symmetrical, and length-dependent polyneuropathy with a slow progression and insidious onset is characteristic of CMT.<br>● **Family History**: The presence of similar symptoms in the mother and severe walking difficulties in the brother strongly suggests a hereditary pattern. CMT is a genetic disorder that often presents with a family history of similar symptoms.<br>● **Pes Cavus**: This foot deformity is a common finding in CMT.<br>● **Nerve Conduction Studies**: Mixed axonal-demyelinating findings are typical in some types of CMT, particularly CMT2, which can present with both axonal loss and demyelination.<br>**2. Hereditary Sensory and Autonomic Neuropathy (HSAN)**<br>**Reasoning**:<br>● **Clinical Presentation**: Although this condition often involves autonomic symptoms, there are variants (e.g., HSAN type II) that predominantly affect sensory and motor nerves with a chronic and progressive course.<br>● **Family History**: The genetic pattern with the mother and brother affected suggests a hereditary neuropathy.<br>● **Nerve Conduction Studies**: A mixed pattern can be seen in HSAN, though it is less common compared to purely sensory or autonomic features.<br>**3. Idiopathic Chronic Axonal Polyneuropathy (ICAP)**<br>**Reasoning**:<br>● **Clinical Presentation**: ICAP presents similarly with a chronic, length-dependent, sensory-motor polyneuropathy that progresses slowly.<br>● **Lab Tests**: Negative screening tests support the diagnosis as no specific cause is identified.<br>● **Nerve Conduction Studies**: Findings of mixed axonal-demyelinating polyneuropathy can be seen in ICAP, though it typically shows a predominant axonal pattern.<br>● **Family History**: ICAP is usually sporadic; the strong family history in this case makes it less likely but still possible if other causes are excluded.<br>**Most Likely Diagnosis**

			Given the clinical presentation, the significant family history, the findings on nerve conduction studies, and the presence of pes cavus, **Charcot-Marie-Tooth Disease (CMT)** is the most likely diagnosis. The hereditary nature, the type of neuropathy, and the specific clinical features such as pes cavus and mild distal weakness strongly point towards this genetic disorder. Further genetic testing to identify specific mutations associated with CMT would be the next step in confirming the diagnosis.
51	A 62-year-old male patient presents with an 8-month history of pure sensory, symmetrical, length dependent polyneuropathy with subacute onset. The patient presents with numbness and tingling and neuropathic pain distally in the lower limbs while he has no tremor, foot deformities, cranial nerve or autonomic involvement. Clinical examination reveals the presence of sensory ataxia. Past medical history is silent. Nerve conduction studies show a demyelinating, sensory-motor polyneuropathy and unilateral carpal tunnel syndrome. Screening lab tests including vitamin B12, folate, vitamin B6, vitamin E, HCV, HBV, ANA, ANCA, ENA, CRP, ESR, glycemia, HbA1c, copper, and cryoglobulins are all negative. Serum electrophoresis shows the presence of IgG paraprotein. There is no family history of polyneuropathy.	CIDP	**Step 1: Summarizing Pertinent Clinical Information**<br>**Patient Information:**<br>● Age: 62 years old<br>● Gender: Male<br>**Clinical Presentation:**<br>● 8-month history of pure sensory, symmetrical, length-dependent polyneuropathy<br>● Symptoms: Numbness, tingling, neuropathic pain in the lower limbs<br>● Onset: Subacute<br>● No tremor, foot deformities, cranial nerve, or autonomic involvement<br>● Presence of sensory ataxia on clinical examination<br>**Investigations:**<br>● Nerve conduction studies: Demyelinating, sensory-motor polyneuropathy; unilateral carpal tunnel syndrome<br>● Screening lab tests (including vitamin B12, folate, vitamin B6, vitamin E, HCV, HBV, ANA, ANCA, ENA, CRP, ESR, glycemia, HbA1c, copper, and cryoglobulins): Negative<br>● Serum electrophoresis: Presence of IgG paraprotein<br>**Additional Information:**<br>● Past medical history: Silent<br>● Family history: No history of polyneuropathy<br>**Step 2: Generating Differential Diagnoses for Polyneuropathy**<br>**1. Monoclonal Gammopathy of Undetermined Significance (MGUS)**<br>**Reasoning:**<br>● The presence of IgG paraprotein in serum electrophoresis is a significant clue.<br>● MGUS is known to be associated with demyelinating polyneuropathy, particularly when it presents with a paraprotein.<br>● Patients with MGUS may develop a sensory-motor polyneuropathy, and the subacute onset fits with the timeline.<br>● MGUS-associated neuropathy is often length-dependent and predominantly sensory, which matches the patient's presentation.<br>**2. Chronic Inflammatory Demyelinating Polyneuropathy (CIDP)**<br>**Reasoning:**<br>● CIDP can present with a demyelinating polyneuropathy, as evidenced by the nerve conduction studies.<br>● The subacute to chronic onset over 8 months is consistent with CIDP, which often progresses over time.<br>● Sensory ataxia is common in CIDP due to the involvement of large sensory fibers.<br>● While CIDP typically has a motor component, sensory-dominant variants exist.<br>● The presence of unilateral carpal tunnel syndrome can be seen in CIDP, as focal entrapment neuropathies are more common.<br>**3. POEMS Syndrome (Polyneuropathy, Organomegaly, Endocrinopathy, Monoclonal gammopathy, Skin changes)**<br>**Reasoning:**<br>● POEMS syndrome includes a monoclonal gammopathy, typically IgG or IgA, which is present in this patient.<br>● The polyneuropathy in POEMS is often demyelinating and can be length-dependent.<br>● Other characteristic features (organomegaly, endocrinopathy, skin changes) need to be excluded, but the presence of sensory ataxia and demyelinating neuropathy with IgG paraprotein raises suspicion.<br>● POEMS is rare but should be considered given the paraprotein and neuropathy.<br>**Step 3: Determining the Most Likely Diagnosis**<br>**Most Likely Diagnosis: Monoclonal Gammopathy of Undetermined Significance (MGUS)-associated Polyneuropathy**<br>**Reasoning:**<br>● The patient's presentation of subacute, sensory-dominant, length-dependent polyneuropathy is highly suggestive of MGUS-associated polyneuropathy, especially given the finding of IgG paraprotein in serum electrophoresis.<br>● CIDP, while a strong consideration, typically has more prominent motor involvement, and the absence of significant motor symptoms leans the diagnosis away from CIDP.<br>● POEMS syndrome is less likely due to the lack of other systemic features that are characteristic of the syndrome.<br>● MGUS is a common cause of polyneuropathy in older adults with an associated paraprotein and fits the clinical and electrophysiological findings in this patient.<br>Thus, the most likely diagnosis is **MGUS-associated polyneuropathy**. Further evaluation, including possible bone marrow biopsy and serial monitoring of the paraprotein, would be warranted to confirm this diagnosis and to rule out progression to multiple myeloma or other related conditions.
52	A 77-year-old male patient presents with a 6-month history of sensory-motor, symmetrical, non-length dependent polyneuropathy with subacute onset. The patient presents with weakness distributed proximally and distally in the lower limbs and distally in the upper limbs, and paresthesia in a stocking-glove distribution. Clinical examination reveals the presence of sensory ataxia, peripheral edema and hypertrichosis. There are no foot deformities, tremor, neuropathic pain, cranial nerve or autonomic involvement.	POEMS	**Summary of Pertinent Clinical Information**<br>● **Patient:** 77-year-old male<br>● **History:**<br>○ **Duration:** 6-month history<br>○ **Type:** Sensory-motor, symmetrical, non-length dependent polyneuropathy with subacute onset<br>○ **Symptoms:**<br>■ Weakness: Proximal and distal in lower limbs, distal in upper limbs<br>■ Paresthesia: Stocking-glove distribution<br>○ **Clinical Examination:**<br>■ Sensory ataxia<br>■ Peripheral edema<br>■ Hypertrichosis<br>○ **Absent Symptoms:** No foot deformities, tremor, neuropathic pain, cranial nerve or autonomic involvement

	Past medical history is positive for diabetes. Nerve conduction studies show mixed demyelinating-axonal, sensory-motor polyneuropathy. Screening lab tests including vitamin B12, folate, vitamin B6, vitamin E, HCV, HBV, ANA, ANCA, ENA, CRP, ESR, glycemia, copper and cryoglobulins are all negative, with positivity for IgM lambda paraprotein and raised HbA1c. There is no family history of polyneuropathy.		• **Past Medical History:** Diabetes<br>• **Nerve Conduction Studies:** Mixed demyelinating-axonal, sensory-motor polyneuropathy<br>• **Screening Lab Tests:**<br>○ Negative: Vitamin B12, folate, vitamin B6, vitamin E, HCV, HBV, ANA, ANCA, ENA, CRP, ESR, glycemia, copper, cryoglobulins<br>○ Positive: IgM lambda paraprotein, raised HbA1c<br>• **Family History:** No family history of polyneuropathy<br>**Differential Diagnoses for Polyneuropathy**<br>1. **Diabetic Neuropathy**<br>○ **Reasoning:**<br>■ Patient has a history of diabetes and raised HbA1c, indicating poor glycemic control.<br>■ Diabetic neuropathy often presents with mixed sensory and motor involvement, as seen in this patient.<br>■ The stocking-glove distribution of paresthesia is characteristic of diabetic neuropathy.<br>■ Sensory ataxia can be a feature in advanced diabetic neuropathy.<br>○ **Against:**<br>■ Diabetic neuropathy usually involves autonomic symptoms and is often length-dependent initially, which is not the case here.<br>■ Presence of IgM lambda paraprotein is not typically associated with diabetic neuropathy.<br>2. **Paraproteinemic Neuropathy (Monoclonal Gammopathy of Undetermined Significance - MGUS)**<br>○ **Reasoning:**<br>■ Presence of IgM lambda paraprotein suggests an underlying paraproteinemia.<br>■ Paraproteinemic neuropathies can present with mixed demyelinating and axonal features on nerve conduction studies.<br>■ Symmetrical, non-length dependent sensory-motor polyneuropathy with subacute onset fits the clinical presentation of MGUS-related neuropathy.<br>■ Peripheral edema and hypertrichosis can be associated with paraproteinemic neuropathy.<br>○ **Against:**<br>■ While sensory ataxia can occur, it is less common.<br>■ Lack of family history and absence of other systemic symptoms commonly seen with more malignant forms of paraproteinemia.<br>3. **Chronic Inflammatory Demyelinating Polyneuropathy (CIDP)**<br>○ **Reasoning:**<br>■ CIDP presents with a chronic, progressive course and can have a subacute onset.<br>■ Symmetrical, sensory-motor involvement with proximal and distal weakness fits the typical presentation of CIDP.<br>■ Mixed demyelinating-axonal features on nerve conduction studies are consistent with CIDP.<br>■ Peripheral edema can occur in CIDP due to protein loss through inflamed nerves.<br>○ **Against:**<br>■ The presence of IgM lambda paraprotein is not typical for CIDP but can occur in cases of paraprotein-associated CIDP.<br>■ CIDP typically has a relapsing-remitting course or steadily progressive course which needs to be corroborated with more detailed history.<br>**Most Likely Diagnosis**<br>Based on the clinical presentation, nerve conduction study findings, and positive IgM lambda paraprotein, the most likely diagnosis is **Paraproteinemic Neuropathy (Monoclonal Gammopathy of Undetermined Significance - MGUS)**. This diagnosis fits well with the subacute onset of symptoms, mixed demyelinating-axonal polyneuropathy, and associated paraprotein. Although diabetic neuropathy and CIDP are also considerations, the specific combination of IgM paraproteinemia and the clinical picture strongly points towards MGUS-related neuropathy. Further hematological workup and potential bone marrow biopsy may be warranted to confirm the underlying paraproteinemia and exclude more serious conditions like multiple myeloma.
53	A 56-year-old male patient presents with a 13-month history of pure motor, asymmetric, non-length dependent polyneuropathy with insidious onset and progressive course. The patient presents with weakness in both hands with asymmetric distribution and reports having frequent cramps and fasciculations. Clinical examination reveals intrinsic hand atrophy. There are no sensory deficits or ataxia, tremor, cranial nerve or autonomic involvement. Past medical history is positive for systemic lupus erythematosus. Nerve conduction studies show a pure motor, demyelinating multineuropathy. Screening lab tests including vitamin B12, folate, vitamin B6, vitamin E, HCV, HBV, ANA, ANCA, ENA, CRP, ESR, glycemia, HbA1c, copper, cryoglobulins, and serum electrophoresis are negative. Cervical spine MRI is normal. There is no family history of polyneuropathy.	MMN	**Summary of Pertinent Clinical Information**<br>• **Patient**: 56-year-old male<br>• **Presenting symptoms**:<br>○ Pure motor polyneuropathy<br>○ Asymmetric distribution<br>○ Non-length dependent<br>○ Insidious onset and progressive over 13 months<br>○ Weakness in both hands with asymmetric distribution<br>○ Frequent cramps and fasciculations<br>• **Physical examination**:<br>○ Intrinsic hand atrophy<br>○ No sensory deficits<br>○ No ataxia, tremor, cranial nerve or autonomic involvement<br>• **Medical history**: Systemic lupus erythematosus (SLE)<br>• **Diagnostic tests**:<br>○ Nerve conduction studies: Pure motor, demyelinating multineuropathy<br>○ Screening lab tests: Negative for vitamin B12, folate, vitamin B6, vitamin E, HCV, HBV, ANA, ANCA, ENA, CRP, ESR, glycemia, HbA1c, copper, cryoglobulins, and serum electrophoresis<br>○ Cervical spine MRI: Normal<br>• **Family history**: No history of polyneuropathy<br>**Differential Diagnoses for Polyneuropathy**<br>1. **Multifocal Motor Neuropathy (MMN)**<br>○ **Reasoning**: MMN typically presents with asymmetric, pure motor symptoms and can cause muscle cramps and fasciculations. It often has a demyelinating pattern on nerve conduction studies and does not involve sensory fibers, aligning with this patient's presentation. MMN can also present with progressive weakness and atrophy in the hands, fitting the clinical course described.<br>○ **Supporting Evidence**:

			■ Asymmetric, pure motor involvement<br>■ Demyelinating pattern on nerve conduction studies<br>■ Hand atrophy and fasciculations without sensory deficits<br>2. **Chronic Inflammatory Demyelinating Polyneuropathy (CIDP) - Pure Motor Variant**<br>○ **Reasoning**: CIDP is an acquired immune-mediated inflammatory disorder. Although it more commonly presents with sensory involvement, a pure motor variant exists. The chronic, progressive course over 13 months and the demyelinating features on nerve conduction studies support this diagnosis.<br>○ **Supporting Evidence**:<br>■ Progressive course over more than 8 weeks (chronic)<br>■ Demyelinating pattern on nerve conduction studies<br>■ No sensory involvement, compatible with the pure motor variant<br>3. **Paraneoplastic Neuropathy**<br>○ **Reasoning**: Paraneoplastic syndromes can cause various neuropathic presentations, including pure motor neuropathy. Although rare, certain cancers can present with neurological symptoms before the tumor is detected. The patient's age and the absence of other common causes suggest considering this etiology, especially given the negative extensive workup.<br>○ **Supporting Evidence**:<br>■ Pure motor presentation<br>■ Progressive and insidious onset<br>■ Extensive negative workup pointing towards less common causes<br>**Most Likely Diagnosis**<br>**Multifocal Motor Neuropathy (MMN)**<br>● **Reasoning**: MMN is the most likely diagnosis based on the following points:<br>○ The asymmetric and purely motor nature of the neuropathy<br>○ Presence of cramps and fasciculations<br>○ Demyelinating features on nerve conduction studies<br>○ Absence of sensory deficits, which distinguishes it from other polyneuropathies like CIDP<br>○ The clinical course and physical findings (hand atrophy) are highly consistent with MMN.<br>Thus, considering the patient's clinical presentation and diagnostic findings, MMN emerges as the most probable diagnosis. Further specific tests for anti-GM1 antibodies could be considered to confirm the diagnosis of MMN.
54	A 74-year-old male patient presents with a 5-year history of sensory-motor, predominantly sensory, symmetrical, length dependent polyneuropathy with insidious onset and slow progressive course. The patient presents with distal paresthesia and hypoesthesia associated with mild neuropathic pain in the feet. At clinical examination there are no tremor, foot deformities, cranial nerve or autonomic involvement. Past medical history is positive for diabetes and cardiac insufficiency. Nerve conduction studies show a sensory-motor, symmetric, length dependent, axonal polyneuropathy. Screening lab tests show hyperglycemia and elevated HbA1c. Remaining exams including vitamin B12, folate, vitamin B6, vitamin E, HBV, HCV, ANA, ANCA, ENA, CRP, ESR, copper, cryoglobulins, and serum electrophoresis are negative. There is family history of polyneuropathy (father had similar symptoms).	Diabetic neuropathy	**Summary of Pertinent Clinical Information**<br>● **Patient Demographics:** 74-year-old male<br>● **History of Present Illness:**<br>○ 5-year history of sensory-motor, predominantly sensory, symmetrical, length-dependent polyneuropathy<br>○ Insidious onset and slow progressive course<br>○ Distal paresthesia and hypoesthesia<br>○ Mild neuropathic pain in the feet<br>● **Clinical Examination:**<br>○ No tremor, foot deformities, cranial nerve or autonomic involvement<br>● **Past Medical History:**<br>○ Diabetes<br>○ Cardiac insufficiency<br>● **Nerve Conduction Studies:**<br>○ Sensory-motor, symmetric, length-dependent, axonal polyneuropathy<br>● **Laboratory Findings:**<br>○ Hyperglycemia<br>○ Elevated HbA1c<br>○ Negative for vitamin B12, folate, vitamin B6, vitamin E, HBV, HCV, ANA, ANCA, ENA, CRP, ESR, copper, cryoglobulins, and serum electrophoresis<br>● **Family History:**<br>○ Father had similar symptoms (suggesting possible hereditary component)<br>**Differential Diagnoses**<br>**1. Diabetic Polyneuropathy**<br>**Reasoning:**<br>● **Diabetes as a Major Risk Factor:** The patient has a history of diabetes with hyperglycemia and elevated HbA1c, indicating poor glycemic control, which is a well-known cause of diabetic neuropathy.<br>● **Typical Clinical Presentation:** The symmetrical, length-dependent, sensory-motor nature of the neuropathy aligns with diabetic polyneuropathy, which commonly presents in this manner.<br>● **Nerve Conduction Studies:** Findings of axonal polyneuropathy are consistent with diabetic neuropathy.<br>● **Exclusion of Other Causes:** Negative screening for other potential causes supports diabetes as a primary factor.<br>**2. Hereditary Sensory and Motor Neuropathy (HSMN)**<br>**Reasoning:**<br>● **Family History:** The patient's father had similar symptoms, suggesting a hereditary component.<br>● **Clinical Presentation:** The insidious onset and slow progression over many years are consistent with hereditary neuropathies, which often present in this manner.<br>● **Symmetric and Length-dependent Nature:** These characteristics are common in hereditary sensory and motor neuropathies.<br>**3. Idiopathic Axonal Polyneuropathy (IAP)**<br>**Reasoning:**<br>● **Common in Elderly:** IAP is relatively common in older adults and often presents similarly to the patient's symptoms.<br>● **Negative Extensive Workup:** The extensive negative screening for other causes could suggest an idiopathic origin.<br>● **Clinical and Electrophysiological Findings:** The symmetric, length-dependent, axonal nature of the neuropathy aligns with IAP.

			**Most Likely Diagnosis**<br>**Diabetic Polyneuropathy**<br>**Reasoning:**<br>● The patient's history of diabetes, poor glycemic control (as evidenced by hyperglycemia and elevated HbA1c), and the typical presentation of diabetic neuropathy strongly point towards this diagnosis.<br>● While hereditary neuropathy is a consideration given the family history, the patient's long-standing diabetes and the characteristic findings on nerve conduction studies make diabetic polyneuropathy the most likely diagnosis.<br>● Idiopathic axonal polyneuropathy remains a possible but less likely diagnosis given the clear presence of diabetes, which is a well-established cause of polyneuropathy in the patient's clinical context.
55	A 70-year-old male patient presents with a 2-year history of sensory-motor, symmetrical, length dependent polyneuropathy with subacute onset. He has been suffering from neuropathic pain in his feet for several years. However, over the past two years, he has also noticed progressive weakness in the feet and wasting of the intrinsic muscles of both hands. Additionally, he reports symptoms suggestive of orthostatic hypothension, and unintentional weight loss of approximately 5 kg over the past year. Clinical examination does not show presence of tremor, foot deformities, or cranial nerve involvement. Past medical history is positive for cardiac insufficiency, diabetes, and IgG lambda MGUS. Nerve conduction studies show a mixed demyelinating-axonal, sensory-motor, symmetric polyneuropathy. Screening lab tests reveal hyperglycemia, elevated HbA1c, presence of IgG lambda paraprotein, and increased creatinine levels. Remaining exams including vitamin B12, folate, vitamin B6, vitamin E, HBV, HCV, ANA, ANCA, ENA, CRP, ESR, copper, and cryoglobulins are normal. There is no family history of polyneuropathy.	Amyloidosis -associated polyneuropathy	**Summary of Pertinent Clinical Information**<br>● **Patient Profile**: 70-year-old male<br>● **History**:<br>○ Sensory-motor, symmetrical, length-dependent polyneuropathy with subacute onset over the past 2 years<br>○ Neuropathic pain in feet for several years<br>○ Progressive weakness in feet<br>○ Wasting of intrinsic muscles of both hands<br>○ Symptoms of orthostatic hypotension<br>○ Unintentional weight loss (~5 kg in the past year)<br>● **Clinical Examination**:<br>○ No tremor<br>○ No foot deformities<br>○ No cranial nerve involvement<br>● **Past Medical History**:<br>○ Cardiac insufficiency<br>○ Diabetes<br>○ IgG lambda monoclonal gammopathy of undetermined significance (MGUS)<br>● **Nerve Conduction Studies**:<br>○ Mixed demyelinating-axonal, sensory-motor, symmetric polyneuropathy<br>● **Screening Lab Tests**:<br>○ Hyperglycemia<br>○ Elevated HbA1c<br>○ Presence of IgG lambda paraprotein<br>○ Increased creatinine levels<br>○ Normal levels: Vitamin B12, folate, vitamin B6, vitamin E, HBV, HCV, ANA, ANCA, ENA, CRP, ESR, copper, cryoglobulins<br>● **Family History**: Negative for polyneuropathy<br>**Differential Diagnoses for Polyneuropathy**<br>**1. Diabetic Polyneuropathy**<br>**Reasoning**:<br>● The patient has a history of diabetes, and hyperglycemia with elevated HbA1c supports poorly controlled diabetes.<br>● Diabetic polyneuropathy often presents as a length-dependent, sensory-motor polyneuropathy, which aligns with the patient's symptoms.<br>● Neuropathic pain and progressive weakness are common in diabetic neuropathy.<br>● However, diabetic neuropathy typically does not cause significant muscle wasting in the hands or significant orthostatic hypotension without other contributing factors.<br>**2. Monoclonal Gammopathy of Undetermined Significance (MGUS)-Related Neuropathy**<br>**Reasoning**:<br>● The patient has IgG lambda MGUS, which is a known cause of neuropathy.<br>● MGUS-related neuropathy can present with a mixed demyelinating-axonal pattern on nerve conduction studies.<br>● Symptoms such as sensory disturbances, motor weakness, and autonomic dysfunction (e.g., orthostatic hypotension) can occur with MGUS-related neuropathy.<br>● The weight loss and lack of other systemic involvement (e.g., no evidence of amyloidosis) also point towards MGUS as a significant consideration.<br>**3. Amyloid Neuropathy (Light Chain Amyloidosis - AL Amyloidosis)**<br>**Reasoning**:<br>● The presence of MGUS raises the possibility of AL amyloidosis, where the monoclonal light chains (lambda in this case) deposit in tissues.<br>● Amyloid neuropathy often presents with a progressive sensory-motor polyneuropathy and can also involve autonomic symptoms (e.g., orthostatic hypotension).<br>● Weight loss and renal impairment (increased creatinine) can be manifestations of systemic amyloidosis.<br>● The mixed demyelinating-axonal pattern on nerve conduction studies is consistent with amyloid neuropathy.<br>**Most Likely Diagnosis**<br>**Amyloid Neuropathy (Light Chain Amyloidosis - AL Amyloidosis)**<br>**Reasoning**:<br>● The presence of IgG lambda paraprotein, progressive neuropathy with both sensory and motor involvement, autonomic symptoms (orthostatic hypotension), and significant weight loss are highly suggestive of AL amyloidosis.<br>● Renal impairment (increased creatinine) further supports the diagnosis, as kidney involvement is common in amyloidosis.<br>● The mixed demyelinating-axonal pattern on nerve conduction studies aligns with amyloid neuropathy.<br>● While diabetic neuropathy and MGUS-related neuropathy are possible, the combination of symptoms and laboratory findings makes amyloid neuropathy the most compelling diagnosis.<br>Further diagnostic work-up, including biopsy (e.g., fat pad, nerve, or renal biopsy) to confirm amyloid deposits and typing of the amyloid (immunohistochemistry, mass spectrometry), would be essential to establish the diagnosis definitively.
56	A 53-year-old female patient presents with a 42-year history of sensory-motor, symmetrical, length-dependent polyneuropathy with insidious onset and	CMT1	**Summary of Pertinent Clinical Information**<br>● **Patient**: 53-year-old female<br>● **Symptoms**:<br>○ 42-year history of sensory-motor, symmetrical, length-dependent polyneuropathy

	slowly progressive course. The patient reports distal leg paresthesia, scoliosis, sensory ataxia, distal weakness in the lower limbs, cramps, hammertoes, and pes cavus. Clinical examination does not reveal tremor, cranial nerve involvement, or autonomic dysfunction. Past medical history is unremarkable. Nerve conduction studies show a demyelinating, symmetric, sensory-motor polyneuropathy. Screening lab tests including vitamin B12, folate, vitamin B6, vitamin E, HBV, HCV, glycemia, HbA1c, ANA, ANCA, ENA, CRP, ESR, copper, cryoglobulins, and serum electrophoresis are all negative. Her mother, brother, and sister have walking difficulties.		○ Distal leg paresthesia<br>○ Sensory ataxia<br>○ Distal weakness in the lower limbs<br>○ Cramps<br>○ Hammertoes<br>○ Pes cavus<br>● **Physical Examination**:<br>○ No tremor<br>○ No cranial nerve involvement<br>○ No autonomic dysfunction<br>● **Past Medical History**: Unremarkable<br>● **Nerve Conduction Studies**: Demyelinating, symmetric, sensory-motor polyneuropathy<br>● **Lab Tests**: All negative (including vitamin B12, folate, vitamin B6, vitamin E, HBV, HCV, glycemia, HbA1c, ANA, ANCA, ENA, CRP, ESR, copper, cryoglobulins, and serum electrophoresis)<br>● **Family History**: Mother, brother, and sister have walking difficulties<br>**Differential Diagnoses for Polyneuropathy**<br>**1. Charcot-Marie-Tooth Disease (CMT)**<br>● **Reasoning**:<br>○ The clinical presentation of a slowly progressive, symmetrical, sensory-motor polyneuropathy with a 42-year history and onset in childhood is highly suggestive of a hereditary neuropathy.<br>○ The presence of distal muscle weakness, sensory ataxia, and characteristic foot deformities like hammertoes and pes cavus are classic features of CMT.<br>○ Family history of walking difficulties in first-degree relatives supports a hereditary pattern, which is common in CMT.<br>○ The demyelinating nature of the polyneuropathy seen on nerve conduction studies aligns with CMT type 1, the most common type associated with demyelination.<br>**2. Hereditary Sensory and Motor Neuropathy (HSMN)**<br>● **Reasoning**:<br>○ HSMN is another hereditary neuropathy that presents with similar features to CMT, including a slowly progressive course, distal sensory loss, and motor weakness.<br>○ The absence of tremor, cranial nerve involvement, and autonomic dysfunction fits with HSMN.<br>○ The negative screening lab tests and unremarkable past medical history rule out many acquired causes, further suggesting a hereditary neuropathy.<br>○ The familial pattern of walking difficulties adds weight to this diagnosis.<br>**3. Chronic Inflammatory Demyelinating Polyneuropathy (CIDP)**<br>● **Reasoning**:<br>○ CIDP can present with a slowly progressive course and can sometimes mimic hereditary neuropathies.<br>○ Demyelination seen on nerve conduction studies is consistent with CIDP.<br>○ However, the extremely long duration (42 years) without significant disability progression is less typical for CIDP, which usually presents with relapsing-remitting or more rapidly progressive symptoms.<br>○ The lack of response to immunosuppressive or immunomodulatory treatments (if any were tried) and the strong family history make CIDP less likely but still a consideration due to overlapping features.<br>**Most Likely Diagnosis**<br>The most likely diagnosis for this patient is **Charcot-Marie-Tooth Disease (CMT)**. This conclusion is based on the following points:<br>● **Long History**: The very long duration of the disease (42 years) with a slowly progressive course is characteristic of CMT.<br>● **Clinical Features**: The presence of distal weakness, sensory ataxia, hammertoes, and pes cavus are hallmark signs of CMT.<br>● **Family History**: The strong family history of walking difficulties suggests a hereditary condition, which is consistent with CMT.<br>● **Nerve Conduction Studies**: The demyelinating nature of the polyneuropathy aligns with CMT type 1.<br>● **Negative Laboratory Tests**: The negative results for various screening tests for other potential causes further support a hereditary etiology like CMT.<br>In conclusion, based on the combination of clinical features, family history, and nerve conduction study findings, Charcot-Marie-Tooth Disease is the most likely diagnosis for this patient.
57	A 65-year old male patient presents with a 3-year history of sensory, symmetrical, length dependent polyneuropathy with insidious onset. The patient presents with sensory ataxia, neuropathic pain, distal hypoesthesia up to the calves and mild dysarthria. Clinical examination does not reveal presence of foot deformities, tremor, or weakness. Past medical history shows chronic cough and mild cardiac insufficiency. Nerve conduction studies show a pure sensory, axonal polyneuropathy. Screening lab tests including vitamin B12, folate, vitamin B6, vitamin E, HBV, HCV, glycemia, HbA1c, ANA, ANCA, ENA, CRP, ESR, copper, cryoglobulins, and serum electrophoresis are all negative. There is no family history of polyneuropathy.	CANVAS	**Step-by-Step Analysis of the Clinical Scenario**<br>**Summary of Pertinent Clinical Information**<br>1. **Patient Details:**<br>○ Age: 65 years old<br>○ Gender: Male<br>2. **Symptom History:**<br>○ Duration: 3 years<br>○ Type: Sensory, symmetrical, length-dependent polyneuropathy<br>○ Onset: Insidious<br>○ Symptoms:<br>■ Sensory ataxia<br>■ Neuropathic pain<br>■ Distal hypoesthesia up to the calves<br>■ Mild dysarthria<br>3. **Clinical Examination:**<br>○ No foot deformities<br>○ No tremor<br>○ No weakness<br>4. **Medical History:**<br>○ Chronic cough<br>○ Mild cardiac insufficiency<br>5. **Diagnostic Findings:**

			○ Nerve Conduction Studies: Pure sensory, axonal polyneuropathy<br>6. **Screening Lab Tests:**<br>○ Negative results for:<br>■ Vitamin B12, folate, vitamin B6, vitamin E<br>■ HBV, HCV<br>■ Glycemia, HbA1c<br>■ ANA, ANCA, ENA, CRP, ESR, copper, cryoglobulins<br>■ Serum electrophoresis<br>7. **Family History:**<br>○ No family history of polyneuropathy<br>**Differential Diagnoses for Polyneuropathy**<br>**1. Idiopathic Sensory Polyneuropathy**<br>**Reasoning:**<br>● **Clinical Presentation:**<br>○ Sensory, symmetrical, length-dependent polyneuropathy without motor involvement is typical for idiopathic sensory polyneuropathy.<br>○ The insidious onset and chronic progression over 3 years fit the pattern.<br>○ Sensory ataxia, neuropathic pain, and distal hypoesthesia are consistent with this diagnosis.<br>● **Negative Screening Tests:**<br>○ Extensive lab workup excluding vitamin deficiencies, infections, autoimmune markers, metabolic causes (e.g., diabetes), and other systemic diseases increases the likelihood of an idiopathic etiology.<br>● **Demographics:**<br>○ This condition is more common in older adults, aligning with the patient's age.<br>**2. Paraneoplastic Neuropathy**<br>**Reasoning:**<br>● **Associated Symptoms:**<br>○ Chronic cough and mild cardiac insufficiency could be indirect signs of an underlying malignancy, which is sometimes associated with paraneoplastic syndromes.<br>● **Neurological Findings:**<br>○ Paraneoplastic neuropathies often present with sensory neuropathy, which can be length-dependent and symmetrical.<br>○ The presence of mild dysarthria might indicate central nervous system involvement, sometimes seen in paraneoplastic syndromes.<br>● **Screening and Examination:**<br>○ Despite the negative lab tests, some paraneoplastic syndromes might not be detected with standard screening and might require specific antibody testing or imaging studies to identify an occult malignancy.<br>**3. Chronic Inflammatory Demyelinating Polyneuropathy (CIDP) with Atypical Presentation**<br>**Reasoning:**<br>● **Clinical Overlap:**<br>○ Although CIDP typically presents with both sensory and motor involvement, some variants can present predominantly with sensory symptoms.<br>● **Symptom Duration and Progression:**<br>○ The chronic and progressive nature of the symptoms aligns with CIDP.<br>● **Negative Initial Workup:**<br>○ The lack of inflammatory markers and normal nerve conduction studies pointing to an axonal rather than demyelinating process is atypical but does not completely exclude CIDP, particularly if there is an atypical presentation.<br>● **Further Testing Required:**<br>○ Additional tests such as cerebrospinal fluid (CSF) analysis or repeat and more detailed electrophysiological studies might be necessary to rule out this diagnosis.<br>**Most Likely Diagnosis**<br>Based on the clinical presentation, history, and diagnostic findings, the most likely diagnosis is **Idiopathic Sensory Polyneuropathy**.<br>**Rationale:**<br>● The pure sensory, symmetrical, length-dependent nature of the polyneuropathy, combined with the negative extensive screening workup, aligns strongly with idiopathic sensory polyneuropathy.<br>● This condition is more common in older adults and fits the patient's age and symptomatology.<br>● The chronic progression over three years without significant motor involvement or other systemic findings makes this the most probable diagnosis.<br>**Next Steps:**<br>● Given the current findings, further evaluation might include more specialized tests if any new symptoms develop, but at this stage, symptomatic management and regular monitoring are appropriate.<br>● If paraneoplastic syndrome remains a concern, targeted cancer screening based on the patient's symptoms (chronic cough and cardiac issues) might be considered.
58	A 71-year-old female patient presents with a 2-year history of chronic, pure sensory, symmetrical, length dependent polyneuropathy with subacute onset. The patient presents with distal leg paresthesia and neuropathic pain associated with cramps. There are no foot deformities, weakness, ataxia, tremor, cranial nerve or autonomic involvement. Past medical history shows diabetes and lymphoma, for which the patient has undergone chemotherapy. Symptoms started during chemotherapy. Nerve conduction studies	Chemotherapy-induced neuropathy	**Summary of Pertinent Clinical Information**<br>● **Patient Profile**: 71-year-old female<br>● **History**: 2-year history of chronic, pure sensory, symmetrical, length-dependent polyneuropathy with subacute onset<br>● **Symptoms**:<br>○ Distal leg paresthesia<br>○ Neuropathic pain<br>○ Cramps<br>● **Examination Findings**: No foot deformities, weakness, ataxia, tremor, cranial nerve or autonomic involvement<br>● **Medical History**:<br>○ Diabetes<br>○ Lymphoma (treated with chemotherapy)<br>● **Symptom Onset**: Symptoms started during chemotherapy<br>● **Nerve Conduction Studies**: Mixed sensory-motor, axonal, symmetric polyneuropathy and unilateral carpal tunnel syndrome

	show mixed sensory-motor, axonal, symmetric polyneuropathy and unilateral carpal tunnel syndrome. Screening lab tests including vitamin B12, folate, vitamin B6, vitamin E, HBV, HCV, ANA, ANCA, ENA, CRP, ESR, glycemia, copper, cryoglobulins and serum electrophoresis are all negative. HbA1c is elevated. There is no family history of polyneuropathy.		● **Laboratory Findings**:<br>○ Negative for vitamin B12, folate, vitamin B6, vitamin E, HBV, HCV, ANA, ANCA, ENA, CRP, ESR, glycemia, copper, cryoglobulins, and serum electrophoresis<br>○ Elevated HbA1c<br>● **Family History**: No family history of polyneuropathy<br>**Differential Diagnoses for Polyneuropathy**<br>1. **Chemotherapy-Induced Peripheral Neuropathy (CIPN)**<br>○ **Reasoning**:<br>■ The onset of symptoms correlates with the period during which the patient was undergoing chemotherapy.<br>■ Lymphoma treatments often involve neurotoxic agents such as platinum compounds (cisplatin, carboplatin), vinca alkaloids (vincristine), or taxanes (paclitaxel), which are known to cause peripheral neuropathy.<br>■ The patient's symptoms are consistent with CIPN, which typically presents as a length-dependent, symmetrical sensory neuropathy.<br>■ CIPN can also manifest with mixed sensory-motor features, aligning with the nerve conduction study findings.<br>2. **Diabetic Polyneuropathy**<br>○ **Reasoning**:<br>■ The patient has a history of diabetes with an elevated HbA1c, indicating poor glycemic control.<br>■ Diabetic polyneuropathy is a common complication of diabetes, often presenting as a distal symmetric polyneuropathy.<br>■ Although typically a more gradual onset, poor glycemic control can accelerate neuropathy progression.<br>■ The clinical presentation of distal paresthesia, pain, and cramps is consistent with diabetic neuropathy.<br>3. **Paraneoplastic Neuropathy**<br>○ **Reasoning**:<br>■ The patient has a history of lymphoma, which can be associated with paraneoplastic syndromes, including neuropathy.<br>■ Paraneoplastic neuropathies are typically subacute in onset and can be pure sensory or mixed sensory-motor.<br>■ The absence of autonomic involvement does not rule out paraneoplastic neuropathy, as it can present variably.<br>■ While less common than chemotherapy-induced or diabetic neuropathy, paraneoplastic processes should be considered given the patient's oncology history.<br>**Most Likely Diagnosis**<br>Based on the provided information, the most likely diagnosis is **Chemotherapy-Induced Peripheral Neuropathy (CIPN)**. The temporal association between the onset of symptoms and the period of chemotherapy, combined with the characteristic presentation of a length-dependent, symmetrical, sensory-dominant polyneuropathy, strongly suggests CIPN. Additionally, nerve conduction studies support a mixed sensory-motor neuropathy, which is compatible with CIPN. While diabetic polyneuropathy is a strong consideration, the correlation with chemotherapy and the timing of symptom onset make CIPN the more likely primary diagnosis.
59	An 80-year-old male patient presents with a 1-year history of sensory-motor, symmetrical, length dependent polyneuropathy with subacute onset. The patient presents with paresthesia and sensory ataxia without tremor, neuropathic pain, foot deformities, cranial nerve or autonomic involvement. Clinical examination shows distal weakness and sensory deficits (hypoesthesia) in the lower limbs. Past medical history is positive for colorectal cancer and plasmacytoma, for which the patient has undergone chemotherapy. Neuropathy symptoms started a few weeks after the last chemotherapy treatment cycle. Nerve conduction studies show a sensory-motor, symmetric, length-dependent, axonal polyneuropathy. Screening lab tests including vitamin B12, folate, vitamin B6, vitamin E, HBV, HCV, ANA, ANCA, ENA, CRP, ESR, glycemia, HbA1c, copper and cryoglobulins are all negative, with positivity for IgG kappa paraprotein. There is no family history of polyneuropathy.	Chemotherapy-induced neuropathy	**Summary of Pertinent Clinical Information**<br>● **Patient Demographics**: 80-year-old male<br>● **History**: 1-year history of sensory-motor, symmetrical, length-dependent polyneuropathy with subacute onset<br>● **Symptoms**:<br>○ Paresthesia and sensory ataxia without tremor<br>○ Neuropathic pain<br>○ No foot deformities<br>○ No cranial nerve involvement<br>○ No autonomic involvement<br>● **Clinical Findings**:<br>○ Distal weakness<br>○ Sensory deficits (hypoesthesia) in the lower limbs<br>● **Past Medical History**:<br>○ Colorectal cancer<br>○ Plasmacytoma (chemotherapy received)<br>● **Timing**: Symptoms started a few weeks after the last chemotherapy treatment cycle<br>● **Nerve Conduction Studies**: Sensory-motor, symmetric, length-dependent, axonal polyneuropathy<br>● **Lab Tests**:<br>○ Normal: Vitamin B12, folate, vitamin B6, vitamin E, HBV, HCV, ANA, ANCA, ENA, CRP, ESR, glycemia, HbA1c, copper, cryoglobulins<br>○ Positive: IgG kappa paraprotein<br>● **Family History**: No family history of polyneuropathy<br>**Differential Diagnoses for Polyneuropathy**<br>1. **Chemotherapy-Induced Peripheral Neuropathy (CIPN)**<br>○ **Reasoning**: The patient's neuropathy symptoms began a few weeks after the last chemotherapy treatment, suggesting a temporal relationship. Chemotherapy agents are known to cause toxic effects on peripheral nerves, leading to neuropathy. The sensory-motor and symmetrical nature of the neuropathy fits with common presentations of CIPN. The subacute onset and length-dependent pattern further support this diagnosis.<br>2. **Paraneoplastic Neuropathy**<br>○ **Reasoning**: The patient has a history of colorectal cancer and plasmacytoma. Paraneoplastic syndromes are rare, but they can present as polyneuropathy. The presence of IgG kappa paraprotein is suggestive of a monoclonal gammopathy, which can be associated with paraneoplastic phenomena. The absence of cranial nerve and autonomic involvement makes this less typical but still possible, given the paraneoplastic context.<br>3. **Monoclonal Gammopathy of Undetermined Significance (MGUS) with Neuropathy**<br>○ **Reasoning**: The detection of IgG kappa paraprotein indicates a monoclonal gammopathy. MGUS can be associated with polyneuropathy, especially in older adults. The clinical presentation of sensory-motor, symmetrical, and axonal polyneuropathy is consistent with neuropathy related to MGUS. While this condition can be indolent, it may explain the chronic and progressive nature of the symptoms.<br>**Most Likely Diagnosis**<br>The most likely diagnosis for this patient is **Chemotherapy-Induced Peripheral Neuropathy (CIPN)**. The temporal relationship between the onset of neuropathy symptoms and the completion of chemotherapy is a strong indicator. The pattern of sensory-motor, symmetrical, and axonal neuropathy is characteristic of CIPN. While other potential diagnoses, such as paraneoplastic neuropathy and MGUS with neuropathy, are considered, the direct link to chemotherapy and the clinical presentation align more closely with CIPN.

60	A 22-year-old male patient presents with a 20-year history of sensory-motor, symmetrical, length dependent polyneuropathy with insidious onset and slow progression. The patient is wheelchair bound and presents with sensory ataxia, severe distal weakness and atrophy in the four limbs, hypoestesia up to calf and pes cavus, with no cranial nerve or autonomic involvement. Past medical history is unremarkable. Nerve conduction studies show a demyelinating, sensory-motor polyneuropathy. Screening lab tests including vitamin B12, folate, vitamin B6, vitamin E, HBV, HCV, glycemia, HbA1c, ANA, ANCA, ENA, CRP, ESR, copper, cryoglobulins, and serum electrophoresis are all negative. One of his five brothers has similar symptoms.	CMT4	**Step-by-Step Clinical Analysis of the Case**<br>**Patient Summary**<br>● **Age/Gender:** 22-year-old male<br>● **Symptoms Duration:** 20-year history<br>● **Symptom Type:** Sensory-motor, symmetrical, length-dependent polyneuropathy<br>● **Onset/Progression:** Insidious onset, slow progression<br>● **Current Condition:** Wheelchair-bound<br>● **Key Symptoms:** Sensory ataxia, severe distal weakness and atrophy in all four limbs, hypoesthesia up to calves, pes cavus<br>● **No Involvement:** Cranial nerves, autonomic system<br>● **Past Medical History:** Unremarkable<br>● **Family History:** One brother with similar symptoms<br>● **Nerve Conduction Studies:** Demyelinating, sensory-motor polyneuropathy<br>● **Screening Lab Tests:** Negative for vitamin B12, folate, vitamin B6, vitamin E, HBV, HCV, glycemia, HbA1c, ANA, ANCA, ENA, CRP, ESR, copper, cryoglobulins, serum electrophoresis<br>**Differential Diagnosis for Polyneuropathy**<br>1. **Charcot-Marie-Tooth Disease (CMT)**<br>○ **Reasoning:**<br>■ **Onset and Progression:** The slow progression and onset in childhood are characteristic of CMT.<br>■ **Symmetrical, Length-Dependent:** CMT commonly presents with symmetrical, length-dependent polyneuropathy.<br>■ **Pes Cavus:** A hallmark feature of CMT is pes cavus, a high-arched foot deformity.<br>■ **Sensory-Motor Polyneuropathy:** Demyelinating nature observed in nerve conduction studies fits with certain subtypes of CMT.<br>■ **Family History:** The presence of similar symptoms in a sibling strongly suggests a hereditary component, consistent with CMT.<br>2. **Hereditary Neuropathy with Liability to Pressure Palsies (HNPP)**<br>○ **Reasoning:**<br>■ **Onset and Progression:** Although HNPP often presents with recurrent pressure palsies, it can sometimes present with a more generalized polyneuropathy that progresses slowly.<br>■ **Sensory-Motor Polyneuropathy:** Demyelinating nature in nerve conduction studies is seen in HNPP.<br>■ **Family History:** Genetic predisposition with family history of similar symptoms supports this diagnosis.<br>3. **Metachromatic Leukodystrophy (MLD)**<br>○ **Reasoning:**<br>■ **Onset and Progression:** MLD can present with progressive polyneuropathy in childhood.<br>■ **Sensory-Motor Polyneuropathy:** Demyelinating polyneuropathy observed in nerve conduction studies can be seen in MLD.<br>■ **Family History:** Autosomal recessive inheritance pattern fits with multiple siblings affected.<br>**Most Likely Diagnosis**<br>Based on the clinical presentation, family history, nerve conduction study findings, and the characteristic physical findings such as pes cavus, the most likely diagnosis is **Charcot-Marie-Tooth Disease (CMT)**. This hereditary neuropathy commonly presents with the described features, and the demyelinating type aligns with specific CMT subtypes, making it the most probable diagnosis. Further genetic testing would be useful to confirm this diagnosis and to identify the specific subtype of CMT.
61	A 71-year-old male patient presents with a 4-year history of pure sensory, symmetrical, length dependent polyneuropathy with insidious onset. The patient presents with neuropathic pain and paresthesia in the feet. There are no sensory ataxia or tremor, foot deformities, cranial nerve or autonomic involvement. Clinical examination shows the presence of tactile hypoesthesia and hyperalgesia in the feet. Past medical history is positive for psoriasis and type 2 diabetes. Nerve conduction studies show a pure sensory, symmetric, axonal, length dependent polyneuropathy and unilateral carpal tunnel syndrome. Screening lab tests show positivity for ANA antibodies, mildly raised CRP and ESR, increased levels of glycemia and HbA1c. Remaining exams, including vitamin B12, vitamin B6, vitamin E, HBV, HCV, ANCA, ENA, copper, cryoglobulins and serum electrophoresis are all negative. There is no family history of polyneuropathy.	Diabetic neuropathy	**Summary of Pertinent Clinical Information**<br>● **Patient Demographics**: 71-year-old male<br>● **Chief Complaint**: 4-year history of pure sensory, symmetrical, length-dependent polyneuropathy with insidious onset<br>● **Symptoms**: Neuropathic pain and paresthesia in the feet<br>● **Negative Symptoms**: No sensory ataxia, tremor, foot deformities, cranial nerve, or autonomic involvement<br>● **Clinical Examination**: Tactile hypoesthesia and hyperalgesia in the feet<br>● **Medical History**: Psoriasis, type 2 diabetes<br>● **Nerve Conduction Studies**: Pure sensory, symmetric, axonal, length-dependent polyneuropathy and unilateral carpal tunnel syndrome<br>● **Lab Findings**:<br>○ Positive ANA antibodies<br>○ Mildly raised CRP and ESR<br>○ Increased glycemia and HbA1c<br>○ Negative for vitamin B12, B6, E, HBV, HCV, ANCA, ENA, copper, cryoglobulins, and serum electrophoresis<br>● **Family History**: No family history of polyneuropathy<br>**Differential Diagnoses for Polyneuropathy**<br>**1. Diabetic Polyneuropathy**<br>**Reasoning**:<br>● **Medical History**: The patient has type 2 diabetes, which is a common cause of polyneuropathy, especially with poor glycemic control.<br>● **Symptoms**: The presentation of pure sensory, symmetrical, length-dependent polyneuropathy with neuropathic pain and paresthesia in the feet is typical of diabetic polyneuropathy.<br>● **Nerve Conduction Studies**: Findings consistent with axonal type polyneuropathy, often seen in diabetes.<br>● **Lab Findings**: Increased glycemia and HbA1c indicate poor diabetes control, supporting diabetic polyneuropathy as a likely diagnosis.<br>**2. Autoimmune-Related Polyneuropathy (e.g., Chronic Inflammatory Demyelinating Polyneuropathy - CIDP)**<br>**Reasoning**:<br>● **Positive ANA Antibodies**: Suggestive of an autoimmune process, which can be associated with polyneuropathy.<br>● **Raised CRP and ESR**: Indicate inflammation, supporting an autoimmune etiology.<br>● **Clinical Features**: The absence of motor symptoms and cranial nerve involvement is less typical for CIDP, but sensory-predominant CIDP can present similarly.<br>**3. Psoriatic Arthritis-Associated Polyneuropathy**<br>**Reasoning**:

			● **Medical History**: The patient has psoriasis, which can be associated with psoriatic arthritis. Psoriatic arthritis can, in some cases, be associated with peripheral neuropathy.<br>● **Inflammatory Markers**: Raised CRP and ESR may suggest an underlying inflammatory condition, such as psoriatic arthritis.<br>● **ANA Positivity**: Although not specific, it can occur in autoimmune or inflammatory conditions like psoriatic arthritis.<br>**Most Likely Diagnosis**<br>The most likely diagnosis in this patient is **diabetic polyneuropathy**. This conclusion is based on the patient's known history of type 2 diabetes, elevated glycemia and HbA1c levels indicating poor glycemic control, and the classic presentation of sensory, symmetrical, length-dependent polyneuropathy. While other conditions such as autoimmune-related polyneuropathy and psoriatic arthritis-associated polyneuropathy are possible, the strong association with diabetes and supporting clinical and lab findings make diabetic polyneuropathy the most probable diagnosis.
62	A 39-year-old male patient presents with a 1-month history of sensory-motor, symmetrical, non-length dependent polyneuropathy with acute onset. The patient presents with weakness, hypoesthesia, paresthesia and neuropathic pain in the four limbs, cramps, weakness of orbicularis oculi muscles, stypsis and urinary incontinence. Clinical examination reveals absence of tremor, ataxia or foot deformities. Past medical history is silent. Nerve conduction studies show a symmetric, demyelinating, sensory-motor polyneuropathy. Screening lab tests including vitamin B12, folate, vitamin B6, vitamin E, HCV, HBV, ANA, ANCA, ENA, CRP, ESR, glycemia, HbA1c, copper, cryoglobulins and serum electrophoresis are all negative. There is no family history of polyneuropathy.	GBS	**Clinical Summary**<br>A 39-year-old male patient presents with:<br>● **History**: 1-month history of sensory-motor, symmetrical, non-length dependent polyneuropathy with acute onset.<br>● **Symptoms**: Weakness, hypoesthesia, paresthesia, neuropathic pain in all four limbs, cramps, weakness of orbicularis oculi muscles, stypsis (constipation), and urinary incontinence.<br>● **Clinical Examination**: No tremor, ataxia, or foot deformities.<br>● **Nerve Conduction Studies**: Symmetric, demyelinating, sensory-motor polyneuropathy.<br>● **Laboratory Tests**: Negative for vitamin B12, folate, vitamin B6, vitamin E, HCV, HBV, ANA, ANCA, ENA, CRP, ESR, glycemia, HbA1c, copper, cryoglobulins, and serum electrophoresis.<br>● **Family History**: No family history of polyneuropathy.<br>**Differential Diagnoses**<br>1. **Guillain-Barré Syndrome (GBS)**<br>○ **Reasoning**: GBS is an acute, demyelinating, symmetrical polyneuropathy that often presents with rapid onset of weakness, sensory disturbances, and areflexia. The patient's acute onset of symptoms, including motor and sensory deficits, urinary incontinence, and weakness of the orbicularis oculi muscles, are consistent with GBS. Nerve conduction studies showing demyelination further support this diagnosis. The absence of tremor, ataxia, and foot deformities also aligns with typical GBS presentations.<br>2. **Chronic Inflammatory Demyelinating Polyneuropathy (CIDP)**<br>○ **Reasoning**: CIDP is another demyelinating polyneuropathy but usually has a more chronic, relapsing-remitting or progressive course rather than an acute onset. However, there are acute variants of CIDP that can mimic GBS. The patient's symptoms, nerve conduction study results, and lack of systemic inflammation markers (negative CRP, ESR) are consistent with CIDP. The presence of autonomic symptoms (urinary incontinence and stypsis) could also fit CIDP.<br>3. **Acute Motor and Sensory Axonal Neuropathy (AMSAN)**<br>○ **Reasoning**: AMSAN is a subtype of GBS characterized by acute onset, severe motor and sensory deficits, and autonomic involvement. It differs from the classic demyelinating form of GBS but can present similarly. The rapid progression and the severe sensory-motor involvement with autonomic features in this patient might suggest AMSAN. However, the nerve conduction studies indicating demyelination would typically favor a diagnosis of GBS or CIDP over AMSAN, which usually shows axonal degeneration.<br>**Most Likely Diagnosis**<br>Based on the acute onset, symmetric, demyelinating polyneuropathy, and the combination of motor, sensory, and autonomic symptoms, **Guillain-Barré Syndrome (GBS)** is the most likely diagnosis. The nerve conduction study findings of demyelination are highly characteristic of GBS. Additionally, the rapid progression and severity of the symptoms, including autonomic involvement, fit well with the typical presentation of GBS. CIDP remains a possibility, especially if the symptoms persist or worsen over a longer period, but the acute nature of the presentation strongly supports GBS.
63	A 57-year-old female patient presents with a 1-month history of sensory-motor, symmetrical, length dependent polyneuropathy with subacute onset. The patient presents with severe sensory ataxia associated with hypoesthesia and paraesthesia in a stocking-glove distribution, as well as weakness of toe dorsiflexion. Clinical examination does not reveal cranial nerve or autonomic involvement, tremor, or foot deformities. Past medical history is positive for chronic autoimmune gastritis, chronic cough, type 2 diabetes, and arterial hypertension. Nerve conduction studies show presence of a sensory motor, length dependent, symmetric, axonal polyneuropathy. Screening lab tests reveal vitamin B12 deficiency, hyperglycaemia, elevated HbA1c, and positive ANA. Remaining exams including folate, vitamin B6, vitamin E, HBV, HCV, ANCA, ENA, CRP, ESR, copper, cryoglobulins, and serum electrophoresis are normal. There is no family history of polyneuropathy.	B12 deficiency neuropathy	**Step-by-Step Clinical Analysis**<br>**Summary of Pertinent Clinical Information**<br>● **History of Present Illness**:<br>○ 1-month history of sensory-motor, symmetrical, length-dependent polyneuropathy with subacute onset.<br>○ Severe sensory ataxia.<br>○ Hypoesthesia and paraesthesia in a stocking-glove distribution.<br>○ Weakness of toe dorsiflexion.<br>○ No cranial nerve or autonomic involvement, tremor, or foot deformities noted.<br>● **Past Medical History**:<br>○ Chronic autoimmune gastritis.<br>○ Chronic cough.<br>○ Type 2 diabetes.<br>○ Arterial hypertension.<br>● **Diagnostic Findings**:<br>○ **Nerve Conduction Studies**: Sensory motor, length-dependent, symmetric, axonal polyneuropathy.<br>○ **Laboratory Findings**:<br>■ Vitamin B12 deficiency.<br>■ Hyperglycaemia.<br>■ Elevated HbA1c.<br>■ Positive ANA.<br>■ Normal folate, vitamin B6, vitamin E, HBV, HCV, ANCA, ENA, CRP, ESR, copper, cryoglobulins, and serum electrophoresis.<br>○ **Family History**: No family history of polyneuropathy.<br>**Differential Diagnoses for Polyneuropathy**<br>**1. Diabetic Polyneuropathy**<br>**Reasoning**:<br>● **Clinical Correlation**: The patient has type 2 diabetes, hyperglycemia, and elevated HbA1c, indicating poor glycemic control.<br>● **Presentation**: Length-dependent, sensory-motor polyneuropathy with a stocking-glove distribution is characteristic of diabetic neuropathy.<br>● **Subacute Onset**: While diabetic neuropathy typically develops more gradually, subacute worsening can occur, especially in cases of poor glycemic control.<br>**2. Vitamin B12 Deficiency Neuropathy**<br>**Reasoning**:<br>● **Clinical Correlation**: Documented vitamin B12 deficiency with a history of chronic autoimmune gastritis, which is often associated with pernicious anemia and B12 deficiency.

			• **Presentation**: Sensory ataxia, hypoesthesia, and paraesthesia are common in vitamin B12 deficiency neuropathy.<br>• **Nerve Conduction Studies**: Axonal polyneuropathy can occur in severe vitamin B12 deficiency.<br>**3. Autoimmune-Related Neuropathy (Possible Sjogren's Syndrome)**<br>**Reasoning**:<br>• **Clinical Correlation**: Positive ANA, which is indicative of an underlying autoimmune disorder.<br>• **Associated Conditions**: Sjogren's syndrome and other connective tissue diseases can cause sensory-motor polyneuropathy.<br>• **Presentation**: While not as typical as the first two diagnoses, autoimmune neuropathies can present with similar symptoms.<br>**Most Likely Diagnosis**<br>**Based on the clinical information, the most likely diagnosis is Diabetic Polyneuropathy**. This conclusion is supported by the patient's history of type 2 diabetes, evidence of poor glycemic control (hyperglycemia, elevated HbA1c), and the characteristic presentation of symmetrical, length-dependent sensory-motor polyneuropathy. Diabetic polyneuropathy is common in patients with long-standing diabetes, especially when glycemic control is suboptimal, which aligns with the patient's medical history and current laboratory findings.<br>**Secondary Considerations**: Vitamin B12 deficiency neuropathy is also highly plausible given the patient's deficiency and clinical presentation. Addressing this deficiency is crucial regardless of the primary diagnosis. Autoimmune-related neuropathy remains a differential diagnosis due to the positive ANA, but it is less likely in the absence of other specific autoimmune symptoms or markers.
64	A 74-year-old male patient presents with a 1-year history of sensory-motor, symmetrical, length dependent polyneuropathy with subacute onset and slow-progressive course. The patient presents with paresthesia and neuropathic pain distally in the lower limbs and sensory ataxia. Clinical examination reveals the presence of mild hand tremor and weakness of toe dorsiflexion. There are no foot deformities, cranial nerve or autonomic involvement. Past medical history is positive for cardiac insufficiency, diabetes and IgM monoclonal gammopathy. Nerve conduction studies show a demyelinating, sensory-motor, symmetric polyneuropathy. Screening lab tests reveal the presence of IgM paraprotein and mildly elevated HbA1c levels. Remaining lab tests including vitamin B12, folate, vitamin B6, vitamin E, HCV, HBV, ANA, ANCA, ENA, CRP, ESR, glycemia, copper, and cryoglobulins are negative. There is no family history of polyneuropathy.	Anti-MAG polyneuropathy	**Summary of Pertinent Clinical Information**<br>**Patient Demographics:**<br>• **Age:** 74-year-old male<br>**Presenting Complaints:**<br>• **Duration:** 1-year history<br>• **Symptoms:** Sensory-motor, symmetrical, length-dependent polyneuropathy with subacute onset and slow-progressive course<br>• **Specific Complaints:**<br>○ Paresthesia<br>○ Neuropathic pain distally in the lower limbs<br>○ Sensory ataxia<br>**Clinical Examination Findings:**<br>• Mild hand tremor<br>• Weakness of toe dorsiflexion<br>• No foot deformities<br>• No cranial nerve involvement<br>• No autonomic involvement<br>**Past Medical History:**<br>• Cardiac insufficiency<br>• Diabetes<br>• IgM monoclonal gammopathy<br>**Investigations:**<br>• **Nerve Conduction Studies:** Demyelinating, sensory-motor, symmetric polyneuropathy<br>• **Lab Tests:**<br>○ **Positive:**<br>■ IgM paraprotein<br>■ Mildly elevated HbA1c levels<br>○ **Negative:**<br>■ Vitamin B12<br>■ Folate<br>■ Vitamin B6<br>■ Vitamin E<br>■ HCV, HBV<br>■ ANA, ANCA, ENA<br>■ CRP, ESR<br>■ Glycemia<br>■ Copper<br>■ Cryoglobulins<br>**Family History:**<br>• No family history of polyneuropathy<br>**Differential Diagnoses for Polyneuropathy**<br>1. **Diabetic Neuropathy**<br>2. **Monoclonal Gammopathy of Undetermined Significance (MGUS)-Related Neuropathy**<br>3. **Chronic Inflammatory Demyelinating Polyneuropathy (CIDP)**<br>**Differential Diagnosis Analysis**<br>**1. Diabetic Neuropathy**<br>**Reasoning:**<br>• **Clinical Features:** The patient's diabetes and elevated HbA1c suggest poorly controlled diabetes, a common cause of polyneuropathy.<br>• **Symptoms Match:** Sensory-motor neuropathy, paresthesia, neuropathic pain, and sensory ataxia are typical in diabetic neuropathy.<br>• **Type of Neuropathy:** Typically, diabetic neuropathy is length-dependent and symmetrical, aligning with this patient's presentation.<br>• **Slow-Progressive Course:** Diabetic neuropathy usually progresses slowly over time.

			**Counterpoints:**<br>● **Demyelination:** Diabetic neuropathy is typically an axonal neuropathy, whereas this patient's nerve conduction studies show demyelination, which is less typical for diabetes alone.<br>**2. Monoclonal Gammopathy of Undetermined Significance (MGUS)-Related Neuropathy**<br>**Reasoning:**<br>● **Monoclonal Protein:** The presence of IgM paraprotein suggests MGUS, which can be associated with neuropathy.<br>● **Symptoms Match:** Sensory-motor symptoms, length-dependent distribution, and sensory ataxia are common in MGUS-related neuropathy.<br>● **Nerve Conduction Studies:** Demyelinating features in nerve conduction studies are consistent with MGUS-related neuropathy.<br>**Counterpoints:**<br>● **Additional Signs:** The absence of autonomic involvement makes MGUS-related neuropathy more likely than other causes, but not definitive.<br>**3. Chronic Inflammatory Demyelinating Polyneuropathy (CIDP)**<br>**Reasoning:**<br>● **Clinical Features:** CIDP can present with subacute onset and progressive sensory-motor polyneuropathy.<br>● **Nerve Conduction Studies:** The demyelinating pattern observed is characteristic of CIDP.<br>● **Symptoms Match:** Symmetrical weakness, sensory loss, and slow progression are typical of CIDP.<br>**Counterpoints:**<br>● **IgM Paraprotein:** While not exclusive to CIDP, the presence of IgM paraprotein makes CIDP less likely unless there is an overlap syndrome.<br>● **Hand Tremor:** Hand tremor is not a classic feature of CIDP, though it can be seen.<br>**Most Likely Diagnosis**<br>**Monoclonal Gammopathy of Undetermined Significance (MGUS)-Related Neuropathy**<br>**Conclusion:** Given the patient's history of IgM monoclonal gammopathy, the demyelinating nature of the neuropathy, and the presence of IgM paraprotein, MGUS-related neuropathy is the most likely diagnosis. The clinical presentation and investigation findings align well with this diagnosis. Diabetic neuropathy and CIDP remain in the differential, but the specific features of the nerve conduction studies and the presence of IgM paraprotein strongly suggest MGUS-related neuropathy as the primary diagnosis.
65	An 83-year-old female patient presents with a 6-week history of pure sensory, symmetrical, length dependent polyneuropathy with subacute onset. The patient presents with distal paresthesia, hypoesthesia, and neuropathic pain in the lower limbs with associated mild sensory ataxia. Clinical examination does not show the presence of weakness, tremor, foot deformities, cranial nerve or autonomic involvement. Romberg test resulted positive with inability to maintain postural station, feeling of instability, and inability to walk with closed eyes. Past medical history is positive for leukopenia, undifferentiated connective tissue disease, and type 2 diabetes. Nerve conduction studies show a pure sensory, symmetric, length dependent, axonal polyneuropathy. Screening lab tests show high glycemia and raised HbA1c, positivity for ANA antibodies, and evidence of vitamin B12 deficiency. Remaining exams including folate, vitamin B6, vitamin E, HCV, HBV, ANCA, CRP, ESR, copper, cryoglobulins, and serum electrophoresis are all negative. There is no family history of polyneuropathy.	Vit B12 deficiency neuropathy	**Step-by-Step Clinical Analysis**<br>**Patient Summary**<br>● **Age:** 83-year-old female<br>● **Symptoms:**<br>○ 6-week history of pure sensory, symmetrical, length-dependent polyneuropathy<br>○ Distal paresthesia, hypoesthesia, neuropathic pain in lower limbs<br>○ Mild sensory ataxia<br>● **Clinical Examination:**<br>○ No weakness, tremor, foot deformities, cranial nerve, or autonomic involvement<br>○ Positive Romberg test: inability to maintain postural station and walk with closed eyes<br>● **Past Medical History:**<br>○ Leukopenia<br>○ Undifferentiated connective tissue disease<br>○ Type 2 diabetes<br>● **Nerve Conduction Studies:** Pure sensory, symmetric, length-dependent, axonal polyneuropathy<br>● **Laboratory Findings:**<br>○ High glycemia and raised HbA1c<br>○ Positive ANA antibodies<br>○ Vitamin B12 deficiency<br>○ Negative for folate, vitamin B6, vitamin E, HCV, HBV, ANCA, CRP, ESR, copper, cryoglobulins, and serum electrophoresis<br>● **Family History:** No family history of polyneuropathy<br>**Differential Diagnoses for Polyneuropathy**<br>1. **Diabetic Neuropathy**<br>2. **Vitamin B12 Deficiency Neuropathy**<br>3. **Paraneoplastic Neuropathy**<br>**Detailed Reasoning for Each Diagnosis**<br>1. **Diabetic Neuropathy:**<br>○ **Reasoning:**<br>■ The patient has a history of type 2 diabetes with high glycemia and elevated HbA1c levels.<br>■ Diabetic neuropathy often presents as a symmetrical, length-dependent, sensory neuropathy, fitting the patient's presentation.<br>■ The subacute onset of symptoms is consistent with diabetic neuropathy, which can worsen rapidly with poor glycemic control.<br>■ Although diabetic neuropathy can sometimes include motor involvement, pure sensory presentations are also common, particularly in older patients with longstanding diabetes.<br>2. **Vitamin B12 Deficiency Neuropathy:**<br>○ **Reasoning:**<br>■ Laboratory findings indicate a vitamin B12 deficiency, which is a known cause of sensory polyneuropathy.<br>■ B12 deficiency neuropathy is often symmetrical, affects the lower limbs first, and can present with paresthesia, hypoesthesia, and neuropathic pain, matching the patient's symptoms.<br>■ Sensory ataxia and a positive Romberg test are typical for B12 deficiency neuropathy due to posterior column involvement in the spinal cord.<br>■ The absence of motor symptoms and normal levels of other vitamins and minerals supports this diagnosis over other nutritional deficiencies.<br>3. **Paraneoplastic Neuropathy:**<br>○ **Reasoning:**

			■ Given the patient's age and recent onset of symptoms, paraneoplastic neuropathy should be considered, particularly in the context of an undifferentiated connective tissue disease, which can sometimes be associated with malignancy.<br>■ Paraneoplastic syndromes often present with subacute onset and can manifest as a pure sensory neuropathy.<br>■ Positive ANA antibodies can be seen in paraneoplastic syndromes, though they are also common in other autoimmune diseases.<br>■ However, the absence of systemic symptoms, rapid weight loss, or other signs of malignancy, along with a negative screening for other common paraneoplastic markers, makes this diagnosis less likely.<br>**Most Likely Diagnosis**<br>Given the clinical presentation and laboratory findings, the most likely diagnosis is **Vitamin B12 Deficiency Neuropathy**. The patient's symptoms, combined with the positive findings of B12 deficiency and the characteristic sensory ataxia with a positive Romberg test, align well with this condition. Furthermore, vitamin B12 deficiency neuropathy can cause sensory symptoms without motor involvement, fitting the clinical scenario presented.<br>**Conclusion**<br>● **Most Likely Diagnosis:** Vitamin B12 Deficiency Neuropathy<br>● **Next Steps:**<br>○ Initiate vitamin B12 supplementation.<br>○ Monitor for clinical improvement and re-evaluate if symptoms persist or worsen, considering the secondary differential diagnoses.<br>○ Ensure tight glycemic control to mitigate any potential contribution from diabetic neuropathy.
66	A 71-year-old female patient presents with a 4-month history of pure sensory, symmetrical, length dependent polyneuropathy with subacute onset. The patient presents with neuropathic pain and paresthesia in the feet. She does not report weakness, ataxia, tremor, cranial nerve or autonomic involvement. Clinical examination shows hyperalgesia and allodynia in the feet. There are no foot deformities, sensory ataxia or weakness. Past medical history is positive for cervical cancer, for which the patient started chemotherapy 6 months ago. Nerve conduction studies show a sensory-motor, symmetric, length dependent polyneuropathy. Screening lab tests show raised CRP and ESR, and positivity for ANA antibodies. Remaining exams including vitamin B12, folate, vitamin B6, vitamin E, HBV, HCV, ANCA, ENA, glycemia, HbA1c, copper, cryoglobulins, and serum electrophoresis are negative. There is no family history of polyneuropathy.	Chemotherapy-induced neuropathy	**Step-by-Step Analysis of Clinical Scenario**<br>**Summary of Pertinent Clinical Information**<br>● **Patient History:**<br>○ 4-month history of pure sensory, symmetrical, length-dependent polyneuropathy.<br>○ Subacute onset.<br>○ Symptoms: Neuropathic pain and paresthesia in the feet.<br>○ No reported weakness, ataxia, tremor, cranial nerve, or autonomic involvement.<br>○ Past medical history includes cervical cancer treated with chemotherapy starting 6 months ago.<br>● **Clinical Examination:**<br>○ Hyperalgesia and allodynia in the feet.<br>○ No foot deformities, sensory ataxia, or weakness.<br>● **Investigations:**<br>○ Nerve conduction studies: Sensory-motor, symmetric, length-dependent polyneuropathy.<br>○ Lab results:<br>■ Raised CRP and ESR.<br>■ Positive ANA antibodies.<br>■ Negative for vitamin B12, folate, vitamin B6, vitamin E, HBV, HCV, ANCA, ENA, glycemia, HbA1c, copper, cryoglobulins, and serum electrophoresis.<br>● **Family History:**<br>○ No family history of polyneuropathy.<br>**Differential Diagnoses for Polyneuropathy**<br>**1. Chemotherapy-Induced Peripheral Neuropathy (CIPN)**<br>**Reasoning:**<br>● The temporal relationship between the onset of polyneuropathy and chemotherapy initiation (6 months ago) is significant.<br>● Chemotherapy, particularly agents used in cervical cancer like platinum-based drugs (e.g., cisplatin), taxanes (e.g., paclitaxel), and others, are well-known causes of peripheral neuropathy.<br>● CIPN typically presents as a length-dependent, symmetrical polyneuropathy, primarily affecting sensory nerves, consistent with this patient's symptoms and clinical findings.<br>**2. Paraneoplastic Neuropathy**<br>**Reasoning:**<br>● The presence of an active malignancy (cervical cancer) raises the possibility of a paraneoplastic syndrome.<br>● Paraneoplastic neuropathies can present with subacute onset and are often associated with positive ANA and elevated inflammatory markers (CRP, ESR).<br>● While motor involvement is less common in paraneoplastic syndromes, sensory neuropathies are well-documented, aligning with this patient's clinical picture.<br>**3. Autoimmune-Related Neuropathy (e.g., Systemic Lupus Erythematosus - SLE)**<br>**Reasoning:**<br>● Positive ANA antibodies suggest an underlying autoimmune process.<br>● Raised CRP and ESR further support systemic inflammation.<br>● Autoimmune diseases like SLE can present with sensory polyneuropathy, and while not common, they must be considered given the serological findings.<br>● However, the lack of other systemic symptoms or organ involvement typically seen in SLE may argue against this diagnosis.<br>**Most Likely Diagnosis**<br>**Chemotherapy-Induced Peripheral Neuropathy (CIPN)** is the most likely diagnosis in this case. The strong temporal association with the initiation of chemotherapy for cervical cancer, the typical presentation of a sensory, length-dependent, symmetrical polyneuropathy, and the absence of other significant systemic or neurological signs point towards CIPN as the primary cause of the patient's symptoms. Paraneoplastic and autoimmune-related neuropathies remain differential considerations but are less likely given the clinical and temporal context.
67	An 83-year-old male patient presents with a 1-year history of sensory-motor, symmetrical, length dependent polyneuropathy with insidious onset and progressive course. The patient presents with distal leg paresthesia, hypoesthesia, and sensory ataxia. Clinical examination confirms the presence of sensory ataxia	Anti-MAG polyneuropathy	**Summary of Pertinent Clinical Information**<br>**Patient:** 83-year-old male<br>**Presenting Complaints:**<br>● 1-year history of sensory-motor, symmetrical, length-dependent polyneuropathy<br>● Insidious onset and progressive course<br>● Distal leg paresthesia, hypoesthesia, and sensory ataxia<br>● Postural hand tremor concurrent with other symptoms<br>**Clinical Findings:**

	with positive Romberg test and inability to walk with closed eyes. There is also postural hand tremor, which appeared concurrently with the other symptoms. Clinical examination does not show the presence of foot deformities, cranial nerve or autonomic involvement. Past medical history is positive for prostatic cancer, for which the patient underwent chemotherapy four years ago. Nerve conduction studies show a demyelinating, sensory-motor, length dependent polyneuropathy. Screening lab tests reveal the presence of IgM paraprotein and positivity for ANA antibodies. Remaining exams including vitamin B12, folate, vitamin B6, vitamin E, HCV, HBV, ANCA, ENA, CRP, ESR, glycemia, HbA1c, copper ,and cryoglobulins are negative. There is no family history of polyneuropathy.		● Positive Romberg test<br>● Inability to walk with closed eyes<br>● No foot deformities<br>● No cranial nerve involvement<br>● No autonomic involvement<br>**Medical History:**<br>● Prostatic cancer, treated with chemotherapy four years ago<br>**Investigations:**<br>● Nerve conduction studies: Demyelinating, sensory-motor, length-dependent polyneuropathy<br>● Lab tests:<br>○ Positive for IgM paraprotein<br>○ Positive for ANA antibodies<br>○ Negative for vitamin B12, folate, vitamin B6, vitamin E, HCV, HBV, ANCA, ENA, CRP, ESR, glycemia, HbA1c, copper, and cryoglobulins<br>**Family History:**<br>● No family history of polyneuropathy<br>**Differential Diagnoses**<br>**1. Monoclonal Gammopathy of Undetermined Significance (MGUS) with Associated Neuropathy**<br>**Reasoning:**<br>● The presence of IgM paraprotein is significant and strongly suggests a monoclonal gammopathy.<br>● MGUS is a common condition in older adults and can be associated with a demyelinating type of polyneuropathy.<br>● The absence of systemic symptoms (e.g., no significant weight loss, organomegaly) and negative results for other paraproteinemia-related conditions (like multiple myeloma) support MGUS.<br>**2. Chronic Inflammatory Demyelinating Polyneuropathy (CIDP)**<br>**Reasoning:**<br>● CIDP is characterized by a progressive or relapsing-remitting course of more than 8 weeks, which aligns with the patient's 1-year history of progressive symptoms.<br>● The demyelinating nature of the neuropathy observed in nerve conduction studies is consistent with CIDP.<br>● The presence of ANA antibodies, although nonspecific, suggests an autoimmune component, which is commonly associated with CIDP.<br>**3. Paraneoplastic Neuropathy**<br>**Reasoning:**<br>● The patient's history of prostatic cancer and subsequent chemotherapy raises the possibility of a paraneoplastic syndrome.<br>● Paraneoplastic neuropathies can present as demyelinating, sensory-motor polyneuropathies and are often associated with an autoimmune response.<br>● The presence of ANA antibodies and IgM paraprotein can be seen in paraneoplastic syndromes, suggesting an immune-mediated process linked to the previous malignancy.<br>**Most Likely Diagnosis**<br>**Monoclonal Gammopathy of Undetermined Significance (MGUS) with Associated Neuropathy**<br>**Justification:**<br>● The patient has IgM paraprotein, which is a hallmark of MGUS, and is a well-documented cause of neuropathy in the elderly.<br>● The demyelinating nature of the polyneuropathy is characteristic of neuropathy associated with MGUS.<br>● CIDP is a close differential; however, MGUS is more likely given the specific presence of IgM paraprotein without significant systemic autoimmune or inflammatory symptoms.<br>● The history of prostatic cancer and chemotherapy could potentially point towards a paraneoplastic neuropathy, but the time gap (four years) and lack of current malignancy signs make MGUS more probable.<br>In conclusion, the most likely diagnosis for this patient is MGUS with associated neuropathy, given the clinical presentation, nerve conduction study findings, and specific lab results.
68	A 46-year-old male patient presents with a 3-year history of sensory-motor, symmetrical, non-length dependent polyneuropathy with insidious onset. The patient presents with leg paresthesia, sensory ataxia, proximal and distal weakness in the four limbs, and cramps. There are no cranial nerve or autonomic involvement, tremor, neuropathic pain or foot deformities. Past medical history shows IgG MGUS and chronic renal insufficiency. Nerve conduction studies show demyelinating, sensory-motor polyneuropathy in the four limbs. Screening lab tests including vitamin B12, folate, vitamin B6, vitamin E, HCV, HBV, ANA, ANCA, ENA, CRP, ESR, glycemia, HbA1c, copper and cryoglobulins are all negative. Serum electrophoresis confirms the presence of IgG monoclonal gammopathy. There is no family history of polyneuropathy.	CIDP	**Summary of Pertinent Clinical Information**<br>● **Patient Profile:** 46-year-old male<br>● **History:** 3-year history of sensory-motor, symmetrical, non-length dependent polyneuropathy with insidious onset<br>● **Symptoms:**<br>○ Leg paresthesia<br>○ Sensory ataxia<br>○ Proximal and distal weakness in the four limbs<br>○ Cramps<br>● **No Symptoms of:** Cranial nerve or autonomic involvement, tremor, neuropathic pain, foot deformities<br>● **Past Medical History:**<br>○ IgG MGUS (Monoclonal Gammopathy of Undetermined Significance)<br>○ Chronic renal insufficiency<br>● **Nerve Conduction Studies:** Demyelinating, sensory-motor polyneuropathy in the four limbs<br>● **Screening Lab Tests:**<br>○ Negative for vitamin B12, folate, vitamin B6, vitamin E, HCV, HBV, ANA, ANCA, ENA, CRP, ESR, glycemia, HbA1c, copper, cryoglobulins<br>● **Serum Electrophoresis:** Confirms the presence of IgG monoclonal gammopathy<br>● **Family History:** No family history of polyneuropathy<br>**Differential Diagnoses**<br>**1. POEMS Syndrome (Polyneuropathy, Organomegaly, Endocrinopathy, Monoclonal protein, Skin changes)**<br>**Reasoning:**<br>● **Monoclonal Gammopathy:** Presence of IgG monoclonal gammopathy is a key feature.<br>● **Polyneuropathy:** The patient has a demyelinating, sensory-motor polyneuropathy which is often seen in POEMS syndrome.<br>● **Additional Features:** While the patient does not present with organomegaly, endocrinopathy, or skin changes (all common in POEMS), these may develop later or be subtle.<br>● **Exclusion of Other Causes:** Extensive lab workup has ruled out other common causes of polyneuropathy, increasing the likelihood of a rarer condition like POEMS.

			**2. Chronic Inflammatory Demyelinating Polyneuropathy (CIDP)**<br>**Reasoning:**<br>● **Polyneuropathy Type:** The demyelinating nature of the polyneuropathy is consistent with CIDP.<br>● **Symptomatology:** CIDP often presents with sensory-motor polyneuropathy, proximal and distal weakness, which the patient exhibits.<br>● **Insidious Onset:** The chronic, insidious onset over 3 years fits with CIDP.<br>● **Response to Treatment:** CIDP often responds to immunotherapy (steroids, IVIg, plasmapheresis), which can be a distinguishing factor.<br>**3. IgG-Related Paraproteinemic Neuropathy**<br>**Reasoning:**<br>● **Monoclonal Gammopathy:** Presence of IgG MGUS.<br>● **Polyneuropathy Association:** Paraproteinemic neuropathies can be associated with MGUS, particularly when the paraprotein is IgG.<br>● **Demonstrated Polyneuropathy:** The demyelinating, sensory-motor polyneuropathy fits the pattern seen in paraproteinemic neuropathy.<br>**Most Likely Diagnosis**<br>**IgG-Related Paraproteinemic Neuropathy**<br>● **Integration of Factors:** The patient's symptoms, the presence of IgG MGUS, and the demyelinating nature of the polyneuropathy make this the most likely diagnosis.<br>● **Rule Out:** While POEMS syndrome and CIDP are strong considerations, the absence of other systemic features (like organomegaly and endocrinopathy in POEMS) and the specific association with IgG MGUS lead to the conclusion of IgG-related paraproteinemic neuropathy.<br>● **Clinical Course:** The insidious onset and chronic nature without response to typical treatments for CIDP further support this diagnosis.
69	A 78-year-old female patient presents with a 2-year history of sensory-motor, symmetrical, length dependent polyneuropathy with subacute onset. Clinical examination reveals presence of numbness, tingling, and neuropathic pain distally in the upper and lower limbs, weakness of ankle dorsiflexion, sensory ataxia, hand tremor and dysphonia, without autonomic involvement or foot deformities. Past medical history reveals diabetes and IgM kappa monoclonal gammopathy. Nerve conduction studies show a length dependent, symmetric, sensory-motor, demyelinating polyneuropathy. Screening lab tests including vitamin B12, folate, vitamin B6, vitamin E, HCV, HBV, ANA, ANCA, ENA, CRP, ESR, glycemia, copper and cryoglobulins are all negative, with positivity for IgM kappa paraprotein and raised HbA1c. There is no family history of polyneuropathy.	Anti-MAG polyneuropathy	**Clinical Summary**<br>**Patient Profile:**<br>● **Age/Sex:** 78-year-old female<br>● **Duration of Symptoms:** 2 years<br>● **Symptoms:**<br>○ Sensory-motor, symmetrical, length-dependent polyneuropathy<br>○ Numbness, tingling, and neuropathic pain in upper and lower limbs<br>○ Weakness of ankle dorsiflexion<br>○ Sensory ataxia<br>○ Hand tremor<br>○ Dysphonia<br>● **Absent Symptoms:**<br>○ Autonomic involvement<br>○ Foot deformities<br>**Clinical Examination:**<br>● Distal neuropathic pain, numbness, and tingling<br>● Weakness of ankle dorsiflexion<br>● Sensory ataxia<br>● Hand tremor<br>● Dysphonia<br>**Past Medical History:**<br>● Diabetes mellitus<br>● IgM kappa monoclonal gammopathy<br>**Nerve Conduction Studies:**<br>● Length-dependent, symmetric, sensory-motor, demyelinating polyneuropathy<br>**Screening Lab Tests:**<br>● Negative for: Vitamin B12, folate, vitamin B6, vitamin E, HCV, HBV, ANA, ANCA, ENA, CRP, ESR, copper, cryoglobulins<br>● Positive for: IgM kappa paraprotein, raised HbA1c<br>**Family History:**<br>● No family history of polyneuropathy<br>**Differential Diagnoses**<br>1. **Diabetic Polyneuropathy**<br>○ **Reasoning:**<br>■ The patient has a history of diabetes and raised HbA1c levels indicating poor glycemic control, which is a well-known risk factor for diabetic polyneuropathy.<br>■ Diabetic polyneuropathy commonly presents as a symmetrical, length-dependent sensory-motor polyneuropathy with symptoms such as numbness, tingling, pain, and weakness.<br>■ Although typically axonal, some cases of diabetic neuropathy can show demyelinating features on nerve conduction studies.<br>○ **Supportive Findings:**<br>■ Presence of neuropathic symptoms in both upper and lower limbs.<br>■ Positive for diabetes with raised HbA1c.<br>2. **Monoclonal Gammopathy of Undetermined Significance (MGUS) Neuropathy**<br>○ **Reasoning:**<br>■ The patient has IgM kappa monoclonal gammopathy, which can be associated with a specific type of neuropathy known as MGUS neuropathy.<br>■ MGUS-related neuropathy often presents as a sensory-motor polyneuropathy and can show demyelinating features on nerve conduction studies.<br>■ The presence of IgM paraprotein supports this diagnosis.<br>○ **Supportive Findings:**<br>■ IgM kappa monoclonal gammopathy.

			■ Symmetric, length-dependent sensory-motor neuropathy.<br>■ Demyelinating features on nerve conduction studies.<br>3. **Chronic Inflammatory Demyelinating Polyneuropathy (CIDP)**<br>○ **Reasoning:**<br>■ CIDP is a chronic, progressive, or relapsing-remitting polyneuropathy that can present with sensory-motor symptoms, weakness, and demyelination on nerve conduction studies.<br>■ The subacute onset and progressive nature of the patient's symptoms over two years align with CIDP.<br>■ CIDP can sometimes be associated with monoclonal gammopathies, although the absence of autonomic involvement and the specific pattern of sensory ataxia, hand tremor, and dysphonia can be less common.<br>○ **Supportive Findings:**<br>■ Length-dependent, symmetric, sensory-motor, demyelinating polyneuropathy.<br>■ Chronic and progressive symptomatology.<br>**Most Likely Diagnosis**<br>**Monoclonal Gammopathy of Undetermined Significance (MGUS) Neuropathy**<br>**Reasoning:**<br>● The presence of IgM kappa monoclonal gammopathy strongly suggests MGUS-related neuropathy.<br>● The clinical features and nerve conduction study findings are consistent with MGUS neuropathy, which typically presents with demyelinating polyneuropathy.<br>● While diabetic polyneuropathy and CIDP are plausible, the specific finding of IgM paraprotein and the matching clinical picture make MGUS neuropathy the most likely diagnosis.
70	A 51-year-old female patient presents with a five-month history of sensory-motor, asymmetrical, non-length-dependent polyneuropathy with subacute onset and progressive course. The patient reports numbness and tingling in a stocking and glove distribution, dysphagia, asymmetric proximal and distal weakness in all four limbs, and mild sensory ataxia. Clinical examination confirms the presence of weakness and sensory deficits in all four limbs, with no evidence of autonomic involvement, tremor, or foot deformities. Past medical history is unremarkable. Nerve conduction studies show a demyelinating, non-length-dependent, sensory-motor polyneuropathy. Screening lab tests including vitamin B12, folate, vitamin B6, vitamin E, HBV, HCV, ANA, ANCA, ENA, CRP, ESR, glycemia, HbA1c, copper, cryoglobulins, and serum electrophoresis are all negative. There is no family history of polyneuropathy.	CIDP	**Summary of Pertinent Clinical Information**<br>● **Patient Demographics**: 51-year-old female<br>● **Symptom Duration**: 5 months<br>● **Symptoms**:<br>○ Sensory-motor polyneuropathy<br>○ Asymmetrical, non-length-dependent<br>○ Numbness and tingling in a stocking and glove distribution<br>○ Dysphagia<br>○ Asymmetric proximal and distal weakness in all four limbs<br>○ Mild sensory ataxia<br>● **Clinical Examination**:<br>○ Weakness and sensory deficits in all four limbs<br>○ No autonomic involvement<br>○ No tremor<br>○ No foot deformities<br>● **Nerve Conduction Studies**: Demyelinating, non-length-dependent, sensory-motor polyneuropathy<br>● **Laboratory Findings**:<br>○ Negative for vitamin deficiencies (B12, folate, B6, E)<br>○ Negative for HBV, HCV<br>○ Negative for ANA, ANCA, ENA<br>○ Normal CRP, ESR, glycemia, HbA1c, copper<br>○ Negative cryoglobulins and serum electrophoresis<br>● **Past Medical History**: Unremarkable<br>● **Family History**: No family history of polyneuropathy<br>**Differential Diagnoses**<br>1. **Chronic Inflammatory Demyelinating Polyneuropathy (CIDP)**<br>○ **Reasoning**: CIDP is a common cause of acquired demyelinating polyneuropathy. The patient's symptoms of progressive, asymmetrical weakness and sensory deficits over several months, along with demyelinating features on nerve conduction studies, fit well with CIDP. The absence of autonomic involvement and the presence of dysphagia and sensory ataxia further support this diagnosis. CIDP can present with a non-length-dependent pattern and affect both proximal and distal muscles, as seen in this patient.<br>2. **Multifocal Motor Neuropathy (MMN)**<br>○ **Reasoning**: MMN is another demyelinating neuropathy that presents with asymmetric, non-length-dependent weakness. Although it primarily affects motor nerves, sensory involvement can sometimes occur. The asymmetrical distribution and the demyelinating nature on nerve conduction studies are suggestive. However, the presence of significant sensory symptoms and sensory ataxia makes MMN less likely than CIDP but still possible.<br>3. **Monoclonal Gammopathy of Undetermined Significance (MGUS) with Polyneuropathy**<br>○ **Reasoning**: MGUS can cause a demyelinating neuropathy similar to CIDP. While serum electrophoresis was negative, there can be instances where MGUS is missed or small monoclonal proteins are not detected. The sensory-motor involvement and the demyelinating features on nerve conduction studies make this a plausible diagnosis. However, the absence of detected monoclonal proteins makes it less likely than CIDP.<br>**Most Likely Diagnosis**<br>The most likely diagnosis for this patient is **Chronic Inflammatory Demyelinating Polyneuropathy (CIDP)**. This conclusion is based on the combination of subacute onset, progressive course, asymmetrical sensory-motor involvement, demyelinating features on nerve conduction studies, and the exclusion of other potential causes through comprehensive laboratory testing. CIDP accounts for the patient's range of symptoms, including the non-length-dependent pattern and the involvement of both proximal and distal muscles.
71	A 69-year-old male patient presents with a 7-month history of sensory-motor, asymmetrical, non-length dependent polyneuropathy with subacute onset and progressive course. The patient presents with paresthesia and neuropathic pain in	CIDP	**Summary of Clinical Information**<br>● **Patient**: 69-year-old male<br>● **History**: 7-month history of sensory-motor, asymmetrical, non-length dependent polyneuropathy with subacute onset and progressive course.<br>● **Symptoms**:<br>○ Paresthesia and neuropathic pain in all four limbs<br>○ Weakness of ankle dorsiflexion

	the four limbs, weakness of ankle dorsiflexion, and mild urinary retention. Clinical examination does not show the presence of tremor, ataxia, foot deformities or cranial nerve involvement. Past medical history is positive for type 2 diabetes. Nerve conduction studies show a demyelinating, sensory-motor, symmetric, polyneuropathy. Screening lab tests including vitamin B12, folate, vitamin B6, vitamin E, HBV, HCV, ANA, ANCA, ENA, CRP, ESR, copper, cryoglobulins and serum electrophoresis are all negative, while glycemia and HbA1c levels are high. There is no family history of polyneuropathy.		○ Mild urinary retention<br>● **Examination Findings**:<br>○ No tremor, ataxia, foot deformities, or cranial nerve involvement<br>● **Past Medical History**: Type 2 diabetes<br>● **Nerve Conduction Studies**: Demyelinating, sensory-motor, symmetric polyneuropathy<br>● **Laboratory Findings**:<br>○ Normal: Vitamin B12, folate, vitamin B6, vitamin E, HBV, HCV, ANA, ANCA, ENA, CRP, ESR, copper, cryoglobulins, serum electrophoresis<br>○ Abnormal: High glycemia and HbA1c levels<br>● **Family History**: No family history of polyneuropathy<br>**Differential Diagnoses for Polyneuropathy**<br>1. **Diabetic Polyneuropathy**<br>○ **Reasoning**: The patient has a history of type 2 diabetes with high glycemia and HbA1c levels, which are known risk factors for diabetic neuropathy. Diabetic polyneuropathy is typically a length-dependent neuropathy, but in some cases, it can present with a non-length dependent pattern, especially with poor glycemic control. The sensory-motor and demyelinating features seen in nerve conduction studies can also be consistent with diabetic neuropathy.<br>2. **Chronic Inflammatory Demyelinating Polyneuropathy (CIDP)**<br>○ **Reasoning**: The patient's subacute onset and progressive course of symptoms, along with the demyelinating nature of the neuropathy seen on nerve conduction studies, fit the profile of CIDP. Additionally, CIDP can present with both sensory and motor involvement and may also cause urinary retention. Although CIDP is typically more symmetrical, asymmetrical forms can occur. The negative autoimmune markers do not rule out CIDP, as not all patients will have positive autoimmune markers.<br>3. **Paraneoplastic Neuropathy**<br>○ **Reasoning**: Paraneoplastic neuropathies can present with subacute onset and progressive, asymmetrical, sensory-motor involvement. Although the typical demographic might be different, the presentation is not exclusive to younger patients. The presence of urinary retention can also be seen in paraneoplastic syndromes. Screening lab tests were negative for common causes, but specific paraneoplastic panels were not mentioned. This diagnosis is less likely given the lack of other signs or known malignancy, but it remains a consideration.<br>**Most Likely Diagnosis**<br>The most likely diagnosis for this patient is **Diabetic Polyneuropathy**. The combination of a history of diabetes with high glycemia and HbA1c levels strongly points towards diabetic polyneuropathy, despite the atypical non-length dependent pattern. Diabetic neuropathy can present in varied forms and the demyelinating features can be secondary to chronic metabolic changes induced by diabetes.<br>**Conclusion**<br>Based on the clinical presentation, history, and diagnostic findings, the most probable diagnosis for this patient's polyneuropathy is **Diabetic Polyneuropathy**, supported by the significant history of diabetes and elevated blood glucose levels. Further management should include optimal glycemic control and symptomatic treatment for neuropathic pain.
72	A 58-year old male patient presents with a 1-year history of sensory-motor, symmetrical, non-length dependent polyneuropathy with subacute onset. The patient presents with paresthesia and weakness in the distal areas of the four limbs. Clinical examination does not show the presence of sensory ataxia, neuropathic pain, tremor, foot deformities, cranial nerve or autonomic involvement. Clinical examination shows cutaneous hemangiomas and hyperpigmentation. Past medical history is positive for IgA lambda monoclonal gammopathy. Nerve conduction studies show mixed demyelinating-axonal, sensory-motor, symmetric polyneuropathy. Screening lab tests including vitamin B12, folate, vitamin B6, vitamin E, HCV, HBV, ANA, ANCA, ENA, CRP, ESR, glycemia, HbA1c, copper and cryoglobulins are all negative, with positivity for IgA lambda paraprotein. There is no family history of polyneuropathy.	POEMS	**Clinical Information Summary**<br>● **Patient**: 58-year-old male<br>● **History**: 1-year history of sensory-motor, symmetrical, non-length dependent polyneuropathy with subacute onset<br>● **Symptoms**: Paresthesia and weakness in distal areas of the four limbs<br>● **Clinical Findings**:<br>○ Absence of sensory ataxia, neuropathic pain, tremor, foot deformities, cranial nerve involvement, or autonomic involvement<br>○ Presence of cutaneous hemangiomas and hyperpigmentation<br>● **Past Medical History**: Positive for IgA lambda monoclonal gammopathy<br>● **Nerve Conduction Studies**: Mixed demyelinating-axonal, sensory-motor, symmetric polyneuropathy<br>● **Screening Lab Results**: Negative for vitamin B12, folate, vitamin B6, vitamin E, HCV, HBV, ANA, ANCA, ENA, CRP, ESR, glycemia, HbA1c, copper, and cryoglobulins; Positive for IgA lambda paraprotein<br>● **Family History**: No family history of polyneuropathy<br>**Differential Diagnoses**<br>**1. Monoclonal Gammopathy of Undetermined Significance (MGUS) with Neuropathy**<br>**Reasoning**:<br>● The patient has a history of IgA lambda monoclonal gammopathy, which is often associated with MGUS.<br>● MGUS can cause polyneuropathy, particularly a mixed demyelinating-axonal type.<br>● The absence of other systemic symptoms and normal lab tests (excluding positive IgA lambda paraprotein) support MGUS as a primary consideration.<br>● Neuropathy in MGUS is often sensory-motor and can present with the described pattern of paresthesia and weakness.<br>**2. Chronic Inflammatory Demyelinating Polyneuropathy (CIDP)**<br>**Reasoning**:<br>● CIDP presents with a sensory-motor, symmetric polyneuropathy which can be both demyelinating and axonal in nature.<br>● The subacute onset and progression over a year fit the typical CIDP timeline.<br>● Although CIDP often involves cranial nerves and can include autonomic symptoms, the absence of these features does not rule out CIDP.<br>● CIDP can be associated with monoclonal gammopathies, adding another layer of potential connection to the patient's IgA lambda paraprotein.<br>**3. Multifocal Motor Neuropathy (MMN) with Conduction Block**<br>**Reasoning**:<br>● MMN typically presents with asymmetric motor neuropathy, but variants can present with symmetric involvement.<br>● The absence of sensory ataxia and neuropathic pain aligns with some presentations of MMN.<br>● The presence of demyelination on nerve conduction studies is consistent with MMN, although the typical association is with motor neuropathy rather than mixed sensory-motor.<br>● Cutaneous findings like hemangiomas and hyperpigmentation are not typical for MMN but may be incidental or secondary findings.<br>**Most Likely Diagnosis**<br>**Monoclonal Gammopathy of Undetermined Significance (MGUS) with Neuropathy** is the most likely diagnosis. The presence of IgA lambda monoclonal gammopathy, the nature of the polyneuropathy, and the negative results for other common causes of neuropathy strongly support this diagnosis. Additionally, the patient's symptoms and nerve conduction study findings are consistent with MGUS-related neuropathy. The cutaneous findings, while not directly indicative of neuropathy, do not contradict this diagnosis and may be incidental findings or related to the underlying monoclonal gammopathy.

73	An 87-year-old male patient presents with a seven-month history of sensory-motor, symmetrical, length-dependent polyneuropathy with subacute onset and progressive course. The patient reports paresthesia and hypoesthesia in the lower limbs, associated with sensory ataxia and mild weakness in ankle dorsiflexion. Clinical examination does not reveal tremor, foot deformities, cranial nerve involvement, or autonomic dysfunction. Past medical history is positive for IgG kappa monoclonal gammopathy and prostate cancer. Nerve conduction studies show a demyelinating, sensory-motor, symmetric polyneuropathy. Screening lab tests including vitamin B12, folate, vitamin B6, vitamin E, HBV, HCV, ANA, ANCA, ENA, CRP, ESR, glycemia, HbA1c, copper, and cryoglobulins are all negative, with positivity for IgG kappa paraprotein. There is no family history of polyneuropathy.	CIDP	**Summary of Pertinent Clinical Information:**<br>● **Patient:** 87-year-old male<br>● **History:**<br>○ **Duration:** Seven-month history<br>○ **Symptoms:** Sensory-motor, symmetrical, length-dependent polyneuropathy with subacute onset and progressive course<br>○ **Specific Complaints:** Paresthesia and hypoesthesia in the lower limbs, sensory ataxia, mild weakness in ankle dorsiflexion<br>○ **Examination Findings:** No tremor, foot deformities, cranial nerve involvement, or autonomic dysfunction<br>● **Past Medical History:**<br>○ IgG kappa monoclonal gammopathy<br>○ Prostate cancer<br>● **Nerve Conduction Studies:** Demyelinating, sensory-motor, symmetric polyneuropathy<br>● **Laboratory Tests:**<br>○ Negative: Vitamin B12, folate, vitamin B6, vitamin E, HBV, HCV, ANA, ANCA, ENA, CRP, ESR, glycemia, HbA1c, copper, cryoglobulins<br>○ Positive: IgG kappa paraprotein<br>● **Family History:** No family history of polyneuropathy<br>**Differential Diagnoses for Polyneuropathy:**<br>1. **Monoclonal Gammopathy of Undetermined Significance (MGUS)-associated Neuropathy:**<br>○ **Reasoning:**<br>■ Presence of IgG kappa monoclonal gammopathy.<br>■ MGUS is known to be associated with neuropathy, especially in the elderly.<br>■ The demyelinating nature of the neuropathy fits well with neuropathies associated with monoclonal gammopathies.<br>■ Exclusion of other common causes (e.g., vitamin deficiencies, infections, diabetes).<br>2. **Paraneoplastic Neuropathy:**<br>○ **Reasoning:**<br>■ History of prostate cancer could indicate a paraneoplastic syndrome.<br>■ Paraneoplastic neuropathies can present with sensory-motor polyneuropathy and are often subacute and progressive.<br>■ However, the absence of autonomic dysfunction and cranial nerve involvement makes this less likely but still possible.<br>3. **Chronic Inflammatory Demyelinating Polyneuropathy (CIDP):**<br>○ **Reasoning:**<br>■ Symmetric, demyelinating, sensory-motor polyneuropathy with a subacute and progressive course.<br>■ CIDP can occur without family history and in older adults.<br>■ Negative autoimmune markers (ANA, ANCA) do not rule out CIDP, as it is often a diagnosis of exclusion with characteristic electrophysiological findings.<br>**Most Likely Diagnosis:**<br>Given the clinical presentation, past medical history, and nerve conduction study findings, **MGUS-associated neuropathy** is the most likely diagnosis. This is supported by the presence of IgG kappa monoclonal gammopathy and the exclusion of other causes through extensive laboratory testing. The demyelinating nature of the polyneuropathy aligns with what is commonly seen in neuropathies associated with MGUS.
74	A 51-year old female patient presents with a 35-year history of chronic, sensory-motor, symmetrical, length dependent polyneuropathy with insidious onset and slow progression. The patient presents with paresthesia, tremor, hypoesthesia up to the calves, bilateral steppage, and hammertoes. Clinical examination does not reveal cranial nerve or autonomic involvement. Past medical history shows chronic renal insufficiency. Nerve conduction studies show an axonal, symmetric, sensory-motor polyneuropathy. Screening lab tests including vitamin B12, folate, vitamin B6, vitamin E, HBV, HCV, glycemia, HbA1c, ANA, ANCA, ENA, CRP, ESR, copper, cryoglobulins, and serum electrophoresis are all negative. Her father and two uncles have walking difficulties.	CMT2	**Step-by-Step Clinical Analysis**<br>**Step 1: Summarize Pertinent Clinical Information**<br>● **Patient Profile:**<br>○ Age/Gender: 51-year-old female<br>○ Chronic, sensory-motor, symmetrical, length-dependent polyneuropathy<br>○ Duration: 35 years<br>○ Onset: Insidious<br>○ Progression: Slow<br>○ Symptoms: Paresthesia, tremor, hypoesthesia up to the calves, bilateral steppage, and hammertoes<br>○ Examination: No cranial nerve or autonomic involvement<br>● **Medical History:**<br>○ Chronic renal insufficiency<br>● **Family History:**<br>○ Father and two uncles with walking difficulties<br>● **Investigations:**<br>○ Nerve Conduction Studies: Axonal, symmetric, sensory-motor polyneuropathy<br>○ Screening Lab Tests: Negative for vitamin B12, folate, vitamin B6, vitamin E, HBV, HCV, glycemia, HbA1c, ANA, ANCA, ENA, CRP, ESR, copper, cryoglobulins, and serum electrophoresis<br>**Step 2: Generate Differential Diagnoses**<br>1. **Hereditary Neuropathy (Charcot-Marie-Tooth Disease)**<br>2. **Uremic Neuropathy**<br>3. **Idiopathic Peripheral Neuropathy**<br>**Step 3: Detailed Reasoning for Each Differential Diagnosis**<br>1. **Hereditary Neuropathy (Charcot-Marie-Tooth Disease)**<br>○ **Reasoning:**<br>■ Family history of walking difficulties in father and two uncles suggests a genetic component.<br>■ Presentation is consistent with Charcot-Marie-Tooth (CMT) disease, which is a hereditary sensory and motor neuropathy.<br>■ Chronic, slowly progressive, length-dependent polyneuropathy with symptoms such as paresthesia, hypoesthesia, steppage gait, and hammertoes are classic for CMT.<br>■ Nerve conduction studies showing axonal, symmetric, sensory-motor polyneuropathy are typical for certain types of CMT, particularly axonal forms like CMT2.

			2. **Uremic Neuropathy**<br>○ **Reasoning:**<br>■ The patient has chronic renal insufficiency, a known cause of uremic neuropathy.<br>■ Uremic neuropathy typically presents as a length-dependent, symmetric sensory-motor polyneuropathy.<br>■ However, uremic neuropathy often has a more rapid progression and is usually seen in patients with severe renal failure, which would need further assessment of renal function and dialysis history.<br>3. **Idiopathic Peripheral Neuropathy**<br>○ **Reasoning:**<br>■ Negative extensive screening for other common causes of neuropathy, including metabolic, infectious, autoimmune, and nutritional deficiencies.<br>■ Idiopathic neuropathy can present with similar symptoms but lacks a definitive family history or identifiable cause.<br>■ Less likely given the strong family history of similar symptoms.<br>**Step 4: Determine the Most Likely Diagnosis**<br>**Most Likely Diagnosis:**<br>● **Hereditary Neuropathy (Charcot-Marie-Tooth Disease)**<br>○ The combination of chronic, progressive sensory-motor neuropathy with a significant family history of similar symptoms strongly suggests a hereditary cause, most likely Charcot-Marie-Tooth disease. The clinical presentation, family history, and nerve conduction study findings are all consistent with this diagnosis.<br>**Conclusion**<br>The most likely diagnosis for the patient is **Hereditary Neuropathy (Charcot-Marie-Tooth Disease)**. Further genetic testing and possibly nerve biopsy might be warranted to confirm the diagnosis and determine the specific type of CMT.
75	An 81-year-old male patient presents with a 13-month history of sensory-motor, symmetrical, non-length dependent polyneuropathy with subacute onset and progressive course. The patient presents with lumbar pain associated with paresthesia, neuropathic pain, and weakness that are distributed both proximally and distally in the four limbs. Clinical examination also reveals the presence of mild sensory ataxia. There are no tremor, cramps, foot deformities, cranial nerve or autonomic involvement. Past medical history is positive for type 2 diabetes and Hashimoto thyroiditis. Nerve conduction studies show a mixed demyelinating-axonal, sensory-motor polyneuropathy. Screening lab tests show the presence of IgG lambda monoclonal gammopathy and raised HbA1c levels. Remaining exams including vitamin B12, folate, vitamin B6, vitamin E, HCV, HBV, ANA, ANCA, ENA, CRP, ESR, copper and cryoglobulins are all negative. Lumbosacral spine x-ray shows the presence of a single osteosclerotic bone lesion. There is no family history of polyneuropathy.	POEMS	**Clinical Summary**<br>An 81-year-old male presents with a 13-month history of sensory-motor, symmetrical, non-length dependent polyneuropathy characterized by subacute onset and progressive course. Symptoms include lumbar pain, paresthesia, neuropathic pain, and weakness affecting both proximal and distal areas in all four limbs. Clinical examination reveals mild sensory ataxia, but no tremor, cramps, foot deformities, cranial nerve, or autonomic involvement. The patient has a history of type 2 diabetes and Hashimoto thyroiditis. Nerve conduction studies indicate a mixed demyelinating-axonal, sensory-motor polyneuropathy. Laboratory findings include IgG lambda monoclonal gammopathy and elevated HbA1c levels. Tests for vitamin deficiencies, viral infections, autoimmune markers, inflammation markers, and other potential causes are negative. A lumbosacral spine x-ray shows a single osteosclerotic bone lesion. There is no family history of polyneuropathy.<br>**Differential Diagnoses for Polyneuropathy**<br>1. **Monoclonal Gammopathy of Undetermined Significance (MGUS) with Associated Polyneuropathy**<br>○ **Reasoning:** The presence of IgG lambda monoclonal gammopathy is significant, as monoclonal gammopathies can be associated with neuropathy. MGUS is a common condition in elderly patients and can lead to demyelinating and axonal neuropathies. The absence of other systemic symptoms or signs of multiple myeloma (such as anemia, hypercalcemia, or renal failure) supports the diagnosis of MGUS-associated neuropathy.<br>○ **Supporting Details:**<br>■ IgG lambda monoclonal gammopathy detected in lab tests.<br>■ Mixed demyelinating-axonal neuropathy on nerve conduction studies.<br>■ Osteosclerotic lesion, although single, might be related but does not indicate multiple myeloma.<br>2. **Diabetic Polyneuropathy**<br>○ **Reasoning:** The patient has a history of type 2 diabetes with raised HbA1c levels, indicating poor glycemic control. Diabetic polyneuropathy typically presents as a length-dependent polyneuropathy, but non-length dependent variants can occur, particularly in long-standing, poorly controlled diabetes. However, the subacute onset and mixed nature of the neuropathy suggest a more complex etiology.<br>○ **Supporting Details:**<br>■ History of type 2 diabetes with elevated HbA1c.<br>■ Sensory-motor involvement consistent with diabetic neuropathy.<br>■ However, the symmetrical, non-length dependent nature is atypical.<br>3. **POEMS Syndrome**<br>○ **Reasoning:** POEMS syndrome (Polyneuropathy, Organomegaly, Endocrinopathy, Monoclonal gammopathy, and Skin changes) can present with polyneuropathy and is associated with monoclonal gammopathy. The presence of an osteosclerotic bone lesion raises suspicion for POEMS syndrome. However, the patient does not exhibit other common features like organomegaly, endocrinopathy, or skin changes, making this less likely but still possible.<br>○ **Supporting Details:**<br>■ IgG lambda monoclonal gammopathy.<br>■ Osteosclerotic bone lesion.<br>■ Sensory-motor polyneuropathy with demyelinating and axonal features.<br>**Most Likely Diagnosis**<br>Based on the clinical presentation, laboratory findings, and the nature of the neuropathy, **Monoclonal Gammopathy of Undetermined Significance (MGUS) with Associated Polyneuropathy** is the most likely diagnosis. The presence of IgG lambda monoclonal gammopathy and the absence of other systemic symptoms or multiple myeloma signs strongly support this diagnosis. While diabetic polyneuropathy is a consideration, the atypical non-length dependent presentation makes it less likely. POEMS syndrome is a possibility given the bone lesion, but the lack of other characteristic features makes it a less probable diagnosis.
76	A 51-year-old female patient presents with a 6-week history of sensory, symmetrical, length dependent polyneuropathy with acute-subacute onset. The patient presents with paresthesia and hypoesthesia with a stocking-glove distribution and sensory ataxia. Clinical examination reveals presence of severe ataxia with inability to stand without support. There is no weakness or neuropathic pain, tremor, foot deformities, cranial nerve or autonomic involvement. Past medical history is positive for type 1 diabetes since the age of 23. Nerve conduction studies	Copper deficiency neuropathy	**Step-by-Step Analysis of Clinical Scenario**<br>**Patient Summary**<br>● **Age/Sex:** 51-year-old female<br>● **History:** Type 1 diabetes mellitus since age 23<br>● **Presentation:** 6-week history of sensory, symmetrical, length-dependent polyneuropathy<br>● **Symptoms:** Paresthesia, hypoesthesia, stocking-glove distribution, sensory ataxia, severe ataxia (unable to stand without support)<br>● **Clinical Findings:**<br>○ Severe ataxia<br>○ No weakness, neuropathic pain, tremor, foot deformities<br>○ No cranial nerve or autonomic involvement<br>● **Nerve Conduction Studies:** Pure sensory, length-dependent, axonal polyneuropathy<br>● **Lab Findings:**<br>○ Hyperglycemia<br>○ Elevated HbA1c levels

	show presence of a pure sensory, length dependent, axonal polyneuropathy. Screening lab tests reveal hyperglycemia, elevated HbA1c levels and severe copper deficiency. Remaining exams including vitamin B12, folate, vitamin B6, vitamin E, HBV, HCV, ANA, ANCA, ENA, CRP, ESR, cryoglobulins, and serum electrophoresis are all negative. There is no family history of polyneuropathy.		○ Severe copper deficiency<br>○ Negative for vitamin B12, folate, vitamin B6, vitamin E, HBV, HCV, ANA, ANCA, ENA, CRP, ESR, cryoglobulins, and serum electrophoresis<br>● **Family History:** Negative for polyneuropathy<br>**Differential Diagnoses for Polyneuropathy**<br>1. **Diabetic Peripheral Neuropathy (DPN)**<br>2. **Copper Deficiency Neuropathy**<br>3. **Chronic Inflammatory Demyelinating Polyneuropathy (CIDP)**<br>**Detailed Reasoning**<br>1. **Diabetic Peripheral Neuropathy (DPN)**<br>○ **Reasoning:**<br>■ The patient has a long-standing history of type 1 diabetes with poor glycemic control, evidenced by hyperglycemia and elevated HbA1c levels.<br>■ DPN is commonly associated with sensory loss and presents in a stocking-glove distribution.<br>■ Although DPN typically involves both sensory and motor fibers, severe sensory neuropathy without motor involvement can occur, especially in cases with poor glycemic control.<br>○ **Against:**<br>■ The acute-subacute onset of symptoms over 6 weeks is less typical for DPN, which generally has a more insidious onset.<br>■ Severe ataxia without significant weakness is unusual for DPN.<br>2. **Copper Deficiency Neuropathy**<br>○ **Reasoning:**<br>■ Severe copper deficiency is noted on screening labs.<br>■ Copper deficiency can cause a sensory ataxic neuropathy due to its role in the proper functioning of the nervous system.<br>■ The pure sensory nature of the polyneuropathy and severe ataxia align well with copper deficiency neuropathy.<br>○ **Against:**<br>■ Copper deficiency neuropathy is typically associated with myelopathy, but this is not reported in the patient's clinical findings.<br>3. **Chronic Inflammatory Demyelinating Polyneuropathy (CIDP)**<br>○ **Reasoning:**<br>■ CIDP can present with a progressive course and sensory ataxia.<br>■ It typically causes a length-dependent polyneuropathy and can present without significant motor involvement initially.<br>○ **Against:**<br>■ The absence of demyelinating features on nerve conduction studies (pure axonal) makes CIDP less likely, as CIDP usually shows demyelinating features.<br>**Most Likely Diagnosis**<br>Based on the clinical presentation, laboratory findings, and nerve conduction study results, the most likely diagnosis is **Copper Deficiency Neuropathy**. The acute-subacute onset of symptoms, severe sensory ataxia, stocking-glove distribution of sensory loss, and the presence of severe copper deficiency strongly support this diagnosis. Additionally, the negative results for other common causes of polyneuropathy (e.g., vitamin deficiencies, inflammatory markers) further support copper deficiency as the primary cause.<br>**Conclusion**<br>The patient's presentation and lab findings, particularly severe copper deficiency, point towards Copper Deficiency Neuropathy as the most likely diagnosis. This condition is consistent with the sensory, symmetrical, length-dependent polyneuropathy and severe ataxia observed in this patient.
77	A 70-year-old female patient presents with a 1-month history of sensory-motor, asymmetrical, non-length dependent, step-wise multineuropathy with acute onset. The patient presents with paresthesia, hypoesthesia, neuropathic pain and weakness in a multifocal distribution. Clinical examination does not reveal presence of ataxia, tremor, foot deformities, cranial nerve or autonomic involvement. Past medical history reveals the presence of intermittent fever and malaise. Nerve conduction studies show a mixed sensory-motor, axonal multineuropathy and unilateral carpal tunnel syndrome. Screening lab tests reveal high levels of CRP and ESR. Remaining lab exams including vitamin B12, folate, vitamin B6, vitamin E, HCV, HBV, ANA, ENA, glycemia, HbA1c, copper, cryoglobulins and serum electrophoresis are normal. There is no family history of polyneuropathy.	Vasculitic neuropathy	**Summary of Pertinent Clinical Information**<br>● **Patient Demographics**: 70-year-old female<br>● **Duration of Symptoms**: 1 month<br>● **Type of Neuropathy**: Sensory-motor, asymmetrical, non-length dependent, step-wise multineuropathy with acute onset<br>● **Symptoms**:<br>○ Paresthesia<br>○ Hypoesthesia<br>○ Neuropathic pain<br>○ Weakness in a multifocal distribution<br>● **Clinical Findings**:<br>○ No ataxia<br>○ No tremor<br>○ No foot deformities<br>○ No cranial nerve involvement<br>○ No autonomic involvement<br>● **Past Medical History**: Intermittent fever and malaise<br>● **Nerve Conduction Studies**: Mixed sensory-motor, axonal multineuropathy and unilateral carpal tunnel syndrome<br>● **Lab Findings**:<br>○ Elevated CRP and ESR<br>○ Normal levels of vitamin B12, folate, vitamin B6, vitamin E, HCV, HBV, ANA, ENA, glycemia, HbA1c, copper, cryoglobulins, and serum electrophoresis<br>● **Family History**: No family history of polyneuropathy<br>**Differential Diagnoses for Polyneuropathy**<br>**1. Vasculitic Neuropathy**<br>● **Reasoning**:<br>○ **Clinical Presentation**: The step-wise, acute onset of asymmetrical, non-length dependent, sensory-motor neuropathy is highly suggestive of vasculitic neuropathy. Vasculitis often causes multifocal neuropathy due to the involvement of blood vessels supplying the peripheral nerves.<br>○ **Associated Symptoms**: The presence of intermittent fever and malaise supports a systemic inflammatory or autoimmune process.

			○ **Laboratory Findings**: Elevated CRP and ESR are markers of inflammation often seen in vasculitic conditions.<br>○ **Nerve Conduction Studies**: Axonal multineuropathy is consistent with the type of damage seen in vasculitic neuropathy.<br>**2. Paraneoplastic Neuropathy**<br>● **Reasoning**:<br>○ **Clinical Presentation**: Although less common, paraneoplastic syndromes can present with multifocal neuropathy and have a rapid onset.<br>○ **Associated Symptoms**: Systemic symptoms like fever and malaise can be part of a paraneoplastic syndrome.<br>○ **Laboratory Findings**: Elevated inflammatory markers (CRP and ESR) can be present in paraneoplastic conditions. Normal routine labs do not rule out paraneoplastic syndromes as they may require specific cancer markers or imaging for diagnosis.<br>○ **Age and Sudden Onset**: The patient's age and the acute, multifocal nature of the symptoms are consistent with paraneoplastic neuropathy.<br>**3. Infectious Neuropathy (e.g., Lyme Disease)**<br>● **Reasoning**:<br>○ **Clinical Presentation**: Acute, multifocal neuropathy can occur in infectious conditions like Lyme disease, particularly if there is an atypical presentation.<br>○ **Associated Symptoms**: Fever and malaise are common systemic symptoms in infections.<br>○ **Laboratory Findings**: Elevated CRP and ESR are nonspecific but could indicate an underlying infection.<br>○ **Nerve Conduction Studies**: The axonal nature of the neuropathy fits with some infectious causes of neuropathy.<br>○ **Normal Screening Tests**: Absence of specific screening for infections like Lyme disease, which is not covered by the given lab tests, means an infectious etiology cannot be ruled out without further specific testing.<br>**Most Likely Diagnosis**<br>Given the step-wise, multifocal distribution, acute onset, and systemic inflammatory markers, **vasculitic neuropathy** is the most likely diagnosis. This condition aligns well with the clinical presentation and nerve conduction study findings, and the systemic symptoms of fever and malaise further support this diagnosis. Additionally, the lack of family history and normal routine labs (excluding those specific for vasculitis) bolster this conclusion. Further diagnostic steps would include specific tests for vasculitis (e.g., ANCA, biopsy) to confirm the diagnosis.
78	A 48-year-old male patient presents with a 4-month history of sensory-motor, asymmetric, length dependent polyneuropathy with subacute onset and rapidly progressive course. The patient presents with paresthesia, hypoesthesia, and weakness in the distal segments of the four limbs in an asymmetric distribution. Clinical evaluation reveals peripheral edema in the lower limbs. The patient reports recent appearance of several skin growths that were diagnosed as cutaneous angiomas by the dermatologist. He also reports losing approximately 10 kg over the past 12 months. There are no tremor, ataxia, neuropathic pain, foot deformities, cranial nerve or autonomic involvement. Past medical history is positive for fibromyalgia and liver steatosis. Nerve conduction studies show a mixed demyelinating-axonal, sensory-motor, symmetric polyneuropathy. Screening lab tests including folate, vitamin B6, vitamin E, HCV, HBV, ANA, ANCA, ENA, CRP, ESR, glycemia, HbA1c, copper and cryoglobulins are negative. Serum electrophoresis shows positivity for IgA lambda paraprotein, and there is evidence of vitamin B12 deficiency. There is no family history of polyneuropathy.	POEMS	**Clinical Summary**<br>● **Patient**: 48-year-old male<br>● **History**:<br>○ 4-month history of sensory-motor, asymmetric, length-dependent polyneuropathy with subacute onset and rapidly progressive course.<br>○ Paresthesia, hypoesthesia, and weakness in distal segments of all four limbs (asymmetric distribution).<br>○ Peripheral edema in lower limbs.<br>○ Recent appearance of cutaneous angiomas.<br>○ 10 kg weight loss over 12 months.<br>● **Examination**:<br>○ No tremor, ataxia, neuropathic pain, foot deformities, cranial nerve, or autonomic involvement.<br>● **Past Medical History**:<br>○ Fibromyalgia<br>○ Liver steatosis<br>● **Investigations**:<br>○ Nerve conduction studies: Mixed demyelinating-axonal, sensory-motor, symmetric polyneuropathy.<br>○ Negative screening lab tests: Folate, vitamin B6, vitamin E, HCV, HBV, ANA, ANCA, ENA, CRP, ESR, glycemia, HbA1c, copper, cryoglobulins.<br>○ Serum electrophoresis: Positive for IgA lambda paraprotein.<br>○ Vitamin B12 deficiency.<br>● **Family History**: Negative for polyneuropathy.<br>**Differential Diagnoses for Polyneuropathy**<br>1. **Monoclonal Gammopathy of Undetermined Significance (MGUS) with associated neuropathy**<br>○ **Reasoning**: The presence of IgA lambda paraprotein in serum electrophoresis strongly suggests a monoclonal gammopathy. MGUS is a common cause of neuropathy, and the neuropathy associated with MGUS is often sensory-motor and can be mixed demyelinating-axonal. The weight loss and rapid progression could be secondary to an evolving hematological condition associated with MGUS, such as smoldering myeloma or multiple myeloma.<br>○ **Supporting Findings**: Positive IgA lambda paraprotein, mixed demyelinating-axonal neuropathy on nerve conduction studies, rapid progression, and weight loss.<br>2. **POEMS Syndrome (Polyneuropathy, Organomegaly, Endocrinopathy, Monoclonal protein, Skin changes)**<br>○ **Reasoning**: POEMS syndrome is a rare paraneoplastic disorder that is characterized by polyneuropathy, monoclonal plasma cell disorder (often lambda), and other systemic symptoms. The presence of peripheral edema, cutaneous angiomas, and weight loss are highly suggestive of POEMS syndrome.<br>○ **Supporting Findings**: Presence of IgA lambda paraprotein, cutaneous angiomas, peripheral edema, weight loss, and the constellation of systemic symptoms.<br>3. **Vitamin B12 Deficiency Neuropathy**<br>○ **Reasoning**: Vitamin B12 deficiency can lead to a sensory-motor polyneuropathy. However, it typically presents with more symmetric symptoms and often includes megaloblastic anemia and involvement of the dorsal columns, leading to ataxia and proprioceptive loss, which are not described here.<br>○ **Supporting Findings**: Identified vitamin B12 deficiency.<br>○ **Contradictory Findings**: Asymmetric distribution of neuropathy, lack of typical signs of B12 deficiency neuropathy (e.g., ataxia, megaloblastic anemia).<br>**Most Likely Diagnosis**<br>**POEMS Syndrome** is the most likely diagnosis based on the comprehensive evaluation of clinical findings, laboratory results, and the rapid progression of symptoms:<br>● The **IgA lambda paraprotein** suggests a monoclonal plasma cell disorder.<br>● The combination of **polyneuropathy, cutaneous angiomas, peripheral edema**, and **weight loss** aligns closely with the criteria for POEMS syndrome.<br>● While MGUS with neuropathy is possible, the presence of additional systemic symptoms like cutaneous angiomas and peripheral edema, typically not seen in isolated MGUS neuropathy, strongly favors POEMS syndrome.<br>● Vitamin B12 deficiency neuropathy is considered less likely due to the asymmetric nature and additional findings that point more towards a paraneoplastic process.<br>**Conclusion**<br>The constellation of findings, especially the presence of IgA lambda paraprotein, cutaneous angiomas, peripheral edema, and weight loss, strongly supports a diagnosis of **POEMS Syndrome**. Further investigations, including imaging studies and possibly a bone marrow biopsy, would be warranted to confirm this diagnosis and guide management.

79	A 55-year-old male patient presents with a 1-year history of a pure sensory, length dependent polyneuropathy with insidious onset and slow progressive course. The patient presents with paresthesia and neuropathic pain in feet. He also reports cramps. Clinical examination does not reveal ataxia, tremor, foot deformities, cranial nerve or autonomic involvement. Past medical history is positive for IgG MGUS and Hashimoto's thyroiditis. Nerve conduction studies show an axonal, sensory-motor, symmetric, length dependent polyneuropathy. Screening lab tests including vitamin B12, folate, vitamin B6, vitamin E, HBV, HCV, glycemia, HbA1c, ANA, ANCA, ENA, CRP, ESR, and copper are all negative, with positivity for IgG monoclonal gammopathy. There is no family history of polyneuropathy.	MGUS-associated neuropathy	**Summary of Pertinent Clinical Information**<br>● **Patient**: 55-year-old male<br>● **History**:<br>○ 1-year history of pure sensory, length-dependent polyneuropathy<br>○ Insidious onset and slow progressive course<br>○ Symptoms: Paresthesia, neuropathic pain in feet, cramps<br>● **Clinical Examination**:<br>○ No ataxia, tremor, foot deformities<br>○ No cranial nerve or autonomic involvement<br>● **Past Medical History**:<br>○ IgG MGUS (Monoclonal Gammopathy of Undetermined Significance)<br>○ Hashimoto's thyroiditis<br>● **Nerve Conduction Studies**:<br>○ Axonal, sensory-motor, symmetric, length-dependent polyneuropathy<br>● **Screening Lab Tests**:<br>○ Negative for vitamin B12, folate, vitamin B6, vitamin E, HBV, HCV, glycemia, HbA1c, ANA, ANCA, ENA, CRP, ESR, copper<br>○ Positive for IgG monoclonal gammopathy<br>● **Family History**: No family history of polyneuropathy<br>**Differential Diagnoses for Polyneuropathy**<br>**1. Paraproteinemic Neuropathy (MGUS-related Neuropathy)**<br>● **Reasoning**:<br>○ Presence of IgG monoclonal gammopathy (MGUS) in the patient's history.<br>○ MGUS is known to be associated with polyneuropathy, particularly presenting as a sensory and length-dependent neuropathy.<br>○ The clinical and electrophysiological findings are consistent with the typical presentation of neuropathy associated with MGUS, which can be axonal or demyelinating in nature.<br>○ Slow progressive course and sensory symptoms align well with MGUS-related neuropathy.<br>**2. Chronic Inflammatory Demyelinating Polyneuropathy (CIDP)**<br>● **Reasoning**:<br>○ Although CIDP typically presents with both motor and sensory involvement, some variants present predominantly with sensory symptoms.<br>○ Hashimoto's thyroiditis, an autoimmune condition, could predispose the patient to other autoimmune conditions like CIDP.<br>○ Length-dependent progression and chronicity could be seen in CIDP, although axonal forms are less common.<br>○ However, lack of motor weakness and normal autonomic function make this less likely compared to paraproteinemic neuropathy.<br>**3. Diabetic Polyneuropathy**<br>● **Reasoning**:<br>○ Even though screening for glycemia and HbA1c is negative, there could be an early diabetic process or a case of impaired glucose tolerance not captured by routine screening.<br>○ Diabetic neuropathy often presents as a length-dependent, sensory-dominant neuropathy.<br>○ However, given the negative screening tests and absence of typical risk factors for diabetes, this is less likely but still a consideration.<br>**Most Likely Diagnosis**<br>Based on the clinical presentation, past medical history, and diagnostic findings, the most likely diagnosis is **Paraproteinemic Neuropathy (MGUS-related Neuropathy)**. The presence of IgG MGUS in the patient, coupled with the characteristic symptoms and nerve conduction study findings, strongly supports this diagnosis. This condition aligns well with the insidious onset, slow progression, and predominantly sensory symptoms noted in the patient.
80	A 70-year-old male patient presents with a 2-year history of pure sensory, symmetrical, length dependent polyneuropathy with insidious onset. The patient presents with neuropathic pain in the lower limbs in a distal distribution. Clinical examination reveals hyperalgesia to pinprick and absence of tactile hypoesthesia. There are no foot deformities, ataxia, tremor, or cranial nerve involvement. The patient reports reduced sweating. Past medical history is negative. Nerve conduction studies show a pure sensory, symmetric, axonal polyneuropathy. Screening lab tests show raised glycemia, elevated HbA1c, and reduced vitamin B12. Remaining exams including folate, vitamin B6, vitamin E, HBV, HCV, ANA, ANCA, ENA, CRP, ESR, copper, cryoglobulins, and serum electrophoresis are all negative. There is no family history of polyneuropathy.	Diabetic neuropathy	**Summary of Clinical Information**<br>● **Patient Profile:** 70-year-old male<br>● **History:**<br>○ Duration: 2-year history<br>○ Type: Pure sensory, symmetrical, length-dependent polyneuropathy<br>○ Onset: Insidious<br>○ Symptoms: Neuropathic pain in distal lower limbs, hyperalgesia to pinprick, absence of tactile hypoesthesia, reduced sweating<br>● **Examination Findings:**<br>○ Hyperalgesia to pinprick<br>○ Absence of tactile hypoesthesia<br>○ No foot deformities, ataxia, tremor, or cranial nerve involvement<br>● **Past Medical History:** Negative<br>● **Investigations:**<br>○ Nerve conduction studies: Pure sensory, symmetric, axonal polyneuropathy<br>○ Lab Tests:<br>■ Raised glycemia<br>■ Elevated HbA1c<br>■ Reduced vitamin B12<br>■ Negative: Folate, vitamin B6, vitamin E, HBV, HCV, ANA, ANCA, ENA, CRP, ESR, copper, cryoglobulins, serum electrophoresis<br>● **Family History:** No family history of polyneuropathy<br>**Differential Diagnoses for Polyneuropathy**<br>**1. Diabetic Neuropathy**<br>**Reasoning:**<br>● **Clinical Presentation:**

			○ Polyneuropathy is common in diabetic patients, often presenting as a distal symmetrical sensory neuropathy.<br>○ Symptoms of neuropathic pain, hyperalgesia, and reduced sweating are typical in diabetic neuropathy.<br>● **Investigations:**<br>○ Raised glycemia and elevated HbA1c indicate poor glycemic control and suggest a diagnosis of diabetes mellitus.<br>○ The absence of other positive findings in extensive screening further supports diabetes as the primary etiology.<br>**2. Vitamin B12 Deficiency Neuropathy**<br>**Reasoning:**<br>● **Clinical Presentation:**<br>○ Vitamin B12 deficiency can lead to a length-dependent sensory neuropathy, which is consistent with the patient's symptoms.<br>○ Hyperalgesia and absence of tactile hypoesthesia can occur in B12 deficiency, although it more commonly presents with combined sensory and motor involvement.<br>● **Investigations:**<br>○ Reduced vitamin B12 levels provide strong evidence supporting this diagnosis.<br>○ While other vitamin deficiencies (B6, folate, vitamin E) are negative, the isolated B12 deficiency remains significant.<br>**3. Idiopathic Sensory Polyneuropathy**<br>**Reasoning:**<br>● **Clinical Presentation:**<br>○ Idiopathic sensory polyneuropathy often presents in older adults with insidious onset of sensory symptoms and no motor involvement.<br>○ This condition is considered when no specific cause is identified after thorough evaluation.<br>● **Investigations:**<br>○ Despite comprehensive testing, no other causes are definitively identified apart from elevated glucose and low B12, which could suggest idiopathic etiology if these factors are not deemed primary.<br>**Most Likely Diagnosis**<br>The most likely diagnosis for this patient is **Diabetic Neuropathy**. This conclusion is based on the following:<br>● **Clinical Consistency:** The patient's symptoms (neuropathic pain, hyperalgesia, reduced sweating) align well with common presentations of diabetic neuropathy.<br>● **Laboratory Evidence:** Elevated glycemia and HbA1c strongly indicate uncontrolled diabetes, which is a known cause of peripheral neuropathy.<br>● **Exclusion of Other Causes:** While vitamin B12 deficiency is present, the clinical picture is more consistent with diabetic neuropathy, especially given the significant findings related to glucose metabolism.<br>Therefore, based on the combination of clinical features and laboratory results, diabetic neuropathy is the most probable diagnosis for this patient.
81	A 67-year-old male patient presents with a 1-week history of pure motor, symmetrical, non-length dependent polyneuropathy with hyperacute onset. The patient presents with facial diplegia, diplopia, and weakness of the intrinsic muscles of the hands. Clinical examination reveals the presence of ophthalmoparesis affecting the right retrolateral muscle and the left superior rectus, facial diplegia, weakness of the intrinsic muscles of both hands, and mild sensory ataxia. There are no neuropathic pain, tremor, foot deformities or autonomic involvement. Past medical history is positive for type 1 diabetes, IgG MGUS, and arterial hypertension. Nerve conduction studies show normal result. Screening lab tests show hyperglycaemia and raised HbA1c levels, IgG monoclonal gammopathy, and mild increase of ESR and CRP levels. Remaining exams including vitamin B12, folate, vitamin B6, vitamin E, HBV, HCV, ANA, ENA, ANCA, copper, and cryoglobulins are normal. There is no family history of polyneuropathy.	GBS-facial diplegia variant	**Summary of Pertinent Clinical Information**<br>● **Patient Demographics:** 67-year-old male<br>● **Chief Complaint:** 1-week history of pure motor, symmetrical, non-length dependent polyneuropathy<br>● **Symptoms:**<br>○ Facial diplegia<br>○ Diplopia<br>○ Weakness of intrinsic muscles of the hands<br>● **Clinical Findings:**<br>○ Ophthalmoparesis affecting right retrolateral muscle and left superior rectus<br>○ Facial diplegia<br>○ Weakness of intrinsic muscles of both hands<br>○ Mild sensory ataxia<br>● **Absent Symptoms:** No neuropathic pain, tremor, foot deformities, or autonomic involvement<br>● **Past Medical History:**<br>○ Type 1 diabetes<br>○ IgG MGUS (Monoclonal Gammopathy of Undetermined Significance)<br>○ Arterial hypertension<br>● **Nerve Conduction Studies:** Normal<br>● **Lab Results:**<br>○ Hyperglycemia and raised HbA1c levels<br>○ IgG monoclonal gammopathy<br>○ Mild increase in ESR and CRP levels<br>○ Normal levels of vitamin B12, folate, vitamin B6, vitamin E, HBV, HCV, ANA, ENA, ANCA, copper, and cryoglobulins<br>● **Family History:** No family history of polyneuropathy<br>**Differential Diagnoses for Polyneuropathy**<br>**1. Guillain-Barré Syndrome (GBS) - Miller Fisher Variant**<br>● **Reasoning:**<br>○ **Acute Onset:** Hyperacute onset of symptoms is characteristic of GBS.<br>○ **Pure Motor Involvement:** GBS typically involves motor symptoms without sensory loss.<br>○ **Facial Diplegia and Ophthalmoparesis:** These are classic features of the Miller Fisher variant of GBS.<br>○ **Normal Nerve Conduction Studies:** Early stages of GBS can present with normal nerve conduction studies.<br>○ **Mild Sensory Ataxia:** While the Miller Fisher variant predominantly involves cranial nerves, mild sensory ataxia can also be observed.<br>○ **Absence of Pain and Autonomic Symptoms:** Consistent with GBS.<br>**2. Chronic Inflammatory Demyelinating Polyneuropathy (CIDP) - Acute Onset**<br>● **Reasoning:**<br>○ **Pure Motor Polyneuropathy:** CIDP can present predominantly with motor involvement.<br>○ **Facial Weakness and Ophthalmoparesis:** Cranial nerve involvement can occur in CIDP.

			○ **Normal Nerve Conduction Studies:** Early CIDP might show normal nerve conduction studies before demyelination is evident.<br>○ **Association with MGUS:** CIDP can be associated with monoclonal gammopathies such as IgG MGUS.<br>○ **Increased ESR and CRP:** Inflammatory markers can be elevated in CIDP.<br>**3. Diabetic Amyotrophy (Diabetic Lumbosacral Radiculoplexus Neuropathy)**<br>● **Reasoning:**<br>○ **Diabetes History:** The patient has a long-standing history of type 1 diabetes with poor glycemic control.<br>○ **Acute Onset of Weakness:** Diabetic amyotrophy can present acutely.<br>○ **Pure Motor Symptoms:** Predominantly motor symptoms without sensory involvement are seen.<br>○ **Normal Nerve Conduction Studies:** Early or mild cases may not show significant changes on nerve conduction studies.<br>○ **Hyperglycemia and Raised HbA1c Levels:** Indicative of poor diabetes control, which is a risk factor for diabetic amyotrophy.<br>**Most Likely Diagnosis**<br>Given the combination of hyperacute onset, pure motor involvement, facial diplegia, ophthalmoparesis, and normal nerve conduction studies, the most likely diagnosis is **Guillain-Barré Syndrome (GBS) - Miller Fisher Variant**. The Miller Fisher variant of GBS classically presents with ophthalmoplegia, ataxia, and areflexia, fitting the patient's presentation of ophthalmoparesis and mild sensory ataxia. The presence of normal nerve conduction studies is consistent with early stages of GBS, and the absence of neuropathic pain and autonomic symptoms aligns well with this diagnosis. Additionally, GBS can have an association with preceding infections or other immune-mediated processes, which could be reflected in the mild increase in ESR and CRP levels.
82	An 87-year-old male patient presents with a 6-month history of pure sensory, symmetrical, length dependent polyneuropathy with subacute onset. The patient presents with leg paresthesia, tremor and sensory ataxia without neuropathic pain, foot deformities, cranial nerve or autonomic involvement. Past medical history is silent. Nerve conduction studies show pure a symmetric, sensory, axonal polyneuropathy and unilateral carpal tunnel syndrome. Screening lab tests including folate, vitamin B6, vitamin E, HCV, HBV, ANA, ANCA, ENA, CRP, ESR, glycemia, HbA1c, copper, cryoglobulins and serum electrophoresis are all negative, with evidence of vitamin B12 deficiency. There is no family history of polyneuropathy.	B12-deficiency polyneuropathy	**Summary of Pertinent Clinical Information**<br>● **Patient:** 87-year-old male<br>● **History:** 6-month history of pure sensory, symmetrical, length-dependent polyneuropathy with subacute onset<br>● **Symptoms:** Leg paresthesia, tremor, sensory ataxia, no neuropathic pain, no foot deformities, no cranial nerve or autonomic involvement<br>● **Past Medical History:** Silent<br>● **Nerve Conduction Studies:** Pure sensory, symmetric, axonal polyneuropathy and unilateral carpal tunnel syndrome<br>● **Screening Lab Tests:**<br>○ **Negative:** Folate, vitamin B6, vitamin E, HCV, HBV, ANA, ANCA, ENA, CRP, ESR, glycemia, HbA1c, copper, cryoglobulins, serum electrophoresis<br>○ **Positive:** Vitamin B12 deficiency<br>● **Family History:** No history of polyneuropathy<br>**Differential Diagnoses for Polyneuropathy**<br>1. **Vitamin B12 Deficiency**<br>○ **Reasoning:** The patient has a confirmed vitamin B12 deficiency, which is a known cause of sensory neuropathy. Vitamin B12 deficiency can lead to subacute combined degeneration of the spinal cord and peripheral neuropathy. The symptoms of leg paresthesia, tremor, and sensory ataxia align well with B12 deficiency neuropathy. Additionally, the absence of foot deformities, cranial nerve, and autonomic involvement supports this diagnosis, as B12 deficiency typically presents with purely sensory symptoms in early stages.<br>2. **Idiopathic Sensory Polyneuropathy**<br>○ **Reasoning:** Given the patient's age and the negative screening for many other common causes, idiopathic sensory polyneuropathy is a plausible diagnosis. This condition is common in older adults and presents with similar symptoms of paresthesia, sensory ataxia, and tremor. The absence of neuropathic pain and autonomic involvement fits well with this diagnosis, although it is a diagnosis of exclusion.<br>3. **Paraneoplastic Neuropathy**<br>○ **Reasoning:** Although less likely given the negative screening labs, paraneoplastic syndromes can present with subacute sensory neuropathy. The age of the patient and the rapid onset of symptoms within 6 months might suggest an underlying malignancy. However, the absence of other systemic signs and the lack of family history or additional paraneoplastic markers makes this less probable but still a consideration.<br>**Most Likely Diagnosis**<br>The most likely diagnosis, based on the patient's clinical presentation and lab results, is **Vitamin B12 Deficiency**. The presence of B12 deficiency and the typical presentation of sensory ataxia and paresthesia without motor involvement or autonomic features strongly support this diagnosis. Addressing the vitamin B12 deficiency through supplementation and monitoring for clinical improvement would be the recommended course of action.
83	A 72-year-old male patient presents with a 1-year history of sensory-motor, symmetrical, non-length dependent polyneuropathy with subacute onset. The patient presents with hand weakness, paresthesia and hypoesthesia in the lower limbs, sensory ataxia, dysphagia, and pes cavus. There are no neuropathic pain or autonomic involvement. Past medical history is silent. Nerve conduction studies show a demyelinating, sensory-motor, asymmetric polyneuropathy. Screening lab tests including vitamin B12, folate, vitamin B6, vitamin E, HCV, HBV, ANA, ANCA, ENA, CRP, ESR, glycemia, HbA1c, copper, cryoglobulins and serum electrophoresis are all negative. There is no family history of polyneuropathy.	CIDP	**Summary of Pertinent Clinical Information**<br>● **Patient Demographics**: 72-year-old male<br>● **History of Present Illness**:<br>○ 1-year history of sensory-motor, symmetrical, non-length dependent polyneuropathy<br>○ Subacute onset<br>○ Hand weakness<br>○ Paresthesia and hypoesthesia in the lower limbs<br>○ Sensory ataxia<br>○ Dysphagia<br>○ Pes cavus (high arch foot)<br>● **Negative Symptoms**: No neuropathic pain, no autonomic involvement<br>● **Past Medical History**: Silent (no significant past medical history)<br>● **Nerve Conduction Studies**: Demyelinating, sensory-motor, asymmetric polyneuropathy<br>● **Laboratory Screening Tests**: Negative for vitamin B12, folate, vitamin B6, vitamin E, HCV, HBV, ANA, ANCA, ENA, CRP, ESR, glycemia, HbA1c, copper, cryoglobulins, and serum electrophoresis<br>● **Family History**: No family history of polyneuropathy<br>**Differential Diagnoses for Polyneuropathy**<br>1. **Chronic Inflammatory Demyelinating Polyneuropathy (CIDP)**<br>○ **Reasoning**: CIDP is a common cause of demyelinating polyneuropathy in older adults. The subacute onset and progressive nature of the symptoms, along with the demyelinating pattern on nerve conduction studies, are characteristic of CIDP. The absence of autonomic involvement and the presence of sensory ataxia, dysphagia, and pes cavus align with the clinical presentation of CIDP. The lack of positive lab tests also fits, as CIDP is primarily diagnosed based on clinical and electrophysiological criteria.<br>2. **Paraneoplastic Neuropathy**<br>○ **Reasoning**: Paraneoplastic syndromes can present with various types of neuropathy, including sensory-motor polyneuropathy. The patient's age increases the risk of malignancy, which can be associated with paraneoplastic neuropathy. The subacute onset, demyelinating nature, and the lack of autonomic involvement can be seen in paraneoplastic syndromes. Although initial screening tests are negative, more specific cancer screening (e.g., imaging, tumor markers) may be needed to rule out an occult malignancy.

			3. **Multifocal Motor Neuropathy (MMN)**<br>○ **Reasoning**: MMN typically presents with asymmetric, distal limb weakness, which could explain the hand weakness seen in the patient. Although MMN usually involves motor nerves, some sensory involvement can occur. The demyelinating nature on nerve conduction studies and the absence of positive screening tests could be consistent with MMN. The lack of autonomic symptoms and the asymmetric pattern are also supportive, although MMN usually presents without sensory ataxia or dysphagia.<br>**Most Likely Diagnosis**<br>Based on the detailed clinical presentation, nerve conduction study findings, and negative screening tests, the most likely diagnosis is **Chronic Inflammatory Demyelinating Polyneuropathy (CIDP)**. The combination of symmetrical sensory-motor polyneuropathy, demyelination on nerve conduction studies, absence of autonomic involvement, and characteristic features such as sensory ataxia and dysphagia strongly point towards CIDP. Further diagnostic steps would include cerebrospinal fluid analysis (looking for elevated protein), MRI of spinal roots (enhancement), and possibly a nerve biopsy to confirm the diagnosis. Early treatment with immunomodulatory therapies (e.g., corticosteroids, IVIG, or plasmapheresis) is critical to improve outcomes.
84	A 60-year-old male patient presents with a 1-month history of pure sensory, symmetrical, length dependent polyneuropathy with subacute onset. The patient presents with frequent cramps, paresthesia, and neuropathic pain in the feet. There are no sensory ataxia, tremor, foot deformities, cranial nerve or autonomic involvement. Past medical history is positive for multiple myeloma, for which the patient has undergone chemotherapy. Symptoms started after the first cycle of chemotherapy. Nerve conduction studies show a pure sensory, symmetric, axonal polyneuropathy. Screening lab tests including vitamin B12, folate, vitamin B6, vitamin E, HBV, HCV, glycemia, HbA1c, ANA, ANCA, ENA, CRP, ESR, copper, and cryoglobulins are all negative, with positivity for IgG kappa paraprotein. There is no family history of polyneuropathy.	Chemotherapy-induced neuropathy	**Summary of Pertinent Clinical Information**<br>● **Patient Demographics**: 60-year-old male<br>● **History of Present Illness**:<br>○ 1-month history of pure sensory, symmetrical, length-dependent polyneuropathy<br>○ Subacute onset with symptoms starting after the first cycle of chemotherapy<br>○ Frequent cramps, paresthesia, and neuropathic pain in the feet<br>● **Symptoms Not Present**:<br>○ No sensory ataxia, tremor, foot deformities<br>○ No cranial nerve or autonomic involvement<br>● **Past Medical History**:<br>○ Multiple myeloma, treated with chemotherapy<br>● **Diagnostic Tests**:<br>○ Nerve conduction studies: pure sensory, symmetric, axonal polyneuropathy<br>○ Screening lab tests: negative for various potential causes (vitamins, infections, metabolic disorders, autoimmune markers, copper, cryoglobulins)<br>○ Positive for IgG kappa paraprotein<br>● **Family History**: No family history of polyneuropathy<br>**Differential Diagnoses**<br>1. **Chemotherapy-induced Peripheral Neuropathy (CIPN)**<br>○ **Reasoning**:<br>■ The temporal relationship between the onset of neuropathy and the initiation of chemotherapy is highly suggestive of CIPN.<br>■ Many chemotherapeutic agents, especially those used in treating multiple myeloma (e.g., bortezomib, thalidomide, vincristine), are known to cause sensory polyneuropathy.<br>■ The clinical presentation (symmetrical, length-dependent, and sensory symptoms) aligns well with CIPN.<br>■ Nerve conduction studies showing axonal damage support this diagnosis, as many chemotherapeutic agents cause axonal degeneration.<br>2. **Monoclonal Gammopathy of Undetermined Significance (MGUS)-associated Neuropathy**<br>○ **Reasoning**:<br>■ The presence of IgG kappa paraprotein suggests a possible monoclonal gammopathy, which can be associated with neuropathy.<br>■ Patients with MGUS can develop a similar clinical presentation of symmetrical, sensory polyneuropathy.<br>■ Multiple myeloma itself can sometimes be associated with MGUS-like neuropathy, and the presence of paraprotein supports this possibility.<br>3. **Paraneoplastic Neuropathy**<br>○ **Reasoning**:<br>■ Although less common, neuropathy can be a paraneoplastic syndrome associated with multiple myeloma.<br>■ Paraneoplastic neuropathies can present with subacute onset, symmetrical, and sensory-dominant features.<br>■ The lack of other systemic signs (like autonomic or cranial nerve involvement) makes this less likely but still possible, considering the patient's cancer history.<br>**Most Likely Diagnosis**<br>Given the temporal relationship between the initiation of chemotherapy and the onset of neuropathy, as well as the clinical presentation and findings on nerve conduction studies, **Chemotherapy-induced Peripheral Neuropathy (CIPN)** is the most likely diagnosis. The absence of other causes on extensive lab testing and the typical sensory, length-dependent, and symmetrical nature of the neuropathy further support this diagnosis.<br>The positive IgG kappa paraprotein could suggest an underlying monoclonal gammopathy, but this is less likely to be the primary cause given the timing of symptom onset and the known neurotoxic potential of chemotherapeutic agents used in multiple myeloma. Therefore, CIPN remains the leading diagnosis.
85	A 63-year-old male patient presents with a 4-year history of sensory-motor, symmetrical, length dependent polyneuropathy with insidious onset and fast progression. The patient presents with severe distal motor weakness and hypoesthesia in the four limbs, dysphagia, dysphonia, and autonomic involvement with no foot deformities or tremor. Past medical history shows lung cancer (20 years ago) but he did not undergo chemotherapy. Nerve conduction studies show an axonal, sensory-motor polyneuropathy with bilateral carpal tunnel syndrome. Screening lab tests including vitamin B12, folate, vitamin B6, vitamin E,	TTR-Amyloidosis	**Step-by-Step Analysis**<br>**1. Clinical Summary**<br>**Patient Details:**<br>● **Age:** 63-year-old male<br>● **Symptom Duration:** 4 years<br>● **Symptom Characteristics:**<br>○ Sensory-motor, symmetrical, length-dependent polyneuropathy<br>○ Severe distal motor weakness<br>○ Hypoesthesia in all four limbs<br>○ Dysphagia (difficulty swallowing)<br>○ Dysphonia (voice changes)<br>○ Autonomic involvement (e.g., possibly orthostatic hypotension, bowel/bladder dysfunction)<br>● **Past Medical History:**<br>○ Lung cancer (20 years ago), no chemotherapy<br>● **Nerve Conduction Studies:**<br>○ Axonal, sensory-motor polyneuropathy

<table>
<tr><td></td><td>HBV, HCV, ANA, ANCA, ENA, glycaemia, HbA1c, CRP, ESR, copper, cryoglobulins, and serum electrophoresis are all negative. There is no family history of polyneuropathy.</td><td></td><td>
Bilateral carpal tunnel syndrome
Laboratory Screening Results: All negative/normal for:
Vitamin B12, folate, vitamin B6, vitamin E
HBV, HCV
ANA, ANCA, ENA
Glycaemia, HbA1c
CRP, ESR
Copper, cryoglobulins
Serum electrophoresis
Family History: No family history of polyneuropathy
2. Differential Diagnoses for Polyneuropathy
Most Probable Diagnoses:
Paraneoplastic Neuropathy
Chronic Inflammatory Demyelinating Polyneuropathy (CIDP)
Amyloidosis
Detailed Reasoning for Differential Diagnoses
1. Paraneoplastic Neuropathy
Reasoning:
Lung Cancer History: The patient's history of lung cancer, even 20 years ago, raises the possibility of a paraneoplastic syndrome, which can manifest as polyneuropathy.
Insidious Onset and Progression: Paraneoplastic neuropathies often have an insidious onset and progressive course.
Dysphagia and Dysphonia: These symptoms, along with autonomic involvement, can be seen in paraneoplastic syndromes.
Negative Lab Tests: Negative autoimmune, infectious, and nutritional workups align with paraneoplastic neuropathy, which is often diagnosed by excluding other causes.
No Family History: Sporadic occurrence fits the profile of paraneoplastic neuropathy.
2. Chronic Inflammatory Demyelinating Polyneuropathy (CIDP)
Reasoning:
Sensory-Motor and Length-Dependent Symptoms: CIDP typically presents with sensory and motor involvement, often symmetrically and in a length-dependent pattern.
Severe Distal Weakness: Common in CIDP.
Nerve Conduction Studies: While CIDP is usually demyelinating, there are axonal variants.
Progressive Course: Fits with the 4-year history of worsening symptoms.
Negative Lab Tests: CIDP can occur with negative routine lab tests, and diagnosis is often clinical and electrophysiological.
Absence of Foot Deformities or Tremor: Consistent with CIDP presentation.
3. Amyloidosis
Reasoning:
Autonomic Involvement: Prominent in amyloidosis-related neuropathy.
Multisystem Involvement: Dysphagia and dysphonia can be related to amyloid deposits affecting multiple organ systems.
Negative Screening Tests: Routine blood tests can often be normal in amyloidosis.
History of Malignancy: Previous malignancy, like lung cancer, can sometimes be associated with secondary amyloidosis.
Nerve Conduction Studies: Amyloid neuropathy can show axonal patterns on nerve conduction studies.
No Family History: Sporadic cases are common in secondary amyloidosis.
Most Likely Diagnosis
Paraneoplastic Neuropathy Reasoning: Given the patient's history of lung cancer, the insidious onset and progression of symptoms, the severe motor and autonomic involvement, and the exclusion of other causes by extensive laboratory testing, paraneoplastic neuropathy is the most likely diagnosis. Although the lung cancer was 20 years ago, paraneoplastic syndromes can present long after the initial malignancy, especially if there is occult malignancy or immune activation.
Conclusion: The patient's clinical presentation, history of lung cancer, and exclusion of other common causes of polyneuropathy strongly suggest paraneoplastic neuropathy as the most probable diagnosis. Further investigations, such as paraneoplastic antibody panels and possibly imaging to rule out occult malignancy, would be warranted to confirm this diagnosis.
</td></tr>
<tr><td>86</td><td>A 39-year-old female patient presents with a 34-year history of sensory-motor, symmetrical, length-dependent polyneuropathy with insidious onset and slowly progressive course. The patient reports paresthesia and neuropathic pain in the hands and feet, sensory ataxia, cramps, distal weakness in all four limbs, moderate calf atrophy, and pes cavus. Clinical examination does not reveal cranial nerve or autonomic involvement. Past medical history is unremarkable. Nerve conduction studies show an axonal, symmetric, length-dependent sensory-motor polyneuropathy. Screening lab tests including vitamin B12, folate, vitamin B6, vitamin E, HBV, HCV, glycemia, HbA1c, ANA, ANCA, ENA, CRP, ESR, copper, cryoglobulins, and serum electrophoresis</td><td>CMT2</td><td>
Summary of Pertinent Clinical Information
Patient: 39-year-old female
History:
Duration: 34-year history
Symptoms:
Sensory-motor, symmetrical, length-dependent polyneuropathy
Paresthesia and neuropathic pain in hands and feet
Sensory ataxia
Cramps
Distal weakness in all four limbs
Moderate calf atrophy
Pes cavus
Onset: Insidious, slowly progressive
Clinical Exam: No cranial nerve or autonomic involvement
Family History:
Father is wheelchair-bound
Brother and sister have walking difficulties
Past Medical History: Unremarkable
Investigations:
</td></tr>
</table>

	are all negative. Her father is wheelchair-bound, and her brother and sister have walking difficulties.		● **Nerve Conduction Studies:** Axonal, symmetric, length-dependent sensory-motor polyneuropathy<br>● **Lab Tests:**<br>○ Vitamin B12, folate, vitamin B6, vitamin E: Negative<br>○ HBV, HCV: Negative<br>○ Glycemia, HbA1c: Negative<br>○ ANA, ANCA, ENA, CRP, ESR: Negative<br>○ Copper, cryoglobulins, serum electrophoresis: Negative<br>**Differential Diagnoses for Polyneuropathy**<br>1. **Hereditary Sensory and Motor Neuropathy (HSMN), particularly Charcot-Marie-Tooth Disease (CMT)**<br>○ **Reasoning:**<br>■ **Family History:** Multiple family members (father, brother, sister) affected suggests a hereditary condition.<br>■ **Pes Cavus:** Common in CMT due to muscle imbalances.<br>■ **Clinical Presentation:** Symmetrical, length-dependent polyneuropathy with a slowly progressive course fits the typical presentation of CMT.<br>■ **Nerve Conduction Studies:** Axonal pattern can be seen in some types of CMT, particularly CMT2 (axonal type).<br>■ **Genetic Inheritance:** The presence of symptoms in multiple family members suggests an autosomal dominant inheritance pattern, common in CMT.<br>2. **Hereditary Neuropathy with Liability to Pressure Palsies (HNPP)**<br>○ **Reasoning:**<br>■ **Family History:** Similar to CMT, HNPP is also inherited and could present with a family history of neuropathic symptoms.<br>■ **Clinical Presentation:** HNPP can present with recurrent, pressure-induced neuropathy but might also present with a chronic, slowly progressive neuropathy in some cases.<br>■ **Nerve Conduction Studies:** Usually show focal demyelination, but there can be secondary axonal loss, especially in longstanding disease.<br>3. **Chronic Inflammatory Demyelinating Polyneuropathy (CIDP) with Axonal Features**<br>○ **Reasoning:**<br>■ **Clinical Presentation:** CIDP typically presents with progressive polyneuropathy, though usually with more prominent motor involvement.<br>■ **Nerve Conduction Studies:** Predominantly demyelinating, but axonal involvement can occur.<br>■ **Response to Treatment:** CIDP often responds to immunomodulatory treatments (steroids, IVIG), which can help distinguish it from purely hereditary neuropathies.<br>**Most Likely Diagnosis**<br>**Hereditary Sensory and Motor Neuropathy (HSMN), particularly Charcot-Marie-Tooth Disease (CMT)**<br>**Reasoning:**<br>● The patient's clinical presentation (symmetrical, length-dependent polyneuropathy with an insidious onset and slowly progressive course), along with the presence of pes cavus and moderate calf atrophy, strongly suggests CMT.<br>● The significant family history of similar symptoms (father, brother, sister) supports a hereditary condition.<br>● Nerve conduction studies showing an axonal pattern align with CMT type 2.<br>● Other hereditary neuropathies, such as HNPP, are less likely given the absence of pressure palsies or recurrent focal neuropathies.<br>● CIDP is less likely due to the lack of cranial nerve involvement, lack of response to immunomodulatory treatments (not mentioned), and the predominant axonal pattern without significant demyelination.<br>Further genetic testing for CMT (e.g., PMP22 duplication/deletion for CMT1A, GJB1 gene for CMTX, or MFN2 gene for CMT2A) would be useful to confirm the diagnosis.
87	A 50-year-old male patient presents with a 1-month history of pure motor multineuropathy with acute onset while hospitalized in ICU for sepsis. Clinical examination reveals the presence of dysphagia, dysphonia, hand tremor, and upper limb weakness in a multifocal distribution. There are no sensory deficits, nor neuropathic pain, tremor or foot deformities. Past medical history is positive for testicular cancer, for which the patient underwent chemotherapy three years ago. Nerve conduction studies show pure motor, axonal multineuropathy with one motor conduction block. Screening lab tests including vitamin B12, folate, vitamin B6, vitamin E, HCV, HBV, ANA, ANCA, ENA, CRP, ESR, glycemia, HbA1c, copper, cryoglobulins and serum electrophoresis are all negative. There is no family history of polyneuropathy.	GBS	**Summary of Clinical Information**<br>**Patient Profile:**<br>● **Age:** 50-year-old male<br>● **Chief Complaint:** 1-month history of pure motor multineuropathy<br>● **Onset:** Acute onset during ICU stay for sepsis<br>**Symptoms:**<br>● Dysphagia (difficulty swallowing)<br>● Dysphonia (difficulty speaking)<br>● Hand tremor<br>● Upper limb weakness in a multifocal distribution<br>**Examination Findings:**<br>● No sensory deficits<br>● No neuropathic pain<br>● No foot deformities<br>**Past Medical History:**<br>● Testicular cancer treated with chemotherapy three years ago<br>**Diagnostic Findings:**<br>● Nerve conduction studies: Pure motor, axonal multineuropathy with one motor conduction block<br>● Screening lab tests: All negative (including vitamin B12, folate, vitamin B6, vitamin E, HCV, HBV, ANA, ANCA, ENA, CRP, ESR, glycemia, HbA1c, copper, cryoglobulins, and serum electrophoresis)<br>● No family history of polyneuropathy<br>**Differential Diagnoses for Polyneuropathy**<br>1. **Paraneoplastic Syndrome:**<br>○ **Reasoning:** The patient's history of testicular cancer and chemotherapy raises the possibility of a paraneoplastic syndrome. Paraneoplastic syndromes can manifest with various neurological symptoms, including motor neuropathies. Although typically associated with sensory or sensorimotor neuropathies, purely motor forms are possible. The acute onset of symptoms and the multifocal distribution of weakness support this diagnosis.<br>2. **Critical Illness Polyneuropathy (CIP):**<br>○ **Reasoning:** The patient's acute onset of symptoms while in the ICU for sepsis is highly suggestive of critical illness polyneuropathy. CIP commonly occurs in patients who have been critically ill, particularly those with sepsis. The condition often presents with predominantly motor symptoms, which aligns with this patient's presentation of pure motor neuropathy, absence of sensory deficits, and the acute nature of onset during critical illness.<br>3. **Chemotherapy-Induced Neurotoxicity:**

			○ **Reasoning:** The patient's history of chemotherapy for testicular cancer can lead to chemotherapy-induced neurotoxicity. Although more commonly associated with sensory neuropathies, motor neuropathy can also occur, particularly with certain chemotherapeutic agents like cisplatin. The temporal gap (three years) since chemotherapy might make this less likely but not impossible, especially if there were residual subclinical effects exacerbated by recent critical illness.<br>**Most Likely Diagnosis**<br>**Critical Illness Polyneuropathy (CIP):** Given the acute onset of pure motor neuropathy during an ICU stay for sepsis, combined with the absence of sensory deficits and other systemic causes ruled out by extensive laboratory testing, critical illness polyneuropathy is the most likely diagnosis. CIP is a well-documented complication of severe sepsis and critical illness, characterized by an acute onset of predominantly motor symptoms. The patient's clinical presentation and history align closely with this condition.<br>**Rationale:**<br>● **Temporal Association:** The neuropathy developed acutely during a critical illness, a hallmark of CIP.<br>● **Symptomatology:** Pure motor involvement without sensory deficits fits CIP, which often presents with similar features.<br>● **Exclusion of Other Causes:** Extensive negative lab workup and absence of family history further support CIP as the primary diagnosis.
88	A 58-year-old male patient presents with a 15-month history of sensory-motor, symmetrical, length-dependent polyneuropathy with insidious onset and progressive course. The patient reports distal paresthesia, numbness, neuropathic pain in the lower limbs in a symmetric distribution, and sensory ataxia. Clinical examination confirms the presence of sensory ataxia, postural hand tremor, and mild weakness in ankle dorsiflexion. There are no foot deformities, cranial nerve involvement, or autonomic dysfunction. Past medical history is positive for IgM kappa MGUS and type 2 diabetes. Nerve conduction studies show a demyelinating, length-dependent, symmetric, sensory-motor polyneuropathy. Screening lab tests including vitamin B12, folate, vitamin B6, vitamin E, HCV, HBV, ANA, ANCA, ENA, CRP, ESR, glycemia, copper, and cryoglobulins are all negative, with evidence of IgM kappa paraprotein and elevated HbA1c levels. There is no family history of polyneuropathy.	Anti-MAG polyneuropathy	**Clinical Summary:**<br>● **Patient Profile**: 58-year-old male<br>● **Duration**: 15-month history<br>● **Symptoms**:<br>○ Sensory-motor, symmetrical, length-dependent polyneuropathy<br>○ Distal paresthesia<br>○ Numbness<br>○ Neuropathic pain in lower limbs (symmetric distribution)<br>○ Sensory ataxia<br>● **Clinical Findings**:<br>○ Sensory ataxia<br>○ Postural hand tremor<br>○ Mild weakness in ankle dorsiflexion<br>● **Exclusions**:<br>○ No foot deformities<br>○ No cranial nerve involvement<br>○ No autonomic dysfunction<br>● **Medical History**:<br>○ IgM kappa MGUS<br>○ Type 2 diabetes<br>● **Diagnostic Studies**:<br>○ Nerve conduction studies: demyelinating, length-dependent, symmetric, sensory-motor polyneuropathy<br>● **Laboratory Tests**:<br>○ Vitamin B12, folate, vitamin B6, vitamin E: Negative<br>○ HCV, HBV: Negative<br>○ ANA, ANCA, ENA, CRP, ESR: Negative<br>○ Glycemia: Elevated HbA1c<br>○ Copper, cryoglobulins: Negative<br>○ IgM kappa paraprotein present<br>**Differential Diagnoses for Polyneuropathy:**<br>1. **Chronic Inflammatory Demyelinating Polyneuropathy (CIDP)**:<br>○ **Reasoning**:<br>■ CIDP is a common cause of acquired demyelinating polyneuropathy.<br>■ It presents with symmetrical sensory and motor deficits, which can progress insidiously.<br>■ Nerve conduction studies showing demyelinating features support this diagnosis.<br>■ CIDP often presents with sensory ataxia and distal weakness.<br>■ However, the presence of IgM paraprotein and diabetes makes this diagnosis less likely without further evidence, but not impossible.<br>2. **Diabetic Neuropathy**:<br>○ **Reasoning**:<br>■ Diabetes is a common cause of polyneuropathy, particularly in patients with elevated HbA1c levels, indicating poor glycemic control.<br>■ Symmetrical, length-dependent sensory-motor polyneuropathy fits the clinical presentation of diabetic neuropathy.<br>■ Diabetic neuropathy can lead to sensory ataxia and distal motor weakness.<br>■ The absence of foot deformities and autonomic dysfunction does not rule out diabetic neuropathy but makes it less typical.<br>3. **Paraproteinemic Neuropathy (related to IgM kappa MGUS)**:<br>○ **Reasoning**:<br>■ Monoclonal gammopathy of undetermined significance (MGUS) is associated with peripheral neuropathy, particularly when IgM paraprotein is present.<br>■ IgM paraproteins can be associated with demyelinating neuropathies, which aligns with nerve conduction study findings.<br>■ The insidious onset, progressive nature, and demyelinating features support this diagnosis.<br>■ Presence of IgM kappa paraprotein in the context of MGUS is highly suggestive.<br>**Most Likely Diagnosis:**<br>**Paraproteinemic Neuropathy (IgM kappa MGUS-associated)**:<br>● **Reasoning**:

			○ The presence of IgM kappa paraprotein along with the clinical presentation and nerve conduction study findings strongly suggest paraproteinemic neuropathy.<br>○ Although diabetic neuropathy and CIDP are plausible, the specificity of the IgM kappa paraprotein in a patient with MGUS makes this the most likely diagnosis.<br>○ The combination of demyelinating features, symmetric distribution, and associated paraprotein supports this conclusion.<br>Further investigations such as a detailed electrophysiological study and possible biopsy, if indicated, could confirm this diagnosis. Additionally, monitoring for progression or transformation of MGUS to a more significant hematologic disorder would be warranted.
89	A 73-year-old male patient presents with a 2-year history of sensory-motor, symmetrical, non-length dependent polyneuropathy with subacute onset. The patient presents with paresthesia and neuropathic pain in a stocking and glove distribution, proximal and distal weakness in the lower limbs, mild sensory ataxia and cramps. There are no foot deformities, tremor, cranial nerve or autonomic involvement. Clinical examination reveals cutaneous hyperpigmentation. Past medical history is positive for IgG lambda monoclonal gammopathy. Nerve conduction studies show a mixed demyelinating-axonal, sensory-motor, symmetric polyneuropathy. Screening lab tests including vitamin B12, folate, vitamin B6, vitamin E, HCV, HBV, ANA, ANCA, ENA, CRP, ESR, glycemia, HbA1c, copper and cryoglobulins are all negative, with positivity for IgG lambda paraprotein. There is no family history of polyneuropathy.	POEMS	**Summary of Pertinent Clinical Information:**<br>● **Patient Demographics:**<br>○ 73-year-old male<br>● **History of Present Illness:**<br>○ 2-year history of sensory-motor, symmetrical, non-length dependent polyneuropathy<br>○ Subacute onset of symptoms<br>○ Paresthesia and neuropathic pain in a stocking and glove distribution<br>○ Proximal and distal weakness in the lower limbs<br>○ Mild sensory ataxia and cramps<br>● **Clinical Examination Findings:**<br>○ Cutaneous hyperpigmentation<br>○ No foot deformities, tremor, cranial nerve, or autonomic involvement<br>● **Past Medical History:**<br>○ IgG lambda monoclonal gammopathy<br>● **Diagnostic Test Results:**<br>○ Nerve conduction studies: mixed demyelinating-axonal, sensory-motor, symmetric polyneuropathy<br>○ Screening labs:<br>■ Negative: vitamin B12, folate, vitamin B6, vitamin E, HCV, HBV, ANA, ANCA, ENA, CRP, ESR, glycemia, HbA1c, copper, and cryoglobulins<br>■ Positive: IgG lambda paraprotein<br>○ No family history of polyneuropathy<br>**Differential Diagnoses:**<br>1. **Monoclonal Gammopathy of Undetermined Significance (MGUS) - Related Neuropathy:**<br>○ **Reasoning:**<br>■ The presence of IgG lambda monoclonal gammopathy is a significant finding in this patient.<br>■ MGUS can lead to a polyneuropathy that is typically symmetrical and can present with both demyelinating and axonal features, consistent with this patient's nerve conduction studies.<br>■ Cutaneous hyperpigmentation is sometimes observed in association with paraproteinemia.<br>■ The subacute onset and mixed type (demyelinating and axonal) of polyneuropathy are typical of MGUS-related neuropathy.<br>■ No family history of polyneuropathy also aligns with an acquired rather than hereditary condition.<br>2. **Chronic Inflammatory Demyelinating Polyneuropathy (CIDP):**<br>○ **Reasoning:**<br>■ CIDP typically presents with a progressive, symmetrical sensory-motor polyneuropathy.<br>■ The mixed demyelinating-axonal nature seen in nerve conduction studies is consistent with CIDP.<br>■ The absence of cranial nerve involvement and the presence of proximal and distal weakness are typical of CIDP.<br>■ However, CIDP often has a more chronic progressive course rather than a subacute onset, making it slightly less likely in this context.<br>■ The presence of a monoclonal gammopathy can sometimes be seen in CIDP, although it is less common.<br>3. **Multifocal Acquired Demyelinating Sensory and Motor Neuropathy (MADSAM, or Lewis-Sumner Syndrome):**<br>○ **Reasoning:**<br>■ MADSAM is a variant of CIDP characterized by asymmetrical and multifocal involvement, but it can occasionally present with more symmetric features.<br>■ The demyelinating-axonal nature and sensory-motor involvement are consistent with MADSAM.<br>■ The subacute onset fits with MADSAM, as it can have an acute to subacute presentation.<br>■ The absence of cranial nerve and autonomic involvement also aligns with MADSAM.<br>**Most Likely Diagnosis:**<br>Given the patient's clinical presentation, past medical history, and diagnostic findings, the most likely diagnosis is **Monoclonal Gammopathy of Undetermined Significance (MGUS) - Related Neuropathy**.<br>● The presence of IgG lambda monoclonal gammopathy is a key indicator.<br>● The clinical presentation of symmetrical, sensory-motor polyneuropathy with mixed demyelinating and axonal features aligns well with MGUS-related neuropathy.<br>● Other conditions like CIDP and MADSAM are considered, but the specific association with IgG lambda monoclonal gammopathy and the absence of other significant findings (such as cranial nerve or autonomic involvement) support MGUS-related neuropathy as the most probable diagnosis.<br>**Conclusion:**<br>The thorough analysis of the patient's history, clinical examination, and diagnostic tests points towards MGUS-related neuropathy as the most likely diagnosis. Further evaluation, including a possible bone marrow biopsy and monitoring of the monoclonal protein levels, might be necessary for confirming the diagnosis and guiding treatment.
90	A 64-year-old male patient presents with a predominantly motor, asymmetrical, non-length dependent polyneuropathy with acute onset that has progressed in the past three months. The patient also reports the presence of neuropathic pain and postural hand tremor. Clinical examination does not	Acute onset CIDP	**Summary of Pertinent Clinical Information**<br>● **Patient Demographics**: 64-year-old male<br>● **Presenting Symptoms**:<br>○ Predominantly motor polyneuropathy<br>○ Asymmetrical and non-length dependent<br>○ Acute onset with progression over three months<br>○ Neuropathic pain

	reveal ataxia, foot deformities, cranial nerve or autonomic involvement. Past medical history is positive for diabetes. Nerve conduction studies show demyelinating, pure motor polyneuropathy with conduction blocks. Screening lab tests including vitamin B12, folate, vitamin B6, vitamin E, HBV, HCV, ANA, ANCA, ENA, CRP, ESR, glycemia, HbA1c, copper, and cryoglobulins are all negative. Serum electrophoresis reveals the presence of IgM monoclonal gammopathy. There is no family history of polyneuropathy.		○ Postural hand tremor<br>● **Clinical Examination**:<br>○ No ataxia<br>○ No foot deformities<br>○ No cranial nerve involvement<br>○ No autonomic involvement<br>● **Medical History**: Diabetes<br>● **Nerve Conduction Studies**: Demyelinating, pure motor polyneuropathy with conduction blocks<br>● **Screening Lab Tests**: Negative for vitamin B12, folate, vitamin B6, vitamin E, HBV, HCV, ANA, ANCA, ENA, CRP, ESR, glycemia, HbA1c, copper, and cryoglobulins<br>● **Serum Electrophoresis**: Presence of IgM monoclonal gammopathy<br>● **Family History**: No family history of polyneuropathy<br>**Differential Diagnoses for Polyneuropathy**<br>1. **Chronic Inflammatory Demyelinating Polyneuropathy (CIDP)**<br>2. **Paraproteinemic Neuropathy**<br>3. **Multifocal Motor Neuropathy (MMN)**<br>**Detailed Reasoning for Each Proposed Diagnosis**<br>**1. Chronic Inflammatory Demyelinating Polyneuropathy (CIDP)**<br>● **Reasoning**: CIDP is a chronic, progressive, or relapsing demyelinating polyneuropathy characterized by motor and sensory deficits. The demyelinating nature and conduction blocks observed in nerve conduction studies are indicative of CIDP. Although CIDP typically presents with more symmetrical symptoms, there are variants that can present asymmetrically and predominantly motor.<br>● **Clinical Correlation**:<br>○ Demyelinating polyneuropathy with conduction blocks is typical of CIDP.<br>○ CIDP can present without ataxia or cranial nerve involvement.<br>○ Acute onset and progression over three months can be seen in rapidly progressive CIDP forms.<br>**2. Paraproteinemic Neuropathy**<br>● **Reasoning**: The presence of IgM monoclonal gammopathy in serum electrophoresis suggests a paraproteinemic neuropathy. This category includes neuropathies associated with monoclonal gammopathies, such as MGUS (Monoclonal Gammopathy of Undetermined Significance), Waldenström's macroglobulinemia, and multiple myeloma. These neuropathies often present with demyelinating features and can be either symmetric or asymmetric.<br>● **Clinical Correlation**:<br>○ IgM monoclonal gammopathy is a strong indicator of a paraproteinemic neuropathy.<br>○ Demyelination and conduction blocks are consistent with this diagnosis.<br>○ Neuropathic pain and tremor can be associated with paraproteinemic neuropathies.<br>**3. Multifocal Motor Neuropathy (MMN)**<br>● **Reasoning**: MMN is a purely motor neuropathy characterized by asymmetric weakness and conduction blocks on nerve conduction studies, similar to the findings in this patient. MMN typically does not involve sensory deficits or autonomic dysfunction and can present with hand tremors.<br>● **Clinical Correlation**:<br>○ Predominantly motor, asymmetrical neuropathy with conduction blocks aligns with MMN.<br>○ The presence of tremor and absence of ataxia, cranial nerve, and autonomic involvement also supports MMN.<br>**Most Likely Diagnosis**<br>The most likely diagnosis for this patient is **Paraproteinemic Neuropathy**.<br>● **Key Factors**:<br>○ The presence of IgM monoclonal gammopathy is a significant finding that directly points towards a paraproteinemic neuropathy.<br>○ Demyelinating features and conduction blocks are consistent with this type of neuropathy.<br>○ Although CIDP and MMN are possible, the IgM monoclonal gammopathy strongly suggests an underlying paraproteinemic process that is driving the neuropathy.<br>To confirm this diagnosis, further evaluation including potential biopsy, detailed hematologic workup, and possibly cerebrospinal fluid analysis could be considered. Treatment would likely involve addressing the underlying monoclonal gammopathy and symptomatic management of the neuropathy.
91	A 78-year-old male patient presents with a 1-year history of a sensory-motor, symmetrical, length dependent polyneuropathy with insidious onset and progressive course. The patient presents with sensory ataxia, hand tremor, weakness of ankle dorsiflexion, and distal leg paraesthesia. He also reports mild neuropathic pain in the feet. Clinical examination does not reveal foot deformities, autonomic or cranial nerve involvement. Past medical history is positive for type 2 diabetes, IgM kappa monoclonal gammopathy, and Waldenström macroglobulinemia. Nerve conduction studies show a demyelinating, sensory-motor, length dependent polyneuropathy. Screening lab tests shows hyperglycaemia, elevated HbA1c, and IgM	Anti-MAG neuropathy	**Clinical Summary**<br>**Patient:** 78-year-old male<br>**Presenting Complaint:** 1-year history of sensory-motor, symmetrical, length-dependent polyneuropathy with insidious onset and progressive course.<br>**Symptoms:**<br>● Sensory ataxia<br>● Hand tremor<br>● Weakness of ankle dorsiflexion<br>● Distal leg paraesthesia<br>● Mild neuropathic pain in the feet<br>**Clinical Examination:**<br>● No foot deformities<br>● No autonomic or cranial nerve involvement<br>**Past Medical History:**<br>● Type 2 diabetes<br>● IgM kappa monoclonal gammopathy<br>● Waldenström macroglobulinemia<br>**Nerve Conduction Studies:**<br>● Demyelinating, sensory-motor, length-dependent polyneuropathy<br>**Lab Findings:**

	monoclonal gammopathy. Remaining exams including vitamin B12, folate, vitamin B6, vitamin E, HCV, ANA, ENA, ANCA, ENA, CRP, copper, and cryoglobulins are negative. There is no family history of polyneuropathy.		● Hyperglycaemia<br>● Elevated HbA1c<br>● IgM monoclonal gammopathy<br>● Negative for vitamin B12, folate, vitamin B6, vitamin E, HCV, ANA, ENA, ANCA, ENA, CRP, copper, and cryoglobulins<br>**Family History:** No family history of polyneuropathy<br>**Differential Diagnoses for Polyneuropathy**<br>**1. Diabetic Polyneuropathy**<br>**Reasoning:**<br>● **History of Type 2 Diabetes:** The patient has a known history of type 2 diabetes, which is a common cause of polyneuropathy.<br>● **Hyperglycaemia and Elevated HbA1c:** These lab findings suggest poor glycemic control, which is a risk factor for diabetic neuropathy.<br>● **Clinical Presentation:** The insidious onset, progressive course, and length-dependent nature of the neuropathy align with typical diabetic polyneuropathy features.<br>● **Sensory-Motor Involvement:** Both sensory and motor fibers are affected, consistent with diabetic polyneuropathy.<br>**2. Monoclonal Gammopathy of Undetermined Significance (MGUS) Neuropathy**<br>**Reasoning:**<br>● **IgM Monoclonal Gammopathy:** The presence of IgM monoclonal gammopathy suggests MGUS, which can be associated with neuropathy.<br>● **Waldenström Macroglobulinemia:** This condition can cause neuropathy due to the deposition of abnormal proteins affecting nerve function.<br>● **Demyelinating Features:** Nerve conduction studies showing demyelinating neuropathy are consistent with MGUS-related neuropathy.<br>● **Lack of Other Causes:** Negative screening for other common causes of neuropathy (vitamin deficiencies, infections, autoimmune markers) supports this diagnosis.<br>**3. Chronic Inflammatory Demyelinating Polyneuropathy (CIDP)**<br>**Reasoning:**<br>● **Demyelinating Polyneuropathy:** Nerve conduction studies show demyelination, which is a hallmark of CIDP.<br>● **Progressive Course:** The progressive nature of symptoms over a year fits the chronic course of CIDP.<br>● **Sensory-Motor Involvement:** CIDP often presents with both sensory and motor involvement.<br>● **Exclusion of Other Causes:** The patient's negative lab results for other conditions (e.g., vitamin deficiencies, infections) support considering CIDP, although no specific autoimmune markers are noted in this case.<br>**Most Likely Diagnosis**<br>**Diabetic Polyneuropathy**<br>**Reasoning:**<br>● The patient's long-standing history of type 2 diabetes, along with hyperglycaemia and elevated HbA1c levels, strongly indicate diabetic polyneuropathy.<br>● The clinical presentation of a sensory-motor, length-dependent neuropathy with sensory ataxia and distal paraesthesia aligns well with diabetic polyneuropathy.<br>● While MGUS neuropathy and CIDP are also possible, the strong correlation between diabetes and polyneuropathy, combined with the patient's poor glycemic control, makes diabetic polyneuropathy the most likely diagnosis.<br>**Conclusion**<br>Based on the patient's clinical presentation, past medical history, and lab findings, diabetic polyneuropathy is the most probable diagnosis. The presence of poorly controlled diabetes, along with the typical pattern of neuropathy, strongly supports this conclusion. MGUS neuropathy and CIDP are less likely but remain considerations in the differential diagnosis.
92	A 58-year-old female patient presents with a 3-month history of sensory-motor, symmetrical, non-length dependent polyneuropathy with acute onset and progressive course. Two weeks after an infectious event, the patient reports the appearance of paraesthesia and weakness in the hands and lower limbs. At nadir she was unable to walk. The symptoms were interpreted as Guillain Barré syndrome and the patient received a course of immunoglobulin therapy with clear benefit. After the therapy she started walking again with some difficulty. About three months after the onset of symptoms, she noticed the reappearance of the paresthesia and a worsening of her strength, so she started using a crutch. Neurological examination shows the presence of weakness, distributed proximally and distally in the lower limbs and distally in the upper limbs, and sensory ataxia. Past medical history is silent. Nerve conduction studies show a demyelinating, sensory-motor polyneuropathy. Screening lab tests including vitamin B12, folate, vitamin B6, vitamin E, HBV, HCV, ANA, ANCA, ENA, CRP, ESR, glycemia, HbA1c, copper, cryoglobulins, and serum electrophoresis	Acute onset CIDP	**Clinical Summary**<br>**Patient:** 58-year-old female<br>**Presenting Symptoms:**<br>● 3-month history of sensory-motor, symmetrical, non-length dependent polyneuropathy<br>● Acute onset with progressive course<br>● Symptoms began two weeks post-infection<br>● Paraesthesia and weakness in hands and lower limbs<br>● At nadir, inability to walk<br>● Initial diagnosis: Guillain Barré syndrome (GBS)<br>● Received immunoglobulin therapy with improvement (able to walk with difficulty)<br>**Current Symptoms (3 months post-onset):**<br>● Reappearance of paresthesia<br>● Worsening of strength, requiring a crutch for walking<br>● Neurological exam: Weakness (proximal and distal in lower limbs, distal in upper limbs), sensory ataxia<br>**Past Medical History:** Silent<br>**Investigations:**<br>● Nerve conduction studies: Demyelinating, sensory-motor polyneuropathy<br>● Screening lab tests: Negative for vitamin B12, folate, vitamin B6, vitamin E, HBV, HCV, ANA, ANCA, ENA, CRP, ESR, glycemia, HbA1c, copper, cryoglobulins, serum electrophoresis<br>● No family history of polyneuropathy<br>**Differential Diagnoses**<br>1. **Chronic Inflammatory Demyelinating Polyneuropathy (CIDP)**<br>○ **Reasoning:**<br>■ CIDP is a chronic disorder characterized by progressive or relapsing-remitting course of demyelinating polyneuropathy.<br>■ The patient's symptoms began acutely and have had a relapsing course, which aligns with CIDP.<br>■ Initial response to immunoglobulin therapy supports this diagnosis, as CIDP often responds to immunomodulatory treatment.<br>■ The recurrence of symptoms after initial improvement is typical in CIDP.<br>■ Sensory-motor involvement with proximal and distal weakness, as well as sensory ataxia, are consistent with CIDP.<br>2. **Multifocal Acquired Demyelinating Sensory and Motor Neuropathy (MADSAM) or Lewis-Sumner Syndrome**<br>○ **Reasoning:**<br>■ MADSAM is a variant of CIDP that presents with multifocal sensory and motor deficits.

	are negative. There is no family history of polyneuropathy.		■ The patient's presentation of both upper and lower limb involvement could fit this diagnosis.<br>■ The demyelinating nature of the neuropathy and the response to immunoglobulin therapy are also supportive.<br>■ However, MADSAM typically has more asymmetric presentation, which is not the case here.<br>■ The progressive and relapsing course is still in line with this condition.<br>3. **Paraneoplastic Neuropathy**<br>○ **Reasoning:**<br>■ Paraneoplastic neuropathies can present as a sensory-motor polyneuropathy and may have a demyelinating nature.<br>■ These neuropathies can have an acute or subacute onset and may follow a relapsing course.<br>■ The absence of a clear family history and the negative initial screening tests do not rule out an underlying malignancy.<br>■ However, the initial benefit from immunoglobulin therapy and the specific relapsing nature after improvement is less characteristic of paraneoplastic processes.<br>**Most Likely Diagnosis**<br>**Chronic Inflammatory Demyelinating Polyneuropathy (CIDP)**<br>● **Reasoning:** The combination of an acute onset, progressive and relapsing course, initial improvement with immunoglobulin therapy, and the demyelinating nature of the neuropathy strongly suggest CIDP. CIDP can present with both proximal and distal weakness, sensory ataxia, and may relapse after initial treatment. The other differential diagnoses are less consistent with the overall clinical picture and response to treatment.
93	A 42-year-old female patient presents with a 6-month history of chronic, sensory-motor, symmetrical, non-length dependent polyneuropathy with subacute onset. The patient presents with paresthesia and severe neuropathic pain in the four limbs in a stocking and glove distribution and sensory ataxia. Clinical examination reveals the presence of postural hand tremor, which the patient reports being present for years. Thereare no foot deformities, cranial nerve or autonomic involvement. Past medical history is positive for rectal cancer, for which the patient has undergone chemotherapy for three years. Neuropathy symptoms started during chemotherapy. Nerve conduction studies show sensory-motor, symmetric, axonal polyneuropathy. Screening lab tests including vitamin B6, vitamin E, HBV, HCV, ANA, ANCA, ENA, CRP, ESR, glycemia, HbA1c, copper, cryoglobulins and serum electrophoresis are all negative, with evidence of vitamin B12 deficiency. There is no family history of polyneuropathy.	Chemotherapy-induced neuropathy	**Summary of Pertinent Clinical Information**<br>● **Patient Details:** 42-year-old female<br>● **Symptom Duration:** 6 months<br>● **Symptom Characteristics:** Chronic, sensory-motor, symmetrical, non-length dependent polyneuropathy with subacute onset<br>● **Symptom Presentation:** Paresthesia and severe neuropathic pain in a stocking and glove distribution, sensory ataxia<br>● **Physical Examination:** Postural hand tremor (present for years), no foot deformities, no cranial nerve or autonomic involvement<br>● **Medical History:** Rectal cancer, undergone chemotherapy for three years, neuropathy symptoms started during chemotherapy<br>● **Nerve Conduction Studies:** Sensory-motor, symmetric, axonal polyneuropathy<br>● **Lab Results:** Negative for various markers (HBV, HCV, ANA, ANCA, ENA, CRP, ESR, glycemia, HbA1c, copper, cryoglobulins, serum electrophoresis), positive for vitamin B12 deficiency<br>● **Family History:** No family history of polyneuropathy<br>**Differential Diagnoses for Polyneuropathy**<br>1. **Chemotherapy-Induced Peripheral Neuropathy (CIPN)**<br>○ **Reasoning:**<br>■ **Temporal Association:** Symptoms started during chemotherapy, which is a common cause of polyneuropathy.<br>■ **Type of Polyneuropathy:** Symmetrical, sensory-motor, axonal polyneuropathy fits the typical presentation of CIPN.<br>■ **Chemotherapy Agents:** Many chemotherapeutic agents, such as platinum-based compounds, taxanes, and vinca alkaloids, are known to cause neuropathy.<br>■ **Exclusion of Other Causes:** Extensive negative screening tests for other common causes of polyneuropathy make CIPN a strong contender.<br>2. **Vitamin B12 Deficiency**<br>○ **Reasoning:**<br>■ **Confirmed Deficiency:** Lab results indicate a vitamin B12 deficiency, which can cause neuropathy.<br>■ **Symptoms Correlation:** Sensory ataxia, paresthesia, and neuropathic pain can all result from vitamin B12 deficiency.<br>■ **Axonal Polyneuropathy:** Vitamin B12 deficiency can lead to axonal damage in peripheral nerves.<br>■ **Pre-existing Hand Tremor:** Although not directly related, it does not rule out B12 deficiency as a contributing factor.<br>3. **Paraneoplastic Neuropathy**<br>○ **Reasoning:**<br>■ **History of Cancer:** The patient's history of rectal cancer raises the possibility of a paraneoplastic syndrome.<br>■ **Subacute Onset:** Paraneoplastic neuropathy often has a subacute onset, similar to this patient's presentation.<br>■ **Negative Screening:** Although screening tests are negative, paraneoplastic neuropathies can sometimes be seronegative.<br>■ **Neuropathy Pattern:** Symmetrical, sensory-motor, axonal polyneuropathy can be consistent with paraneoplastic syndromes.<br>**Most Likely Diagnosis**<br>Based on the information provided, the most likely diagnosis is **Chemotherapy-Induced Peripheral Neuropathy (CIPN)**. This conclusion is supported by the strong temporal association between the onset of symptoms and the chemotherapy treatment for rectal cancer. Additionally, the symmetrical, sensory-motor, axonal nature of the polyneuropathy aligns well with the typical presentation of CIPN. The presence of vitamin B12 deficiency is noted but is less likely the primary cause given the clear correlation with chemotherapy timing and the typical presentation of CIPN. Paraneoplastic neuropathy remains a consideration but is less likely due to the lack of supporting laboratory evidence and the strong alternative explanation provided by the chemotherapy history.
94	A 73-year-old female patient presents with a 1-year history of pure sensory, symmetrical, length dependent polyneuropathy with insidious onset. The patient presents with paresthesia, neuropathic pain and sensory ataxia without weakness, cranial nerve or autonomic involvement. There are also no foot deformities or tremor. Past medical history is positive for sarcoidosis. Nerve conduction studies show pure a pure sensory, axonal polyneuropathy. Screening lab tests including vitamin B12, folate, vitamin B6, vitamin E, HCV, HBV, ANCA, ENA, CRP, ESR, glycemia, HbA1c, copper,	Sarcoidosis associated polyneuropathy	**Step-by-Step Analysis of the Clinical Scenario**<br>**Summary of Pertinent Clinical Information**<br>● **Patient Profile:**<br>○ **Age:** 73-year-old<br>○ **Sex:** Female<br>● **Presenting Symptoms:**<br>○ **Duration:** 1 year<br>○ **Type:** Pure sensory, symmetrical, length-dependent polyneuropathy<br>○ **Onset:** Insidious<br>○ **Symptoms:** Paresthesia, neuropathic pain, sensory ataxia<br>○ **Absent Symptoms:** Weakness, cranial nerve involvement, autonomic involvement, foot deformities, tremor<br>● **Past Medical History:**<br>○ Sarcoidosis<br>● **Investigations:**<br>○ **Nerve Conduction Studies:** Pure sensory, axonal polyneuropathy

	cryoglobulins and serum electrophoresis are all negative, with positivity for ANA autoantibodies. There is no family history of polyneuropathy.		○ **Screening Lab Tests:**<br>■ **Negative Results:** Vitamin B12, folate, vitamin B6, vitamin E, HCV, HBV, ANCA, ENA, CRP, ESR, glycemia, HbA1c, copper, cryoglobulins, serum electrophoresis<br>■ **Positive Result:** ANA autoantibodies<br>● **Family History:** No family history of polyneuropathy<br>**Differential Diagnoses for Polyneuropathy**<br>**1. Sarcoidosis-Related Neuropathy**<br>**Reasoning:**<br>● **Sarcoidosis Background:** Sarcoidosis can involve peripheral nerves, leading to neuropathy. Though it often causes a mixed sensory and motor neuropathy, it can occasionally present with a purely sensory form.<br>● **Evidence from Case:** The patient has a known history of sarcoidosis, making sarcoidosis-related neuropathy a strong consideration. The chronic nature of sarcoidosis aligns with the insidious onset of the neuropathy.<br>● **Nerve Conduction Findings:** The pure sensory, axonal nature of the neuropathy can occur in sarcoidosis.<br>**2. ANA-Associated Autoimmune Neuropathy**<br>**Reasoning:**<br>● **Autoimmune Association:** The presence of ANA autoantibodies suggests an underlying autoimmune process.<br>● **Typical Presentation:** Autoimmune neuropathies can present as pure sensory neuropathies and can be symmetrical and length-dependent.<br>● **Supporting Evidence:** The patient's positive ANA result, absence of other common causes of neuropathy, and the autoimmune context provided by sarcoidosis make an ANA-associated autoimmune neuropathy a plausible diagnosis.<br>**3. Idiopathic Sensory Neuropathy**<br>**Reasoning:**<br>● **Common in Elderly:** In the absence of a clear secondary cause, idiopathic sensory neuropathy is a common diagnosis in older adults.<br>● **Typical Presentation:** It often presents as a symmetrical, length-dependent sensory neuropathy, similar to this patient's presentation.<br>● **Lack of Other Causes:** Extensive negative workup for other common causes (e.g., vitamin deficiencies, diabetes, infections, and other autoimmune markers) makes idiopathic etiology a consideration.<br>**Most Likely Diagnosis**<br>**Most Likely Diagnosis: ANA-Associated Autoimmune Neuropathy**<br>**Detailed Reasoning:**<br>● **Autoimmune Link:** The patient's positive ANA, in the context of a history of sarcoidosis (an autoimmune disease), strongly suggests an autoimmune basis for the neuropathy.<br>● **Clinical Presentation:** The pure sensory, symmetrical, length-dependent nature of the neuropathy, along with the absence of motor, cranial nerve, or autonomic involvement, fits well with an autoimmune sensory neuropathy.<br>● **Exclusion of Other Causes:** The comprehensive negative screening for other common etiologies (nutritional deficiencies, infections, metabolic causes) supports an autoimmune process as the underlying cause.<br>**Conclusion**<br>Based on the clinical presentation, past medical history, and investigative findings, the most likely diagnosis for this patient is **ANA-associated autoimmune neuropathy**. This conclusion is drawn considering the patient's positive ANA autoantibodies and history of sarcoidosis, an autoimmune condition, coupled with the characteristic presentation and exclusion of other common causes of polyneuropathy.
95	A 62-year-old male patient presents with a 5-month history of sensory-motor, symmetrical, length dependent polyneuropathy with subacute onset. The patient presents with distal paresthesia and neuropathic pain in the lower limbs, associated with weakness of toe dorsiflexion. Clinical examination also reveals the presence of orthostatic hypotension. There are no sensory ataxia, tremor, foot deformities or cranial nerve involvement. Past medical history is positive for diabetes since the age of 50, and plasmacytoma, for which the patient has undergone chemotherapy. Symptoms started during treatment with chemotherapy. Nerve conduction studies show a mixed sensory-motor, symmetric, axonal polyneuropathy. Screening lab tests including vitamin B12, folate, vitamin B6, vitamin E, HBV, HCV, ANA, ANCA, ENA, CRP, ESR, copper, cryoglobulins and serum electrophoresis are all negative, with evidence of hyperglycaemia and elevated HbA1c. There is no family history of polyneuropathy.	Chemotherapy-induced neuropathy	**Step-by-Step Clinical Analysis of the Case:**<br>**Summary of Pertinent Clinical Information:**<br>● **Patient Demographics:**<br>○ 62-year-old male<br>● **History of Present Illness:**<br>○ 5-month history of sensory-motor, symmetrical, length-dependent polyneuropathy with subacute onset<br>○ Symptoms include distal paresthesia and neuropathic pain in the lower limbs<br>○ Weakness in toe dorsiflexion<br>○ Presence of orthostatic hypotension<br>● **Clinical Examination Findings:**<br>○ No sensory ataxia, tremor, foot deformities, or cranial nerve involvement<br>● **Past Medical History:**<br>○ Diabetes mellitus since the age of 50<br>○ Plasmacytoma treated with chemotherapy<br>● **Onset of Symptoms:**<br>○ During chemotherapy treatment<br>● **Nerve Conduction Studies:**<br>○ Mixed sensory-motor, symmetric, axonal polyneuropathy<br>● **Screening Lab Tests:**<br>○ Negative for vitamin B12, folate, vitamin B6, vitamin E, HBV, HCV, ANA, ANCA, ENA, CRP, ESR, copper, cryoglobulins, and serum electrophoresis<br>○ Evidence of hyperglycaemia and elevated HbA1c<br>● **Family History:**<br>○ No family history of polyneuropathy<br>**Differential Diagnoses for Polyneuropathy:**<br>1. **Diabetic Polyneuropathy:**<br>○ **Reasoning:**<br>■ The patient has a long history of diabetes (12 years), which is a well-known risk factor for developing polyneuropathy.<br>■ Elevated HbA1c and hyperglycaemia support poorly controlled diabetes, which can exacerbate neuropathic symptoms.<br>■ Diabetic neuropathy typically presents as a symmetrical, length-dependent polyneuropathy, matching the patient's presentation.<br>■ The presence of orthostatic hypotension can be associated with diabetic autonomic neuropathy.<br>2. **Chemotherapy-Induced Polyneuropathy:**<br>○ **Reasoning:**<br>■ Symptoms started during chemotherapy treatment for plasmacytoma, suggesting a temporal relationship.<br>■ Many chemotherapeutic agents, such as vinca alkaloids, platinum compounds, and taxanes, are known to cause peripheral neuropathy.

			■ The pattern of sensory-motor and axonal involvement is consistent with chemotherapy-induced neuropathy.<br>■ Orthostatic hypotension can also be seen in the context of autonomic involvement in chemotherapy-induced neuropathy.<br>3. **Paraneoplastic Neuropathy:**<br>○ **Reasoning:**<br>■ The patient has a history of plasmacytoma, which can be associated with paraneoplastic syndromes, including neuropathies.<br>■ Although less common, paraneoplastic neuropathies can present with similar clinical features, including sensory-motor involvement and autonomic dysfunction.<br>■ The temporal association with cancer and treatment may raise suspicion, although no specific paraneoplastic markers were noted in the lab tests.<br>**Most Likely Diagnosis:**<br>Based on the detailed clinical information and the differential diagnoses discussed, the most likely diagnosis is **Chemotherapy-Induced Polyneuropathy**. This conclusion is drawn from the close temporal relationship between the onset of neuropathy symptoms and the chemotherapy treatment, the typical pattern of sensory-motor and axonal involvement, and the absence of other lab markers pointing towards an alternative cause. Diabetic polyneuropathy is also a strong contender given the patient's history and uncontrolled diabetes, but the acute onset during chemotherapy makes the chemotherapy-induced etiology more compelling in this scenario.<br>The diagnosis should be confirmed and managed in close collaboration with the patient's oncologist, considering the potential need for adjusting chemotherapy regimens and addressing neuropathic pain and autonomic symptoms.
96	An 86-year-old male patient presents with a 1-year history of sensory-motor, symmetrical, non-length dependent polyneuropathy with subacute onset and progressive course. The patient presents with distal paresthesia in the upper limbs and sensory ataxia with weakness of ankle dorsiflexion. Clinical examination reveals mild hand tremor. There are no neuropathic pain, foot deformities, cranial nerve or autonomic involvement. Past medical history shows IgG kappa monoclonal gammopathy and basal cell skin cancer. Nerve conduction studies show a demyelinating, sensory-motor polyneuropathy. Screening lab tests including vitamin B12, folate, vitamin B6, vitamin E, HCV, ANA, ANCA, ENA, CRP, ESR, glycemia, HbA1c, copper and cryoglobulins are all negative, with evidence of chronic HBV infection and positivity for IgG kappa paraprotein. There is no family history of polyneuropathy.	CIDP	**Summary of Pertinent Clinical Information**<br>**Patient Demographics:**<br>● Age: 86 years<br>● Gender: Male<br>**Presenting Symptoms:**<br>● Duration: 1 year<br>● Type: Sensory-motor, symmetrical, non-length dependent polyneuropathy<br>● Onset: Subacute<br>● Course: Progressive<br>● Specific symptoms:<br>○ Distal paresthesia in the upper limbs<br>○ Sensory ataxia<br>○ Weakness of ankle dorsiflexion<br>○ Mild hand tremor<br>● Absent symptoms:<br>○ Neuropathic pain<br>○ Foot deformities<br>○ Cranial nerve involvement<br>○ Autonomic involvement<br>**Past Medical History:**<br>● IgG kappa monoclonal gammopathy<br>● Basal cell skin cancer<br>**Investigations:**<br>● Nerve conduction studies: Demyelinating, sensory-motor polyneuropathy<br>● Lab tests:<br>○ Negative for vitamin B12, folate, vitamin B6, vitamin E, HCV, ANA, ANCA, ENA, CRP, ESR, glycemia, HbA1c, copper, cryoglobulins<br>○ Positive for chronic HBV infection and IgG kappa paraprotein<br>**Family History:**<br>● No family history of polyneuropathy<br>**Differential Diagnoses for Polyneuropathy**<br>1. **Monoclonal Gammopathy of Undetermined Significance (MGUS) Associated Neuropathy**<br>○ **Reasoning:**<br>■ The patient has an IgG kappa monoclonal gammopathy, which can be associated with MGUS.<br>■ MGUS is a known cause of demyelinating neuropathy.<br>■ The absence of systemic symptoms and the chronic, progressive nature of the neuropathy align with MGUS-associated neuropathy.<br>■ Negative screening for other common causes of polyneuropathy supports this diagnosis.<br>2. **Chronic Inflammatory Demyelinating Polyneuropathy (CIDP)**<br>○ **Reasoning:**<br>■ CIDP typically presents with a chronic, progressive course similar to this patient's presentation.<br>■ The demyelinating nature of the neuropathy on nerve conduction studies is characteristic of CIDP.<br>■ Although CIDP often has a more relapsing-remitting course, a chronic progressive form is possible.<br>■ The lack of significant autonomic involvement and the symmetrical nature of the symptoms are consistent with CIDP.<br>3. **Paraneoplastic Neuropathy**<br>○ **Reasoning:**<br>■ Although the patient's history of basal cell carcinoma is not typically associated with paraneoplastic syndromes, the presence of a monoclonal gammopathy raises suspicion.<br>■ Paraneoplastic neuropathy can present as a sensory-motor polyneuropathy with demyelinating features.<br>■ This diagnosis is less likely given the negative systemic inflammatory markers and the specific cancer history, but it remains a consideration due to the monoclonal gammopathy.<br>**Most Likely Diagnosis**<br>The most likely diagnosis for this patient is **Monoclonal Gammopathy of Undetermined Significance (MGUS) Associated Neuropathy**. This is based on the presence of IgG kappa monoclonal gammopathy, the demyelinating nature of the neuropathy, and the exclusion of other common causes through comprehensive screening. MGUS-associated neuropathy fits well with the clinical presentation, particularly the chronic, progressive course and the symmetrical, non-length dependent distribution of symptoms.

97	A 57-year-old male patient presents with a 1-year history of sensory-motor, asymmetric, non-length dependent polyneuropathy with subacute onset and progressive course. The patient presents with weakness and paresthesia in the proximal and distal areas of the upper limbs in an asymmetric distribution. Clinical examination excludes the presence of ataxia, tremor, neuropathic pain, foot deformities, cranial nerve or autonomic involvement. Past medical history is silent. Nerve conduction studies show a demyelinating, sensory-motor multineuropathy. Screening lab tests reveal the presence of folate deficiency. Remaining exams including vitamin B12, vitamin B6, vitamin E, HBV, HCV, ANA, ANCA, ENA, CRP, ESR, glycemia, HbA1c, copper, cryoglobulins and serum electrophoresis are all negative. There is no family history of polyneuropathy.	CIDP	**Summary of Pertinent Clinical Information**<br>**Patient Demographics:**<br>● 57-year-old male<br>**Presenting Symptoms:**<br>● 1-year history of sensory-motor, asymmetric, non-length dependent polyneuropathy<br>● Subacute onset and progressive course<br>● Weakness and paresthesia in proximal and distal areas of the upper limbs with an asymmetric distribution<br>**Clinical Examination Findings:**<br>● No ataxia, tremor, neuropathic pain, foot deformities, cranial nerve, or autonomic involvement<br>**Past Medical History:**<br>● Silent (no significant past medical issues)<br>**Investigations:**<br>● Nerve conduction studies: Demyelinating, sensory-motor multineuropathy<br>● Screening lab tests:<br>○ Positive: Folate deficiency<br>○ Negative: Vitamin B12, Vitamin B6, Vitamin E, HBV, HCV, ANA, ANCA, ENA, CRP, ESR, glycemia, HbA1c, copper, cryoglobulins, serum electrophoresis<br>**Family History:**<br>● No family history of polyneuropathy<br>**Differential Diagnoses for Polyneuropathy**<br>**1. Chronic Inflammatory Demyelinating Polyneuropathy (CIDP)**<br>**Reasoning:**<br>● **Clinical Presentation:** CIDP often presents with progressive sensory and motor deficits, which can be asymmetric and proximal/distal in distribution, similar to this patient's presentation.<br>● **Nerve Conduction Studies:** Demyelination seen in the nerve conduction studies is characteristic of CIDP.<br>● **Subacute Onset and Progression:** CIDP typically has a progressive course over weeks to months, aligning with the patient's history of a 1-year progression.<br>● **Exclusion of Other Causes:** Extensive negative screening tests for other potential causes of neuropathy, such as diabetes, infections (HBV, HCV), and autoimmune markers (ANA, ANCA), support the consideration of CIDP.<br>● **Lack of Systemic Symptoms:** The absence of systemic involvement (e.g., autonomic or cranial nerve involvement) is also consistent with CIDP.<br>**2. Multifocal Motor Neuropathy (MMN)**<br>**Reasoning:**<br>● **Clinical Presentation:** MMN often presents with asymmetric, multifocal motor involvement without significant sensory loss, though sensory involvement can sometimes occur.<br>● **Nerve Conduction Studies:** While MMN typically shows conduction block in motor nerves, demyelination may be present in severe cases.<br>● **Asymmetric and Proximal Involvement:** The asymmetric, proximal, and distal motor weakness fits the pattern seen in MMN.<br>● **Lack of Systemic Symptoms:** Like CIDP, MMN usually does not involve systemic symptoms or significant sensory impairment, which aligns with the clinical findings in this patient.<br>**3. Multifocal Acquired Sensory and Motor Neuropathy (MADSAM) or Lewis-Sumner Syndrome**<br>**Reasoning:**<br>● **Clinical Presentation:** MADSAM is characterized by asymmetric, multifocal sensory and motor neuropathy. The patient's presentation of both proximal and distal sensory-motor involvement fits well with this diagnosis.<br>● **Nerve Conduction Studies:** Demyelination in nerve conduction studies is also seen in MADSAM, supporting this diagnosis.<br>● **Progressive Course:** The subacute onset and progressive nature of the symptoms are consistent with MADSAM.<br>● **Exclusion of Other Causes:** The negative extensive workup for other etiologies further supports the consideration of MADSAM.<br>**Most Likely Diagnosis**<br>**Chronic Inflammatory Demyelinating Polyneuropathy (CIDP)** is the most likely diagnosis for this patient. The reasoning includes:<br>● The combination of sensory and motor involvement with demyelinating features on nerve conduction studies.<br>● The progressive, subacute course over a year.<br>● The exclusion of other causes through extensive negative testing.<br>● The presence of asymmetric distribution, which, although less common, can occur in CIDP.<br>While MMN and MADSAM are also possible, CIDP remains the most comprehensive diagnosis given the patient's symptomatology, examination findings, and nerve conduction study results. The identification and treatment of folate deficiency is also important, but it is unlikely to be the primary cause of the demyelinating neuropathy observed in this patient.
98	A 74-year-old male patient presents with a 1-month of sensory-motor, multifocal neuropathy with subacute onset and stepwise progression. The patient presents with paresthesia, neuropathic pain, and weakness with a multifocal distribution in the four limbs. He also reports arthralgia and headache, which began in the last two weeks. Clinical examination excludes the presence of sensory ataxia, tremor, foot deformities, cranial nerve or autonomic involvement. Past medical history is positive for arterial hypertension, Hashimoto's thyroiditis, chronic HBV infection, and type 2 diabetes mellitus. Nerve conduction studies show a multifocal, axonal, sensory-motor	Vasculitic neuropathy	**Summary of Pertinent Clinical Information**<br>● **Patient Demographics**: 74-year-old male<br>● **Symptoms**:<br>○ Sensory-motor neuropathy (paresthesia, neuropathic pain, weakness)<br>○ Multifocal distribution in four limbs<br>○ Subacute onset with stepwise progression<br>○ Arthralgia and headache (last two weeks)<br>● **Clinical Examination**:<br>○ No sensory ataxia, tremor, foot deformities<br>○ No cranial nerve or autonomic involvement<br>● **Past Medical History**:<br>○ Arterial hypertension<br>○ Hashimoto's thyroiditis<br>○ Chronic HBV infection<br>○ Type 2 diabetes mellitus<br>● **Nerve Conduction Studies**: Multifocal, axonal, sensory-motor neuropathy<br>● **Lab Results**:

	neuropathy. Screening lab tests reveal elevated glycemia and HbA1c leves, vitamin B12 deficiency, and raised ESR and CRP. Remaining exams including folate, vitamin B6, vitamin E, HCV, ANA, ANCA, ENA, copper, cryoglobulins, and serum electrophoresis are normal. There is no family history of polyneuropathy.		○ Elevated glycemia and HbA1c<br>○ Vitamin B12 deficiency<br>○ Raised ESR and CRP<br>○ Normal: Folate, vitamin B6, vitamin E, HCV, ANA, ANCA, ENA, copper, cryoglobulins, and serum electrophoresis<br>● **Family History**: No polyneuropathy<br>**Differential Diagnoses for Polyneuropathy**<br>1. **Diabetic Neuropathy**<br>○ **Reasoning**: The patient has type 2 diabetes mellitus, which is a common cause of polyneuropathy. Elevated glycemia and HbA1c levels indicate poor glycemic control, which contributes to nerve damage. Although diabetic neuropathy typically presents with distal symmetric polyneuropathy, multifocal involvement can occur, especially in the context of poor glycemic control.<br>○ **Supporting Features**:<br>■ Elevated glycemia and HbA1c<br>■ Chronic diabetes mellitus<br>○ **Contradicting Features**:<br>■ The multifocal nature and stepwise progression are somewhat atypical for classic diabetic neuropathy.<br>2. **Vasculitic Neuropathy (e.g., Polyarteritis Nodosa)**<br>○ **Reasoning**: The combination of subacute onset, stepwise progression, multifocal involvement, elevated ESR and CRP, and associated systemic symptoms (arthralgia and headache) suggests a vasculitic process. Polyarteritis nodosa, a type of vasculitis, can cause multifocal, axonal, sensory-motor neuropathy due to ischemic damage to the nerves.<br>○ **Supporting Features**:<br>■ Subacute, stepwise progression of symptoms<br>■ Multifocal neuropathy<br>■ Elevated ESR and CRP<br>■ Systemic symptoms (arthralgia and headache)<br>○ **Contradicting Features**:<br>■ Absence of specific positive autoimmune markers (ANA, ANCA)<br>■ Lack of other systemic involvement typically seen in vasculitis<br>3. **Vitamin B12 Deficiency Neuropathy**<br>○ **Reasoning**: Vitamin B12 deficiency is known to cause a sensory-motor neuropathy. However, it more commonly presents with a distal symmetric pattern rather than a multifocal distribution. The patient's confirmed vitamin B12 deficiency could contribute to neuropathic symptoms, especially in the context of other underlying conditions.<br>○ **Supporting Features**:<br>■ Confirmed vitamin B12 deficiency<br>■ Sensory-motor neuropathy fitting the profile of B12 deficiency<br>○ **Contradicting Features**:<br>■ Multifocal distribution is less typical for B12 deficiency neuropathy<br>■ Rapid, stepwise progression is also less typical<br>**Most Likely Diagnosis**<br>Considering the patient's clinical presentation, medical history, and diagnostic findings, **vasculitic neuropathy** (e.g., Polyarteritis Nodosa) is the most likely diagnosis. The multifocal, stepwise progression of symptoms along with systemic signs such as arthralgia and headache, and elevated inflammatory markers (ESR and CRP) point towards a vasculitic process. While diabetic neuropathy and vitamin B12 deficiency neuropathy are also possible, the clinical features and progression align more closely with vasculitic neuropathy. Further evaluation, including a nerve biopsy and possibly a detailed angiographic study, may be warranted to confirm the diagnosis and guide treatment.
99	A 71-year-old male patient presents with a 2-year history of sensory-motor, symmetrical, length dependent polyneuropathy with insidious onset and fast disease progression. The patient presents with moderate distal weakness and hypoesthesia in the four limbs, sensory ataxia and autonomic involvement with no foot deformities or neuropathic pain. Past medical history is positive for cardiac insufficiency. Nerve conduction studies show an axonal, sensory-motor polyneuropathy with bilateral carpal tunnel syndrome. Screening lab tests including vitamin B12, folate, vitamin B6, vitamin E, HBV, HCV, ANA, ANCA, ENA, glycaemia, HbA1c, CRP, ESR, copper, cryoglobulins, and serum electrophoresis are all negative. His brother is wheelchair bound.	TTR-Amyloidosis	**Step 1: Summarize Pertinent Clinical Information**<br>**Patient Information:**<br>● **Age/Gender:** 71-year-old male<br>● **Symptom Duration:** 2 years<br>● **Symptom Type:** Sensory-motor, symmetrical, length-dependent polyneuropathy<br>● **Onset:** Insidious<br>● **Progression:** Fast<br>● **Weakness:** Moderate distal weakness in four limbs<br>● **Hypoesthesia:** Present in four limbs<br>● **Sensory Ataxia:** Present<br>● **Autonomic Involvement:** Present<br>● **Foot Deformities:** None<br>● **Neuropathic Pain:** None<br>**Medical History:**<br>● **Cardiac Insufficiency**<br>● **Family History:** Brother is wheelchair-bound<br>**Nerve Conduction Studies:**<br>● Axonal, sensory-motor polyneuropathy<br>● Bilateral carpal tunnel syndrome<br>**Screening Lab Tests:**<br>● All negative: vitamin B12, folate, vitamin B6, vitamin E, HBV, HCV, ANA, ANCA, ENA, glycaemia, HbA1c, CRP, ESR, copper, cryoglobulins, and serum electrophoresis<br>**Step 2: Generate Three Most Probable Differential Diagnoses**<br>**1. Amyloid Neuropathy (Familial Amyloid Polyneuropathy)**<br>● **Reasoning:** The presence of a rapidly progressive, sensory-motor, symmetrical polyneuropathy with autonomic involvement in an elderly patient is highly suggestive of amyloid neuropathy. The negative screening tests for metabolic and infectious causes, combined with the family history (brother being wheelchair-bound), point towards a genetic component, which is consistent with hereditary amyloidosis.

			**2. Hereditary Neuropathy (Charcot-Marie-Tooth Disease)**<br>● **Reasoning:** Given the patient's age and the insidious onset of symptoms, a hereditary neuropathy like Charcot-Marie-Tooth (CMT) disease could be considered. Although CMT typically presents earlier in life, there are later-onset forms. The family history of neuropathy and the presence of bilateral carpal tunnel syndrome align with CMT features. However, the rapid progression and autonomic involvement are less typical for CMT, making this less likely than amyloid neuropathy.<br>**3. Chronic Inflammatory Demyelinating Polyneuropathy (CIDP)**<br>● **Reasoning:** CIDP could present with a progressive, sensory-motor polyneuropathy, but it usually shows more prominent demyelinating features on nerve conduction studies. The presence of autonomic involvement and rapid progression is more consistent with amyloid neuropathy, but CIDP should be considered due to its variability in presentation. The negative inflammatory markers (CRP, ESR) and absence of response to typical CIDP treatments (assuming no prior treatment) make this diagnosis less probable.<br>**Step 3: Determine the Most Likely Diagnosis**<br>**Most Likely Diagnosis: Amyloid Neuropathy (Familial Amyloid Polyneuropathy)**<br>**Reasoning:**<br>● The patient's clinical presentation (rapidly progressive, sensory-motor, symmetrical polyneuropathy with autonomic involvement) strongly suggests amyloid neuropathy.<br>● The negative extensive metabolic and infectious screening tests point away from other common causes of polyneuropathy.<br>● Family history of a similar severe neuropathy supports a hereditary etiology, which is consistent with familial amyloid polyneuropathy.<br>● The presence of bilateral carpal tunnel syndrome also supports this diagnosis, as amyloid deposits can often affect the carpal tunnel.<br>Given these factors, familial amyloid polyneuropathy is the most fitting diagnosis for this patient's clinical presentation. Further confirmatory tests, such as genetic testing for transthyretin mutations or a biopsy showing amyloid deposits, would solidify this diagnosis.
100	A 76-year-old female patient presents with a 6-year history of chronic, pure sensory, symmetrical, length dependent polyneuropathy with insidious onset and slow progressive course. The main symptom is severe burning and tingling pain in the feet. Clinical examination reveals hyperalgesia upon pinprick testing, along with redness and warmth in the feet. There are no weakness or tactile hypoesthesia, sensory ataxia, tremor, foot deformities, or cranial nerve involvement. Past medical history is silent. Nerve conduction studies are normal with no signs of polyneuropathy. Screening lab tests including vitamin B12, folate, vitamin B6, vitamin E, HBV, HCV, ANA, ANCA, ENA, CRP, ESR, glycemia, HbA1c, copper, cryoglobulins, and serum electrophoresis are negative. There is family history of polyneuropathy.	Small fibre neuropathy	**Step-by-Step Analysis of the Clinical Scenario**<br>**Patient Summary**<br>● **Demographics**: 76-year-old female<br>● **History**: 6-year history of chronic, pure sensory, symmetrical, length-dependent polyneuropathy<br>● **Symptoms**: Severe burning and tingling pain in the feet<br>● **Onset and Course**: Insidious onset, slow progressive course<br>● **Clinical Examination**:<br>○ Hyperalgesia upon pinprick testing<br>○ Redness and warmth in the feet<br>○ No weakness, tactile hypoesthesia, sensory ataxia, tremor, foot deformities, or cranial nerve involvement<br>● **Past Medical History**: Silent<br>● **Family History**: Polyneuropathy<br>● **Diagnostic Tests**:<br>○ Nerve conduction studies: Normal<br>○ Screening lab tests (including vitamin levels, infections, autoimmune markers, inflammatory markers, glycemia, HbA1c, copper, cryoglobulins, serum electrophoresis): Negative<br>**Differential Diagnoses for Polyneuropathy**<br>1. **Small Fiber Neuropathy (SFN)**<br>○ **Reasoning**:<br>■ **Clinical Presentation**: The patient presents with pure sensory symptoms (burning and tingling pain) and hyperalgesia, which are characteristic of SFN.<br>■ **Normal Nerve Conduction Studies**: SFN typically affects small unmyelinated fibers that are not detected by standard nerve conduction studies, explaining the normal results.<br>■ **Redness and Warmth**: These symptoms can be due to autonomic involvement, often seen in SFN.<br>■ **Family History**: There can be a genetic predisposition to SFN.<br>○ **Additional Tests**:<br>■ Skin biopsy to assess intraepidermal nerve fiber density (IENFD)<br>■ Quantitative sensory testing (QST)<br>2. **Hereditary Sensory and Autonomic Neuropathy (HSAN)**<br>○ **Reasoning**:<br>■ **Clinical Presentation**: Chronic and slowly progressive sensory neuropathy with autonomic features (redness and warmth) fits HSAN.<br>■ **Family History**: The presence of a family history supports a hereditary cause.<br>■ **Normal Nerve Conduction Studies**: HSAN can primarily affect small fibers, leading to normal nerve conduction studies.<br>○ **Additional Tests**:<br>■ Genetic testing for mutations associated with HSAN<br>■ Detailed family history and possibly examining affected family members<br>3. **Idiopathic Small Fiber Neuropathy**<br>○ **Reasoning**:<br>■ **Clinical Presentation**: Similar to SFN with pure sensory symptoms and autonomic features.<br>■ **Normal Nerve Conduction Studies**: Again, normal studies align with small fiber involvement.<br>■ **Screening Lab Tests Negative**: When no underlying cause is identified, idiopathic SFN is considered.<br>○ **Additional Tests**:<br>■ Same as for SFN: Skin biopsy and QST to confirm small fiber involvement.<br>**Most Likely Diagnosis**<br>The most likely diagnosis for this patient is **Small Fiber Neuropathy (SFN)**. The clinical presentation with severe burning pain, hyperalgesia, and autonomic features like redness and warmth, combined with normal nerve conduction studies and a family history of polyneuropathy, strongly suggest SFN. Furthermore, the absence of any abnormal findings in extensive lab workups supports this diagnosis.<br>To confirm SFN, a skin biopsy to measure intraepidermal nerve fiber density (IENFD) would be the next step, along with quantitative sensory testing (QST) to further evaluate small fiber function. Genetic testing could also be considered given the family history, to rule out or confirm hereditary forms of SFN or HSAN.

Supplementary Table 2. Trial 1 – Case presentations and GPT-4o output to 2nd prompt (confirmatory test)

LEGEND: Anti-MAG neuropathy, anti-myelin-associated glycoprotein neuropathy; CANVAS, cerebellar ataxia, neuropathy, and vestibular areflexia syndrome; CIDP, chronic inflammatory demyelinating polyneuropathy; CMT, Charcot-Marie-Tooth disease; GBS, Guillain-Barré syndrome; HBV, hepatitis B virus; HCV, hepatitis C virus; HNPP, hereditary neuropathy with liability to pressure palsies; MGUS, monoclonal gammopathy of undetermined significance; MMN, multifocal motor neuropathy; POEMS, polyneuropathy, organomegaly, endocrinopathy, monoclonal gammopathy, and skin changes syndrome; TTR-Amyloidosis, transthyretin amyloidosis-associated neuropathy;

N°	Case presentation and Diagnosis	Diagnosis	Prompt 2 output
1	**Case presentation:** A 32-year old male patient presents with a 26-year history of sensory-motor, symmetrical, length dependent polyneuropathy with insidious onset and slow progression. The patient presents with scoliosis, neuropathic pain, sensory ataxia, pes cavus, distal weakness in the four limbs, severe calf atrophy with no cranial nerve or autonomic involvement. Past medical history is silent. Nerve conduction studies show an axonal, length dependent, symmetric, sensory-motor polyneuropathy. Screening lab tests including vitamin B12, folate, vitamin B6, vitamin E, HBV, HCV, glycemia, HbA1c, ANA, ANCA, ENA, CRP, ESR, copper, cryoglobulins, and serum electrophoresis are all negative. Both her father and grandfather have foot deformities and walking difficulties.	CMT2	**Additional Examinations**<br>1. Genetic Testing<br>○ Reasoning: Given the strong family history of similar symptoms and the hereditary nature of conditions like Charcot-Marie-Tooth disease, genetic testing is crucial. It can identify specific mutations in genes associated with CMT (e.g., PMP22, MPZ, GJB1, MFN2 for CMT1 and CMT2 subtypes). Identifying a genetic mutation would confirm the diagnosis and differentiate between the various types of hereditary neuropathies.<br>2. Nerve Biopsy<br>○ Reasoning: While not routinely performed, a nerve biopsy can be helpful in ambiguous cases. It can show characteristic features of hereditary neuropathies, such as onion bulb formations in demyelinating types or loss of myelinated fibers in axonal types. This would help in further characterizing the type of neuropathy.<br>3. Electromyography (EMG)<br>○ Reasoning: EMG can complement nerve conduction studies by assessing the electrical activity of muscles. It can help in distinguishing between neuropathic and myopathic processes, providing additional information on the extent and distribution of muscle involvement.<br>4. Magnetic Resonance Imaging (MRI) of the Spine<br>○ Reasoning: An MRI of the spine can assess the extent of scoliosis and look for any other spinal cord or nerve root abnormalities that could contribute to the neuropathy. This is important in evaluating the structural aspects and ruling out other causes of the symptoms.<br>5. Autonomic Function Tests<br>○ Reasoning: Even though there are no current signs of autonomic involvement, autonomic function tests can be useful. They can help identify subclinical autonomic neuropathy which may not be apparent through symptoms alone but could be present in some hereditary neuropathies.<br>6. Quantitative Sensory Testing (QST)<br>○ Reasoning: QST can assess the functional status of the sensory nerves, quantifying the thresholds for detecting sensory stimuli like vibration, temperature, and pain. This can provide detailed information about sensory deficits and contribute to a more comprehensive understanding of the sensory involvement.<br>7. Lumbar Puncture<br>○ Reasoning: Analyzing cerebrospinal fluid (CSF) can help rule out inflammatory or infectious causes of neuropathy. Elevated protein levels with normal cell count can be seen in chronic inflammatory demyelinating polyneuropathy (CIDP), which needs to be differentiated from hereditary neuropathies.<br>8. Ophthalmological Examination<br>○ Reasoning: Some hereditary neuropathies can have associated ocular manifestations. A thorough eye examination can identify subclinical involvement that might help in narrowing down the diagnosis.<br>**Conclusion: Most Crucial Test**<br>The most crucial test from the list is Genetic Testing. This test directly targets the underlying cause of the symptoms, given the strong hereditary pattern and characteristic presentation. Identifying a genetic mutation associated with CMT or another hereditary neuropathy would not only confirm the diagnosis but also guide management and genetic counseling for the patient and their family.<br>**Detailed Analysis and Reasoning**<br>1. Genetic Testing<br>○ Details: This test involves analyzing the patient's DNA for known mutations associated with hereditary neuropathies. Panels for CMT can include many genes like PMP22, MPZ, GJB1, MFN2, among others.<br>○ Reasoning: The family history and clinical presentation strongly suggest a genetic disorder. Identifying the specific mutation will confirm the diagnosis and help differentiate between types of hereditary neuropathies.<br>2. Nerve Biopsy<br>○ Details: A small sample of a peripheral nerve (usually the sural nerve) is taken and examined microscopically.<br>○ Reasoning: This can show structural abnormalities typical of hereditary neuropathies, such as axonal degeneration or demyelination with onion bulb formations.<br>3. Electromyography (EMG)<br>○ Details: This test measures the electrical activity of muscles at rest and during contraction.<br>○ Reasoning: It helps to confirm the presence of neuropathy by showing denervation and reinnervation patterns. It also helps differentiate between neuropathic and myopathic causes of muscle weakness.<br>4. MRI of the Spine<br>○ Details: An MRI provides detailed images of the spine and spinal cord.<br>○ Reasoning: It helps evaluate the structural aspects of scoliosis and can rule out other spinal cord pathologies that might mimic or contribute to the neuropathy.<br>5. Autonomic Function Tests<br>○ Details: These tests assess the function of the autonomic nervous system through various physiological measurements.<br>○ Reasoning: They can detect subclinical autonomic involvement, which, while not reported in this case, could provide additional diagnostic information.<br>6. Quantitative Sensory Testing (QST)<br>○ Details: QST assesses sensory nerve function by determining the threshold for detecting different sensory stimuli.<br>○ Reasoning: It provides a quantitative measure of sensory deficits, adding detail to the sensory examination findings.<br>7. Lumbar Puncture<br>○ Details: This involves taking a sample of CSF from the lower back to analyze its composition.<br>○ Reasoning: It helps rule out inflammatory or infectious causes of neuropathy and can show elevated protein levels indicative of chronic inflammatory conditions.<br>8. Ophthalmological Examination<br>○ Details: This includes a comprehensive eye exam to check for any ocular abnormalities.<br>○ Reasoning: Some hereditary neuropathies have associated eye findings, and an ophthalmological exam can help identify these and narrow down the differential diagnosis.<br>**Conclusion**<br>The Genetic Testing is the most crucial test. It is directly aligned with the patient's clinical presentation and family history, and it has the highest potential to provide a definitive diagnosis by identifying specific genetic mutations associated with hereditary neuropathies.

2	**Case presentation:** An 88-year-old male patient presents with a 19-month history of pure sensory, symmetrical, length dependent polyneuropathy with insidious onset and slow progressive course. The patient presents with hypoesthesia, paresthesia, and cramps in the lower limbs in a stocking distribution. Clinical examination does not reveal weakness or sensory ataxia, neuropathic pain, tremor, foot deformities, cranial nerve or autonomic involvement. Past medical history is positive for colorectal cancer, for which the patient underwent chemotherapy 5 years ago, and chronic HCV infection. Nerve conduction studies show a pure sensory, symmetric, length-dependent axonal polyneuropathy with unilateral carpal tunnel syndrome. Screening lab tests including vitamin B12, folate, vitamin B6, vitamin E, HBV, glycemia, HbA1c, ANA, ANCA, ENA, CRP, ESR, copper, cryoglobulins, and serum electrophoresis are normal, with positivity for HCV IgG. There is no family history of polyneuropathy.	HCV-associated neuropathy	**Step-by-Step Analysis**<br>**Step 1: Review Initial Findings and Differential Diagnoses**<br>**Key Points:**<br>● 88-year-old male with a 19-month history of pure sensory, symmetrical, length-dependent polyneuropathy.<br>● Symptoms: Hypoesthesia, paresthesia, and cramps in lower limbs.<br>● Past medical history: Colorectal cancer (chemotherapy 5 years ago), chronic HCV infection.<br>● Nerve conduction studies: Pure sensory, symmetric, length-dependent axonal polyneuropathy.<br>● Lab tests: Normal except for positive HCV IgG.<br>● No family history of polyneuropathy.<br>**Differential Diagnoses:**<br>1. Hepatitis C Virus (HCV)-Related Neuropathy<br>2. Chemotherapy-Induced Neuropathy<br>3. Idiopathic Sensory Polyneuropathy<br>**Step 2: Identify Gaps and Additional Tests Needed**<br>To further narrow down the differential diagnoses, additional tests should focus on:<br>● Evaluating the extent and specific characteristics of the neuropathy.<br>● Identifying any other potential underlying causes or contributing factors.<br>● Confirming or ruling out HCV-related complications.<br>**Step 3: List of Additional Examinations**<br>1. HCV RNA Quantification and Genotyping<br>○ Reasoning: Quantifying HCV RNA levels can determine the viral load, indicating active infection. Genotyping can provide insights into the specific strain, which may influence the likelihood of neuropathy.<br>2. Nerve Biopsy<br>○ Reasoning: A biopsy of a sensory nerve can provide direct histological evidence of axonal degeneration, inflammation, or vasculitis, helping to differentiate between HCV-related neuropathy and other causes.<br>3. Skin Biopsy for Intraepidermal Nerve Fiber Density (IENFD)<br>○ Reasoning: Assessing small fiber involvement through skin biopsy can detect early and subtle changes in peripheral neuropathy, which may not be apparent on nerve conduction studies.<br>4. Anti-Neuronal Antibodies (e.g., Anti-Hu, Anti-Ri, Anti-Yo)<br>○ Reasoning: Testing for paraneoplastic antibodies can identify a possible paraneoplastic syndrome, especially given the patient's history of colorectal cancer.<br>5. Lumbar Puncture with Cerebrospinal Fluid (CSF) Analysis<br>○ Reasoning: CSF analysis can help rule out central nervous system involvement or inflammatory neuropathies by checking for elevated protein, pleocytosis, or other abnormalities.<br>6. MRI of the Spine<br>○ Reasoning: MRI can evaluate for structural causes of neuropathy, such as spinal stenosis, which can mimic peripheral neuropathy symptoms.<br>7. HCV-Related Autoimmune Marker Panel (e.g., Rheumatoid Factor, Complement Levels)<br>○ Reasoning: Assessing autoimmune markers can help identify immune-mediated mechanisms of neuropathy related to HCV, such as mixed cryoglobulinemia or other autoimmune conditions.<br>8. Electromyography (EMG)<br>○ Reasoning: EMG can further characterize the neuropathy by assessing muscle response to nerve stimulation, helping to distinguish between purely sensory and sensorimotor neuropathy.<br>**Step 4: Determine the Most Crucial Test**<br>**Most Crucial Test: HCV RNA Quantification and Genotyping**<br>Reasoning: This test is crucial because:<br>● It confirms active HCV infection and viral load.<br>● It provides genotype information that can influence the interpretation of HCV-related complications.<br>● Establishing the activity and type of HCV infection can significantly impact management decisions and confirm the suspected primary etiology of the neuropathy.<br>**Conclusion**<br>To narrow down the differential diagnoses, the most crucial test from the list is HCV RNA Quantification and Genotyping. This test will provide essential information about the active infection status and the specific HCV strain, helping to confirm whether the neuropathy is indeed related to chronic HCV infection.
3	**Case presentation:** A 75-year-old female patient presents with a 1-year history of sensory-motor, symmetrical, length dependent polyneuropathy with subacute onset. The patient presents with paresthesia and hypoesthesia in the lower limbs and sensory ataxia, without tremor, foot deformities, neuropathic pain, cranial nerve or autonomic involvement. Past medical history shows breast cancer, for which the patient has undergone chemotherapy. Neuropathy symptoms started during chemotherapy. Nerve conduction studies show a symmetric, sensory-motor, axonal polyneuropathy. Screening lab tests including vitamin B12, folate, vitamin B6, vitamin E, HCV, HBV, ANA, ANCA, ENA, CRP, ESR, glycemia,	Chemotherapy induced polyneuropathy	**Step-by-Step Analysis and Additional Examinations:**<br>**1. Review and Analysis of Existing Data:**<br>○ Current Findings: Symmetrical, sensory-motor, axonal polyneuropathy, onset during chemotherapy for breast cancer, negative comprehensive screening labs.<br>○ Clinical Presentation: Paresthesia, hypoesthesia, sensory ataxia, absence of tremor, foot deformities, neuropathic pain, cranial nerve, and autonomic involvement.<br>**2. Identifying Gaps in Current Data:**<br>○ Chemotherapy-Induced Neuropathy: Consider ruling out other causes to confirm this diagnosis.<br>○ Paraneoplastic and Other Neuropathies: Further investigation required to rule out rarer causes.<br>**3. Proposed Additional Examinations:**<br>**a. Quantitative Sensory Testing (QST):**<br>○ **Reasoning:** QST assesses the sensory threshold to mechanical, thermal, and pain stimuli. This test can quantify the extent of sensory dysfunction and differentiate between small fiber and large fiber involvement, which might help to better characterize the neuropathy type.<br>**b. Skin Biopsy for Intraepidermal Nerve Fiber Density (IENFD):**<br>○ **Reasoning:** This test helps in evaluating small fiber neuropathy, which may not be evident on nerve conduction studies. A reduction in intraepidermal nerve fiber density can support a diagnosis of small fiber involvement, which is common in chemotherapy-induced neuropathy.<br>**c. Anti-Hu and Anti-Yo Antibody Testing:**<br>○ **Reasoning:** These tests detect antibodies associated with paraneoplastic syndromes. Given the history of breast cancer, paraneoplastic neuropathy should be considered. Although autoimmune markers were negative, these specific antibodies can sometimes be present in paraneoplastic conditions.<br>**d. Lumbar Puncture and Cerebrospinal Fluid (CSF) Analysis:**

	HbA1c, copper, cryoglobulins and serum electrophoresis are all negative. There is no family history of polyneuropathy.		○ **Reasoning:** CSF analysis can provide information about inflammatory or infectious causes of neuropathy. Elevated protein levels with normal cell counts (albuminocytologic dissociation) can be indicative of Guillain-Barré syndrome or chronic inflammatory demyelinating polyneuropathy (CIDP), while abnormal cells or proteins might suggest a paraneoplastic process.<br>**e. Genetic Testing for Hereditary Neuropathies:**<br>○ **Reasoning:** While there is no family history, some hereditary neuropathies can present later in life and might not have clear familial patterns. Genetic testing can help rule out hereditary causes of axonal neuropathy, such as Charcot-Marie-Tooth disease.<br>**f. Electromyography (EMG):**<br>○ **Reasoning:** EMG can further characterize the type and extent of neuropathy by assessing muscle electrical activity, which can help differentiate between neuropathic and myopathic processes and provide additional information on the extent of axonal damage.<br>**g. Serum Protein Electrophoresis with Immunofixation:**<br>○ **Reasoning:** While initial serum electrophoresis was negative, immunofixation can be more sensitive in detecting monoclonal gammopathies, which can be associated with neuropathy. Multiple myeloma and monoclonal gammopathy of undetermined significance (MGUS) are potential considerations.<br>**4. Determining the Most Crucial Test:**<br>Skin Biopsy for Intraepidermal Nerve Fiber Density (IENFD) is likely the most crucial test. This test specifically evaluates small fiber neuropathy, which is common in chemotherapy-induced neuropathy. Given the patient's clinical presentation and history of chemotherapy, confirming the involvement of small fibers would significantly support the diagnosis of CIPN. It also provides direct evidence of nerve damage that can correlate with clinical symptoms.<br>**Summary:**<br>While several additional tests can aid in narrowing down the differential diagnosis, the Skin Biopsy for Intraepidermal Nerve Fiber Density (IENFD) is the most crucial due to its direct assessment of small fiber neuropathy, which is pertinent to confirming chemotherapy-induced peripheral neuropathy. Other tests, such as anti-Hu and anti-Yo antibody testing and lumbar puncture, should be considered based on initial findings from the skin biopsy and further clinical suspicion.
4	**Case presentation:** An 81-year-old male patient presents with a 9-week history of sensory-motor, asymmetrical multineuropathy with subacute onset and progressive course. The patient presents with paresthesia, hypoesthesia, neuropathic pain, and weakness with a multifocal distribution. Clinical examination does not reveal sensory ataxia nor tremor, foot deformities, cranial nerve or autonomic involvement. Past medical history is positive for giant cell arteritis. Nerve conduction studies show a sensory-motor, axonal multineuropathy. Screening lab tests including vitamin B12, folate, vitamin B6, vitamin E, HBV, HCV, glycemia, HbA1c, ANA, ENA, copper, cryoglobulins, and serum electrophoresis are all negative, with evidence of elevated CRP and ESR. There is no family history of polyneuropathy.	Vasculitic neuropathy	**Step-by-Step Analysis and Additional Examinations**<br>**Step 1: Detailed Clinical History and Examination**<br>● Revisit the Detailed Medical History: Obtain more details about the patient's previous diagnosis and treatment for giant cell arteritis. Note any medications used and their duration.<br>● Neurological Examination: Conduct a thorough neurological examination to document the exact pattern of sensory and motor deficits, reflex changes, and any subtle signs that may have been initially overlooked.<br>**Step 2: Additional Laboratory Tests**<br>1. Anti-Neutrophil Cytoplasmic Antibodies (ANCA)<br>○ Reasoning: ANCA-associated vasculitides (e.g., granulomatosis with polyangiitis) can present with neuropathy. Given the patient's history of vasculitis, ANCA testing is pertinent.<br>2. Serum Immunofixation Electrophoresis (IFE)<br>○ Reasoning: To rule out monoclonal gammopathies, which can be associated with peripheral neuropathies, including those due to paraneoplastic syndromes.<br>3. Anti-Hu, Anti-Ri, and Anti-Yo Antibodies<br>○ Reasoning: These are paraneoplastic antibodies that can help identify underlying malignancies associated with neuropathies.<br>4. Lyme Disease Serology (ELISA and Western Blot)<br>○ Reasoning: Lyme disease can present with neuropathic symptoms and may be relevant, particularly if there is any history of tick exposure or residence in endemic areas.<br>5. HIV and Syphilis Serology<br>○ Reasoning: Both HIV and syphilis can present with neuropathy. Despite low suspicion based on history, screening is prudent due to treatability.<br>6. CSF Analysis (Lumbar Puncture)<br>○ Reasoning: Elevated protein levels and specific cell counts can indicate inflammatory or infectious processes. Useful for diagnosing conditions like CIDP or vasculitic neuropathies.<br>**Step 3: Additional Instrumental Examinations**<br>**1. Nerve Biopsy**<br>○ **Reasoning**: Provides direct evidence of vasculitis or other pathological changes in the nerve. Essential for definitive diagnosis of vasculitic neuropathy.<br>**2. Skin Biopsy (for Small Fiber Neuropathy)**<br>○ Reasoning: Can be useful to identify small fiber neuropathy, which might not be evident on routine nerve conduction studies.<br>**3. MRI of Brain and Spinal Cord**<br>○ **Reasoning:** To rule out any central nervous system pathology that could contribute to neuropathic symptoms. Specific sequences can identify nerve root or plexus involvement.<br>**4. PET-CT Scan**<br>○ **Reasoning:** To detect occult malignancies that could be causing paraneoplastic syndromes.<br>**5. Repeat Electromyography (EMG) and Nerve Conduction Studies (NCS)**<br>○ **Reasoning:** To confirm initial findings and assess progression or new areas of involvement.<br>**Step 4: Targeted Investigations Based on History and Examination**<br>**1. Temporal Artery Biopsy (if recurrent GCA suspected)**<br>○ **Reasoning:** Though the patient is known to have GCA, a repeat biopsy can confirm active disease if there is suspicion of recurrence.<br>**2. Autoimmune Panel (comprehensive)**<br>○ **Reasoning:** Includes tests like anti-MAG antibodies, which can identify specific immune-mediated neuropathies.<br>**Most Crucial Test**<br>**Nerve Biopsy**<br>● **Reasoning:** Given the high suspicion of vasculitic neuropathy based on clinical history, subacute presentation, progressive course, and elevated inflammatory markers (CRP, ESR), a nerve biopsy is the most crucial test. It can provide definitive pathological evidence of vasculitis affecting the nerves, guiding targeted immunosuppressive therapy. This test directly addresses the most likely differential diagnosis and has significant implications for treatment and prognosis.<br>**Conclusion**<br>While multiple tests are necessary to fully evaluate this patient's condition and rule out other differential diagnoses, the nerve biopsy stands out as the most critical test. It will provide direct evidence of vasculitic involvement, confirming the diagnosis and allowing for appropriate management.
5	A 61-year-old male patient presents with a 1-year history of sensory-motor, asymmetric, non-length dependent	POEMS	**Step-by-Step Analysis for Additional Examinations:**<br>**1. Summary of Clinical Data:**<br>● **Patient Profile: 61-year-old male with psoriatic arthritis**

	polyneuropathy with subacute onset and progressive course. The patient presents with asymmetric distal paresthesia and neuropathic pain distributed in the four limbs, associated with weakness in the four limbs both proximally and distally. Clinical examination also shows the presence of mild sensory ataxia, peripheral edema in the feet, and mild cutaneous hyperpigmentation in the lower limbs. There are no autonomic symptoms or foot deformities. Past medical history is positive for psoriatic arthritis. Nerve conduction studies show a demyelinating sensory-motor polyneuropathy. Screening lab tests show presence of IgG lambda paraprotein and elevated CRP. Remaining exams, including vitamin B12, folate, vitamin B6, vitamin E, HBV, HCV, ANA, ENA, ANCA, ESR, glycemia, HbA1c, copper, and cryoglobulins are negative. There is no family history of polyneuropathy.		• **Symptoms: Sensory-motor, asymmetric, non-length dependent polyneuropathy; subacute onset; progressive course; asymmetric distal paresthesia; neuropathic pain; proximal and distal limb weakness; mild sensory ataxia; peripheral edema; cutaneous hyperpigmentation**<br>• **Nerve Conduction Studies: Demyelinating sensory-motor polyneuropathy**<br>• **Lab Findings: Positive IgG lambda paraprotein; elevated CRP**<br>• **Negative Tests: Vitamin B12, folate, vitamin B6, vitamin E, HBV, HCV, ANA, ENA, ANCA, ESR, glycemia, HbA1c, copper, cryoglobulins**<br>• **Family History: No family history of polyneuropathy**<br>**2. Additional Examinations:**<br>**a) Serum Free Light Chains (sFLC) Assay:**<br>• **Reasoning:**<br>○ To quantify free kappa and lambda light chains.<br>○ Helps in evaluating the monoclonal protein further, confirming its type and concentration.<br>○ Useful in distinguishing MGUS from more serious plasma cell disorders like multiple myeloma or POEMS syndrome.<br>**b) Bone Marrow Biopsy:**<br>• **Reasoning:**<br>○ To assess for plasma cell dyscrasia or other hematological malignancies.<br>○ Essential in confirming POEMS syndrome or diagnosing multiple myeloma.<br>○ Helps in identifying any abnormal plasma cells or amyloid deposits.<br>**c) VEGF (Vascular Endothelial Growth Factor) Levels:**<br>• **Reasoning:**<br>○ Elevated VEGF levels are a diagnostic criterion for POEMS syndrome.<br>○ Helps to confirm the diagnosis in conjunction with other clinical and laboratory findings.<br>**d) Imaging Studies (CT or PET-CT scan of Chest, Abdomen, and Pelvis):**<br>• **Reasoning:**<br>○ To identify osteosclerotic lesions, lymphadenopathy, or organomegaly.<br>○ Useful in diagnosing POEMS syndrome as these features are often present.<br>○ Helps in ruling out other malignancies or metastatic diseases.<br>**e) Nerve Biopsy:**<br>• **Reasoning:**<br>○ To provide direct histological evidence of demyelination, inflammation, or amyloidosis.<br>○ Useful in confirming the type of neuropathy and differentiating between CIDP, POEMS, and other neuropathies.<br>**f) CSF Analysis:**<br>• **Reasoning:**<br>○ To check for elevated protein levels and absence of significant pleocytosis.<br>○ Helps in supporting the diagnosis of CIDP and ruling out infectious or malignant causes of neuropathy.<br>**g) Immunofixation Electrophoresis (IFE) of Serum and Urine:**<br>• **Reasoning:**<br>○ To further characterize the monoclonal protein.<br>○ Helps in confirming the type of paraproteinemia and evaluating for Bence Jones proteins in urine, indicating light chain disease.<br>**3. Most Crucial Test:**<br>**Bone Marrow Biopsy**<br>• **Reasoning:**<br>○ Bone marrow biopsy is crucial as it provides definitive evidence of plasma cell disorders.<br>○ It can confirm the presence of clonal plasma cells, amyloid deposits, and other marrow abnormalities.<br>○ Essential in diagnosing POEMS syndrome, multiple myeloma, and other plasma cell dyscrasias which are critical differential diagnoses in this patient.<br>○ Coupled with other tests, it solidifies the diagnosis and helps in planning appropriate treatment.<br>**Conclusion:**<br>The most crucial test from the list is bone marrow biopsy, as it directly assesses the presence and extent of plasma cell disorders, which are highly relevant given the patient's clinical presentation and lab findings, particularly the presence of IgG lambda paraprotein.
6	A 73-year-old female patient presents with an 8-month history of pure sensory, symmetrical, length dependent polyneuropathy with subacute onset and progressive course. The patient presents with distal paresthesia in the upper and lower limbs without weakness or neuropathic pain. Clinical examination reveals reduced tactile sensation in the feet. There are no tremor or ataxia, foot deformities, cranial nerve or autonomic involvement. Past medical history is positive for episodic diarrhea. Nerve conduction studies show a pure sensory,	Vit B12 deficiency neuropathy	**Step-by-Step Analysis of the Clinical Scenario**<br>**Patient Summary:**<br>• 73-year-old female<br>• 8-month history of pure sensory, symmetrical, length-dependent polyneuropathy with subacute onset and progressive course<br>• Distal paresthesia in upper and lower limbs without weakness or neuropathic pain<br>• Reduced tactile sensation in the feet<br>• Past medical history includes episodic diarrhea<br>• Nerve conduction studies indicate pure sensory, symmetric, length-dependent, axonal polyneuropathy<br>• Screening lab tests reveal vitamin B12 deficiency, hyperglycemia, and elevated HbA1c<br>• Negative tests: folate, vitamin B6, vitamin E, HCV, HBV, ANA, ENA, ANCA, CRP, ESR, copper, cryoglobulins, serum electrophoresis<br>• No family history of polyneuropathy<br>**Additional Examinations Needed**<br>1. **Oral Glucose Tolerance Test (OGTT)**

	symmetric, length dependent, axonal polyneuropathy. Screening lab tests reveal the presence of vitamin B12 deficiency, hyperglycaemia, and elevated HbA1c. Remaining exams including folate, vitamin B6, vitamin E, HCV, HBV, ANA, ENA, ANCA, CRP, ESR, copper, cryoglobulins and, serum electrophoresis are negative. There is no family history of polyneuropathy.		○ **Reasoning:** While hyperglycemia and elevated HbA1c are indicative of diabetes, an OGTT can help confirm diabetes or prediabetes by evaluating the body's response to glucose intake. This test is especially useful if fasting glucose levels are not conclusive.<br>2. **Serum Methylmalonic Acid (MMA) and Homocysteine Levels**<br>○ **Reasoning:** Elevated levels of MMA and homocysteine can confirm vitamin B12 deficiency, as these metabolites accumulate when B12 is deficient. This can help confirm if the neuropathy is due to B12 deficiency.<br>3. **Anti-transglutaminase Antibodies (tTG-IgA) and Total IgA Levels**<br>○ **Reasoning:** To rule out celiac disease as a cause of the episodic diarrhea and potential malabsorption, which can lead to secondary neuropathy. Total IgA is necessary to ensure accurate interpretation of tTG-IgA levels, as IgA deficiency can lead to false negatives.<br>4. **Electromyography (EMG)**<br>○ **Reasoning:** While nerve conduction studies provide information on the type and pattern of neuropathy, EMG can help further assess the extent of axonal damage and differentiate between axonal and demyelinating neuropathies.<br>5. **Lumbar Puncture (CSF Analysis)**<br>○ **Reasoning:** This can help identify any underlying inflammatory or infectious causes of the neuropathy, such as chronic inflammatory demyelinating polyneuropathy (CIDP) or other neuropathies with an inflammatory component.<br>6. **Gastrointestinal Evaluation (e.g., Endoscopy with Biopsy)**<br>○ **Reasoning:** Given the patient's history of episodic diarrhea and potential celiac disease, a gastrointestinal evaluation can help identify any structural or functional abnormalities, including celiac disease or other malabsorptive conditions.<br>7. **Repeat Vitamin B12, Folate, and Iron Studies**<br>○ **Reasoning:** Since vitamin deficiencies can fluctuate, repeating these tests can confirm persistent deficiencies and help assess if supplementation has been effective or if there is ongoing malabsorption.<br>8. **Thyroid Function Tests (TSH, Free T4)**<br>○ **Reasoning:** Hypothyroidism can also cause neuropathy and should be ruled out given the patient's age and clinical presentation.<br>9. **Paraneoplastic Panel (including anti-Hu, anti-Ri, anti-Yo antibodies)**<br>○ **Reasoning:** Considering the patient's age, a paraneoplastic syndrome should be considered, especially if there are no clear metabolic or nutritional causes identified.<br>**Most Crucial Test**<br>**Oral Glucose Tolerance Test (OGTT)**<br>● **Primary Reason:** Confirming the diagnosis of diabetes or prediabetes is crucial given the hyperglycemia and elevated HbA1c. Diabetes is a common cause of sensory polyneuropathy, and precise diagnosis and management of blood glucose levels can significantly impact the patient's neuropathy and overall health.<br>● **Impact on Management:** Establishing diabetes would direct treatment towards stringent glycemic control, potentially halting the progression of neuropathy and preventing further complications.<br>**Conclusion**<br>In conclusion, while several tests are necessary to comprehensively evaluate the differential diagnoses, the Oral Glucose Tolerance Test (OGTT) is the most crucial. Confirming or ruling out diabetes is essential given the patient's clinical presentation and lab findings, and it will significantly guide the management and therapeutic approach for this patient's polyneuropathy.
7	A 70-year-old male patient presents with a 5-week history of pure sensory, symmetrical, length dependent polyneuropathy with subacute onset and progressive course. The patient presents with distal paresthesia and hypoesthesia in the four limbs and sensory ataxia. Clinical examination does not show weakness, tremor, foot deformities, autonomic or cranial nerve involvement. Past medical history is positive for obesity treated with bariatric surgery, anemia, hypercholesterolemia, and type 2 diabetes mellitus. Nerve conduction studies show a pure sensory, symmetric, axonal polyneuropathy. Screening lab tests shows slightly elevated glycemia and HbA1c levels, and severe folate deficiency. Remaining exams including vitamin B12, E, HBV, HCV, ANA, ANCA, ENA, CRP, ESR, copper, cryoglobulins, and serum electrophoresis are normal. There is no family history of polyneuropathy.	Folate deficiency neuropathy	**Step-by-Step Analysis of Additional Examinations**<br>**Comprehensive Data Review**<br>● Patient: 70-year-old male<br>● Symptoms: 5-week history of pure sensory, symmetrical, length-dependent polyneuropathy, distal paresthesia and hypoesthesia, sensory ataxia<br>● Clinical Examination: No weakness, tremor, foot deformities, autonomic, or cranial nerve involvement<br>● Past Medical History: Obesity (treated with bariatric surgery), anemia, hypercholesterolemia, type 2 diabetes mellitus<br>● Nerve Conduction Studies: Pure sensory, symmetric, axonal polyneuropathy<br>● Lab Results: Slightly elevated glycemia and HbA1c levels, severe folate deficiency, normal levels of vitamin B12, vitamin E, HBV, HCV, ANA, ANCA, ENA, CRP, ESR, copper, cryoglobulins, and serum electrophoresis<br>● Family History: No family history of polyneuropathy<br>**Necessary Additional Examinations**<br>**1. Complete Blood Count (CBC) with Differential**<br>**Reasoning:**<br>● **Anemia Assessment:** Given the patient's history of anemia, a complete blood count can provide information on the type and severity of anemia, which might be linked to nutritional deficiencies.<br>● **Infection/Inflammation:** A differential can help identify any hidden infections or inflammatory processes that might contribute to neuropathy.<br>**2. Red Blood Cell Folate Levels**<br>**Reasoning:**<br>● **Folate Status Confirmation:** While serum folate levels indicate current folate status, red blood cell folate levels give a better indication of long-term folate stores, which can provide a more accurate assessment of folate deficiency and its potential contribution to neuropathy.<br>**3. Serum Homocysteine and Methylmalonic Acid (MMA) Levels**<br>**Reasoning:**<br>● Folate and B12 Metabolism: Elevated homocysteine levels can indicate folate or vitamin B12 deficiency, while elevated MMA levels are more specific for vitamin B12 deficiency. These tests can help differentiate between folate and B12 deficiencies and their contributions to neuropathy.<br>**4. Oral Glucose Tolerance Test (OGTT)**<br>**Reasoning:**<br>● **Glucose Control:** Despite slightly elevated glycemia and HbA1c, an OGTT can provide a more detailed assessment of glucose metabolism and control, which is crucial in diagnosing and managing diabetic neuropathy.<br>**5. Serum Protein Electrophoresis with Immunofixation**<br>**Reasoning:**<br>● **Paraproteinemia:** This test can detect monoclonal gammopathies (e.g., multiple myeloma, MGUS), which can present with neuropathy. Although initial serum electrophoresis was normal, immunofixation can be more sensitive for detecting monoclonal proteins.<br>**6. Lumbar Puncture (CSF Analysis)**<br>**Reasoning:**<br>● **Infectious or Inflammatory Causes:** Analysis of cerebrospinal fluid can help rule out infections, inflammatory conditions, or malignancies affecting the nervous system.

			**7. Skin Biopsy for Intraepidermal Nerve Fiber Density**<br>**Reasoning:**<br>● **Small Fiber Neuropathy:** This test can diagnose small fiber neuropathy, which may not be detected by standard nerve conduction studies but could explain the patient's sensory symptoms.<br>**8. Electromyography (EMG)**<br>**Reasoning:**<br>● **Muscle and Nerve Function:** EMG can provide additional information on the involvement of muscles and nerves, helping to distinguish between different types of polyneuropathies.<br>**Most Crucial Test**<br>The most crucial test from this list is Serum Homocysteine and Methylmalonic Acid (MMA) Levels.<br>**Reasoning:**<br>● **Differentiating Deficiencies:** This test is pivotal in differentiating between folate and vitamin B12 deficiencies, both of which can cause neuropathy and present with similar clinical features. Since the patient has a severe folate deficiency and normal B12 levels, confirming elevated homocysteine (indicative of folate deficiency) without elevated MMA (which would indicate B12 deficiency) will solidify the diagnosis of folate deficiency neuropathy.<br>● **Direct Impact on Treatment:** The results will directly influence the treatment strategy, confirming whether folate supplementation alone is sufficient or if additional investigation into B12 metabolism is required.
8	A 74-year-old male patient presents with a 9-month history of pure sensory, symmetrical, length dependent polyneuropathy with subacute onset while hospitalised for lymphoma. The patient developed neuropathic pain in the feet with no sensory deficits or weakness. Clinical examination reveals absence of ataxia, tremor, foot deformities, cranial nerve or autonomic involvement. Past medical history is positive for diabetes since the age of 43, and lymphoma for which the patient has undergone chemotherapy. Neuropathy symptoms started during chemotherapy. Nerve conduction studies show a sensory-motor, symmetric, axonal polyneuropathy. Screening lab tests including vitamin B12, folate, vitamin B6, vitamin E, HCV, ANA, ANCA, ENA, CRP, ESR, copper, cryoglobulins and serum electrophoresis are all negative. HbA1c and glycemia levels are elevated and there is evidence of chronic HBV infection. There is no family history of polyneuropathy.	Chemotherapy-induced neuropathy	**Step-by-Step Analysis**<br>**1. Review Clinical Data**<br>○ Patient: 74-year-old male<br>○ Symptoms: 9-month history of pure sensory, symmetrical, length-dependent polyneuropathy with subacute onset during chemotherapy for lymphoma.<br>○ Examination: No ataxia, tremor, foot deformities, cranial nerve or autonomic involvement.<br>○ History: Diabetes since age 43, lymphoma treated with chemotherapy.<br>○ Nerve Conduction Studies: Sensory-motor, symmetric, axonal polyneuropathy.<br>○ Laboratory Tests: Negative for vitamin deficiencies, infections (HCV), autoimmune markers, inflammatory markers, copper levels, cryoglobulins, and serum electrophoresis. Elevated HbA1c and glycemia. Chronic HBV infection.<br>○ Family History: No history of polyneuropathy.<br>**2. List of Necessary Additional Examinations**<br>○ **Electromyography (EMG)**<br>■ **Reasoning:** To assess the electrical activity of muscles and further differentiate between axonal and demyelinating neuropathies. EMG can provide detailed information about the severity and distribution of the neuropathy.<br>○ **Lumbar Puncture (CSF Analysis)**<br>■ **Reasoning:** To check for elevated protein levels without pleocytosis, which is indicative of CIDP. This can help to rule in or out an inflammatory cause of the neuropathy.<br>○ **Skin Biopsy**<br>■ **Reasoning:** To evaluate intraepidermal nerve fiber density, which can be reduced in small fiber neuropathy. This test can provide insight into the presence of small fiber involvement, which might not be detected in nerve conduction studies.<br>○ **Glucose Tolerance Test**<br>■ **Reasoning:** Although HbA1c and glycemia are elevated, a glucose tolerance test can provide a more detailed assessment of glucose metabolism and potential undiagnosed diabetic neuropathy.<br>○ **Repeat Nerve Conduction Studies**<br>■ **Reasoning:** To monitor the progression of neuropathy and distinguish between different patterns of nerve involvement over time. This can also help in assessing the response to any interventions.<br>○ **Serum Immunofixation Electrophoresis (IFE)**<br>■ **Reasoning:** To detect monoclonal gammopathies, which can be associated with neuropathies. Although initial serum electrophoresis was negative, IFE is more sensitive.<br>○ **Autoantibody Panel (including anti-MAG, anti-GM1, and others)**<br>■ **Reasoning:** To detect specific autoantibodies that might be associated with paraneoplastic syndromes or other autoimmune neuropathies. This is particularly relevant given the history of lymphoma.<br>○ **HBV DNA Quantification**<br>■ **Reasoning:** To assess the viral load of chronic HBV infection, as active HBV replication can sometimes be associated with neuropathies. This can also help guide potential treatment adjustments for HBV.<br>**3. Most Crucial Test**<br>○ **Lumbar Puncture (CSF Analysis)**<br>■ **Reasoning:** Given the possibility of CIDP or another inflammatory neuropathy, CSF analysis can provide critical diagnostic information. Elevated protein levels in the absence of significant white blood cells would support a diagnosis of CIDP, guiding specific immunotherapy treatment. This test is crucial because it can significantly narrow down the differential diagnosis and directly influence management and therapeutic decisions.<br>**Conclusion**<br>The most crucial test to be conducted in this clinical scenario is a lumbar puncture (CSF analysis). This test is pivotal in identifying potential inflammatory causes of the neuropathy, such as CIDP, which could significantly alter the patient's treatment plan and prognosis.
9	A 77-year-old female patient presents with a 3-week history of pure sensory, symmetrical, length dependent polyneuropathy with subacute onset and progressive course. The patient presents with hypoesthesia and mild neuropathic pain in the feet. Clinical examination reveals also mild sensory ataxia. There are no tremor, foot deformities, cranial nerve or autonomic involvement. Past medical history is negative. Nerve conduction studies show a pure sensory, length dependent, symmetric, axonal	B6 deficiency neuropathy	**Step-by-Step Analysis and Additional Examinations**<br>**Step 1: Review of Clinical Scenario**<br>● 77-year-old female with a 3-week history of pure sensory, symmetrical, length-dependent polyneuropathy<br>● Symptoms include hypoesthesia, mild neuropathic pain in the feet, and mild sensory ataxia<br>● Nerve conduction studies show pure sensory, length-dependent, symmetric, axonal polyneuropathy<br>● Lab tests reveal decreased serum vitamin B6 and slightly increased transaminase levels<br>● Other screening tests are negative, including vitamin B12, folate, HBV, HCV, glycemia, HbA1c, ANA, ANCA, ENA, CRP, ESR, copper, cryoglobulins, and serum electrophoresis<br>● No significant past medical history or family history of polyneuropathy<br>**Step 2: Identify Potential Gaps and Additional Examinations Needed**<br>Given the clinical scenario and initial test results, additional examinations are needed to further narrow down the differential diagnoses and rule out other potential causes of polyneuropathy.<br>**Proposed Additional Examinations:**<br>**1. Liver Function Tests (LFTs) - Detailed Panel**

	polyneuropathy. Screening lab tests reveal decreased serum level of vitamin B6 and a slight increase in transaminase levels (aspartate aminotransferase/alanine aminotransferase). Remaining exams including vitamin B12, folate, vitamin E, HBV, HCV, glycemia, HbA1c, ANA, ANCA, ENA, CRP, ESR, copper, cryoglobulins, and serum electrophoresis are negative. There is no family history of polyneuropathy.		○ **Reasoning:** The patient has slightly elevated transaminase levels. A comprehensive liver function panel (including bilirubin, alkaline phosphatase, gamma-glutamyl transferase, and albumin) can help to better assess liver function and rule out chronic liver disease as a contributing factor to the neuropathy.<br>○ **Specific Tests:** Total and direct bilirubin, alkaline phosphatase, gamma-glutamyl transferase (GGT), and albumin.<br>2. **Serum Protein Electrophoresis with Immunofixation (SPEP/IFE)**<br>○ **Reasoning:** While the initial serum electrophoresis was negative, performing an immunofixation can help detect low-level monoclonal proteins that might not be picked up on routine electrophoresis. This is particularly useful for identifying conditions such as monoclonal gammopathy of undetermined significance (MGUS) or multiple myeloma.<br>○ **Specific Tests:** Serum protein electrophoresis with immunofixation.<br>3. **Anti-Neuronal Antibodies (e.g., Anti-Hu, Anti-Ri, Anti-Yo)**<br>○ **Reasoning:** Some paraneoplastic syndromes can present with neuropathy. Testing for anti-neuronal antibodies can help identify if there is an underlying malignancy causing the neuropathy.<br>○ **Specific Tests:** Anti-Hu, Anti-Ri, Anti-Yo antibodies.<br>4. **Quantitative Sensory Testing (QST)**<br>○ **Reasoning:** QST can provide objective data on sensory nerve function, helping to further characterize the sensory deficits and guide diagnosis and management.<br>○ **Specific Tests:** Quantitative sensory testing for vibration, temperature, and pain thresholds.<br>5. **Heavy Metal Screen**<br>○ **Reasoning:** Exposure to heavy metals like lead, arsenic, or mercury can cause sensory neuropathy. Although there is no specific exposure history provided, this test can rule out this potential cause.<br>○ **Specific Tests:** Blood and urine levels of lead, arsenic, and mercury.<br>6. **Autonomic Function Tests**<br>○ **Reasoning:** Even though there are no signs of autonomic involvement reported, subtle autonomic dysfunction can sometimes be present in peripheral neuropathies. Autonomic function testing can help rule this out.<br>○ **Specific Tests:** Heart rate variability, tilt-table test, and sudomotor function tests.<br>7. **Lumbar Puncture with CSF Analysis**<br>○ **Reasoning:** Inflammatory or infectious causes of neuropathy may be identified through cerebrospinal fluid (CSF) analysis. This can help in detecting elevated protein levels, pleocytosis, or the presence of specific pathogens.<br>○ **Specific Tests:** CSF analysis for cell count, protein, glucose, and culture.<br>8. **Genetic Testing for Hereditary Neuropathies**<br>○ **Reasoning:** Although there is no family history of polyneuropathy, some hereditary neuropathies can present later in life. Genetic testing can help identify mutations associated with hereditary sensory and autonomic neuropathies (HSAN) or Charcot-Marie-Tooth disease.<br>○ **Specific Tests:** Genetic panel for hereditary neuropathies.<br>**Step 3: Determine the Most Crucial Test**<br>**Most Crucial Test: Serum Protein Electrophoresis with Immunofixation (SPEP/IFE)**<br>**Reasoning:**<br>● Despite the initial serum electrophoresis being negative, immunofixation can detect low-level monoclonal proteins that might not be visible on standard electrophoresis. Detecting a monoclonal protein would significantly alter the differential diagnosis and management plan, pointing towards conditions like MGUS or multiple myeloma, which are crucial to identify and treat early.<br>● The presence of an underlying monoclonal gammopathy would directly impact the diagnosis and could explain the neuropathy, making this test highly relevant and potentially diagnostic.<br>**Conclusion**<br>Based on the comprehensive review of the patient's clinical scenario and initial test results, the most crucial additional test is Serum Protein Electrophoresis with Immunofixation (SPEP/IFE). This test can provide critical information that may reveal an underlying condition responsible for the polyneuropathy, guiding further management and treatment.
10	A 76-year-old female patient presents with a 2-year history of sensory-motor, symmetrical, length dependent polyneuropathy with subacute onset. The patient presents with paresthesia, neuropathic pain, cramps, orthostatic hypotension, and weakness of toe dorsiflexion. There are no foot deformities, cranial nerve involvement, ataxia, or tremor. Past medical history is positive for chronic renal insufficiency and multiple myeloma, for which the patient has undergone chemotherapy. Neuropathy symptoms started at the end of the chemotherapy treatment cycles. Nerve conduction studies show a symmetric, pure sensory, axonal polyneuropathy and unilateral carpal tunnel syndrome. Screening lab tests including vitamin B12, folate, vitamin B6, vitamin E, HBV, HCV, ANA, ANCA, ENA, CRP, ESR, glycemia, HbA1c, copper and cryoglobulins are all negative, with positivity for IgG kappa paraprotein. There is no family history of polyneuropathy.	Chemotherapy-induced neuropathy	**Step-by-Step Analysis and Additional Examinations:**<br>1. **Re-evaluation of Current Data:**<br>○ Clinical Symptoms: Sensory-motor, symmetrical, length-dependent polyneuropathy with paresthesia, neuropathic pain, cramps, orthostatic hypotension, and weakness of toe dorsiflexion.<br>○ Medical History: Chronic renal insufficiency, multiple myeloma with chemotherapy, and presence of IgG kappa paraprotein.<br>○ Nerve Conduction Studies: Symmetric, pure sensory, axonal polyneuropathy and unilateral carpal tunnel syndrome.<br>○ Negative Lab Tests: Extensive negative screening including vitamin deficiencies, viral infections, autoimmune markers, metabolic markers, and cryoglobulins.<br>2. **List of Necessary Additional Examinations:**<br>**1. Serum Protein Electrophoresis (SPEP) and Immunofixation Electrophoresis (IFE)**<br>● **Reasoning:** To further evaluate the significance of the IgG kappa paraprotein and assess for the presence of a monoclonal gammopathy (e.g., multiple myeloma, MGUS, or other plasma cell dyscrasias). This will help determine if the paraprotein is contributing to the neuropathy.<br>**2. Skin Biopsy**<br>● **Reasoning:** To evaluate for small fiber neuropathy, which might not be evident in nerve conduction studies. This can provide information on intraepidermal nerve fiber density and help detect small fiber involvement, which is common in chemotherapy-induced neuropathy.<br>**3. Electromyography (EMG)**<br>● **Reasoning:** To complement nerve conduction studies and further assess the extent and type of neuromuscular involvement. This can help distinguish between axonal and demyelinating processes and provide more detail on motor involvement.<br>**4. Autonomic Function Tests**<br>● **Reasoning:** To assess the extent of autonomic dysfunction, given the patient's symptoms of orthostatic hypotension. Tests may include heart rate variability, quantitative sudomotor axon reflex test (QSART), and tilt-table testing.<br>**5. Repeat Nerve Conduction Studies**<br>● **Reasoning:** To monitor progression and re-evaluate the pattern of neuropathy over time. This can help identify any new developments or changes in the neuropathy pattern, particularly after cessation of chemotherapy.<br>**6. Renal Function Tests (including electrolytes)**<br>● **Reasoning:** To monitor the progression of chronic renal insufficiency and assess for any electrolyte imbalances that might contribute to neuropathy symptoms.<br>**7. Bone Marrow Biopsy and Aspirate**<br>● **Reasoning:** Given the history of multiple myeloma and the presence of IgG kappa paraprotein, this can help assess the current status of the disease and rule out progression or relapse.<br>**8. Heavy Metal Screening**<br>● **Reasoning:** To exclude heavy metal poisoning (e.g., lead, mercury, arsenic) as a potential cause of neuropathy, which can present similarly.<br>**Most Crucial Test:**

			The most crucial test from the list is Serum Protein Electrophoresis (SPEP) and Immunofixation Electrophoresis (IFE). This test is essential to further evaluate the presence and significance of the IgG kappa paraprotein found in the patient. Understanding whether this paraprotein is associated with a plasma cell disorder (such as multiple myeloma, MGUS, or other related conditions) is critical in narrowing down the differential diagnoses and guiding further management. This test directly relates to both the patient's medical history of multiple myeloma and the neuropathy, making it the most pivotal examination to conduct next.
11	A 76-year-old female patient presents with a 1-year history of sensory-motor multineuropathy with subacute onset. The patient presents with paresthesia and weakness in a multifocal distribution, without ataxia, neuropathic pain, tremor, foot deformities, cranial nerve or autonomic involvement. Past medical history shows diabetes, anaemia, chronic renal insufficiency, undifferentiated connective tissue disease, osteoarthritis and ANCA vasculitis. Nerve conduction studies show a sensory-motor, axonal multineuropathy. Screening lab tests including vitamin B12, folate, vitamin B6, vitamin E, HCV, HBV, ENA, CRP, ESR, glycemia, HbA1c, copper, cryoglobulins and serum electrophoresis are all negative, with positivity for ANA and ANCA autoantibodies. There is no family history of polyneuropathy.	Vasculitic neuropathy	**Step-by-Step Approach to Additional Examinations:**<br>**1. Detailed Review of Existing Data:**<br>● **Current Findings:**<br>○ Sensory-motor multineuropathy with subacute onset.<br>○ Paresthesia and weakness in a multifocal distribution.<br>○ Absence of ataxia, neuropathic pain, tremor, foot deformities, cranial nerve, or autonomic involvement.<br>○ History of diabetes, anaemia, chronic renal insufficiency, undifferentiated connective tissue disease, osteoarthritis, and ANCA vasculitis.<br>○ Nerve conduction studies showing sensory-motor, axonal multineuropathy.<br>○ Positive ANA and ANCA autoantibodies.<br>**2. Additional Examinations:**<br>**A. Laboratory Tests:**<br>**1. Complete Blood Count (CBC) with Differential:**<br>○ **Reasoning:** To check for signs of infection, inflammation, or hematologic abnormalities that could be contributing to the neuropathy.<br>**2. Erythrocyte Sedimentation Rate (ESR) and C-Reactive Protein (CRP):**<br>○ **Reasoning:** Even though initial screening was negative, repeat testing can help assess ongoing inflammation which might suggest vasculitic activity.<br>**3. Renal Function Tests (BUN, Creatinine, eGFR):**<br>○ **Reasoning:** To monitor the extent of chronic renal insufficiency and its possible contribution to neuropathy.<br>**4. Immunoglobulin Levels (IgG, IgA, IgM):**<br>○ **Reasoning:** To identify any underlying immune deficiency or gammopathy that could be associated with neuropathy.<br>**5. ANCA Subtyping (c-ANCA and p-ANCA):**<br>○ **Reasoning:** To determine the specific type of ANCA vasculitis, which can guide diagnosis and management.<br>**6. Anti-Ro/SSA and Anti-La/SSB Antibodies:**<br>○ **Reasoning:** Given the undifferentiated connective tissue disease, these tests can help identify specific autoimmune conditions like Sjögren's syndrome.<br>**7. Liver Function Tests (LFTs):**<br>○ **Reasoning:** To assess hepatic function and rule out liver-related causes of neuropathy.<br>**8. Serum Protein Electrophoresis (SPEP) and Immunofixation Electrophoresis (IFE):**<br>○ **Reasoning:** To detect monoclonal gammopathy which can be associated with conditions like multiple myeloma or amyloidosis, contributing to neuropathy.<br>**B. Instrumental Tests:**<br>**1. Electromyography (EMG):**<br>○ **Reasoning:** To further evaluate the electrical activity of muscles and confirm the type of neuropathy (axonal vs demyelinating).<br>**2. Skin Biopsy:**<br>○ **Reasoning:** To assess for small fiber neuropathy, which may not be detected on standard nerve conduction studies.<br>**3. Magnetic Resonance Imaging (MRI) of the Spine and Brain:**<br>○ **Reasoning:** To rule out any central nervous system pathology or spinal cord involvement that might mimic peripheral neuropathy.<br>**4. Nerve Biopsy:**<br>○ **Reasoning:** In cases where vasculitis is strongly suspected, a nerve biopsy can provide definitive histological evidence of vasculitis.<br>**5. Autonomic Testing (if autonomic symptoms develop):**<br>○ **Reasoning:** To evaluate the involvement of the autonomic nervous system, which might become relevant if symptoms progress.<br>**Most Crucial Test:**<br>**Nerve Biopsy**<br>**Reasoning:**<br>● Definitive Diagnosis: A nerve biopsy can provide histopathological confirmation of vasculitic neuropathy, distinguishing it from other causes such as diabetic neuropathy or connective tissue disease-related neuropathy.<br>● Targeted Treatment: Confirming the presence of vasculitis can guide specific immunosuppressive therapy, crucial for patient management.<br>● Rule Out Other Causes: It can help rule out other conditions like amyloidosis or other inflammatory neuropathies.<br>**Conclusion:**<br>Based on the comprehensive analysis, a nerve biopsy stands out as the most crucial test. It provides definitive histopathological evidence, which is essential for accurate diagnosis and effective management of the patient's condition.
12	An 18-year old male patient presents with a 16-year history of sensory-motor, symmetrical, length dependent polyneuropathy with insidious onset and slow progression. The patient presents with sensory ataxia, distal weakness in the lower limbs, severe calf atrophy, pes planus, and hammertoes with no cranial nerve or autonomic involvement. Past medical history is silent. Nerve conduction studies show an axonal, sensory-motor polyneuropathy. Screening lab tests including vitamin B12, folate, vitamin B6, vitamin E, HBV, HCV, glycemia, HbA1c,	CMT2	**Step-by-Step Approach to Additional Examinations:**<br>**1. Genetic Testing for Charcot-Marie-Tooth Disease (CMT)**<br>● **Reasoning:** Given the strong suspicion of CMT based on clinical presentation, family history, and nerve conduction studies, genetic testing can confirm the diagnosis. Testing for common mutations associated with CMT, such as PMP22 duplication (CMT1A) or mutations in the MFN2 gene (CMT2A), can provide definitive evidence.<br>**2. Electromyography (EMG)**<br>● **Reasoning:** EMG can complement nerve conduction studies by providing detailed information about the electrical activity of muscles. This can help differentiate between neuropathic and myopathic processes and assess the severity and distribution of the neuropathy.<br>**3. Magnetic Resonance Imaging (MRI) of the Lower Limbs**<br>● **Reasoning:** MRI can visualize the extent of muscle atrophy and fatty replacement in the lower limbs, providing additional evidence of chronic neuropathy. It can also rule out other structural causes of the patient's symptoms.<br>**4. Lumbar Puncture for Cerebrospinal Fluid (CSF) Analysis**<br>● **Reasoning:** While less likely given the current evidence, CSF analysis can rule out inflammatory or infectious causes of neuropathy (e.g., chronic inflammatory demyelinating polyneuropathy (CIDP), Guillain-Barré syndrome). Elevated protein levels without pleocytosis can indicate CIDP.<br>**5. Skin Biopsy**

	ANA, ANCA, ENA, CRP, ESR, copper, cryoglobulins, and serum electrophoresis are all negative. Her mother and grandmother have walking difficulties. Her 5-year-old sister has pes cavus.		• **Reasoning:** A skin biopsy with intraepidermal nerve fiber density (IENFD) analysis can help assess small fiber involvement, which might not be apparent in standard nerve conduction studies. This can be useful in cases of hereditary sensory neuropathies.<br>**6. Ophthalmologic Examination**<br>• **Reasoning:** An ophthalmologic examination, including fundoscopy, can detect retinal changes associated with certain hereditary neuropathies, such as optic atrophy seen in some CMT types or Friedreich's ataxia.<br>**7. Echocardiogram and Cardiac Evaluation**<br>• **Reasoning:** Although the patient does not report cardiac symptoms, some hereditary neuropathies, particularly Friedreich's ataxia, are associated with cardiomyopathy. An echocardiogram can help rule out subclinical cardiac involvement.<br>**Most Crucial Test:**<br>**Genetic Testing for Charcot-Marie-Tooth Disease (CMT)**<br>• **Reasoning:** Genetic testing is the most crucial test because it can definitively confirm or rule out CMT, the leading diagnosis based on clinical presentation and family history. Identifying specific genetic mutations associated with CMT will provide a clear diagnosis, guide management, and inform family planning and genetic counseling.
13	An 82-year-old female patient presents with a 1-year history of sensory-motor, symmetrical, length dependent polyneuropathy with subacute onset and progressive course. The patient presents with paraesthesia and neuropathic pain in the hands and feet and bilateral foot drop. Clinical examination excludes sensory ataxia, cranial nerve or autonomic involvement, tremor or foot deformities. Past medical history is negative. Nerve conduction studies show a demyelinating, sensory-motor, symmetric polyneuropathy. Screening lab tests reveal presence of polycythaemia, IgG lambda paraprotein, positivity for ANA, hyperglycemia, and elevated HbA1c. Remaining exams including vitamin B12, folate, vitamin B6, vitamin E, ANCA, CRP, ESR, copper, and cryoglobulins are negative. There is no family history of polyneuropathy.	POEMS	**Step-by-Step Approach to Additional Examinations**<br>**1. Detailed Patient History and Clinical Examination**<br>• **Reasoning:** To ensure that no subtle signs or symptoms have been overlooked that could influence the differential diagnosis. Detailed questioning about the onset, duration, and progression of symptoms as well as any potential exposures, infections, or medications is crucial.<br>**2. Serum Protein Electrophoresis (SPEP) and Immunofixation Electrophoresis (IFE)**<br>• **Reasoning:** Given the presence of IgG lambda paraprotein, further characterization of the paraprotein is essential. These tests help confirm and detail monoclonal gammopathies, which could point towards MGUS, multiple myeloma, or other plasma cell disorders.<br>**3. Lumbar Puncture with Cerebrospinal Fluid (CSF) Analysis**<br>• **Reasoning:** To assess for elevated protein levels without pleocytosis, which is indicative of CIDP. This test can help differentiate between inflammatory and other causes of polyneuropathy.<br>**4. Hemoglobin A1c (HbA1c) and Fasting Glucose Reassessment**<br>• **Reasoning:** Although initial tests indicate hyperglycemia and elevated HbA1c, reassessment helps confirm persistent hyperglycemia and the diagnosis of diabetes, ensuring accurate interpretation of diabetic neuropathy.<br>**5. Serum Free Light Chain Assay**<br>• **Reasoning:** This test quantifies free light chains in the blood, providing further information on the extent and type of monoclonal gammopathy, assisting in differentiating between MGUS and more malignant conditions.<br>**6. Autoimmune Panel (including anti-Ro, anti-La, anti-dsDNA, anti-Smith, and complement levels)**<br>• **Reasoning:** Given the positive ANA, further autoimmune workup is warranted to exclude systemic autoimmune diseases that can present with neuropathy, such as lupus or Sjogren's syndrome.<br>**7. MRI of the Spine with Contrast**<br>• **Reasoning:** To look for any evidence of nerve root or spinal cord involvement, which could be seen in CIDP and other demyelinating conditions. It can also rule out compressive causes of neuropathy.<br>**8. Nerve Biopsy**<br>• **Reasoning:** If other tests remain inconclusive, a nerve biopsy can provide direct evidence of the type of nerve pathology present, such as inflammation, demyelination, or amyloid deposition.<br>**Most Crucial Test**<br>The most crucial test in this scenario is Serum Protein Electrophoresis (SPEP) and Immunofixation Electrophoresis (IFE).<br>**Reasoning:**<br>• Presence of IgG Lambda Paraprotein: The detection of a monoclonal paraprotein is a significant finding that needs further characterization. SPEP and IFE are essential to confirm the presence and type of paraprotein, which helps in diagnosing or excluding conditions like MGUS, multiple myeloma, or other related disorders.<br>• Impact on Management: Identifying the nature of the paraprotein can drastically change the management and prognosis. For instance, MGUS requires monitoring, while multiple myeloma needs more aggressive treatment.<br>• Diagnostic Clarity: This test provides clarity on whether the neuropathy is related to a hematologic malignancy or a benign monoclonal gammopathy, thereby significantly narrowing down the differential diagnoses.<br>By conducting SPEP and IFE, the clinical team can better determine the relevance of the IgG lambda paraprotein in relation to the patient's neuropathy, guiding subsequent investigations and management.
14	A 22-year old male patient presents with a 20-year history of chronic, sensory-motor, symmetrical, length dependent polyneuropathy with insidious onset and slow progression. The patient presents with severe distal weakness and atrophy in the four limbs, hypoesthesia up to calves, pes planus, and scoliosis with no cranial nerve or autonomic involvement. He uses a rollator for walking. Past medical history is silent. Nerve conduction studies show demyelinating, symmetric, sensory-motor polyneuropathy. Screening lab tests including vitamin B12, folate, vitamin B6, vitamin E, HBV, HCV, glycemia, HbA1c, ANA, ANCA, ENA, CRP, ESR, copper, cryoglobulins, and serum electrophoresis are all negative. There is no family history of polyneuropathy.	CMT4	**Step-by-Step Analysis of Additional Examinations**<br>**1. Genetic Testing for Charcot-Marie-Tooth Disease:**<br>• **Reasoning:** Given the clinical presentation and the nerve conduction study findings, genetic testing for mutations in the PMP22 gene (commonly associated with CMT1A) and other genes linked to CMT is crucial. Despite the absence of a family history, de novo mutations can occur.<br>• **Specifics:** Sequencing and multiplex ligation-dependent probe amplification (MLPA) can detect common duplications or deletions in the PMP22 gene and other relevant genetic mutations.<br>**2. Lumbar Puncture for Cerebrospinal Fluid (CSF) Analysis:**<br>• **Reasoning:** To rule out chronic inflammatory demyelinating polyneuropathy (CIDP), a CSF analysis should be performed. Elevated protein levels with a normal cell count (albuminocytologic dissociation) are indicative of CIDP.<br>• **Specifics:** Measure CSF protein, cell count, glucose, and perform cytological analysis.<br>**3. Nerve Biopsy:**<br>• **Reasoning:** A nerve biopsy can provide histopathological evidence to distinguish between hereditary neuropathies and acquired demyelinating neuropathies like CIDP. Typical findings in CMT include onion bulb formations and demyelination.<br>• **Specifics:** Sural nerve biopsy is typically performed, and the sample is analyzed for signs of demyelination, remyelination, and axonal loss.<br>**4. Electromyography (EMG):**<br>• **Reasoning:** EMG can complement nerve conduction studies by assessing muscle electrical activity, helping to differentiate between neuropathic and myopathic processes and to evaluate the extent of nerve and muscle involvement.<br>• **Specifics:** Perform EMG on the affected limbs to assess for denervation changes and to correlate with the nerve conduction study findings.<br>**5. MRI of the Spine:**<br>• **Reasoning:** To evaluate for structural abnormalities that might contribute to scoliosis and to exclude compressive lesions or other spinal pathologies that could mimic or contribute to neuropathy.<br>• Specifics: MRI of the cervical, thoracic, and lumbar spine with and without contrast.<br>**6. Skin Biopsy for Intraepidermal Nerve Fiber Density (IENFD):**<br>• **Reasoning:** A skin biopsy can assess small nerve fiber involvement, which might be under-represented in standard nerve conduction studies. This can help in distinguishing between large and small fiber neuropathy.<br>• **Specifics:** Skin punch biopsy from the distal leg and thigh, followed by immunostaining for nerve fibers.<br>**7. Quantitative Sensory Testing (QST):**<br>• **Reasoning:** QST can objectively quantify sensory deficits, providing a comprehensive evaluation of sensory nerve function, particularly for small fiber involvement.

			● **Specifics:** Thermal and vibratory threshold testing on the distal limbs.<br>**Determining the Most Crucial Test**<br>Among the listed examinations, Genetic Testing for Charcot-Marie-Tooth Disease is the most crucial. Given the long-standing nature of the polyneuropathy, the specific pattern of demyelination, and the absence of systemic or autonomic involvement, confirming or excluding a genetic etiology such as CMT will significantly narrow the differential diagnosis and guide further management. This test directly addresses the most probable diagnosis and can provide a definitive answer regarding the hereditary nature of the neuropathy.
15	A 72-year-old female patient presents with a 2-year history of pure sensory, symmetrical, length dependent polyneuropathy with insidious onset. The patient presents with distal paresthesia and neuropathic pain in the upper and lower limbs in a stocking and glove distribution, without ataxia or tremor, foot deformities, cranial nerve or autonomic involvement. Past medical history shows multiple myeloma, for which the patient has undergone chemotherapy. Neuropathy symptoms started 2 months after chemotherapy. Nerve conduction studies show pure sensory, symmetric, axonal polyneuropathy. Screening lab tests including vitamin B12, folate, vitamin B6, vitamin E, HBV, HCV, ANA, ANCA, ENA, CRP, ESR, glycemia, HbA1c, copper and cryoglobulins are all negative, with positivity for IgG kappa paraprotein. There is no family history of polyneuropathy.	Chemotherapy-induced neuropathy	**Step-by-Step Analysis for Additional Examinations:**<br>**1. Comprehensive Blood Work:**<br>○ **Reasoning:** Although initial screening lab tests are negative, additional specific tests can provide more information on potential underlying causes or contributing factors.<br>○ **Tests:**<br>■ Serum Protein Electrophoresis (SPEP) and Immunofixation: To further characterize and quantify the monoclonal protein and determine if there's a significant paraproteinemia that could contribute to neuropathy.<br>■ Free Light Chain Assay: To detect free light chains which can be elevated in multiple myeloma and related disorders.<br>■ Anti-Hu and Anti-MAG antibodies: To identify possible paraneoplastic or autoimmune neuropathies.<br>■ Heavy Metal Screen (Lead, Arsenic, Mercury): Chronic exposure can cause peripheral neuropathy and may not be covered by standard lab panels.<br>**2. Lumbar Puncture (CSF Analysis):**<br>○ **Reasoning:** To evaluate for any inflammatory or infectious process that might not be evident in peripheral blood tests.<br>○ **Tests:**<br>■ Cell Count and Differential: To check for inflammatory cells.<br>■ Protein and Glucose Levels: Elevated protein levels can indicate a breakdown of the blood-nerve barrier, common in inflammatory neuropathies.<br>■ Oligoclonal Bands: Presence might suggest an autoimmune process.<br>**3. Skin Biopsy:**<br>○ **Reasoning:** To evaluate small fiber neuropathy which can be missed on nerve conduction studies that typically assess large fibers.<br>○ **Procedure:**<br>■ Epidermal Nerve Fiber Density (ENFD) Measurement: Reduced density would confirm small fiber neuropathy.<br>**4. MRI of the Brain and Spinal Cord:**<br>○ **Reasoning:** To rule out central nervous system involvement or structural abnormalities that might be contributing to the neuropathy.<br>○ **Areas:**<br>■ Brain: To rule out any paraneoplastic or demyelinating disease.<br>■ Spinal Cord: To check for any compressive lesions or other abnormalities.<br>**5. Nerve Biopsy:**<br>○ **Reasoning:** To get a direct examination of the nerve pathology. This can be particularly useful if the diagnosis remains unclear after non-invasive tests.<br>○ **Procedure:**<br>■ Sural Nerve Biopsy: Examined for evidence of axonal degeneration, demyelination, and potential infiltration indicative of paraneoplastic syndromes.<br>**6. Repeat Nerve Conduction Studies/EMG:**<br>○ **Reasoning:** To assess the progression of the neuropathy and gather more detailed data on the nature of the nerve damage.<br>○ **Procedure:**<br>■ Electromyography (EMG): Can help differentiate between axonal and demyelinating processes and identify any subtle motor involvement not evident clinically.<br>**Most Crucial Test:**<br>Serum Protein Electrophoresis (SPEP) and Immunofixation are the most crucial tests. Given the patient's history of multiple myeloma and the presence of IgG kappa paraprotein, further characterization of the monoclonal protein is essential. This test will help determine the significance of the paraprotein and whether it might be contributing to the neuropathy through mechanisms such as MGUS-related neuropathy or progression/relapse of multiple myeloma.<br>**Conclusion:**<br>By performing these additional examinations, we can narrow down the differential diagnoses, specifically focusing on the distinction between chemotherapy-induced neuropathy, paraneoplastic syndrome, and MGUS-related neuropathy. The comprehensive blood work, especially SPEP and immunofixation, will provide crucial insights into the monoclonal protein and its potential role in the patient's neuropathy.
16	A 59-year-old female patient presents with a 1-year history of pure sensory, symmetrical, non-length dependent polyneuropathy with insidious onset. The patient presents with paresthesia distributed proximally and distally in the upper and lower limbs in an asymmetric fashion, and sensory ataxia. There are no foot deformities, tremor, neuropathic pain, cranial nerve or autonomic involvement. Past medical history is positive for IgG lambda monoclonal gammopathy and lung cancer, for which she underwent chemotherapy 4 years ago. Nerve conduction studies show a demyelinating, non-length dependent, sensory-motor polyneuropathy with conduction blocks. Screening lab tests including vitamin B12, folate, vitamin B6, vitamin E, HCV, ANA, ANCA, ENA, CRP, ESR, glycemia, HbA1c,	CIDP	**Step-by-Step Analysis and Additional Examinations**<br>**Step 1: Reassess Initial Findings**<br>**1. Clinical Presentation:**<br>○ **Pure sensory, symmetrical, non-length dependent polyneuropathy**<br>○ **Paresthesia proximally and distally in an asymmetric fashion**<br>○ **Sensory ataxia**<br>○ **No foot deformities, tremor, neuropathic pain, cranial nerve or autonomic involvement**<br>**2. Past Medical History:**<br>○ **IgG lambda monoclonal gammopathy**<br>○ **Lung cancer treated with chemotherapy 4 years ago**<br>**3. Nerve Conduction Studies:**<br>○ **Demyelinating, non-length dependent, sensory-motor polyneuropathy with conduction blocks**<br>**4. Laboratory Findings:**<br>○ **Positive for IgG lambda paraprotein**<br>○ **Chronic HBV infection**<br>○ **Negative for vitamin B12, folate, vitamin B6, vitamin E, HCV, ANA, ANCA, ENA, CRP, ESR, glycemia, HbA1c, copper, cryoglobulins**<br>**5. Family History:**<br>○ **No history of polyneuropathy**<br>**Step 2: List Additional Examinations**<br>**1. Serum Immunofixation Electrophoresis (IFE) and Free Light Chain (FLC) Assay:**

	copper and cryoglobulins are all negative, with positivity for IgG lambda paraprotein and evidence of chronic HBV infection. There is no family history of polyneuropathy.		○ **Reasoning: To further characterize the monoclonal gammopathy. IFE can identify the exact type of monoclonal protein, and FLC assay can detect free light chains in the serum, providing more details on the monoclonal gammopathy.**<br>○ **Clinical Relevance: This helps confirm MGUS and rule out multiple myeloma or other plasma cell disorders.**<br>**2. Cerebrospinal Fluid (CSF) Analysis:**<br>○ **Reasoning: To assess for albuminocytologic dissociation and exclude infections or inflammatory conditions affecting the nervous system.**<br>○ **Clinical Relevance: Albuminocytologic dissociation is common in CIDP. Presence of abnormal cells or infectious agents would suggest other etiologies.**<br>**3. Paraneoplastic Antibody Panel:**<br>○ **Reasoning: To detect antibodies associated with paraneoplastic syndromes.**<br>○ **Clinical Relevance: Given the history of lung cancer, this can help identify if the neuropathy is paraneoplastic in nature.**<br>**4. Repeat Nerve Conduction Studies and Electromyography (EMG):**<br>○ **Reasoning: To further characterize the pattern and progression of neuropathy, and to assess muscle involvement.**<br>○ **Clinical Relevance: This can provide additional information to distinguish between CIDP, MGUS-related neuropathy, and other causes.**<br>**5. Serum Hepatitis B Viral Load and Liver Function Tests:**<br>○ **Reasoning: To assess the activity of chronic HBV infection and liver function.**<br>○ **Clinical Relevance: Active HBV infection could contribute to neuropathy through a paraneoplastic-like mechanism or through systemic effects.**<br>**6. Positron Emission Tomography (PET) Scan:**<br>○ **Reasoning: To check for recurrence of lung cancer or presence of other malignancies.**<br>○ **Clinical Relevance: If paraneoplastic syndrome is suspected, PET scan can help detect occult tumors.**<br>**7. Autoimmune Panel Including Antiganglioside Antibodies (e.g., Anti-GM1, Anti-GQ1b):**<br>○ **Reasoning: To detect autoimmune neuropathies.**<br>○ **Clinical Relevance: These antibodies are associated with specific types of neuropathy (e.g., multifocal motor neuropathy, Miller Fisher syndrome) that may present with similar features.**<br>**8. Bone Marrow Biopsy:**<br>○ **Reasoning: To evaluate for multiple myeloma or other hematologic malignancies.**<br>○ **Clinical Relevance: Especially important if serum IFE and FLC assay are suggestive of a more significant plasma cell disorder.**<br>**Step 3: Determining the Most Crucial Test**<br>**Most Crucial Test: Serum Immunofixation Electrophoresis (IFE) and Free Light Chain (FLC) Assay**<br>● **Reasoning: This test is critical to further characterize the monoclonal gammopathy identified in the initial workup. Confirming the type and nature of the monoclonal protein can significantly narrow down the differential diagnosis, particularly between MGUS, multiple myeloma, and other plasma cell dyscrasias. Accurate identification of the monoclonal protein type will help determine if the polyneuropathy is related to MGUS or if it points towards a more serious underlying hematologic condition that might require more aggressive treatment.**
17	A 73-year-old female patient presents with a 1-year history of sensory-motor, symmetrical, polyneuropathy with subacute onset. The patient presents with distal paresthesia and neuropathic pain in the four limbs, sensory ataxia, cramps, orthostatic hypotension and diarrhoea but with no cranial nerve involvement or foot deformities. Clinical examination shows cutaneous angiomas and hyperpigmentation. Laboratory tests reveal presence of thrombocytosis and IgG lambda monoclonal gammopathy. Nerve conduction studies show a symmetric, sensory-motor, demyelinating-axonal polyneuropathy. Screening lab tests including vitamin B12, folate, vitamin B6, vitamin E, HCV, HBV, ANA, ANCA, ENA, CRP, ESR, glycemia, HbA1c, copper and cryoglobulins are negative, with positivity for IgG lambda paraprotein. There is no family history of polyneuropathy.	POEMS	**Step-by-Step Analysis of Additional Examinations**<br>**1. Serum and Urine Protein Electrophoresis with Immunofixation**<br>● **Reasoning:**<br>○ **To confirm the presence and type of monoclonal gammopathy (IgG lambda in this case).**<br>○ **Helps in distinguishing between MGUS, multiple myeloma, and AL amyloidosis.**<br>○ **Immunofixation can further classify the monoclonal protein and assess the monoclonal protein's burden.**<br>**2. Bone Marrow Biopsy and Aspirate**<br>● **Reasoning:**<br>○ **Essential for assessing plasma cell disorders such as multiple myeloma or AL amyloidosis.**<br>○ **Provides information on plasma cell infiltration and helps rule out malignancies like multiple myeloma.**<br>○ **Bone marrow findings can differentiate between MGUS and more severe plasma cell dyscrasias.**<br>**3. Fat Pad Biopsy or Rectal Biopsy with Congo Red Staining**<br>● **Reasoning:**<br>○ **Used to detect amyloid deposits, which are indicative of AL amyloidosis.**<br>○ **Congo red staining of the biopsy specimen, with examination under polarized light, can confirm amyloid presence.**<br>○ **Less invasive than other biopsy sites and highly sensitive for systemic amyloidosis.**<br>**4. Autonomic Function Tests**<br>● **Reasoning:**<br>○ **To evaluate the extent of autonomic dysfunction, including orthostatic hypotension and gastrointestinal symptoms.**<br>○ **Tests such as tilt-table testing, heart rate variability, and QSART (quantitative sudomotor axon reflex test) can provide comprehensive autonomic evaluation.**<br>○ **Helps in characterizing the autonomic involvement, which can be significant in AL amyloidosis and CIDP.**<br>**5. Skin Biopsy**<br>● **Reasoning:**<br>○ **Can be used to assess small fiber neuropathy, which is often missed on nerve conduction studies.**<br>○ **Immunohistochemistry can detect amyloid deposits or other pathologies affecting small nerve fibers.**<br>○ **Supports diagnosis in cases where cutaneous symptoms such as angiomas and hyperpigmentation are present.**<br>**6. Serum Free Light Chains (FLC) Assay**<br>● **Reasoning:**<br>○ **Measures the amount of free kappa and lambda light chains in the blood, which can be elevated in conditions like AL amyloidosis and multiple myeloma.**<br>○ **Provides an assessment of clonal plasma cell activity and helps monitor disease burden.**<br>○ **A high kappa/lambda ratio could indicate a more malignant process rather than MGUS.**<br>**7. Nerve Biopsy**<br>● **Reasoning:**

			○ Direct examination of the nerve tissue can reveal amyloid deposits or inflammatory infiltrates.<br>○ Helps in confirming the nature of the neuropathy (demyelinating vs. axonal, inflammatory vs. non-inflammatory).<br>○ Although more invasive, it provides definitive pathological information.<br>**8. Echocardiogram**<br>● **Reasoning:**<br>○ Useful for detecting cardiac involvement in amyloidosis, which may present as restrictive cardiomyopathy.<br>○ Assessing cardiac function can provide indirect evidence of systemic amyloidosis.<br>○ Cardiac involvement is a common and serious complication of AL amyloidosis.<br>**Most Crucial Test**<br>Based on the differential diagnoses and the clinical scenario, the most crucial test to perform is the Bone Marrow Biopsy and Aspirate.<br>● **Reasoning:**<br>○ It is critical for distinguishing between MGUS, multiple myeloma, and AL amyloidosis by providing direct evidence of plasma cell dyscrasia.<br>○ Provides information on the percentage of plasma cells, the presence of amyloid deposits, and other hematological abnormalities.<br>○ Can confirm or exclude malignancies like multiple myeloma, which have significant treatment and prognostic implications.<br>Conducting a bone marrow biopsy will yield the most definitive information to narrow down the diagnosis and guide further management of the patient's condition.
18	A 55-year-old male patient presents with a 5-month history of pure sensory, asymmetric, non-length dependent polyneuropathy with subacute onset and progressive course. The patient presents with paresthesia and hypoesthesia in the distal areas of the four limbs, severe sensory ataxia, orthostatic hypotension, and delayed gastric and intestinal emptying. The patient also reports xerostomia and xerophthalmia. Clinical examination reveals reduced pupillary reflex and anhidrosis. There are no neuropathic pain, tremor, foot deformities or cranial nerve involvement. Past medical history is positive for Hashimoto's thyroiditis. Nerve conduction studies show a pure sensory, non-length dependent, axonal polyneuropathy. Screening lab tests reveal folate deficiency and low-titre ENA positivity. Remaining exams including vitamin B12, vitamin B6, vitamin E, HBV, HCV, ANA, ANCA, CRP, ESR, copper, glycemia, HbA1c, cryoglobulins, and serum electrophoresis are normal. There is no family history of polyneuropathy.	Sjogren-associated ganglionopathy	**Step-by-Step Analysis and Additional Examinations**<br>**1. Ganglionic Acetylcholine Receptor (AChR) Antibodies**<br>● **Reasoning:**<br>○ AAG is strongly suggested by the clinical presentation, and ganglionic AChR antibodies are specific markers for this condition.<br>○ Positive antibodies would confirm the diagnosis and guide appropriate immunotherapy.<br>**2. Anti-Ro/SSA and Anti-La/SSB Antibodies**<br>● **Reasoning:**<br>○ Given the symptoms of xerostomia and xerophthalmia, along with the presence of Hashimoto's thyroiditis, Sjogren's syndrome should be considered.<br>○ Anti-Ro/SSA and Anti-La/SSB antibodies are highly specific for Sjogren's syndrome.<br>○ These tests would help differentiate between Sjogren's syndrome and other autoimmune neuropathies.<br>**3. Paraneoplastic Antibody Panel**<br>● **Reasoning:**<br>○ Paraneoplastic syndromes can present with polyneuropathy and autonomic dysfunction.<br>○ This panel includes various antibodies (e.g., anti-Hu, anti-Yo, anti-Ri) associated with paraneoplastic neurological syndromes.<br>○ Identifying a paraneoplastic etiology could lead to early detection of an occult malignancy.<br>**4. Salivary Gland Biopsy**<br>● **Reasoning:**<br>○ If Sjogren's syndrome is suspected, a minor salivary gland biopsy can provide histopathological confirmation.<br>○ This test would show lymphocytic infiltration typical of Sjogren's syndrome, aiding in diagnosis.<br>**5. Autonomic Function Testing (AFT)**<br>● **Reasoning:**<br>○ Comprehensive autonomic function tests (e.g., tilt table test, heart rate variability, QSART) can objectively measure the extent of autonomic dysfunction.<br>○ This would provide quantifiable data on the autonomic involvement and help in monitoring disease progression or response to treatment.<br>**6. Gastric Emptying Study**<br>● **Reasoning:**<br>○ Given the symptoms of delayed gastric emptying, a gastric emptying study would quantify gastric motility and provide evidence of autonomic gastrointestinal dysfunction.<br>○ This helps to correlate clinical symptoms with objective findings.<br>**7. Skin Biopsy**<br>● **Reasoning:**<br>○ A skin biopsy with assessment of intraepidermal nerve fiber density can detect small fiber neuropathy.<br>○ It can also show loss of autonomic nerve fibers, which would support the diagnosis of an autonomic neuropathy.<br>**8. MRI of the Brain and Spinal Cord**<br>● **Reasoning:**<br>○ Although cranial nerve involvement is absent, ruling out central nervous system pathology is important, especially given the sensory ataxia.<br>○ MRI can exclude structural causes of neuropathy, such as demyelinating diseases or paraneoplastic syndromes affecting the central nervous system.<br>**9. Whole Body PET-CT Scan**<br>● **Reasoning:**<br>○ To identify any occult malignancy that might be responsible for a paraneoplastic syndrome.<br>○ This imaging can detect small tumors that might not be apparent on other imaging modalities.<br>**Most Crucial Test**<br>**Ganglionic Acetylcholine Receptor (AChR) Antibodies**<br>**Reasoning:**<br>● Given the strong clinical suspicion of Autoimmune Autonomic Ganglionopathy (AAG) based on the patient's symptoms and history, testing for ganglionic AChR antibodies is crucial.<br>● This test is highly specific for AAG and would provide definitive evidence to confirm the diagnosis.<br>● Confirmation of AAG would lead to targeted treatment with immunotherapy, which can significantly improve the patient's quality of life and prognosis.
19	A 70-year-old male patient presents with a 5-year history of sensory-motor,	TTR-Amyloidosis	**Step-by-Step Analysis of Additional Examinations**<br>**Given the clinical scenario, the following additional examinations (both laboratory and instrumental) are necessary to narrow down the differential diagnoses of polyneuropathy:**

	symmetrical, length dependent polyneuropathy with insidious onset and fast disease progression. The patient presents with moderate-to-severe distal weakness and hypoesthesia in the four limbs in a distal and symmetric distribution, sensory ataxia, tongue fasciculations and autonomic involvement with no foot deformities or tremor. Past medical history is positive for cardiac insufficiency. Nerve conduction studies show an axonal, sensory-motor polyneuropathy with bilateral carpal tunnel syndrome. Screening lab tests including vitamin B12, vitamin B9, folate, vitamin E, HBV, HCV, ANA, ANCA, ENA, glycemia, HbA1c, CRP, ESR, copper, cryoglobulins, and serum electrophoresis are all negative. His father, who was wheelchair bound, died from cardiac insufficiency.		**1. Genetic Testing for Transthyretin (TTR) Mutations**<br>● **Reasoning: Familial Amyloid Polyneuropathy (FAP) is often caused by mutations in the TTR gene. Given the patient's family history and symptoms, genetic testing can confirm the presence of a TTR mutation, providing a definitive diagnosis.**<br>● **Process: Blood sample for DNA analysis, focusing on the TTR gene.**<br>**2. Nerve Biopsy**<br>● **Reasoning: A nerve biopsy can help detect amyloid deposits, which are indicative of amyloidosis, including FAP. It can also provide detailed information on the type of neuropathy (axonal vs. demyelinating).**<br>● **Process: Typically involves taking a small sample of a peripheral nerve, such as the sural nerve, and analyzing it histologically.**<br>**3. Serum and Urine Immunofixation Electrophoresis**<br>● **Reasoning: This test can help identify monoclonal proteins that are often seen in conditions like light chain amyloidosis (AL amyloidosis). While serum electrophoresis was negative, immunofixation is more sensitive.**<br>● **Process: Blood and urine samples are analyzed to detect monoclonal proteins.**<br>**4. Quantitative Sensory Testing (QST)**<br>● **Reasoning: QST helps assess the function of small and large nerve fibers. This can provide a more detailed understanding of the sensory deficits and their progression.**<br>● **Process: Involves applying controlled stimuli (thermal and mechanical) to assess sensory thresholds.**<br>**5. Autonomic Function Tests**<br>● **Reasoning: Given the autonomic involvement (e.g., orthostatic hypotension, gastrointestinal symptoms), detailed autonomic function testing can provide objective evidence of autonomic dysfunction.**<br>● **Process: Includes tests like heart rate variability, blood pressure response to tilt-table testing, and quantitative sudomotor axon reflex testing (QSART).**<br>**6. Anti-GM1 Antibody Testing**<br>● **Reasoning: Although MMN is less likely, it is important to rule out. Anti-GM1 antibodies are often present in MMN and their presence can support this diagnosis.**<br>● **Process: Blood sample analysis for anti-GM1 antibodies.**<br>**7. Cardiac Imaging and Function Tests**<br>● **Reasoning: Given the patient's and his father's history of cardiac insufficiency, and the potential for cardiac involvement in amyloidosis, detailed cardiac assessment is crucial. Echocardiography and cardiac MRI can help identify amyloid deposits in the heart.**<br>● **Process: Non-invasive imaging studies, such as echocardiogram and cardiac MRI, to assess heart structure and function.**<br>**Most Crucial Test**<br>**Genetic Testing for Transthyretin (TTR) Mutations**<br>**This test is the most crucial because:**<br>● **It directly addresses the most likely diagnosis, Familial Amyloid Polyneuropathy (FAP).**<br>● **Positive genetic testing for a TTR mutation provides a definitive diagnosis.**<br>● **It has significant implications for the patient's management and family screening.**<br>**Conclusion**<br>While multiple additional examinations are important to comprehensively evaluate the patient and rule out other conditions, genetic testing for TTR mutations stands out as the most critical test due to its potential to provide a conclusive diagnosis of FAP, which aligns with the clinical presentation and family history.
20	A 64-year-old male patient presents with an 18-month history of pure motor, asymmetric, non-length dependent polyneuropathy with insidious onset. The patient presents with weakness and atrophy in a multifocal distribution in the upper limbs. He also reports the presence of subjective paresthesia in the hands but at clinical examination objective tactile sensation is normal. There are no neuropathic pain, ataxia, tremor, foot deformities, cranial nerve or autonomic involvement. Past medical history is silent. Nerve conduction studies show pure motor, demyelinating multineuropathy in the upper limbs. Screening lab tests including vitamin B12, folate, vitamin B6, vitamin E, HCV, HBV, ANA, ANCA, ENA, CRP, ESR, glycemia, HbA1c, copper, cryoglobulins and serum electrophoresis are negative. Cervical spine MRI is normal. There is no family history of polyneuropathy.	MMN	**Step-by-Step Analysis and Additional Examinations**<br>**Step 1: Review of Current Data**<br>● **Patient Demographics: 64-year-old male**<br>● **Symptoms:**<br>○ **18-month history of pure motor, asymmetric, non-length dependent polyneuropathy with insidious onset**<br>○ **Weakness and atrophy in a multifocal distribution in the upper limbs**<br>○ **Subjective paresthesia in the hands but normal objective tactile sensation**<br>○ **No neuropathic pain, ataxia, tremor, foot deformities, cranial nerve, or autonomic involvement**<br>● **Current Investigations:**<br>○ **Nerve conduction studies: Pure motor, demyelinating multineuropathy in the upper limbs**<br>○ **Screening lab tests: Negative for vitamin B12, folate, vitamin B6, vitamin E, HCV, HBV, ANA, ANCA, ENA, CRP, ESR, glycemia, HbA1c, copper, cryoglobulins, serum electrophoresis**<br>○ **Cervical spine MRI: Normal**<br>● **History: No past medical history or family history of polyneuropathy**<br>**Step 2: Identifying Additional Examinations Given the current data, several additional tests can be helpful to further narrow down the differential diagnoses, particularly focusing on MMN, CIDP, and MADSAM.**<br>**Additional Examinations**<br>**1. Anti-GM1 Antibodies Test**<br>○ **Reasoning:**<br>■ **Anti-GM1 antibodies are frequently present in patients with Multifocal Motor Neuropathy (MMN).**<br>■ **Testing for these antibodies can provide significant diagnostic value, as their presence strongly supports MMN.**<br>○ **Specific Case Relevance:**<br>■ **The patient's presentation of asymmetric, multifocal motor weakness aligns well with MMN, making this test particularly relevant.**<br>**2. Lumbar Puncture and Cerebrospinal Fluid (CSF) Analysis**<br>○ **Reasoning:**<br>■ **CSF analysis can help distinguish between MMN, CIDP, and other inflammatory neuropathies.**<br>■ **Elevated CSF protein without pleocytosis is often seen in CIDP.**<br>■ **MMN typically does not show significant CSF abnormalities.**<br>○ **Specific Case Relevance:**<br>■ **Identifying elevated CSF protein could point towards CIDP, which is a key differential diagnosis.**<br>**3. Repetitive Nerve Stimulation (RNS) or Single Fiber Electromyography (SFEMG)**<br>○ **Reasoning:**<br>■ **These tests can help differentiate between disorders of neuromuscular transmission and motor neuron diseases.**

			■ They are particularly useful in identifying neuromuscular junction disorders like myasthenia gravis, which, although less likely, could present with pure motor symptoms.<br>○ **Specific Case Relevance:**<br>■ Given the pure motor nature of the neuropathy, ruling out neuromuscular junction disorders is prudent.<br>4. **Paraneoplastic Panel**<br>○ **Reasoning:**<br>■ Paraneoplastic syndromes can present with motor neuropathies and are associated with various malignancies.<br>■ This panel can detect antibodies related to paraneoplastic neurological syndromes.<br>○ **Specific Case Relevance:**<br>■ The patient's age and the chronic nature of symptoms warrant ruling out an underlying malignancy.<br>5. **Muscle and Nerve Biopsy**<br>○ **Reasoning:**<br>■ Biopsy can provide definitive evidence of demyelination, inflammation, and other pathological changes.<br>■ It is particularly useful when other diagnostic tests are inconclusive.<br>○ **Specific Case Relevance:**<br>■ Direct tissue examination can confirm demyelinating neuropathy and differentiate between MMN and CIDP.<br>**Most Crucial Test**<br>**Anti-GM1 Antibodies Test**<br>**Reasoning:**<br>● The presence of anti-GM1 antibodies would strongly support a diagnosis of MMN, which is the most likely diagnosis based on the clinical presentation and current investigations.<br>● This test is less invasive compared to lumbar puncture and biopsies and can provide clear diagnostic direction, thus significantly influencing the treatment plan.<br>**Conclusion:** While several additional examinations are warranted to fully evaluate the patient, the anti-GM1 antibodies test is the most crucial at this stage due to its high diagnostic yield for MMN, which is the leading differential diagnosis given the patient's clinical presentation.
21	A 74-year-old male patient presents with a 3-month history of sensory-motor, asymmetric, length dependent polyneuropathy with subacute onset and progressive course. The patient presents with sensory ataxia associated with neuropathic pain, paraesthesia, and hypoesthesia in the feet, and weakness with distal, asymmetric distribution. Clinical examination does not show presence of tremor, foot deformities, cranial nerve or autonomic involvement. Past medical history is positive for multiple myeloma, in remission for the past three years with no need for maintenance therapy (last chemotherapy cycle performed three years ago). Nerve conduction studies show a demyelinating, sensory-motor polyneuropathy. Screening lab tests show presence of IgG lambda paraprotein. Remaining exams including vitamin B12, folate, vitamin B6, vitamin E, HBV, HCV, ANA, ANCA, ENA, CRP, ESR, glycemia, HbA1c, copper, and cryoglobulins are negative. There is no family history of polyneuropathy.	CIDP	**Step-by-Step Analysis of Additional Examinations**<br>**1. Serum Immunofixation Electrophoresis and Free Light Chain Assay:**<br>● **Reasoning:**<br>○ The presence of IgG lambda paraprotein needs further characterization to determine its significance.<br>○ Immunofixation can help identify and quantify the specific monoclonal protein.<br>○ A free light chain assay can detect abnormalities in the kappa and lambda light chains, providing additional information about the monoclonal protein.<br>**2. Lumbar Puncture with Cerebrospinal Fluid (CSF) Analysis:**<br>● **Reasoning:**<br>○ CSF analysis can help detect elevated protein levels with a normal white cell count (albuminocytologic dissociation), which is typical in CIDP.<br>○ It can also help rule out infectious or inflammatory causes of neuropathy.<br>○ Oligoclonal bands and other markers can provide clues to immune-mediated neuropathies.<br>**3. MRI of the Spine with Contrast:**<br>● **Reasoning:**<br>○ MRI can help visualize nerve roots and the spinal cord.<br>○ It can identify hypertrophy of nerve roots, which is indicative of CIDP.<br>○ This can also help rule out other structural causes of neuropathy.<br>**4. Repeat Nerve Conduction Studies and Electromyography (EMG):**<br>● **Reasoning:**<br>○ Serial nerve conduction studies can monitor the progression of neuropathy and help differentiate between axonal and demyelinating processes.<br>○ EMG can provide additional details about the muscle and nerve function, helping to refine the diagnosis.<br>**5. Serum Protein Electrophoresis (SPEP) and Urine Protein Electrophoresis (UPEP):**<br>● **Reasoning:**<br>○ These tests can quantify monoclonal proteins and detect Bence Jones proteins in the urine.<br>○ They help assess the burden of monoclonal gammopathy and differentiate between multiple myeloma and MGUS.<br>**6. Biopsy of the Affected Nerve (Sural Nerve Biopsy):**<br>● **Reasoning:**<br>○ A biopsy can provide definitive histopathological evidence of the type of neuropathy.<br>○ It can reveal demyelination, axonal loss, or inflammatory infiltrates, aiding in the diagnosis of CIDP or paraneoplastic neuropathy.<br>**7. Anti-MAG (Myelin-Associated Glycoprotein) Antibody Testing:**<br>● **Reasoning:**<br>○ Anti-MAG antibodies are often present in patients with demyelinating neuropathies associated with monoclonal gammopathies.<br>○ Detection of these antibodies can support the diagnosis of MGUS-related neuropathy.<br>**Conclusion**<br>**Most Crucial Test:**<br>**Serum Immunofixation Electrophoresis and Free Light Chain Assay**<br>This test is the most crucial as it directly targets the characterization and quantification of the monoclonal protein (IgG lambda paraprotein) found in the patient's serum. Understanding the nature and extent of this paraprotein is vital in determining whether the neuropathy is related to MGUS, a more severe form of monoclonal gammopathy like multiple myeloma, or another related condition. This test is foundational in narrowing down the differential diagnoses and guiding further diagnostic and therapeutic steps.
22	An 82-year-old female patient presents with a 2-year history of pure sensory, symmetrical, length dependent	HCV-associated neuropathy	**Step-by-Step Analysis of Additional Examinations:**<br>**1. Comprehensive Blood Panel:**<br>● **Reasoning:**

	polyneuropathy with insidious onset and slow progressive course. The patient presents with paresthesia, hypoesthesia, and mild neuropathic pain in the feet, associated with diffused cramps in the lower limbs. Clinical examination does not reveal sensory ataxia, tremor, foot deformities, cranial nerve or autonomic involvement. Past medical history is positive for chronic HCV infection, psoriatic arthritis, and fibromyalgia. Nerve conduction studies show presence of a pure sensory, length dependent, symmetric, axonal polyneuropathy with unilateral carpal tunnel syndrome. Screening lab tests including vitamin B12, folate, vitamin B6, vitamin E, HBV, ANCA, ENA, CRP, ESR, copper, cryoglobulins and serum electrophoresis are all negative, with positivity for ANA autoantibodies and evidence of chronic HCV infection. There is no family history of polyneuropathy.		○ To rule out other potential metabolic and systemic causes of neuropathy, even though initial screening tests were negative.<br>● Tests:<br>○ Complete Blood Count (CBC): To check for any underlying hematological conditions.<br>○ Blood glucose levels and HbA1c: To rule out diabetes mellitus, a common cause of peripheral neuropathy.<br>○ Renal function tests (creatinine, BUN): To assess for renal insufficiency which can contribute to neuropathy.<br>○ Liver function tests: To further evaluate the liver function given her chronic HCV infection.<br>2. Immunological and Inflammatory Markers:<br>● Reasoning:<br>○ To further investigate the autoimmune component suggested by the positive ANA.<br>● Tests:<br>○ Anti-double stranded DNA (dsDNA) antibodies: To check for systemic lupus erythematosus (SLE).<br>○ Anti-Ro/SSA and Anti-La/SSB antibodies: To evaluate for Sjögren's syndrome.<br>○ Rheumatoid factor (RF) and Anti-cyclic citrullinated peptide (anti-CCP): To investigate rheumatoid arthritis and related conditions.<br>○ Complement levels (C3, C4): Low levels can indicate an ongoing autoimmune process.<br>3. CSF Analysis (Lumbar Puncture):<br>● Reasoning:<br>○ To rule out chronic inflammatory demyelinating polyneuropathy (CIDP) or other inflammatory neuropathies.<br>● Tests:<br>○ CSF protein levels: Elevated in CIDP.<br>○ Cell count and differential: To rule out infectious or inflammatory processes.<br>○ Oligoclonal bands: To assess for central nervous system autoimmune processes.<br>4. Nerve Biopsy:<br>● Reasoning:<br>○ To obtain a definitive diagnosis if less invasive tests are inconclusive, especially for distinguishing between axonal and demyelinating neuropathies or for diagnosing vasculitic neuropathy.<br>● Details:<br>○ Sural nerve biopsy: To look for signs of vasculitis, amyloidosis, or other infiltrative diseases.<br>5. Electromyography (EMG) and Repeated Nerve Conduction Studies (NCS):<br>● Reasoning:<br>○ To further characterize the type and extent of neuropathy, and to monitor progression.<br>● Details:<br>○ EMG: To assess for muscle denervation which can suggest a more chronic or severe process.<br>○ NCS: To re-evaluate for changes since initial studies and to confirm findings.<br>6. Cryoglobulin and Hepatitis C Virus RNA Quantification:<br>● Reasoning:<br>○ Despite initial negative cryoglobulin test, a repeat test can be useful as cryoglobulin levels can fluctuate.<br>● Details:<br>○ Repeat cryoglobulins: To ensure initial negative result wasn't a false negative.<br>○ HCV RNA quantification: To assess the viral load, which can correlate with disease activity and potential neuropathic involvement.<br>Most Crucial Test:<br>Comprehensive Blood Panel: Among the additional tests, the most crucial is the comprehensive blood panel, particularly focusing on blood glucose levels and HbA1c to rule out diabetes mellitus, which is a common and treatable cause of peripheral neuropathy. Despite the focus on her chronic HCV infection, diabetes should be definitively excluded due to its high prevalence and potential for contributing to neuropathic symptoms.<br>By systematically eliminating other common causes with this panel, a more accurate diagnosis of HCV-associated neuropathy or another underlying condition can be confirmed.
23	A 59-year-old male patient presents with a 1-year history of sensory-motor, symmetrical, non-length dependent polyneuropathy with subacute onset and progressive course. The patient presents with paresthesia, neuropathic pain and weakness distributed proximally and distally in the lower limbs. The patient also reports that in the last months he has noticed the appearance of white nails. Clinical examination does not reveal ataxia, tremor, foot deformities, cranial nerve or autonomic involvement. Past medical history is positive for alcohol abuse. Screening lab tests show the presence of thrombocytosis and IgG lambda monoclonal gammopathy. Vitamin B12, folate, vitamin B6, E, HCV, HBV, ANA, ANCA, ENA, CRP, ESR, glycemia, HbA1c, copper and cryoglobulins are normal.	POEMS	Step-by-Step Approach to Additional Examinations<br>Step 1: Review Current Information<br>● Patient Profile: 59-year-old male<br>● Symptoms: Sensory-motor, symmetrical, non-length dependent polyneuropathy with paresthesia, neuropathic pain, and weakness<br>● Medical History: Alcohol abuse<br>● Lab Findings: Thrombocytosis, IgG lambda monoclonal gammopathy<br>● Normal Labs: Vitamin B12, folate, vitamin B6, vitamin E, HCV, HBV, ANA, ANCA, ENA, CRP, ESR, glycemia, HbA1c, copper, cryoglobulins<br>● Nerve Conduction Studies: Mixed demyelinating-axonal, sensory-motor polyneuropathy<br>● Clinical Exam: No ataxia, tremor, cranial nerve or autonomic involvement, or foot deformities<br>Step 2: Identify Key Areas for Further Investigation<br>1. Monoclonal Gammopathy<br>2. Alcoholic Neuropathy<br>3. Other Neuropathies (e.g., CIDP, Paraneoplastic, Toxic)<br>Step 3: Additional Examinations<br>1. Serum and Urine Immunofixation Electrophoresis:<br>○ Reasoning: To further characterize the monoclonal gammopathy. This will help determine if there is a higher level of monoclonal protein or presence of light chains, indicating possible multiple myeloma or other related disorders.<br>2. Bone Marrow Biopsy:<br>○ Reasoning: To evaluate for plasma cell dyscrasia, including multiple myeloma. This is particularly important given the IgG lambda monoclonal gammopathy and thrombocytosis.<br>3. CT Scan or PET Scan:

	Nerve conduction studies show a mixed demyelinating-axonal, sensory-motor polyneuropathy. There is no family history of polyneuropathy.		○ **Reasoning: To look for any signs of malignancy, including lymphoproliferative disorders, which could be associated with the monoclonal gammopathy.**<br>**4. Liver Function Tests and Gamma-Glutamyl Transferase (GGT):**<br>○ **Reasoning: To assess the impact of alcohol abuse on the liver, as liver dysfunction can contribute to peripheral neuropathy.**<br>**5. Magnetic Resonance Imaging (MRI) of the Spine and Brain:**<br>○ **Reasoning: To rule out central nervous system involvement, which can occasionally present with similar symptoms and might reveal lesions associated with CIDP or other demyelinating diseases.**<br>**6. CSF Analysis:**<br>○ **Reasoning: To check for elevated protein levels and presence of oligoclonal bands, which could support a diagnosis of CIDP.**<br>**7. Serum Free Light Chain Assay:**<br>○ **Reasoning: To quantify free light chains in the blood, which can indicate the severity and type of monoclonal gammopathy.**<br>**8. Autonomic Function Tests:**<br>○ **Reasoning: Although the clinical exam showed no autonomic involvement, these tests can help confirm the absence and rule out autonomic neuropathy.**<br>**9. Electromyography (EMG):**<br>○ **Reasoning: To further assess muscle function and pinpoint the extent and nature of nerve damage.**<br>**Step 4: Determine the Most Crucial Test**<br>**Most Crucial Test: Bone Marrow Biopsy**<br>**Reasoning:**<br>● **The presence of an IgG lambda monoclonal gammopathy combined with thrombocytosis raises significant concern for plasma cell dyscrasias, such as multiple myeloma or related disorders.**<br>● **A bone marrow biopsy can provide definitive information on the presence of abnormal plasma cells or other hematologic malignancies.**<br>● **Identifying a potential underlying hematologic malignancy can have profound implications for treatment and prognosis, making it the most crucial test in this context.**<br>**Conclusion**<br>**The bone marrow biopsy stands out as the most critical test to perform next. It will help clarify the nature of the monoclonal gammopathy and its possible role in the patient's polyneuropathy. Other tests listed should follow based on the results of the bone marrow biopsy to provide a comprehensive diagnostic picture.**
24	A 77-year-old male patient presents with a 1-year history of sensory-motor, symmetrical, length dependent polyneuropathy with subacute onset. The patient presents with distal leg paresthesia and neuropathic pain, distal weakness in the upper and lower limbs, without tremor, ataxia, foot deformities, cranial nerve or autonomic involvement. Past medical history shows cutaneous angiomas and IgG lambda monoclonal gammopathy. Nerve conduction studies show a demyelinating, symmetric, sensory-motor polyneuropathy. Screening lab tests including vitamin B12, folate, vitamin B6, vitamin E, HCV, HBV, ANA, ANCA, ENA, CRP, ESR, glycemia, HbA1c, copper and cryoglobulins are all negative, with positivity for IgG lambda paraprotein. There is no family history of polyneuropathy.	POEMS	**Step-by-Step Analysis and Additional Examinations**<br>**1. Confirm Diagnosis of Monoclonal Gammopathy and Evaluate for MGUS or Other Plasma Cell Disorders:**<br>● **Serum and Urine Protein Electrophoresis (SPEP and UPEP) with Immunofixation:**<br>○ **Reasoning: To confirm the presence and quantify the monoclonal protein (IgG lambda) and rule out multiple myeloma or other plasma cell dyscrasias. These tests can help differentiate between MGUS and more malignant conditions.**<br>● **Serum Free Light Chains (FLC) Assay:**<br>○ **Reasoning: To evaluate the kappa and lambda light chain levels and their ratio, which can provide further insight into the nature of the monoclonal gammopathy.**<br>**2. Assess for POEMS Syndrome:**<br>● **VEGF (Vascular Endothelial Growth Factor) Levels:**<br>○ **Reasoning: Elevated VEGF levels are a hallmark of POEMS syndrome. Given the patient's cutaneous angiomas, this test would help in confirming or ruling out POEMS.**<br>● **Full Body Imaging (CT or PET-CT Scan):**<br>○ **Reasoning: To identify organomegaly, sclerotic bone lesions, and lymphadenopathy, which are components of POEMS syndrome.**<br>● **Endocrine Evaluation (Thyroid Function Tests, Blood Glucose, Testosterone, Cortisol Levels):**<br>○ **Reasoning: To check for endocrinopathies, another component of POEMS syndrome.**<br>**3. Evaluate for CIDP:**<br>● **Lumbar Puncture for Cerebrospinal Fluid (CSF) Analysis:**<br>○ **Reasoning: Elevated protein without pleocytosis in CSF is characteristic of CIDP. This can help differentiate CIDP from other causes of polyneuropathy.**<br>**4. Rule Out Amyloidosis:**<br>● **Abdominal Fat Pad Biopsy with Congo Red Staining:**<br>○ **Reasoning: To check for amyloid deposits, as amyloidosis can present with a similar clinical picture and is often associated with monoclonal gammopathy.**<br>**5. Detailed Neurological Evaluation:**<br>● **Quantitative Sensory Testing (QST):**<br>○ **Reasoning: To objectively measure sensory function and detect small fiber neuropathy, which can provide additional information on the nature of the polyneuropathy.**<br>● **Skin Biopsy:**<br>○ **Reasoning: To assess for intraepidermal nerve fiber density, which can help identify small fiber neuropathy not detected by nerve conduction studies.**<br>**Most Crucial Test**<br>**Serum and Urine Protein Electrophoresis (SPEP and UPEP) with Immunofixation:**<br>● **Reasoning: This test is crucial because it will confirm the presence of the monoclonal protein and its type (IgG lambda), helping to differentiate between MGUS, multiple myeloma, and other plasma cell disorders. This differentiation is vital for guiding further management and treatment, as the therapeutic approach and prognosis differ significantly between these conditions.**<br>**Conclusion**<br>**By systematically conducting these additional examinations, the differential diagnoses can be narrowed down significantly. The SPEP and UPEP with immunofixation are paramount in confirming the diagnosis of MGUS and ruling out more serious plasma cell disorders, which is the most likely cause given the patient's clinical presentation and current laboratory findings.**
25	A 78-year-old male patient presents with an 8-month history of sensory-motor, symmetrical, length dependent polyneuropathy with insidious onset and progressive course. The patient presents with paresthesia, neuropathic pain and weakness distributed distally in the lower limbs. Clinical examination also reveals the	anti-MAG polyneuropathy	**Step-by-Step Analysis of Necessary Additional Examinations**<br>**Initial Clinical Information Recap**<br>● **Patient Profile:**<br>○ **78-year-old male with 8-month history of sensory-motor, symmetrical, length-dependent polyneuropathy.**<br>○ **Symptoms include paresthesia, neuropathic pain, weakness in lower limbs, and sensory ataxia.**<br>○ **Known IgM monoclonal gammopathy.**<br>● **Diagnostic Findings:**<br>○ **Nerve conduction studies show demyelinating, symmetric, sensory-motor polyneuropathy.**

	presence sensory ataxia. There are no foot deformities, tremor, cranial nerve or autonomic involvement. Past medical history is positive for IgM monoclonal gammopathy. Nerve conduction studies show a demyelinating, symmetric, sensory-motor polyneuropathy. Screening lab tests including vitamin B12, folate, vitamin B6, vitamin E, HCV, HBV, ANA, ANCA, ENA, CRP, ESR, glycemia, HbA1c, copper and cryoglobulins are all negative, with positivity for IgM paraprotein. There is no family history of polyneuropathy.		○ **Comprehensive lab tests are mostly negative except for the presence of IgM paraprotein.**<br>**Additional Examinations**<br>**1. Serum Protein Electrophoresis (SPEP) and Immunofixation Electrophoresis (IFE):**<br>○ **Reasoning:**<br>■ **These tests will help to quantify the monoclonal protein and confirm the type of paraprotein.**<br>■ **Important for differentiating between MGUS, WM, and other monoclonal gammopathies.**<br>■ **Can provide information on the monoclonal spike and its characteristics.**<br>**2. Bone Marrow Biopsy:**<br>○ **Reasoning:**<br>■ **Essential for diagnosing Waldenström Macroglobulinemia by identifying lymphoplasmacytic lymphoma.**<br>■ **Will provide detailed information on bone marrow involvement, which is crucial for staging and management.**<br>■ **Helps to rule out other hematologic malignancies that might present with a monoclonal gammopathy.**<br>**3. Serum Viscosity:**<br>○ **Reasoning:**<br>■ **Hyperviscosity syndrome is a potential complication of WM due to high levels of IgM.**<br>■ **Elevated serum viscosity can cause symptoms and needs to be monitored and managed appropriately.**<br>**4. Magnetic Resonance Imaging (MRI) of the Spine:**<br>○ **Reasoning:**<br>■ **To evaluate any structural causes for neuropathy, especially if spinal involvement is suspected.**<br>■ **Can help rule out spinal stenosis or other compressive neuropathies.**<br>**5. Positron Emission Tomography-Computed Tomography (PET-CT):**<br>○ **Reasoning:**<br>■ **To assess for systemic involvement and to identify potential sites of lymphoplasmacytic infiltration.**<br>■ **Useful for staging WM and evaluating for other malignancies.**<br>**6. Quantitative Immunoglobulins (IgG, IgA, IgM):**<br>○ **Reasoning:**<br>■ **To assess the levels of different immunoglobulins.**<br>■ **Useful for understanding the extent of monoclonal gammopathy and potential suppression of normal immunoglobulin production.**<br>**7. Antiglycolipid Antibodies (e.g., anti-MAG antibodies):**<br>○ **Reasoning:**<br>■ **Monoclonal gammopathy-associated neuropathies often have specific antibodies, such as anti-MAG.**<br>■ **Presence of these antibodies can confirm a specific subtype of neuropathy associated with monoclonal gammopathy.**<br>**8. Cerebrospinal Fluid (CSF) Analysis:**<br>○ **Reasoning:**<br>■ **To evaluate for inflammatory markers and oligoclonal bands that might suggest an inflammatory neuropathy.**<br>■ **Helpful in ruling out other central nervous system involvements.**<br>**9. Nerve Biopsy:**<br>○ **Reasoning:**<br>■ **Although invasive, it can provide definitive histopathological confirmation of demyelination and potential monoclonal protein deposition in nerves.**<br>■ **Useful when diagnosis remains unclear after less invasive tests.**<br>**Most Crucial Test**<br>The most crucial test in this scenario is the Bone Marrow Biopsy. This test is pivotal because:<br>● It directly assesses bone marrow involvement, which is key to diagnosing Waldenström Macroglobulinemia.<br>● Provides histological and cytological details necessary for differentiating WM from other potential diagnoses such as MGUS or other hematologic malignancies.<br>● Helps in determining the extent of the disease, which is crucial for planning appropriate treatment and management.<br>By performing the bone marrow biopsy, clinicians can obtain critical diagnostic information that aligns with the patient's clinical presentation and the presence of IgM monoclonal gammopathy, leading to a more accurate and definitive diagnosis.
26	An 80-year-old male patient presents with a 2-year history of sensory-motor, asymmetrical, non-length dependent polyneuropathy with subacute onset and a rapid progressive course. The patient presents with paraesthesia, sensory ataxia, neuropathic pain, proximal and distal weakness in the four limbs, and tremor. Clinical examination shows symmetric proximal and distal weakness in the upper and lower limbs, hypoesthesia in a stocking and glove distribution, sensory ataxia, mild intrinsic hand atrophy, and cutaneous angiomas. Clinical examination does not show the presence of foot deformities, cranial nerve, or autonomic involvement.	POEMS	**Step-by-Step Analysis of Additional Examinations**<br>**1. Review of Initial Information:**<br>○ **Patient: 80-year-old male**<br>○ **Symptoms: Sensory-motor polyneuropathy, paraesthesia, sensory ataxia, neuropathic pain, proximal and distal weakness, tremor**<br>○ **Clinical findings: Symmetric proximal and distal weakness, hypoesthesia in stocking and glove distribution, sensory ataxia, mild intrinsic hand atrophy, cutaneous angiomas**<br>○ **Past medical history: Chronic HCV infection**<br>○ **Nerve conduction studies: Mixed demyelinating-axonal, symmetric, sensory-motor polyneuropathy**<br>○ **Positive tests: Polycythaemia, IgM paraprotein, chronic HCV infection**<br>○ **Negative tests: Vitamin B12, folate, vitamin B6, vitamin E, HBV, ANA, ANCA, ENA, CRP, ESR, copper, cryoglobulins**<br>○ **No family history of polyneuropathy**<br>**Additional Examinations**<br>**1. Serum Protein Electrophoresis (SPEP) and Immunofixation Electrophoresis (IFE):**<br>○ **Reasoning: To further characterize and quantify the IgM paraprotein. This can help in diagnosing monoclonal gammopathies such as Waldenström's Macroglobulinemia (WM) or multiple myeloma.**<br>**2. Bone Marrow Biopsy and Aspirate:**<br>○ **Reasoning: To evaluate for the presence of lymphoplasmacytic lymphoma or other hematologic malignancies. This is crucial for diagnosing WM, where bone marrow infiltration by malignant cells would be expected.**<br>**3. HCV RNA Quantification:**

	Past medical history is positive for chronic HCV infection. Nerve conduction studies show a mixed demyelinating-axonal, symmetric, sensory-motor polyneuropathy. Screening lab tests reveal the presence of polycythaemia, IgM paraprotein, and chronic HCV infection. Remaining lab tests, including vitamin B12, folate, vitamin B6, vitamin E, HBV, ANA, ANCA, ENA, CRP, ESR, copper and cryoglobulins are all negative. There is no family history of polyneuropathy.		○ Reasoning: To assess the viral load and confirm active infection. This can help determine the role of HCV in the patient's neuropathy and guide treatment decisions.<br>4. Anti-MAG (Myelin-Associated Glycoprotein) Antibodies:<br>○ Reasoning: To evaluate for the presence of antibodies associated with demyelinating neuropathies, particularly in the context of paraproteinemia. These antibodies are often found in patients with neuropathy associated with IgM paraproteins.<br>5. Cerebrospinal Fluid (CSF) Analysis:<br>○ Reasoning: To look for elevated protein levels, oligoclonal bands, and other markers of inflammation that can support a diagnosis of CIDP or other inflammatory neuropathies.<br>6. MRI of the Brain and Spine:<br>○ Reasoning: To rule out central nervous system involvement and to evaluate for any structural abnormalities that might be contributing to the neuropathy.<br>7. Ophthalmologic Examination:<br>○ Reasoning: To check for retinal changes such as Waldenström's retinopathy, which can provide further evidence for WM.<br>8. Autonomic Testing:<br>○ Reasoning: Although no autonomic involvement was noted on initial examination, a thorough autonomic assessment can help rule out subtle autonomic neuropathy.<br>9. Skin Biopsy:<br>○ Reasoning: To examine the cutaneous angiomas and to assess for small fiber neuropathy if there is suspicion of such involvement.<br>Most Crucial Test<br>Bone Marrow Biopsy and Aspirate<br>● Reasoning: This test is pivotal because it can confirm the diagnosis of Waldenström's Macroglobulinemia by identifying lymphoplasmacytic infiltration in the bone marrow. It also helps differentiate between other potential causes of paraproteinemia and neuropathy. A positive finding here would significantly narrow the differential diagnosis and guide appropriate treatment.<br>Conclusion<br>While several additional examinations are necessary to fully understand the patient's condition, a bone marrow biopsy and aspirate are the most crucial tests. This test will provide definitive evidence to confirm or rule out Waldenström's Macroglobulinemia, thereby significantly influencing the diagnostic and therapeutic approach for this patient.
27	A 76-year-old female patient presents with a three-year history of pure sensory, symmetrical, length-dependent polyneuropathy with insidious onset and progressive course. The patient reports paraesthesia, severe neuropathic pain, and cramps in the feet in a symmetric distribution, which began approximately three years ago but significantly worsened after the third cycle of chemotherapy. Clinical examination does not reveal ataxia, tremor, foot deformities, cranial nerve or autonomic dysfunction. Past medical history is positive for type 2 diabetes, non-Hodgkin lymphoma, and breast cancer, for which she has undergone chemotherapy. Nerve conduction studies show a sensorimotor, length-dependent, symmetric, axonal polyneuropathy. Screening lab tests including vitamin B12, folate, vitamin B6, vitamin E, HCV, ANA, ANCA, ENA, CRP, ESR, copper, cryoglobulins, and serum electrophoresis are all negative. However, there is evidence of hyperglycemia, elevated HbA1c, and chronic HBV infection. There is no family history of polyneuropathy.	Superimposed chemotherapy-induced neuropathy on diabetic polyneuropathy	Step-by-Step Approach to Additional Examinations<br>1. Detailed Patient History and Physical Examination<br>● Reasoning: A thorough re-evaluation of the patient's history and physical examination can provide additional clues and ensure that no relevant information is overlooked. This may include more specific questions about pain characteristics, any other symptoms not initially reported, and a detailed family history.<br>2. Glucose Tolerance Test (GTT)<br>● Reasoning: Given the patient's history of type 2 diabetes and hyperglycemia, it is crucial to assess the extent of glucose intolerance. This test will provide a comprehensive understanding of her glucose metabolism and help quantify the severity of her diabetes, which is closely related to diabetic polyneuropathy.<br>● Expected Outcome: This will confirm if the patient's glucose levels are consistently high and help correlate the severity of neuropathy with glycemic control.<br>3. Comprehensive Metabolic Panel (CMP)<br>● Reasoning: A CMP can provide detailed information about the patient's overall metabolic state, including liver and kidney function, electrolytes, and blood glucose levels.<br>● Expected Outcome: Identifies any metabolic derangements that might contribute to neuropathy.<br>4. Hemoglobin A1c (HbA1c)<br>● Reasoning: While elevated HbA1c was already noted, repeating this test can help determine current glycemic control and correlate it with neuropathy progression.<br>● Expected Outcome: Confirms chronic hyperglycemia if persistently elevated, supporting diabetic neuropathy.<br>5. Liver Function Tests (LFTs)<br>● Reasoning: Considering the chronic HBV infection, LFTs are necessary to evaluate the liver's status, as hepatic dysfunction can contribute to neuropathy.<br>● Expected Outcome: Identifies any liver dysfunction that might be exacerbating neuropathy.<br>6. Nerve Biopsy<br>● Reasoning: Although invasive, a nerve biopsy can provide definitive information about the type of nerve damage and help distinguish between different causes of neuropathy, such as diabetes versus other inflammatory or toxic causes.<br>● Expected Outcome: Histopathological findings may indicate characteristic features of diabetic neuropathy or reveal alternative etiologies.<br>7. Skin Biopsy for Intraepidermal Nerve Fiber Density (IENFD)<br>● Reasoning: This less invasive test measures small nerve fiber density and can be particularly useful in diagnosing small fiber neuropathy, which might not be detected on routine nerve conduction studies.<br>● Expected Outcome: Reduced nerve fiber density would support a diagnosis of small fiber neuropathy, common in diabetes.<br>8. Quantitative Sensory Testing (QST)<br>● Reasoning: QST measures sensory nerve function and can help quantify the severity of sensory loss and pain, providing objective data to support the clinical diagnosis.<br>● Expected Outcome: Helps correlate sensory dysfunction with clinical findings.<br>9. HBV DNA Quantification and Hepatitis B Serology Panel<br>● Reasoning: To assess the activity of chronic HBV infection and potential ongoing liver inflammation, which could contribute to neuropathy.<br>● Expected Outcome: High viral load or signs of active hepatitis might suggest an HBV-related component to neuropathy.<br>10. Autoantibody Panel (Expanded)<br>● Reasoning: Although initial screening was negative, a more comprehensive panel could uncover less common autoimmune causes of neuropathy.<br>● Expected Outcome: Identifies any autoimmune involvement not detected by initial screening.<br>11. Lumbar Puncture (CSF Analysis)<br>● Reasoning: To rule out inflammatory or infectious causes of neuropathy by analyzing cerebrospinal fluid.<br>● Expected Outcome: Elevated protein or presence of inflammatory cells might suggest an inflammatory neuropathy.<br>Most Crucial Test<br>Among these tests, Hemoglobin A1c (HbA1c) is the most crucial test. This test provides essential information on the patient's long-term glycemic control, which is directly related to the development and progression of diabetic polyneuropathy. Given the patient's history of diabetes and the significant impact of glycemic control on neuropathy, confirming and closely monitoring HbA1c levels will be pivotal in managing her condition and addressing the primary cause of her symptoms.<br>HbA1c can guide therapeutic adjustments and help correlate the relationship between blood sugar levels and neuropathic symptoms, ensuring targeted and effective treatment.

28	A 37-year-old male patient presents with a 2-week history of sensory-motor, symmetrical, non-length dependent polyneuropathy with acute onset. The patient presents with distal paresthesia in the upper and lower limbs, neuropathic pain, and weakness in the proximal and distal areas of the upper and lower limbs. There are no ataxia, tremor, foot deformities, cranial nerve or autonomic involvement. Past medical history is silent. Nerve conduction studies show a demyelinating, sensory-motor polyneuropathy. Screening lab tests including vitamin B12, folate, vitamin B6, vitamin E, HCV, HBV, ANA, ANCA, ENA, CRP, ESR, glycemia, HbA1c, copper, cryoglobulins and serum electrophoresis are all negative. There is no family history of polyneuropathy.	GBS	**Step-by-Step Analysis of Additional Examinations**<br>**Step 1: Lumbar Puncture (CSF Analysis)**<br>● **Reasoning: In the context of suspected Guillain-Barré Syndrome (GBS), a lumbar puncture can provide critical information. The cerebrospinal fluid (CSF) in GBS typically shows an elevated protein level with a normal white cell count (albuminocytologic dissociation).**<br>● **Expected Findings: Elevated protein without significant pleocytosis.**<br>● **Impact: This finding would strongly support the diagnosis of GBS and help differentiate it from other causes of polyneuropathy.**<br>**Step 2: Repeat Nerve Conduction Studies (NCS) and Electromyography (EMG)**<br>● **Reasoning: Given the initial findings of a demyelinating polyneuropathy, repeat studies can provide more detailed information on the progression of demyelination and any signs of axonal damage. EMG can help in assessing muscle involvement and confirming the demyelinating nature.**<br>● **Expected Findings: Further evidence of demyelination, possibly with temporal dispersion and conduction block.**<br>● **Impact: Helps in distinguishing between AIDP (GBS) and CIDP, and monitoring disease progression.**<br>**Step 3: MRI of the Spine with Contrast**<br>● **Reasoning: An MRI can reveal nerve root enhancement or thickening, which is often seen in GBS due to inflammation of the nerve roots. This can also help in ruling out other conditions such as CIDP or structural abnormalities.**<br>● **Expected Findings: Enhancement of the cauda equina or nerve roots.**<br>● **Impact: Supports the diagnosis of GBS and helps exclude other structural causes of neuropathy.**<br>**Step 4: Autoimmune Panel (including Anti-GM1 antibodies)**<br>● **Reasoning: While the initial autoimmune screening was negative, specific antibodies, such as anti-GM1, can be associated with GBS variants like multifocal motor neuropathy (MMN) or other autoimmune neuropathies.**<br>● **Expected Findings: Positive anti-GM1 antibodies would indicate a likelihood of MMN or a specific GBS variant.**<br>● **Impact: Provides more specific information regarding the underlying cause of the neuropathy, particularly if MMN is considered.**<br>**Step 5: Infectious Disease Panel**<br>● **Reasoning: Certain infections (e.g., Campylobacter jejuni, Cytomegalovirus, Epstein-Barr virus, Mycoplasma pneumoniae) have been associated with the onset of GBS. Testing for these pathogens can help identify a possible trigger.**<br>● **Expected Findings: Recent or active infection with known GBS-associated pathogens.**<br>● **Impact: Identification of a triggering infection can support the diagnosis of GBS and guide appropriate treatment strategies.**<br>**Step 6: Immunoglobulin and Complement Levels**<br>● **Reasoning: Measuring serum immunoglobulins (IgG, IgA, IgM) and complement levels (C3, C4) can help in identifying underlying immune deficiencies or dysregulations that might predispose to neuropathies.**<br>● **Expected Findings: Abnormal immunoglobulin or complement levels could indicate an underlying immunological disorder contributing to the neuropathy.**<br>● **Impact: Provides a broader understanding of the patient's immune status and potential secondary causes of neuropathy.**<br>**Determining the Most Crucial Test**<br>**Among the tests listed, the Lumbar Puncture (CSF Analysis) is the most crucial. This test is particularly important in differentiating GBS from other potential causes of polyneuropathy due to its characteristic findings (elevated protein with normal white cell count).**<br>**Conclusion**<br>**Lumbar Puncture (CSF Analysis) is prioritized as it provides direct and significant evidence for diagnosing Guillain-Barré Syndrome (GBS) and helps to rule out other differential diagnoses, thereby narrowing down the cause of the polyneuropathy efficiently. This step is critical in confirming the working diagnosis and guiding subsequent management and treatment strategies.**
29	A 75-year-old male patient presents with a 4-year history of pure sensory, symmetrical, length dependent polyneuropathy with insidious onset and slow progressive course. The patient presents with paresthesia and neuropathic pain in the feet. Clinical examination does not showtremor or sensory ataxia. There are no signs of foot deformities, cranial nerve or autonomic involvement. Past medical history is positive for type 2 diabetes and arterial hypertension. Nerve conduction studies show a mixed sensory-motor, length dependent, axonal polyneuropathy. Screening lab tests reveal hyperglycaemia and elevated HbA1c. Remaining exams including vitamin B12, vitamin B6, vitamin E, HBV, HCV, ANA, ANCA, ENA, CRP, ESR, copper, cryoglobulin, and serum electrophoresis are normal. There is no family history of polyneuropathy.	Diabetic neuropathy	**Additional Examinations for Narrowing Down Differential Diagnoses:**<br>**1. Glycemic Control Monitoring:**<br>○ **Tests: Fasting Blood Glucose, Oral Glucose Tolerance Test (OGTT)**<br>○ **Reasoning:**<br>■ **While hyperglycemia and elevated HbA1c are indicative of diabetes, it's crucial to assess the current state of glycemic control more precisely. These tests help to confirm the consistency and extent of hyperglycemia over time and can provide further evidence for diabetic neuropathy.**<br>**2. Detailed Neurological Examination:**<br>○ **Tests: Quantitative Sensory Testing (QST), Electromyography (EMG)**<br>○ **Reasoning:**<br>■ **QST can evaluate the patient's sensory thresholds and identify deficits in different sensory modalities (e.g., thermal, vibratory). EMG can help assess the extent of axonal damage and distinguish between axonal and demyelinating neuropathies. These tests provide a deeper understanding of the neuropathy's characteristics and severity.**<br>**3. Autoimmune Screening:**<br>○ **Tests: Anti-Hu, Anti-Ro, Anti-La antibodies, Paraneoplastic panel**<br>○ **Reasoning:**<br>■ **Although initial autoimmune markers (ANA, ANCA) were normal, specific antibody testing could reveal an underlying autoimmune process, especially considering the age of the patient and the chronic nature of the neuropathy. Paraneoplastic syndromes can present similarly, and this would be pertinent given the patient's age.**<br>**4. Nutritional Deficiencies Assessment:**<br>○ **Tests: Homocysteine, Methylmalonic Acid (MMA), Folate levels**<br>○ **Reasoning:**<br>■ **Although vitamin B12 levels are normal, MMA and homocysteine are more sensitive markers for B12 deficiency. Folate levels should be checked as its deficiency can also cause neuropathy. These assessments are crucial to rule out any nutritional causes.**<br>**5. Electrolyte and Renal Function Tests:**<br>○ **Tests: Serum creatinine, Blood Urea Nitrogen (BUN), Serum electrolytes (Sodium, Potassium, Calcium, Magnesium)**<br>○ **Reasoning:**<br>■ **Electrolyte imbalances and renal dysfunction can contribute to neuropathic symptoms. These tests ensure there are no underlying metabolic disturbances contributing to the patient's condition.**<br>**6. Toxic Exposure Screening:**<br>○ **Tests: Heavy Metals Panel (Lead, Mercury, Arsenic)**<br>○ **Reasoning:**<br>■ **Chronic exposure to heavy metals can cause peripheral neuropathy. This screening helps to rule out environmental or occupational exposure that might not have been initially considered.**

			7. **Imaging Studies:**<br>○ **Tests: MRI of the spine and brain, Ultrasound of peripheral nerves**<br>○ **Reasoning:**<br>■ **MRI can help exclude central causes of neuropathy and structural lesions that might contribute to the symptoms. Ultrasound of peripheral nerves can provide information about nerve enlargement or compression, which can aid in differentiating various neuropathies.**<br>**Most Crucial Test:**<br>**Quantitative Sensory Testing (QST) is the most crucial test from the list. This test provides detailed information on the functional status of different sensory modalities, which is key in a patient presenting with pure sensory symptoms. It helps to objectively quantify sensory deficits, differentiating between small and large fiber neuropathy, and can guide further diagnostic and therapeutic steps specifically tailored to the patient's sensory neuropathy.**
30	A 60-year-old male patient presents with a 17-month history of sensory, symmetrical, length dependent polyneuropathy with an insidious onset and a slow progressive course. The first occurred symptom was neuropathic pain in the feet, followed by erectile dysfunction, hyperhidrosis and mild distal paraesthesia. Clinical examination does not show presence of sensory ataxia, tremor or foot deformities. Past medical history is silent. Nerve conduction studies show a mixed sensory-motor, symmetric, axonal polyneuropathy and bilateral carpal tunnel syndrome. Screening lab tests including vitamin B12, folate, vitamin B6, vitamin E, HBV, HCV, ANA, ANCA, ENA, CRP, ESR, glycemia, HbA1c, copper, and cryoglobulins are normal. Serum electrophoresis shows presence of IgG lambda paraprotein. There is no family history of polyneuropathy.	Amyloidosis associated polyneuropathy	**Step-by-Step Analysis and Additional Examinations**<br>**Step 1: Review Clinical Data**<br>● **Patient Demographics: 60-year-old male**<br>● **Symptom History: 17-month history of sensory, symmetrical, length-dependent polyneuropathy; insidious onset, slow progression**<br>● **Initial and Subsequent Symptoms: Neuropathic pain in feet, erectile dysfunction, hyperhidrosis, mild distal paraesthesia**<br>● **Clinical Findings: No sensory ataxia, tremor, or foot deformities**<br>● **Nerve Conduction Studies: Mixed sensory-motor, symmetric, axonal polyneuropathy; bilateral carpal tunnel syndrome**<br>● **Laboratory Findings: Normal B12, folate, B6, vitamin E, HBV, HCV, ANA, ANCA, ENA, CRP, ESR, glycemia, HbA1c, copper, cryoglobulins; presence of IgG lambda paraprotein**<br>● **Family History: No polyneuropathy**<br>**Step 2: Establish Differential Diagnoses**<br>1. **Monoclonal Gammopathy of Undetermined Significance (MGUS)**<br>2. **Light Chain (AL) Amyloidosis**<br>3. **Chronic Inflammatory Demyelinating Polyneuropathy (CIDP)**<br>**Step 3: List Additional Examinations**<br>1. **Bone Marrow Biopsy and Aspiration**<br>○ **Reasoning: To evaluate for clonal plasma cell disorders like multiple myeloma or AL amyloidosis, which can present with neuropathy and paraproteinemia.**<br>2. **Serum Free Light Chain Assay**<br>○ **Reasoning: To quantify free kappa and lambda light chains, helping to distinguish MGUS from AL amyloidosis or multiple myeloma.**<br>3. **Urine Immunofixation Electrophoresis (IFE)**<br>○ **Reasoning: To detect Bence Jones proteins, which are indicative of monoclonal light chains in urine, supporting a diagnosis of AL amyloidosis or multiple myeloma.**<br>4. **Nerve Biopsy**<br>○ **Reasoning: To directly examine the nerve tissue for amyloid deposits, vasculitis, or other pathological changes indicative of specific neuropathies.**<br>5. **Electrocardiogram (ECG) and Echocardiogram**<br>○ **Reasoning: To assess for cardiac involvement, particularly amyloid cardiomyopathy, which is common in AL amyloidosis and can significantly affect prognosis and management.**<br>6. **Autonomic Function Tests**<br>○ **Reasoning: To quantify the extent of autonomic dysfunction (e.g., heart rate variability, sweat tests) and correlate with the autonomic symptoms like erectile dysfunction and hyperhidrosis.**<br>7. **CT or MRI of the Spine**<br>○ **Reasoning: To rule out structural causes of neuropathy or radiculopathy, particularly if there's suspicion of spinal involvement or compression.**<br>8. **Quantitative Sensory Testing (QST)**<br>○ **Reasoning: To provide a detailed assessment of sensory nerve function, helping to correlate clinical symptoms with objective measures of sensory deficits.**<br>9. **Positron Emission Tomography (PET) Scan**<br>○ **Reasoning: To evaluate for systemic involvement and detect any hidden malignancies that could be associated with paraneoplastic syndromes causing neuropathy.**<br>**Most Crucial Test**<br>**Bone Marrow Biopsy and Aspiration**<br>● **Reasoning: This test is critical for definitively identifying the presence and type of plasma cell disorder. Given the detected IgG lambda paraprotein, a bone marrow biopsy will help determine if there is a significant plasma cell population indicating MGUS, multiple myeloma, or AL amyloidosis. It provides direct evidence of clonal plasma cell disorders, which are crucial in differentiating between the primary suspected diagnoses.**<br>**Conclusion**<br>**The Bone Marrow Biopsy and Aspiration is the most crucial test, as it will help confirm or rule out significant plasma cell disorders, providing a definitive direction for diagnosis and subsequent management.**
31	A 54-year-old male patient presents with a 2-year history of chronic, pure sensory, symmetrical, length dependent polyneuropathy with insidious onset. The patient presents with distal paresthesia and neuropathic pain in the lower limbs, without ataxia. Past medical history reveals high alcohol consumption. Clinical examination reveals absence of foot deformities, tremor, cranial nerve or autonomic involvement. Nerve conduction studies show a length-dependent polyneuropathy with symmetric, pure sensory, axonal involvement. Screening lab tests including vitamin B12, folate, vitamin B6, vitamin E, HCV, HBV, ANA, ANCA, ENA, CRP, ESR, glycemia, HbA1c, copper,	Alcohol-associated neuropathy	**Step-by-Step Analysis and Additional Examinations**<br>1. **Detailed Summary of the Case:**<br>○ **Patient Profile: 54-year-old male**<br>○ **Symptom Duration: 2 years**<br>○ **Symptom Description: Chronic, pure sensory, symmetrical, length-dependent polyneuropathy with distal paresthesia and neuropathic pain in lower limbs.**<br>○ **Onset: Insidious**<br>○ **Past Medical History: High alcohol consumption**<br>○ **Clinical Findings: No foot deformities, tremor, cranial nerve, or autonomic involvement.**<br>○ **Nerve Conduction Studies: Symmetric, pure sensory, axonal involvement in a length-dependent pattern.**<br>○ **Screening Lab Tests: Negative for various vitamin deficiencies, infections, autoantibodies, inflammatory markers, and metabolic conditions. Elevated serum GGT levels.**<br>○ **Family History: No family history of polyneuropathy.**<br>2. **Additional Examinations:**<br>○ **Laboratory Tests:**<br>1. **Thiamine (Vitamin B1) Levels:**<br>■ **Reasoning: Chronic alcohol consumption can lead to thiamine deficiency, contributing to peripheral neuropathy. Measuring thiamine levels can help identify or rule out this deficiency.**<br>2. **Magnesium Levels:**<br>■ **Reasoning: Alcohol abuse can cause hypomagnesemia, which may contribute to neurological symptoms. Evaluating magnesium levels can help assess electrolyte imbalances.**

	cryoglobulins and serum electrophoresis are all negative. Serum gamma-glutamyl transferase (GGT) levels are elevated. There is no family history of polyneuropathy.		**3. Liver Function Tests (LFTs) including AST, ALT, and Bilirubin:**<br>■ **Reasoning: Elevated GGT levels suggest liver involvement. Comprehensive LFTs can provide a fuller picture of liver function and damage extent, which can affect neurological health.**<br>**4. Homocysteine Levels:**<br>■ **Reasoning: Elevated homocysteine can be a marker for vitamin deficiencies (B6, B12, folate) and other metabolic disorders that could contribute to neuropathy. This test may offer additional insights into underlying metabolic issues.**<br>**5. Heavy Metal Screen (including lead, mercury, arsenic):**<br>■ **Reasoning: Chronic exposure to heavy metals can cause peripheral neuropathy. Testing for these metals can rule out toxic neuropathies.**<br>○ **Instrumental Tests:**<br>**1. Skin Biopsy for Intraepidermal Nerve Fiber Density (IENFD):**<br>■ **Reasoning: A skin biopsy can assess small fiber neuropathy, which may not be evident on nerve conduction studies but can cause significant sensory symptoms.**<br>**2. Quantitative Sensory Testing (QST):**<br>■ **Reasoning: QST can help quantify sensory deficits and differentiate between large and small fiber neuropathy. This non-invasive test can provide detailed sensory profiling.**<br>**3. Autonomic Function Testing (AFT):**<br>■ **Reasoning: Although the patient does not report autonomic symptoms, AFT can help detect subclinical autonomic involvement, which can be present in some neuropathies.**<br>**4. MRI of the Brain and Spine:**<br>■ **Reasoning: MRI can rule out central causes of neuropathy, such as spinal cord or brain lesions that may present with similar symptoms.**<br>**5. Lumbar Puncture with Cerebrospinal Fluid (CSF) Analysis:**<br>■ **Reasoning: CSF analysis can help rule out infectious, inflammatory, and paraneoplastic causes of neuropathy by detecting abnormal cells, proteins, or antibodies.**<br>**3. Most Crucial Test:**<br>**Thiamine (Vitamin B1) Levels**<br>○ **Reasoning: Given the patient's history of high alcohol consumption and the associated risk of thiamine deficiency leading to Wernicke-Korsakoff syndrome and other neuropathies, measuring thiamine levels is crucial. Identifying a thiamine deficiency is directly actionable with supplementation, potentially reversing or halting the progression of neuropathy. This test is highly relevant to the patient's history and can significantly impact management and prognosis.**<br>**Conclusion:**<br>**To narrow down the differential diagnoses for this patient's chronic, sensory, length-dependent polyneuropathy, additional laboratory and instrumental tests are necessary. The most crucial test among these is the measurement of thiamine (Vitamin B1) levels due to its strong association with chronic alcohol consumption and potential for causing reversible neuropathy.**
32	An 80-year-old male patient presents with a 3-year history of neuropathic pain and skin redness in the hands and feet. Symptoms started insidiously but progressed over the years. Clinical examination reveals hyperalgesia and allodynia in the hands and feet, where the skin is warm and red. There are no weakness, deficit in tactile sensation, sensory ataxia, tremor, foot deformities, cranial nerve or autonomic involvement. Past medical history is positive for prostate cancer and chronic renal insufficiency. Nerve conduction studies are normal. Screening lab tests including vitamin B12, folate, vitamin B6, vitamin E, HBV, HCV, ANA, ANCA, ENA, CRP, ESR, glycemia, HbA1c, copper, cryoglobulins and serum electrophoresis are negative. There is no family history of polyneuropathy.	Small fibre neuropathy	**Step-by-Step Analysis:**<br>**1. Review of Current Clinical Data:**<br>○ **Symptoms: Neuropathic pain and skin redness in hands and feet, hyperalgesia, and allodynia.**<br>○ **Medical History: Prostate cancer, chronic renal insufficiency.**<br>○ **Examination: Warm and red skin in affected areas, no weakness, no sensory ataxia, no tremor, no cranial nerve or autonomic involvement.**<br>○ **Investigations: Normal nerve conduction studies, negative screening lab tests (B12, folate, B6, vitamin E, HBV, HCV, ANA, ANCA, ENA, CRP, ESR, glycemia, HbA1c, copper, cryoglobulins, and serum electrophoresis), no family history of polyneuropathy.**<br>**2. Identification of Differential Diagnoses:**<br>○ **Erythromelalgia**<br>○ **Small Fiber Neuropathy (SFN)**<br>○ **Paraneoplastic Neuropathy**<br>**3. Additional Examinations Needed:**<br>**1. Skin Biopsy with Intraepidermal Nerve Fiber Density (IENFD) Analysis:**<br>● **Reasoning:**<br>○ **This test can help diagnose Small Fiber Neuropathy by quantifying the density of small nerve fibers in the skin. SFN often involves a reduction in these fibers.**<br>○ **It can also rule out erythromelalgia and other neuropathies by assessing the condition of small fibers.**<br>**2. Thermoregulatory Sweat Test (TST):**<br>● **Reasoning:**<br>○ **This test evaluates autonomic nerve function by assessing sweat production in response to a controlled environment.**<br>○ **It helps in diagnosing SFN and can also detect autonomic involvement that may not be clinically apparent.**<br>**3. Serum and Urine Protein Electrophoresis with Immunofixation:**<br>● **Reasoning:**<br>○ **This test can identify monoclonal gammopathies which are associated with paraneoplastic syndromes and certain types of neuropathies.**<br>○ **Considering the patient's history of prostate cancer, this test can help identify a paraneoplastic etiology.**<br>**4. Genetic Testing for Familial Erythromelalgia:**<br>● **Reasoning:**<br>○ **Despite the lack of family history, genetic mutations (e.g., SCN9A mutation) can sporadically occur and cause erythromelalgia.**<br>○ **This test can confirm a diagnosis of erythromelalgia if a genetic predisposition is found.**<br>**5. Quantitative Sensory Testing (QST):**<br>● **Reasoning:**<br>○ **QST assesses the sensory threshold for different stimuli (thermal, vibratory, pain), which helps evaluate the function of small and large nerve fibers.**<br>○ **It aids in diagnosing SFN and distinguishing it from other neuropathies.**<br>**6. Paraneoplastic Antibody Panel:**<br>● **Reasoning:**<br>○ **This test checks for the presence of antibodies associated with paraneoplastic syndromes.**<br>○ **It is particularly relevant given the patient's history of prostate cancer, as it can reveal a paraneoplastic etiology.**<br>**Most Crucial Test:**

			**Skin Biopsy with Intraepidermal Nerve Fiber Density (IENFD) Analysis is the most crucial test from the list.**<br>**Reasoning:**<br>● **It provides direct evidence of small fiber pathology, which is central to diagnosing Small Fiber Neuropathy (SFN), a leading differential in this case.**<br>● **It can also help rule out other conditions by showing the status of small nerve fibers, thus narrowing the differential diagnosis significantly.**<br>● **Given the patient's symptoms and normal nerve conduction studies, this test is likely to yield the most definitive and actionable information.**
33	A 24-year old male patient presents with a 4-year history of sensory-motor, symmetrical, length dependent polyneuropathy with insidious onset and slow progression. The patient presents with severe walking difficulties, bilateral foot-drop, severe distal atrophy in the lower limbs, and hypoestesia up to calves. There is no cranial nerve or autonomic involvement. Past medical history is silent. Nerve conduction studies show a mixed axonal-demyelinating, symmetric, sensory-motor polyneuropathy. Screening lab tests including vitamin B12, folate, vitamin B6, vitamin E, HBV, HCV, glycemia, HbA1c, ANA, ANCA, ENA, CRP, ESR, copper, cryoglobulins, and serum electrophoresis are all negative. His mother and sister have mild walking difficulties.	X-linked CMT	**Step-by-Step Approach for Additional Examinations:**<br>**1. Genetic Testing**<br>● **Reasoning: Given the strong family history and clinical presentation suggestive of a hereditary neuropathy, genetic testing for Charcot-Marie-Tooth (CMT) disease and other hereditary neuropathies is warranted. This will help identify specific genetic mutations associated with CMT and confirm the diagnosis.**<br>● **Specific Tests:**<br>○ **CMT panel (includes common mutations in PMP22, MPZ, GJB1, MFN2, etc.)**<br>○ **Whole exome sequencing if initial panel is inconclusive**<br>**2. Nerve Biopsy**<br>● **Reasoning: A nerve biopsy can provide histopathological evidence of demyelination, remyelination, and axonal loss, which can help differentiate between hereditary neuropathies and acquired conditions like CIDP. This is especially useful if genetic tests are inconclusive.**<br>● **Specific Tests:**<br>○ **Sural nerve biopsy with light microscopy and electron microscopy**<br>**3. Cerebrospinal Fluid (CSF) Analysis**<br>● **Reasoning: CSF analysis can help differentiate between hereditary neuropathies and CIDP. Elevated CSF protein without pleocytosis is typical of CIDP. Normal CSF findings are more common in hereditary neuropathies.**<br>● **Specific Tests:**<br>○ **CSF protein and cell count**<br>**4. Electromyography (EMG) and Repeated Nerve Conduction Studies**<br>● **Reasoning: While initial nerve conduction studies have been performed, repeating these along with EMG can help in further characterizing the pattern of neuropathy and monitoring progression. EMG can provide additional information on muscle involvement.**<br>● **Specific Tests:**<br>○ **EMG**<br>○ **Repeat nerve conduction studies with more comprehensive testing**<br>**5. Advanced Imaging (MRI)**<br>● **Reasoning: MRI of the spine and lower limbs can help assess for structural causes of neuropathy and can show nerve root or plexus involvement, which might be seen in some acquired neuropathies. It can also show muscle atrophy and fatty replacement typical in hereditary neuropathies.**<br>● **Specific Tests:**<br>○ **MRI of the lumbosacral spine and lower limbs**<br>**6. Autonomic Testing**<br>● **Reasoning: Although there is no cranial nerve or autonomic involvement reported, comprehensive autonomic testing can help rule out subtle autonomic dysfunction that might be missed on clinical examination alone. This is more relevant to differentiate certain types of neuropathies.**<br>● **Specific Tests:**<br>○ **Quantitative sudomotor axon reflex test (QSART)**<br>○ **Heart rate variability test**<br>○ **Tilt table test**<br>**Most Crucial Test:**<br>**The most crucial test from this list is Genetic Testing (CMT panel and possibly whole exome sequencing). Given the clinical presentation and strong family history suggestive of a hereditary neuropathy, identifying a specific genetic mutation would provide a definitive diagnosis and guide management. Genetic testing directly targets the most likely diagnosis based on the provided clinical information and family history, making it the highest yield investigation.**<br>**Summary of Additional Examinations:**<br>**1. Genetic Testing (CMT panel and possibly whole exome sequencing)**<br>**2. Nerve Biopsy (sural nerve)**<br>**3. CSF Analysis (protein and cell count)**<br>**4. Electromyography (EMG) and Repeated Nerve Conduction Studies**<br>**5. MRI of the lumbosacral spine and lower limbs**<br>**6. Autonomic Testing (QSART, heart rate variability, tilt table test)**<br>**These tests will provide a comprehensive evaluation and help narrow down the differential diagnoses, with genetic testing being the most critical in confirming the suspected diagnosis of Charcot-Marie-Tooth disease.**
34	An 83-year-old male patient presents with a 1-year history of pure sensory, symmetrical, length dependent polyneuropathy with insidious onset and progressive course. The patient presents with distal paresthesia and hypoesthesia in the four limbs, mild hand tremor and cramps but no neuropathic pain. He also reports the appearance of gait instability in the past few months. Clinical examination confirms the presence of sensory ataxia with difficulty walking without support.	Vit B12 deficiency neuropathy	**Step-by-Step Analysis of Additional Examinations**<br>**1. Serum Immunofixation Electrophoresis (IFE) and Free Light Chain (FLC) Assay**<br>○ **Reasoning:**<br>■ **Although IgG kappa monoclonal gammopathy is already identified, these tests provide more detailed information about the monoclonal protein and its free light chain levels, which helps in confirming MGUS and distinguishing it from more serious conditions like multiple myeloma or AL amyloidosis.**<br>○ **Purpose: To quantify and characterize the monoclonal protein and assess the risk of progression to a malignancy.**<br>**2. Bone Marrow Biopsy**<br>○ **Reasoning:**<br>■ **This test can provide definitive evidence of clonal plasma cells or other hematologic malignancies. It's important for ruling out multiple myeloma or other plasma cell disorders.**<br>○ **Purpose: To confirm the diagnosis of MGUS and exclude multiple myeloma or other related disorders.**<br>**3. Lumbar Puncture with Cerebrospinal Fluid (CSF) Analysis**<br>○ **Reasoning:**

	There are no foot deformities, cranial nerve or autonomic involvement. Past medical history shows IgG kappa monoclonal gammopathy, thrombocytopenia, and prostatic cancer. Nerve conduction studies show a pure sensory, symmetric, length-dependent, axonal polyneuropathy. Screening lab tests show the presence of IgG kappa paraprotein and evidence of vitamin B12 deficiency. Remaining exams including folate, vitamin B6, vitamin E, HCV, HBV, ANA, ENA, ANCA, CRP, ESR, glycemia, HbA1c, copper and cryoglobulins are all negative. There is no family history of polyneuropathy.		■ **CSF analysis can help detect the presence of an inflammatory or infectious cause of neuropathy, such as chronic inflammatory demyelinating polyneuropathy (CIDP) or other autoimmune neuropathies, which might not be obvious from blood tests alone.**<br>○ **Purpose: To evaluate for inflammatory, infectious, or malignant cells in the CSF that might contribute to neuropathy.**<br>**4. Magnetic Resonance Imaging (MRI) of the Spine**<br>○ **Reasoning:**<br>■ **MRI can help identify structural abnormalities or compressive lesions that might contribute to neuropathy. It also helps to rule out any paraneoplastic syndromes involving the central nervous system or spinal cord.**<br>○ **Purpose: To visualize the spine for any structural issues, tumors, or other abnormalities that could cause or contribute to the patient's symptoms.**<br>**5. Electromyography (EMG) and Repeat Nerve Conduction Studies (NCS)**<br>○ **Reasoning:**<br>■ **Repeat and more detailed NCS/EMG can help differentiate between demyelinating and axonal processes more clearly and assess the extent of nerve damage.**<br>○ **Purpose: To provide a detailed assessment of the type and extent of neuropathy and help guide treatment.**<br>**6. Antibody Testing for Paraneoplastic Syndromes**<br>○ **Reasoning:**<br>■ **Given the history of prostatic cancer, testing for paraneoplastic antibodies can help identify a paraneoplastic neuropathy that might be associated with cancer.**<br>○ **Purpose: To detect paraneoplastic antibodies that might indicate an immune response associated with the patient's cancer.**<br>**7. Vitamin B12 and Methylmalonic Acid (MMA) Levels**<br>○ **Reasoning:**<br>■ **Confirming vitamin B12 deficiency with MMA levels, as elevated MMA levels indicate a functional B12 deficiency even if B12 levels appear borderline.**<br>○ **Purpose: To ensure that vitamin B12 deficiency is accurately diagnosed and appropriately treated.**<br>**8. Serum Protein Electrophoresis (SPEP) and Urine Protein Electrophoresis (UPEP)**<br>○ **Reasoning:**<br>■ **These tests help quantify monoclonal protein in both serum and urine, providing further information on the extent of monoclonal gammopathy.**<br>○ **Purpose: To further characterize and quantify monoclonal proteins and assess the potential kidney involvement.**<br>**Determining the Most Crucial Test**<br>**Most Crucial Test: Bone Marrow Biopsy**<br>**Reasoning:**<br>● **A bone marrow biopsy is crucial because it provides definitive information about the presence and percentage of clonal plasma cells or other abnormal cells. This test can confirm MGUS and rule out other serious hematologic conditions such as multiple myeloma or amyloidosis. Given the patient's monoclonal gammopathy and significant neuropathy, excluding these serious conditions is paramount for accurate diagnosis and appropriate management.**<br>**This step ensures a thorough investigation and narrows down the differential diagnosis while confirming the underlying cause of the neuropathy.**
35	An 82-year-old male patient presents with a 3-year history of pure sensory, symmetrical, length dependent polyneuropathy with insidious onset and slow progressive course. The patient presents with paraesthesia and hypoesthesia in the feet. Clinical examination reveals hyperalgesia during pinprick testing and tactile hypoesthesia in the feet, while there are no weakness, sensory ataxia, tremor, foot deformities, cranial nerve or autonomic dysfunction. Past medical history is positive for IgG kappa MGUS, prostate and bladder cancer, for which he underwent chemotherapy five years ago. Nerve conduction studies show a sensory-motor, symmetric, length dependent, axonal polyneuropathy. Screening lab tests reveal presence of IgG kappa paraprotein. Remaining exams including vitamin B12, folate, vitamin B6, vitamin E, HBV, HCV, glycaemia, HbA1c, ANA, ANCA, ENA, CRP, ESR, copper ,and cryoglobulins are normal. There is no family history of polyneuropathy.	Paraproteinemic neuropathy	**Step-by-Step Analysis of Additional Examinations**<br>**Step 1: Detailed Review of Case Summary**<br>● **Patient Profile:**<br>○ **82-year-old male with a history of pure sensory, symmetrical, length-dependent polyneuropathy.**<br>○ **Known IgG kappa MGUS and past chemotherapy for prostate and bladder cancer.**<br>● **Symptoms:**<br>○ **Paraesthesia and hypoesthesia in the feet.**<br>○ **Hyperalgesia during pinprick testing and tactile hypoesthesia.**<br>● **Nerve Conduction Studies:**<br>○ **Sensory-motor, symmetric, length-dependent, axonal polyneuropathy.**<br>● **Lab Tests:**<br>○ **Presence of IgG kappa paraprotein.**<br>○ **Normal levels of vitamin B12, folate, vitamin B6, vitamin E, HBV, HCV, glycaemia, HbA1c, ANA, ANCA, ENA, CRP, ESR, copper, and cryoglobulins.**<br>**Step 2: Additional Examinations**<br>**1. Serum Free Light Chains (FLC) and Immunofixation Electrophoresis:**<br>○ **Reasoning: These tests help in further characterizing the monoclonal protein detected and are crucial for diagnosing conditions like multiple myeloma or light chain amyloidosis, which can present with neuropathy.**<br>○ **Purpose: To assess the risk of progression from MGUS to a malignant plasma cell disorder.**<br>**2. Bone Marrow Biopsy:**<br>○ **Reasoning: Given the presence of IgG kappa paraprotein and history of MGUS, a bone marrow biopsy can determine if there is any underlying hematologic malignancy such as multiple myeloma.**<br>○ **Purpose: To rule out or confirm hematologic malignancies.**<br>**3. Anti-MAG (Myelin-Associated Glycoprotein) Antibodies:**<br>○ **Reasoning: Anti-MAG antibodies are often associated with demyelinating neuropathies in patients with paraproteinemias.**<br>○ **Purpose: To determine if there is an immune-mediated component to the neuropathy.**<br>**4. Autonomic Testing (e.g., QSART, Tilt Table Test):**<br>○ **Reasoning: Despite the lack of reported autonomic symptoms, a thorough evaluation can reveal subclinical autonomic dysfunction, which can be associated with certain neuropathies.**<br>○ **Purpose: To evaluate the autonomic nervous system involvement, which can alter management.**<br>**5. Skin Biopsy for Intraepidermal Nerve Fiber Density (IENFD):**<br>○ **Reasoning: Skin biopsy can quantify small fiber neuropathy, which might not be detected on routine nerve conduction studies.**<br>○ **Purpose: To assess small fiber involvement in neuropathy.**<br>**6. PET/CT Scan:**<br>○ **Reasoning: To evaluate for any malignancy recurrence or metastasis, particularly given the history of prostate and bladder cancer.**<br>○ **Purpose: To rule out cancer recurrence contributing to the neuropathy.**

<table>
<tr><td></td><td></td><td></td><td>

7. **Genetic Testing for Hereditary Neuropathies:**
   - **Reasoning: Although there is no family history, late-onset hereditary neuropathies can occur sporadically.**
   - **Purpose: To exclude genetic causes of neuropathy.**

Step 3: Most Crucial Test
Bone Marrow Biopsy

- **Reasoning: Given the presence of IgG kappa paraprotein and the patient's history of MGUS, a bone marrow biopsy is crucial. It helps in diagnosing potential malignant transformation to multiple myeloma or other hematologic malignancies, which can present with neuropathy. Early detection and treatment can significantly affect the patient's prognosis and management plan.**

Conclusion
The bone marrow biopsy is the most crucial test in this clinical scenario. It directly addresses the potential for malignant progression from MGUS, which is highly relevant given the patient's symptoms, history, and the presence of paraprotein.
</td></tr>
<tr><td>36</td><td>A 75-year-old male patient presents with a four-year history of pure sensory, symmetrical, length-dependent polyneuropathy with insidious onset and slow progression. The patient reports sensory ataxia, hypoesthesia in the distal lower limbs up to the calves, mild dysphagia and dysarthria, with no distal weakness. Past medical history is positive for chronic cough. Nerve conduction studies show an axonal, symmetric, length-dependent, pure sensory polyneuropathy. Screening lab tests, including vitamin B12, folate, vitamin B6, vitamin E, HBV, HCV, glycemia, HbA1c, ANA, ANCA, CRP, ESR, copper, cryoglobulins, and serum electrophoresis are all negative, with positivity for ENA autoantibodies. There is no family history of polyneuropathy.</td><td>CANVAS</td><td>

Additional Examinations for Further Analysis

1. **CT Scan of the Chest:**
   - **Reasoning: Given the patient's history of chronic cough and the suspicion of paraneoplastic syndrome, a CT scan of the chest can help identify potential malignancies such as lung cancer, which is commonly associated with paraneoplastic syndromes.**
2. **Positron Emission Tomography (PET) Scan:**
   - **Reasoning: A PET scan is highly sensitive for detecting malignancies throughout the body. It can identify both primary tumors and metastatic disease, which would support the diagnosis of a paraneoplastic syndrome.**
3. **Serum and Urine Protein Electrophoresis with Immunofixation:**
   - **Reasoning: To rule out multiple myeloma or other monoclonal gammopathies, which can also present with sensory polyneuropathy. Although initial serum electrophoresis was negative, immunofixation provides greater sensitivity.**
4. **Anti-Hu, Anti-Ri, and Anti-Yo Antibodies:**
   - **Reasoning: These are specific paraneoplastic antibodies often associated with sensory neuropathies and various malignancies, particularly small cell lung cancer and other solid tumors.**
5. **Salivary Gland Biopsy or Lip Biopsy:**
   - **Reasoning: If Sjogren's syndrome is suspected, a salivary gland or lip biopsy can confirm the presence of lymphocytic infiltrates characteristic of this autoimmune disease, despite the ENA positivity.**
6. **High-Resolution Esophageal Manometry and Videofluoroscopic Swallow Study:**
   - **Reasoning: To evaluate the degree and nature of dysphagia and dysarthria. This can help in understanding whether these symptoms are due to neuropathy or other underlying structural or functional issues.**
7. **Repeat Nerve Conduction Studies and Electromyography (EMG):**
   - **Reasoning: Further evaluation of the extent and progression of the neuropathy, looking specifically for any evolving features that might suggest a different type of neuropathy, such as a mixed or motor component.**
8. **Lumbar Puncture with Cerebrospinal Fluid (CSF) Analysis:**
   - **Reasoning: To rule out chronic inflammatory demyelinating polyradiculoneuropathy (CIDP) or other inflammatory neuropathies that might not be detected by serum studies alone. CSF analysis can reveal elevated protein levels and other markers indicative of inflammation or malignancy-related processes.**

Conclusion: Most Crucial Test
CT Scan of the Chest
Given the clinical presentation, history of chronic cough, and the high suspicion of a paraneoplastic syndrome, the most crucial test is a CT scan of the chest. This imaging modality is essential for identifying potential underlying malignancies, particularly lung cancer, which is a common cause of paraneoplastic sensory neuropathy. Detecting a primary tumor can significantly narrow the differential diagnosis and guide further management, including oncological assessment and treatment.
</td></tr>
<tr><td>37</td><td>A 58-year-old female patient presents with a 13-month history of sensory-motor, symmetrical, length dependent polyneuropathy with subacute onset and progressive course. The patient presents with distal weakness, neuropathic pain, and paresthesia in the four limbs. Clinical examination shows the presence of mild intrinsic hand atrophy and a peripheral edema. There are no sensory ataxia, tremor, foot deformities, cranial nerve or autonomic involvement. Past medical history is positive for occasional alcohol use. Nerve conduction studies show a mixed demyelinating-axonal, length dependent, sensory-motor polyneuropathy. Screening lab tests including vitamin B12, folate, vitamin B6, vitamin E, HCV, HBV, ANA, ANCA, ENA, CRP, ESR, glycemia, HbA1c, copper, and cryoglobulins are negative, while serum electrophoresis shows positivity for IgG lambda paraprotein. A cardiac ultrasound, performed to investigate edema in the lower limbs, revealed the presence of pericardial effusion. There is no family history of polyneuropathy.</td><td>POEMS</td><td>

Step-by-Step Analysis of Additional Examinations
1. Bone Marrow Biopsy and Aspiration
Reasoning:

- **To evaluate for plasma cell dyscrasias such as multiple myeloma or other hematologic malignancies.**
- **The presence of IgG lambda paraprotein raises suspicion for a monoclonal gammopathy, which can sometimes progress to multiple myeloma or be part of other malignancies.**
- **A bone marrow biopsy will help confirm or rule out these conditions.**

2. Serum Free Light Chain Assay
Reasoning:

- **To quantify free kappa and lambda light chains and assess the kappa/lambda ratio.**
- **This test is useful in identifying and monitoring monoclonal plasma cell disorders, especially in the context of MGUS, multiple myeloma, or AL amyloidosis.**
- **Abnormalities in free light chains can support the diagnosis and provide information about disease burden.**

3. CT Scan or MRI of Chest, Abdomen, and Pelvis
Reasoning:

- **To evaluate for organomegaly, lymphadenopathy, or other structural abnormalities that may be associated with POEMS syndrome or other malignancies.**
- **This imaging will help detect any organ involvement that was not apparent on clinical examination.**

4. Echocardiogram
Reasoning:

- **Given the presence of pericardial effusion, a detailed echocardiogram can provide more information on cardiac function and the extent of effusion.**
- **This can help determine if there is any underlying cardiac involvement, which can be seen in conditions like amyloidosis or POEMS syndrome.**

5. Skin Biopsy
Reasoning:

- **If any skin changes are observed upon further detailed examination, a skin biopsy can help identify amyloid deposits or other histological abnormalities.**
- **Skin biopsy can be useful in diagnosing conditions like amyloidosis, which can present with neuropathy and other systemic features.**

6. CSF Analysis
Reasoning:

- **To evaluate for the presence of elevated protein or malignant cells, which can be seen in CIDP and other inflammatory or neoplastic processes.**
- **CSF analysis can provide additional diagnostic clues in cases of suspected CIDP or CNS involvement in systemic diseases.**

7. Electromyography (EMG)
Reasoning:
</td></tr>
</table>

			● To complement nerve conduction studies and provide further information on the type and extent of neuromuscular involvement.<br>● EMG can help differentiate between primary nerve and muscle pathology, which is crucial in comprehensive neuropathy assessment.<br>Most Crucial Test<br>Bone Marrow Biopsy and Aspiration<br>Reasoning:<br>● This test is pivotal in evaluating the source and nature of the monoclonal protein detected in serum electrophoresis.<br>● A bone marrow biopsy can confirm or rule out multiple myeloma, MGUS, and other hematologic conditions, providing a definitive diagnosis or significantly narrowing down the differential diagnoses.<br>● Given the potential for serious underlying conditions like multiple myeloma or amyloidosis that require prompt treatment, this test is the most critical to perform.<br>In summary, while multiple tests are necessary to fully elucidate the underlying cause of the polyneuropathy, the bone marrow biopsy and aspiration stand out as the most crucial due to their direct ability to identify or rule out hematologic malignancies and other serious conditions associated with monoclonal gammopathy.
38	A 30-year-old female patient presents with a six-month history of right peroneal mononeuropathy (foot drop) with acute onset. She also reports the presence of mild paresthesia in the fourth and fifth fingers of the left hand over the past two years, and frequent hand paresthesia after using scissors at work. Nerve conduction studies show a mild demyelinating, sensory-motor polyneuropathy, with right carpal tunnel syndrome, left ulnar entrapment at the elbow, and right peroneal nerve entrapment at the fibular head. Screening lab tests including vitamin B12, folate, vitamin B6, vitamin E, HBV, HCV, glycemia, HbA1c, ANA, ANCA, ENA, CRP, ESR, copper, cryoglobulins, and serum electrophoresis are all negative. There is no family history of polyneuropathy.	HNPP	Step-by-Step Analysis of Additional Examinations<br>To narrow down the differential diagnoses for this patient with polyneuropathy, further diagnostic tests are necessary. Here is a structured approach to determine the necessary additional examinations:<br>Additional Laboratory Tests<br>1. Genetic Testing for PMP22 Gene Deletion/Duplication<br>○ Reasoning: Given the high suspicion for Hereditary Neuropathy with Liability to Pressure Palsies (HNPP), testing for deletions in the PMP22 gene, which is implicated in HNPP, would be crucial. Identifying this genetic mutation would confirm the diagnosis.<br>2. Anti-GM1 Antibody Testing<br>○ Reasoning: Anti-GM1 antibodies are often elevated in Multifocal Motor Neuropathy (MMN). Although MMN is less likely, this test can help rule it out or support its diagnosis.<br>3. CSF Analysis (Lumbar Puncture)<br>○ Reasoning: For Chronic Inflammatory Demyelinating Polyneuropathy (CIDP), cerebrospinal fluid (CSF) analysis often shows elevated protein levels without pleocytosis. This can support or rule out CIDP.<br>4. Repeating Serum Electrophoresis and Immunofixation<br>○ Reasoning: While initial tests were negative, repeating serum electrophoresis and immunofixation might be helpful to rule out any potential late-onset monoclonal gammopathy or multiple myeloma that could be causing a polyneuropathy.<br>Additional Instrumental Tests<br>5. MRI of the Brain and Spine<br>○ Reasoning: To rule out any structural abnormalities or lesions in the central nervous system that might contribute to the patient's symptoms. MRI can also help detect nerve root or plexus involvement that might not be evident in nerve conduction studies.<br>6. High-Resolution Ultrasonography of Peripheral Nerves<br>○ Reasoning: This imaging technique can provide detailed visualization of nerve morphology and identify sites of nerve compression or focal nerve enlargement which can support the diagnosis of entrapment neuropathies or HNPP.<br>7. Skin Biopsy with Intraepidermal Nerve Fiber Density (IENFD) Analysis<br>○ Reasoning: This test can help in the assessment of small fiber neuropathy which might not be detected in nerve conduction studies but can coexist with large fiber neuropathies.<br>Most Crucial Test<br>Genetic Testing for PMP22 Gene Deletion/Duplication is the most crucial test. Given the clinical presentation of multiple entrapment neuropathies and the demyelinating pattern on nerve conduction studies, confirming the diagnosis of HNPP through genetic testing will provide definitive evidence and guide appropriate management.<br>Summary of Proposed Additional Examinations<br>1. Genetic Testing for PMP22 Gene Deletion/Duplication<br>2. Anti-GM1 Antibody Testing<br>3. CSF Analysis (Lumbar Puncture)<br>4. Repeating Serum Electrophoresis and Immunofixation<br>5. MRI of the Brain and Spine<br>6. High-Resolution Ultrasonography of Peripheral Nerves<br>7. Skin Biopsy with IENFD Analysis<br>Conclusion: The most crucial test from this list is the Genetic Testing for PMP22 Gene Deletion/Duplication, which can definitively confirm the diagnosis of HNPP, the most likely cause of the patient's symptoms based on the initial clinical scenario.
39	A 76-year-old male patient presents with an 8-year history of chronic, pure sensory, symmetrical, length dependent polyneuropathy with insidious onset and slow progressive course. The patient presents with severe neuropathic pain and heat intolerance in the feet. He does not report weakness, ataxia, paresthesia, tremor, cranial nerve or autonomic involvement. Clinical examination reveals the presence of allodynia and hyperalgesia in the feet. There are no foot deformities. Past medical history is positive for prostatic cancer. Nerve conduction studies are normal. Screening lab tests including vitamin B12, folate, vitamin B6, vitamin E, HBV, HCV, ANA, ANCA, ENA, CRP, ESR, glycemia, HbA1c, copper, cryoglobulins,	Small fibre neuropathy	Step-by-Step Analysis and Additional Examinations:<br>1. Step 1: Review Initial Findings and Identify Gaps<br>○ The patient has a chronic, pure sensory, symmetrical, length-dependent polyneuropathy.<br>○ Negative screening lab tests and normal nerve conduction studies.<br>○ History of prostatic cancer.<br>2. Step 2: Consider Differential Diagnoses<br>○ Paraneoplastic neuropathy<br>○ Idiopathic Small Fiber Neuropathy (SFN)<br>○ Chemotherapy-induced neuropathy<br>3. Step 3: Identify Necessary Additional Examinations<br>a. Skin Biopsy for Intraepidermal Nerve Fiber Density (IENFD)<br>○ Reasoning: This test can help confirm the diagnosis of small fiber neuropathy by measuring the density of small nerve fibers in the skin. It is particularly useful in cases where nerve conduction studies are normal, as it targets the small fibers that are typically involved in SFN.<br>○ Clinical Relevance: Given the patient's symptoms of severe neuropathic pain and heat intolerance, confirming small fiber involvement is critical.<br>4. b. Quantitative Sensory Testing (QST)<br>○ Reasoning: QST evaluates the function of small and large sensory nerve fibers by assessing responses to controlled thermal and mechanical stimuli. This can provide objective evidence of sensory dysfunction.<br>○ Clinical Relevance: This test can help quantify the extent of sensory loss and hyperalgesia, which aligns with the patient's symptoms.<br>5. c. Paraneoplastic Antibody Panel

	and serum electrophoresis are negative. There is no family history of polyneuropathy.		○ Reasoning: This panel can detect antibodies associated with paraneoplastic syndromes, which can occur in patients with cancer. Identifying these antibodies can help confirm or rule out a paraneoplastic cause of the neuropathy.<br>○ Clinical Relevance: Given the patient's history of prostatic cancer, this test is important to explore potential paraneoplastic etiologies.<br>6. d. Autonomic Function Testing<br>○ Reasoning: Even though the patient does not report autonomic symptoms, autonomic function testing can sometimes reveal subclinical autonomic involvement, which can help in the diagnosis of certain neuropathies.<br>○ Clinical Relevance: This test can provide additional information about the involvement of small fibers, which can be affected in SFN.<br>7. e. Repeat Comprehensive Metabolic Panel and Electrolytes<br>○ Reasoning: Some metabolic disorders can cause neuropathy and might not have been fully ruled out. A repeat and comprehensive review of metabolic panel and electrolytes can identify any missed abnormalities.<br>○ Clinical Relevance: Ensures no secondary causes of neuropathy are overlooked.<br>8. f. Genetic Testing for Hereditary Neuropathies<br>○ Reasoning: Although there is no family history, genetic testing can sometimes reveal mutations associated with hereditary sensory neuropathies that can present later in life.<br>○ Clinical Relevance: Helps rule out or confirm a genetic etiology, particularly in idiopathic cases.<br>Most Crucial Test:<br>● Skin Biopsy for Intraepidermal Nerve Fiber Density (IENFD)<br>○ Rationale: This test directly assesses the small nerve fibers in the skin, which are implicated in the patient's symptoms. It provides definitive evidence of small fiber neuropathy, especially when nerve conduction studies are normal. Confirming small fiber neuropathy will significantly narrow down the differential diagnosis and guide appropriate management.
40	A 79-year-old male patient presents with a 2-year history of pure sensory, symmetrical, length dependent polyneuropathy with insidious onset. The patient presents with paresthesia, hypoesthesia and neuropathic pain in the distal areas of the lower limbs. There are no ataxia, tremor, foot deformities, cranial nerve or autonomic involvement. Past medical history is silent. Nerve conduction studies show pure sensory, axonal polyneuropathy. Screening lab tests including vitamin B12, folate, vitamin B6, vitamin E, HCV, ANA, ANCA, ENA, CRP, ESR, glycemia, HbA1c, copper, cryoglobulins and serum electrophoresis are all negative, with evidence of chronic HBV infection. There is no family history of polyneuropathy.	HBV-associated neuropathy	Step-by-Step Approach to Analysis and Additional Examinations<br>Step 1: Review Clinical Presentation and Current Workup<br>● Symptoms: Sensory, symmetrical, length-dependent polyneuropathy with paresthesia, hypoesthesia, and neuropathic pain in distal lower limbs.<br>● Nerve Conduction Studies: Pure sensory, axonal polyneuropathy.<br>● Lab Results: Negative for common causes (vitamin deficiencies, infections, autoimmune markers, diabetes, cryoglobulins), positive for chronic HBV infection.<br>● No family history of polyneuropathy.<br>Step 2: Identify Gaps in Current Workup<br>● While common causes have been ruled out, further specific tests might be necessary to explore less common causes and confirm the most likely diagnosis.<br>Step 3: Propose Additional Examinations<br>1. HBV DNA Quantification and Liver Function Tests (LFTs)<br>○ Reasoning: Chronic HBV infection is a known factor, and quantifying viral load along with assessing liver function (ALT, AST, ALP, bilirubin) can help determine the extent of viral activity and liver involvement, which can impact the severity and progression of neuropathy.<br>2. Cancer Screening<br>○ Reasoning: Given the patient's age and the possibility of paraneoplastic syndromes causing sensory neuropathy, screening for underlying malignancies is crucial. This could include:<br>■ Chest X-ray or CT Scan: To detect lung cancer, particularly small cell lung cancer.<br>■ CT/MRI of the Abdomen and Pelvis: To identify potential tumors in these regions.<br>■ PSA Test (for prostate cancer in males): Given the age of the patient.<br>■ Complete Blood Count (CBC) and Basic Metabolic Panel (BMP): To look for any hematologic malignancies or abnormalities that might suggest cancer.<br>3. Anti-Hu and Other Paraneoplastic Autoantibodies<br>○ Reasoning: Paraneoplastic syndromes can present with sensory neuropathy, and detecting specific autoantibodies can help confirm this diagnosis.<br>4. Lumbar Puncture (CSF Analysis)<br>○ Reasoning: To check for any inflammatory or infectious processes that might not be evident in blood tests. This includes looking for elevated protein levels or the presence of malignant cells.<br>5. Skin Biopsy for Intraepidermal Nerve Fiber Density (IENFD)<br>○ Reasoning: This test can help in diagnosing small fiber neuropathy, which might not be detected on nerve conduction studies but could explain the sensory symptoms.<br>6. Genetic Testing<br>○ Reasoning: Despite the lack of family history, genetic mutations can occur de novo and lead to polyneuropathy. Genetic testing for hereditary neuropathies might be considered if other tests are inconclusive.<br>7. Heavy Metal Screening (Blood and Urine)<br>○ Reasoning: Although less common, heavy metal toxicity (e.g., lead, arsenic, mercury) can cause sensory neuropathy and should be ruled out.<br>Conclusion: Most Crucial Test<br>The most crucial test from the list is:<br>HBV DNA Quantification and Liver Function Tests (LFTs)<br>● Justification:<br>○ Given the established presence of chronic HBV infection and the potential for HBV-associated neuropathy, determining the viral load and assessing liver function are critical for understanding the extent of HBV's role in the patient's neuropathy.<br>○ This test will guide further management of the HBV infection, which could directly impact the neuropathy if the infection is contributing to the condition.<br>○ Other tests, while important, are more exploratory in nature. The HBV DNA quantification and LFTs directly address a known variable in the patient's clinical picture.<br>Next Steps<br>1. Conduct HBV DNA Quantification and Liver Function Tests.<br>2. Based on the results, consider additional tests as outlined (e.g., cancer screening, paraneoplastic antibodies) to further narrow down the differential diagnoses if necessary.
41	An 82-year-old female patient presents with a 4-year history of pure sensory, length dependent, symmetric polyneuropathy with insidious onset and slow progressive course. The patient presents with paresthesia and mild neuropathic pain in the feet. Clinical examination does not show weakness or	MGUS-associated neuropathy	Step-by-Step Analysis of Additional Examinations<br>1. Serum Immunofixation Electrophoresis (IFE)<br>● Reasoning: This test is crucial to confirm the type and amount of monoclonal protein (paraprotein) present in the blood. Given the patient's known IgA kappa monoclonal gammopathy, detailed characterization of the monoclonal protein can provide further insight into its potential pathogenicity and association with neuropathy.<br>2. Bone Marrow Biopsy<br>● Reasoning: This test helps to assess the extent of plasma cell infiltration, which can differentiate MGUS from more serious conditions like multiple myeloma or Waldenström macroglobulinemia. Although the patient has been diagnosed with monoclonal gammopathy, it is essential to rule out progression to malignancy, which can influence the treatment plan and prognosis.<br>3. Serum Free Light Chains (FLC) Assay

	sensory ataxia, tremor, foot deformities, cranial nerve or autonomic involvement. Past medical history is positive for IgA kappa monoclonal gammopathy, arterial hypertension, gout, hypercholesterolemia, and cataract. Nerve conduction studies show presence of a pure sensory, length dependent, symmetric, axonal polyneuropathy. Screening lab tests including vitamin B12, folate, vitamin B6, vitamin E, HBV, HCV, glycemia, HbA1c, ANA, ANCA, ENA, CRP, ESR, copper, and cryoglobulins are negative, with positivity to IgA kappa paraprotein. There is no family history of polyneuropathy.		● Reasoning: This assay measures the free kappa and lambda light chains in the blood, which helps in identifying light chain amyloidosis or other plasma cell disorders. The ratio of kappa to lambda light chains can be particularly useful in further characterizing monoclonal gammopathies and detecting low-level monoclonal light chain production.<br>4. Lumbar Puncture (CSF Analysis)<br>● Reasoning: Cerebrospinal fluid (CSF) analysis can help to exclude chronic inflammatory demyelinating polyneuropathy (CIDP) and other inflammatory or infectious causes of neuropathy. Elevated protein levels with normal cell count (albuminocytologic dissociation) are typically seen in CIDP.<br>5. Quantitative Sensory Testing (QST)<br>● Reasoning: This test helps to evaluate sensory nerve function more precisely and can quantify the degree of sensory impairment. It provides objective data on the severity and distribution of sensory deficits, which can support the diagnosis and monitor progression.<br>6. Skin Biopsy<br>● Reasoning: Skin biopsy with assessment of intraepidermal nerve fiber density can be used to diagnose small fiber neuropathy. This can provide direct evidence of peripheral nerve involvement, especially in cases where standard nerve conduction studies might not detect small fiber dysfunction.<br>7. Antibody Testing for Paraprotein-associated Neuropathy<br>● Reasoning: Testing for anti-myelin-associated glycoprotein (anti-MAG) antibodies, which are often associated with IgM MGUS, can help to identify specific antibody-mediated neuropathies. Although the patient has IgA kappa monoclonal gammopathy, testing for a broader range of antibodies can be useful.<br>Most Crucial Test<br>Serum Immunofixation Electrophoresis (IFE)<br>The most crucial test in this scenario is Serum Immunofixation Electrophoresis (IFE). This test provides a detailed characterization of the monoclonal protein, which is essential for confirming the presence and type of paraprotein. Given the patient's IgA kappa monoclonal gammopathy and its association with neuropathy, this test will help to establish a more definitive diagnosis and guide further management. It can also identify any changes in the monoclonal protein that may indicate progression to a more severe hematologic disorder.
42	A 47-year-old male patient presents with a 1-year history of pure motor, asymmetrical multineuropathy with subacute onset. Clinical examination reveals the presence of weakness and atrophy in a multifocal distribution in the upper limbs. There are no sensory deficits or subjective numbness, tingling or pain, and physical examination does not show the presence of sensory ataxia, tremor, foot deformities, cranial nerve or autonomic involvement. Past medical history is silent. Nerve conduction studies show pure motor, demyelinating multineuropathy with nerve conduction blocks. Screening lab tests including vitamin B12, folate, vitamin B6, vitamin E, HCV, HBV, ANA, ANCA, ENA, CRP, ESR, glycemia, HbA1c, copper, cryoglobulins and serum electrophoresis are all negative. There is no family history of polyneuropathy.	MMN	Step-by-Step Analysis of the Clinical Scenario<br>Summary of the Clinical Information<br>● Patient: 47-year-old male<br>● History: 1-year history of pure motor, asymmetrical multineuropathy with subacute onset<br>● Clinical Examination: Multifocal weakness and atrophy in the upper limbs, no sensory deficits<br>● Nerve Conduction Studies: Pure motor, demyelinating multineuropathy with conduction blocks<br>● Screening Lab Tests: Negative for various potential causes (vitamin deficiencies, infections, autoimmune markers, glycemia, cryoglobulins, etc.)<br>● Family History: No family history of polyneuropathy<br>Additional Examinations Needed<br>1. Anti-GM1 Antibody Testing<br>Reasoning:<br>● MMN Association: Anti-GM1 antibodies are frequently found in patients with MMN. Detecting these antibodies can strongly support the diagnosis of MMN.<br>● Pure Motor Involvement: This test is particularly useful in cases with pure motor neuropathy and conduction blocks, aligning with the patient's presentation.<br>2. Lumbar Puncture (CSF Analysis)<br>Reasoning:<br>● CIDP Diagnosis: Elevated protein levels without pleocytosis in cerebrospinal fluid can support the diagnosis of CIDP.<br>● Rule Out Other Causes: CSF analysis can also help exclude infections or other inflammatory conditions affecting the nervous system.<br>3. MRI of the Brachial Plexus<br>Reasoning:<br>● Structural Causes: MRI can help identify structural lesions or nerve root compressions that could explain multifocal motor neuropathy.<br>● Inflammatory Changes: In CIDP and MMN, MRI may show nerve root or plexus enlargement, which can be helpful for diagnosis.<br>4. Paraneoplastic Panel (including onconeural antibodies)<br>Reasoning:<br>● Paraneoplastic Syndromes: Screening for onconeural antibodies can identify paraneoplastic etiologies, which can present with similar motor neuropathy features.<br>● Thorough Screening: Given the patient's age and presentation, ruling out an occult malignancy is prudent.<br>5. Genetic Testing<br>Reasoning:<br>● Hereditary Neuropathies: Although there is no family history, some genetic mutations can present de novo and cause motor neuropathies. Testing for mutations linked to hereditary motor neuropathies can be considered if other tests are inconclusive.<br>Most Crucial Test<br>The most crucial test from the list is Anti-GM1 Antibody Testing. This test is specifically targeted towards confirming MMN, which is the most likely diagnosis based on the clinical presentation and electrophysiological findings. Positive results for anti-GM1 antibodies would strongly support the diagnosis of MMN, allowing for targeted therapy to begin promptly.<br>Summary of Necessary Additional Examinations<br>1. Anti-GM1 Antibody Testing - To support diagnosis of MMN.<br>2. Lumbar Puncture (CSF Analysis) - To check for elevated protein levels and rule out infections or inflammatory conditions.<br>3. MRI of the Brachial Plexus - To identify structural or inflammatory changes in the nerves.<br>4. Paraneoplastic Panel (including onconeural antibodies) - To screen for paraneoplastic syndromes.<br>5. Genetic Testing - To investigate possible hereditary motor neuropathies.<br>Conclusion<br>The additional examinations listed above are crucial in narrowing down the differential diagnoses. However, Anti-GM1 Antibody Testing stands out as the most crucial test due to its high specificity for MMN, which remains the most probable diagnosis based on the current clinical scenario.
43	An 88-year-old male patient presents with a 4-year history of sensory-motor, symmetrical, length dependent	Diabetic neuropathy	Step-by-Step Analysis of Additional Examinations<br>1. Detailed Clinical Neurological Examination

	polyneuropathy with insidious onset and slow progressive course. The patient presents with paresthesia and neuropathic pain distally in the lower limbs, with no ataxia, tremor, foot deformities, cranial nerve or autonomic involvement. Past medical history is positive for type 2 diabetes, cardiac insufficiency and lung cancer, for which he underwent chemotherapy 15 years ago. Nerve conduction studies show a sensory-motor, symmetric, axonal polyneuropathy. Screening lab tests including vitamin B12, folate, vitamin B6, vitamin E, HBV, HCV, ANA, ANCA, ENA, CRP, ESR, copper, cryoglobulins and serum electrophoresis are all negative, with evidence of hyperglycaemia and elevated HbA1c. There is no family history of polyneuropathy.		● **Reasoning: To further characterize the extent and pattern of the neuropathy, a detailed neurological examination can provide more insights into sensory deficits, motor strength, reflexes, and gait abnormalities. This helps in confirming the symmetry and length-dependence of the neuropathy.**<br>**2. Nerve Conduction Studies (Repeat/Extended) and Electromyography (EMG)**<br>● **Reasoning: Although initial nerve conduction studies have confirmed axonal polyneuropathy, repeat or more detailed studies can help quantify the severity, progression, and exact distribution of nerve damage. EMG can help identify any additional motor unit changes that might have been missed initially.**<br>**3. Skin Biopsy with Intraepidermal Nerve Fiber Density (IENFD) Measurement**<br>● **Reasoning: This test can provide a direct assessment of small fiber neuropathy, which may not be fully captured by nerve conduction studies. It helps in evaluating the integrity of small sensory fibers, which are often affected in diabetic neuropathy.**<br>**4. Quantitative Sensory Testing (QST)**<br>● **Reasoning: QST can measure the sensory threshold for vibration, temperature, and pain, offering additional information about the functional status of sensory nerves, particularly small fibers.**<br>**5. Serum Immunofixation Electrophoresis and Free Light Chains**<br>● **Reasoning: This test can detect monoclonal proteins that may not be apparent on standard serum electrophoresis, helping to rule out paraproteinemias and monoclonal gammopathies, which can be associated with neuropathy.**<br>**6. Oral Glucose Tolerance Test (OGTT)**<br>● **Reasoning: Despite known diabetes, OGTT can provide a more detailed assessment of glucose metabolism and help in identifying any postprandial spikes that may contribute to neuropathy.**<br>**7. Anti-Ganglioside Antibodies**<br>● **Reasoning: These antibodies can be associated with certain types of immune-mediated neuropathies. Testing for anti-GM1 and other anti-ganglioside antibodies can help rule out chronic inflammatory demyelinating polyneuropathy (CIDP) and other autoimmune neuropathies.**<br>**8. Lumbar Puncture for Cerebrospinal Fluid (CSF) Analysis**<br>● **Reasoning: CSF analysis can help identify elevated protein levels, which are often seen in CIDP and other inflammatory neuropathies. It can also help rule out infectious or neoplastic causes.**<br>**9. Genetic Testing for Hereditary Neuropathies**<br>● **Reasoning: Although there is no family history, late-onset hereditary neuropathies can occur. Genetic testing can identify mutations in genes associated with Charcot-Marie-Tooth disease and other hereditary neuropathies.**<br>**10. Autonomic Function Tests**<br>● **Reasoning: Given the absence of autonomic symptoms but to be thorough, autonomic function testing can confirm the absence of autonomic involvement, which might suggest certain types of neuropathy over others.**<br>**Most Crucial Test**<br>**Nerve Conduction Studies (Repeat/Extended) and Electromyography (EMG)**<br>● **Rationale: While many tests are useful, repeat and extended nerve conduction studies combined with EMG are crucial for the following reasons:**<br>○ **Detailed Analysis: They provide a comprehensive understanding of both the sensory and motor nerve involvement, allowing for a precise characterization of the neuropathy.**<br>○ **Progression Assessment: They help in determining the progression and severity of the neuropathy over time.**<br>○ **Differential Diagnosis: They can help distinguish between purely axonal damage and demyelinating processes, which can significantly narrow down the differential diagnoses.**<br>○ **Baseline for Comparison: They serve as a baseline for future comparisons to assess disease progression or response to treatment.**
44	An 81-year-old male patient presents with a six-month history of sensory-motor, symmetrical, length-dependent polyneuropathy with subacute onset and slow progression. The patient reports neuropathic pain in the lower limbs in a distal, symmetric distribution, along with sensory ataxia. Clinical examination does not reveal weakness, tremor, foot deformities, cranial nerve or autonomic involvement. Past medical history is positive for IgM kappa monoclonal gammopathy, Waldenström macroglobulinemia, and colorectal cancer, for which he underwent chemotherapy five years ago. Nerve conduction studies show a length-dependent, sensorimotor, symmetric, demyelinating polyneuropathy. Screening lab tests, including vitamin B12, folate, vitamin B6, vitamin E, HCV, HBV, ANA, ANCA, ENA, CRP, ESR, glycemia, HbA1c, copper, and cryoglobulins are all normal, with evidence of IgM kappa paraprotein. There is no family history of polyneuropathy.	Anti-MAG polyneuropathy	**Step-by-Step Analysis and Additional Examinations**<br>**1. Detailed Patient History and Examination**<br>● **Reasoning: A thorough history and physical examination are essential to identify any subtle signs that may have been missed initially and to gather more context about the patient's symptoms and their progression.**<br>● **Specifics: Revisit history of chemotherapy drugs used, any other comorbidities not initially reported, medication history, dietary habits, and detailed neurological examination.**<br>**2. Repeat and Additional Blood Tests**<br>● **Serum Immunofixation Electrophoresis (IFE) and Free Light Chain Assay**<br>○ **Reasoning: To confirm the presence and type of monoclonal gammopathy, quantify the paraprotein, and assess the free light chain ratio.**<br>○ **Specifics: IgM kappa monoclonal gammopathy needs confirmation and quantification to assess the extent of its role in neuropathy.**<br>● **Anti-myelin-associated glycoprotein (MAG) antibodies**<br>○ **Reasoning: Anti-MAG antibodies are often associated with demyelinating neuropathies in patients with IgM monoclonal gammopathy.**<br>○ **Specifics: The presence of anti-MAG antibodies would support the diagnosis of a neuropathy associated with Waldenström macroglobulinemia.**<br>● **Electrolytes, Renal, and Liver Function Tests**<br>○ **Reasoning: To rule out other metabolic causes of neuropathy such as renal insufficiency or liver dysfunction.**<br>○ **Specifics: Abnormalities in these tests could suggest alternative or contributing causes of neuropathy.**<br>● **Thyroid Function Tests (TFTs)**<br>○ **Reasoning: Hypothyroidism can cause peripheral neuropathy.**<br>○ **Specifics: Evaluating TSH, free T4, and free T3 levels.**<br>**3. Lumbar Puncture (LP)**<br>● **Cerebrospinal Fluid (CSF) Analysis**<br>○ **Reasoning: To check for elevated protein levels, which can indicate chronic inflammatory demyelinating polyneuropathy (CIDP) or other inflammatory causes of neuropathy.**<br>○ **Specifics: CSF protein level, cell count, and electrophoresis.**<br>**4. MRI of Spine and Brain**<br>● **Reasoning: To exclude structural causes of neuropathy such as spinal stenosis, and to look for evidence of CNS involvement that could suggest CIDP or other inflammatory disorders.**<br>● **Specifics: MRI with contrast can show nerve root enhancement in CIDP.**<br>**5. Skin Biopsy**<br>● **Reasoning: To assess intraepidermal nerve fiber density, which can provide evidence of small fiber neuropathy that may not be detected on nerve conduction studies.**<br>● **Specifics: Punch biopsy for histopathological analysis.**<br>**6. Repeat Nerve Conduction Studies and Electromyography (EMG)**<br>● **Reasoning: To confirm the findings of the initial study and to monitor any progression or changes in the pattern of neuropathy.**<br>● **Specifics: Detailed assessment of motor and sensory nerve function.**<br>**7. Bone Marrow Biopsy**<br>● **Reasoning: To further characterize the extent of the monoclonal gammopathy and rule out other hematologic malignancies.**

			● Specifics: Histopathological and cytogenetic analysis.<br>**Most Crucial Test**<br>**Serum Immunofixation Electrophoresis (IFE) and Free Light Chain Assay**<br>**This test is the most crucial because it will confirm the presence and type of monoclonal gammopathy and quantify the paraprotein levels. Given the patient's history of Waldenström macroglobulinemia and IgM kappa monoclonal gammopathy, this test is critical in correlating the paraprotein levels with the neuropathy. It directly targets the suspected underlying cause and helps in determining the extent of the disease's contribution to the neuropathy.**<br>**This step-by-step approach ensures a comprehensive evaluation, considering both common and rare causes, and using targeted investigations to narrow down the differential diagnoses effectively.**
45	An 83-year-old male patient presents with a 3-year history of sensory, symmetrical, length dependent polyneuropathy with insidious onset. The patient presents with paresthesia and hypoesthesia in the lower limbs without weakness or sensory ataxia. Clinical examination does not show the presence of tremor, neuropathic pain, foot deformities, cranial nerve or autonomic involvement. Past medical history is positive for IgG lambda monoclonal gammopathy. Nerve conduction studies show axonal, length dependent, sensory-motor polyneuropathy and unilateral carpal tunnel syndrome. Screening lab tests including vitamin B12, folate, vitamin B6, vitamin E, HCV, HBV, ANA, ANCA, ENA, CRP, ESR, glycemia, HbA1c, copper, and cryoglobulins are all negative, with positivity for IgG lambda paraprotein. There is no family history of polyneuropathy.	MGUS-associated neuropathy	**Step-by-Step Analysis and Additional Examinations**<br>**Initial Summary**<br>**The patient is an 83-year-old male with a 3-year history of sensory, symmetrical, length-dependent polyneuropathy, characterized by paresthesia and hypoesthesia in the lower limbs. He has a known IgG lambda monoclonal gammopathy. Extensive initial laboratory testing has been negative for common causes of polyneuropathy.**<br>**Step 1: Detailed Analysis of the Case**<br>**Given the patient's clinical presentation and history, further testing is necessary to narrow down the differential diagnoses, particularly focusing on ruling out specific conditions that can mimic or coexist with MGUS-related neuropathy.**<br>**Step 2: Additional Examinations**<br>**1. Serum Protein Electrophoresis (SPEP) and Immunofixation Electrophoresis (IFE):**<br>○ **Reasoning: To further characterize the monoclonal gammopathy. While MGUS is a common cause, multiple myeloma or other plasma cell dyscrasias must be ruled out, especially in the presence of an axonal neuropathy.**<br>**2. Bone Marrow Biopsy:**<br>○ **Reasoning: If SPEP and IFE suggest a clonal plasma cell disorder beyond MGUS, a bone marrow biopsy would be necessary to evaluate for plasma cell percentage, which can help diagnose multiple myeloma or other plasma cell disorders.**<br>**3. Anti-MAG Antibodies:**<br>○ **Reasoning: Anti-myelin-associated glycoprotein (MAG) antibodies are often associated with monoclonal gammopathy-related neuropathies, particularly in IgM paraproteinemias, but can also be relevant in IgG cases.**<br>**4. Autonomic Function Tests:**<br>○ **Reasoning: To confirm the absence of autonomic involvement. This can help distinguish between different types of polyneuropathies, as some, such as diabetic neuropathy, may have autonomic features even if not initially evident.**<br>**5. Skin Biopsy for Intraepidermal Nerve Fiber Density (IENFD):**<br>○ **Reasoning: To assess small fiber involvement, which might not be detected in routine nerve conduction studies. This test is useful if there is a clinical suspicion of small fiber neuropathy despite normal large fiber studies.**<br>**6. Quantitative Sensory Testing (QST):**<br>○ **Reasoning: To assess sensory function more precisely and quantify the degree of sensory loss or dysfunction, which can assist in characterizing the neuropathy more accurately.**<br>**7. Genetic Testing for Hereditary Neuropathies:**<br>○ **Reasoning: Although the family history is negative, genetic mutations can sometimes occur de novo. Testing for mutations commonly associated with hereditary sensory and motor neuropathies (e.g., Charcot-Marie-Tooth disease) might be warranted if the clinical picture is unclear after other tests.**<br>**8. Repeat Glycemic Monitoring:**<br>○ **Reasoning: To rule out intermittent hyperglycemia or pre-diabetes that may not have been detected in initial tests. Continuous glucose monitoring or an oral glucose tolerance test could provide additional information.**<br>**Step 3: Most Crucial Test**<br>**Serum Protein Electrophoresis (SPEP) and Immunofixation Electrophoresis (IFE)**<br>**Reasoning:**<br>● **These tests are crucial to definitively characterize the monoclonal protein and to distinguish between benign monoclonal gammopathy (MGUS) and more malignant conditions such as multiple myeloma or other plasma cell dyscrasias.**<br>● **Accurate identification of the type and amount of monoclonal protein can guide further management and help in narrowing down the exact cause of the polyneuropathy.**<br>● **Given the patient's history of IgG lambda monoclonal gammopathy, it is essential to ensure that this is not progressing to a more serious condition, which can significantly affect the clinical approach and prognosis.**<br>**By focusing on SPEP and IFE, we can gain a deeper understanding of the monoclonal gammopathy, which is likely the underlying cause of the patient's polyneuropathy, and determine if additional, more invasive tests like a bone marrow biopsy are needed.**
46	A 71-year-old female patient presents with a 2-year history of sensory-motor, asymmetrical multineuropathy with relatively slow onset. The patient presents with weakness, numbness and tingling in a multifocal distribution in the four limbs, with hand tremor and mild sensory ataxia without pain. Clinical examination reveals absence of foot deformities, cranial nerve or autonomic involvement. Past medical history is positive for breast and kidney cancer, for which the patient underwent chemotherapy five years ago, and which were considered cured. Nerve conduction studies show a multifocal, demyelinating, sensory-motor neuropathy with motor nerve conduction blocks. Screening lab tests including vitamin B12, folate, vitamin B6, vitamin E, HCV, HBV, ANA, ANCA, ENA, CRP, ESR, glycemia, HbA1c, copper,	Multifocal CIDP	**Step-by-Step Analysis of Additional Examinations**<br>**To further narrow down the differential diagnoses for the patient with sensory-motor, asymmetrical multineuropathy, the following additional examinations should be conducted:**<br>**1. Cerebrospinal Fluid (CSF) Analysis**<br>○ **Reasoning:**<br>■ **CSF analysis can help in diagnosing CIDP. An elevated protein level without significant pleocytosis (an increase in white blood cells) is characteristic of CIDP.**<br>■ **CSF analysis can also rule out infectious causes and other inflammatory conditions that might affect the nervous system.**<br>**2. Anti-GM1 Antibody Testing**<br>○ **Reasoning:**<br>■ **This test is useful for diagnosing Multifocal Motor Neuropathy (MMN), as anti-GM1 antibodies are often elevated in MMN patients.**<br>■ **The presence of these antibodies would strongly support a diagnosis of MMN, particularly in the context of motor nerve conduction blocks.**<br>**3. Repeat Nerve Conduction Studies and Electromyography (EMG)**<br>○ **Reasoning:**<br>■ **Additional nerve conduction studies can provide more detailed information on the pattern of demyelination and conduction blocks.**<br>■ **EMG can help differentiate between axonal and demyelinating processes and can identify the presence of active denervation.**<br>**4. Paraneoplastic Panel**<br>○ **Reasoning:**<br>■ **Given the patient's history of cancer, testing for a broad panel of paraneoplastic antibodies (e.g., anti-Hu, anti-Ri, anti-Yo) can help identify a paraneoplastic syndrome.**<br>■ **Although initial screening was negative, specific paraneoplastic antibodies may provide more insight into potential immune-mediated neuropathies associated with previous malignancies.**<br>**5. MRI of the Brachial and Lumbosacral Plexus**<br>○ **Reasoning:**

	cryoglobulins and serum electrophoresis are all negative. There is no family history of polyneuropathy.		■ **MRI can detect structural abnormalities or inflammatory changes in the peripheral nerves and plexuses.**<br>■ **It can help identify any compressive lesions, inflammatory infiltrates, or other structural causes of neuropathy.**<br>6. **Serum Immunofixation Electrophoresis and Free Light Chains**<br>○ **Reasoning:**<br>■ **These tests can detect monoclonal gammopathies, which may be associated with demyelinating neuropathies such as POEMS syndrome or amyloidosis.**<br>■ **Monoclonal gammopathies can sometimes present with neuropathic symptoms even in the absence of systemic disease.**<br>7. **Skin Biopsy**<br>○ **Reasoning:**<br>■ **A skin biopsy with analysis of intraepidermal nerve fiber density can help in diagnosing small fiber neuropathies, which might present with sensory symptoms.**<br>■ **Although less likely in this case given the demyelinating nature of the neuropathy, it can rule out small fiber involvement.**<br>**Most Crucial Test**<br>**Cerebrospinal Fluid (CSF) Analysis**<br>● **Justification:**<br>○ **CSF analysis is the most crucial test because it provides direct evidence of the inflammatory process characteristic of CIDP (elevated protein level without significant pleocytosis).**<br>○ **It can also rule out infectious causes and other inflammatory or neoplastic conditions affecting the nervous system.**<br>○ **Given the clinical suspicion of CIDP, confirming the diagnosis with CSF findings would be highly valuable for guiding appropriate treatment and management.**<br>**By performing these additional tests, clinicians can narrow down the differential diagnoses and determine the most accurate diagnosis for the patient's polyneuropathy.**
47	An 80-year-old male patient presents with a 4-year history of pure sensory, symmetrical, length dependent polyneuropathy with insidious onset. The patient presents with distal paresthesia, neuropathic pain and cramps in the lower limbs with no ataxia, tremor, foot deformities, cranial nerve or autonomic involvement. Past medical history shows diabetes, psoriatic arthritis, polymyalgia rheumatica and chemotherapy for lung cancer. Neuropathy symptoms started four years after chemotherapy. Nerve conduction studies show mixed sensory-motor, symmetric, axonal polyneuropathy. Screening lab tests including vitamin B12, folate, vitamin B6, vitamin E, HCV, ANA, ANCA, ENA, CRP, ESR, glycemia, copper, cryoglobulins and serum electrophoresis are all negative, with evidence of chronic HBV infection. HbA1c levels are elevated. There is no family history of polyneuropathy.	Diabetic neuropathy	**Step-by-Step Analysis and Additional Examinations**<br>**Given the comprehensive data from the previous clinical scenario, it is important to further investigate to narrow down the differential diagnoses of the patient's polyneuropathy. Here are the necessary additional examinations that should be conducted:**<br>**1. HbA1c and Fasting Blood Glucose Levels**<br>● **Reasoning: While the patient already has elevated HbA1c, confirming current glycemic control and ruling out any acute hyperglycemic states is crucial. Diabetic neuropathy is a leading cause, and these tests will confirm the chronicity and control of diabetes.**<br>● **Test: Repeat HbA1c and fasting blood glucose levels.**<br>**2. Oral Glucose Tolerance Test (OGTT)**<br>● **Reasoning: In some cases, diabetic neuropathy can occur even with normal fasting glucose levels. An OGTT can help in diagnosing diabetes or impaired glucose tolerance that might not be evident with fasting glucose alone.**<br>● **Test: OGTT.**<br>**3. Anti-Ganglioside Antibodies**<br>● **Reasoning: These are associated with various neuropathies, including chronic inflammatory demyelinating polyneuropathy (CIDP) and multifocal motor neuropathy. Though less likely, ruling out immune-mediated causes is important.**<br>● **Test: Serum anti-ganglioside antibodies (GM1, GD1a, GD1b, GQ1b).**<br>**4. Nerve Biopsy**<br>● **Reasoning: In cases of unclear etiology, a nerve biopsy can provide definitive histopathological evidence of the type and cause of neuropathy, particularly useful for distinguishing between axonal and demyelinating processes and identifying vasculitis or amyloidosis.**<br>● **Test: Sural nerve biopsy.**<br>**5. Lumbar Puncture and CSF Analysis**<br>● **Reasoning: This can help identify inflammatory or infectious causes of neuropathy, including elevated protein levels seen in CIDP or chronic infections.**<br>● **Test: Cerebrospinal fluid (CSF) analysis.**<br>**6. Skin Biopsy for Intraepidermal Nerve Fiber Density (IENFD)**<br>● **Reasoning: This test can assess small fiber neuropathy, which might not be detected on standard nerve conduction studies. It can be particularly useful in diabetic neuropathy to assess the degree of small fiber involvement.**<br>● **Test: Skin biopsy for IENFD.**<br>**7. Hepatitis B Virus (HBV) DNA Quantification**<br>● **Reasoning: Quantifying the viral load can provide insights into the activity of the chronic HBV infection, which could contribute to neuropathy.**<br>● **Test: HBV DNA quantification.**<br>**8. Thyroid Function Tests (TFT)**<br>● **Reasoning: Hypothyroidism can cause polyneuropathy, and thyroid dysfunction should be ruled out.**<br>● **Test: Serum TSH, Free T4.**<br>**9. Paraneoplastic Antibody Panel**<br>● **Reasoning: Given the patient's history of lung cancer, a paraneoplastic syndrome could be a cause of neuropathy. Identifying specific antibodies can support this diagnosis.**<br>● **Test: Paraneoplastic antibody panel (anti-Hu, anti-Ri, anti-Yo, etc.).**<br>**10. Heavy Metal Screening**<br>● **Reasoning: Chronic exposure to heavy metals such as lead, arsenic, or mercury can cause peripheral neuropathy. Screening for these can rule out toxic causes.**<br>● **Test: Blood and urine heavy metal levels.**<br>**Most Crucial Test**<br>**Out of all the proposed tests, the HbA1c and fasting blood glucose levels are the most crucial. These tests provide immediate and essential information about the patient's glycemic control, which is the most likely cause of his symptoms given the history of diabetes and elevated HbA1c levels. Ensuring accurate and up-to-date glycemic data will help to either confirm diabetic neuropathy or prompt further investigation if the results are not consistent with poor glycemic control.**<br>**Conclusion**<br>**By conducting these additional examinations, the differential diagnosis can be further refined. Given the patient's history and clinical presentation, prioritizing tests related to diabetes management and glycemic control is crucial. Hence, repeating the HbA1c and fasting blood glucose levels stands out as the most critical step in narrowing down the differential diagnosis of this patient's polyneuropathy.**
48	A 45-year-old female patient presents with a 1-year history of pure sensory, symmetrical, length dependent	Chemotherapy-induced neuropathy	**Step-by-Step Analysis and Additional Examinations**<br>**Step 1: Review of Current Information**<br>● **The patient has a 1-year history of pure sensory, symmetrical, length-dependent polyneuropathy with subacute onset, following chemotherapy for multiple myeloma.**

	polyneuropathy with subacute onset. The patient presents with distal leg paresthesia and neuropathic pain, with no ataxia, tremor, foot deformities, cranial nerve or autonomic involvement. Clinical examination shows hyperalgesia at pinprick test with normal tactile sensation. Past medical history is positive for multiple myeloma, for which the patient has undergone chemotherapy. Neuropathy symptoms started after the first chemotherapy cycle and remained stable ever since. Nerve conduction studies show pure a sensory, symmetric, axonal polyneuropathy. Screening lab tests including vitamin B12, folate, vitamin B6, vitamin E, HBV, HCV, ANA, ANCA, ENA, CRP, ESR, glycemia, HbA1c, copper and cryoglobulins are all negative, with positivity for IgG lambda paraprotein. There is no family history of polyneuropathy.		**● Clinical features include distal leg paresthesia, neuropathic pain, and hyperalgesia.**<br>**● Past medical history includes multiple myeloma treated with chemotherapy.**<br>**● Lab tests showed positivity for IgG lambda paraprotein.**<br>**● Negative results for vitamin deficiencies, infections, autoimmune markers, glycemic control, copper, and cryoglobulins.**<br>**● Nerve conduction studies show pure sensory, symmetric, axonal polyneuropathy.**<br>**Step 2: Additional Examinations**<br>**1. Electromyography (EMG)**<br>**○ Reasoning: To further characterize the type and extent of nerve damage. EMG can help differentiate between axonal and demyelinating neuropathies and identify any muscle involvement that might not be apparent clinically.**<br>**2. Quantitative Sensory Testing (QST)**<br>**○ Reasoning: To assess the functional status of sensory nerves more precisely. QST can help to quantify sensory deficits and differentiate between different types of sensory fiber involvement (small vs. large fibers).**<br>**3. Skin Biopsy with Intraepidermal Nerve Fiber Density (IENFD) Analysis**<br>**○ Reasoning: To evaluate small fiber neuropathy, which might not be detected by nerve conduction studies. This test can help confirm the diagnosis of small fiber involvement in sensory neuropathy.**<br>**4. Serum Immunofixation Electrophoresis**<br>**○ Reasoning: To better characterize the IgG lambda paraprotein and evaluate the possibility of an underlying monoclonal gammopathy contributing to the neuropathy. It can help differentiate between different types of paraproteinemias and assess the disease burden of multiple myeloma or MGUS.**<br>**5. Cerebrospinal Fluid (CSF) Analysis**<br>**○ Reasoning: To rule out chronic inflammatory demyelinating polyradiculoneuropathy (CIDP) or other inflammatory neuropathies. Elevated protein levels in the CSF can indicate an ongoing inflammatory process affecting the peripheral nerves.**<br>**6. MRI of the Lumbar Spine**<br>**○ Reasoning: To rule out any compressive lesions or other structural abnormalities that might contribute to the patient's symptoms. MRI can provide detailed imaging of the nerve roots and spinal cord.**<br>**7. Anti-MAG (Myelin-Associated Glycoprotein) Antibodies**<br>**○ Reasoning: To rule out an immune-mediated neuropathy. Anti-MAG neuropathy is associated with IgM paraproteins but can also be seen in cases with IgG paraproteins.**<br>**8. Repeat Nerve Conduction Studies**<br>**○ Reasoning: To assess any progression or stability in the neuropathy over time. Repeating these studies can provide insight into the chronicity and response to any treatments.**<br>**Step 3: Determining the Most Crucial Test**<br>**Among these additional examinations, Skin Biopsy with Intraepidermal Nerve Fiber Density (IENFD) Analysis is the most crucial.**<br>**Reasoning:**<br>**● Diagnostic Clarity: The patient's presentation includes pure sensory symptoms, which may involve small fiber neuropathy. Skin biopsy can directly assess small fiber density and provide a definitive diagnosis.**<br>**● Nerve Conduction Limitation: Traditional nerve conduction studies and EMG may not effectively detect small fiber involvement. Skin biopsy complements these studies by offering additional diagnostic information.**<br>**● Treatment Implications: Accurate diagnosis of small fiber neuropathy can guide specific therapeutic strategies, especially if the neuropathy is secondary to chemotherapy or an underlying plasma cell dyscrasia.**<br>**Conclusion**<br>**The most crucial test to conduct is the Skin Biopsy with Intraepidermal Nerve Fiber Density (IENFD) Analysis, as it provides definitive evidence for small fiber neuropathy, aligning with the patient's symptoms and enhancing the diagnostic accuracy beyond what nerve conduction studies alone can offer. This test will significantly contribute to narrowing down the differential diagnoses and guiding appropriate management.**
49	A 79-year-old female patient presents with a 1-year history of pure sensory, symmetrical, length dependent polyneuropathy with insidious onset and progressive course. The patient presents with distal leg paresthesia, severe ataxia and postural tremor, which she reported to appear along with the other symptoms. Clinical examination does not show presence of foot deformities, cranial nerve or autonomic involvement. Past medical history is positive for diabetes. Nerve conduction studies show demyelinating, sensory-motor, symmetric polyneuropathy. Screening lab tests show the presence of IgM kappa monoclonal gammopathy, while other exams including vitamin B12, folate, vitamin B6, vitamin E, HCV, HBV, ANA, ANCA, ENA, CRP, ESR, glycemia, HbA1c, copper and cryoglobulins are all negative. There is no family history of polyneuropathy.	Anti-MAG polyneuropathy	**Step-by-Step Analysis and Additional Examinations**<br>**Step 1: Review of Current Data**<br>**● Patient Details: 79-year-old female with a 1-year history of pure sensory, symmetrical, length-dependent polyneuropathy**<br>**● Symptoms: Distal leg paresthesia, severe ataxia, postural tremor**<br>**● Clinical Findings: No foot deformities, cranial nerve, or autonomic involvement**<br>**● Medical History: Diabetes**<br>**● Nerve Conduction Studies: Demyelinating, sensory-motor, symmetric polyneuropathy**<br>**● Lab Results: Positive IgM kappa monoclonal gammopathy; negative for vitamin deficiencies, infections (HCV, HBV), autoimmune markers (ANA, ANCA, ENA), and other potential causes**<br>**● Family History: No polyneuropathy**<br>**Step 2: Identify Gaps and Need for Additional Examinations**<br>**Given the patient's presentation and initial test results, further examinations are required to narrow down the differential diagnoses and to confirm the suspected CIDP or MGUS-related neuropathy.**<br>**Additional Examinations**<br>**1. Serum Protein Electrophoresis (SPEP) and Immunofixation Electrophoresis (IFE)**<br>**○ Reasoning:**<br>**■ To further characterize the IgM kappa monoclonal gammopathy detected in initial screenings. This will help differentiate between benign MGUS and potentially malignant conditions like Waldenström's macroglobulinemia or multiple myeloma, which can also present with neuropathy.**<br>**2. Cerebrospinal Fluid (CSF) Analysis**<br>**○ Reasoning:**<br>**■ A lumbar puncture with CSF analysis can help identify elevated protein levels without pleocytosis, which is supportive of CIDP. It also helps rule out infections or malignancies that could cause similar symptoms.**<br>**3. Anti-MAG Antibodies**<br>**○ Reasoning:**<br>**■ Anti-myelin-associated glycoprotein (MAG) antibodies are often present in patients with demyelinating neuropathy associated with IgM monoclonal gammopathy. This test can help confirm MGUS-related neuropathy.**<br>**4. Nerve Biopsy**<br>**○ Reasoning:**<br>**■ A nerve biopsy can provide definitive histopathological evidence of demyelination and inflammation typical of CIDP. It can also help exclude other causes of neuropathy, such as vasculitis or amyloidosis.**<br>**5. MRI of the Spine and Brachial/Lumbosacral Plexus**<br>**○ Reasoning:**

			■ **MRI can identify hypertrophy or inflammation of the nerve roots and plexus, which is indicative of CIDP. It helps exclude compressive neuropathies or other structural abnormalities.**<br>6. **Bone Marrow Biopsy**<br>○ **Reasoning:**<br>■ **To evaluate the extent of monoclonal gammopathy and rule out underlying hematologic malignancies, such as multiple myeloma or Waldenström's macroglobulinemia, which can present with neuropathy.**<br>7. **Retinal Examination**<br>○ **Reasoning:**<br>■ **Paraneoplastic retinopathy can present with neuropathy and is associated with certain monoclonal gammopathies. This test helps to identify any related retinal pathology.**<br>8. **Autoimmune Panel Expansion**<br>○ **Reasoning:**<br>■ **Although some autoimmune markers were tested, a broader panel including anti-GM1, anti-GQ1b, and other ganglioside antibodies can be helpful. These are associated with various neuropathies and might provide additional diagnostic information.**<br>**Step 3: Most Crucial Test**<br>**Cerebrospinal Fluid (CSF) Analysis**<br>● **Justification:**<br>○ **CSF analysis provides direct evidence of an inflammatory process with elevated protein levels, which is a hallmark of CIDP.**<br>○ **It helps exclude other potential causes such as infections or malignancies.**<br>○ **It is less invasive than a nerve biopsy and provides critical diagnostic information that can strongly support or refute the diagnosis of CIDP.**<br>○ **The presence of elevated protein without pleocytosis is a key feature in diagnosing CIDP and distinguishing it from other conditions.**<br>**In conclusion, while all the proposed additional tests are valuable, CSF analysis stands out as the most crucial test for confirming the diagnosis of CIDP in this patient.**
50	A 57-year-old female patient presents with a five-year history of chronic, sensory-motor, symmetrical, length-dependent polyneuropathy with insidious onset and slow progression. The patient reports neuropathic pain in the feet, mild distal weakness in the lower limbs, and pes cavus, with no cranial nerve or autonomic involvement. Past medical history is unremarkable. Nerve conduction studies show a mixed axonal-demyelinating, sensory-motor polyneuropathy. Screening lab tests including vitamin B12, folate, vitamin B6, vitamin E, HBV, HCV, glycemia, HbA1c, ANA, ANCA, ENA, CRP, ESR, copper, cryoglobulins, and serum electrophoresis are all negative. Her mother has similar symptoms, while her brother has severe walking difficulties.	X-linked CMT	**Additional Examinations for Narrowing Down Differential Diagnoses**<br>**Step-by-Step Analysis**<br>1. **Genetic Testing**<br>○ **Reasoning: Given the strong family history of neuropathy, genetic testing can identify mutations associated with hereditary neuropathies such as Charcot-Marie-Tooth Disease (CMT) or Hereditary Sensory and Autonomic Neuropathy (HSAN). Specific gene panels can target common mutations (e.g., PMP22 duplication/deletion for CMT1A, MFN2 for CMT2A).**<br>○ **Tests:**<br>■ **CMT gene panel**<br>■ **Whole exome sequencing if initial gene panel is inconclusive**<br>2. **Nerve Biopsy**<br>○ **Reasoning: A nerve biopsy can provide histopathological confirmation of the type of neuropathy, showing features such as axonal degeneration, demyelination, and inflammatory infiltrates. This can help differentiate between various hereditary neuropathies and other conditions.**<br>○ **Tests:**<br>■ **Sural nerve biopsy**<br>3. **Skin Biopsy**<br>○ **Reasoning: This test can assess small fiber involvement and density, helping to identify conditions like small fiber neuropathy, which can sometimes accompany hereditary neuropathies.**<br>○ **Tests:**<br>■ **Skin biopsy with intraepidermal nerve fiber density (IENFD) analysis**<br>4. **Lumbar Puncture with Cerebrospinal Fluid (CSF) Analysis**<br>○ **Reasoning: Analyzing CSF can rule out inflammatory causes of neuropathy, such as chronic inflammatory demyelinating polyneuropathy (CIDP), which can present similarly but would show elevated protein levels and other abnormalities.**<br>○ **Tests:**<br>■ **CSF protein, glucose, cell count**<br>■ **Oligoclonal bands**<br>5. **Electromyography (EMG) and Repeated Nerve Conduction Studies (NCS)**<br>○ **Reasoning: EMG and repeated NCS can provide more detailed information about the pattern and progression of nerve involvement, distinguishing between axonal and demyelinating components.**<br>○ **Tests:**<br>■ **Detailed EMG**<br>■ **Repeat NCS with focus on multiple nerve sites**<br>6. **Immunofixation Electrophoresis of Serum and Urine**<br>○ **Reasoning: To rule out paraproteinemias and monoclonal gammopathies, which can sometimes present with neuropathy, even if initial serum electrophoresis was negative.**<br>○ **Tests:**<br>■ **Serum and urine immunofixation electrophoresis**<br>7. **Autonomic Function Tests**<br>○ **Reasoning: Even though there is no reported autonomic involvement, detailed autonomic testing can help identify subtle autonomic dysfunction that might support a diagnosis of HSAN or other neuropathies with mild autonomic features.**<br>○ **Tests:**<br>■ **Tilt table test**<br>■ **Quantitative sudomotor axon reflex test (QSART)**<br>■ **Heart rate variability analysis**<br>**Conclusion: Most Crucial Test**<br>**Genetic Testing is the most crucial test. Given the strong family history and the clinical features suggestive of a hereditary neuropathy (e.g., pes cavus, symmetrical sensory-motor polyneuropathy, and family members with similar symptoms), genetic testing can directly identify specific mutations responsible for the neuropathy, confirming the diagnosis of conditions like Charcot-Marie-Tooth Disease (CMT) or Hereditary Sensory and Autonomic Neuropathy (HSAN). This test can provide a definitive diagnosis and guide management and genetic counseling for the patient and her family.**

51	A 62-year-old male patient presents with an 8-month history of pure sensory, symmetrical, length dependent polyneuropathy with subacute onset. The patient presents with numbness and tingling and neuropathic pain distally in the lower limbs while he has no tremor, foot deformities, cranial nerve or autonomic involvement. Clinical examination reveals the presence of sensory ataxia. Past medical history is silent. Nerve conduction studies show a demyelinating, sensory-motor polyneuropathy and unilateral carpal tunnel syndrome. Screening lab tests including vitamin B12, folate, vitamin B6, vitamin E, HCV, HBV, ANA, ANCA, ENA, CRP, ESR, glycemia, HbA1c, copper, and cryoglobulins are all negative. Serum electrophoresis shows the presence of IgG paraprotein. There is no family history of polyneuropathy.	CIDP	**Step-by-Step Analysis of Additional Examinations**<br>**1. Bone Marrow Biopsy and Aspiration**<br>**Reasoning:**<br>● **Purpose: To evaluate for plasma cell disorders such as multiple myeloma, Waldenström macroglobulinemia, or other hematologic malignancies that may present with monoclonal gammopathy.**<br>● **Case Specifics: The presence of IgG paraprotein suggests a possible plasma cell dyscrasia. A bone marrow biopsy can confirm the presence of abnormal plasma cells or other hematologic abnormalities.**<br>**2. Serum Free Light Chains (FLC) Assay**<br>**Reasoning:**<br>● **Purpose: To detect light chain disease and provide additional information about the monoclonal protein burden.**<br>● **Case Specifics: This test can identify free light chains that might not be detected on standard serum electrophoresis, helping to assess the risk of progression to multiple myeloma or related disorders.**<br>**3. Immunofixation Electrophoresis (IFE)**<br>**Reasoning:**<br>● **Purpose: To further characterize the type of monoclonal protein (IgG subtype) and assess clonality.**<br>● **Case Specifics: This test can help to confirm the presence and type of paraprotein detected on serum electrophoresis, providing more detail about the monoclonal gammopathy.**<br>**4. Lumbar Puncture with Cerebrospinal Fluid (CSF) Analysis**<br>**Reasoning:**<br>● **Purpose: To assess for CSF protein levels, oligoclonal bands, and other signs of inflammation or infection that might be indicative of CIDP or another central nervous system process.**<br>● **Case Specifics: In patients with suspected CIDP, elevated CSF protein without pleocytosis (increased white blood cells) is often seen. CSF analysis can help differentiate between CIDP and other polyneuropathies.**<br>**5. Skin Biopsy**<br>**Reasoning:**<br>● **Purpose: To assess for small fiber neuropathy by examining intraepidermal nerve fiber density.**<br>● **Case Specifics: While the patient primarily presents with large fiber involvement, a skin biopsy can help rule out or confirm small fiber neuropathy, which may coexist with large fiber neuropathies.**<br>**6. Imaging Studies (MRI or CT Scan)**<br>**Reasoning:**<br>● **Purpose: To evaluate for possible organomegaly, masses, or other structural abnormalities associated with POEMS syndrome.**<br>● **Case Specifics: Although POEMS syndrome is less likely, imaging can help rule out associated organomegaly or tumors that might suggest this diagnosis.**<br>**7. Autonomic Testing**<br>**Reasoning:**<br>● **Purpose: To assess for autonomic dysfunction, which can sometimes accompany polyneuropathy.**<br>● **Case Specifics: Even though the patient has no overt autonomic symptoms, autonomic testing can provide a comprehensive assessment of the neuropathy's extent.**<br>**Conclusion: Most Crucial Test**<br>**Most Crucial Test: Bone Marrow Biopsy and Aspiration**<br>**Reasoning:**<br>● **This test directly addresses the presence of IgG paraprotein and helps determine whether there is an underlying hematologic malignancy, such as multiple myeloma or another plasma cell dyscrasia, which could explain the patient's neuropathy.**<br>● **Identifying or ruling out a significant hematologic condition is crucial for directing appropriate management and treatment.**<br>● **Given the patient's age and the presence of a paraprotein, it is essential to exclude serious underlying conditions that could have significant implications for prognosis and treatment.**<br>**Therefore, the bone marrow biopsy and aspiration is the most crucial additional examination to further narrow down the differential diagnoses and guide the patient's management plan.**
52	A 77-year-old male patient presents with a 6-month history of sensory-motor, symmetrical, non-length dependent polyneuropathy with subacute onset. The patient presents with weakness distributed proximally and distally in the lower limbs and distally in the upper limbs, and paresthesia in a stocking-glove distribution. Clinical examination reveals the presence of sensory ataxia, peripheral edema and hypertrichosis. There are no foot deformities, tremor, neuropathic pain, cranial nerve or autonomic involvement. Past medical history is positive for diabetes. Nerve conduction studies show mixed demyelinating-axonal, sensory-motor polyneuropathy. Screening lab tests including vitamin B12, folate, vitamin B6, vitamin E, HCV, HBV, ANA, ANCA, ENA, CRP, ESR, glycemia, copper and cryoglobulins are all negative, with positivity for IgM lambda paraprotein and raised HbA1c. There is no family history of polyneuropathy.	POEMS	**Step-by-Step Analysis and Additional Examinations**<br>**1. Review of Initial Clinical and Diagnostic Data:**<br>○ **77-year-old male with subacute onset sensory-motor, symmetrical, non-length dependent polyneuropathy.**<br>○ **Weakness proximally and distally in lower limbs, distally in upper limbs.**<br>○ **Paresthesia in a stocking-glove distribution.**<br>○ **Sensory ataxia, peripheral edema, hypertrichosis.**<br>○ **Diabetes with raised HbA1c.**<br>○ **Nerve conduction studies showing mixed demyelinating-axonal, sensory-motor polyneuropathy.**<br>○ **Positive IgM lambda paraprotein.**<br>○ **Negative for various other laboratory tests.**<br>**2. Necessary Additional Examinations:**<br>**Laboratory Tests**<br>**1. Serum Protein Electrophoresis (SPEP) and Immunofixation Electrophoresis (IFE):**<br>○ **Reasoning: To quantify the paraprotein and identify the type of monoclonal protein (IgM lambda in this case). This helps in diagnosing and distinguishing between MGUS, Waldenström's macroglobulinemia, and other plasma cell dyscrasias.**<br>**2. Serum Free Light Chain Assay:**<br>○ **Reasoning: To assess the ratio of kappa to lambda light chains, which can provide additional information about the nature and extent of monoclonal gammopathy and aid in diagnosing conditions like AL amyloidosis.**<br>**3. Bone Marrow Biopsy:**<br>○ **Reasoning: To evaluate for plasma cell dyscrasias, including multiple myeloma or Waldenström's macroglobulinemia. This is crucial in determining if the paraprotein is due to a malignant condition.**<br>**4. Anti-MAG Antibody Testing:**<br>○ **Reasoning: Anti-myelin-associated glycoprotein (MAG) antibodies are often associated with IgM paraproteinemic neuropathy. Their presence would support the diagnosis of a paraprotein-related neuropathy.**<br>**5. HbA1c and Fasting Glucose:**<br>○ **Reasoning: To confirm and further evaluate the level of glycemic control, as diabetic neuropathy is a significant differential diagnosis.**<br>**Instrumental Tests**<br>**6. MRI of the Spine:**

			○ **Reasoning: To evaluate for structural abnormalities that might contribute to neuropathy, particularly given the proximal weakness in the lower limbs. This can help rule out spinal stenosis or other compressive etiologies.**<br>7. **Lumbar Puncture with Cerebrospinal Fluid (CSF) Analysis:**<br>○ **Reasoning: To check for elevated protein levels and exclude infections or inflammatory processes involving the nervous system. High CSF protein with few cells (albuminocytologic dissociation) supports CIDP.**<br>8. **Autonomic Function Tests:**<br>○ **Reasoning: Although there is no current autonomic involvement, these tests can confirm or exclude subclinical autonomic neuropathy, which can refine the differential diagnosis.**<br>**Most Crucial Test**<br>**The most crucial test from the list is Serum Protein Electrophoresis (SPEP) and Immunofixation Electrophoresis (IFE).**<br>**Reasoning: This test is essential because it will confirm the presence and quantify the monoclonal protein, providing critical information about the type and level of paraprotein, which is central to diagnosing paraproteinemic neuropathy. It will help differentiate between MGUS and more severe conditions like multiple myeloma or Waldenström's macroglobulinemia. Given the presence of IgM lambda paraprotein, understanding its significance is pivotal in guiding further diagnostic and therapeutic steps.**
53	A 56-year-old male patient presents with a 13-month history of pure motor, asymmetric, non-length dependent polyneuropathy with insidious onset and progressive course. The patient presents with weakness in both hands with asymmetric distribution and reports having frequent cramps and fasciculations. Clinical examination reveals intrinsic hand atrophy. There are no sensory deficits or ataxia, tremor, cranial nerve or autonomic involvement. Past medical history is positive for systemic lupus erythematosus. Nerve conduction studies show a pure motor, demyelinating multineuropathy. Screening lab tests including vitamin B12, folate, vitamin B6, vitamin E, HCV, HBV, ANA, ANCA, ENA, CRP, ESR, glycemia, HbA1c, copper, cryoglobulins, and serum electrophoresis are negative. Cervical spine MRI is normal. There is no family history of polyneuropathy.	MMN	**Step-by-Step Analysis of Additional Examinations**<br>**Step 1: Review Current Information and Identify Gaps**<br>● **Patient Information: 56-year-old male, 13-month history of pure motor, asymmetric, non-length dependent polyneuropathy.**<br>● **Key Findings: Weakness, cramps, fasciculations, intrinsic hand atrophy, no sensory deficits, negative extensive lab work, normal cervical spine MRI.**<br>● **Considered Differential Diagnoses: MMN, CIDP (pure motor variant), paraneoplastic neuropathy.**<br>**Step 2: Identify Necessary Additional Examinations**<br>**To narrow down the differential diagnoses, the following additional tests can be considered:**<br>1. **Anti-GM1 Antibody Testing**<br>○ **Reasoning: Anti-GM1 antibodies are often present in patients with MMN. This test is crucial as a positive result would strongly support the diagnosis of MMN.**<br>○ **Details: Enzyme-linked immunosorbent assay (ELISA) or other immunoassays to detect the presence of anti-GM1 antibodies.**<br>2. **Lumbar Puncture with Cerebrospinal Fluid (CSF) Analysis**<br>○ **Reasoning: CSF analysis can help differentiate between various demyelinating conditions. For example, elevated protein levels without pleocytosis (increased white blood cells) can be seen in CIDP, while MMN typically has normal CSF findings.**<br>○ **Details: Measurement of CSF protein, cell count, glucose, and oligoclonal bands.**<br>3. **Repeat Electromyography (EMG) and Nerve Conduction Studies (NCS) with Focus on Different Nerves**<br>○ **Reasoning: To further characterize the pattern and extent of nerve involvement, which can aid in distinguishing MMN from other motor neuropathies and CIDP.**<br>○ **Details: Conduct EMG and NCS on additional muscles and nerves not previously tested to confirm demyelination and rule out other patterns.**<br>4. **Imaging Studies: Whole Body PET-CT Scan**<br>○ **Reasoning: To rule out occult malignancy as a cause of paraneoplastic neuropathy. PET-CT is sensitive for detecting malignancies and can help identify any hidden tumors.**<br>○ **Details: Whole body scan to detect abnormal metabolic activity indicative of malignancy.**<br>5. **Immunoelectrophoresis of Serum and Urine**<br>○ **Reasoning: To detect monoclonal proteins (paraproteins) that may be associated with conditions like POEMS syndrome (Polyneuropathy, Organomegaly, Endocrinopathy, Monoclonal protein, and Skin changes) or other paraneoplastic syndromes.**<br>○ **Details: Assess for the presence of monoclonal gammopathy in serum and urine samples.**<br>6. **Genetic Testing for Hereditary Neuropathies**<br>○ **Reasoning: Although there is no family history, late-onset hereditary motor neuropathies can occasionally present without a clear familial pattern. Testing for mutations in relevant genes can be useful if other tests are inconclusive.**<br>○ **Details: Genetic panels for hereditary motor neuropathies.**<br>7. **Autoimmune Panel Expansion**<br>○ **Reasoning: Given the patient's history of SLE, expanding the autoimmune panel could help identify other potential autoimmune causes of neuropathy.**<br>○ **Details: Additional tests for antibodies such as anti-MAG (myelin-associated glycoprotein), anti-GQ1b, anti-sulfatide, and others.**<br>**Step 3: Prioritize and Determine the Most Crucial Test**<br>**Most Crucial Test: Anti-GM1 Antibody Testing**<br>● **Reasoning: Based on the clinical presentation and nerve conduction study results, MMN is the most likely diagnosis. Anti-GM1 antibody testing is highly specific for MMN and can confirm the diagnosis, guiding appropriate treatment with immunoglobulin therapy.**<br>● **Impact: A positive anti-GM1 antibody test would significantly narrow the diagnosis to MMN, allowing for targeted treatment and improving prognosis.**<br>**Summary of Proposed Tests with Reasoning**<br>1. **Anti-GM1 Antibody Testing: High specificity for MMN, essential for confirming diagnosis.**<br>2. **Lumbar Puncture with CSF Analysis: Differentiates between CIDP and MMN.**<br>3. **Repeat EMG and NCS: Further characterizes neuropathy pattern.**<br>4. **Whole Body PET-CT Scan: Rules out occult malignancy in paraneoplastic syndrome.**<br>5. **Immunoelectrophoresis of Serum and Urine: Detects monoclonal proteins associated with paraneoplastic syndromes.**<br>6. **Genetic Testing for Hereditary Neuropathies: Rules out hereditary causes if other tests are inconclusive.**<br>7. **Expanded Autoimmune Panel: Identifies other autoimmune neuropathies.**<br>**By focusing on the anti-GM1 antibody test first, we can confirm the most likely diagnosis and proceed with appropriate treatment efficiently.**
54	A 74-year-old male patient presents with a 5-year history of sensory-motor, predominantly sensory, symmetrical, length dependent polyneuropathy with insidious onset and slow progressive course. The patient presents with distal	Diabetic neuropathy	**Step-by-Step Analysis of the Clinical Scenario**<br>1. **Review Patient Data:**<br>○ **74-year-old male with 5-year history of sensory-motor, symmetrical, length-dependent polyneuropathy**<br>○ **Symptoms: distal paresthesia, hypoesthesia, mild neuropathic pain in the feet**<br>○ **No tremor, foot deformities, cranial nerve or autonomic involvement**<br>○ **Past medical history: diabetes and cardiac insufficiency**

	paresthesia and hypoesthesia associated with mild neuropathic pain in the feet. At clinical examination there are no tremor, foot deformities, cranial nerve or autonomic involvement. Past medical history is positive for diabetes and cardiac insufficiency. Nerve conduction studies show a sensory-motor, symmetric, length dependent, axonal polyneuropathy. Screening lab tests show hyperglycemia and elevated HbA1c. Remaining exams including vitamin B12, folate, vitamin B6, vitamin E, HBV, HCV, ANA, ANCA, ENA, CRP, ESR, copper, cryoglobulins, and serum electrophoresis are negative. There is family history of polyneuropathy (father had similar symptoms).		○ **Nerve conduction studies: sensory-motor, symmetric, length-dependent, axonal polyneuropathy**<br>○ **Laboratory findings: hyperglycemia, elevated HbA1c, negative for various other tests**<br>○ **Family history of polyneuropathy**<br>2. **Identify Differential Diagnoses:**<br>○ **Diabetic polyneuropathy**<br>○ **Hereditary sensory and motor neuropathy (HSMN)**<br>○ **Idiopathic axonal polyneuropathy (IAP)**<br>**Additional Examinations**<br>1. **Genetic Testing:**<br>○ **Reasoning: Given the family history of similar symptoms, genetic testing can help identify specific mutations associated with hereditary neuropathies such as Charcot-Marie-Tooth disease.**<br>○ **Tests: Genetic panels for hereditary neuropathy (e.g., PMP22 duplication/deletion, GJB1, MPZ mutations).**<br>2. **Skin Biopsy:**<br>○ **Reasoning: A skin biopsy can be used to assess intraepidermal nerve fiber density, which is reduced in small fiber neuropathies. This can help differentiate between large and small fiber neuropathies.**<br>○ **Tests: Intraepidermal nerve fiber density (IENFD) assessment.**<br>3. **Glucose Tolerance Test (GTT):**<br>○ **Reasoning: To evaluate the patient's glucose metabolism more comprehensively, particularly if diabetes is poorly controlled or if there are concerns about the accuracy of HbA1c.**<br>○ **Tests: Oral glucose tolerance test (OGTT).**<br>4. **Autonomic Testing:**<br>○ **Reasoning: Although the patient does not have autonomic symptoms, autonomic testing can provide additional information on the extent of neuropathy.**<br>○ **Tests: Heart rate variability, quantitative sudomotor axon reflex test (QSART).**<br>5. **Electromyography (EMG):**<br>○ **Reasoning: To provide more detailed information on the extent and distribution of nerve and muscle involvement.**<br>○ **Tests: Electromyography with nerve conduction studies.**<br>6. **Lumbar Puncture:**<br>○ **Reasoning: To assess cerebrospinal fluid (CSF) for signs of inflammation or infection, which can sometimes be associated with neuropathies.**<br>○ **Tests: CSF analysis (protein levels, cell count, oligoclonal bands).**<br>7. **Serum Protein Electrophoresis with Immunofixation (SPEP/IFE):**<br>○ **Reasoning: To check for monoclonal gammopathies, which can be associated with neuropathy.**<br>○ **Tests: Serum protein electrophoresis with immunofixation.**<br>**Most Crucial Test**<br>**Genetic Testing**<br>**Reasoning:**<br>● **Given the family history of similar symptoms, genetic testing is crucial to determine if there is an underlying hereditary neuropathy. Identifying specific genetic mutations can confirm a diagnosis of hereditary sensory and motor neuropathy, which will significantly influence management and prognosis. This test will help differentiate between diabetic polyneuropathy and hereditary neuropathy, particularly since the patient has a clear family history suggesting a genetic component.**
55	A 70-year-old male patient presents with a 2-year history of sensory-motor, symmetrical, length dependent polyneuropathy with subacute onset. He has been suffering from neuropathic pain in his feet for several years. However, over the past two years, he has also noticed progressive weakness in the feet and wasting of the intrinsic muscles of both hands. Additionally, he reports symptoms suggestive of orthostatic hypothension, and unintentional weight loss of approximately 5 kg over the past year. Clinical examination does not show presence of tremor, foot deformities, or cranial nerve involvement. Past medical history is positive for cardiac insufficiency, diabetes, and IgG lambda MGUS. Nerve conduction studies show a mixed demyelinating-axonal, sensory-motor, symmetric polyneuropathy. Screening lab tests reveal hyperglycemia, elevated HbA1c, presence of IgG lambda paraprotein, and increased creatinine levels. Remaining exams including vitamin B12, folate, vitamin B6, vitamin E, HBV, HCV, ANA, ANCA, ENA, CRP, ESR, copper,	Amyloidosis-associated polyneuropathy	**Additional Examinations for Narrowing Down Differential Diagnoses**<br>**1. Serum and Urine Immunofixation Electrophoresis**<br>**Reasoning:**<br>● **To detect and characterize monoclonal proteins (paraproteins) more accurately.**<br>● **Given the patient's history of IgG lambda MGUS, confirming the presence and type of paraprotein is crucial.**<br>● **Helps differentiate between MGUS, smoldering myeloma, and active multiple myeloma.**<br>**2. Serum Free Light Chain Assay**<br>**Reasoning:**<br>● **Measures the levels of free kappa and lambda light chains in the blood.**<br>● **Helps in assessing the burden of monoclonal light chains, which is important in the context of amyloidosis.**<br>● **An abnormal kappa/lambda ratio can suggest light chain amyloidosis.**<br>**3. Fat Pad Biopsy**<br>**Reasoning:**<br>● **A minimally invasive procedure to check for amyloid deposits in the subcutaneous fat.**<br>● **Positive amyloid deposits would strongly support a diagnosis of amyloidosis, particularly AL amyloidosis.**<br>**4. Nerve Biopsy**<br>**Reasoning:**<br>● **Provides direct evidence of amyloid deposits or other pathologic changes in the peripheral nerves.**<br>● **Can help confirm amyloid neuropathy and exclude other causes of neuropathy.**<br>**5. Electrocardiogram (ECG) and Echocardiogram**<br>**Reasoning:**<br>● **To assess for cardiac involvement which is common in AL amyloidosis.**<br>● **ECG can show low voltage or pseudo-infarction patterns.**<br>● **Echocardiogram can reveal features of amyloidosis such as thickened ventricular walls and diastolic dysfunction.**<br>**6. Cardiac Biomarkers (NT-proBNP and Troponin T)**<br>**Reasoning:**<br>● **Elevated levels can indicate cardiac involvement in amyloidosis.**

	and cryoglobulins are normal. There is no family history of polyneuropathy.		● **Helps in staging and prognosis of AL amyloidosis.**<br>**7. Autonomic Function Tests**<br>**Reasoning:**<br>● **To objectively evaluate the severity of autonomic dysfunction, which is prominent in amyloid neuropathy.**<br>● **Tests include tilt table testing, heart rate variability, and sudomotor function tests.**<br>**8. 24-Hour Urine Protein and Creatinine Clearance**<br>**Reasoning:**<br>● **To assess for renal involvement, which is common in amyloidosis.**<br>● **Helps in quantifying proteinuria and evaluating renal function.**<br>**9. Positron Emission Tomography (PET) or Whole-Body MRI**<br>**Reasoning:**<br>● **For systemic evaluation of amyloid deposits and ruling out systemic involvement of MGUS or myeloma.**<br>● **PET scans can identify areas of increased metabolic activity suggestive of malignancy or inflammation.**<br>**10. Bone Marrow Biopsy**<br>**Reasoning:**<br>● **To evaluate for plasma cell disorders such as multiple myeloma or amyloidosis.**<br>● **Essential in cases with suspected light chain amyloidosis to determine the percentage of plasma cells and presence of amyloid deposits.**<br>**Most Crucial Test**<br>**Fat Pad Biopsy**<br>**Reasoning:**<br>● **Given the high suspicion of amyloidosis based on clinical presentation and the presence of IgG lambda paraprotein, confirming the diagnosis is essential.**<br>● **Fat pad biopsy is a minimally invasive procedure with a high diagnostic yield for detecting amyloid deposits.**<br>● **Positive results would provide definitive evidence supporting AL amyloidosis, guiding further specific treatment strategies.**<br>**Conclusion**<br>● **Most crucial test: Fat pad biopsy, due to its high diagnostic yield and minimally invasive nature, is essential for confirming amyloid deposits and establishing a definitive diagnosis of AL amyloidosis. This will enable targeted management and treatment.**
56	A 53-year-old female patient presents with a 42-year history of sensory-motor, symmetrical, length-dependent polyneuropathy with insidious onset and slowly progressive course. The patient reports distal leg paresthesia, scoliosis, sensory ataxia, distal weakness in the lower limbs, cramps, hammertoes, and pes cavus. Clinical examination does not reveal tremor, cranial nerve involvement, or autonomic dysfunction. Past medical history is unremarkable. Nerve conduction studies show a demyelinating, symmetric, sensory-motor polyneuropathy. Screening lab tests including vitamin B12, folate, vitamin B6, vitamin E, HBV, HCV, glycemia, HbA1c, ANA, ANCA, ENA, CRP, ESR, copper, cryoglobulins, and serum electrophoresis are all negative. Her mother, brother, and sister have walking difficulties.	CMT1	**Step-by-Step Analysis**<br>**Comprehensive Data Recap**<br>**1. Patient Details:**<br>○ **53-year-old female with a 42-year history of slowly progressive sensory-motor, symmetrical, length-dependent polyneuropathy.**<br>○ **Symptoms: Distal leg paresthesia, sensory ataxia, distal weakness in the lower limbs, cramps, hammertoes, pes cavus.**<br>○ **No tremor, cranial nerve involvement, or autonomic dysfunction.**<br>○ **Family history of walking difficulties.**<br>**2. Previous Test Results:**<br>○ **Nerve conduction studies: Demyelinating, symmetric, sensory-motor polyneuropathy.**<br>○ **Negative lab tests: Vitamin B12, folate, vitamin B6, vitamin E, HBV, HCV, glycemia, HbA1c, ANA, ANCA, ENA, CRP, ESR, copper, cryoglobulins, and serum electrophoresis.**<br>**Additional Examinations**<br>**1. Genetic Testing for Hereditary Neuropathies**<br>● **Reasoning:**<br>○ **Given the family history and clinical presentation suggestive of a hereditary neuropathy (e.g., CMT), genetic testing can confirm the diagnosis.**<br>○ **Genetic panels for CMT can identify mutations in genes such as PMP22, MPZ, GJB1, and others commonly associated with hereditary neuropathies.**<br>○ **This test is crucial because a positive result would confirm the diagnosis and guide management and genetic counseling.**<br>**2. Nerve Biopsy**<br>● **Reasoning:**<br>○ **A nerve biopsy can provide histological evidence of demyelination, remyelination, and axonal loss, which are characteristic of specific neuropathies.**<br>○ **This can help differentiate between hereditary and acquired causes of demyelinating neuropathy.**<br>○ **It can also help identify other rare neuropathies that might not be detected through genetic testing.**<br>**3. Electromyography (EMG)**<br>● **Reasoning:**<br>○ **EMG can further characterize the nature and extent of muscle involvement and distinguish between neuropathic and myopathic changes.**<br>○ **This test complements nerve conduction studies by providing additional information on muscle denervation and reinnervation.**<br>**4. MRI of the Spine and Peripheral Nerves**<br>● **Reasoning:**<br>○ **MRI can detect structural abnormalities in the spinal cord and peripheral nerves, which might contribute to or mimic polyneuropathy.**<br>○ **It can help rule out compressive lesions, such as herniated discs or tumors, and provide detailed imaging of nerve roots and plexuses.**<br>**5. Lumbar Puncture with CSF Analysis**<br>● **Reasoning:**<br>○ **Analyzing cerebrospinal fluid (CSF) can help identify inflammatory or infectious causes of neuropathy.**<br>○ **Elevated protein levels with a normal cell count (albumin-cytologic dissociation) are indicative of CIDP.**<br>○ **This test helps rule out other potential causes of polyneuropathy such as infections, malignancies, or autoimmune disorders.**<br>**6. Detailed Autonomic Function Testing**<br>● **Reasoning:**<br>○ **Even though there is no autonomic dysfunction on initial examination, detailed testing can uncover subtle autonomic involvement.**

<table>
<tr><td></td><td></td><td></td><td>○ Tests like tilt-table testing, heart rate variability, and sweat tests can provide a comprehensive evaluation of the autonomic nervous system.
Most Crucial Test
The most crucial test from the list is Genetic Testing for Hereditary Neuropathies. This test is fundamental because:
● The clinical presentation and family history strongly suggest a hereditary neuropathy such as Charcot-Marie-Tooth Disease.
● Genetic testing can provide a definitive diagnosis, which is critical for guiding management, prognosis, and genetic counseling for the patient and her family.
● Confirming a genetic mutation associated with CMT or another hereditary neuropathy will significantly narrow down the differential diagnoses and eliminate the need for some of the more invasive tests, such as nerve biopsy.
Conclusion
Genetic testing for hereditary neuropathies is the most crucial test to conduct in this patient due to the high suspicion of Charcot-Marie-Tooth Disease based on clinical, family history, and previous test results. This will confirm the diagnosis and guide appropriate management strategies.</td></tr>
<tr><td>57</td><td>A 65-year old male patient presents with a 3-year history of sensory, symmetrical, length dependent polyneuropathy with insidious onset. The patient presents with sensory ataxia, neuropathic pain, distal hypoesthesia up to the calves and mild dysarthria. Clinical examination does not reveal presence of foot deformities, tremor, or weakness. Past medical history shows chronic cough and mild cardiac insufficiency. Nerve conduction studies show a pure sensory, axonal polyneuropathy. Screening lab tests including vitamin B12, folate, vitamin B6, vitamin E, HBV, HCV, glycemia, HbA1c, ANA, ANCA, ENA, CRP, ESR, copper, cryoglobulins, and serum electrophoresis are all negative. There is no family history of polyneuropathy.</td><td>CANVAS</td><td>Step-by-Step Analysis for Additional Examinations
Summary of Previous Analysis
1. Patient Details:
○ 65-year-old male
2. Symptom History:
○ 3-year history of sensory, symmetrical, length-dependent polyneuropathy with insidious onset
○ Sensory ataxia, neuropathic pain, distal hypoesthesia up to the calves, mild dysarthria
3. Clinical Examination:
○ No foot deformities, tremor, or weakness
4. Medical History:
○ Chronic cough, mild cardiac insufficiency
5. Diagnostic Findings:
○ Nerve conduction studies: Pure sensory, axonal polyneuropathy
6. Screening Lab Tests:
○ Negative results for: Vitamin B12, folate, vitamin B6, vitamin E, HBV, HCV, glycemia, HbA1c, ANA, ANCA, ENA, CRP, ESR, copper, cryoglobulins, serum electrophoresis
7. Family History:
○ No family history of polyneuropathy
Additional Examinations
1. Cerebrospinal Fluid (CSF) Analysis
Reasoning:
● CIDP Diagnosis:
○ CSF analysis can detect elevated protein levels with normal cell count (albuminocytologic dissociation), which is a hallmark of CIDP.
○ Given the chronic nature and atypical presentation, ruling out CIDP with CSF analysis is crucial.
● Infection or Inflammation:
○ CSF analysis can also help identify infections, inflammatory processes, or malignant cells not detected in blood tests.
2. Anti-Hu and Other Paraneoplastic Antibodies
Reasoning:
● Paraneoplastic Syndromes:
○ Testing for paraneoplastic antibodies (e.g., anti-Hu, anti-Yo, anti-Ri) can help identify a paraneoplastic syndrome, which could explain the sensory neuropathy and mild dysarthria.
● Associated Malignancies:
○ Chronic cough and cardiac issues might be indirect signs of an occult malignancy. Identifying paraneoplastic antibodies can guide further cancer screening.
3. Chest X-ray or Chest CT Scan
Reasoning:
● Chronic Cough Evaluation:
○ Chronic cough can be a symptom of lung pathology, including malignancy, which might present with paraneoplastic syndrome.
○ A chest X-ray or CT scan can identify any lung masses, infections, or other abnormalities contributing to the patient's symptoms.
4. Serum Immunofixation Electrophoresis (IFE)
Reasoning:
● Monoclonal Gammopathy:
○ Serum IFE can detect monoclonal proteins not identified by standard serum electrophoresis. This is important for diagnosing conditions like monoclonal gammopathy of undetermined significance (MGUS) or multiple myeloma, which can be associated with sensory polyneuropathy.
5. Autoimmune Panel:
Reasoning:
● Expanded Autoimmune Testing:
○ Although initial autoimmune tests were negative, further testing for specific antibodies (e.g., Sjögren's syndrome antibodies, ganglioside antibodies) can help identify autoimmune causes of neuropathy.
6. High-Resolution Ultrasound of Peripheral Nerves
Reasoning:
● Peripheral Nerve Pathology:
○ High-resolution ultrasound can help visualize nerve enlargement or structural abnormalities, supporting the diagnosis of CIDP or other peripheral nerve conditions.
Most Crucial Test
The most crucial test to perform is Cerebrospinal Fluid (CSF) Analysis.
Rationale:
● CSF analysis is highly valuable in diagnosing CIDP, which remains a probable differential diagnosis given the chronic progression and sensory nature of the neuropathy.
● It can provide insights into possible inflammatory, infectious, or paraneoplastic processes that might not be evident in blood tests.</td></tr>
</table>

			● Given the patient's symptoms and negative initial lab tests, CSF analysis offers the potential for a definitive diagnosis, guiding further management and treatment strategies.<br>**Conclusion**<br>**Performing a CSF analysis will be pivotal in confirming or excluding CIDP, identifying other possible causes like infections or malignancies, and ultimately narrowing down the differential diagnoses in this complex clinical scenario.**
58	A 71-year-old female patient presents with a 2-year history of chronic, pure sensory, symmetrical, length dependent polyneuropathy with subacute onset. The patient presents with distal leg paresthesia and neuropathic pain associated with cramps. There are no foot deformities, weakness, ataxia, tremor, cranial nerve or autonomic involvement. Past medical history shows diabetes and lymphoma, for which the patient has undergone chemotherapy. Symptoms started during chemotherapy. Nerve conduction studies show mixed sensory-motor, axonal, symmetric polyneuropathy and unilateral carpal tunnel syndrome. Screening lab tests including vitamin B12, folate, vitamin B6, vitamin E, HBV, HCV, ANA, ANCA, ENA, CRP, ESR, glycemia, copper, cryoglobulins and serum electrophoresis are all negative. HbA1c is elevated. There is no family history of polyneuropathy.	Chemotherapy-induced neuropathy	**Step-by-Step Analysis of Comprehensive Data and Additional Examinations**<br>**Step 1: Review of Clinical Information**<br>● **Patient Profile: 71-year-old female**<br>● **History: 2-year history of chronic, pure sensory, symmetrical, length-dependent polyneuropathy with subacute onset**<br>● **Symptoms: Distal leg paresthesia, neuropathic pain, cramps**<br>● **Examination Findings: No foot deformities, weakness, ataxia, tremor, cranial nerve or autonomic involvement**<br>● **Medical History: Diabetes, lymphoma (chemotherapy)**<br>● **Symptom Onset: During chemotherapy**<br>● **Nerve Conduction Studies: Mixed sensory-motor, axonal, symmetric polyneuropathy and unilateral carpal tunnel syndrome**<br>● **Laboratory Findings: Negative for multiple tests, elevated HbA1c**<br>● **Family History: No polyneuropathy**<br>**Step 2: Identification of Additional Examinations**<br>**1. Skin Biopsy for Intraepidermal Nerve Fiber Density (IENFD)**<br>○ **Reasoning:**<br>■ **Evaluates small fiber neuropathy which can be missed in nerve conduction studies.**<br>■ **Useful in diagnosing sensory neuropathies and understanding the degree of nerve fiber loss.**<br>■ **Relevant due to the patient's chronic sensory symptoms.**<br>**2. Quantitative Sensory Testing (QST)**<br>○ **Reasoning:**<br>■ **Assesses sensory nerve function.**<br>■ **Measures thresholds for vibration, temperature, and pain.**<br>■ **Helps in differentiating small fiber neuropathy from other types.**<br>**3. Autonomic Function Testing**<br>○ **Reasoning:**<br>■ **Despite no current autonomic symptoms, autonomic involvement can be subtle.**<br>■ **Tests like heart rate variability, sweat tests, and tilt-table testing.**<br>■ **Important for comprehensive neuropathy assessment.**<br>**4. Lumbar Puncture for Cerebrospinal Fluid (CSF) Analysis**<br>○ **Reasoning:**<br>■ **To rule out inflammatory or infectious causes of neuropathy.**<br>■ **Useful if paraneoplastic neuropathy is suspected.**<br>■ **CSF analysis can reveal elevated proteins or malignant cells.**<br>**5. Serum Immunofixation Electrophoresis and Free Light Chain Assay**<br>○ **Reasoning:**<br>■ **Detects monoclonal gammopathies, which can cause neuropathy.**<br>■ **Important due to the patient's lymphoma history.**<br>**6. Antineuronal Antibody Testing (e.g., Anti-Hu, Anti-Ri)**<br>○ **Reasoning:**<br>■ **Evaluates for paraneoplastic neuropathy.**<br>■ **Antibodies associated with malignancies like lymphoma.**<br>**7. MRI of the Lumbar Spine**<br>○ **Reasoning:**<br>■ **Assess for any spinal pathologies contributing to neuropathy.**<br>■ **Identifies potential nerve compressions or lesions.**<br>**8. Glucose Tolerance Test**<br>○ **Reasoning:**<br>■ **Despite elevated HbA1c, further assessment of glucose metabolism.**<br>■ **Identifies glucose intolerance or undiagnosed diabetes contributing to neuropathy.**<br>**Step 3: Most Crucial Test**<br>**Skin Biopsy for Intraepidermal Nerve Fiber Density (IENFD)**<br>**Reasoning:**<br>● **The patient's symptoms are predominantly sensory and consistent with small fiber involvement, which may not be detected by nerve conduction studies.**<br>● **Skin biopsy provides direct evidence of small fiber neuropathy and can confirm the diagnosis by showing a reduction in nerve fiber density.**<br>● **It is a minimally invasive test with high diagnostic yield for sensory neuropathies, particularly useful in chemotherapy-induced and diabetic neuropathy assessment.**<br>**Conclusion**<br>**The Skin Biopsy for Intraepidermal Nerve Fiber Density (IENFD) is the most crucial test to narrow down the differential diagnosis in this patient. It offers specific insights into small fiber involvement, aiding in confirming the type and extent of neuropathy, thus guiding further management.**
59	An 80-year-old male patient presents with a 1-year history of sensory-motor, symmetrical, length dependent	Chemotherapy-induced neuropathy	**Step-by-Step Analysis and Additional Examinations**<br>**Step 1: Review of the Current Information**<br>● **Clinical Presentation: Sensory-motor, symmetrical, length-dependent polyneuropathy; paresthesia; sensory ataxia; distal weakness; hypoesthesia in lower limbs; no cranial or autonomic involvement.**

	polyneuropathy with subacute onset. The patient presents with paresthesia and sensory ataxia without tremor, neuropathic pain, foot deformities, cranial nerve or autonomic involvement. Clinical examination shows distal weakness and sensory deficits (hypoesthesia) in the lower limbs. Past medical history is positive for colorectal cancer and plasmacytoma, for which the patient has undergone chemotherapy. Neuropathy symptoms started a few weeks after the last chemotherapy treatment cycle. Nerve conduction studies show a sensory-motor, symmetric, length-dependent, axonal polyneuropathy. Screening lab tests including vitamin B12, folate, vitamin B6, vitamin E, HBV, HCV, ANA, ANCA, ENA, CRP, ESR, glycemia, HbA1c, copper and cryoglobulins are all negative, with positivity for IgG kappa paraprotein. There is no family history of polyneuropathy.		**● Past Medical History: Colorectal cancer, plasmacytoma (chemotherapy received).**<br>**● Nerve Conduction Studies: Sensory-motor, symmetric, length-dependent, axonal polyneuropathy.**<br>**● Lab Tests: Normal vitamin levels, negative viral and autoimmune markers, positive IgG kappa paraprotein.**<br>**Step 2: Formulate Differential Diagnoses**<br>**● Chemotherapy-Induced Peripheral Neuropathy (CIPN)**<br>**● Paraneoplastic Neuropathy**<br>**● Monoclonal Gammopathy of Undetermined Significance (MGUS) with Neuropathy**<br>**Step 3: Identify Gaps and Additional Examinations**<br>**1. Serum and Urine Protein Electrophoresis (SPEP/UPEP) and Immunofixation**<br>**○ Reasoning: To quantify and characterize the monoclonal protein detected (IgG kappa paraprotein). This helps in differentiating MGUS from multiple myeloma or other plasma cell dyscrasias. UPEP can detect Bence Jones proteins indicative of multiple myeloma.**<br>**2. Bone Marrow Biopsy and Aspiration**<br>**○ Reasoning: If the monoclonal protein levels are concerning, a bone marrow biopsy can help assess for multiple myeloma or other hematologic malignancies. This is crucial given the patient's history of plasmacytoma and the presence of IgG kappa paraprotein.**<br>**3. Lumbar Puncture with Cerebrospinal Fluid (CSF) Analysis**<br>**○ Reasoning: To evaluate for paraneoplastic neuropathy and other inflammatory or infectious causes of neuropathy. CSF analysis can detect abnormalities such as elevated protein levels or the presence of malignant cells.**<br>**4. Anti-Hu, Anti-Yo, and Other Paraneoplastic Antibodies**<br>**○ Reasoning: To assess for paraneoplastic neuropathy, which can occur in the context of the patient's cancer history. These antibodies are markers for paraneoplastic syndromes.**<br>**5. Skin Biopsy**<br>**○ Reasoning: To assess for small fiber neuropathy if large fiber involvement is not definitive. This can provide additional insights into the neuropathy's characteristics.**<br>**6. Quantitative Sensory Testing (QST)**<br>**○ Reasoning: To objectively measure sensory nerve function and quantify the extent of sensory deficits, providing a comprehensive profile of the sensory abnormalities.**<br>**7. MRI of the Spine**<br>**○ Reasoning: To exclude compressive radiculopathy or other structural causes of neuropathy, which might not be apparent on initial evaluations.**<br>**8. Positron Emission Tomography (PET) Scan**<br>**○ Reasoning: To identify any occult malignancies that could be contributing to a paraneoplastic syndrome. This is pertinent given the patient's cancer history.**<br>**Step 4: Prioritize Examinations**<br>**Most Crucial Test: Serum and Urine Protein Electrophoresis (SPEP/UPEP) and Immunofixation**<br>**Reasoning: Given the presence of IgG kappa paraprotein, it is essential to further characterize and quantify this protein to differentiate between MGUS, multiple myeloma, or other plasma cell dyscrasias. This test is crucial for identifying the underlying cause of the neuropathy and guiding subsequent diagnostic and therapeutic steps.**
60	A 22-year-old male patient presents with a 20-year history of sensory-motor, symmetrical, length dependent polyneuropathy with insidious onset and slow progression. The patient is wheelchair bound and presents with sensory ataxia, severe distal weakness and atrophy in the four limbs, hypoestesia up to calf and pes cavus, with no cranial nerve or autonomic involvement. Past medical history is unremarkable. Nerve conduction studies show a demyelinating, sensory-motor polyneuropathy. Screening lab tests including vitamin B12, folate, vitamin B6, vitamin E, HBV, HCV, glycemia, HbA1c, ANA, ANCA, ENA, CRP, ESR, copper, cryoglobulins, and serum electrophoresis are all negative. One of his five brothers has similar symptoms.	CMT4	**Step-by-Step Analysis for Additional Examinations**<br>**Summary of Pertinent Clinical Information**<br>**● Patient: 22-year-old male**<br>**● Symptoms: Sensory-motor, symmetrical, length-dependent polyneuropathy with insidious onset and slow progression, sensory ataxia, severe distal weakness and atrophy, hypoesthesia up to calves, pes cavus**<br>**● Family History: One brother with similar symptoms**<br>**● Nerve Conduction Studies: Demyelinating, sensory-motor polyneuropathy**<br>**● Screening Lab Tests: Negative for vitamin B12, folate, vitamin B6, vitamin E, HBV, HCV, glycemia, HbA1c, ANA, ANCA, ENA, CRP, ESR, copper, cryoglobulins, serum electrophoresis**<br>**● Unremarkable past medical history, no cranial nerve or autonomic involvement**<br>**Additional Examinations**<br>**1. Genetic Testing**<br>**○ Reasoning:**<br>**■ Charcot-Marie-Tooth Disease (CMT): Genetic testing for PMP22 duplication/deletion (CMT1A), MFN2 mutations (CMT2A), and other CMT-related genes.**<br>**■ Hereditary Neuropathy with Liability to Pressure Palsies (HNPP): Genetic testing for PMP22 deletion.**<br>**■ Metachromatic Leukodystrophy (MLD): Testing for ARSA gene mutations.**<br>**○ Impact: Direct confirmation of a hereditary neuropathy and identification of specific genetic mutations associated with the suspected conditions.**<br>**2. Electromyography (EMG)**<br>**○ Reasoning:**<br>**■ Differentiation of Neuropathy: Further characterization of the polyneuropathy, distinguishing between demyelinating and axonal types.**<br>**■ Severity and Distribution: Assessment of the extent and severity of muscle involvement.**<br>**○ Impact: Provides detailed information on the muscle response and helps to corroborate findings from nerve conduction studies.**<br>**3. Lumbar Puncture (CSF Analysis)**<br>**○ Reasoning:**<br>**■ Exclude Inflammatory Neuropathies: Check for elevated protein levels which can indicate chronic inflammatory demyelinating polyneuropathy (CIDP) or other inflammatory conditions.**<br>**○ Impact: Helps exclude or support inflammatory causes of neuropathy which may present similarly but require different management.**<br>**4. MRI of Brain and Spinal Cord**<br>**○ Reasoning:**<br>**■ Metachromatic Leukodystrophy (MLD): Assess for white matter changes typical of leukodystrophies.**<br>**■ Structural Anomalies: Identify any structural or compressive causes of neuropathy.**<br>**○ Impact: Provides visual evidence of central nervous system involvement or other structural abnormalities.**<br>**5. Repeat Nerve Conduction Studies and Segmental Nerve Biopsy**<br>**○ Reasoning:**<br>**■ Detailed Characterization: Confirm and expand on initial findings, ensuring accurate characterization of the neuropathy.**<br>**■ Biopsy: Histological examination to identify specific pathological features such as onion bulb formations (indicative of hereditary neuropathy).**

			○ Impact: Further corroborates findings and may reveal specific pathological features supportive of certain diagnoses.<br>6. Serum and Urine Arylsulfatase A Activity<br>○ Reasoning:<br>■ Metachromatic Leukodystrophy (MLD): Arylsulfatase A deficiency is a hallmark of MLD.<br>○ Impact: Direct biochemical confirmation of MLD.<br>**Most Crucial Test**<br>Genetic Testing is the most crucial test in this case. Given the strong family history and the clinical presentation suggestive of a hereditary neuropathy, genetic testing can provide a definitive diagnosis by identifying specific mutations associated with Charcot-Marie-Tooth disease, HNPP, or other hereditary neuropathies. This test will not only confirm the diagnosis but also aid in genetic counseling and management planning for the patient and potentially affected family members.
61	A 71-year-old male patient presents with a 4-year history of pure sensory, symmetrical, length dependent polyneuropathy with insidious onset. The patient presents with neuropathic pain and paresthesia in the feet. There are no sensory ataxia or tremor, foot deformities, cranial nerve or autonomic involvement. Clinical examination shows the presence of tactile hypoesthesia and hyperalgesia in the feet. Past medical history is positive for psoriasis and type 2 diabetes. Nerve conduction studies show a pure sensory, symmetric, axonal, length dependent polyneuropathy and unilateral carpal tunnel syndrome. Screening lab tests show positivity for ANA antibodies, mildly raised CRP and ESR, increased levels of glycemia and HbA1c. Remaining exams, including vitamin B12, vitamin B6, vitamin E, HBV, HCV, ANCA, ENA, copper, cryoglobulins and serum electrophoresis are all negative. There is no family history of polyneuropathy.	Diabetic neuropathy	**Step-by-Step Analysis and Additional Examinations**<br>**1. Comprehensive Blood Panel**<br>● Reasoning: To ensure no other systemic conditions are contributing to the neuropathy.<br>○ Complete Blood Count (CBC): To rule out anemia or infection.<br>○ Liver Function Tests (LFTs): To check for liver disease.<br>○ Kidney Function Tests (KFTs): To check for renal insufficiency, which can be associated with neuropathy.<br>**2. Autoimmune Panel**<br>● Reasoning: Positive ANA antibodies and raised CRP/ESR suggest a possible autoimmune process.<br>○ Anti-Double-Stranded DNA (Anti-dsDNA): To check for systemic lupus erythematosus (SLE).<br>○ Anti-Ro/SSA and Anti-La/SSB: To check for Sjogren's syndrome.<br>○ Rheumatoid Factor (RF) and Anti-Citrullinated Protein Antibody (ACPA): To check for rheumatoid arthritis.<br>**3. Glucose Tolerance Test (GTT)**<br>● Reasoning: To evaluate glucose metabolism and confirm or rule out prediabetes/diabetes more accurately.<br>○ Oral Glucose Tolerance Test (OGTT): Can provide detailed information on glucose handling and potential postprandial hyperglycemia.<br>**4. Nerve Conduction Studies (NCS) and Electromyography (EMG)**<br>● Reasoning: Further detail on the type and distribution of neuropathy, particularly to distinguish between axonal and demyelinating processes.<br>○ Repeat NCS/EMG: To monitor progression and severity of neuropathy.<br>**5. Skin Biopsy**<br>● Reasoning: To evaluate small fiber neuropathy, which might not be detected on standard NCS.<br>○ Intraepidermal Nerve Fiber Density (IENFD) Measurement: Can provide information on small fiber involvement.<br>**6. Cerebrospinal Fluid (CSF) Analysis**<br>● Reasoning: If autoimmune neuropathy is strongly suspected, CSF analysis can help.<br>○ CSF Protein and Cell Count: Elevated protein with normal cell count can support chronic inflammatory demyelinating polyneuropathy (CIDP).<br>**7. Imaging Studies**<br>● Reasoning: To rule out structural causes of neuropathy.<br>○ MRI of Lumbar Spine: To check for spinal stenosis or other compressive neuropathies.<br>○ Ultrasound of Peripheral Nerves: To assess for nerve entrapments or tumors.<br>**8. Immunoglobulin and Serum Protein Electrophoresis**<br>● Reasoning: To check for monoclonal gammopathy, which can be associated with neuropathy.<br>○ Immunofixation Electrophoresis: To detect monoclonal proteins.<br>○ Free Light Chains: To evaluate for light chain amyloidosis.<br>**9. Genetic Testing**<br>● Reasoning: Although there is no family history, late-onset hereditary neuropathies should be considered.<br>○ Genetic Panel for Hereditary Neuropathies: To identify genetic mutations.<br>**Most Crucial Test**<br>**Glucose Tolerance Test (GTT)**<br>● Reasoning: Given the patient's history of type 2 diabetes and elevated HbA1c, confirming the extent of glucose dysregulation is crucial. A GTT will provide a clear picture of glucose metabolism, which is fundamental in diagnosing diabetic polyneuropathy. Proper management of blood glucose levels is critical for treating and potentially slowing the progression of diabetic neuropathy. Thus, this test is not only diagnostic but also essential for guiding treatment.<br>**Conclusion**<br>The most crucial test from the list is the Glucose Tolerance Test (GTT), as it will provide critical information on the patient's glucose metabolism, which is central to confirming and managing diabetic polyneuropathy.
62	A 39-year-old male patient presents with a 1-month history of sensory-motor, symmetrical, non-length dependent polyneuropathy with acute onset. The patient presents with weakness, hypoesthesia, paresthesia and neuropathic pain in the four limbs, cramps, weakness of orbicularis oculi muscles, stypsis and urinary incontinence. Clinical examination reveals absence of tremor, ataxia or foot deformities. Past medical history is silent. Nerve conduction studies show a symmetric, demyelinating, sensory-motor	GBS	**Step-by-Step Analysis and Additional Examinations**<br>1. Lumbar Puncture (CSF Analysis)<br>○ Reasoning: In Guillain-Barré Syndrome (GBS), cerebrospinal fluid (CSF) analysis often shows elevated protein levels with a normal white cell count (albuminocytologic dissociation). This is a key diagnostic criterion for GBS. CIDP may show similar findings. This test is crucial in distinguishing between GBS/CIDP and other causes of polyneuropathy.<br>2. MRI of the Spine<br>○ Reasoning: MRI can help rule out structural causes of neuropathy such as spinal cord compression, which can mimic polyneuropathy. Additionally, it can show nerve root enhancement in cases of CIDP and sometimes GBS.<br>3. Antiganglioside Antibodies (e.g., Anti-GM1, Anti-GQ1b)<br>○ Reasoning: These antibodies can be present in variants of GBS, such as the Miller Fisher syndrome (associated with Anti-GQ1b) and can help differentiate between subtypes of GBS and other neuropathies.<br>4. Electromyography (EMG)<br>○ Reasoning: EMG can provide additional information about the type and extent of nerve damage, helping to differentiate between demyelinating and axonal neuropathies. It can also help in assessing the muscle involvement.<br>5. Autonomic Function Tests

	polyneuropathy. Screening lab tests including vitamin B12, folate, vitamin B6, vitamin E, HCV, HBV, ANA, ANCA, ENA, CRP, ESR, glycemia, HbA1c, copper, cryoglobulins and serum electrophoresis are all negative. There is no family history of polyneuropathy.		○ Reasoning: Given the patient's autonomic symptoms (urinary incontinence and constipation), autonomic function tests (e.g., heart rate variability, blood pressure response to tilt) can help assess the extent of autonomic involvement, which is more common in GBS and certain forms of CIDP.<br>6. Infectious Disease Panel (e.g., HIV, Lyme Disease, CMV, EBV)<br>○ Reasoning: Some infections can cause acute neuropathies. While initial screening tests were negative, more specific infectious panels can be useful, especially for conditions like HIV or Lyme disease that can present with similar symptoms.<br>7. Paraneoplastic Panel<br>○ Reasoning: Although less likely given the acute onset, paraneoplastic syndromes can cause neuropathies. This panel can help rule out underlying malignancies associated with neuropathies.<br>8. Repeat Nerve Conduction Studies<br>○ Reasoning: As the disease progresses, repeat studies can help monitor changes and the effectiveness of treatment, as well as provide further confirmation of the type of neuropathy.<br>Most Crucial Test<br>Lumbar Puncture (CSF Analysis) is the most crucial test from this list. This test is essential for confirming the diagnosis of GBS by demonstrating albuminocytologic dissociation, which is a hallmark of the disease. It also helps differentiate GBS from other potential causes of polyneuropathy, such as infectious, inflammatory, or neoplastic etiologies. CSF analysis can provide rapid and specific information that guides further management and treatment.
63	A 57-year-old female patient presents with a 1-month history of sensory-motor, symmetrical, length dependent polyneuropathy with subacute onset. The patient presents with severe sensory ataxia associated with hypoesthesia and paraesthesia in a stocking-glove distribution, as well as weakness of toe dorsiflexion. Clinical examination does not reveal cranial nerve or autonomic involvement, tremor, or foot deformities. Past medical history is positive for chronic autoimmune gastritis, chronic cough, type 2 diabetes, and arterial hypertension. Nerve conduction studies show presence of a sensory motor, length dependent, symmetric, axonal polyneuropathy. Screening lab tests reveal vitamin B12 deficiency, hyperglycaemia, elevated HbA1c, and positive ANA. Remaining exams including folate, vitamin B6, vitamin E, HBV, HCV, ANCA, ENA, CRP, ESR, copper, cryoglobulins, and serum electrophoresis are normal. There is no family history of polyneuropathy.	B12 deficiency neuropathy	Step-by-Step Additional Examination Analysis<br>1. Comprehensive Analysis of Previous Clinical Scenario<br>● History: Sensory-motor, symmetrical, length-dependent polyneuropathy, subacute onset, severe sensory ataxia, hypoesthesia, paraesthesia, weakness of toe dorsiflexion.<br>● Past Medical History: Chronic autoimmune gastritis, chronic cough, type 2 diabetes, arterial hypertension.<br>● Diagnostic Findings: Axonal polyneuropathy on nerve conduction studies, vitamin B12 deficiency, hyperglycemia, elevated HbA1c, positive ANA.<br>● Screening Lab Tests: Normal folate, vitamin B6, vitamin E, HBV, HCV, ANCA, ENA, CRP, ESR, copper, cryoglobulins, serum electrophoresis.<br>● No family history of polyneuropathy.<br>Additional Examinations to Narrow Down Differential Diagnoses<br>1. HbA1c and Blood Glucose Monitoring<br>● Reasoning: To confirm and further assess the extent of hyperglycemia and diabetes management. Diabetic neuropathy is a likely cause given the patient's history of type 2 diabetes and elevated HbA1c.<br>● Tests:<br>○ Continuous glucose monitoring (CGM).<br>○ Repeat HbA1c.<br>2. Methylmalonic Acid (MMA) and Homocysteine Levels<br>● Reasoning: Elevated MMA and homocysteine levels can help confirm the diagnosis of vitamin B12 deficiency as these markers are more sensitive than serum B12 alone.<br>● Tests:<br>○ Serum methylmalonic acid (MMA).<br>○ Serum homocysteine.<br>3. Autoimmune Panel<br>● Reasoning: Given the positive ANA, it is essential to screen for specific autoimmune diseases that could cause neuropathy, such as Sjogren's syndrome, systemic lupus erythematosus (SLE), and rheumatoid arthritis (RA).<br>● Tests:<br>○ Anti-Ro (SSA) and Anti-La (SSB) antibodies (for Sjogren's syndrome).<br>○ Anti-dsDNA, anti-Smith antibodies (for SLE).<br>○ Rheumatoid factor (RF) and anti-CCP (for RA).<br>4. CSF Analysis<br>● Reasoning: To rule out inflammatory or infectious causes of neuropathy. Particularly useful if considering Guillain-Barré syndrome or chronic inflammatory demyelinating polyneuropathy (CIDP).<br>● Tests:<br>○ Cerebrospinal fluid (CSF) analysis (including protein levels, cell count, oligoclonal bands).<br>5. Electromyography (EMG)<br>● Reasoning: To further characterize the nature and extent of neuropathy, helping to differentiate between axonal and demyelinating processes.<br>● Tests:<br>○ Electromyography (EMG) study.<br>6. Serum Protein Electrophoresis (SPEP) and Immunofixation Electrophoresis (IFE)<br>● Reasoning: To identify monoclonal gammopathies, which can be associated with peripheral neuropathy (e.g., multiple myeloma, MGUS).<br>● Tests:<br>○ Serum protein electrophoresis (SPEP).<br>○ Immunofixation electrophoresis (IFE).<br>7. Genetic Testing for Hereditary Neuropathies<br>● Reasoning: Although there's no family history, sporadic mutations can still occur. This helps in diagnosing conditions like Charcot-Marie-Tooth disease.<br>● Tests:<br>○ Genetic testing for common mutations causing hereditary neuropathies.<br>8. Skin Biopsy for Intraepidermal Nerve Fiber Density (IENFD)<br>● Reasoning: To evaluate for small fiber neuropathy, which might not be detected in routine nerve conduction studies.<br>● Tests:<br>○ Skin biopsy.<br>Most Crucial Test<br>The most crucial test is the Methylmalonic Acid (MMA) and Homocysteine Levels.<br>Reasoning:<br>● The patient has a documented vitamin B12 deficiency, which can cause severe neuropathy and ataxia.<br>● Elevated levels of MMA and homocysteine would confirm functional B12 deficiency, which is a treatable condition.<br>● Addressing and correcting B12 deficiency could potentially alleviate the neuropathic symptoms, making it imperative to confirm this diagnosis.

			**Conclusion**<br>**While many of these tests are important to rule out other differential diagnoses, confirming vitamin B12 deficiency through MMA and homocysteine levels is the most crucial next step due to its direct impact on treatment and potential for symptom reversal.**
64	A 74-year-old male patient presents with a 1-year history of sensory-motor, symmetrical, length dependent polyneuropathy with subacute onset and slow-progressive course. The patient presents with paresthesia and neuropathic pain distally in the lower limbs and sensory ataxia. Clinical examination reveals the presence of mild hand tremor and weakness of toe dorsiflexion. There are no foot deformities, cranial nerve or autonomic involvement. Past medical history is positive for cardiac insufficiency, diabetes and IgM monoclonal gammopathy. Nerve conduction studies show a demyelinating, sensory-motor, symmetric polyneuropathy. Screening lab tests reveal the presence of IgM paraprotein and mildly elevated HbA1c levels. Remaining lab tests including vitamin B12, folate, vitamin B6, vitamin E, HCV, HBV, ANA, ANCA, ENA, CRP, ESR, glycemia, copper, and cryoglobulins are negative. There is no family history of polyneuropathy.	Anti-MAG polyneuropathy	**Additional Examinations**<br>**1. Serum Protein Electrophoresis (SPEP) and Immunofixation Electrophoresis (IFE)**<br>**Reasoning:**<br>● **Purpose: To quantify and further characterize the monoclonal protein detected (IgM paraprotein) and to differentiate between MGUS, Waldenström macroglobulinemia, or other related conditions.**<br>● **Relevance: Helps confirm the presence of a monoclonal protein and provide more detail about its nature and potential clinical significance.**<br>**2. Nerve Biopsy**<br>**Reasoning:**<br>● **Purpose: To provide histological confirmation of the type of neuropathy, particularly useful in distinguishing between CIDP, MGUS-related neuropathy, and other forms of neuropathy.**<br>● **Relevance: Can show demyelination, axonal loss, and inflammatory infiltrates, providing direct evidence of the underlying pathophysiology.**<br>**3. Lumbar Puncture (CSF Analysis)**<br>**Reasoning:**<br>● **Purpose: To analyze cerebrospinal fluid (CSF) for elevated protein levels and the presence of cells, which can help in diagnosing CIDP and other inflammatory neuropathies.**<br>● **Relevance: Elevated CSF protein without pleocytosis is characteristic of CIDP, while the presence of a monoclonal protein in CSF could indicate a monoclonal gammopathy affecting the nervous system.**<br>**4. Anti-MAG (Myelin-Associated Glycoprotein) Antibodies**<br>**Reasoning:**<br>● **Purpose: To test for the presence of anti-MAG antibodies, which are often associated with IgM paraprotein-related neuropathies.**<br>● **Relevance: Helps differentiate MGUS-related neuropathy from other forms of neuropathy, as anti-MAG antibodies are commonly found in patients with IgM-related neuropathy.**<br>**5. MRI of the Spine with Gadolinium**<br>**Reasoning:**<br>● **Purpose: To evaluate for nerve root or spinal cord involvement and to identify any abnormalities that might suggest an inflammatory or demyelinating process.**<br>● **Relevance: Useful for diagnosing CIDP, as enhancement of the nerve roots can be indicative of an inflammatory process.**<br>**6. Quantitative Sensory Testing (QST)**<br>**Reasoning:**<br>● **Purpose: To assess the functional status of small and large nerve fibers.**<br>● **Relevance: Provides detailed information about the extent and distribution of sensory impairment, which can be helpful in characterizing the neuropathy.**<br>**7. Skin Biopsy**<br>**Reasoning:**<br>● **Purpose: To assess intraepidermal nerve fiber density.**<br>● **Relevance: Can help diagnose small fiber neuropathy, which may not be detected on nerve conduction studies.**<br>**8. Autonomic Function Testing**<br>**Reasoning:**<br>● **Purpose: To evaluate autonomic involvement which may be subtle and not apparent on routine clinical examination.**<br>● **Relevance: Although the patient does not have overt autonomic symptoms, subclinical autonomic involvement can be present in many forms of neuropathy, including diabetic and paraproteinemic neuropathies.**<br>**Most Crucial Test**<br>**Serum Protein Electrophoresis (SPEP) and Immunofixation Electrophoresis (IFE)**<br>**Conclusion: SPEP and IFE are the most crucial tests because they will provide detailed information about the monoclonal protein present in the patient's serum. This is essential for distinguishing between different types of monoclonal gammopathies, such as MGUS, Waldenström macroglobulinemia, or multiple myeloma, and understanding their potential role in the patient's neuropathy. Confirming and characterizing the monoclonal protein will significantly narrow down the differential diagnoses and guide further management.**
65	An 83-year-old female patient presents with a 6-week history of pure sensory, symmetrical, length dependent polyneuropathy with subacute onset. The patient presents with distal paresthesia, hypoesthesia, and neuropathic pain in the lower limbs with associated mild sensory ataxia. Clinical examination does not show the presence of weakness, tremor, foot deformities, cranial nerve or autonomic involvement. Romberg test resulted positive with inability to maintain postural station, feeling of instability, and inability to walk with closed eyes. Past medical history is positive for leukopenia, undifferentiated connective tissue disease, and type 2 diabetes. Nerve conduction studies show a pure sensory, symmetric, length dependent, axonal polyneuropathy. Screening lab tests show high glycemia and raised HbA1c, positivity for ANA	Vit B12 deficiency neuropathy	**Step-by-Step Analysis for Additional Examinations**<br>**Summary of Initial Findings**<br>● **Patient: 83-year-old female**<br>● **Symptoms: 6-week history of pure sensory, symmetrical, length-dependent polyneuropathy; distal paresthesia, hypoesthesia, neuropathic pain in lower limbs; mild sensory ataxia**<br>● **Past Medical History: Leukopenia, undifferentiated connective tissue disease, type 2 diabetes**<br>● **Nerve Conduction Studies: Pure sensory, symmetric, length-dependent, axonal polyneuropathy**<br>● **Laboratory Findings: High glycemia, raised HbA1c, positive ANA antibodies, vitamin B12 deficiency; negative for other relevant tests**<br>● **Clinical Examination: No weakness, tremor, foot deformities, cranial nerve or autonomic involvement; positive Romberg test**<br>**Additional Examinations and Reasoning**<br>**1. MRI of the Spine:**<br>○ **Reasoning:**<br>■ **To rule out structural causes of sensory neuropathy such as spinal cord compression, tumors, or myelopathy.**<br>■ **MRI can detect lesions in the posterior columns, which could explain the sensory ataxia and positive Romberg test.**<br>■ **Helps to identify any secondary complications related to undifferentiated connective tissue disease.**<br>**2. Serum Protein Electrophoresis and Immunofixation:**<br>○ **Reasoning:**<br>■ **To screen for monoclonal gammopathies such as multiple myeloma or MGUS, which can present as a sensory polyneuropathy.**<br>■ **The patient's age and unexplained leukopenia make these conditions more likely.**<br>**3. Autoantibody Panel (Anti-Ro, Anti-La, Anti-dsDNA, Anti-Sm, Anti-RNP, Anti-CCP):**<br>○ **Reasoning:**<br>■ **To further evaluate the underlying undifferentiated connective tissue disease.**

	antibodies, and evidence of vitamin B12 deficiency. Remaining exams including folate, vitamin B6, vitamin E, HCV, HBV, ANCA, CRP, ESR, copper, cryoglobulins, and serum electrophoresis are all negative. There is no family history of polyneuropathy.		■ **Positive ANA antibodies suggest an autoimmune etiology, and specific autoantibodies can help narrow down the diagnosis (e.g., systemic lupus erythematosus, Sjögren's syndrome, rheumatoid arthritis).**<br>4. **Oral Glucose Tolerance Test (OGTT) and Continuous Glucose Monitoring (CGM):**<br>○ **Reasoning:**<br>■ **To obtain a more comprehensive assessment of glucose metabolism and control.**<br>■ **Diabetes can significantly contribute to neuropathy, and better glucose monitoring can help tailor treatment strategies.**<br>5. **CSF Analysis (Cerebrospinal Fluid Analysis):**<br>○ **Reasoning:**<br>■ **To rule out inflammatory or infectious causes of neuropathy.**<br>■ **CSF analysis can help identify elevated protein levels, oligoclonal bands, or other markers of central nervous system involvement.**<br>6. **Skin Biopsy with Intraepidermal Nerve Fiber Density (IENFD) Testing:**<br>○ **Reasoning:**<br>■ **To assess for small fiber neuropathy, which may not be detected on routine nerve conduction studies.**<br>■ **Useful in cases where symptoms suggest neuropathy but conventional testing is inconclusive.**<br>7. **Complete Blood Count (CBC) with Differential and Peripheral Blood Smear:**<br>○ **Reasoning:**<br>■ **To investigate the cause of leukopenia and identify any hematological disorders.**<br>■ **Leukopenia could be related to an underlying malignancy, bone marrow disorder, or autoimmune condition.**<br>8. **Electromyography (EMG):**<br>○ **Reasoning:**<br>■ **To assess the electrical activity of muscles and help differentiate between axonal and demyelinating neuropathies.**<br>■ **Provides additional information on the extent and nature of nerve involvement.**<br>9. **Anti-Ganglioside Antibodies (e.g., Anti-GM1, Anti-GQ1b):**<br>○ **Reasoning:**<br>■ **To evaluate for possible immune-mediated neuropathies such as Guillain-Barré syndrome or chronic inflammatory demyelinating polyneuropathy (CIDP).**<br>■ **Although less likely given the sensory-only presentation, it is still worth considering in the differential diagnosis.**<br>**Most Crucial Test**<br>**Based on the initial clinical presentation and the need to clarify the underlying etiology of the neuropathy, serum protein electrophoresis and immunofixation is the most crucial test. This test can help detect monoclonal gammopathies, which are common in older adults and can present with sensory neuropathy. It also addresses the patient's leukopenia, which could be indicative of an underlying hematological disorder.**<br>**Conclusion**<br>● **Most Crucial Test: Serum protein electrophoresis and immunofixation**<br>● **Next Steps: Perform the test to assess for monoclonal gammopathies and then proceed with further testing based on the results to refine the diagnosis and guide treatment.**
66	A 71-year-old female patient presents with a 4-month history of pure sensory, symmetrical, length dependent polyneuropathy with subacute onset. The patient presents with neuropathic pain and paresthesia in the feet. She does not report weakness, ataxia, tremor, cranial nerve or autonomic involvement. Clinical examination shows hyperalgesia and allodynia in the feet. There are no foot deformities, sensory ataxia or weakness. Past medical history is positive for cervical cancer, for which the patient started chemotherapy 6 months ago. Nerve conduction studies show a sensory-motor, symmetric, length dependent polyneuropathy. Screening lab tests show raised CRP and ESR, and positivity for ANA antibodies. Remaining exams including vitamin B12, folate, vitamin B6, vitamin E, HBV, HCV, ANCA, ENA, glycemia, HbA1c, copper, cryoglobulins, and serum electrophoresis are negative. There is no family history of polyneuropathy.	Chemotherapy-induced neuropathy	**Step-by-Step Analysis for Additional Examinations**<br>**Summary of Case**<br>● **Clinical Presentation:**<br>○ **4-month history of sensory, symmetrical, length-dependent polyneuropathy.**<br>○ **Symptoms: Neuropathic pain and paresthesia in feet.**<br>○ **No weakness, ataxia, tremor, cranial nerve, or autonomic involvement.**<br>○ **Past medical history of cervical cancer treated with chemotherapy 6 months ago.**<br>● **Investigations:**<br>○ **Nerve conduction studies: Sensory-motor, symmetric, length-dependent polyneuropathy.**<br>○ **Lab results: Raised CRP and ESR, positive ANA antibodies, negative for other screening tests.**<br>○ **No family history of polyneuropathy.**<br>**Additional Examinations**<br>**1. CSF Analysis**<br>**Reasoning:**<br>● **To evaluate for inflammatory or infectious causes of neuropathy.**<br>● **Can help in diagnosing paraneoplastic neuropathy (e.g., presence of onconeural antibodies).**<br>● **Useful in ruling out Guillain-Barré syndrome and chronic inflammatory demyelinating polyneuropathy (CIDP).**<br>**2. Paraneoplastic Antibody Panel**<br>**Reasoning:**<br>● **Given the history of cervical cancer, it is crucial to evaluate for paraneoplastic syndromes.**<br>● **Can identify specific onconeural antibodies associated with paraneoplastic neuropathies, which might not be detected by ANA alone.**<br>**3. Skin Biopsy**<br>**Reasoning:**<br>● **To assess small fiber neuropathy, which may not be evident in nerve conduction studies.**<br>● **Can provide insights into intraepidermal nerve fiber density, helpful in diagnosing conditions like small fiber neuropathy due to autoimmune or paraneoplastic causes.**<br>**4. MRI of the Cervical and Lumbar Spine**<br>**Reasoning:**<br>● **To rule out compressive lesions, spinal cord abnormalities, or metastases that could contribute to neuropathy.**<br>● **Important to assess the structural integrity of the spinal cord and nerve roots, especially in a patient with a history of cancer.**<br>**5. Comprehensive Autoimmune Panel**<br>**Reasoning:**

			● **Given the positive ANA and elevated inflammatory markers, further testing for specific autoimmune markers (e.g., dsDNA, SSA/SSB, RNP, SM antibodies) could help diagnose systemic autoimmune conditions like SLE, Sjögren's syndrome, or mixed connective tissue disease.**<br>**6. PET-CT Scan**<br>**Reasoning:**<br>● **To detect any occult malignancies or metastases that might be contributing to a paraneoplastic syndrome.**<br>● **Provides comprehensive imaging to evaluate for any cancer recurrence or new malignancy, which can be associated with paraneoplastic neuropathies.**<br>**7. Quantitative Sensory Testing (QST)**<br>**Reasoning:**<br>● **To assess sensory nerve function in more detail.**<br>● **Can help quantify the degree of sensory impairment and distinguish between large and small fiber involvement.**<br>**Most Crucial Test**<br>**Paraneoplastic Antibody Panel**<br>**Reasoning:**<br>● **The history of cervical cancer and the timing of neuropathy onset strongly suggest a possible paraneoplastic etiology.**<br>● **Identifying specific onconeural antibodies can directly link the neuropathy to a paraneoplastic syndrome, guiding further management and treatment.**<br>● **This test can confirm or rule out a paraneoplastic cause, which has significant implications for prognosis and therapeutic decisions.**<br>**In conclusion, while all listed tests are important for a comprehensive evaluation, the paraneoplastic antibody panel is the most crucial due to its potential to directly identify a paraneoplastic syndrome related to the patient's cervical cancer history. This will help in confirming the diagnosis and tailoring the appropriate management plan.**
67	An 83-year-old male patient presents with a 1-year history of sensory-motor, symmetrical, length dependent polyneuropathy with insidious onset and progressive course. The patient presents with distal leg paresthesia, hypoesthesia, and sensory ataxia. Clinical examination confirms the presence of sensory ataxia with positive Romberg test and inability to walk with closed eyes. There is also postural hand tremor, which appeared concurrently with the other symptoms. Clinical examination does not show the presence of foot deformities, cranial nerve or autonomic involvement. Past medical history is positive for prostatic cancer, for which the patient underwent chemotherapy four years ago. Nerve conduction studies show a demyelinating, sensory-motor, length dependent polyneuropathy. Screening lab tests reveal the presence of IgM paraprotein and positivity for ANA antibodies. Remaining exams including vitamin B12, folate, vitamin B6, vitamin E, HCV, HBV, ANCA, ENA, CRP, ESR, glycemia, HbA1c, copper ,and cryoglobulins are negative. There is no family history of polyneuropathy.	Anti-MAG polyneuropathy	**Step-by-Step Analysis and Additional Examinations**<br>**Current Clinical Scenario Recap:**<br>● **Patient: 83-year-old male**<br>● **Symptoms: Sensory-motor, symmetrical, length-dependent polyneuropathy with insidious onset and progressive course over 1 year, distal leg paresthesia, hypoesthesia, sensory ataxia, and postural hand tremor.**<br>● **Medical History: Prostatic cancer treated with chemotherapy four years ago.**<br>● **Investigations: Demyelinating, sensory-motor polyneuropathy on nerve conduction studies, positive for IgM paraprotein and ANA antibodies, negative for a comprehensive list of other tests (e.g., vitamins, HCV, HBV, ANCA, ENA, CRP, ESR, glycemia, HbA1c, copper, cryoglobulins).**<br>● **Family History: No polyneuropathy.**<br>**Additional Examinations**<br>**1. Serum and Urine Immunofixation Electrophoresis (IFE)**<br>○ **Reasoning: To further characterize the monoclonal protein detected (IgM paraprotein) and assess for potential multiple myeloma or other plasma cell dyscrasias. IFE is more sensitive than standard serum protein electrophoresis (SPEP).**<br>**2. Bone Marrow Biopsy**<br>○ **Reasoning: If serum and urine IFE confirm monoclonal gammopathy, a bone marrow biopsy would help evaluate for multiple myeloma or other marrow-involving hematologic malignancies.**<br>**3. Anti-MAG Antibodies (Myelin-Associated Glycoprotein)**<br>○ **Reasoning: Anti-MAG antibodies are often found in patients with IgM paraproteinemic neuropathy, particularly those with a demyelinating pattern. This would support a diagnosis of MGUS-related neuropathy.**<br>**4. Lumbar Puncture with Cerebrospinal Fluid (CSF) Analysis**<br>○ **Reasoning: To assess for elevated protein without pleocytosis, which is a hallmark of CIDP. It can also help exclude infections or malignancies involving the central nervous system.**<br>**5. Skin Biopsy**<br>○ **Reasoning: To evaluate intraepidermal nerve fiber density (IENFD), which can help differentiate between axonal and demyelinating neuropathies, and provide evidence for small fiber neuropathy if suspected.**<br>**6. Serum Free Light Chain Assay**<br>○ **Reasoning: To detect light chain disease, which might not be evident in standard serum protein electrophoresis but can be detected in cases of MGUS, multiple myeloma, or other related disorders.**<br>**7. PET-CT Scan**<br>○ **Reasoning: To screen for occult malignancies given the history of prostatic cancer and potential for paraneoplastic syndrome. This can help identify any current neoplastic processes contributing to the neuropathy.**<br>**8. Detailed Autoimmune Panel**<br>○ **Reasoning: Although ANA is positive, further testing for specific autoantibodies (e.g., anti-dsDNA, anti-Smith, anti-Ro/SSA, anti-La/SSB) can help rule out or confirm underlying autoimmune conditions contributing to the neuropathy.**<br>**Most Crucial Test**<br>**Serum and Urine Immunofixation Electrophoresis (IFE)**<br>**Justification:**<br>● **The identification and characterization of the monoclonal protein are critical in determining whether the IgM paraprotein detected is indicative of MGUS, multiple myeloma, or another plasma cell disorder.**<br>● **This test provides essential information on the type of monoclonal protein, guiding further diagnostic steps such as bone marrow biopsy.**<br>● **It can help distinguish between benign monoclonal gammopathy and more serious hematologic conditions.**<br>**By conducting this test, the clinician can obtain a more precise understanding of the monoclonal gammopathy, which is a pivotal factor in the patient's neuropathy and subsequent diagnostic pathway.**
68	A 46-year-old male patient presents with a 3-year history of sensory-motor, symmetrical, non-length dependent polyneuropathy with insidious onset. The patient presents with leg paresthesia, sensory ataxia, proximal and distal weakness in the four limbs, and cramps. There are no cranial nerve or autonomic involvement, tremor, neuropathic pain or foot deformities. Past medical history	CIDP	**Step-by-Step Analysis and Additional Examinations**<br>**1. Detailed Blood and Serum Tests**<br>● **Reasoning: Further blood and serum tests can help identify or rule out additional causes of neuropathy and related conditions.**<br>○ **Serum Immunofixation Electrophoresis (IFE):**<br>■ **Reasoning: To further characterize the monoclonal protein and determine its light chain type (kappa or lambda). This can help differentiate between MGUS and more malignant conditions like multiple myeloma or amyloidosis.**<br>○ **Free Light Chain Assay:**<br>■ **Reasoning: Measures free kappa and lambda light chains in the blood. Useful in identifying light chain disease or amyloidosis, which may be associated with neuropathy.**<br>○ **Antibody Screening (Anti-MAG, anti-GM1, anti-GQ1b, anti-sulfatide):**<br>■ **Reasoning: To identify antibodies associated with specific neuropathies, particularly those seen in paraproteinemic neuropathies or other immune-mediated conditions.**

	shows IgG MGUS and chronic renal insufficiency. Nerve conduction studies show demyelinating, sensory-motor polyneuropathy in the four limbs. Screening lab tests including vitamin B12, folate, vitamin B6, vitamin E, HCV, HBV, ANA, ANCA, ENA, CRP, ESR, glycemia, HbA1c, copper and cryoglobulins are all negative. Serum electrophoresis confirms the presence of IgG monoclonal gammopathy. There is no family history of polyneuropathy.		**2. Urine Tests**<br>● **Reasoning: To evaluate for light chains and further investigate renal involvement.**<br>○ **24-Hour Urine Protein Electrophoresis:**<br>■ **Reasoning: To detect Bence Jones proteins (free light chains), which are indicative of multiple myeloma or other plasma cell dyscrasias.**<br>**3. Imaging Studies**<br>● **Reasoning: To assess for organomegaly or other systemic involvement.**<br>○ **Whole-Body PET/CT Scan:**<br>■ **Reasoning: To detect hidden organomegaly, lymphadenopathy, or other malignancies that might be contributing to the polyneuropathy. This is particularly relevant for POEMS syndrome and other malignancies.**<br>○ **MRI of the Spine:**<br>■ **Reasoning: To evaluate for spinal cord involvement or nerve root disease, which could contribute to the neuropathy.**<br>**4. Nerve and Muscle Biopsy**<br>● **Reasoning: To provide direct evidence of the nature of the neuropathy.**<br>○ **Sural Nerve Biopsy:**<br>■ **Reasoning: To evaluate for amyloid deposits, vasculitis, or other structural abnormalities in the nerves.**<br>○ **Muscle Biopsy:**<br>■ **Reasoning: To rule out myopathy or amyloid infiltration in muscles.**<br>**5. Cerebrospinal Fluid (CSF) Analysis**<br>● **Reasoning: To assess for inflammatory markers and other abnormalities.**<br>○ **Lumbar Puncture for CSF Analysis:**<br>■ **Reasoning: To look for elevated protein, oligoclonal bands, or other signs of inflammatory or infectious neuropathy. Elevated CSF protein without pleocytosis is a hallmark of CIDP.**<br>**Most Crucial Test**<br>**Serum Immunofixation Electrophoresis (IFE)**<br>● **Reasoning: While several tests are essential for a comprehensive evaluation, Serum Immunofixation Electrophoresis (IFE) is crucial because it provides detailed information about the monoclonal protein. Identifying the specific type and light chain of the monoclonal protein can significantly narrow down the differential diagnoses, distinguishing between benign MGUS and conditions such as multiple myeloma, Waldenström's macroglobulinemia, or amyloidosis. This test directly relates to the patient's existing IgG MGUS and its potential connection to the neuropathy.**<br>**Next Steps:**<br>● **Conduct the proposed additional examinations in a stepwise manner, starting with the most non-invasive and relevant tests, while continuously evaluating the results to refine the differential diagnosis further.**
69	A 78-year-old female patient presents with a 2-year history of sensory-motor, symmetrical, length dependent polyneuropathy with subacute onset. Clinical examination reveals presence of numbness, tingling, and neuropathic pain distally in the upper and lower limbs, weakness of ankle dorsiflexion, sensory ataxia, hand tremor and dysphonia, without autonomic involvement or foot deformities. Past medical history reveals diabetes and IgM kappa monoclonal gammopathy. Nerve conduction studies show a length dependent, symmetric, sensory-motor, demyelinating polyneuropathy. Screening lab tests including vitamin B12, folate, vitamin B6, vitamin E, HCV, HBV, ANA, ANCA, ENA, CRP, ESR, glycemia, copper and cryoglobulins are all negative, with positivity for IgM kappa paraprotein and raised HbA1c. There is no family history of polyneuropathy.	Anti-MAG polyneuropathy	**Step-by-Step Analysis and Additional Examinations**<br>**1. Comprehensive Data Review**<br>● **Patient Profile: 78-year-old female**<br>● **Symptoms Duration: 2 years**<br>● **Symptoms: Sensory-motor, symmetrical, length-dependent polyneuropathy, numbness, tingling, pain, weakness of ankle dorsiflexion, sensory ataxia, hand tremor, dysphonia**<br>● **Medical History: Diabetes mellitus, IgM kappa monoclonal gammopathy**<br>● **Nerve Conduction Studies: Demyelinating polyneuropathy**<br>● **Screening Lab Tests: Positive for IgM kappa paraprotein, raised HbA1c**<br>● **Negative Tests: Vitamin B12, folate, vitamin B6, vitamin E, HCV, HBV, ANA, ANCA, ENA, CRP, ESR, copper, cryoglobulins**<br>● **Family History: No polyneuropathy**<br>**2. Additional Examinations**<br>**1. Serum Immunofixation Electrophoresis (IFE)**<br>○ **Reasoning: To further characterize the monoclonal protein and confirm the presence and type of monoclonal gammopathy (IgM kappa). This is essential for confirming MGUS and ruling out more serious plasma cell dyscrasias like Waldenström's macroglobulinemia or multiple myeloma.**<br>**2. Quantitative Immunoglobulins (IgG, IgA, IgM)**<br>○ **Reasoning: To assess for immunoglobulin levels which can provide insight into immune system function and help identify immune-mediated neuropathies. Abnormal levels can indicate an underlying condition contributing to the neuropathy.**<br>**3. Lumbar Puncture with Cerebrospinal Fluid (CSF) Analysis**<br>○ **Reasoning: To check for elevated CSF protein levels, which are often seen in CIDP. CSF analysis can help distinguish between different types of neuropathies and is crucial for diagnosing inflammatory conditions like CIDP.**<br>**4. Anti-MAG (Myelin-Associated Glycoprotein) Antibodies**<br>○ **Reasoning: Anti-MAG antibodies are often found in patients with IgM monoclonal gammopathy and demyelinating neuropathy. Their presence would support a diagnosis of MGUS neuropathy associated with IgM paraprotein.**<br>**5. Nerve Biopsy**<br>○ **Reasoning: To provide histopathological evidence of the type of neuropathy (e.g., demyelination, axonal loss) and identify specific pathological features that could indicate a specific cause, such as amyloid deposits or inflammatory infiltrates.**<br>**6. Electromyography (EMG)**<br>○ **Reasoning: To complement nerve conduction studies and provide further information on the extent and pattern of nerve damage, helping to differentiate between axonal and demyelinating processes.**<br>**7. Serum Free Light Chains (FLC) and Urine Bence Jones Protein**<br>○ **Reasoning: To detect light chain monoclonal proteins which can be associated with multiple myeloma or other plasma cell disorders. This can help differentiate MGUS from more serious hematologic malignancies.**<br>**8. Positron Emission Tomography-Computed Tomography (PET-CT)**<br>○ **Reasoning: To rule out malignancies, especially lymphoproliferative disorders that can present with neuropathy and monoclonal gammopathy. PET-CT can identify hypermetabolic lesions that might not be evident on other imaging studies.**<br>**Most Crucial Test**<br>**Anti-MAG (Myelin-Associated Glycoprotein) Antibodies**

			**Reasoning:**<br>● The presence of IgM kappa monoclonal gammopathy and demyelinating neuropathy strongly suggests MGUS neuropathy. Anti-MAG antibodies are specifically associated with IgM paraprotein and demyelinating polyneuropathy.<br>● Identifying anti-MAG antibodies would directly support the diagnosis of MGUS neuropathy and help differentiate it from other potential causes like CIDP and diabetic neuropathy.<br>● This test is both specific and sensitive for confirming the suspected diagnosis based on the clinical presentation and existing laboratory findings.
70	A 51-year-old female patient presents with a five-month history of sensory-motor, asymmetrical, non-length-dependent polyneuropathy with subacute onset and progressive course. The patient reports numbness and tingling in a stocking and glove distribution, dysphagia, asymmetric proximal and distal weakness in all four limbs, and mild sensory ataxia. Clinical examination confirms the presence of weakness and sensory deficits in all four limbs, with no evidence of autonomic involvement, tremor, or foot deformities. Past medical history is unremarkable. Nerve conduction studies show a demyelinating, non-length-dependent, sensory-motor polyneuropathy. Screening lab tests including vitamin B12, folate, vitamin B6, vitamin E, HBV, HCV, ANA, ANCA, ENA, CRP, ESR, glycemia, HbA1c, copper, cryoglobulins, and serum electrophoresis are all negative. There is no family history of polyneuropathy.	CIDP	**Step-by-Step Analysis and Additional Examinations**<br>**Step 1: Review Clinical Presentation and Initial Findings**<br>● Patient Presentation:<br>○ 51-year-old female<br>○ 5-month history of sensory-motor, asymmetrical, non-length-dependent polyneuropathy<br>○ Numbness, tingling, dysphagia, asymmetric proximal and distal weakness, mild sensory ataxia<br>● Clinical Examination: Weakness and sensory deficits in all four limbs<br>● Nerve Conduction Studies: Demyelinating, non-length-dependent, sensory-motor polyneuropathy<br>● Initial Lab Results: Negative for vitamin deficiencies, infections (HBV, HCV), autoimmune markers (ANA, ANCA, ENA), inflammatory markers (CRP, ESR), glycemic control (glycemia, HbA1c), copper, cryoglobulins, and serum electrophoresis.<br>**Step 2: Identify Key Areas for Further Investigation**<br>1. Confirmation of Demyelinating Nature:<br>○ Reasoning: Verify the demyelinating process to solidify the diagnosis.<br>2. Assessment for Autoimmune/Paraneoplastic Causes:<br>○ Reasoning: Exclude autoimmune or paraneoplastic syndromes, which can cause similar neuropathies.<br>3. Investigation for Rare or Overlooked Conditions:<br>○ Reasoning: Rule out less common causes or those that may not have been detected initially.<br>**Additional Examinations**<br>1. Lumbar Puncture for Cerebrospinal Fluid (CSF) Analysis:<br>○ Reasoning: CSF analysis can reveal elevated protein levels with normal cell counts (albuminocytologic dissociation), which is typical in CIDP. It also helps exclude other central nervous system infections or inflammations.<br>○ Specifics: Test for protein levels, cell count, glucose, and possible oligoclonal bands.<br>2. Repeat Serum Immunofixation Electrophoresis and Serum Free Light Chain Analysis:<br>○ Reasoning: Ensure no monoclonal gammopathy has been missed. Serum immunofixation is more sensitive than standard electrophoresis. MGUS-related neuropathy should be ruled out with certainty.<br>○ Specifics: Look for monoclonal proteins or abnormal free light chains.<br>3. Antibodies Associated with Neuropathies:<br>○ Reasoning: Autoantibodies like anti-GM1 (associated with multifocal motor neuropathy) and anti-MAG (myelin-associated glycoprotein, linked with demyelinating neuropathies) can help differentiate types of neuropathies.<br>○ Specifics: Test for anti-GM1, anti-MAG, and other relevant antibodies.<br>4. MRI of the Spine and Brachial/Lumbosacral Plexus:<br>○ Reasoning: MRI can detect nerve root or plexus enhancement, which is indicative of inflammatory processes like CIDP. It also helps rule out structural causes.<br>○ Specifics: Look for gadolinium enhancement of nerve roots or plexus.<br>5. Genetic Testing for Hereditary Neuropathies:<br>○ Reasoning: Although there is no family history, some hereditary neuropathies can present sporadically or have late-onset.<br>○ Specifics: Test for mutations linked with hereditary demyelinating polyneuropathies, such as PMP22, MPZ, and others.<br>6. Skin or Nerve Biopsy:<br>○ Reasoning: A biopsy can provide direct evidence of demyelination or inflammatory infiltrates, supporting a diagnosis of CIDP or other neuropathies.<br>○ Specifics: Perform a biopsy of a sural nerve or skin to assess small fiber neuropathy and immune deposits.<br>**Conclusion: Most Crucial Test**<br>**Lumbar Puncture for Cerebrospinal Fluid (CSF) Analysis**<br>● Reasoning: CSF analysis is crucial for confirming CIDP, as it typically shows elevated protein levels with normal cell counts (albuminocytologic dissociation). This test is highly informative and directly related to diagnosing and differentiating demyelinating polyneuropathies from other conditions. It also helps rule out central nervous system infections and other inflammatory disorders.<br>In summary, while a comprehensive set of tests is necessary to narrow down the differential diagnoses, the most crucial test in this scenario is the lumbar puncture for CSF analysis due to its direct relevance to diagnosing CIDP and its ability to provide definitive evidence supporting the demyelinating process observed in nerve conduction studies.
71	A 69-year-old male patient presents with a 7-month history of sensory-motor, asymmetrical, non-length dependent polyneuropathy with subacute onset and progressive course. The patient presents with paresthesia and neuropathic pain in the four limbs, weakness of ankle dorsiflexion, and mild urinary retention. Clinical examination does not show the presence of tremor, ataxia, foot deformities or cranial nerve involvement. Past medical history is positive for type 2 diabetes. Nerve conduction studies show a demyelinating, sensory-motor, symmetric,	CIDP	**Step-by-Step Analysis and Additional Examinations**<br>**Step 1: Review Clinical Data**<br>● Symptoms: Paresthesia, neuropathic pain in four limbs, ankle dorsiflexion weakness, mild urinary retention<br>● Clinical Examination: No tremor, ataxia, foot deformities, or cranial nerve involvement<br>● Nerve Conduction Studies: Demyelinating, sensory-motor, symmetric polyneuropathy<br>● History: Type 2 diabetes, high glycemia, and HbA1c levels<br>● Negative Tests: Vitamin B12, folate, vitamin B6, vitamin E, HBV, HCV, ANA, ANCA, ENA, CRP, ESR, copper, cryoglobulins, serum electrophoresis<br>● Family History: No family history of polyneuropathy<br>**Step 2: Additional Examinations**<br>1. Lumbar Puncture (CSF Analysis)<br>○ Reasoning: A cerebrospinal fluid (CSF) analysis can help differentiate between inflammatory and non-inflammatory causes of neuropathy. Elevated protein levels with normal cell count (albuminocytologic dissociation) are characteristic of CIDP.<br>2. MRI of the Spine<br>○ Reasoning: MRI can detect structural causes of neuropathy, such as nerve root compression, spinal cord lesions, or radiculopathy, which may mimic polyneuropathy symptoms.

	polyneuropathy. Screening lab tests including vitamin B12, folate, vitamin B6, vitamin E, HBV, HCV, ANA, ANCA, ENA, CRP, ESR, copper, cryoglobulins and serum electrophoresis are all negative, while glycemia and HbA1c levels are high. There is no family history of polyneuropathy.		3. **Autoimmune Panel (Extended)**<br>○ **Reasoning: Although initial autoimmune markers (ANA, ANCA, ENA) were negative, an extended autoimmune panel including anti-GM1, anti-MAG, and anti-sulfatide antibodies can be useful in identifying atypical autoimmune neuropathies.**<br>4. **Paraneoplastic Antibody Panel**<br>○ **Reasoning: Screening for paraneoplastic antibodies (e.g., anti-Hu, anti-Yo, anti-Ri) can help identify a paraneoplastic syndrome, especially in the absence of other identifiable causes and given the progressive nature of symptoms.**<br>5. **Nerve Biopsy**<br>○ **Reasoning: In cases where the diagnosis remains unclear, a nerve biopsy can provide definitive histopathological evidence of the type of neuropathy, distinguishing between demyelinating and axonal types, and can also identify specific causes like vasculitis.**<br>6. **Electromyography (EMG)**<br>○ **Reasoning: EMG can complement nerve conduction studies by evaluating the electrical activity of muscles, further characterizing the nature and distribution of the neuropathy.**<br>7. **Glucose Tolerance Test**<br>○ **Reasoning: While the patient already has diabetes, a glucose tolerance test can provide more detailed information about glucose metabolism, potentially identifying glucose fluctuations that could contribute to neuropathy.**<br>**Step 3: Most Crucial Test**<br>**Based on the current clinical scenario and differential diagnoses, the most crucial test is the Lumbar Puncture (CSF Analysis).**<br>**Conclusion**<br>**Lumbar Puncture (CSF Analysis) is the most critical test for this patient. It provides essential information that can confirm or rule out CIDP by demonstrating elevated CSF protein levels with normal cell count. This information, combined with the clinical presentation and nerve conduction study findings, will significantly narrow down the differential diagnosis and guide further management.**
72	A 58-year old male patient presents with a 1-year history of sensory-motor, symmetrical, non-length dependent polyneuropathy with subacute onset. The patient presents with paresthesia and weakness in the distal areas of the four limbs. Clinical examination does not show the presence of sensory ataxia, neuropathic pain, tremor, foot deformities, cranial nerve or autonomic involvement. Clinical examination shows cutaneous hemangiomas and hyperpigmentation. Past medical history is positive for IgA lambda monoclonal gammopathy. Nerve conduction studies show mixed demyelinating-axonal, sensory-motor, symmetric polyneuropathy. Screening lab tests including vitamin B12, folate, vitamin B6, vitamin E, HCV, HBV, ANA, ANCA, ENA, CRP, ESR, glycemia, HbA1c, copper and cryoglobulins are all negative, with positivity for IgA lambda paraprotein. There is no family history of polyneuropathy.	POEMS	**Step-by-Step Analysis and Additional Examinations**<br>**Step 1: Review of Clinical Presentation**<br>● **Symptoms: Sensory-motor, symmetrical, non-length dependent polyneuropathy with paresthesia and weakness in distal limbs.**<br>● **Duration: 1 year with subacute onset.**<br>● **Clinical Findings: Cutaneous hemangiomas, hyperpigmentation, no sensory ataxia, neuropathic pain, tremor, foot deformities, cranial nerve or autonomic involvement.**<br>● **Past Medical History: IgA lambda monoclonal gammopathy.**<br>● **Nerve Conduction Studies: Mixed demyelinating-axonal, sensory-motor, symmetric polyneuropathy.**<br>● **Screening Lab Tests: Extensive negative results, positive for IgA lambda paraprotein.**<br>**Step 2: Formulate Additional Tests**<br>1. **Serum Immunofixation Electrophoresis (IFE) and Serum Free Light Chains (FLC) Assay**<br>○ **Reasoning: To further characterize the monoclonal protein and quantify light chains, helping to distinguish between MGUS, smoldering myeloma, or multiple myeloma.**<br>2. **Bone Marrow Biopsy**<br>○ **Reasoning: To assess for plasma cell dyscrasia or other hematological malignancies, especially given the presence of a monoclonal gammopathy.**<br>3. **CSF Analysis**<br>○ **Reasoning: To check for elevated protein levels and oligoclonal bands, which can support a diagnosis of CIDP or other inflammatory neuropathies.**<br>4. **Anti-MAG Antibodies Testing**<br>○ **Reasoning: Anti-MAG (myelin-associated glycoprotein) antibodies are associated with IgM paraproteinemic neuropathy, which can present similarly to MGUS neuropathy.**<br>5. **Nerve Biopsy**<br>○ **Reasoning: To provide definitive evidence of the type of neuropathy, helping to distinguish between demyelinating and axonal damage and to identify inflammatory or amyloid deposits.**<br>6. **Imaging Studies (MRI of the Spine and Brain)**<br>○ **Reasoning: To rule out structural causes of neuropathy and to check for any CNS involvement which might suggest an alternate diagnosis like multiple sclerosis or CIDP.**<br>7. **Quantitative Sensory Testing (QST)**<br>○ **Reasoning: To provide objective measurement of sensory function and help differentiate between large and small fiber neuropathy.**<br>8. **Autonomic Function Tests**<br>○ **Reasoning: Despite the lack of autonomic symptoms, these tests can provide comprehensive evaluation, as subtle autonomic dysfunction can sometimes accompany neuropathies.**<br>**Step 3: Determine the Most Crucial Test**<br>**Bone Marrow Biopsy is the most crucial test. Given the presence of IgA lambda monoclonal gammopathy, it is essential to rule out or confirm a plasma cell dyscrasia, which can significantly impact management and prognosis. Bone marrow biopsy can differentiate between MGUS, smoldering multiple myeloma, or multiple myeloma, and provide insights into the extent of any underlying hematologic condition that might be contributing to the neuropathy.**<br>**Summary of Additional Tests and Their Importance**<br>1. **Serum Immunofixation Electrophoresis and FLC Assay**<br>○ **Detailed characterization and quantification of the monoclonal protein.**<br>2. **Bone Marrow Biopsy**<br>○ **Assessment for plasma cell dyscrasia (most crucial test).**<br>3. **CSF Analysis**<br>○ **Identification of inflammatory markers supporting CIDP or other neuropathies.**<br>4. **Anti-MAG Antibodies Testing**<br>○ **Detection of specific antibodies linked to paraproteinemic neuropathy.**<br>5. **Nerve Biopsy**<br>○ **Direct examination of nerve tissue for definitive diagnosis.**<br>6. **Imaging Studies**<br>○ **Exclusion of structural CNS causes.**<br>7. **Quantitative Sensory Testing**<br>○ **Objective measurement of sensory function.**<br>8. **Autonomic Function Tests**

			○ **Comprehensive evaluation of potential autonomic involvement.**<br>**By conducting these additional tests, a more precise diagnosis can be established, guiding appropriate treatment and management for the patient's polyneuropathy.**
73	An 87-year-old male patient presents with a seven-month history of sensory-motor, symmetrical, length-dependent polyneuropathy with subacute onset and progressive course. The patient reports paresthesia and hypoesthesia in the lower limbs, associated with sensory ataxia and mild weakness in ankle dorsiflexion. Clinical examination does not reveal tremor, foot deformities, cranial nerve involvement, or autonomic dysfunction. Past medical history is positive for IgG kappa monoclonal gammopathy and prostate cancer. Nerve conduction studies show a demyelinating, sensory-motor, symmetric polyneuropathy. Screening lab tests including vitamin B12, folate, vitamin B6, vitamin E, HBV, HCV, ANA, ANCA, ENA, CRP, ESR, glycemia, HbA1c, copper, and cryoglobulins are all negative, with positivity for IgG kappa paraprotein. There is no family history of polyneuropathy.	CIDP	**Step-by-Step Analysis and Additional Examinations**<br>**Step 1: Review of Case Details**<br>● **Symptoms and Signs:**<br>○ **Sensory-motor, symmetrical, length-dependent polyneuropathy**<br>○ **Subacute onset, progressive course**<br>○ **Paresthesia, hypoesthesia, sensory ataxia, mild weakness in ankle dorsiflexion**<br>○ **No tremor, foot deformities, cranial nerve involvement, or autonomic dysfunction**<br>● **Past Medical History:**<br>○ **IgG kappa monoclonal gammopathy**<br>○ **Prostate cancer**<br>● **Nerve Conduction Studies:**<br>○ **Demyelinating, sensory-motor, symmetric polyneuropathy**<br>● **Laboratory Tests:**<br>○ **Negative: Vitamin B12, folate, vitamin B6, vitamin E, HBV, HCV, ANA, ANCA, ENA, CRP, ESR, glycemia, HbA1c, copper, cryoglobulins**<br>○ **Positive: IgG kappa paraprotein**<br>**Step 2: List of Necessary Additional Examinations**<br>1. **Serum Protein Electrophoresis (SPEP) and Immunofixation Electrophoresis (IFE):**<br>○ **Reasoning: To confirm the presence and type of monoclonal protein (IgG kappa) and to assess the quantity of the paraprotein.**<br>○ **Details: These tests help in confirming MGUS or progressing towards multiple myeloma or other plasma cell dyscrasias.**<br>2. **Bone Marrow Biopsy:**<br>○ **Reasoning: Given the history of IgG kappa monoclonal gammopathy, this test will help evaluate the extent of plasma cell infiltration, which is crucial for diagnosing multiple myeloma or other related conditions.**<br>○ **Details: Important to differentiate between MGUS and more serious hematologic malignancies.**<br>3. **Anti-MAG (Myelin-Associated Glycoprotein) Antibody Test:**<br>○ **Reasoning: Anti-MAG antibodies are often present in patients with demyelinating neuropathies associated with monoclonal gammopathies.**<br>○ **Details: Helps in diagnosing specific types of neuropathy related to monoclonal gammopathies.**<br>4. **Cerebrospinal Fluid (CSF) Analysis:**<br>○ **Reasoning: CSF protein elevation can support the diagnosis of CIDP.**<br>○ **Details: Includes CSF protein, cell count, and oligoclonal bands.**<br>5. **Paraneoplastic Panel (including anti-Hu, anti-Yo, anti-Ri, etc.):**<br>○ **Reasoning: To rule out paraneoplastic syndromes, especially given the patient's history of prostate cancer.**<br>○ **Details: Helps identify any underlying autoimmune response related to malignancy.**<br>6. **Skin Biopsy for Intraepidermal Nerve Fiber Density:**<br>○ **Reasoning: Useful for diagnosing small fiber neuropathies which might be missed by nerve conduction studies.**<br>○ **Details: Can provide information on the involvement of small fibers, complementing nerve conduction study findings.**<br>7. **Quantitative Sensory Testing (QST):**<br>○ **Reasoning: To objectively assess sensory thresholds and confirm the extent of sensory involvement.**<br>○ **Details: Provides detailed information about sensory deficits.**<br>8. **MRI of the Spine:**<br>○ **Reasoning: To rule out compressive causes of polyneuropathy, especially since the patient has a history of cancer.**<br>○ **Details: Helps exclude structural lesions causing neuropathic symptoms.**<br>**Step 3: Determining the Most Crucial Test**<br>**Among the tests listed, the Serum Protein Electrophoresis (SPEP) and Immunofixation Electrophoresis (IFE) are the most crucial. These tests will confirm the presence and type of monoclonal protein, which is essential in diagnosing and differentiating between MGUS, multiple myeloma, and other plasma cell dyscrasias. Confirming the monoclonal protein type and quantity will direct further diagnostic steps and management plans.**
74	A 51-year old female patient presents with a 35-year history of chronic, sensory-motor, symmetrical, length dependent polyneuropathy with insidious onset and slow progression. The patient presents with paresthesia, tremor, hypoesthesia up to the calves, bilateral steppage, and hammertoes. Clinical examination does not reveal cranial nerve or autonomic involvement. Past medical history shows chronic renal insufficiency. Nerve conduction studies show an axonal, symmetric, sensory-motor polyneuropathy. Screening lab tests including vitamin B12, folate, vitamin B6, vitamin E, HBV, HCV, glycemia, HbA1c, ANA, ANCA, ENA, CRP, ESR, copper, cryoglobulins, and serum	CMT2	**Step-by-Step Analysis for Additional Examinations**<br>**Step 1: List Necessary Additional Examinations**<br>1. **Genetic Testing for Hereditary Neuropathies**<br>2. **Renal Function Tests (including eGFR)**<br>3. **Skin or Nerve Biopsy**<br>4. **Electromyography (EMG)**<br>5. **Lumbar Puncture (CSF Analysis)**<br>6. **Imaging Studies (MRI of Spine)**<br>7. **Repeat Nerve Conduction Studies with Emphasis on Sensory and Motor Distribution**<br>**Step 2: Detailed Reasoning for Each Proposed Test**<br>1. **Genetic Testing for Hereditary Neuropathies**<br>○ **Reasoning:**<br>■ **Given the strong family history and clinical presentation suggestive of Charcot-Marie-Tooth (CMT) disease, genetic testing can identify specific mutations associated with hereditary neuropathies.**<br>■ **Common genetic tests for CMT include PMP22, MPZ, GJB1, and MFN2 mutations.**<br>2. **Renal Function Tests (including eGFR)**<br>○ **Reasoning:**<br>■ **Chronic renal insufficiency can lead to uremic neuropathy, which might contribute to or exacerbate the polyneuropathy.**<br>■ **Assessing the current state of renal function can help rule out or confirm uremic neuropathy as a contributing factor.**

	electrophoresis are all negative. Her father and two uncles have walking difficulties.		■ **Tests include serum creatinine, blood urea nitrogen (BUN), and estimated glomerular filtration rate (eGFR).**<br>**3. Skin or Nerve Biopsy**<br>○ **Reasoning:**<br>■ **A nerve biopsy can provide detailed pathological information, such as the presence of axonal degeneration or demyelination, helping to differentiate between various types of neuropathies.**<br>■ **It can also help identify specific hereditary neuropathies or other rare causes.**<br>**4. Electromyography (EMG)**<br>○ **Reasoning:**<br>■ **EMG can complement nerve conduction studies by assessing the electrical activity of muscles, helping to differentiate between neuropathic and myopathic conditions.**<br>■ **It provides additional information on the extent and distribution of motor involvement.**<br>**5. Lumbar Puncture (CSF Analysis)**<br>○ **Reasoning:**<br>■ **Analyzing cerebrospinal fluid (CSF) can help rule out inflammatory, infectious, or paraneoplastic causes of polyneuropathy.**<br>■ **Useful in cases where an immune-mediated process is suspected.**<br>**6. Imaging Studies (MRI of Spine)**<br>○ **Reasoning:**<br>■ **MRI can identify structural abnormalities in the spine that might contribute to neuropathy, such as nerve root compression or spinal stenosis.**<br>■ **Although less likely given the symmetrical nature of the neuropathy, it helps rule out any contributing factors from the spinal column.**<br>**7. Repeat Nerve Conduction Studies with Emphasis on Sensory and Motor Distribution**<br>○ **Reasoning:**<br>■ **Repeating and expanding the nerve conduction studies to include more nerves and a detailed analysis of sensory and motor responses can provide a comprehensive overview of the neuropathy's characteristics.**<br>■ **Helps in detecting any progression or changes over time.**<br>**Step 3: Determine the Most Crucial Test**<br>**Most Crucial Test:**<br>● **Genetic Testing for Hereditary Neuropathies**<br>○ **Reasoning:**<br>■ **Given the strong family history of similar symptoms and the clinical presentation consistent with Charcot-Marie-Tooth disease, genetic testing is the most crucial examination. It can provide a definitive diagnosis by identifying specific genetic mutations associated with hereditary neuropathies. This will confirm the diagnosis and allow for appropriate management and genetic counseling for the patient and her family.**<br>**Conclusion**<br>**The most crucial test from the list is Genetic Testing for Hereditary Neuropathies. This test will provide definitive information on the presence of hereditary neuropathies like Charcot-Marie-Tooth disease, which is the leading suspected diagnosis based on the patient's clinical presentation and family history.**
75	An 81-year-old male patient presents with a 13-month history of sensory-motor, symmetrical, non-length dependent polyneuropathy with subacute onset and progressive course. The patient presents with lumbar pain associated with paresthesia, neuropathic pain, and weakness that are distributed both proximally and distally in the four limbs. Clinical examination also reveals the presence of mild sensory ataxia. There are no tremor, cramps, foot deformities, cranial nerve or autonomic involvement. Past medical history is positive for type 2 diabetes and Hashimoto thyroiditis. Nerve conduction studies show a mixed demyelinating-axonal, sensory-motor polyneuropathy. Screening lab tests show the presence of IgG lambda monoclonal gammopathy and raised HbA1c levels. Remaining exams including vitamin B12, folate, vitamin B6, vitamin E, HCV, HBV, ANA, ANCA, ENA, CRP, ESR, copper and cryoglobulins are all negative. Lumbosacral spine x-ray shows the presence of a single osteosclerotic bone lesion. There is no family history of polyneuropathy.	POEMS	**Step-by-Step Analysis and Additional Examinations**<br>**Step 1: Review Current Data**<br>● **Patient Details: 81-year-old male**<br>● **History: 13-month sensory-motor, symmetrical, non-length dependent polyneuropathy; subacute onset; progressive course; lumbar pain; paresthesia; neuropathic pain; weakness in all four limbs; mild sensory ataxia.**<br>● **Clinical Findings: No tremor, cramps, foot deformities, cranial nerve or autonomic involvement.**<br>● **Medical History: Type 2 diabetes, Hashimoto thyroiditis.**<br>● **Nerve Conduction Studies: Mixed demyelinating-axonal, sensory-motor polyneuropathy.**<br>● **Lab Findings: IgG lambda monoclonal gammopathy, elevated HbA1c. Negative for vitamin deficiencies, viral infections, autoimmune markers, inflammation markers, other potential causes.**<br>● **Imaging: Lumbosacral spine x-ray shows a single osteosclerotic bone lesion.**<br>● **Family History: No polyneuropathy.**<br>**Step 2: Identify Gaps in Information**<br>● **Further evaluation of monoclonal gammopathy.**<br>● **Assessment for possible underlying malignancy or POEMS syndrome.**<br>● **Exclusion of other potential causes of neuropathy not yet ruled out by current tests.**<br>**Step 3: Propose Additional Examinations**<br>**1. Serum Free Light Chain Assay and Serum Immunofixation Electrophoresis (IFE)**<br>○ **Reasoning: To further characterize the monoclonal gammopathy. Serum free light chain assay helps in quantifying light chains and detecting light chain abnormalities. IFE provides detailed information on the type and amount of monoclonal protein.**<br>○ **Details: Necessary to determine if the gammopathy is part of a more significant hematologic disorder like multiple myeloma or MGUS.**<br>**2. Bone Marrow Biopsy and Aspirate**<br>○ **Reasoning: To assess for clonal plasma cells or other hematologic malignancies. Given the monoclonal gammopathy and osteosclerotic lesion, this is crucial for diagnosing conditions like multiple myeloma or POEMS syndrome.**<br>○ **Details: Helps confirm or rule out malignancies that may be contributing to the neuropathy.**<br>**3. Whole-Body Imaging (PET-CT or MRI)**<br>○ **Reasoning: To identify any other osteosclerotic lesions, organomegaly, or other signs of systemic involvement indicative of POEMS syndrome or other malignancies.**<br>○ **Details: Comprehensive imaging can detect other areas of involvement not seen on a single lumbosacral x-ray.**<br>**4. Cerebrospinal Fluid (CSF) Analysis**<br>○ **Reasoning: To evaluate for inflammatory or infectious causes of neuropathy that might not have been detected by blood tests. CSF analysis can detect abnormal cells, protein levels, and infections.**<br>○ **Details: Especially important if there is suspicion of conditions like chronic inflammatory demyelinating polyneuropathy (CIDP) or paraneoplastic syndromes.**<br>**5. Anti-MAG (Myelin-Associated Glycoprotein) Antibody Testing**<br>○ **Reasoning: To evaluate for specific antibodies associated with demyelinating neuropathies. Anti-MAG antibodies are often seen in patients with monoclonal gammopathies.**

			○ Details: Can help differentiate between different types of neuropathies associated with monoclonal gammopathies.<br>6. Endocrine Evaluation<br>○ Reasoning: To check for endocrinopathies as part of POEMS syndrome. This includes thyroid function tests (beyond those for Hashimoto's thyroiditis), adrenal function tests, and glucose tolerance tests.<br>○ Details: Endocrine disorders can contribute to neuropathy and need to be assessed comprehensively.<br>7. Nerve Biopsy<br>○ Reasoning: To provide histological confirmation of the type of neuropathy (demyelinating vs. axonal) and to look for specific pathological features.<br>○ Details: Helps in cases where non-invasive tests are inconclusive.<br>**Step 4: Determine the Most Crucial Test**<br>**Bone Marrow Biopsy and Aspirate**<br>● Reasoning: Given the monoclonal gammopathy and osteosclerotic lesion, this test is pivotal in diagnosing or excluding hematologic malignancies such as multiple myeloma or POEMS syndrome. It provides direct evidence of clonal plasma cells, which are critical in confirming these diagnoses.<br>**Conclusion**<br>The bone marrow biopsy and aspirate are the most crucial tests in this scenario. This examination will yield definitive information about the presence of hematologic malignancies, helping to narrow down the differential diagnoses significantly and guiding further management and treatment.
76	A 51-year-old female patient presents with a 6-week history of sensory, symmetrical, length dependent polyneuropathy with acute-subacute onset. The patient presents with paresthesia and hypoesthesia with a stocking-glove distribution and sensory ataxia. Clinical examination reveals presence of severe ataxia with inability to stand without support. There is no weakness or neuropathic pain, tremor, foot deformities, cranial nerve or autonomic involvement. Past medical history is positive for type 1 diabetes since the age of 23. Nerve conduction studies show presence of a pure sensory, length dependent, axonal polyneuropathy. Screening lab tests reveal hyperglycemia, elevated HbA1c levels and severe copper deficiency. Remaining exams including vitamin B12, folate, vitamin B6, vitamin E, HBV, HCV, ANA, ANCA, ENA, CRP, ESR, cryoglobulins, and serum electrophoresis are all negative. There is no family history of polyneuropathy.	Copper deficiency neuropathy	**Step-by-Step Analysis for Additional Examinations**<br>**Patient Summary (for reference)**<br>● Age/Sex: 51-year-old female<br>● History: Type 1 diabetes mellitus since age 23<br>● Presentation: 6-week history of sensory, symmetrical, length-dependent polyneuropathy<br>● Symptoms: Paresthesia, hypoesthesia, stocking-glove distribution, sensory ataxia, severe ataxia (unable to stand without support)<br>● Clinical Findings:<br>○ Severe ataxia<br>○ No weakness, neuropathic pain, tremor, foot deformities<br>○ No cranial nerve or autonomic involvement<br>● Nerve Conduction Studies: Pure sensory, length-dependent, axonal polyneuropathy<br>● Lab Findings:<br>○ Hyperglycemia<br>○ Elevated HbA1c levels<br>○ Severe copper deficiency<br>○ Negative for vitamin B12, folate, vitamin B6, vitamin E, HBV, HCV, ANA, ANCA, ENA, CRP, ESR, cryoglobulins, and serum electrophoresis<br>● Family History: Negative for polyneuropathy<br>**Additional Examinations**<br>1. Serum and Urine Copper and Ceruloplasmin Levels<br>○ Reasoning:<br>■ Confirm the extent of copper deficiency and investigate possible underlying causes such as malabsorption or genetic disorders (e.g., Menkes disease).<br>■ Low serum copper and ceruloplasmin levels support copper deficiency neuropathy.<br>○ Expected Outcome: These tests will help confirm and quantify the copper deficiency.<br>2. MRI of the Brain and Spinal Cord<br>○ Reasoning:<br>■ Evaluate for potential myelopathy which can accompany copper deficiency.<br>■ Identify any other neurological abnormalities or lesions that might explain the severe ataxia.<br>○ Expected Outcome: Findings may reveal spinal cord degeneration or other structural abnormalities consistent with copper deficiency myelopathy.<br>3. Upper and Lower Gastrointestinal Endoscopy with Biopsies<br>○ Reasoning:<br>■ Assess for gastrointestinal causes of malabsorption which can lead to copper deficiency (e.g., celiac disease, Crohn's disease).<br>■ Biopsies can provide histological evidence of malabsorptive disorders.<br>○ Expected Outcome: Detection of any underlying malabsorptive pathology contributing to copper deficiency.<br>4. Genetic Testing for ATP7A and ATP7B Mutations<br>○ Reasoning:<br>■ Evaluate for genetic causes of copper metabolism disorders (e.g., Wilson's disease, Menkes disease).<br>■ Relevant for understanding the etiology of severe copper deficiency in the absence of clear secondary causes.<br>○ Expected Outcome: Identification of mutations would confirm a hereditary copper metabolism disorder.<br>5. Repeat Nerve Conduction Studies and Electromyography (EMG)<br>○ Reasoning:<br>■ Confirm the initial findings and evaluate for any progression or changes in the pattern of neuropathy.<br>■ EMG can provide additional information on muscle involvement and help differentiate between axonal and demyelinating processes.<br>○ Expected Outcome: Corroborate the diagnosis of pure sensory axonal neuropathy.<br>6. Vitamin A and Zinc Levels<br>○ Reasoning:<br>■ Zinc excess can interfere with copper absorption and lead to deficiency.<br>■ Vitamin A levels can be affected in malabsorptive states.<br>○ Expected Outcome: Elevated zinc or low vitamin A levels could suggest malabsorption or excessive supplementation interfering with copper metabolism.<br>**Most Crucial Test**

			**Serum and Urine Copper and Ceruloplasmin Levels**<br>**Reasoning:**<br>● **Confirming the diagnosis of copper deficiency neuropathy is paramount since this aligns with the clinical presentation and the initial finding of severe copper deficiency.**<br>● **Establishing the degree of deficiency will guide further management and supplementation.**<br>● **These tests are critical for diagnosing copper deficiency and distinguishing it from other causes of neuropathy, thereby directing appropriate treatment and identifying the need for further investigation into underlying causes.**<br>**Conclusion**<br>**The most crucial test from the list is Serum and Urine Copper and Ceruloplasmin Levels. These tests are essential to confirm the diagnosis of copper deficiency neuropathy, quantify the deficiency, and guide further management and investigation into the underlying cause of the deficiency.**
77	A 70-year-old female patient presents with a 1-month history of sensory-motor, asymmetrical, non-length dependent, step-wise multineuropathy with acute onset. The patient presents with paresthesia, hypoesthesia, neuropathic pain and weakness in a multifocal distribution. Clinical examination does not reveal presence of ataxia, tremor, foot deformities, cranial nerve or autonomic involvement. Past medical history reveals the presence of intermittent fever and malaise. Nerve conduction studies show a mixed sensory-motor, axonal multineuropathy and unilateral carpal tunnel syndrome. Screening lab tests reveal high levels of CRP and ESR. Remaining lab exams including vitamin B12, folate, vitamin B6, vitamin E, HCV, HBV, ANA, ENA, glycemia, HbA1c, copper, cryoglobulins and serum electrophoresis are normal. There is no family history of polyneuropathy.	Vasculitic neuropathy	**Step-by-Step Analysis**<br>**Step 1: Comprehensive Data Analysis**<br>● **Patient Demographics: 70-year-old female**<br>● **History:**<br>○ **1-month history of sensory-motor, asymmetrical, non-length dependent, step-wise multineuropathy with acute onset**<br>○ **Symptoms: Paresthesia, hypoesthesia, neuropathic pain, weakness in a multifocal distribution**<br>○ **Intermittent fever and malaise**<br>● **Clinical Findings: No ataxia, tremor, foot deformities, cranial nerve, or autonomic involvement**<br>● **Nerve Conduction Studies: Mixed sensory-motor, axonal multineuropathy and unilateral carpal tunnel syndrome**<br>● **Lab Findings: Elevated CRP and ESR; normal levels of vitamin B12, folate, vitamin B6, vitamin E, HCV, HBV, ANA, ENA, glycemia, HbA1c, copper, cryoglobulins, and serum electrophoresis**<br>● **Family History: No family history of polyneuropathy**<br>**Step 2: Additional Examinations Needed**<br>1. **ANCA (Antineutrophil Cytoplasmic Antibodies) Testing**<br>○ **Reasoning: To evaluate for vasculitic neuropathy, as ANCA is often positive in systemic vasculitides like granulomatosis with polyangiitis or microscopic polyangiitis. This test can help confirm the presence of a vasculitic process.**<br>2. **Nerve and Muscle Biopsy**<br>○ **Reasoning: A biopsy can provide direct evidence of vasculitis by showing inflammation of the blood vessels. It is crucial for definitive diagnosis of vasculitic neuropathy.**<br>3. **CSF Analysis (Lumbar Puncture)**<br>○ **Reasoning: To rule out inflammatory or infectious causes affecting the central nervous system. Elevated protein levels with or without pleocytosis can indicate an inflammatory neuropathy or an infectious etiology.**<br>4. **Cancer Screening (CT Scan of Chest, Abdomen, Pelvis)**<br>○ **Reasoning: To investigate for potential paraneoplastic syndromes. Given the patient's age and systemic symptoms, screening for hidden malignancies is essential.**<br>5. **Specific Infectious Disease Tests (e.g., Lyme Disease Serology)**<br>○ **Reasoning: To rule out infectious etiologies not covered in the initial screening. Lyme disease, for example, can present with multifocal neuropathy.**<br>6. **Rheumatoid Factor (RF) and Anti-CCP Antibodies**<br>○ **Reasoning: To check for rheumatoid arthritis, which can be associated with vasculitis and secondary neuropathy.**<br>7. **PET Scan**<br>○ **Reasoning: To detect occult malignancies and evaluate for systemic inflammatory processes. This can be useful if initial cancer screenings are inconclusive.**<br>8. **Anti-Ganglioside Antibodies**<br>○ **Reasoning: To evaluate for immune-mediated neuropathies such as Multifocal Motor Neuropathy (MMN) or Chronic Inflammatory Demyelinating Polyneuropathy (CIDP), which can present with multifocal neuropathy.**<br>**Step 3: Determining the Most Crucial Test**<br>**Nerve and Muscle Biopsy**<br>● **Reasoning: This test is pivotal because it provides direct histological evidence of vasculitis or other pathological processes affecting the nerves and muscles. While ANCA testing can support a diagnosis of vasculitic neuropathy, a biopsy can confirm it by showing characteristic inflammatory changes in the blood vessels. This is especially important given the acute, multifocal nature of the neuropathy, elevated inflammatory markers, and the need to definitively diagnose or rule out vasculitis as the underlying cause.**<br>**Conclusion**<br>**The most crucial test from the list is the nerve and muscle biopsy. It provides definitive diagnostic information regarding vasculitic neuropathy, which is highly suspected based on the patient's clinical presentation, nerve conduction study results, and elevated inflammatory markers.**
78	A 48-year-old male patient presents with a 4-month history of sensory-motor, asymmetric, length dependent polyneuropathy with subacute onset and rapidly progressive course. The patient presents with paresthesia, hypoesthesia, and weakness in the distal segments of the four limbs in an asymmetric distribution. Clinical evaluation reveals peripheral edema in the lower limbs. The patient reports recent appearance of several skin growths that were diagnosed as cutaneous angiomas by the dermatologist. He also reports losing approximately 10 kg over the past 12 months. There are no tremor, ataxia, neuropathic pain, foot deformities, cranial nerve or autonomic involvement.	POEMS	**Step-by-Step Analysis**<br>**Step 1: Review the Clinical Scenario**<br>**Patient Summary:**<br>● **48-year-old male**<br>● **4-month history of rapidly progressive sensory-motor, asymmetric, length-dependent polyneuropathy**<br>● **Clinical findings: Paresthesia, hypoesthesia, weakness in distal limbs, peripheral edema, cutaneous angiomas, significant weight loss**<br>● **Past medical history: Fibromyalgia, liver steatosis**<br>● **Investigations: Mixed demyelinating-axonal, sensory-motor polyneuropathy, positive IgA lambda paraprotein, vitamin B12 deficiency, negative screening lab tests**<br>**Step 2: Identify Differential Diagnoses**<br>1. **POEMS Syndrome**<br>2. **MGUS-related neuropathy**<br>3. **Vitamin B12 Deficiency Neuropathy**<br>**Step 3: Determine Additional Examinations**<br>**Objective: Confirm the diagnosis and rule out other potential causes.**<br>1. **Bone Marrow Biopsy and Aspiration**<br>○ **Reasoning: To evaluate for plasma cell dyscrasias, including multiple myeloma, POEMS syndrome, and other hematologic malignancies.**<br>○ **Details: Assessing for monoclonal plasma cells, amyloidosis, or other hematologic abnormalities.**

	Past medical history is positive for fibromyalgia and liver steatosis. Nerve conduction studies show a mixed demyelinating-axonal, sensory-motor, symmetric polyneuropathy. Screening lab tests including folate, vitamin B6, vitamin E, HCV, HBV, ANA, ANCA, ENA, CRP, ESR, glycemia, HbA1c, copper and cryoglobulins are negative. Serum electrophoresis shows positivity for IgA lambda paraprotein, and there is evidence of vitamin B12 deficiency. There is no family history of polyneuropathy.		2. Serum Vascular Endothelial Growth Factor (VEGF)<br>○ Reasoning: VEGF is often elevated in POEMS syndrome and is part of the diagnostic criteria.<br>○ Details: Measuring VEGF levels can support the diagnosis of POEMS.<br>3. Imaging Studies: Whole-Body PET-CT or MRI<br>○ Reasoning: To detect organomegaly, sclerotic bone lesions, or other signs consistent with POEMS syndrome or malignancies.<br>○ Details: Identifying characteristic findings such as bone lesions or organ involvement.<br>4. Electromyography (EMG)<br>○ Reasoning: To further characterize the extent and type of neuropathy.<br>○ Details: Differentiating between axonal and demyelinating processes and confirming the distribution.<br>5. Complete Blood Count (CBC) with Differential<br>○ Reasoning: To identify any hematologic abnormalities such as anemia or thrombocytosis, which can be associated with POEMS syndrome.<br>○ Details: Assessing for blood dyscrasias or cytopenias.<br>6. Serum Immunofixation Electrophoresis (IFE)<br>○ Reasoning: To further characterize the monoclonal protein and assess for additional paraproteins.<br>○ Details: Identifying the specific type of paraprotein and its clonality.<br>7. 24-Hour Urine Protein Electrophoresis<br>○ Reasoning: To detect Bence Jones proteins or other monoclonal proteins in urine, which are indicative of plasma cell disorders.<br>○ Details: Complementing serum studies to evaluate for multiple myeloma or related conditions.<br>8. Skin Biopsy (if clinically indicated)<br>○ Reasoning: To histologically examine the cutaneous angiomas and rule out other dermatologic conditions.<br>○ Details: Confirming the nature of skin growths and assessing for specific findings related to POEMS.<br>Step 4: Determine the Most Crucial Test<br>Most Crucial Test: Bone Marrow Biopsy and Aspiration<br>● Reasoning: This test is pivotal for diagnosing plasma cell disorders, including POEMS syndrome and multiple myeloma. It provides definitive information on the presence of monoclonal plasma cells, amyloidosis, and other hematologic abnormalities. Given the positive IgA lambda paraprotein and the constellation of symptoms, this test will significantly narrow down the diagnosis and guide subsequent management.<br>Conclusion<br>The comprehensive analysis of the clinical scenario highlights the need for targeted additional examinations to confirm the diagnosis. The bone marrow biopsy and aspiration are the most crucial tests, providing essential diagnostic information for confirming POEMS syndrome or other plasma cell disorders.
79	A 55-year-old male patient presents with a 1-year history of a pure sensory, length dependent polyneuropathy with insidious onset and slow progressive course. The patient presents with paresthesia and neuropathic pain in feet. He also reports cramps. Clinical examination does not reveal ataxia, tremor, foot deformities, cranial nerve or autonomic involvement. Past medical history is positive for IgG MGUS and Hashimoto's thyroiditis. Nerve conduction studies show an axonal, sensory-motor, symmetric, length dependent polyneuropathy. Screening lab tests including vitamin B12, folate, vitamin B6, vitamin E, HBV, HCV, glycemia, HbA1c, ANA, ANCA, ENA, CRP, ESR, and copper are all negative, with positivity for IgG monoclonal gammopathy. There is no family history of polyneuropathy.	MGUS-associated neuropathy	Step-by-Step Analysis<br>1. Review Initial Findings:<br>○ The patient presents with a sensory, length-dependent polyneuropathy.<br>○ Nerve conduction studies show an axonal, sensory-motor polyneuropathy.<br>○ Initial lab tests (vitamin B12, folate, vitamin B6, vitamin E, HBV, HCV, glycemia, HbA1c, ANA, ANCA, ENA, CRP, ESR, copper) are negative except for IgG monoclonal gammopathy.<br>○ Medical history includes IgG MGUS and Hashimoto's thyroiditis.<br>2. Determine Additional Examinations: Based on the provided information, additional tests are necessary to further narrow down the differential diagnoses, particularly focusing on MGUS-related neuropathy, CIDP, and potential hidden diabetes or other causes.<br>Additional Examinations<br>Laboratory Tests<br>1. Serum Immunofixation Electrophoresis and Free Light Chains<br>○ Reasoning: To characterize the monoclonal protein further and to rule out conditions like multiple myeloma or other plasma cell dyscrasias that could cause neuropathy. This will help in understanding the significance of the IgG MGUS and its potential progression.<br>2. Anti-MAG Antibodies<br>○ Reasoning: Anti-myelin-associated glycoprotein (MAG) antibodies can be present in patients with paraproteinemic neuropathy, particularly those with IgM MGUS. While IgG MGUS is more common, checking for anti-MAG antibodies can help rule out an immune-mediated neuropathy associated with monoclonal gammopathy.<br>3. Oral Glucose Tolerance Test (OGTT)<br>○ Reasoning: Although initial glucose tests are negative, an OGTT can identify impaired glucose tolerance or early diabetes not captured by fasting glucose or HbA1c. Diabetic polyneuropathy remains a potential differential diagnosis.<br>4. TSH, Free T4, and Anti-thyroid Antibodies<br>○ Reasoning: Re-evaluation of thyroid function given the history of Hashimoto's thyroiditis. Thyroid dysfunction can contribute to neuropathic symptoms, and ensuring adequate thyroid hormone replacement or control is essential.<br>5. CSF Analysis (Lumbar Puncture)<br>○ Reasoning: CSF analysis can help rule out CIDP. Elevated protein levels with a normal cell count (albuminocytologic dissociation) are characteristic of CIDP. This test is crucial for distinguishing between CIDP and other forms of neuropathy.<br>Instrumental Tests<br>6. Skin Biopsy<br>○ Reasoning: To assess small fiber neuropathy, which can be present in various neuropathies including those related to diabetes, MGUS, or autoimmune causes. Intraepidermal nerve fiber density (IENFD) measurement can provide valuable information.<br>7. MRI of the Spine<br>○ Reasoning: To exclude structural causes of neuropathy, such as compressive radiculopathy or spinal cord lesions, which might mimic or contribute to the symptoms.<br>Most Crucial Test<br>Given the patient's history and current findings, Serum Immunofixation Electrophoresis and Free Light Chains is the most crucial test. This test will help further characterize the monoclonal protein, distinguish MGUS from more serious plasma cell disorders like multiple myeloma, and provide clarity on the significance of the detected IgG monoclonal gammopathy. Accurate characterization of the monoclonal protein is essential for appropriate management and prognosis.

80	A 70-year-old male patient presents with a 2-year history of pure sensory, symmetrical, length dependent polyneuropathy with insidious onset. The patient presents with neuropathic pain in the lower limbs in a distal distribution. Clinical examination reveals hyperalgesia to pinprick and absence of tactile hypoesthesia. There are no foot deformities, ataxia, tremor, or cranial nerve involvement. The patient reports reduced sweating. Past medical history is negative. Nerve conduction studies show a pure sensory, symmetric, axonal polyneuropathy. Screening lab tests show raised glycemia, elevated HbA1c, and reduced vitamin B12. Remaining exams including folate, vitamin B6, vitamin E, HBV, HCV, ANA, ANCA, ENA, CRP, ESR, copper, cryoglobulins, and serum electrophoresis are all negative. There is no family history of polyneuropathy.	Diabetic neuropathy	**Step-by-Step Analysis of Comprehensive Data**<br>1. **Review of Clinical Presentation:**<br>○ **Symptoms: Pure sensory, symmetrical, length-dependent polyneuropathy with neuropathic pain in lower limbs, hyperalgesia, and reduced sweating.**<br>○ **Physical Examination: Hyperalgesia to pinprick, absence of tactile hypoesthesia, no ataxia, tremor, or cranial nerve involvement.**<br>2. **Review of Initial Investigations:**<br>○ **Nerve Conduction Studies: Pure sensory, symmetric, axonal polyneuropathy.**<br>○ **Laboratory Tests:**<br>■ **Positive Findings: Raised glycemia, elevated HbA1c, reduced vitamin B12.**<br>■ **Negative Findings: Folate, vitamin B6, vitamin E, HBV, HCV, ANA, ANCA, ENA, CRP, ESR, copper, cryoglobulins, serum electrophoresis.**<br>3. **Review of Medical and Family History:**<br>○ **Past Medical History: Negative.**<br>○ **Family History: No family history of polyneuropathy.**<br>**Additional Examinations to Narrow Down Differential Diagnoses**<br>**1. Oral Glucose Tolerance Test (OGTT)**<br>● **Reasoning: To confirm the diagnosis of diabetes mellitus, especially in the presence of elevated glycemia and HbA1c. This test can detect impaired glucose tolerance or diabetes not previously diagnosed.**<br>● **Rationale: Given the elevated glycemia and HbA1c, confirming the glucose metabolism status is critical in establishing diabetic neuropathy.**<br>**2. Methylmalonic Acid (MMA) and Homocysteine Levels**<br>● **Reasoning: Elevated levels of MMA and homocysteine are more sensitive markers of vitamin B12 deficiency than serum B12 levels alone.**<br>● **Rationale: These tests help confirm vitamin B12 deficiency, as reduced B12 levels were noted. Elevated MMA and homocysteine levels would strengthen the diagnosis of B12 deficiency neuropathy.**<br>**3. Quantitative Sensory Testing (QST)**<br>● **Reasoning: To assess the function of small and large sensory nerve fibers and quantify sensory deficits, which can help differentiate between various types of neuropathies.**<br>● **Rationale: This test can provide more detailed information on the type and extent of sensory loss, which can be useful in distinguishing diabetic neuropathy from other sensory neuropathies.**<br>**4. Skin Biopsy with Intraepidermal Nerve Fiber Density (IENFD)**<br>● **Reasoning: To evaluate small fiber neuropathy, which may not be detected by standard nerve conduction studies.**<br>● **Rationale: This test is particularly useful in patients with sensory symptoms but normal or inconclusive nerve conduction studies, helping to confirm or rule out small fiber neuropathy.**<br>**5. Autonomic Function Tests**<br>● **Reasoning: To assess the involvement of the autonomic nervous system, particularly because the patient reports reduced sweating.**<br>● **Rationale: These tests can help evaluate the extent of autonomic involvement, which is common in diabetic neuropathy but can also occur in other types of polyneuropathies.**<br>**Determining the Most Crucial Test**<br>**Based on the case specifics and the need to confirm the most likely diagnosis:**<br>**Oral Glucose Tolerance Test (OGTT)**<br>● **Reasoning: The elevated glycemia and HbA1c strongly suggest a diabetic etiology, and the OGTT is critical for confirming the diagnosis of diabetes mellitus.**<br>● **Importance: Establishing a definitive diagnosis of diabetes will provide a clear pathway for management and support the diagnosis of diabetic neuropathy. Furthermore, effective management of diabetes can potentially prevent the progression of neuropathy and improve symptoms.**<br>**Conclusion**<br>**The most crucial test from the list is the Oral Glucose Tolerance Test (OGTT). Confirming diabetes mellitus is paramount, as it not only solidifies the diagnosis of diabetic neuropathy but also guides further management and treatment strategies for the patient.**
81	A 67-year-old male patient presents with a 1-week history of pure motor, symmetrical, non-length dependent polyneuropathy with hyperacute onset. The patient presents with facial diplegia, diplopia, and weakness of the intrinsic muscles of the hands. Clinical examination reveals the presence of ophthalmoparesis affecting the right retrolateral muscle and the left superior rectus, facial diplegia, weakness of the intrinsic muscles of both hands, and mild sensory ataxia. There are no neuropathic pain, tremor, foot deformities or autonomic involvement. Past medical history is positive for type 1 diabetes, IgG MGUS, and arterial hypertension. Nerve conduction studies show normal result. Screening lab tests show hyperglycaemia and raised HbA1c levels, IgG monoclonal gammopathy, and mild increase of ESR and CRP levels. Remaining exams including vitamin B12, folate, vitamin B6, vitamin E, HBV, HCV, ANA, ENA, ANCA, copper, and cryoglobulins are normal. There is no family history of polyneuropathy.	GBS-facial diplegia variant	**Step-by-Step Analysis and Additional Examinations**<br>**Step 1: Review of Clinical Presentation and Initial Findings**<br>● **Clinical Presentation:**<br>○ **67-year-old male with a 1-week history of pure motor, symmetrical, non-length dependent polyneuropathy**<br>○ **Facial diplegia, diplopia, weakness of intrinsic hand muscles, and ophthalmoparesis**<br>○ **Mild sensory ataxia without pain, tremor, foot deformities, or autonomic involvement**<br>● **Past Medical History:**<br>○ **Type 1 diabetes, IgG MGUS, arterial hypertension**<br>● **Initial Findings:**<br>○ **Normal nerve conduction studies**<br>○ **Hyperglycemia, raised HbA1c levels, IgG monoclonal gammopathy, mildly increased ESR and CRP**<br>○ **Normal levels of vitamin B12, folate, vitamin B6, vitamin E, HBV, HCV, ANA, ENA, ANCA, copper, and cryoglobulins**<br>○ **No family history of polyneuropathy**<br>**Step 2: Identifying Key Clinical Questions**<br>1. **What is causing the acute onset of motor symptoms?**<br>2. **Is there an underlying immune-mediated or inflammatory process?**<br>3. **Is there an involvement of any specific pathogens or antibodies?**<br>4. **How does the patient's history of diabetes and MGUS influence the differential diagnoses?**<br>**Step 3: Proposing Additional Examinations**<br>1. **Lumbar Puncture (CSF Analysis)**<br>○ **Reasoning:**<br>■ **To evaluate for albuminocytologic dissociation, which is characteristic of Guillain-Barré Syndrome (GBS).**<br>■ **Increased protein levels with normal white blood cell count in CSF support the diagnosis of GBS.**<br>■ **Helps in ruling out other infectious or inflammatory conditions of the CNS.**<br>2. **Anti-GQ1b Antibody Testing**<br>○ **Reasoning:**

			■ **Highly specific for Miller Fisher variant of GBS.**<br>■ **The presence of anti-GQ1b antibodies can confirm the diagnosis given the clinical presentation of ophthalmoparesis and ataxia.**<br>**3. MRI of the Brain and Spinal Cord**<br>○ **Reasoning:**<br>■ **To rule out central nervous system lesions, such as those caused by multiple sclerosis or stroke, which could mimic peripheral neuropathy.**<br>■ **Can provide evidence of nerve root enhancement that may be seen in GBS or CIDP.**<br>**4. Repeat Nerve Conduction Studies (NCS) and Electromyography (EMG)**<br>○ **Reasoning:**<br>■ **To detect any late-onset changes that may not have been apparent in initial studies.**<br>■ **Helps in differentiating between demyelinating and axonal neuropathies.**<br>■ **Provides further information on the extent and nature of nerve involvement.**<br>**5. Serum Electrophoresis and Immunofixation**<br>○ **Reasoning:**<br>■ **Detailed analysis of the monoclonal gammopathy (IgG MGUS) to assess the possibility of progression to multiple myeloma or related disorders.**<br>■ **MGUS can be associated with neuropathies, and a thorough evaluation is necessary.**<br>**6. Autoantibody Panel (including Anti-MAG, Anti-Hu, Anti-Ri)**<br>○ **Reasoning:**<br>■ **To evaluate for paraneoplastic syndromes or other autoimmune causes of neuropathy.**<br>■ **These antibodies can be associated with peripheral neuropathies and can guide treatment.**<br>**7. Infectious Disease Screening (including HIV, Lyme Disease)**<br>○ **Reasoning:**<br>■ **To rule out infections that can present with neuropathy.**<br>■ **These infections can have neurological manifestations that may present similarly.**<br>**8. Glycated Hemoglobin (HbA1c) and Extended Diabetes Panel**<br>○ **Reasoning:**<br>■ **To further evaluate the patient's diabetes control.**<br>■ **Poorly controlled diabetes can lead to diabetic neuropathy, and understanding the extent of diabetes management can guide the diagnosis.**<br>**Step 4: Determining the Most Crucial Test**<br>**Given the clinical presentation and initial findings, the most crucial test to narrow down the differential diagnoses is:**<br>**Lumbar Puncture (CSF Analysis)**<br>● **Reasoning:**<br>○ **CSF analysis for albuminocytologic dissociation is a key diagnostic criterion for Guillain-Barré Syndrome (GBS).**<br>○ **This test can provide immediate and direct evidence supporting the diagnosis of GBS, especially in the hyperacute setting.**<br>○ **While other tests are also important, CSF analysis is crucial for confirming or ruling out GBS, which is a medical emergency requiring prompt treatment.**<br>**This approach ensures a comprehensive evaluation of the patient's condition, focusing on the most likely and urgent diagnoses while systematically ruling out other potential causes.**
82	An 87-year-old male patient presents with a 6-month history of pure sensory, symmetrical, length dependent polyneuropathy with subacute onset. The patient presents with leg paresthesia, tremor and sensory ataxia without neuropathic pain, foot deformities, cranial nerve or autonomic involvement. Past medical history is silent. Nerve conduction studies show pure a symmetric, sensory, axonal polyneuropathy and unilateral carpal tunnel syndrome. Screening lab tests including folate, vitamin B6, vitamin E, HCV, HBV, ANA, ANCA, ENA, CRP, ESR, glycemia, HbA1c, copper, cryoglobulins and serum electrophoresis are all negative, with evidence of vitamin B12 deficiency. There is no family history of polyneuropathy.	B12-deficiency polyneuropathy	**Step-by-Step Analysis of Additional Examinations**<br>**Initial Clinical Information Recap**<br>● **Patient: 87-year-old male**<br>● **History: 6-month pure sensory, symmetrical, length-dependent polyneuropathy with subacute onset**<br>● **Symptoms: Leg paresthesia, tremor, sensory ataxia, no neuropathic pain, no foot deformities, no cranial nerve or autonomic involvement**<br>● **Nerve Conduction Studies: Pure sensory, symmetric, axonal polyneuropathy and unilateral carpal tunnel syndrome**<br>● **Screening Lab Tests: Negative for common causes except for vitamin B12 deficiency**<br>● **Family History: No history of polyneuropathy**<br>**Additional Examinations and Reasoning**<br>**1. Methylmalonic Acid (MMA) and Homocysteine Levels**<br>○ **Reasoning: These are more sensitive markers of vitamin B12 deficiency. Elevated levels can confirm the functional deficiency of B12 even if serum B12 levels are borderline or slightly low. This can provide further evidence supporting vitamin B12 deficiency as the cause of the neuropathy.**<br>**2. MRI of the Brain and Spinal Cord**<br>○ **Reasoning: MRI can help identify structural abnormalities or lesions in the central nervous system that may contribute to sensory ataxia and tremor. It can also show the characteristic findings of subacute combined degeneration of the spinal cord, which is associated with vitamin B12 deficiency.**<br>**3. Lumbar Puncture and Cerebrospinal Fluid (CSF) Analysis**<br>○ **Reasoning: This can help rule out other inflammatory or infectious causes of neuropathy. Elevated protein levels with normal cell counts can be seen in chronic inflammatory demyelinating polyneuropathy (CIDP) or paraneoplastic syndromes.**<br>**4. Skin Biopsy for Intraepidermal Nerve Fiber Density**<br>○ **Reasoning: This test is useful for diagnosing small fiber neuropathy, which might not be detected by standard nerve conduction studies. It helps to assess the density of small nerve fibers in the skin, providing information on peripheral nerve integrity.**<br>**5. Genetic Testing**<br>○ **Reasoning: While family history is negative, late-onset hereditary neuropathies should be considered, especially if no clear acquired cause is identified. Testing for mutations associated with hereditary sensory and autonomic neuropathies (HSAN) and other genetic neuropathies can be helpful.**<br>**6. Serum and Urine Protein Electrophoresis and Immunofixation**<br>○ **Reasoning: To rule out monoclonal gammopathy of undetermined significance (MGUS), multiple myeloma, or amyloidosis. These conditions can cause axonal polyneuropathy and might not be detected with routine serum electrophoresis alone.**<br>**7. Anti-Ganglioside Antibodies (e.g., anti-GM1, anti-GQ1b)**

			○ **Reasoning: These antibodies are associated with certain autoimmune neuropathies like Guillain-Barré syndrome variants and multifocal motor neuropathy. Though less likely, they should be considered if autoimmune etiology is suspected.**<br>**Most Crucial Test**<br>**The most crucial test from this list is Methylmalonic Acid (MMA) and Homocysteine Levels. Given the confirmed vitamin B12 deficiency and the patient's symptoms, these tests can substantiate the functional impact of the deficiency. Elevated MMA and homocysteine levels would strongly support vitamin B12 deficiency as the primary cause of the polyneuropathy and guide appropriate treatment.**<br>**Conclusion**<br>**Based on the additional examinations proposed and the patient's clinical scenario, measuring Methylmalonic Acid (MMA) and Homocysteine Levels is crucial. It confirms the functional deficiency of vitamin B12, helping to solidify the diagnosis and guide targeted treatment, which is critical in preventing further neurological damage and potentially reversing the symptoms.**
83	A 72-year-old male patient presents with a 1-year history of sensory-motor, symmetrical, non-length dependent polyneuropathy with subacute onset. The patient presents with hand weakness, paresthesia and hypoesthesia in the lower limbs, sensory ataxia, dysphagia, and pes cavus. There are no neuropathic pain or autonomic involvement. Past medical history is silent. Nerve conduction studies show a demyelinating, sensory-motor, asymmetric polyneuropathy. Screening lab tests including vitamin B12, folate, vitamin B6, vitamin E, HCV, HBV, ANA, ANCA, ENA, CRP, ESR, glycemia, HbA1c, copper, cryoglobulins and serum electrophoresis are all negative. There is no family history of polyneuropathy.	CIDP	**Step-by-Step Analysis for Additional Examinations**<br>**1. Cerebrospinal Fluid (CSF) Analysis**<br>● **Reasoning: A CSF analysis can provide valuable information regarding inflammatory and infectious processes affecting the nervous system. In CIDP, an elevated protein level without a significant increase in white blood cells (albuminocytologic dissociation) is often observed. This test helps to confirm the diagnosis of CIDP and differentiate it from other types of neuropathy.**<br>● **Tests: Protein levels, white blood cell count, glucose, oligoclonal bands.**<br>**2. MRI of the Spine and Nerve Roots**<br>● **Reasoning: MRI can detect nerve root enhancement and thickening, which are indicative of CIDP. It can also help rule out structural causes of neuropathy and look for signs of paraneoplastic syndromes that may involve the nervous system.**<br>● **Tests: MRI with gadolinium contrast of the cervical, thoracic, and lumbosacral spine.**<br>**3. Anti-GM1 Antibodies**<br>● **Reasoning: The presence of anti-GM1 antibodies is suggestive of multifocal motor neuropathy (MMN), which can present with asymmetric demyelinating neuropathy. Although MMN primarily affects motor nerves, sensory involvement can occasionally occur. Testing for these antibodies can help differentiate MMN from other demyelinating neuropathies.**<br>● **Tests: Serum anti-GM1 antibody titers.**<br>**4. Repeat Nerve Conduction Studies with Electromyography (EMG)**<br>● **Reasoning: A detailed repeat nerve conduction study combined with EMG can help further characterize the pattern and extent of demyelination and axonal damage. This can help differentiate between CIDP, MMN, and paraneoplastic neuropathy.**<br>● **Tests: Nerve conduction studies, EMG of both upper and lower limbs.**<br>**5. Serum and Urine Protein Electrophoresis with Immunofixation**<br>● **Reasoning: This test can help identify monoclonal gammopathies, which are associated with certain types of neuropathy such as POEMS syndrome and multiple myeloma-related neuropathy. It is important to rule out these conditions as potential causes of demyelinating polyneuropathy.**<br>● **Tests: Serum protein electrophoresis (SPEP), urine protein electrophoresis (UPEP), and immunofixation.**<br>**6. Paraneoplastic Antibody Panel**<br>● **Reasoning: Testing for paraneoplastic antibodies can help identify a hidden malignancy associated with neuropathy. Paraneoplastic syndromes often present with subacute onset neuropathy and can be associated with cancers like small cell lung cancer and lymphoma.**<br>● **Tests: Serum paraneoplastic panel (e.g., anti-Hu, anti-Ri, anti-Yo antibodies).**<br>**7. Autoimmune Panel**<br>● **Reasoning: A comprehensive autoimmune panel can help identify other autoimmune conditions that could present with neuropathy. Although initial tests were negative, more specific autoimmune markers might be necessary.**<br>● **Tests: Anti-MAG (myelin-associated glycoprotein), anti-GQ1b antibodies, anti-MOG antibodies, anti-neurofascin antibodies.**<br>**8. Full Body PET-CT Scan**<br>● **Reasoning: To detect any occult malignancy that could be causing paraneoplastic neuropathy, a full body PET-CT scan is warranted. This imaging modality is sensitive for identifying cancers that might not be evident on conventional imaging.**<br>● **Tests: Whole-body PET-CT scan.**<br>**Conclusion: Most Crucial Test**<br>**The most crucial test from this list is the Cerebrospinal Fluid (CSF) Analysis. Given the clinical presentation and the findings on nerve conduction studies, CSF analysis is key for diagnosing CIDP, as it can reveal the characteristic albuminocytologic dissociation. This test will significantly aid in confirming CIDP as the most likely diagnosis and will help differentiate it from other potential causes of demyelinating polyneuropathy.**
84	A 60-year-old male patient presents with a 1-month history of pure sensory, symmetrical, length dependent polyneuropathy with subacute onset. The patient presents with frequent cramps, paresthesia, and neuropathic pain in the feet. There are no sensory ataxia, tremor, foot deformities, cranial nerve or autonomic involvement. Past medical history is positive for multiple myeloma, for which the patient has undergone chemotherapy. Symptoms started after the first cycle of chemotherapy. Nerve conduction studies show a pure sensory, symmetric, axonal polyneuropathy. Screening lab tests including vitamin B12, folate, vitamin B6, vitamin E, HBV, HCV, glycemia, HbA1c, ANA, ANCA, ENA, CRP, ESR, copper, and cryoglobulins are all negative, with positivity for IgG kappa	Chemotherapy-induced neuropathy	**Step-by-Step Analysis and Additional Examinations**<br>**1. Review of Current Data:**<br>○ **60-year-old male with a history of multiple myeloma.**<br>○ **Developed pure sensory, symmetrical, length-dependent polyneuropathy post-chemotherapy.**<br>○ **Symptoms include cramps, paresthesia, and neuropathic pain in the feet.**<br>○ **Nerve conduction studies indicate axonal polyneuropathy.**<br>○ **Extensive screening tests were negative except for the presence of IgG kappa paraprotein.**<br>**2. Goals of Additional Testing:**<br>○ **Confirm the diagnosis of chemotherapy-induced neuropathy.**<br>○ **Rule out other potential causes of neuropathy.**<br>○ **Assess the extent and severity of the neuropathy.**<br>○ **Evaluate for any treatable or reversible conditions.**<br>**Additional Examinations and Reasoning**<br>**1. Quantitative Sensory Testing (QST)**<br>○ **Reasoning: QST can objectively measure sensory nerve function, providing detailed information about the type and severity of sensory deficits. It helps in differentiating between small and large fiber neuropathies, which is crucial in assessing the impact of chemotherapy on different nerve fibers.**<br>**2. Skin Biopsy with Intraepidermal Nerve Fiber Density (IENFD)**<br>○ **Reasoning: A skin biopsy can provide a quantitative measure of small fiber neuropathy, which may not be detected on nerve conduction studies. This is particularly useful if the patient's symptoms are out of proportion to the findings on nerve conduction studies.**<br>**3. Electromyography (EMG)**

	paraprotein. There is no family history of polyneuropathy.		○ **Reasoning: EMG can help assess for any motor involvement that may not be clinically apparent. Although the current presentation is purely sensory, subtle motor involvement can sometimes be detected with EMG, providing a more comprehensive assessment of the neuropathy.**<br>**4. Serum and Urine Immunofixation Electrophoresis**<br>○ **Reasoning: This test is more sensitive than standard protein electrophoresis and can detect monoclonal proteins, helping to further evaluate the significance of the IgG kappa paraprotein. It is important in distinguishing MGUS-related neuropathy from other causes.**<br>**5. Repeat Nerve Conduction Studies**<br>○ **Reasoning: Given the subacute onset, repeating nerve conduction studies can provide information about the progression of the neuropathy and help in monitoring response to any interventions.**<br>**6. Antibodies Testing (anti-MAG, anti-GM1, anti-Hu, anti-Yo)**<br>○ **Reasoning: Testing for antibodies associated with paraneoplastic syndromes and other immune-mediated neuropathies can help identify less common causes of neuropathy that may be related to the underlying malignancy or its treatment.**<br>**7. Lumbar Puncture with Cerebrospinal Fluid (CSF) Analysis**<br>○ **Reasoning: CSF analysis can help rule out central nervous system involvement and infections/inflammatory causes. Elevated protein levels with normal cell count (albuminocytologic dissociation) can suggest a paraneoplastic or demyelinating process.**<br>**8. Whole-Body PET/CT Scan**<br>○ **Reasoning: This imaging modality can detect any additional malignancies or metastatic disease that might be contributing to the neuropathy, providing a comprehensive assessment of the patient's oncological status.**<br>**Most Crucial Test**<br>**Serum and Urine Immunofixation Electrophoresis**<br>**This test is crucial because it can provide a definitive assessment of the monoclonal protein detected in the patient's serum. Given the presence of IgG kappa paraprotein, it is essential to determine whether this is related to MGUS or multiple myeloma, which could significantly influence the management and prognosis of the patient's neuropathy. This test can help differentiate between a paraneoplastic syndrome and other potential causes of polyneuropathy, thereby guiding further management strategies.**
85	A 63-year-old male patient presents with a 4-year history of sensory-motor, symmetrical, length dependent polyneuropathy with insidious onset and fast progression. The patient presents with severe distal motor weakness and hypoesthesia in the four limbs, dysphagia, dysphonia, and autonomic involvement with no foot deformities or tremor. Past medical history shows lung cancer (20 years ago) but he did not undergo chemotherapy. Nerve conduction studies show an axonal, sensory-motor polyneuropathy with bilateral carpal tunnel syndrome. Screening lab tests including vitamin B12, folate, vitamin B6, vitamin E, HBV, HCV, ANA, ANCA, ENA, glycaemia, HbA1c, CRP, ESR, copper, cryoglobulins, and serum electrophoresis are all negative. There is no family history of polyneuropathy.	TTR-Amyloidosis	**Step-by-Step Analysis of Additional Examinations**<br>**1. Comprehensive Data Review**<br>**Clinical Summary:**<br>● **63-year-old male**<br>● **4-year history of sensory-motor, symmetrical, length-dependent polyneuropathy**<br>● **Severe distal motor weakness**<br>● **Hypoesthesia in all four limbs**<br>● **Dysphagia and dysphonia**<br>● **Autonomic involvement**<br>● **Past history of lung cancer (20 years ago), no chemotherapy**<br>● **Nerve conduction studies: axonal, sensory-motor polyneuropathy, bilateral carpal tunnel syndrome**<br>● **Negative screening lab tests for multiple potential causes**<br>● **No family history of polyneuropathy**<br>**2. Additional Examinations**<br>**Objective: To further investigate the etiology of the polyneuropathy and narrow down differential diagnoses, particularly focusing on paraneoplastic syndrome, CIDP, and amyloidosis.**<br>**Proposed Tests:**<br>**1. Paraneoplastic Antibody Panel**<br>○ **Reasoning: Given the patient's history of lung cancer and the clinical suspicion of paraneoplastic neuropathy, testing for paraneoplastic antibodies (e.g., anti-Hu, anti-Yo, anti-Ri, anti-CV2/CRMP5) is critical. These antibodies can help identify an immune response related to an underlying malignancy, even years after the initial cancer diagnosis.**<br>**2. Serum and Urine Immunofixation Electrophoresis (IFE)**<br>○ **Reasoning: To rule out multiple myeloma or related plasma cell disorders, which can present with neuropathy. Monoclonal gammopathies can be detected by this test, which might not show up on routine serum electrophoresis.**<br>**3. Fat Pad Biopsy or Nerve Biopsy**<br>○ **Reasoning: To confirm or exclude amyloidosis, a fat pad biopsy can be done to detect amyloid deposits. If negative and suspicion remains high, a nerve biopsy could be considered, although more invasive.**<br>**4. Lumbar Puncture with Cerebrospinal Fluid (CSF) Analysis**<br>○ **Reasoning: For suspected CIDP, CSF analysis can reveal elevated protein levels with a normal cell count (albuminocytologic dissociation). It can also help exclude infectious or neoplastic processes.**<br>**5. Imaging Studies: PET-CT or Full-Body CT Scan**<br>○ **Reasoning: To detect occult malignancies which might be contributing to paraneoplastic syndrome. A PET-CT can be particularly useful for identifying metabolically active lesions.**<br>**6. Autonomic Function Tests**<br>○ **Reasoning: To objectively assess the extent of autonomic involvement, tests like tilt-table testing, heart rate variability studies, and sudomotor testing can be performed. This can help quantify autonomic dysfunction and guide management.**<br>**7. Genetic Testing**<br>○ **Reasoning: Although there is no family history, some hereditary neuropathies can present later in life or be sporadic. Genetic testing can help rule out hereditary causes of polyneuropathy, such as hereditary transthyretin amyloidosis.**<br>**Most Crucial Test**<br>**Paraneoplastic Antibody Panel Reasoning: Given the patient's history of lung cancer and the clinical picture suggesting paraneoplastic neuropathy, identifying paraneoplastic antibodies can directly point towards an immune-mediated process related to a remote or occult malignancy. This test is essential because it not only supports the diagnosis but also may guide further investigations for occult cancers and influence treatment strategies.**<br>**Conclusion**<br>**Conducting the paraneoplastic antibody panel is the most crucial step in this patient's further workup. This test can confirm the presence of an immune-mediated process related to his previous malignancy and prompt further investigations to detect any underlying or recurrent cancer.**
86	A 39-year-old female patient presents with a 34-year history of sensory-motor,	CMT2	**Step-by-Step Analysis and Additional Examinations**<br>**Step 1: Review Initial Clinical and Diagnostic Findings**

	symmetrical, length-dependent polyneuropathy with insidious onset and slowly progressive course. The patient reports paresthesia and neuropathic pain in the hands and feet, sensory ataxia, cramps, distal weakness in all four limbs, moderate calf atrophy, and pes cavus. Clinical examination does not reveal cranial nerve or autonomic involvement. Past medical history is unremarkable. Nerve conduction studies show an axonal, symmetric, length-dependent sensory-motor polyneuropathy. Screening lab tests including vitamin B12, folate, vitamin B6, vitamin E, HBV, HCV, glycemia, HbA1c, ANA, ANCA, ENA, CRP, ESR, copper, cryoglobulins, and serum electrophoresis are all negative. Her father is wheelchair-bound, and her brother and sister have walking difficulties.		**Summary of Clinical and Diagnostic Findings:**<br>● Patient: 39-year-old female with 34-year history of sensory-motor, symmetrical, length-dependent polyneuropathy.<br>● Symptoms: Paresthesia, neuropathic pain, sensory ataxia, cramps, distal weakness, moderate calf atrophy, pes cavus.<br>● Family History: Father wheelchair-bound; brother and sister have walking difficulties.<br>● Initial Lab Tests: Negative for vitamin deficiencies, infections, autoimmune markers, diabetes, cryoglobulins, serum electrophoresis.<br>● Nerve Conduction Studies: Axonal, symmetric, length-dependent sensory-motor polyneuropathy.<br>**Step 2: List Additional Examinations**<br>1. Genetic Testing for Hereditary Neuropathies<br>○ Reasoning: Given the strong family history and clinical presentation, genetic testing can identify specific mutations associated with Charcot-Marie-Tooth disease (CMT) and other hereditary neuropathies. Tests may include:<br>■ PMP22 gene duplication/deletion (CMT1A)<br>■ GJB1 gene mutation (CMTX)<br>■ MFN2 gene mutation (CMT2A)<br>■ Other relevant genetic panels.<br>○ Impact: Confirmation of a genetic mutation will definitively diagnose a hereditary neuropathy, guiding prognosis and genetic counseling.<br>2. Nerve Biopsy<br>○ Reasoning: While invasive, a nerve biopsy can provide histopathological confirmation of axonal versus demyelinating neuropathy and help identify any inflammatory or vasculitic processes.<br>○ Impact: It can distinguish between hereditary neuropathies and acquired conditions like CIDP or vasculitic neuropathies, which might present with similar clinical features.<br>3. Autonomic Function Testing<br>○ Reasoning: Although the clinical exam did not reveal autonomic involvement, subclinical autonomic dysfunction could be present. Tests might include:<br>■ Heart rate variability<br>■ Tilt-table test<br>■ Sudomotor function tests.<br>○ Impact: Identifying autonomic involvement can help differentiate between various types of polyneuropathies and guide management.<br>4. Advanced Imaging (MRI) of Nerves<br>○ Reasoning: MRI can visualize nerve roots and peripheral nerves, potentially identifying hypertrophy, inflammation, or other structural abnormalities.<br>○ Impact: Can provide supportive evidence for demyelinating conditions or reveal compressive neuropathies not evident on clinical exam and nerve conduction studies.<br>5. CSF Analysis (Lumbar Puncture)<br>○ Reasoning: Analysis of cerebrospinal fluid (CSF) can identify elevated protein levels and presence of cells, which is indicative of an inflammatory neuropathy like CIDP.<br>○ Impact: Useful in distinguishing CIDP from hereditary neuropathies.<br>**Step 3: Determine the Most Crucial Test**<br>**Most Crucial Test: Genetic Testing for Hereditary Neuropathies**<br>**Reasoning:**<br>● High Yield: Given the strong family history and typical presentation of a hereditary neuropathy, genetic testing is likely to yield a definitive diagnosis.<br>● Specificity: Genetic testing can precisely identify the mutation responsible, confirming the diagnosis of conditions like CMT.<br>● Impact on Management: A confirmed genetic diagnosis will influence long-term management, prognosis, and genetic counseling for the patient and family members.<br>● Non-Invasive: Compared to nerve biopsy or lumbar puncture, genetic testing is non-invasive and has fewer risks.<br>Conclusion: Given the patient's clinical presentation, family history, and initial diagnostic findings, genetic testing for hereditary neuropathies is the most crucial test. It has the highest potential to provide a definitive diagnosis and significantly impacts clinical management and family counseling.
87	A 50-year-old male patient presents with a 1-month history of pure motor multineuropathy with acute onset while hospitalized in ICU for sepsis. Clinical examination reveals the presence of dysphagia, dysphonia, hand tremor, and upper limb weakness in a multifocal distribution. There are no sensory deficits, nor neuropathic pain, tremor or foot deformities. Past medical history is positive for testicular cancer, for which the patient underwent chemotherapy three years ago. Nerve conduction studies show pure motor, axonal multineuropathy with one motor conduction block. Screening lab tests including vitamin B12, folate, vitamin B6, vitamin E, HCV, HBV, ANA, ANCA, ENA, CRP, ESR, glycemia, HbA1c, copper, cryoglobulins and serum electrophoresis are all negative. There is no family history of polyneuropathy.	GBS	**Step-by-Step Analysis of the Clinical Scenario**<br>**Patient Profile and History Recap:**<br>● 50-year-old male<br>● Acute onset of pure motor multineuropathy during ICU stay for sepsis<br>● Symptoms: Dysphagia, dysphonia, hand tremor, upper limb weakness (multifocal distribution)<br>● No sensory deficits, neuropathic pain, or foot deformities<br>● Past medical history: Testicular cancer treated with chemotherapy three years ago<br>● Nerve conduction studies: Pure motor, axonal multineuropathy with one motor conduction block<br>● Screening lab tests (vitamin B12, folate, vitamin B6, vitamin E, HCV, HBV, ANA, ANCA, ENA, CRP, ESR, glycemia, HbA1c, copper, cryoglobulins, serum electrophoresis) are all negative<br>● No family history of polyneuropathy<br>**Additional Examinations**<br>**1. Cerebrospinal Fluid (CSF) Analysis:**<br>● Reasoning: CSF analysis can help identify any inflammatory or infectious processes affecting the nervous system, such as Guillain-Barré syndrome (GBS) or other inflammatory neuropathies. Elevated protein levels with normal cell counts (albuminocytologic dissociation) are indicative of GBS.<br>● Tests: CSF protein, glucose, cell count, and cultures<br>**2. MRI of the Brain and Spinal Cord:**<br>● Reasoning: MRI can reveal structural abnormalities, demyelination, or other pathologies in the central nervous system that might be contributing to the neuropathy. It can also help rule out metastatic lesions, given the patient's history of cancer.<br>● Tests: MRI with contrast of the brain and cervical/thoracic spine<br>**3. Repeat Electromyography (EMG) and Nerve Conduction Studies (NCS):**<br>● Reasoning: A repeat EMG/NCS can provide more detailed information about the progression and characteristics of the neuropathy. It can help distinguish between axonal and demyelinating processes and assess the extent of motor involvement.<br>● Tests: Detailed EMG/NCS focusing on different muscle groups and nerves

			4. Autoantibody Panel:<br>● Reasoning: Testing for additional autoantibodies can help identify paraneoplastic syndromes or other autoimmune neuropathies that may not be covered by the initial screening. This includes testing for anti-Hu, anti-Yo, anti-Ri, and anti-MAG antibodies.<br>● Tests: Comprehensive paraneoplastic antibody panel, anti-GM1, anti-MAG<br>5. Serum Onconeural Antibodies:<br>● Reasoning: As part of the investigation for a paraneoplastic syndrome, checking for onconeural antibodies associated with testicular cancer (e.g., anti-Ma2) can help identify a possible paraneoplastic cause of the neuropathy.<br>● Tests: Serum onconeural antibodies specific for testicular cancer<br>6. Muscle and Nerve Biopsy:<br>● Reasoning: A biopsy can provide definitive histopathological evidence of the type of neuropathy, showing signs of inflammation, vasculitis, or other specific pathologies that may not be evident through non-invasive tests.<br>● Tests: Biopsy of an affected muscle and adjacent nerve<br>Most Crucial Test<br>Cerebrospinal Fluid (CSF) Analysis<br>Rationale:<br>● Diagnostic Yield: CSF analysis can quickly identify or rule out Guillain-Barré syndrome, which is an important differential diagnosis given the acute onset and purely motor nature of the neuropathy.<br>● Speed and Specificity: It provides timely and specific information that can guide further management, such as the need for intravenous immunoglobulin or plasmapheresis in the case of GBS.<br>● Exclusion of Central Nervous System Infection or Inflammation: It helps rule out infectious or inflammatory processes, which is crucial for directing appropriate treatment.<br>By conducting a CSF analysis, we can narrow down the differential diagnoses effectively and determine the next steps in the patient's management plan.
88	A 58-year-old male patient presents with a 15-month history of sensory-motor, symmetrical, length-dependent polyneuropathy with insidious onset and progressive course. The patient reports distal paresthesia, numbness, neuropathic pain in the lower limbs in a symmetric distribution, and sensory ataxia. Clinical examination confirms the presence of sensory ataxia, postural hand tremor, and mild weakness in ankle dorsiflexion. There are no foot deformities, cranial nerve involvement, or autonomic dysfunction. Past medical history is positive for IgM kappa MGUS and type 2 diabetes. Nerve conduction studies show a demyelinating, length-dependent, symmetric, sensory-motor polyneuropathy. Screening lab tests including vitamin B12, folate, vitamin B6, vitamin E, HCV, HBV, ANA, ANCA, ENA, CRP, ESR, glycemia, copper, and cryoglobulins are all negative, with evidence of IgM kappa paraprotein and elevated HbA1c levels. There is no family history of polyneuropathy.	Anti-MAG polyneuropathy	Step-by-Step Analysis for Additional Examinations:<br>1. Review Initial Data and Identify Gaps:<br>○ Current findings include demyelinating polyneuropathy, elevated HbA1c, and IgM kappa paraprotein.<br>○ Exclusions of other common causes like vitamin deficiencies and infections have already been performed.<br>○ The differential diagnoses are CIDP, diabetic neuropathy, and paraproteinemic neuropathy.<br>2. Establish Additional Tests Needed:<br>A. Electrophysiological Studies:<br>○ Rationale: Detailed electrophysiological studies, including repetitive nerve stimulation and single-fiber electromyography (EMG), can provide more insight into the nature of demyelination and help differentiate between CIDP and paraproteinemic neuropathy.<br>○ Purpose: Confirm demyelinating patterns and evaluate for conduction blocks or other features specific to CIDP.<br>3. B. Serum Immunofixation Electrophoresis and Free Light Chain Assay:<br>○ Rationale: To quantify and further characterize the IgM kappa paraprotein.<br>○ Purpose: Distinguish between benign MGUS and potential malignant transformation (e.g., Waldenström's macroglobulinemia or multiple myeloma).<br>4. C. Lumbar Puncture (CSF Analysis):<br>○ Rationale: Analysis of cerebrospinal fluid (CSF) for elevated protein without pleocytosis can support a diagnosis of CIDP.<br>○ Purpose: Detect albuminocytologic dissociation which is typical in CIDP.<br>5. D. Skin Biopsy with Nerve Fiber Density Analysis:<br>○ Rationale: Assess small fiber neuropathy, which can be seen in diabetes and paraproteinemic neuropathy.<br>○ Purpose: Quantify intraepidermal nerve fiber density to differentiate between small fiber involvement.<br>6. E. Nerve Biopsy:<br>○ Rationale: Direct histological examination of the nerve can reveal characteristic changes of CIDP, amyloid deposits, or vasculitis.<br>○ Purpose: Differentiate between CIDP, amyloidosis, and paraproteinemic neuropathy.<br>7. F. Serum Protein Electrophoresis (SPEP) and Urine Protein Electrophoresis (UPEP):<br>○ Rationale: To detect and quantify monoclonal protein in serum and urine.<br>○ Purpose: Further evaluate the extent of paraproteinemia and rule out multiple myeloma or related disorders.<br>8. G. Hematological Evaluation:<br>○ Rationale: Comprehensive blood tests, including bone marrow biopsy, if needed.<br>○ Purpose: Assess for underlying hematologic malignancies associated with IgM kappa paraprotein.<br>9. Most Crucial Test:<br>○ CSF Analysis via Lumbar Puncture:<br>■ Reasoning: This test is critical as it can provide definitive support for CIDP through detection of elevated protein levels with normal white cell count (albuminocytologic dissociation).<br>■ Impact: Positive findings would strongly suggest CIDP, guiding towards immunomodulatory treatment, while negative findings would shift focus towards a paraproteinemic etiology.<br>Detailed Test Reasoning:<br>● Electrophysiological Studies: These help refine the understanding of the polyneuropathy's demyelinating nature and may reveal features typical of CIDP, such as conduction blocks.<br>● Serum Immunofixation and Free Light Chain Assay: These tests provide a deeper analysis of the IgM paraprotein, necessary for differentiating between MGUS and potential malignancies.<br>● Lumbar Puncture (CSF Analysis): This can confirm CIDP through typical CSF findings.<br>● Skin Biopsy: This can uncover small fiber involvement, useful in differentiating diabetic neuropathy and paraproteinemic neuropathy.<br>● Nerve Biopsy: Direct examination of nerve tissue can identify histopathological features specific to different neuropathies, including CIDP and amyloidosis.<br>● SPEP and UPEP: These quantify monoclonal proteins, essential for diagnosing related plasma cell disorders.<br>● Hematological Evaluation: Comprehensive blood analysis and possibly bone marrow biopsy help detect underlying hematologic conditions associated with paraproteinemia.<br>By performing these additional tests, a more accurate and comprehensive diagnosis can be reached, ensuring appropriate management and treatment for the patient.
89	A 73-year-old male patient presents with a 2-year history of sensory-motor, symmetrical, non-length dependent polyneuropathy with subacute onset. The patient presents with paresthesia and	POEMS	Step-by-Step Analysis:<br>Step 1: Review Clinical Presentation and Initial Findings<br>● History: 2-year history of sensory-motor, symmetrical, non-length dependent polyneuropathy with subacute onset.<br>● Symptoms: Paresthesia, neuropathic pain, proximal and distal weakness, mild sensory ataxia, cramps.<br>● Examination: Cutaneous hyperpigmentation, no foot deformities, tremor, cranial nerve or autonomic involvement.

	neuropathic pain in a stocking and glove distribution, proximal and distal weakness in the lower limbs, mild sensory ataxia and cramps. There are no foot deformities, tremor, cranial nerve or autonomic involvement. Clinical examination reveals cutaneous hyperpigmentation. Past medical history is positive for IgG lambda monoclonal gammopathy. Nerve conduction studies show a mixed demyelinating-axonal, sensory-motor, symmetric polyneuropathy. Screening lab tests including vitamin B12, folate, vitamin B6, vitamin E, HCV, HBV, ANA, ANCA, ENA, CRP, ESR, glycemia, HbA1c, copper and cryoglobulins are all negative, with positivity for IgG lambda paraprotein. There is no family history of polyneuropathy.		● Past Medical History: IgG lambda monoclonal gammopathy.<br>● Nerve Conduction Studies: Mixed demyelinating-axonal, sensory-motor, symmetric polyneuropathy.<br>● Screening Labs: Negative for vitamin B12, folate, vitamin B6, vitamin E, HCV, HBV, ANA, ANCA, ENA, CRP, ESR, glycemia, HbA1c, copper, cryoglobulins; Positive for IgG lambda paraprotein.<br>● Family History: No polyneuropathy.<br>Step 2: Identify Potential Causes and Differential Diagnoses<br>● Primary Differential Diagnoses:<br>1. MGUS-related neuropathy<br>2. CIDP<br>3. MADSAM (Lewis-Sumner Syndrome)<br>Step 3: Determine Necessary Additional Examinations<br>● Objective: Narrow down differential diagnoses and determine the specific cause of polyneuropathy.<br>Proposed Additional Examinations<br>1. Bone Marrow Biopsy and Serum Free Light Chain Assay<br>○ Reasoning: To evaluate for multiple myeloma or other plasma cell dyscrasias that may be associated with MGUS.<br>○ Details: A bone marrow biopsy can confirm the presence and extent of plasma cell infiltration. Serum free light chain assay helps quantify the monoclonal protein burden.<br>2. Cerebrospinal Fluid (CSF) Analysis<br>○ Reasoning: To look for evidence of inflammation or infection that could support a diagnosis of CIDP or other immune-mediated neuropathies.<br>○ Details: CSF analysis may show elevated protein levels without pleocytosis in CIDP. Oligoclonal bands could also be assessed.<br>3. MRI of the Spine with Contrast<br>○ Reasoning: To assess for nerve root enhancement which is often seen in CIDP and MADSAM.<br>○ Details: MRI can reveal hypertrophy or enhancement of nerve roots, supporting the diagnosis of an inflammatory neuropathy.<br>4. Quantitative Sensory Testing (QST)<br>○ Reasoning: To provide a detailed assessment of sensory nerve function, which can help differentiate between different types of neuropathies.<br>○ Details: QST can quantify the degree and pattern of sensory deficits, aiding in the characterization of the neuropathy.<br>5. Autoimmune Panel (Extended)<br>○ Reasoning: To rule out autoimmune causes that were not covered in the initial screening.<br>○ Details: Includes anti-GM1, anti-MAG, and anti-ganglioside antibodies, which can be associated with various types of immune-mediated neuropathies.<br>6. Electromyography (EMG)<br>○ Reasoning: To complement nerve conduction studies by assessing muscle response to nerve stimulation and identifying patterns typical of demyelinating vs. axonal neuropathies.<br>○ Details: EMG can provide detailed information about muscle and nerve function, helping to differentiate between different neuropathic processes.<br>Most Crucial Test<br>Bone Marrow Biopsy and Serum Free Light Chain Assay<br>● Reasoning: Given the patient's history of IgG lambda monoclonal gammopathy, it is crucial to determine whether this represents a benign MGUS or if it is indicative of a more serious plasma cell disorder such as multiple myeloma. This test will provide definitive information on the clonal proliferation of plasma cells, which can significantly impact the management and prognosis of the patient's condition. It directly addresses the most likely underlying cause of the patient's symptoms and can help differentiate between MGUS-related neuropathy and other potential causes like CIDP or MADSAM.<br>Conclusion<br>In this comprehensive analysis, the bone marrow biopsy and serum free light chain assay are the most crucial tests to perform next. This approach ensures a targeted and effective evaluation to confirm or rule out the most probable diagnosis and guide appropriate treatment strategies.
90	A 64-year-old male patient presents with a predominantly motor, asymmetrical, non-length dependent polyneuropathy with acute onset that has progressed in the past three months. The patient also reports the presence of neuropathic pain and postural hand tremor. Clinical examination does not reveal ataxia, foot deformities, cranial nerve or autonomic involvement. Past medical history is positive for diabetes. Nerve conduction studies show demyelinating, pure motor polyneuropathy with conduction blocks. Screening lab tests including vitamin B12, folate, vitamin B6, vitamin E, HBV, HCV, ANA, ANCA, ENA, CRP, ESR, glycemia, HbA1c, copper, and cryoglobulins are all negative. Serum electrophoresis reveals the presence of IgM monoclonal gammopathy. There is no family history of polyneuropathy.	Acute onset CIDP	Step-by-Step Analysis of Additional Examinations<br>1. Serum Immunofixation Electrophoresis (IFE)<br>● Reasoning: To further characterize the IgM monoclonal gammopathy found in serum electrophoresis. Immunofixation can help differentiate between benign and malignant monoclonal gammopathies and provide insight into the potential underlying disorder (e.g., MGUS, Waldenström's macroglobulinemia, multiple myeloma).<br>● Impact: Precise identification of the monoclonal protein type is crucial for diagnosis and management.<br>2. Bone Marrow Biopsy<br>● Reasoning: To evaluate for hematologic malignancies such as multiple myeloma or Waldenström's macroglobulinemia, which are associated with IgM monoclonal gammopathy. Bone marrow biopsy can reveal plasma cell dyscrasia or lymphoproliferative disorders.<br>● Impact: Helps confirm or rule out malignancies, guiding further treatment.<br>3. Nerve Biopsy<br>● Reasoning: To assess the histopathological characteristics of the peripheral nerves, which can help differentiate between CIDP, paraproteinemic neuropathy, and other demyelinating neuropathies. It can also provide evidence of vasculitis, amyloid deposition, or other specific pathology.<br>● Impact: Direct tissue examination can reveal the underlying pathological process affecting the nerves.<br>4. CSF Analysis (Lumbar Puncture)<br>● Reasoning: To check for elevated protein levels without pleocytosis, which is typical in CIDP. The presence of monoclonal proteins in the CSF could also support the diagnosis of a paraproteinemic neuropathy or hematologic malignancy.<br>● Impact: Can help distinguish between CIDP and other inflammatory or malignant neuropathies.<br>5. PET-CT Scan<br>● Reasoning: To identify systemic involvement, lymphadenopathy, or hidden malignancies associated with paraproteinemic neuropathies. It provides detailed imaging that can reveal lymphoproliferative disorders or metastatic disease.<br>● Impact: Provides a comprehensive evaluation of systemic involvement, aiding in diagnosis and staging of malignancies.<br>6. Anti-MAG Antibodies Test<br>● Reasoning: To test for the presence of anti-myelin-associated glycoprotein (MAG) antibodies, which are often found in IgM paraproteinemic neuropathies. Positive anti-MAG antibodies would strongly support the diagnosis of a paraproteinemic neuropathy.<br>● Impact: Specific for confirming paraproteinemic neuropathy associated with IgM monoclonal gammopathy.

			7. FISH (Fluorescence In Situ Hybridization) Analysis<br>● Reasoning: To detect genetic abnormalities associated with hematologic malignancies such as multiple myeloma and Waldenström's macroglobulinemia. It helps identify specific chromosomal abnormalities or translocations.<br>● Impact: Provides detailed genetic information to guide diagnosis and treatment planning.<br>8. Quantitative Immunoglobulins and Free Light Chains Assay<br>● Reasoning: To measure levels of immunoglobulins and free light chains, which can provide information about the monoclonal protein production and potential underlying hematologic disorders.<br>● Impact: Helps in the assessment of disease burden and monitoring response to treatment.<br>Most Crucial Test<br>Serum Immunofixation Electrophoresis (IFE) is the most crucial test.<br>● Reasoning: While other tests are important, IFE is essential because it will provide a definitive characterization of the monoclonal protein found on the initial serum electrophoresis. This information is pivotal in confirming the presence and type of monoclonal gammopathy, which is central to diagnosing and managing conditions like MGUS, Waldenström's macroglobulinemia, or multiple myeloma. Without this step, further specific diagnoses cannot be accurately pursued.<br>By conducting the IFE, we can narrow down the differential diagnosis significantly and decide on the next appropriate steps, such as a bone marrow biopsy or anti-MAG antibodies test, based on the findings.
91	A 78-year-old male patient presents with a 1-year history of a sensory-motor, symmetrical, length dependent polyneuropathy with insidious onset and progressive course. The patient presents with sensory ataxia, hand tremor, weakness of ankle dorsiflexion, and distal leg paraesthesia. He also reports mild neuropathic pain in the feet. Clinical examination does not reveal foot deformities, autonomic or cranial nerve involvement. Past medical history is positive for type 2 diabetes, IgM kappa monoclonal gammopathy, and Waldenström macroglobulinemia. Nerve conduction studies show a demyelinating, sensory-motor, length dependent polyneuropathy. Screening lab tests shows hyperglycaemia, elevated HbA1c, and IgM monoclonal gammopathy. Remaining exams including vitamin B12, folate, vitamin B6, vitamin E, HCV, ANA, ENA, ANCA, ENA, CRP, copper, and cryoglobulins are negative. There is no family history of polyneuropathy.	Anti-MAG neuropathy	Step-by-Step Analysis and Additional Examinations<br>Step 1: Review the Clinical Summary<br>● Patient: 78-year-old male<br>● Symptoms: Sensory-motor, symmetrical, length-dependent polyneuropathy with sensory ataxia, hand tremor, weakness of ankle dorsiflexion, distal leg paraesthesia, and mild neuropathic pain in the feet.<br>● Medical History: Type 2 diabetes, IgM kappa monoclonal gammopathy, Waldenström macroglobulinemia.<br>● Nerve Conduction Studies: Demyelinating, sensory-motor, length-dependent polyneuropathy.<br>● Lab Findings: Hyperglycaemia, elevated HbA1c, IgM monoclonal gammopathy; negative for various vitamin deficiencies, infections, and autoimmune markers.<br>Step 2: Identify Additional Examinations<br>To further narrow down the differential diagnoses (Diabetic Polyneuropathy, MGUS Neuropathy, and CIDP), the following additional examinations are necessary:<br>1. Serum Protein Electrophoresis (SPEP) and Immunofixation Electrophoresis (IFE):<br>○ Reasoning: These tests will further characterize the monoclonal gammopathy and determine the specific type and amount of monoclonal protein. This is crucial for differentiating between MGUS and more severe plasma cell disorders such as multiple myeloma or Waldenström macroglobulinemia-related neuropathy.<br>2. Lumbar Puncture with Cerebrospinal Fluid (CSF) Analysis:<br>○ Reasoning: CSF analysis can help identify elevated protein levels without pleocytosis, which is indicative of CIDP. This can differentiate CIDP from other causes of demyelinating polyneuropathy.<br>3. Anti-MAG Antibodies:<br>○ Reasoning: Anti-myelin-associated glycoprotein (MAG) antibodies are often present in patients with IgM paraproteinemic neuropathy, particularly those related to Waldenström macroglobulinemia. This can help confirm the diagnosis of MGUS neuropathy.<br>4. Skin Biopsy:<br>○ Reasoning: A skin biopsy can help assess small fiber neuropathy, which might not be detected by nerve conduction studies. This is particularly useful if there is a suspicion of small fiber involvement in diabetic neuropathy.<br>5. Oral Glucose Tolerance Test (OGTT):<br>○ Reasoning: This test can provide a more detailed assessment of glucose metabolism, potentially identifying any glucose intolerance or undiagnosed diabetes, which can contribute to neuropathy.<br>6. Electromyography (EMG):<br>○ Reasoning: EMG can help further characterize the neuropathy, distinguishing between axonal and demyelinating processes and identifying any secondary muscle involvement.<br>7. Autonomic Function Testing:<br>○ Reasoning: Even though there is no apparent autonomic involvement on initial examination, detailed autonomic testing can uncover subtle autonomic dysfunction, which may be relevant in diabetic neuropathy or CIDP.<br>8. Serum Free Light Chain Assay:<br>○ Reasoning: This test can help assess for light chain deposition diseases and further characterize the monoclonal gammopathy, which is useful in differentiating MGUS from more significant hematologic disorders.<br>Step 3: Determine the Most Crucial Test<br>Among the listed additional examinations, the most crucial test for this patient would be:<br>Lumbar Puncture with Cerebrospinal Fluid (CSF) Analysis<br>Reasoning:<br>● Direct Assessment of Demyelination: CSF analysis can reveal elevated protein levels without pleocytosis, which is highly indicative of CIDP.<br>● Differentiation from Other Causes: It provides direct evidence to differentiate CIDP from other causes of demyelinating neuropathy, such as diabetic polyneuropathy and MGUS-related neuropathy.<br>● Impact on Treatment: A definitive diagnosis of CIDP would significantly influence treatment decisions, potentially leading to immunomodulatory therapies that can alter the disease course.<br>Conclusion<br>The comprehensive analysis of the patient's clinical presentation and medical history suggests that further testing is essential to narrow down the differential diagnoses. Among the proposed additional examinations, lumbar puncture with cerebrospinal fluid analysis is the most crucial, as it provides critical information to confirm or exclude CIDP, directly impacting management and therapeutic approaches.
92	A 58-year-old female patient presents with a 3-month history of sensory-motor, symmetrical, non-length dependent polyneuropathy with acute onset and progressive course. Two weeks after an infectious event, the patient reports the appearance of paraesthesia and weakness in the hands and lower limbs. At nadir she was unable to walk. The symptoms were interpreted as Guillain Barré syndrome and the patient received a course of immunoglobulin therapy with clear benefit.	Acute onset CIDP	Step-by-Step Analysis<br>1. Initial Clinical Summary:<br>● 58-year-old female with a 3-month history of sensory-motor, symmetrical, non-length dependent polyneuropathy.<br>● Symptoms began acutely two weeks post-infection and were progressive.<br>● Initial improvement with immunoglobulin therapy but relapse of symptoms three months later.<br>● Neurological examination revealed proximal and distal weakness in lower limbs, distal weakness in upper limbs, and sensory ataxia.<br>● Nerve conduction studies indicated demyelinating, sensory-motor polyneuropathy.<br>● Extensive initial screening lab tests were negative.<br>2. Differential Diagnoses:<br>● Chronic Inflammatory Demyelinating Polyneuropathy (CIDP)<br>● Multifocal Acquired Demyelinating Sensory and Motor Neuropathy (MADSAM)<br>● Paraneoplastic Neuropathy

	After the therapy she started walking again with some difficulty. About three months after the onset of symptoms, she noticed the reappearance of the paresthesia and a worsening of her strength, so she started using a crutch. Neurological examination shows the presence of weakness, distributed proximally and distally in the lower limbs and distally in the upper limbs, and sensory ataxia. Past medical history is silent. Nerve conduction studies show a demyelinating, sensory-motor polyneuropathy. Screening lab tests including vitamin B12, folate, vitamin B6, vitamin E, HBV, HCV, ANA, ANCA, ENA, CRP, ESR, glycemia, HbA1c, copper, cryoglobulins, and serum electrophoresis are negative. There is no family history of polyneuropathy.		**Additional Examinations**<br>**Laboratory Tests:**<br>**1. Lumbar Puncture (CSF Analysis):**<br>○ **Reasoning: Elevated protein with normal white cell count (albuminocytologic dissociation) is typical in CIDP. This test can help distinguish between CIDP and other types of neuropathies, including paraneoplastic syndromes.**<br>**2. Anti-GM1, Anti-GQ1b, and Other Ganglioside Antibodies:**<br>○ **Reasoning: These antibodies are often elevated in immune-mediated neuropathies, including CIDP and its variants. They can provide supportive evidence for an autoimmune etiology.**<br>**3. Anti-MAG Antibodies:**<br>○ **Reasoning: Myelin-associated glycoprotein (MAG) antibodies are associated with demyelinating neuropathies and can help identify specific subtypes of immune-mediated neuropathies.**<br>**4. Onconeural Antibodies (e.g., anti-Hu, anti-Yo, anti-Ri):**<br>○ **Reasoning: These antibodies are often elevated in paraneoplastic neuropathies. Testing for these can help rule out a paraneoplastic process.**<br>**5. Repeat Comprehensive Metabolic Panel and Thyroid Function Tests:**<br>○ **Reasoning: To rule out metabolic causes and thyroid dysfunction that might not have been captured initially.**<br>**Instrumental Tests:**<br>**1. Repeat Nerve Conduction Studies (NCS) and Electromyography (EMG):**<br>○ **Reasoning: Repeating these studies can provide updated information on the progression of demyelination and axonal damage, and can help differentiate between CIDP and other neuropathies.**<br>**2. Magnetic Resonance Imaging (MRI) of the Spine and Brain:**<br>○ **Reasoning: MRI can reveal nerve root or spinal cord lesions, which are common in CIDP. Enhancement of nerve roots can support a diagnosis of CIDP.**<br>**3. Chest, Abdominal, and Pelvic CT or PET-CT Scan:**<br>○ **Reasoning: To rule out an underlying malignancy that could be causing a paraneoplastic syndrome, particularly since the initial screening was negative.**<br>**4. Skin Biopsy:**<br>○ **Reasoning: To evaluate for small fiber neuropathy, which can sometimes be missed on NCS/EMG. It can provide information on nerve fiber density and help identify immune-mediated neuropathies.**<br>**Most Crucial Test**<br>**Lumbar Puncture (CSF Analysis)**<br>● **Reasoning: This test is crucial for its ability to detect albuminocytologic dissociation, which is highly suggestive of CIDP. Given the patient's clinical presentation, history of response to immunoglobulin therapy, and relapsing nature of the symptoms, confirming this finding can significantly narrow down the diagnosis to CIDP. It is also less invasive and more straightforward compared to some of the other suggested tests like biopsies or advanced imaging.**<br>**Conclusion**<br>**While all the proposed additional tests are valuable, the Lumbar Puncture (CSF Analysis) stands out as the most crucial. It directly assesses for the characteristic features of CIDP and can substantially aid in confirming the diagnosis, thereby guiding further treatment and management strategies.**
93	A 42-year-old female patient presents with a 6-month history of chronic, sensory-motor, symmetrical, non-length dependent polyneuropathy with subacute onset. The patient presents with paresthesia and severe neuropathic pain in the four limbs in a stocking and glove distribution and sensory ataxia. Clinical examination reveals the presence of postural hand tremor, which the patient reports being present for years. Thereare no foot deformities, cranial nerve or autonomic involvement. Past medical history is positive for rectal cancer, for which the patient has undergone chemotherapy for three years. Neuropathy symptoms started during chemotherapy. Nerve conduction studies show sensory-motor, symmetric, axonal polyneuropathy. Screening lab tests including vitamin B6, vitamin E, HBV, HCV, ANA, ANCA, ENA, CRP, ESR, glycemia, HbA1c, copper, cryoglobulins and serum electrophoresis are all negative, with evidence of vitamin B12 deficiency. There is no family history of polyneuropathy.	Chemotherapy-induced neuropathy	**Step-by-Step Analysis of Comprehensive Data**<br>**1. Review Current Data:**<br>○ **Symptoms: Chronic, sensory-motor, symmetrical, non-length dependent polyneuropathy with subacute onset; paresthesia, severe neuropathic pain, sensory ataxia.**<br>○ **Physical Exam: Postural hand tremor, no foot deformities, no cranial nerve or autonomic involvement.**<br>○ **History: Rectal cancer treated with chemotherapy; symptoms started during chemotherapy.**<br>○ **Nerve Conduction Studies: Sensory-motor, symmetric, axonal polyneuropathy.**<br>○ **Lab Results: Vitamin B12 deficiency, other screenings negative.**<br>○ **Family History: No family history of polyneuropathy.**<br>**2. Determine Additional Examinations:**<br>○ **Based on the current clinical picture, it is crucial to conduct additional examinations to further narrow down the differential diagnoses.**<br>**Additional Examinations**<br>**1. Complete Blood Count (CBC) and Peripheral Blood Smear**<br>○ **Reasoning:**<br>■ **Evaluate for hematologic disorders that could contribute to neuropathy, such as myelodysplastic syndromes or leukemia.**<br>■ **Assess overall health and detect any abnormalities in blood cells that might indicate other underlying conditions.**<br>**2. Lumbar Puncture (Cerebrospinal Fluid Analysis)**<br>○ **Reasoning:**<br>■ **Check for elevated protein levels, which can indicate an inflammatory or demyelinating process (e.g., Guillain-Barré syndrome or chronic inflammatory demyelinating polyneuropathy).**<br>■ **Exclude central nervous system involvement, which could present with similar symptoms.**<br>**3. Autoimmune Panel (Extended)**<br>○ **Reasoning:**<br>■ **Further test for autoimmune neuropathies. Include tests for paraneoplastic antibodies not covered by initial screening, such as anti-Hu, anti-Ri, anti-Yo.**<br>■ **Paraneoplastic syndromes may present with negative standard autoimmune tests but can still be seropositive for specific paraneoplastic antibodies.**<br>**4. Electromyography (EMG)**<br>○ **Reasoning:**<br>■ **Confirm the axonal nature of the neuropathy.**<br>■ **Differentiate between axonal and demyelinating processes.**<br>■ **Provide detailed information on the extent and distribution of the neuropathy.**<br>**5. Skin Biopsy for Intraepidermal Nerve Fiber Density**<br>○ **Reasoning:**<br>■ **Assess small fiber neuropathy, which might not be evident on standard nerve conduction studies.**<br>■ **Provide additional information about the sensory component of the neuropathy.**<br>**6. Quantitative Sensory Testing (QST)**

			○ **Reasoning:**<br>■ **Assess sensory nerve function more precisely.**<br>■ **Help determine the extent of sensory involvement and distinguish between different types of sensory neuropathy.**<br>7. **Magnetic Resonance Imaging (MRI) of the Spine**<br>○ **Reasoning:**<br>■ **Rule out any structural causes of neuropathy, such as spinal cord compression or tumors.**<br>■ **Evaluate for other possible neurological conditions affecting the spine and nerve roots.**<br>8. **Serum Protein Electrophoresis (SPEP) and Immunofixation Electrophoresis (IFE)**<br>○ **Reasoning:**<br>■ **Detect monoclonal gammopathies, such as multiple myeloma or POEMS syndrome, which can present with neuropathy.**<br>■ **Identify other protein abnormalities that could contribute to neuropathic symptoms.**<br>**Determining the Most Crucial Test**<br>**Lumbar Puncture (Cerebrospinal Fluid Analysis)**<br>● **Reasoning:**<br>○ **This test provides critical information about the presence of inflammatory or infectious processes in the central nervous system that could contribute to the neuropathy.**<br>○ **Elevated protein levels or the presence of specific antibodies in the cerebrospinal fluid can confirm or rule out several differential diagnoses, including Guillain-Barré syndrome, chronic inflammatory demyelinating polyneuropathy (CIDP), and paraneoplastic syndromes.**<br>○ **The lumbar puncture can reveal whether the neuropathy is part of a more extensive neuro-inflammatory process, guiding further management and treatment.**
94	A 73-year-old female patient presents with a 1-year history of pure sensory, symmetrical, length dependent polyneuropathy with insidious onset. The patient presents with paresthesia, neuropathic pain and sensory ataxia without weakness, cranial nerve or autonomic involvement. There are also no foot deformities or tremor. Past medical history is positive for sarcoidosis. Nerve conduction studies show pure a pure sensory, axonal polyneuropathy. Screening lab tests including vitamin B12, folate, vitamin B6, vitamin E, HCV, HBV, ANCA, ENA, CRP, ESR, glycemia, HbA1c, copper, cryoglobulins and serum electrophoresis are all negative, with positivity for ANA autoantibodies. There is no family history of polyneuropathy.	Sarcoidosis associated polyneuropathy	**Step-by-Step Analysis and Recommendations for Additional Examinations**<br>**Summary of Pertinent Clinical Information**<br>● **Patient Profile:**<br>○ **Age: 73-year-old**<br>○ **Sex: Female**<br>● **Presenting Symptoms:**<br>○ **Duration: 1 year**<br>○ **Type: Pure sensory, symmetrical, length-dependent polyneuropathy**<br>○ **Onset: Insidious**<br>○ **Symptoms: Paresthesia, neuropathic pain, sensory ataxia**<br>○ **Absent Symptoms: Weakness, cranial nerve involvement, autonomic involvement, foot deformities, tremor**<br>● **Past Medical History:**<br>○ **Sarcoidosis**<br>● **Investigations:**<br>○ **Nerve Conduction Studies: Pure sensory, axonal polyneuropathy**<br>○ **Screening Lab Tests:**<br>■ **Negative Results: Vitamin B12, folate, vitamin B6, vitamin E, HCV, HBV, ANCA, ENA, CRP, ESR, glycemia, HbA1c, copper, cryoglobulins, serum electrophoresis**<br>■ **Positive Result: ANA autoantibodies**<br>● **Family History: No family history of polyneuropathy**<br>**Additional Examinations**<br>**1. Anti-Ro (SSA) and Anti-La (SSB) Antibodies**<br>**Reasoning:**<br>● **Association with ANA Positivity: These antibodies are often associated with ANA positivity and can be indicative of Sjögren's syndrome, which can present with a sensory neuropathy.**<br>● **Relevance to Symptoms: Sjögren's syndrome frequently presents with sensory neuropathy, and considering the patient's ANA positivity, it is important to rule out this condition.**<br>**2. Nerve Biopsy**<br>**Reasoning:**<br>● **Detailed Pathological Insight: A nerve biopsy can provide detailed pathological information about the type and extent of nerve damage, which can help differentiate between various causes of neuropathy.**<br>● **Sarcoidosis Confirmation: It can confirm or rule out sarcoid granulomas in the nerve tissue, which would support a diagnosis of sarcoidosis-related neuropathy.**<br>**3. Cerebrospinal Fluid (CSF) Analysis**<br>**Reasoning:**<br>● **Inflammatory Markers: CSF analysis can reveal elevated protein levels and the presence of inflammatory cells, which might indicate an autoimmune or inflammatory neuropathy.**<br>● **Exclusion of Other Causes: It can help exclude other potential causes like infections or paraneoplastic syndromes that might present with similar neuropathic symptoms.**<br>**4. Serum Immunofixation Electrophoresis**<br>**Reasoning:**<br>● **Monoclonal Gammopathy Detection: This test is more sensitive than standard serum electrophoresis and can detect monoclonal gammopathies, which can be associated with neuropathy, particularly in elderly patients.**<br>● **Relevance: Despite a negative serum electrophoresis, a more sensitive test is warranted given the age of the patient and the unexplained neuropathy.**<br>**5. Thyroid Function Tests**<br>**Reasoning:**<br>● **Common Cause of Neuropathy: Hypothyroidism can cause a sensory neuropathy and is relatively common in older adults.**<br>● **Symptom Overlap: While the patient's symptoms are not overtly suggestive of hypothyroidism, it is a common and easily treatable cause that should be ruled out.**<br>**Most Crucial Test**<br>**Most Crucial Test: Anti-Ro (SSA) and Anti-La (SSB) Antibodies**<br>**Detailed Reasoning:**<br>● **Autoimmune Connection: Given the patient's ANA positivity and history of sarcoidosis (another autoimmune condition), it is critical to explore other potential autoimmune causes. Sjögren's syndrome is a common cause of sensory neuropathy associated with ANA positivity.**

			● **Impact on Management: Identifying Sjögren's syndrome would directly impact the management and treatment plan for the patient, possibly leading to targeted immunosuppressive therapy.**<br>**Conclusion**<br>**To narrow down the differential diagnoses for the patient's polyneuropathy, the most crucial test to conduct is the evaluation for Anti-Ro (SSA) and Anti-La (SSB) antibodies. This test is pivotal in identifying Sjögren's syndrome, which is a plausible and treatable cause of the patient's symptoms, given the ANA positivity and the clinical presentation.**
95	A 62-year-old male patient presents with a 5-month history of sensory-motor, symmetrical, length dependent polyneuropathy with subacute onset. The patient presents with distal paresthesia and neuropathic pain in the lower limbs, associated with weakness of toe dorsiflexion. Clinical examination also reveals the presence of orthostatic hypotension. There are no sensory ataxia, tremor, foot deformities or cranial nerve involvement. Past medical history is positive for diabetes since the age of 50, and plasmacytoma, for which the patient has undergone chemotherapy. Symptoms started during treatment with chemotherapy. Nerve conduction studies show a mixed sensory-motor, symmetric, axonal polyneuropathy. Screening lab tests including vitamin B12, folate, vitamin B6, vitamin E, HBV, HCV, ANA, ANCA, ENA, CRP, ESR, copper, cryoglobulins and serum electrophoresis are all negative, with evidence of hyperglycaemia and elevated HbA1c. There is no family history of polyneuropathy.	Chemotherapy-induced neuropathy	**Step-by-Step Analysis for Additional Examinations**<br>**Review of Clinical Information:**<br>● **Patient Demographics:**<br>○ **62-year-old male**<br>● **History of Present Illness:**<br>○ **5-month history of sensory-motor, symmetrical, length-dependent polyneuropathy with subacute onset**<br>○ **Distal paresthesia, neuropathic pain in lower limbs, weakness in toe dorsiflexion**<br>○ **Orthostatic hypotension**<br>● **Clinical Examination Findings:**<br>○ **No sensory ataxia, tremor, foot deformities, or cranial nerve involvement**<br>● **Past Medical History:**<br>○ **Diabetes mellitus since age 50**<br>○ **Plasmacytoma treated with chemotherapy**<br>● **Onset of Symptoms:**<br>○ **During chemotherapy treatment**<br>● **Nerve Conduction Studies:**<br>○ **Mixed sensory-motor, symmetric, axonal polyneuropathy**<br>● **Screening Lab Tests:**<br>○ **Negative for multiple conditions, hyperglycaemia, and elevated HbA1c**<br>● **Family History:**<br>○ **No family history of polyneuropathy**<br>**Additional Examinations:**<br>**1. Complete Blood Count (CBC) and Comprehensive Metabolic Panel (CMP):**<br>○ **Reasoning:**<br>■ **A CBC can help rule out anemia or other hematological conditions that might contribute to neuropathy.**<br>■ **A CMP can assess liver and kidney function, which can also impact nerve health.**<br>**2. Electromyography (EMG):**<br>○ **Reasoning:**<br>■ **EMG can provide further detail about the nature and distribution of the neuropathy, distinguishing between axonal and demyelinating processes, and assessing the extent of muscle involvement.**<br>**3. Lumbar Puncture (CSF Analysis):**<br>○ **Reasoning:**<br>■ **CSF analysis can help rule out inflammatory or infectious causes of neuropathy, such as Guillain-Barré syndrome or chronic inflammatory demyelinating polyneuropathy (CIDP).**<br>**4. Quantitative Sensory Testing (QST):**<br>○ **Reasoning:**<br>■ **QST can objectively assess sensory nerve function, providing detailed information on the severity and type of sensory involvement.**<br>**5. Autonomic Testing (e.g., Tilt Table Test, Heart Rate Variability):**<br>○ **Reasoning:**<br>■ **Given the orthostatic hypotension, autonomic testing can help assess the extent of autonomic nervous system involvement, which is crucial in both diabetic and chemotherapy-induced neuropathy.**<br>**6. Skin Biopsy for Intraepidermal Nerve Fiber Density (IENFD):**<br>○ **Reasoning:**<br>■ **A skin biopsy can quantify small nerve fiber density, which is useful in diagnosing small fiber neuropathy often seen in diabetes and chemotherapy-induced neuropathy.**<br>**7. Paraneoplastic Panel:**<br>○ **Reasoning:**<br>■ **Since the patient has a history of plasmacytoma, testing for paraneoplastic antibodies can help identify if the neuropathy is paraneoplastic in origin.**<br>**8. Serum Protein Electrophoresis (SPEP) and Immunofixation Electrophoresis (IFE):**<br>○ **Reasoning:**<br>■ **Although previous tests showed negative results, repeating these tests can rule out the presence of monoclonal gammopathy, which can be associated with neuropathy in the context of a hematological malignancy.**<br>**Most Crucial Test:**<br>**Electromyography (EMG):**<br>● **Reasoning:**<br>○ **EMG is crucial as it will provide detailed information on the type and extent of nerve and muscle involvement, distinguishing between axonal and demyelinating processes. This distinction is key to differentiating between diabetic polyneuropathy, chemotherapy-induced neuropathy, and other potential causes such as paraneoplastic neuropathy.**<br>○ **EMG results will guide further diagnostic steps and therapeutic approaches.**<br>**Conclusion:**<br>**Conducting the EMG should be prioritized to gain detailed insights into the neuropathy's characteristics, helping to narrow down the differential diagnoses and guide further targeted investigations. This test will provide essential information about the nerve and muscle involvement, which is critical for a more precise diagnosis and appropriate management plan.**
96	An 86-year-old male patient presents with a 1-year history of sensory-motor,	CIDP	**Step-by-Step Analysis and Additional Examinations**<br>**Step 1: Review Existing Data**

	symmetrical, non-length dependent polyneuropathy with subacute onset and progressive course. The patient presents with distal paresthesia in the upper limbs and sensory ataxia with weakness of ankle dorsiflexion. Clinical examination reveals mild hand tremor. There are no neuropathic pain, foot deformities, cranial nerve or autonomic involvement. Past medical history shows IgG kappa monoclonal gammopathy and basal cell skin cancer. Nerve conduction studies show a demyelinating, sensory-motor polyneuropathy. Screening lab tests including vitamin B12, folate, vitamin B6, vitamin E, HCV, ANA, ANCA, ENA, CRP, ESR, glycemia, HbA1c, copper and cryoglobulins are all negative, with evidence of chronic HBV infection and positivity for IgG kappa paraprotein. There is no family history of polyneuropathy.		● **Symptoms: Sensory-motor, symmetrical, non-length dependent polyneuropathy; distal paresthesia in the upper limbs; sensory ataxia; weakness of ankle dorsiflexion; mild hand tremor.**<br>● **Past Medical History: IgG kappa monoclonal gammopathy; basal cell skin cancer.**<br>● **Investigations: Demyelinating, sensory-motor polyneuropathy on nerve conduction studies; comprehensive screening negative for many common causes; chronic HBV infection and IgG kappa paraprotein present.**<br>**Step 2: Identify Additional Necessary Examinations**<br>**1. Serum Protein Electrophoresis (SPEP) and Immunofixation Electrophoresis (IFE)**<br>● **Reasoning: These tests will help quantify and characterize the monoclonal protein present. SPEP and IFE can differentiate MGUS from multiple myeloma or other plasma cell disorders, which can also cause neuropathy.**<br>**2. Bone Marrow Biopsy**<br>● **Reasoning: Since the patient has IgG kappa monoclonal gammopathy, a bone marrow biopsy can determine if there is underlying plasma cell dyscrasia, such as multiple myeloma, which might contribute to the neuropathy.**<br>**3. Cerebrospinal Fluid (CSF) Analysis**<br>● **Reasoning: Lumbar puncture and CSF analysis can help identify inflammatory markers or elevated protein levels indicative of CIDP. The presence of oligoclonal bands can also be assessed.**<br>**4. Anti-MAG Antibodies and Other Anti-Neuronal Antibodies**<br>● **Reasoning: Testing for anti-myelin-associated glycoprotein (MAG) antibodies can help diagnose demyelinating neuropathy associated with MGUS. Additional anti-neuronal antibodies can screen for other paraneoplastic syndromes.**<br>**5. Magnetic Resonance Imaging (MRI) of the Spine**<br>● **Reasoning: MRI can assess for structural causes of neuropathy, such as spinal cord compression or lesions that could explain the symptoms.**<br>**6. Quantitative Sensory Testing (QST)**<br>● **Reasoning: QST can help evaluate the sensory deficits in more detail, distinguishing between large and small fiber involvement, which can guide the differential diagnosis further.**<br>**7. Repeat Nerve Conduction Studies (NCS) and Electromyography (EMG)**<br>● **Reasoning: Repeating these studies can monitor the progression and characterize the neuropathy in more detail, including distinguishing between axonal and demyelinating features more precisely.**<br>**Proposed Tests and Their Detailed Reasoning**<br>1. **Serum Protein Electrophoresis (SPEP) and Immunofixation Electrophoresis (IFE)**<br>○ **These tests will help characterize the monoclonal protein and determine if it's linked to more severe conditions like multiple myeloma.**<br>2. **Bone Marrow Biopsy**<br>○ **Important for identifying or ruling out underlying plasma cell dyscrasias, which might significantly alter the patient's treatment and prognosis.**<br>3. **Cerebrospinal Fluid (CSF) Analysis**<br>○ **CSF analysis will help identify CIDP by looking for elevated protein levels or inflammatory markers, which are not typically seen in MGUS alone.**<br>4. **Anti-MAG Antibodies and Other Anti-Neuronal Antibodies**<br>○ **Testing for these antibodies can confirm a diagnosis of MGUS-related neuropathy and exclude other paraneoplastic neuropathies.**<br>5. **Magnetic Resonance Imaging (MRI) of the Spine**<br>○ **MRI can reveal any structural abnormalities in the spine that might contribute to neuropathy, helping to exclude compressive or neoplastic causes.**<br>6. **Quantitative Sensory Testing (QST)**<br>○ **QST provides a detailed sensory profile that can differentiate between types of nerve fiber involvement, which is crucial for diagnosis.**<br>7. **Repeat Nerve Conduction Studies (NCS) and Electromyography (EMG)**<br>○ **These tests will help in assessing the progression and type of neuropathy more accurately over time, helping to refine the diagnosis.**<br>**Most Crucial Test**<br>**The most crucial test from this list is the Serum Protein Electrophoresis (SPEP) and Immunofixation Electrophoresis (IFE). This test will provide critical information about the monoclonal protein present, which is directly related to the patient's known IgG kappa monoclonal gammopathy. It will help distinguish between benign MGUS and more severe plasma cell disorders such as multiple myeloma, thereby significantly influencing the diagnostic and therapeutic approach.**
97	A 57-year-old male patient presents with a 1-year history of sensory-motor, asymmetric, non-length dependent polyneuropathy with subacute onset and progressive course. The patient presents with weakness and paresthesia in the proximal and distal areas of the upper limbs in an asymmetric distribution. Clinical examination excludes the presence of ataxia, tremor, neuropathic pain, foot deformities, cranial nerve or autonomic involvement. Past medical history is silent. Nerve conduction studies show a demyelinating, sensory-motor multineuropathy. Screening lab tests reveal the presence of folate deficiency. Remaining exams including vitamin B12, vitamin B6, vitamin E, HBV, HCV, ANA, ANCA, ENA, CRP, ESR, glycemia, HbA1c, copper, cryoglobulins and serum electrophoresis are all negative. There is no family history of polyneuropathy.	CIDP	**Step-by-Step Analysis and Additional Examinations**<br>**To narrow down the differential diagnoses and arrive at a definitive diagnosis for the patient's peripheral neuropathy, the following additional examinations should be conducted:**<br>**1. Cerebrospinal Fluid (CSF) Analysis**<br>**Reasoning:**<br>● **CIDP Diagnostic Support: Elevated CSF protein levels with a normal cell count (albuminocytologic dissociation) are commonly seen in CIDP.**<br>● **Exclusion of Other Inflammatory/Immune Disorders: CSF analysis can help rule out other inflammatory or infectious causes of neuropathy, such as Guillain-Barré syndrome, sarcoidosis, or chronic infections like Lyme disease and HIV.**<br>**2. Serum Protein Electrophoresis with Immunofixation**<br>**Reasoning:**<br>● **Detection of Monoclonal Gammopathies: Conditions such as MGUS (Monoclonal Gammopathy of Undetermined Significance) or multiple myeloma can present with neuropathy. Even though initial serum electrophoresis was negative, immunofixation is more sensitive and can detect smaller monoclonal proteins.**<br>● **Association with Demyelinating Neuropathy: Monoclonal gammopathies are associated with demyelinating neuropathies and should be ruled out.**<br>**3. Anti-GM1 Ganglioside Antibodies**<br>**Reasoning:**<br>● **MMN Diagnostic Support: Anti-GM1 antibodies are often present in Multifocal Motor Neuropathy (MMN). Testing for these antibodies can help differentiate MMN from other neuropathies, especially since MMN can present with demyelinating features.**<br>**4. MRI of the Brachial Plexus**<br>**Reasoning:**<br>● **Structural and Inflammatory Lesions: MRI can detect structural lesions, inflammation, or other abnormalities in the brachial plexus that might explain asymmetric upper limb involvement.**<br>● **CIDP and MMN Evaluation: MRI can also show hypertrophy or enhancement of the brachial plexus in conditions like CIDP or MMN.**<br>**5. Repeat Nerve Conduction Studies with Focus on Proximal Segments**<br>**Reasoning:**<br>● **Detailed Mapping of Nerve Involvement: Repeating and extending nerve conduction studies to include proximal segments can help better characterize the pattern and extent of demyelination.**<br>● **Support for Demyelinating Disorders: More extensive studies can provide further support for diagnoses like CIDP or MMN by demonstrating conduction block or segmental demyelination.**<br>**6. Skin Biopsy with Intraepidermal Nerve Fiber Density (IENFD) Measurement**<br>**Reasoning:**

			● **Small Fiber Neuropathy: Although not the primary suspect, a skin biopsy can help rule out small fiber neuropathy, which may not be detected by standard nerve conduction studies.**<br>● **Assessment of Nerve Fiber Involvement: This can provide additional information on nerve fiber density and integrity, aiding in the differential diagnosis.**<br>**Most Crucial Test**<br>**Cerebrospinal Fluid (CSF) Analysis is the most crucial test from the list.**<br>**Reasoning:**<br>● **High Diagnostic Yield for CIDP: Elevated CSF protein with normal cell count (albuminocytologic dissociation) is a hallmark of CIDP, providing strong support for this diagnosis.**<br>● **Exclusion of Other Conditions: It can help rule out other inflammatory or infectious causes that might present similarly.**<br>● **Guiding Further Management: Positive findings would guide treatment towards immunomodulatory therapy, which is critical for CIDP management.**<br>**By conducting a CSF analysis, we can significantly narrow down the differential diagnoses, particularly in distinguishing CIDP from other potential causes of the patient's neuropathy.**
98	A 74-year-old male patient presents with a 1-month of sensory-motor, multifocal neuropathy with subacute onset and stepwise progression. The patient presents with paresthesia, neuropathic pain, and weakness with a multifocal distribution in the four limbs. He also reports arthralgia and headache, which began in the last two weeks. Clinical examination excludes the presence of sensory ataxia, tremor, foot deformities, cranial nerve or autonomic involvement. Past medical history is positive for arterial hypertension, Hashimoto's thyroiditis, chronic HBV infection, and type 2 diabetes mellitus. Nerve conduction studies show a multifocal, axonal, sensory-motor neuropathy. Screening lab tests reveal elevated glycemia and HbA1c leves, vitamin B12 deficiency, and raised ESR and CRP. Remaining exams including folate, vitamin B6, vitamin E, HCV, ANA, ANCA, ENA, copper, cryoglobulins, and serum electrophoresis are normal. There is no family history of polyneuropathy.	Vasculitic neuropathy	**Step-by-Step Analysis and Additional Examinations**<br>**1. Review Clinical Information**<br>● **74-year-old male with subacute, multifocal, sensory-motor neuropathy.**<br>● **Symptoms: paresthesia, neuropathic pain, weakness, arthralgia, headache.**<br>● **Exam: no sensory ataxia, tremor, foot deformities, cranial nerve, or autonomic involvement.**<br>● **Past medical history: hypertension, Hashimoto's thyroiditis, chronic HBV infection, type 2 diabetes mellitus.**<br>● **Labs: elevated glycemia, HbA1c, vitamin B12 deficiency, raised ESR and CRP.**<br>● **Normal: folate, vitamin B6, vitamin E, HCV, ANA, ANCA, ENA, copper, cryoglobulins, serum electrophoresis.**<br>● **Nerve conduction studies: multifocal, axonal, sensory-motor neuropathy.**<br>● **No family history of polyneuropathy.**<br>**2. Additional Examinations**<br>**A. Laboratory Tests**<br>1. **Anti-neutrophil cytoplasmic antibodies (c-ANCA, p-ANCA)**<br>○ **Reasoning: Despite initial ANCA being normal, specific subtypes (c-ANCA, p-ANCA) can sometimes be more indicative of vasculitis.**<br>○ **Purpose: Detect underlying vasculitis (e.g., granulomatosis with polyangiitis).**<br>2. **Hepatitis C Virus (HCV) Antibody and RNA**<br>○ **Reasoning: Although initial HCV was negative, a re-test or more sensitive testing may be needed since chronic HCV can lead to cryoglobulinemia and vasculitic neuropathy.**<br>○ **Purpose: Exclude HCV-related cryoglobulinemic vasculitis.**<br>3. **Serum Protein Electrophoresis and Immunofixation**<br>○ **Reasoning: To detect monoclonal gammopathies (e.g., multiple myeloma, POEMS syndrome) that can present with neuropathy.**<br>○ **Purpose: Identify monoclonal proteins, amyloidosis, or other plasma cell dyscrasias.**<br>4. **Comprehensive Metabolic Panel (CMP)**<br>○ **Reasoning: Evaluate liver and kidney function, electrolyte imbalances that may contribute to neuropathy.**<br>○ **Purpose: Rule out metabolic causes of neuropathy.**<br>5. **Autoimmune Panel (Rheumatoid factor, Complement levels, Anti-SSA/Ro, Anti-SSB/La, etc.)**<br>○ **Reasoning: Further assess for autoimmune disorders that could cause systemic and peripheral nerve involvement.**<br>○ **Purpose: Identify other autoimmune conditions.**<br>**B. Instrumental Tests**<br>1. **Nerve Biopsy**<br>○ **Reasoning: Direct examination of nerve tissue can identify vasculitis, amyloidosis, or other structural nerve pathologies.**<br>○ **Purpose: Confirm diagnosis of vasculitic neuropathy or other infiltrative processes.**<br>2. **Skin Biopsy with Immunofluorescence**<br>○ **Reasoning: Can reveal small-fiber neuropathy and vasculitis if present.**<br>○ **Purpose: Complement nerve biopsy findings.**<br>3. **MRI of Brain and Spinal Cord**<br>○ **Reasoning: Exclude central nervous system involvement or structural abnormalities that may contribute to symptoms.**<br>○ **Purpose: Rule out CNS causes of neuropathy and systemic symptoms.**<br>4. **Electromyography (EMG)**<br>○ **Reasoning: Further characterize the neuropathy, differentiating between axonal and demyelinating processes.**<br>○ **Purpose: Provide additional details on the type and extent of nerve damage.**<br>5. **Angiography (MR or CT)**<br>○ **Reasoning: Assess for evidence of systemic vasculitis affecting larger vessels.**<br>○ **Purpose: Detect vascular changes consistent with vasculitis.**<br>6. **Lumbar Puncture with CSF Analysis**<br>○ **Reasoning: Evaluate for infectious, inflammatory, or paraneoplastic processes.**<br>○ **Purpose: Analyze CSF for signs of infection, inflammation, or malignancy.**<br>**Most Crucial Test**<br>**Nerve Biopsy**<br>● **Reasoning: Given the presentation of multifocal, axonal neuropathy with systemic inflammatory signs (elevated ESR and CRP), and the suspicion of vasculitis, a nerve biopsy is the most direct and definitive test. It can confirm the presence of vasculitis, amyloidosis, or other infiltrative processes that are difficult to diagnose through non-invasive means.**<br>● **Purpose: Confirm diagnosis of vasculitic neuropathy, guiding targeted treatment.**
99	A 71-year-old male patient presents with a 2-year history of sensory-motor, symmetrical, length dependent	TTR-Amyloidosis	**Step-by-Step Analysis of Additional Examinations**<br>**Step 1: Summarize Clinical Data**<br>● **Patient Demographics: 71-year-old male**

	polyneuropathy with insidious onset and fast disease progression. The patient presents with moderate distal weakness and hypoesthesia in the four limbs, sensory ataxia and autonomic involvement with no foot deformities or neuropathic pain. Past medical history is positive for cardiac insufficiency. Nerve conduction studies show an axonal, sensory-motor polyneuropathy with bilateral carpal tunnel syndrome. Screening lab tests including vitamin B12, folate, vitamin B6, vitamin E, HBV, HCV, ANA, ANCA, ENA, glycaemia, HbA1c, CRP, ESR, copper, cryoglobulins, and serum electrophoresis are all negative. His brother is wheelchair bound.		**● Symptoms: 2-year history of sensory-motor, symmetrical, length-dependent polyneuropathy with insidious onset and fast progression, moderate distal weakness, hypoesthesia, sensory ataxia, autonomic involvement**<br>**● No Foot Deformities or Neuropathic Pain**<br>**● Medical History: Cardiac insufficiency**<br>**● Family History: Brother is wheelchair-bound**<br>**● Nerve Conduction Studies: Axonal, sensory-motor polyneuropathy, bilateral carpal tunnel syndrome**<br>**● Negative Screening Lab Tests: vitamin B12, folate, vitamin B6, vitamin E, HBV, HCV, ANA, ANCA, ENA, glycaemia, HbA1c, CRP, ESR, copper, cryoglobulins, serum electrophoresis**<br>**Step 2: List Additional Necessary Examinations**<br>**1. Genetic Testing for Transthyretin (TTR) Mutations**<br>**● Reasoning: Familial amyloid polyneuropathy (FAP) is most commonly associated with mutations in the TTR gene. Given the patient's family history and clinical presentation, genetic testing can confirm the diagnosis of hereditary amyloidosis.**<br>**2. Biopsy (Nerve and/or Abdominal Fat Pad)**<br>**● Reasoning: A biopsy can provide direct evidence of amyloid deposits. Congo red staining with apple-green birefringence under polarized light can confirm amyloid presence, which is diagnostic for amyloidosis.**<br>**3. Serum and Urine Immunofixation Electrophoresis (IFE)**<br>**● Reasoning: While initial serum electrophoresis was negative, IFE is more sensitive for detecting monoclonal proteins associated with light chain (AL) amyloidosis. This test can help rule out AL amyloidosis if FAP is not confirmed.**<br>**4. Autonomic Function Tests**<br>**● Reasoning: To objectively evaluate the extent of autonomic involvement, tests such as heart rate variability, sweat test, and blood pressure response to tilt-table testing can provide detailed information.**<br>**5. Echocardiogram and Cardiac MRI**<br>**● Reasoning: Cardiac involvement is common in amyloidosis, especially familial TTR amyloidosis. An echocardiogram can detect thickened ventricular walls and other features of amyloid cardiomyopathy. Cardiac MRI can provide detailed tissue characterization.**<br>**6. CSF Analysis (if CIDP is still a consideration)**<br>**● Reasoning: Although less likely, CIDP should be definitively ruled out. Elevated protein levels in cerebrospinal fluid (CSF) without pleocytosis can support a diagnosis of CIDP.**<br>**7. Quantitative Sensory Testing (QST)**<br>**● Reasoning: QST can help quantify the extent and distribution of sensory deficits, providing additional functional data that complements nerve conduction studies.**<br>**8. Genetic Testing for Common Hereditary Neuropathies (e.g., PMP22 duplication/deletion for CMT1A)**<br>**● Reasoning: Although less likely, testing for common hereditary neuropathies can be considered given the family history and the need to rule out other genetic causes of neuropathy.**<br>**Step 3: Determine the Most Crucial Test**<br>**Most Crucial Test: Genetic Testing for Transthyretin (TTR) Mutations**<br>**Reasoning:**<br>**● The clinical presentation strongly suggests familial amyloid polyneuropathy (FAP), which is most commonly caused by TTR mutations.**<br>**● Confirming a TTR mutation will not only solidify the diagnosis but also guide treatment options and genetic counseling for the patient and family members.**<br>**● This test directly addresses the most likely differential diagnosis based on the patient's history, family history, and clinical findings.**<br>**Conclusion**<br>**Given the patient's symptoms, family history, and current clinical data, genetic testing for transthyretin (TTR) mutations is the most critical test to confirm the suspected diagnosis of familial amyloid polyneuropathy. Other tests, such as biopsies and detailed cardiac evaluations, will provide supportive information and help assess the extent of the disease but are secondary to confirming the genetic basis of the suspected condition.**
100	A 76-year-old female patient presents with a 6-year history of chronic, pure sensory, symmetrical, length dependent polyneuropathy with insidious onset and slow progressive course. The main symptom is severe burning and tingling pain in the feet. Clinical examination reveals hyperalgesia upon pinprick testing, along with redness and warmth in the feet. There are no weakness or tactile hypoesthesia, sensory ataxia, tremor, foot deformities, or cranial nerve involvement. Past medical history is silent. Nerve conduction studies are normal with no signs of polyneuropathy. Screening lab tests including vitamin B12, folate, vitamin B6, vitamin E, HBV, HCV, ANA, ANCA, ENA, CRP, ESR, glycemia, HbA1c, copper, cryoglobulins, and serum electrophoresis are negative. There is family history of polyneuropathy.	Small fibre neuropathy	**Step-by-Step Approach to Additional Examinations**<br>**1. Skin Biopsy**<br>**● Reasoning: A skin biopsy is used to assess intraepidermal nerve fiber density (IENFD), which helps diagnose small fiber neuropathy (SFN). This test is crucial because SFN often involves small unmyelinated fibers that are not detected by standard nerve conduction studies, which were normal in this patient.**<br>**● Procedure: A small skin sample is taken, typically from the lower leg, and analyzed under a microscope to count nerve fibers.**<br>**● Expected Outcome: Reduced nerve fiber density would support a diagnosis of SFN.**<br>**2. Quantitative Sensory Testing (QST)**<br>**● Reasoning: QST evaluates the function of small sensory nerve fibers by measuring responses to various stimuli, such as temperature and vibration. This can help confirm SFN and provide information on the extent of sensory dysfunction.**<br>**● Procedure: Non-invasive testing where stimuli are applied to the skin, and the patient's responses are recorded and analyzed.**<br>**● Expected Outcome: Abnormal results indicating sensory dysfunction would support the diagnosis of SFN.**<br>**3. Genetic Testing**<br>**● Reasoning: Given the family history of polyneuropathy, genetic testing can identify hereditary forms of neuropathy, such as hereditary sensory and autonomic neuropathy (HSAN). This is particularly important since many hereditary neuropathies can present with similar symptoms.**<br>**● Procedure: Blood sample or saliva sample for DNA analysis, looking for mutations known to cause HSAN or other hereditary neuropathies.**<br>**● Expected Outcome: Identification of a specific genetic mutation would confirm a hereditary neuropathy.**<br>**4. Autonomic Function Testing**<br>**● Reasoning: Autonomic testing can assess the involvement of the autonomic nervous system, which may be indicated by the redness and warmth in the feet. These tests evaluate heart rate variability, blood pressure response to positional changes, and sweating responses.**<br>**● Procedure: Series of tests including tilt table test, deep breathing test, and thermoregulatory sweat test.**<br>**● Expected Outcome: Abnormal results would indicate autonomic dysfunction, supporting a diagnosis of conditions like SFN or HSAN.**<br>**5. Serum and Urine Protein Electrophoresis with Immunofixation**<br>**● Reasoning: This test can identify monoclonal gammopathies, such as multiple myeloma or POEMS syndrome, which can cause neuropathy. While the initial serum electrophoresis was negative, immunofixation is more sensitive.**<br>**● Procedure: Blood and urine samples are analyzed to detect abnormal proteins.**<br>**● Expected Outcome: Detection of monoclonal proteins would indicate a paraneoplastic or hematologic disorder causing neuropathy.**<br>**6. CSF Analysis**<br>**● Reasoning: Cerebrospinal fluid (CSF) analysis can help rule out inflammatory or infectious causes of neuropathy, such as Guillain-Barré syndrome or chronic inflammatory demyelinating polyneuropathy (CIDP), even though these are less likely given the normal nerve conduction studies.**<br>**● Procedure: Lumbar puncture to collect CSF for analysis of cell count, protein levels, and presence of oligoclonal bands.**

			• **Expected Outcome: Elevated protein without pleocytosis might suggest CIDP, whereas normal results would help exclude inflammatory neuropathies.**<br>Most Crucial Test<br>**The most crucial test from the list is the skin biopsy to assess intraepidermal nerve fiber density (IENFD). This test directly evaluates the presence and density of small nerve fibers, which are implicated in small fiber neuropathy (SFN). Given the patient's clinical presentation of pure sensory symptoms, normal nerve conduction studies, and the need to differentiate SFN from other potential diagnoses, this test provides essential diagnostic information.**<br>Conclusion<br>**Based on the patient's clinical presentation, normal nerve conduction studies, and family history, the following additional examinations are recommended:**<br>**1. Skin biopsy for IENFD**<br>**2. Quantitative sensory testing (QST)**<br>**3. Genetic testing**<br>**4. Autonomic function testing**<br>**5. Serum and urine protein electrophoresis with immunofixation**<br>**6. CSF analysis**<br>**The skin biopsy for IENFD is the most crucial test to confirm the diagnosis of small fiber neuropathy, given the specificity of the symptoms and the limitations of nerve conduction studies in detecting small fiber involvement.**

<u>Table 3.</u> Trial 2 – Case presentations and GPT-4 output to 1st prompt (diagnosis)

LEGEND: Anti-MAG neuropathy, anti-myelin-associated glycoprotein neuropathy; CANVAS, cerebellar ataxia, neuropathy, and vestibular areflexia syndrome; CIDP, chronic inflammatory demyelinating polyneuropathy; CMT, Charcot-Marie-Tooth disease; GBS, Guillain-Barré syndrome; HBV, hepatitis B virus; HCV, hepatitis C virus; HNPP, hereditary neuropathy with liability to pressure palsies; MGUS, monoclonal gammopathy of undetermined significance; MMN, multifocal motor neuropathy; POEMS, polyneuropathy, organomegaly, endocrinopathy, monoclonal gammopathy, and skin changes syndrome; TTR-Amyloidosis, transthyretin amyloidosis-associated neuropathy;

N°	Case presentation and Diagnosis	Diagnosis	Prompt 1 output
1	**Case presentation:** A 32-year old male patient presents with a 26-year history of sensory-motor, symmetrical, length dependent polyneuropathy with insidious onset and slow progression. The patient presents with scoliosis, neuropathic pain, sensory ataxia, pes cavus, distal weakness in the four limbs, severe calf atrophy with no cranial nerve or autonomic involvement. Past medical history is silent. Nerve conduction studies show an axonal, length dependent, symmetric, sensory-motor polyneuropathy. Screening lab tests including vitamin B12, folate, vitamin B6, vitamin E, HBV, HCV, glycemia, HbA1c, ANA, ANCA, ENA, CRP, ESR, copper, cryoglobulins, and serum electrophoresis are all negative. Both her father and grandfather have foot deformities and walking difficulties.	CMT2	**Step-by-Step Analysis**<br>**Summary of Pertinent Clinical Information**<br>● **Patient Profile:**<br>○ **Age/Gender:** 32-year-old male<br>○ **Presentation Duration:** 26-year history<br>○ **Symptoms:** Sensory-motor, symmetrical, length-dependent polyneuropathy with insidious onset and slow progression<br>○ **Additional Findings:**<br>■ Scoliosis<br>■ Neuropathic pain<br>■ Sensory ataxia<br>■ Pes cavus (high-arched feet)<br>■ Distal weakness in all four limbs<br>■ Severe calf atrophy<br>■ No cranial nerve or autonomic involvement<br>● **Past Medical History:** Unremarkable<br>● **Investigations:**<br>○ **Nerve Conduction Studies:** Axonal, length-dependent, symmetric, sensory-motor polyneuropathy<br>○ **Laboratory Tests:** Negative for vitamin B12, folate, vitamin B6, vitamin E, HBV, HCV, glycemia, HbA1c, ANA, ANCA, ENA, CRP, ESR, copper, cryoglobulins, serum electrophoresis<br>● **Family History:**<br>○ **Father and Grandfather:** Both have foot deformities and walking difficulties<br>**Differential Diagnoses for Polyneuropathy**<br>1. **Charcot-Marie-Tooth Disease (CMT)**<br>○ **Reasoning:**<br>■ **Hereditary Pattern:** The patient's father and grandfather also have foot deformities and walking difficulties, suggesting a familial hereditary pattern.<br>■ **Clinical Features:** Pes cavus, scoliosis, distal muscle weakness, and severe calf atrophy are characteristic of CMT. The sensory-motor polyneuropathy with length dependency fits well with CMT presentation.<br>■ **Nerve Conduction Studies:** Axonal polyneuropathy observed in CMT2 (axonal type) matches the findings in this patient.<br>2. **Hereditary Neuropathy with Liability to Pressure Palsies (HNPP)**<br>○ **Reasoning:**<br>■ **Hereditary Pattern:** Although this condition is less likely to present with a 26-year progressive history, the family history and foot deformities suggest consideration.<br>■ **Clinical Features:** Sensory-motor neuropathy and foot deformities can occur, but scoliosis and severe calf atrophy are less typical.<br>■ **Nerve Conduction Studies:** Usually show focal demyelination rather than the diffuse axonal pattern seen in this patient.<br>3. **Friedreich's Ataxia**<br>○ **Reasoning:**<br>■ **Hereditary Pattern:** Inherited in an autosomal recessive pattern which could explain family history if parents were carriers.<br>■ **Clinical Features:** Includes sensory ataxia, scoliosis, and progressive weakness, often accompanied by pes cavus.<br>■ **Nerve Conduction Studies:** Can show mixed axonal and demyelinating features, but the absence of cranial nerve involvement and the specific family history make it a less likely fit compared to CMT.<br>**Most Likely Diagnosis**<br>Based on the clinical presentation, nerve conduction study findings, and family history, **Charcot-Marie-Tooth Disease (CMT)** is the most likely diagnosis. The insidious onset, slow progression, and combination of sensory-motor polyneuropathy with scoliosis, pes cavus, distal weakness, and severe calf atrophy strongly point towards CMT. The family history of similar symptoms further supports this diagnosis, particularly CMT type 2 (CMT2) given the axonal nature of the neuropathy observed in the nerve conduction studies.
2	**Case presentation:** An 88-year-old male patient presents with a 19-month history of pure sensory, symmetrical, length dependent polyneuropathy with insidious onset and slow progressive course. The patient presents with hypoesthesia, paresthesia, and cramps in the lower limbs in a stocking distribution. Clinical examination does not reveal weakness or sensory ataxia, neuropathic pain, tremor, foot deformities, cranial nerve or autonomic involvement. Past medical history is positive for colorectal cancer, for which the patient underwent chemotherapy 5 years ago, and chronic	HCV-associated neuropathy	**Summary of Pertinent Clinical Information**<br>● **Patient Demographics**: 88-year-old male<br>● **Symptom History**:<br>○ 19-month history<br>○ Pure sensory, symmetrical, length-dependent polyneuropathy<br>○ Insidious onset, slow progressive course<br>○ Symptoms: hypoesthesia, paresthesia, cramps in lower limbs in a stocking distribution<br>● **Clinical Examination**:<br>○ No weakness<br>○ No sensory ataxia<br>○ No neuropathic pain, tremor, foot deformities<br>○ No cranial nerve or autonomic involvement<br>● **Past Medical History**:<br>○ Colorectal cancer (chemotherapy 5 years ago)<br>○ Chronic HCV infection

	HCV infection. Nerve conduction studies show a pure sensory, symmetric, length-dependent axonal polyneuropathy with unilateral carpal tunnel syndrome. Screening lab tests including vitamin B12, folate, vitamin B6, vitamin E, HBV, glycemia, HbA1c, ANA, ANCA, ENA, CRP, ESR, copper, cryoglobulins, and serum electrophoresis are normal, with positivity for HCV IgG. There is no family history of polyneuropathy.		● **Nerve Conduction Studies**:<br>○ Pure sensory, symmetric, length-dependent axonal polyneuropathy<br>○ Unilateral carpal tunnel syndrome<br>● **Laboratory Tests**:<br>○ Normal: vitamin B12, folate, vitamin B6, vitamin E, HBV, glycemia, HbA1c, ANA, ANCA, ENA, CRP, ESR, copper, cryoglobulins, serum electrophoresis<br>○ Positive: HCV IgG<br>● **Family History**: Negative for polyneuropathy<br>**Differential Diagnoses for Polyneuropathy**<br>1. **Chronic HCV-Associated Neuropathy**<br>○ **Reasoning**:<br>■ HCV infection is known to cause chronic peripheral neuropathy, primarily due to cryoglobulinemia, although the cryoglobulin test is negative here.<br>■ The presence of HCV IgG indicates chronic HCV infection, which can lead to a slow-progressing sensory neuropathy.<br>■ The absence of systemic inflammatory markers (CRP, ESR) and other typical signs (such as vasculitis) does not rule out HCV-associated neuropathy.<br>■ The patient's history of chronic HCV infection and corresponding sensory symptoms align with this diagnosis.<br>2. **Chemotherapy-Induced Peripheral Neuropathy (CIPN)**<br>○ **Reasoning**:<br>■ Chemotherapy, particularly drugs used in colorectal cancer treatment (such as oxaliplatin), is a well-known cause of peripheral neuropathy.<br>■ Although the chemotherapy was 5 years ago, neuropathy can be a late-onset side effect in some cases.<br>■ The sensory nature of the neuropathy without motor involvement and the stocking distribution of symptoms are consistent with CIPN.<br>■ The patient's past medical history of chemotherapy for colorectal cancer supports this diagnosis.<br>3. **Idiopathic Sensory Neuropathy**<br>○ **Reasoning**:<br>■ Despite thorough screening, many cases of sensory polyneuropathy remain idiopathic, especially in the elderly.<br>■ The normal results of extensive laboratory testing and the lack of a family history make an idiopathic cause a reasonable consideration.<br>■ The presentation of a length-dependent, symmetrical, pure sensory neuropathy with no motor involvement fits the pattern of idiopathic sensory neuropathy.<br>■ Age-related degeneration and subtle metabolic changes not detected by standard tests could contribute to this form of neuropathy.<br>**Most Likely Diagnosis**<br>**Chronic HCV-Associated Neuropathy** is the most likely diagnosis in this patient. The presence of chronic HCV infection, coupled with the typical presentation of a sensory, symmetrical, length-dependent polyneuropathy, supports this diagnosis. Despite the absence of cryoglobulins, HCV can still lead to neuropathy through other mechanisms. The patient's clinical history and laboratory findings strongly suggest this etiology over the others considered.
3	**Case presentation:** A 75-year-old female patient presents with a 1-year history of sensory-motor, symmetrical, length dependent polyneuropathy with subacute onset. The patient presents with paresthesia and hypoesthesia in the lower limbs and sensory ataxia, without tremor, foot deformities, neuropathic pain, cranial nerve or autonomic involvement. Past medical history shows breast cancer, for which the patient has undergone chemotherapy. Neuropathy symptoms started during chemotherapy. Nerve conduction studies show a symmetric, sensory-motor, axonal polyneuropathy. Screening lab tests including vitamin B12, folate, vitamin B6, vitamin E, HCV, HBV, ANA, ANCA, ENA, CRP, ESR, glycemia, HbA1c, copper, cryoglobulins and serum electrophoresis are all negative. There is no family history of polyneuropathy.	Chemotherapy induced polyneuropathy	**Summary of Pertinent Clinical Information**<br>**Patient Demographics:**<br>● **Age:** 75-year-old<br>● **Gender:** Female<br>**Clinical Presentation:**<br>● **Duration of Symptoms:** 1-year history<br>● **Onset:** Subacute<br>● **Type:** Sensory-motor, symmetrical, length-dependent polyneuropathy<br>● **Symptoms:**<br>○ Paresthesia and hypoesthesia in the lower limbs<br>○ Sensory ataxia<br>● **Absent Symptoms:**<br>○ No tremor<br>○ No foot deformities<br>○ No neuropathic pain<br>○ No cranial nerve involvement<br>○ No autonomic involvement<br>**Past Medical History:**<br>● **Breast cancer:** Underwent chemotherapy<br>**Timeline:** Neuropathy symptoms started during chemotherapy.<br>**Diagnostic Findings:**<br>● **Nerve Conduction Studies:** Symmetric, sensory-motor, axonal polyneuropathy<br>● **Lab Tests:** Negative for vitamin B12, folate, vitamin B6, vitamin E, HCV, HBV, ANA, ANCA, ENA, CRP, ESR, glycemia, HbA1c, copper, cryoglobulins, and serum electrophoresis<br>● **Family History:** No family history of polyneuropathy<br>**Differential Diagnoses for Polyneuropathy**<br>**1. Chemotherapy-Induced Peripheral Neuropathy (CIPN):**<br>● **Reasoning:**<br>○ The patient's neuropathy symptoms started during chemotherapy for breast cancer.<br>○ Chemotherapy agents, especially taxanes, platinum compounds, and vinca alkaloids, are well-known causes of peripheral neuropathy.<br>○ The presentation of sensory-motor, symmetrical, length-dependent polyneuropathy aligns with typical CIPN patterns.<br>○ The absence of neuropathic pain and cranial nerve involvement fits with CIPN, which primarily affects the distal extremities.<br>**2. Paraneoplastic Neuropathy:**<br>● **Reasoning:**<br>○ Although less common than CIPN, paraneoplastic syndromes can present as polyneuropathy.

			○ The temporal association with breast cancer raises this possibility.<br>○ However, paraneoplastic neuropathies often present with additional systemic or neurological symptoms, which are absent in this case.<br>○ Negative autoimmune markers (ANA, ANCA, ENA) make this less likely, but not impossible.<br>**3. Idiopathic Axonal Polyneuropathy:**<br>● **Reasoning:**<br>○ Given the extensive negative workup (including nutritional, infectious, autoimmune, and metabolic causes), idiopathic polyneuropathy remains a consideration.<br>○ This diagnosis is one of exclusion, typically considered when no specific cause is found after thorough investigation.<br>○ The age of the patient also aligns with idiopathic polyneuropathy, which is more common in older adults.<br>**Most Likely Diagnosis**<br>**Most Likely Diagnosis: Chemotherapy-Induced Peripheral Neuropathy (CIPN)**<br>**Detailed Reasoning:**<br>● The temporal correlation between the onset of neuropathy symptoms and chemotherapy is strong.<br>● The specific chemotherapy agents used for breast cancer treatment are known to cause peripheral neuropathy.<br>● The clinical pattern of sensory-motor, symmetrical, length-dependent polyneuropathy without other systemic or neurological involvements strongly supports CIPN.<br>● The negative extensive lab workup rules out many other potential causes of polyneuropathy, further supporting CIPN as the most probable diagnosis.<br>In conclusion, considering the patient's clinical history, symptomatology, and diagnostic findings, Chemotherapy-Induced Peripheral Neuropathy (CIPN) is the most likely diagnosis.
4	**Case presentation:** An 81-year-old male patient presents with a 9-week history of sensory-motor, asymmetrical multineuropathy with subacute onset and progressive course. The patient presents with paresthesia, hypoesthesia, neuropathic pain, and weakness with a multifocal distribution. Clinical examination does not reveal sensory ataxia nor tremor, foot deformities, cranial nerve or autonomic involvement. Past medical history is positive for giant cell arteritis. Nerve conduction studies show a sensory-motor, axonal multineuropathy. Screening lab tests including vitamin B12, folate, vitamin B6, vitamin E, HBV, HCV, glycemia, HbA1c, ANA, ENA, copper, cryoglobulins, and serum electrophoresis are all negative, with evidence of elevated CRP and ESR. There is no family history of polyneuropathy.	Vasculitic neuropathy	**Summary of Clinical Information**<br>● **Patient Details**: 81-year-old male<br>● **Symptom History**: 9-week history of sensory-motor, asymmetrical multineuropathy with subacute onset and progressive course<br>○ **Symptoms**: Paresthesia, hypoesthesia, neuropathic pain, and weakness<br>○ **Distribution**: Multifocal<br>● **Clinical Examination**:<br>○ Absence of sensory ataxia<br>○ No tremor<br>○ No foot deformities<br>○ No cranial nerve involvement<br>○ No autonomic involvement<br>● **Past Medical History**: Positive for giant cell arteritis<br>● **Nerve Conduction Studies**: Sensory-motor, axonal multineuropathy<br>● **Screening Lab Tests**: Negative for vitamin B12, folate, vitamin B6, vitamin E, HBV, HCV, glycemia, HbA1c, ANA, ENA, copper, cryoglobulins, and serum electrophoresis<br>● **Inflammatory Markers**: Elevated CRP and ESR<br>● **Family History**: No family history of polyneuropathy<br>**Differential Diagnoses for Polyneuropathy**<br>1. **Vasculitic Neuropathy (Secondary to Giant Cell Arteritis)**<br>○ **Reasoning**:<br>■ The patient has a known history of giant cell arteritis, an inflammatory vasculitis that can affect multiple organ systems including the peripheral nerves.<br>■ The subacute onset and progressive nature of the neuropathy align with vasculitic processes which can cause asymmetrical, multifocal neuropathy.<br>■ Elevated CRP and ESR are indicative of an ongoing inflammatory process, which is consistent with active vasculitis.<br>■ The lack of sensory ataxia, cranial nerve involvement, and autonomic symptoms also fits the profile of vasculitic neuropathy.<br>2. **Monoclonal Gammopathy of Undetermined Significance (MGUS) with Peripheral Neuropathy**<br>○ **Reasoning**:<br>■ While screening lab tests including serum electrophoresis were negative, it is worth considering that MGUS could still be a possibility due to the age of the patient.<br>■ MGUS can present with a sensory-motor, axonal neuropathy, and the multifocal distribution of symptoms could be a feature.<br>■ Although not the most likely given the absence of a monoclonal protein, it is still a consideration in an elderly patient with progressive neuropathy.<br>3. **Chronic Inflammatory Demyelinating Polyneuropathy (CIDP)**<br>○ **Reasoning**:<br>■ CIDP typically presents with a progressive course of motor and sensory dysfunction.<br>■ The absence of sensory ataxia, foot deformities, and cranial nerve involvement can still fit within the spectrum of CIDP presentations, although less common.<br>■ Elevated inflammatory markers (CRP, ESR) could be associated with an underlying inflammatory neuropathy.<br>■ The patient's nerve conduction studies indicate axonal involvement, which is less common in CIDP but still possible in a subset of patients.<br>**Most Likely Diagnosis**<br>Given the patient's past medical history of giant cell arteritis, the subacute and progressive course of asymmetrical multineuropathy, elevated inflammatory markers (CRP and ESR), and the exclusion of other common causes of neuropathy, the most likely diagnosis is **vasculitic neuropathy secondary to giant cell arteritis**. This diagnosis accounts for the multifocal distribution of symptoms and the patient's existing condition that predisposes him to such neuropathic manifestations. Further diagnostic steps could include nerve biopsy and further rheumatologic evaluation to confirm the vasculitic process and guide appropriate treatment.
5	A 61-year-old male patient presents with a 1-year history of sensory-motor, asymmetric, non-length dependent polyneuropathy with subacute onset and progressive course. The patient presents with asymmetric distal paresthesia and neuropathic pain distributed in the four limbs, associated with weakness in the four limbs both proximally and distally. Clinical	POEMS	**Summary of Pertinent Clinical Information**<br>● **Patient:** 61-year-old male<br>● **History:** 1-year history of sensory-motor, asymmetric, non-length dependent polyneuropathy with subacute onset and progressive course<br>● **Symptoms:**<br>○ Asymmetric distal paresthesia<br>○ Neuropathic pain in four limbs<br>○ Weakness in four limbs, both proximally and distally<br>○ Mild sensory ataxia<br>○ Peripheral edema in the feet

	examination also shows the presence of mild sensory ataxia, peripheral edema in the feet, and mild cutaneous hyperpigmentation in the lower limbs. There are no autonomic symptoms or foot deformities. Past medical history is positive for psoriatic arthritis. Nerve conduction studies show a demyelinating sensory-motor polyneuropathy. Screening lab tests show presence of IgG lambda paraprotein and elevated CRP. Remaining exams, including vitamin B12, folate, vitamin B6, vitamin E, HBV, HCV, ANA, ENA, ANCA, ESR, glycemia, HbA1c, copper, and cryoglobulins are negative. There is no family history of polyneuropathy.		○ Mild cutaneous hyperpigmentation in lower limbs<br>● **Clinical Examination:** No autonomic symptoms or foot deformities<br>● **Past Medical History:** Psoriatic arthritis<br>● **Nerve Conduction Studies:** Demyelinating sensory-motor polyneuropathy<br>● **Lab Results:**<br>○ Positive IgG lambda paraprotein<br>○ Elevated CRP<br>○ Negative for vitamin B12, folate, vitamin B6, vitamin E, HBV, HCV, ANA, ENA, ANCA, ESR, glycemia, HbA1c, copper, and cryoglobulins<br>● **Family History:** No family history of polyneuropathy<br>**Differential Diagnoses for Polyneuropathy**<br>1. **Monoclonal Gammopathy of Undetermined Significance (MGUS) with Associated Polyneuropathy**<br>○ **Reasoning:**<br>■ Presence of IgG lambda paraprotein suggests a possible underlying monoclonal gammopathy.<br>■ MGUS is a condition where a paraprotein is present without evidence of multiple myeloma or other malignancies and can be associated with polyneuropathy.<br>■ The clinical presentation of sensory-motor polyneuropathy, particularly with demyelination on nerve conduction studies, is consistent with neuropathy associated with MGUS.<br>■ Elevated CRP indicates an inflammatory process, which can be seen in MGUS-related neuropathies.<br>2. **Chronic Inflammatory Demyelinating Polyneuropathy (CIDP)**<br>○ **Reasoning:**<br>■ CIDP is an acquired immune-mediated inflammatory disorder of the peripheral nervous system characterized by progressive or relapsing-remitting course.<br>■ The subacute onset and progressive nature of the patient's symptoms align with CIDP.<br>■ Demyelinating features on nerve conduction studies are hallmark findings in CIDP.<br>■ The presence of elevated CRP supports an inflammatory etiology, and mild sensory ataxia and weakness fit the clinical picture of CIDP.<br>■ Peripheral edema and cutaneous hyperpigmentation are less typical but can occur.<br>3. **Multifocal Motor Neuropathy (MMN)**<br>○ **Reasoning:**<br>■ MMN is characterized by asymmetric motor neuropathy with demyelinating features, often without significant sensory involvement.<br>■ The patient's asymmetric distribution and weakness both proximally and distally could be indicative of MMN.<br>■ Although MMN typically involves motor nerves, sensory involvement can occasionally be present.<br>■ Elevated CRP suggests an inflammatory process, which could be seen in MMN.<br>■ The absence of autonomic symptoms aligns with MMN, though peripheral edema is unusual.<br>**Most Likely Diagnosis**<br>Based on the clinical presentation, nerve conduction study results, and laboratory findings, the most likely diagnosis is **Chronic Inflammatory Demyelinating Polyneuropathy (CIDP)**. This is supported by the progressive, subacute onset, demyelinating nature on nerve conduction studies, elevated CRP indicating inflammation, and the pattern of sensory-motor involvement with ataxia and weakness. While MGUS with associated polyneuropathy is a strong contender given the presence of IgG lambda paraprotein, CIDP is more consistent with the overall clinical picture, including the lack of systemic symptoms typically associated with more severe hematologic malignancies.
6	A 73-year-old female patient presents with an 8-month history of pure sensory, symmetrical, length dependent polyneuropathy with subacute onset and progressive course. The patient presents with distal paresthesia in the upper and lower limbs without weakness or neuropathic pain. Clinical examination reveals reduced tactile sensation in the feet. There are no tremor or ataxia, foot deformities, cranial nerve or autonomic involvement. Past medical history is positive for episodic diarrhea. Nerve conduction studies show a pure sensory, symmetric, length dependent, axonal polyneuropathy. Screening lab tests reveal the presence of vitamin B12 deficiency, hyperglycaemia, and elevated HbA1c. Remaining exams including folate, vitamin B6, vitamin E, HCV, HBV, ANA, ENA, ANCA, CRP, ESR, copper, cryoglobulins and, serum electrophoresis are negative. There is no family history of polyneuropathy.	Vit B12 deficiency neuropathy	**Summary of Pertinent Clinical Information**<br>**Patient Profile:**<br>● **Age:** 73 years<br>● **Gender:** Female<br>● **Duration of Symptoms:** 8 months<br>● **Symptom Description:** Pure sensory, symmetrical, length-dependent polyneuropathy<br>● **Onset and Course:** Subacute onset, progressive course<br>● **Symptoms:**<br>○ Distal paresthesia in upper and lower limbs<br>○ No weakness or neuropathic pain<br>○ Reduced tactile sensation in feet<br>● **Examination Findings:**<br>○ Reduced tactile sensation in the feet<br>○ No tremor, ataxia, foot deformities, cranial nerve, or autonomic involvement<br>**Past Medical History:**<br>● Episodic diarrhea<br>**Nerve Conduction Studies:**<br>● Pure sensory, symmetric, length-dependent, axonal polyneuropathy<br>**Laboratory Findings:**<br>● Vitamin B12 deficiency<br>● Hyperglycemia<br>● Elevated HbA1c<br>● Negative for:<br>○ Folate<br>○ Vitamin B6, Vitamin E<br>○ Hepatitis C Virus (HCV), Hepatitis B Virus (HBV)<br>○ Antinuclear Antibodies (ANA), Extractable Nuclear Antigen Antibodies (ENA), Antineutrophil Cytoplasmic Antibodies (ANCA)<br>○ C-Reactive Protein (CRP), Erythrocyte Sedimentation Rate (ESR)<br>○ Copper, cryoglobulins

			○ Serum electrophoresis<br>**Family History:**<br>● No family history of polyneuropathy<br>**Differential Diagnoses for Polyneuropathy**<br>1. **Diabetic Neuropathy:**<br>○ **Reasoning:** The patient has hyperglycemia and an elevated HbA1c, which are indicative of diabetes or impaired glucose tolerance. Diabetic neuropathy is a common complication of diabetes, typically presenting as a length-dependent, symmetric, sensory polyneuropathy. This aligns with the patient's clinical presentation and nerve conduction study findings.<br>○ **Supportive Evidence:**<br>■ Hyperglycemia and elevated HbA1c<br>■ Pure sensory, symmetrical, length-dependent polyneuropathy<br>2. **Vitamin B12 Deficiency Neuropathy:**<br>○ **Reasoning:** The presence of vitamin B12 deficiency is a significant finding as it can lead to sensory neuropathy, often presenting with paresthesias and sensory deficits. Although vitamin B12 deficiency more commonly presents with combined system disease affecting both the spinal cord and peripheral nerves, a pure sensory neuropathy is possible.<br>○ **Supportive Evidence:**<br>■ Confirmed vitamin B12 deficiency<br>■ Sensory deficits without weakness or autonomic involvement<br>3. **Idiopathic Sensory Neuropathy:**<br>○ **Reasoning:** In the absence of identifiable causes (e.g., diabetes, nutritional deficiencies), idiopathic sensory neuropathy could be considered, especially in the elderly population. However, this is more of a diagnosis of exclusion when common etiologies have been ruled out.<br>○ **Supportive Evidence:**<br>■ Negative results for extensive screening of other potential causes (e.g., infections, autoimmune markers)<br>■ Age-related increase in idiopathic cases<br>**Most Likely Diagnosis**<br>**Diabetic Neuropathy** is the most likely diagnosis given the presence of hyperglycemia and elevated HbA1c, which strongly suggest diabetes or prediabetes. The patient's clinical presentation of a pure sensory, symmetric, length-dependent polyneuropathy fits well with the typical manifestations of diabetic neuropathy.<br>**Conclusion**<br>Considering the clinical presentation, nerve conduction study results, and laboratory findings, **diabetic neuropathy** is the most probable diagnosis. The presence of hyperglycemia and elevated HbA1c, combined with the sensory polyneuropathy without other significant findings, strongly supports this diagnosis. Management should include optimizing glycemic control and addressing the vitamin B12 deficiency to prevent further complications.
7	A 70-year-old male patient presents with a 5-week history of pure sensory, symmetrical, length dependent polyneuropathy with subacute onset and progressive course. The patient presents with distal paresthesia and hypoesthesia in the four limbs and sensory ataxia. Clinical examination does not show weakness, tremor, foot deformities, autonomic or cranial nerve involvement. Past medical history is positive for obesity treated with bariatric surgery, anemia, hypercholesterolemia, and type 2 diabetes mellitus. Nerve conduction studies show a pure sensory, symmetric, axonal polyneuropathy. Screening lab tests shows slightly elevated glycemia and HbA1c levels, and severe folate deficiency. Remaining exams including vitamin B12, E, HBV, HCV, ANA, ANCA, ENA, CRP, ESR, copper, cryoglobulins, and serum electrophoresis are normal. There is no family history of polyneuropathy.	Folate deficiency neuropathy	**Summary of Clinical Information**<br>● **Patient Details:**<br>○ 70-year-old male<br>○ History: 5 weeks of sensory, symmetrical, length-dependent polyneuropathy<br>○ Symptoms: Distal paresthesia, hypoesthesia in all four limbs, sensory ataxia<br>○ Examination: No weakness, tremor, foot deformities, autonomic or cranial nerve involvement<br>● **Past Medical History:**<br>○ Obesity (treated with bariatric surgery)<br>○ Anemia<br>○ Hypercholesterolemia<br>○ Type 2 diabetes mellitus<br>● **Nerve Conduction Studies:**<br>○ Pure sensory, symmetric, axonal polyneuropathy<br>● **Lab Tests:**<br>○ Slightly elevated glycemia and HbA1c<br>○ Severe folate deficiency<br>○ Normal levels of vitamin B12, E, HBV, HCV, ANA, ANCA, ENA, CRP, ESR, copper, cryoglobulins, and serum electrophoresis<br>● **Family History:**<br>○ No family history of polyneuropathy<br>**Differential Diagnoses**<br>1. **Diabetic Polyneuropathy:**<br>○ **Reasoning:**<br>■ The patient has a history of type 2 diabetes mellitus with slightly elevated glycemia and HbA1c levels, indicating suboptimal glycemic control.<br>■ Diabetic polyneuropathy typically presents as a symmetrical, length-dependent polyneuropathy, predominantly affecting sensory nerves initially.<br>■ While autonomic involvement is common in advanced stages, early diabetic neuropathy often presents with sensory symptoms like paresthesia and hypoesthesia.<br>○ **Supporting Evidence:**<br>■ History of diabetes<br>■ Symmetrical, sensory, length-dependent neuropathy<br>■ Elevated glycemia and HbA1c<br>2. **Folate Deficiency Neuropathy:**<br>○ **Reasoning:**<br>■ The patient has severe folate deficiency, which can lead to neuropathy.<br>■ Folate deficiency can cause sensory neuropathy, presenting with paresthesia, hypoesthesia, and sensory ataxia, without motor involvement.<br>■ The patient's history of bariatric surgery increases the risk of malabsorption and vitamin deficiencies, including folate.<br>○ **Supporting Evidence:**

<table>
<tr><td></td><td></td><td></td><td>■ Severe folate deficiency
■ History of bariatric surgery (risk for malabsorption)
■ Sensory symptoms without motor involvement
3. Idiopathic Axonal Polyneuropathy:
○ Reasoning:
■ This condition is a diagnosis of exclusion and often presents in elderly patients with a subacute or chronic course.
■ It is characterized by sensory symptoms and can be length-dependent, as seen in this patient.
■ The absence of significant findings in extensive laboratory testing supports the possibility of an idiopathic cause.
○ Supporting Evidence:
■ Age of the patient
■ Symmetrical, sensory, length-dependent polyneuropathy
■ Negative extensive laboratory workup (excluding folate deficiency)
Most Likely Diagnosis
Folate Deficiency Neuropathy is the most likely diagnosis given the severity of the folate deficiency, the patient's history of bariatric surgery (which predisposes to malabsorption syndromes), and the typical presentation of sensory neuropathy without motor involvement. While diabetic polyneuropathy is also a strong contender, the acute and progressive nature over 5 weeks, along with the significant folate deficiency, points more convincingly towards a nutritional deficiency neuropathy. Correcting the folate deficiency and monitoring the patient's response would be a prudent next step in management.</td></tr>
<tr><td>8</td><td>A 74-year-old male patient presents with a 9-month history of pure sensory, symmetrical, length dependent polyneuropathy with subacute onset while hospitalised for lymphoma. The patient developed neuropathic pain in the feet with no sensory deficits or weakness. Clinical examination reveals absence of ataxia, tremor, foot deformities, cranial nerve or autonomic involvement. Past medical history is positive for diabetes since the age of 43, and lymphoma for which the patient has undergone chemotherapy. Neuropathy symptoms started during chemotherapy. Nerve conduction studies show a sensory-motor, symmetric, axonal polyneuropathy. Screening lab tests including vitamin B12, folate, vitamin B6, vitamin E, HCV, ANA, ANCA, ENA, CRP, ESR, copper, cryoglobulins and serum electrophoresis are all negative. HbA1c and glycemia levels are elevated and there is evidence of chronic HBV infection. There is no family history of polyneuropathy.</td><td>Chemotherapy-induced neuropathy</td><td>Summary of Clinical Information
● Patient: 74-year-old male
● Presentation: 9-month history of pure sensory, symmetrical, length-dependent polyneuropathy
○ Onset: Subacute, during hospitalization for lymphoma
○ Symptoms: Neuropathic pain in the feet, no sensory deficits or weakness
● Clinical Examination:
○ Absence of ataxia, tremor, foot deformities, cranial nerve or autonomic involvement
● Past Medical History:
○ Diabetes since age 43
○ Lymphoma, treated with chemotherapy
● Nerve Conduction Studies: Sensory-motor, symmetric, axonal polyneuropathy
● Screening Lab Tests: Negative for vitamin B12, folate, vitamin B6, vitamin E, HCV, ANA, ANCA, ENA, CRP, ESR, copper, cryoglobulins, and serum electrophoresis
● Additional Findings:
○ Elevated HbA1c and glycemia levels
○ Chronic HBV infection
● Family History: No family history of polyneuropathy
Differential Diagnoses for Polyneuropathy
1. Chemotherapy-Induced Peripheral Neuropathy (CIPN)
○ Reasoning: The neuropathy symptoms began during chemotherapy for lymphoma, a common cause of polyneuropathy. Chemotherapy agents, particularly those used in treating lymphoma, such as platinum-based compounds, taxanes, and vinca alkaloids, are well-known to cause sensory, axonal polyneuropathy. The subacute onset and pure sensory nature of the neuropathy align with typical presentations of CIPN.
2. Diabetic Neuropathy
○ Reasoning: The patient has a long-standing history of diabetes (over 30 years), with elevated HbA1c and glycemia levels indicating poorly controlled diabetes. Diabetic neuropathy is a common cause of polyneuropathy, characterized by sensory symptoms and axonal damage, as seen in nerve conduction studies. The symmetrical, length-dependent distribution of neuropathic pain also fits the typical presentation of diabetic neuropathy.
3. Chronic Hepatitis B Virus (HBV)-Related Neuropathy
○ Reasoning: Although less common, chronic HBV infection can lead to neuropathy, potentially through immune-mediated mechanisms or direct viral effects. Given the chronic HBV infection in this patient, it is a plausible contributing factor. However, the absence of specific markers of immune-mediated conditions (e.g., negative ANA, ANCA) makes this a less likely primary cause compared to the first two differentials.
Most Likely Diagnosis
Chemotherapy-Induced Peripheral Neuropathy (CIPN)
● Conclusion: The most likely diagnosis is CIPN, given the clear temporal relationship between the onset of neuropathy and the administration of chemotherapy. The specific characteristics of the neuropathy (sensory, symmetrical, axonal) align with the typical presentation of CIPN. While diabetic neuropathy is also a strong contender due to the patient's long history of diabetes, the sudden onset of symptoms during chemotherapy favors CIPN as the primary cause. Chronic HBV-related neuropathy remains a consideration but is less likely to be the primary cause given the context and negative immune markers.</td></tr>
<tr><td>9</td><td>A 77-year-old female patient presents with a 3-week history of pure sensory, symmetrical, length dependent polyneuropathy with subacute onset and progressive course. The patient presents with hypoesthesia and mild neuropathic pain in the feet. Clinical examination reveals also mild sensory ataxia. There are no tremor, foot deformities, cranial nerve or autonomic involvement. Past medical history is negative. Nerve conduction studies show a pure sensory, length dependent, symmetric, axonal polyneuropathy. Screening lab tests reveal decreased serum level of vitamin B6 and a slight increase in transaminase levels</td><td>B6 deficiency neuropathy</td><td>Clinical Summary
Patient: 77-year-old female
Presentation:
● 3-week history of pure sensory, symmetrical, length-dependent polyneuropathy
● Subacute onset with progressive course
● Symptoms: Hypoesthesia and mild neuropathic pain in the feet
● Clinical examination: Mild sensory ataxia, no tremor, foot deformities, cranial nerve, or autonomic involvement
Past Medical History: Negative
Diagnostic Findings:
● Nerve Conduction Studies: Pure sensory, length-dependent, symmetric, axonal polyneuropathy
● Lab Tests:
○ Decreased serum vitamin B6 levels
○ Slight increase in transaminase levels (AST/ALT)
○ Negative for: Vitamin B12, folate, vitamin E, HBV, HCV, glycemia, HbA1c, ANA, ANCA, ENA, CRP, ESR, copper, cryoglobulins, and serum electrophoresis
Family History: Negative for polyneuropathy
Differential Diagnoses</td></tr>
</table>

	(aspartate aminotransferase/alanine aminotransferase). Remaining exams including vitamin B12, folate, vitamin E, HBV, HCV, glycemia, HbA1c, ANA, ANCA, ENA, CRP, ESR, copper, cryoglobulins, and serum electrophoresis are negative. There is no family history of polyneuropathy.		**1. Vitamin B6 Deficiency Neuropathy**<br>**Reasoning:**<br>● **Clinical Features:** Pure sensory neuropathy, mild neuropathic pain, sensory ataxia.<br>● **Lab Findings:** Decreased serum vitamin B6 level.<br>● **Mechanism:** Vitamin B6 is crucial for normal nerve function. Its deficiency can lead to sensory neuropathy, characterized by hypoesthesia and sensory ataxia. The subacute onset and progressive nature align with a nutritional deficiency causing nerve damage over time.<br>**2. Alcoholic Neuropathy**<br>**Reasoning:**<br>● **Clinical Features:** Symmetrical, length-dependent polyneuropathy, mild sensory ataxia.<br>● **Lab Findings:** Slight increase in transaminase levels (AST/ALT), which can be indicative of liver damage due to alcohol use.<br>● **Mechanism:** Chronic alcohol consumption can lead to nutritional deficiencies (e.g., vitamin B6) and direct toxic effects on nerves, causing axonal polyneuropathy. This fits the pattern of a progressive sensory neuropathy.<br>**3. Cryptogenic Sensory Polyneuropathy (Idiopathic Neuropathy)**<br>**Reasoning:**<br>● **Clinical Features:** Symmetrical, sensory, length-dependent neuropathy with subacute onset and progressive course, mild sensory ataxia.<br>● **Lab Findings:** Extensive negative workup for common causes of neuropathy (e.g., vitamin B12, folate, diabetes, infections).<br>● **Mechanism:** In some older adults, a cause for polyneuropathy cannot be identified despite extensive testing. These cases are classified as idiopathic or cryptogenic. Given the negative comprehensive lab results and the patient's age, this remains a possibility.<br>**Most Likely Diagnosis**<br>**Vitamin B6 Deficiency Neuropathy** is the most likely diagnosis due to:<br>● **Direct Evidence:** Decreased serum vitamin B6 level, which is a well-known cause of sensory neuropathy.<br>● **Consistency:** The clinical presentation (pure sensory neuropathy, sensory ataxia) aligns well with known effects of vitamin B6 deficiency.<br>● **Progression:** The subacute onset and progressive course are typical for a deficiency-related neuropathy, which can worsen over weeks to months if not corrected.<br>**Conclusion**<br>Based on the provided clinical information, **Vitamin B6 Deficiency Neuropathy** emerges as the most probable diagnosis. This conclusion is drawn from the specific finding of decreased serum vitamin B6 levels combined with a clinical picture that fits the sensory and progressive nature of the neuropathy described. Replenishment of vitamin B6 and monitoring of the patient’s response to treatment would be the next appropriate steps in management.
10	A 76-year-old female patient presents with a 2-year history of sensory-motor, symmetrical, length dependent polyneuropathy with subacute onset. The patient presents with paresthesia, neuropathic pain, cramps, orthostatic hypotension, and weakness of toe dorsiflexion. There are no foot deformities, cranial nerve involvement, ataxia, or tremor. Past medical history is positive for chronic renal insufficiency and multiple myeloma, for which the patient has undergone chemotherapy. Neuropathy symptoms started at the end of the chemotherapy treatment cycles. Nerve conduction studies show a symmetric, pure sensory, axonal polyneuropathy and unilateral carpal tunnel syndrome. Screening lab tests including vitamin B12, folate, vitamin B6, vitamin E, HBV, HCV, ANA, ANCA, ENA, CRP, ESR, glycemia, HbA1c, copper and cryoglobulins are all negative, with positivity for IgG kappa paraprotein. There is no family history of polyneuropathy.	Chemotherapy-induced neuropathy	**Summary of Pertinent Clinical Information**<br>● **Patient**: 76-year-old female<br>● **Symptoms**:<br>○ 2-year history of sensory-motor, symmetrical, length-dependent polyneuropathy with subacute onset<br>○ Paresthesia<br>○ Neuropathic pain<br>○ Cramps<br>○ Orthostatic hypotension<br>○ Weakness of toe dorsiflexion<br>● **Absences**:<br>○ No foot deformities<br>○ No cranial nerve involvement<br>○ No ataxia<br>○ No tremor<br>● **Past Medical History**:<br>○ Chronic renal insufficiency<br>○ Multiple myeloma treated with chemotherapy<br>● **Onset**: Neuropathy symptoms began at the end of chemotherapy cycles<br>● **Nerve Conduction Studies**:<br>○ Symmetric, pure sensory, axonal polyneuropathy<br>○ Unilateral carpal tunnel syndrome<br>● **Laboratory Tests**:<br>○ Negative for vitamin B12, folate, vitamin B6, vitamin E, HBV, HCV, ANA, ANCA, ENA, CRP, ESR, glycemia, HbA1c, copper, and cryoglobulins<br>○ Positive for IgG kappa paraprotein<br>● **Family History**: No family history of polyneuropathy<br>**Differential Diagnoses for Polyneuropathy**<br>1. **Chemotherapy-Induced Peripheral Neuropathy (CIPN)**<br>○ **Reasoning**:<br>■ Temporal relationship with the onset of neuropathy symptoms and chemotherapy treatment.<br>■ Common in multiple myeloma patients treated with chemotherapeutic agents like bortezomib, thalidomide, or vincristine, which are known to cause neurotoxic effects.<br>■ The presence of sensory symptoms (paresthesia, pain) and motor symptoms (toe dorsiflexion weakness) aligns with the known clinical presentation of CIPN.<br>■ Axonal neuropathy seen in nerve conduction studies is a typical finding in CIPN.<br>2. **Monoclonal Gammopathy of Undetermined Significance (MGUS) - related neuropathy**<br>○ **Reasoning**:<br>■ Presence of IgG kappa paraprotein is indicative of a monoclonal gammopathy, which can be associated with peripheral neuropathy.<br>■ MGUS-related neuropathy can present with similar sensory and motor symptoms and often involves a length-dependent pattern.<br>■ The chronicity and progression of symptoms over two years fit the pattern seen in MGUS-associated neuropathy.<br>■ Axonal involvement on nerve conduction studies aligns with findings in MGUS-related neuropathy.

			3. **Uremic Neuropathy**<br>○ **Reasoning**:<br>■ Chronic renal insufficiency is a known risk factor for uremic neuropathy.<br>■ Uremic neuropathy typically presents as a symmetrical, length-dependent sensory and motor polyneuropathy.<br>■ Symptoms such as paresthesia, cramps, and weakness are common in uremic neuropathy.<br>■ The presence of orthostatic hypotension can be related to autonomic dysfunction, which is seen in uremic neuropathy.<br>■ The patient's renal function impairment could contribute to the neuropathic symptoms observed.<br>**Most Likely Diagnosis**<br>The most likely diagnosis is **Chemotherapy-Induced Peripheral Neuropathy (CIPN)** due to the clear temporal relationship between the onset of symptoms and the completion of chemotherapy cycles. The clinical presentation, including sensory and motor symptoms, fits well with CIPN, and the axonal nature of the neuropathy seen in nerve conduction studies supports this diagnosis. Additionally, while MGUS-related neuropathy and uremic neuropathy are possible, the direct association with recent chemotherapy makes CIPN the leading diagnosis.
11	A 76-year-old female patient presents with a 1-year history of sensory-motor multineuropathy with subacute onset. The patient presents with paresthesia and weakness in a multifocal distribution, without ataxia, neuropathic pain, tremor, foot deformities, cranial nerve or autonomic involvement. Past medical history shows diabetes, anaemia, chronic renal insufficiency, undifferentiated connective tissue disease, osteoarthritis and ANCA vasculitis. Nerve conduction studies show a sensory-motor, axonal multineuropathy. Screening lab tests including vitamin B12, folate, vitamin B6, vitamin E, HCV, HBV, ENA, CRP, ESR, glycemia, HbA1c, copper, cryoglobulins and serum electrophoresis are all negative, with positivity for ANA and ANCA autoantibodies. There is no family history of polyneuropathy.	Vasculitic neuropathy	**Clinical Information Summary**<br>**Patient Details:**<br>● **Age:** 76<br>● **Gender:** Female<br>● **Duration of Symptoms:** 1 year<br>● **Symptom Onset:** Subacute<br>● **Symptoms:**<br>○ Paresthesia<br>○ Weakness in a multifocal distribution<br>● **Absent Symptoms:**<br>○ Ataxia<br>○ Neuropathic pain<br>○ Tremor<br>○ Foot deformities<br>○ Cranial nerve involvement<br>○ Autonomic involvement<br>**Past Medical History:**<br>● Diabetes<br>● Anemia<br>● Chronic renal insufficiency<br>● Undifferentiated connective tissue disease<br>● Osteoarthritis<br>● ANCA vasculitis<br>**Diagnostic Findings:**<br>● **Nerve Conduction Studies:** Sensory-motor, axonal multineuropathy<br>● **Screening Lab Tests:** Negative for vitamin B12, folate, vitamin B6, vitamin E, HCV, HBV, ENA, CRP, ESR, glycemia, HbA1c, copper, cryoglobulins, serum electrophoresis<br>● **Positive Findings:** ANA and ANCA autoantibodies<br>● **Family History:** No family history of polyneuropathy<br>**Differential Diagnoses for Polyneuropathy**<br>1. **Diabetic Neuropathy:**<br>○ **Reasoning:** The patient has a history of diabetes, which is a common cause of polyneuropathy. Diabetic neuropathy often presents with sensory-motor symptoms and can have a subacute or chronic onset. However, the negative HbA1c and glycemia tests make this less likely, suggesting good glycemic control or a non-diabetic cause for the neuropathy.<br>2. **Vasculitic Neuropathy (ANCA-Associated Vasculitis):**<br>○ **Reasoning:** The presence of ANCA vasculitis in the patient's history and positive ANCA autoantibodies suggests a vasculitic process. Vasculitic neuropathy typically presents with a sensory-motor, axonal pattern and can be multifocal due to the patchy nature of blood vessel involvement. The absence of systemic symptoms (like fever or weight loss) and a lack of response to common vasculitis treatments would need to be considered, but this remains a strong possibility given the patient's history.<br>3. **Chronic Inflammatory Demyelinating Polyneuropathy (CIDP):**<br>○ **Reasoning:** CIDP is a chronic inflammatory condition that can present with sensory-motor polyneuropathy. While typically demyelinating in nature, axonal forms can occur. The multifocal distribution of weakness and the chronic course are consistent with CIDP. The presence of ANA autoantibodies could indicate an overlap with an autoimmune condition, although CIDP itself typically does not involve ANCA positivity.<br>**Most Likely Diagnosis**<br>**Vasculitic Neuropathy (ANCA-Associated Vasculitis):**<br>● **Detailed Reasoning:** Considering the patient's history of ANCA vasculitis, positive ANCA autoantibodies, and the multifocal distribution of sensory-motor symptoms, vasculitic neuropathy is the most likely diagnosis. The axonal nature of the neuropathy and the patient's chronic renal insufficiency further support this. The absence of other systemic symptoms can occur in localized vasculitis, and the chronicity aligns with the patient's presentation.<br>This diagnosis should be further evaluated with a focus on confirming vasculitic activity through potential biopsy of affected nerves or muscles, imaging to assess vascular involvement, and possibly starting or adjusting immunosuppressive therapy based on current disease activity.
12	An 18-year old male patient presents with a 16-year history of sensory-motor, symmetrical, length dependent polyneuropathy with insidious onset and slow progression. The patient presents with sensory ataxia, distal weakness in the lower limbs, severe calf atrophy, pes	CMT2	**Summary of Clinical Information:**<br>● **Patient Information:** 18-year-old male<br>● **History:** 16-year history of sensory-motor, symmetrical, length-dependent polyneuropathy with insidious onset and slow progression<br>● **Symptoms:**<br>○ Sensory ataxia<br>○ Distal weakness in the lower limbs<br>○ Severe calf atrophy

	planus, and hammertoes with no cranial nerve or autonomic involvement. Past medical history is silent. Nerve conduction studies show an axonal, sensory-motor polyneuropathy. Screening lab tests including vitamin B12, folate, vitamin B6, vitamin E, HBV, HCV, glycemia, HbA1c, ANA, ANCA, ENA, CRP, ESR, copper, cryoglobulins, and serum electrophoresis are all negative. Her mother and grandmother have walking difficulties. Her 5-year-old sister has pes cavus.		○ Pes planus (flat feet)<br>○ Hammertoes<br>● **Examination Findings:** No cranial nerve or autonomic involvement<br>● **Past Medical History:** Silent<br>● **Family History:**<br>○ Mother and grandmother have walking difficulties<br>○ 5-year-old sister has pes cavus (high-arched feet)<br>● **Nerve Conduction Studies:** Axonal, sensory-motor polyneuropathy<br>● **Screening Lab Tests:** All negative (including vitamins, infectious markers, autoimmune markers, glycemic control, inflammatory markers, copper, cryoglobulins, and serum electrophoresis)<br>**Differential Diagnoses:**<br>1. **Charcot-Marie-Tooth Disease (CMT):**<br>○ **Reasoning:**<br>■ **Clinical Features:** CMT typically presents with a similar history of sensory-motor polyneuropathy, distal muscle weakness, atrophy, and foot deformities (pes cavus, pes planus, hammertoes).<br>■ **Family History:** The presence of walking difficulties in the patient's mother and grandmother, and pes cavus in the sister, suggest a hereditary pattern.<br>■ **Nerve Conduction Studies:** The axonal type of polyneuropathy fits with certain types of CMT (particularly CMT2, which is an axonal variant).<br>■ **Slow Progression:** CMT often has a chronic and slowly progressive course.<br>2. **Hereditary Sensory and Autonomic Neuropathy (HSAN):**<br>○ **Reasoning:**<br>■ **Clinical Features:** HSAN can present with sensory ataxia and motor involvement. Although autonomic involvement is typical, some subtypes may predominantly affect sensory and motor nerves.<br>■ **Family History:** The hereditary aspect fits with the patient's family history.<br>■ **Nerve Conduction Studies:** Axonal polyneuropathy is consistent with HSAN.<br>■ **Onset and Progression:** Insidious onset and slow progression are characteristic of some HSAN subtypes.<br>3. **Refsum Disease:**<br>○ **Reasoning:**<br>■ **Clinical Features:** Refsum disease is characterized by sensory-motor polyneuropathy, ataxia, and distal weakness. However, it also typically presents with retinitis pigmentosa, hearing loss, and cardiomyopathy, which are not mentioned in this case.<br>■ **Family History:** It is a hereditary disorder, which fits the family history.<br>■ **Nerve Conduction Studies:** Axonal polyneuropathy is seen in Refsum disease.<br>■ **Screening Lab Tests:** Although not specifically mentioned, plasma phytanic acid levels are required for diagnosis and would not be covered by the typical lab tests listed.<br>**Most Likely Diagnosis:**<br>**Charcot-Marie-Tooth Disease (CMT):**<br>Given the clinical presentation of a long history of sensory-motor polyneuropathy, distal muscle weakness, severe calf atrophy, foot deformities (pes planus and hammertoes), and the strong family history of similar symptoms, Charcot-Marie-Tooth Disease is the most likely diagnosis. The axonal type seen in nerve conduction studies aligns with CMT2. The lack of cranial nerve or autonomic involvement further supports this diagnosis, as CMT typically does not involve these systems. A genetic test to confirm specific mutations associated with CMT would be the next step for a definitive diagnosis.
13	An 82-year-old female patient presents with a 1-year history of sensory-motor, symmetrical, length dependent polyneuropathy with subacute onset and progressive course. The patient presents with paraesthesia and neuropathic pain in the hands and feet and bilateral foot drop. Clinical examination excludes sensory ataxia, cranial nerve or autonomic involvement, tremor or foot deformities. Past medical history is negative. Nerve conduction studies show a demyelinating, sensory-motor, symmetric polyneuropathy. Screening lab tests reveal presence of polycythaemia, IgG lambda paraprotein, positivity for ANA, hyperglycemia, and elevated HbA1c. Remaining exams including vitamin B12, folate, vitamin B6, vitamin E, ANCA, CRP, ESR, copper, and cryoglobulins are negative. There is no family history of polyneuropathy.	POEMS	**Summary of Clinical Information**<br>**Patient Demographics:**<br>● Age: 82 years<br>● Gender: Female<br>**Chief Complaints:**<br>● 1-year history of sensory-motor, symmetrical, length-dependent polyneuropathy<br>● Subacute onset with progressive course<br>● Paraesthesia and neuropathic pain in hands and feet<br>● Bilateral foot drop<br>**Clinical Examination:**<br>● No sensory ataxia<br>● No cranial nerve involvement<br>● No autonomic involvement<br>● No tremor<br>● No foot deformities<br>**Past Medical History:**<br>● Negative for other significant conditions<br>**Nerve Conduction Studies:**<br>● Demyelinating, sensory-motor, symmetric polyneuropathy<br>**Laboratory Findings:**<br>● Polycythaemia<br>● Presence of IgG lambda paraprotein<br>● Positive ANA (antinuclear antibody)<br>● Hyperglycemia<br>● Elevated HbA1c<br>**Negative Laboratory Findings:**<br>● Vitamin B12, folate, vitamin B6, vitamin E<br>● ANCA (antineutrophil cytoplasmic antibodies)<br>● CRP (C-reactive protein)

			● ESR (erythrocyte sedimentation rate)<br>● Copper<br>● Cryoglobulins<br>**Family History:**<br>● No family history of polyneuropathy<br>**Differential Diagnoses for Polyneuropathy**<br>1. **Diabetic Polyneuropathy**<br>2. **Paraproteinemic Neuropathy**<br>3. **Chronic Inflammatory Demyelinating Polyneuropathy (CIDP)**<br>**Detailed Reasoning for Differential Diagnoses**<br>1. **Diabetic Polyneuropathy:**<br>○ **Clinical Reasoning:** The patient presents with hyperglycemia and elevated HbA1c, which are indicative of diabetes mellitus. Diabetic polyneuropathy is a common complication of diabetes, characterized by a symmetrical, length-dependent pattern of sensory and motor nerve involvement, which matches the patient's presentation.<br>○ **Supporting Evidence:**<br>■ Hyperglycemia<br>■ Elevated HbA1c<br>○ **Counterpoints:** The presence of other laboratory abnormalities such as IgG lambda paraprotein and ANA positivity suggests that diabetes alone might not fully explain the neuropathy.<br>2. **Paraproteinemic Neuropathy:**<br>○ **Clinical Reasoning:** The presence of IgG lambda paraprotein indicates a possible monoclonal gammopathy, which can be associated with neuropathy. Paraproteinemic neuropathies are often demyelinating and can present with sensory and motor deficits.<br>○ **Supporting Evidence:**<br>■ IgG lambda paraprotein<br>■ Demyelinating neuropathy on nerve conduction studies<br>○ **Counterpoints:** While paraproteinemic neuropathy is likely, it does not explain the hyperglycemia and elevated HbA1c, suggesting a potential multifactorial etiology.<br>3. **Chronic Inflammatory Demyelinating Polyneuropathy (CIDP):**<br>○ **Clinical Reasoning:** CIDP is a chronic, progressive, demyelinating polyneuropathy. It typically presents with sensory and motor involvement and can be symmetrical. Although CIDP usually presents with more prominent motor symptoms, it remains a differential to consider given the demyelinating nature of the neuropathy and the presence of ANA positivity, which may indicate an autoimmune component.<br>○ **Supporting Evidence:**<br>■ Demyelinating nature of the neuropathy<br>■ ANA positivity (suggestive of an autoimmune process)<br>○ **Counterpoints:** The lack of cranial nerve or autonomic involvement and the presence of IgG lambda paraprotein might make CIDP less likely compared to paraproteinemic neuropathy.<br>**Most Likely Diagnosis**<br>The most likely diagnosis for this patient is **Paraproteinemic Neuropathy**. This conclusion is based on the presence of IgG lambda paraprotein, which strongly suggests a monoclonal gammopathy, and the demyelinating nature of the neuropathy observed in nerve conduction studies. Although the patient also has hyperglycemia and elevated HbA1c, which suggest diabetes mellitus, the specific finding of IgG lambda paraprotein cannot be ignored and strongly supports a paraproteinemic etiology.<br>Further diagnostic work-up, including additional hematologic evaluation and possibly a bone marrow biopsy, may be warranted to confirm the underlying cause of the paraproteinemia and guide appropriate treatment.
14	A 22-year old male patient presents with a 20-year history of chronic, sensory-motor, symmetrical, length dependent polyneuropathy with insidious onset and slow progression. The patient presents with severe distal weakness and atrophy in the four limbs, hypoesthesia up to calves, pes planus, and scoliosis with no cranial nerve or autonomic involvement. He uses a rollator for walking. Past medical history is silent. Nerve conduction studies show demyelinating, symmetric, sensory-motor polyneuropathy. Screening lab tests including vitamin B12, folate, vitamin B6, vitamin E, HBV, HCV, glycemia, HbA1c, ANA, ANCA, ENA, CRP, ESR, copper, cryoglobulins, and serum electrophoresis are all negative. There is no family history of polyneuropathy.	CMT4	**Summary of Pertinent Clinical Information**<br>● **Patient**: 22-year-old male<br>● **History**:<br>○ 20-year history of chronic, sensory-motor, symmetrical, length-dependent polyneuropathy<br>○ Insidious onset and slow progression<br>● **Symptoms**:<br>○ Severe distal weakness and atrophy in all four limbs<br>○ Hypoesthesia up to the calves<br>○ Pes planus (flat feet)<br>○ Scoliosis<br>○ Uses a rollator for walking<br>○ No cranial nerve or autonomic involvement<br>● **Nerve Conduction Studies**: Demyelinating, symmetric, sensory-motor polyneuropathy<br>● **Laboratory Tests**: Negative results for vitamin B12, folate, vitamin B6, vitamin E, HBV, HCV, glycemia, HbA1c, ANA, ANCA, ENA, CRP, ESR, copper, cryoglobulins, and serum electrophoresis<br>● **Family History**: No family history of polyneuropathy<br>● **Past Medical History**: Unremarkable<br>**Differential Diagnoses for Polyneuropathy**<br>1. **Charcot-Marie-Tooth Disease (CMT)**<br>○ **Reasoning**:<br>■ The patient's chronic, length-dependent, symmetrical polyneuropathy with insidious onset and slow progression is highly suggestive of CMT, a hereditary motor and sensory neuropathy.<br>■ Severe distal weakness and atrophy, pes planus, and scoliosis are common in CMT.<br>■ Demyelinating neuropathy seen in nerve conduction studies is characteristic of CMT type 1.<br>■ Lack of family history does not exclude CMT as new mutations can occur, or family members might have subclinical disease.<br>■ Absence of cranial nerve and autonomic involvement aligns with CMT.<br>2. **Chronic Inflammatory Demyelinating Polyneuropathy (CIDP)**<br>○ **Reasoning**:<br>■ CIDP can present with a chronic, progressive course and can be symmetrical and length-dependent.

			■ Demyelinating polyneuropathy on nerve conduction studies is consistent with CIDP.<br>■ Severe distal weakness and atrophy fit with CIDP presentations.<br>■ However, the very slow progression over 20 years and the absence of inflammatory markers (CRP, ESR) or response to treatment usually seen in CIDP make this less likely compared to hereditary causes.<br>3. **Hereditary Neuropathy with Liability to Pressure Palsies (HNPP)**<br>○ **Reasoning**:<br>■ HNPP can present with features of demyelinating polyneuropathy and has hereditary transmission, although it is typically more characterized by recurrent focal neuropathies rather than a purely length-dependent polyneuropathy.<br>■ The absence of family history can occur with new mutations or variable expressivity.<br>■ The patient's presentation is more suggestive of a continuous process rather than the episodic nature of HNPP, making this a less likely but still possible diagnosis.<br>**Most Likely Diagnosis**<br>Given the patient's history of a 20-year chronic, slowly progressive sensory-motor, symmetrical polyneuropathy, severe distal weakness and atrophy, pes planus, scoliosis, demyelinating features on nerve conduction studies, and negative screening lab tests for common causes of acquired neuropathies, the most likely diagnosis is **Charcot-Marie-Tooth Disease (CMT)**.<br>CMT, specifically CMT type 1, fits well with the clinical presentation and electrophysiological findings. Although there is no family history, the possibility of a de novo mutation or subclinical disease in family members should be considered. Genetic testing for common CMT mutations (e.g., PMP22 duplication/deletion) would be the next step to confirm the diagnosis.
15	A 72-year-old female patient presents with a 2-year history of pure sensory, symmetrical, length dependent polyneuropathy with insidious onset. The patient presents with distal paresthesia and neuropathic pain in the upper and lower limbs in a stocking and glove distribution, without ataxia or tremor, foot deformities, cranial nerve or autonomic involvement. Past medical history shows multiple myeloma, for which the patient has undergone chemotherapy. Neuropathy symptoms started 2 months after chemotherapy. Nerve conduction studies show pure sensory, symmetric, axonal polyneuropathy. Screening lab tests including vitamin B12, folate, vitamin B6, vitamin E, HBV, HCV, ANA, ANCA, ENA, CRP, ESR, glycemia, HbA1c, copper and cryoglobulins are all negative, with positivity for IgG kappa paraprotein. There is no family history of polyneuropathy.	Chemotherapy-induced neuropathy	**Clinical Summary**<br>● **Patient**: 72-year-old female<br>● **History**: 2-year history of pure sensory, symmetrical, length-dependent polyneuropathy with insidious onset<br>● **Symptoms**:<br>○ Distal paresthesia and neuropathic pain in upper and lower limbs<br>○ Stocking and glove distribution<br>○ No ataxia, tremor, foot deformities, cranial nerve or autonomic involvement<br>● **Past Medical History**: Multiple myeloma treated with chemotherapy<br>● **Onset of Symptoms**: 2 months after starting chemotherapy<br>● **Nerve Conduction Studies**: Pure sensory, symmetric, axonal polyneuropathy<br>● **Screening Lab Results**:<br>○ Normal: Vitamin B12, folate, vitamin B6, vitamin E, HBV, HCV, ANA, ANCA, ENA, CRP, ESR, glycemia, HbA1c, copper, cryoglobulins<br>○ Positive: IgG kappa paraprotein<br>● **Family History**: No family history of polyneuropathy<br>**Differential Diagnoses**<br>1. **Chemotherapy-Induced Peripheral Neuropathy (CIPN)**<br>○ **Reasoning**: The patient has a history of multiple myeloma and chemotherapy, with neuropathy symptoms starting 2 months post-chemotherapy. CIPN is a common side effect of many chemotherapeutic agents, particularly those used in multiple myeloma, such as bortezomib, thalidomide, and vincristine. The presentation of pure sensory, symmetrical, length-dependent polyneuropathy is consistent with CIPN.<br>2. **Monoclonal Gammopathy of Undetermined Significance (MGUS) Neuropathy**<br>○ **Reasoning**: The presence of IgG kappa paraprotein suggests a monoclonal gammopathy, which is often associated with neuropathy. MGUS can lead to a similar clinical presentation of sensory, symmetrical polyneuropathy. While this patient has multiple myeloma, the paraproteinemia could still contribute to the neuropathy, independent of the chemotherapy.<br>3. **Multiple Myeloma-Related Neuropathy**<br>○ **Reasoning**: Multiple myeloma itself can cause peripheral neuropathy, typically through direct nerve infiltration, paraneoplastic syndromes, or amyloidosis. However, the patient's normal screening labs, including those for cryoglobulins and other specific markers, make this less likely compared to the other diagnoses. Nevertheless, the presence of multiple myeloma and the onset of neuropathy post-chemotherapy keep this diagnosis in consideration.<br>**Most Likely Diagnosis**<br>**Chemotherapy-Induced Peripheral Neuropathy (CIPN)** is the most likely diagnosis for the following reasons:<br>● The temporal relationship between the onset of neuropathy and chemotherapy is strong, with symptoms starting 2 months post-chemotherapy.<br>● The type of neuropathy (pure sensory, symmetric, axonal) aligns well with common presentations of CIPN.<br>● The negative lab results for other potential causes and the absence of family history of polyneuropathy further support this diagnosis.<br>Thus, considering the clinical history, symptoms, and diagnostic findings, chemotherapy-induced peripheral neuropathy is the most probable cause of the patient's condition.
16	A 59-year-old female patient presents with a 1-year history of pure sensory, symmetrical, non-length dependent polyneuropathy with insidious onset. The patient presents with paresthesia distributed proximally and distally in the upper and lower limbs in an asymmetric fashion, and sensory ataxia. There are no foot deformities, tremor, neuropathic pain, cranial nerve or autonomic involvement. Past medical history is positive for IgG lambda monoclonal gammopathy and lung cancer, for which she underwent chemotherapy 4 years ago. Nerve conduction studies show a demyelinating, non-length dependent, sensory-motor polyneuropathy with conduction blocks. Screening lab tests including vitamin B12,	CIDP	**Clinical Summary**<br>**Patient Information:**<br>● **Age:** 59-year-old female<br>● **History:** 1-year history of pure sensory, symmetrical, non-length dependent polyneuropathy with insidious onset<br>● **Symptoms:**<br>○ Paresthesia distributed proximally and distally in the upper and lower limbs in an asymmetric fashion<br>○ Sensory ataxia<br>○ No foot deformities, tremor, neuropathic pain, cranial nerve or autonomic involvement<br>**Past Medical History:**<br>● IgG lambda monoclonal gammopathy<br>● Lung cancer (treated with chemotherapy 4 years ago)<br>**Nerve Conduction Studies:**<br>● Demyelinating, non-length dependent, sensory-motor polyneuropathy with conduction blocks<br>**Laboratory Tests:**<br>● Negative: Vitamin B12, folate, vitamin B6, vitamin E, HCV, ANA, ANCA, ENA, CRP, ESR, glycemia, HbA1c, copper, cryoglobulins<br>● Positive: IgG lambda paraprotein, chronic HBV infection<br>**Family History:**<br>● No family history of polyneuropathy

	folate, vitamin B6, vitamin E, HCV, ANA, ANCA, ENA, CRP, ESR, glycemia, HbA1c, copper and cryoglobulins are all negative, with positivity for IgG lambda paraprotein and evidence of chronic HBV infection. There is no family history of polyneuropathy.		**Differential Diagnosis**<br>1. **Monoclonal Gammopathy of Undetermined Significance (MGUS) associated Neuropathy:**<br>○ **Reasoning:**<br>■ The patient has a history of IgG lambda monoclonal gammopathy.<br>■ MGUS can be associated with demyelinating neuropathy, often presenting with sensory symptoms and ataxia.<br>■ The nerve conduction studies showing demyelination and conduction blocks are consistent with neuropathy associated with monoclonal gammopathy.<br>2. **Paraneoplastic Neuropathy:**<br>○ **Reasoning:**<br>■ The patient has a history of lung cancer treated with chemotherapy 4 years ago.<br>■ Paraneoplastic syndromes can present with neuropathies, often with sensory symptoms and ataxia.<br>■ The insidious onset and non-length dependent nature of the neuropathy can be associated with a paraneoplastic process.<br>■ However, the lack of recent cancer activity or evidence of recurrence makes this less likely but still plausible.<br>3. **Chronic Inflammatory Demyelinating Polyneuropathy (CIDP):**<br>○ **Reasoning:**<br>■ CIDP often presents with symmetrical, sensory, and motor polyneuropathy.<br>■ The nerve conduction studies showing demyelinating polyneuropathy with conduction blocks are characteristic of CIDP.<br>■ The chronic progressive nature of symptoms over one year is consistent with CIDP.<br>■ Although there is no evidence of cranial nerve involvement or autonomic symptoms, CIDP can present with pure sensory manifestations.<br>**Most Likely Diagnosis**<br>**Monoclonal Gammopathy of Undetermined Significance (MGUS) associated Neuropathy:**<br>● The presence of IgG lambda monoclonal gammopathy strongly suggests this diagnosis.<br>● The clinical presentation and nerve conduction study findings are highly consistent with neuropathy associated with MGUS.<br>● While paraneoplastic neuropathy and CIDP are also considerations, the specific association with IgG lambda monoclonal gammopathy makes MGUS-related neuropathy the most probable diagnosis in this case.<br>**Conclusion**<br>The most likely diagnosis for this 59-year-old female patient is MGUS-associated neuropathy, given her clinical presentation, history of IgG lambda monoclonal gammopathy, and consistent nerve conduction study findings.
17	A 73-year-old female patient presents with a 1-year history of sensory-motor, symmetrical, polyneuropathy with subacute onset. The patient presents with distal paresthesia and neuropathic pain in the four limbs, sensory ataxia, cramps, orthostatic hypotension and diarrhoea but with no cranial nerve involvement or foot deformities. Clinical examination shows cutaneous angiomas and hyperpigmentation. Laboratory tests reveal presence of thrombocytosis and IgG lambda monoclonal gammopathy. Nerve conduction studies show a symmetric, sensory-motor, demyelinating-axonal polyneuropathy. Screening lab tests including vitamin B12, folate, vitamin B6, vitamin E, HCV, HBV, ANA, ANCA, ENA, CRP, ESR, glycemia, HbA1c, copper and cryoglobulins are negative, with positivity for IgG lambda paraprotein. There is no family history of polyneuropathy.	POEMS	**Summary of Pertinent Clinical Information**<br>● **Patient Demographics:** 73-year-old female<br>● **History:**<br>○ Duration: 1 year<br>○ Type: Sensory-motor, symmetrical, polyneuropathy with subacute onset<br>○ Symptoms: Distal paresthesia, neuropathic pain in four limbs, sensory ataxia, cramps, orthostatic hypotension, and diarrhea<br>○ No cranial nerve involvement or foot deformities<br>● **Clinical Examination:**<br>○ Cutaneous angiomas<br>○ Hyperpigmentation<br>● **Laboratory Findings:**<br>○ Thrombocytosis<br>○ IgG lambda monoclonal gammopathy<br>○ Negative screening tests: vitamin B12, folate, vitamin B6, vitamin E, HCV, HBV, ANA, ANCA, ENA, CRP, ESR, glycemia, HbA1c, copper, and cryoglobulins<br>○ Positive: IgG lambda paraprotein<br>● **Nerve Conduction Studies:** Symmetric, sensory-motor, demyelinating-axonal polyneuropathy<br>● **Family History:** No family history of polyneuropathy<br>**Differential Diagnoses for Polyneuropathy**<br>1. **Monoclonal Gammopathy of Undetermined Significance (MGUS) with Peripheral Neuropathy**<br>○ **Reasoning:**<br>■ Presence of IgG lambda monoclonal gammopathy suggests an underlying plasma cell disorder.<br>■ MGUS can be associated with a range of neuropathic symptoms, including sensory-motor polyneuropathy.<br>■ Demyelinating-axonal features on nerve conduction studies align with the neuropathy seen in MGUS-related cases.<br>■ The absence of systemic disease or malignancy supports MGUS as a potential diagnosis.<br>■ Other laboratory tests are negative, ruling out other common causes of neuropathy.<br>2. **Primary Systemic Amyloidosis (AL Amyloidosis)**<br>○ **Reasoning:**<br>■ Amyloidosis is associated with monoclonal gammopathy (IgG lambda in this case).<br>■ Cutaneous angiomas and hyperpigmentation can be seen in amyloidosis due to amyloid deposits in the skin.<br>■ Neuropathic pain, sensory ataxia, and autonomic dysfunction (orthostatic hypotension, diarrhea) are characteristic of amyloidosis.<br>■ Thrombocytosis can occur in amyloidosis.<br>■ The demyelinating-axonal nature of the neuropathy fits with amyloid involvement of the nerves.<br>■ Further investigation for amyloid deposits (e.g., fat pad biopsy) would be necessary to confirm this diagnosis.<br>3. **Chronic Inflammatory Demyelinating Polyneuropathy (CIDP)**<br>○ **Reasoning:**<br>■ CIDP typically presents with symmetrical, sensory-motor polyneuropathy.<br>■ The subacute onset and progressive nature are consistent with CIDP.<br>■ Demyelinating features on nerve conduction studies are a hallmark of CIDP.

			■ CIDP can be associated with monoclonal gammopathy (paraproteinemic neuropathy).<br>■ While the presence of IgG lambda monoclonal gammopathy is not specific, it can be a paraneoplastic or autoimmune marker.<br>■ The lack of response to typical inflammatory markers (negative ANA, ANCA, CRP, ESR) makes this less likely but still plausible.<br>**Most Likely Diagnosis**<br>Based on the clinical presentation, laboratory findings, and nerve conduction studies, the most likely diagnosis is **Primary Systemic Amyloidosis (AL Amyloidosis)**. The presence of IgG lambda monoclonal gammopathy, cutaneous angiomas, hyperpigmentation, neuropathic pain, autonomic dysfunction, and the specific pattern of neuropathy strongly suggest amyloidosis. Further confirmatory tests, such as a fat pad biopsy to detect amyloid deposits, would be critical to establish this diagnosis definitively.
18	A 55-year-old male patient presents with a 5-month history of pure sensory, asymmetric, non-length dependent polyneuropathy with subacute onset and progressive course. The patient presents with paresthesia and hypoesthesia in the distal areas of the four limbs, severe sensory ataxia, orthostatic hypotension, and delayed gastric and intestinal emptying. The patient also reports xerostomia and xerophthalmia. Clinical examination reveals reduced pupillary reflex and anhidrosis. There are no neuropathic pain, tremor, foot deformities or cranial nerve involvement. Past medical history is positive for Hashimoto's thyroiditis. Nerve conduction studies show a pure sensory, non-length dependent, axonal polyneuropathy. Screening lab tests reveal folate deficiency and low-titre ENA positivity. Remaining exams including vitamin B12, vitamin B6, vitamin E, HBV, HCV, ANA, ANCA, CRP, ESR, copper, glycemia, HbA1c, cryoglobulins, and serum electrophoresis are normal. There is no family history of polyneuropathy.	Sjogren-associated ganglionopathy	**Summary of Pertinent Clinical Information**<br>● **Patient Profile**: 55-year-old male<br>● **Chief Complaint**: 5-month history of pure sensory, asymmetric, non-length dependent polyneuropathy<br>● **Symptom Description**:<br>○ Paresthesia and hypoesthesia in distal areas of all four limbs<br>○ Severe sensory ataxia<br>○ Autonomic symptoms: orthostatic hypotension, delayed gastric and intestinal emptying, xerostomia, xerophthalmia<br>○ Reduced pupillary reflex, anhidrosis<br>● **Absent Symptoms**: Neuropathic pain, tremor, foot deformities, cranial nerve involvement<br>● **Past Medical History**: Hashimoto's thyroiditis<br>● **Nerve Conduction Studies**: Pure sensory, non-length dependent, axonal polyneuropathy<br>● **Lab Findings**:<br>○ Positive: Folate deficiency, low-titre ENA positivity<br>○ Normal: Vitamin B12, Vitamin B6, Vitamin E, HBV, HCV, ANA, ANCA, CRP, ESR, copper, glycemia, HbA1c, cryoglobulins, serum electrophoresis<br>● **Family History**: No family history of polyneuropathy<br>**Differential Diagnoses for Polyneuropathy**<br>1. **Sjogren's Syndrome-Associated Neuropathy**<br>○ **Reasoning**: Sjogren's syndrome is an autoimmune disorder that frequently presents with xerostomia (dry mouth) and xerophthalmia (dry eyes), which the patient reports. Autonomic dysfunctions like orthostatic hypotension and gastrointestinal symptoms are common in Sjogren's-related neuropathy. The presence of low-titre ENA positivity further supports this possibility, as ENA antibodies are often associated with autoimmune conditions including Sjogren's syndrome. Additionally, Hashimoto's thyroiditis is another autoimmune disease, and patients with one autoimmune condition are at higher risk for others.<br>2. **Paraneoplastic Neuropathy**<br>○ **Reasoning**: Paraneoplastic syndromes can cause a wide range of neurological symptoms due to immune responses elicited by a malignancy. These can include asymmetric and non-length dependent sensory neuropathies with prominent autonomic features like those seen in this patient (orthostatic hypotension, gastrointestinal dysmotility). Although there is no direct evidence of malignancy in the initial workup, the subacute onset and the complex autonomic involvement raise the possibility of a paraneoplastic process. A comprehensive malignancy screening would be warranted.<br>3. **Vitamin B1 (Thiamine) Deficiency**<br>○ **Reasoning**: Thiamine deficiency, also known as beriberi, can cause a sensory polyneuropathy along with autonomic dysfunction, including gastrointestinal issues and orthostatic hypotension. Although this patient's condition is not length-dependent and there is no history of alcohol abuse (a common cause of thiamine deficiency), dietary deficiency or malabsorption syndromes could be considered. The folate deficiency found might hint at potential nutritional deficiencies that could extend to thiamine.<br>**Most Likely Diagnosis**<br>Given the clinical presentation, the combination of autonomic dysfunction, sensory ataxia, dry mouth, and dry eyes, along with the history of autoimmune thyroiditis and low-titre ENA positivity, **Sjogren's syndrome-associated neuropathy** is the most likely diagnosis. This condition encompasses all the patient's symptoms, and the lab findings support an autoimmune etiology. Further testing for specific Sjogren's antibodies (anti-SSA/Ro, anti-SSB/La) and a lip biopsy could confirm the diagnosis.
19	A 70-year-old male patient presents with a 5-year history of sensory-motor, symmetrical, length dependent polyneuropathy with insidious onset and fast disease progression. The patient presents with moderate-to-severe distal weakness and hypoesthesia in the four limbs in a distal and symmetric distribution, sensory ataxia, tongue fasciculations and autonomic involvement with no foot deformities or tremor. Past medical history is positive for cardiac insufficiency. Nerve conduction studies show an axonal, sensory-motor polyneuropathy with bilateral carpal tunnel syndrome. Screening lab tests including vitamin B12, vitamin B9, folate, vitamin E, HBV, HCV, ANA, ANCA, ENA, glycemia, HbA1c, CRP, ESR, copper, cryoglobulins, and serum electrophoresis are all negative. His father, who was wheelchair bound, died from cardiac insufficiency.	TTR-Amyloidosis	**Clinical Summary**<br>● **Patient Profile:** 70-year-old male<br>● **History of Present Illness:** 5-year history of sensory-motor, symmetrical, length-dependent polyneuropathy with insidious onset and rapid progression<br>● **Symptoms:**<br>○ Moderate-to-severe distal weakness<br>○ Hypoesthesia in all four limbs (distal and symmetric)<br>○ Sensory ataxia<br>○ Tongue fasciculations<br>○ Autonomic involvement (details unspecified)<br>● **Past Medical History:** Cardiac insufficiency<br>● **Nerve Conduction Studies:** Axonal, sensory-motor polyneuropathy with bilateral carpal tunnel syndrome<br>● **Screening Lab Results:** Negative for vitamin B12, vitamin B9, folate, vitamin E, HBV, HCV, ANA, ANCA, ENA, glycemia, HbA1c, CRP, ESR, copper, cryoglobulins, and serum electrophoresis<br>● **Family History:** Father, who was wheelchair-bound, died from cardiac insufficiency<br>**Differential Diagnoses for Polyneuropathy**<br>1. **Hereditary Transthyretin Amyloidosis (hATTR)**<br>○ **Reasoning:**<br>■ **Age of Onset:** Although more common in mid-adulthood, it can present in older age.<br>■ **Symptom Profile:** Length-dependent polyneuropathy, autonomic involvement, and cardiac insufficiency are classic features.<br>■ **Family History:** The patient's father had cardiac insufficiency, suggesting a possible genetic link.<br>■ **Nerve Conduction Studies:** Axonal polyneuropathy fits with amyloidosis.<br>■ **Additional Features:** Tongue fasciculations may be indicative of amyloidosis affecting cranial nerves.<br>■ **Exclusion of Other Causes:** Extensive negative lab workup for other causes supports this diagnosis.<br>2. **Chronic Inflammatory Demyelinating Polyneuropathy (CIDP)**<br>○ **Reasoning:**

			■ **Symptom Profile:** Progressive weakness and sensory loss over several years; symmetrical and distal pattern.<br>■ **Nerve Conduction Studies:** Typically shows demyelination, but can sometimes present with axonal features especially in advanced stages.<br>■ **Autonomic Involvement:** Can be seen in CIDP, though less common.<br>■ **Progression and Chronicity:** Fits the description of a chronic and progressively worsening neuropathy.<br>■ **Treatment Response:** CIDP may respond to immunosuppressive therapy, which can be a distinguishing feature.<br>3. **Diabetic Polyneuropathy**<br>○ **Reasoning:**<br>■ **Symptom Profile:** Length-dependent sensory-motor neuropathy, symmetrical and distal, autonomic features.<br>■ **Nerve Conduction Studies:** Axonal pattern is typical.<br>■ **Exclusion Criteria:** Despite the negative glycemia and HbA1c, it's possible early stages or undiagnosed diabetes could contribute.<br>■ **Additional Considerations:** Given the negative lab results, this is less likely but remains a differential due to the commonality of diabetes.<br>**Most Likely Diagnosis**<br>**Hereditary Transthyretin Amyloidosis (hATTR)**<br>**Justification:**<br>● **Family History:** The patient's father had cardiac insufficiency and was wheelchair-bound, suggesting a possible genetic component.<br>● **Cardiac Involvement:** The patient's cardiac insufficiency aligns with hATTR, known to cause both cardiac and neuropathic issues.<br>● **Nerve Conduction and Symptom Profile:** Axonal, sensory-motor polyneuropathy with autonomic involvement fits well with hATTR.<br>● **Negative Lab Tests:** The comprehensive negative screening for other causes strengthens the likelihood of a hereditary condition.<br>To confirm hATTR, genetic testing for TTR mutations and possibly a biopsy (e.g., fat pad, nerve, or cardiac) to identify amyloid deposits would be warranted.
20	A 64-year-old male patient presents with an 18-month history of pure motor, asymmetric, non-length dependent polyneuropathy with insidious onset. The patient presents with weakness and atrophy in a multifocal distribution in the upper limbs. He also reports the presence of subjective paresthesia in the hands but at clinical examination objective tactile sensation is normal. There are no neuropathic pain, ataxia, tremor, foot deformities, cranial nerve or autonomic involvement. Past medical history is silent. Nerve conduction studies show pure motor, demyelinating multineuropathy in the upper limbs. Screening lab tests including vitamin B12, folate, vitamin B6, vitamin E, HCV, HBV, ANA, ANCA, ENA, CRP, ESR, glycemia, HbA1c, copper, cryoglobulins and serum electrophoresis are negative. Cervical spine MRI is normal. There is no family history of polyneuropathy.	MMN	**Summary of Pertinent Clinical Information**<br>● **Patient Demographics**: 64-year-old male<br>● **Duration of Symptoms**: 18 months<br>● **Symptom Characteristics**:<br>○ Pure motor, asymmetric, non-length dependent polyneuropathy<br>○ Insidious onset<br>○ Weakness and atrophy in a multifocal distribution in the upper limbs<br>○ Subjective paresthesia in the hands, but normal objective tactile sensation<br>○ No neuropathic pain, ataxia, tremor, foot deformities, cranial nerve or autonomic involvement<br>● **Past Medical History**: Silent<br>● **Nerve Conduction Studies**: Pure motor, demyelinating multineuropathy in the upper limbs<br>● **Screening Lab Tests**: Negative for vitamin deficiencies (B12, B6, folate, E), HCV, HBV, ANA, ANCA, ENA, CRP, ESR, glycemia, HbA1c, copper, cryoglobulins, serum electrophoresis<br>● **Imaging**: Cervical spine MRI normal<br>● **Family History**: No family history of polyneuropathy<br>**Differential Diagnoses**<br>**1. Multifocal Motor Neuropathy (MMN)**<br>● **Reasoning**:<br>○ **Clinical Features**: MMN often presents as an asymmetric, purely motor neuropathy with multifocal distribution, matching the patient's presentation.<br>○ **Nerve Conduction Studies**: The presence of demyelinating features and multifocal motor conduction block is characteristic of MMN.<br>○ **Negative Labs**: Absence of systemic inflammatory markers and normal cervical MRI aligns with MMN, which typically lacks systemic involvement.<br>○ **Immune-mediated**: MMN is an immune-mediated disorder without sensory loss, fitting the patient's normal tactile sensation despite subjective paresthesia.<br>**2. Amyotrophic Lateral Sclerosis (ALS)**<br>● **Reasoning**:<br>○ **Clinical Features**: ALS can present with asymmetric motor weakness and atrophy, initially affecting the upper limbs in a multifocal pattern.<br>○ **Lack of Sensory Involvement**: The normal tactile sensation and absence of sensory symptoms align with ALS, which primarily affects motor neurons.<br>○ **Progressive Weakness**: The insidious onset and progressive nature of symptoms over 18 months are consistent with ALS.<br>○ **Nerve Conduction Studies**: ALS typically shows motor neuron disease patterns on nerve conduction studies, although demyelinating features are less common.<br>**3. Chronic Inflammatory Demyelinating Polyneuropathy (CIDP) - Multifocal Variant**<br>● **Reasoning**:<br>○ **Clinical Features**: CIDP can present with motor predominant, asymmetric polyneuropathy, particularly in the multifocal variant known as Lewis-Sumner Syndrome.<br>○ **Nerve Conduction Studies**: Demyelinating polyneuropathy fits with CIDP.<br>○ **Lack of Systemic Involvement**: The absence of systemic inflammatory markers and normal screening tests are consistent with CIDP, which may not always show systemic involvement.<br>○ **Immunological Aspect**: CIDP is an immune-mediated disorder, and normal MRI of the cervical spine helps rule out other structural causes.<br>**Most Likely Diagnosis**<br>**Multifocal Motor Neuropathy (MMN)** is the most likely diagnosis based on the following points:<br>● The clinical presentation of asymmetric, purely motor neuropathy with multifocal distribution.<br>● The presence of demyelinating features on nerve conduction studies.<br>● Normal sensory examination despite subjective paresthesia.<br>● Negative screening lab tests and normal cervical spine MRI, excluding other systemic and structural causes.<br>MMN is an immune-mediated disorder that aligns well with the patient's symptomatology and diagnostic findings.
21	A 74-year-old male patient presents with a 3-month history of sensory-motor, asymmetric, length dependent polyneuropathy with subacute onset and	CIDP	**Summary of Pertinent Clinical Information**<br>**Patient Profile:**<br>● Age: 74-year-old male<br>● Presenting with: 3-month history of sensory-motor, asymmetric, length-dependent polyneuropathy

	progressive course. The patient presents with sensory ataxia associated with neuropathic pain, paraesthesia, and hypoesthesia in the feet, and weakness with distal, asymmetric distribution. Clinical examination does not show presence of tremor, foot deformities, cranial nerve or autonomic involvement. Past medical history is positive for multiple myeloma, in remission for the past three years with no need for maintenance therapy (last chemotherapy cycle performed three years ago). Nerve conduction studies show a demyelinating, sensory-motor polyneuropathy. Screening lab tests show presence of IgG lambda paraprotein. Remaining exams including vitamin B12, folate, vitamin B6, vitamin E, HBV, HCV, ANA, ANCA, ENA, CRP, ESR, glycemia, HbA1c, copper, and cryoglobulins are negative. There is no family history of polyneuropathy.		● Symptoms: Sensory ataxia, neuropathic pain, paraesthesia, hypoesthesia in feet, and distal, asymmetric weakness<br>● Onset: Subacute with a progressive course<br>● Clinical Findings: No tremor, foot deformities, cranial nerve or autonomic involvement<br>**Medical History:**<br>● Multiple myeloma: In remission for the past three years, no maintenance therapy required, last chemotherapy cycle three years ago<br>**Investigations:**<br>● Nerve conduction studies: Demyelinating, sensory-motor polyneuropathy<br>● Screening lab tests: Presence of IgG lambda paraprotein<br>● Negative Tests: Vitamin B12, folate, vitamin B6, vitamin E, HBV, HCV, ANA, ANCA, ENA, CRP, ESR, glycemia, HbA1c, copper, and cryoglobulins<br>● Family History: Negative for polyneuropathy<br>**Differential Diagnoses**<br>1. **Monoclonal Gammopathy of Undetermined Significance (MGUS) Related Neuropathy**<br>○ **Reasoning:**<br>■ Presence of IgG lambda paraprotein suggests monoclonal gammopathy.<br>■ Multiple myeloma history aligns with the likelihood of MGUS, particularly in remission.<br>■ Demyelinating pattern on nerve conduction studies fits with neuropathy associated with monoclonal gammopathies.<br>■ MGUS is known to cause sensory-motor polyneuropathy with subacute onset and progressive course, consistent with this patient's presentation.<br>2. **Chronic Inflammatory Demyelinating Polyneuropathy (CIDP)**<br>○ **Reasoning:**<br>■ CIDP can present with a progressive sensory-motor polyneuropathy.<br>■ Demyelinating nerve conduction study findings are characteristic of CIDP.<br>■ Although CIDP often has a more symmetric presentation, it can present asymmetrically in some cases.<br>■ Lack of autonomic involvement and subacute progression also align with CIDP.<br>3. **Chemotherapy-Induced Peripheral Neuropathy (CIPN)**<br>○ **Reasoning:**<br>■ Patient's history of chemotherapy for multiple myeloma three years ago could contribute to persistent or late-onset neuropathy.<br>■ Sensory-motor polyneuropathy with a progressive course can be a residual or delayed effect of chemotherapeutic agents.<br>■ The demyelinating pattern on nerve conduction studies can be seen in CIPN.<br>■ Although typically CIPN is more symmetric, variability in presentation could explain asymmetric findings.<br>**Most Likely Diagnosis**<br>Given the clinical presentation, history of multiple myeloma with current remission, and the presence of IgG lambda paraprotein, the most likely diagnosis is:<br>**Monoclonal Gammopathy of Undetermined Significance (MGUS) Related Neuropathy**<br>**Supporting Points:**<br>● The presence of a monoclonal protein (IgG lambda paraprotein) strongly suggests a monoclonal gammopathy.<br>● MGUS is a common cause of neuropathy in patients with a history of multiple myeloma in remission.<br>● The demyelinating pattern seen in nerve conduction studies is consistent with neuropathies associated with monoclonal gammopathies.<br>● The clinical features of sensory-motor polyneuropathy, including sensory ataxia, neuropathic pain, and paraesthesia, align well with MGUS-related neuropathy.<br>In conclusion, MGUS-related neuropathy is the most probable diagnosis, supported by the patient's history, clinical findings, and investigative results. Further confirmation with serum protein electrophoresis and immunofixation could solidify this diagnosis and help guide management and monitoring.
22	An 82-year-old female patient presents with a 2-year history of pure sensory, symmetrical, length dependent polyneuropathy with insidious onset and slow progressive course. The patient presents with paresthesia, hypoesthesia, and mild neuropathic pain in the feet, associated with diffused cramps in the lower limbs. Clinical examination does not reveal sensory ataxia, tremor, foot deformities, cranial nerve or autonomic involvement. Past medical history is positive for chronic HCV infection, psoriatic arthritis, and fibromyalgia. Nerve conduction studies show presence of a pure sensory, length dependent, symmetric, axonal polyneuropathy with unilateral carpal tunnel syndrome. Screening lab tests including vitamin B12, folate, vitamin B6, vitamin E, HBV, ANCA, ENA, CRP, ESR, copper, cryoglobulins and serum electrophoresis are all negative, with positivity for ANA autoantibodies and	HCV-associated neuropathy	**Step-by-Step Clinical Analysis**<br>**Summarizing Pertinent Clinical Information**<br>● **Patient Demographics:**<br>○ 82-year-old female<br>● **History of Present Illness:**<br>○ 2-year history of pure sensory, symmetrical, length-dependent polyneuropathy<br>○ Insidious onset with a slow progressive course<br>○ Symptoms: paresthesia, hypoesthesia, mild neuropathic pain in the feet, diffused cramps in the lower limbs<br>● **Clinical Examination:**<br>○ No sensory ataxia, tremor, foot deformities, cranial nerve or autonomic involvement<br>● **Past Medical History:**<br>○ Chronic HCV infection<br>○ Psoriatic arthritis<br>○ Fibromyalgia<br>● **Diagnostic Tests:**<br>○ Nerve conduction studies: pure sensory, length-dependent, symmetric, axonal polyneuropathy; unilateral carpal tunnel syndrome<br>○ Screening lab tests: Negative for vitamin B12, folate, vitamin B6, vitamin E, HBV, ANCA, ENA, CRP, ESR, copper, cryoglobulins, and serum electrophoresis<br>○ Positive for ANA autoantibodies<br>○ Evidence of chronic HCV infection<br>● **Family History:**<br>○ No family history of polyneuropathy<br>**Differential Diagnoses for Polyneuropathy**<br>1. **Chronic Hepatitis C Virus (HCV) Infection-Associated Neuropathy:**<br>○ **Reasoning:**

	evidence of chronic HCV infection. There is no family history of polyneuropathy.		■ The patient has a known history of chronic HCV infection, which can be associated with cryoglobulinemia and subsequent peripheral neuropathy.<br>■ Although cryoglobulins were negative in this patient, HCV can still cause neuropathy through immune-mediated mechanisms or direct viral effects.<br>■ The slow progressive course and symmetrical, length-dependent sensory involvement fit the profile of HCV-associated neuropathy.<br>2. **Autoimmune Polyneuropathy (Possibly Sjögren's Syndrome):**<br>○ **Reasoning:**<br>■ Presence of ANA autoantibodies suggests an autoimmune process.<br>■ Sjögren's syndrome is associated with sensory neuropathy and can present with ANA positivity.<br>■ Although there is no mention of sicca symptoms (dry eyes, dry mouth), Sjögren's syndrome can present with neuropathy even in the absence of typical glandular symptoms.<br>3. **Paraneoplastic Neuropathy:**<br>○ **Reasoning:**<br>■ In older adults, paraneoplastic syndromes should be considered, especially with an unexplained polyneuropathy.<br>■ The patient has not been noted to have any signs or symptoms of malignancy, but the presence of ANA autoantibodies raises the possibility of an underlying autoimmune process potentially related to a malignancy.<br>■ This would need further investigation with imaging studies and possibly tumor markers.<br>**Most Likely Diagnosis**<br>The most likely diagnosis in this case is **Chronic HCV Infection-Associated Neuropathy**. This conclusion is based on the combination of the patient's known chronic HCV infection, the fitting clinical profile of slow progressive, symmetrical, sensory polyneuropathy, and the presence of ANA autoantibodies, which can be seen in chronic HCV infection.<br>**Additional Considerations**<br>● **Further Investigation:**<br>○ To confirm the diagnosis and rule out other possible causes, additional tests such as a detailed autoimmune panel, screening for Sjögren's syndrome (e.g., anti-Ro/SSA and anti-La/SSB antibodies), and potentially a paraneoplastic panel could be considered.<br>○ Given the age of the patient, a malignancy screen might also be warranted to exclude paraneoplastic syndrome.<br>This comprehensive analysis ensures a thorough evaluation of the patient's condition and helps in narrowing down the most likely cause of her symptoms while considering other plausible differential diagnoses.
23	A 59-year-old male patient presents with a 1-year history of sensory-motor, symmetrical, non-length dependent polyneuropathy with subacute onset and progressive course. The patient presents with paresthesia, neuropathic pain and weakness distributed proximally and distally in the lower limbs. The patient also reports that in the last months he has noticed the appearance of white nails. Clinical examination does not reveal ataxia, tremor, foot deformities, cranial nerve or autonomic involvement. Past medical history is positive for alcohol abuse. Screening lab tests show the presence of thrombocytosis and IgG lambda monoclonal gammopathy. Vitamin B12, folate, vitamin B6, E, HCV, HBV, ANA, ANCA, ENA, CRP, ESR, glycemia, HbA1c, copper and cryoglobulins are normal. Nerve conduction studies show a mixed demyelinating-axonal, sensory-motor polyneuropathy. There is no family history of polyneuropathy.	POEMS	**Summary of Pertinent Clinical Information**<br>● **Patient Profile**: 59-year-old male<br>● **History**:<br>○ 1-year history of sensory-motor, symmetrical, non-length dependent polyneuropathy<br>○ Subacute onset and progressive course<br>○ Symptoms: paresthesia, neuropathic pain, and weakness in lower limbs, both proximally and distally<br>○ Recent appearance of white nails<br>● **Examination**:<br>○ No ataxia, tremor, foot deformities, cranial nerve or autonomic involvement<br>● **Medical History**:<br>○ Positive for alcohol abuse<br>● **Laboratory Findings**:<br>○ Thrombocytosis<br>○ IgG lambda monoclonal gammopathy<br>○ Normal levels of Vitamin B12, folate, vitamin B6, E, HCV, HBV, ANA, ANCA, ENA, CRP, ESR, glycemia, HbA1c, copper, and cryoglobulins<br>● **Nerve Conduction Studies**:<br>○ Mixed demyelinating-axonal, sensory-motor polyneuropathy<br>● **Family History**:<br>○ No family history of polyneuropathy<br>**Differential Diagnoses**<br>1. **Chronic Inflammatory Demyelinating Polyneuropathy (CIDP)**<br>○ **Reasoning**: CIDP is a common cause of acquired demyelinating polyneuropathy, presenting with progressive symmetrical weakness and sensory changes in both proximal and distal muscles, as observed in this patient. The mixed demyelinating-axonal pattern on nerve conduction studies supports this diagnosis. Additionally, the absence of autonomic involvement aligns with typical CIDP presentations. The patient's progressive course over a year is also characteristic of CIDP.<br>2. **Monoclonal Gammopathy of Undetermined Significance (MGUS) Neuropathy**<br>○ **Reasoning**: The presence of IgG lambda monoclonal gammopathy is a significant finding. MGUS can be associated with polyneuropathy, typically presenting as a mixed sensorimotor neuropathy, as seen in this patient. MGUS-related neuropathy can cause a similar pattern of demyelination and axonal damage. Although MGUS is often asymptomatic, it can sometimes lead to neuropathic symptoms, especially when the paraprotein exerts a toxic effect on peripheral nerves.<br>3. **Alcohol-related Neuropathy**<br>○ **Reasoning**: The patient's history of alcohol abuse is a strong risk factor for developing polyneuropathy. Alcohol-related neuropathy usually presents with a gradual onset of sensory and motor symptoms, affecting both distal and proximal muscles. The lack of improvement with cessation of alcohol use and the normal vitamin B levels, which rules out nutritional deficiencies often associated with alcoholism, still make this a plausible diagnosis due to direct neurotoxic effects of alcohol.<br>**Most Likely Diagnosis**<br>**Chronic Inflammatory Demyelinating Polyneuropathy (CIDP)**<br>**Rationale**: CIDP is the most likely diagnosis given the combination of clinical features, nerve conduction study findings, and the progressive nature of the disease. The patient's symmetrical, sensory-motor involvement affecting both proximal and distal muscles, along with the mixed demyelinating-axonal neuropathy on electrophysiological studies, is highly suggestive of CIDP. While MGUS and alcohol-related neuropathy are also considerations, the specific electrophysiological pattern and the absence of other systemic or nutritional abnormalities further support CIDP as the most probable diagnosis. The presence of white nails could be an incidental finding or related to a comorbid condition, and does not necessarily detract from the CIDP diagnosis. Further diagnostic steps such as cerebrospinal fluid analysis showing elevated protein without pleocytosis or a nerve biopsy showing characteristic changes could confirm CIDP.
24	A 77-year-old male patient presents with a 1-year history of sensory-motor,	POEMS	**Step 1: Summary of Pertinent Clinical Information**<br>**Patient Demographics:**

	symmetrical, length dependent polyneuropathy with subacute onset. The patient presents with distal leg paresthesia and neuropathic pain, distal weakness in the upper and lower limbs, without tremor, ataxia, foot deformities, cranial nerve or autonomic involvement. Past medical history shows cutaneous angiomas and IgG lambda monoclonal gammopathy. Nerve conduction studies show a demyelinating, symmetric, sensory-motor polyneuropathy. Screening lab tests including vitamin B12, folate, vitamin B6, vitamin E, HCV, HBV, ANA, ANCA, ENA, CRP, ESR, glycemia, HbA1c, copper and cryoglobulins are all negative, with positivity for IgG lambda paraprotein. There is no family history of polyneuropathy.		● 77-year-old male<br>**History of Present Illness:**<br>● 1-year history of sensory-motor, symmetrical, length-dependent polyneuropathy<br>● Subacute onset<br>● Distal leg paresthesia and neuropathic pain<br>● Distal weakness in upper and lower limbs<br>**Negative Symptoms:**<br>● No tremor<br>● No ataxia<br>● No foot deformities<br>● No cranial nerve involvement<br>● No autonomic involvement<br>**Past Medical History:**<br>● Cutaneous angiomas<br>● IgG lambda monoclonal gammopathy<br>**Nerve Conduction Studies:**<br>● Demyelinating, symmetric, sensory-motor polyneuropathy<br>**Laboratory Results:**<br>● Negative for: Vitamin B12, folate, vitamin B6, vitamin E, HCV, HBV, ANA, ANCA, ENA, CRP, ESR, glycemia, HbA1c, copper, and cryoglobulins<br>● Positive for IgG lambda paraprotein<br>**Family History:**<br>● No family history of polyneuropathy<br>**Step 2: Differential Diagnosis for Polyneuropathy**<br>**Differential Diagnosis 1: POEMS Syndrome (Polyneuropathy, Organomegaly, Endocrinopathy, Monoclonal gammopathy, Skin changes)**<br>**Reasoning:**<br>● **Polyneuropathy**: Present with sensory-motor polyneuropathy which is typically demyelinating in POEMS syndrome.<br>● **Monoclonal Gammopathy**: The patient has an IgG lambda monoclonal gammopathy, consistent with POEMS.<br>● **Cutaneous Angiomas**: Skin changes, such as angiomas, are common in POEMS syndrome.<br>● **Other features**: While organomegaly, endocrinopathy, and other characteristic features were not mentioned, the presence of the monoclonal protein and skin changes in conjunction with neuropathy makes this a strong possibility.<br>**Differential Diagnosis 2: Chronic Inflammatory Demyelinating Polyneuropathy (CIDP)**<br>**Reasoning:**<br>● **Polyneuropathy**: Symmetric, sensory-motor, demyelinating polyneuropathy fits the classic presentation of CIDP.<br>● **Course**: The subacute onset over a year is consistent with CIDP, which typically has a progressive or relapsing-remitting course.<br>● **Exclusion of Other Causes**: The negative laboratory screening for infections, vitamin deficiencies, autoimmune markers, and diabetes makes CIDP more likely.<br>**Differential Diagnosis 3: Monoclonal Gammopathy of Undetermined Significance (MGUS) associated neuropathy**<br>**Reasoning:**<br>● **Monoclonal Gammopathy**: Presence of IgG lambda paraprotein is consistent with MGUS.<br>● **Polyneuropathy**: MGUS can be associated with demyelinating polyneuropathy.<br>● **Exclusion of Other Causes**: The negative screening for other potential causes supports the diagnosis of neuropathy secondary to MGUS.<br>**Step 3: Most Likely Diagnosis**<br>**Most Likely Diagnosis: POEMS Syndrome Reasoning:**<br>● The combination of demyelinating polyneuropathy, IgG lambda monoclonal gammopathy, and cutaneous angiomas strongly suggests POEMS syndrome. While not all characteristic features (like organomegaly and endocrinopathy) are documented, the presence of these key elements aligns well with the diagnosis. Given the specific monoclonal gammopathy (IgG lambda) and the presence of skin manifestations, POEMS syndrome is more probable than CIDP or MGUS alone.<br>**Conclusion**<br>The clinical scenario of a 77-year-old male with subacute, symmetric, sensory-motor demyelinating polyneuropathy, cutaneous angiomas, and an IgG lambda monoclonal gammopathy is most consistent with POEMS syndrome. This diagnosis accounts for the range of symptoms and clinical findings presented, making it the most likely diagnosis given the provided information.
25	A 78-year-old male patient presents with an 8-month history of sensory-motor, symmetrical, length dependent polyneuropathy with insidious onset and progressive course. The patient presents with paresthesia, neuropathic pain and weakness distributed distally in the lower limbs. Clinical examination also reveals the presence sensory ataxia. There are no foot deformities, tremor, cranial nerve or autonomic involvement. Past medical history is positive for IgM monoclonal gammopathy. Nerve conduction studies show a demyelinating, symmetric, sensory-motor polyneuropathy. Screening lab tests including vitamin B12, folate, vitamin B6,	anti-MAG polyneuropathy	**Step-by-Step Clinical Analysis**<br>**Summary of Pertinent Clinical Information:**<br>1. **Patient Demographics:**<br>○ 78-year-old male<br>2. **Presenting Complaints:**<br>○ 8-month history of sensory-motor, symmetrical, length-dependent polyneuropathy<br>○ Insidious onset and progressive course<br>○ Paresthesia (abnormal sensations)<br>○ Neuropathic pain<br>○ Weakness distributed distally in the lower limbs<br>○ Sensory ataxia<br>3. **Clinical Findings:**<br>○ Symmetrical sensory-motor deficits in the lower limbs<br>○ No foot deformities, tremor, cranial nerve, or autonomic involvement<br>4. **Past Medical History:**<br>○ IgM monoclonal gammopathy

	vitamin E, HCV, HBV, ANA, ANCA, ENA, CRP, ESR, glycemia, HbA1c, copper and cryoglobulins are all negative, with positivity for IgM paraprotein. There is no family history of polyneuropathy.		5. **Diagnostic Studies:**<br>○ Nerve conduction studies: Demyelinating, symmetric, sensory-motor polyneuropathy<br>○ Screening lab tests: Negative for vitamin B12, folate, vitamin B6, vitamin E, HCV, HBV, ANA, ANCA, ENA, CRP, ESR, glycemia, HbA1c, copper, cryoglobulins<br>○ Positive for IgM paraprotein<br>6. **Family History:**<br>○ No family history of polyneuropathy<br>**Differential Diagnoses for Polyneuropathy:**<br>1. **Waldenström Macroglobulinemia (WM):**<br>○ **Reasoning:**<br>■ WM is characterized by the presence of IgM monoclonal gammopathy.<br>■ Demyelinating neuropathy is commonly associated with WM.<br>■ The patient's presentation with sensory-motor polyneuropathy, IgM paraproteinemia, and the absence of other systemic involvement supports this diagnosis.<br>■ Sensory ataxia and distal weakness are consistent with WM-related neuropathy.<br>2. **Chronic Inflammatory Demyelinating Polyneuropathy (CIDP):**<br>○ **Reasoning:**<br>■ CIDP is an acquired immune-mediated inflammatory disorder.<br>■ It often presents with progressive, symmetrical, sensory-motor neuropathy and can be associated with monoclonal gammopathies.<br>■ Nerve conduction studies showing demyelination align with CIDP.<br>■ The lack of other systemic symptoms and progressive nature over 8 months is consistent with CIDP.<br>■ However, the presence of IgM monoclonal gammopathy leans more towards a paraneoplastic or hematologic etiology.<br>3. **Monoclonal Gammopathy of Undetermined Significance (MGUS)-related Polyneuropathy:**<br>○ **Reasoning:**<br>■ MGUS is often asymptomatic but can be associated with peripheral neuropathy.<br>■ The presence of IgM paraprotein and neuropathy without other systemic disease supports MGUS-related neuropathy.<br>■ Demyelinating neuropathy can be seen in MGUS patients.<br>■ This diagnosis fits well with the patient's presentation, although MGUS itself is a diagnosis of exclusion and requires ruling out more severe hematologic conditions like WM.<br>**Most Likely Diagnosis:**<br>Based on the clinical presentation, past medical history, and diagnostic findings, the most likely diagnosis is **Waldenström Macroglobulinemia (WM)**. The presence of IgM monoclonal gammopathy, the characteristic demyelinating neuropathy, and the exclusion of other causes make WM the most probable diagnosis. Additionally, the patient's symptoms and the findings from nerve conduction studies strongly align with this condition.
26	An 80-year-old male patient presents with a 2-year history of sensory-motor, asymmetrical, non-length dependent polyneuropathy with subacute onset and a rapid progressive course. The patient presents with paraesthesia, sensory ataxia, neuropathic pain, proximal and distal weakness in the four limbs, and tremor. Clinical examination shows symmetric proximal and distal weakness in the upper and lower limbs, hypoesthesia in a stocking and glove distribution, sensory ataxia, mild intrinsic hand atrophy, and cutaneous angiomas. Clinical examination does not show the presence of foot deformities, cranial nerve, or autonomic involvement. Past medical history is positive for chronic HCV infection. Nerve conduction studies show a mixed demyelinating-axonal, symmetric, sensory-motor polyneuropathy. Screening lab tests reveal the presence of polycythaemia, IgM paraprotein, and chronic HCV infection. Remaining lab tests, including vitamin B12, folate, vitamin B6, vitamin E, HBV, ANA, ANCA, ENA, CRP, ESR, copper and cryoglobulins are all negative. There is no family history of polyneuropathy.	POEMS	**Step-by-Step Clinical Scenario Analysis**<br>**Summary of Pertinent Clinical Information**<br>**Patient Profile:**<br>● Age: 80-year-old male<br>● Duration: 2-year history<br>● Neuropathy Type: Sensory-motor, asymmetrical, non-length dependent polyneuropathy<br>● Onset: Subacute onset with rapid progression<br>**Symptoms:**<br>● Paraesthesia<br>● Sensory ataxia<br>● Neuropathic pain<br>● Proximal and distal weakness in all four limbs<br>● Tremor<br>**Clinical Findings:**<br>● Symmetric proximal and distal weakness in upper and lower limbs<br>● Hypoesthesia in a stocking and glove distribution<br>● Sensory ataxia<br>● Mild intrinsic hand atrophy<br>● Cutaneous angiomas<br>● Absence of foot deformities, cranial nerve, and autonomic involvement<br>**Past Medical History:**<br>● Chronic HCV infection<br>**Investigations:**<br>● Nerve Conduction Studies: Mixed demyelinating-axonal, symmetric, sensory-motor polyneuropathy<br>● Laboratory Findings: Polycythaemia, IgM paraprotein, chronic HCV infection<br>● Negative Tests: Vitamin B12, folate, vitamin B6, vitamin E, HBV, ANA, ANCA, ENA, CRP, ESR, copper, cryoglobulins<br>● Family History: No family history of polyneuropathy<br>**Differential Diagnoses for Polyneuropathy**<br>**1. Hepatitis C Virus (HCV)-Related Mixed Cryoglobulinemia**<br>**Reasoning:**<br>● **Chronic HCV Infection:** The patient has a known history of chronic HCV infection, which is strongly associated with mixed cryoglobulinemia, a condition that can lead to vasculitis and neuropathy.<br>● **Symptoms:** The presence of paraesthesia, sensory ataxia, neuropathic pain, and proximal and distal weakness aligns with the clinical manifestations of cryoglobulinemic neuropathy.<br>● **Cutaneous Angiomas:** These may be indicative of underlying vasculitis or other vascular abnormalities associated with cryoglobulinemia.<br>● **IgM Paraprotein:** Often found in cases of cryoglobulinemia, supporting the diagnosis.

			**2. Monoclonal Gammopathy of Undetermined Significance (MGUS) with Peripheral Neuropathy**<br>**Reasoning:**<br>● **IgM Paraprotein:** The presence of an IgM paraprotein suggests the possibility of MGUS, which can be associated with peripheral neuropathy.<br>● **Neuropathy Type:** MGUS-related neuropathy often presents with a mixed sensory and motor neuropathy, which fits the patient's presentation.<br>● **Rapid Progression:** MGUS-associated neuropathy can have a variable course, including rapid progression in some cases.<br>**3. Polycythemia Vera with Secondary Neuropathy**<br>**Reasoning:**<br>● **Polycythaemia:** The presence of polycythaemia may indicate polycythemia vera, a myeloproliferative disorder that can cause secondary neuropathy.<br>● **Symptoms and Findings:** Neuropathy in polycythemia vera can be due to hyperviscosity and microvascular occlusions, leading to similar clinical features as described in the patient, including sensory-motor involvement and neuropathic pain.<br>● **Proximal and Distal Weakness:** Fits with the type of neuropathy seen in polycythemia vera.<br>**Most Likely Diagnosis**<br>Given the patient's history of chronic HCV infection, the presence of IgM paraprotein, and the clinical presentation of polyneuropathy, the most likely diagnosis is **Hepatitis C Virus (HCV)-Related Mixed Cryoglobulinemia**. This condition can cause a mixed demyelinating-axonal neuropathy, aligning with the nerve conduction study results, and is consistent with the presence of systemic symptoms like cutaneous angiomas.<br>**Conclusion**<br>The patient's clinical presentation and laboratory findings most strongly suggest HCV-related mixed cryoglobulinemia as the underlying cause of his polyneuropathy. This diagnosis accounts for his chronic HCV infection, rapid progression of symptoms, and the presence of IgM paraprotein. Further workup, including specific testing for cryoglobulins, could confirm this diagnosis and guide appropriate treatment.
27	A 76-year-old female patient presents with a three-year history of pure sensory, symmetrical, length-dependent polyneuropathy with insidious onset and progressive course. The patient reports paraesthesia, severe neuropathic pain, and cramps in the feet in a symmetric distribution, which began approximately three years ago but significantly worsened after the third cycle of chemotherapy. Clinical examination does not reveal ataxia, tremor, foot deformities, cranial nerve or autonomic dysfunction. Past medical history is positive for type 2 diabetes, non-Hodgkin lymphoma, and breast cancer, for which she has undergone chemotherapy. Nerve conduction studies show a sensorimotor, length-dependent, symmetric, axonal polyneuropathy. Screening lab tests including vitamin B12, folate, vitamin B6, vitamin E, HCV, ANA, ANCA, ENA, CRP, ESR, copper, cryoglobulins, and serum electrophoresis are all negative. However, there is evidence of hyperglycemia, elevated HbA1c, and chronic HBV infection. There is no family history of polyneuropathy.	Superimposed chemotherapy-induced neuropathy on diabetic polyneuropathy	**Summary of Clinical Information**<br>● **Patient:** 76-year-old female<br>● **Presentation:** Three-year history of pure sensory, symmetrical, length-dependent polyneuropathy with insidious onset and progressive course.<br>● **Symptoms:** Paraesthesia, severe neuropathic pain, and cramps in the feet, symmetric distribution.<br>● **Onset:** Symptoms began approximately three years ago, worsened after the third cycle of chemotherapy.<br>● **Clinical Examination:** No ataxia, tremor, foot deformities, cranial nerve or autonomic dysfunction.<br>● **Past Medical History:**<br>○ Type 2 diabetes<br>○ Non-Hodgkin lymphoma<br>○ Breast cancer (treated with chemotherapy)<br>● **Nerve Conduction Studies:** Sensorimotor, length-dependent, symmetric, axonal polyneuropathy.<br>● **Screening Lab Tests:** Negative for vitamin B12, folate, vitamin B6, vitamin E, HCV, ANA, ANCA, ENA, CRP, ESR, copper, cryoglobulins, and serum electrophoresis.<br>● **Positive Lab Findings:** Hyperglycemia, elevated HbA1c, chronic HBV infection.<br>● **Family History:** No family history of polyneuropathy.<br>**Differential Diagnoses for Polyneuropathy**<br>1. **Diabetic Polyneuropathy**<br>○ **Reasoning:**<br>■ The patient has a history of type 2 diabetes with evidence of hyperglycemia and elevated HbA1c, indicating poor glycemic control.<br>■ Diabetic polyneuropathy is a common complication of diabetes and typically presents as a symmetrical, length-dependent polyneuropathy, which matches the patient's symptoms.<br>■ The insidious onset and progressive nature of the polyneuropathy, along with severe neuropathic pain and paraesthesia, are characteristic of diabetic neuropathy.<br>2. **Chemotherapy-Induced Peripheral Neuropathy (CIPN)**<br>○ **Reasoning:**<br>■ The patient has a history of chemotherapy for breast cancer, and her symptoms significantly worsened after the third cycle of chemotherapy.<br>■ Chemotherapeutic agents, particularly those used in the treatment of breast cancer and non-Hodgkin lymphoma (such as platinum-based drugs, taxanes, and vinca alkaloids), are known to cause peripheral neuropathy.<br>■ CIPN typically presents as a symmetrical, length-dependent polyneuropathy with sensory symptoms and can have a delayed onset and progressive course even after cessation of chemotherapy.<br>3. **Chronic Hepatitis B Virus (HBV) Infection-Related Neuropathy**<br>○ **Reasoning:**<br>■ The patient has chronic HBV infection, which can be associated with peripheral neuropathy through immune-mediated mechanisms or direct viral effects.<br>■ Although less common, HBV-related neuropathy can present as a symmetrical, axonal polyneuropathy, which aligns with the patient's nerve conduction study findings.<br>■ The absence of other common causes (negative screening lab tests) and the chronic nature of HBV infection support this as a potential contributing factor.<br>**Most Likely Diagnosis**<br>Based on the clinical information provided, the most likely diagnosis for this patient's polyneuropathy is **Diabetic Polyneuropathy**. The key factors supporting this diagnosis include the presence of type 2 diabetes with poor glycemic control (elevated HbA1c), the typical presentation of symmetrical, length-dependent polyneuropathy, and the progressive nature of the symptoms. While chemotherapy and chronic HBV infection are potential contributors, the prominence of diabetic risk factors and the classic presentation strongly suggest diabetic polyneuropathy as the primary diagnosis.
28	A 37-year-old male patient presents with a 2-week history of sensory-motor, symmetrical, non-length dependent polyneuropathy with acute onset. The patient presents with distal paresthesia in the upper and lower limbs, neuropathic pain, and weakness in the proximal and distal areas of the upper and lower limbs. There are no ataxia, tremor, foot deformities, cranial nerve or autonomic involvement. Past medical history is silent.	GBS	**Step-by-Step Analysis of Clinical Scenario**<br>**Patient Summary**<br>● **Patient Demographics:** 37-year-old male<br>● **Presenting Symptoms:**<br>○ Acute onset, 2-week history<br>○ Sensory-motor, symmetrical, non-length dependent polyneuropathy<br>○ Distal paresthesia in upper and lower limbs<br>○ Neuropathic pain<br>○ Weakness in both proximal and distal areas of upper and lower limbs<br>● **Clinical Findings:**<br>○ No ataxia, tremor, foot deformities, cranial nerve, or autonomic involvement

	Nerve conduction studies show a demyelinating, sensory-motor polyneuropathy. Screening lab tests including vitamin B12, folate, vitamin B6, vitamin E, HCV, HBV, ANA, ANCA, ENA, CRP, ESR, glycemia, HbA1c, copper, cryoglobulins and serum electrophoresis are all negative. There is no family history of polyneuropathy.		● **Past Medical History:** Unremarkable<br>● **Nerve Conduction Studies:** Demyelinating, sensory-motor polyneuropathy<br>● **Screening Lab Tests:** Negative (vitamin B12, folate, vitamin B6, vitamin E, HCV, HBV, ANA, ANCA, ENA, CRP, ESR, glycemia, HbA1c, copper, cryoglobulins, serum electrophoresis)<br>● **Family History:** No polyneuropathy<br>**Differential Diagnoses for Polyneuropathy**<br>1. **Guillain-Barré Syndrome (GBS)**<br>○ **Reasoning:**<br>■ **Acute Onset:** GBS typically presents with an acute onset, often progressing rapidly over days to weeks.<br>■ **Symmetrical, Sensory-Motor Involvement:** GBS usually involves symmetrical weakness and can include both motor and sensory deficits.<br>■ **Demyelinating Polyneuropathy:** The demyelinating nature observed in nerve conduction studies is consistent with GBS, particularly the acute inflammatory demyelinating polyneuropathy (AIDP) variant.<br>■ **Lack of Autonomic and Cranial Nerve Involvement:** While autonomic and cranial nerve involvement can occur in GBS, their absence does not rule it out.<br>2. **Chronic Inflammatory Demyelinating Polyneuropathy (CIDP)**<br>○ **Reasoning:**<br>■ **Sensory-Motor, Symmetrical Polyneuropathy:** CIDP can present with both sensory and motor involvement, often symmetrical.<br>■ **Demyelinating Features:** Nerve conduction studies consistent with demyelination are typical for CIDP.<br>■ **Time Course:** CIDP generally has a more chronic course, but subacute presentations can occur, making it a differential to consider.<br>■ **No Systemic Involvement:** The absence of systemic disease or other organ involvement aligns with CIDP.<br>3. **Multifocal Motor Neuropathy (MMN)**<br>○ **Reasoning:**<br>■ **Motor Predominance:** MMN is primarily motor, but the sensory involvement here makes it less likely, yet still a consideration.<br>■ **Demyelinating Features:** Demyelination can be observed in MMN.<br>■ **Lack of Autonomic Involvement:** Similar to the current case, MMN lacks autonomic involvement.<br>■ **Rapid Onset:** While MMN usually has a more chronic progression, it can sometimes present more acutely.<br>**Most Likely Diagnosis**<br>**Guillain-Barré Syndrome (GBS)**<br>● The most likely diagnosis given the acute onset, symmetrical sensory-motor involvement, and demyelinating nature observed in nerve conduction studies. Although there is no cranial nerve or autonomic involvement, these are not mandatory for the diagnosis of GBS. The rapid progression and demyelinating features strongly support GBS, particularly the AIDP variant.<br>**Conclusion**<br>The patient's presentation is most consistent with Guillain-Barré Syndrome, particularly the AIDP variant. This is based on the acute onset of symptoms, symmetrical sensory-motor involvement, and findings from nerve conduction studies indicating demyelination. Further confirmatory tests such as lumbar puncture (to check for elevated protein in cerebrospinal fluid without pleocytosis) and possibly MRI may be warranted to solidify the diagnosis. Early intervention with IV immunoglobulins or plasmapheresis should be considered to improve outcomes.
29	A 75-year-old male patient presents with a 4-year history of pure sensory, symmetrical, length dependent polyneuropathy with insidious onset and slow progressive course. The patient presents with paresthesia and neuropathic pain in the feet. Clinical examination does not showtremor or sensory ataxia. There are no signs of foot deformities, cranial nerve or autonomic involvement. Past medical history is positive for type 2 diabetes and arterial hypertension. Nerve conduction studies show a mixed sensory-motor, length dependent, axonal polyneuropathy. Screening lab tests reveal hyperglycaemia and elevated HbA1c. Remaining exams including vitamin B12, vitamin B6, vitamin E, HBV, HCV, ANA, ANCA, ENA, CRP, ESR, copper, cryoglobulin, and serum electrophoresis are normal. There is no family history of polyneuropathy.	Diabetic neuropathy	**Step-by-Step Clinical Analysis**<br>**1. Summary of Pertinent Clinical Information:**<br>● **Patient:** 75-year-old male<br>● **History Duration:** 4 years<br>● **Symptoms:**<br>○ Pure sensory, symmetrical, length-dependent polyneuropathy<br>○ Paresthesia and neuropathic pain in the feet<br>○ Insidious onset and slow progressive course<br>● **Clinical Examination:**<br>○ No tremor or sensory ataxia<br>○ No foot deformities<br>○ No cranial nerve or autonomic involvement<br>● **Past Medical History:**<br>○ Type 2 diabetes<br>○ Arterial hypertension<br>● **Nerve Conduction Studies:**<br>○ Mixed sensory-motor, length-dependent, axonal polyneuropathy<br>● **Screening Lab Results:**<br>○ Hyperglycaemia and elevated HbA1c<br>○ Normal levels of vitamin B12, vitamin B6, vitamin E, HBV, HCV, ANA, ANCA, ENA, CRP, ESR, copper, cryoglobulin, and serum electrophoresis<br>● **Family History:**<br>○ No family history of polyneuropathy<br>**2. Differential Diagnoses for Polyneuropathy:**<br>Based on the clinical presentation and the available information, the three most probable differential diagnoses for this patient's polyneuropathy are:<br>1. **Diabetic Polyneuropathy:**<br>○ **Reasoning:** The patient has a history of type 2 diabetes with hyperglycaemia and elevated HbA1c levels. Diabetic polyneuropathy is the most common cause of peripheral neuropathy and typically presents as a symmetrical, length-dependent sensory neuropathy with a slow progression, consistent with the patient's symptoms and nerve conduction study results.<br>2. **Chronic Idiopathic Axonal Polyneuropathy (CIAP):**<br>○ **Reasoning:** Given the insidious onset, slow progression, and lack of other specific etiological findings from the extensive lab work, CIAP is a plausible diagnosis. CIAP typically presents in older adults with a similar sensory-predominant neuropathy and normal laboratory findings apart from those related to diabetes.<br>3. **Paraneoplastic Neuropathy:**

			○ **Reasoning:** Although less likely due to the absence of systemic symptoms or a known malignancy, paraneoplastic neuropathy should be considered in older adults with unexplained neuropathy. The extensive negative workup makes this less probable, but it remains a differential to consider, especially if other causes are ruled out.<br>**3. Most Likely Diagnosis:**<br>Based on the patient's clinical history, examination, and diagnostic findings, the most likely diagnosis is **Diabetic Polyneuropathy**. This diagnosis is supported by:<br>● The presence of type 2 diabetes with poorly controlled blood sugar levels (hyperglycaemia and elevated HbA1c).<br>● The typical presentation of a sensory, symmetrical, length-dependent neuropathy.<br>● Nerve conduction studies showing a mixed sensory-motor, axonal pattern consistent with diabetic neuropathy.<br>**Conclusion**<br>The patient's symptoms and diagnostic findings strongly point towards **Diabetic Polyneuropathy** as the most likely diagnosis. Continuous management of blood glucose levels and symptomatic treatment for neuropathic pain should be prioritized in this patient's care plan. Further follow-up and monitoring for potential complications related to diabetes are also essential.
30	A 60-year-old male patient presents with a 17-month history of sensory, symmetrical, length dependent polyneuropathy with an insidious onset and a slow progressive course. The first occurred symptom was neuropathic pain in the feet, followed by erectile dysfunction, hyperhidrosis and mild distal paraesthesia. Clinical examination does not show presence of sensory ataxia, tremor or foot deformities. Past medical history is silent. Nerve conduction studies show a mixed sensory-motor, symmetric, axonal polyneuropathy and bilateral carpal tunnel syndrome. Screening lab tests including vitamin B12, folate, vitamin B6, vitamin E, HBV, HCV, ANA, ANCA, ENA, CRP, ESR, glycemia, HbA1c, copper, and cryoglobulins are normal. Serum electrophoresis shows presence of IgG lambda paraprotein. There is no family history of polyneuropathy.	Amyloidosis associated polyneuropathy	**Clinical Information Summary**<br>● **Patient**: 60-year-old male<br>● **History**: 17 months of sensory, symmetrical, length-dependent polyneuropathy with an insidious onset and slow progressive course<br>○ **Initial symptom**: Neuropathic pain in feet<br>○ **Subsequent symptoms**: Erectile dysfunction, hyperhidrosis, mild distal paraesthesia<br>● **Clinical Examination**:<br>○ No sensory ataxia, tremor, or foot deformities<br>● **Past Medical History**: No significant history<br>● **Nerve Conduction Studies**: Mixed sensory-motor, symmetric, axonal polyneuropathy and bilateral carpal tunnel syndrome<br>● **Lab Tests**:<br>○ Normal: Vitamin B12, folate, vitamin B6, vitamin E, HBV, HCV, ANA, ANCA, ENA, CRP, ESR, glycemia, HbA1c, copper, cryoglobulins<br>○ Abnormal: Serum electrophoresis shows IgG lambda paraprotein<br>● **Family History**: No family history of polyneuropathy<br>**Differential Diagnoses for Polyneuropathy**<br>1. **Monoclonal Gammopathy of Undetermined Significance (MGUS)**<br>○ **Reasoning**:<br>■ Presence of IgG lambda paraprotein in serum electrophoresis is highly suggestive of MGUS.<br>■ MGUS can cause a slowly progressive, length-dependent polyneuropathy.<br>■ The patient's age and absence of family history align with typical MGUS presentations.<br>■ The normal screening tests for other common causes and the specific paraprotein finding support this diagnosis.<br>2. **Diabetic Polyneuropathy**<br>○ **Reasoning**:<br>■ Diabetic polyneuropathy is a common cause of polyneuropathy, particularly in the elderly.<br>■ The patient's glycemia and HbA1c are normal, which might exclude active diabetes. However, prediabetes or a history of undiagnosed diabetes cannot be entirely ruled out without more specific testing or glucose tolerance tests.<br>■ Symptoms such as neuropathic pain, erectile dysfunction, and autonomic symptoms (hyperhidrosis) are consistent with diabetic neuropathy.<br>■ The absence of family history and the presence of bilateral carpal tunnel syndrome also fit the profile, although the normal HbA1c and glycemia make this less likely.<br>3. **Amyloidosis**<br>○ **Reasoning**:<br>■ Amyloidosis, particularly AL amyloidosis, can present with a slowly progressive axonal polyneuropathy.<br>■ The presence of a paraprotein (IgG lambda) raises the suspicion of amyloidosis, as monoclonal gammopathy is often associated with this condition.<br>■ Symptoms such as neuropathic pain and autonomic involvement (erectile dysfunction, hyperhidrosis) are characteristic of amyloid neuropathy.<br>■ Absence of other organ involvement (such as renal, cardiac, or hepatic) does not exclude the diagnosis as neuropathy can be an early manifestation.<br>**Most Likely Diagnosis**<br>**Monoclonal Gammopathy of Undetermined Significance (MGUS) with Associated Polyneuropathy**<br>**Conclusion**: The most likely diagnosis is MGUS-associated polyneuropathy. This conclusion is supported by the presence of IgG lambda paraprotein, the pattern of sensory and motor axonal polyneuropathy, and the exclusion of other common causes through comprehensive screening. While diabetic polyneuropathy and amyloidosis remain considerations, the specific finding of IgG lambda paraprotein strongly points towards MGUS as the primary etiology. Further diagnostic steps could include bone marrow biopsy and imaging to rule out progression to multiple myeloma or amyloidosis, and additional tests for nerve biopsy if amyloidosis remains a concern.
31	A 54-year-old male patient presents with a 2-year history of chronic, pure sensory, symmetrical, length dependent polyneuropathy with insidious onset. The patient presents with distal paresthesia and neuropathic pain in the lower limbs, without ataxia. Past medical history reveals high alcohol consumption. Clinical examination reveals absence of foot deformities, tremor, cranial nerve or autonomic involvement. Nerve conduction studies show a length-dependent polyneuropathy with symmetric, pure sensory, axonal involvement. Screening lab tests including vitamin B12, folate, vitamin	Alcohol-associated neuropathy	**Summary of Pertinent Clinical Information**<br>● **Patient Profile**: 54-year-old male<br>● **Duration of Symptoms**: 2 years<br>● **Symptom Characteristics**:<br>○ Chronic, pure sensory, symmetrical, length-dependent polyneuropathy<br>○ Insidious onset<br>○ Distal paresthesia and neuropathic pain in the lower limbs<br>○ No ataxia<br>● **Past Medical History**: High alcohol consumption<br>● **Clinical Examination**:<br>○ Absence of foot deformities, tremor, cranial nerve involvement, or autonomic involvement<br>● **Nerve Conduction Studies**:<br>○ Length-dependent polyneuropathy with symmetric, pure sensory, axonal involvement<br>● **Screening Lab Tests**:<br>○ Negative: vitamin B12, folate, vitamin B6, vitamin E, HCV, HBV, ANA, ANCA, ENA, CRP, ESR, glycemia, HbA1c, copper, cryoglobulins, serum electrophoresis

	B6, vitamin E, HCV, HBV, ANA, ANCA, ENA, CRP, ESR, glycemia, HbA1c, copper, cryoglobulins and serum electrophoresis are all negative. Serum gamma-glutamyl transferase (GGT) levels are elevated. There is no family history of polyneuropathy.		○ Elevated: Serum gamma-glutamyl transferase (GGT) levels<br>● **Family History**: No family history of polyneuropathy<br>**Differential Diagnoses for Polyneuropathy**<br>1. **Alcoholic Polyneuropathy**<br>○ **Reasoning**:<br>■ The patient has a history of high alcohol consumption, which is a well-known risk factor for developing polyneuropathy.<br>■ Chronic alcohol use can lead to nutritional deficiencies (such as thiamine deficiency) and direct neurotoxic effects, contributing to axonal degeneration.<br>■ Elevated serum GGT levels support ongoing significant alcohol consumption and potential liver involvement.<br>■ The clinical presentation of a pure sensory, symmetric, length-dependent polyneuropathy aligns well with typical manifestations of alcoholic polyneuropathy.<br>2. **Idiopathic Sensory Polyneuropathy**<br>○ **Reasoning**:<br>■ Despite extensive screening, no specific metabolic, infectious, or autoimmune causes were identified, making idiopathic origins a possibility.<br>■ The absence of family history and negative extensive screening tests could suggest an idiopathic nature.<br>■ Idiopathic sensory polyneuropathy often presents with similar features: chronic, symmetric, length-dependent sensory symptoms without motor involvement.<br>3. **Toxic Neuropathy (Non-Alcoholic)**<br>○ **Reasoning**:<br>■ While the patient's history is significant for alcohol, other toxic exposures should be considered, especially given the negative screening for metabolic and infectious causes.<br>■ Common non-alcoholic toxins include heavy metals (like lead or arsenic), certain medications, and industrial chemicals.<br>■ The detailed history did not provide specifics on occupational or environmental exposures, but the possibility remains given the chronic nature and negative metabolic workup.<br>**Most Likely Diagnosis**<br>Based on the clinical presentation and the patient's significant history of high alcohol consumption, **Alcoholic Polyneuropathy** is the most likely diagnosis. This conclusion is supported by:<br>● A well-documented history of alcohol use, which is a major etiological factor for polyneuropathy.<br>● Elevated serum GGT, indicating chronic alcohol use.<br>● The absence of other identifiable causes through extensive lab workup.<br>**Conclusion**<br>The patient's chronic, symmetric, pure sensory, length-dependent polyneuropathy with a history of high alcohol consumption, supported by elevated GGT levels and a lack of other identifiable causes, points most strongly to **Alcoholic Polyneuropathy** as the most likely diagnosis. Treatment should focus on cessation of alcohol consumption and addressing any nutritional deficiencies, particularly thiamine, to halt the progression and potentially improve symptoms.
32	An 80-year-old male patient presents with a 3-year history of neuropathic pain and skin redness in the hands and feet. Symptoms started insidiously but progressed over the years. Clinical examination reveals hyperalgesia and allodynia in the hands and feet, where the skin is warm and red. There are no weakness, deficit in tactile sensation, sensory ataxia, tremor, foot deformities, cranial nerve or autonomic involvement. Past medical history is positive for prostate cancer and chronic renal insufficiency. Nerve conduction studies are normal. Screening lab tests including vitamin B12, folate, vitamin B6, vitamin E, HBV, HCV, ANA, ANCA, ENA, CRP, ESR, glycemia, HbA1c, copper, cryoglobulins and serum electrophoresis are negative. There is no family history of polyneuropathy.	Small fibre neuropathy	**Clinical Summary**<br>An 80-year-old male presents with a 3-year history of neuropathic pain and skin redness in the hands and feet. Symptoms started insidiously and have progressively worsened. Clinical findings include:<br>● Hyperalgesia and allodynia in the hands and feet<br>● Warm and red skin in affected areas<br>● Absence of weakness, tactile sensation deficit, sensory ataxia, tremor, foot deformities, cranial nerve, or autonomic involvement<br>Relevant medical history includes:<br>● Prostate cancer<br>● Chronic renal insufficiency<br>Diagnostic findings:<br>● Normal nerve conduction studies<br>● Negative screening lab tests for vitamin B12, folate, vitamin B6, vitamin E, HBV, HCV, ANA, ANCA, ENA, CRP, ESR, glycemia, HbA1c, copper, cryoglobulins, and serum electrophoresis<br>● No family history of polyneuropathy<br>**Differential Diagnoses for Polyneuropathy**<br>**1. Small Fiber Neuropathy (SFN)**<br>**Reasoning:**<br>● **Clinical Presentation:** SFN typically presents with neuropathic pain, hyperalgesia, and allodynia. The warm and red skin in the hands and feet is consistent with the involvement of small nerve fibers responsible for pain and temperature sensation.<br>● **Normal Nerve Conduction Studies:** Nerve conduction studies often remain normal in SFN as they primarily assess large fibers.<br>● **Negative Screening Tests:** Common causes of SFN (such as diabetes and vitamin deficiencies) have been ruled out by the negative lab tests, but SFN can still occur idiopathically or secondary to other less common conditions.<br>**2. Erythromelalgia**<br>**Reasoning:**<br>● **Clinical Presentation:** Erythromelalgia is characterized by episodes of red, hot, painful extremities, which align with the patient's symptoms.<br>● **Triggers and Relief:** Typically, symptoms are triggered by heat and relieved by cooling, although this information is not provided in the case history.<br>● **Associations:** Erythromelalgia can be primary (genetic) or secondary to myeloproliferative disorders. While there is no mention of such disorders, the chronic renal insufficiency and history of prostate cancer could indirectly contribute to vascular or neuropathic conditions mimicking erythromelalgia.<br>**3. Chronic Inflammatory Demyelinating Polyneuropathy (CIDP) – Atypical Presentation**<br>**Reasoning:**<br>● **Clinical Presentation:** CIDP usually presents with progressive weakness and sensory loss, but atypical forms can present with predominant pain and sensory disturbances.<br>● **Normal Nerve Conduction Studies:** CIDP usually shows abnormalities in nerve conduction studies, but there are rare cases where early or mild disease may not yet show these changes.<br>● **Immune-Mediated Mechanism:** Despite the negative autoimmune screening, CIDP or other immune-mediated neuropathies might not always have positive serologies, particularly in elderly patients.<br>**Most Likely Diagnosis**<br>**Small Fiber Neuropathy (SFN)** is the most likely diagnosis based on the clinical presentation of neuropathic pain, hyperalgesia, and allodynia with normal nerve conduction studies and negative extensive lab workup. The clinical features and diagnostic findings are highly suggestive of SFN, especially given the lack of large fiber involvement and the normal nerve conduction studies. Although erythromelalgia is a close differential, the insidious progression and lack of typical triggering factors make SFN more plausible. CIDP is less likely due to the absence of motor involvement and the normal nerve conduction studies.<br>**Conclusion**

			Based on the patient's symptoms, clinical findings, and diagnostic results, the most likely diagnosis is **Small Fiber Neuropathy (SFN)**. Further confirmatory tests, such as skin biopsy or quantitative sensory testing (QST), could be considered to support the diagnosis of SFN.
33	A 24-year old male patient presents with a 4-year history of sensory-motor, symmetrical, length dependent polyneuropathy with insidious onset and slow progression. The patient presents with severe walking difficulties, bilateral foot-drop, severe distal atrophy in the lower limbs, and hypoesthesia up to calves. There is no cranial nerve or autonomic involvement. Past medical history is silent. Nerve conduction studies show a mixed axonal-demyelinating, symmetric, sensory-motor polyneuropathy. Screening lab tests including vitamin B12, folate, vitamin B6, vitamin E, HBV, HCV, glycemia, HbA1c, ANA, ANCA, ENA, CRP, ESR, copper, cryoglobulins, and serum electrophoresis are all negative. His mother and sister have mild walking difficulties.	X-linked CMT	**Step-by-Step Analysis**<br>**1. Summary of Clinical Information:**<br>● **Patient Information:** 24-year-old male<br>● **Presenting Complaint:** Sensory-motor, symmetrical, length-dependent polyneuropathy with insidious onset and slow progression over 4 years<br>● **Symptoms:**<br>○ Severe walking difficulties<br>○ Bilateral foot-drop<br>○ Severe distal atrophy in lower limbs<br>○ Hypoesthesia up to the calves<br>● **Examination Findings:**<br>○ No cranial nerve involvement<br>○ No autonomic involvement<br>● **Past Medical History:** Unremarkable<br>● **Family History:**<br>○ Mother and sister with mild walking difficulties<br>● **Investigations:**<br>○ Nerve conduction studies: Mixed axonal-demyelinating, symmetric, sensory-motor polyneuropathy<br>○ Screening lab tests: Negative (vitamin B12, folate, vitamin B6, vitamin E, HBV, HCV, glycemia, HbA1c, ANA, ANCA, ENA, CRP, ESR, copper, cryoglobulins, serum electrophoresis)<br>**2. Differential Diagnoses for Polyneuropathy:**<br>**A. Charcot-Marie-Tooth Disease (CMT)**<br>● **Reasoning:**<br>○ **Clinical Features:** The slow progression of sensory-motor polyneuropathy, distal atrophy, and foot-drop are characteristic features of CMT.<br>○ **Family History:** The presence of walking difficulties in the mother and sister suggests a hereditary pattern.<br>○ **Nerve Conduction Studies:** Mixed axonal-demyelinating features are consistent with CMT, which can present with both axonal and demyelinating forms.<br>○ **Onset and Progression:** Insidious onset and slow progression over years align with the typical course of CMT.<br>**B. Hereditary Neuropathy with Liability to Pressure Palsies (HNPP)**<br>● **Reasoning:**<br>○ **Clinical Features:** HNPP can present with sensory-motor polyneuropathy and recurrent focal neuropathies, but it typically involves more focal, episodic pressure-related neuropathies rather than the continuous, length-dependent progression seen here.<br>○ **Family History:** Positive family history supports a genetic etiology.<br>○ **Nerve Conduction Studies:** While HNPP often shows focal demyelination, the mixed axonal-demyelinating pattern could overlap with HNPP in some cases.<br>○ **Onset and Progression:** The progressive, length-dependent nature and severe distal involvement make HNPP less likely compared to CMT.<br>**C. Chronic Inflammatory Demyelinating Polyneuropathy (CIDP)**<br>● **Reasoning:**<br>○ **Clinical Features:** CIDP often presents with a progressive sensory-motor polyneuropathy and can have mixed axonal-demyelinating features on nerve conduction studies.<br>○ **Exclusion of Autoimmune Markers:** The absence of systemic inflammatory markers (CRP, ESR) and negative autoimmune screening (ANA, ANCA, ENA) is less supportive of CIDP, although not definitive.<br>○ **Onset and Progression:** CIDP typically has a more variable onset, and the family history is less indicative of a hereditary condition compared to CMT.<br>**3. Most Likely Diagnosis:**<br>Based on the detailed clinical presentation, the family history, and the findings from nerve conduction studies, **Charcot-Marie-Tooth Disease (CMT)** is the most probable diagnosis. The key factors include:<br>● The symmetrical, sensory-motor polyneuropathy with distal atrophy and foot-drop.<br>● The slow progression and length-dependent nature of symptoms.<br>● The strong family history suggesting a hereditary condition.<br>● The mixed axonal-demyelinating pattern on nerve conduction studies.<br>Further genetic testing to identify specific mutations associated with CMT would be the next step in confirming the diagnosis.
34	An 83-year-old male patient presents with a 1-year history of pure sensory, symmetrical, length dependent polyneuropathy with insidious onset and progressive course. The patient presents with distal paresthesia and hypoesthesia in the four limbs, mild hand tremor and cramps but no neuropathic pain. He also reports the appearance of gait instability in the past few months. Clinical examination confirms the presence of sensory ataxia with difficulty walking without support. There are no foot deformities, cranial nerve or autonomic involvement. Past medical history shows IgG kappa monoclonal gammopathy, thrombocytopenia, and prostatic cancer.	Vit B12 deficiency neuropathy	**Step-by-Step Clinical Scenario Analysis**<br>**Summary of Pertinent Clinical Information**<br>1. **Patient Demographics**: 83-year-old male<br>2. **Symptoms**:<br>○ 1-year history of pure sensory, symmetrical, length-dependent polyneuropathy<br>○ Distal paresthesia and hypoesthesia in all four limbs<br>○ Mild hand tremor and cramps, no neuropathic pain<br>○ Recent gait instability, sensory ataxia, difficulty walking without support<br>3. **Clinical Examination**:<br>○ Sensory ataxia, no foot deformities<br>○ No cranial nerve or autonomic involvement<br>4. **Past Medical History**:<br>○ IgG kappa monoclonal gammopathy<br>○ Thrombocytopenia<br>○ Prostatic cancer<br>5. **Diagnostic Tests**:<br>○ Nerve conduction studies: pure sensory, symmetric, length-dependent, axonal polyneuropathy

	Nerve conduction studies show a pure sensory, symmetric, length-dependent, axonal polyneuropathy. Screening lab tests show the presence of IgG kappa paraprotein and evidence of vitamin B12 deficiency. Remaining exams including folate, vitamin B6, vitamin E, HCV, HBV, ANA, ENA, ANCA, CRP, ESR, glycemia, HbA1c, copper and cryoglobulins are all negative. There is no family history of polyneuropathy.		○ Screening labs: presence of IgG kappa paraprotein, vitamin B12 deficiency<br>○ Negative for folate, vitamin B6, vitamin E, HCV, HBV, ANA, ENA, ANCA, CRP, ESR, glycemia, HbA1c, copper, and cryoglobulins<br>6. **Family History**: No family history of polyneuropathy<br>**Differential Diagnoses for Polyneuropathy**<br>1. **Monoclonal Gammopathy of Undetermined Significance (MGUS) Associated Neuropathy**:<br>○ **Reasoning**: The patient has a documented IgG kappa monoclonal gammopathy. MGUS can lead to a sensorimotor polyneuropathy, often presenting with symptoms similar to those described in this case. The presence of IgG kappa paraprotein further supports this diagnosis.<br>○ **Supporting Features**:<br>■ IgG kappa monoclonal gammopathy<br>■ Symmetrical, length-dependent polyneuropathy<br>■ Axonal nature of the neuropathy on nerve conduction studies<br>○ **Against**: MGUS typically presents without systemic involvement, but the patient's thrombocytopenia and history of prostatic cancer need consideration for a more systemic process.<br>2. **Vitamin B12 Deficiency**:<br>○ **Reasoning**: Vitamin B12 deficiency can lead to a subacute combined degeneration of the spinal cord and peripheral neuropathy. Sensory ataxia and gait instability are consistent with B12 deficiency.<br>○ **Supporting Features**:<br>■ Documented vitamin B12 deficiency<br>■ Sensory ataxia<br>■ Length-dependent axonal polyneuropathy<br>○ **Against**: Typically, B12 deficiency neuropathy would also affect motor nerves and present with more systemic signs of deficiency like anemia or cognitive changes, which are not prominent in this case.<br>3. **Paraneoplastic Neuropathy**:<br>○ **Reasoning**: The patient has a history of prostatic cancer, which could lead to a paraneoplastic syndrome affecting peripheral nerves. Paraneoplastic neuropathies can present as pure sensory neuropathies and often have an insidious onset with progressive course.<br>○ **Supporting Features**:<br>■ History of prostatic cancer<br>■ Symmetrical, sensory, length-dependent polyneuropathy<br>■ Absence of other common causes (negative extensive workup)<br>○ **Against**: Lack of specific paraneoplastic antibodies in the workup, and no clear temporal relation to cancer treatment or progression.<br>**Most Likely Diagnosis**<br>Considering the detailed clinical history, diagnostic findings, and the nature of the patient's neuropathy, the most likely diagnosis is **Monoclonal Gammopathy of Undetermined Significance (MGUS) Associated Neuropathy**. This diagnosis aligns well with the presence of IgG kappa monoclonal gammopathy, the nature of the neuropathy (pure sensory, length-dependent, axonal), and the exclusion of other significant causes through extensive testing.<br>**Conclusion**<br>The patient's presentation, clinical findings, and diagnostic results most strongly suggest MGUS-associated neuropathy. The presence of IgG kappa monoclonal gammopathy and the specific pattern of neuropathy support this diagnosis, making it the most probable cause of his symptoms. Further management should include monitoring of the MGUS for potential progression and addressing the vitamin B12 deficiency to see if there is any improvement in symptoms.
35	An 82-year-old male patient presents with a 3-year history of pure sensory, symmetrical, length dependent polyneuropathy with insidious onset and slow progressive course. The patient presents with paraesthesia and hypoesthesia in the feet. Clinical examination reveals hyperalgesia during pinprick testing and tactile hypoesthesia in the feet, while there are no weakness, sensory ataxia, tremor, foot deformities, cranial nerve or autonomic dysfunction. Past medical history is positive for IgG kappa MGUS, prostate and bladder cancer, for which he underwent chemotherapy five years ago. Nerve conduction studies show a sensory-motor, symmetric, length dependent, axonal polyneuropathy. Screening lab tests reveal presence of IgG kappa paraprotein. Remaining exams including vitamin B12, folate, vitamin B6, vitamin E, HBV, HCV, glycaemia, HbA1c, ANA, ANCA, ENA, CRP, ESR, copper ,and cryoglobulins are normal. There is no family history of polyneuropathy.	Paraprotein emic neuropathy	**Summary of Pertinent Clinical Information**<br>● **Patient:** 82-year-old male<br>● **Duration of symptoms:** 3 years<br>● **Type of neuropathy:** Pure sensory, symmetrical, length-dependent polyneuropathy<br>● **Onset and progression:** Insidious onset with slow progressive course<br>● **Symptoms:** Paraesthesia and hypoesthesia in the feet<br>● **Clinical examination:**<br>○ Hyperalgesia during pinprick testing<br>○ Tactile hypoesthesia in the feet<br>○ No weakness<br>○ No sensory ataxia<br>○ No tremor<br>○ No foot deformities<br>○ No cranial nerve or autonomic dysfunction<br>● **Past medical history:**<br>○ IgG kappa monoclonal gammopathy of undetermined significance (MGUS)<br>○ Prostate and bladder cancer (chemotherapy 5 years ago)<br>● **Nerve conduction studies:** Sensory-motor, symmetric, length-dependent, axonal polyneuropathy<br>● **Lab tests:**<br>○ IgG kappa paraprotein present<br>○ Normal levels of vitamin B12, folate, vitamin B6, vitamin E, HBV, HCV, glycaemia, HbA1c, ANA, ANCA, ENA, CRP, ESR, copper, and cryoglobulins<br>● **Family history:** No family history of polyneuropathy<br>**Differential Diagnoses for Polyneuropathy**<br>1. **Monoclonal Gammopathy of Undetermined Significance (MGUS)-associated Polyneuropathy**<br>○ **Reasoning:**<br>■ The presence of IgG kappa paraprotein suggests an association with MGUS.<br>■ MGUS is known to be associated with a chronic, length-dependent, sensory neuropathy.<br>■ The patient's clinical presentation aligns with the typical manifestations of MGUS-associated neuropathy: pure sensory symptoms, symmetrical distribution, and slow progression.<br>■ Nerve conduction studies showing axonal polyneuropathy support this diagnosis as MGUS often causes axonal damage.<br>2. **Chemotherapy-induced Peripheral Neuropathy (CIPN)**

			○ **Reasoning:**<br>■ The patient has a history of chemotherapy for prostate and bladder cancer five years ago, which is a well-known risk factor for peripheral neuropathy.<br>■ Chemotherapy agents, especially platinum-based compounds and taxanes, can cause a sensory-predominant neuropathy that persists for years after treatment.<br>■ The absence of new findings in nerve conduction studies (sensory-motor, axonal) is consistent with the chronic effects of previous chemotherapy.<br>■ However, the long interval (5 years) since chemotherapy makes it less likely compared to more immediate post-chemotherapy onset, though delayed neuropathy is possible.<br>3. **Idiopathic Sensory Polyneuropathy**<br>○ **Reasoning:**<br>■ This diagnosis is considered when all specific causes of neuropathy have been excluded.<br>■ The patient's normal screening labs for common causes of neuropathy (vitamin deficiencies, diabetes, infections, inflammatory markers) support this as a potential diagnosis.<br>■ Idiopathic sensory polyneuropathy typically presents with a similar clinical picture: insidious onset, slow progression, and predominantly sensory symptoms without motor involvement or systemic disease.<br>**Most Likely Diagnosis**<br>**Monoclonal Gammopathy of Undetermined Significance (MGUS)-associated Polyneuropathy**<br>● **Reasoning:**<br>○ The presence of IgG kappa paraprotein and the patient's history of MGUS strongly suggest a link between his neuropathy and MGUS.<br>○ MGUS-associated neuropathy typically presents as a sensory-predominant, symmetrical, length-dependent neuropathy, fitting the patient's clinical presentation perfectly.<br>○ Other common causes of neuropathy have been ruled out with normal lab tests.<br>○ Although chemotherapy-induced neuropathy is possible, the timeline and clinical course are more consistent with MGUS-associated neuropathy.
36	A 75-year-old male patient presents with a four-year history of pure sensory, symmetrical, length-dependent polyneuropathy with insidious onset and slow progression. The patient reports sensory ataxia, hypoesthesia in the distal lower limbs up to the calves, mild dysphagia and dysarthria, with no distal weakness. Past medical history is positive for chronic cough. Nerve conduction studies show an axonal, symmetric, length-dependent, pure sensory polyneuropathy. Screening lab tests, including vitamin B12, folate, vitamin B6, vitamin E, HBV, HCV, glycemia, HbA1c, ANA, ANCA, CRP, ESR, copper, cryoglobulins, and serum electrophoresis are all negative, with positivity for ENA autoantibodies. There is no family history of polyneuropathy.	CANVAS	**Step-by-Step Analysis of the Clinical Scenario**<br>**Patient Summary:**<br>● **Age/Gender:** 75-year-old male<br>● **History:**<br>○ Four-year history of pure sensory, symmetrical, length-dependent polyneuropathy<br>○ Insidious onset and slow progression<br>○ Symptoms: sensory ataxia, hypoesthesia in distal lower limbs up to the calves, mild dysphagia and dysarthria<br>○ No distal weakness<br>● **Past Medical History:** Chronic cough<br>● **Nerve Conduction Studies:** Axonal, symmetric, length-dependent, pure sensory polyneuropathy<br>● **Laboratory Findings:**<br>○ Negative: Vitamin B12, folate, vitamin B6, vitamin E, HBV, HCV, glycemia, HbA1c, ANA, ANCA, CRP, ESR, copper, cryoglobulins, and serum electrophoresis<br>○ Positive: ENA (Extractable Nuclear Antigen) autoantibodies<br>● **Family History:** No family history of polyneuropathy<br>**Differential Diagnoses**<br>1. **Paraneoplastic Neuropathy**<br>○ **Reasoning:**<br>■ **Symptoms:** The presence of sensory ataxia and sensory polyneuropathy with an insidious onset and slow progression fits the profile of paraneoplastic neuropathy. The chronic cough might be a sign of an underlying malignancy (e.g., lung cancer), which is a common association.<br>■ **Positive ENA autoantibodies:** These are often seen in paraneoplastic syndromes.<br>■ **Age and presentation:** The elderly population is at a higher risk for malignancies, making this a plausible diagnosis.<br>2. **Primary Sjögren's Syndrome (PSS)**<br>○ **Reasoning:**<br>■ **Symptoms:** Sensory neuropathy, especially without motor involvement, is common in PSS. The patient's mild dysphagia and dysarthria may be indicative of sicca symptoms often seen in Sjögren's syndrome.<br>■ **Positive ENA autoantibodies:** Autoantibodies such as anti-Ro/SSA and anti-La/SSB, which are part of the ENA panel, are commonly positive in PSS.<br>■ **Chronic cough:** This could be secondary to xerotrachea, another manifestation of Sjögren's syndrome.<br>3. **Chronic Inflammatory Demyelinating Polyneuropathy (CIDP) with Sensory Predominance**<br>○ **Reasoning:**<br>■ **Symptoms:** Although CIDP typically has motor involvement, there are sensory predominant variants. The length-dependent, symmetrical nature of the neuropathy fits.<br>■ **ENA autoantibodies:** While not typical, there can be some overlap with autoimmune features.<br>■ **Chronic cough:** This is less directly related but might be incidental or a manifestation of a mild systemic inflammatory state.<br>**Most Likely Diagnosis**<br>Given the patient's positive ENA autoantibodies, the chronic cough, and the sensory-dominant nature of the polyneuropathy, the most likely diagnosis is **Primary Sjögren's Syndrome (PSS)**. This is because PSS commonly presents with sensory polyneuropathy and can be associated with other systemic features such as dry mouth (leading to dysphagia and dysarthria) and dry airways (leading to chronic cough). The presence of ENA autoantibodies strongly supports this autoimmune etiology.<br>**Conclusion**<br>The most likely diagnosis for this patient is Primary Sjögren's Syndrome (PSS). This diagnosis fits the clinical presentation and the serological findings, explaining the sensory neuropathy and other accompanying symptoms. Further investigations, such as a minor salivary gland biopsy or further serological testing for specific antibodies like anti-Ro/SSA and anti-La/SSB, may be warranted to confirm the diagnosis.
37	A 58-year-old female patient presents with a 13-month history of sensory-motor, symmetrical, length dependent polyneuropathy with subacute onset and progressive course. The patient presents with distal weakness, neuropathic pain, and paresthesia in the four limbs. Clinical examination shows the presence of mild	POEMS	**Clinical Information Summary**<br>● **Patient Profile**: 58-year-old female<br>● **Symptom Duration**: 13-month history<br>● **Symptoms**: Sensory-motor, symmetrical, length-dependent polyneuropathy with subacute onset and progressive course; distal weakness, neuropathic pain, and paresthesia in all four limbs; mild intrinsic hand atrophy; peripheral edema.<br>● **Clinical Findings**: No sensory ataxia, tremor, foot deformities, cranial nerve or autonomic involvement.<br>● **Past Medical History**: Occasional alcohol use.<br>● **Nerve Conduction Studies**: Mixed demyelinating-axonal, length-dependent, sensory-motor polyneuropathy.

	intrinsic hand atrophy and a peripheral edema. There are no sensory ataxia, tremor, foot deformities, cranial nerve or autonomic involvement. Past medical history is positive for occasional alcohol use. Nerve conduction studies show a mixed demyelinating-axonal, length dependent, sensory-motor polyneuropathy. Screening lab tests including vitamin B12, folate, vitamin B6, vitamin E, HCV, HBV, ANA, ANCA, ENA, CRP, ESR, glycemia, HbA1c, copper, and cryoglobulins are negative, while serum electrophoresis shows positivity for IgG lambda paraprotein. A cardiac ultrasound, performed to investigate edema in the lower limbs, revealed the presence of pericardial effusion. There is no family history of polyneuropathy.		● **Screening Lab Tests**: Negative for vitamin B12, folate, vitamin B6, vitamin E, HCV, HBV, ANA, ANCA, ENA, CRP, ESR, glycemia, HbA1c, copper, and cryoglobulins.<br>● **Serum Electrophoresis**: Positive for IgG lambda paraprotein.<br>● **Cardiac Ultrasound**: Presence of pericardial effusion.<br>● **Family History**: No family history of polyneuropathy.<br>**Differential Diagnoses**<br>**1. Monoclonal Gammopathy of Undetermined Significance (MGUS) with Neuropathy**<br>**Reasoning**:<br>● **IgG Lambda Paraprotein**: The presence of monoclonal gammopathy (IgG lambda paraprotein) is a significant finding. MGUS is known to cause neuropathy in some cases.<br>● **Neuropathy Type**: The mixed demyelinating-axonal pattern fits with neuropathy associated with monoclonal gammopathy.<br>● **Subacute and Progressive Course**: This matches the typical course of neuropathy associated with MGUS.<br>● **Pericardial Effusion**: This can occur in MGUS due to amyloid deposits or other mechanisms, though it is not highly specific.<br>**2. Chronic Inflammatory Demyelinating Polyneuropathy (CIDP)**<br>**Reasoning**:<br>● **Clinical Features**: Symmetrical, sensory-motor polyneuropathy with both demyelinating and axonal features is characteristic of CIDP.<br>● **Progressive Course**: The subacute onset and progressive nature align with CIDP.<br>● **Negative Laboratory Results**: CIDP is often a diagnosis of exclusion when common causes of neuropathy are ruled out.<br>● **Response to Treatment**: CIDP is potentially treatable with immunotherapy, which can be a distinguishing factor.<br>**3. Amyloidosis**<br>**Reasoning**:<br>● **Peripheral Edema and Pericardial Effusion**: These are classic signs of systemic amyloidosis, which can also cause neuropathy.<br>● **Paraprotein Presence**: The IgG lambda paraprotein could indicate an underlying plasma cell dyscrasia associated with amyloidosis.<br>● **Neuropathy Type**: Amyloid neuropathy often presents as a length-dependent, sensory-motor polyneuropathy with both axonal and demyelinating features.<br>● **Subacute and Progressive Course**: Fits the pattern of neuropathy in amyloidosis.<br>**Most Likely Diagnosis**<br>The most likely diagnosis is **Monoclonal Gammopathy of Undetermined Significance (MGUS) with Neuropathy**. This diagnosis is supported by the presence of the IgG lambda paraprotein, which is a hallmark of MGUS, and the clinical and electrophysiological characteristics of the patient's neuropathy. Additionally, while pericardial effusion and peripheral edema could suggest amyloidosis, the absence of other typical signs and the predominant findings support MGUS-related neuropathy. Further investigations, such as bone marrow biopsy and evaluation for amyloidosis, might be necessary to confirm the diagnosis and rule out related conditions.
38	A 30-year-old female patient presents with a six-month history of right peroneal mononeuropathy (foot drop) with acute onset. She also reports the presence of mild paresthesia in the fourth and fifth fingers of the left hand over the past two years, and frequent hand paresthesia after using scissors at work. Nerve conduction studies show a mild demyelinating, sensory-motor polyneuropathy, with right carpal tunnel syndrome, left ulnar entrapment at the elbow, and right peroneal nerve entrapment at the fibular head. Screening lab tests including vitamin B12, folate, vitamin B6, vitamin E, HBV, HCV, glycemia, HbA1c, ANA, ANCA, ENA, CRP, ESR, copper, cryoglobulins, and serum electrophoresis are all negative. There is no family history of polyneuropathy.	HNPP	**Clinical Summary**<br>A 30-year-old female patient presents with:<br>● **Primary Complaint:** Six-month history of right peroneal mononeuropathy (foot drop) with acute onset.<br>● **Additional Symptoms:**<br>○ Mild paresthesia in the fourth and fifth fingers of the left hand over the past two years.<br>○ Frequent hand paresthesia after using scissors at work.<br>● **Nerve Conduction Studies Findings:**<br>○ Mild demyelinating, sensory-motor polyneuropathy.<br>○ Right carpal tunnel syndrome.<br>○ Left ulnar entrapment at the elbow.<br>○ Right peroneal nerve entrapment at the fibular head.<br>● **Laboratory Findings:** Screening lab tests are all negative, including vitamin B12, folate, vitamin B6, vitamin E, HBV, HCV, glycemia, HbA1c, ANA, ANCA, ENA, CRP, ESR, copper, cryoglobulins, and serum electrophoresis.<br>● **Family History:** No family history of polyneuropathy.<br>**Differential Diagnoses for Polyneuropathy**<br>1. **Hereditary Neuropathy with Liability to Pressure Palsies (HNPP)**<br>○ **Reasoning:**<br>■ **Multiple Nerve Entrapments:** The patient has multiple entrapment neuropathies (right carpal tunnel syndrome, left ulnar entrapment, right peroneal nerve entrapment), which is characteristic of HNPP.<br>■ **Demographic:** Onset in a young adult (30 years old) is consistent with HNPP, which often presents in early adulthood.<br>■ **Negative Lab Tests:** The negative results for various inflammatory and metabolic conditions make a hereditary cause more likely.<br>■ **Symptom Fluctuation:** The mild paresthesia in the hands exacerbated by repetitive activities (using scissors) aligns with the intermittent and activity-related nature of HNPP.<br>2. **Chronic Inflammatory Demyelinating Polyneuropathy (CIDP)**<br>○ **Reasoning:**<br>■ **Demyelinating Neuropathy:** The nerve conduction studies show mild demyelination, which can be seen in CIDP.<br>■ **Sensory-Motor Involvement:** The presence of sensory-motor polyneuropathy is consistent with CIDP, which can affect both sensory and motor fibers.<br>■ **Chronic Course:** The progressive nature of symptoms over months to years fits with the chronic progression seen in CIDP.<br>■ **Negative Laboratory Tests:** While CIDP is an autoimmune disorder, the lack of specific serum markers does not rule it out, as many patients can have negative autoimmune panels.<br>3. **Multifocal Motor Neuropathy (MMN)**<br>○ **Reasoning:**<br>■ **Motor Predominance:** Although typically a motor neuropathy, MMN can present with asymmetric motor deficits like the acute onset of foot drop.<br>■ **Electrodiagnostic Findings:** The demyelinating features on nerve conduction studies could be seen in MMN.<br>■ **Entrapment Neuropathies:** Multiple nerve entrapments could suggest an underlying multifocal neuropathy.<br>■ **Autoimmune Nature:** Similar to CIDP, MMN is an immune-mediated condition and might not always show positive specific serum markers in early stages.<br>**Most Likely Diagnosis**<br>The most likely diagnosis for this patient is **Hereditary Neuropathy with Liability to Pressure Palsies (HNPP)**. This conclusion is drawn based on the following points:<br>● The presence of multiple entrapment neuropathies, which is a hallmark of HNPP.<br>● The young age of onset and the lack of family history, which does not rule out HNPP as it can present with de novo mutations.

			● Negative lab results for a wide range of other possible causes, strengthening the likelihood of a hereditary etiology.<br>● The pattern of intermittent and activity-related symptoms (such as paresthesia exacerbated by using scissors), which is typical of HNPP.<br>Further genetic testing, specifically for the PMP22 gene deletion or mutation, would be needed to confirm the diagnosis of HNPP.
39	A 76-year-old male patient presents with an 8-year history of chronic, pure sensory, symmetrical, length dependent polyneuropathy with insidious onset and slow progressive course. The patient presents with severe neuropathic pain and heat intolerance in the feet. He does not report weakness, ataxia, paresthesia, tremor, cranial nerve or autonomic involvement. Clinical examination reveals the presence of allodynia and hyperalgesia in the feet. There are no foot deformities. Past medical history is positive for prostatic cancer. Nerve conduction studies are normal. Screening lab tests including vitamin B12, folate, vitamin B6, vitamin E, HBV, HCV, ANA, ANCA, ENA, CRP, ESR, glycemia, HbA1c, copper, cryoglobulins, and serum electrophoresis are negative. There is no family history of polyneuropathy.	Small fibre neuropathy	**Summary of Pertinent Clinical Information**<br>● **Patient Demographics**: 76-year-old male<br>● **Duration and Course**: 8-year history, chronic, pure sensory, symmetrical, length-dependent polyneuropathy with insidious onset and slow progression<br>● **Symptoms**: Severe neuropathic pain and heat intolerance in the feet<br>● **Examination Findings**: Allodynia and hyperalgesia in the feet, no foot deformities<br>● **Negative Findings**: No weakness, ataxia, paresthesia, tremor, cranial nerve involvement, or autonomic involvement<br>● **Past Medical History**: Prostatic cancer<br>● **Diagnostic Studies**:<br>○ Nerve conduction studies: Normal<br>○ Screening lab tests: Negative for vitamin B12, folate, vitamin B6, vitamin E, HBV, HCV, ANA, ANCA, ENA, CRP, ESR, glycemia, HbA1c, copper, cryoglobulins, serum electrophoresis<br>● **Family History**: Negative for polyneuropathy<br>**Differential Diagnoses for Polyneuropathy**<br>1. **Idiopathic Small Fiber Neuropathy**<br>○ **Reasoning**:<br>■ The presentation of severe neuropathic pain, allodynia, and hyperalgesia with normal nerve conduction studies strongly suggests small fiber involvement.<br>■ The chronic, length-dependent nature, and absence of large fiber involvement (no weakness or ataxia) support this diagnosis.<br>■ Normal nerve conduction studies often occur in small fiber neuropathy as these tests primarily assess large fiber function.<br>■ The absence of metabolic, autoimmune, and infectious causes on screening tests suggests an idiopathic origin.<br>2. **Chemotherapy-Induced Peripheral Neuropathy (CIPN)**<br>○ **Reasoning**:<br>■ The patient's history of prostatic cancer may imply prior exposure to chemotherapeutic agents, which are known to cause peripheral neuropathy.<br>■ CIPN can present as a pure sensory neuropathy and typically affects the feet in a length-dependent pattern.<br>■ The chronic nature and progression could be consistent with delayed or persistent neuropathy post-chemotherapy.<br>■ Normal nerve conduction studies can be seen in small fiber CIPN.<br>3. **Paraneoplastic Neuropathy**<br>○ **Reasoning**:<br>■ Although less likely given the chronic, slow progression, paraneoplastic neuropathy must be considered due to the patient's history of prostatic cancer.<br>■ These neuropathies can present with pure sensory symptoms and are often length-dependent.<br>■ The insidious onset and absence of autonomic symptoms are consistent, though paraneoplastic syndromes often present more acutely and with other neurological signs.<br>■ Negative paraneoplastic antibody panels (ANA, ANCA, ENA) make this less likely, but paraneoplastic syndromes can still occur without detectable antibodies.<br>**Most Likely Diagnosis**<br>The most likely diagnosis is **Idiopathic Small Fiber Neuropathy**. This diagnosis aligns best with the patient's presentation of severe neuropathic pain, heat intolerance, allodynia, and hyperalgesia in the feet, coupled with normal nerve conduction studies. The absence of significant findings on an extensive array of metabolic, infectious, and autoimmune tests supports an idiopathic etiology. Additionally, the patient's symptoms are characteristic of small fiber involvement, which is often not detected on standard nerve conduction studies.
40	A 79-year-old male patient presents with a 2-year history of pure sensory, symmetrical, length dependent polyneuropathy with insidious onset. The patient presents with paresthesia, hypoesthesia and neuropathic pain in the distal areas of the lower limbs. There are no ataxia, tremor, foot deformities, cranial nerve or autonomic involvement. Past medical history is silent. Nerve conduction studies show pure sensory, axonal polyneuropathy. Screening lab tests including vitamin B12, folate, vitamin B6, vitamin E, HCV, ANA, ANCA, ENA, CRP, ESR, glycemia, HbA1c, copper, cryoglobulins and serum electrophoresis are all negative, with evidence of chronic HBV infection. There is no family history of polyneuropathy.	HBV-associated neuropathy	**Summary of Pertinent Clinical Information**<br>**Patient Profile:**<br>● **Age:** 79-year-old male<br>● **History:** 2-year history of pure sensory, symmetrical, length-dependent polyneuropathy with insidious onset<br>● **Symptoms:** Paresthesia, hypoesthesia, and neuropathic pain in distal areas of lower limbs<br>● **Negative Symptoms:** No ataxia, tremor, foot deformities, cranial nerve or autonomic involvement<br>**Investigations:**<br>● **Nerve Conduction Studies:** Pure sensory, axonal polyneuropathy<br>● **Screening Lab Tests:**<br>○ Negative: Vitamin B12, folate, vitamin B6, vitamin E, HCV, ANA, ANCA, ENA, CRP, ESR, glycemia, HbA1c, copper, cryoglobulins, serum electrophoresis<br>○ Positive: Chronic HBV infection<br>● **Family History:** No family history of polyneuropathy<br>**Differential Diagnoses for Polyneuropathy**<br>1. **Chronic HBV Infection-Associated Neuropathy**<br>○ **Reasoning:**<br>■ The patient has a confirmed chronic HBV infection.<br>■ Chronic viral infections, including HBV, can be associated with peripheral neuropathy due to immune-mediated mechanisms or direct viral effects on the nerves.<br>■ Although less common than HCV, HBV can cause similar neuropathic conditions.<br>2. **Idiopathic Sensory Neuropathy**<br>○ **Reasoning:**<br>■ A significant proportion of patients with pure sensory axonal polyneuropathy are ultimately diagnosed with idiopathic neuropathy when no definitive cause is identified.<br>■ The extensive negative workup for common causes (e.g., diabetes, nutritional deficiencies, autoimmune diseases) suggests that the neuropathy might be idiopathic.<br>■ The patient's advanced age further increases the likelihood of idiopathic neuropathy, as it is more common in older adults.<br>3. **Paraneoplastic Neuropathy**<br>○ **Reasoning:**<br>■ The insidious onset and pure sensory nature of the neuropathy can sometimes be associated with a paraneoplastic syndrome, where cancer triggers an immune response that affects the nerves.

			■ Although the patient's past medical history is silent and no cancer screening results are mentioned, paraneoplastic syndromes can present with a variety of neuropathic patterns.<br>■ The absence of other common causes makes it necessary to consider paraneoplastic neuropathy, especially in an older patient who may be at higher risk for occult malignancies.<br>**Most Likely Diagnosis**<br>**Chronic HBV Infection-Associated Neuropathy** appears to be the most likely diagnosis given the confirmed chronic HBV infection and the exclusion of other common causes through comprehensive testing. This diagnosis is supported by the known association between chronic HBV and peripheral neuropathy, although it is less common compared to HCV-related neuropathy. The patient's age and negative screening results for other conditions make idiopathic neuropathy a close differential, but the presence of HBV infection is a significant finding that aligns with the clinical presentation.
41	An 82-year-old female patient presents with a 4-year history of pure sensory, length dependent, symmetric polyneuropathy with insidious onset and slow progressive course. The patient presents with paresthesia and mild neuropathic pain in the feet. Clinical examination does not show weakness or sensory ataxia, tremor, foot deformities, cranial nerve or autonomic involvement. Past medical history is positive for IgA kappa monoclonal gammopathy, arterial hypertension, gout, hypercholesterolemia, and cataract. Nerve conduction studies show presence of a pure sensory, length dependent, symmetric, axonal polyneuropathy. Screening lab tests including vitamin B12, folate, vitamin B6, vitamin E, HBV, HCV, glycemia, HbA1c, ANA, ANCA, ENA, CRP, ESR, copper, and cryoglobulins are negative, with positivity to IgA kappa paraprotein. There is no family history of polyneuropathy.	MGUS-associated neuropathy	**Summary of Clinical Information**<br>**Patient Profile:**<br>● Age: 82-year-old female<br>● History: 4-year history of pure sensory, length-dependent, symmetric polyneuropathy<br>● Symptoms: Paresthesia and mild neuropathic pain in the feet<br>● Onset: Insidious and slow progressive course<br>**Clinical Examination:**<br>● No weakness<br>● No sensory ataxia<br>● No tremor<br>● No foot deformities<br>● No cranial nerve involvement<br>● No autonomic involvement<br>**Past Medical History:**<br>● IgA kappa monoclonal gammopathy<br>● Arterial hypertension<br>● Gout<br>● Hypercholesterolemia<br>● Cataract<br>**Diagnostic Studies:**<br>● Nerve conduction studies: Pure sensory, length-dependent, symmetric, axonal polyneuropathy<br>● Lab tests:<br>○ Negative: Vitamin B12, folate, vitamin B6, vitamin E, HBV, HCV, glycemia, HbA1c, ANA, ANCA, ENA, CRP, ESR, copper, cryoglobulins<br>○ Positive: IgA kappa paraprotein<br>**Family History:**<br>● No family history of polyneuropathy<br>**Differential Diagnoses for Polyneuropathy**<br>1. **Monoclonal Gammopathy of Undetermined Significance (MGUS)-Related Neuropathy**<br>○ **Reasoning:**<br>■ Presence of IgA kappa monoclonal gammopathy, which is associated with MGUS.<br>■ MGUS can be asymptomatic or present with polyneuropathy, often of the axonal type, matching the nerve conduction study findings.<br>■ The slow progression and sensory nature of the neuropathy fit the typical presentation of MGUS-related neuropathy.<br>2. **Chronic Idiopathic Axonal Polyneuropathy (CIAP)**<br>○ **Reasoning:**<br>■ Common in elderly patients with a slowly progressive, length-dependent sensory polyneuropathy.<br>■ Negative lab tests for metabolic, infectious, and autoimmune causes support this diagnosis.<br>■ The absence of systemic symptoms and specific laboratory abnormalities aligns with idiopathic causes.<br>3. **Paraproteinemic Neuropathy**<br>○ **Reasoning:**<br>■ The presence of IgA kappa paraprotein raises the possibility of a neuropathy associated with paraproteinemia.<br>■ Paraproteinemic neuropathies can present similarly with a sensory-predominant, length-dependent, and slowly progressive course.<br>■ This condition often lacks additional systemic involvement, aligning with the patient's clinical presentation.<br>**Most Likely Diagnosis**<br>The most likely diagnosis for this patient is **Monoclonal Gammopathy of Undetermined Significance (MGUS)-Related Neuropathy**. This diagnosis is strongly supported by the presence of IgA kappa monoclonal gammopathy, the clinical presentation of pure sensory, symmetric polyneuropathy, and the exclusion of other common causes through comprehensive lab testing. MGUS is known to be associated with peripheral neuropathy, particularly in the elderly, and fits well with the patient's symptoms and test results.
42	A 47-year-old male patient presents with a 1-year history of pure motor, asymmetrical multineuropathy with subacute onset. Clinical examination reveals the presence of weakness and atrophy in a multifocal distribution in the upper limbs. There are no sensory deficits or subjective numbness, tingling or pain, and physical examination does not show the presence of sensory ataxia, tremor, foot deformities, cranial nerve or autonomic involvement. Past	MMN	**Summary of Pertinent Clinical Information**<br>● **Patient Demographics**: 47-year-old male<br>● **Symptoms**: 1-year history of pure motor, asymmetrical multineuropathy with subacute onset<br>● **Clinical Findings**:<br>○ Weakness and atrophy in a multifocal distribution in the upper limbs<br>○ No sensory deficits (no numbness, tingling, or pain)<br>○ No sensory ataxia, tremor, foot deformities<br>○ No cranial nerve or autonomic involvement<br>● **Past Medical History**: Unremarkable<br>● **Nerve Conduction Studies**: Pure motor, demyelinating multineuropathy with nerve conduction blocks<br>● **Lab Results**: Negative for vitamin B12, folate, vitamin B6, vitamin E, HCV, HBV, ANA, ANCA, ENA, CRP, ESR, glycemia, HbA1c, copper, cryoglobulins, and serum electrophoresis

	medical history is silent. Nerve conduction studies show pure motor, demyelinating multineuropathy with nerve conduction blocks. Screening lab tests including vitamin B12, folate, vitamin B6, vitamin E, HCV, HBV, ANA, ANCA, ENA, CRP, ESR, glycemia, HbA1c, copper, cryoglobulins and serum electrophoresis are all negative. There is no family history of polyneuropathy.		● **Family History**: No family history of polyneuropathy<br>**Differential Diagnoses for Polyneuropathy**<br>**1. Multifocal Motor Neuropathy (MMN)**<br>● **Reasoning**:<br>○ **Clinical Presentation**: MMN typically presents with pure motor deficits without sensory involvement, which aligns with the patient's symptoms.<br>○ **Electrophysiological Findings**: The presence of demyelinating features with nerve conduction blocks is characteristic of MMN.<br>○ **Pattern of Weakness**: Asymmetrical and multifocal weakness, as seen in this patient, is a hallmark of MMN.<br>○ **Age of Onset**: The patient's age fits within the common age range for MMN presentation.<br>● **Supporting Evidence**: MMN is a likely diagnosis due to the combination of pure motor involvement, demyelinating features on nerve conduction studies, and the pattern of weakness.<br>**2. Amyotrophic Lateral Sclerosis (ALS)**<br>● **Reasoning**:<br>○ **Clinical Presentation**: ALS often presents with motor neuron degeneration leading to weakness and atrophy. However, ALS typically involves both upper and lower motor neuron signs and progresses more rapidly.<br>○ **Electrophysiological Findings**: ALS can show motor neuron involvement, but demyelinating features are not typical.<br>○ **Absence of Sensory Involvement**: The absence of sensory deficits fits ALS, but the asymmetry and nerve conduction blocks are less typical.<br>● **Supporting Evidence**: While the pure motor involvement and lack of sensory deficits are consistent with ALS, the demyelinating features and nerve conduction blocks make it less likely.<br>**3. Chronic Inflammatory Demyelinating Polyneuropathy (CIDP)**<br>● **Reasoning**:<br>○ **Clinical Presentation**: CIDP can present with both motor and sensory involvement, but pure motor CIDP is a recognized variant.<br>○ **Electrophysiological Findings**: Demyelinating features and conduction blocks are common in CIDP.<br>○ **Pattern of Weakness**: CIDP typically presents with symmetrical weakness, though asymmetrical forms can occur.<br>○ **Screening Labs**: Negative lab tests are not unusual in CIDP, but other supporting evidence such as elevated protein in cerebrospinal fluid (CSF) might be required.<br>● **Supporting Evidence**: CIDP is a consideration due to the demyelinating features, but the pure motor and asymmetrical presentation are less typical.<br>**Most Likely Diagnosis**<br>The most likely diagnosis for this patient is **Multifocal Motor Neuropathy (MMN)**. This is based on the following points:<br>● The clinical presentation of pure motor deficits without sensory involvement.<br>● The asymmetrical and multifocal distribution of weakness and atrophy.<br>● The electrophysiological findings of demyelinating features with conduction blocks, which are highly characteristic of MMN.<br>● The age of the patient fits within the common range for MMN.<br>Given the combination of these factors, MMN stands out as the diagnosis that most closely matches the patient's clinical picture and investigation results.
43	An 88-year-old male patient presents with a 4-year history of sensory-motor, symmetrical, length dependent polyneuropathy with insidious onset and slow progressive course. The patient presents with paresthesia and neuropathic pain distally in the lower limbs, with no ataxia, tremor, foot deformities, cranial nerve or autonomic involvement. Past medical history is positive for type 2 diabetes, cardiac insufficiency and lung cancer, for which he underwent chemotherapy 15 years ago. Nerve conduction studies show a sensory-motor, symmetric, axonal polyneuropathy. Screening lab tests including vitamin B12, folate, vitamin B6, vitamin E, HBV, HCV, ANA, ANCA, ENA, CRP, ESR, copper, cryoglobulins and serum electrophoresis are all negative, with evidence of hyperglycaemia and elevated HbA1c. There is no family history of polyneuropathy.	Diabetic neuropathy	**Summary of Pertinent Clinical Information**<br>● **Patient Profile:** 88-year-old male<br>● **History:**<br>○ 4-year history of sensory-motor, symmetrical, length-dependent polyneuropathy<br>○ Insidious onset and slow progressive course<br>○ Paresthesia and neuropathic pain in lower limbs<br>○ No ataxia, tremor, foot deformities, cranial nerve, or autonomic involvement<br>● **Past Medical History:**<br>○ Type 2 diabetes mellitus<br>○ Cardiac insufficiency<br>○ Lung cancer (chemotherapy 15 years ago)<br>● **Diagnostic Findings:**<br>○ Nerve conduction studies: sensory-motor, symmetric, axonal polyneuropathy<br>○ Screening lab tests: Negative for vitamin B12, folate, vitamin B6, vitamin E, HBV, HCV, ANA, ANCA, ENA, CRP, ESR, copper, cryoglobulins, and serum electrophoresis<br>○ Hyperglycemia and elevated HbA1c<br>● **Family History:** No family history of polyneuropathy<br>**Differential Diagnoses for Polyneuropathy**<br>1. **Diabetic Polyneuropathy (DPN):**<br>○ **Reasoning:**<br>■ The patient has a history of type 2 diabetes mellitus with hyperglycemia and elevated HbA1c levels, indicating poor glycemic control.<br>■ Diabetic polyneuropathy is the most common cause of polyneuropathy in diabetic patients.<br>■ The clinical presentation of symmetrical, distal, sensory-motor polyneuropathy with a gradual onset and progression is typical of diabetic neuropathy.<br>■ Absence of autonomic involvement and cranial nerve involvement is consistent with the patient's presentation.<br>2. **Chemotherapy-Induced Peripheral Neuropathy (CIPN):**<br>○ **Reasoning:**<br>■ The patient underwent chemotherapy for lung cancer 15 years ago, and certain chemotherapeutic agents (e.g., platinum compounds, taxanes, and vinca alkaloids) are known to cause delayed-onset peripheral neuropathy.<br>■ The pattern of sensory-motor, symmetrical, axonal polyneuropathy could be consistent with CIPN, although the long interval since chemotherapy makes this diagnosis less likely.<br>■ The absence of progression over a decade after chemotherapy and the presence of ongoing hyperglycemia favor other diagnoses.<br>3. **Chronic Idiopathic Axonal Polyneuropathy (CIAP):**<br>○ **Reasoning:**<br>■ CIAP typically presents in older adults with a slowly progressive, length-dependent sensory-motor polyneuropathy.<br>■ The absence of a specific cause after extensive workup, including negative screening for vitamin deficiencies, infections, autoimmune markers, and normal serum electrophoresis, supports CIAP as a potential diagnosis.

			■ The patient's age and gradual progression of symptoms without any other identifiable cause fit the profile for CIAP.<br>**Most Likely Diagnosis**<br>Based on the clinical presentation, past medical history, and diagnostic findings, the most likely diagnosis is **Diabetic Polyneuropathy (DPN)**. The patient's long-standing history of diabetes mellitus, poor glycemic control (evidenced by hyperglycemia and elevated HbA1c), and the characteristic pattern of neuropathy strongly support this diagnosis. The slow progression and absence of autonomic or cranial nerve involvement further align with diabetic neuropathy, making it the most probable cause of the patient's symptoms.
44	An 81-year-old male patient presents with a six-month history of sensory-motor, symmetrical, length-dependent polyneuropathy with subacute onset and slow progression. The patient reports neuropathic pain in the lower limbs in a distal, symmetric distribution, along with sensory ataxia. Clinical examination does not reveal weakness, tremor, foot deformities, cranial nerve or autonomic involvement. Past medical history is positive for IgM kappa monoclonal gammopathy, Waldenström macroglobulinemia, and colorectal cancer, for which he underwent chemotherapy five years ago. Nerve conduction studies show a length-dependent, sensorimotor, symmetric, demyelinating polyneuropathy. Screening lab tests, including vitamin B12, folate, vitamin B6, vitamin E, HCV, HBV, ANA, ANCA, ENA, CRP, ESR, glycemia, HbA1c, copper, and cryoglobulins are all normal, with evidence of IgM kappa paraprotein. There is no family history of polyneuropathy.	Anti-MAG polyneuropathy	**Clinical Summary**<br>**Patient Information:**<br>● **Age:** 81-year-old male<br>● **History Duration:** 6 months<br>● **Symptoms:**<br>○ Sensory-motor, symmetrical, length-dependent polyneuropathy<br>○ Subacute onset with slow progression<br>○ Neuropathic pain in lower limbs (distal, symmetric distribution)<br>○ Sensory ataxia<br>**Clinical Examination:**<br>● No weakness<br>● No tremor<br>● No foot deformities<br>● No cranial nerve or autonomic involvement<br>**Past Medical History:**<br>● IgM kappa monoclonal gammopathy<br>● Waldenström macroglobulinemia<br>● Colorectal cancer (chemotherapy completed 5 years ago)<br>**Investigations:**<br>● **Nerve Conduction Studies:** Length-dependent, sensorimotor, symmetric, demyelinating polyneuropathy<br>● **Screening Lab Tests:** Normal results for:<br>○ Vitamin B12, folate, vitamin B6, vitamin E<br>○ Hepatitis C Virus (HCV), Hepatitis B Virus (HBV)<br>○ ANA, ANCA, ENA<br>○ CRP, ESR<br>○ Glycemia, HbA1c<br>○ Copper<br>○ Cryoglobulins<br>● Evidence of IgM kappa paraprotein<br>● No family history of polyneuropathy<br>**Differential Diagnoses for Polyneuropathy**<br>1. **Waldenström Macroglobulinemia-related Neuropathy**<br>○ **Reasoning:**<br>■ The patient has a history of Waldenström macroglobulinemia, which is associated with IgM monoclonal gammopathy.<br>■ IgM paraproteins can cause a demyelinating neuropathy by binding to myelin-associated glycoprotein (MAG) or other neural antigens, leading to nerve damage.<br>■ The clinical picture of a slowly progressive, length-dependent, demyelinating polyneuropathy fits well with Waldenström macroglobulinemia-related neuropathy.<br>■ Absence of autonomic and cranial nerve involvement also aligns with this diagnosis.<br>2. **Monoclonal Gammopathy of Undetermined Significance (MGUS) Neuropathy**<br>○ **Reasoning:**<br>■ The patient has IgM kappa monoclonal gammopathy, which can be a part of MGUS.<br>■ MGUS can be associated with a peripheral neuropathy, often presenting with similar symptoms as described (symmetrical, length-dependent, and demyelinating).<br>■ The absence of other systemic features and normal results in a wide array of screening tests suggest the neuropathy could be related to the monoclonal protein itself.<br>■ MGUS-related neuropathy can be indolent and slowly progressive, which matches the patient's clinical course.<br>3. **Chemotherapy-induced Neuropathy**<br>○ **Reasoning:**<br>■ The patient underwent chemotherapy for colorectal cancer 5 years ago, which could have led to a delayed onset of neuropathy.<br>■ Some chemotherapeutic agents are known to cause delayed neuropathy, particularly those that are platinum-based or taxanes.<br>■ However, chemotherapy-induced neuropathy typically presents as axonal rather than demyelinating, making this less likely compared to the other two diagnoses.<br>■ The time lapse of five years post-chemotherapy also makes this diagnosis less probable as a sole cause.<br>**Most Likely Diagnosis**<br>**Waldenström Macroglobulinemia-related Neuropathy**<br>● Given the patient's history of Waldenström macroglobulinemia, the presence of IgM kappa paraprotein, and the typical presentation of a demyelinating polyneuropathy, this diagnosis is the most likely.<br>● The subacute onset, slow progression, and specific electrophysiological findings (demyelinating polyneuropathy) strongly support this diagnosis over MGUS or chemotherapy-induced neuropathy.<br>● Management would involve addressing the underlying hematological condition, potentially with therapies targeting the IgM paraprotein or the Waldenström macroglobulinemia itself.
45	An 83-year-old male patient presents with a 3-year history of sensory, symmetrical, length dependent polyneuropathy with insidious onset. The patient presents with	MGUS-associated neuropathy	**Step-by-Step Analysis of Clinical Scenario**<br>**Clinical Summary**<br>● **Patient Demographics**: 83-year-old male<br>● **History**: 3-year history of sensory, symmetrical, length-dependent polyneuropathy with an insidious onset

	paresthesia and hypoesthesia in the lower limbs without weakness or sensory ataxia. Clinical examination does not show the presence of tremor, neuropathic pain, foot deformities, cranial nerve or autonomic involvement. Past medical history is positive for IgG lambda monoclonal gammopathy. Nerve conduction studies show axonal, length dependent, sensory-motor polyneuropathy and unilateral carpal tunnel syndrome. Screening lab tests including vitamin B12, folate, vitamin B6, vitamin E, HCV, HBV, ANA, ANCA, ENA, CRP, ESR, glycemia, HbA1c, copper, and cryoglobulins are all negative, with positivity for IgG lambda paraprotein. There is no family history of polyneuropathy.		● **Symptoms**: Paresthesia and hypoesthesia in the lower limbs without associated weakness or sensory ataxia<br>● **Clinical Examination**:<br>○ No tremor, neuropathic pain, foot deformities<br>○ No cranial nerve or autonomic involvement<br>● **Past Medical History**: IgG lambda monoclonal gammopathy<br>● **Nerve Conduction Studies**:<br>○ Axonal, length-dependent, sensory-motor polyneuropathy<br>○ Unilateral carpal tunnel syndrome<br>● **Screening Lab Tests**:<br>○ Negative: Vitamin B12, folate, vitamin B6, vitamin E, HCV, HBV, ANA, ANCA, ENA, CRP, ESR, glycemia, HbA1c, copper, cryoglobulins<br>○ Positive: IgG lambda paraprotein<br>● **Family History**: No family history of polyneuropathy<br>**Differential Diagnoses for Polyneuropathy**<br>1. **Monoclonal Gammopathy of Undetermined Significance (MGUS)-Related Neuropathy**<br>○ **Reasoning**:<br>■ Presence of IgG lambda monoclonal gammopathy is a significant finding.<br>■ MGUS can be associated with a slowly progressive, sensory-dominant, length-dependent polyneuropathy.<br>■ Lack of other systemic symptoms or significant abnormalities in other lab tests supports MGUS-related neuropathy.<br>■ Nerve conduction study findings of axonal polyneuropathy align with typical presentations in MGUS-associated cases.<br>2. **Chronic Inflammatory Demyelinating Polyneuropathy (CIDP)**<br>○ **Reasoning**:<br>■ Although CIDP is typically demyelinating, some cases present with mixed or predominantly axonal features.<br>■ Chronic, slowly progressive neuropathy can fit CIDP, though the absence of weakness is less typical.<br>■ The monoclonal gammopathy could be coincidental or secondary in the context of CIDP.<br>■ Nerve conduction studies would ideally show demyelination, but axonal involvement can also occur.<br>3. **Diabetic Polyneuropathy**<br>○ **Reasoning**:<br>■ Diabetic polyneuropathy is common, but the patient's glycemia and HbA1c are within normal ranges.<br>■ If diabetes were present or undiagnosed, it would be a strong candidate due to the typical length-dependent sensory features.<br>■ However, the absence of hyperglycemia or elevated HbA1c makes this less likely.<br>**Most Likely Diagnosis**<br>**Monoclonal Gammopathy of Undetermined Significance (MGUS)-Related Neuropathy**<br>● **Reasoning**:<br>○ The presence of IgG lambda paraprotein in the context of a sensory, length-dependent polyneuropathy with an axonal pattern is highly suggestive of MGUS-related neuropathy.<br>○ The absence of other significant abnormalities in extensive laboratory work-up further supports this diagnosis.<br>○ MGUS is known to be associated with such neuropathies, and given the patient's clinical presentation and history, it is the most probable diagnosis.<br>Thus, the most likely diagnosis for this patient is MGUS-related neuropathy. Further management should include monitoring the progression of the neuropathy and regular follow-up to assess for potential evolution into a more systemic plasma cell disorder.
46	A 71-year-old female patient presents with a 2-year history of sensory-motor, asymmetrical multineuropathy with relatively slow onset. The patient presents with weakness, numbness and tingling in a multifocal distribution in the four limbs, with hand tremor and mild sensory ataxia without pain. Clinical examination reveals absence of foot deformities, cranial nerve or autonomic involvement. Past medical history is positive for breast and kidney cancer, for which the patient underwent chemotherapy five years ago, and which were considered cured. Nerve conduction studies show a multifocal, demyelinating, sensory-motor neuropathy with motor nerve conduction blocks. Screening lab tests including vitamin B12, folate, vitamin B6, vitamin E, HCV, HBV, ANA, ANCA, ENA, CRP, ESR, glycemia, HbA1c, copper, cryoglobulins and serum electrophoresis are all negative. There is no family history of polyneuropathy.	Multifocal CIDP	**Step-by-Step Clinical Analysis**<br>**Patient Summary:**<br>● **Age/Gender:** 71-year-old female<br>● **Presenting Complaints:**<br>○ 2-year history of sensory-motor, asymmetrical multineuropathy<br>○ Weakness, numbness, and tingling in a multifocal distribution in the four limbs<br>○ Hand tremor<br>○ Mild sensory ataxia without pain<br>● **Clinical Examination:**<br>○ Absence of foot deformities<br>○ No cranial nerve involvement<br>○ No autonomic involvement<br>● **Past Medical History:**<br>○ Breast and kidney cancer, treated with chemotherapy five years ago and considered cured<br>● **Investigations:**<br>○ Nerve conduction studies show multifocal, demyelinating, sensory-motor neuropathy with motor nerve conduction blocks<br>○ Screening lab tests (vitamin B12, folate, vitamin B6, vitamin E, HCV, HBV, ANA, ANCA, ENA, CRP, ESR, glycemia, HbA1c, copper, cryoglobulins, serum electrophoresis) are all negative<br>● **Family History:** No family history of polyneuropathy<br>**Differential Diagnoses for Polyneuropathy**<br>1. **Chronic Inflammatory Demyelinating Polyneuropathy (CIDP)**<br>○ **Reasoning:** CIDP is a chronic immune-mediated neuropathy that presents with slowly progressive or relapsing-remitting sensory and motor symptoms. The patient's multifocal distribution, demyelinating features on nerve conduction studies, and motor conduction blocks are characteristic of CIDP. The absence of cranial nerve and autonomic involvement supports CIDP, as these features are not commonly seen in this condition.<br>2. **Paraneoplastic Neuropathy**<br>○ **Reasoning:** Given the patient's history of breast and kidney cancer, paraneoplastic neuropathy must be considered. Paraneoplastic syndromes can cause peripheral neuropathy through immune-mediated mechanisms. The slow onset and multifocal nature of the neuropathy align with paraneoplastic processes. However, the absence of other systemic symptoms and the negative screening tests may make this less likely than CIDP.

			3. **Multifocal Motor Neuropathy (MMN)**<br>○ **Reasoning:** MMN is characterized by asymmetric, predominantly motor neuropathy with conduction blocks. The patient's presentation with multifocal weakness, hand tremor, and motor nerve conduction blocks on NCS fit MMN. However, MMN typically does not have significant sensory involvement, which is noted in this patient, making it a less probable diagnosis compared to CIDP.<br>**Most Likely Diagnosis**<br>Given the clinical presentation, nerve conduction study findings, and exclusion of other common causes through extensive negative screening tests, **Chronic Inflammatory Demyelinating Polyneuropathy (CIDP)** is the most likely diagnosis. The multifocal, demyelinating, and sensory-motor features with motor conduction blocks are highly indicative of CIDP. The lack of autonomic, cranial nerve involvement, and negative screening for other systemic diseases or deficiencies further support this diagnosis.<br>The next steps would include considering a trial of immunomodulatory treatment (e.g., corticosteroids, intravenous immunoglobulin, or plasmapheresis) to assess response, which can help confirm the diagnosis of CIDP.
47	An 80-year-old male patient presents with a 4-year history of pure sensory, symmetrical, length dependent polyneuropathy with insidious onset. The patient presents with distal paresthesia, neuropathic pain and cramps in the lower limbs with no ataxia, tremor, foot deformities, cranial nerve or autonomic involvement. Past medical history shows diabetes, psoriatic arthritis, polymyalgia rheumatica and chemotherapy for lung cancer. Neuropathy symptoms started four years after chemotherapy. Nerve conduction studies show mixed sensory-motor, symmetric, axonal polyneuropathy. Screening lab tests including vitamin B12, folate, vitamin B6, vitamin E, HCV, ANA, ANCA, ENA, CRP, ESR, glycemia, copper, cryoglobulins and serum electrophoresis are all negative, with evidence of chronic HBV infection. HbA1c levels are elevated. There is no family history of polyneuropathy.	Diabetic neuropathy	**Summary of Clinical Information**<br>**Patient Profile:**<br>● **Age:** 80 years old<br>● **Gender:** Male<br>**Clinical Presentation:**<br>● **History Duration:** 4 years<br>● **Type of Neuropathy:** Pure sensory, symmetrical, length-dependent polyneuropathy<br>● **Symptoms:**<br>○ Distal paresthesia (tingling or prickling sensation)<br>○ Neuropathic pain and cramps in the lower limbs<br>● **Neurological Findings:**<br>○ No ataxia (loss of coordination)<br>○ No tremor<br>○ No foot deformities<br>○ No cranial nerve or autonomic involvement<br>**Past Medical History:**<br>● Diabetes<br>● Psoriatic arthritis<br>● Polymyalgia rheumatica<br>● Chemotherapy for lung cancer (neuropathy symptoms started four years post-chemotherapy)<br>**Investigations:**<br>● **Nerve Conduction Studies:** Mixed sensory-motor, symmetric, axonal polyneuropathy<br>● **Screening Laboratory Tests:**<br>○ Negative for vitamin B12, folate, vitamin B6, vitamin E, HCV, ANA, ANCA, ENA, CRP, ESR, glycemia, copper, cryoglobulins, and serum electrophoresis<br>○ Chronic HBV infection<br>○ Elevated HbA1c levels<br>**Family History:**<br>● No family history of polyneuropathy<br>**Differential Diagnoses for Polyneuropathy**<br>1. **Diabetic Neuropathy**<br>○ **Reasoning:**<br>■ The patient has a history of diabetes with elevated HbA1c levels, indicating poor glycemic control.<br>■ Diabetic neuropathy typically presents as a length-dependent, symmetrical, sensory polyneuropathy, which matches the patient's presentation.<br>■ The absence of autonomic involvement and the purely sensory nature align with common presentations of diabetic neuropathy in early stages.<br>■ The patient's long-term diabetes increases the likelihood of developing neuropathy due to chronic hyperglycemia-induced nerve damage.<br>2. **Chemotherapy-Induced Peripheral Neuropathy (CIPN)**<br>○ **Reasoning:**<br>■ The patient has a history of chemotherapy for lung cancer, and neuropathy symptoms began four years after chemotherapy.<br>■ Many chemotherapeutic agents, such as platinum-based drugs, taxanes, and vinca alkaloids, are known to cause axonal polyneuropathy.<br>■ CIPN often presents as a symmetrical, sensory, or sensorimotor polyneuropathy with length-dependent distribution, consistent with the patient's symptoms.<br>■ The chronicity and gradual onset of symptoms, even years after chemotherapy, can be seen in CIPN, as neurotoxic effects can be delayed or progressive.<br>3. **Chronic Hepatitis B Virus (HBV) Infection-Associated Neuropathy**<br>○ **Reasoning:**<br>■ The patient has chronic HBV infection, which can lead to peripheral neuropathy, although it is less common.<br>■ HBV-associated neuropathy is typically related to immune-mediated mechanisms or cryoglobulinemia; however, the patient tested negative for cryoglobulins.<br>■ The pattern of sensory, length-dependent neuropathy with mixed sensory-motor involvement could fit, although HBV-related neuropathy often has other systemic signs or laboratory abnormalities, which are absent in this case.<br>**Most Likely Diagnosis**<br>**Diabetic Neuropathy**<br>**Reasoning:**<br>● The patient's long-standing diabetes and elevated HbA1c levels strongly suggest diabetic neuropathy as the most likely cause.<br>● The pattern of pure sensory, symmetrical, length-dependent polyneuropathy is classic for diabetic neuropathy.<br>● The chronic nature of the condition fits the four-year history of progressive symptoms.<br>● While chemotherapy-induced neuropathy and HBV-associated neuropathy are plausible, the presence of diabetes with poor glycemic control makes diabetic neuropathy the most probable diagnosis given the clinical and investigative findings.

48	A 45-year-old female patient presents with a 1-year history of pure sensory, symmetrical, length dependent polyneuropathy with subacute onset. The patient presents with distal leg paresthesia and neuropathic pain, with no ataxia, tremor, foot deformities, cranial nerve or autonomic involvement. Clinical examination shows hyperalgesia at pinprick test with normal tactile sensation. Past medical history is positive for multiple myeloma, for which the patient has undergone chemotherapy. Neuropathy symptoms started after the first chemotherapy cycle and remained stable ever since. Nerve conduction studies show pure a sensory, symmetric, axonal polyneuropathy. Screening lab tests including vitamin B12, folate, vitamin B6, vitamin E, HBV, HCV, ANA, ANCA, ENA, CRP, ESR, glycemia, HbA1c, copper and cryoglobulins are all negative, with positivity for IgG lambda paraprotein. There is no family history of polyneuropathy.	Chemotherapy-induced neuropathy	**Clinical Summary**<br>● **Patient Information**: 45-year-old female<br>● **History**:<br>○ 1-year history of pure sensory, symmetrical, length-dependent polyneuropathy<br>○ Subacute onset<br>○ Symptoms: Distal leg paresthesia, neuropathic pain<br>○ No ataxia, tremor, foot deformities, cranial nerve or autonomic involvement<br>● **Clinical Examination**:<br>○ Hyperalgesia at pinprick test<br>○ Normal tactile sensation<br>● **Past Medical History**:<br>○ Multiple myeloma<br>○ Underwent chemotherapy<br>○ Neuropathy symptoms started after the first chemotherapy cycle and remained stable<br>● **Investigations**:<br>○ Nerve conduction studies: Pure sensory, symmetric, axonal polyneuropathy<br>○ Screening lab tests: Negative for vitamin B12, folate, vitamin B6, vitamin E, HBV, HCV, ANA, ANCA, ENA, CRP, ESR, glycemia, HbA1c, copper, and cryoglobulins<br>○ Positive for IgG lambda paraprotein<br>● **Family History**: No family history of polyneuropathy<br>**Differential Diagnoses for Polyneuropathy**<br>1. **Chemotherapy-induced Peripheral Neuropathy (CIPN)**<br>○ **Reasoning**:<br>■ The temporal relationship between the onset of neuropathy symptoms and the initiation of chemotherapy strongly suggests a causative link.<br>■ Chemotherapy, particularly drugs used in the treatment of multiple myeloma such as bortezomib, thalidomide, and platinum-based agents, is well-known to cause sensory neuropathy.<br>■ The stability of symptoms post-chemotherapy initiation is consistent with CIPN, which often does not progress once the offending agent is discontinued or completed.<br>■ Absence of other systemic or autoimmune markers further supports this diagnosis.<br>2. **Paraproteinemic Neuropathy**<br>○ **Reasoning**:<br>■ Presence of IgG lambda paraprotein indicates a possible monoclonal gammopathy, which is associated with multiple myeloma and can lead to neuropathy.<br>■ Paraproteinemic neuropathies are typically length-dependent and symmetric, fitting the patient's presentation.<br>■ Paraproteins can damage peripheral nerves through various mechanisms, including deposition and direct toxicity, leading to sensory neuropathy.<br>■ The lack of other laboratory abnormalities makes this a plausible diagnosis in conjunction with the known multiple myeloma.<br>3. **Vitamin B6 Toxicity**<br>○ **Reasoning**:<br>■ Although screening tests for vitamin B6 were negative, this remains a differential due to the classic presentation of sensory neuropathy.<br>■ Vitamin B6 toxicity is known to cause a length-dependent sensory neuropathy with symptoms such as distal paresthesia and neuropathic pain.<br>■ The history of chemotherapy could theoretically include supplementation practices, but this is less likely given the stability of the symptoms post-chemotherapy.<br>**Most Likely Diagnosis**<br>The most likely diagnosis for this patient is **Chemotherapy-induced Peripheral Neuropathy (CIPN)**. The onset of symptoms correlates closely with the initiation of chemotherapy, a common cause of pure sensory, symmetrical, length-dependent polyneuropathy in patients undergoing treatment for multiple myeloma. The stability of symptoms following the initial chemotherapy cycle further supports this diagnosis. The negative extensive laboratory workup for other common causes of polyneuropathy and the presence of IgG lambda paraprotein, although indicative of an underlying hematologic disorder, align more strongly with CIPN given the temporal relationship and typical clinical course observed in such cases.
49	A 79-year-old female patient presents with a 1-year history of pure sensory, symmetrical, length dependent polyneuropathy with insidious onset and progressive course. The patient presents with distal leg paresthesia, severe ataxia and postural tremor, which she reported to appear along with the other symptoms. Clinical examination does not show presence of foot deformities, cranial nerve or autonomic involvement. Past medical history is positive for diabetes. Nerve conduction studies show demyelinating, sensory-motor, symmetric polyneuropathy. Screening lab tests show the presence of IgM kappa monoclonal gammopathy, while other exams including vitamin B12, folate, vitamin B6, vitamin E, HCV, HBV, ANA, ANCA, ENA, CRP, ESR, glycemia, HbA1c, copper and cryoglobulins are all negative. There is no family history of polyneuropathy.	Anti-MAG polyneuropathy	**Summary of Pertinent Clinical Information**<br>● **Patient**: 79-year-old female<br>● **Symptoms**:<br>○ 1-year history of pure sensory, symmetrical, length-dependent polyneuropathy<br>○ Distal leg paresthesia<br>○ Severe ataxia<br>○ Postural tremor<br>● **Course**: Insidious onset and progressive<br>● **Clinical Examination**:<br>○ No foot deformities<br>○ No cranial nerve involvement<br>○ No autonomic involvement<br>● **Past Medical History**: Positive for diabetes<br>● **Nerve Conduction Studies**: Demyelinating, sensory-motor, symmetric polyneuropathy<br>● **Lab Tests**:<br>○ Presence of IgM kappa monoclonal gammopathy<br>○ Negative for vitamin B12, folate, vitamin B6, vitamin E, HCV, HBV, ANA, ANCA, ENA, CRP, ESR, glycemia, HbA1c, copper, and cryoglobulins<br>● **Family History**: No family history of polyneuropathy<br>**Differential Diagnoses**<br>1. **Monoclonal Gammopathy of Undetermined Significance (MGUS) Neuropathy**<br>○ **Reasoning**: The presence of IgM kappa monoclonal gammopathy suggests a possible link to neuropathy. MGUS is known to cause neuropathy, particularly in older adults. The demyelinating pattern on nerve conduction studies supports this diagnosis, as MGUS-associated neuropathy often presents with demyelination. The progressive and insidious onset aligns with the typical presentation of MGUS neuropathy.

			2. **Chronic Inflammatory Demyelinating Polyneuropathy (CIDP)**<br>○ **Reasoning**: CIDP can present similarly with a progressive and insidious onset of polyneuropathy, often with both sensory and motor involvement. The demyelinating pattern seen in nerve conduction studies is characteristic of CIDP. However, the presence of IgM kappa monoclonal gammopathy suggests a specific etiology beyond typical CIDP, but CIDP could still coexist or be triggered by the monoclonal gammopathy.<br>3. **Diabetic Neuropathy**<br>○ **Reasoning**: Although the patient has a history of diabetes, the negative glycemia and HbA1c reduce the likelihood of current poor glycemic control as the cause. Diabetic neuropathy typically presents with a length-dependent, symmetrical pattern and can include both sensory and motor involvement. However, diabetic neuropathy usually does not show a demyelinating pattern on nerve conduction studies, making this diagnosis less likely but still possible considering the patient's history.<br>**Most Likely Diagnosis**<br>**Monoclonal Gammopathy of Undetermined Significance (MGUS) Neuropathy** is the most likely diagnosis. The presence of IgM kappa monoclonal gammopathy strongly points toward MGUS-related neuropathy, especially given the demyelinating nature of the polyneuropathy observed in nerve conduction studies. This condition often presents with sensory symptoms and can progress insidiously, matching the clinical course of the patient's symptoms. Other differentials like CIDP and diabetic neuropathy are less likely due to the specificity of the monoclonal gammopathy finding and the typical presentation patterns.
50	A 57-year-old female patient presents with a five-year history of chronic, sensory-motor, symmetrical, length-dependent polyneuropathy with insidious onset and slow progression. The patient reports neuropathic pain in the feet, mild distal weakness in the lower limbs, and pes cavus, with no cranial nerve or autonomic involvement. Past medical history is unremarkable. Nerve conduction studies show a mixed axonal-demyelinating, sensory-motor polyneuropathy. Screening lab tests including vitamin B12, folate, vitamin B6, vitamin E, HBV, HCV, glycemia, HbA1c, ANA, ANCA, ENA, CRP, ESR, copper, cryoglobulins, and serum electrophoresis are all negative. Her mother has similar symptoms, while her brother has severe walking difficulties.	X-linked CMT	**Clinical Summary**<br>**Patient Profile:**<br>● **Age/Gender:** 57-year-old female<br>● **History Duration:** 5 years<br>● **Symptoms:** Chronic, sensory-motor, symmetrical, length-dependent polyneuropathy with:<br>○ Neuropathic pain in the feet<br>○ Mild distal weakness in lower limbs<br>○ Pes cavus<br>● **Onset/Progression:** Insidious onset, slow progression<br>● **Cranial/Autonomic Involvement:** None<br>**Medical History:**<br>● **Past Medical History:** Unremarkable<br>**Family History:**<br>● **Mother:** Similar symptoms<br>● **Brother:** Severe walking difficulties<br>**Nerve Conduction Studies:**<br>● Mixed axonal-demyelinating, sensory-motor polyneuropathy<br>**Laboratory Tests:**<br>● Negative for: Vitamin B12, folate, vitamin B6, vitamin E, HBV, HCV, glycemia, HbA1c, ANA, ANCA, ENA, CRP, ESR, copper, cryoglobulins, and serum electrophoresis<br>**Differential Diagnoses for Polyneuropathy**<br>**1. Hereditary Neuropathy with Liability to Pressure Palsies (HNPP)**<br>**Reasoning:**<br>● **Clinical Presentation:** The patient's chronic, length-dependent polyneuropathy with a slow progression is consistent with hereditary neuropathies. The presence of pes cavus and a positive family history (mother and brother) strongly suggests a genetic cause.<br>● **Nerve Conduction Studies:** Mixed axonal and demyelinating features are characteristic of hereditary neuropathies.<br>● **Family History:** Hereditary patterns, especially autosomal dominant, could explain similar symptoms in her mother and more severe symptoms in her brother.<br>● **Genetic Testing:** Confirmation would require genetic testing for PMP22 gene deletions or duplications.<br>**2. Charcot-Marie-Tooth Disease (CMT) Type 1**<br>**Reasoning:**<br>● **Clinical Presentation:** Charcot-Marie-Tooth disease, particularly type 1, typically presents with distal muscle weakness, sensory loss, and foot deformities like pes cavus.<br>● **Nerve Conduction Studies:** The mixed axonal-demyelinating pattern can be seen in CMT, though CMT1 often shows predominant demyelination.<br>● **Family History:** The hereditary aspect of CMT aligns with the family history provided. Autosomal dominant inheritance could explain the pattern observed in the family.<br>● **Genetic Testing:** Specific genetic mutations in genes such as PMP22, MPZ, and GJB1 (Cx32) would confirm the diagnosis.<br>**3. Hereditary Sensory and Motor Neuropathy (HSMN)**<br>**Reasoning:**<br>● **Clinical Presentation:** HSMN encompasses a group of genetic disorders, including CMT, characterized by sensory and motor neuropathy. The slow progression and symmetric nature fit this group.<br>● **Nerve Conduction Studies:** A mixed axonal-demyelinating pattern supports this diagnosis.<br>● **Family History:** A hereditary neuropathy like HSMN could explain the multi-generational presentation in the patient's family.<br>● **Genetic Testing:** Identification of specific mutations in related genes (e.g., MFN2, GDAP1) would support the diagnosis.<br>**Most Likely Diagnosis**<br>Given the clinical presentation, nerve conduction study results, and strong family history, **Charcot-Marie-Tooth Disease (CMT) Type 1** emerges as the most likely diagnosis. The presence of pes cavus, a hallmark of CMT, combined with the mixed axonal-demyelinating pattern on nerve conduction studies and the autosomal dominant inheritance pattern seen in the patient's family, strongly supports this diagnosis. Genetic testing targeting known CMT-related genes would provide definitive confirmation.
51	A 62-year-old male patient presents with an 8-month history of pure sensory, symmetrical, length dependent polyneuropathy with subacute onset. The patient presents with numbness and tingling and neuropathic pain distally in the lower limbs while he has no tremor, foot deformities, cranial nerve or autonomic involvement. Clinical examination reveals	CIDP	**Summary of Pertinent Clinical Information**<br>● **Patient Information:** 62-year-old male<br>● **History:** 8-month history of pure sensory, symmetrical, length-dependent polyneuropathy with subacute onset<br>● **Symptoms:** Numbness, tingling, neuropathic pain distally in the lower limbs<br>● **Examination Findings:** Sensory ataxia, no tremor, foot deformities, cranial nerve, or autonomic involvement<br>● **Past Medical History:** Silent<br>● **Nerve Conduction Studies:** Demyelinating, sensory-motor polyneuropathy; unilateral carpal tunnel syndrome<br>● **Screening Lab Tests:** Negative for vitamin B12, folate, vitamin B6, vitamin E, HCV, HBV, ANA, ANCA, ENA, CRP, ESR, glycemia, HbA1c, copper, and cryoglobulins<br>● **Serum Electrophoresis:** Presence of IgG paraprotein

	the presence of sensory ataxia. Past medical history is silent. Nerve conduction studies show a demyelinating, sensory-motor polyneuropathy and unilateral carpal tunnel syndrome. Screening lab tests including vitamin B12, folate, vitamin B6, vitamin E, HCV, HBV, ANA, ANCA, ENA, CRP, ESR, glycemia, HbA1c, copper, and cryoglobulins are all negative. Serum electrophoresis shows the presence of IgG paraprotein. There is no family history of polyneuropathy.		● **Family History:** No family history of polyneuropathy<br>**Differential Diagnoses for Polyneuropathy**<br>1. **Monoclonal Gammopathy of Undetermined Significance (MGUS) with IgG Paraprotein**<br>○ **Reasoning:**<br>■ The presence of IgG paraprotein in serum electrophoresis is significant.<br>■ MGUS is a common cause of polyneuropathy in older adults and often presents with demyelinating features on nerve conduction studies.<br>■ Sensory ataxia and pure sensory symptoms without autonomic involvement are typical in MGUS-related neuropathy.<br>■ Subacute onset and progressive nature align with MGUS-associated neuropathy.<br>2. **Chronic Inflammatory Demyelinating Polyneuropathy (CIDP)**<br>○ **Reasoning:**<br>■ CIDP can present with subacute onset and progressive course, similar to the patient's history.<br>■ Demyelinating sensory-motor polyneuropathy observed in nerve conduction studies is a hallmark of CIDP.<br>■ Sensory ataxia is consistent with CIDP.<br>■ However, CIDP often has motor involvement, which seems less pronounced in this case.<br>■ Lack of response to initial inflammatory markers (CRP, ESR) makes CIDP slightly less likely but still a consideration.<br>3. **Paraneoplastic Neuropathy**<br>○ **Reasoning:**<br>■ Paraneoplastic syndromes can cause polyneuropathy and are associated with various malignancies.<br>■ IgG paraprotein might indicate an underlying neoplastic process.<br>■ Subacute onset and demyelinating nature fit with paraneoplastic neuropathies.<br>■ Absence of other typical paraneoplastic markers and clinical features makes this less likely than MGUS and CIDP but should be considered.<br>**Most Likely Diagnosis**<br>Given the clinical presentation, nerve conduction study findings, and the presence of IgG paraprotein without other significant laboratory abnormalities or family history, **Monoclonal Gammopathy of Undetermined Significance (MGUS) with IgG Paraprotein** is the most likely diagnosis. This aligns well with the patient's age, subacute onset, demyelinating polyneuropathy, and sensory ataxia. MGUS is known to be associated with neuropathy, and the absence of other systemic involvement or inflammatory markers supports this diagnosis over CIDP or paraneoplastic neuropathy.
52	A 77-year-old male patient presents with a 6-month history of sensory-motor, symmetrical, non-length dependent polyneuropathy with subacute onset. The patient presents with weakness distributed proximally and distally in the lower limbs and distally in the upper limbs, and paresthesia in a stocking-glove distribution. Clinical examination reveals the presence of sensory ataxia, peripheral edema and hypertrichosis. There are no foot deformities, tremor, neuropathic pain, cranial nerve or autonomic involvement. Past medical history is positive for diabetes. Nerve conduction studies show mixed demyelinating-axonal, sensory-motor polyneuropathy. Screening lab tests including vitamin B12, folate, vitamin B6, vitamin E, HCV, HBV, ANA, ANCA, ENA, CRP, ESR, glycemia, copper and cryoglobulins are all negative, with positivity for IgM lambda paraprotein and raised HbA1c. There is no family history of polyneuropathy.	POEMS	**Clinical Information Summary**<br>● **Patient Profile:**<br>○ 77-year-old male<br>○ 6-month history of sensory-motor, symmetrical, non-length dependent polyneuropathy<br>○ Subacute onset<br>● **Symptoms:**<br>○ Weakness proximally and distally in lower limbs<br>○ Weakness distally in upper limbs<br>○ Paresthesia in stocking-glove distribution<br>○ Sensory ataxia<br>○ Peripheral edema<br>○ Hypertrichosis<br>● **Clinical Findings:**<br>○ No foot deformities<br>○ No tremor<br>○ No neuropathic pain<br>○ No cranial nerve involvement<br>○ No autonomic involvement<br>● **Medical History:**<br>○ Positive for diabetes<br>● **Nerve Conduction Studies:**<br>○ Mixed demyelinating-axonal, sensory-motor polyneuropathy<br>● **Laboratory Results:**<br>○ Negative for vitamin B12, folate, vitamin B6, vitamin E, HCV, HBV, ANA, ANCA, ENA, CRP, ESR, glycemia, copper, and cryoglobulins<br>○ Positive for IgM lambda paraprotein<br>○ Raised HbA1c<br>● **Family History:**<br>○ No family history of polyneuropathy<br>**Differential Diagnoses for Polyneuropathy**<br>1. **Diabetic Polyneuropathy:**<br>○ **Reasoning:**<br>■ Patient has a history of diabetes with raised HbA1c indicating poor glycemic control.<br>■ Diabetic polyneuropathy commonly presents with a stocking-glove pattern of paresthesia and mixed sensory-motor involvement.<br>■ However, the presence of proximal weakness, subacute onset, and non-length dependent features are atypical for classic diabetic neuropathy.<br>2. **POEMS Syndrome (Polyneuropathy, Organomegaly, Endocrinopathy, Monoclonal gammopathy, and Skin changes):**<br>○ **Reasoning:**<br>■ The presence of peripheral edema and hypertrichosis aligns with skin changes seen in POEMS syndrome.<br>■ IgM lambda paraprotein is consistent with a monoclonal gammopathy component of POEMS.

			■ Sensory-motor polyneuropathy with both demyelinating and axonal features is typical in POEMS syndrome.<br>■ Other POEMS features such as organomegaly and endocrine abnormalities should be further investigated.<br>3. **Chronic Inflammatory Demyelinating Polyneuropathy (CIDP) with Paraproteinemia:**<br>○ **Reasoning:**<br>■ CIDP can present with symmetrical, sensory-motor polyneuropathy and mixed demyelinating-axonal features.<br>■ Presence of paraproteinemia (IgM lambda) may suggest a paraproteinemic variant of CIDP.<br>■ The subacute onset and progression over 6 months with sensory ataxia are consistent with CIDP.<br>**Most Likely Diagnosis**<br>Considering the clinical presentation, examination findings, and the laboratory results, the most likely diagnosis is **POEMS Syndrome**.<br>● **Supporting Points:**<br>○ The combination of sensory-motor polyneuropathy, peripheral edema, hypertrichosis, and IgM lambda paraprotein strongly suggests POEMS syndrome.<br>○ The mixed demyelinating-axonal features on nerve conduction studies are typical of POEMS-associated neuropathy.<br>○ The atypical features for diabetic neuropathy and CIDP, such as the non-length dependent pattern and associated clinical signs (edema, hypertrichosis), further support POEMS syndrome.<br>**Conclusion**<br>The patient's presentation of a sensory-motor, symmetrical, non-length dependent polyneuropathy with associated clinical signs and the presence of IgM lambda paraprotein is most consistent with POEMS syndrome. Further investigations to confirm the diagnosis of POEMS syndrome may include a detailed evaluation for other components such as organomegaly, endocrine abnormalities, and additional monoclonal protein studies.
53	A 56-year-old male patient presents with a 13-month history of pure motor, asymmetric, non-length dependent polyneuropathy with insidious onset and progressive course. The patient presents with weakness in both hands with asymmetric distribution and reports having frequent cramps and fasciculations. Clinical examination reveals intrinsic hand atrophy. There are no sensory deficits or ataxia, tremor, cranial nerve or autonomic involvement. Past medical history is positive for systemic lupus erythematosus. Nerve conduction studies show a pure motor, demyelinating multineuropathy. Screening lab tests including vitamin B12, folate, vitamin B6, vitamin E, HCV, HBV, ANA, ANCA, ENA, CRP, ESR, glycemia, HbA1c, copper, cryoglobulins, and serum electrophoresis are negative. Cervical spine MRI is normal. There is no family history of polyneuropathy.	MMN	**Clinical Information Summary**<br>● **Patient Demographics**: 56-year-old male<br>● **Chief Complaint**: 13-month history of pure motor, asymmetric, non-length dependent polyneuropathy with insidious onset and progressive course<br>● **Symptoms**:<br>○ Weakness in both hands with asymmetric distribution<br>○ Frequent cramps and fasciculations<br>● **Clinical Findings**:<br>○ Intrinsic hand atrophy<br>○ No sensory deficits, ataxia, tremor, cranial nerve, or autonomic involvement<br>● **Past Medical History**: Systemic lupus erythematosus (SLE)<br>● **Investigations**:<br>○ Nerve conduction studies: Pure motor, demyelinating multineuropathy<br>○ Negative screening lab tests: Vitamin B12, folate, vitamin B6, vitamin E, HCV, HBV, ANA, ANCA, ENA, CRP, ESR, glycemia, HbA1c, copper, cryoglobulins, serum electrophoresis<br>○ Normal cervical spine MRI<br>● **Family History**: No family history of polyneuropathy<br>**Differential Diagnoses for Polyneuropathy**<br>**1. Multifocal Motor Neuropathy (MMN)**<br>**Reasoning**:<br>● **Clinical Presentation**: MMN typically presents with pure motor symptoms, asymmetry, and involves the upper limbs more than the lower limbs. The patient's symptoms of weakness in the hands, cramps, and fasciculations align with MMN.<br>● **Nerve Conduction Studies**: MMN is characterized by demyelinating features without sensory involvement, which matches the patient's study results.<br>● **Course**: The insidious and progressive nature of the disease is consistent with MMN.<br>● **Lack of Sensory Involvement**: The absence of sensory deficits is a hallmark of MMN.<br>**2. Amyotrophic Lateral Sclerosis (ALS)**<br>**Reasoning**:<br>● **Clinical Presentation**: ALS presents with asymmetric motor symptoms, including weakness, atrophy, fasciculations, and cramps, similar to the patient's symptoms.<br>● **Upper and Lower Motor Neuron Signs**: Although the patient mainly has lower motor neuron signs (atrophy, fasciculations), ALS could still be considered if early upper motor neuron signs were subtle or missed.<br>● **Progression**: ALS is progressive and typically involves both upper and lower motor neurons over time.<br>● **Exclusion of Sensory and Autonomic Involvement**: The absence of sensory and autonomic involvement fits ALS.<br>**3. Chronic Inflammatory Demyelinating Polyneuropathy (CIDP)**<br>**Reasoning**:<br>● **Clinical Presentation**: CIDP can present with motor-predominant symptoms and demyelinating features on nerve conduction studies. Although it typically has both sensory and motor involvement, pure motor variants exist.<br>● **Nerve Conduction Studies**: The demyelinating nature fits CIDP.<br>● **Progression**: The chronic and progressive course aligns with CIDP.<br>● **Autoimmune Association**: The patient's history of systemic lupus erythematosus, an autoimmune disease, makes an autoimmune etiology like CIDP plausible.<br>**Most Likely Diagnosis**<br>**Multifocal Motor Neuropathy (MMN)**<br>**Justification**:<br>● The patient's presentation of pure motor symptoms, including weakness, cramps, and fasciculations, along with the absence of sensory deficits, strongly points towards MMN.<br>● The demyelinating features on nerve conduction studies without sensory involvement are classic for MMN.<br>● ALS, although possible, typically presents with both upper and lower motor neuron signs, which are not prominent in this case.<br>● CIDP is less likely due to the absence of sensory involvement, although a motor-predominant variant is possible, it is less common.<br>Thus, Multifocal Motor Neuropathy is the most probable diagnosis given the clinical presentation and investigation findings.
54	A 74-year-old male patient presents with a 5-year history of sensory-motor, predominantly sensory, symmetrical, length dependent polyneuropathy with	Diabetic neuropathy	**Summary of Pertinent Clinical Information**<br>● **Patient Demographics:** 74-year-old male<br>● **Duration of Symptoms:** 5-year history<br>● **Symptoms:** Sensory-motor, predominantly sensory, symmetrical, length-dependent polyneuropathy with distal paresthesia and hypoesthesia; mild neuropathic pain in the feet.

	insidious onset and slow progressive course. The patient presents with distal paresthesia and hypoesthesia associated with mild neuropathic pain in the feet. At clinical examination there are no tremor, foot deformities, cranial nerve or autonomic involvement. Past medical history is positive for diabetes and cardiac insufficiency. Nerve conduction studies show a sensory-motor, symmetric, length dependent, axonal polyneuropathy. Screening lab tests show hyperglycemia and elevated HbA1c. Remaining exams including vitamin B12, folate, vitamin B6, vitamin E, HBV, HCV, ANA, ANCA, ENA, CRP, ESR, copper, cryoglobulins, and serum electrophoresis are negative. There is family history of polyneuropathy (father had similar symptoms).		● **Clinical Findings:** No tremor, foot deformities, cranial nerve involvement, or autonomic involvement.<br>● **Past Medical History:** Diabetes, cardiac insufficiency<br>● **Nerve Conduction Studies:** Sensory-motor, symmetric, length-dependent, axonal polyneuropathy<br>● **Lab Results:**<br>○ **Positive:** Hyperglycemia, elevated HbA1c<br>○ **Negative:** Vitamin B12, folate, vitamin B6, vitamin E, HBV, HCV, ANA, ANCA, ENA, CRP, ESR, copper, cryoglobulins, serum electrophoresis<br>● **Family History:** Father had similar symptoms<br>**Differential Diagnoses for Polyneuropathy**<br>1. **Diabetic Polyneuropathy**<br>○ **Reasoning:**<br>■ The patient has a history of diabetes, which is a well-known cause of polyneuropathy.<br>■ Elevated HbA1c and hyperglycemia support the diagnosis of poorly controlled diabetes.<br>■ Diabetic polyneuropathy typically presents with a length-dependent, sensory-motor neuropathy, which matches the patient's symptoms.<br>■ Slow, progressive course of symptoms over several years is consistent with diabetic neuropathy.<br>2. **Hereditary Sensory and Motor Neuropathy (HSMN)**<br>○ **Reasoning:**<br>■ Family history of polyneuropathy (father with similar symptoms) suggests a possible genetic component.<br>■ Insidious onset and slow progression are characteristic of hereditary neuropathies.<br>■ Sensory-motor involvement without cranial nerve or autonomic symptoms can be seen in HSMN.<br>■ Negative screening tests for other causes make hereditary neuropathy more likely.<br>3. **Chronic Inflammatory Demyelinating Polyneuropathy (CIDP)**<br>○ **Reasoning:**<br>■ Although CIDP often has an autoimmune basis, the absence of autoimmune markers does not rule it out completely.<br>■ CIDP can present with a slowly progressive course and sensory-motor involvement.<br>■ Typically, CIDP shows demyelinating features on nerve conduction studies, but chronic cases can exhibit axonal changes.<br>■ The negative inflammatory markers and normal serum electrophoresis make this diagnosis less likely, but it remains a consideration due to clinical overlap.<br>**Most Likely Diagnosis**<br>**Diabetic Polyneuropathy** is the most likely diagnosis for the following reasons:<br>● Strong correlation between the patient's history of diabetes and the presentation of a length-dependent, sensory-motor polyneuropathy.<br>● Hyperglycemia and elevated HbA1c levels strongly suggest poorly controlled diabetes as the underlying cause.<br>● The pattern of symptoms and nerve conduction study findings are consistent with diabetic neuropathy.<br>● While hereditary neuropathy is a consideration, the presence of diabetes as a clear risk factor and the typical presentation make diabetic polyneuropathy the most plausible diagnosis.
55	A 70-year-old male patient presents with a 2-year history of sensory-motor, symmetrical, length dependent polyneuropathy with subacute onset. He has been suffering from neuropathic pain in his feet for several years. However, over the past two years, he has also noticed progressive weakness in the feet and wasting of the intrinsic muscles of both hands. Additionally, he reports symptoms suggestive of orthostatic hypothension, and unintentional weight loss of approximately 5 kg over the past year. Clinical examination does not show presence of tremor, foot deformities, or cranial nerve involvement. Past medical history is positive for cardiac insufficiency, diabetes, and IgG lambda MGUS. Nerve conduction studies show a mixed demyelinating-axonal, sensory-motor, symmetric polyneuropathy. Screening lab tests reveal hyperglycemia, elevated HbA1c, presence of IgG lambda paraprotein, and increased creatinine levels. Remaining exams including vitamin B12, folate, vitamin B6, vitamin E, HBV, HCV, ANA, ANCA, ENA, CRP, ESR, copper, and cryoglobulins are normal. There is no family history of polyneuropathy.	Amyloidosis-associated polyneuropathy	**Clinical Information Summary**<br>● **Patient**: 70-year-old male<br>● **Symptoms**:<br>○ Sensory-motor, symmetrical, length-dependent polyneuropathy (2-year history)<br>○ Neuropathic pain in feet (several years)<br>○ Progressive weakness in feet (over past two years)<br>○ Wasting of intrinsic muscles of both hands<br>○ Symptoms of orthostatic hypotension<br>○ Unintentional weight loss (5 kg over the past year)<br>● **Clinical Examination**:<br>○ No tremor<br>○ No foot deformities<br>○ No cranial nerve involvement<br>● **Past Medical History**:<br>○ Cardiac insufficiency<br>○ Diabetes<br>○ IgG lambda MGUS (Monoclonal Gammopathy of Undetermined Significance)<br>● **Nerve Conduction Studies**:<br>○ Mixed demyelinating-axonal, sensory-motor, symmetric polyneuropathy<br>● **Lab Findings**:<br>○ Hyperglycemia<br>○ Elevated HbA1c<br>○ Presence of IgG lambda paraprotein<br>○ Increased creatinine levels<br>○ Normal levels of: vitamin B12, folate, vitamin B6, vitamin E, HBV, HCV, ANA, ANCA, ENA, CRP, ESR, copper, cryoglobulins<br>● **Family History**: No family history of polyneuropathy<br>**Differential Diagnoses for Polyneuropathy**<br>1. **Diabetic Neuropathy**<br>○ **Reasoning**:<br>■ The patient has a history of diabetes with hyperglycemia and elevated HbA1c, indicating poorly controlled diabetes, which is a common cause of sensory-motor polyneuropathy.<br>■ Diabetic neuropathy typically presents as a length-dependent polyneuropathy, similar to the patient's description.

			■ Neuropathic pain and progressive weakness are also consistent with diabetic neuropathy.<br>2. **Amyloid Light-Chain (AL) Amyloidosis**<br>○ **Reasoning**:<br>■ The presence of IgG lambda MGUS suggests a monoclonal gammopathy, which can be associated with AL amyloidosis.<br>■ Symptoms like orthostatic hypotension, weight loss, and mixed demyelinating-axonal polyneuropathy are suggestive of amyloidosis.<br>■ Cardiac insufficiency could be related to amyloid deposits in the heart, contributing to the patient's overall clinical picture.<br>■ Progressive muscle wasting in hands and autonomic involvement also align with amyloidosis manifestations.<br>3. **Chronic Inflammatory Demyelinating Polyneuropathy (CIDP)**<br>○ **Reasoning**:<br>■ CIDP often presents with progressive, symmetrical, sensory-motor polyneuropathy with a mix of demyelinating and axonal features on nerve conduction studies.<br>■ The subacute onset and progressive course over two years fit the CIDP profile.<br>■ However, the presence of diabetes and MGUS makes CIDP less likely compared to diabetic neuropathy and AL amyloidosis.<br>**Most Likely Diagnosis**<br>**Amyloid Light-Chain (AL) Amyloidosis** is the most likely diagnosis.<br>● **Supporting Points**:<br>○ The presence of IgG lambda paraprotein (MGUS) strongly suggests the possibility of amyloid deposits.<br>○ Symptoms like orthostatic hypotension, significant weight loss, and the mixed pattern of demyelination and axonal damage on nerve conduction studies are indicative of AL amyloidosis.<br>○ The combination of cardiac insufficiency, progressive muscle wasting, and autonomic symptoms further supports this diagnosis over diabetic neuropathy and CIDP.<br>Given the clinical presentation and the laboratory findings, AL amyloidosis should be thoroughly investigated, including further tests such as serum and urine immunofixation electrophoresis, free light chain assay, and possibly a biopsy to confirm amyloid deposits.
56	A 53-year-old female patient presents with a 42-year history of sensory-motor, symmetrical, length-dependent polyneuropathy with insidious onset and slowly progressive course. The patient reports distal leg paresthesia, scoliosis, sensory ataxia, distal weakness in the lower limbs, cramps, hammertoes, and pes cavus. Clinical examination does not reveal tremor, cranial nerve involvement, or autonomic dysfunction. Past medical history is unremarkable. Nerve conduction studies show a demyelinating, symmetric, sensory-motor polyneuropathy. Screening lab tests including vitamin B12, folate, vitamin B6, vitamin E, HBV, HCV, glycemia, HbA1c, ANA, ANCA, ENA, CRP, ESR, copper, cryoglobulins, and serum electrophoresis are all negative. Her mother, brother, and sister have walking difficulties.	CMT1	**Clinical Summary**<br>● **Patient Information**:<br>○ 53-year-old female.<br>○ 42-year history of sensory-motor, symmetrical, length-dependent polyneuropathy.<br>○ Insidious onset with a slowly progressive course.<br>● **Symptoms**:<br>○ Distal leg paresthesia.<br>○ Sensory ataxia.<br>○ Distal weakness in lower limbs.<br>○ Cramps.<br>○ Hammertoes.<br>○ Pes cavus.<br>○ Scoliosis.<br>● **Clinical Examination**:<br>○ No tremor.<br>○ No cranial nerve involvement.<br>○ No autonomic dysfunction.<br>● **Nerve Conduction Studies**:<br>○ Demyelinating, symmetric, sensory-motor polyneuropathy.<br>● **Laboratory Tests**:<br>○ Negative results for vitamin B12, folate, vitamin B6, vitamin E, HBV, HCV, glycemia, HbA1c, ANA, ANCA, ENA, CRP, ESR, copper, cryoglobulins, and serum electrophoresis.<br>● **Family History**:<br>○ Mother, brother, and sister have walking difficulties.<br>**Differential Diagnoses**<br>1. **Charcot-Marie-Tooth Disease (CMT)**<br>○ **Reasoning**:<br>■ CMT is a group of inherited disorders that cause nerve damage. The patient's slowly progressive, symmetrical, sensory-motor neuropathy with a demyelinating pattern on nerve conduction studies is characteristic of CMT.<br>■ The presence of pes cavus, hammertoes, and scoliosis are commonly associated with CMT.<br>■ The strong family history of walking difficulties supports a hereditary condition.<br>■ Lack of cranial nerve involvement and autonomic dysfunction is consistent with CMT.<br>2. **Hereditary Sensory and Autonomic Neuropathy (HSAN)**<br>○ **Reasoning**:<br>■ HSAN can present with sensory loss and motor symptoms, but typically also includes autonomic dysfunction, which the patient does not have.<br>■ The patient's clinical presentation of distal paresthesia, sensory ataxia, and lack of autonomic symptoms makes this diagnosis less likely.<br>■ However, the hereditary nature and long duration of symptoms do align with HSAN characteristics.<br>3. **Chronic Inflammatory Demyelinating Polyneuropathy (CIDP)**<br>○ **Reasoning**:<br>■ CIDP is an acquired immune-mediated inflammatory disorder of the peripheral nervous system, but typically does not present with such a long-standing history (42 years) without more significant progression or relapses.<br>■ CIDP can present with a demyelinating, symmetric, sensory-motor polyneuropathy, but usually with a more variable course and often responds to immunotherapy.<br>■ The absence of systemic inflammation markers (CRP, ESR) and negative autoimmune panels makes CIDP less likely, but it remains a consideration due to the demyelinating nature of the neuropathy.<br>**Most Likely Diagnosis**

			Given the chronic nature of the neuropathy, the family history of similar symptoms, and the characteristic clinical features such as pes cavus, hammertoes, and scoliosis, **Charcot-Marie-Tooth Disease (CMT)** is the most likely diagnosis. The patient's clinical presentation aligns well with this hereditary neuropathy, and the absence of systemic or autonomic involvement further supports this diagnosis.
57	A 65-year old male patient presents with a 3-year history of sensory, symmetrical, length dependent polyneuropathy with insidious onset. The patient presents with sensory ataxia, neuropathic pain, distal hypoesthesia up to the calves and mild dysarthria. Clinical examination does not reveal presence of foot deformities, tremor, or weakness. Past medical history shows chronic cough and mild cardiac insufficiency. Nerve conduction studies show a pure sensory, axonal polyneuropathy. Screening lab tests including vitamin B12, folate, vitamin B6, vitamin E, HBV, HCV, glycemia, HbA1c, ANA, ANCA, ENA, CRP, ESR, copper, cryoglobulins, and serum electrophoresis are all negative. There is no family history of polyneuropathy.	CANVAS	**Summary of Clinical Information:**<br>● **Patient Profile**: 65-year-old male<br>● **History**:<br>○ 3-year history of sensory, symmetrical, length-dependent polyneuropathy<br>○ Insidious onset<br>○ Symptoms: Sensory ataxia, neuropathic pain, distal hypoesthesia up to the calves, mild dysarthria<br>● **Clinical Examination**:<br>○ No foot deformities<br>○ No tremor<br>○ No weakness<br>● **Past Medical History**:<br>○ Chronic cough<br>○ Mild cardiac insufficiency<br>● **Diagnostic Tests**:<br>○ Nerve conduction studies: Pure sensory, axonal polyneuropathy<br>○ Screening lab tests: Negative for vitamin B12, folate, vitamin B6, vitamin E, HBV, HCV, glycemia, HbA1c, ANA, ANCA, ENA, CRP, ESR, copper, cryoglobulins, and serum electrophoresis<br>● **Family History**: No family history of polyneuropathy<br>**Differential Diagnoses for Polyneuropathy:**<br>1. **Idiopathic Sensory Axonal Neuropathy**<br>○ **Reasoning**: The patient has a long-standing history of sensory polyneuropathy with a pure sensory axonal pattern on nerve conduction studies. Given the extensive negative laboratory workup and the absence of a family history, idiopathic sensory axonal neuropathy remains a significant consideration. This diagnosis is often made when no specific cause can be identified despite comprehensive testing.<br>○ **Supporting Details**:<br>■ Insidious onset over several years<br>■ Pure sensory involvement without motor symptoms<br>■ Negative extensive laboratory workup<br>2. **Paraneoplastic Neuropathy**<br>○ **Reasoning**: The presence of chronic cough and mild cardiac insufficiency could be suggestive of an underlying malignancy, which sometimes presents with paraneoplastic syndromes including neuropathy. Although not definitive, the symptoms and the lack of other identifiable causes warrant consideration of this diagnosis.<br>○ **Supporting Details**:<br>■ Chronic cough could indicate an occult lung carcinoma, which can be associated with paraneoplastic syndromes<br>■ Lack of other causes despite thorough investigation<br>■ Mild dysarthria might be associated with central nervous system involvement often seen in paraneoplastic syndromes<br>3. **Toxic Neuropathy (Possible Heavy Metal Exposure)**<br>○ **Reasoning**: Although specific toxins were not identified in the initial screening, chronic exposure to heavy metals like lead, arsenic, or mercury can cause a pure sensory axonal neuropathy. A detailed occupational and environmental history could reveal potential exposures not initially considered.<br>○ **Supporting Details**:<br>■ Chronic nature and progression of symptoms<br>■ Pure sensory involvement consistent with certain toxic neuropathies<br>■ Need to rule out exposure to substances not covered by initial tests<br>**Most Likely Diagnosis:**<br>Based on the provided information and considering the negative extensive laboratory and family history, the most likely diagnosis for this patient is **Idiopathic Sensory Axonal Neuropathy**. This diagnosis is often reached by exclusion after a thorough workup fails to reveal a specific cause. The patient's clinical presentation, with pure sensory symptoms, insidious onset, and the negative results of a comprehensive battery of tests, supports this conclusion. Further monitoring and symptomatic management would be the primary approach, with continued vigilance for any signs that might suggest an alternative etiology in the future.
58	A 71-year-old female patient presents with a 2-year history of chronic, pure sensory, symmetrical, length dependent polyneuropathy with subacute onset. The patient presents with distal leg paresthesia and neuropathic pain associated with cramps. There are no foot deformities, weakness, ataxia, tremor, cranial nerve or autonomic involvement. Past medical history shows diabetes and lymphoma, for which the patient has undergone chemotherapy. Symptoms started during chemotherapy. Nerve conduction studies show mixed sensory-motor, axonal, symmetric polyneuropathy and unilateral carpal tunnel syndrome. Screening lab tests including vitamin B12, folate, vitamin B6, vitamin E, HBV, HCV, ANA, ANCA, ENA,	Chemotherapy-induced neuropathy	**Clinical Summary**<br>**Patient:** 71-year-old female<br>**Presentation:**<br>● **Symptoms:**<br>○ Chronic, pure sensory, symmetrical, length-dependent polyneuropathy<br>○ Distal leg paresthesia<br>○ Neuropathic pain associated with cramps<br>● **Onset:** Subacute, started during chemotherapy<br>● **Duration:** 2 years<br>**Physical Examination:**<br>● No foot deformities<br>● No weakness<br>● No ataxia<br>● No tremor<br>● No cranial nerve involvement<br>● No autonomic involvement<br>**Past Medical History:**<br>● Diabetes mellitus (elevated HbA1c)

	CRP, ESR, glycemia, copper, cryoglobulins and serum electrophoresis are all negative. HbA1c is elevated. There is no family history of polyneuropathy.		● Lymphoma (underwent chemotherapy)<br>**Nerve Conduction Studies:**<br>● Mixed sensory-motor, axonal, symmetric polyneuropathy<br>● Unilateral carpal tunnel syndrome<br>**Laboratory Tests:**<br>● Negative: Vitamin B12, folate, vitamin B6, vitamin E, HBV, HCV, ANA, ANCA, ENA, CRP, ESR, glycemia, copper, cryoglobulins, serum electrophoresis<br>**Family History:**<br>● No family history of polyneuropathy<br>**Differential Diagnoses for Polyneuropathy**<br>1. **Chemotherapy-Induced Peripheral Neuropathy (CIPN)**<br>○ **Reasoning:**<br>■ The onset of symptoms during chemotherapy strongly suggests a temporal relationship.<br>■ Chemotherapeutic agents, especially those used to treat lymphoma (e.g., platinum-based drugs, taxanes, vinca alkaloids), are known to cause sensory axonal neuropathy.<br>■ The patient's pure sensory symptoms, symmetrical pattern, and distal involvement are consistent with CIPN.<br>■ The absence of other significant findings (e.g., cranial nerve, autonomic involvement) fits the typical presentation of CIPN.<br>2. **Diabetic Peripheral Neuropathy (DPN)**<br>○ **Reasoning:**<br>■ The patient has a history of diabetes with elevated HbA1c, indicating poor glycemic control, a major risk factor for DPN.<br>■ DPN commonly presents as a length-dependent, symmetrical sensory neuropathy with neuropathic pain, which matches the patient's presentation.<br>■ While the timing of symptoms starting during chemotherapy may initially suggest CIPN, it's plausible that pre-existing subclinical DPN became symptomatic due to chemotherapy-induced stress on the nerves.<br>3. **Paraneoplastic Neuropathy**<br>○ **Reasoning:**<br>■ Lymphoma, along with other cancers, can be associated with paraneoplastic syndromes, including neuropathies.<br>■ Although less common, paraneoplastic neuropathy could present as a subacute, symmetrical, sensory-dominant polyneuropathy.<br>■ The presence of lymphoma and the temporal association with cancer treatment (though not exclusive to chemotherapy) support considering this diagnosis.<br>■ Negative autoantibody screens (e.g., ANA, ANCA) make this less likely but do not entirely rule it out as paraneoplastic neuropathies can sometimes be seronegative.<br>**Most Likely Diagnosis**<br>**Chemotherapy-Induced Peripheral Neuropathy (CIPN)** is the most likely diagnosis given the temporal onset of symptoms during chemotherapy, typical presentation of sensory polyneuropathy without motor, cranial nerve, or autonomic involvement, and the known neurotoxic effects of chemotherapeutic agents used in lymphoma treatment. Additionally, while diabetes and poor glycemic control could contribute, the clear correlation with chemotherapy initiation makes CIPN the leading diagnosis.<br>**Conclusion**<br>Given the patient's history, clinical presentation, and the temporal relationship with chemotherapy, CIPN is the most probable diagnosis. However, the contribution of diabetic neuropathy cannot be entirely excluded and may play a secondary role. Close monitoring and addressing both potential contributing factors (chemotherapy and diabetes) would be prudent in managing this patient's condition.
59	An 80-year-old male patient presents with a 1-year history of sensory-motor, symmetrical, length dependent polyneuropathy with subacute onset. The patient presents with paresthesia and sensory ataxia without tremor, neuropathic pain, foot deformities, cranial nerve or autonomic involvement. Clinical examination shows distal weakness and sensory deficits (hypoesthesia) in the lower limbs. Past medical history is positive for colorectal cancer and plasmacytoma, for which the patient has undergone chemotherapy. Neuropathy symptoms started a few weeks after the last chemotherapy treatment cycle. Nerve conduction studies show a sensory-motor, symmetric, length-dependent, axonal polyneuropathy. Screening lab tests including vitamin B12, folate, vitamin B6, vitamin E, HBV, HCV, ANA, ANCA, ENA, CRP, ESR, glycemia, HbA1c, copper and cryoglobulins are all negative, with positivity for IgG kappa paraprotein. There is no family history of polyneuropathy.	Chemotherapy-induced neuropathy	**Summary of Pertinent Clinical Information**<br>**Patient Profile:**<br>● **Age:** 80-year-old male<br>● **Presenting Symptoms:**<br>○ 1-year history of sensory-motor, symmetrical, length-dependent polyneuropathy with subacute onset<br>○ Paresthesia and sensory ataxia without tremor<br>○ Neuropathic pain, no foot deformities, cranial nerve or autonomic involvement<br>○ Distal weakness and sensory deficits (hypoesthesia) in the lower limbs<br>**Medical History:**<br>● Colorectal cancer<br>● Plasmacytoma<br>● Chemotherapy for colorectal cancer and plasmacytoma<br>**Clinical Findings:**<br>● Nerve conduction studies indicate sensory-motor, symmetric, length-dependent, axonal polyneuropathy<br>**Laboratory Findings:**<br>● Screening lab tests negative for:<br>○ Vitamin B12, folate, vitamin B6, vitamin E<br>○ HBV, HCV<br>○ ANA, ANCA, ENA<br>○ CRP, ESR<br>○ Glycemia, HbA1c<br>○ Copper<br>○ Cryoglobulins<br>● Positive for IgG kappa paraprotein<br>**Family History:**<br>● No family history of polyneuropathy<br>**Differential Diagnoses for Polyneuropathy**<br>1. **Chemotherapy-Induced Peripheral Neuropathy (CIPN)**<br>○ **Reasoning:**<br>■ The onset of symptoms is closely related to the timing of chemotherapy treatment.

			■ Chemotherapy, particularly agents like platinum compounds, taxanes, and vinca alkaloids, is known to cause axonal polyneuropathy.<br>■ Symptoms began a few weeks after the last chemotherapy cycle, fitting the typical timeline for CIPN.<br>2. **Paraproteinemic Neuropathy**<br>○ **Reasoning:**<br>■ The patient has a history of plasmacytoma, and IgG kappa paraproteinemia was detected.<br>■ Paraproteins can be associated with neuropathies due to their deposition in nerves or through autoimmune mechanisms.<br>■ Symmetrical sensory-motor neuropathy is a common presentation of paraproteinemic neuropathy.<br>3. **Paraneoplastic Neuropathy**<br>○ **Reasoning:**<br>■ The patient's history of colorectal cancer raises the possibility of paraneoplastic syndromes, which can include peripheral neuropathy.<br>■ Although less common, paraneoplastic neuropathy can present with subacute sensory-motor symptoms.<br>■ Absence of other more common causes (negative extensive lab tests) supports considering paraneoplastic causes.<br>**Most Likely Diagnosis**<br>**Chemotherapy-Induced Peripheral Neuropathy (CIPN)**<br>**Reasoning:**<br>● The timing of symptom onset following chemotherapy is a key indicator.<br>● Nerve conduction study findings of axonal polyneuropathy are consistent with CIPN.<br>● While the presence of IgG kappa paraprotein suggests the possibility of a paraproteinemic neuropathy, the temporal relationship with chemotherapy is stronger evidence for CIPN.<br>● Lack of family history and negative extensive screening tests further reduce the likelihood of other causes.<br>Considering the clinical context, chemotherapy-induced peripheral neuropathy stands out as the most likely diagnosis given the direct temporal correlation and typical presentation.
60	A 22-year-old male patient presents with a 20-year history of sensory-motor, symmetrical, length dependent polyneuropathy with insidious onset and slow progression. The patient is wheelchair bound and presents with sensory ataxia, severe distal weakness and atrophy in the four limbs, hypoesthesia up to calf and pes cavus, with no cranial nerve or autonomic involvement. Past medical history is unremarkable. Nerve conduction studies show a demyelinating, sensory-motor polyneuropathy. Screening lab tests including vitamin B12, folate, vitamin B6, vitamin E, HBV, HCV, glycemia, HbA1c, ANA, ANCA, ENA, CRP, ESR, copper, cryoglobulins, and serum electrophoresis are all negative. One of his five brothers has similar symptoms.	CMT4	**Step-by-Step Clinical Analysis**<br>**Summary of Pertinent Clinical Information:**<br>1. **Patient Profile:**<br>○ 22-year-old male<br>○ Wheelchair-bound<br>○ 20-year history of symptoms<br>2. **Symptoms:**<br>○ Sensory-motor, symmetrical, length-dependent polyneuropathy<br>○ Insidious onset, slow progression<br>○ Severe distal weakness and atrophy in all four limbs<br>○ Sensory ataxia<br>○ Hypoesthesia up to calf level<br>○ Pes cavus (high-arched feet)<br>3. **Neurological Findings:**<br>○ No cranial nerve involvement<br>○ No autonomic involvement<br>4. **Diagnostic Findings:**<br>○ Nerve conduction studies: demyelinating, sensory-motor polyneuropathy<br>5. **Laboratory Tests:**<br>○ Negative for vitamin B12, folate, vitamin B6, vitamin E, HBV, HCV, glycemia, HbA1c, ANA, ANCA, ENA, CRP, ESR, copper, cryoglobulins, and serum electrophoresis<br>6. **Family History:**<br>○ One of five brothers has similar symptoms<br>**Differential Diagnosis for Polyneuropathy:**<br>1. **Charcot-Marie-Tooth Disease (CMT):**<br>○ **Reasoning:**<br>■ CMT is a hereditary neuropathy that typically presents with distal muscle weakness, atrophy, sensory loss, and pes cavus.<br>■ The patient's symmetrical and length-dependent polyneuropathy, slow progression, and the presence of pes cavus are classic features.<br>■ The demyelinating type of CMT (CMT1) fits with the demyelinating findings on nerve conduction studies.<br>■ Family history of similar symptoms in a brother suggests a genetic component, which is consistent with CMT.<br>2. **Hereditary Sensory and Autonomic Neuropathy (HSAN):**<br>○ **Reasoning:**<br>■ HSAN can present with sensory and motor neuropathy, often with sensory ataxia.<br>■ However, HSAN typically involves autonomic symptoms, which are not present in this patient.<br>■ The lack of autonomic involvement makes HSAN less likely, but it cannot be completely ruled out.<br>3. **Refsum Disease:**<br>○ **Reasoning:**<br>■ Refsum disease is a hereditary metabolic disorder that can present with a peripheral neuropathy, sensory ataxia, and other systemic features like retinitis pigmentosa, which are not mentioned here.<br>■ The demyelinating polyneuropathy and pes cavus can be seen in Refsum disease.<br>■ Absence of systemic features like ichthyosis and retinitis pigmentosa reduces the likelihood of this diagnosis, but the sensory-motor polyneuropathy fits.<br>**Most Likely Diagnosis:**<br>Based on the provided clinical information, the most likely diagnosis is **Charcot-Marie-Tooth Disease (CMT)**. This diagnosis is supported by the following factors:<br>● The patient's clinical presentation with a long history of progressive, symmetrical, length-dependent polyneuropathy with severe distal weakness and atrophy, sensory ataxia, hypoesthesia, and pes cavus.<br>● The demyelinating nature of the neuropathy seen in nerve conduction studies.

			● The family history of a similar condition in one of his brothers, suggesting a hereditary component.<br>CMT is the most consistent with the overall clinical picture and family history. Further genetic testing would be required to confirm the diagnosis and identify the specific subtype of CMT.
61	A 71-year-old male patient presents with a 4-year history of pure sensory, symmetrical, length dependent polyneuropathy with insidious onset. The patient presents with neuropathic pain and paresthesia in the feet. There are no sensory ataxia or tremor, foot deformities, cranial nerve or autonomic involvement. Clinical examination shows the presence of tactile hypoesthesia and hyperalgesia in the feet. Past medical history is positive for psoriasis and type 2 diabetes. Nerve conduction studies show a pure sensory, symmetric, axonal, length dependent polyneuropathy and unilateral carpal tunnel syndrome. Screening lab tests show positivity for ANA antibodies, mildly raised CRP and ESR, increased levels of glycemia and HbA1c. Remaining exams, including vitamin B12, vitamin B6, vitamin E, HBV, HCV, ANCA, ENA, copper, cryoglobulins and serum electrophoresis are all negative. There is no family history of polyneuropathy.	Diabetic neuropathy	**Step-by-Step Clinical Analysis of the Case**<br>**Summarizing Pertinent Clinical Information**<br>● **Patient Profile:** 71-year-old male<br>● **History:**<br>○ 4-year history of pure sensory, symmetrical, length-dependent polyneuropathy<br>○ Neuropathic pain and paresthesia in the feet<br>○ No sensory ataxia, tremor, foot deformities, cranial nerve, or autonomic involvement<br>● **Clinical Examination:**<br>○ Tactile hypoesthesia and hyperalgesia in the feet<br>● **Past Medical History:**<br>○ Psoriasis<br>○ Type 2 diabetes<br>● **Nerve Conduction Studies:**<br>○ Pure sensory, symmetric, axonal, length-dependent polyneuropathy<br>○ Unilateral carpal tunnel syndrome<br>● **Lab Tests:**<br>○ Positive for ANA antibodies<br>○ Mildly raised CRP and ESR<br>○ Increased levels of glycemia and HbA1c<br>○ Negative for vitamin B12, vitamin B6, vitamin E, HBV, HCV, ANCA, ENA, copper, cryoglobulins, serum electrophoresis<br>● **Family History:** No family history of polyneuropathy<br>**Differential Diagnoses for Polyneuropathy**<br>**1. Diabetic Neuropathy**<br>**Reasoning:**<br>● **Correlation with Diabetes:** The patient has a history of type 2 diabetes, which is a well-known cause of polyneuropathy. Increased levels of glycemia and HbA1c indicate poor glycemic control, which can contribute to the development and progression of diabetic neuropathy.<br>● **Clinical Features:** The symmetrical, length-dependent pattern of sensory neuropathy with pain and paresthesia in the feet aligns with typical presentations of diabetic neuropathy.<br>● **Exclusion of Other Causes:** Negative results for other potential causes (e.g., vitamin deficiencies, infections, inflammatory markers) further support diabetic neuropathy.<br>**2. Chronic Inflammatory Demyelinating Polyneuropathy (CIDP)**<br>**Reasoning:**<br>● **Autoimmune Association:** The positive ANA antibodies and mildly raised CRP and ESR suggest an autoimmune component. CIDP is a chronic autoimmune condition affecting the peripheral nerves.<br>● **Clinical Presentation:** CIDP often presents with symmetrical, sensory, and motor involvement. Although the patient has a purely sensory presentation, atypical forms can exist.<br>● **Nerve Conduction Study Findings:** CIDP typically shows demyelinating features, but mixed or predominantly axonal forms can also be seen, making it a consideration despite the axonal findings.<br>**3. Small Fiber Neuropathy**<br>**Reasoning:**<br>● **Symptoms and Examination:** Neuropathic pain, paresthesia, tactile hypoesthesia, and hyperalgesia are consistent with small fiber neuropathy, which affects the small sensory fibers.<br>● **Association with Diabetes:** Small fiber neuropathy is commonly associated with diabetes and can present early in the disease course or with poor glycemic control.<br>● **Lab Findings:** The positive ANA antibodies and mild inflammatory markers could suggest an underlying autoimmune small fiber neuropathy, although this is less specific than in CIDP.<br>**Determining the Most Likely Diagnosis**<br>Considering the clinical presentation, history, and investigation results, the most likely diagnosis is **Diabetic Neuropathy**. This conclusion is based on:<br>● **Strong association with diabetes:** The patient's long history of type 2 diabetes and evidence of poor glycemic control strongly support diabetic neuropathy as the primary cause.<br>● **Symmetrical, length-dependent sensory involvement:** The clinical pattern is classic for diabetic neuropathy.<br>● **Exclusion of other potential causes:** Negative results for a broad range of other potential etiologies further narrow down diabetic neuropathy as the leading diagnosis.<br>While autoimmune elements and other forms of neuropathy cannot be completely ruled out, the alignment of symptoms, clinical findings, and the patient's medical history strongly favor diabetic neuropathy.
62	A 39-year-old male patient presents with a 1-month history of sensory-motor, symmetrical, non-length dependent polyneuropathy with acute onset. The patient presents with weakness, hypoesthesia, paresthesia and neuropathic pain in the four limbs, cramps, weakness of orbicularis oculi muscles, stypsis and urinary incontinence. Clinical examination reveals absence of tremor, ataxia or foot deformities. Past medical history is silent. Nerve conduction studies show a symmetric, demyelinating, sensory-motor polyneuropathy. Screening lab tests including vitamin B12, folate, vitamin B6, vitamin E, HCV, HBV, ANA, ANCA, ENA, CRP, ESR, glycemia, HbA1c, copper,	GBS	**Summary of Pertinent Clinical Information:**<br>● **Patient**: 39-year-old male<br>● **Duration**: 1-month history<br>● **Symptoms**:<br>○ Sensory-motor, symmetrical, non-length dependent polyneuropathy<br>○ Weakness, hypoesthesia (reduced sensation), paresthesia (tingling), and neuropathic pain in all four limbs<br>○ Muscle cramps<br>○ Weakness of orbicularis oculi muscles<br>○ Constipation (stypsis)<br>○ Urinary incontinence<br>● **Clinical Examination**:<br>○ Absence of tremor, ataxia, or foot deformities<br>● **Nerve Conduction Studies**: Symmetric, demyelinating, sensory-motor polyneuropathy<br>● **Lab Tests**: Negative for vitamin B12, folate, vitamin B6, vitamin E, HCV, HBV, ANA, ANCA, ENA, CRP, ESR, glycemia, HbA1c, copper, cryoglobulins, and serum electrophoresis<br>● **Family History**: No family history of polyneuropathy<br>**Differential Diagnoses for Polyneuropathy:**<br>1. **Guillain-Barré Syndrome (GBS) - Acute Inflammatory Demyelinating Polyneuropathy (AIDP)**

	cryoglobulins and serum electrophoresis are all negative. There is no family history of polyneuropathy.		○ **Reasoning**:<br>■ **Acute Onset**: GBS often presents with a rapid onset, progressing over days to weeks, similar to this case's 1-month history.<br>■ **Symmetrical Polyneuropathy**: GBS typically presents with symmetrical weakness and sensory symptoms.<br>■ **Demyelinating Pattern**: The nerve conduction studies show a demyelinating pattern, which is characteristic of GBS.<br>■ **Additional Symptoms**: Weakness of orbicularis oculi muscles, urinary incontinence, and constipation can occur in GBS due to autonomic involvement.<br>■ **Absence of Family History**: GBS is not inherited, aligning with the lack of family history.<br>2. **Chronic Inflammatory Demyelinating Polyneuropathy (CIDP)**<br>○ **Reasoning**:<br>■ **Similar Presentation**: CIDP can present similarly to GBS but usually progresses over a longer period. The 1-month duration is somewhat atypical but could represent an acute-on-chronic presentation.<br>■ **Symmetric, Demyelinating Polyneuropathy**: The demyelinating pattern in nerve conduction studies and the symmetric nature of the symptoms align with CIDP.<br>■ **Autonomic Symptoms**: CIDP can also present with autonomic symptoms like urinary incontinence and constipation.<br>■ **Negative Labs**: CIDP often has no specific lab abnormalities, consistent with the negative screening tests.<br>3. **Paraneoplastic Neuropathy**<br>○ **Reasoning**:<br>■ **Rapid Onset**: Paraneoplastic neuropathies can present acutely and resemble GBS or CIDP.<br>■ **Symmetrical Polyneuropathy**: These neuropathies often present with symmetrical sensory-motor involvement.<br>■ **Demyelinating Pattern**: Paraneoplastic syndromes can cause a demyelinating pattern on nerve conduction studies.<br>■ **Associated Symptoms**: Autonomic symptoms like urinary incontinence and constipation are possible.<br>■ **Screening for Malignancy**: The absence of specific screening tests for malignancy in the given labs suggests that further investigation for an underlying cancer might be necessary.<br>**Most Likely Diagnosis:**<br>Given the acute onset, symmetrical and sensory-motor involvement, demyelinating pattern on nerve conduction studies, and the presence of autonomic symptoms, **Guillain-Barré Syndrome (GBS)**, particularly the Acute Inflammatory Demyelinating Polyneuropathy (AIDP) variant, is the most likely diagnosis. The patient's presentation is highly consistent with GBS, especially given the rapid progression and the demyelinating features observed. CIDP is less likely due to its typically more chronic course, and paraneoplastic neuropathy is a consideration but less likely without clear evidence of an underlying malignancy or more specific symptoms pointing to a neoplastic process.
63	A 57-year-old female patient presents with a 1-month history of sensory-motor, symmetrical, length dependent polyneuropathy with subacute onset. The patient presents with severe sensory ataxia associated with hypoesthesia and paraesthesia in a stocking-glove distribution, as well as weakness of toe dorsiflexion. Clinical examination does not reveal cranial nerve or autonomic involvement, tremor, or foot deformities. Past medical history is positive for chronic autoimmune gastritis, chronic cough, type 2 diabetes, and arterial hypertension. Nerve conduction studies show presence of a sensory motor, length dependent, symmetric, axonal polyneuropathy. Screening lab tests reveal vitamin B12 deficiency, hyperglycaemia, elevated HbA1c, and positive ANA. Remaining exams including folate, vitamin B6, vitamin E, HBV, HCV, ANCA, ENA, CRP, ESR, copper, cryoglobulins, and serum electrophoresis are normal. There is no family history of polyneuropathy.	B12 deficiency neuropathy	**Summary of Pertinent Clinical Information**<br>**Patient Details:**<br>● **Age/Gender:** 57-year-old female<br>● **Symptoms:**<br>○ 1-month history of sensory-motor, symmetrical, length-dependent polyneuropathy<br>○ Subacute onset<br>○ Severe sensory ataxia<br>○ Hypoesthesia and paraesthesia in a stocking-glove distribution<br>○ Weakness of toe dorsiflexion<br>● **Clinical Findings:**<br>○ No cranial nerve involvement<br>○ No autonomic involvement<br>○ No tremor or foot deformities<br>● **Past Medical History:**<br>○ Chronic autoimmune gastritis<br>○ Chronic cough<br>○ Type 2 diabetes<br>○ Arterial hypertension<br>● **Nerve Conduction Studies:** Sensory-motor, length-dependent, symmetric, axonal polyneuropathy<br>● **Laboratory Findings:**<br>○ Vitamin B12 deficiency<br>○ Hyperglycaemia<br>○ Elevated HbA1c<br>○ Positive ANA<br>○ Normal folate, vitamin B6, vitamin E, HBV, HCV, ANCA, ENA, CRP, ESR, copper, cryoglobulins, and serum electrophoresis<br>● **Family History:** No family history of polyneuropathy<br>**Differential Diagnoses for Polyneuropathy**<br>**1. Diabetic Polyneuropathy**<br>**Reasoning:**<br>● **Hyperglycaemia and Elevated HbA1c:** The patient has a history of type 2 diabetes, and the presence of hyperglycaemia and elevated HbA1c suggests poor glycemic control, which is a significant risk factor for diabetic polyneuropathy.<br>● **Symmetrical, Length-Dependent Polyneuropathy:** Diabetic polyneuropathy typically presents as a symmetrical, length-dependent polyneuropathy, matching the patient's presentation.<br>● **Stocking-Glove Distribution:** The sensory symptoms in a stocking-glove distribution are characteristic of diabetic neuropathy.<br>● **Weakness:** Motor involvement, including weakness of toe dorsiflexion, can occur in diabetic polyneuropathy, especially in advanced cases.<br>**2. Vitamin B12 Deficiency Neuropathy**<br>**Reasoning:**<br>● **Vitamin B12 Deficiency:** The patient has documented vitamin B12 deficiency, which is a known cause of neuropathy.<br>● **Sensory Ataxia and Hypoesthesia:** Vitamin B12 deficiency often presents with sensory ataxia and hypoesthesia, aligning with the patient's symptoms.<br>● **Subacute Onset:** Neuropathy due to vitamin B12 deficiency can have a subacute onset, similar to the patient's timeline.<br>● **Autoimmune Gastritis:** The patient's history of chronic autoimmune gastritis could be contributing to malabsorption and subsequent vitamin B12 deficiency.<br>**3. Autoimmune-Related Polyneuropathy**

			**Reasoning:**<br>● **Positive ANA:** The presence of positive ANA indicates an autoimmune component, which can be associated with various autoimmune neuropathies.<br>● **Chronic Autoimmune Gastritis:** This suggests a predisposition to other autoimmune conditions that could involve the peripheral nervous system.<br>● **Sensory-Motor Polyneuropathy:** Autoimmune polyneuropathies can present with a mixed sensory-motor pattern and a length-dependent distribution.<br>**Most Likely Diagnosis**<br>Based on the presented clinical information, the most likely diagnosis is **Diabetic Polyneuropathy**. The reasoning is as follows:<br>● The patient has a clear history of type 2 diabetes with evidence of poor glycemic control (hyperglycaemia and elevated HbA1c).<br>● The clinical presentation of a symmetrical, length-dependent, sensory-motor polyneuropathy with stocking-glove distribution is classic for diabetic neuropathy.<br>● While vitamin B12 deficiency and autoimmune factors are also present and can contribute to neuropathy, the overall clinical picture is most consistent with diabetic polyneuropathy given the patient's diabetes and associated findings.<br>Further management should include optimization of glycemic control, vitamin B12 supplementation, and consideration of further evaluation for autoimmune conditions if symptoms persist or worsen despite addressing these primary factors.
64	A 74-year-old male patient presents with a 1-year history of sensory-motor, symmetrical, length dependent polyneuropathy with subacute onset and slow-progressive course. The patient presents with paresthesia and neuropathic pain distally in the lower limbs and sensory ataxia. Clinical examination reveals the presence of mild hand tremor and weakness of toe dorsiflexion. There are no foot deformities, cranial nerve or autonomic involvement. Past medical history is positive for cardiac insufficiency, diabetes and IgM monoclonal gammopathy. Nerve conduction studies show a demyelinating, sensory-motor, symmetric polyneuropathy. Screening lab tests reveal the presence of IgM paraprotein and mildly elevated HbA1c levels. Remaining lab tests including vitamin B12, folate, vitamin B6, vitamin E, HCV, HBV, ANA, ANCA, ENA, CRP, ESR, glycemia, copper, and cryoglobulins are negative. There is no family history of polyneuropathy.	Anti-MAG polyneuropathy	**Step-by-Step Analysis**<br>**Summary of Pertinent Clinical Information**<br>● **Patient Demographics**: 74-year-old male<br>● **Chief Complaint**: 1-year history of sensory-motor, symmetrical, length-dependent polyneuropathy<br>● **Onset and Course**: Subacute onset with a slow-progressive course<br>● **Symptoms**:<br>○ Paresthesia and neuropathic pain in the lower limbs<br>○ Sensory ataxia<br>● **Clinical Examination**:<br>○ Mild hand tremor<br>○ Weakness of toe dorsiflexion<br>○ No foot deformities, cranial nerve involvement, or autonomic involvement<br>● **Medical History**:<br>○ Cardiac insufficiency<br>○ Diabetes<br>○ IgM monoclonal gammopathy<br>● **Nerve Conduction Studies**: Demyelinating, sensory-motor, symmetric polyneuropathy<br>● **Laboratory Findings**:<br>○ Presence of IgM paraprotein<br>○ Mildly elevated HbA1c levels<br>○ Negative for vitamin B12, folate, vitamin B6, vitamin E, HCV, HBV, ANA, ANCA, ENA, CRP, ESR, glycemia, copper, and cryoglobulins<br>● **Family History**: No family history of polyneuropathy<br>**Differential Diagnoses for Polyneuropathy**<br>1. **Diabetic Polyneuropathy**<br>2. **Monoclonal Gammopathy of Undetermined Significance (MGUS)-related Polyneuropathy**<br>3. **Chronic Inflammatory Demyelinating Polyneuropathy (CIDP)**<br>**Detailed Reasoning**<br>1. **Diabetic Polyneuropathy**:<br>○ **Supporting Factors**: The patient has a history of diabetes with mildly elevated HbA1c levels, indicating poor glycemic control. Diabetic polyneuropathy is common in long-standing diabetes and presents with a similar clinical picture of symmetrical, distal, sensory-motor neuropathy.<br>○ **Counterpoints**: The demyelinating nature of the neuropathy seen on nerve conduction studies is more typical of CIDP or MGUS-related neuropathy rather than the axonal pattern usually seen in diabetic polyneuropathy.<br>2. **Monoclonal Gammopathy of Undetermined Significance (MGUS)-related Polyneuropathy**:<br>○ **Supporting Factors**: The presence of IgM monoclonal gammopathy and IgM paraprotein are strong indicators of MGUS-related neuropathy. This type of neuropathy can present with a demyelinating pattern on nerve conduction studies and can cause a chronic, slowly progressive, sensory-motor polyneuropathy.<br>○ **Clinical Features**: MGUS-related neuropathy often involves distal extremities and can be associated with sensory ataxia and neuropathic pain, aligning well with this patient's symptoms.<br>3. **Chronic Inflammatory Demyelinating Polyneuropathy (CIDP)**:<br>○ **Supporting Factors**: CIDP presents with a chronic, slowly progressive course of sensory-motor neuropathy with a demyelinating pattern on nerve conduction studies. The lack of autonomic involvement and the presence of a mild hand tremor can be seen in CIDP.<br>○ **Counterpoints**: CIDP typically requires the exclusion of other causes of demyelinating neuropathy, such as MGUS. The presence of IgM monoclonal gammopathy makes MGUS-related neuropathy a more likely primary diagnosis in this context.<br>**Most Likely Diagnosis**<br>**Monoclonal Gammopathy of Undetermined Significance (MGUS)-related Polyneuropathy**<br>● The combination of IgM monoclonal gammopathy, the demyelinating nature of the neuropathy, and the clinical presentation of sensory-motor polyneuropathy with sensory ataxia strongly suggest MGUS-related neuropathy as the primary diagnosis. While diabetic polyneuropathy is a consideration due to the patient's diabetes, the demyelinating pattern and the presence of monoclonal protein are more indicative of MGUS-related neuropathy. CIDP is also a possibility, but the monoclonal gammopathy points more convincingly towards MGUS-related neuropathy.
65	An 83-year-old female patient presents with a 6-week history of pure sensory, symmetrical, length dependent polyneuropathy with subacute onset. The patient presents with distal paresthesia, hypoesthesia, and neuropathic pain in the lower limbs with associated mild sensory ataxia. Clinical examination does not show	Vit B12 deficiency neuropathy	**Summary of Pertinent Clinical Information**<br>**Patient Profile:**<br>● **Age:** 83-year-old female<br>● **Presenting Complaint:** 6-week history of pure sensory, symmetrical, length-dependent polyneuropathy with subacute onset<br>**Symptoms:**<br>● Distal paresthesia, hypoesthesia, and neuropathic pain in the lower limbs<br>● Mild sensory ataxia<br>● Positive Romberg test (instability, inability to walk with closed eyes)

	the presence of weakness, tremor, foot deformities, cranial nerve or autonomic involvement. Romberg test resulted positive with inability to maintain postural station, feeling of instability, and inability to walk with closed eyes. Past medical history is positive for leukopenia, undifferentiated connective tissue disease, and type 2 diabetes. Nerve conduction studies show a pure sensory, symmetric, length dependent, axonal polyneuropathy. Screening lab tests show high glycemia and raised HbA1c, positivity for ANA antibodies, and evidence of vitamin B12 deficiency. Remaining exams including folate, vitamin B6, vitamin E, HCV, HBV, ANCA, CRP, ESR, copper, cryoglobulins, and serum electrophoresis are all negative. There is no family history of polyneuropathy.		**Clinical Findings:**<br>● No weakness, tremor, foot deformities, cranial nerve, or autonomic involvement<br>**Past Medical History:**<br>● Leukopenia<br>● Undifferentiated connective tissue disease<br>● Type 2 diabetes<br>**Investigations:**<br>● **Nerve Conduction Studies:** Pure sensory, symmetric, length-dependent, axonal polyneuropathy<br>● **Lab Tests:**<br>○ High glycemia and raised HbA1c<br>○ Positive ANA antibodies<br>○ Vitamin B12 deficiency<br>○ Negative for folate, vitamin B6, vitamin E, HCV, HBV, ANCA, CRP, ESR, copper, cryoglobulins, and serum electrophoresis<br>● **Family History:** No family history of polyneuropathy<br>**Differential Diagnoses for Polyneuropathy**<br>**1. Diabetic Polyneuropathy**<br>**Reasoning:**<br>● **Clinical Correlation:** The patient has a history of type 2 diabetes, which is a common cause of polyneuropathy. The raised HbA1c and high glycemia levels support poorly controlled diabetes.<br>● **Symptoms Alignment:** Diabetic polyneuropathy typically presents with distal sensory symptoms such as paresthesia, hypoesthesia, and neuropathic pain, aligning with the patient's presentation.<br>● **Pattern of Neuropathy:** The length-dependent, symmetrical, sensory axonal polyneuropathy seen in nerve conduction studies is characteristic of diabetic neuropathy.<br>**2. Vitamin B12 Deficiency Neuropathy**<br>**Reasoning:**<br>● **Clinical Correlation:** The patient has evidence of vitamin B12 deficiency, which is known to cause neuropathy.<br>● **Symptoms Alignment:** Vitamin B12 deficiency often presents with distal sensory symptoms and can include sensory ataxia and a positive Romberg test.<br>● **Pattern of Neuropathy:** The sensory nature of the neuropathy without motor involvement fits the profile of B12 deficiency neuropathy. Sensory ataxia due to posterior column involvement is also common in B12 deficiency.<br>**3. Connective Tissue Disease-Associated Neuropathy**<br>**Reasoning:**<br>● **Clinical Correlation:** The patient has a history of undifferentiated connective tissue disease and positive ANA antibodies, indicating an autoimmune component that can contribute to neuropathy.<br>● **Symptoms Alignment:** Autoimmune conditions can lead to polyneuropathy, often presenting with sensory symptoms.<br>● **Pattern of Neuropathy:** While less common than the other causes, connective tissue disease-associated neuropathy can present as a sensory neuropathy, potentially exacerbated by immune-mediated mechanisms.<br>**Most Likely Diagnosis**<br>**Diabetic Polyneuropathy** is the most likely diagnosis given the patient's history of type 2 diabetes, poorly controlled blood glucose levels, and the characteristic presentation of length-dependent, sensory axonal polyneuropathy. The presence of raised HbA1c and high glycemia supports this diagnosis.<br>**Conclusion**<br>While vitamin B12 deficiency and connective tissue disease-associated neuropathy are plausible, the overall clinical picture aligns most closely with diabetic polyneuropathy. Management should focus on optimizing blood glucose control and addressing the vitamin B12 deficiency to improve symptoms and prevent progression.
66	A 71-year-old female patient presents with a 4-month history of pure sensory, symmetrical, length dependent polyneuropathy with subacute onset. The patient presents with neuropathic pain and paresthesia in the feet. She does not report weakness, ataxia, tremor, cranial nerve or autonomic involvement. Clinical examination shows hyperalgesia and allodynia in the feet. There are no foot deformities, sensory ataxia or weakness. Past medical history is positive for cervical cancer, for which the patient started chemotherapy 6 months ago. Nerve conduction studies show a sensory-motor, symmetric, length dependent polyneuropathy. Screening lab tests show raised CRP and ESR, and positivity for ANA antibodies. Remaining exams including vitamin B12, folate, vitamin B6, vitamin E, HBV, HCV, ANCA, ENA, glycemia, HbA1c, copper, cryoglobulins, and serum electrophoresis are negative. There is no family history of polyneuropathy.	Chemotherapy-induced neuropathy	**Summary of Pertinent Clinical Information:**<br>● **Patient Demographics**: 71-year-old female<br>● **Chief Complaint**: 4-month history of pure sensory, symmetrical, length-dependent polyneuropathy with subacute onset<br>● **Symptoms**: Neuropathic pain and paresthesia in the feet, hyperalgesia, and allodynia in the feet<br>● **Physical Examination**: No weakness, ataxia, tremor, cranial nerve, or autonomic involvement; no foot deformities or sensory ataxia<br>● **Past Medical History**: Cervical cancer, started chemotherapy 6 months ago<br>● **Nerve Conduction Studies**: Sensory-motor, symmetric, length-dependent polyneuropathy<br>● **Lab Results**:<br>○ Raised CRP and ESR<br>○ Positive ANA antibodies<br>○ Negative for vitamin B12, folate, vitamin B6, vitamin E, HBV, HCV, ANCA, ENA, glycemia, HbA1c, copper, cryoglobulins, and serum electrophoresis<br>● **Family History**: No family history of polyneuropathy<br>**Differential Diagnoses for Polyneuropathy:**<br>1. **Chemotherapy-Induced Peripheral Neuropathy (CIPN)**:<br>○ **Reasoning**: The patient's neuropathy began shortly after the initiation of chemotherapy for cervical cancer. Chemotherapy is a well-known cause of peripheral neuropathy, particularly in older patients. The temporal relationship between the start of chemotherapy and the onset of symptoms strongly suggests a causative link.<br>○ **Supporting Evidence**: The subacute onset and pure sensory nature of the neuropathy, along with the hyperalgesia and allodynia, are consistent with CIPN. Raised inflammatory markers (CRP, ESR) and positive ANA might be incidental or related to the underlying malignancy and treatment, rather than the primary cause of neuropathy.<br>2. **Paraneoplastic Neuropathy**:<br>○ **Reasoning**: Paraneoplastic syndromes can occur in the context of cancer and might lead to various neuropathies. The presence of a malignancy (cervical cancer) increases the likelihood of a paraneoplastic process.<br>○ **Supporting Evidence**: The raised CRP and ESR, and positive ANA antibodies might suggest an immune-mediated mechanism, which is a characteristic of paraneoplastic syndromes. However, the pure sensory nature without other systemic signs makes this less likely compared to chemotherapy-induced neuropathy.<br>3. **Autoimmune-Related Neuropathy**:<br>○ **Reasoning**: The presence of positive ANA antibodies could indicate an underlying autoimmune process, such as systemic lupus erythematosus (SLE) or Sjögren's syndrome, which can cause neuropathy.<br>○ **Supporting Evidence**: Raised inflammatory markers (CRP, ESR) also support an inflammatory process. However, the lack of other systemic autoimmune symptoms (e.g., rash, joint pain) makes this less likely compared to the other differentials. Additionally, other specific autoimmune tests (ANCA, ENA) were negative.<br>**Most Likely Diagnosis:**

			Based on the clinical information, the most likely diagnosis is **Chemotherapy-Induced Peripheral Neuropathy (CIPN)**. The strong temporal association with chemotherapy initiation, the nature of the neuropathy (pure sensory, length-dependent), and the absence of other systemic autoimmune features or family history support this diagnosis. The raised CRP and ESR and positive ANA might be related to the underlying cancer or chemotherapy but are not specifically indicative of another primary cause.
67	An 83-year-old male patient presents with a 1-year history of sensory-motor, symmetrical, length dependent polyneuropathy with insidious onset and progressive course. The patient presents with distal leg paresthesia, hypoesthesia, and sensory ataxia. Clinical examination confirms the presence of sensory ataxia with positive Romberg test and inability to walk with closed eyes. There is also postural hand tremor, which appeared concurrently with the other symptoms. Clinical examination does not show the presence of foot deformities, cranial nerve or autonomic involvement. Past medical history is positive for prostatic cancer, for which the patient underwent chemotherapy four years ago. Nerve conduction studies show a demyelinating, sensory-motor, length dependent polyneuropathy. Screening lab tests reveal the presence of IgM paraprotein and positivity for ANA antibodies. Remaining exams including vitamin B12, folate, vitamin B6, vitamin E, HCV, HBV, ANCA, ENA, CRP, ESR, glycemia, HbA1c, copper ,and cryoglobulins are negative. There is no family history of polyneuropathy.	Anti-MAG polyneuropathy	**Summary of Pertinent Clinical Information**<br>**Patient Profile:**<br>● Age: 83 years old<br>● Sex: Male<br>● Past Medical History: Prostatic cancer treated with chemotherapy 4 years ago<br>**Presenting Symptoms (1-year history):**<br>● Sensory-motor, symmetrical, length-dependent polyneuropathy<br>● Distal leg paresthesia<br>● Hypoesthesia<br>● Sensory ataxia<br>● Positive Romberg test<br>● Inability to walk with closed eyes<br>● Postural hand tremor (concurrent with other symptoms)<br>● No foot deformities<br>● No cranial nerve involvement<br>● No autonomic involvement<br>**Clinical Findings:**<br>● Demyelinating, sensory-motor, length-dependent polyneuropathy (Nerve conduction studies)<br>● IgM paraprotein (Screening lab test)<br>● Positive ANA antibodies (Screening lab test)<br>● Negative for vitamin B12, folate, vitamin B6, vitamin E, HCV, HBV, ANCA, ENA, CRP, ESR, glycemia, HbA1c, copper, and cryoglobulins<br>● No family history of polyneuropathy<br>**Differential Diagnoses for Polyneuropathy**<br>1. **Chronic Inflammatory Demyelinating Polyneuropathy (CIDP)**<br>○ **Reasoning:**<br>■ The clinical presentation of a progressive, sensory-motor polyneuropathy with a demyelinating pattern on nerve conduction studies is characteristic of CIDP.<br>■ The presence of IgM paraprotein and positive ANA antibodies may suggest an underlying immune-mediated mechanism, which aligns with the pathophysiology of CIDP.<br>■ CIDP often presents with sensory ataxia, positive Romberg sign, and progressive weakness and sensory loss in a length-dependent fashion.<br>■ The patient's age and chronic course are also consistent with CIDP, which is more common in older adults.<br>2. **Paraneoplastic Neuropathy**<br>○ **Reasoning:**<br>■ The patient's history of prostatic cancer treated with chemotherapy raises the possibility of a paraneoplastic syndrome.<br>■ Paraneoplastic neuropathies can present with a similar pattern of sensory-motor involvement and are often associated with a progressive course.<br>■ The presence of IgM paraprotein could be related to a monoclonal gammopathy of undetermined significance (MGUS), which can be seen in paraneoplastic syndromes.<br>■ However, the absence of autonomic involvement and cranial nerve involvement makes a purely paraneoplastic syndrome less likely but still possible.<br>3. **Monoclonal Gammopathy of Undetermined Significance (MGUS) Neuropathy**<br>○ **Reasoning:**<br>■ MGUS can present with a chronic, progressive, sensory-motor neuropathy and is associated with the presence of IgM paraprotein.<br>■ The demyelinating nature of the neuropathy on nerve conduction studies is consistent with neuropathies seen in association with MGUS.<br>■ Positive ANA antibodies could be a coincidental finding but may also indicate an underlying autoimmune process that could contribute to the neuropathy.<br>■ The age of the patient and the chronicity of the symptoms are typical of MGUS-related neuropathy.<br>**Most Likely Diagnosis**<br>Based on the clinical presentation, nerve conduction study findings, and the presence of IgM paraprotein and ANA antibodies, **Chronic Inflammatory Demyelinating Polyneuropathy (CIDP)** is the most likely diagnosis. The demyelinating nature of the neuropathy, the chronic progressive course, and the sensory-motor involvement with sensory ataxia are all consistent with CIDP. Additionally, the presence of IgM paraprotein and positive ANA antibodies suggests an immune-mediated process, further supporting the diagnosis of CIDP.<br>While paraneoplastic neuropathy and MGUS-related neuropathy are also considerations, the overall clinical picture fits best with CIDP.
68	A 46-year-old male patient presents with a 3-year history of sensory-motor, symmetrical, non-length dependent polyneuropathy with insidious onset. The patient presents with leg paresthesia, sensory ataxia, proximal and distal weakness in the four limbs, and cramps. There are no cranial nerve or autonomic involvement, tremor, neuropathic pain or foot deformities. Past medical history shows IgG MGUS and chronic renal insufficiency. Nerve conduction studies show demyelinating, sensory-motor polyneuropathy in the four limbs.	CIDP	**Summary of Pertinent Clinical Information**<br>● **Patient:** 46-year-old male<br>● **History:**<br>○ 3-year history of sensory-motor, symmetrical, non-length dependent polyneuropathy<br>○ Insidious onset<br>○ Symptoms: leg paresthesia, sensory ataxia, proximal and distal weakness in all four limbs, and cramps<br>● **Absent Symptoms:** No cranial nerve involvement, autonomic involvement, tremor, neuropathic pain, or foot deformities<br>● **Past Medical History:**<br>○ IgG MGUS (Monoclonal Gammopathy of Undetermined Significance)<br>○ Chronic renal insufficiency<br>● **Nerve Conduction Studies:** Demyelinating, sensory-motor polyneuropathy in all four limbs<br>● **Lab Results:**<br>○ Negative for vitamin B12, folate, vitamin B6, vitamin E, HCV, HBV, ANA, ANCA, ENA, CRP, ESR, glycemia, HbA1c, copper, and cryoglobulins<br>○ Serum electrophoresis: Presence of IgG monoclonal gammopathy

	Screening lab tests including vitamin B12, folate, vitamin B6, vitamin E, HCV, HBV, ANA, ANCA, ENA, CRP, ESR, glycemia, HbA1c, copper and cryoglobulins are all negative. Serum electrophoresis confirms the presence of IgG monoclonal gammopathy. There is no family history of polyneuropathy.		● **Family History:** No family history of polyneuropathy<br>**Differential Diagnoses for Polyneuropathy**<br>1. **IgG Monoclonal Gammopathy of Undetermined Significance (MGUS) associated neuropathy:**<br>○ **Reasoning:** MGUS is commonly associated with a demyelinating polyneuropathy. The patient's history of IgG MGUS and the presence of a demyelinating pattern on nerve conduction studies support this diagnosis. MGUS can cause a chronic, progressive sensory-motor polyneuropathy, and the absence of other metabolic or infectious causes further supports this diagnosis.<br>○ **Features supporting:** Presence of IgG MGUS, demyelinating sensory-motor polyneuropathy, symmetrical involvement, absence of alternative causes on extensive lab testing.<br>2. **Chronic Inflammatory Demyelinating Polyneuropathy (CIDP):**<br>○ **Reasoning:** CIDP is a chronic, progressive demyelinating polyneuropathy that can present with both proximal and distal weakness, sensory deficits, and an insidious onset. The patient's clinical presentation, including symmetrical involvement, proximal and distal weakness, and sensory ataxia, is consistent with CIDP. While the patient's MGUS is notable, CIDP can occur independently or in association with paraproteinemias like MGUS.<br>○ **Features supporting:** Symmetrical sensory-motor involvement, demyelinating pattern on nerve conduction studies, chronic progressive course, and exclusion of other common causes.<br>3. **Uremic Neuropathy:**<br>○ **Reasoning:** Chronic renal insufficiency can lead to uremic neuropathy, characterized by a mixed sensory and motor polyneuropathy. However, uremic neuropathy typically presents with a length-dependent pattern, which is less consistent with this patient's non-length dependent presentation. Despite this, the chronic renal insufficiency and associated metabolic disturbances could contribute to neuropathic symptoms.<br>○ **Features supporting:** Chronic renal insufficiency, mixed sensory-motor involvement.<br>○ **Features against:** Non-length dependent nature of the neuropathy, demyelinating rather than axonal pattern on nerve conduction studies.<br>**Most Likely Diagnosis**<br>**IgG MGUS-associated neuropathy** is the most likely diagnosis for this patient. This conclusion is drawn from the presence of IgG MGUS, the demyelinating pattern of the polyneuropathy on nerve conduction studies, and the exclusion of other potential causes through comprehensive lab testing. MGUS-associated neuropathy is known to cause a chronic, progressive sensory-motor polyneuropathy that fits well with the patient's clinical presentation. CIDP remains a close differential but given the specific association with IgG MGUS, the most probable diagnosis is MGUS-associated neuropathy.
69	A 78-year-old female patient presents with a 2-year history of sensory-motor, symmetrical, length dependent polyneuropathy with subacute onset. Clinical examination reveals presence of numbness, tingling, and neuropathic pain distally in the upper and lower limbs, weakness of ankle dorsiflexion, sensory ataxia, hand tremor and dysphonia, without autonomic involvement or foot deformities. Past medical history reveals diabetes and IgM kappa monoclonal gammopathy. Nerve conduction studies show a length dependent, symmetric, sensory-motor, demyelinating polyneuropathy. Screening lab tests including vitamin B12, folate, vitamin B6, vitamin E, HCV, HBV, ANA, ANCA, ENA, CRP, ESR, glycemia, copper and cryoglobulins are all negative, with positivity for IgM kappa paraprotein and raised HbA1c. There is no family history of polyneuropathy.	Anti-MAG polyneuropathy	**Clinical Summary**<br>**Patient Details:**<br>● **Age:** 78-year-old female<br>● **Duration of Symptoms:** 2 years<br>● **Type of Neuropathy:** Sensory-motor, symmetrical, length-dependent polyneuropathy<br>● **Onset:** Subacute<br>**Clinical Examination Findings:**<br>● **Symptoms:**<br>○ Numbness<br>○ Tingling<br>○ Neuropathic pain distally in upper and lower limbs<br>● **Weakness:** Ankle dorsiflexion<br>● **Sensory Ataxia**<br>● **Hand Tremor**<br>● **Dysphonia**<br>● **No Autonomic Involvement**<br>● **No Foot Deformities**<br>**Past Medical History:**<br>● Diabetes<br>● IgM kappa monoclonal gammopathy<br>**Nerve Conduction Studies:**<br>● Length-dependent, symmetric, sensory-motor, demyelinating polyneuropathy<br>**Screening Lab Tests:**<br>● **Negative for:**<br>○ Vitamin B12, Folate, Vitamin B6, Vitamin E<br>○ Hepatitis C Virus (HCV)<br>○ Hepatitis B Virus (HBV)<br>○ Antinuclear Antibody (ANA)<br>○ Antineutrophil Cytoplasmic Antibody (ANCA)<br>○ Extractable Nuclear Antigen (ENA)<br>○ C-Reactive Protein (CRP)<br>○ Erythrocyte Sedimentation Rate (ESR)<br>○ Copper<br>○ Cryoglobulins<br>● **Positive for:**<br>○ IgM kappa paraprotein<br>○ Raised HbA1c<br>**Family History:**<br>● No family history of polyneuropathy<br>**Differential Diagnoses for Polyneuropathy**<br>1. **Diabetic Polyneuropathy**<br>○ **Reasoning:**<br>■ **Prevalence:** Diabetes is a common cause of polyneuropathy.<br>■ **Symptoms:** The patient has raised HbA1c, indicating poorly controlled diabetes, which can lead to sensory-motor neuropathy.

			■ **Pattern:** Length-dependent, symmetric polyneuropathy is typical in diabetes.<br>■ **Examination:** The combination of sensory symptoms, motor weakness, and pain fits diabetic neuropathy.<br>2. **Monoclonal Gammopathy of Undetermined Significance (MGUS)-Related Neuropathy**<br>○ **Reasoning:**<br>■ **Presence of Paraprotein:** The patient has IgM kappa monoclonal gammopathy.<br>■ **Symptoms:** MGUS can lead to a demyelinating polyneuropathy, often symmetric and length-dependent.<br>■ **Nerve Conduction Studies:** Demyelinating features align with MGUS-related neuropathy.<br>■ **Prevalence:** MGUS is common in older adults and can be associated with neuropathy.<br>3. **Chronic Inflammatory Demyelinating Polyradiculoneuropathy (CIDP)**<br>○ **Reasoning:**<br>■ **Symptoms and Course:** CIDP typically presents with progressive, symmetrical, sensory-motor neuropathy over months to years, fitting the subacute onset in this case.<br>■ **Nerve Conduction Studies:** The demyelinating pattern is consistent with CIDP.<br>■ **Exclusion of Other Causes:** Negative screening tests for other potential causes of demyelinating neuropathy support CIDP as a differential.<br>**Most Likely Diagnosis**<br>Based on the clinical presentation, nerve conduction studies, and laboratory findings, the most likely diagnosis is **Monoclonal Gammopathy of Undetermined Significance (MGUS)-Related Neuropathy**.<br>● The presence of IgM kappa monoclonal gammopathy is a significant finding that strongly suggests MGUS-related neuropathy.<br>● The demyelinating nature of the neuropathy on nerve conduction studies is more characteristic of MGUS-related neuropathy than diabetic polyneuropathy, which typically shows axonal features.<br>● While diabetic polyneuropathy is a strong contender due to the raised HbA1c, the specific demyelinating pattern and the presence of monoclonal gammopathy lean towards MGUS-related neuropathy.<br>Further evaluation by a hematologist to assess the extent and nature of the monoclonal gammopathy and possible treatment options for the neuropathy is recommended.
70	A 51-year-old female patient presents with a five-month history of sensory-motor, asymmetrical, non-length-dependent polyneuropathy with subacute onset and progressive course. The patient reports numbness and tingling in a stocking and glove distribution, dysphagia, asymmetric proximal and distal weakness in all four limbs, and mild sensory ataxia. Clinical examination confirms the presence of weakness and sensory deficits in all four limbs, with no evidence of autonomic involvement, tremor, or foot deformities. Past medical history is unremarkable. Nerve conduction studies show a demyelinating, non-length-dependent, sensory-motor polyneuropathy. Screening lab tests including vitamin B12, folate, vitamin B6, vitamin E, HBV, HCV, ANA, ANCA, ENA, CRP, ESR, glycemia, HbA1c, copper, cryoglobulins, and serum electrophoresis are all negative. There is no family history of polyneuropathy.	CIDP	**Clinical Scenario Summary**<br>A 52-year-old male presents to the outpatient clinic with symptoms of peripheral neuropathy. He reports a 6-month history of progressively worsening numbness, tingling, and burning sensations in his feet, which has recently started affecting his hands. He also notes muscle weakness, particularly in his lower extremities. His medical history includes Type 2 diabetes mellitus, hypertension, and hyperlipidemia. He is on metformin, lisinopril, and atorvastatin. He does not smoke and drinks alcohol occasionally. Physical examination reveals decreased sensation to light touch and pinprick in a stocking-glove distribution, diminished ankle reflexes, and mild weakness of the distal muscles in the lower extremities. Laboratory tests show elevated fasting blood glucose levels, HbA1c of 8.5%, normal renal function, and no evidence of vitamin deficiencies. Nerve conduction studies indicate a length-dependent axonal polyneuropathy.<br>**Differential Diagnoses**<br>1. **Diabetic Peripheral Neuropathy (DPN)**<br>○ **Reasoning**: The patient has a history of poorly controlled Type 2 diabetes mellitus, evidenced by elevated fasting blood glucose levels and an HbA1c of 8.5%. DPN is the most common cause of peripheral neuropathy in diabetic patients, characterized by a length-dependent axonal neuropathy. The symptoms of numbness, tingling, and burning sensations, along with decreased sensation in a stocking-glove distribution, are classic for DPN. Additionally, the presence of muscle weakness and diminished reflexes further supports this diagnosis.<br>2. **Chronic Inflammatory Demyelinating Polyneuropathy (CIDP)**<br>○ **Reasoning**: CIDP is an acquired immune-mediated disorder that presents with progressive, symmetric limb weakness, and sensory loss. Although it is less common than diabetic neuropathy, it should be considered, especially if there is a progressive course and significant motor involvement. However, the typical presentation includes both proximal and distal muscle weakness, which this patient lacks. Furthermore, nerve conduction studies usually show demyelinating features rather than axonal damage, which does not align with the patient's study results.<br>3. **Alcoholic Neuropathy**<br>○ **Reasoning**: The patient reports occasional alcohol consumption, but there is no indication of heavy or chronic alcohol use, which is typically required for the development of alcoholic neuropathy. This condition also presents with a similar pattern of distal sensory loss and motor weakness, but it usually requires a significant history of alcohol abuse. Given the patient's description of his alcohol use, this is less likely.<br>**Most Likely Diagnosis**<br>The most likely diagnosis for this patient is **Diabetic Peripheral Neuropathy (DPN)**. This conclusion is based on the patient's history of poorly controlled diabetes, the classic symptoms of a length-dependent axonal polyneuropathy, and the nerve conduction study findings. While other causes of neuropathy are possible, the patient's clinical presentation and history strongly support DPN as the primary diagnosis.
71	A 69-year-old male patient presents with a 7-month history of sensory-motor, asymmetrical, non-length dependent polyneuropathy with subacute onset and progressive course. The patient presents with paresthesia and neuropathic pain in the four limbs, weakness of ankle dorsiflexion, and mild urinary retention. Clinical examination does not show the presence of tremor, ataxia, foot deformities or cranial nerve involvement. Past medical history is positive for type 2 diabetes. Nerve conduction studies show a demyelinating, sensory-motor, symmetric, polyneuropathy. Screening lab tests including vitamin B12, folate, vitamin B6, vitamin E, HBV, HCV, ANA, ANCA, ENA, CRP, ESR, copper, cryoglobulins and serum electrophoresis are all negative, while glycemia and HbA1c levels are high. There is no family history of polyneuropathy.	CIDP	**Summary of Pertinent Clinical Information**<br>● **Patient Demographics**: 69-year-old male<br>● **Chief Complaints**:<br>○ Sensory-motor, asymmetrical, non-length dependent polyneuropathy<br>○ Paresthesia and neuropathic pain in all four limbs<br>○ Weakness of ankle dorsiflexion<br>○ Mild urinary retention<br>● **Onset and Course**:<br>○ Subacute onset, 7-month history, progressive course<br>● **Clinical Examination Findings**:<br>○ No tremor<br>○ No ataxia<br>○ No foot deformities<br>○ No cranial nerve involvement<br>● **Past Medical History**:<br>○ Type 2 diabetes<br>● **Nerve Conduction Studies**:<br>○ Demyelinating, sensory-motor, symmetric polyneuropathy<br>● **Laboratory Findings**:<br>○ Screening tests (vitamin B12, folate, vitamin B6, vitamin E, HBV, HCV, ANA, ANCA, ENA, CRP, ESR, copper, cryoglobulins, serum electrophoresis) are negative<br>○ High glycemia and HbA1c levels

			● **Family History**:<br>○ No family history of polyneuropathy<br>**Differential Diagnoses for Polyneuropathy**<br>**1. Diabetic Polyneuropathy**<br>**Reasoning**:<br>● **Association with Diabetes**: The patient has a known history of type 2 diabetes with high glycemia and HbA1c levels, indicating poorly controlled diabetes.<br>● **Common Presentation**: Diabetic polyneuropathy typically presents with a symmetric sensory-motor polyneuropathy, which can be either length-dependent or non-length dependent.<br>● **Nerve Conduction Studies**: Diabetic neuropathy often shows a demyelinating pattern, although it can also be axonal.<br>● **Symptomatic Overlap**: The patient's symptoms of paresthesia, neuropathic pain, and motor weakness are consistent with diabetic neuropathy.<br>● **Urinary Retention**: Autonomic involvement can occur in diabetic neuropathy, explaining the mild urinary retention.<br>**2. Chronic Inflammatory Demyelinating Polyneuropathy (CIDP)**<br>**Reasoning**:<br>● **Demyelinating Nature**: CIDP is characterized by a demyelinating polyneuropathy, which matches the nerve conduction study findings.<br>● **Clinical Course**: The subacute onset and progressive nature of the symptoms fit well with CIDP, which often progresses over months.<br>● **Symmetric Polyneuropathy**: CIDP typically presents with symmetric involvement, aligning with the patient's presentation.<br>● **Exclusion of Other Causes**: The negative results for various screening tests (vitamin deficiencies, infections, autoimmune markers) further support CIDP as other causes have been ruled out.<br>**3. Paraneoplastic Neuropathy**<br>**Reasoning**:<br>● **Age and Cancer Risk**: At 69 years old, the patient is at an increased risk for malignancies, which can be associated with paraneoplastic syndromes.<br>● **Subacute Onset and Progression**: Paraneoplastic neuropathies can present with a subacute onset and progressive course.<br>● **Demyelinating Pattern**: Paraneoplastic neuropathies can also show a demyelinating pattern on nerve conduction studies.<br>● **Symptomatology**: The presentation of sensory-motor polyneuropathy with autonomic involvement (urinary retention) can be seen in paraneoplastic syndromes.<br>**Most Likely Diagnosis**<br>Based on the provided clinical information, the most likely diagnosis is **Diabetic Polyneuropathy**. The patient's history of type 2 diabetes, poorly controlled blood glucose levels (evidenced by high glycemia and HbA1c), and the typical presentation of symmetric, sensory-motor polyneuropathy all strongly support this diagnosis. Additionally, the presence of mild urinary retention can be attributed to autonomic involvement in diabetic neuropathy. The findings from the nerve conduction studies and the exclusion of other causes through negative screening tests further consolidate diabetic polyneuropathy as the most probable diagnosis.
72	A 58-year old male patient presents with a 1-year history of sensory-motor, symmetrical, non-length dependent polyneuropathy with subacute onset. The patient presents with paresthesia and weakness in the distal areas of the four limbs. Clinical examination does not show the presence of sensory ataxia, neuropathic pain, tremor, foot deformities, cranial nerve or autonomic involvement. Clinical examination shows cutaneous hemangiomas and hyperpigmentation. Past medical history is positive for IgA lambda monoclonal gammopathy. Nerve conduction studies show mixed demyelinating-axonal, sensory-motor, symmetric polyneuropathy. Screening lab tests including vitamin B12, folate, vitamin B6, vitamin E, HCV, HBV, ANA, ANCA, ENA, CRP, ESR, glycemia, HbA1c, copper and cryoglobulins are all negative, with positivity for IgA lambda paraprotein. There is no family history of polyneuropathy.	POEMS	**Summary of Pertinent Clinical Information:**<br>● **Patient Profile:**<br>○ Age: 58-year-old male<br>○ Duration: 1-year history<br>○ Type: Sensory-motor, symmetrical, non-length dependent polyneuropathy<br>○ Onset: Subacute<br>● **Symptoms:**<br>○ Paresthesia and weakness in distal areas of all four limbs<br>○ No sensory ataxia<br>○ No neuropathic pain<br>○ No tremor<br>○ No foot deformities<br>○ No cranial nerve involvement<br>○ No autonomic involvement<br>● **Clinical Findings:**<br>○ Cutaneous hemangiomas<br>○ Hyperpigmentation<br>● **Past Medical History:**<br>○ IgA lambda monoclonal gammopathy<br>● **Nerve Conduction Studies:**<br>○ Mixed demyelinating-axonal, sensory-motor, symmetric polyneuropathy<br>● **Screening Lab Results:**<br>○ Negative: Vitamin B12, folate, vitamin B6, vitamin E, HCV, HBV, ANA, ANCA, ENA, CRP, ESR, glycemia, HbA1c, copper, cryoglobulins<br>○ Positive: IgA lambda paraprotein<br>● **Family History:**<br>○ No family history of polyneuropathy<br>**Differential Diagnoses for Polyneuropathy:**<br>1. **POEMS Syndrome (Polyneuropathy, Organomegaly, Endocrinopathy, Monoclonal Gammopathy, Skin changes)**<br>○ **Reasoning:**<br>■ The presence of IgA lambda monoclonal gammopathy strongly suggests a plasma cell disorder, which is a hallmark of POEMS syndrome.<br>■ The cutaneous hemangiomas and hyperpigmentation are characteristic skin changes seen in POEMS.<br>■ Mixed demyelinating and axonal neuropathy fits the typical neuropathic pattern observed in POEMS.<br>■ The absence of other systemic involvement (organomegaly, endocrinopathy) doesn't rule out POEMS as these may not be clinically apparent or fully developed yet.<br>2. **Chronic Inflammatory Demyelinating Polyneuropathy (CIDP) associated with Monoclonal Gammopathy**<br>○ **Reasoning:**<br>■ CIDP can present with a mixed demyelinating-axonal, sensory-motor polyneuropathy.<br>■ Monoclonal gammopathies, particularly IgA, can be associated with CIDP.

			■ The subacute onset and symmetrical distribution are consistent with CIDP.<br>■ The lack of systemic involvement (autonomic, cranial nerves) and sensory ataxia is also consistent with CIDP.<br>3. **Monoclonal Gammopathy of Undetermined Significance (MGUS) Neuropathy**<br>○ **Reasoning:**<br>■ The patient has an IgA lambda monoclonal gammopathy which can be associated with MGUS.<br>■ MGUS can lead to a neuropathy that is often symmetric and can have mixed sensory and motor involvement.<br>■ The absence of systemic symptoms and the chronic, subacute onset fit with MGUS neuropathy.<br>■ The negative screening for other causes and the specific nerve conduction study findings align with MGUS neuropathy.<br>**Most Likely Diagnosis:**<br>The most likely diagnosis is **POEMS Syndrome**. This conclusion is supported by the presence of monoclonal gammopathy (IgA lambda), cutaneous manifestations (hemangiomas and hyperpigmentation), and the characteristic nerve conduction study findings (mixed demyelinating-axonal neuropathy). While CIDP and MGUS neuropathy are also possibilities, the specific skin changes and the type of monoclonal gammopathy lean more towards POEMS syndrome. A thorough evaluation for other features of POEMS (such as organomegaly and endocrinopathy) would be warranted to confirm this diagnosis.
73	An 87-year-old male patient presents with a seven-month history of sensory-motor, symmetrical, length-dependent polyneuropathy with subacute onset and progressive course. The patient reports paresthesia and hypoesthesia in the lower limbs, associated with sensory ataxia and mild weakness in ankle dorsiflexion. Clinical examination does not reveal tremor, foot deformities, cranial nerve involvement, or autonomic dysfunction. Past medical history is positive for IgG kappa monoclonal gammopathy and prostate cancer. Nerve conduction studies show a demyelinating, sensory-motor, symmetric polyneuropathy. Screening lab tests including vitamin B12, folate, vitamin B6, vitamin E, HBV, HCV, ANA, ANCA, ENA, CRP, ESR, glycemia, HbA1c, copper, and cryoglobulins are all negative, with positivity for IgG kappa paraprotein. There is no family history of polyneuropathy.	CIDP	**Summary of Pertinent Clinical Information**<br>**Patient Profile:**<br>● **Age/Gender:** 87-year-old male<br>● **History:** Seven-month history of sensory-motor, symmetrical, length-dependent polyneuropathy<br>● **Onset:** Subacute onset with progressive course<br>● **Symptoms:** Paresthesia, hypoesthesia in lower limbs, sensory ataxia, mild weakness in ankle dorsiflexion<br>● **Examination:** No tremor, foot deformities, cranial nerve involvement, or autonomic dysfunction<br>**Past Medical History:**<br>● IgG kappa monoclonal gammopathy<br>● Prostate cancer<br>**Investigations:**<br>● **Nerve Conduction Studies:** Demyelinating, sensory-motor, symmetric polyneuropathy<br>● **Screening Lab Tests:** Negative for vitamin B12, folate, vitamin B6, vitamin E, HBV, HCV, ANA, ANCA, ENA, CRP, ESR, glycemia, HbA1c, copper, and cryoglobulins<br>● **Positive Lab Findings:** IgG kappa paraprotein<br>● **Family History:** No family history of polyneuropathy<br>**Differential Diagnoses for Polyneuropathy**<br>1. **Monoclonal Gammopathy of Undetermined Significance (MGUS) related Polyneuropathy**<br>○ **Reasoning:**<br>■ The patient has a documented IgG kappa monoclonal gammopathy.<br>■ MGUS is known to be associated with neuropathy, often presenting as a sensory-motor polyneuropathy.<br>■ The demyelinating pattern on nerve conduction studies is consistent with MGUS-related neuropathy.<br>■ The absence of other causes of neuropathy (negative extensive lab workup) strengthens this diagnosis.<br>■ Subacute onset and progression align with the typical course of MGUS-related neuropathy.<br>2. **Chronic Inflammatory Demyelinating Polyneuropathy (CIDP)**<br>○ **Reasoning:**<br>■ CIDP is a common cause of demyelinating, sensory-motor polyneuropathy.<br>■ The subacute onset and progressive nature of symptoms fit the presentation of CIDP.<br>■ Sensory ataxia and weakness in ankle dorsiflexion are consistent with CIDP.<br>■ CIDP can occur in association with monoclonal gammopathy, although this is less common.<br>■ The absence of cranial nerve involvement and autonomic dysfunction does not exclude CIDP but makes other causes more likely.<br>3. **Paraneoplastic Polyneuropathy**<br>○ **Reasoning:**<br>■ The patient has a history of prostate cancer, raising the possibility of a paraneoplastic syndrome.<br>■ Paraneoplastic neuropathies can present as a demyelinating, sensory-motor polyneuropathy.<br>■ However, paraneoplastic neuropathies often have a more rapid onset and may present with other neurological signs, which are not seen in this case.<br>■ The presence of a monoclonal protein (IgG kappa) can sometimes be associated with paraneoplastic syndromes, though this is less typical for prostate cancer.<br>**Most Likely Diagnosis**<br>The most likely diagnosis for this patient is **Monoclonal Gammopathy of Undetermined Significance (MGUS) related Polyneuropathy**. This conclusion is based on the presence of IgG kappa monoclonal gammopathy, the demyelinating pattern on nerve conduction studies, and the exclusion of other common causes of polyneuropathy through extensive lab testing. MGUS is known to cause a sensory-motor polyneuropathy, and the patient's clinical presentation aligns well with this condition.
74	A 51-year old female patient presents with a 35-year history of chronic, sensory-motor, symmetrical, length dependent polyneuropathy with insidious onset and slow progression. The patient presents with paresthesia, tremor, hypoesthesia up to the calves, bilateral steppage, and hammertoes. Clinical examination does not reveal cranial nerve or autonomic involvement. Past medical history shows	CMT2	**Clinical Summary**<br>● **Patient:** 51-year-old female<br>● **History:**<br>○ 35-year history of chronic, sensory-motor, symmetrical, length-dependent polyneuropathy<br>○ Symptoms: paresthesia, tremor, hypoesthesia up to the calves, bilateral steppage, and hammertoes<br>○ No cranial nerve or autonomic involvement<br>● **Past Medical History:**<br>○ Chronic renal insufficiency<br>● **Family History:**<br>○ Father and two uncles with walking difficulties

	chronic renal insufficiency. Nerve conduction studies show an axonal, symmetric, sensory-motor polyneuropathy. Screening lab tests including vitamin B12, folate, vitamin B6, vitamin E, HBV, HCV, glycemia, HbA1c, ANA, ANCA, ENA, CRP, ESR, copper, cryoglobulins, and serum electrophoresis are all negative. Her father and two uncles have walking difficulties.		● **Nerve Conduction Studies:**<br>○ Axonal, symmetric, sensory-motor polyneuropathy<br>● **Screening Lab Tests:**<br>○ All negative (vitamin B12, folate, vitamin B6, vitamin E, HBV, HCV, glycemia, HbA1c, ANA, ANCA, ENA, CRP, ESR, copper, cryoglobulins, and serum electrophoresis)<br>**Differential Diagnoses for Polyneuropathy**<br>**1. Hereditary Motor and Sensory Neuropathy (HMSN), also known as Charcot-Marie-Tooth (CMT) Disease**<br>● **Reasoning:**<br>○ **Chronic and progressive:** The 35-year history of slowly progressive neuropathy fits the typical presentation of hereditary neuropathies.<br>○ **Sensory-motor involvement:** Symmetrical sensory and motor symptoms are characteristic of CMT.<br>○ **Family history:** The presence of walking difficulties in her father and uncles strongly suggests a genetic component.<br>○ **Clinical features:** Hammertoes, bilateral steppage gait, and length-dependent hypoesthesia are typical manifestations.<br>○ **Nerve conduction studies:** Axonal neuropathy is consistent with certain types of CMT.<br>**2. Chronic Renal Failure-associated Polyneuropathy**<br>● **Reasoning:**<br>○ **Past medical history:** Chronic renal insufficiency is a known cause of polyneuropathy.<br>○ **Chronic course:** The slow progression of symptoms can be associated with chronic kidney disease.<br>○ **Sensory-motor neuropathy:** Renal failure can cause mixed sensory and motor neuropathies.<br>○ **Symmetric involvement:** Typically affects both sides of the body similarly, as described in this case.<br>○ **Exclusion of other causes:** Given the negative extensive laboratory workup, renal insufficiency remains a potential underlying condition.<br>**3. Idiopathic Polyneuropathy**<br>● **Reasoning:**<br>○ **No identifiable cause:** Despite an extensive workup, no specific cause has been found.<br>○ **Symmetric, sensory-motor neuropathy:** Fits the clinical and nerve conduction study findings.<br>○ **Chronic progression:** The insidious onset and long-term progression are consistent with idiopathic forms.<br>○ **Common in middle-aged and older adults:** Idiopathic neuropathies are not uncommon in this age group.<br>**Most Likely Diagnosis**<br>Based on the clinical presentation, family history, and negative extensive laboratory workup, **Hereditary Motor and Sensory Neuropathy (Charcot-Marie-Tooth Disease)** is the most likely diagnosis. The combination of a chronic, slowly progressive course, typical sensory-motor features, characteristic foot deformities, and a family history of similar symptoms strongly points towards a hereditary neuropathy. CMT encompasses various genetic subtypes, and the presence of axonal neuropathy on nerve conduction studies is consistent with certain types of CMT. Further genetic testing could confirm the specific subtype.
75	An 81-year-old male patient presents with a 13-month history of sensory-motor, symmetrical, non-length dependent polyneuropathy with subacute onset and progressive course. The patient presents with lumbar pain associated with paresthesia, neuropathic pain, and weakness that are distributed both proximally and distally in the four limbs. Clinical examination also reveals the presence of mild sensory ataxia. There are no tremor, cramps, foot deformities, cranial nerve or autonomic involvement. Past medical history is positive for type 2 diabetes and Hashimoto thyroiditis. Nerve conduction studies show a mixed demyelinating-axonal, sensory-motor polyneuropathy. Screening lab tests show the presence of IgG lambda monoclonal gammopathy and raised HbA1c levels. Remaining exams including vitamin B12, folate, vitamin B6, vitamin E, HCV, HBV, ANA, ANCA, ENA, CRP, ESR, copper and cryoglobulins are all negative. Lumbosacral spine x-ray shows the presence of a single osteosclerotic bone lesion. There is no family history of polyneuropathy.	POEMS	**Summary of Pertinent Clinical Information**<br>**Patient Information:**<br>● **Age:** 81 years<br>● **Sex:** Male<br>● **Duration of Symptoms:** 13 months<br>**Symptoms:**<br>● Sensory-motor, symmetrical, non-length dependent polyneuropathy<br>● Subacute onset with progressive course<br>● Lumbar pain<br>● Paresthesia<br>● Neuropathic pain<br>● Weakness (proximal and distal in all four limbs)<br>● Mild sensory ataxia<br>**Negative Findings:**<br>● No tremor<br>● No cramps<br>● No foot deformities<br>● No cranial nerve involvement<br>● No autonomic involvement<br>**Past Medical History:**<br>● Type 2 diabetes<br>● Hashimoto thyroiditis<br>**Diagnostic Findings:**<br>● **Nerve Conduction Studies:** Mixed demyelinating-axonal, sensory-motor polyneuropathy<br>● **Lab Results:**<br>○ IgG lambda monoclonal gammopathy present<br>○ Raised HbA1c levels<br>○ Negative for vitamin B12, folate, vitamin B6, vitamin E, HCV, HBV, ANA, ANCA, ENA, CRP, ESR, copper, and cryoglobulins<br>**Imaging:**<br>● Lumbosacral spine x-ray shows a single osteosclerotic bone lesion<br>**Family History:** No family history of polyneuropathy<br>**Differential Diagnoses for Polyneuropathy**<br>1. **Monoclonal Gammopathy of Undetermined Significance (MGUS) with Peripheral Neuropathy**

			○ **Reasoning:** The presence of IgG lambda monoclonal gammopathy suggests MGUS, which can be associated with a peripheral neuropathy, particularly with a mixed demyelinating and axonal pattern. MGUS-related neuropathy often presents with sensory-motor involvement and can be progressive. The osteosclerotic bone lesion could potentially indicate a plasma cell dyscrasia, although MGUS itself is not typically associated with osteosclerotic lesions.<br>2. **Diabetic Polyneuropathy**<br>○ **Reasoning:** The patient has a history of type 2 diabetes with raised HbA1c levels, indicating poorly controlled diabetes. Diabetic polyneuropathy is common and typically presents with a sensory-motor pattern. However, diabetic neuropathy usually presents as a length-dependent neuropathy, which is less consistent with the described non-length dependent distribution.<br>3. **POEMS Syndrome (Polyneuropathy, Organomegaly, Endocrinopathy, Monoclonal gammopathy, and Skin changes)**<br>○ **Reasoning:** POEMS syndrome should be considered due to the combination of polyneuropathy and monoclonal gammopathy. The presence of an osteosclerotic bone lesion is a key diagnostic feature of POEMS syndrome. Additionally, the patient's sensory ataxia and symmetrical nature of the polyneuropathy align with POEMS, even though other features like organomegaly and skin changes are not mentioned, they might not be overt or have been evaluated yet.<br>**Most Likely Diagnosis**<br>**POEMS Syndrome**<br>● **Detailed Reasoning:** POEMS syndrome is the most likely diagnosis considering the constellation of findings: sensory-motor polyneuropathy, presence of an IgG lambda monoclonal gammopathy, and a single osteosclerotic bone lesion. The neuropathy in POEMS is typically non-length dependent, as described in this patient, and is often associated with significant pain, which the patient has. Additionally, POEMS is often progressive and fits with the patient's clinical course. The absence of other typical features (organomegaly, endocrinopathy, skin changes) does not exclude the diagnosis but suggests that further evaluation might be necessary to identify these features. The patient's diabetes could contribute to his neuropathy but is less likely to be the primary cause given the non-length dependent nature and other associated findings.
76	A 51-year-old female patient presents with a 6-week history of sensory, symmetrical, length dependent polyneuropathy with acute-subacute onset. The patient presents with paresthesia and hypoesthesia with a stocking-glove distribution and sensory ataxia. Clinical examination reveals presence of severe ataxia with inability to stand without support. There is no weakness or neuropathic pain, tremor, foot deformities, cranial nerve or autonomic involvement. Past medical history is positive for type 1 diabetes since the age of 23. Nerve conduction studies show presence of a pure sensory, length dependent, axonal polyneuropathy. Screening lab tests reveal hyperglycemia, elevated HbA1c levels and severe copper deficiency. Remaining exams including vitamin B12, folate, vitamin B6, vitamin E, HBV, HCV, ANA, ANCA, ENA, CRP, ESR, cryoglobulins, and serum electrophoresis are all negative. There is no family history of polyneuropathy.	Copper deficiency neuropathy	**Step-by-Step Clinical Analysis of the Case**<br>**Patient Summary:**<br>● **Age:** 51 years old<br>● **Gender:** Female<br>● **Symptoms:**<br>○ Sensory, symmetrical, length-dependent polyneuropathy<br>○ Acute-subacute onset<br>○ Paresthesia and hypoesthesia with a stocking-glove distribution<br>○ Sensory ataxia<br>○ Severe ataxia with inability to stand without support<br>● **Clinical Examination:**<br>○ Severe ataxia<br>○ No weakness or neuropathic pain<br>○ No tremor, foot deformities<br>○ No cranial nerve or autonomic involvement<br>● **Medical History:**<br>○ Type 1 diabetes since age 23<br>● **Nerve Conduction Studies:**<br>○ Pure sensory, length-dependent, axonal polyneuropathy<br>● **Laboratory Findings:**<br>○ Hyperglycemia<br>○ Elevated HbA1c levels<br>○ Severe copper deficiency<br>○ Negative for vitamin B12, folate, vitamin B6, vitamin E, HBV, HCV, ANA, ANCA, ENA, CRP, ESR, cryoglobulins, serum electrophoresis<br>● **Family History:**<br>○ No family history of polyneuropathy<br>**Differential Diagnosis for Polyneuropathy**<br>**1. Diabetic Neuropathy**<br>● **Reasoning:**<br>○ **Medical History:** The patient has a long history of type 1 diabetes with poorly controlled hyperglycemia, as evidenced by elevated HbA1c levels.<br>○ **Clinical Features:** Diabetic neuropathy often presents as a length-dependent sensory polyneuropathy, similar to the patient's symptoms.<br>○ **Nerve Conduction Studies:** The findings of pure sensory, axonal polyneuropathy can be consistent with diabetic neuropathy.<br>○ **Typical Presentation:** Diabetic neuropathy commonly presents with sensory symptoms in a stocking-glove distribution and may include sensory ataxia.<br>● **Against:**<br>○ Diabetic neuropathy usually presents more gradually over years rather than acutely or subacutely over six weeks.<br>**2. Copper Deficiency Neuropathy**<br>● **Reasoning:**<br>○ **Laboratory Findings:** The patient has severe copper deficiency, which is a known cause of neuropathy.<br>○ **Clinical Features:** Copper deficiency can cause a sensory ataxic neuropathy due to the degeneration of the dorsal columns of the spinal cord and peripheral nerves.<br>○ **Acute-Subacute Onset:** Copper deficiency neuropathy can present with relatively rapid onset of symptoms, fitting the patient's timeline.<br>● **Against:**<br>○ Copper deficiency neuropathy is less common compared to other causes like diabetic neuropathy, and typically requires a significant deficiency, which appears to be the case here.<br>**3. Idiopathic Sensory Neuropathy**<br>● **Reasoning:**<br>○ **Clinical Features:** When no clear etiology is identified despite extensive workup, idiopathic sensory neuropathy is a diagnosis of exclusion.<br>○ **Nerve Conduction Studies:** A pure sensory, length-dependent, axonal polyneuropathy without clear cause can sometimes be classified as idiopathic.<br>● **Against:**

			○ Given the identified severe copper deficiency and history of diabetes, these should be thoroughly considered before concluding idiopathy.<br>**Most Likely Diagnosis**<br>**Copper Deficiency Neuropathy** is the most likely diagnosis considering the severe copper deficiency found in the patient. While diabetic neuropathy is also a strong contender due to the patient's long-standing type 1 diabetes, the acute-subacute onset of symptoms and specific laboratory finding of copper deficiency strongly suggest that copper deficiency is playing a significant role in the patient's neuropathy.<br>**Conclusion**<br>The patient's clinical presentation, nerve conduction study results, and laboratory findings strongly support the diagnosis of copper deficiency neuropathy as the primary cause of her symptoms. The acute-subacute onset, sensory ataxia, and the specific deficiency identified make this the most probable diagnosis. Diabetic neuropathy could also be contributing, given her history and poor glucose control, but the copper deficiency stands out as a more immediate and addressable cause.
77	A 70-year-old female patient presents with a 1-month history of sensory-motor, asymmetrical, non-length dependent, step-wise multineuropathy with acute onset. The patient presents with paresthesia, hypoesthesia, neuropathic pain and weakness in a multifocal distribution. Clinical examination does not reveal presence of ataxia, tremor, foot deformities, cranial nerve or autonomic involvement. Past medical history reveals the presence of intermittent fever and malaise. Nerve conduction studies show a mixed sensory-motor, axonal multineuropathy and unilateral carpal tunnel syndrome. Screening lab tests reveal high levels of CRP and ESR. Remaining lab exams including vitamin B12, folate, vitamin B6, vitamin E, HCV, HBV, ANA, ENA, glycemia, HbA1c, copper, cryoglobulins and serum electrophoresis are normal. There is no family history of polyneuropathy.	Vasculitic neuropathy	**Summary of Pertinent Clinical Information**<br>**Patient Demographics:**<br>● 70-year-old female<br>**Presenting Complaints:**<br>● 1-month history of sensory-motor, asymmetrical, non-length dependent, step-wise multineuropathy<br>● Acute onset<br>● Symptoms include paresthesia, hypoesthesia, neuropathic pain, and weakness in a multifocal distribution<br>**Clinical Examination Findings:**<br>● No ataxia<br>● No tremor<br>● No foot deformities<br>● No cranial nerve involvement<br>● No autonomic involvement<br>**Past Medical History:**<br>● Intermittent fever<br>● Malaise<br>**Nerve Conduction Studies:**<br>● Mixed sensory-motor, axonal multineuropathy<br>● Unilateral carpal tunnel syndrome<br>**Laboratory Findings:**<br>● High levels of CRP and ESR<br>● Normal levels of:<br>○ Vitamin B12<br>○ Folate<br>○ Vitamin B6<br>○ Vitamin E<br>○ HCV, HBV<br>○ ANA, ENA<br>○ Glycemia, HbA1c<br>○ Copper<br>○ Cryoglobulins<br>○ Serum electrophoresis<br>**Family History:**<br>● No family history of polyneuropathy<br>**Differential Diagnoses for Polyneuropathy**<br>1. **Vasculitic Neuropathy**<br>○ **Reasoning:**<br>■ The acute, step-wise progression of asymmetrical sensory-motor symptoms suggests a vasculitic process, which often presents with mononeuritis multiplex (a pattern of damage affecting multiple nerves in an asymmetrical fashion).<br>■ Elevated CRP and ESR are consistent with an inflammatory process, common in vasculitis.<br>■ The presence of intermittent fever and malaise supports a systemic inflammatory condition.<br>■ Normal levels of cryoglobulins, ANA, ENA do not rule out vasculitis, as some forms (e.g., non-cryoglobulinemic vasculitis) may present with normal serological markers.<br>2. **Paraneoplastic Neuropathy**<br>○ **Reasoning:**<br>■ Paraneoplastic syndromes can present with a variety of neuropathic patterns, including asymmetric, multifocal neuropathies.<br>■ The patient's age increases the likelihood of an underlying malignancy.<br>■ Symptoms like fever and malaise could be paraneoplastic in nature.<br>■ Elevated inflammatory markers (CRP and ESR) can be associated with paraneoplastic syndromes.<br>■ Absence of autonomic involvement does not rule out paraneoplastic etiology, as manifestations can vary.<br>3. **Infectious Neuropathy (e.g., Lyme Disease)**<br>○ **Reasoning:**<br>■ Although uncommon, some infectious agents can cause asymmetrical, multifocal neuropathy.<br>■ The patient's intermittent fever and elevated inflammatory markers raise the possibility of an ongoing infection.<br>■ Lyme disease can present with a variety of neurological symptoms, including multineuropathy, especially in endemic areas.<br>■ The absence of HCV, HBV does not exclude other infectious causes that may not have been tested for.<br>**Most Likely Diagnosis**

			The most likely diagnosis for this patient, given the acute onset, step-wise progression, asymmetrical distribution, and systemic inflammatory signs (fever, malaise, elevated CRP and ESR), is **vasculitic neuropathy**. This diagnosis is supported by the pattern of nerve involvement, the inflammatory markers, and the absence of other common causes of neuropathy such as diabetes or nutritional deficiencies. Additionally, vasculitic neuropathy often presents with mononeuritis multiplex, which fits the described nerve conduction study findings. Further investigations, such as nerve biopsy or additional serological tests specific for vasculitis (e.g., ANCA), may be warranted to confirm this diagnosis.
78	A 48-year-old male patient presents with a 4-month history of sensory-motor, asymmetric, length dependent polyneuropathy with subacute onset and rapidly progressive course. The patient presents with paresthesia, hypoesthesia, and weakness in the distal segments of the four limbs in an asymmetric distribution. Clinical evaluation reveals peripheral edema in the lower limbs. The patient reports recent appearance of several skin growths that were diagnosed as cutaneous angiomas by the dermatologist. He also reports losing approximately 10 kg over the past 12 months. There are no tremor, ataxia, neuropathic pain, foot deformities, cranial nerve or autonomic involvement. Past medical history is positive for fibromyalgia and liver steatosis. Nerve conduction studies show a mixed demyelinating-axonal, sensory-motor, symmetric polyneuropathy. Screening lab tests including folate, vitamin B6, vitamin E, HCV, HBV, ANA, ANCA, ENA, CRP, ESR, glycemia, HbA1c, copper and cryoglobulins are negative. Serum electrophoresis shows positivity for IgA lambda paraprotein, and there is evidence of vitamin B12 deficiency. There is no family history of polyneuropathy.	POEMS	**Summary of Clinical Information**<br>**Patient Presentation:**<br>● **History:** 4-month history of sensory-motor, asymmetric, length-dependent polyneuropathy with subacute onset and rapid progression.<br>● **Symptoms:** Paresthesia, hypoesthesia, and weakness in the distal segments of the four limbs in an asymmetric distribution.<br>● **Physical Findings:** Peripheral edema in the lower limbs.<br>● **Skin:** Recent appearance of several skin growths diagnosed as cutaneous angiomas.<br>● **Weight Loss:** Approximately 10 kg lost over the past 12 months.<br>● **No:** Tremor, ataxia, neuropathic pain, foot deformities, cranial nerve, or autonomic involvement.<br>● **Past Medical History:** Fibromyalgia and liver steatosis.<br>● **Nerve Conduction Studies:** Mixed demyelinating-axonal, sensory-motor, symmetric polyneuropathy.<br>● **Screening Lab Tests:** Negative for folate, vitamin B6, vitamin E, HCV, HBV, ANA, ANCA, ENA, CRP, ESR, glycemia, HbA1c, copper, and cryoglobulins.<br>● **Serum Electrophoresis:** Positive for IgA lambda paraprotein.<br>● **Vitamin Deficiency:** Evidence of vitamin B12 deficiency.<br>● **Family History:** No family history of polyneuropathy.<br>**Differential Diagnoses for Polyneuropathy**<br>1. **Paraneoplastic Polyneuropathy:**<br>○ **Reasoning:** The rapid progression and asymmetric nature of the neuropathy, significant weight loss, and presence of cutaneous angiomas suggest a paraneoplastic syndrome. Paraneoplastic polyneuropathy can present as a mixed demyelinating-axonal neuropathy, and the presence of a monoclonal gammopathy (IgA lambda paraprotein) strengthens this suspicion. Additionally, paraneoplastic syndromes can be associated with certain cancers that might explain the systemic signs and symptoms.<br>2. **Monoclonal Gammopathy of Undetermined Significance (MGUS):**<br>○ **Reasoning:** The presence of IgA lambda paraprotein in the serum electrophoresis is characteristic of MGUS, which can be associated with peripheral neuropathy. MGUS-related neuropathy typically presents as a mixed demyelinating-axonal neuropathy. However, the rapid progression and weight loss are less typical for MGUS and more suggestive of a malignant transformation, possibly to multiple myeloma or a related disorder.<br>3. **Vitamin B12 Deficiency:**<br>○ **Reasoning:** Vitamin B12 deficiency can cause subacute combined degeneration of the spinal cord and peripheral neuropathy. It can present with a sensory-motor neuropathy, although it is usually symmetric. The presence of vitamin B12 deficiency in this patient could contribute to the neuropathy but might not fully explain the asymmetric presentation and rapid progression.<br>**Most Likely Diagnosis**<br>**Paraneoplastic Polyneuropathy:**<br>Based on the clinical presentation, the most likely diagnosis is paraneoplastic polyneuropathy. The rapid progression of symptoms, asymmetric distribution, significant weight loss, and presence of cutaneous angiomas (which can be paraneoplastic manifestations) strongly point towards an underlying malignancy. The detection of IgA lambda paraprotein further supports this possibility, as certain cancers, especially hematologic malignancies, can produce monoclonal gammopathies. Further investigations to identify an underlying malignancy would be warranted to confirm this diagnosis.
79	A 55-year-old male patient presents with a 1-year history of a pure sensory, length dependent polyneuropathy with insidious onset and slow progressive course. The patient presents with paresthesia and neuropathic pain in feet. He also reports cramps. Clinical examination does not reveal ataxia, tremor, foot deformities, cranial nerve or autonomic involvement. Past medical history is positive for IgG MGUS and Hashimoto's thyroiditis. Nerve conduction studies show an axonal, sensory-motor, symmetric, length dependent polyneuropathy. Screening lab tests including vitamin B12, folate, vitamin B6, vitamin E, HBV, HCV, glycemia, HbA1c, ANA, ANCA, ENA, CRP, ESR, and copper are all negative, with positivity for IgG monoclonal gammopathy. There is no family history of polyneuropathy.	MGUS-associated neuropathy	**Summary of Pertinent Clinical Information**<br>**Patient Profile:**<br>● Age: 55 years<br>● Sex: Male<br>**Presenting Symptoms:**<br>● 1-year history of pure sensory, length-dependent polyneuropathy<br>● Insidious onset and slow progressive course<br>● Paresthesia and neuropathic pain in feet<br>● Reports of cramps<br>**Clinical Examination:**<br>● No ataxia<br>● No tremor<br>● No foot deformities<br>● No cranial nerve involvement<br>● No autonomic involvement<br>**Medical History:**<br>● IgG MGUS (Monoclonal Gammopathy of Undetermined Significance)<br>● Hashimoto's thyroiditis<br>**Nerve Conduction Studies:**<br>● Axonal, sensory-motor, symmetric, length-dependent polyneuropathy<br>**Laboratory Tests:**<br>● Negative: Vitamin B12, folate, vitamin B6, vitamin E, HBV, HCV, glycemia, HbA1c, ANA, ANCA, ENA, CRP, ESR, and copper<br>● Positive: IgG monoclonal gammopathy<br>**Family History:**<br>● No family history of polyneuropathy<br>**Differential Diagnoses for Polyneuropathy**<br>**1. Monoclonal Gammopathy of Undetermined Significance (MGUS)-related Neuropathy**<br>**Reasoning:**<br>● The patient has a confirmed diagnosis of IgG MGUS, which is known to be associated with neuropathy.<br>● MGUS can lead to a slowly progressive sensory or sensorimotor neuropathy, often length-dependent and axonal, as seen in this patient.

			● The absence of other significant findings or systemic involvement further supports this diagnosis.<br>**2. Chronic Inflammatory Demyelinating Polyneuropathy (CIDP)**<br>**Reasoning:**<br>● CIDP typically presents with a progressive course and can be associated with both sensory and motor symptoms.<br>● However, the patient's nerve conduction studies indicate an axonal rather than a demyelinating pattern, making CIDP less likely but still a consideration as it can occasionally present with axonal features.<br>● The chronic, progressive nature and the patient's immune-related conditions (Hashimoto's thyroiditis) might suggest an underlying immune-mediated neuropathy.<br>**3. Idiopathic Axonal Neuropathy**<br>**Reasoning:**<br>● Given the negative screening for common causes of neuropathy and the absence of family history, idiopathic axonal neuropathy remains a possibility.<br>● This diagnosis is often one of exclusion, especially in older adults where the neuropathy is slowly progressive and length-dependent.<br>**Most Likely Diagnosis**<br>The most likely diagnosis for this patient is **Monoclonal Gammopathy of Undetermined Significance (MGUS)-related Neuropathy**. This conclusion is supported by the patient's history of IgG MGUS, the typical presentation of a length-dependent, axonal polyneuropathy, and the exclusion of other common causes through comprehensive lab testing. The association between MGUS and neuropathy is well-documented, and the clinical presentation aligns with what is expected in MGUS-related neuropathy.
80	A 70-year-old male patient presents with a 2-year history of pure sensory, symmetrical, length dependent polyneuropathy with insidious onset. The patient presents with neuropathic pain in the lower limbs in a distal distribution. Clinical examination reveals hyperalgesia to pinprick and absence of tactile hypoesthesia. There are no foot deformities, ataxia, tremor, or cranial nerve involvement. The patient reports reduced sweating. Past medical history is negative. Nerve conduction studies show a pure sensory, symmetric, axonal polyneuropathy. Screening lab tests show raised glycemia, elevated HbA1c, and reduced vitamin B12. Remaining exams including folate, vitamin B6, vitamin E, HBV, HCV, ANA, ANCA, ENA, CRP, ESR, copper, cryoglobulins, and serum electrophoresis are all negative. There is no family history of polyneuropathy.	Diabetic neuropathy	**Step-by-Step Clinical Scenario Analysis**<br>**Step 1: Summarize Pertinent Clinical Information**<br>● **Patient Profile:** 70-year-old male<br>● **Symptoms:**<br>○ 2-year history of pure sensory, symmetrical, length-dependent polyneuropathy<br>○ Insidious onset<br>○ Neuropathic pain in lower limbs (distal distribution)<br>○ Hyperalgesia to pinprick<br>○ Absence of tactile hypoesthesia<br>○ Reduced sweating<br>● **Clinical Findings:**<br>○ No foot deformities, ataxia, tremor, or cranial nerve involvement<br>● **Past Medical History:** Negative<br>● **Nerve Conduction Studies:** Pure sensory, symmetric, axonal polyneuropathy<br>● **Lab Results:**<br>○ Raised glycemia<br>○ Elevated HbA1c<br>○ Reduced vitamin B12<br>○ Normal levels of folate, vitamin B6, vitamin E, HBV, HCV, ANA, ANCA, ENA, CRP, ESR, copper, cryoglobulins, serum electrophoresis<br>● **Family History:** No polyneuropathy<br>**Step 2: Generate Differential Diagnoses for Polyneuropathy**<br>1. **Diabetic Neuropathy**<br>2. **Vitamin B12 Deficiency Neuropathy**<br>3. **Idiopathic Neuropathy**<br>**Step 3: Detailed Reasoning for Each Proposed Diagnosis**<br>1. **Diabetic Neuropathy:**<br>○ **Supporting Evidence:**<br>■ Raised glycemia and elevated HbA1c indicate poorly controlled diabetes mellitus.<br>■ Diabetic neuropathy commonly presents as a distal, symmetrical, sensory polyneuropathy, matching the patient's symptoms and nerve conduction study findings.<br>■ The insidious onset and chronic nature align with typical diabetic neuropathy progression.<br>■ Reduced sweating is a common autonomic symptom in diabetic neuropathy.<br>○ **Conclusion:** Given the elevated glycemia and HbA1c, diabetic neuropathy is highly probable.<br>2. **Vitamin B12 Deficiency Neuropathy:**<br>○ **Supporting Evidence:**<br>■ Reduced vitamin B12 levels are noted, which is known to cause sensory neuropathy.<br>■ Vitamin B12 deficiency can lead to demyelination and axonal damage, resulting in sensory symptoms.<br>■ Neuropathy from vitamin B12 deficiency is typically symmetrical and can involve sensory nerves.<br>○ **Counterpoints:**<br>■ Vitamin B12 deficiency neuropathy often includes symptoms such as gait disturbances and ataxia, which are absent in this patient.<br>○ **Conclusion:** While vitamin B12 deficiency is a potential contributing factor, the absence of more widespread neurological signs reduces its likelihood as the primary diagnosis.<br>3. **Idiopathic Neuropathy:**<br>○ **Supporting Evidence:**<br>■ In the absence of clear etiological factors, idiopathic neuropathy can be considered.<br>■ The patient's history and normal results for a wide array of other potential causes (e.g., infections, autoimmune markers, other vitamin deficiencies) support idiopathic etiology as a differential.<br>○ **Counterpoints:**<br>■ Elevated glycemia and HbA1c provide a more specific alternative diagnosis.<br>○ **Conclusion:** Idiopathic neuropathy remains a consideration but is less likely than diabetic neuropathy given the specific laboratory findings.<br>**Step 4: Determine the Most Likely Diagnosis**<br>**Most Likely Diagnosis: Diabetic Neuropathy**

			● The combination of raised glycemia and elevated HbA1c strongly suggests that diabetes is a significant contributing factor.<br>● The clinical presentation of distal symmetrical sensory polyneuropathy with autonomic features (reduced sweating) fits well with diabetic neuropathy.<br>● While vitamin B12 deficiency may play a role in exacerbating symptoms, the primary diagnosis is more consistent with diabetic neuropathy.<br>**Conclusion**<br>The most likely diagnosis for this 70-year-old male patient presenting with a 2-year history of pure sensory, symmetrical, length-dependent polyneuropathy, raised glycemia, and elevated HbA1c, is **Diabetic Neuropathy**. This diagnosis is supported by both clinical presentation and laboratory findings, making it the primary consideration in this case.
81	A 67-year-old male patient presents with a 1-week history of pure motor, symmetrical, non-length dependent polyneuropathy with hyperacute onset. The patient presents with facial diplegia, diplopia, and weakness of the intrinsic muscles of the hands. Clinical examination reveals the presence of ophthalmoparesis affecting the right retrolateral muscle and the left superior rectus, facial diplegia, weakness of the intrinsic muscles of both hands, and mild sensory ataxia. There are no neuropathic pain, tremor, foot deformities or autonomic involvement. Past medical history is positive for type 1 diabetes, IgG MGUS, and arterial hypertension. Nerve conduction studies show normal result. Screening lab tests show hyperglycaemia and raised HbA1c levels, IgG monoclonal gammopathy, and mild increase of ESR and CRP levels. Remaining exams including vitamin B12, folate, vitamin B6, vitamin E, HBV, HCV, ANA, ENA, ANCA, copper, and cryoglobulins are normal. There is no family history of polyneuropathy.	GBS-facial diplegia variant	**Clinical Summary**<br>**Patient Demographics:**<br>● **Age:** 67 years<br>● **Sex:** Male<br>**Presenting Symptoms (1-week history):**<br>● **Pure motor, symmetrical, non-length dependent polyneuropathy**<br>● **Facial diplegia**<br>● **Diplopia**<br>● **Weakness of intrinsic muscles of the hands**<br>● **Ophthalmoparesis:**<br>○ Right retrolateral muscle<br>○ Left superior rectus<br>● **Mild sensory ataxia**<br>**Clinical Findings:**<br>● **Facial diplegia**<br>● **Weakness of intrinsic muscles of both hands**<br>● **Mild sensory ataxia**<br>**Negative Findings:**<br>● No neuropathic pain<br>● No tremor<br>● No foot deformities<br>● No autonomic involvement<br>**Past Medical History:**<br>● **Type 1 diabetes**<br>● **IgG MGUS (monoclonal gammopathy of undetermined significance)**<br>● **Arterial hypertension**<br>**Investigations:**<br>● **Nerve conduction studies:** Normal<br>● **Lab Tests:**<br>○ Hyperglycaemia<br>○ Raised HbA1c levels<br>○ IgG monoclonal gammopathy<br>○ Mild increase in ESR and CRP<br>○ Normal levels of vitamin B12, folate, vitamin B6, vitamin E, HBV, HCV, ANA, ENA, ANCA, copper, and cryoglobulins<br>**Family History:**<br>● No family history of polyneuropathy<br>**Differential Diagnoses for Polyneuropathy**<br>1. **Guillain-Barré Syndrome (GBS), particularly Miller Fisher Syndrome (MFS) Variant**<br>○ **Reasoning:**<br>■ Hyperacute onset of motor symptoms and cranial nerve involvement (ophthalmoparesis, facial diplegia).<br>■ Presence of ataxia and ophthalmoparesis aligns with Miller Fisher syndrome, a variant of GBS.<br>■ Normal nerve conduction studies can occur early in the disease course of GBS/MFS.<br>■ Elevated ESR and CRP can be consistent with an acute inflammatory process.<br>2. **Diabetic Amyotrophy (Diabetic Lumbosacral Radiculoplexus Neuropathy)**<br>○ **Reasoning:**<br>■ Rapid onset of motor symptoms in a diabetic patient.<br>■ Although typically affecting lower limbs, a variant or an atypical presentation can involve upper limbs.<br>■ Hyperglycaemia and raised HbA1c indicate poor glycemic control, potentially contributing to the neuropathy.<br>■ However, the symmetrical nature and involvement of cranial nerves are less typical for this condition.<br>3. **Multifocal Motor Neuropathy (MMN)**<br>○ **Reasoning:**<br>■ Pure motor neuropathy with focal involvement can present similarly.<br>■ Facial diplegia and hand muscle weakness are consistent with MMN.<br>■ Presence of IgG MGUS can be associated with immune-mediated neuropathies like MMN.<br>■ However, the acute onset and cranial nerve involvement are less typical for MMN, making it a less likely diagnosis compared to GBS/MFS.<br>**Most Likely Diagnosis**<br>**Guillain-Barré Syndrome (GBS), particularly the Miller Fisher Syndrome (MFS) Variant**

			**Reasoning:**<br>● The hyperacute onset of symptoms within one week.<br>● Pure motor, symmetrical, non-length dependent polyneuropathy with cranial nerve involvement (ophthalmoparesis and facial diplegia).<br>● Mild sensory ataxia and specific pattern of muscle involvement (intrinsic muscles of hands).<br>● The presence of IgG MGUS could indicate a paraneoplastic or autoimmune etiology, but the clinical presentation aligns more closely with GBS/MFS than MMN.<br>● Normal nerve conduction studies can be seen in the early phase of GBS/MFS.<br>● Elevated ESR and CRP suggest an inflammatory process which is consistent with GBS/MFS.<br>Thus, the combination of clinical presentation, rapid progression, and the involvement of cranial nerves strongly suggests Guillain-Barré Syndrome, particularly the Miller Fisher Syndrome variant, as the most likely diagnosis.
82	An 87-year-old male patient presents with a 6-month history of pure sensory, symmetrical, length dependent polyneuropathy with subacute onset. The patient presents with leg paresthesia, tremor and sensory ataxia without neuropathic pain, foot deformities, cranial nerve or autonomic involvement. Past medical history is silent. Nerve conduction studies show pure a symmetric, sensory, axonal polyneuropathy and unilateral carpal tunnel syndrome. Screening lab tests including folate, vitamin B6, vitamin E, HCV, HBV, ANA, ANCA, ENA, CRP, ESR, glycemia, HbA1c, copper, cryoglobulins and serum electrophoresis are all negative, with evidence of vitamin B12 deficiency. There is no family history of polyneuropathy.	B12-deficiency polyneuropathy	**Step 1: Summarize Pertinent Clinical Information**<br>**Patient Profile:**<br>● **Age/Gender:** 87-year-old male<br>● **Presenting Symptoms:**<br>○ 6-month history of pure sensory, symmetrical, length-dependent polyneuropathy with subacute onset<br>○ Leg paresthesia<br>○ Tremor<br>○ Sensory ataxia<br>● **Negative Symptoms:**<br>○ No neuropathic pain<br>○ No foot deformities<br>○ No cranial nerve involvement<br>○ No autonomic involvement<br>**Past Medical History:**<br>● Silent (no significant past medical conditions noted)<br>**Diagnostic Findings:**<br>● **Nerve Conduction Studies:**<br>○ Pure sensory, symmetric, axonal polyneuropathy<br>○ Unilateral carpal tunnel syndrome<br>● **Screening Laboratory Tests:**<br>○ Negative: folate, vitamin B6, vitamin E, HCV, HBV, ANA, ANCA, ENA, CRP, ESR, glycemia, HbA1c, copper, cryoglobulins, serum electrophoresis<br>○ Positive: Vitamin B12 deficiency<br>● **Family History:** No family history of polyneuropathy<br>**Step 2: Generate Differential Diagnoses for Polyneuropathy**<br>1. **Vitamin B12 Deficiency**<br>○ **Reasoning:**<br>■ The patient has confirmed vitamin B12 deficiency.<br>■ Vitamin B12 deficiency is a well-known cause of sensory polyneuropathy, often presenting with paresthesia, sensory ataxia, and tremor.<br>■ The absence of neuropathic pain and autonomic involvement aligns with the presentation of vitamin B12 deficiency-related neuropathy.<br>■ Sensory ataxia and tremor can occur due to subacute combined degeneration of the spinal cord, a complication of severe vitamin B12 deficiency.<br>2. **Chronic Inflammatory Demyelinating Polyneuropathy (CIDP)**<br>○ **Reasoning:**<br>■ Although less likely given the axonal rather than demyelinating pattern on nerve conduction studies, CIDP can present with a pure sensory variant.<br>■ The symmetrical and progressive nature over six months with sensory involvement fits CIDP's chronic course.<br>■ However, the lack of response to inflammation markers (CRP, ESR) and absence of motor involvement makes CIDP less probable.<br>3. **Idiopathic Sensory Polyneuropathy**<br>○ **Reasoning:**<br>■ In elderly patients, idiopathic sensory polyneuropathy is a common diagnosis when other causes are excluded.<br>■ The symmetrical, length-dependent nature without motor or autonomic involvement is typical for idiopathic sensory polyneuropathy.<br>■ The patient's age and presentation without other systemic symptoms could suggest this diagnosis.<br>**Step 3: Determine the Most Likely Diagnosis**<br>**Most Likely Diagnosis: Vitamin B12 Deficiency**<br>● **Supporting Points:**<br>○ Confirmed laboratory evidence of vitamin B12 deficiency.<br>○ Clinical symptoms (paresthesia, sensory ataxia, and tremor) are consistent with vitamin B12 deficiency neuropathy.<br>○ No evidence of other systemic or inflammatory causes in the screening tests.<br>○ The pure sensory, axonal nature of the neuropathy on nerve conduction studies aligns with vitamin B12 deficiency.<br>○ The absence of neuropathic pain, autonomic involvement, and a silent past medical history further supports this diagnosis.<br>**Conclusion:** The most likely diagnosis for this patient's polyneuropathy is vitamin B12 deficiency. This conclusion is drawn from the clinical presentation, laboratory findings, and exclusion of other potential causes through comprehensive screening.
83	A 72-year-old male patient presents with a 1-year history of sensory-motor, symmetrical, non-length dependent polyneuropathy with subacute onset. The patient presents with hand weakness,	CIDP	**Step 1: Summarize Pertinent Clinical Information**<br>● **Patient Demographics:**<br>○ 72-year-old male<br>● **Clinical Presentation:**<br>○ 1-year history of sensory-motor, symmetrical, non-length dependent polyneuropathy

	paresthesia and hypoesthesia in the lower limbs, sensory ataxia, dysphagia, and pes cavus. There are no neuropathic pain or autonomic involvement. Past medical history is silent. Nerve conduction studies show a demyelinating, sensory-motor, asymmetric polyneuropathy. Screening lab tests including vitamin B12, folate, vitamin B6, vitamin E, HCV, HBV, ANA, ANCA, ENA, CRP, ESR, glycemia, HbA1c, copper, cryoglobulins and serum electrophoresis are all negative. There is no family history of polyneuropathy.		○ Subacute onset<br>○ Symptoms:<br>■ Hand weakness<br>■ Paresthesia and hypoesthesia in the lower limbs<br>■ Sensory ataxia<br>■ Dysphagia<br>■ Pes cavus<br>○ No neuropathic pain or autonomic involvement<br>● **Past Medical History:**<br>○ Silent (no significant past medical history)<br>● **Diagnostic Findings:**<br>○ Nerve conduction studies: Demyelinating, sensory-motor, asymmetric polyneuropathy<br>○ Screening lab tests: Negative for vitamin B12, folate, vitamin B6, vitamin E, HCV, HBV, ANA, ANCA, ENA, CRP, ESR, glycemia, HbA1c, copper, cryoglobulins, and serum electrophoresis<br>● **Family History:**<br>○ No family history of polyneuropathy<br>**Step 2: Generate Differential Diagnoses**<br>**Differential Diagnosis 1: Chronic Inflammatory Demyelinating Polyneuropathy (CIDP)**<br>**Reasoning:**<br>● CIDP is characterized by a chronic progressive or relapsing sensory and motor neuropathy.<br>● The subacute onset, symmetrical presentation, and demyelinating nature on nerve conduction studies align with CIDP.<br>● Sensory ataxia and muscle weakness are common features of CIDP.<br>● The absence of neuropathic pain and autonomic involvement is consistent, as these are not universal in CIDP.<br>● CIDP often requires exclusion of other causes, and the negative screening tests support this diagnosis.<br>**Differential Diagnosis 2: Multifocal Motor Neuropathy (MMN) with Conduction Block**<br>**Reasoning:**<br>● MMN typically presents with asymmetric motor neuropathy but can have sensory involvement in some cases.<br>● The patient's asymmetric findings on nerve conduction studies could suggest MMN.<br>● Hand weakness and sensory deficits are compatible with MMN, though it is predominantly motor.<br>● MMN can present with demyelination, consistent with the patient's nerve conduction studies.<br>● Negative screening tests do not rule out MMN, as the diagnosis is primarily clinical and electrophysiological.<br>**Differential Diagnosis 3: Hereditary Neuropathy with Liability to Pressure Palsies (HNPP)**<br>**Reasoning:**<br>● HNPP can present in older adults with chronic polyneuropathy.<br>● The demyelinating, sensory-motor neuropathy fits the electrophysiological findings of HNPP.<br>● Pes cavus and sensory ataxia are features that can be seen in hereditary neuropathies.<br>● The absence of a family history is not definitive as HNPP can sometimes present de novo.<br>● Negative routine screening tests support the consideration of a genetic neuropathy.<br>**Step 3: Determine the Most Likely Diagnosis**<br>**Most Likely Diagnosis: Chronic Inflammatory Demyelinating Polyneuropathy (CIDP)**<br>**Reasoning:**<br>● The chronic, progressive nature of the symptoms over one year with a subacute onset is highly suggestive of CIDP.<br>● The symmetrical sensory-motor polyneuropathy with demyelinating features on nerve conduction studies strongly supports CIDP.<br>● Sensory ataxia, hand weakness, and absence of pain and autonomic symptoms align well with CIDP.<br>● The negative screening tests further support a diagnosis of CIDP, as they help rule out other secondary causes of polyneuropathy.<br>● CIDP often requires a diagnosis of exclusion, and the provided data make CIDP the most fitting diagnosis based on the overall clinical picture.
84	A 60-year-old male patient presents with a 1-month history of pure sensory, symmetrical, length dependent polyneuropathy with subacute onset. The patient presents with frequent cramps, paresthesia, and neuropathic pain in the feet. There are no sensory ataxia, tremor, foot deformities, cranial nerve or autonomic involvement. Past medical history is positive for multiple myeloma, for which the patient has undergone chemotherapy. Symptoms started after the first cycle of chemotherapy. Nerve conduction studies show a pure sensory, symmetric, axonal polyneuropathy. Screening lab tests including vitamin B12, folate, vitamin B6, vitamin E, HBV, HCV, glycemia, HbA1c, ANA, ANCA, ENA, CRP,	Chemotherapy-induced neuropathy	**Clinical Information Summary**<br>**Patient Profile:**<br>● **Age:** 60-year-old male<br>● **Symptom Onset:** 1-month history<br>● **Symptoms:** Pure sensory, symmetrical, length-dependent polyneuropathy, subacute onset; frequent cramps, paresthesia, neuropathic pain in the feet<br>● **Negative Symptoms:** No sensory ataxia, tremor, foot deformities, cranial nerve, or autonomic involvement<br>● **Past Medical History:** Multiple myeloma, chemotherapy (symptoms started after the first cycle)<br>● **Nerve Conduction Studies:** Pure sensory, symmetric, axonal polyneuropathy<br>● **Screening Lab Tests:**<br>○ Negative: Vitamin B12, folate, vitamin B6, vitamin E, HBV, HCV, glycemia, HbA1c, ANA, ANCA, ENA, CRP, ESR, copper, cryoglobulins<br>○ Positive: IgG kappa paraprotein<br>● **Family History:** No family history of polyneuropathy<br>**Differential Diagnoses for Polyneuropathy**<br>**1. Chemotherapy-Induced Peripheral Neuropathy (CIPN)**<br>**Reasoning:**<br>● **Temporal Association:** Symptoms began after the first cycle of chemotherapy, indicating a strong temporal relationship.<br>● **Type of Neuropathy:** Chemotherapy-induced neuropathy is commonly sensory and symmetrical, consistent with the patient's presentation.<br>● **Underlying Condition:** The patient's history of multiple myeloma and chemotherapy is significant since many chemotherapeutic agents, especially those used in hematologic malignancies, are known to cause peripheral neuropathy.

	ESR, copper, and cryoglobulins are all negative, with positivity for IgG kappa paraprotein. There is no family history of polyneuropathy.		● **Mechanism:** Chemotherapeutic agents can cause axonal damage or impair neuronal repair mechanisms, leading to the symptoms described.<br>**2. Paraneoplastic Neuropathy**<br>**Reasoning:**<br>● **Association with Cancer:** The patient has multiple myeloma, which is known to be associated with paraneoplastic syndromes, including neuropathy.<br>● **IgG Kappa Paraprotein:** Presence of IgG kappa paraprotein suggests a possible paraneoplastic process, which is common in multiple myeloma.<br>● **Clinical Presentation:** Paraneoplastic neuropathies can present as a subacute, symmetric, sensory neuropathy.<br>● **Mechanism:** The immune response to the tumor or the presence of monoclonal proteins can lead to nerve damage.<br>**3. Monoclonal Gammopathy of Undetermined Significance (MGUS)-Related Neuropathy**<br>**Reasoning:**<br>● **Paraprotein Presence:** The positive finding of IgG kappa paraprotein might indicate MGUS, which is often associated with neuropathy.<br>● **Clinical Features:** MGUS-related neuropathy typically presents as a length-dependent, sensory, and symmetrical polyneuropathy, which matches the patient's clinical picture.<br>● **Subacute Onset:** The subacute onset of symptoms can be seen in MGUS-related neuropathy.<br>**Most Likely Diagnosis**<br>**Chemotherapy-Induced Peripheral Neuropathy (CIPN)**<br>● **Strong Temporal Association:** The onset of symptoms closely following chemotherapy initiation is highly indicative of CIPN.<br>● **Common Presentation:** Sensory, symmetrical, length-dependent polyneuropathy is a hallmark of CIPN.<br>● **Exclusion of Other Causes:** Negative results on extensive lab testing for other potential causes, combined with the presence of a clear temporal relationship with chemotherapy, make CIPN the most likely diagnosis.<br>**Conclusion**<br>Given the patient's history of multiple myeloma and recent initiation of chemotherapy, along with the subacute onset of pure sensory, symmetrical, length-dependent polyneuropathy and the absence of other identified causes, **Chemotherapy-Induced Peripheral Neuropathy (CIPN)** is the most likely diagnosis.
85	A 63-year-old male patient presents with a 4-year history of sensory-motor, symmetrical, length dependent polyneuropathy with insidious onset and fast progression. The patient presents with severe distal motor weakness and hypoesthesia in the four limbs, dysphagia, dysphonia, and autonomic involvement with no foot deformities or tremor. Past medical history shows lung cancer (20 years ago) but he did not undergo chemotherapy. Nerve conduction studies show an axonal, sensory-motor polyneuropathy with bilateral carpal tunnel syndrome. Screening lab tests including vitamin B12, folate, vitamin B6, vitamin E, HBV, HCV, ANA, ANCA, ENA, glycaemia, HbA1c, CRP, ESR, copper, cryoglobulins, and serum electrophoresis are all negative. There is no family history of polyneuropathy.	TTR-Amyloidosis	**Summary of Pertinent Clinical Information:**<br>● **Patient Profile**: 63-year-old male<br>● **Symptoms**:<br>○ Sensory-motor, symmetrical, length-dependent polyneuropathy<br>○ Severe distal motor weakness<br>○ Hypoesthesia in four limbs<br>○ Dysphagia (difficulty swallowing)<br>○ Dysphonia (difficulty speaking)<br>○ Autonomic involvement (symptoms not specified)<br>● **History**:<br>○ Insidious onset with fast progression over 4 years<br>○ Past medical history of lung cancer 20 years ago (no chemotherapy)<br>● **Physical Examination**:<br>○ No foot deformities<br>○ No tremor<br>● **Nerve Conduction Studies**: Axonal, sensory-motor polyneuropathy with bilateral carpal tunnel syndrome<br>● **Laboratory Tests**:<br>○ Vitamin B12, folate, vitamin B6, vitamin E, HBV, HCV, ANA, ANCA, ENA, glycaemia, HbA1c, CRP, ESR, copper, cryoglobulins, and serum electrophoresis are all negative<br>● **Family History**: No family history of polyneuropathy<br>**Differential Diagnoses for Polyneuropathy:**<br>1. **Paraneoplastic Neuropathy**:<br>○ **Reasoning**: The patient has a history of lung cancer, which can be associated with paraneoplastic syndromes including neuropathy. Paraneoplastic neuropathy is often insidious and progressive, and it can present with sensory-motor symptoms and autonomic involvement.<br>○ **Supporting Points**:<br>■ History of lung cancer (albeit 20 years ago)<br>■ Presence of autonomic involvement (common in paraneoplastic syndromes)<br>■ Negative screening lab tests, which is consistent with paraneoplastic neuropathy since it is often diagnosed clinically and with specific antibody testing not included in the basic screening.<br>2. **Chronic Inflammatory Demyelinating Polyradiculoneuropathy (CIDP)**:<br>○ **Reasoning**: CIDP typically presents with progressive, symmetrical, sensory-motor neuropathy. Autonomic involvement can also occur, and CIDP can be associated with axonal damage as seen in nerve conduction studies.<br>○ **Supporting Points**:<br>■ Symmetrical and progressive nature of the neuropathy<br>■ Severe distal motor weakness and hypoesthesia are common in CIDP<br>■ Negative screening labs can be consistent as CIDP diagnosis is often clinical with support from CSF analysis and nerve biopsy if necessary.<br>3. **Amyloidosis (Hereditary or Acquired)**:<br>○ **Reasoning**: Amyloidosis can present with a similar clinical picture, including progressive sensory-motor neuropathy and autonomic involvement. It can be hereditary or acquired (e.g., secondary to multiple myeloma).<br>○ **Supporting Points**:<br>■ The patient's symptoms match the common presentation of amyloidosis-related neuropathy.<br>■ Autonomic involvement (dysphagia, dysphonia, and autonomic symptoms) is common in amyloidosis.<br>■ Negative routine lab tests but serum electrophoresis alone may not detect amyloidosis; further specific tests like biopsy or fat pad aspirate could be necessary.<br>**Most Likely Diagnosis:**<br>**Paraneoplastic Neuropathy**<br>**Reasoning**: The patient's history of lung cancer, although treated 20 years ago, significantly raises the suspicion for paraneoplastic syndrome. The progressive nature of the neuropathy, coupled with the autonomic symptoms and the broad negative lab workup, points towards a paraneoplastic etiology. Additionally, the lack of familial history and negative tests for more common causes of polyneuropathy make paraneoplastic neuropathy a strong contender.

			Further diagnostic steps should include specific antibody testing for paraneoplastic syndromes (e.g., anti-Hu, anti-Yo, anti-Ri), and potentially imaging to rule out any recurrence of malignancy. If paraneoplastic antibodies are positive, this would strongly support the diagnosis.
86	A 39-year-old female patient presents with a 34-year history of sensory-motor, symmetrical, length-dependent polyneuropathy with insidious onset and slowly progressive course. The patient reports paresthesia and neuropathic pain in the hands and feet, sensory ataxia, cramps, distal weakness in all four limbs, moderate calf atrophy, and pes cavus. Clinical examination does not reveal cranial nerve or autonomic involvement. Past medical history is unremarkable. Nerve conduction studies show an axonal, symmetric, length-dependent sensory-motor polyneuropathy. Screening lab tests including vitamin B12, folate, vitamin B6, vitamin E, HBV, HCV, glycemia, HbA1c, ANA, ANCA, ENA, CRP, ESR, copper, cryoglobulins, and serum electrophoresis are all negative. Her father is wheelchair-bound, and her brother and sister have walking difficulties.	CMT2	**Clinical Summary**<br>● **Patient**: 39-year-old female<br>● **History**:<br>○ 34-year history of sensory-motor, symmetrical, length-dependent polyneuropathy<br>○ Insidious onset, slowly progressive<br>○ Symptoms: Paresthesia, neuropathic pain in hands and feet, sensory ataxia, cramps, distal weakness in all four limbs, moderate calf atrophy, pes cavus<br>● **Clinical Examination**:<br>○ No cranial nerve or autonomic involvement<br>● **Past Medical History**: Unremarkable<br>● **Nerve Conduction Studies**: Axonal, symmetric, length-dependent sensory-motor polyneuropathy<br>● **Lab Tests**: Negative for vitamin B12, folate, vitamin B6, vitamin E, HBV, HCV, glycemia, HbA1c, ANA, ANCA, ENA, CRP, ESR, copper, cryoglobulins, and serum electrophoresis<br>● **Family History**:<br>○ Father is wheelchair-bound<br>○ Brother and sister have walking difficulties<br>**Differential Diagnoses**<br>**1. Charcot-Marie-Tooth Disease (CMT)**<br>● **Reasoning**:<br>○ **Clinical Presentation**: The patient's long-standing history of sensory-motor, symmetrical, length-dependent polyneuropathy with insidious onset and slow progression is highly characteristic of CMT. The presence of pes cavus, distal weakness, and calf atrophy further supports this diagnosis.<br>○ **Nerve Conduction Studies**: CMT often shows axonal degeneration or demyelination depending on the subtype. The patient's axonal neuropathy aligns with certain types of CMT, such as CMT2 (axonal form).<br>○ **Family History**: The strong family history of similar symptoms (father wheelchair-bound, siblings with walking difficulties) suggests a hereditary neuropathy. CMT is a common hereditary neuropathy with an autosomal dominant inheritance pattern.<br>**2. Hereditary Sensory and Autonomic Neuropathy (HSAN)**<br>● **Reasoning**:<br>○ **Clinical Presentation**: HSAN presents with a similar picture of sensory-motor neuropathy, often with neuropathic pain and sensory ataxia. However, autonomic involvement is typically more pronounced in HSAN, which is not observed in this patient.<br>○ **Nerve Conduction Studies**: HSAN can show axonal degeneration. The sensory ataxia and neuropathic pain are consistent with HSAN.<br>○ **Family History**: The hereditary nature of HSAN aligns with the family history of similar symptoms, though the absence of autonomic symptoms in the patient and family members makes this diagnosis less likely compared to CMT.<br>**3. Hereditary Amyloidosis**<br>● **Reasoning**:<br>○ **Clinical Presentation**: Hereditary amyloidosis can present with progressive polyneuropathy, often affecting sensory and motor nerves symmetrically. Symptoms like neuropathic pain and paresthesia fit this diagnosis.<br>○ **Nerve Conduction Studies**: Axonal neuropathy is typical in hereditary amyloidosis.<br>○ **Family History**: The progressive nature of the disease and family history of similar symptoms suggest a genetic component. Amyloidosis is a possibility, though less common than CMT.<br>**Most Likely Diagnosis**<br>Based on the clinical presentation, nerve conduction studies, and family history, **Charcot-Marie-Tooth Disease (CMT)**is the most likely diagnosis. The patient's symptoms and the strong familial pattern align well with this hereditary neuropathy, making it the most probable diagnosis among the differentials considered. Further genetic testing can confirm the specific type of CMT and guide management and counseling.
87	A 50-year-old male patient presents with a 1-month history of pure motor multineuropathy with acute onset while hospitalized in ICU for sepsis. Clinical examination reveals the presence of dysphagia, dysphonia, hand tremor, and upper limb weakness in a multifocal distribution. There are no sensory deficits, nor neuropathic pain, tremor or foot deformities. Past medical history is positive for testicular cancer, for which the patient underwent chemotherapy three years ago. Nerve conduction studies show pure motor, axonal multineuropathy with one motor conduction block. Screening lab tests including vitamin B12, folate, vitamin B6, vitamin E, HCV, HBV, ANA, ANCA, ENA, CRP, ESR, glycemia, HbA1c, copper, cryoglobulins and serum electrophoresis are all negative. There is no family history of polyneuropathy.	GBS	**Summary of Pertinent Clinical Information**<br>● **Patient:** 50-year-old male<br>● **Presenting complaint:** 1-month history of pure motor multineuropathy<br>● **Onset:** Acute onset while hospitalized in ICU for sepsis<br>● **Symptoms:**<br>○ Dysphagia<br>○ Dysphonia<br>○ Hand tremor<br>○ Upper limb weakness in a multifocal distribution<br>● **Examination findings:**<br>○ Pure motor involvement<br>○ Multifocal distribution of upper limb weakness<br>○ No sensory deficits<br>○ No neuropathic pain, foot deformities, or additional tremors noted<br>● **Past medical history:**<br>○ Testicular cancer treated with chemotherapy three years ago<br>● **Nerve conduction studies:** Pure motor, axonal multineuropathy with one motor conduction block<br>● **Laboratory results:**<br>○ Negative for vitamin B12, folate, vitamin B6, vitamin E, HCV, HBV, ANA, ANCA, ENA, CRP, ESR, glycemia, HbA1c, copper, cryoglobulins, and serum electrophoresis<br>● **Family history:** Negative for polyneuropathy<br>**Differential Diagnoses for Polyneuropathy**<br>**1. Paraneoplastic Neuropathy**<br>● **Reasoning:**<br>○ The patient's history of testicular cancer treated with chemotherapy raises the possibility of paraneoplastic syndromes, which can include motor neuropathies.

			○ Paraneoplastic syndromes can present with acute or subacute onset of motor symptoms without sensory involvement.<br>○ The negative laboratory tests make autoimmune and metabolic causes less likely, supporting a paraneoplastic etiology.<br>○ Paraneoplastic neuropathy can sometimes present after the primary cancer treatment, linked to immune-mediated damage.<br>**2. Critical Illness Polyneuropathy (CIP)**<br>● **Reasoning:**<br>○ The acute onset of symptoms in the context of an ICU stay for sepsis strongly suggests CIP.<br>○ CIP commonly presents with symmetric motor and sensory deficits, but purely motor forms have been documented.<br>○ The patient's dysphagia, dysphonia, and multifocal upper limb weakness are consistent with CIP affecting cranial nerves and upper extremities.<br>○ The normal laboratory findings support this diagnosis, as CIP is primarily diagnosed clinically and electrophysiologically.<br>**3. Chemotherapy-Induced Peripheral Neuropathy (CIPN)**<br>● **Reasoning:**<br>○ The patient has a history of chemotherapy, a known risk factor for peripheral neuropathy.<br>○ CIPN typically presents with a mixed sensorimotor neuropathy, but pure motor involvement is also possible.<br>○ The onset of neuropathy several years post-chemotherapy is unusual but not impossible, especially with prior severe sepsis potentially unmasking latent neuropathy.<br>○ Electrophysiological findings of motor axonal neuropathy with conduction block align with potential chemotherapy-induced damage.<br>**Most Likely Diagnosis**<br>**Critical Illness Polyneuropathy (CIP):**<br>● The clinical context of acute onset during ICU hospitalization for sepsis, combined with the pure motor presentation and electrophysiological findings, strongly suggests CIP.<br>● Although the patient's history of cancer and chemotherapy raises suspicion for paraneoplastic or chemotherapy-induced neuropathy, the timing and clinical scenario more convincingly point toward CIP.<br>● The absence of sensory involvement, which is typical in CIP, further supports this diagnosis.<br>Thus, considering the clinical presentation and the patient's recent medical history, Critical Illness Polyneuropathy is the most likely diagnosis.
88	A 58-year-old male patient presents with a 15-month history of sensory-motor, symmetrical, length-dependent polyneuropathy with insidious onset and progressive course. The patient reports distal paresthesia, numbness, neuropathic pain in the lower limbs in a symmetric distribution, and sensory ataxia. Clinical examination confirms the presence of sensory ataxia, postural hand tremor, and mild weakness in ankle dorsiflexion. There are no foot deformities, cranial nerve involvement, or autonomic dysfunction. Past medical history is positive for IgM kappa MGUS and type 2 diabetes. Nerve conduction studies show a demyelinating, length-dependent, symmetric, sensory-motor polyneuropathy. Screening lab tests including vitamin B12, folate, vitamin B6, vitamin E, HCV, HBV, ANA, ANCA, ENA, CRP, ESR, glycemia, copper, and cryoglobulins are all negative, with evidence of IgM kappa paraprotein and elevated HbA1c levels. There is no family history of polyneuropathy.	Anti-MAG polyneuropathy	**Summary of Clinical Information**<br>● **Patient**: 58-year-old male<br>● **History**: 15-month history of sensory-motor, symmetrical, length-dependent polyneuropathy with insidious onset and progressive course<br>● **Symptoms**:<br>○ Distal paresthesia and numbness<br>○ Neuropathic pain in lower limbs (symmetric distribution)<br>○ Sensory ataxia<br>○ Postural hand tremor<br>○ Mild weakness in ankle dorsiflexion<br>● **Clinical Findings**:<br>○ Sensory ataxia<br>○ Postural hand tremor<br>○ Mild weakness in ankle dorsiflexion<br>● **No**:<br>○ Foot deformities<br>○ Cranial nerve involvement<br>○ Autonomic dysfunction<br>● **Past Medical History**:<br>○ IgM kappa MGUS (Monoclonal Gammopathy of Undetermined Significance)<br>○ Type 2 diabetes<br>● **Nerve Conduction Studies**: Demyelinating, length-dependent, symmetric, sensory-motor polyneuropathy<br>● **Lab Results**:<br>○ Negative: Vitamin B12, folate, vitamin B6, vitamin E, HCV, HBV, ANA, ANCA, ENA, CRP, ESR, glycemia, copper, cryoglobulins<br>○ Positive: IgM kappa paraprotein, elevated HbA1c<br>● **Family History**: No family history of polyneuropathy<br>**Differential Diagnoses for Polyneuropathy**<br>1. **Diabetic Polyneuropathy**<br>○ **Reasoning**:<br>■ The patient has a history of type 2 diabetes and elevated HbA1c levels, indicating poor glycemic control, which is a well-known risk factor for diabetic polyneuropathy.<br>■ Diabetic polyneuropathy is typically a length-dependent, symmetrical, sensory-motor neuropathy.<br>■ Symptoms such as distal paresthesia, numbness, neuropathic pain, and mild motor weakness align well with diabetic polyneuropathy.<br>■ Sensory ataxia can occur in diabetic patients due to loss of proprioception.<br>2. **Monoclonal Gammopathy of Undetermined Significance (MGUS)-associated Neuropathy**<br>○ **Reasoning**:<br>■ The presence of IgM kappa paraprotein suggests a monoclonal gammopathy, which can be associated with neuropathy.<br>■ MGUS can cause a demyelinating neuropathy, consistent with the nerve conduction study findings.<br>■ Patients with MGUS often present with symptoms similar to those described, including sensory disturbances and mild motor deficits.<br>■ The absence of autonomic dysfunction and cranial nerve involvement fits with MGUS-associated neuropathy.<br>3. **Chronic Inflammatory Demyelinating Polyneuropathy (CIDP)**<br>○ **Reasoning**:<br>■ CIDP is a chronic progressive neuropathy with demyelinating features on nerve conduction studies.<br>■ It typically presents with sensory and motor involvement, and sensory ataxia is common.

			■ Although there is no family history or foot deformities, the progressive course over 15 months fits CIDP.<br>■ However, the absence of autonomic dysfunction and cranial nerve involvement is somewhat less typical but still possible.<br>**Most Likely Diagnosis**<br>Considering the patient's clinical presentation, history of type 2 diabetes with poor glycemic control, and the presence of IgM kappa paraprotein, the most likely diagnosis is **Diabetic Polyneuropathy**. The combination of elevated HbA1c, typical symptoms of diabetic neuropathy, and length-dependent demyelinating features on nerve conduction studies supports this diagnosis. While MGUS-associated neuropathy and CIDP are also plausible, the strong link between diabetes and polyneuropathy in the context of poor glycemic control makes diabetic polyneuropathy the most probable diagnosis.
89	A 73-year-old male patient presents with a 2-year history of sensory-motor, symmetrical, non-length dependent polyneuropathy with subacute onset. The patient presents with paresthesia and neuropathic pain in a stocking and glove distribution, proximal and distal weakness in the lower limbs, mild sensory ataxia and cramps. There are no foot deformities, tremor, cranial nerve or autonomic involvement. Clinical examination reveals cutaneous hyperpigmentation. Past medical history is positive for IgG lambda monoclonal gammopathy. Nerve conduction studies show a mixed demyelinating-axonal, sensory-motor, symmetric polyneuropathy. Screening lab tests including vitamin B12, folate, vitamin B6, vitamin E, HCV, HBV, ANA, ANCA, ENA, CRP, ESR, glycemia, HbA1c, copper and cryoglobulins are all negative, with positivity for IgG lambda paraprotein. There is no family history of polyneuropathy.	POEMS	**Summary of Pertinent Clinical Information**<br>**Patient Demographics:**<br>● Age: 73 years old<br>● Gender: Male<br>**Clinical Presentation:**<br>● 2-year history of sensory-motor, symmetrical, non-length dependent polyneuropathy<br>● Subacute onset<br>● Symptoms: paresthesia and neuropathic pain in a stocking and glove distribution, proximal and distal weakness in the lower limbs, mild sensory ataxia, and cramps<br>● Physical exam: cutaneous hyperpigmentation<br>● No foot deformities, tremor, cranial nerve or autonomic involvement<br>**Past Medical History:**<br>● IgG lambda monoclonal gammopathy<br>**Diagnostic Findings:**<br>● Nerve conduction studies: mixed demyelinating-axonal, sensory-motor, symmetric polyneuropathy<br>● Screening lab tests: Negative for vitamin B12, folate, vitamin B6, vitamin E, HCV, HBV, ANA, ANCA, ENA, CRP, ESR, glycemia, HbA1c, copper, and cryoglobulins<br>● Positive for IgG lambda paraprotein<br>● No family history of polyneuropathy<br>**Differential Diagnoses for Polyneuropathy**<br>1. **Monoclonal Gammopathy of Undetermined Significance (MGUS)-related Neuropathy:**<br>○ **Reasoning:** The patient has a known history of IgG lambda monoclonal gammopathy, which is commonly associated with polyneuropathy. MGUS can lead to a similar presentation of sensory-motor polyneuropathy, with both demyelinating and axonal features on nerve conduction studies. The presence of IgG lambda paraprotein further supports this diagnosis.<br>○ **Supporting Clinical Findings:** Symmetrical polyneuropathy, mixed demyelinating-axonal features, and presence of IgG lambda paraprotein.<br>2. **Chronic Inflammatory Demyelinating Polyneuropathy (CIDP):**<br>○ **Reasoning:** CIDP often presents with a chronic, progressive course of symmetrical sensory and motor polyneuropathy, which can include proximal and distal weakness. The subacute onset over two years, mixed demyelinating-axonal features on nerve conduction studies, and sensory ataxia are consistent with CIDP.<br>○ **Supporting Clinical Findings:** Symmetrical polyneuropathy, mixed demyelinating-axonal features, sensory ataxia, and proximal/distal weakness.<br>3. **POEMS Syndrome (Polyneuropathy, Organomegaly, Endocrinopathy, Monoclonal gammopathy, Skin changes):**<br>○ **Reasoning:** POEMS syndrome is characterized by polyneuropathy associated with monoclonal gammopathy, and the presence of cutaneous hyperpigmentation is suggestive of skin changes seen in this syndrome. The symmetric polyneuropathy and positive IgG lambda paraprotein are also consistent with POEMS.<br>○ **Supporting Clinical Findings:** Symmetrical polyneuropathy, IgG lambda paraprotein, cutaneous hyperpigmentation.<br>**Most Likely Diagnosis**<br>Given the combination of a known IgG lambda monoclonal gammopathy, symmetrical sensory-motor polyneuropathy with mixed demyelinating-axonal features, and the absence of other typical causes based on the extensive screening, the most likely diagnosis is **Monoclonal Gammopathy of Undetermined Significance (MGUS)-related Neuropathy**. This diagnosis is supported by the presence of IgG lambda paraprotein and the clinical presentation which aligns with the known manifestations of MGUS-related neuropathy.<br>While CIDP and POEMS syndrome are also strong considerations, the lack of other POEMS features (such as organomegaly or endocrinopathy) and the specific association with MGUS make MGUS-related neuropathy the most probable diagnosis in this clinical scenario.
90	A 64-year-old male patient presents with a predominantly motor, asymmetrical, non-length dependent polyneuropathy with acute onset that has progressed in the past three months. The patient also reports the presence of neuropathic pain and postural hand tremor. Clinical examination does not reveal ataxia, foot deformities, cranial nerve or autonomic involvement. Past medical history is positive for diabetes. Nerve conduction studies show demyelinating, pure motor polyneuropathy with conduction blocks. Screening lab tests including vitamin B12, folate, vitamin B6, vitamin E, HBV, HCV, ANA, ANCA, ENA, CRP, ESR, glycemia, HbA1c, copper, and cryoglobulins are all negative. Serum electrophoresis reveals the presence of IgM monoclonal gammopathy. There is no family history of polyneuropathy.	Acute onset CIDP	**Summary of Pertinent Clinical Information**<br>● **Patient Profile**: 64-year-old male<br>● **Clinical Presentation**: Predominantly motor, asymmetrical, non-length dependent polyneuropathy<br>○ Acute onset with progression over the past three months<br>○ Neuropathic pain<br>○ Postural hand tremor<br>● **Clinical Examination**:<br>○ No ataxia<br>○ No foot deformities<br>○ No cranial nerve involvement<br>○ No autonomic involvement<br>● **Past Medical History**: Diabetes<br>● **Nerve Conduction Studies**: Demyelinating, pure motor polyneuropathy with conduction blocks<br>● **Laboratory Tests**:<br>○ Negative: Vitamin B12, folate, vitamin B6, vitamin E, HBV, HCV, ANA, ANCA, ENA, CRP, ESR, glycemia, HbA1c, copper, cryoglobulins<br>○ Positive: Serum electrophoresis reveals IgM monoclonal gammopathy<br>● **Family History**: No family history of polyneuropathy<br>**Differential Diagnoses for Polyneuropathy**<br>**1. Multifocal Motor Neuropathy (MMN)**<br>**Reasoning**:<br>● **Predominantly Motor Involvement**: MMN typically presents with a predominantly motor neuropathy without significant sensory involvement.

			● **Asymmetrical Distribution**: The asymmetrical pattern of weakness is characteristic of MMN.<br>● **Nerve Conduction Studies**: The presence of demyelinating features with conduction blocks strongly supports MMN.<br>● **IgM Monoclonal Gammopathy**: While not definitive, the presence of IgM monoclonal gammopathy can be associated with MMN.<br>● **Lack of Autonomic and Cranial Nerve Involvement**: MMN typically does not involve autonomic or cranial nerves, consistent with this patient's presentation.<br>**2. Chronic Inflammatory Demyelinating Polyneuropathy (CIDP) with Atypical Presentation**<br>**Reasoning**:<br>● **Motor Predominance**: CIDP can present with predominantly motor symptoms, though it often includes sensory symptoms which are minimal or absent in this case.<br>● **Acute Onset and Progression**: While CIDP usually has a more chronic onset, acute exacerbations can occur, leading to a more rapid progression.<br>● **Demyelinating Polyneuropathy**: The nerve conduction study findings are consistent with CIDP.<br>● **IgM Monoclonal Gammopathy**: Some cases of CIDP are associated with monoclonal gammopathies.<br>● **No Cranial or Autonomic Involvement**: While CIDP can involve these systems, its absence does not rule out the diagnosis, particularly in atypical presentations.<br>**3. Paraneoplastic Neuropathy**<br>**Reasoning**:<br>● **Asymmetrical and Acute Onset**: Paraneoplastic neuropathies can present with rapid onset and asymmetrical involvement.<br>● **Predominantly Motor Symptoms**: This can also occur in paraneoplastic syndromes.<br>● **Monoclonal Gammopathy**: The presence of an IgM monoclonal gammopathy raises the suspicion of an underlying malignancy, potentially contributing to a paraneoplastic syndrome.<br>● **No Cranial or Autonomic Involvement**: This presentation can vary widely in paraneoplastic syndromes.<br>**Most Likely Diagnosis**<br>Based on the clinical presentation, nerve conduction study findings, and the presence of IgM monoclonal gammopathy, the most likely diagnosis is **Multifocal Motor Neuropathy (MMN)**. MMN is characterized by its predominantly motor involvement, asymmetrical distribution, demyelinating features with conduction blocks, and lack of sensory, cranial nerve, or autonomic involvement, all of which align closely with the patient's presentation. The presence of IgM monoclonal gammopathy further supports this diagnosis, although it is not definitive on its own.<br>Therefore, MMN stands out as the most probable diagnosis given the combination of clinical and electrophysiological findings in this case.
91	A 78-year-old male patient presents with a 1-year history of a sensory-motor, symmetrical, length dependent polyneuropathy with insidious onset and progressive course. The patient presents with sensory ataxia, hand tremor, weakness of ankle dorsiflexion, and distal leg paraesthesia. He also reports mild neuropathic pain in the feet. Clinical examination does not reveal foot deformities, autonomic or cranial nerve involvement. Past medical history is positive for type 2 diabetes, IgM kappa monoclonal gammopathy, and Waldenström macroglobulinemia. Nerve conduction studies show a demyelinating, sensory-motor, length dependent polyneuropathy. Screening lab tests shows hyperglycaemia, elevated HbA1c, and IgM monoclonal gammopathy. Remaining exams including vitamin B12, folate, vitamin B6, vitamin E, HCV, ANA, ENA, ANCA, ENA, CRP, copper, and cryoglobulins are negative. There is no family history of polyneuropathy.	Anti-MAG neuropathy	**Summary of Pertinent Clinical Information**<br>**Patient Profile:**<br>● **Age:** 78 years old<br>● **Gender:** Male<br>● **Presentation:** 1-year history of sensory-motor, symmetrical, length-dependent polyneuropathy<br>● **Symptoms:** Sensory ataxia, hand tremor, weakness of ankle dorsiflexion, distal leg paraesthesia, mild neuropathic pain in the feet<br>● **Clinical Examination:** No foot deformities, autonomic or cranial nerve involvement<br>● **Past Medical History:**<br>○ Type 2 diabetes<br>○ IgM kappa monoclonal gammopathy<br>○ Waldenström macroglobulinemia<br>● **Nerve Conduction Studies:** Demyelinating, sensory-motor, length-dependent polyneuropathy<br>● **Screening Lab Tests:**<br>○ Hyperglycaemia<br>○ Elevated HbA1c<br>○ IgM monoclonal gammopathy<br>○ Negative for vitamin B12, folate, vitamin B6, vitamin E, HCV, ANA, ENA, ANCA, CRP, copper, and cryoglobulins<br>● **Family History:** No family history of polyneuropathy<br>**Differential Diagnoses for Polyneuropathy**<br>1. **Diabetic Polyneuropathy**<br>○ **Reasoning:**<br>■ **Type 2 Diabetes History:** The patient has a history of type 2 diabetes with hyperglycaemia and elevated HbA1c, which are indicative of poor glycemic control.<br>■ **Symptoms Match:** Sensory-motor polyneuropathy, mild neuropathic pain, and distal leg paraesthesia are common in diabetic neuropathy.<br>■ **Progression and Symmetry:** The insidious onset and progressive course of the neuropathy align with the typical presentation of diabetic neuropathy.<br>○ **Conclusion:** Given the patient's significant history of diabetes and corresponding symptoms, diabetic polyneuropathy is a strong contender.<br>2. **Monoclonal Gammopathy of Undetermined Significance (MGUS) Neuropathy**<br>○ **Reasoning:**<br>■ **Presence of IgM Monoclonal Gammopathy:** The patient has IgM kappa monoclonal gammopathy, which is known to be associated with neuropathy in MGUS.<br>■ **Waldenström Macroglobulinemia:** This condition itself can be associated with neuropathy due to the presence of monoclonal proteins affecting nerve function.<br>■ **Demyelinating Polyneuropathy:** Nerve conduction studies showing demyelination are consistent with neuropathies associated with monoclonal gammopathies.<br>○ **Conclusion:** The presence of IgM monoclonal gammopathy and Waldenström macroglobulinemia strongly suggest that MGUS-related neuropathy could be the underlying cause.<br>3. **Chronic Inflammatory Demyelinating Polyneuropathy (CIDP)**<br>○ **Reasoning:**<br>■ **Demyelinating Neuropathy:** The nerve conduction studies indicate a demyelinating process, which is a hallmark of CIDP.<br>■ **Symmetrical Sensory-Motor Involvement:** CIDP typically presents with symmetrical, progressive polyneuropathy affecting both sensory and motor nerves.<br>■ **Length-dependent Polyneuropathy:** CIDP can present with a length-dependent pattern similar to what is described in this case.<br>○ **Conclusion:** CIDP is a possible diagnosis given the demyelinating nature of the neuropathy and the clinical presentation.<br>**Most Likely Diagnosis**<br>**Diabetic Polyneuropathy**<br>**Reasoning:**<br>● The patient has a long-standing history of type 2 diabetes with poor glycemic control, evidenced by hyperglycaemia and elevated HbA1c.

			• The clinical features of sensory-motor polyneuropathy, mild neuropathic pain, and distal leg paraesthesia are consistent with diabetic neuropathy.<br>• While monoclonal gammopathy and CIDP are also plausible, the strong correlation between diabetes and neuropathy in this context makes diabetic polyneuropathy the most likely diagnosis.
92	A 58-year-old female patient presents with a 3-month history of sensory-motor, symmetrical, non-length dependent polyneuropathy with acute onset and progressive course. Two weeks after an infectious event, the patient reports the appearance of paraesthesia and weakness in the hands and lower limbs. At nadir she was unable to walk. The symptoms were interpreted as Guillain Barré syndrome and the patient received a course of immunoglobulin therapy with clear benefit. After the therapy she started walking again with some difficulty. About three months after the onset of symptoms, she noticed the reappearance of the paresthesia and a worsening of her strength, so she started using a crutch. Neurological examination shows the presence of weakness, distributed proximally and distally in the lower limbs and distally in the upper limbs, and sensory ataxia. Past medical history is silent. Nerve conduction studies show a demyelinating, sensory-motor polyneuropathy. Screening lab tests including vitamin B12, folate, vitamin B6, vitamin E, HBV, HCV, ANA, ANCA, ENA, CRP, ESR, glycemia, HbA1c, copper, cryoglobulins, and serum electrophoresis are negative. There is no family history of polyneuropathy.	Acute onset CIDP	**Summary of Pertinent Clinical Information**<br>• **Patient:** 58-year-old female<br>• **History Duration:** 3 months<br>• **Symptoms:** Sensory-motor, symmetrical, non-length dependent polyneuropathy<br>• **Onset:** Acute onset with progressive course<br>• **Initial Event:** Symptoms appeared two weeks after an infectious event<br>• **Initial Symptoms:** Paraesthesia and weakness in hands and lower limbs<br>• **Condition at Nadir:** Unable to walk<br>• **Initial Diagnosis and Treatment:** Guillain Barré syndrome; treated with immunoglobulin therapy with benefit (improved walking)<br>• **Current Status:** Reappearance of paresthesia and worsening strength three months after initial symptoms; using a crutch<br>• **Neurological Examination:** Weakness proximally and distally in lower limbs, distally in upper limbs, sensory ataxia<br>• **Past Medical History:** Silent<br>• **Nerve Conduction Studies:** Demyelinating, sensory-motor polyneuropathy<br>• **Screening Lab Tests:** Negative (vitamin B12, folate, vitamin B6, vitamin E, HBV, HCV, ANA, ANCA, ENA, CRP, ESR, glycemia, HbA1c, copper, cryoglobulins, serum electrophoresis)<br>• **Family History:** No family history of polyneuropathy<br>**Differential Diagnoses for Polyneuropathy**<br>1. **Chronic Inflammatory Demyelinating Polyneuropathy (CIDP)**<br>○ **Reasoning:**<br>■ **Clinical Course:** The patient's symptoms, which improved with immunoglobulin therapy but then recurred, suggest a chronic relapsing-remitting course typical of CIDP.<br>■ **Response to Treatment:** The benefit from immunoglobulin therapy supports this diagnosis as CIDP is known to respond to such treatments.<br>■ **Nerve Conduction Studies:** The demyelinating sensory-motor polyneuropathy pattern is consistent with CIDP.<br>■ **Temporal Relationship:** Onset following an infectious event and progressive course align with CIDP.<br>2. **Multifocal Motor Neuropathy (MMN)**<br>○ **Reasoning:**<br>■ **Clinical Features:** MMN typically presents with asymmetric weakness, predominantly in the upper limbs, which does not entirely fit this patient's symmetrical and sensory-motor symptoms.<br>■ **Nerve Conduction Studies:** MMN often shows conduction block in motor nerves without sensory involvement, which is not observed in this case.<br>■ **Response to Treatment:** MMN also responds to immunoglobulin therapy, but the overall presentation does not align as closely as CIDP.<br>3. **Acute-onset Chronic Polyneuropathy (e.g., Paraneoplastic Syndrome)**<br>○ **Reasoning:**<br>■ **Clinical Course:** Acute onset with a progressive course can be seen in some paraneoplastic syndromes, although typically other signs of malignancy would be expected.<br>■ **Nerve Conduction Studies:** Demyelinating pattern can be seen in paraneoplastic neuropathies.<br>■ **Screening Tests:** Negative screening labs make this less likely, although it cannot be completely ruled out without further investigation for occult malignancy.<br>■ **Response to Treatment:** Paraneoplastic neuropathies may have variable responses to immunotherapy.<br>**Most Likely Diagnosis**<br>Based on the clinical presentation, response to treatment, nerve conduction study results, and the exclusion of other common causes through negative screening labs, the most likely diagnosis is:<br>**Chronic Inflammatory Demyelinating Polyneuropathy (CIDP)**<br>This diagnosis accounts for the relapsing-remitting course, demyelinating nature of the polyneuropathy, response to immunoglobulin therapy, and the symmetrical sensory-motor involvement. CIDP often follows an infectious event and can present with a chronic course with intermittent exacerbations, matching the patient's clinical picture.
93	A 42-year-old female patient presents with a 6-month history of chronic, sensory-motor, symmetrical, non-length dependent polyneuropathy with subacute onset. The patient presents with paresthesia and severe neuropathic pain in the four limbs in a stocking and glove distribution and sensory ataxia. Clinical examination reveals the presence of postural hand tremor, which the patient reports being present for years. Thereare no foot deformities, cranial nerve or autonomic involvement. Past medical history is positive for rectal cancer, for which the patient has undergone chemotherapy for three years. Neuropathy symptoms started during chemotherapy. Nerve conduction studies show sensory-motor, symmetric, axonal polyneuropathy. Screening lab tests including vitamin B6, vitamin E, HBV, HCV, ANA, ANCA, ENA, CRP, ESR, glycemia, HbA1c, copper, cryoglobulins and serum electrophoresis are all negative, with evidence of vitamin	Chemotherapy-induced neuropathy	**Summary of Pertinent Clinical Information**<br>• **Patient Information**: 42-year-old female<br>• **Presenting Symptoms**:<br>○ 6-month history of chronic, sensory-motor, symmetrical, non-length dependent polyneuropathy<br>○ Subacute onset of symptoms<br>○ Paresthesia and severe neuropathic pain in a stocking and glove distribution<br>○ Sensory ataxia<br>• **Clinical Examination**:<br>○ Postural hand tremor (present for years)<br>○ No foot deformities<br>○ No cranial nerve involvement<br>○ No autonomic involvement<br>• **Medical History**:<br>○ Rectal cancer, treated with chemotherapy for three years<br>○ Neuropathy symptoms began during chemotherapy<br>• **Investigations**:<br>○ Nerve conduction studies: sensory-motor, symmetric, axonal polyneuropathy<br>○ Screening lab tests:<br>■ Vitamin B6, vitamin E, HBV, HCV, ANA, ANCA, ENA, CRP, ESR, glycemia, HbA1c, copper, cryoglobulins, and serum electrophoresis all negative<br>■ Vitamin B12 deficiency present<br>○ No family history of polyneuropathy<br>**Differential Diagnoses**<br>1. **Chemotherapy-Induced Peripheral Neuropathy (CIPN)**

	B12 deficiency. There is no family history of polyneuropathy.		○ **Reasoning**:<br>■ The onset of neuropathy symptoms coincided with chemotherapy treatment.<br>■ Chemotherapeutic agents, particularly those used for rectal cancer (e.g., platinum-based drugs like oxaliplatin), are well-known to cause peripheral neuropathy.<br>■ The sensory-motor, symmetrical, and axonal nature of the neuropathy is consistent with chemotherapy-induced damage.<br>■ The stocking and glove distribution is typical for CIPN.<br>2. **Vitamin B12 Deficiency Neuropathy**<br>○ **Reasoning**:<br>■ The patient has documented vitamin B12 deficiency.<br>■ Vitamin B12 deficiency can cause subacute combined degeneration, affecting both the sensory and motor pathways, leading to peripheral neuropathy.<br>■ Sensory ataxia and paresthesia are common in B12 deficiency neuropathy.<br>■ Although the neuropathy started during chemotherapy, B12 deficiency might have contributed or exacerbated the neuropathic symptoms.<br>3. **Paraneoplastic Neuropathy**<br>○ **Reasoning**:<br>■ History of rectal cancer raises the possibility of paraneoplastic syndromes.<br>■ Paraneoplastic neuropathies can present as subacute or chronic, symmetrical, sensory-motor neuropathies.<br>■ However, the absence of cranial nerve involvement and autonomic features makes this less likely compared to CIPN and B12 deficiency.<br>■ Negative results for autoimmune markers (ANA, ANCA, ENA) and normal serum electrophoresis make this diagnosis less probable.<br>**Most Likely Diagnosis**<br>Based on the detailed clinical history, timing of symptom onset, nature of the symptoms, and laboratory findings, the most likely diagnosis is **Chemotherapy-Induced Peripheral Neuropathy (CIPN)**. The temporal relationship between the onset of neuropathy and chemotherapy, coupled with the typical clinical presentation of CIPN, strongly supports this diagnosis. The presence of vitamin B12 deficiency may contribute to the neuropathic symptoms but is likely secondary to the primary issue caused by chemotherapy.<br>**Conclusion**<br>The patient's polyneuropathy is most likely due to the toxic effects of chemotherapeutic agents used in her cancer treatment, specifically a condition known as Chemotherapy-Induced Peripheral Neuropathy (CIPN). Addressing the vitamin B12 deficiency is also important to potentially improve overall neurological function.
94	A 73-year-old female patient presents with a 1-year history of pure sensory, symmetrical, length dependent polyneuropathy with insidious onset. The patient presents with paresthesia, neuropathic pain and sensory ataxia without weakness, cranial nerve or autonomic involvement. There are also no foot deformities or tremor. Past medical history is positive for sarcoidosis. Nerve conduction studies show pure a pure sensory, axonal polyneuropathy. Screening lab tests including vitamin B12, folate, vitamin B6, vitamin E, HCV, HBV, ANCA, ENA, CRP, ESR, glycemia, HbA1c, copper, cryoglobulins and serum electrophoresis are all negative, with positivity for ANA autoantibodies. There is no family history of polyneuropathy.	Sarcoidosis associated polyneuropathy	**Summary of Clinical Information**<br>**Patient Demographics:**<br>● 73-year-old female<br>**Chief Complaint:**<br>● 1-year history of pure sensory, symmetrical, length-dependent polyneuropathy<br>**Symptoms:**<br>● Paresthesia<br>● Neuropathic pain<br>● Sensory ataxia<br>● No weakness, cranial nerve involvement, or autonomic involvement<br>**Physical Exam Findings:**<br>● No foot deformities<br>● No tremor<br>**Past Medical History:**<br>● Sarcoidosis<br>**Diagnostic Findings:**<br>● Nerve conduction studies: Pure sensory, axonal polyneuropathy<br>● Screening lab tests:<br>○ Negative: Vitamin B12, folate, vitamin B6, vitamin E, HCV, HBV, ANCA, ENA, CRP, ESR, glycemia, HbA1c, copper, cryoglobulins, serum electrophoresis<br>○ Positive: ANA autoantibodies<br>● No family history of polyneuropathy<br>**Differential Diagnoses**<br>**1. Sarcoidosis-related Neuropathy:**<br>**Reasoning:**<br>● The patient has a known history of sarcoidosis, an inflammatory disease that can affect multiple organs, including the nervous system.<br>● Sarcoidosis can cause a variety of neuropathic presentations, including sensory polyneuropathy.<br>● Though less common, sarcoidosis-related neuropathy typically involves granulomatous inflammation, which might not always be detectable on routine lab tests.<br>● The presence of ANA autoantibodies, while non-specific, can be associated with autoimmune conditions like sarcoidosis.<br>**2. Paraneoplastic Neuropathy:**<br>**Reasoning:**<br>● The presence of ANA autoantibodies could suggest an autoimmune process, potentially paraneoplastic in nature, considering the patient's age.<br>● Paraneoplastic syndromes can present as subacute or chronic polyneuropathy and are often associated with sensory manifestations.<br>● The negative screening labs do not rule out the possibility of an occult malignancy, especially in the context of an elderly patient with a recent onset of polyneuropathy.<br>● Further investigations might be needed to exclude malignancy, such as imaging studies or specific paraneoplastic antibody panels.<br>**3. Idiopathic Sensory Polyneuropathy:**<br>**Reasoning:**<br>● Despite extensive testing, a significant proportion of sensory polyneuropathy cases remain idiopathic, especially in the elderly.<br>● The clinical presentation of pure sensory, symmetrical, length-dependent polyneuropathy with insidious onset aligns with idiopathic sensory polyneuropathy.<br>● This diagnosis is often considered after exclusion of other potential causes, as seen with the extensive negative workup in this case.

			**Most Likely Diagnosis**<br>**Sarcoidosis-related Neuropathy:**<br>Given the patient's history of sarcoidosis and the exclusion of other common causes through extensive testing, sarcoidosis-related neuropathy stands out as the most likely diagnosis. While sarcoidosis primarily affects the lungs and lymph nodes, it can have neurological manifestations, including peripheral neuropathy. The positive ANA autoantibodies, although not specific, support an autoimmune etiology, which can be associated with sarcoidosis. Further, the absence of other diagnostic clues in the extensive negative workup leans toward this diagnosis. Additional tests, such as a biopsy of accessible tissue (e.g., skin, nerve) or advanced imaging, might be considered to confirm granulomatous inflammation consistent with sarcoidosis.
95	A 62-year-old male patient presents with a 5-month history of sensory-motor, symmetrical, length dependent polyneuropathy with subacute onset. The patient presents with distal paresthesia and neuropathic pain in the lower limbs, associated with weakness of toe dorsiflexion. Clinical examination also reveals the presence of orthostatic hypotension. There are no sensory ataxia, tremor, foot deformities or cranial nerve involvement. Past medical history is positive for diabetes since the age of 50, and plasmacytoma, for which the patient has undergone chemotherapy. Symptoms started during treatment with chemotherapy. Nerve conduction studies show a mixed sensory-motor, symmetric, axonal polyneuropathy. Screening lab tests including vitamin B12, folate, vitamin B6, vitamin E, HBV, HCV, ANA, ANCA, ENA, CRP, ESR, copper, cryoglobulins and serum electrophoresis are all negative, with evidence of hyperglycaemia and elevated HbA1c. There is no family history of polyneuropathy.	Chemotherapy-induced neuropathy	**Summary of Pertinent Clinical Information:**<br>● **Patient:** 62-year-old male<br>● **Duration of Symptoms:** 5 months<br>● **Symptoms:**<br>○ Sensory-motor, symmetrical, length-dependent polyneuropathy<br>○ Distal paresthesia and neuropathic pain in lower limbs<br>○ Weakness of toe dorsiflexion<br>○ Orthostatic hypotension<br>● **Negative Findings:**<br>○ No sensory ataxia<br>○ No tremor<br>○ No foot deformities<br>○ No cranial nerve involvement<br>● **Past Medical History:**<br>○ Diabetes since age 50<br>○ Plasmacytoma treated with chemotherapy<br>● **Symptom Onset:** During chemotherapy treatment<br>● **Nerve Conduction Studies:** Mixed sensory-motor, symmetric, axonal polyneuropathy<br>● **Screening Lab Results:**<br>○ Negative for vitamin B12, folate, vitamin B6, vitamin E, HBV, HCV, ANA, ANCA, ENA, CRP, ESR, copper, cryoglobulins, and serum electrophoresis<br>○ Hyperglycemia and elevated HbA1c<br>● **Family History:** No family history of polyneuropathy<br>**Differential Diagnoses for Polyneuropathy:**<br>1. **Chemotherapy-Induced Peripheral Neuropathy (CIPN):**<br>○ **Reasoning:** The onset of symptoms coincides with the start of chemotherapy for plasmacytoma, making CIPN a strong contender. Chemotherapeutic agents, particularly those used for treating plasmacytoma (such as bortezomib or thalidomide), are known to cause peripheral neuropathy as a common side effect.<br>○ **Supporting Features:**<br>■ Subacute onset of symptoms during chemotherapy<br>■ Symmetrical sensory-motor polyneuropathy<br>■ Absence of other potential causes from the negative lab tests<br>2. **Diabetic Polyneuropathy:**<br>○ **Reasoning:** The patient has a long-standing history of diabetes with evidence of poor glycemic control (hyperglycemia and elevated HbA1c), which is a well-known risk factor for developing diabetic neuropathy.<br>○ **Supporting Features:**<br>■ Long history of diabetes<br>■ Elevated HbA1c indicating poor glucose control<br>■ Symmetrical, length-dependent nature of the neuropathy, which is typical of diabetic neuropathy<br>○ **Against Features:**<br>■ The relatively rapid onset (over 5 months) and significant neuropathic pain might be more aggressive than typical diabetic neuropathy, suggesting another contributing factor.<br>3. **Amyloid Neuropathy:**<br>○ **Reasoning:** Plasmacytoma can be associated with light chain amyloidosis (AL amyloidosis), which can cause peripheral neuropathy. While screening tests for serum electrophoresis were negative, this does not entirely rule out amyloidosis, especially if the deposition is in peripheral nerves.<br>○ **Supporting Features:**<br>■ Association with plasmacytoma<br>■ Presence of autonomic symptoms such as orthostatic hypotension, which is a hallmark of amyloid neuropathy<br>○ **Against Features:**<br>■ Lack of confirmatory evidence from serum electrophoresis or other specific tests for amyloidosis<br>**Most Likely Diagnosis:**<br>**Chemotherapy-Induced Peripheral Neuropathy (CIPN):** Given the temporal relationship between the onset of symptoms and the initiation of chemotherapy for plasmacytoma, alongside the subacute nature of the neuropathy, CIPN appears to be the most likely diagnosis. The rapid development of symptoms and the combination of sensory, motor, and autonomic involvement are characteristic of CIPN. Additionally, the negative screening tests for other common causes and the patient's ongoing chemotherapy treatment further support this diagnosis.<br>**Diabetic Polyneuropathy** remains a contributing factor due to the patient's long-standing diabetes and poor glycemic control. However, the aggressive onset and additional autonomic symptoms suggest that chemotherapy is the primary cause exacerbating the underlying diabetic neuropathy.<br>**Amyloid Neuropathy** should be considered, especially in the context of a plasmacytoma, but the lack of specific confirmatory evidence makes it less likely in this scenario without further diagnostic workup.
96	An 86-year-old male patient presents with a 1-year history of sensory-motor, symmetrical, non-length dependent polyneuropathy with subacute onset and	CIDP	**Clinical Summary**<br>● **Patient**: 86-year-old male<br>● **History**: 1-year history of sensory-motor, symmetrical, non-length dependent polyneuropathy<br>○ **Onset**: Subacute

	progressive course. The patient presents with distal paresthesia in the upper limbs and sensory ataxia with weakness of ankle dorsiflexion. Clinical examination reveals mild hand tremor. There are no neuropathic pain, foot deformities, cranial nerve or autonomic involvement. Past medical history shows IgG kappa monoclonal gammopathy and basal cell skin cancer. Nerve conduction studies show a demyelinating, sensory-motor polyneuropathy. Screening lab tests including vitamin B12, folate, vitamin B6, vitamin E, HCV, ANA, ANCA, ENA, CRP, ESR, glycemia, HbA1c, copper and cryoglobulins are all negative, with evidence of chronic HBV infection and positivity for IgG kappa paraprotein. There is no family history of polyneuropathy.		○ **Course**: Progressive<br>● **Symptoms**:<br>○ Distal paresthesia in upper limbs<br>○ Sensory ataxia<br>○ Weakness of ankle dorsiflexion<br>● **Clinical Findings**:<br>○ Mild hand tremor<br>○ No neuropathic pain, foot deformities, cranial nerve, or autonomic involvement<br>● **Past Medical History**:<br>○ IgG kappa monoclonal gammopathy<br>○ Basal cell skin cancer<br>● **Nerve Conduction Studies**: Demyelinating, sensory-motor polyneuropathy<br>● **Lab Tests**:<br>○ Negative: Vitamin B12, folate, vitamin B6, vitamin E, HCV, ANA, ANCA, ENA, CRP, ESR, glycemia, HbA1c, copper, cryoglobulins<br>○ Positive: Chronic HBV infection, IgG kappa paraprotein<br>● **Family History**: No polyneuropathy<br>**Differential Diagnoses for Polyneuropathy**<br>1. **Monoclonal Gammopathy of Undetermined Significance (MGUS)-related Polyneuropathy**:<br>○ **Reasoning**: The patient has an IgG kappa monoclonal gammopathy, which can be associated with polyneuropathy. MGUS can cause a demyelinating neuropathy, often presenting with a sensory-motor pattern and can be symmetrical. The absence of other systemic features, the chronic HBV infection, and the specific type of paraprotein (IgG kappa) align with this diagnosis.<br>2. **Chronic Inflammatory Demyelinating Polyneuropathy (CIDP)**:<br>○ **Reasoning**: CIDP is characterized by a progressive, demyelinating, sensory-motor polyneuropathy, which can present in a non-length dependent pattern. The subacute onset and progressive nature fit well with CIDP. CIDP often does not show systemic involvement, similar to this case, and can be associated with paraproteinemia.<br>3. **Paraneoplastic Neuropathy**:<br>○ **Reasoning**: While the patient has a history of basal cell carcinoma, which is not commonly associated with paraneoplastic syndromes, the presence of an unexplained polyneuropathy raises the suspicion. However, the demyelinating nature of the neuropathy and the absence of other systemic or cranial nerve involvement make this diagnosis less likely compared to MGUS and CIDP.<br>**Most Likely Diagnosis**<br>The most likely diagnosis is **Monoclonal Gammopathy of Undetermined Significance (MGUS)-related Polyneuropathy**. This diagnosis is supported by the presence of IgG kappa monoclonal gammopathy, which is known to be associated with demyelinating, sensory-motor polyneuropathy. The clinical presentation of progressive, symmetrical, non-length dependent polyneuropathy, and the absence of other systemic involvements such as cranial nerve or autonomic signs further support this diagnosis. CIDP remains a close differential, but the specific paraprotein association with MGUS makes it more probable in this context.
97	A 57-year-old male patient presents with a 1-year history of sensory-motor, asymmetric, non-length dependent polyneuropathy with subacute onset and progressive course. The patient presents with weakness and paresthesia in the proximal and distal areas of the upper limbs in an asymmetric distribution. Clinical examination excludes the presence of ataxia, tremor, neuropathic pain, foot deformities, cranial nerve or autonomic involvement. Past medical history is silent. Nerve conduction studies show a demyelinating, sensory-motor multineuropathy. Screening lab tests reveal the presence of folate deficiency. Remaining exams including vitamin B12, vitamin B6, vitamin E, HBV, HCV, ANA, ANCA, ENA, CRP, ESR, glycemia, HbA1c, copper, cryoglobulins and serum electrophoresis are all negative. There is no family history of polyneuropathy.	CIDP	**Summary of Pertinent Clinical Information**<br>● **Patient Profile**: 57-year-old male<br>● **History**: 1-year history of sensory-motor, asymmetric, non-length dependent polyneuropathy<br>● **Onset and Course**: Subacute onset with progressive course<br>● **Symptoms**: Weakness and paresthesia in proximal and distal areas of the upper limbs in an asymmetric distribution<br>● **Examination Findings**:<br>○ No ataxia, tremor, neuropathic pain, foot deformities, cranial nerve or autonomic involvement<br>● **Nerve Conduction Studies**: Demyelinating, sensory-motor multineuropathy<br>● **Laboratory Findings**:<br>○ Positive for folate deficiency<br>○ Negative for vitamin B12, vitamin B6, vitamin E, HBV, HCV, ANA, ANCA, ENA, CRP, ESR, glycemia, HbA1c, copper, cryoglobulins, and serum electrophoresis<br>● **Family History**: No family history of polyneuropathy<br>**Differential Diagnoses for Polyneuropathy**<br>**1. Chronic Inflammatory Demyelinating Polyneuropathy (CIDP)**<br>● **Reasoning**:<br>○ **Presentation**: CIDP often presents with progressive, asymmetric, sensory-motor polyneuropathy, which matches the patient's symptoms.<br>○ **Nerve Conduction Studies**: The demyelinating pattern on nerve conduction studies is characteristic of CIDP.<br>○ **Exclusion of Other Causes**: Negative screening for other common causes (e.g., diabetes, nutritional deficiencies other than folate) supports this diagnosis.<br>○ **Progression**: The subacute onset and progressive nature are typical of CIDP rather than more acute causes like Guillain-Barré Syndrome.<br>**2. Multifocal Motor Neuropathy (MMN)**<br>● **Reasoning**:<br>○ **Presentation**: MMN typically presents with asymmetric, motor predominant neuropathy. While MMN usually spares sensory function, sensory involvement can sometimes be seen, albeit less common.<br>○ **Nerve Conduction Studies**: The presence of demyelination and conduction block on nerve conduction studies can also be indicative of MMN.<br>○ **Clinical Course**: The progressive and asymmetric nature without systemic symptoms or autonomic involvement aligns with MMN.<br>○ **Lab Tests**: The negative results for inflammatory markers (e.g., CRP, ESR) and autoimmune panels are consistent with MMN.<br>**3. Paraneoplastic Neuropathy**<br>● **Reasoning**:<br>○ **Presentation**: Paraneoplastic syndromes can present with subacute or chronic neuropathy, often with an asymmetric distribution.<br>○ **Association with Cancer**: Though the patient's history lacks explicit evidence of cancer, paraneoplastic neuropathies should be considered, particularly in an older patient.<br>○ **Nerve Conduction Studies**: Demyelinating patterns can be seen in paraneoplastic neuropathies.<br>○ **Exclusion of Common Causes**: The extensive negative workup for other causes makes this a differential worth considering.<br>○ **Clinical Course**: The absence of systemic symptoms does not rule out paraneoplastic syndrome, as neuropathy can sometimes precede cancer diagnosis.<br>**Most Likely Diagnosis**

			The most likely diagnosis for this patient is **Chronic Inflammatory Demyelinating Polyneuropathy (CIDP)**.<br>● **Alignment with Clinical Presentation**: The asymmetric, sensory-motor nature of the neuropathy, its subacute onset, and progressive course align well with CIDP.<br>● **Nerve Conduction Studies**: The demyelinating nature on nerve conduction studies is highly suggestive of CIDP.<br>● **Exclusion of Other Causes**: Extensive negative workup for other potential causes of neuropathy supports CIDP as a diagnosis of exclusion.<br>● **Response to Treatment**: CIDP often responds well to immunomodulatory treatments such as corticosteroids, IVIG, or plasmapheresis, which can further confirm the diagnosis upon clinical improvement.<br>Given the clinical presentation, diagnostic findings, and exclusion of other common causes, CIDP emerges as the most probable diagnosis for this patient. Further management should include consideration of immunotherapy and close monitoring of clinical response.
98	A 74-year-old male patient presents with a 1-month of sensory-motor, multifocal neuropathy with subacute onset and stepwise progression. The patient presents with paresthesia, neuropathic pain, and weakness with a multifocal distribution in the four limbs. He also reports arthralgia and headache, which began in the last two weeks. Clinical examination excludes the presence of sensory ataxia, tremor, foot deformities, cranial nerve or autonomic involvement. Past medical history is positive for arterial hypertension, Hashimoto's thyroiditis, chronic HBV infection, and type 2 diabetes mellitus. Nerve conduction studies show a multifocal, axonal, sensory-motor neuropathy. Screening lab tests reveal elevated glycemia and HbA1c leves, vitamin B12 deficiency, and raised ESR and CRP. Remaining exams including folate, vitamin B6, vitamin E, HCV, ANA, ANCA, ENA, copper, cryoglobulins, and serum electrophoresis are normal. There is no family history of polyneuropathy.	Vasculitic neuropathy	**Clinical Summary**<br>**Patient Information:**<br>● **Age/Gender:** 74-year-old male<br>● **Presenting Symptoms:**<br>○ 1-month history of sensory-motor, multifocal neuropathy<br>○ Subacute onset with stepwise progression<br>○ Paresthesia, neuropathic pain, and weakness in all four limbs<br>○ Arthralgia and headache in the last two weeks<br>**Clinical Examination:**<br>● No sensory ataxia<br>● No tremor<br>● No foot deformities<br>● No cranial nerve or autonomic involvement<br>**Medical History:**<br>● Arterial hypertension<br>● Hashimoto's thyroiditis<br>● Chronic HBV infection<br>● Type 2 diabetes mellitus<br>**Diagnostic Tests:**<br>● **Nerve Conduction Studies:** Multifocal, axonal, sensory-motor neuropathy<br>● **Lab Tests:**<br>○ Elevated glycemia and HbA1c<br>○ Vitamin B12 deficiency<br>○ Raised ESR and CRP<br>○ Normal: folate, vitamin B6, vitamin E, HCV, ANA, ANCA, ENA, copper, cryoglobulins, serum electrophoresis<br>**Family History:**<br>● No family history of polyneuropathy<br>**Differential Diagnoses for Polyneuropathy**<br>1. **Diabetic Neuropathy:**<br>○ **Reasoning:**<br>■ The patient has a known history of type 2 diabetes mellitus with elevated glycemia and HbA1c levels, which are indicative of poor glucose control.<br>■ Diabetic neuropathy typically presents as a distal symmetric polyneuropathy but can also manifest in a multifocal pattern, especially with poor glycemic control.<br>■ Symptoms of paresthesia, neuropathic pain, and weakness are consistent with diabetic neuropathy.<br>■ However, the rapid, subacute onset and stepwise progression are less typical for diabetic neuropathy, which usually develops gradually over time.<br>2. **Vasculitic Neuropathy:**<br>○ **Reasoning:**<br>■ The combination of neuropathy, arthralgia, and headache suggests a systemic inflammatory process.<br>■ Elevated ESR and CRP levels support the presence of an inflammatory or vasculitic condition.<br>■ Vasculitic neuropathy often presents with a subacute onset and stepwise progression, which matches the clinical presentation.<br>■ The multifocal distribution of neuropathy is typical for vasculitic involvement of the vasa nervorum.<br>■ Although ANA, ANCA, and other specific autoantibodies were negative, vasculitis can sometimes be present without these markers.<br>3. **Vitamin B12 Deficiency Neuropathy:**<br>○ **Reasoning:**<br>■ The patient has a documented vitamin B12 deficiency.<br>■ Vitamin B12 deficiency can cause a sensory-motor neuropathy, with symptoms including paresthesia, neuropathic pain, and weakness.<br>■ However, B12 deficiency typically causes a more symmetric neuropathy and may be associated with other findings like megaloblastic anemia or subacute combined degeneration of the spinal cord, which are not mentioned in this case.<br>■ The patient's multifocal and rapidly progressing symptoms are less typical for B12 deficiency neuropathy.<br>**Most Likely Diagnosis**<br>**Vasculitic Neuropathy:**<br>● Considering the subacute onset, stepwise progression, multifocal distribution of the neuropathy, and associated systemic symptoms (arthralgia and headache), vasculitic neuropathy is the most probable diagnosis.<br>● Elevated inflammatory markers (ESR and CRP) further support this diagnosis, despite the negative specific autoimmune markers.<br>● The patient's multiple comorbid conditions, including type 2 diabetes and chronic HBV infection, could predispose to a vasculitic process, making this diagnosis more likely.<br>**Conclusion**<br>The most likely diagnosis for the patient's presentation of subacute, multifocal sensory-motor neuropathy with systemic inflammatory signs is **vasculitic neuropathy**. This is supported by the clinical picture, elevated inflammatory markers, and the exclusion of other causes through the lab tests performed. Further diagnostic workup, including possibly a nerve biopsy and more specific tests for vasculitis, would help confirm this diagnosis.

99	A 71-year-old male patient presents with a 2-year history of sensory-motor, symmetrical, length dependent polyneuropathy with insidious onset and fast disease progression. The patient presents with moderate distal weakness and hypoesthesia in the four limbs, sensory ataxia and autonomic involvement with no foot deformities or neuropathic pain. Past medical history is positive for cardiac insufficiency. Nerve conduction studies show an axonal, sensory-motor polyneuropathy with bilateral carpal tunnel syndrome. Screening lab tests including vitamin B12, folate, vitamin B6, vitamin E, HBV, HCV, ANA, ANCA, ENA, glycaemia, HbA1c, CRP, ESR, copper, cryoglobulins, and serum electrophoresis are all negative. His brother is wheelchair bound.	TTR-Amyloidosis	**Summary of Pertinent Clinical Information:**<br>● **Patient Demographics:** 71-year-old male<br>● **Clinical Presentation:**<br>○ 2-year history of sensory-motor, symmetrical, length-dependent polyneuropathy<br>○ Insidious onset with rapid disease progression<br>○ Moderate distal weakness and hypoesthesia in all four limbs<br>○ Sensory ataxia<br>○ Autonomic involvement<br>○ No foot deformities or neuropathic pain<br>● **Past Medical History:** Cardiac insufficiency<br>● **Nerve Conduction Studies:** Axonal, sensory-motor polyneuropathy with bilateral carpal tunnel syndrome<br>● **Screening Lab Tests:** Negative for vitamin B12, folate, vitamin B6, vitamin E, HBV, HCV, ANA, ANCA, ENA, glycaemia, HbA1c, CRP, ESR, copper, cryoglobulins, and serum electrophoresis<br>● **Family History:** Brother is wheelchair-bound<br>**Differential Diagnoses for Polyneuropathy:**<br>1. **Hereditary Transthyretin Amyloidosis (hATTR):**<br>○ **Reasoning:** The patient's rapid disease progression, autonomic involvement, and family history of a similarly affected sibling strongly suggest a hereditary condition. hATTR is a common cause of familial amyloidosis with polyneuropathy, presenting with axonal sensorimotor neuropathy, autonomic dysfunction, and cardiac involvement, which aligns with the patient's cardiac insufficiency.<br>○ **Supportive Findings:** Sensory-motor neuropathy with autonomic features, family history of a similarly affected sibling, cardiac involvement.<br>2. **Chronic Inflammatory Demyelinating Polyneuropathy (CIDP):**<br>○ **Reasoning:** Although CIDP typically presents with a more demyelinating pattern on nerve conduction studies, the chronic progressive nature and symmetrical sensory-motor involvement with autonomic features can occasionally mimic axonal neuropathy. However, the absence of elevated inflammatory markers (CRP, ESR) and negative autoantibodies (ANA, ANCA) make this less likely.<br>○ **Supportive Findings:** Chronic progressive nature, symmetrical sensory-motor involvement, autonomic symptoms, but nerve conduction studies more indicative of axonal rather than demyelinating process.<br>3. **Amyloidosis (AL or ATTR):**<br>○ **Reasoning:** Amyloidosis, particularly ATTR (hereditary or wild-type), often presents with a similar clinical picture of progressive sensory-motor polyneuropathy with autonomic involvement and cardiac manifestations. Although AL amyloidosis typically presents with a monoclonal protein spike on serum electrophoresis, which was negative in this patient, the clinical presentation is still highly suggestive of amyloidosis.<br>○ **Supportive Findings:** Sensory-motor neuropathy, autonomic symptoms, cardiac insufficiency, and family history indicative of a genetic component.<br>**Most Likely Diagnosis:**<br>**Hereditary Transthyretin Amyloidosis (hATTR):**<br>● The combination of rapidly progressive sensory-motor polyneuropathy with autonomic involvement, cardiac insufficiency, and a family history of a similarly affected sibling strongly points towards hATTR. This form of amyloidosis is known for its familial nature and can present with both neurological and cardiac symptoms, fitting the patient's clinical profile well. The absence of typical inflammatory markers and negative autoimmune panel further supports this diagnosis over other inflammatory or immune-mediated neuropathies.
100	A 76-year-old female patient presents with a 6-year history of chronic, pure sensory, symmetrical, length dependent polyneuropathy with insidious onset and slow progressive course. The main symptom is severe burning and tingling pain in the feet. Clinical examination reveals hyperalgesia upon pinprick testing, along with redness and warmth in the feet. There are no weakness or tactile hypoesthesia, sensory ataxia, tremor, foot deformities, or cranial nerve involvement. Past medical history is silent. Nerve conduction studies are normal with no signs of polyneuropathy. Screening lab tests including vitamin B12, folate, vitamin B6, vitamin E, HBV, HCV, ANA, ANCA, ENA, CRP, ESR, glycemia, HbA1c, copper, cryoglobulins, and serum electrophoresis are negative. There is family history of polyneuropathy.	Small fibre neuropathy	**Summary of Pertinent Clinical Information:**<br>● **Patient:** 76-year-old female<br>● **History:**<br>○ Chronic, pure sensory, symmetrical, length-dependent polyneuropathy<br>○ 6-year history with insidious onset and slow progressive course<br>○ Severe burning and tingling pain in the feet<br>● **Clinical Examination:**<br>○ Hyperalgesia upon pinprick testing<br>○ Redness and warmth in the feet<br>○ No weakness, tactile hypoesthesia, sensory ataxia, tremor, foot deformities, or cranial nerve involvement<br>● **Nerve Conduction Studies:** Normal, no signs of polyneuropathy<br>● **Screening Lab Tests:** Negative for vitamin B12, folate, vitamin B6, vitamin E, HBV, HCV, ANA, ANCA, ENA, CRP, ESR, glycemia, HbA1c, copper, cryoglobulins, and serum electrophoresis<br>● **Family History:** Presence of polyneuropathy<br>**Differential Diagnoses:**<br>1. **Small Fiber Neuropathy (SFN):**<br>○ **Reasoning:**<br>■ The clinical presentation of severe burning and tingling pain, particularly in the feet, along with hyperalgesia, redness, and warmth, is characteristic of small fiber neuropathy.<br>■ Small fiber neuropathy often affects the small A-delta and C fibers, which are responsible for pain and temperature sensation, and these symptoms align with the patient's experience.<br>■ Normal nerve conduction studies are typical in SFN since these studies primarily assess large fibers, which are not affected in SFN.<br>■ The chronic, slowly progressive nature fits the usual course of SFN.<br>■ Family history of polyneuropathy can sometimes be associated with inherited forms of SFN, such as those caused by mutations in the SCN9A or SCN10A genes.<br>2. **Idiopathic Small Fiber Neuropathy:**<br>○ **Reasoning:**<br>■ Given the extensive negative screening tests for common causes of polyneuropathy, an idiopathic etiology is possible.<br>■ Many cases of small fiber neuropathy remain idiopathic despite thorough evaluation.<br>■ The lack of systemic disease signs or laboratory abnormalities suggests an idiopathic cause, which is relatively common in elderly patients with chronic polyneuropathy.<br>3. **Hereditary Sensory and Autonomic Neuropathy (HSAN):**<br>○ **Reasoning:**<br>■ The family history of polyneuropathy raises the possibility of a hereditary condition.<br>■ HSAN can present with pure sensory symptoms, including pain and autonomic features such as redness and warmth due to dysautonomia.<br>■ While typically earlier in onset, late-onset forms can occur, particularly in milder or atypical presentations.<br>■ Genetic testing would be necessary to confirm this diagnosis, especially given the family history.

			**Most Likely Diagnosis:**<br>The most likely diagnosis for this patient is **Small Fiber Neuropathy (SFN)**. This is supported by the clinical presentation of severe burning and tingling pain, hyperalgesia, and the normal nerve conduction studies, which are all hallmarks of SFN. Additionally, the family history of polyneuropathy and the absence of laboratory abnormalities common to other causes of polyneuropathy further support this diagnosis. If the small fiber neuropathy is confirmed through further testing, such as skin biopsy or corneal confocal microscopy, it would strengthen this conclusion. Given the negative extensive workup for secondary causes, an idiopathic or hereditary form of SFN remains the primary consideration.

Table 4. Trial 2 – Case presentations and GPT-4 output to 2nd prompt (confirmatory test)

LEGEND: Anti-MAG neuropathy, anti-myelin-associated glycoprotein neuropathy; CANVAS, cerebellar ataxia, neuropathy, and vestibular areflexia syndrome; CIDP, chronic inflammatory demyelinating polyneuropathy; CMT, Charcot-Marie-Tooth disease; GBS, Guillain-Barré syndrome; HBV, hepatitis B virus; HCV, hepatitis C virus; HNPP, hereditary neuropathy with liability to pressure palsies; MGUS, monoclonal gammopathy of undetermined significance; MMN, multifocal motor neuropathy; POEMS, polyneuropathy, organomegaly, endocrinopathy, monoclonal gammopathy, and skin changes syndrome; TTR-Amyloidosis, transthyretin amyloidosis-associated neuropathy;

N°	Case presentation and Diagnosis	Diagnosis	Prompt 2 output
1	**Case presentation:** A 32-year old male patient presents with a 26-year history of sensory-motor, symmetrical, length dependent polyneuropathy with insidious onset and slow progression. The patient presents with scoliosis, neuropathic pain, sensory ataxia, pes cavus, distal weakness in the four limbs, severe calf atrophy with no cranial nerve or autonomic involvement. Past medical history is silent. Nerve conduction studies show an axonal, length dependent, symmetric, sensory-motor polyneuropathy. Screening lab tests including vitamin B12, folate, vitamin B6, vitamin E, HBV, HCV, glycemia, HbA1c, ANA, ANCA, ENA, CRP, ESR, copper, cryoglobulins, and serum electrophoresis are all negative. Both her father and grandfather have foot deformities and walking difficulties.	CMT2	**Step-by-Step Analysis**<br>**Summary of Pertinent Clinical Information**<br>● **Patient Profile:**<br>○ **Age/Gender: 32-year-old male**<br>○ **Presentation Duration: 26-year history**<br>○ **Symptoms: Sensory-motor, symmetrical, length-dependent polyneuropathy with insidious onset and slow progression**<br>○ **Additional Findings:**<br>■ **Scoliosis**<br>■ **Neuropathic pain**<br>■ **Sensory ataxia**<br>■ **Pes cavus (high-arched feet)**<br>■ **Distal weakness in all four limbs**<br>■ **Severe calf atrophy**<br>■ **No cranial nerve or autonomic involvement**<br>● **Past Medical History: Unremarkable**<br>● **Investigations:**<br>○ **Nerve Conduction Studies: Axonal, length-dependent, symmetric, sensory-motor polyneuropathy**<br>○ **Laboratory Tests: Negative for vitamin B12, folate, vitamin B6, vitamin E, HBV, HCV, glycemia, HbA1c, ANA, ANCA, ENA, CRP, ESR, copper, cryoglobulins, serum electrophoresis**<br>● **Family History:**<br>○ **Father and Grandfather: Both have foot deformities and walking difficulties**<br>**Additional Examinations**<br>**1. Genetic Testing**<br>● **Reasoning:**<br>○ **Given the hereditary nature suggested by the family history and the specific clinical presentation (e.g., pes cavus, scoliosis, and neuropathy), genetic testing for known mutations associated with Charcot-Marie-Tooth disease (CMT) is warranted. Common genes include PMP22, MPZ, GJB1, and MFN2.**<br>○ **Genetic testing can definitively diagnose or rule out CMT and distinguish between types (e.g., CMT1 vs. CMT2).**<br>**2. MRI of the Spine**<br>● **Reasoning:**<br>○ **To assess the degree of scoliosis and evaluate any spinal cord or nerve root involvement that might contribute to neuropathic symptoms.**<br>○ **MRI can provide detailed imaging of soft tissue structures and help rule out other potential causes of neuropathy such as spinal stenosis or tumors.**<br>**3. Electromyography (EMG)**<br>● **Reasoning:**<br>○ **To complement the nerve conduction studies by assessing the electrical activity of muscles, which can help differentiate between neurogenic and myogenic causes of muscle weakness.**<br>○ **EMG can provide additional information about the severity and distribution of the neuropathy.**<br>**4. Detailed Family History and Pedigree Analysis**<br>● **Reasoning:**<br>○ **To further delineate the inheritance pattern and identify other potentially affected family members.**<br>○ **This can provide clues about the type of hereditary neuropathy and guide genetic testing.**<br>**5. Skin Biopsy for Intraepidermal Nerve Fiber Density (IENFD)**<br>● **Reasoning:**<br>○ **This test can help assess the density of small nerve fibers, which is particularly useful in cases of length-dependent neuropathy.**<br>○ **It can confirm the presence of a neuropathy even when nerve conduction studies are inconclusive.**<br>**6. Repeat Comprehensive Laboratory Panel**<br>● **Reasoning:**<br>○ **Although the initial screening was extensive, a repeat or extended panel could include less common causes of neuropathy.**<br>○ **Tests might include paraneoplastic antibodies, Lyme disease serology, HIV testing, and a more comprehensive toxicology screen.**<br>**Most Crucial Test**<br>**Genetic Testing for CMT is the most crucial test. It has the potential to confirm the diagnosis, differentiate between types, and provide definitive evidence of a hereditary neuropathy, which aligns closely with the patient's clinical presentation and family history.**<br>**Conclusion**<br>**In conclusion, while several additional examinations can provide valuable information, genetic testing stands out as the most critical due to its ability to confirm the diagnosis of Charcot-Marie-Tooth disease, which is highly suspected in this patient based on the clinical features and family history**
2	**Case presentation:** An 88-year-old male patient presents with a 19-month history of pure sensory, symmetrical, length dependent polyneuropathy with insidious	HCV-associated neuropathy	**Step-by-Step Analysis and Additional Examinations**<br>**Given the patient's clinical presentation and history, further tests are necessary to narrow down the differential diagnoses. Here are the recommended additional examinations, along with the reasoning for each:**<br>**1. HCV RNA Quantitative Test**<br>● **Reasoning:**

	onset and slow progressive course. The patient presents with hypoesthesia, paresthesia, and cramps in the lower limbs in a stocking distribution. Clinical examination does not reveal weakness or sensory ataxia, neuropathic pain, tremor, foot deformities, cranial nerve or autonomic involvement. Past medical history is positive for colorectal cancer, for which the patient underwent chemotherapy 5 years ago, and chronic HCV infection. Nerve conduction studies show a pure sensory, symmetric, length-dependent axonal polyneuropathy with unilateral carpal tunnel syndrome. Screening lab tests including vitamin B12, folate, vitamin B6, vitamin E, HBV, glycemia, HbA1c, ANA, ANCA, ENA, CRP, ESR, copper, cryoglobulins, and serum electrophoresis are normal, with positivity for HCV IgG. There is no family history of polyneuropathy.		○ **Although the patient is positive for HCV IgG, confirming active HCV replication is crucial. The HCV RNA test will provide information on the viral load, indicating whether the virus is actively replicating and possibly contributing to neuropathy.**<br>○ **This test is especially important if antiviral treatment is being considered.**<br>**2. Liver Function Tests (LFTs)**<br>● **Reasoning:**<br>○ **Chronic HCV infection can lead to liver dysfunction, which may have systemic effects contributing to neuropathy.**<br>○ **Abnormal liver function tests might support the hypothesis that HCV is causing systemic effects, including peripheral neuropathy.**<br>**3. Skin Biopsy**<br>● **Reasoning:**<br>○ **A skin biopsy can be used to assess small fiber neuropathy, which might not be detected on routine nerve conduction studies.**<br>○ **This test can reveal intraepidermal nerve fiber density, providing insight into sensory nerve involvement, which could be linked to HCV or another etiology.**<br>**4. Antineuronal Antibody Panel**<br>● **Reasoning:**<br>○ **Screening for paraneoplastic syndromes, particularly given the patient's history of colorectal cancer, is essential. This panel can detect antibodies associated with paraneoplastic neurological syndromes.**<br>○ **Although less likely due to the long interval since cancer treatment, it remains a consideration.**<br>**5. Cerebrospinal Fluid (CSF) Analysis**<br>● **Reasoning:**<br>○ **Lumbar puncture and CSF analysis can help rule out central nervous system involvement and infections or inflammatory causes of neuropathy.**<br>○ **Elevated protein levels without pleocytosis in CSF can support a diagnosis of chronic inflammatory demyelinating polyneuropathy (CIDP), which can sometimes present with sensory-predominant symptoms.**<br>**6. Genetic Testing for Hereditary Neuropathies**<br>● **Reasoning:**<br>○ **Despite no family history, late-onset hereditary neuropathies can present in older adults. Genetic testing can identify mutations associated with hereditary neuropathies, such as transthyretin amyloidosis or other genetic causes.**<br>○ **This is particularly relevant if other common causes are ruled out.**<br>**7. Electromyography (EMG)**<br>● **Reasoning:**<br>○ **Although nerve conduction studies have been performed, EMG can provide additional information on muscle involvement and help distinguish between axonal and demyelinating processes.**<br>○ **This can further clarify the nature of the neuropathy and rule out subclinical motor involvement.**<br>**Most Crucial Test**<br>**HCV RNA Quantitative Test is the most crucial test from the list.**<br>**Reasoning:**<br>● **Confirming active HCV replication is essential in establishing a direct link between the chronic HCV infection and the patient's neuropathy. The presence of active viral replication would strongly suggest that the HCV infection is contributing to the neuropathy.**<br>● **This information is vital for determining the need for antiviral therapy, which could potentially alleviate the neuropathy if the HCV infection is treated effectively.**<br>● **Given the patient's positive HCV IgG and the potential for HCV to cause neuropathy, confirming the viral load is a key step in the diagnostic process.**<br>**Conducting this test will provide a clear direction for further management and potential treatment options, making it the most critical next step in the evaluation process.**
3	**Case presentation:** A 75-year-old female patient presents with a 1-year history of sensory-motor, symmetrical, length dependent polyneuropathy with subacute onset. The patient presents with paresthesia and hypoesthesia in the lower limbs and sensory ataxia, without tremor, foot deformities, neuropathic pain, cranial nerve or autonomic involvement. Past medical history shows breast cancer, for which the patient has undergone chemotherapy. Neuropathy symptoms started during chemotherapy. Nerve conduction studies show a symmetric, sensory-motor, axonal polyneuropathy. Screening lab tests including vitamin B12, folate, vitamin B6, vitamin E, HCV, HBV, ANA, ANCA, ENA, CRP, ESR, glycemia, HbA1c, copper, cryoglobulins and serum electrophoresis are all negative. There is no family history of polyneuropathy.	Chemotherapy induced polyneuropathy	**Additional Examinations: Detailed Analysis and Reasoning**<br>**Given the comprehensive data provided, additional examinations can further narrow down the differential diagnoses for this patient's polyneuropathy. Here is a step-by-step approach to the necessary additional tests:**<br>**1. Detailed Chemotherapy Regimen Review:**<br>○ **Reasoning: Identifying the specific chemotherapy agents used will help determine if they are known to cause peripheral neuropathy. This can strengthen the diagnosis of CIPN.**<br>○ **Specifics: Taxanes, platinum compounds, and vinca alkaloids are commonly associated with neuropathy.**<br>**2. Skin Biopsy for Intraepidermal Nerve Fiber Density (IENFD):**<br>○ **Reasoning: This test can detect small fiber neuropathy, which might not show up on nerve conduction studies.**<br>○ **Specifics: A reduced density of intraepidermal nerve fibers can confirm small fiber involvement, supporting a diagnosis of chemotherapy-induced neuropathy if large fiber involvement is also present.**<br>**3. CSF Analysis:**<br>○ **Reasoning: While less likely, paraneoplastic syndromes or other immune-mediated neuropathies could present with inflammatory markers in the CSF.**<br>○ **Specifics: Analysis for elevated protein, presence of malignant cells, or oligoclonal bands can help exclude or confirm these conditions.**<br>**4. Genetic Testing:**<br>○ **Reasoning: Although the patient has no family history, late-onset genetic neuropathies can occur, especially in elderly patients.**<br>○ **Specifics: Tests for mutations associated with Charcot-Marie-Tooth disease or other hereditary neuropathies.**<br>**5. Repeat Nerve Conduction Studies and Electromyography (EMG):**<br>○ **Reasoning: To track the progression and characteristics of the neuropathy over time, and to ensure no new findings develop.**<br>○ **Specifics: Reassessment can provide information on whether the neuropathy is worsening or stabilizing, aiding in the management plan.**<br>**6. Heavy Metal Screening (Lead, Arsenic, Mercury, and Thallium):**<br>○ **Reasoning: Although not directly suggested by the initial workup, chronic exposure to heavy metals can cause axonal polyneuropathy.**<br>○ **Specifics: Blood and urine tests for these metals can rule out toxic neuropathies.**<br>**7. Autonomic Function Tests:**<br>○ **Reasoning: To definitively rule out subtle autonomic involvement that the patient may not have noticed.**<br>○ **Specifics: Tests like heart rate variability, sweat testing, and tilt-table test can detect autonomic dysfunction.**<br>**8. MRI of the Spine:**<br>○ **Reasoning: To exclude compressive myelopathies or other spinal cord pathologies that might contribute to the patient's symptoms.**<br>○ **Specifics: An MRI can visualize structural abnormalities that could explain the symptoms if present.**<br>**Most Crucial Test: Detailed Chemotherapy Regimen Review**

			**Reasoning:**<br>● **Understanding the specific agents used in chemotherapy and their timelines will directly support or challenge the diagnosis of chemotherapy-induced peripheral neuropathy (CIPN).**<br>● **This review can identify known neurotoxic agents and correlate them with the onset and progression of the neuropathy symptoms.**<br>● **It is a non-invasive, cost-effective approach that can provide definitive information about the likely etiology of the patient's condition.**<br>**By confirming the specific chemotherapeutic agents used, and their known neurotoxic profiles, the likelihood of CIPN can be assessed more accurately. This test will guide the necessity and urgency of other more invasive or expensive tests.**
4	**Case presentation:** An 81-year-old male patient presents with a 9-week history of sensory-motor, asymmetrical multineuropathy with subacute onset and progressive course. The patient presents with paresthesia, hypoesthesia, neuropathic pain, and weakness with a multifocal distribution. Clinical examination does not reveal sensory ataxia nor tremor, foot deformities, cranial nerve or autonomic involvement. Past medical history is positive for giant cell arteritis. Nerve conduction studies show a sensory-motor, axonal multineuropathy. Screening lab tests including vitamin B12, folate, vitamin B6, vitamin E, HBV, HCV, glycemia, HbA1c, ANA, ENA, copper, cryoglobulins, and serum electrophoresis are all negative, with evidence of elevated CRP and ESR. There is no family history of polyneuropathy.	Vasculitic neuropathy	**Step-by-Step Analysis and Additional Examinations**<br>**Step 1: Review of Current Information**<br>● **Patient Details: 81-year-old male**<br>● **Symptom History: 9-week history of sensory-motor, asymmetrical multineuropathy**<br>● **Symptoms: Paresthesia, hypoesthesia, neuropathic pain, weakness**<br>● **Distribution: Multifocal**<br>● **Clinical Examination: No sensory ataxia, tremor, foot deformities, cranial nerve, or autonomic involvement**<br>● **Past Medical History: Giant cell arteritis**<br>● **Nerve Conduction Studies: Sensory-motor, axonal multineuropathy**<br>● **Screening Lab Tests: Negative for B12, folate, B6, vitamin E, HBV, HCV, glycemia, HbA1c, ANA, ENA, copper, cryoglobulins, serum electrophoresis**<br>● **Inflammatory Markers: Elevated CRP and ESR**<br>● **Family History: No family history of polyneuropathy**<br>**Step 2: Identifying Additional Examinations**<br>1. **Nerve Biopsy**<br>○ **Reasoning: A nerve biopsy, particularly of a sensory nerve, can provide direct evidence of vasculitic changes, including necrotizing vasculitis and inflammatory infiltrates. This is crucial for diagnosing vasculitic neuropathy.**<br>○ **Details: A sural nerve biopsy would be appropriate in this case.**<br>2. **MRI of the Peripheral Nerves (Neurography)**<br>○ **Reasoning: MRI can help visualize nerve inflammation, enlargement, or structural abnormalities, which can be indicative of vasculitic neuropathy or other inflammatory neuropathies like CIDP.**<br>○ **Details: Imaging of the affected limbs and nerve plexuses can provide detailed structural information.**<br>3. **CSF Analysis (Lumbar Puncture)**<br>○ **Reasoning: Cerebrospinal fluid (CSF) analysis can reveal elevated protein levels without pleocytosis, which is suggestive of CIDP. Inflammatory markers or atypical cells may suggest an underlying inflammatory or infectious process.**<br>○ **Details: Analysis should include cell count, protein, glucose, oligoclonal bands, and cytology.**<br>4. **Repeat Serum Electrophoresis and Immunofixation Electrophoresis**<br>○ **Reasoning: To re-evaluate for the presence of monoclonal gammopathy, which may have been missed initially. Even small amounts of monoclonal protein can be clinically significant.**<br>○ **Details: This test would help identify MGUS or multiple myeloma if present.**<br>5. **PET-CT Scan**<br>○ **Reasoning: To detect systemic involvement of vasculitis and assess for large vessel involvement associated with giant cell arteritis. It can also identify other potential malignancies.**<br>○ **Details: A whole-body PET-CT can reveal metabolic activity suggestive of active vasculitis or malignancy.**<br>6. **Anti-Neutrophil Cytoplasmic Antibodies (ANCA)**<br>○ **Reasoning: While not specific for giant cell arteritis, ANCA can help rule out other vasculitic syndromes like granulomatosis with polyangiitis (Wegener's) which can also present with neuropathy.**<br>○ **Details: Testing for c-ANCA and p-ANCA should be performed.**<br>7. **Detailed Rheumatologic Panel**<br>○ **Reasoning: Further rheumatologic tests including anti-dsDNA, anti-RNP, anti-Smith, and anti-cardiolipin antibodies can help identify other systemic autoimmune conditions that may present with neuropathy.**<br>○ **Details: Comprehensive testing for other potential autoimmune causes.**<br>**Step 3: Most Crucial Test**<br>**Nerve Biopsy**<br>● **Reasoning: A nerve biopsy provides direct pathological evidence of vasculitis affecting the peripheral nerves, which is crucial in confirming a diagnosis of vasculitic neuropathy. Given the patient's history of giant cell arteritis and the clinical presentation of multifocal neuropathy, this test is paramount in establishing the presence of vasculitic changes and guiding appropriate treatment.**<br>**Conclusion**<br>**A nerve biopsy is the most crucial test as it provides definitive pathological evidence to confirm or rule out vasculitic neuropathy, especially in a patient with a known history of giant cell arteritis and a clinical picture suggestive of an inflammatory neuropathy.**
5	A 61-year-old male patient presents with a 1-year history of sensory-motor, asymmetric, non-length dependent polyneuropathy with subacute onset and progressive course. The patient presents with asymmetric distal paresthesia and neuropathic pain distributed in the four limbs, associated with weakness in the four limbs both proximally and distally. Clinical examination also shows the presence of mild sensory ataxia, peripheral edema in the feet, and mild cutaneous hyperpigmentation in the lower limbs.	POEMS	**Step-by-Step Analysis and Additional Examinations**<br>**1. Detailed Immunological Workup**<br>● **Serum Immunofixation Electrophoresis (IFE):**<br>○ **Reasoning: To precisely characterize the paraprotein detected (IgG lambda). IFE is more sensitive than serum protein electrophoresis and can help in diagnosing monoclonal gammopathies.**<br>● **Free Light Chain Assay:**<br>○ **Reasoning: To quantify free light chains in the serum, which can be elevated in conditions like multiple myeloma, MGUS, or other plasma cell dyscrasias. This test is particularly useful for detecting light chain myeloma.**<br>**2. Neurological and CSF Studies**<br>● **Lumbar Puncture with Cerebrospinal Fluid (CSF) Analysis:**<br>○ **Reasoning: To look for elevated protein levels without pleocytosis, which is typical in CIDP. CSF analysis can also rule out other infectious or inflammatory conditions affecting the nervous system.**<br>● **MRI of the Spine and Nerve Roots:**<br>○ **Reasoning: To detect nerve root hypertrophy or other structural abnormalities. This imaging can support the diagnosis of CIDP by showing hypertrophic nerves or roots.**<br>**3. Detailed Nerve Studies**

	There are no autonomic symptoms or foot deformities. Past medical history is positive for psoriatic arthritis. Nerve conduction studies show a demyelinating sensory-motor polyneuropathy. Screening lab tests show presence of IgG lambda paraprotein and elevated CRP. Remaining exams, including vitamin B12, folate, vitamin B6, vitamin E, HBV, HCV, ANA, ENA, ANCA, ESR, glycemia, HbA1c, copper, and cryoglobulins are negative. There is no family history of polyneuropathy.		**● Repeat Nerve Conduction Studies (NCS) and Electromyography (EMG):**<br>○ **Reasoning: To confirm and further detail the extent and nature of the demyelinating process. Repeated studies can also help in monitoring disease progression or response to therapy.**<br>**● Nerve Biopsy (if needed):**<br>○ **Reasoning: In cases where diagnosis remains uncertain, a nerve biopsy can provide histological evidence of demyelination, inflammation, or amyloid deposits.**<br>**4. Hematological Evaluation**<br>**● Bone Marrow Biopsy:**<br>○ **Reasoning: To rule out multiple myeloma or other hematologic malignancies, given the presence of IgG lambda paraprotein. This can confirm or exclude conditions like multiple myeloma, amyloidosis, or other plasma cell dyscrasias.**<br>**5. Comprehensive Inflammatory and Autoimmune Panel**<br>**● Anti-MAG Antibodies:**<br>○ **Reasoning: To test for myelin-associated glycoprotein antibodies, which can be associated with certain types of demyelinating neuropathies, particularly those associated with paraproteinemias.**<br>**● ESR and C-reactive protein (CRP):**<br>○ **Reasoning: Rechecking these inflammatory markers to monitor the inflammatory status, as initial results showed elevated CRP.**<br>**6. Systemic Involvement Tests**<br>**● Echocardiogram:**<br>○ **Reasoning: To check for cardiac involvement in amyloidosis, especially if amyloidosis is suspected from biopsy results or other tests.**<br>**Most Crucial Test**<br>**Based on the patient's presentation and the need to narrow down the differential diagnoses, Serum Immunofixation Electrophoresis (IFE) emerges as the most crucial test. This test will precisely characterize the IgG lambda paraprotein detected in initial screening. Determining the exact nature and quantity of the paraprotein is essential for diagnosing conditions like MGUS, multiple myeloma, or related disorders, which can have a significant impact on the management and prognosis of the patient. Furthermore, the results of this test will guide the need for further hematological investigations, such as a bone marrow biopsy, making it a critical step in the diagnostic process.**
6	A 73-year-old female patient presents with an 8-month history of pure sensory, symmetrical, length dependent polyneuropathy with subacute onset and progressive course. The patient presents with distal paresthesia in the upper and lower limbs without weakness or neuropathic pain. Clinical examination reveals reduced tactile sensation in the feet. There are no tremor or ataxia, foot deformities, cranial nerve or autonomic involvement. Past medical history is positive for episodic diarrhea. Nerve conduction studies show a pure sensory, symmetric, length dependent, axonal polyneuropathy. Screening lab tests reveal the presence of vitamin B12 deficiency, hyperglycaemia, and elevated HbA1c. Remaining exams including folate, vitamin B6, vitamin E, HCV, HBV, ANA, ENA, ANCA, CRP, ESR, copper, cryoglobulins and, serum electrophoresis are negative. There is no family history of polyneuropathy.	Vit B12 deficiency neuropathy	**Step-by-Step Analysis and Additional Examinations**<br>**Step 1: Review Current Data**<br>**Clinical Presentation:**<br>**● Symptoms: Pure sensory, symmetrical, length-dependent polyneuropathy with distal paresthesia in the upper and lower limbs, no weakness, neuropathic pain, tremor, ataxia, foot deformities, cranial nerve, or autonomic involvement.**<br>**● History: Episodic diarrhea, vitamin B12 deficiency, hyperglycemia, elevated HbA1c.**<br>**● Nerve Conduction Studies: Pure sensory, symmetric, length-dependent, axonal polyneuropathy.**<br>**● Laboratory Tests: Negative for folate, vitamin B6, vitamin E, HCV, HBV, ANA, ENA, ANCA, CRP, ESR, copper, cryoglobulins, and serum electrophoresis.**<br>**● Family History: No family history of polyneuropathy.**<br>**Step 2: Additional Examinations**<br>**1. Hemoglobin A1c (HbA1c) Repeat and Fasting Blood Glucose:**<br>**● Reasoning: Confirm the diagnosis of diabetes or prediabetes. Elevated HbA1c and hyperglycemia need to be reconfirmed to ensure that diabetic neuropathy is indeed the most likely cause.**<br>**● Test Description: Measure HbA1c to assess long-term glucose control and fasting blood glucose levels to confirm current glucose status.**<br>**2. Oral Glucose Tolerance Test (OGTT):**<br>**● Reasoning: If diabetes is suspected but not confirmed by HbA1c and fasting glucose, an OGTT can help diagnose impaired glucose tolerance or diabetes.**<br>**● Test Description: Patient drinks a glucose solution, and blood glucose levels are measured at intervals to assess glucose metabolism.**<br>**3. Comprehensive Metabolic Panel (CMP):**<br>**● Reasoning: To check liver and kidney function, electrolytes, and overall metabolic status, which can influence neuropathy.**<br>**● Test Description: Blood test measuring glucose, calcium, proteins, electrolytes, and liver and kidney function.**<br>**4. Serum Immunofixation Electrophoresis:**<br>**● Reasoning: Although the initial serum electrophoresis was negative, immunofixation can detect monoclonal gammopathies more sensitively. Monoclonal gammopathies can cause neuropathy.**<br>**● Test Description: Blood test to detect monoclonal proteins (M-proteins).**<br>**5. Anti-Ganglioside Antibodies (GM1, GQ1b, GD1a, GD1b):**<br>**● Reasoning: To evaluate for immune-mediated neuropathies, such as chronic inflammatory demyelinating polyneuropathy (CIDP) or multifocal motor neuropathy (MMN), which may present with pure sensory symptoms in early stages.**<br>**● Test Description: Blood test to detect specific antibodies associated with autoimmune neuropathies.**<br>**6. Lumbar Puncture with Cerebrospinal Fluid (CSF) Analysis:**<br>**● Reasoning: To assess for infections, inflammatory conditions, or malignancies affecting the nervous system.**<br>**● Test Description: CSF analysis for cell count, protein, glucose, and specific biomarkers or infectious agents.**<br>**7. MRI of the Spine:**<br>**● Reasoning: To exclude structural causes such as spinal stenosis or tumors that might present with similar symptoms.**<br>**● Test Description: Imaging study to visualize spinal structures and potential compression or lesions.**<br>**8. Gastrointestinal Workup:**<br>**● Reasoning: Given the history of episodic diarrhea, assess for malabsorption syndromes or celiac disease, which can cause vitamin deficiencies leading to neuropathy.**<br>**● Test Description: Endoscopy, biopsy, and specific tests for celiac disease (tissue transglutaminase antibodies).**<br>**9. Repeat Vitamin and Mineral Levels:**<br>**● Reasoning: To ensure no other deficiencies (such as zinc or copper) are contributing to the neuropathy.**<br>**● Test Description: Blood tests for comprehensive vitamin and mineral levels.**<br>**Step 3: Determining the Most Crucial Test**<br>**Most Crucial Test: Hemoglobin A1c (HbA1c) Repeat and Fasting Blood Glucose**<br>**Reasoning:**<br>**● Primary Importance: Given the initial findings of hyperglycemia and elevated HbA1c, confirming diabetes or prediabetes is critical. Diabetic neuropathy is a common cause of polyneuropathy, and appropriate management of blood glucose levels can significantly impact the patient's symptoms and overall prognosis.**

			● **Immediate Impact: A definitive diagnosis of diabetes will direct immediate and specific management strategies, potentially improving symptoms and preventing further progression of neuropathy.**<br>● **Correlation with Symptoms: The clinical presentation aligns well with diabetic neuropathy, making this the most logical next step to confirm the diagnosis and guide treatment.**<br>**Conclusion**<br>**The most crucial test to perform is a repeat Hemoglobin A1c (HbA1c) and fasting blood glucose to confirm the diagnosis of diabetes. This will provide a definitive basis for diagnosing diabetic neuropathy and guide further management and treatment plans for the patient.**
7	A 70-year-old male patient presents with a 5-week history of pure sensory, symmetrical, length dependent polyneuropathy with subacute onset and progressive course. The patient presents with distal paresthesia and hypoesthesia in the four limbs and sensory ataxia. Clinical examination does not show weakness, tremor, foot deformities, autonomic or cranial nerve involvement. Past medical history is positive for obesity treated with bariatric surgery, anemia, hypercholesterolemia, and type 2 diabetes mellitus. Nerve conduction studies show a pure sensory, symmetric, axonal polyneuropathy. Screening lab tests shows slightly elevated glycemia and HbA1c levels, and severe folate deficiency. Remaining exams including vitamin B12, E, HBV, HCV, ANA, ANCA, ENA, CRP, ESR, copper, cryoglobulins, and serum electrophoresis are normal. There is no family history of polyneuropathy.	Folate deficiency neuropathy	**Step-by-Step Analysis**<br>**1. Review of Clinical Data**<br>○ **Patient's history: 70-year-old male, sensory, symmetrical, length-dependent polyneuropathy.**<br>○ **Symptoms: Distal paresthesia, hypoesthesia, sensory ataxia.**<br>○ **Examination: No motor involvement, autonomic or cranial nerve symptoms.**<br>○ **Past medical history: Bariatric surgery, anemia, hypercholesterolemia, type 2 diabetes mellitus.**<br>○ **Nerve conduction studies: Pure sensory, symmetric, axonal polyneuropathy.**<br>○ **Lab results: Slightly elevated glycemia and HbA1c, severe folate deficiency, normal vitamin B12, E, HBV, HCV, ANA, ANCA, ENA, CRP, ESR, copper, cryoglobulins, serum electrophoresis.**<br>○ **No family history of polyneuropathy.**<br>**2. Initial Differential Diagnoses**<br>○ **Diabetic Polyneuropathy**<br>○ **Folate Deficiency Neuropathy**<br>○ **Idiopathic Axonal Polyneuropathy**<br>**Additional Examinations**<br>**1. Serum Methylmalonic Acid (MMA) and Homocysteine Levels**<br>○ **Reasoning: To further assess the metabolic status related to vitamin deficiencies. Elevated MMA and homocysteine are indicative of functional vitamin B12 deficiency even when serum B12 levels are normal.**<br>○ **Case Specifics: Although vitamin B12 levels are normal, the severe folate deficiency and history of bariatric surgery increase the risk for mixed deficiencies.**<br>**2. Oral Glucose Tolerance Test (OGTT)**<br>○ **Reasoning: To evaluate glucose metabolism more comprehensively. This test can help detect glucose intolerance or postprandial hyperglycemia not evident in fasting glucose or HbA1c levels.**<br>○ **Case Specifics: The patient's history of diabetes and slightly elevated glycemia and HbA1c warrant a more detailed assessment of glycemic control.**<br>**3. Comprehensive Metabolic Panel (CMP)**<br>○ **Reasoning: To check for any metabolic imbalances, including liver and kidney function, which can contribute to neuropathy.**<br>○ **Case Specifics: The patient has a history of multiple metabolic conditions, making it crucial to rule out any contributing metabolic disturbances.**<br>**4. Quantitative Sensory Testing (QST)**<br>○ **Reasoning: To quantify the extent of sensory dysfunction and differentiate between small and large fiber involvement.**<br>○ **Case Specifics: The patient presents with sensory ataxia and distal hypoesthesia, and QST can provide more detailed information on sensory nerve function.**<br>**5. Skin Biopsy for Intraepidermal Nerve Fiber Density (IENFD)**<br>○ **Reasoning: To assess small fiber neuropathy, which might not be evident in standard nerve conduction studies.**<br>○ **Case Specifics: Pure sensory symptoms suggest possible involvement of small fibers, especially in a setting of diabetes and nutritional deficiencies.**<br>**6. Lumbar Puncture for Cerebrospinal Fluid (CSF) Analysis**<br>○ **Reasoning: To rule out any inflammatory or infectious causes of neuropathy.**<br>○ **Case Specifics: Subacute onset and progressive nature of symptoms require exclusion of inflammatory neuropathies, such as chronic inflammatory demyelinating polyneuropathy (CIDP).**<br>**Most Crucial Test**<br>**Serum Methylmalonic Acid (MMA) and Homocysteine Levels**<br>● **Reasoning: This test is the most crucial because it helps clarify the functional status of vitamin B12 and folate metabolism, which is directly relevant given the patient's severe folate deficiency and history of bariatric surgery. Elevated levels would confirm a functional deficiency contributing to the neuropathy, even if serum B12 appears normal. This would significantly influence the treatment plan and prognosis.**
8	A 74-year-old male patient presents with a 9-month history of pure sensory, symmetrical, length dependent polyneuropathy with subacute onset while hospitalised for lymphoma. The patient developed neuropathic pain in the feet with no sensory deficits or weakness. Clinical examination reveals absence of ataxia, tremor, foot deformities, cranial nerve or autonomic involvement. Past medical history is positive for diabetes since the age of 43, and lymphoma for which the patient has undergone chemotherapy. Neuropathy symptoms started during chemotherapy. Nerve conduction studies show a sensory-motor, symmetric, axonal polyneuropathy. Screening lab tests including vitamin B12, folate, vitamin B6, vitamin E, HCV, ANA, ANCA, ENA, CRP, ESR, copper, cryoglobulins and serum electrophoresis are all negative. HbA1c and glycemia levels	Chemotherapy-induced neuropathy	**Step-by-Step Approach for Additional Examinations**<br>To further narrow down the differential diagnoses of polyneuropathy in this patient, several additional tests should be conducted. These tests aim to provide more detailed information on potential underlying causes and to rule out other contributing factors.<br>**1. Detailed Blood Tests**<br>● **Reasoning**: While initial screening tests were negative, further detailed blood tests can provide more insights into metabolic and nutritional deficiencies, infections, and immune responses.<br>● **Tests**:<br>○ **HbA1c and Fasting Blood Glucose**: To confirm the extent of glycemic control, which could further support the diagnosis of diabetic neuropathy.<br>○ **Liver Function Tests (LFTs)**: To assess liver function and rule out hepatic causes of neuropathy, especially given the chronic HBV infection.<br>○ **Kidney Function Tests (KFTs)**: To evaluate renal function, as chronic kidney disease can also lead to neuropathy.<br>○ **Serum Protein Electrophoresis and Immunofixation**: To rule out monoclonal gammopathy, which can be associated with neuropathy.<br>**2. Additional Vitamin and Mineral Levels**<br>● **Reasoning**: Some vitamin deficiencies, despite the initial negative screening, can still play a role in neuropathy.<br>● **Tests**:<br>○ **Vitamin D**: Deficiency can contribute to neuropathy symptoms.<br>○ **Magnesium and Zinc Levels**: Deficiencies can cause or exacerbate neuropathy.<br>**3. Imaging Studies**<br>● **Reasoning**: Imaging can help identify structural or compressive causes of neuropathy and assess any related complications from underlying conditions like lymphoma.<br>● **Tests**:<br>○ **MRI of the Spine**: To rule out compressive neuropathies, especially if there is suspicion of spinal involvement or paraneoplastic syndromes.<br>○ **Ultrasound of the Liver**: To evaluate for any structural changes due to chronic HBV infection that might be indirectly affecting neuropathy.<br>**4. Advanced Electrophysiological Studies**<br>● **Reasoning**: Detailed nerve conduction studies can provide more specific information about the type and extent of neuropathy.

	are elevated and there is evidence of chronic HBV infection. There is no family history of polyneuropathy.		● **Tests**:<br>○ **Quantitative Sensory Testing (QST)**: To assess sensory nerve function in more detail.<br>○ **Electromyography (EMG)**: To further evaluate the extent of motor involvement and distinguish between different types of neuropathy.<br>**5. Autoimmune and Paraneoplastic Panel**<br>● **Reasoning**: Given the history of lymphoma, it is important to rule out paraneoplastic neuropathy, which can present similarly.<br>● **Tests**:<br>○ **Paraneoplastic Antibody Panel**: To detect antibodies associated with paraneoplastic syndromes.<br>○ **Comprehensive Autoimmune Panel**: Despite initial negative ANA, further detailed testing can identify other autoimmune neuropathies.<br>**6. Biopsy**<br>● **Reasoning**: Invasive but definitive tests like biopsies can provide conclusive evidence of specific types of neuropathy.<br>● **Tests**:<br>○ **Nerve Biopsy**: To look for specific pathological changes in peripheral nerves.<br>○ **Skin Biopsy**: To evaluate small fiber neuropathy, which might not be detected by nerve conduction studies.<br>**Most Crucial Test**<br>**Comprehensive Autoimmune and Paraneoplastic Panel**<br>● **Conclusion**: Given the patient's history of lymphoma and the temporal association of neuropathy onset with chemotherapy, the most crucial test would be the comprehensive autoimmune and paraneoplastic panel. This test can identify underlying paraneoplastic syndromes or autoimmune processes that might be contributing to the neuropathy, which is critical in a patient with a history of cancer. This information would directly impact the management and treatment approach for the patient's neuropathy.
9	A 77-year-old female patient presents with a 3-week history of pure sensory, symmetrical, length dependent polyneuropathy with subacute onset and progressive course. The patient presents with hypoesthesia and mild neuropathic pain in the feet. Clinical examination reveals also mild sensory ataxia. There are no tremor, foot deformities, cranial nerve or autonomic involvement. Past medical history is negative. Nerve conduction studies show a pure sensory, length dependent, symmetric, axonal polyneuropathy. Screening lab tests reveal decreased serum level of vitamin B6 and a slight increase in transaminase levels (aspartate aminotransferase/alanine aminotransferase). Remaining exams including vitamin B12, folate, vitamin E, HBV, HCV, glycemia, HbA1c, ANA, ANCA, ENA, CRP, ESR, copper, cryoglobulins, and serum electrophoresis are negative. There is no family history of polyneuropathy.	B6 deficiency neuropathy	**Step-by-Step Approach to Additional Examinations**<br>**Step 1: Review Initial Findings**<br>**Clinical Presentation:**<br>● 3-week history of pure sensory, symmetrical, length-dependent polyneuropathy<br>● Mild neuropathic pain in the feet<br>● Mild sensory ataxia<br>**Initial Laboratory Results:**<br>● Decreased serum vitamin B6<br>● Slight increase in transaminase levels (AST/ALT)<br>● Negative for vitamin B12, folate, vitamin E, HBV, HCV, glycemia, HbA1c, ANA, ANCA, ENA, CRP, ESR, copper, cryoglobulins, serum electrophoresis<br>**Step 2: Identify Additional Necessary Examinations**<br>**1. Serum Protein Electrophoresis (SPEP) and Immunofixation Electrophoresis (IFE) Reasoning:**<br>● **Relevance:** Despite the initial negative serum electrophoresis, repeat testing with immunofixation can help detect low levels of monoclonal proteins (M-protein) which might be missed initially.<br>● **Purpose:** Identify monoclonal gammopathies (e.g., MGUS, multiple myeloma) which can present with neuropathy.<br>**2. Liver Function Tests (LFTs) Reasoning:**<br>● **Relevance:** Slight increase in AST/ALT warrants further investigation.<br>● **Purpose:** Assess for more detailed liver function parameters including bilirubin, alkaline phosphatase, gamma-glutamyl transferase (GGT), and albumin to evaluate for underlying liver disease potentially contributing to neuropathy.<br>**3. Vitamin B6 Level and Functional Assessment Reasoning:**<br>● **Relevance:** Initial finding of decreased vitamin B6 needs confirmation and functional impact assessment.<br>● **Purpose:** Confirm deficiency and assess functional status (e.g., homocysteine levels, as vitamin B6 is involved in its metabolism).<br>**4. Heavy Metal Screen (e.g., Lead, Arsenic) Reasoning:**<br>● **Relevance:** Peripheral neuropathy can result from heavy metal toxicity.<br>● **Purpose:** Detect heavy metals that could cause axonal polyneuropathy.<br>**5. Thyroid Function Tests (TFTs) Reasoning:**<br>● **Relevance:** Hypothyroidism can present with sensory neuropathy.<br>● **Purpose:** Assess TSH, free T4 levels to rule out thyroid dysfunction as a contributing factor.<br>**6. Oral Glucose Tolerance Test (OGTT) Reasoning:**<br>● **Relevance:** Despite normal glycemia and HbA1c, early diabetes or glucose intolerance can still cause neuropathy.<br>● **Purpose:** Evaluate glucose metabolism more comprehensively.<br>**7. Autoimmune Panel Extension Reasoning:**<br>● **Relevance:** Further autoimmune causes could be involved despite initial negative ANA, ANCA, ENA.<br>● **Purpose:** Include anti-ganglioside antibodies (e.g., anti-GM1), paraneoplastic panels which can be associated with neuropathies.<br>**8. Nerve Biopsy Reasoning:**<br>● **Relevance:** Can provide histological diagnosis if clinical and laboratory findings are inconclusive.<br>● **Purpose:** Identify characteristic features of specific neuropathies (e.g., vasculitis, amyloidosis).<br>**9. Electromyography (EMG) Reasoning:**<br>● **Relevance:** Complements nerve conduction studies and helps in differentiating axonal vs. demyelinating neuropathies.<br>● **Purpose:** Assess muscle response and further characterize the type of neuropathy.<br>**Step 3: Determine the Most Crucial Test**<br>**Most Crucial Test: Serum Protein Electrophoresis (SPEP) and Immunofixation Electrophoresis (IFE)**<br>● **Reasoning:** Given the patient's age and the pure sensory nature of the neuropathy, ruling out monoclonal gammopathy of undetermined significance (MGUS) or multiple myeloma is critical. These conditions can present subtly and may not be detected with initial screening. Detecting a monoclonal protein would significantly alter the management and prognosis of the patient.<br>**Conclusion**

			The most crucial additional test in this scenario is **Serum Protein Electrophoresis (SPEP) and Immunofixation Electrophoresis (IFE)**. This test is essential to rule out monoclonal gammopathies, which can present with sensory neuropathy and are more common in the elderly. Detecting such a condition would have significant implications for the patient's diagnosis and treatment plan.
10	A 76-year-old female patient presents with a 2-year history of sensory-motor, symmetrical, length dependent polyneuropathy with subacute onset. The patient presents with paresthesia, neuropathic pain, cramps, orthostatic hypotension, and weakness of toe dorsiflexion. There are no foot deformities, cranial nerve involvement, ataxia, or tremor. Past medical history is positive for chronic renal insufficiency and multiple myeloma, for which the patient has undergone chemotherapy. Neuropathy symptoms started at the end of the chemotherapy treatment cycles. Nerve conduction studies show a symmetric, pure sensory, axonal polyneuropathy and unilateral carpal tunnel syndrome. Screening lab tests including vitamin B12, folate, vitamin B6, vitamin E, HBV, HCV, ANA, ANCA, ENA, CRP, ESR, glycemia, HbA1c, copper and cryoglobulins are all negative, with positivity for IgG kappa paraprotein. There is no family history of polyneuropathy.	Chemotherapy-induced neuropathy	**Step-by-Step Analysis and Additional Examinations**<br>1. **Review of Current Data**:<br>○ Patient: 76-year-old female with sensory-motor, symmetrical, length-dependent polyneuropathy.<br>○ Symptoms: Paresthesia, neuropathic pain, cramps, orthostatic hypotension, toe dorsiflexion weakness.<br>○ Past Medical History: Chronic renal insufficiency, multiple myeloma treated with chemotherapy.<br>○ Nerve Conduction Studies: Symmetric, pure sensory, axonal polyneuropathy; unilateral carpal tunnel syndrome.<br>○ Lab Tests: Positive for IgG kappa paraprotein; other tests negative.<br>○ No family history of polyneuropathy.<br>2. **Differential Diagnoses**:<br>○ Chemotherapy-Induced Peripheral Neuropathy (CIPN)<br>○ Monoclonal Gammopathy of Undetermined Significance (MGUS)-related neuropathy<br>○ Uremic Neuropathy<br>3. **Necessary Additional Examinations**:<br>○ **Electromyography (EMG)**:<br>■ **Reasoning**: To complement nerve conduction studies by providing detailed information on muscle activity and detecting potential myopathic changes or radiculopathy. This can help distinguish between axonal versus demyelinating neuropathy patterns.<br>○ **Serum Immunofixation Electrophoresis**:<br>■ **Reasoning**: To further characterize the IgG kappa paraprotein and confirm the presence of a monoclonal protein, which can aid in diagnosing MGUS or multiple myeloma relapse.<br>○ **Quantitative Sensory Testing (QST)**:<br>■ **Reasoning**: To evaluate the functional aspects of sensory nerve fibers and assess the degree of sensory loss or hypersensitivity, providing insight into the extent of sensory neuropathy.<br>○ **Autonomic Function Testing**:<br>■ **Reasoning**: To assess the autonomic nervous system's involvement given the patient's orthostatic hypotension, which may indicate autonomic neuropathy. Tests could include heart rate variability, tilt-table testing, and sweat testing.<br>○ **Skin Biopsy for Intraepidermal Nerve Fiber Density (IENFD)**:<br>■ **Reasoning**: To quantify the small nerve fibers' density, which can help diagnose small fiber neuropathy that might not be detected in nerve conduction studies.<br>○ **Serum and Urine Free Light Chains (FLC) Assay**:<br>■ **Reasoning**: To evaluate the levels of free light chains in the serum and urine, aiding in the diagnosis and monitoring of multiple myeloma or MGUS.<br>○ **Kidney Function Tests (including detailed creatinine clearance)**:<br>■ **Reasoning**: To assess the severity of chronic renal insufficiency and its contribution to uremic neuropathy, given the patient's history of renal impairment.<br>○ **Vitamin and Mineral Levels (recheck)**:<br>■ **Reasoning**: Despite initial negative results, rechecking vitamin B12, folate, vitamin B6, and copper levels could be warranted to rule out deficiencies as they can occasionally be intermittent or low-normal.<br>○ **Magnetic Resonance Imaging (MRI) of the Spine**:<br>■ **Reasoning**: To rule out any structural abnormalities or spinal cord lesions that might contribute to the neuropathy.<br>4. **Most Crucial Test**:<br>The **Serum Immunofixation Electrophoresis** is the most crucial test. Given the positive IgG kappa paraprotein and history of multiple myeloma, confirming the presence and type of monoclonal protein is vital. This test will help differentiate between MGUS-related neuropathy and multiple myeloma relapse, significantly impacting the management plan and guiding further treatment decisions.
11	A 76-year-old female patient presents with a 1-year history of sensory-motor multineuropathy with subacute onset. The patient presents with paresthesia and weakness in a multifocal distribution, without ataxia, neuropathic pain, tremor, foot deformities, cranial nerve or autonomic involvement. Past medical history shows diabetes, anaemia, chronic renal insufficiency, undifferentiated connective tissue disease, osteoarthritis and ANCA vasculitis. Nerve conduction studies show a sensory-motor, axonal multineuropathy. Screening lab tests including vitamin B12, folate, vitamin B6, vitamin E, HCV, HBV, ENA, CRP, ESR, glycemia, HbA1c, copper, cryoglobulins and serum electrophoresis are all negative, with positivity for ANA and ANCA autoantibodies. There is no family history of polyneuropathy.	Vasculitic neuropathy	**Step-by-Step Analysis and Additional Examinations**<br>Given the clinical scenario, further diagnostic testing is necessary to narrow down the differential diagnoses. The aim is to confirm or rule out potential causes such as diabetic neuropathy, vasculitic neuropathy (ANCA-associated), and CIDP. Below are the additional examinations recommended:<br>**1. Detailed Neurological Examination**<br>● **Reasoning:** To assess the extent and distribution of sensory and motor deficits, reflexes, and any subtle signs that might have been missed. This will help in differentiating between peripheral and central causes and might reveal patterns indicative of specific neuropathies.<br>**2. Repeat and Expanded Nerve Conduction Studies (NCS) and Electromyography (EMG)**<br>● **Reasoning:** To further characterize the neuropathy (axonal vs. demyelinating), assess for temporal dispersion, conduction block, and to rule out superimposed conditions like myopathy. Expanded testing can provide a more comprehensive view of nerve involvement and progression.<br>**3. Serum and Urine Immunofixation Electrophoresis**<br>● **Reasoning:** To detect monoclonal gammopathies which can be associated with neuropathies, such as monoclonal gammopathy of undetermined significance (MGUS) or multiple myeloma.<br>**4. Lumbar Puncture and Cerebrospinal Fluid (CSF) Analysis**<br>● **Reasoning:** To assess for elevated protein levels without pleocytosis, which is typical in CIDP. This can also help rule out infectious or neoplastic causes of neuropathy.<br>**5. Skin Biopsy**<br>● **Reasoning:** To evaluate for small fiber neuropathy and possible vasculitic changes affecting small nerves and vessels. Immunohistochemistry can also help detect inflammatory infiltrates.<br>**6. Nerve Biopsy (Sural or Superficial Peroneal Nerve)**<br>● **Reasoning:** To obtain direct evidence of vasculitis or inflammatory infiltration in the peripheral nerves. This is particularly useful in suspected vasculitic neuropathy.<br>**7. Magnetic Resonance Imaging (MRI) of Nerves or Plexus**<br>● **Reasoning:** To assess for nerve root or plexus involvement, which can be seen in conditions like CIDP or vasculitis. MRI can also detect nerve enlargement or signal changes indicative of inflammation.<br>**8. Autoimmune Panel and Further Immunological Tests**<br>● **Reasoning:** To expand on the positive ANA and ANCA results and test for other autoantibodies that might be present in connective tissue diseases or autoimmune neuropathies (e.g., anti-MAG, anti-GM1 antibodies).<br>**9. Renal Function Tests and Renal Ultrasound**<br>● **Reasoning:** To further evaluate the extent of chronic renal insufficiency and any potential secondary causes of neuropathy related to renal disease.<br>**10. Blood Glucose Monitoring and Extended Diabetes Workup**

			● **Reasoning:** Despite negative HbA1c and glycemia, periodic monitoring and possibly continuous glucose monitoring (CGM) could provide a more accurate picture of glycemic control over time, which might still implicate diabetic neuropathy.<br>**Most Crucial Test**<br>**Nerve Biopsy (Sural or Superficial Peroneal Nerve):**<br>● **Reasoning:** This test is the most crucial because it can provide definitive evidence of vasculitis affecting the nerves, which is strongly suspected given the patient's history of ANCA vasculitis and the clinical presentation. A positive nerve biopsy showing vasculitic changes would solidify the diagnosis and directly influence the management plan, including immunosuppressive therapy.<br>**Conclusion**<br>A comprehensive approach involving these additional examinations will help in narrowing down the differential diagnoses by confirming the presence of specific pathological changes, thereby guiding targeted treatment strategies. The nerve biopsy stands out as the most critical test for its potential to provide conclusive evidence of vasculitic neuropathy.
12	An 18-year old male patient presents with a 16-year history of sensory-motor, symmetrical, length dependent polyneuropathy with insidious onset and slow progression. The patient presents with sensory ataxia, distal weakness in the lower limbs, severe calf atrophy, pes planus, and hammertoes with no cranial nerve or autonomic involvement. Past medical history is silent. Nerve conduction studies show an axonal, sensory-motor polyneuropathy. Screening lab tests including vitamin B12, folate, vitamin B6, vitamin E, HBV, HCV, glycemia, HbA1c, ANA, ANCA, ENA, CRP, ESR, copper, cryoglobulins, and serum electrophoresis are all negative. Her mother and grandmother have walking difficulties. Her 5-year-old sister has pes cavus.	CMT2	**Step-by-Step Analysis and Additional Examinations:**<br>1. **Detailed Family History and Pedigree Analysis:**<br>○ **Reasoning:** To further assess the hereditary pattern of the neuropathy, a detailed family history and pedigree analysis is essential. This can help confirm the mode of inheritance (e.g., autosomal dominant, autosomal recessive, or X-linked).<br>○ **Additional Information:** Questions should include specific ages of onset, types of symptoms, and any similar conditions in extended family members.<br>2. **Genetic Testing:**<br>○ **Reasoning:** Given the strong family history and the clinical presentation, genetic testing is critical for confirming the diagnosis of a hereditary neuropathy such as Charcot-Marie-Tooth disease (CMT) or Hereditary Sensory and Autonomic Neuropathy (HSAN). Testing for mutations in genes known to be associated with these conditions (e.g., PMP22, GJB1, MFN2 for CMT) would be appropriate.<br>○ **Specific Tests:** Panels for CMT and other hereditary neuropathies.<br>3. **Electromyography (EMG):**<br>○ **Reasoning:** While nerve conduction studies have already shown axonal neuropathy, EMG can provide additional information on the extent and pattern of muscle involvement, which can help differentiate between different types of neuropathies.<br>○ **Additional Information:** EMG can confirm chronic denervation and reinnervation, supporting the diagnosis of a long-standing neuropathy.<br>4. **Skin Biopsy:**<br>○ **Reasoning:** A skin biopsy can be useful to assess the density of intraepidermal nerve fibers, which can help in diagnosing small fiber neuropathy. Though not specifically indicated by the current symptoms, it can rule out any additional underlying small fiber involvement.<br>○ **Additional Information:** Examination for nerve fiber density and any signs of axonal degeneration.<br>5. **Phytanic Acid Levels:**<br>○ **Reasoning:** To rule out Refsum disease, a test for elevated phytanic acid levels in the plasma should be conducted, given its association with sensory-motor polyneuropathy and hereditary nature.<br>○ **Additional Information:** Phytanic acid accumulation leads to a variety of neurological symptoms and other systemic features.<br>6. **Whole Exome Sequencing (WES):**<br>○ **Reasoning:** If specific gene panels do not yield results, WES can be a comprehensive approach to identify rare or novel mutations associated with hereditary neuropathies.<br>○ **Additional Information:** This test can identify mutations in genes not typically included in standard panels.<br>7. **Neuromuscular Ultrasound:**<br>○ **Reasoning:** This non-invasive imaging can be used to visualize peripheral nerves and muscles, helping to assess nerve size, structure, and muscle morphology.<br>○ **Additional Information:** Can provide supportive evidence for neuropathy and muscle atrophy.<br>**Conclusion and Most Crucial Test:**<br>**Most Crucial Test: Genetic Testing**<br>● **Reasoning:** Given the strong family history and the typical presentation of a hereditary neuropathy, genetic testing specifically for Charcot-Marie-Tooth disease (CMT) and related hereditary neuropathies is the most crucial test. It provides a definitive diagnosis by identifying pathogenic mutations, guiding further management, and allowing for genetic counseling for the patient and family members.<br>**Summary of Additional Examinations:**<br>1. Detailed Family History and Pedigree Analysis<br>2. Genetic Testing (CMT and related neuropathy panels)<br>3. Electromyography (EMG)<br>4. Skin Biopsy (for intraepidermal nerve fiber density)<br>5. Phytanic Acid Levels (for Refsum disease)<br>6. Whole Exome Sequencing (WES)<br>7. Neuromuscular Ultrasound
13	An 82-year-old female patient presents with a 1-year history of sensory-motor, symmetrical, length dependent polyneuropathy with subacute onset and progressive course. The patient presents with paraesthesia and neuropathic pain in the hands and feet and bilateral foot drop. Clinical examination excludes sensory ataxia, cranial nerve or autonomic involvement, tremor or foot deformities. Past medical history is negative. Nerve conduction studies show a demyelinating, sensory-motor, symmetric polyneuropathy. Screening lab tests reveal presence of polycythaemia, IgG lambda paraprotein,	POEMS	**Step-by-Step Approach to Additional Examinations**<br>**1. Repeat Basic Metabolic Panel and Comprehensive Blood Work**<br>**Reasoning:**<br>● To confirm and further evaluate the hyperglycemia and elevated HbA1c levels.<br>● Assess kidney and liver function, which can be affected in diabetes and other systemic conditions.<br>**Tests:**<br>● Fasting blood glucose<br>● Repeat HbA1c<br>● Serum electrolytes<br>● Liver function tests (LFTs)<br>● Kidney function tests (KFTs)<br>**2. Specific Hematologic and Oncologic Tests**<br>**Reasoning:**<br>● To evaluate the significance of the IgG lambda paraprotein, which suggests a monoclonal gammopathy.<br>● These tests will help identify conditions like multiple myeloma, Waldenström's macroglobulinemia, or other monoclonal gammopathies.

	positivity for ANA, hyperglycemia, and elevated HbA1c. Remaining exams including vitamin B12, folate, vitamin B6, vitamin E, ANCA, CRP, ESR, copper, and cryoglobulins are negative. There is no family history of polyneuropathy.		**Tests:**<br>● Serum protein electrophoresis (SPEP)<br>● Immunofixation electrophoresis (IFE)<br>● Serum free light chains (FLC) assay<br>● Bone marrow biopsy (if indicated by the results of the above tests)<br>● Complete blood count (CBC) with differential<br>**3. Autoimmune and Inflammatory Markers**<br>**Reasoning:**<br>● Positive ANA can indicate an autoimmune process. Further autoimmune testing can help rule out conditions like systemic lupus erythematosus (SLE) or other connective tissue diseases that may have overlapping symptoms with neuropathy.<br>**Tests:**<br>● ANA panel with specific antibodies (e.g., anti-dsDNA, anti-Ro/SSA, anti-La/SSB, anti-Smith)<br>● Rheumatoid factor (RF)<br>● Anti-cyclic citrullinated peptide (anti-CCP) antibodies<br>**4. Nerve Biopsy**<br>**Reasoning:**<br>● To provide histological confirmation of the type of neuropathy (e.g., demyelinating vs. axonal) and to identify specific causes like vasculitis, amyloidosis, or other infiltrative processes.<br>**5. Imaging Studies**<br>**Reasoning:**<br>● To rule out structural causes of neuropathy and evaluate the extent of nerve involvement.<br>● Imaging can also help identify any hidden malignancies or other systemic conditions.<br>**Tests:**<br>● MRI of the spine and brain<br>● Ultrasound or MRI of peripheral nerves (if indicated)<br>**6. Genetic Testing (if indicated)**<br>**Reasoning:**<br>● Although there is no family history of polyneuropathy, late-onset genetic conditions can sometimes present without a family history. This can be considered if no other cause is identified.<br>**Tests:**<br>● Genetic panel for hereditary neuropathies<br>**Detailed Reasoning for Each Test**<br>1. **Fasting Blood Glucose and Repeat HbA1c:**<br>○ Confirms the diagnosis of diabetes and monitors blood sugar control, which is crucial since poor glycemic control can exacerbate neuropathy.<br>2. **Serum Protein Electrophoresis (SPEP), Immunofixation Electrophoresis (IFE), Serum Free Light Chains (FLC) Assay:**<br>○ Essential for characterizing the monoclonal protein and diagnosing conditions like multiple myeloma or MGUS (Monoclonal Gammopathy of Undetermined Significance).<br>3. **Bone Marrow Biopsy:**<br>○ Indicated if SPEP, IFE, or FLC tests suggest multiple myeloma or another plasma cell dyscrasia. It provides definitive diagnosis and staging.<br>4. **ANA Panel with Specific Antibodies, RF, Anti-CCP:**<br>○ Helps to identify or rule out specific autoimmune conditions that could present with neuropathy.<br>5. **Nerve Biopsy:**<br>○ Provides definitive histological diagnosis of the type and cause of neuropathy, particularly useful if there's suspicion of conditions like vasculitis or amyloidosis.<br>6. **MRI of the Spine and Brain, Peripheral Nerve Ultrasound or MRI:**<br>○ Rules out central causes and structural abnormalities that might be contributing to neuropathy.<br>7. **Genetic Testing:**<br>○ Considered in the absence of a clear diagnosis from other tests, especially if a hereditary neuropathy is suspected.<br>**Most Crucial Test**<br>**Serum Protein Electrophoresis (SPEP) and Immunofixation Electrophoresis (IFE)**<br>**Reasoning:**<br>● Given the presence of IgG lambda paraprotein, these tests are crucial for identifying and characterizing the monoclonal gammopathy. The results will help determine if the patient has multiple myeloma, MGUS, or another related condition, which significantly impacts the diagnosis and management plan. The identification of a paraproteinemic neuropathy will guide further specific interventions and management strategies.
14	A 22-year old male patient presents with a 20-year history of chronic, sensory-motor, symmetrical, length dependent polyneuropathy with insidious onset and slow progression. The patient presents with severe distal weakness and atrophy in the four limbs, hypoesthesia up to calves, pes planus, and scoliosis with no cranial nerve or autonomic involvement. He uses a rollator for walking. Past medical history is silent. Nerve conduction studies show demyelinating, symmetric, sensory-motor polyneuropathy. Screening lab tests including vitamin B12, folate, vitamin B6,	CMT4	**Step-by-Step Analysis and Additional Examinations**<br>**1. Clinical Re-evaluation:**<br>○ **Neurological Examination: Detailed re-evaluation to assess the extent of motor and sensory deficits, reflexes, and muscle tone.**<br>■ **Reasoning: To provide a baseline and detect any subtle changes that may guide further testing.**<br>**2. Genetic Testing:**<br>○ **Genetic Panel for Charcot-Marie-Tooth Disease:**<br>■ **Reasoning: Given the high suspicion of CMT, genetic testing for common mutations (e.g., PMP22 duplication/deletion for CMT1A) is crucial. This can confirm the diagnosis and determine the specific subtype of CMT.**<br>○ **Whole Exome Sequencing:**<br>■ **Reasoning: If initial genetic panel is negative, whole exome sequencing can identify less common mutations or other hereditary neuropathies.**<br>**3. Nerve Biopsy:**<br>○ **Sural Nerve Biopsy:**<br>■ **Reasoning: To look for characteristic features of demyelination, remyelination, and axonal loss, which can help differentiate between hereditary and acquired neuropathies.**<br>**4. Advanced Neuroimaging:**

	vitamin E, HBV, HCV, glycemia, HbA1c, ANA, ANCA, ENA, CRP, ESR, copper, cryoglobulins, and serum electrophoresis are all negative. There is no family history of polyneuropathy.		○ **MRI of the Spine:**<br>■ **Reasoning: To assess for spinal abnormalities contributing to scoliosis and to rule out other causes of neuropathy like compressive lesions.**<br>○ **MRI with Nerve-Specific Protocols:**<br>■ **Reasoning: High-resolution imaging of peripheral nerves can reveal patterns of nerve damage specific to certain neuropathies.**<br>**5. Laboratory Tests:**<br>○ **Genetic Metabolic Panel:**<br>■ **Reasoning: To rule out metabolic disorders that can cause neuropathy, such as metachromatic leukodystrophy or adrenoleukodystrophy.**<br>○ **Paraneoplastic Panel:**<br>■ **Reasoning: Although less likely given the chronicity and progression, ruling out paraneoplastic syndromes associated with neuropathies can be considered.**<br>○ **Repeat Electrodiagnostic Studies:**<br>■ **Reasoning: To confirm the findings of the initial studies and assess for any progression or changes in the neuropathy pattern.**<br>**Most Crucial Test**<br>**Genetic Testing for Charcot-Marie-Tooth Disease (specifically a genetic panel including PMP22 duplication/deletion analysis) is the most crucial test.**<br>**Detailed Reasoning:**<br>**1. Genetic Panel for Charcot-Marie-Tooth Disease:**<br>○ **Purpose: Confirm diagnosis and subtype of CMT.**<br>○ **Justification: High clinical suspicion based on the patient's presentation and electrophysiological findings.**<br>○ **Impact: Positive result would provide a definitive diagnosis, guide management, and offer genetic counseling for the patient and family.**<br>**2. Whole Exome Sequencing:**<br>○ **Purpose: Identify less common mutations or other hereditary neuropathies.**<br>○ **Justification: If the initial genetic panel is negative, this test can provide a broader genetic analysis.**<br>○ **Impact: Helps in diagnosing rare genetic causes of polyneuropathy.**<br>**3. Sural Nerve Biopsy:**<br>○ **Purpose: Histological confirmation of neuropathy type.**<br>○ **Justification: Can differentiate between hereditary and acquired causes based on pathology.**<br>○ **Impact: Provides detailed structural information on the nerve, supporting the diagnosis.**<br>**4. MRI of the Spine and with Nerve-Specific Protocols:**<br>○ **Purpose: Rule out spinal causes and assess peripheral nerve involvement.**<br>○ **Justification: Structural abnormalities can contribute to neuropathy; imaging can reveal specific patterns.**<br>○ **Impact: Non-invasive way to rule out other differential diagnoses and support clinical findings.**<br>**By conducting these additional examinations, particularly focusing on genetic testing, the diagnosis can be confirmed or further clarified, ensuring accurate management and prognostication for the patient.**
15	A 72-year-old female patient presents with a 2-year history of pure sensory, symmetrical, length dependent polyneuropathy with insidious onset. The patient presents with distal paresthesia and neuropathic pain in the upper and lower limbs in a stocking and glove distribution, without ataxia or tremor, foot deformities, cranial nerve or autonomic involvement. Past medical history shows multiple myeloma, for which the patient has undergone chemotherapy. Neuropathy symptoms started 2 months after chemotherapy. Nerve conduction studies show pure sensory, symmetric, axonal polyneuropathy. Screening lab tests including vitamin B12, folate, vitamin B6, vitamin E, HBV, HCV, ANA, ANCA, ENA, CRP, ESR, glycemia, HbA1c, copper and cryoglobulins are all negative, with positivity for IgG kappa paraprotein. There is no family history of polyneuropathy.	Chemotherapy-induced neuropathy	**Step-by-Step Approach for Further Analysis**<br>**Step 1: Review and Confirm Current Data**<br>● **Clinical Presentation**: Pure sensory, symmetrical, length-dependent polyneuropathy<br>● **History**: Multiple myeloma with chemotherapy, onset of neuropathy 2 months post-chemotherapy<br>● **Nerve Conduction Studies**: Pure sensory, symmetric, axonal polyneuropathy<br>● **Laboratory Results**: Positive for IgG kappa paraprotein, other tests negative<br>● **Family History**: No family history of polyneuropathy<br>**Step 2: Identify Additional Necessary Examinations**<br>1. **Serum Immunofixation Electrophoresis (IFE)**<br>○ **Reasoning**: To further characterize the IgG kappa paraprotein and determine if there is an underlying monoclonal gammopathy such as MGUS or progression to multiple myeloma. This can help differentiate between CIPN and MGUS-related neuropathy.<br>2. **Quantitative Immunoglobulins (IgG, IgA, IgM)**<br>○ **Reasoning**: Assess levels of immunoglobulins to check for abnormalities that might indicate an underlying hematologic disorder affecting the nerves.<br>3. **Bone Marrow Biopsy**<br>○ **Reasoning**: Given the history of multiple myeloma and presence of paraprotein, this can help assess the current status of the disease, rule out amyloidosis, and check for potential relapse or progression affecting the nerves.<br>4. **Skin Biopsy for Intraepidermal Nerve Fiber Density (IENFD)**<br>○ **Reasoning**: This can help confirm small fiber neuropathy, which may not be detected by nerve conduction studies, and provide additional information on the pattern and severity of nerve damage.<br>5. **MRI of Spine**<br>○ **Reasoning**: To rule out spinal pathology such as nerve compression or infiltration by myeloma, which could contribute to the neuropathic symptoms.<br>6. **Autonomic Testing (e.g., Quantitative Sudomotor Axon Reflex Test - QSART)**<br>○ **Reasoning**: Although there is no current autonomic involvement, autonomic testing can provide a more comprehensive assessment of peripheral neuropathy, especially in cases where symptoms may evolve.<br>7. **Urine Protein Electrophoresis and Immunofixation**<br>○ **Reasoning**: To detect Bence-Jones proteins, which could indicate multiple myeloma or light chain disease affecting the nerves.<br>8. **Lumbar Puncture for CSF Analysis**<br>○ **Reasoning**: To rule out any paraneoplastic syndromes or infections that could be contributing to the neuropathy.<br>**Step 3: Determine the Most Crucial Test**<br>● **Bone Marrow Biopsy**<br>○ **Reasoning**: Given the history of multiple myeloma, the presence of IgG kappa paraprotein, and the need to rule out disease progression, a bone marrow biopsy is the most crucial test. This will provide definitive information on the status of the multiple myeloma, assess for potential amyloidosis, and help determine if the neuropathy is related to a relapse or progression of myeloma or an associated hematologic disorder.<br>**Conclusion**

			While all proposed tests are valuable in narrowing down the differential diagnoses, the **Bone Marrow Biopsy** stands out as the most crucial. It directly addresses the potential causes related to the patient's history of multiple myeloma and the presence of paraprotein, providing essential information to guide further treatment and management.
16	A 59-year-old female patient presents with a 1-year history of pure sensory, symmetrical, non-length dependent polyneuropathy with insidious onset. The patient presents with paresthesia distributed proximally and distally in the upper and lower limbs in an asymmetric fashion, and sensory ataxia. There are no foot deformities, tremor, neuropathic pain, cranial nerve or autonomic involvement. Past medical history is positive for IgG lambda monoclonal gammopathy and lung cancer, for which she underwent chemotherapy 4 years ago. Nerve conduction studies show a demyelinating, non-length dependent, sensory-motor polyneuropathy with conduction blocks. Screening lab tests including vitamin B12, folate, vitamin B6, vitamin E, HCV, ANA, ANCA, ENA, CRP, ESR, glycemia, HbA1c, copper and cryoglobulins are all negative, with positivity for IgG lambda paraprotein and evidence of chronic HBV infection. There is no family history of polyneuropathy.	CIDP	**Step-by-Step Analysis and Additional Examinations**<br>Given the complexity of the patient's presentation, it is important to conduct further tests to narrow down the differential diagnoses. Below is a detailed list of additional examinations, along with their reasoning:<br>1. **Serum and Urine Immunofixation Electrophoresis (IFE)**<br>○ **Reasoning:**<br>■ This test will help characterize the monoclonal protein (IgG lambda) more precisely.<br>■ MGUS, multiple myeloma, and other plasma cell dyscrasias can be better evaluated with detailed immunofixation.<br>○ **Expected Outcome:** Confirm the presence and type of monoclonal protein, which can support the diagnosis of MGUS or related conditions.<br>2. **Bone Marrow Biopsy**<br>○ **Reasoning:**<br>■ To assess the extent of monoclonal gammopathy and rule out multiple myeloma or other hematologic malignancies.<br>■ Helps evaluate for any underlying malignancy that could contribute to neuropathy.<br>○ **Expected Outcome:** Identify clonal plasma cell population, ruling out or confirming conditions like multiple myeloma.<br>3. **Anti-MAG (Myelin-Associated Glycoprotein) Antibodies**<br>○ **Reasoning:**<br>■ Anti-MAG antibodies are often associated with demyelinating neuropathies related to monoclonal gammopathies.<br>■ Presence of these antibodies can help confirm a diagnosis of MGUS-associated neuropathy.<br>○ **Expected Outcome:** Positive anti-MAG antibodies would support the diagnosis of MGUS-associated neuropathy.<br>4. **CSF Analysis (Cerebrospinal Fluid)**<br>○ **Reasoning:**<br>■ To check for elevated protein levels and oligoclonal bands which can support the diagnosis of CIDP.<br>■ Rule out infectious or inflammatory processes within the central nervous system.<br>○ **Expected Outcome:** Elevated protein without pleocytosis would support CIDP.<br>5. **Paraneoplastic Panel (Anti-Hu, Anti-Yo, Anti-Ri, etc.)**<br>○ **Reasoning:**<br>■ Paraneoplastic syndromes can cause neuropathy and are associated with certain cancers, including lung cancer.<br>■ Identifying specific autoantibodies can help link the neuropathy to a paraneoplastic syndrome.<br>○ **Expected Outcome:** Presence of specific paraneoplastic antibodies would suggest a paraneoplastic etiology.<br>6. **Repeat Nerve Conduction Studies/EMG (Electromyography)**<br>○ **Reasoning:**<br>■ To further characterize the neuropathy and monitor progression.<br>■ Differentiates between axonal and demyelinating processes and assesses the severity.<br>○ **Expected Outcome:** Provide detailed information on the nature and extent of nerve involvement.<br>7. **Skin Biopsy for Intraepidermal Nerve Fiber Density**<br>○ **Reasoning:**<br>■ To evaluate small fiber neuropathy, which can sometimes be present even in demyelinating neuropathies.<br>■ Helps rule out other causes of sensory neuropathy.<br>○ **Expected Outcome:** Reduced nerve fiber density can indicate small fiber neuropathy, adding to the overall neuropathy picture.<br>8. **Lung Cancer Surveillance (CT scan)**<br>○ **Reasoning:**<br>■ To check for recurrence or metastasis of lung cancer, which could cause paraneoplastic neuropathy.<br>■ Ensure no new malignancies are contributing to the neuropathy.<br>○ **Expected Outcome:** Clear imaging would reduce the likelihood of a current paraneoplastic syndrome related to lung cancer.<br>**Most Crucial Test**<br>**Serum and Urine Immunofixation Electrophoresis (IFE):**<br>● This test is most crucial because it will provide detailed information on the monoclonal protein present. It helps to confirm the presence and type of monoclonal gammopathy, which is central to the suspected diagnosis of MGUS-associated neuropathy.<br>● By identifying the specific characteristics of the monoclonal protein, it can significantly narrow down the differential diagnosis and guide further specific testing and management.<br>**Conclusion**<br>A thorough analysis of the clinical scenario indicates that serum and urine immunofixation electrophoresis is the most crucial test. This will provide essential information to confirm the diagnosis of MGUS-associated neuropathy and exclude other potential hematologic malignancies. The other listed tests are also important to further narrow down the differential diagnoses and provide a comprehensive assessment of the patient's condition.
17	A 73-year-old female patient presents with a 1-year history of sensory-motor, symmetrical, polyneuropathy with subacute onset. The patient presents with distal paresthesia and neuropathic pain in the four limbs, sensory ataxia, cramps, orthostatic hypotension and diarrhoea but with no cranial nerve involvement or foot deformities. Clinical examination shows cutaneous angiomas and hyperpigmentation. Laboratory tests reveal	POEMS	**Step-by-Step Approach to Additional Examinations**<br>**Step 1: Laboratory Tests**<br>**1. Serum Free Light Chains (FLC) Assay**<br>○ **Reasoning:**<br>■ **Measures the levels of free light chains (kappa and lambda) in the blood.**<br>■ **Important for diagnosing and monitoring monoclonal gammopathies and AL amyloidosis.**<br>■ **Discrepancies in the kappa/lambda ratio can indicate a clonal plasma cell disorder.**<br>**2. Serum and Urine Protein Electrophoresis (SPEP and UPEP) with Immunofixation**<br>○ **Reasoning:**<br>■ **Identifies the presence and type of monoclonal protein (M-protein) in the blood and urine.**<br>■ **Essential for diagnosing multiple myeloma, AL amyloidosis, and other plasma cell dyscrasias.**

	presence of thrombocytosis and IgG lambda monoclonal gammopathy. Nerve conduction studies show a symmetric, sensory-motor, demyelinating-axonal polyneuropathy. Screening lab tests including vitamin B12, folate, vitamin B6, vitamin E, HCV, HBV, ANA, ANCA, ENA, CRP, ESR, glycemia, HbA1c, copper and cryoglobulins are negative, with positivity for IgG lambda paraprotein. There is no family history of polyneuropathy.		**3. NT-proBNP (N-terminal pro b-type Natriuretic Peptide) and Troponin Levels**<br>○ **Reasoning:**<br>■ **Helps in assessing cardiac involvement in amyloidosis.**<br>■ **Elevated levels are associated with cardiac amyloidosis, which can present with similar neuropathic and autonomic symptoms.**<br>**4. Complete Blood Count (CBC) with Differential**<br>○ **Reasoning:**<br>■ **Evaluates for any hematologic abnormalities that might suggest a plasma cell disorder or other systemic disease.**<br>■ **Thrombocytosis noted in the patient may need further clarification.**<br>**Step 2: Imaging and Instrumental Tests**<br>**1. Echocardiogram**<br>○ **Reasoning:**<br>■ **Non-invasive method to evaluate cardiac structure and function.**<br>■ **Important for detecting cardiac amyloidosis, which can present with thickened ventricular walls and other characteristic findings.**<br>**2. Abdominal Fat Pad Biopsy**<br>○ **Reasoning:**<br>■ **Minimally invasive procedure to detect amyloid deposits.**<br>■ **High sensitivity for diagnosing systemic amyloidosis.**<br>■ **Stained with Congo red to confirm the presence of amyloid fibrils.**<br>**3. Nerve Biopsy**<br>○ **Reasoning:**<br>■ **Can provide direct evidence of amyloid deposition in nerves.**<br>■ **Helps to differentiate between other causes of neuropathy.**<br>**4. Bone Marrow Biopsy**<br>○ **Reasoning:**<br>■ **Assess for plasma cell dyscrasia, myeloma, or amyloid infiltration.**<br>■ **Useful in diagnosing AL amyloidosis and evaluating the extent of clonal plasma cells.**<br>**Step 3: Advanced Tests**<br>**1. Positron Emission Tomography (PET) Scan**<br>○ **Reasoning:**<br>■ **Provides detailed imaging of systemic involvement in amyloidosis.**<br>■ **Useful for assessing organ involvement and the extent of disease spread.**<br>**2. Autonomic Function Tests**<br>○ **Reasoning:**<br>■ **Evaluates the extent of autonomic dysfunction, which is significant in this patient.**<br>■ **Tests like tilt-table test, heart rate variability, and QSART (Quantitative Sudomotor Axon Reflex Test).**<br>**Most Crucial Test**<br>**Abdominal Fat Pad Biopsy**<br>● **Reasoning:**<br>○ **This test is minimally invasive and has a high sensitivity for detecting amyloid deposits.**<br>○ **Direct confirmation of amyloid presence in the body is critical for diagnosing systemic amyloidosis.**<br>○ **Given the clinical presentation, positive IgG lambda monoclonal gammopathy, and the combination of neuropathic and autonomic symptoms, confirming amyloidosis would provide a definitive diagnosis and guide appropriate treatment.**<br>**Summary of Additional Examinations and Most Crucial Test**<br>**1. Laboratory Tests:**<br>○ **Serum Free Light Chains (FLC) Assay**<br>○ **Serum and Urine Protein Electrophoresis (SPEP and UPEP) with Immunofixation**<br>○ **NT-proBNP and Troponin Levels**<br>○ **Complete Blood Count (CBC) with Differential**<br>**2. Imaging and Instrumental Tests:**<br>○ **Echocardiogram**<br>○ **Abdominal Fat Pad Biopsy (Most crucial test)**<br>○ **Nerve Biopsy**<br>○ **Bone Marrow Biopsy**<br>**3. Advanced Tests:**<br>○ **Positron Emission Tomography (PET) Scan**<br>○ **Autonomic Function Tests**<br>**By conducting these tests, the diagnosis can be further narrowed down and confirmed, particularly focusing on ruling in or out AL amyloidosis, which appears to be the most likely diagnosis based on the current data.**
18	A 55-year-old male patient presents with a 5-month history of pure sensory, asymmetric, non-length dependent polyneuropathy with subacute onset and progressive course. The patient presents with paresthesia and hypoesthesia in the	Sjogren-associated ganglionopathy	**Step-by-Step Analysis and Additional Examinations**<br>**Step 1: Review of Case Details**<br>● **Chief Complaint**: Pure sensory, asymmetric, non-length dependent polyneuropathy<br>● **Symptoms**: Paresthesia, hypoesthesia, severe sensory ataxia, orthostatic hypotension, delayed gastric and intestinal emptying, xerostomia, xerophthalmia, reduced pupillary reflex, anhidrosis<br>● **Medical History**: Hashimoto's thyroiditis<br>● **Lab Findings**: Folate deficiency, low-titre ENA positivity, normal vitamin B12, B6, E, HBV, HCV, ANA, ANCA, CRP, ESR, copper, glycemia, HbA1c, cryoglobulins, serum electrophoresis

	distal areas of the four limbs, severe sensory ataxia, orthostatic hypotension, and delayed gastric and intestinal emptying. The patient also reports xerostomia and xerophthalmia. Clinical examination reveals reduced pupillary reflex and anhidrosis. There are no neuropathic pain, tremor, foot deformities or cranial nerve involvement. Past medical history is positive for Hashimoto's thyroiditis. Nerve conduction studies show a pure sensory, non-length dependent, axonal polyneuropathy. Screening lab tests reveal folate deficiency and low-titre ENA positivity. Remaining exams including vitamin B12, vitamin B6, vitamin E, HBV, HCV, ANA, ANCA, CRP, ESR, copper, glycemia, HbA1c, cryoglobulins, and serum electrophoresis are normal. There is no family history of polyneuropathy.		● **Nerve Conduction Studies**: Pure sensory, non-length dependent, axonal polyneuropathy<br>● **Family History**: No family history of polyneuropathy<br>**Step 2: List of Additional Examinations**<br>1. **Anti-SSA/Ro and Anti-SSB/La Antibodies**<br>○ **Reasoning**: These antibodies are specific for Sjogren's syndrome, which is a strong differential diagnosis given the patient's xerostomia, xerophthalmia, and autonomic symptoms. Positive results would support Sjogren's syndrome-associated neuropathy.<br>2. **Salivary Gland Biopsy (Lip Biopsy)**<br>○ **Reasoning**: A lip biopsy can reveal focal lymphocytic sialadenitis, which is diagnostic for Sjogren's syndrome. This test is particularly useful in cases with dry mouth and eyes, as in this patient.<br>3. **Paraneoplastic Panel**<br>○ **Reasoning**: This panel includes tests for antibodies associated with paraneoplastic neurological syndromes (e.g., anti-Hu, anti-Yo, anti-Ri). Given the subacute onset and complex symptoms, ruling out a paraneoplastic etiology is essential, especially with the possibility of an underlying malignancy.<br>4. **Thiamine (Vitamin B1) Levels**<br>○ **Reasoning**: Although the history does not strongly suggest thiamine deficiency, the presence of multiple sensory and autonomic symptoms warrants checking for nutritional deficiencies that could contribute to the neuropathy.<br>5. **Autonomic Function Tests**<br>○ **Reasoning**: These tests, including tilt-table testing, quantitative sudomotor axon reflex test (QSART), and heart rate variability analysis, can provide objective evidence of autonomic dysfunction, helping to quantify the extent and severity of autonomic involvement.<br>6. **Comprehensive Cancer Screening**<br>○ **Reasoning**: This may include imaging studies (CT, PET scans), tumor markers, and a thorough physical examination. Given the potential paraneoplastic nature of the symptoms, it is crucial to rule out an occult malignancy.<br>7. **Folate and Homocysteine Levels**<br>○ **Reasoning**: To reassess the extent of folate deficiency and its potential impact on the neuropathy. Elevated homocysteine levels can indicate functional folate deficiency.<br>8. **Anti-Ganglionic Acetylcholine Receptor Antibody (Anti-gAChR)**<br>○ **Reasoning**: This test can help diagnose autoimmune autonomic ganglionopathy, which could explain the severe autonomic symptoms (e.g., orthostatic hypotension, gastrointestinal dysmotility).<br>**Step 3: Determining the Most Crucial Test**<br>The most crucial test from this list is **Anti-SSA/Ro and Anti-SSB/La Antibodies**.<br>**Detailed Reasoning**<br>● **Relevance to Symptoms**: The patient has prominent symptoms of Sjogren's syndrome (xerostomia, xerophthalmia, autonomic dysfunction), and these antibodies are highly specific for the diagnosis.<br>● **Correlation with Existing Findings**: Low-titre ENA positivity and Hashimoto's thyroiditis (another autoimmune condition) strengthen the suspicion of an autoimmune etiology.<br>● **Diagnostic Value**: Positive results for these antibodies would confirm the diagnosis of Sjogren's syndrome, providing a clear direction for treatment and management.<br>**Conclusion**<br>The **Anti-SSA/Ro and Anti-SSB/La Antibodies** test is the most crucial examination to perform next, as it directly targets the most likely diagnosis (Sjogren's syndrome) and would significantly impact the clinical management of the patient.
19	A 70-year-old male patient presents with a 5-year history of sensory-motor, symmetrical, length dependent polyneuropathy with insidious onset and fast disease progression. The patient presents with moderate-to-severe distal weakness and hypoesthesia in the four limbs in a distal and symmetric distribution, sensory ataxia, tongue fasciculations and autonomic involvement with no foot deformities or tremor. Past medical history is positive for cardiac insufficiency. Nerve conduction studies show an axonal, sensory-motor polyneuropathy with bilateral carpal tunnel syndrome. Screening lab tests including vitamin B12, vitamin B9, folate, vitamin E, HBV, HCV, ANA, ANCA, ENA, glycemia, HbA1c, CRP, ESR, copper, cryoglobulins, and serum electrophoresis are all negative. His father, who was wheelchair bound, died from cardiac insufficiency.	TTR-Amyloidosis	**Step-by-Step Analysis of the Clinical Scenario**<br>**Summary of Pertinent Clinical Information**<br>● **Patient Profile:** 70-year-old male<br>● **History of Present Illness:** 5-year history of sensory-motor, symmetrical, length-dependent polyneuropathy with insidious onset and rapid progression<br>● **Symptoms:**<br>○ Moderate-to-severe distal weakness<br>○ Hypoesthesia in all four limbs (distal and symmetric)<br>○ Sensory ataxia<br>○ Tongue fasciculations<br>○ Autonomic involvement (details unspecified)<br>● **Past Medical History:** Cardiac insufficiency<br>● **Nerve Conduction Studies:** Axonal, sensory-motor polyneuropathy with bilateral carpal tunnel syndrome<br>● **Screening Lab Results:** Negative for vitamin B12, vitamin B9, folate, vitamin E, HBV, HCV, ANA, ANCA, ENA, glycemia, HbA1c, CRP, ESR, copper, cryoglobulins, and serum electrophoresis<br>● **Family History:** Father, who was wheelchair-bound, died from cardiac insufficiency<br>**Necessary Additional Examinations**<br>1. **Genetic Testing for Transthyretin (TTR) Mutations**<br>○ **Reasoning:** Given the strong suspicion for hereditary transthyretin amyloidosis (hATTR), identifying a mutation in the TTR gene would confirm the diagnosis. Family history of cardiac issues and neuropathy also supports this testing.<br>2. **Biopsy for Amyloid Deposits (Fat Pad, Nerve, or Cardiac)**<br>○ **Reasoning:** To confirm amyloidosis, a biopsy of the abdominal fat pad, nerve, or cardiac tissue can reveal amyloid deposits. Congo red staining followed by immunohistochemistry for TTR can specifically diagnose hATTR.<br>3. **Electrocardiogram (ECG) and Echocardiogram**<br>○ **Reasoning:** These tests are critical to assess the extent of cardiac involvement, which is common in amyloidosis. They can help identify restrictive cardiomyopathy or other cardiac abnormalities associated with amyloid deposits.<br>4. **Serum and Urine Protein Electrophoresis with Immunofixation**<br>○ **Reasoning:** Although initial serum electrophoresis was negative, immunofixation can detect smaller amounts of monoclonal proteins. This helps rule out other causes like monoclonal gammopathy of undetermined significance (MGUS) or multiple myeloma, which can present similarly.<br>5. **Autonomic Function Tests**<br>○ **Reasoning:** Detailed assessment of autonomic dysfunction through tests like tilt-table testing, heart rate variability, and sweat tests can provide more information on the extent and nature of autonomic involvement.<br>6. **Lumbar Puncture with Cerebrospinal Fluid (CSF) Analysis**<br>○ **Reasoning:** Examining CSF can rule out inflammatory or infectious causes of polyneuropathy, such as CIDP or paraneoplastic syndromes. Elevated protein without pleocytosis can be indicative of CIDP.

			7. **MRI of the Brain and Spine**<br>○ **Reasoning:** To exclude central nervous system involvement and to provide additional information on the extent of peripheral nerve involvement.<br>**Most Crucial Test**<br>**Genetic Testing for Transthyretin (TTR) Mutations**<br>**Justification:**<br>● **Direct Confirmation:** Identifying a mutation in the TTR gene directly confirms the diagnosis of hereditary transthyretin amyloidosis (hATTR).<br>● **Non-Invasive:** Genetic testing is less invasive compared to a biopsy.<br>● **Family History:** The patient's family history strongly suggests a hereditary component, making this test highly relevant.<br>● **Diagnostic Certainty:** It provides definitive evidence of hATTR, guiding specific treatment and family counseling.<br>**Conclusion**<br>Given the clinical presentation and family history, genetic testing for TTR mutations is the most crucial test. It will directly confirm the diagnosis of hereditary transthyretin amyloidosis, which aligns with the patient's symptoms and family history, facilitating appropriate management and genetic counseling for the patient and his family.
20	A 64-year-old male patient presents with an 18-month history of pure motor, asymmetric, non-length dependent polyneuropathy with insidious onset. The patient presents with weakness and atrophy in a multifocal distribution in the upper limbs. He also reports the presence of subjective paresthesia in the hands but at clinical examination objective tactile sensation is normal. There are no neuropathic pain, ataxia, tremor, foot deformities, cranial nerve or autonomic involvement. Past medical history is silent. Nerve conduction studies show pure motor, demyelinating multineuropathy in the upper limbs. Screening lab tests including vitamin B12, folate, vitamin B6, vitamin E, HCV, HBV, ANA, ANCA, ENA, CRP, ESR, glycemia, HbA1c, copper, cryoglobulins and serum electrophoresis are negative. Cervical spine MRI is normal. There is no family history of polyneuropathy.	MMN	**Step-by-Step Analysis**<br>1. **Review Clinical Presentation and Initial Findings**:<br>○ 64-year-old male with 18-month history of asymmetric, non-length dependent, pure motor polyneuropathy.<br>○ Multifocal weakness and atrophy in the upper limbs.<br>○ Subjective paresthesia without objective sensory loss.<br>○ Nerve conduction studies show pure motor, demyelinating multineuropathy.<br>○ Extensive lab tests negative, including vitamin levels, infection markers, autoimmune markers, metabolic panels, and serum electrophoresis.<br>○ Normal cervical spine MRI.<br>○ No family history of polyneuropathy.<br>2. **Evaluate Initial Differential Diagnoses**:<br>○ Multifocal Motor Neuropathy (MMN)<br>○ Amyotrophic Lateral Sclerosis (ALS)<br>○ Chronic Inflammatory Demyelinating Polyneuropathy (CIDP) - Multifocal Variant<br>3. **Determine Additional Examinations Needed**:<br>**Laboratory Tests**<br>1. **Anti-GM1 Antibodies**:<br>○ **Reasoning**: MMN is often associated with anti-GM1 antibodies. Detecting these can help confirm the diagnosis.<br>○ **Relevance**: Since MMN is high on the differential list, this test is crucial for supporting or excluding MMN.<br>2. **CSF Analysis (Lumbar Puncture)**:<br>○ **Reasoning**: Elevated protein with normal cell count (albuminocytologic dissociation) is indicative of CIDP. It can also help rule out other central nervous system disorders.<br>○ **Relevance**: Differentiates between CIDP and other neuropathies, and excludes infectious or inflammatory causes.<br>**Instrumental Examinations**<br>3. **Repeat Nerve Conduction Studies with F-Wave Analysis**:<br>○ **Reasoning**: Detailed conduction studies, including F-wave latency, can provide further evidence of demyelination or conduction block typical of MMN or CIDP.<br>○ **Relevance**: Differentiates between MMN and CIDP by evaluating the extent and pattern of conduction abnormalities.<br>4. **MRI of the Brachial Plexus**:<br>○ **Reasoning**: Helps identify any structural causes of nerve damage, such as nerve sheath tumors or compressive neuropathies, which could present similarly.<br>○ **Relevance**: Although less likely given normal cervical MRI, it rules out compressive etiologies contributing to the polyneuropathy.<br>5. **Electromyography (EMG)**:<br>○ **Reasoning**: Helps assess the extent of motor neuron involvement and differentiate between neurogenic and myopathic causes of muscle weakness.<br>○ **Relevance**: Provides additional data to distinguish ALS from MMN and CIDP.<br>**Immunological and Genetic Tests**<br>6. **Paraneoplastic Panel**:<br>○ **Reasoning**: To rule out paraneoplastic syndromes, which can present with neuropathies.<br>○ **Relevance**: Important for excluding rare but possible causes of neuropathy in the absence of a clear primary tumor.<br>7. **Genetic Testing for Hereditary Neuropathy**:<br>○ **Reasoning**: Despite the lack of family history, genetic forms of motor neuropathy can sometimes present sporadically.<br>○ **Relevance**: Useful if all other tests are inconclusive.<br>**Most Crucial Test**<br>**Anti-GM1 Antibodies Test** is the most crucial test to conduct next.<br>**Reasoning**:<br>● Given the strong suspicion of MMN based on clinical presentation and initial findings, detecting anti-GM1 antibodies can provide a definitive diagnosis.<br>● It is a relatively specific test for MMN, distinguishing it from CIDP and ALS, which do not typically present with anti-GM1 antibodies.<br>● Positive anti-GM1 antibodies would confirm MMN and guide appropriate immunomodulatory treatment, such as IVIg therapy, which is effective for MMN.<br>By conducting this test, the diagnosis of MMN can be either confirmed or excluded, significantly narrowing the differential and directing subsequent management strategies.
21	A 74-year-old male patient presents with a 3-month history of sensory-motor, asymmetric, length dependent polyneuropathy with subacute onset and progressive course. The patient presents with sensory ataxia associated with	CIDP	**Step-by-Step Analysis and Additional Examinations**<br>**Step 1: Detailed Review of the Case**<br>● 74-year-old male with a 3-month history of sensory-motor, asymmetric, length-dependent polyneuropathy.<br>● Sensory ataxia, neuropathic pain, paraesthesia, hypoesthesia in feet, and distal, asymmetric weakness.<br>● Multiple myeloma in remission, last chemotherapy three years ago.<br>● Nerve conduction studies show demyelinating, sensory-motor polyneuropathy.

	neuropathic pain, paraesthesia, and hypoesthesia in the feet, and weakness with distal, asymmetric distribution. Clinical examination does not show presence of tremor, foot deformities, cranial nerve or autonomic involvement. Past medical history is positive for multiple myeloma, in remission for the past three years with no need for maintenance therapy (last chemotherapy cycle performed three years ago). Nerve conduction studies show a demyelinating, sensory-motor polyneuropathy. Screening lab tests show presence of IgG lambda paraprotein. Remaining exams including vitamin B12, folate, vitamin B6, vitamin E, HBV, HCV, ANA, ANCA, ENA, CRP, ESR, glycemia, HbA1c, copper, and cryoglobulins are negative. There is no family history of polyneuropathy.		● Presence of IgG lambda paraprotein.<br>● Negative for vitamin deficiencies, hepatitis B and C, autoimmune markers, inflammatory markers, glucose abnormalities, copper, and cryoglobulins.<br>● No family history of polyneuropathy.<br>**Step 2: Identifying Additional Examinations**<br>1. **Serum Protein Electrophoresis (SPEP) and Immunofixation Electrophoresis (IFE)**<br>○ **Reasoning:** To confirm the presence and type of monoclonal protein (IgG lambda). This helps in differentiating between MGUS, multiple myeloma, or other plasma cell dyscrasias.<br>○ **Details:** These tests identify and quantify monoclonal proteins and are crucial in diagnosing conditions like MGUS or relapse of multiple myeloma.<br>2. **Lumbar Puncture with Cerebrospinal Fluid (CSF) Analysis**<br>○ **Reasoning:** To check for albuminocytologic dissociation which is often seen in CIDP. This test can help differentiate between CIDP and other neuropathies.<br>○ **Details:** Elevated protein levels without significant pleocytosis (increased white cells) would support a diagnosis of CIDP.<br>3. **Anti-MAG (Myelin-Associated Glycoprotein) Antibodies**<br>○ **Reasoning:** These antibodies are often associated with demyelinating neuropathies linked to monoclonal gammopathies, particularly IgM MGUS.<br>○ **Details:** Positive anti-MAG antibodies would support the diagnosis of a paraproteinemic neuropathy.<br>4. **Bone Marrow Biopsy**<br>○ **Reasoning:** To evaluate for potential relapse of multiple myeloma or other hematologic malignancies that might explain the neuropathy.<br>○ **Details:** This test would help determine if the multiple myeloma is in fact in remission or if there has been a recurrence.<br>5. **Skin Biopsy for Intraepidermal Nerve Fiber Density (IENFD)**<br>○ **Reasoning:** To evaluate small fiber neuropathy which may not be detected on nerve conduction studies.<br>○ **Details:** A reduction in nerve fiber density can indicate small fiber neuropathy, which can coexist with other neuropathies.<br>6. **Genetic Testing for Hereditary Neuropathies**<br>○ **Reasoning:** To rule out hereditary neuropathies given the patient's age and presentation, despite no family history.<br>○ **Details:** Although less likely, identifying genetic mutations could be pivotal in diagnosis.<br>7. **MRI of the Spine and Brain**<br>○ **Reasoning:** To rule out structural lesions or central nervous system involvement which might contribute to the symptoms.<br>○ **Details:** This can help exclude compressive neuropathies or other central causes of neuropathy.<br>**Conclusion: Most Crucial Test**<br>**Serum Protein Electrophoresis (SPEP) and Immunofixation Electrophoresis (IFE)**<br>**Reasoning:**<br>● Given the presence of IgG lambda paraprotein and the history of multiple myeloma, it is essential to confirm and quantify the monoclonal protein.<br>● This test will help determine whether the neuropathy is related to MGUS, relapse of multiple myeloma, or another plasma cell disorder.<br>● The results will guide further management and therapeutic decisions, including the need for more intensive investigations like bone marrow biopsy or specific antibody testing.<br>**Summary:** Performing SPEP and IFE will provide critical information regarding the monoclonal protein's presence and type, thereby significantly narrowing the differential diagnosis and guiding subsequent management steps.
22	An 82-year-old female patient presents with a 2-year history of pure sensory, symmetrical, length dependent polyneuropathy with insidious onset and slow progressive course. The patient presents with paresthesia, hypoesthesia, and mild neuropathic pain in the feet, associated with diffused cramps in the lower limbs. Clinical examination does not reveal sensory ataxia, tremor, foot deformities, cranial nerve or autonomic involvement. Past medical history is positive for chronic HCV infection, psoriatic arthritis, and fibromyalgia. Nerve conduction studies show presence of a pure sensory, length dependent, symmetric, axonal polyneuropathy with unilateral carpal tunnel syndrome. Screening lab tests including vitamin B12, folate, vitamin B6, vitamin E, HBV, ANCA, ENA, CRP, ESR, copper, cryoglobulins and serum electrophoresis are all negative, with positivity for ANA autoantibodies and evidence of chronic HCV infection. There is no family history of polyneuropathy.	HCV-associated neuropathy	**Step-by-Step Analysis and Additional Examinations**<br>**Analyzing the Clinical Scenario**<br>● **Patient Demographics:**<br>○ 82-year-old female<br>● **History of Present Illness:**<br>○ 2-year history of pure sensory, symmetrical, length-dependent polyneuropathy<br>○ Symptoms: paresthesia, hypoesthesia, mild neuropathic pain, cramps in lower limbs<br>● **Clinical Examination:**<br>○ No sensory ataxia, tremor, foot deformities, cranial nerve or autonomic involvement<br>● **Past Medical History:**<br>○ Chronic HCV infection<br>○ Psoriatic arthritis<br>○ Fibromyalgia<br>● **Diagnostic Tests:**<br>○ Nerve conduction studies: pure sensory, length-dependent, symmetric, axonal polyneuropathy; unilateral carpal tunnel syndrome<br>○ Screening lab tests: Negative for vitamin B12, folate, vitamin B6, vitamin E, HBV, ANCA, ENA, CRP, ESR, copper, cryoglobulins, serum electrophoresis; positive for ANA autoantibodies and chronic HCV infection<br>● **Family History:**<br>○ No family history of polyneuropathy<br>**Necessary Additional Examinations**<br>**Laboratory Tests**<br>1. **Anti-Ro/SSA and Anti-La/SSB Antibodies:**<br>○ **Reasoning:**<br>■ These antibodies are associated with Sjögren's syndrome, which can present with sensory neuropathy even without classic sicca symptoms.<br>■ The presence of ANA autoantibodies raises suspicion for autoimmune etiologies.<br>2. **Paraneoplastic Antibody Panel:**<br>○ **Reasoning:**<br>■ Older adults with unexplained neuropathy should be screened for paraneoplastic syndromes.<br>■ Paraneoplastic antibodies (e.g., anti-Hu, anti-Yo, anti-Ri) can help identify an underlying malignancy causing neuropathy.<br>3. **HCV RNA Quantitative Test:**<br>○ **Reasoning:**<br>■ To assess the viral load and activity of chronic HCV infection.

			■ Correlation between viral load and neuropathy severity can guide management.<br>4. **Serum and Urine Immunofixation Electrophoresis:**<br>○ **Reasoning:**<br>■ To detect monoclonal gammopathy, which can be associated with multiple myeloma or MGUS, both of which can cause neuropathy.<br>■ Despite previous serum electrophoresis being negative, immunofixation is more sensitive.<br>5. **Comprehensive Metabolic Panel and Thyroid Function Tests:**<br>○ **Reasoning:**<br>■ To exclude metabolic causes (e.g., diabetes, renal dysfunction) and thyroid dysfunction, both of which can cause polyneuropathy.<br>**Instrumental Tests**<br>6. **Electromyography (EMG):**<br>○ **Reasoning:**<br>■ To further characterize the extent and type of neuropathy, distinguishing between axonal and demyelinating processes.<br>■ Can help in assessing the severity and progression of the neuropathy.<br>7. **Magnetic Resonance Imaging (MRI) of the Spine:**<br>○ **Reasoning:**<br>■ To exclude spinal pathologies such as spinal stenosis or tumors that might contribute to the patient's symptoms.<br>■ Useful in detecting structural abnormalities that might compress peripheral nerves.<br>8. **High-Resolution Ultrasound of Peripheral Nerves:**<br>○ **Reasoning:**<br>■ To visualize the peripheral nerves and identify any focal abnormalities or compressions not evident on nerve conduction studies.<br>9. **Whole-Body Positron Emission Tomography-Computed Tomography (PET-CT):**<br>○ **Reasoning:**<br>■ To screen for malignancies that might be causing paraneoplastic neuropathy.<br>■ Provides a comprehensive assessment for hidden neoplasms.<br>**Determining the Most Crucial Test**<br>Among these proposed tests, the most crucial test appears to be the **Anti-Ro/SSA and Anti-La/SSB Antibodies** test. This test is essential because:<br>● The presence of ANA autoantibodies suggests an autoimmune process, and Sjögren's syndrome can present with neuropathy even without classic symptoms.<br>● Identifying Sjögren's syndrome would significantly impact the management and treatment strategy for this patient.<br>● It directly targets a probable differential diagnosis considering the current clinical findings and known autoimmune background (psoriatic arthritis).<br>**Conclusion**<br>Given the comprehensive nature of the presented case and the need to narrow down the differential diagnoses, additional examinations, particularly focused on autoimmune and paraneoplastic causes, are warranted. Among these, testing for Anti-Ro/SSA and Anti-La/SSB antibodies stands out as the most critical step in furthering the diagnostic process.
23	A 59-year-old male patient presents with a 1-year history of sensory-motor, symmetrical, non-length dependent polyneuropathy with subacute onset and progressive course. The patient presents with paresthesia, neuropathic pain and weakness distributed proximally and distally in the lower limbs. The patient also reports that in the last months he has noticed the appearance of white nails. Clinical examination does not reveal ataxia, tremor, foot deformities, cranial nerve or autonomic involvement. Past medical history is positive for alcohol abuse. Screening lab tests show the presence of thrombocytosis and IgG lambda monoclonal gammopathy. Vitamin B12, folate, vitamin B6, E, HCV, HBV, ANA, ANCA, ENA, CRP, ESR, glycemia, HbA1c, copper and cryoglobulins are normal. Nerve conduction studies show a mixed demyelinating-axonal, sensory-motor polyneuropathy. There is no family history of polyneuropathy.	POEMS	**Step-by-Step Analysis and Additional Examinations**<br>1. **Cerebrospinal Fluid (CSF) Analysis**<br>○ **Reasoning**: CSF analysis can reveal elevated protein levels with a normal white cell count (albuminocytologic dissociation), which is characteristic of CIDP. This test helps to confirm CIDP and exclude other infectious or inflammatory causes of polyneuropathy.<br>2. **Serum Immunofixation Electrophoresis and Free Light Chain Assay**<br>○ **Reasoning**: To further characterize the monoclonal gammopathy detected in initial screening. This test can help differentiate between MGUS and more severe hematologic disorders like multiple myeloma or Waldenström's macroglobulinemia, which can also present with neuropathy.<br>3. **Nerve Biopsy**<br>○ **Reasoning**: A nerve biopsy can provide definitive histological evidence of CIDP, showing segmental demyelination and remyelination. It can also help to rule out other causes of neuropathy, such as amyloid neuropathy or vasculitis.<br>4. **MRI of the Lumbosacral Plexus**<br>○ **Reasoning**: MRI can reveal hypertrophy of spinal roots or plexuses and contrast enhancement, which are indicative of CIDP. It can help in visualizing structural changes that support the diagnosis.<br>5. **Autonomic Testing**<br>○ **Reasoning**: Although the patient does not have apparent autonomic symptoms, autonomic testing can help rule out autonomic neuropathies which might not be evident clinically. Tests include heart rate variability, blood pressure response to standing, and sweating tests.<br>6. **Bone Marrow Biopsy**<br>○ **Reasoning**: Given the presence of thrombocytosis and monoclonal gammopathy, a bone marrow biopsy can help rule out plasma cell dyscrasias, myeloproliferative disorders, or hematologic malignancies that could be associated with neuropathy.<br>7. **Liver Function Tests and Serum Transaminases**<br>○ **Reasoning**: Chronic alcohol abuse can lead to liver dysfunction. Liver function tests can help assess the extent of liver damage, which might contribute to the patient's neuropathy.<br>8. **Genetic Testing for Hereditary Neuropathies**<br>○ **Reasoning**: Although there is no family history of polyneuropathy, genetic testing can help exclude hereditary neuropathies such as Charcot-Marie-Tooth disease, especially since the clinical presentation is non-length dependent.<br>**Determining the Most Crucial Test**<br>**Cerebrospinal Fluid (CSF) Analysis**<br>**Rationale**: CSF analysis is the most crucial test in this scenario as it can provide direct supportive evidence for CIDP by showing elevated protein levels without pleocytosis. This finding is highly indicative of CIDP and can help confirm the diagnosis in conjunction with the clinical presentation and nerve conduction study results. Additionally, it helps to exclude other potential infectious or inflammatory causes of polyneuropathy, making it a pivotal diagnostic tool in this clinical setting.
24	A 77-year-old male patient presents with a 1-year history of sensory-motor,	POEMS	**Step-by-Step Analysis and Recommendations for Additional Examinations**<br>**Step 1: Summary of Clinical Scenario**

	symmetrical, length dependent polyneuropathy with subacute onset. The patient presents with distal leg paresthesia and neuropathic pain, distal weakness in the upper and lower limbs, without tremor, ataxia, foot deformities, cranial nerve or autonomic involvement. Past medical history shows cutaneous angiomas and IgG lambda monoclonal gammopathy. Nerve conduction studies show a demyelinating, symmetric, sensory-motor polyneuropathy. Screening lab tests including vitamin B12, folate, vitamin B6, vitamin E, HCV, HBV, ANA, ANCA, ENA, CRP, ESR, glycemia, HbA1c, copper and cryoglobulins are all negative, with positivity for IgG lambda paraprotein. There is no family history of polyneuropathy.		**Patient Details:**<br>● 77-year-old male<br>● 1-year history of sensory-motor, symmetrical, length-dependent polyneuropathy<br>● Subacute onset<br>● Distal leg paresthesia, neuropathic pain, and distal weakness in upper and lower limbs<br>● No tremor, ataxia, foot deformities, cranial nerve, or autonomic involvement<br>● Past medical history: cutaneous angiomas, IgG lambda monoclonal gammopathy<br>● Nerve conduction studies: demyelinating, symmetric, sensory-motor polyneuropathy<br>● Screening lab tests: negative for vitamin B12, folate, vitamin B6, vitamin E, HCV, HBV, ANA, ANCA, ENA, CRP, ESR, glycemia, HbA1c, copper, and cryoglobulins; positive for IgG lambda paraprotein<br>● No family history of polyneuropathy<br>**Step 2: Additional Examinations**<br>**1. Serum VEGF (Vascular Endothelial Growth Factor) Levels**<br>**Reasoning:**<br>● Elevated VEGF levels are a hallmark of POEMS syndrome and are critical for diagnosis.<br>● VEGF is involved in angiogenesis and increased levels can explain the cutaneous angiomas.<br>● This test can help confirm the diagnosis of POEMS syndrome.<br>**2. Whole-body PET-CT or CT Scan**<br>**Reasoning:**<br>● Imaging to assess for organomegaly (especially liver and spleen), lymphadenopathy, and osteosclerotic lesions, which are common in POEMS syndrome.<br>● Helps in identifying any underlying malignancies or other structural abnormalities that may explain the neuropathy.<br>**3. Serum and Urine Immunofixation Electrophoresis**<br>**Reasoning:**<br>● To further characterize the monoclonal gammopathy and rule out multiple myeloma or related plasma cell disorders.<br>● Identifies the type of monoclonal protein and its concentration, which is crucial for diagnosing conditions like POEMS or other gammopathies.<br>**4. Lumbar Puncture with Cerebrospinal Fluid (CSF) Analysis**<br>**Reasoning:**<br>● Helps to evaluate for increased protein concentration, which is often seen in CIDP.<br>● CSF analysis can help rule out other inflammatory or infectious causes of neuropathy.<br>**5. Endocrine Evaluation (including thyroid function tests, glucose tolerance test, and sex hormones)**<br>**Reasoning:**<br>● POEMS syndrome often presents with endocrine abnormalities (e.g., hypothyroidism, diabetes, hypogonadism).<br>● Comprehensive endocrine testing can help identify these abnormalities and support the diagnosis.<br>**6. Bone Marrow Biopsy**<br>**Reasoning:**<br>● To assess for plasma cell dyscrasias, amyloidosis, or other hematologic malignancies.<br>● This test is particularly useful if multiple myeloma or POEMS syndrome is suspected.<br>**Step 3: Determining the Most Crucial Test**<br>**Most Crucial Test: Serum VEGF Levels Reasoning:**<br>● Elevated VEGF levels are highly specific for POEMS syndrome and are considered a major criterion for its diagnosis.<br>● Given the patient's clinical presentation and the presence of an IgG lambda monoclonal gammopathy, confirming elevated VEGF levels would significantly narrow the diagnosis towards POEMS syndrome, aiding in early and targeted management.<br>**Conclusion**<br>For the given clinical scenario, the most crucial test is the measurement of serum VEGF levels. This test will help confirm the diagnosis of POEMS syndrome, which is highly suspected based on the patient's clinical presentation and laboratory findings. The other suggested tests will provide additional supportive information and help rule out other differential diagnoses.
25	A 78-year-old male patient presents with an 8-month history of sensory-motor, symmetrical, length dependent polyneuropathy with insidious onset and progressive course. The patient presents with paresthesia, neuropathic pain and weakness distributed distally in the lower limbs. Clinical examination also reveals the presence sensory ataxia. There are no foot deformities, tremor, cranial nerve or autonomic involvement. Past medical history is positive for IgM monoclonal gammopathy. Nerve conduction studies show a demyelinating, symmetric, sensory-motor polyneuropathy. Screening lab tests including vitamin B12, folate, vitamin B6, vitamin E, HCV, HBV, ANA, ANCA, ENA, CRP, ESR, glycemia, HbA1c, copper and cryoglobulins are all negative, with	anti-MAG polyneuropathy	**Step-by-Step Approach for Additional Examinations**<br>**1. Additional Examinations:**<br>1. **Serum Protein Electrophoresis (SPEP) and Immunofixation Electrophoresis (IFE):**<br>○ **Reasoning:**<br>■ These tests are essential to quantify and characterize the monoclonal protein (M-protein).<br>■ They can help confirm the presence and type of monoclonal gammopathy, distinguishing between different types such as MGUS, Waldenström Macroglobulinemia, and multiple myeloma.<br>■ IFE is particularly sensitive in identifying the specific type of immunoglobulin and light chain involved, which is crucial for diagnosis and monitoring of plasma cell disorders.<br>2. **Bone Marrow Biopsy and Aspirate:**<br>○ **Reasoning:**<br>■ A bone marrow examination can reveal the presence of abnormal plasma cells or lymphoplasmacytic cells, which are indicative of conditions like Waldenström Macroglobulinemia or multiple myeloma.<br>■ It can also help assess the degree of bone marrow infiltration and rule out other hematologic malignancies.<br>3. **Quantitative Immunoglobulins:**<br>○ **Reasoning:**<br>■ Measuring the levels of IgG, IgA, and IgM can provide additional information on the monoclonal gammopathy and help in distinguishing between different disorders.<br>■ Elevated levels of IgM are characteristic of Waldenström Macroglobulinemia.<br>4. **Anti-MAG Antibodies:**<br>○ **Reasoning:**<br>■ Anti-myelin-associated glycoprotein (MAG) antibodies are often associated with demyelinating neuropathies linked to IgM monoclonal gammopathy.<br>■ Their presence would support a diagnosis of a neuropathy associated with IgM paraproteinemia, such as in Waldenström Macroglobulinemia.

	positivity for IgM paraprotein. There is no family history of polyneuropathy.		5. **Cerebrospinal Fluid (CSF) Analysis:**<br>○ **Reasoning:**<br>■ CSF analysis can help rule out central nervous system involvement and inflammatory causes of neuropathy.<br>■ Elevated protein levels with few or no cells (albumin-cytological dissociation) can be seen in CIDP.<br>6. **PET-CT or CT Scan of the Chest, Abdomen, and Pelvis:**<br>○ **Reasoning:**<br>■ Imaging studies can help identify lymphadenopathy, organomegaly, or other masses that might suggest a lymphoproliferative disorder like Waldenström Macroglobulinemia.<br>■ These scans are useful for staging and assessing the extent of disease.<br>7. **Serum Free Light Chain Assay:**<br>○ **Reasoning:**<br>■ This test measures the free light chains of immunoglobulins (kappa and lambda) in the blood and can detect abnormalities not visible in SPEP.<br>■ It is particularly useful for diagnosing light chain multiple myeloma or AL amyloidosis.<br>8. **Cryoglobulin Test:**<br>○ **Reasoning:**<br>■ Although the initial cryoglobulin test was negative, repeating it might be beneficial as cryoglobulins can sometimes be intermittently present and associated with monoclonal gammopathies.<br>■ This test can help identify cryoglobulinemia, which can present with similar symptoms.<br>**2. Most Crucial Test:**<br>The **Bone Marrow Biopsy and Aspirate** is the most crucial test. It provides definitive diagnostic information about the bone marrow's cellular composition, the presence of abnormal lymphoplasmacytic or plasma cells, and the extent of infiltration. This test is essential for confirming or ruling out Waldenström Macroglobulinemia or other hematologic malignancies, making it indispensable for narrowing down the differential diagnoses in this patient.
26	An 80-year-old male patient presents with a 2-year history of sensory-motor, asymmetrical, non-length dependent polyneuropathy with subacute onset and a rapid progressive course. The patient presents with paraesthesia, sensory ataxia, neuropathic pain, proximal and distal weakness in the four limbs, and tremor. Clinical examination shows symmetric proximal and distal weakness in the upper and lower limbs, hypoesthesia in a stocking and glove distribution, sensory ataxia, mild intrinsic hand atrophy, and cutaneous angiomas. Clinical examination does not show the presence of foot deformities, cranial nerve, or autonomic involvement. Past medical history is positive for chronic HCV infection. Nerve conduction studies show a mixed demyelinating-axonal, symmetric, sensory-motor polyneuropathy. Screening lab tests reveal the presence of polycythaemia, IgM paraprotein, and chronic HCV infection. Remaining lab tests, including vitamin B12, folate, vitamin B6, vitamin E, HBV, ANA, ANCA, ENA, CRP, ESR, copper and cryoglobulins are all negative. There is no family history of polyneuropathy.	POEMS	**Step-by-Step Analysis of Additional Examinations**<br>**Necessary Additional Examinations**<br>**1. Serum Cryoglobulin Testing**<br>**Reasoning:**<br>● **HCV-related Cryoglobulinemia:** Given the patient's chronic HCV infection and symptoms, testing for cryoglobulins is crucial. Mixed cryoglobulinemia is a common complication of chronic HCV infection and can lead to vasculitis and neuropathy.<br>● **Supporting Evidence:** Presence of cutaneous angiomas and neuropathic symptoms support the likelihood of cryoglobulinemia.<br>**2. Bone Marrow Biopsy**<br>**Reasoning:**<br>● **Polycythaemia:** The patient's polycythaemia could be due to a myeloproliferative disorder like polycythemia vera, which requires a bone marrow biopsy for confirmation.<br>● **MGUS Diagnosis:** If IgM paraprotein is present, bone marrow examination can help assess the presence of plasma cell dyscrasias, such as MGUS or multiple myeloma.<br>**3. Serum and Urine Protein Electrophoresis (SPEP and UPEP)**<br>**Reasoning:**<br>● **IgM Paraprotein:** To further evaluate the presence and quantity of monoclonal proteins, which can indicate conditions like MGUS, multiple myeloma, or Waldenström's macroglobulinemia.<br>● **Differentiation:** Helps distinguish between different types of paraproteinemias and their potential neuropathic complications.<br>**4. Magnetic Resonance Imaging (MRI) of the Brain and Spine**<br>**Reasoning:**<br>● **Neurological Symptoms:** MRI can identify structural lesions or other CNS involvement that may contribute to the patient's symptoms, such as demyelinating diseases or spinal cord compression.<br>● **Rule Out Other Causes:** Ensures there are no other underlying conditions contributing to the neuropathy, such as spinal stenosis or brain lesions.<br>**5. Skin Biopsy with Immunofluorescence**<br>**Reasoning:**<br>● **Cutaneous Angiomas:** To assess for vasculitis or other dermatological manifestations of systemic disease, particularly in the context of suspected cryoglobulinemia.<br>● **Direct Evidence:** Can provide direct evidence of vasculitis or immune complex deposition in the skin.<br>**6. Hepatitis C Virus RNA Quantification and Genotyping**<br>**Reasoning:**<br>● **HCV Infection:** Quantifying HCV RNA levels and determining the genotype can guide treatment decisions and provide a baseline for monitoring therapy response.<br>● **Treatment Implications:** Important for tailoring antiviral therapy, which may impact neuropathy if the underlying HCV is treated effectively.<br>**7. Autonomic Testing**<br>**Reasoning:**<br>● **Comprehensive Assessment:** Although no autonomic involvement was noted on initial examination, detailed autonomic testing can provide a more comprehensive assessment, ensuring no subtle autonomic dysfunction is present.<br>● **Rule Out Subclinical Involvement:** This testing can rule out subclinical autonomic involvement, which might be associated with some systemic diseases.<br>**8. PET-CT Scan**<br>**Reasoning:**<br>● **Systemic Disease Screening:** To identify any systemic malignancy or other underlying conditions not apparent through initial screening, especially given the age of the patient and the presence of polycythaemia and IgM paraprotein.<br>● **Comprehensive Evaluation:** Provides a thorough evaluation of potential underlying causes, such as lymphoproliferative disorders.<br>**Most Crucial Test**<br>**Serum Cryoglobulin Testing**<br>**Reasoning:**<br>● **High Probability Diagnosis:** Given the patient’s history of chronic HCV infection, polyneuropathy symptoms, and presence of IgM paraprotein, cryoglobulinemia is a leading diagnostic consideration.<br>● **Confirmatory Nature:** This test is directly confirmatory for mixed cryoglobulinemia, which can explain the patient's neuropathic symptoms and systemic findings.<br>● **Guidance for Treatment:** Positive cryoglobulin results would significantly influence the management plan, including potential antiviral and immunosuppressive therapies.<br>**Conclusion**

			Serum cryoglobulin testing is the most crucial test in this scenario due to its potential to confirm a high-probability diagnosis of HCV-related mixed cryoglobulinemia. This would not only provide a definitive explanation for the patient's symptoms but also guide targeted treatment strategies.
27	A 76-year-old female patient presents with a three-year history of pure sensory, symmetrical, length-dependent polyneuropathy with insidious onset and progressive course. The patient reports paraesthesia, severe neuropathic pain, and cramps in the feet in a symmetric distribution, which began approximately three years ago but significantly worsened after the third cycle of chemotherapy. Clinical examination does not reveal ataxia, tremor, foot deformities, cranial nerve or autonomic dysfunction. Past medical history is positive for type 2 diabetes, non-Hodgkin lymphoma, and breast cancer, for which she has undergone chemotherapy. Nerve conduction studies show a sensorimotor, length-dependent, symmetric, axonal polyneuropathy. Screening lab tests including vitamin B12, folate, vitamin B6, vitamin E, HCV, ANA, ANCA, ENA, CRP, ESR, copper, cryoglobulins, and serum electrophoresis are all negative. However, there is evidence of hyperglycemia, elevated HbA1c, and chronic HBV infection. There is no family history of polyneuropathy.	Superimposed chemotherapy-induced neuropathy on diabetic polyneuropathy	**Additional Examinations**<br>To further narrow down the differential diagnoses, the following additional examinations should be conducted:<br>1. **HbA1c and Fasting Blood Glucose Levels**<br>○ **Reasoning:** While the patient already has documented hyperglycemia and elevated HbA1c, repeat testing can provide updated information on current glycemic control. Tight glucose management is crucial in diabetic neuropathy management.<br>○ **Purpose:** Confirm and assess the severity of diabetes.<br>2. **Electromyography (EMG)**<br>○ **Reasoning:** EMG can provide additional details on the extent and type of nerve damage, helping to differentiate between axonal and demyelinating neuropathies.<br>○ **Purpose:** Determine the presence of muscle involvement and further characterize the neuropathy type.<br>3. **Serum Protein Electrophoresis with Immunofixation**<br>○ **Reasoning:** To rule out paraproteinemias or monoclonal gammopathies, which can be associated with peripheral neuropathies.<br>○ **Purpose:** Identify any monoclonal proteins that might indicate a plasma cell disorder or other hematological condition.<br>4. **Quantitative Sensory Testing (QST)**<br>○ **Reasoning:** QST assesses the functional status of sensory nerves, including small fiber function, which might not be fully captured in nerve conduction studies.<br>○ **Purpose:** Evaluate sensory nerve function in detail.<br>5. **Skin Biopsy**<br>○ **Reasoning:** Useful for diagnosing small fiber neuropathies by measuring intraepidermal nerve fiber density.<br>○ **Purpose:** Identify small fiber involvement, which is often seen in diabetic and chemotherapy-induced neuropathies.<br>6. **Liver Function Tests (LFTs)**<br>○ **Reasoning:** Chronic HBV infection can impact liver function, and any liver dysfunction could contribute to neuropathic symptoms.<br>○ **Purpose:** Assess liver function and rule out hepatic contribution to the neuropathy.<br>7. **Anti-Hu, Anti-Yo, and Anti-Ri Antibodies**<br>○ **Reasoning:** These paraneoplastic antibodies can be associated with neuropathies, particularly in patients with a history of cancer.<br>○ **Purpose:** Rule out paraneoplastic syndromes as a cause of the neuropathy.<br>8. **Thyroid Function Tests (TFTs)**<br>○ **Reasoning:** Hypothyroidism can cause peripheral neuropathy.<br>○ **Purpose:** Evaluate thyroid function to rule out thyroid dysfunction as a contributing factor.<br>**Most Crucial Test**<br>The most crucial test from this list is **Electromyography (EMG)**.<br>**Reasoning for Electromyography (EMG)**<br>● **Detailed Characterization:** EMG provides a detailed assessment of the type and extent of nerve damage, distinguishing between axonal and demyelinating neuropathies, which is critical in narrowing down the differential diagnosis.<br>● **Muscle Involvement:** It can also detect any muscle involvement, which could indicate a more widespread neuromuscular disorder.<br>● **Diagnostic Clarity:** EMG findings can help differentiate between diabetic polyneuropathy and chemotherapy-induced peripheral neuropathy, as both conditions can present similarly but have different management and prognostic implications.<br>By performing EMG, clinicians can obtain a more comprehensive understanding of the patient's neuropathy, leading to more targeted and effective management strategies.
28	A 37-year-old male patient presents with a 2-week history of sensory-motor, symmetrical, non-length dependent polyneuropathy with acute onset. The patient presents with distal paresthesia in the upper and lower limbs, neuropathic pain, and weakness in the proximal and distal areas of the upper and lower limbs. There are no ataxia, tremor, foot deformities, cranial nerve or autonomic involvement. Past medical history is silent. Nerve conduction studies show a demyelinating, sensory-motor polyneuropathy. Screening lab tests including vitamin B12, folate, vitamin B6, vitamin E, HCV, HBV, ANA, ANCA, ENA, CRP, ESR, glycemia, HbA1c, copper, cryoglobulins and serum electrophoresis are all negative. There is no family history of polyneuropathy.	GBS	**Step-by-Step Analysis of Additional Examinations**<br>**Patient Summary Review**<br>● **Patient Demographics:** 37-year-old male<br>● **Presenting Symptoms:**<br>○ Acute onset, 2-week history<br>○ Sensory-motor, symmetrical, non-length dependent polyneuropathy<br>○ Distal paresthesia in upper and lower limbs<br>○ Neuropathic pain<br>○ Weakness in both proximal and distal areas of upper and lower limbs<br>● **Clinical Findings:**<br>○ No ataxia, tremor, foot deformities, cranial nerve, or autonomic involvement<br>● **Past Medical History:** Unremarkable<br>● **Nerve Conduction Studies:** Demyelinating, sensory-motor polyneuropathy<br>● **Screening Lab Tests:** Negative (vitamin B12, folate, vitamin B6, vitamin E, HCV, HBV, ANA, ANCA, ENA, CRP, ESR, glycemia, HbA1c, copper, cryoglobulins, serum electrophoresis)<br>● **Family History:** No polyneuropathy<br>**Additional Examinations**<br>1. **Lumbar Puncture (LP)**<br>○ **Reasoning:**<br>■ **Cerebrospinal Fluid (CSF) Analysis:** Important for diagnosing Guillain-Barré Syndrome (GBS). Typically, elevated protein levels with normal cell count (albumin-cytological dissociation) are observed.<br>■ **Exclusion of Other Conditions:** Can help rule out infections or other inflammatory conditions affecting the nervous system.<br>2. **MRI of the Spine**<br>○ **Reasoning:**<br>■ **Imaging of Nerve Roots and Spinal Cord:** Can identify nerve root enhancement, which is often seen in GBS.<br>■ **Rule Out Structural Causes:** Ensures there are no compressive or structural lesions contributing to the neuropathy.<br>3. **Repetitive Nerve Stimulation (RNS) Test**

			○ **Reasoning:**<br>■ **Neuromuscular Junction Disorders:** Helps exclude conditions like myasthenia gravis or Lambert-Eaton myasthenic syndrome, although less likely given the clinical presentation.<br>4. **Anti-GM1 and Other Ganglioside Antibodies**<br>○ **Reasoning:**<br>■ **GBS and CIDP:** These antibodies are often elevated in patients with GBS and some subtypes of CIDP. Presence of these antibodies can support the diagnosis.<br>5. **Serum Immunofixation Electrophoresis (IFE) and Free Light Chains**<br>○ **Reasoning:**<br>■ **Monoclonal Gammopathies:** Important for ruling out conditions like POEMS syndrome or other paraproteinemias that can present with demyelinating polyneuropathy.<br>6. **HIV Testing**<br>○ **Reasoning:**<br>■ **Infectious Etiologies:** Although initial viral screenings were negative, HIV can present with neuropathic symptoms and should be ruled out.<br>7. **Paraneoplastic Panel**<br>○ **Reasoning:**<br>■ **Paraneoplastic Syndromes:** These can present with neuropathies, and identifying any associated antibodies can guide further cancer screening and diagnosis.<br>**Determining the Most Crucial Test**<br>**Lumbar Puncture (LP)**<br>● **Reasoning:** The most crucial test in this scenario is the lumbar puncture for CSF analysis. Given the acute onset of symptoms and the demyelinating nature observed in nerve conduction studies, confirming the presence of albumin-cytological dissociation would be highly indicative of Guillain-Barré Syndrome (GBS). This test is pivotal because it can strongly support the diagnosis of GBS, allowing for prompt initiation of treatment with IV immunoglobulins or plasmapheresis, which can significantly impact the patient's prognosis.
29	A 75-year-old male patient presents with a 4-year history of pure sensory, symmetrical, length dependent polyneuropathy with insidious onset and slow progressive course. The patient presents with paresthesia and neuropathic pain in the feet. Clinical examination does not showtremor or sensory ataxia. There are no signs of foot deformities, cranial nerve or autonomic involvement. Past medical history is positive for type 2 diabetes and arterial hypertension. Nerve conduction studies show a mixed sensory-motor, length dependent, axonal polyneuropathy. Screening lab tests reveal hyperglycaemia and elevated HbA1c. Remaining exams including vitamin B12, vitamin B6, vitamin E, HBV, HCV, ANA, ANCA, ENA, CRP, ESR, copper, cryoglobulin, and serum electrophoresis are normal. There is no family history of polyneuropathy.	Diabetic neuropathy	**Step-by-Step Clinical Analysis for Additional Examinations**<br>**1. Review of Pertinent Clinical Information:**<br>● **Patient:** 75-year-old male<br>● **History Duration:** 4 years<br>● **Symptoms:** Pure sensory, symmetrical, length-dependent polyneuropathy with paresthesia and neuropathic pain in the feet<br>● **Clinical Examination:** No tremor, sensory ataxia, foot deformities, cranial nerve or autonomic involvement<br>● **Past Medical History:** Type 2 diabetes and arterial hypertension<br>● **Nerve Conduction Studies:** Mixed sensory-motor, length-dependent, axonal polyneuropathy<br>● **Screening Lab Results:** Hyperglycaemia, elevated HbA1c, normal levels of vitamin B12, vitamin B6, vitamin E, HBV, HCV, ANA, ANCA, ENA, CRP, ESR, copper, cryoglobulin, and serum electrophoresis<br>● **Family History:** No family history of polyneuropathy<br>**2. Additional Examinations to Narrow Down Differential Diagnoses:**<br>1. **Glucose Tolerance Test (Oral Glucose Tolerance Test - OGTT):**<br>○ **Reasoning:** This test assesses how well the body processes glucose and can help confirm or rule out glucose intolerance or pre-diabetes, which can contribute to neuropathy. Given the patient's known diabetes, it can provide additional data on glucose metabolism.<br>2. **Serum Protein Electrophoresis with Immunofixation:**<br>○ **Reasoning:** Although initial serum electrophoresis was normal, immunofixation can detect low levels of monoclonal proteins that might be missed otherwise. This is crucial for ruling out monoclonal gammopathies such as MGUS, multiple myeloma, or POEMS syndrome, which can present with polyneuropathy.<br>3. **Thyroid Function Tests (TFTs):**<br>○ **Reasoning:** Hypothyroidism can cause polyneuropathy. Assessing thyroid function through TSH, Free T4, and Free T3 can rule out this potential cause. The patient's age and diabetes history increase the relevance of evaluating thyroid function.<br>4. **CSF Analysis (Lumbar Puncture):**<br>○ **Reasoning:** CSF analysis can help in diagnosing inflammatory or infectious causes of polyneuropathy such as chronic inflammatory demyelinating polyneuropathy (CIDP), Guillain-Barré syndrome variants, or other central nervous system infections. Elevated protein without pleocytosis can indicate CIDP.<br>5. **Nerve Biopsy:**<br>○ **Reasoning:** In cases where the diagnosis remains unclear, a nerve biopsy can provide histopathological confirmation of axonal degeneration or demyelination and help identify vasculitic, amyloid, or infiltrative neuropathies.<br>6. **MRI of the Spine:**<br>○ **Reasoning:** MRI can help rule out spinal stenosis or other structural causes that could compress nerve roots and mimic polyneuropathy symptoms.<br>7. **HbA1c Re-test and Continuous Glucose Monitoring (CGM):**<br>○ **Reasoning:** Reassessing long-term glycemic control and variability in blood sugar levels can provide a better understanding of the patient's diabetes management and its impact on neuropathy.<br>8. **Autonomic Testing:**<br>○ **Reasoning:** Even though there are no clinical signs of autonomic involvement, autonomic function tests (e.g., heart rate variability, tilt-table test) can detect subclinical autonomic neuropathy, which is common in diabetic patients.<br>**3. Determining the Most Crucial Test:**<br>Given the comprehensive initial workup and the specifics of this case, the most crucial additional test would be **Serum Protein Electrophoresis with Immunofixation**. This test is pivotal because:<br>● It provides a definitive exclusion of monoclonal gammopathies, which can present with similar neuropathic symptoms.<br>● The patient's age and the chronic nature of his symptoms necessitate thorough evaluation for underlying malignancies or pre-malignant conditions that can be subtle yet serious causes of polyneuropathy.<br>**Conclusion**<br>While multiple additional examinations can provide valuable information, **Serum Protein Electrophoresis with Immunofixation** stands out as the most crucial test. This test is essential to rule out monoclonal gammopathies and ensure that the polyneuropathy is not due to a treatable underlying malignancy or related condition.
30	A 60-year-old male patient presents with a 17-month history of sensory, symmetrical, length dependent polyneuropathy with an insidious onset and a slow progressive	Amyloidosis associated polyneuropathy	**Step-by-Step Analysis of the Clinical Scenario**<br>**Clinical Data Recap:**<br>● **Patient**: 60-year-old male<br>● **Symptoms**: 17-month history of sensory, symmetrical, length-dependent polyneuropathy, neuropathic pain, erectile dysfunction, hyperhidrosis, mild distal paraesthesia

	course. The first occurred symptom was neuropathic pain in the feet, followed by erectile dysfunction, hyperhidrosis and mild distal paraesthesia. Clinical examination does not show presence of sensory ataxia, tremor or foot deformities. Past medical history is silent. Nerve conduction studies show a mixed sensory-motor, symmetric, axonal polyneuropathy and bilateral carpal tunnel syndrome. Screening lab tests including vitamin B12, folate, vitamin B6, vitamin E, HBV, HCV, ANA, ANCA, ENA, CRP, ESR, glycemia, HbA1c, copper, and cryoglobulins are normal. Serum electrophoresis shows presence of IgG lambda paraprotein. There is no family history of polyneuropathy.		● **Clinical Examination**: No sensory ataxia, tremor, or foot deformities<br>● **Nerve Conduction Studies**: Mixed sensory-motor, symmetric, axonal polyneuropathy and bilateral carpal tunnel syndrome<br>● **Lab Results**: Normal (various), except for IgG lambda paraprotein on serum electrophoresis<br>● **Family History**: No polyneuropathy<br>**Necessary Additional Examinations**<br>1. **Bone Marrow Biopsy**<br>○ **Reasoning**:<br>■ To evaluate for plasma cell disorders like multiple myeloma or amyloidosis, which are potential causes of monoclonal gammopathy.<br>■ Bone marrow examination can confirm the presence of abnormal plasma cells and help differentiate MGUS from more severe conditions like multiple myeloma or amyloidosis.<br>2. **Serum Free Light Chain Assay**<br>○ **Reasoning**:<br>■ To quantify free light chains (kappa and lambda) in the serum, which can help in diagnosing conditions like light chain amyloidosis (AL amyloidosis).<br>■ An abnormal kappa/lambda ratio can support the diagnosis of plasma cell dyscrasias or amyloidosis.<br>3. **24-Hour Urine Protein Electrophoresis (UPEP)**<br>○ **Reasoning**:<br>■ To detect Bence Jones proteins, which are free light chains excreted in the urine and associated with multiple myeloma and AL amyloidosis.<br>■ UPEP can help in identifying renal involvement and further characterizing the paraprotein.<br>4. **Nerve Biopsy**<br>○ **Reasoning**:<br>■ To obtain a definitive diagnosis if amyloidosis is suspected.<br>■ Nerve biopsy can reveal amyloid deposits in the nerves and help confirm or exclude amyloid neuropathy.<br>5. **Electrocardiogram (ECG) and Echocardiogram**<br>○ **Reasoning**:<br>■ To assess for cardiac involvement, which can occur in amyloidosis and is crucial for prognosis and management.<br>■ Cardiac amyloidosis can manifest with specific ECG changes and cardiac dysfunction detectable on echocardiogram.<br>6. **Autonomic Function Testing**<br>○ **Reasoning**:<br>■ Given the presence of erectile dysfunction and hyperhidrosis, autonomic function tests can help assess the extent of autonomic nervous system involvement.<br>■ Tests may include heart rate variability, tilt-table testing, and sudomotor function testing.<br>7. **Genetic Testing for Hereditary Neuropathies**<br>○ **Reasoning**:<br>■ Despite the lack of family history, genetic mutations could still play a role.<br>■ Testing for hereditary neuropathies, such as hereditary transthyretin amyloidosis, can be considered based on the clinical picture and exclusion of other causes.<br>**Most Crucial Test**<br>**Bone Marrow Biopsy**<br>**Reasoning**:<br>● Given the presence of IgG lambda paraprotein, a bone marrow biopsy is crucial to determine the underlying cause of the monoclonal gammopathy.<br>● It can help differentiate between MGUS, multiple myeloma, and amyloidosis.<br>● A biopsy will provide detailed information about plasma cell infiltration, which is critical for diagnosing and managing the patient's condition.<br>**Conclusion**<br>A thorough analysis of the patient's clinical scenario necessitates several additional examinations to narrow down the differential diagnoses. Among these, the bone marrow biopsy stands out as the most crucial test. It directly addresses the potential underlying plasma cell disorder associated with the IgG lambda paraprotein, thereby guiding further diagnostic and therapeutic decisions.
31	A 54-year-old male patient presents with a 2-year history of chronic, pure sensory, symmetrical, length dependent polyneuropathy with insidious onset. The patient presents with distal paresthesia and neuropathic pain in the lower limbs, without ataxia. Past medical history reveals high alcohol consumption. Clinical examination reveals absence of foot deformities, tremor, cranial nerve or autonomic involvement. Nerve conduction studies show a length-dependent polyneuropathy with symmetric, pure sensory, axonal involvement. Screening lab tests including vitamin B12, folate, vitamin B6, vitamin E, HCV, HBV, ANA, ANCA, ENA, CRP, ESR, glycemia, HbA1c, copper, cryoglobulins and serum electrophoresis are all negative. Serum gamma-glutamyl transferase (GGT) levels are elevated.	Alcohol-associated neuropathy	**Step-by-Step Analysis and Additional Examinations**<br>**Step 1: Review and Summary**<br>**Patient Profile**: 54-year-old male with chronic, pure sensory, symmetrical, length-dependent polyneuropathy. **History**: High alcohol consumption. **Clinical Findings**:<br>● Distal paresthesia and neuropathic pain in the lower limbs.<br>● No ataxia, no foot deformities, no tremor, no cranial nerve or autonomic involvement. **Nerve Conduction Studies**: Length-dependent, symmetric, pure sensory, axonal polyneuropathy. **Initial Labs**: Negative for vitamin B12, folate, vitamin B6, vitamin E, HCV, HBV, ANA, ANCA, ENA, CRP, ESR, glycemia, HbA1c, copper, cryoglobulins, serum electrophoresis. **Positive Lab Finding**: Elevated serum gamma-glutamyl transferase (GGT) levels. **Family History**: No history of polyneuropathy.<br>**Step 2: Identify Possible Differential Diagnoses**<br>1. **Alcoholic Polyneuropathy**<br>2. **Idiopathic Sensory Polyneuropathy**<br>3. **Toxic Neuropathy (Non-Alcoholic)**<br>**Step 3: List Additional Examinations**<br>1. **Thiamine (Vitamin B1) Level**<br>○ **Reasoning**: Chronic alcohol consumption can lead to thiamine deficiency, contributing to peripheral neuropathy. Measuring thiamine levels can help confirm if a deficiency is present and contributing to the neuropathy.<br>○ **Specifics**: Thiamine deficiency is common in alcoholics due to poor nutritional intake and impaired absorption.<br>2. **Liver Function Tests (LFTs)**<br>○ **Reasoning**: Alcoholic liver disease can contribute to polyneuropathy. A comprehensive liver function panel, including AST, ALT, alkaline phosphatase, and bilirubin, can provide more insight into liver health.<br>○ **Specifics**: Elevated GGT suggests liver involvement; other liver enzymes can provide a more complete picture of liver function.<br>3. **Heavy Metal Screening (e.g., Lead, Arsenic)**<br>○ **Reasoning**: Chronic exposure to heavy metals can cause toxic neuropathy. Screening for these can rule out non-alcoholic toxic causes.

	There is no family history of polyneuropathy.		○ **Specifics**: Heavy metal exposure can be insidious and present with similar neuropathic symptoms.<br>4. **Serum Protein Electrophoresis and Immunofixation Electrophoresis**<br>○ **Reasoning**: To detect monoclonal gammopathies or multiple myeloma, which can present with similar neuropathy.<br>○ **Specifics**: While initial screening was negative, more specific tests could detect subtle abnormalities.<br>5. **Autonomic Function Tests**<br>○ **Reasoning**: To rule out subclinical autonomic neuropathy which might not be apparent on clinical examination but could be present in cases of chronic alcohol consumption.<br>○ **Specifics**: Autonomic tests include heart rate variability, blood pressure response to standing, and sweating tests.<br>6. **Genetic Testing for Hereditary Neuropathies**<br>○ **Reasoning**: While there's no family history, genetic mutations can occur de novo. Testing can rule out rare hereditary conditions.<br>○ **Specifics**: Tests would include sequencing for common genes involved in hereditary sensory and autonomic neuropathies (HSAN).<br>7. **MRI of the Brain and Spinal Cord**<br>○ **Reasoning**: To rule out structural causes of neuropathy and to ensure there is no central nervous system involvement mimicking peripheral neuropathy.<br>○ **Specifics**: Useful in excluding other causes like multiple sclerosis or spinal cord compression.<br>8. **Quantitative Sensory Testing (QST)**<br>○ **Reasoning**: To objectively measure sensory thresholds and better characterize the sensory involvement.<br>○ **Specifics**: Helps in differentiating between different types of sensory neuropathy (e.g., large vs. small fiber).<br>**Step 4: Determine the Most Crucial Test**<br>**Most Crucial Test: Thiamine (Vitamin B1) Level**<br>**Reasoning**: Given the patient's significant history of alcohol consumption and the known association between alcohol use and thiamine deficiency leading to peripheral neuropathy, measuring thiamine levels is critical. This test is highly specific to the patient's likely condition (Alcoholic Polyneuropathy) and has direct implications for treatment. Correcting a thiamine deficiency can potentially halt progression and improve symptoms, making it the most immediate and impactful test.<br>**Conclusion**<br>The most crucial additional examination is the **Thiamine (Vitamin B1) Level** test. This will help confirm or rule out a significant cause of neuropathy in a patient with a history of high alcohol consumption, guiding appropriate treatment and management.
32	An 80-year-old male patient presents with a 3-year history of neuropathic pain and skin redness in the hands and feet. Symptoms started insidiously but progressed over the years. Clinical examination reveals hyperalgesia and allodynia in the hands and feet, where the skin is warm and red. There are no weakness, deficit in tactile sensation, sensory ataxia, tremor, foot deformities, cranial nerve or autonomic involvement. Past medical history is positive for prostate cancer and chronic renal insufficiency. Nerve conduction studies are normal. Screening lab tests including vitamin B12, folate, vitamin B6, vitamin E, HBV, HCV, ANA, ANCA, ENA, CRP, ESR, glycemia, HbA1c, copper, cryoglobulins and serum electrophoresis are negative. There is no family history of polyneuropathy.	Small fibre neuropathy	**Step-by-Step Analysis of Additional Examinations**<br>**Step 1: Comprehensive Clinical Re-evaluation**<br>A thorough re-evaluation of the patient's history and physical examination should be conducted to ensure no details have been missed. This includes:<br>● Detailed history of symptom triggers and alleviating factors.<br>● Comprehensive physical examination focusing on other areas potentially affected.<br>**Step 2: Additional Laboratory Tests**<br>1. **Skin Biopsy for Intraepidermal Nerve Fiber Density (IENFD)**<br>○ **Reasoning:** This test can confirm small fiber neuropathy by quantifying the density of small nerve fibers in the skin. Reduced nerve fiber density is diagnostic for SFN.<br>2. **Serum and Urine Protein Electrophoresis with Immunofixation**<br>○ **Reasoning:** To rule out monoclonal gammopathies like multiple myeloma or amyloidosis, which can cause neuropathy. The patient's age and history of cancer necessitate this test.<br>3. **Anti-Hu and Anti-Ri Antibodies**<br>○ **Reasoning:** These antibodies can be associated with paraneoplastic syndromes, particularly in patients with a history of cancer. Neuropathy can be a manifestation of such syndromes.<br>4. **Celiac Disease Screening (Tissue Transglutaminase Antibodies)**<br>○ **Reasoning:** Celiac disease can present with neuropathy even in the absence of gastrointestinal symptoms.<br>5. **Vitamin D Levels**<br>○ **Reasoning:** Vitamin D deficiency has been associated with neuropathic pain and might contribute to the patient's symptoms.<br>6. **Lyme Disease Serology (ELISA followed by Western Blot)**<br>○ **Reasoning:** Lyme disease can present with neuropathy, and considering the patient's age and symptoms, it's prudent to rule out.<br>**Step 3: Additional Instrumental Tests**<br>1. **Quantitative Sensory Testing (QST)**<br>○ **Reasoning:** QST assesses the function of small and large nerve fibers and can help in confirming the diagnosis of SFN by evaluating pain and temperature sensation.<br>2. **Autonomic Function Tests**<br>○ **Reasoning:** Although there is no apparent autonomic involvement, these tests can uncover subclinical autonomic dysfunction, which might support a diagnosis of SFN or other neuropathies.<br>3. **Thermography**<br>○ **Reasoning:** Thermography can help document the warm and red areas in hands and feet, providing objective evidence for disorders like erythromelalgia.<br>4. **Skin Temperature Measurement and Blood Flow Studies**<br>○ **Reasoning:** These tests can help evaluate erythromelalgia by measuring skin temperature and blood flow changes in response to various stimuli.<br>**Step 4: Imaging Studies**<br>1. **MRI of Brain and Spine**<br>○ **Reasoning:** To rule out central causes of neuropathy, although less likely in this case, considering the normal neurological examination findings.<br>**Step 5: Genetic Testing**<br>1. **Genetic Testing for SCN9A and SCN10A Mutations**<br>○ **Reasoning:** These mutations are associated with inherited erythromelalgia. Given the symptoms, ruling out a genetic cause could be beneficial.<br>**Most Crucial Test**<br>**Skin Biopsy for Intraepidermal Nerve Fiber Density (IENFD)** is the most crucial test from the list. It provides direct evidence for small fiber neuropathy, which is highly suspected given the clinical presentation and normal nerve conduction studies. Confirming SFN will guide further management and potentially narrow down the etiology based on additional test results.<br>**Conclusion**<br>The skin biopsy for IENFD is the pivotal test to confirm small fiber neuropathy, which aligns with the patient's symptoms and normal nerve conduction study results. Subsequent tests should then be guided by the results of this biopsy, focusing on identifying underlying causes.

33	A 24-year old male patient presents with a 4-year history of sensory-motor, symmetrical, length dependent polyneuropathy with insidious onset and slow progression. The patient presents with severe walking difficulties, bilateral foot-drop, severe distal atrophy in the lower limbs, and hypoesthesia up to calves. There is no cranial nerve or autonomic involvement. Past medical history is silent. Nerve conduction studies show a mixed axonal-demyelinating, symmetric, sensory-motor polyneuropathy. Screening lab tests including vitamin B12, folate, vitamin B6, vitamin E, HBV, HCV, glycemia, HbA1c, ANA, ANCA, ENA, CRP, ESR, copper, cryoglobulins, and serum electrophoresis are all negative. His mother and sister have mild walking difficulties.	X-linked CMT	**Step-by-Step Analysis for Additional Examinations**<br>**1. Additional Laboratory Tests:**<br>**A. Genetic Testing for Charcot-Marie-Tooth Disease (CMT)**<br>● **Reasoning:** Given the strong family history and clinical presentation, genetic testing can identify specific mutations associated with different types of CMT (e.g., PMP22, GJB1, MPZ, MFN2).<br>● **Purpose:** Confirm diagnosis of CMT, subtype classification for prognostic and management purposes.<br>**B. Nerve Biopsy**<br>● **Reasoning:** Nerve biopsy can provide histological evidence of demyelination, axonal loss, and other structural changes.<br>● **Purpose:** Differentiate between hereditary and acquired neuropathies, and provide supportive evidence for a diagnosis like CMT or CIDP.<br>**C. Cerebrospinal Fluid (CSF) Analysis**<br>● **Reasoning:** Elevated protein levels without pleocytosis can suggest CIDP, whereas normal CSF may support a hereditary neuropathy.<br>● **Purpose:** Help differentiate CIDP from hereditary neuropathies and other acquired polyneuropathies.<br>**D. Anti-GM1 Antibodies and Other Paraneoplastic Panels**<br>● **Reasoning:** Anti-GM1 antibodies are sometimes present in CIDP and multifocal motor neuropathy. Paraneoplastic syndromes can also present with neuropathy.<br>● **Purpose:** Rule out autoimmune and paraneoplastic etiologies.<br>**2. Additional Instrumental Tests:**<br>**A. MRI of Spine and Brachial/Lumbosacral Plexus**<br>● **Reasoning:** MRI can identify hypertrophy of peripheral nerves, root enhancement, and other structural abnormalities.<br>● **Purpose:** Assess for CIDP or other compressive/infiltrative causes of neuropathy.<br>**B. High-Resolution Ultrasound of Peripheral Nerves**<br>● **Reasoning:** Can detect nerve enlargement, focal nerve abnormalities, and differentiate between demyelinating and axonal neuropathies.<br>● **Purpose:** Non-invasive assessment of nerve pathology, especially useful in hereditary neuropathies.<br>**C. Somatosensory Evoked Potentials (SSEPs)**<br>● **Reasoning:** SSEPs can assess the functional integrity of the sensory pathways.<br>● **Purpose:** Evaluate the extent and distribution of sensory pathway involvement, providing supportive evidence for conditions like CMT or CIDP.<br>**3. Evaluation of Most Crucial Test:**<br>**Most Crucial Test: Genetic Testing for Charcot-Marie-Tooth Disease (CMT)**<br>● **Reasoning:** The combination of the clinical presentation, strong family history, and nerve conduction study findings strongly suggest a hereditary neuropathy, particularly CMT. Genetic testing is definitive for confirming the diagnosis of CMT and identifying the specific subtype, which is critical for prognosis and management. It also has implications for family counseling and potential future therapies.<br>**Summary of Recommended Tests:**<br>1. **Genetic Testing for CMT** (most crucial)<br>2. Nerve Biopsy<br>3. CSF Analysis<br>4. Anti-GM1 Antibodies and Paraneoplastic Panels<br>5. MRI of Spine and Brachial/Lumbosacral Plexus<br>6. High-Resolution Ultrasound of Peripheral Nerves<br>7. Somatosensory Evoked Potentials (SSEPs)<br>**Conclusion:**<br>Genetic testing for Charcot-Marie-Tooth Disease is the most crucial test in this clinical scenario, as it provides definitive evidence for a hereditary neuropathy, which aligns closely with the patient's clinical and family history.
34	An 83-year-old male patient presents with a 1-year history of pure sensory, symmetrical, length dependent polyneuropathy with insidious onset and progressive course. The patient presents with distal paresthesia and hypoesthesia in the four limbs, mild hand tremor and cramps but no neuropathic pain. He also reports the appearance of gait instability in the past few months. Clinical examination confirms the presence of sensory ataxia with difficulty walking without support. There are no foot deformities, cranial nerve or autonomic involvement. Past medical history shows IgG kappa monoclonal gammopathy, thrombocytopenia, and prostatic cancer. Nerve conduction studies show a pure sensory, symmetric, length-dependent, axonal polyneuropathy. Screening lab tests show the presence of IgG kappa paraprotein and evidence of vitamin B12 deficiency. Remaining exams including folate, vitamin B6, vitamin E, HCV, HBV, ANA, ENA, ANCA, CRP, ESR, glycemia,	Vit B12 deficiency neuropathy	**Step-by-Step Approach to Additional Examinations**<br>**Step 1: Review of the Provided Data**<br>1. **Patient Demographics**: 83-year-old male<br>2. **Symptoms**: Sensory, symmetrical, length-dependent polyneuropathy; distal paresthesia and hypoesthesia; mild hand tremor and cramps; gait instability; sensory ataxia.<br>3. **Past Medical History**: IgG kappa monoclonal gammopathy, thrombocytopenia, prostatic cancer.<br>4. **Diagnostic Tests**: Nerve conduction studies indicate pure sensory, symmetric, length-dependent, axonal polyneuropathy; presence of IgG kappa paraprotein; vitamin B12 deficiency; other labs negative.<br>5. **Clinical Findings**: No cranial nerve or autonomic involvement; no family history of polyneuropathy.<br>**Step 2: Identify Knowledge Gaps**<br>The existing data provide a strong basis for the differential diagnosis, but some gaps remain, particularly in ruling out other potential causes of neuropathy. Additional tests can help refine the diagnosis by excluding other conditions or confirming suspicions.<br>**Step 3: List of Necessary Additional Examinations**<br>1. **Serum Free Light Chains (FLC) Assay**:<br>○ **Reasoning**: To further evaluate the monoclonal gammopathy. Abnormal FLC ratios could suggest a progression from MGUS to multiple myeloma or other plasma cell disorders.<br>○ **Specifics**: MGUS can sometimes progress to more serious hematologic conditions which can cause neuropathy.<br>2. **Serum Immunofixation Electrophoresis (IFE)**:<br>○ **Reasoning**: To confirm the presence and type of monoclonal protein. It provides more detailed information on monoclonal proteins compared to standard electrophoresis.<br>○ **Specifics**: Helps differentiate MGUS from more aggressive forms of plasma cell dyscrasias.<br>3. **Bone Marrow Biopsy**:<br>○ **Reasoning**: To rule out multiple myeloma or other hematologic malignancies given the history of monoclonal gammopathy and thrombocytopenia.<br>○ **Specifics**: Useful in confirming the diagnosis of a plasma cell disorder or other hematological abnormalities.<br>4. **Lumbar Puncture with Cerebrospinal Fluid (CSF) Analysis**:<br>○ **Reasoning**: To exclude inflammatory or infectious causes of neuropathy and to check for the presence of oligoclonal bands which might suggest a paraneoplastic or autoimmune process.<br>○ **Specifics**: Can help detect conditions like chronic inflammatory demyelinating polyneuropathy (CIDP) or infections.<br>5. **Anti-Hu and Other Paraneoplastic Antibody Panel**:<br>○ **Reasoning**: To identify paraneoplastic syndromes, which can present with sensory neuropathies in the context of malignancy.

	HbA1c, copper and cryoglobulins are all negative. There is no family history of polyneuropathy.		○ **Specifics**: Given the history of prostatic cancer, ruling out a paraneoplastic etiology is crucial.<br>6. **Skin Biopsy**:<br>○ **Reasoning**: To assess small fiber neuropathy, which might not be detected on standard nerve conduction studies.<br>○ **Specifics**: Useful in diagnosing small fiber involvement which can cause sensory symptoms.<br>7. **Re-assessment of Vitamin B12 with Methylmalonic Acid (MMA) and Homocysteine Levels**:<br>○ **Reasoning**: To confirm the functional deficiency of B12, as elevated levels of MMA and homocysteine are more sensitive indicators of B12 deficiency than serum B12 levels alone.<br>○ **Specifics**: Confirms whether the neuropathy is related to B12 deficiency.<br>8. **Magnetic Resonance Imaging (MRI) of the Spine**:<br>○ **Reasoning**: To rule out compressive lesions or other structural abnormalities that might contribute to sensory ataxia and gait instability.<br>○ **Specifics**: Especially useful if there are any signs suggesting spinal involvement.<br>**Step 4: Determine the Most Crucial Test**<br>**Most Crucial Test: Bone Marrow Biopsy**<br>● **Reasoning**: Given the patient's age, history of IgG kappa monoclonal gammopathy, thrombocytopenia, and prostatic cancer, ruling out multiple myeloma or other hematologic malignancies is paramount. Bone marrow biopsy will provide definitive information on the status of the plasma cells and help determine if there is a progression from MGUS to multiple myeloma or other conditions, directly influencing management and prognosis.<br>**Conclusion**<br>The comprehensive analysis of the patient's clinical scenario and the recommended additional examinations focuses on narrowing down the differential diagnosis and confirming the primary underlying cause of the polyneuropathy. The bone marrow biopsy stands out as the most crucial test to perform next, given its potential to definitively rule out or confirm multiple myeloma or other hematologic conditions that could significantly impact the patient's treatment plan.
35	An 82-year-old male patient presents with a 3-year history of pure sensory, symmetrical, length dependent polyneuropathy with insidious onset and slow progressive course. The patient presents with paraesthesia and hypoesthesia in the feet. Clinical examination reveals hyperalgesia during pinprick testing and tactile hypoesthesia in the feet, while there are no weakness, sensory ataxia, tremor, foot deformities, cranial nerve or autonomic dysfunction. Past medical history is positive for IgG kappa MGUS, prostate and bladder cancer, for which he underwent chemotherapy five years ago. Nerve conduction studies show a sensory-motor, symmetric, length dependent, axonal polyneuropathy. Screening lab tests reveal presence of IgG kappa paraprotein. Remaining exams including vitamin B12, folate, vitamin B6, vitamin E, HBV, HCV, glycaemia, HbA1c, ANA, ANCA, ENA, CRP, ESR, copper ,and cryoglobulins are normal. There is no family history of polyneuropathy.	Paraprotein emic neuropathy	**Step-by-Step Approach to Additional Examinations**<br>1. **Repeat Serum Protein Electrophoresis and Immunofixation**<br>○ **Reasoning:**<br>■ To confirm the presence and quantify the level of IgG kappa paraprotein.<br>■ Evaluate the stability or progression of MGUS, as a rise in paraprotein levels may suggest progression to a more significant plasma cell disorder.<br>■ This test is crucial because an increase in paraprotein levels can correlate with more severe neuropathy and may change the clinical management.<br>2. **Anti-MAG (Myelin-Associated Glycoprotein) Antibodies**<br>○ **Reasoning:**<br>■ Anti-MAG antibodies are often present in patients with neuropathy associated with MGUS.<br>■ Detection of these antibodies can help to confirm the diagnosis of paraproteinemic neuropathy and distinguish it from other causes.<br>■ These antibodies specifically target myelin and can lead to a predominantly sensory neuropathy, matching the patient's symptoms.<br>3. **Lumbar Puncture with Cerebrospinal Fluid (CSF) Analysis**<br>○ **Reasoning:**<br>■ CSF analysis can help exclude other causes of polyneuropathy such as chronic inflammatory demyelinating polyneuropathy (CIDP) or central nervous system involvement.<br>■ Presence of a high protein level without pleocytosis (albuminocytologic dissociation) can support the diagnosis of CIDP or other inflammatory neuropathies.<br>■ Oligoclonal bands in the CSF might indicate an immune-mediated process.<br>4. **Autonomic Function Testing**<br>○ **Reasoning:**<br>■ Although the patient does not currently have autonomic symptoms, comprehensive autonomic testing can uncover subclinical autonomic involvement.<br>■ This would be particularly important if considering the broader spectrum of neuropathies associated with monoclonal gammopathies.<br>■ Tests such as heart rate variability, blood pressure response to tilt-table testing, and quantitative sudomotor axon reflex testing can provide insights into the autonomic nervous system status.<br>5. **Skin Biopsy**<br>○ **Reasoning:**<br>■ To assess intraepidermal nerve fiber density, which can provide direct evidence of small fiber neuropathy.<br>■ This test is minimally invasive and can detect small fiber involvement that may not be evident on nerve conduction studies.<br>■ Useful for diagnosing neuropathies where large fiber studies are inconclusive but clinical symptoms suggest small fiber involvement.<br>6. **Serum Free Light Chain Assay**<br>○ **Reasoning:**<br>■ To measure the levels of free kappa and lambda light chains in the serum.<br>■ This test can help detect light chain deposition diseases and provide further evidence of plasma cell dyscrasias.<br>■ Abnormal ratios or high levels can suggest progression from MGUS to smoldering multiple myeloma or multiple myeloma.<br>**Most Crucial Test**<br>**Anti-MAG (Myelin-Associated Glycoprotein) Antibodies**<br>● **Reasoning:**<br>○ Given the patient's history of MGUS and the presence of IgG kappa paraprotein, testing for anti-MAG antibodies is most crucial.<br>○ Anti-MAG antibodies are specifically associated with MGUS-related neuropathy and can provide a definitive link between the patient's neuropathy and their monoclonal gammopathy.<br>○ Detection of these antibodies would confirm the diagnosis of MGUS-associated neuropathy and guide appropriate treatment strategies.<br>By focusing on this specific test, the diagnosis can be clarified and treatment tailored to the underlying etiology, potentially improving patient outcomes.
36	A 75-year-old male patient presents with a four-year history of pure sensory, symmetrical, length-dependent polyneuropathy with insidious onset and slow progression. The patient reports sensory ataxia, hypoesthesia in the distal lower limbs up to the calves, mild dysphagia and dysarthria, with no distal	CANVAS	**Step-by-Step Analysis for Additional Examinations**<br>Given the clinical scenario of a 75-year-old male with sensory, symmetrical, length-dependent polyneuropathy and positive ENA autoantibodies, additional examinations are necessary to narrow down the differential diagnoses. Here is a structured approach:<br>1. **Serological Tests for Specific Autoantibodies**<br>○ **Anti-Ro/SSA and Anti-La/SSB Antibodies:** These are specific for Sjögren's Syndrome.<br>■ **Reasoning:** The presence of ENA autoantibodies suggests an autoimmune etiology. Sjögren's Syndrome commonly presents with these antibodies and could explain the patient's neuropathy and symptoms such as dysphagia and dysarthria.<br>2. **Salivary Gland Biopsy (Lip Biopsy)**

	weakness. Past medical history is positive for chronic cough. Nerve conduction studies show an axonal, symmetric, length-dependent, pure sensory polyneuropathy. Screening lab tests, including vitamin B12, folate, vitamin B6, vitamin E, HBV, HCV, glycemia, HbA1c, ANA, ANCA, CRP, ESR, copper, cryoglobulins, and serum electrophoresis are all negative, with positivity for ENA autoantibodies. There is no family history of polyneuropathy.		○ **Reasoning:** A minor salivary gland biopsy can confirm the diagnosis of Sjögren's Syndrome by showing focal lymphocytic sialadenitis. This is a highly specific test for PSS, especially when serology is equivocal or requires confirmation.<br>3. **Chest Imaging (Chest X-ray or CT Scan)**<br>○ **Reasoning:** Given the chronic cough and the patient's age, it is important to rule out an underlying malignancy, such as lung cancer, which could be associated with paraneoplastic neuropathy. Chest imaging can identify masses, lymphadenopathy, or other abnormalities.<br>4. **CT or PET Scan for Whole Body Screening**<br>○ **Reasoning:** To further investigate for hidden malignancies that might cause paraneoplastic neuropathy. This helps in identifying primary tumors or metastatic disease not evident on initial imaging.<br>5. **Lumbar Puncture with Cerebrospinal Fluid (CSF) Analysis**<br>○ **Reasoning:** CSF analysis can help in diagnosing chronic inflammatory conditions like CIDP. Elevated protein levels with a normal cell count (albuminocytologic dissociation) are typical findings in CIDP.<br>6. **Schirmer's Test and Ocular Surface Staining**<br>○ **Reasoning:** These tests help to confirm dry eye symptoms indicative of Sjögren's Syndrome. They measure tear production and ocular surface damage, respectively.<br>7. **Pulmonary Function Tests (PFTs)**<br>○ **Reasoning:** To evaluate the extent of lung involvement, particularly in the context of Sjögren's Syndrome, which can cause interstitial lung disease or other pulmonary manifestations.<br>8. **Repetitive Nerve Stimulation or Single Fiber EMG**<br>○ **Reasoning:** These tests can help rule out myasthenia gravis, which can present with dysphagia and dysarthria. Though less likely, this should be considered if initial tests are inconclusive.<br>9. **Anti-Hu, Anti-Yo, and Anti-Ri Antibodies**<br>○ **Reasoning:** These paraneoplastic antibodies are commonly associated with neurological syndromes related to cancer. Testing for these can help confirm or rule out a paraneoplastic syndrome.<br>**Most Crucial Test**<br>**Anti-Ro/SSA and Anti-La/SSB Antibodies Testing:**<br>● **Reasoning:** Given the positive ENA autoantibodies, specific testing for Sjögren's Syndrome-related antibodies is the most direct and essential next step. These antibodies (anti-Ro/SSA and anti-La/SSB) are highly specific for Sjögren's Syndrome and can solidify the diagnosis, explaining the patient's neuropathy and other systemic symptoms.<br>**Conclusion**<br>The most crucial test to perform next is **Anti-Ro/SSA and Anti-La/SSB Antibodies Testing**. This test targets the most likely diagnosis (Primary Sjögren's Syndrome), given the clinical presentation and initial laboratory findings, and will significantly narrow down the differential diagnosis.
37	A 58-year-old female patient presents with a 13-month history of sensory-motor, symmetrical, length dependent polyneuropathy with subacute onset and progressive course. The patient presents with distal weakness, neuropathic pain, and paresthesia in the four limbs. Clinical examination shows the presence of mild intrinsic hand atrophy and a peripheral edema. There are no sensory ataxia, tremor, foot deformities, cranial nerve or autonomic involvement. Past medical history is positive for occasional alcohol use. Nerve conduction studies show a mixed demyelinating-axonal, length dependent, sensory-motor polyneuropathy. Screening lab tests including vitamin B12, folate, vitamin B6, vitamin E, HCV, HBV, ANA, ANCA, ENA, CRP, ESR, glycemia, HbA1c, copper, and cryoglobulins are negative, while serum electrophoresis shows positivity for IgG lambda paraprotein. A cardiac ultrasound, performed to investigate edema in the lower limbs, revealed the presence of pericardial effusion. There is no family history of polyneuropathy.	POEMS	**Step-by-Step Analysis and Additional Examinations**<br>1. **Review and Analysis of Current Data**<br>○ Patient Profile: 58-year-old female with a 13-month history of sensory-motor, symmetrical, length-dependent polyneuropathy.<br>○ Key Symptoms: Distal weakness, neuropathic pain, paresthesia, mild intrinsic hand atrophy, peripheral edema.<br>○ Examination Findings: No sensory ataxia, tremor, foot deformities, cranial nerve or autonomic involvement.<br>○ Laboratory Results: Negative for common neuropathy-related lab tests but positive for IgG lambda paraprotein.<br>○ Imaging: Cardiac ultrasound shows pericardial effusion.<br>○ Nerve Conduction Studies: Mixed demyelinating-axonal polyneuropathy.<br>○ Past Medical History: Occasional alcohol use.<br>○ No family history of polyneuropathy.<br>2. **Additional Examinations Needed**<br>**Laboratory Tests**<br>1. **Serum Free Light Chain Assay**<br>○ **Reasoning**: To evaluate for light chain multiple myeloma or AL amyloidosis, which can be associated with monoclonal gammopathy.<br>○ **Details**: Measures free kappa and lambda light chains in serum; an abnormal ratio indicates clonal plasma cell disorders.<br>2. **Bone Marrow Biopsy**<br>○ **Reasoning**: To assess for underlying plasma cell dyscrasias such as multiple myeloma or amyloidosis.<br>○ **Details**: Provides direct visualization and quantification of plasma cells, amyloid deposits, and other abnormalities.<br>3. **Serum and Urine Immunofixation Electrophoresis**<br>○ **Reasoning**: To further characterize the paraprotein and detect other monoclonal proteins.<br>○ **Details**: Detects and characterizes monoclonal proteins in serum and urine, providing additional information on the type of paraprotein.<br>4. **NT-proBNP or BNP Levels**<br>○ **Reasoning**: To evaluate for cardiac involvement in amyloidosis or other conditions causing pericardial effusion.<br>○ **Details**: Elevated levels suggest heart failure or amyloid cardiomyopathy.<br>**Instrumental Examinations**<br>5. **Abdominal Fat Pad Biopsy**<br>○ **Reasoning**: To detect amyloid deposits, which are often present in systemic amyloidosis.<br>○ **Details**: Fat pad biopsy is a minimally invasive procedure that can detect amyloid with Congo red staining and apple-green birefringence under polarized light.<br>6. **MRI of the Lumbar Spine**<br>○ **Reasoning**: To rule out structural causes of neuropathy and to assess for nerve root involvement.<br>○ **Details**: Provides detailed images of the lumbar spine and nerve roots, helping to identify compressive neuropathies.<br>7. **Echocardiogram with Strain Imaging**<br>○ **Reasoning**: To assess for cardiac amyloidosis, which can present with pericardial effusion and neuropathy.<br>○ **Details**: Strain imaging detects subtle changes in myocardial function, often seen in cardiac amyloidosis.<br>8. **PET-CT Scan**<br>○ **Reasoning**: To detect systemic involvement of diseases like amyloidosis or to rule out malignancies.<br>○ **Details**: Provides whole-body imaging to detect organ involvement and systemic disease.<br>**Most Crucial Test**

<table>
<tr><td></td><td></td><td></td><td>The Serum Free Light Chain Assay is the most crucial test from the list. This test provides essential information about the presence of clonal plasma cell disorders, such as light chain multiple myeloma or AL amyloidosis, which are highly relevant given the patient's presentation and positive serum electrophoresis for IgG lambda paraprotein. Detecting an abnormal light chain ratio would significantly narrow down the differential diagnoses and guide further diagnostic and therapeutic steps.</td></tr>
<tr><td>38</td><td>A 30-year-old female patient presents with a six-month history of right peroneal mononeuropathy (foot drop) with acute onset. She also reports the presence of mild paresthesia in the fourth and fifth fingers of the left hand over the past two years, and frequent hand paresthesia after using scissors at work. Nerve conduction studies show a mild demyelinating, sensory-motor polyneuropathy, with right carpal tunnel syndrome, left ulnar entrapment at the elbow, and right peroneal nerve entrapment at the fibular head. Screening lab tests including vitamin B12, folate, vitamin B6, vitamin E, HBV, HCV, glycemia, HbA1c, ANA, ANCA, ENA, CRP, ESR, copper, cryoglobulins, and serum electrophoresis are all negative. There is no family history of polyneuropathy.</td><td>HNPP</td><td>Step-by-Step Analysis of Additional Examinations Needed

Genetic Testing for PMP22 Gene Deletion or Mutation
Reasoning: Hereditary Neuropathy with Liability to Pressure Palsies (HNPP) is often caused by a deletion or mutation in the PMP22 gene. Given the clinical presentation of multiple entrapment neuropathies and negative laboratory results, genetic testing is crucial to confirm or rule out HNPP.
Test: DNA testing for PMP22 gene deletion or duplication (via multiplex ligation-dependent probe amplification (MLPA) or comparative genomic hybridization (CGH)).
Lumbar Puncture for Cerebrospinal Fluid (CSF) Analysis
Reasoning: Chronic Inflammatory Demyelinating Polyneuropathy (CIDP) often shows elevated protein levels in the CSF without pleocytosis. This test can help distinguish CIDP from other demyelinating neuropathies.
Test: Lumbar puncture with CSF analysis including protein levels and cell count.
MRI of the Brachial and Lumbar Plexus
Reasoning: MRI can provide detailed imaging of peripheral nerves and detect any structural abnormalities, such as nerve enlargement or inflammation, which could indicate an inflammatory or compressive etiology.
Test: MRI of the brachial and lumbar plexus with gadolinium contrast.
Anti-GM1 Antibodies Testing
Reasoning: Multifocal Motor Neuropathy (MMN) is often associated with the presence of anti-GM1 antibodies. Testing for these antibodies can help in the diagnosis of MMN, particularly in cases of asymmetric motor neuropathy.
Test: Blood test for anti-GM1 antibodies.
Repeat Nerve Conduction Studies and Electromyography (EMG)
Reasoning: Detailed electrodiagnostic studies can help delineate the pattern and severity of nerve involvement. Repeated studies can show progression or stability of neuropathy and help differentiate between various types of neuropathies.
Test: Repeat comprehensive nerve conduction studies and EMG.
Skin Biopsy for Intraepidermal Nerve Fiber Density (IENFD)
Reasoning: A skin biopsy can help evaluate small fiber neuropathy, which might not be detected in routine nerve conduction studies. This can provide additional information about the integrity of small nerve fibers.
Test: Skin biopsy with intraepidermal nerve fiber density (IENFD) measurement.

Most Crucial Test
The most crucial test in this clinical scenario is genetic testing for the PMP22 gene deletion or mutation. This test directly addresses the high likelihood of HNPP based on the clinical presentation and the negative results from a broad range of other tests. Confirming or ruling out HNPP would significantly narrow down the differential diagnosis and guide further management.
Summary of Tests and Reasoning

Genetic Testing for PMP22: Essential to confirm or rule out HNPP.
Lumbar Puncture for CSF Analysis: Important for identifying elevated protein levels indicative of CIDP.
MRI of Brachial and Lumbar Plexus: Helpful for visualizing nerve structure and detecting inflammation or compressive lesions.
Anti-GM1 Antibodies Testing: Critical for diagnosing MMN, especially in cases of asymmetric motor neuropathy.
Repeat Nerve Conduction Studies and EMG: Necessary for detailed assessment of nerve function and progression of neuropathy.
Skin Biopsy for IENFD: Useful for detecting small fiber neuropathy.

The genetic test for PMP22 is the priority due to its direct relevance to the suspected diagnosis of HNPP, which is supported by the patient's clinical presentation and negative laboratory results.</td></tr>
<tr><td>39</td><td>A 76-year-old male patient presents with an 8-year history of chronic, pure sensory, symmetrical, length dependent polyneuropathy with insidious onset and slow progressive course. The patient presents with severe neuropathic pain and heat intolerance in the feet. He does not report weakness, ataxia, paresthesia, tremor, cranial nerve or autonomic involvement. Clinical examination reveals the presence of allodynia and hyperalgesia in the feet. There are no foot deformities. Past medical history is positive for prostatic cancer. Nerve conduction studies are normal. Screening lab tests including vitamin B12, folate, vitamin B6, vitamin E, HBV, HCV, ANA, ANCA, ENA, CRP, ESR, glycemia, HbA1c, copper, cryoglobulins, and serum electrophoresis are negative. There is no family history of polyneuropathy.</td><td>Small fibre neuropathy</td><td>Step-by-Step Analysis and Additional Examinations
Given the clinical scenario, the goal is to conduct additional examinations that can further narrow down the differential diagnoses for the patient's chronic, pure sensory, symmetrical, length-dependent polyneuropathy.
Step 1: Detailed Review of Clinical Presentation

Symptomatology: Chronic, insidious onset, pure sensory polyneuropathy with severe neuropathic pain, heat intolerance, allodynia, and hyperalgesia in the feet.
Negative Findings: No weakness, ataxia, paresthesia, tremor, cranial nerve involvement, or autonomic involvement.
Medical History: Prostatic cancer.
Negative Tests: Normal nerve conduction studies; screening lab tests negative for vitamin deficiencies, infections, autoimmune markers, metabolic and other systemic conditions.

Step 2: Identification of Potential Pathologies

Idiopathic Small Fiber Neuropathy
Chemotherapy-Induced Peripheral Neuropathy (CIPN)
Paraneoplastic Neuropathy

Step 3: Additional Examinations

Skin Biopsy for Intraepidermal Nerve Fiber Density (IENFD)
Reasoning: This test can confirm small fiber neuropathy by quantifying the density of small nerve fibers in the skin. A reduced density supports a diagnosis of small fiber neuropathy.
Relevance: Given the normal nerve conduction studies and clinical features of small fiber involvement (pain, allodynia), this test is crucial.
Quantitative Sensory Testing (QST)
Reasoning: QST assesses the function of small and large sensory fibers by measuring responses to thermal and vibratory stimuli. It helps differentiate small fiber neuropathy from other sensory disorders.
Relevance: It provides functional evidence of sensory fiber impairment and correlates with the patient's symptoms of heat intolerance and neuropathic pain.
Autonomic Function Tests
Reasoning: Though the patient does not report autonomic symptoms, small fiber neuropathy can sometimes involve autonomic fibers. Tests include heart rate variability, blood pressure response to tilt, and sudomotor function.
Relevance: These tests can identify subtle autonomic involvement, which might not be evident clinically but would support a small fiber neuropathy diagnosis.
Paraneoplastic Panel (Expanded)
Reasoning: A more comprehensive paraneoplastic antibody panel, including antibodies not covered in the initial screening, could detect paraneoplastic syndromes associated with sensory neuropathy.
Relevance: Given the patient's history of prostatic cancer, a thorough evaluation for paraneoplastic antibodies is warranted despite negative initial tests.
Chemotherapy History Review
Reasoning: Detailed review of the patient’s chemotherapy regimen, including specific agents and timing, to assess the likelihood of CIPN.
</td></tr>
</table>

			○ **Relevance**: Certain chemotherapeutic agents (e.g., platinum-based drugs, taxanes) are more likely to cause neuropathy. Correlating symptom onset with chemotherapy exposure can help confirm CIPN.<br>6. **Nerve Biopsy**<br>○ **Reasoning**: While more invasive, a nerve biopsy can provide definitive histological evidence of neuropathy and rule out other causes such as amyloidosis or vasculitis.<br>○ **Relevance**: Typically reserved for cases where less invasive tests are inconclusive, or when specific neuropathic conditions are suspected.<br>**Step 4: Most Crucial Test**<br>The **Skin Biopsy for Intraepidermal Nerve Fiber Density (IENFD)** is the most crucial test. It provides direct evidence of small fiber neuropathy, which is strongly suggested by the patient's symptoms and the normal nerve conduction studies. This test can confirm a diagnosis of small fiber neuropathy, thereby narrowing down the differential diagnoses significantly and guiding further management.<br>**Conclusion**<br>In this case, the skin biopsy for IENFD is essential for confirming the presence of small fiber neuropathy, which is the leading consideration given the clinical presentation and normal nerve conduction studies. This test will either confirm the diagnosis or prompt further investigations if results are inconclusive.
40	A 79-year-old male patient presents with a 2-year history of pure sensory, symmetrical, length dependent polyneuropathy with insidious onset. The patient presents with paresthesia, hypoesthesia and neuropathic pain in the distal areas of the lower limbs. There are no ataxia, tremor, foot deformities, cranial nerve or autonomic involvement. Past medical history is silent. Nerve conduction studies show pure sensory, axonal polyneuropathy. Screening lab tests including vitamin B12, folate, vitamin B6, vitamin E, HCV, ANA, ANCA, ENA, CRP, ESR, glycemia, HbA1c, copper, cryoglobulins and serum electrophoresis are all negative, with evidence of chronic HBV infection. There is no family history of polyneuropathy.	HBV-associated neuropathy	**Additional Examinations for Narrowing Down Differential Diagnoses**<br>**1. HBV DNA Quantification and Liver Function Tests**<br>● **Reasoning:**<br>○ To assess the viral load and activity of HBV infection.<br>○ Elevated HBV DNA levels and abnormal liver function tests can indicate active infection and potential systemic effects, including neuropathy.<br>● **Tests:**<br>○ HBV DNA (PCR)<br>○ ALT, AST, ALP, GGT, bilirubin levels<br>**2. CSF Analysis**<br>● **Reasoning:**<br>○ To identify any signs of inflammation, infection, or malignancy-related changes in the central nervous system.<br>○ CSF analysis can reveal elevated protein levels or pleocytosis, which may support the diagnosis of paraneoplastic or infectious neuropathy.<br>● **Tests:**<br>○ CSF protein, glucose, cell count, cytology, oligoclonal bands<br>**3. Paraneoplastic Panel**<br>● **Reasoning:**<br>○ To detect antibodies associated with paraneoplastic syndromes that can cause neuropathy.<br>○ This is particularly important given the patient's age and the possibility of an occult malignancy.<br>● **Tests:**<br>○ Anti-Hu, Anti-Ri, Anti-Yo, Anti-Ma2, and other relevant antibodies<br>**4. Nerve Biopsy**<br>● **Reasoning:**<br>○ To obtain direct histopathological evidence of nerve pathology.<br>○ This can help differentiate between various causes of axonal neuropathy, including vasculitis, amyloidosis, or other infiltrative processes.<br>● **Test:**<br>○ Sural nerve biopsy<br>**5. Whole-Body PET-CT Scan**<br>● **Reasoning:**<br>○ To screen for occult malignancies, which could be causing paraneoplastic neuropathy.<br>○ PET-CT is sensitive for detecting metabolically active tumors and can guide further diagnostic workup.<br>● **Test:**<br>○ Whole-body PET-CT<br>**6. Quantitative Sensory Testing (QST)**<br>● **Reasoning:**<br>○ To provide a detailed assessment of sensory function and quantify sensory deficits.<br>○ QST can help characterize the neuropathy more precisely and differentiate between small and large fiber involvement.<br>● **Test:**<br>○ Quantitative Sensory Testing (QST)<br>**Most Crucial Test**<br>**Nerve Biopsy** is the most crucial test from the list. It provides direct histopathological evidence that can significantly narrow down the differential diagnoses by identifying specific pathological changes in the nerve, such as vasculitis, amyloidosis, or other infiltrative processes. This information is critical in confirming or ruling out potential causes of neuropathy that might not be evident through other less invasive tests.
41	An 82-year-old female patient presents with a 4-year history of pure sensory, length dependent, symmetric polyneuropathy with insidious onset and slow progressive course. The patient presents with paresthesia and mild neuropathic pain in the feet. Clinical examination does not show weakness or sensory ataxia, tremor, foot deformities, cranial nerve or autonomic involvement. Past medical history is positive for IgA kappa monoclonal gammopathy, arterial	MGUS-associated neuropathy	**Step-by-Step Analysis and Additional Examinations**<br>**Step 1: Clinical Assessment and Detailed History**<br>● **Re-evaluate patient history**: Confirm the history of paresthesia, pain, and any other sensory abnormalities. Assess the progression and any possible triggers or associated factors.<br>● **Thorough physical examination**: Repeat and ensure detailed neurological examination focusing on sensory modalities, reflexes, and any subtle signs that might have been previously missed.<br>**Step 2: Necessary Additional Examinations**<br>1. **Serum Protein Electrophoresis (SPEP) and Immunofixation Electrophoresis (IFE)**<br>○ **Reasoning**: Given the presence of IgA kappa monoclonal gammopathy, it is essential to further characterize the monoclonal protein. These tests can help differentiate between MGUS and multiple myeloma or other plasma cell dyscrasias.<br>○ **Expected Outcome**: Identify the type and quantity of monoclonal protein, which can help in assessing the significance of the gammopathy.<br>2. **Bone Marrow Biopsy**<br>○ **Reasoning**: To assess for plasma cell disorders, especially if SPEP and IFE suggest a high burden of monoclonal protein. This test can help rule out multiple myeloma or other hematologic malignancies.<br>○ **Expected Outcome**: Detailed assessment of bone marrow plasma cell percentage and possible clonal abnormalities.

	hypertension, gout, hypercholesterolemia, and cataract. Nerve conduction studies show presence of a pure sensory, length dependent, symmetric, axonal polyneuropathy. Screening lab tests including vitamin B12, folate, vitamin B6, vitamin E, HBV, HCV, glycemia, HbA1c, ANA, ANCA, ENA, CRP, ESR, copper, and cryoglobulins are negative, with positivity to IgA kappa paraprotein. There is no family history of polyneuropathy.		3. **Serum Free Light Chain Assay**<br>○ **Reasoning**: This test helps quantify free light chains in the blood, which can provide additional information on the monoclonal protein load and is useful in diagnosing and monitoring plasma cell disorders.<br>○ **Expected Outcome**: Abnormal kappa/lambda ratio indicative of clonal plasma cell disorder.<br>4. **Quantitative Sensory Testing (QST)**<br>○ **Reasoning**: To objectively measure sensory nerve function and assess the extent of sensory loss. This can provide detailed information on the sensory modalities affected.<br>○ **Expected Outcome**: Quantification of sensory deficits, confirming the length-dependent nature and type of sensory involvement.<br>5. **Skin Biopsy for Intraepidermal Nerve Fiber Density (IENFD)**<br>○ **Reasoning**: To assess for small fiber neuropathy, which might not be detected on nerve conduction studies. This can provide direct evidence of nerve fiber loss in the skin.<br>○ **Expected Outcome**: Reduced intraepidermal nerve fiber density confirming small fiber involvement.<br>6. **Magnetic Resonance Imaging (MRI) of the Spine**<br>○ **Reasoning**: To exclude structural causes of neuropathy such as spinal stenosis or radiculopathy, which can sometimes present with similar symptoms.<br>○ **Expected Outcome**: Normal spinal imaging, ruling out compressive or structural causes.<br>7. **CSF Analysis (Lumbar Puncture)**<br>○ **Reasoning**: To exclude central nervous system involvement and look for any inflammatory or infectious causes of neuropathy. Elevated protein levels can sometimes be seen in chronic inflammatory demyelinating polyneuropathy (CIDP).<br>○ **Expected Outcome**: Normal CSF analysis, which helps in ruling out CIDP and central nervous system involvement.<br>8. **Genetic Testing for Hereditary Neuropathy**<br>○ **Reasoning**: Despite the lack of family history, late-onset genetic neuropathies should be considered. Testing for common mutations can help identify hereditary neuropathies that might not present with family history due to de novo mutations.<br>○ **Expected Outcome**: Negative genetic testing, supporting an acquired rather than hereditary neuropathy.<br>**Conclusion: Most Crucial Test**<br>The most crucial test from the list is **Serum Protein Electrophoresis (SPEP) and Immunofixation Electrophoresis (IFE)**. This test is critical because it will help to further characterize the IgA kappa monoclonal protein. Differentiating between benign monoclonal gammopathy of undetermined significance (MGUS) and more serious conditions such as multiple myeloma or other related plasma cell dyscrasias is essential for directing appropriate management and therapy. This test will provide key information about the significance of the gammopathy in relation to the patient's neuropathy.
42	A 47-year-old male patient presents with a 1-year history of pure motor, asymmetrical multineuropathy with subacute onset. Clinical examination reveals the presence of weakness and atrophy in a multifocal distribution in the upper limbs. There are no sensory deficits or subjective numbness, tingling or pain, and physical examination does not show the presence of sensory ataxia, tremor, foot deformities, cranial nerve or autonomic involvement. Past medical history is silent. Nerve conduction studies show pure motor, demyelinating multineuropathy with nerve conduction blocks. Screening lab tests including vitamin B12, folate, vitamin B6, vitamin E, HCV, HBV, ANA, ANCA, ENA, CRP, ESR, glycemia, HbA1c, copper, cryoglobulins and serum electrophoresis are all negative. There is no family history of polyneuropathy.	MMN	**Step-by-Step Analysis and Additional Examinations**<br>**Initial Clinical Scenario Recap**<br>● **Patient Demographics**: 47-year-old male<br>● **Symptoms**: 1-year history of pure motor, asymmetrical multineuropathy with subacute onset<br>● **Clinical Findings**: Weakness and atrophy in a multifocal distribution in the upper limbs; no sensory deficits; no sensory ataxia, tremor, foot deformities; no cranial nerve or autonomic involvement<br>● **Past Medical History**: Unremarkable<br>● **Nerve Conduction Studies**: Pure motor, demyelinating multineuropathy with nerve conduction blocks<br>● **Lab Results**: Negative for vitamin B12, folate, vitamin B6, vitamin E, HCV, HBV, ANA, ANCA, ENA, CRP, ESR, glycemia, HbA1c, copper, cryoglobulins, and serum electrophoresis<br>● **Family History**: No family history of polyneuropathy<br>**Additional Examinations and Reasoning**<br>**1. Serum Anti-GM1 Antibodies**<br>● **Reasoning**: Multifocal Motor Neuropathy (MMN) is often associated with anti-GM1 antibodies. Testing for these antibodies can support the diagnosis of MMN.<br>● **Expected Outcome**: Presence of anti-GM1 antibodies would strongly support MMN as the diagnosis.<br>**2. Cerebrospinal Fluid (CSF) Analysis**<br>● **Reasoning**: CSF analysis can help distinguish between inflammatory and non-inflammatory neuropathies. Elevated protein levels with normal cell counts (albuminocytologic dissociation) are common in Chronic Inflammatory Demyelinating Polyneuropathy (CIDP).<br>● **Expected Outcome**: Elevated protein levels without pleocytosis would support a diagnosis of CIDP.<br>**3. Magnetic Resonance Imaging (MRI) of the Brachial Plexus**<br>● **Reasoning**: MRI can help identify structural causes of neuropathy, such as nerve root or plexus lesions, which might explain asymmetrical motor involvement.<br>● **Expected Outcome**: Any structural abnormalities or inflammatory changes would need further investigation to determine their relevance.<br>**4. Repetitive Nerve Stimulation and Single-Fiber Electromyography (SFEMG)**<br>● **Reasoning**: These tests can help differentiate between presynaptic and postsynaptic disorders of neuromuscular transmission, which can present with pure motor symptoms.<br>● **Expected Outcome**: Abnormal results may suggest disorders such as Myasthenia Gravis, which typically presents with different clinical features but needs to be ruled out.<br>**5. Muscle and Nerve Biopsy**<br>● **Reasoning**: Biopsy can provide histopathological evidence of specific neuropathies, including inflammatory and inherited forms.<br>● **Expected Outcome**: Findings consistent with inflammatory neuropathy would support CIDP, while other findings might suggest alternative diagnoses.<br>**6. Genetic Testing for Hereditary Neuropathies**<br>● **Reasoning**: Although there is no family history, genetic mutations can sometimes present sporadically. Genetic testing can rule out hereditary motor neuropathies.<br>● **Expected Outcome**: Identifying specific genetic mutations would confirm a hereditary neuropathy diagnosis.<br>**7. Serum Immunoglobulins and Electrophoresis for Paraproteins**<br>● **Reasoning**: Although initial serum electrophoresis was negative, checking for monoclonal gammopathy can help identify paraproteinemic neuropathies, such as POEMS syndrome or MGUS.<br>● **Expected Outcome**: Detection of paraproteins would direct attention towards related neuropathies.<br>**Most Crucial Test**<br>The most crucial test to perform next is **Serum Anti-GM1 Antibodies**. This test is particularly relevant for confirming the diagnosis of Multifocal Motor Neuropathy (MMN), which is the most likely diagnosis based on the clinical scenario provided. Detection of anti-GM1 antibodies would strongly support the diagnosis of MMN, guiding further management and treatment decisions effectively.<br>**Summary of Recommended Tests**<br>1. Serum Anti-GM1 Antibodies<br>2. Cerebrospinal Fluid (CSF) Analysis<br>3. MRI of the Brachial Plexus

			4. Repetitive Nerve Stimulation and Single-Fiber Electromyography (SFEMG)<br>5. Muscle and Nerve Biopsy<br>6. Genetic Testing for Hereditary Neuropathies<br>7. Serum Immunoglobulins and Electrophoresis for Paraproteins<br>Conducting these tests in a stepwise manner, starting with serum anti-GM1 antibodies, will help narrow down the differential diagnoses and confirm the most likely diagnosis of MMN.
43	An 88-year-old male patient presents with a 4-year history of sensory-motor, symmetrical, length dependent polyneuropathy with insidious onset and slow progressive course. The patient presents with paresthesia and neuropathic pain distally in the lower limbs, with no ataxia, tremor, foot deformities, cranial nerve or autonomic involvement. Past medical history is positive for type 2 diabetes, cardiac insufficiency and lung cancer, for which he underwent chemotherapy 15 years ago. Nerve conduction studies show a sensory-motor, symmetric, axonal polyneuropathy. Screening lab tests including vitamin B12, folate, vitamin B6, vitamin E, HBV, HCV, ANA, ANCA, ENA, CRP, ESR, copper, cryoglobulins and serum electrophoresis are all negative, with evidence of hyperglycaemia and elevated HbA1c. There is no family history of polyneuropathy.	Diabetic neuropathy	**Step-by-Step Analysis and Additional Examinations**<br>Given the comprehensive clinical scenario of an 88-year-old male with sensory-motor, symmetrical, length-dependent polyneuropathy, the following additional examinations are necessary to narrow down the differential diagnoses. Each proposed test is justified based on the specifics of the case and medical knowledge.<br>**1. Electromyography (EMG):**<br>● **Reasoning:** EMG is crucial for distinguishing between axonal and demyelinating polyneuropathies. It can provide detailed information about the nature and severity of the neuropathy. Given the nerve conduction studies show an axonal pattern, EMG can help confirm and further characterize this finding.<br>**2. Glucose Tolerance Test (GTT):**<br>● **Reasoning:** While the patient has a known history of type 2 diabetes and elevated HbA1c, a GTT can provide more detailed information on glucose metabolism and help evaluate the extent of glucose dysregulation. This is important in confirming the diagnosis of diabetic polyneuropathy.<br>**3. Serum Protein Electrophoresis with Immunofixation:**<br>● **Reasoning:** This test is useful for detecting monoclonal gammopathies, such as multiple myeloma or POEMS syndrome, which can present with peripheral neuropathy. Despite a normal initial serum electrophoresis, immunofixation provides higher sensitivity for detecting monoclonal proteins.<br>**4. Anti-Hu and Anti-CV2/CRMP5 Antibodies:**<br>● **Reasoning:** These antibodies are associated with paraneoplastic syndromes, which can present with peripheral neuropathy. Given the patient's history of lung cancer, screening for paraneoplastic neuropathies is warranted.<br>**5. Skin Biopsy:**<br>● **Reasoning:** A skin biopsy with evaluation of intraepidermal nerve fiber density can help diagnose small fiber neuropathy, which might not be detected on standard nerve conduction studies. This could be particularly relevant if there is a suspected component of small fiber involvement in the patient's neuropathy.<br>**6. Quantitative Sensory Testing (QST):**<br>● **Reasoning:** QST assesses the function of small and large sensory nerve fibers. This non-invasive test can help quantify the extent of sensory dysfunction and complement findings from nerve conduction studies and EMG.<br>**7. Magnetic Resonance Imaging (MRI) of the Spine:**<br>● **Reasoning:** MRI can rule out structural causes of neuropathy such as spinal stenosis or other compressive lesions that could contribute to the patient's symptoms. Given his age, degenerative spine disease could be a contributing factor.<br>**8. Autonomic Function Testing:**<br>● **Reasoning:** Even though the patient does not have overt autonomic symptoms, subtle autonomic dysfunction can be present in diabetic polyneuropathy. This test can help in identifying any autonomic involvement which might not be clinically apparent.<br>**Determining the Most Crucial Test**<br>Considering the patient's history, clinical presentation, and initial findings, the most crucial test from the above list is the **Glucose Tolerance Test (GTT)**. This test is essential to evaluate the extent of glucose dysregulation, which is directly relevant to confirming the diagnosis of diabetic polyneuropathy. Given the elevated HbA1c and known diabetes, understanding the precise glucose handling and control will guide the management of the patient's neuropathy and overall diabetic care.<br>**Conclusion**<br>In summary, while several additional examinations are justified to narrow down the differential diagnoses, the Glucose Tolerance Test stands out as the most critical. It provides vital information on glucose metabolism, crucial for confirming diabetic polyneuropathy, which is the most likely diagnosis in this case.
44	An 81-year-old male patient presents with a six-month history of sensory-motor, symmetrical, length-dependent polyneuropathy with subacute onset and slow progression. The patient reports neuropathic pain in the lower limbs in a distal, symmetric distribution, along with sensory ataxia. Clinical examination does not reveal weakness, tremor, foot deformities, cranial nerve or autonomic involvement. Past medical history is positive for IgM kappa monoclonal gammopathy, Waldenström macroglobulinemia, and colorectal cancer, for which he underwent chemotherapy five years ago. Nerve conduction studies show a length-dependent, sensorimotor, symmetric, demyelinating polyneuropathy. Screening lab tests, including vitamin B12, folate, vitamin B6, vitamin E, HCV, HBV, ANA, ANCA, ENA, CRP, ESR, glycemia, HbA1c, copper, and cryoglobulins are all normal, with evidence of IgM kappa paraprotein. There is no family history of polyneuropathy.	Anti-MAG polyneuropathy	**Step-by-Step Analysis and Additional Examinations**<br>Given the clinical scenario, further examinations are necessary to narrow down the differential diagnoses and confirm the underlying cause of the patient's polyneuropathy.<br>**1. Serum Immunofixation Electrophoresis**<br>**Reasoning:**<br>● To further characterize the IgM paraprotein detected in the initial tests. This test helps in identifying the specific type of monoclonal protein, which can differentiate between Waldenström macroglobulinemia and MGUS.<br>**2. Bone Marrow Biopsy and Aspiration**<br>**Reasoning:**<br>● To evaluate the extent and activity of the Waldenström macroglobulinemia or to confirm MGUS. This can reveal the percentage of lymphoplasmacytic cells and other hematological abnormalities.<br>**3. Quantitative Immunoglobulins and Serum Free Light Chains**<br>**Reasoning:**<br>● To assess the levels of various immunoglobulins and the ratio of kappa to lambda light chains, which can provide further information on the monoclonal protein's impact and help differentiate between different plasma cell disorders.<br>**4. Anti-MAG Antibodies**<br>**Reasoning:**<br>● To detect antibodies against myelin-associated glycoprotein (MAG), which are often present in patients with neuropathy associated with IgM monoclonal gammopathy. A positive result would strongly suggest a paraproteinemic neuropathy.<br>**5. Lumbar Puncture (CSF Analysis)**<br>**Reasoning:**<br>● To check for elevated protein levels and the presence of monoclonal proteins in the cerebrospinal fluid, which can be indicative of a neuropathy associated with monoclonal gammopathy or Waldenström macroglobulinemia.<br>**6. PET-CT Scan**<br>**Reasoning:**<br>● To assess for systemic involvement of Waldenström macroglobulinemia and to rule out other malignancies or lymphoproliferative disorders that may present with similar symptoms.<br>**7. Skin Biopsy with Immunohistochemistry**<br>**Reasoning:**<br>● If small fiber neuropathy is suspected, a skin biopsy can provide evidence of small fiber involvement, which may not be detected in nerve conduction studies.<br>**8. Repeat Nerve Conduction Studies and EMG**

			**Reasoning:**<br>● To monitor progression and further characterize the type of neuropathy (axonal vs. demyelinating), providing additional data on the pattern and severity of nerve involvement.<br>**Most Crucial Test**<br>**Anti-MAG Antibodies**<br>● **Reasoning:**<br>○ Detecting anti-MAG antibodies can be highly specific for neuropathies associated with IgM monoclonal gammopathies. This test directly addresses the potential mechanism of the patient's demyelinating polyneuropathy in the context of his known IgM kappa paraproteinemia.<br>○ A positive result would provide strong evidence for a paraproteinemic neuropathy, particularly linked to Waldenström macroglobulinemia or MGUS.<br>**Summary**<br>The comprehensive analysis of the patient's clinical scenario suggests that identifying the presence of anti-MAG antibodies is the most crucial next step. This test will directly contribute to confirming or ruling out the diagnosis of a paraproteinemic neuropathy associated with the patient's IgM kappa monoclonal gammopathy. Other tests will further characterize the extent and nature of the underlying hematological disorder and assess for additional systemic involvement.
45	An 83-year-old male patient presents with a 3-year history of sensory, symmetrical, length dependent polyneuropathy with insidious onset. The patient presents with paresthesia and hypoesthesia in the lower limbs without weakness or sensory ataxia. Clinical examination does not show the presence of tremor, neuropathic pain, foot deformities, cranial nerve or autonomic involvement. Past medical history is positive for IgG lambda monoclonal gammopathy. Nerve conduction studies show axonal, length dependent, sensory-motor polyneuropathy and unilateral carpal tunnel syndrome. Screening lab tests including vitamin B12, folate, vitamin B6, vitamin E, HCV, HBV, ANA, ANCA, ENA, CRP, ESR, glycemia, HbA1c, copper, and cryoglobulins are all negative, with positivity for IgG lambda paraprotein. There is no family history of polyneuropathy.	MGUS-associated neuropathy	**Step-by-Step Analysis for Additional Examinations**<br>**Clinical Summary Review**<br>● **Patient Demographics**: 83-year-old male<br>● **Primary Complaint**: 3-year history of sensory, symmetrical, length-dependent polyneuropathy<br>● **Symptoms**: Paresthesia and hypoesthesia in lower limbs, no weakness, sensory ataxia, or other systemic involvement<br>● **Medical History**: IgG lambda monoclonal gammopathy<br>● **Nerve Conduction Studies**: Axonal, length-dependent, sensory-motor polyneuropathy; unilateral carpal tunnel syndrome<br>● **Initial Screening Labs**: Negative for various vitamins, viral infections, autoimmune markers, inflammatory markers, glycemia, and cryoglobulins; positive for IgG lambda paraprotein<br>**Additional Examinations Needed**<br>1. **Serum Immunofixation Electrophoresis (IFE) and Free Light Chain Assay**<br>○ **Reasoning**: Further characterization of the monoclonal protein is essential to differentiate between MGUS and other plasma cell dyscrasias such as multiple myeloma or amyloidosis. The free light chain assay can provide insight into the presence of light chain disease.<br>2. **Bone Marrow Biopsy**<br>○ **Reasoning**: To evaluate the extent of plasma cell involvement and rule out multiple myeloma or other hematologic malignancies. This is particularly important given the patient's age and presence of monoclonal gammopathy.<br>3. **Anti-MAG Antibody Testing**<br>○ **Reasoning**: Anti-myelin-associated glycoprotein (MAG) neuropathy is associated with IgM monoclonal gammopathy, but testing is also valuable in IgG cases. It can help identify a specific subtype of paraproteinemic neuropathy.<br>4. **Skin Biopsy for Intraepidermal Nerve Fiber Density (IENFD)**<br>○ **Reasoning**: This test helps assess small fiber neuropathy, which can present with sensory symptoms and be associated with paraproteinemias. The patient's symptoms suggest sensory involvement, which warrants this examination.<br>5. **Cerebrospinal Fluid (CSF) Analysis**<br>○ **Reasoning**: Lumbar puncture with CSF analysis can help rule out inflammatory or infectious causes of neuropathy. Elevated protein without pleocytosis can support a diagnosis of CIDP, and the presence of oligoclonal bands could indicate an inflammatory process.<br>6. **Quantitative Sensory Testing (QST)**<br>○ **Reasoning**: To objectively assess the functional status of large and small nerve fibers, providing a detailed profile of sensory deficits that could guide diagnosis and management.<br>7. **Autonomic Testing**<br>○ **Reasoning**: While there is no clinical indication of autonomic involvement, autonomic testing can confirm the absence of autonomic neuropathy, further narrowing the differential diagnoses.<br>8. **Genetic Testing**<br>○ **Reasoning**: Although there is no family history of polyneuropathy, late-onset hereditary neuropathies should be considered. Genetic testing can rule out these rare causes.<br>**Most Crucial Test**<br>**Serum Immunofixation Electrophoresis (IFE) and Free Light Chain Assay**<br>● **Reasoning**:<br>○ Detailed characterization of the monoclonal protein is pivotal in differentiating MGUS from other plasma cell disorders such as multiple myeloma or AL amyloidosis, which could have significant treatment and prognostic implications.<br>○ Determining the free light chain ratio can provide additional information regarding the presence of light chain disease.<br>○ These tests are non-invasive and can provide critical diagnostic information that guides further investigations and management.<br>By performing Serum Immunofixation Electrophoresis and Free Light Chain Assay, we can gain essential insights into the nature of the monoclonal gammopathy, which is crucial for narrowing down the differential diagnosis and determining the appropriate clinical management.
46	A 71-year-old female patient presents with a 2-year history of sensory-motor, asymmetrical multineuropathy with relatively slow onset. The patient presents with weakness, numbness and tingling in a multifocal distribution in the four limbs, with hand tremor and mild sensory ataxia without pain. Clinical examination reveals absence of foot deformities, cranial nerve or autonomic involvement. Past medical history is positive for breast and kidney cancer, for which the patient underwent	Multifocal CIDP	**Step-by-Step Analysis and Additional Examinations**<br>**Patient Summary Recap:**<br>● **Age/Gender:** 71-year-old female<br>● **Presenting Complaints:** Sensory-motor, asymmetrical multineuropathy with weakness, numbness, tingling, hand tremor, and mild sensory ataxia over 2 years.<br>● **Clinical Examination:** Absence of foot deformities, cranial nerve, and autonomic involvement.<br>● **Past Medical History:** Treated for breast and kidney cancer with chemotherapy five years ago.<br>● **Investigations:** Multifocal, demyelinating, sensory-motor neuropathy with motor nerve conduction blocks on NCS. Extensive negative screening lab tests.<br>● **Family History:** No family history of polyneuropathy.<br>**Additional Examinations**<br>1. **Cerebrospinal Fluid (CSF) Analysis**<br>○ **Reasoning:** CSF analysis can help differentiate between CIDP and other neuropathies. Elevated protein levels without a significant increase in white blood cells (albuminocytologic dissociation) is characteristic of CIDP.<br>○ **Tests:** CSF protein, glucose, cell count, and oligoclonal bands.

	chemotherapy five years ago, and which were considered cured. Nerve conduction studies show a multifocal, demyelinating, sensory-motor neuropathy with motor nerve conduction blocks. Screening lab tests including vitamin B12, folate, vitamin B6, vitamin E, HCV, HBV, ANA, ANCA, ENA, CRP, ESR, glycemia, HbA1c, copper, cryoglobulins and serum electrophoresis are all negative. There is no family history of polyneuropathy.		2. **Anti-GM1 Antibodies**<br>○ **Reasoning:** Anti-GM1 antibodies are often present in Multifocal Motor Neuropathy (MMN) and can help differentiate MMN from CIDP, as MMN typically shows these antibodies in a significant number of cases.<br>3. **Paraneoplastic Panel**<br>○ **Reasoning:** Considering the patient's history of cancer, a comprehensive paraneoplastic panel including anti-Hu, anti-Yo, and anti-Ri antibodies among others could identify a paraneoplastic syndrome, which might not have been covered by initial broad screening tests.<br>4. **Immunoglobulins and Electrophoresis (Serum and Urine)**<br>○ **Reasoning:** Testing for monoclonal gammopathies (e.g., multiple myeloma, POEMS syndrome) can be relevant since these conditions can present with demyelinating neuropathy. Serum and urine immunofixation electrophoresis, along with free light chain assays, should be performed.<br>5. **Nerve Biopsy**<br>○ **Reasoning:** In cases where diagnosis remains uncertain after non-invasive tests, a nerve biopsy can provide definitive information on the type of neuropathy, revealing specific pathological features such as inflammation, demyelination, or vasculitis.<br>6. **MRI of Brachial and Lumbar Plexus**<br>○ **Reasoning:** MRI can reveal hypertrophy or inflammation of the nerve roots and plexuses, which can support the diagnosis of CIDP. It can also exclude other causes of neuropathy such as structural lesions.<br>7. **Genetic Testing**<br>○ **Reasoning:** Although there's no family history, some genetic neuropathies can present sporadically. Genetic testing for hereditary neuropathies (e.g., Charcot-Marie-Tooth disease) might be considered if other investigations are inconclusive.<br>**Most Crucial Test**<br>The **Cerebrospinal Fluid (CSF) Analysis** is the most crucial test. It can provide direct evidence supporting the diagnosis of CIDP through the identification of elevated protein levels with normal cell count (albuminocytologic dissociation). This test is essential because it directly addresses the most likely diagnosis (CIDP) based on the clinical presentation and initial test results.<br>**Summary of Recommended Tests with Detailed Reasoning:**<br>1. **CSF Analysis:** To confirm CIDP through albuminocytologic dissociation.<br>2. **Anti-GM1 Antibodies:** To rule out MMN.<br>3. **Paraneoplastic Panel:** To identify or rule out a paraneoplastic syndrome.<br>4. **Immunoglobulins and Electrophoresis:** To exclude monoclonal gammopathies.<br>5. **Nerve Biopsy:** For definitive histopathological diagnosis if other tests are inconclusive.<br>6. **MRI of Brachial and Lumbar Plexus:** To support CIDP diagnosis or rule out other structural causes.<br>7. **Genetic Testing:** To identify potential hereditary neuropathies in the absence of other causes.<br>These additional examinations will help narrow down the differential diagnoses, ultimately confirming the most likely diagnosis and guiding appropriate treatment.
47	An 80-year-old male patient presents with a 4-year history of pure sensory, symmetrical, length dependent polyneuropathy with insidious onset. The patient presents with distal paresthesia, neuropathic pain and cramps in the lower limbs with no ataxia, tremor, foot deformities, cranial nerve or autonomic involvement. Past medical history shows diabetes, psoriatic arthritis, polymyalgia rheumatica and chemotherapy for lung cancer. Neuropathy symptoms started four years after chemotherapy. Nerve conduction studies show mixed sensory-motor, symmetric, axonal polyneuropathy. Screening lab tests including vitamin B12, folate, vitamin B6, vitamin E, HCV, ANA, ANCA, ENA, CRP, ESR, glycemia, copper, cryoglobulins and serum electrophoresis are all negative, with evidence of chronic HBV infection. HbA1c levels are elevated. There is no family history of polyneuropathy.	Diabetic neuropathy	**Step-by-Step Analysis and Additional Examinations**<br>**Step 1: Review Initial Findings**<br>**Key Points:**<br>● Pure sensory, symmetrical, length-dependent polyneuropathy<br>● Symptoms: distal paresthesia, neuropathic pain, cramps in lower limbs<br>● No ataxia, tremor, foot deformities, cranial nerve, or autonomic involvement<br>● Past medical history: diabetes, psoriatic arthritis, polymyalgia rheumatica, chemotherapy for lung cancer<br>● Chronic HBV infection and elevated HbA1c<br>● Nerve conduction studies: mixed sensory-motor, symmetric, axonal polyneuropathy<br>● Screening lab tests: largely unremarkable<br>**Step 2: Identify Gaps in Information**<br>To narrow down the differential diagnoses, we need to gather more specific information on potential causes. Additional tests will help clarify whether diabetic neuropathy, chemotherapy-induced neuropathy, or HBV-associated neuropathy is the most likely cause.<br>**Step 3: List Additional Examinations**<br>1. **Detailed Glucose Monitoring and HbA1c Trend Analysis**<br>○ **Reasoning:**<br>■ To confirm the diagnosis of diabetic neuropathy, it is crucial to have detailed data on the patient's glucose control over time. This includes current fasting glucose, postprandial glucose, and HbA1c levels over the past few years.<br>○ **Test:**<br>■ Continuous glucose monitoring<br>■ HbA1c trend analysis (last 5 years)<br>2. **Electromyography (EMG)**<br>○ **Reasoning:**<br>■ EMG can help differentiate between axonal and demyelinating neuropathy and assess the extent of motor involvement, which can be crucial in distinguishing between diabetic neuropathy and other causes.<br>○ **Test:**<br>■ Electromyography to assess muscle response to nerve stimulation<br>3. **Skin Biopsy for Intraepidermal Nerve Fiber Density (IENFD)**<br>○ **Reasoning:**<br>■ To evaluate small fiber neuropathy, which is common in diabetic neuropathy and can present with purely sensory symptoms. This test measures nerve fiber density and can help confirm diabetic etiology.<br>○ **Test:**<br>■ Skin biopsy with IENFD analysis<br>4. **Lumbar Puncture for Cerebrospinal Fluid (CSF) Analysis**<br>○ **Reasoning:**<br>■ To rule out inflammatory or infectious causes of neuropathy that may not have been detected in initial blood tests. CSF analysis can detect elevated protein levels, indicative of chronic inflammatory demyelinating polyneuropathy (CIDP) or other neuroinflammatory conditions.

			○ **Test:**<br>■ Lumbar puncture with CSF analysis (protein, glucose, cytology, immunoglobulin, oligoclonal bands)<br>5. **Quantitative Sensory Testing (QST)**<br>○ **Reasoning:**<br>■ To objectively measure sensory thresholds and confirm the presence and extent of sensory neuropathy. This can be particularly useful in distinguishing between different types of sensory neuropathy.<br>○ **Test:**<br>■ Quantitative sensory testing (vibration, thermal thresholds)<br>6. **Serum Immunofixation Electrophoresis**<br>○ **Reasoning:**<br>■ To check for monoclonal gammopathy, which can be associated with certain types of neuropathies, including those seen in plasma cell dyscrasias or POEMS syndrome.<br>○ **Test:**<br>■ Serum immunofixation electrophoresis<br>7. **Comprehensive Hepatitis B Virus Workup**<br>○ **Reasoning:**<br>■ To assess the extent of HBV infection and rule out HBV-related neuropathy through markers of viral replication and liver function.<br>○ **Test:**<br>■ HBV DNA quantification<br>■ Liver function tests (LFTs)<br>**Step 4: Determine the Most Crucial Test**<br>**Most Crucial Test: Detailed Glucose Monitoring and HbA1c Trend Analysis**<br>**Reasoning:**<br>● Given the strong suspicion of diabetic neuropathy based on the elevated HbA1c and the patient's history of diabetes, understanding the patient's long-term glycemic control is vital.<br>● Continuous glucose monitoring and a comprehensive analysis of HbA1c trends will provide definitive evidence of whether chronic hyperglycemia has been a consistent issue, supporting the diagnosis of diabetic neuropathy.<br>● This test will also guide the management plan for optimizing glucose control, which is crucial for preventing further nerve damage.
48	A 45-year-old female patient presents with a 1-year history of pure sensory, symmetrical, length dependent polyneuropathy with subacute onset. The patient presents with distal leg paresthesia and neuropathic pain, with no ataxia, tremor, foot deformities, cranial nerve or autonomic involvement. Clinical examination shows hyperalgesia at pinprick test with normal tactile sensation. Past medical history is positive for multiple myeloma, for which the patient has undergone chemotherapy. Neuropathy symptoms started after the first chemotherapy cycle and remained stable ever since. Nerve conduction studies show pure a sensory, symmetric, axonal polyneuropathy. Screening lab tests including vitamin B12, folate, vitamin B6, vitamin E, HBV, HCV, ANA, ANCA, ENA, CRP, ESR, glycemia, HbA1c, copper and cryoglobulins are all negative, with positivity for IgG lambda paraprotein. There is no family history of polyneuropathy.	Chemotherapy-induced neuropathy	**Step-by-Step Analysis and Additional Examinations**<br>**Step 1: Detailed Patient History and Clinical Examination**<br>● **Review Symptoms**: Ensure a thorough evaluation of the patient's sensory symptoms, progression, and any subtle signs of motor involvement.<br>● **Past Medical History**: Re-assess the history of multiple myeloma and specific chemotherapy agents used.<br>● **Medication Review**: Evaluate all medications and supplements taken by the patient, including over-the-counter and herbal remedies.<br>**Step 2: Additional Laboratory Tests**<br>1. **Serum Protein Electrophoresis (SPEP) and Immunofixation Electrophoresis (IFE)**<br>○ **Reasoning**: To quantify and further characterize the IgG lambda paraprotein. This will help determine the extent of monoclonal gammopathy and its potential role in neuropathy.<br>2. **Complete Blood Count (CBC) with Differential**<br>○ **Reasoning**: To assess overall hematologic status and rule out anemia, infection, or other hematologic abnormalities associated with multiple myeloma.<br>3. **Renal Function Tests (Blood Urea Nitrogen, Creatinine)**<br>○ **Reasoning**: Chemotherapy and multiple myeloma can impact renal function, which can indirectly affect nerve health.<br>4. **Thyroid Function Tests (TSH, Free T4)**<br>○ **Reasoning**: Hypothyroidism can present with similar sensory neuropathy symptoms and should be excluded.<br>5. **Serum Immunoglobulins (IgA, IgM, IgG) and Free Light Chains**<br>○ **Reasoning**: To evaluate the immune status and identify any abnormalities related to multiple myeloma or associated disorders.<br>6. **Liver Function Tests (ALT, AST, ALP, Bilirubin)**<br>○ **Reasoning**: To rule out hepatic causes of neuropathy.<br>7. **Heavy Metal Screening (Lead, Mercury, Arsenic)**<br>○ **Reasoning**: Chronic exposure to heavy metals can cause sensory neuropathy and should be ruled out if there's any suspicion.<br>**Step 3: Instrumental Tests**<br>1. **Nerve Biopsy**<br>○ **Reasoning**: To obtain a definitive diagnosis if the cause of neuropathy remains unclear. It can provide information on the type of nerve damage (axonal vs. demyelinating) and specific pathological features.<br>2. **Skin Biopsy for Intraepidermal Nerve Fiber Density (IENFD)**<br>○ **Reasoning**: To quantify small fiber neuropathy, which may not be detected in standard nerve conduction studies.<br>3. **Lumbar Puncture (CSF Analysis)**<br>○ **Reasoning**: To rule out inflammatory or infectious causes of neuropathy, especially in the context of possible paraneoplastic syndromes.<br>4. **Magnetic Resonance Imaging (MRI) of the Spine**<br>○ **Reasoning**: To exclude compressive lesions or other structural abnormalities that could contribute to neuropathy symptoms.<br>**Most Crucial Test**<br>The most crucial test from the list is **Serum Protein Electrophoresis (SPEP) and Immunofixation Electrophoresis (IFE)**. This test is essential because it will provide a detailed characterization of the paraprotein, which is critical in understanding the role of monoclonal gammopathy in the patient's neuropathy. Identifying the specific properties and levels of the paraprotein can directly impact the management and treatment strategy, especially in the context of multiple myeloma and its complications.
49	A 79-year-old female patient presents with a 1-year history of pure sensory, symmetrical, length dependent polyneuropathy with insidious onset and progressive course. The patient presents	Anti-MAG polyneuropathy	**Additional Examinations**<br>1. **Serum Immunofixation Electrophoresis (IFE) and Serum Free Light Chain (FLC) Assay**<br>○ **Reasoning**: These tests further characterize the monoclonal protein detected by identifying the specific type and quantity of light chains. This is important for confirming the diagnosis of MGUS and excluding other plasma cell dyscrasias, such as multiple myeloma or Waldenström's macroglobulinemia.<br>2. **Lumbar Puncture with Cerebrospinal Fluid (CSF) Analysis**

	with distal leg paresthesia, severe ataxia and postural tremor, which she reported to appear along with the other symptoms. Clinical examination does not show presence of foot deformities, cranial nerve or autonomic involvement. Past medical history is positive for diabetes. Nerve conduction studies show demyelinating, sensory-motor, symmetric polyneuropathy. Screening lab tests show the presence of IgM kappa monoclonal gammopathy, while other exams including vitamin B12, folate, vitamin B6, vitamin E, HCV, HBV, ANA, ANCA, ENA, CRP, ESR, glycemia, HbA1c, copper and cryoglobulins are all negative. There is no family history of polyneuropathy.		○ **Reasoning**: CSF analysis can help in diagnosing CIDP by identifying elevated protein levels without pleocytosis. Oligoclonal bands in CSF may also support an inflammatory cause of neuropathy. This test can help distinguish CIDP from other demyelinating neuropathies.<br>3. **Skin Biopsy for Intraepidermal Nerve Fiber Density (IENFD)**<br>○ **Reasoning**: A skin biopsy can assess small fiber neuropathy, which might not be detected by nerve conduction studies. Reduced IENFD can indicate small fiber involvement, which may not be solely due to MGUS or diabetes.<br>4. **Antibody Testing for Myelin-Associated Glycoprotein (MAG) and Other Paraproteins**<br>○ **Reasoning**: Anti-MAG antibodies are often associated with IgM monoclonal gammopathies and can cause demyelinating neuropathy. Testing for these and other relevant antibodies (e.g., anti-GM1, anti-GQ1b) can help pinpoint the specific cause of the demyelination.<br>5. **MRI of the Spine**<br>○ **Reasoning**: MRI can reveal nerve root enhancement or thickening, which is often seen in CIDP. It can also exclude compressive causes of neuropathy and provide visual confirmation of demyelination.<br>6. **Nerve Biopsy**<br>○ **Reasoning**: Although invasive, a nerve biopsy can provide definitive evidence of the type of neuropathy (axonal vs. demyelinating) and identify characteristic features of conditions like CIDP, amyloidosis, or vasculitis-associated neuropathy.<br>**Most Crucial Test**<br>**Serum Immunofixation Electrophoresis (IFE) and Serum Free Light Chain (FLC) Assay** are the most crucial tests. Given the presence of IgM kappa monoclonal gammopathy, these tests are essential to further characterize the monoclonal protein. This will confirm the diagnosis of MGUS and exclude other plasma cell disorders that may require different management. Identifying the specific type and quantity of the monoclonal protein is pivotal in understanding the etiology of the neuropathy and guiding further diagnostic and therapeutic steps.
50	A 57-year-old female patient presents with a five-year history of chronic, sensory-motor, symmetrical, length-dependent polyneuropathy with insidious onset and slow progression. The patient reports neuropathic pain in the feet, mild distal weakness in the lower limbs, and pes cavus, with no cranial nerve or autonomic involvement. Past medical history is unremarkable. Nerve conduction studies show a mixed axonal-demyelinating, sensory-motor polyneuropathy. Screening lab tests including vitamin B12, folate, vitamin B6, vitamin E, HBV, HCV, glycemia, HbA1c, ANA, ANCA, ENA, CRP, ESR, copper, cryoglobulins, and serum electrophoresis are all negative. Her mother has similar symptoms, while her brother has severe walking difficulties.	X-linked CMT	**Step-by-Step Analysis**<br>**Clinical Data Summary:**<br>● **57-year-old female** with chronic, sensory-motor, symmetrical, length-dependent polyneuropathy.<br>● **Symptoms:** Neuropathic pain in feet, mild distal weakness in lower limbs, pes cavus.<br>● **Onset:** Insidious, slow progression.<br>● **Nerve Conduction Studies:** Mixed axonal-demyelinating, sensory-motor polyneuropathy.<br>● **Family History:** Similar symptoms in mother, severe walking difficulties in brother.<br>● **Initial Laboratory Tests:** Negative for vitamin deficiencies, infections, metabolic disorders, autoimmune markers, and other common neuropathy causes.<br>**Additional Examinations**<br>**1. Genetic Testing**<br>**Reasoning:**<br>● Given the family history and clinical presentation, genetic testing is essential to identify potential hereditary neuropathies, such as CMT or HNPP.<br>● **Target Genes:** PMP22, MPZ, GJB1 (Cx32), MFN2, GDAP1, and others associated with hereditary neuropathies.<br>**2. Nerve Biopsy**<br>**Reasoning:**<br>● A nerve biopsy can provide detailed information on the type of nerve damage (axonal vs. demyelinating) and any specific pathological features.<br>● Useful for differentiating between various types of hereditary and acquired neuropathies.<br>**3. Skin Biopsy**<br>**Reasoning:**<br>● To assess intraepidermal nerve fiber density (IENFD) and identify small fiber neuropathy, which may not be apparent on nerve conduction studies.<br>● Small fiber involvement is common in many hereditary neuropathies and some acquired conditions.<br>**4. Lumbar Puncture (CSF Analysis)**<br>**Reasoning:**<br>● To exclude inflammatory or infectious causes of neuropathy that might not have been detected in initial screening.<br>● Analysis of CSF can reveal elevated protein levels without pleocytosis, which can be seen in some hereditary neuropathies.<br>**5. Electromyography (EMG)**<br>**Reasoning:**<br>● To further evaluate the extent of muscle involvement and differentiate between neurogenic and myopathic changes.<br>● Complementary to nerve conduction studies to provide a comprehensive view of neuromuscular function.<br>**6. Autonomic Function Testing**<br>**Reasoning:**<br>● Even though there is no reported autonomic involvement, testing can reveal subclinical autonomic dysfunction, which might be present in some hereditary neuropathies.<br>**Most Crucial Test**<br>**Genetic Testing** is the most crucial test in this scenario. It directly targets the suspected hereditary nature of the neuropathy, given the strong family history and clinical presentation. Identifying a specific genetic mutation would confirm the diagnosis, guide management, and provide prognostic information.<br>**Conclusion**<br>Based on the analysis, genetic testing should be prioritized to identify specific mutations associated with hereditary neuropathies such as Charcot-Marie-Tooth Disease or Hereditary Neuropathy with Liability to Pressure Palsies. This test is pivotal in confirming the diagnosis, given the patient's family history and clinical features.
51	A 62-year-old male patient presents with an 8-month history of pure sensory, symmetrical, length dependent polyneuropathy with subacute onset. The patient presents with numbness and tingling and neuropathic pain distally in the lower limbs while he has no tremor, foot	CIDP	**Step-by-Step Analysis**<br>**Initial Assessment**<br>1. **Patient Demographics and History:**<br>○ 62-year-old male<br>○ 8-month history of pure sensory, symmetrical, length-dependent polyneuropathy<br>○ Subacute onset, with numbness, tingling, and neuropathic pain distally in the lower limbs<br>○ Sensory ataxia, no tremor, foot deformities, cranial nerve, or autonomic involvement

	deformities, cranial nerve or autonomic involvement. Clinical examination reveals the presence of sensory ataxia. Past medical history is silent. Nerve conduction studies show a demyelinating, sensory-motor polyneuropathy and unilateral carpal tunnel syndrome. Screening lab tests including vitamin B12, folate, vitamin B6, vitamin E, HCV, HBV, ANA, ANCA, ENA, CRP, ESR, glycemia, HbA1c, copper, and cryoglobulins are all negative. Serum electrophoresis shows the presence of IgG paraprotein. There is no family history of polyneuropathy.		2. **Clinical Examination:**<br>○ Sensory ataxia<br>○ Unilateral carpal tunnel syndrome<br>3. **Initial Laboratory Tests:**<br>○ Negative: Vitamin B12, folate, vitamin B6, vitamin E, HCV, HBV, ANA, ANCA, ENA, CRP, ESR, glycemia, HbA1c, copper, and cryoglobulins<br>4. **Nerve Conduction Studies:**<br>○ Demyelinating, sensory-motor polyneuropathy<br>5. **Serum Electrophoresis:**<br>○ Presence of IgG paraprotein<br>6. **Family History:**<br>○ No family history of polyneuropathy<br>**Additional Examinations**<br>1. **Bone Marrow Biopsy:**<br>○ **Reasoning:** To assess for plasma cell dyscrasias (e.g., multiple myeloma, Waldenström's macroglobulinemia) which can present with paraproteinemia and neuropathy.<br>○ **Case Specifics:** Presence of IgG paraprotein raises suspicion for an underlying plasma cell disorder.<br>2. **Quantitative Immunoglobulins and Immunofixation Electrophoresis:**<br>○ **Reasoning:** To further characterize the IgG paraprotein and evaluate for monoclonal gammopathies.<br>○ **Case Specifics:** Identifying the specific type of paraprotein can help diagnose MGUS, multiple myeloma, or other related conditions.<br>3. **Serum Free Light Chain Assay:**<br>○ **Reasoning:** To detect light chain abnormalities which are common in monoclonal gammopathies.<br>○ **Case Specifics:** Complements the findings from serum electrophoresis and helps in the diagnosis of conditions like light chain myeloma.<br>4. **Lumbar Puncture with Cerebrospinal Fluid (CSF) Analysis:**<br>○ **Reasoning:** To evaluate for inflammatory or infectious causes of neuropathy, including CIDP.<br>○ **Case Specifics:** Elevated protein without pleocytosis in CSF can support CIDP diagnosis. Helps rule out infections or other inflammatory conditions.<br>5. **CT or PET Scan:**<br>○ **Reasoning:** To screen for underlying malignancies that could be associated with paraneoplastic neuropathy.<br>○ **Case Specifics:** Paraneoplastic syndromes often present with neuropathy and can be associated with cancers such as small cell lung carcinoma.<br>6. **Anti-MAG Antibodies:**<br>○ **Reasoning:** To evaluate for antibodies associated with neuropathy and MGUS.<br>○ **Case Specifics:** Anti-MAG (myelin-associated glycoprotein) antibodies are often found in patients with neuropathy related to MGUS.<br>7. **Genetic Testing:**<br>○ **Reasoning:** To rule out hereditary neuropathies, especially given the demyelinating pattern seen in nerve conduction studies.<br>○ **Case Specifics:** While no family history is reported, late-onset hereditary neuropathies can still occur.<br>8. **Skin Biopsy:**<br>○ **Reasoning:** To evaluate for small fiber neuropathy which may not be detected on nerve conduction studies.<br>○ **Case Specifics:** Useful if there is a clinical suspicion of small fiber involvement despite normal nerve conduction study findings.<br>**Most Crucial Test**<br>**Bone Marrow Biopsy**<br>● **Reasoning:** The detection of IgG paraprotein in the context of polyneuropathy strongly suggests a potential plasma cell dyscrasia. Bone marrow biopsy is crucial for diagnosing multiple myeloma, MGUS, or other related conditions, which could directly impact the patient's management and prognosis. This test provides definitive information about the presence and extent of clonal plasma cells or lymphoplasmacytic cells, which is central to confirming or ruling out a diagnosis of multiple myeloma or other plasma cell disorders.<br>**Conclusion**<br>Conducting a bone marrow biopsy is the most crucial test given the presence of IgG paraprotein, which may indicate an underlying plasma cell dyscrasia such as MGUS or multiple myeloma, conditions directly associated with the patient's neuropathy symptoms.
52	A 77-year-old male patient presents with a 6-month history of sensory-motor, symmetrical, non-length dependent polyneuropathy with subacute onset. The patient presents with weakness distributed proximally and distally in the lower limbs and distally in the upper limbs, and paresthesia in a stocking-glove distribution. Clinical examination reveals the presence of sensory ataxia, peripheral edema and hypertrichosis. There are no foot deformities, tremor, neuropathic pain, cranial nerve or autonomic involvement. Past medical history is positive for diabetes. Nerve conduction studies show mixed demyelinating-axonal, sensory-motor polyneuropathy. Screening lab tests including vitamin B12, folate, vitamin B6, vitamin E, HCV, HBV, ANA, ANCA, ENA,	POEMS	**Step-by-Step Analysis and Additional Examinations**<br>To narrow down the differential diagnoses of the patient's polyneuropathy, further investigations are needed. These additional tests will help confirm or rule out specific conditions such as POEMS syndrome, diabetic polyneuropathy, and CIDP with paraproteinemia.<br>**Step 1: Imaging Studies**<br>1. **Whole Body CT Scan or MRI:**<br>○ **Reasoning:**<br>■ To identify organomegaly (hepatomegaly, splenomegaly, lymphadenopathy) associated with POEMS syndrome.<br>■ To detect any other masses or lesions indicative of malignancies, which can be related to monoclonal gammopathies.<br>2. **Bone Marrow Biopsy:**<br>○ **Reasoning:**<br>■ To detect the presence of plasma cell dyscrasia or myeloma, which is a part of POEMS syndrome.<br>■ To evaluate for hematologic malignancies associated with monoclonal gammopathies.<br>**Step 2: Specific Laboratory Tests**<br>3. **Serum VEGF Levels:**<br>○ **Reasoning:**<br>■ Vascular Endothelial Growth Factor (VEGF) levels are typically elevated in POEMS syndrome and can be a diagnostic marker.<br>4. **Serum Protein Electrophoresis (SPEP) and Immunofixation Electrophoresis (IFE):**<br>○ **Reasoning:**<br>■ To further characterize the monoclonal protein and determine its exact nature.

	CRP, ESR, glycemia, copper and cryoglobulins are all negative, with positivity for IgM lambda paraprotein and raised HbA1c. There is no family history of polyneuropathy.		■ To quantify the paraprotein level, which can help in diagnosing monoclonal gammopathies and POEMS syndrome.<br>5. **Thyroid Function Tests (TFTs):**<br>○ **Reasoning:**<br>■ Endocrinopathies, including thyroid abnormalities, are part of POEMS syndrome. TFTs will help identify any thyroid dysfunction.<br>6. **Serum Free Light Chains (FLC) Assay:**<br>○ **Reasoning:**<br>■ To evaluate the kappa and lambda light chains in the blood, which can be abnormal in POEMS syndrome and other monoclonal gammopathies.<br>**Step 3: Functional Tests**<br>7. **Electromyography (EMG):**<br>○ **Reasoning:**<br>■ To further assess the extent and pattern of nerve and muscle involvement, which can help differentiate between neuropathies like CIDP and POEMS syndrome.<br>8. **Echocardiogram:**<br>○ **Reasoning:**<br>■ To evaluate for cardiac abnormalities, including heart failure or restrictive cardiomyopathy, which can be associated with POEMS syndrome.<br>**Step 4: Specialized Tests**<br>9. **Nerve Biopsy:**<br>○ **Reasoning:**<br>■ To examine the pathological changes in the peripheral nerves, which can provide definitive evidence of demyelination or axonal damage.<br>■ To help differentiate between CIDP and other types of neuropathies.<br>**Conclusion: Most Crucial Test**<br>**Serum VEGF Levels**<br>● **Reasoning:**<br>○ Elevated VEGF levels are a hallmark of POEMS syndrome and can significantly aid in confirming the diagnosis.<br>○ Given the patient's presentation and the positive IgM lambda paraprotein, a high VEGF level would strongly support POEMS syndrome over other differential diagnoses.<br>By conducting these additional examinations, particularly focusing on the serum VEGF levels, the diagnostic process will be more comprehensive and directed towards confirming the most likely diagnosis of POEMS syndrome.
53	A 56-year-old male patient presents with a 13-month history of pure motor, asymmetric, non-length dependent polyneuropathy with insidious onset and progressive course. The patient presents with weakness in both hands with asymmetric distribution and reports having frequent cramps and fasciculations. Clinical examination reveals intrinsic hand atrophy. There are no sensory deficits or ataxia, tremor, cranial nerve or autonomic involvement. Past medical history is positive for systemic lupus erythematosus. Nerve conduction studies show a pure motor, demyelinating multineuropathy. Screening lab tests including vitamin B12, folate, vitamin B6, vitamin E, HCV, HBV, ANA, ANCA, ENA, CRP, ESR, glycemia, HbA1c, copper, cryoglobulins, and serum electrophoresis are negative. Cervical spine MRI is normal. There is no family history of polyneuropathy.	MMN	**Step-by-Step Analysis of the Clinical Scenario**<br>**Clinical Information Recap**<br>● **Symptoms**: Asymmetric motor weakness in both hands, cramps, fasciculations, intrinsic hand atrophy<br>● **History**: Systemic lupus erythematosus (SLE)<br>● **Examinations**: Pure motor demyelinating neuropathy on nerve conduction studies, normal cervical spine MRI, comprehensive negative lab work, no sensory deficits, no family history of polyneuropathy<br>**Additional Examinations**<br>1. **Anti-GM1 Antibodies Testing**<br>○ **Reasoning**: Anti-GM1 antibodies are often elevated in patients with Multifocal Motor Neuropathy (MMN). Testing for these antibodies can help confirm MMN, which is high on the differential list due to the patient's pure motor symptoms and demyelinating neuropathy.<br>○ **Importance**: Presence of anti-GM1 antibodies would strongly support MMN as the diagnosis.<br>2. **Lumbar Puncture with Cerebrospinal Fluid (CSF) Analysis**<br>○ **Reasoning**: CSF analysis can help differentiate between ALS and other demyelinating conditions. Elevated protein without pleocytosis (increased white blood cells) is common in CIDP. While ALS often shows normal CSF findings, CIDP would typically show elevated protein.<br>○ **Importance**: Elevated CSF protein would support CIDP, while normal CSF findings would make ALS more likely.<br>3. **Repetitive Nerve Stimulation (RNS)**<br>○ **Reasoning**: RNS is used to evaluate neuromuscular transmission disorders. Although less specific, it can help rule out conditions like myasthenia gravis, which can sometimes present with motor symptoms.<br>○ **Importance**: Helps rule out other neuromuscular junction disorders that might mimic the patient's symptoms.<br>4. **Genetic Testing for Hereditary Neuropathies**<br>○ **Reasoning**: Although there is no family history, some hereditary neuropathies can present sporadically. Genetic testing can help identify conditions like hereditary motor neuropathies that might not have a clear family history.<br>○ **Importance**: Negative results would help focus on acquired causes.<br>5. **MRI Brain and Spine**<br>○ **Reasoning**: An MRI can help rule out structural lesions or other CNS causes of motor weakness that might not have been detected in the cervical spine MRI. ALS sometimes shows corticospinal tract degeneration.<br>○ **Importance**: To ensure there is no central nervous system pathology contributing to the symptoms.<br>6. **EMG (Electromyography)**<br>○ **Reasoning**: EMG can help differentiate between neuropathic and myopathic processes and can show denervation patterns typical of ALS.<br>○ **Importance**: Supports the diagnosis of ALS if there is widespread denervation.<br>**Most Crucial Test**<br>**Anti-GM1 Antibodies Testing**<br>**Justification**:<br>● The patient's clinical presentation and nerve conduction study findings align closely with Multifocal Motor Neuropathy (MMN). Confirming the presence of anti-GM1 antibodies would be a strong indicator of MMN and could lead to appropriate treatment (e.g., IVIg therapy).<br>● This test is less invasive compared to lumbar puncture and provides specific diagnostic information that directly targets the leading differential diagnosis based on the presented case.<br>**Conclusion**<br>The additional examinations listed aim to further narrow down the differential diagnoses, primarily focusing on distinguishing between MMN, ALS, and CIDP. Among these, testing for anti-GM1 antibodies is the most crucial as it directly targets the most likely diagnosis (MMN) based on the clinical scenario and available data.
54	A 74-year-old male patient presents with a 5-year history of sensory-motor,	Diabetic neuropathy	**Step-by-Step Analysis and Additional Examinations**<br>Given the clinical scenario, here is a structured approach to further evaluate the patient's condition:

	predominantly sensory, symmetrical, length dependent polyneuropathy with insidious onset and slow progressive course. The patient presents with distal paresthesia and hypoesthesia associated with mild neuropathic pain in the feet. At clinical examination there are no tremor, foot deformities, cranial nerve or autonomic involvement. Past medical history is positive for diabetes and cardiac insufficiency. Nerve conduction studies show a sensory-motor, symmetric, length dependent, axonal polyneuropathy. Screening lab tests show hyperglycemia and elevated HbA1c. Remaining exams including vitamin B12, folate, vitamin B6, vitamin E, HBV, HCV, ANA, ANCA, ENA, CRP, ESR, copper, cryoglobulins, and serum electrophoresis are negative. There is family history of polyneuropathy (father had similar symptoms).		1. **Review of Existing Data**<br>○ History and physical exam indicate a sensory-motor, predominantly sensory, length-dependent polyneuropathy.<br>○ Past medical history includes diabetes and cardiac insufficiency.<br>○ Lab tests show hyperglycemia and elevated HbA1c.<br>○ Nerve conduction studies show axonal polyneuropathy.<br>○ Family history of similar symptoms suggests possible hereditary neuropathy.<br>2. **Necessary Additional Examinations**<br>**Laboratory Tests**<br>**1. Glycated Albumin:**<br>● **Reasoning:** Provides information on short-term glucose control, useful to correlate with long-term HbA1c levels and assess ongoing glycemic variability.<br>**2. Genetic Testing for Hereditary Neuropathies:**<br>● **Reasoning:** Given the family history, testing for genetic mutations associated with hereditary sensory and motor neuropathies (HSMN) like Charcot-Marie-Tooth disease can confirm a genetic cause.<br>**3. Glucose Tolerance Test:**<br>● **Reasoning:** To better characterize glucose metabolism abnormalities that might not be evident with fasting glucose or HbA1c alone, particularly in patients with suspected prediabetes or early diabetes.<br>**4. Immunofixation Electrophoresis and Free Light Chains:**<br>● **Reasoning:** To rule out monoclonal gammopathy of undetermined significance (MGUS), multiple myeloma, or other plasma cell dyscrasias, which can present with polyneuropathy.<br>**5. Antibody Testing for Paraneoplastic Syndromes:**<br>● **Reasoning:** Despite the lack of systemic signs, paraneoplastic syndromes can sometimes present primarily with neuropathy. Testing for antibodies like anti-Hu, anti-Ri, and anti-Yo can help rule this out.<br>**6. CSF Analysis (Lumbar Puncture):**<br>● **Reasoning:** To evaluate for elevated protein levels or other markers indicative of inflammatory or infectious causes of neuropathy, such as chronic inflammatory demyelinating polyneuropathy (CIDP) or Guillain-Barré syndrome.<br>**Instrumental Tests**<br>**1. MRI of the Spine and Brain:**<br>● **Reasoning:** To rule out structural causes of neuropathy such as compressive lesions, tumors, or demyelinating diseases like multiple sclerosis.<br>**2. Skin Biopsy:**<br>● **Reasoning:** To evaluate intraepidermal nerve fiber density, which can help in diagnosing small fiber neuropathy, especially if standard nerve conduction studies are inconclusive.<br>**3. Autonomic Testing:**<br>● **Reasoning:** Though there is no overt autonomic involvement, subtle autonomic dysfunction could be present. Tests include heart rate variability, tilt-table testing, and sudomotor function tests.<br>**4. Repeated Nerve Conduction Studies and EMG:**<br>● **Reasoning:** To monitor progression and help differentiate between axonal and demyelinating neuropathies, especially if initial results were borderline.<br>**Most Crucial Test**<br>**Genetic Testing for Hereditary Neuropathies**<br>● **Reasoning:** Given the significant family history of similar symptoms, genetic testing is critical to confirm or rule out hereditary sensory and motor neuropathies. Identifying a genetic cause will not only aid in diagnosis but also impact management and counseling for the patient and his family members.<br>This test stands out as the most crucial due to the hereditary component observed in the patient's history, which aligns with the clinical presentation and progression of the disease.
55	A 70-year-old male patient presents with a 2-year history of sensory-motor, symmetrical, length dependent polyneuropathy with subacute onset. He has been suffering from neuropathic pain in his feet for several years. However, over the past two years, he has also noticed progressive weakness in the feet and wasting of the intrinsic muscles of both hands. Additionally, he reports symptoms suggestive of orthostatic hypothension, and unintentional weight loss of approximately 5 kg over the past year. Clinical examination does not show presence of tremor, foot deformities, or cranial nerve involvement. Past medical history is positive for cardiac insufficiency, diabetes, and IgG lambda MGUS. Nerve conduction studies show a mixed demyelinating-axonal, sensory-motor, symmetric polyneuropathy. Screening lab tests reveal hyperglycemia, elevated HbA1c, presence of IgG lambda paraprotein, and increased creatinine levels. Remaining exams including vitamin B12, folate, vitamin B6, vitamin E, HBV, HCV, ANA, ANCA, ENA, CRP, ESR, copper, and cryoglobulins are normal. There is no family history of polyneuropathy.	Amyloidosis-associated polyneuropathy	**Step-by-Step Analysis and Additional Examinations**<br>1. **Review of Current Data**<br>○ **Symptoms**: Sensory-motor, symmetrical polyneuropathy; neuropathic pain; progressive weakness; muscle wasting; orthostatic hypotension; weight loss.<br>○ **History**: Diabetes, cardiac insufficiency, IgG lambda MGUS.<br>○ **Nerve Conduction Studies**: Mixed demyelinating-axonal polyneuropathy.<br>○ **Lab Findings**: Hyperglycemia, elevated HbA1c, IgG lambda paraprotein, increased creatinine, normal vitamin and infection panels.<br>○ **Examination**: No tremor, foot deformities, or cranial nerve involvement.<br>2. **List of Necessary Additional Examinations**<br>**a. Serum and Urine Immunofixation Electrophoresis (IFE) and Free Light Chain Assay**<br>○ **Reasoning**: To quantify and further characterize the monoclonal protein and to detect the presence of free light chains, which are crucial for diagnosing AL amyloidosis.<br>3. **b. Fat Pad Biopsy or Minor Salivary Gland Biopsy**<br>○ **Reasoning**: To confirm the presence of amyloid deposits, which would support a diagnosis of AL amyloidosis. These sites are minimally invasive and often yield diagnostic material.<br>4. **c. Echocardiogram**<br>○ **Reasoning**: To evaluate cardiac function and detect amyloid infiltration, which can manifest as cardiac insufficiency.<br>5. **d. Autonomic Function Testing**<br>○ **Reasoning**: To objectively assess the extent of autonomic dysfunction, which is suggested by symptoms of orthostatic hypotension.<br>6. **e. Electromyography (EMG)**<br>○ **Reasoning**: To provide additional detail on the nature of the neuropathy, distinguishing between demyelinating and axonal components and assessing the extent of muscle involvement.<br>7. **f. Serum Protein Electrophoresis (SPEP)**<br>○ **Reasoning**: To further quantify monoclonal protein and assess for potential multiple myeloma or other plasma cell disorders.<br>8. **g. Bone Marrow Biopsy**<br>○ **Reasoning**: To investigate underlying hematologic disorders such as multiple myeloma or other plasma cell dyscrasias, especially in the presence of MGUS and a polyneuropathy suggestive of AL amyloidosis.<br>9. **h. Renal Function Tests (including 24-hour urine protein)**<br>○ **Reasoning**: To assess for renal involvement, which can be affected in both diabetes and AL amyloidosis, especially given the elevated creatinine levels.<br>10. **i. Holter Monitoring**<br>○ **Reasoning**: To detect any cardiac arrhythmias or conduction abnormalities which might not be apparent on a routine EKG and could be associated with amyloidosis.<br>11. **Most Crucial Test**<br>The **Fat Pad Biopsy or Minor Salivary Gland Biopsy** is the most crucial test.<br>● **Reasoning**:

			○ It directly tests for the presence of amyloid deposits, which is pivotal in confirming or ruling out AL amyloidosis.<br>○ Given the patient's history of IgG lambda MGUS and the clinical signs suggestive of amyloidosis, confirming the presence of amyloid is essential for accurate diagnosis and subsequent management.<br>**Conclusion**<br>While all proposed tests provide valuable information and help narrow down the differential diagnoses, the fat pad biopsy or minor salivary gland biopsy directly addresses the suspected underlying cause—amyloid deposition—thereby making it the most crucial step in the diagnostic workup for this patient.
56	A 53-year-old female patient presents with a 42-year history of sensory-motor, symmetrical, length-dependent polyneuropathy with insidious onset and slowly progressive course. The patient reports distal leg paresthesia, scoliosis, sensory ataxia, distal weakness in the lower limbs, cramps, hammertoes, and pes cavus. Clinical examination does not reveal tremor, cranial nerve involvement, or autonomic dysfunction. Past medical history is unremarkable. Nerve conduction studies show a demyelinating, symmetric, sensory-motor polyneuropathy. Screening lab tests including vitamin B12, folate, vitamin B6, vitamin E, HBV, HCV, glycemia, HbA1c, ANA, ANCA, ENA, CRP, ESR, copper, cryoglobulins, and serum electrophoresis are all negative. Her mother, brother, and sister have walking difficulties.	CMT1	**Step-by-Step Analysis and Additional Examinations**<br>**Step 1: Review Clinical Presentation and Initial Findings**<br>● **Patient Details**: 53-year-old female with a 42-year history of sensory-motor, symmetrical, length-dependent polyneuropathy.<br>● **Symptoms**: Distal leg paresthesia, scoliosis, sensory ataxia, distal weakness in lower limbs, cramps, hammertoes, pes cavus.<br>● **Family History**: Mother, brother, and sister with walking difficulties.<br>● **Initial Tests**: Negative for vitamin B12, folate, vitamin B6, vitamin E, HBV, HCV, glycemia, HbA1c, ANA, ANCA, ENA, CRP, ESR, copper, cryoglobulins, and serum electrophoresis.<br>● **Nerve Conduction Studies**: Demyelinating, symmetric, sensory-motor polyneuropathy.<br>**Step 2: Identifying Additional Examinations**<br>1. **Genetic Testing for Hereditary Neuropathies**<br>○ **Reasoning**: Given the family history and clinical presentation, genetic testing can help identify specific mutations associated with Charcot-Marie-Tooth disease (CMT) and other hereditary neuropathies (e.g., PMP22, GJB1, MFN2, MPZ genes).<br>2. **Electromyography (EMG)**<br>○ **Reasoning**: EMG can help distinguish between primary nerve and muscle disorders, providing additional information on the extent and type of neuromuscular involvement.<br>3. **Lumbar Puncture with Cerebrospinal Fluid (CSF) Analysis**<br>○ **Reasoning**: While unlikely in a hereditary condition, CSF analysis can help rule out chronic inflammatory demyelinating polyneuropathy (CIDP) by detecting elevated protein levels without pleocytosis.<br>4. **Nerve Biopsy**<br>○ **Reasoning**: In cases where the diagnosis remains unclear, a nerve biopsy can provide definitive histopathological evidence of demyelination, axonal loss, or inflammatory changes.<br>5. **MRI of the Spine and Lower Limbs**<br>○ **Reasoning**: MRI can detect structural abnormalities, nerve root or plexus involvement, and can help in the assessment of scoliosis and other skeletal deformities related to neuropathy.<br>6. **Ophthalmologic Examination**<br>○ **Reasoning**: Some hereditary neuropathies can be associated with retinal abnormalities (e.g., optic neuropathy in certain types of CMT), so an ophthalmologic evaluation can provide supportive evidence.<br>7. **Autonomic Testing**<br>○ **Reasoning**: To definitively rule out autonomic involvement, comprehensive autonomic testing (e.g., heart rate variability, sweat test) can provide additional information.<br>**Step 3: Prioritizing the Most Crucial Test**<br>● **Most Crucial Test**: **Genetic Testing for Hereditary Neuropathies**<br>○ **Reasoning**: Genetic testing is the most definitive and non-invasive method to confirm the diagnosis of hereditary neuropathies like Charcot-Marie-Tooth disease. Given the patient's family history and the clinical presentation, identifying the specific genetic mutation can confirm the diagnosis and guide management and genetic counseling for the family.<br>**Conclusion**<br>The most crucial test in this case is **genetic testing for hereditary neuropathies**. This test will provide a definitive diagnosis, clarify the specific type of neuropathy, and support appropriate management and genetic counseling for the patient and her family.
57	A 65-year old male patient presents with a 3-year history of sensory, symmetrical, length dependent polyneuropathy with insidious onset. The patient presents with sensory ataxia, neuropathic pain, distal hypoesthesia up to the calves and mild dysarthria. Clinical examination does not reveal presence of foot deformities, tremor, or weakness. Past medical history shows chronic cough and mild cardiac insufficiency. Nerve conduction studies show a pure sensory, axonal polyneuropathy. Screening lab tests including vitamin B12, folate, vitamin B6, vitamin E, HBV, HCV, glycemia, HbA1c, ANA, ANCA, ENA, CRP, ESR, copper, cryoglobulins, and serum electrophoresis are all negative. There is no family history of polyneuropathy.	CANVAS	**Step-by-Step Analysis and Additional Examinations**<br>**Step 1: Review of Clinical and Diagnostic Data**<br>● **Patient Profile**: 65-year-old male<br>● **Symptoms**: Sensory ataxia, neuropathic pain, distal hypoesthesia up to the calves, mild dysarthria<br>● **Nerve Conduction Studies**: Pure sensory, axonal polyneuropathy<br>● **Negative Screening Tests**: Vitamin B12, folate, vitamin B6, vitamin E, HBV, HCV, glycemia, HbA1c, ANA, ANCA, ENA, CRP, ESR, copper, cryoglobulins, serum electrophoresis<br>● **Past Medical History**: Chronic cough, mild cardiac insufficiency<br>● **Family History**: No family history of polyneuropathy<br>**Step 2: Identification of Necessary Additional Examinations**<br>1. **High-Resolution Chest CT Scan**<br>○ **Reasoning**: Given the chronic cough and mild cardiac insufficiency, an occult malignancy such as lung cancer could be present, which might lead to paraneoplastic neuropathy. A chest CT can help identify any hidden tumors or abnormalities in the thoracic region.<br>○ **Specifics**: Detection of neoplasms or lymphadenopathy that might not be evident on basic imaging.<br>2. **CSF Analysis (Lumbar Puncture)**<br>○ **Reasoning**: Examining cerebrospinal fluid (CSF) can help identify inflammatory or infectious processes, and certain cancers that might be missed by blood tests. Protein levels, cell count, and specific markers can be assessed.<br>○ **Specifics**: Elevated protein without pleocytosis might indicate chronic inflammatory demyelinating polyneuropathy (CIDP) or a paraneoplastic syndrome.<br>3. **Heavy Metal Screening (Blood and Urine)**<br>○ **Reasoning**: Although initial screening tests were negative, detailed testing for heavy metals like lead, arsenic, and mercury could identify toxic neuropathy, especially if there's a history of exposure.<br>○ **Specifics**: Detailed heavy metal panel to quantify levels in blood and urine.<br>4. **PET-CT Scan**<br>○ **Reasoning**: If there's a strong suspicion of paraneoplastic syndrome and the chest CT is inconclusive, a PET-CT can help detect occult malignancies throughout the body.<br>○ **Specifics**: Detects metabolic activity of cancer cells, aiding in locating hidden tumors.<br>5. **Anti-Hu, Anti-Ri, and Anti-Yo Antibodies (Paraneoplastic Panel)**<br>○ **Reasoning**: Testing for these antibodies can help diagnose paraneoplastic syndromes. These are associated with cancers such as small-cell lung cancer and can cause neuropathy.<br>○ **Specifics**: Presence of these antibodies would strongly suggest a paraneoplastic syndrome.<br>6. **Genetic Testing for Hereditary Neuropathies**

			○ **Reasoning**: Despite the absence of family history, late-onset hereditary neuropathies can occur sporadically. Genetic testing can identify mutations associated with conditions like hereditary sensory and autonomic neuropathy (HSAN).<br>○ **Specifics**: Sequencing for common mutations associated with hereditary neuropathies.<br>7. **Comprehensive Autoimmune Panel**<br>○ **Reasoning**: While some autoimmune markers have been tested, an extended panel including antibodies not initially covered might be revealing (e.g., anti-MAG, anti-GM1, anti-GAD).<br>○ **Specifics**: Broader assessment of potential autoimmune etiologies.<br>8. **Repeat Nerve Biopsy**<br>○ **Reasoning**: If all other tests are inconclusive, a repeat nerve biopsy might provide histopathological insights into the neuropathy, identifying small fiber involvement or inflammatory infiltrates.<br>○ **Specifics**: Examination of nerve tissue for structural changes, inflammation, or amyloid deposits.<br>**Step 3: Determining the Most Crucial Test**<br>Based on the clinical scenario and the need to identify or rule out serious underlying conditions, the **most crucial test** to conduct is a **High-Resolution Chest CT Scan**. This test is prioritized due to the patient's chronic cough and mild cardiac insufficiency, which raise concerns about an occult malignancy that could be causing a paraneoplastic syndrome leading to polyneuropathy.<br>● **High-Resolution Chest CT Scan**:<br>○ **Reasoning**: It directly addresses the potential underlying cause related to the patient's respiratory symptoms and mild cardiac insufficiency. It is a non-invasive test that can reveal significant pathologies not evident in standard screening tests.<br>○ **Expected Outcome**: Identification or exclusion of pulmonary or mediastinal tumors, which would significantly impact the differential diagnosis and subsequent management.<br>By conducting this scan, a significant step can be made towards either confirming or ruling out a serious underlying cause, thereby guiding further necessary investigations and treatment plans.
58	A 71-year-old female patient presents with a 2-year history of chronic, pure sensory, symmetrical, length dependent polyneuropathy with subacute onset. The patient presents with distal leg paresthesia and neuropathic pain associated with cramps. There are no foot deformities, weakness, ataxia, tremor, cranial nerve or autonomic involvement. Past medical history shows diabetes and lymphoma, for which the patient has undergone chemotherapy. Symptoms started during chemotherapy. Nerve conduction studies show mixed sensory-motor, axonal, symmetric polyneuropathy and unilateral carpal tunnel syndrome. Screening lab tests including vitamin B12, folate, vitamin B6, vitamin E, HBV, HCV, ANA, ANCA, ENA, CRP, ESR, glycemia, copper, cryoglobulins and serum electrophoresis are all negative. HbA1c is elevated. There is no family history of polyneuropathy.	Chemotherapy-induced neuropathy	**Step-by-Step Analysis and Additional Examinations**<br>**Step 1: Review Current Information**<br>● **Patient History:**<br>○ 71-year-old female<br>○ 2-year history of chronic, pure sensory, symmetrical, length-dependent polyneuropathy<br>○ Symptoms started during chemotherapy for lymphoma<br>○ History of diabetes mellitus with elevated HbA1c<br>● **Current Symptoms:**<br>○ Distal leg paresthesia, neuropathic pain, and cramps<br>○ No weakness, ataxia, tremor, cranial nerve, or autonomic involvement<br>● **Nerve Conduction Studies:**<br>○ Mixed sensory-motor, axonal, symmetric polyneuropathy<br>○ Unilateral carpal tunnel syndrome<br>● **Laboratory Results:**<br>○ Negative for various nutritional deficiencies, infections, autoimmune markers, and other common causes of neuropathy<br>● **Family History:**<br>○ No family history of polyneuropathy<br>**Step 2: Additional Examinations**<br>1. **Detailed Nerve Conduction Studies (NCS) and Electromyography (EMG)**<br>○ **Reasoning:**<br>■ To further characterize the extent and type of neuropathy, differentiating between axonal and demyelinating patterns.<br>■ To identify any subtle motor involvement that might have been missed previously.<br>○ **Expected Outcome:**<br>■ Confirmation of sensory-motor axonal neuropathy and assessment of any progression or new findings.<br>2. **Lumbar Puncture with Cerebrospinal Fluid (CSF) Analysis**<br>○ **Reasoning:**<br>■ To rule out inflammatory or infectious causes of neuropathy, which might not be evident in blood tests.<br>■ CSF analysis can help identify protein levels, cells, and markers indicative of paraneoplastic syndromes or other neuropathies.<br>○ **Expected Outcome:**<br>■ Normal CSF findings would support a diagnosis of CIPN or DPN over inflammatory neuropathies.<br>3. **Skin Biopsy**<br>○ **Reasoning:**<br>■ To assess small fiber neuropathy, which might not be detectable on NCS/EMG.<br>■ Small fiber neuropathy can cause significant sensory symptoms and is often associated with diabetes and chemotherapy.<br>○ **Expected Outcome:**<br>■ Abnormal epidermal nerve fiber density would indicate small fiber neuropathy.<br>4. **Autoantibody Panel (Expanded)**<br>○ **Reasoning:**<br>■ To detect less common or specific autoantibodies that might be missed in routine panels.<br>■ Especially relevant in the context of a potential paraneoplastic syndrome.<br>○ **Expected Outcome:**<br>■ Negative results would support non-autoimmune etiologies like CIPN or DPN.<br>5. **Quantitative Sensory Testing (QST)**<br>○ **Reasoning:**<br>■ To quantify sensory deficits and pain thresholds, providing objective measures of sensory function.<br>■ Helps in differentiating the extent and type of sensory involvement (thermal, mechanical, etc.).

			○ **Expected Outcome:**<br>■ Results consistent with the clinical presentation of sensory neuropathy.<br>6. **HbA1c and Blood Glucose Monitoring**<br>○ **Reasoning:**<br>■ To assess and manage blood sugar levels, as poor glycemic control can exacerbate neuropathy.<br>■ Helps in differentiating diabetic neuropathy from other causes.<br>○ **Expected Outcome:**<br>■ Elevated HbA1c would support diabetic neuropathy as a contributing factor.<br>**Most Crucial Test**<br>**Lumbar Puncture with Cerebrospinal Fluid (CSF) Analysis** is the most crucial test to perform. This test is essential because it can help rule out or confirm inflammatory, infectious, and paraneoplastic causes of neuropathy. Given the patient's history of lymphoma and the onset of symptoms during chemotherapy, CSF analysis can provide critical information about the underlying pathology, differentiating between CIPN, paraneoplastic neuropathy, or other potential causes.<br>**Conclusion**<br>To narrow down the differential diagnoses for this patient's polyneuropathy, the following additional examinations are recommended:<br>1. Detailed NCS and EMG<br>2. Lumbar puncture with CSF analysis<br>3. Skin biopsy<br>4. Expanded autoantibody panel<br>5. Quantitative sensory testing<br>6. HbA1c and blood glucose monitoring<br>Among these, the **lumbar puncture with CSF analysis** is the most crucial as it can provide decisive information to rule out serious underlying conditions like paraneoplastic syndrome and other inflammatory or infectious causes of neuropathy.
59	An 80-year-old male patient presents with a 1-year history of sensory-motor, symmetrical, length dependent polyneuropathy with subacute onset. The patient presents with paresthesia and sensory ataxia without tremor, neuropathic pain, foot deformities, cranial nerve or autonomic involvement. Clinical examination shows distal weakness and sensory deficits (hypoesthesia) in the lower limbs. Past medical history is positive for colorectal cancer and plasmacytoma, for which the patient has undergone chemotherapy. Neuropathy symptoms started a few weeks after the last chemotherapy treatment cycle. Nerve conduction studies show a sensory-motor, symmetric, length-dependent, axonal polyneuropathy. Screening lab tests including vitamin B12, folate, vitamin B6, vitamin E, HBV, HCV, ANA, ANCA, ENA, CRP, ESR, glycemia, HbA1c, copper and cryoglobulins are all negative, with positivity for IgG kappa paraprotein. There is no family history of polyneuropathy.	Chemotherapy-induced neuropathy	**Step-by-Step Analysis**<br>To thoroughly analyze the patient's clinical scenario, we will list additional necessary examinations, providing detailed reasoning for each.<br>**1. Serum and Urine Protein Electrophoresis with Immunofixation**<br>**Reasoning:**<br>● The presence of IgG kappa paraprotein necessitates further evaluation to determine the extent and significance of monoclonal gammopathy.<br>● Protein electrophoresis and immunofixation can identify and quantify monoclonal proteins, distinguishing between monoclonal gammopathy of undetermined significance (MGUS), multiple myeloma, or related disorders.<br>● This is crucial to differentiate paraproteinemic neuropathy from other causes.<br>**2. Nerve Biopsy**<br>**Reasoning:**<br>● Although invasive, a nerve biopsy can provide definitive information about the type of neuropathy.<br>● It can reveal amyloid deposits (indicative of amyloidosis) or inflammatory changes (indicative of vasculitis or other inflammatory neuropathies).<br>● Given the patient's history of cancer and paraproteinemia, ruling out amyloid neuropathy or vasculitic neuropathy is critical.<br>**3. Electromyography (EMG)**<br>**Reasoning:**<br>● EMG can complement nerve conduction studies by assessing muscle innervation and distinguishing between axonal and demyelinating neuropathies.<br>● It helps in confirming the extent and type of neuropathic involvement, providing a more detailed neuromuscular assessment.<br>**4. Bone Marrow Biopsy**<br>**Reasoning:**<br>● Given the history of plasmacytoma and presence of IgG kappa paraprotein, a bone marrow biopsy is indicated to assess for multiple myeloma or other plasma cell dyscrasias.<br>● This test will help determine if the paraproteinemia is related to a systemic condition that could contribute to neuropathy.<br>**5. Anti-Hu, Anti-Ri, and Other Paraneoplastic Antibodies**<br>**Reasoning:**<br>● Paraneoplastic neuropathies can be associated with antibodies against neuronal proteins.<br>● Testing for anti-Hu, anti-Ri, and other paraneoplastic antibodies can help diagnose paraneoplastic syndromes, particularly given the history of colorectal cancer.<br>**6. MRI of the Spine and Brain**<br>**Reasoning:**<br>● Imaging studies can help rule out structural causes of neuropathy and identify potential central nervous system involvement.<br>● MRI can detect spinal cord compression, demyelinating lesions, or metastatic disease that may present with similar neurological symptoms.<br>**Most Crucial Test**<br>**Serum and Urine Protein Electrophoresis with Immunofixation**<br>**Reasoning:**<br>● This test directly assesses the presence and type of monoclonal protein, which is highly relevant given the detected IgG kappa paraprotein.<br>● It provides critical information to distinguish between different hematologic conditions, such as MGUS, multiple myeloma, or other plasma cell dyscrasias, which can influence the treatment approach and prognosis.<br>● Given the clinical scenario, this test is most directly related to the patient's symptoms and has the potential to significantly impact clinical management and further diagnostic pathways.<br>**Summary of Additional Examinations**<br>1. Serum and Urine Protein Electrophoresis with Immunofixation<br>2. Nerve Biopsy<br>3. Electromyography (EMG)<br>4. Bone Marrow Biopsy<br>5. Anti-Hu, Anti-Ri, and Other Paraneoplastic Antibodies<br>6. MRI of the Spine and Brain<br>By following this step-by-step approach and prioritizing serum and urine protein electrophoresis with immunofixation, the clinician can narrow down the differential diagnoses effectively and tailor the patient's management plan accordingly.

60	A 22-year-old male patient presents with a 20-year history of sensory-motor, symmetrical, length dependent polyneuropathy with insidious onset and slow progression. The patient is wheelchair bound and presents with sensory ataxia, severe distal weakness and atrophy in the four limbs, hypoesthesia up to calf and pes cavus, with no cranial nerve or autonomic involvement. Past medical history is unremarkable. Nerve conduction studies show a demyelinating, sensory-motor polyneuropathy. Screening lab tests including vitamin B12, folate, vitamin B6, vitamin E, HBV, HCV, glycemia, HbA1c, ANA, ANCA, ENA, CRP, ESR, copper, cryoglobulins, and serum electrophoresis are all negative. One of his five brothers has similar symptoms.	CMT4	**Step-by-Step Analysis and Additional Examinations**<br>**Comprehensive Data Analysis:**<br>● **Symptoms**: Sensory-motor, symmetrical, length-dependent polyneuropathy; insidious onset, slow progression; sensory ataxia; severe distal weakness and atrophy in four limbs; hypoesthesia up to calf; pes cavus.<br>● **Neurological Findings**: No cranial nerve involvement; no autonomic involvement.<br>● **Diagnostic Findings**: Demyelinating, sensory-motor polyneuropathy on nerve conduction studies.<br>● **Laboratory Tests**: Negative for vitamin B12, folate, vitamin B6, vitamin E, HBV, HCV, glycemia, HbA1c, ANA, ANCA, ENA, CRP, ESR, copper, cryoglobulins, serum electrophoresis.<br>● **Family History**: One of five brothers with similar symptoms.<br>**Additional Examinations:**<br>1. **Genetic Testing:**<br>○ **Reasoning**: Given the strong suspicion of a hereditary neuropathy like Charcot-Marie-Tooth disease (CMT), genetic testing is essential. Identifying mutations in genes such as PMP22, MPZ, GJB1, or MFN2 can confirm the diagnosis of CMT and specify the subtype.<br>○ **Specifics**: Whole exome sequencing or targeted gene panels for known CMT-associated genes.<br>2. **Nerve Biopsy:**<br>○ **Reasoning**: A nerve biopsy can provide histological confirmation of the type of neuropathy, showing features like onion bulb formations characteristic of demyelinating neuropathies. It is especially useful if genetic testing is inconclusive.<br>○ **Specifics**: Sural nerve biopsy to examine nerve fiber degeneration, demyelination, and potential inflammatory infiltrates.<br>3. **MRI of the Spinal Cord and Brain:**<br>○ **Reasoning**: MRI can help rule out central nervous system causes of ataxia and provide detailed images of peripheral nerve involvement. It can identify nerve hypertrophy typical of CMT.<br>○ **Specifics**: MRI focusing on the lumbosacral plexus and spinal cord to visualize any structural abnormalities.<br>4. **Electromyography (EMG):**<br>○ **Reasoning**: EMG complements nerve conduction studies and provides information on the extent of muscle involvement and the nature of neuropathic damage. It helps in differentiating between axonal and demyelinating neuropathies.<br>○ **Specifics**: EMG of distal and proximal muscles in all four limbs.<br>5. **Skin Biopsy:**<br>○ **Reasoning**: A skin biopsy can help in diagnosing small fiber neuropathies and some forms of HSAN. It provides information on intraepidermal nerve fiber density.<br>○ **Specifics**: Skin biopsy from the distal leg to assess small fiber neuropathy.<br>6. **Serum Phytanic Acid Levels:**<br>○ **Reasoning**: To rule out Refsum disease, which is a differential diagnosis given the clinical presentation. Elevated phytanic acid levels are diagnostic for Refsum disease.<br>○ **Specifics**: Quantitative measurement of phytanic acid in blood.<br>**Most Crucial Test:**<br>The most crucial test from this list is **Genetic Testing**. It directly addresses the high suspicion of a hereditary neuropathy like CMT, is non-invasive compared to a nerve biopsy, and can definitively diagnose or rule out specific genetic mutations responsible for the patient's condition. Given the family history and clinical presentation, confirming the genetic mutation will provide a clear diagnosis and guide further management and genetic counseling for the family.
61	A 71-year-old male patient presents with a 4-year history of pure sensory, symmetrical, length dependent polyneuropathy with insidious onset. The patient presents with neuropathic pain and paresthesia in the feet. There are no sensory ataxia or tremor, foot deformities, cranial nerve or autonomic involvement. Clinical examination shows the presence of tactile hypoesthesia and hyperalgesia in the feet. Past medical history is positive for psoriasis and type 2 diabetes. Nerve conduction studies show a pure sensory, symmetric, axonal, length dependent polyneuropathy and unilateral carpal tunnel syndrome. Screening lab tests show positivity for ANA antibodies, mildly raised CRP and ESR, increased levels of glycemia and HbA1c. Remaining exams, including vitamin B12, vitamin B6, vitamin E, HBV, HCV, ANCA, ENA, copper, cryoglobulins and serum electrophoresis are all negative. There is no family history of polyneuropathy.	Diabetic neuropathy	**Step-by-Step Additional Examinations Analysis**<br>To narrow down the differential diagnoses for the patient’s polyneuropathy, a comprehensive approach involving additional laboratory and instrumental examinations is necessary. Here’s a detailed breakdown of the necessary tests and their rationale:<br>**1. Glucose Tolerance Test (GTT)**<br>**Reasoning:**<br>● **Purpose:** To assess the degree of glucose intolerance or insulin resistance, providing more detail about the patient’s glycemic control beyond HbA1c.<br>● **Rationale:** Although the patient has a known history of diabetes, the GTT can offer insights into postprandial glucose spikes, which may contribute to neuropathic symptoms.<br>**2. Nerve Biopsy**<br>**Reasoning:**<br>● **Purpose:** To obtain histopathological information about the nerve, which can help differentiate between various types of neuropathies, including inflammatory, vasculitic, and amyloid neuropathies.<br>● **Rationale:** In a case with overlapping clinical features, a nerve biopsy can provide definitive evidence regarding the nature of the neuropathy, particularly if there is suspicion of CIDP or other atypical neuropathies.<br>**3. Skin Biopsy**<br>**Reasoning:**<br>● **Purpose:** To evaluate small nerve fiber density, which is useful for diagnosing small fiber neuropathy.<br>● **Rationale:** The patient’s symptoms of hyperalgesia and paresthesia in the feet are suggestive of small fiber involvement. Skin biopsy is a minimally invasive method to confirm this.<br>**4. Autoimmune Panel (Extended)**<br>**Reasoning:**<br>● **Purpose:** To screen for additional autoimmune markers such as anti-Ro, anti-La, anti-dsDNA, anti-Smith, anti-RNP, and complement levels (C3, C4).<br>● **Rationale:** The presence of positive ANA and mildly raised CRP and ESR suggests an autoimmune process. An extended autoimmune panel can help identify specific conditions like Sjogren's syndrome or lupus, which can present with neuropathy.<br>**5. Serum Immunofixation Electrophoresis (IFE)**<br>**Reasoning:**<br>● **Purpose:** To detect monoclonal proteins that may indicate a paraproteinemic neuropathy, such as multiple myeloma or MGUS (Monoclonal Gammopathy of Undetermined Significance).<br>● **Rationale:** Although initial serum electrophoresis was negative, IFE is more sensitive and can detect smaller amounts of monoclonal proteins.<br>**6. Lumbar Puncture and Cerebrospinal Fluid (CSF) Analysis**<br>**Reasoning:**<br>● **Purpose:** To evaluate the CSF for elevated protein levels, oligoclonal bands, and cells.<br>● **Rationale:** Elevated protein in the absence of pleocytosis can support a diagnosis of CIDP. CSF analysis can also rule out infectious or malignant causes of neuropathy.<br>**7. Magnetic Resonance Imaging (MRI) of the Lumbar Spine**<br>**Reasoning:**

			● **Purpose:** To identify any structural causes of neuropathy such as spinal stenosis or disc herniation, which might mimic or exacerbate neuropathic symptoms.<br>● **Rationale:** Although less likely given the symmetric nature of the neuropathy, ruling out compressive neuropathies is important, especially in older adults.<br>**8. Quantitative Sensory Testing (QST)**<br>**Reasoning:**<br>● **Purpose:** To objectively measure sensory nerve function.<br>● **Rationale:** QST can help quantify the degree of sensory loss and hyperalgesia, providing additional objective data to support clinical findings.<br>**Most Crucial Test**<br>**Nerve Biopsy**<br>**Reasoning:**<br>● **Comprehensive Diagnostic Information:** Nerve biopsy provides histopathological evidence, which can definitively distinguish between diabetic neuropathy, CIDP, vasculitic neuropathy, amyloid neuropathy, and other less common causes.<br>● **Specificity:** Given the overlap of clinical features among different types of neuropathies and the positive ANA suggesting a possible autoimmune process, nerve biopsy is likely to yield the most specific diagnostic information.<br>**Conclusion**<br>While several additional tests are important for a comprehensive evaluation, the nerve biopsy stands out as the most crucial test. It offers definitive diagnostic information that can accurately narrow down the differential diagnoses and guide appropriate management for this patient's polyneuropathy.
62	A 39-year-old male patient presents with a 1-month history of sensory-motor, symmetrical, non-length dependent polyneuropathy with acute onset. The patient presents with weakness, hypoesthesia, paresthesia and neuropathic pain in the four limbs, cramps, weakness of orbicularis oculi muscles, stypsis and urinary incontinence. Clinical examination reveals absence of tremor, ataxia or foot deformities. Past medical history is silent. Nerve conduction studies show a symmetric, demyelinating, sensory-motor polyneuropathy. Screening lab tests including vitamin B12, folate, vitamin B6, vitamin E, HCV, HBV, ANA, ANCA, ENA, CRP, ESR, glycemia, HbA1c, copper, cryoglobulins and serum electrophoresis are all negative. There is no family history of polyneuropathy.	GBS	**Step-by-Step Analysis and Additional Examinations**<br>**Step 1: Review of Clinical Scenario**<br>● **Key Findings**: Acute onset of sensory-motor, symmetrical, non-length dependent polyneuropathy with demyelination; associated with autonomic symptoms.<br>● **Initial Differential Diagnoses**: Guillain-Barré Syndrome (GBS), Chronic Inflammatory Demyelinating Polyneuropathy (CIDP), and Paraneoplastic Neuropathy.<br>**Step 2: Additional Examinations**<br>1. **Lumbar Puncture with Cerebrospinal Fluid (CSF) Analysis**<br>○ **Reasoning**:<br>■ In GBS, CSF analysis typically shows elevated protein levels with a normal white cell count (albuminocytologic dissociation).<br>■ This is a crucial test to differentiate GBS from other causes of acute polyneuropathy, such as infections or inflammatory disorders.<br>○ **Expected Findings**:<br>■ **GBS**: Elevated protein, normal cell count.<br>■ **CIDP**: May show elevated protein but not always as pronounced as in GBS.<br>■ **Paraneoplastic**: Variable findings, not specific.<br>2. **MRI of the Spine with Contrast**<br>○ **Reasoning**:<br>■ MRI can help visualize nerve root enhancement, which is often seen in GBS due to inflammation and demyelination.<br>■ Useful in differentiating CIDP (which might show similar but more chronic changes) and in identifying possible structural causes of neuropathy.<br>○ **Expected Findings**:<br>■ **GBS**: Nerve root enhancement.<br>■ **CIDP**: Possible nerve root thickening or enhancement.<br>■ **Paraneoplastic**: Could show nerve root involvement but less specific.<br>3. **Anti-Ganglioside Antibodies (e.g., Anti-GM1, Anti-GQ1b)**<br>○ **Reasoning**:<br>■ Certain variants of GBS, like Miller Fisher Syndrome, are associated with specific antibodies.<br>■ These antibodies can provide strong evidence supporting a diagnosis of GBS.<br>○ **Expected Findings**:<br>■ **GBS**: Presence of anti-ganglioside antibodies (e.g., Anti-GM1 in GBS, Anti-GQ1b in Miller Fisher variant).<br>■ **CIDP/Paraneoplastic**: Typically negative.<br>4. **CT Scan of the Chest, Abdomen, and Pelvis**<br>○ **Reasoning**:<br>■ This imaging helps in identifying any underlying malignancy, which could point towards a paraneoplastic neuropathy.<br>■ Particularly important if there are no other signs or symptoms suggesting cancer.<br>○ **Expected Findings**:<br>■ **Paraneoplastic**: Possible identification of a primary malignancy.<br>■ **GBS/CIDP**: Usually no findings relevant to polyneuropathy.<br>5. **Autoimmune Panel and Paraneoplastic Panel (e.g., Anti-Hu, Anti-Yo, Anti-Ri Antibodies)**<br>○ **Reasoning**:<br>■ Detecting autoantibodies associated with paraneoplastic syndromes can help confirm a diagnosis of paraneoplastic neuropathy.<br>■ These panels can identify underlying autoimmune processes that might not be evident with initial lab tests.<br>○ **Expected Findings**:<br>■ **Paraneoplastic**: Presence of specific paraneoplastic antibodies.<br>■ **GBS/CIDP**: Typically negative.<br>6. **Electromyography (EMG) and Repetitive Nerve Stimulation Tests**<br>○ **Reasoning**:<br>■ EMG can further characterize the extent and pattern of nerve involvement, and repetitive nerve stimulation can assess neuromuscular junction disorders.<br>■ Helps in differentiating between various types of neuropathies.<br>○ **Expected Findings**:<br>■ **GBS/CIDP**: Conduction block and demyelination.

			■ **Paraneoplastic**: Variable, depending on the underlying condition.<br>**Step 3: Determination of the Most Crucial Test**<br>The most crucial test from this list is **Lumbar Puncture with Cerebrospinal Fluid (CSF) Analysis**.<br>● **Reasoning**:<br>○ It is pivotal for diagnosing GBS, given the characteristic finding of albuminocytologic dissociation (elevated protein with normal cell count).<br>○ It provides immediate, critical diagnostic information that can strongly differentiate GBS from other causes of acute polyneuropathy.<br>○ GBS is a medical emergency that requires prompt treatment, making rapid and accurate diagnosis essential.<br>By performing a lumbar puncture and analyzing the CSF, the clinical team can confirm or rule out GBS and thus direct appropriate and timely management.
63	A 57-year-old female patient presents with a 1-month history of sensory-motor, symmetrical, length dependent polyneuropathy with subacute onset. The patient presents with severe sensory ataxia associated with hypoesthesia and paraesthesia in a stocking-glove distribution, as well as weakness of toe dorsiflexion. Clinical examination does not reveal cranial nerve or autonomic involvement, tremor, or foot deformities. Past medical history is positive for chronic autoimmune gastritis, chronic cough, type 2 diabetes, and arterial hypertension. Nerve conduction studies show presence of a sensory motor, length dependent, symmetric, axonal polyneuropathy. Screening lab tests reveal vitamin B12 deficiency, hyperglycaemia, elevated HbA1c, and positive ANA. Remaining exams including folate, vitamin B6, vitamin E, HBV, HCV, ANCA, ENA, CRP, ESR, copper, cryoglobulins, and serum electrophoresis are normal. There is no family history of polyneuropathy.	B12 deficiency neuropathy	**Step-by-Step Analysis and Additional Examinations**<br>**Step 1: Review Initial Findings**<br>● **History:** Type 2 diabetes, chronic autoimmune gastritis, chronic cough, arterial hypertension<br>● **Symptoms:** Sensory-motor, symmetrical, length-dependent polyneuropathy, severe sensory ataxia, hypoesthesia, paraesthesia, weakness of toe dorsiflexion<br>● **Physical Examination:** No cranial nerve, autonomic involvement, tremor, or foot deformities<br>● **Lab Results:** Vitamin B12 deficiency, hyperglycemia, elevated HbA1c, positive ANA, normal other tests<br>● **Nerve Conduction Studies:** Sensory-motor, length-dependent, symmetric, axonal polyneuropathy<br>**Step 2: Identify Additional Examinations Needed**<br>1. **HbA1c and Fasting Blood Glucose (Repeated)**<br>○ **Reasoning:** To confirm and evaluate the current glycemic control status. Poorly controlled diabetes is a major cause of polyneuropathy.<br>2. **Serum Methylmalonic Acid and Homocysteine**<br>○ **Reasoning:** These tests are more sensitive indicators of vitamin B12 deficiency. Elevated levels would confirm the deficiency and its role in neuropathy.<br>3. **Anti-Parietal Cell and Intrinsic Factor Antibodies**<br>○ **Reasoning:** Given the patient's history of autoimmune gastritis, these antibodies can confirm pernicious anemia as the cause of vitamin B12 deficiency.<br>4. **Oral Glucose Tolerance Test (OGTT)**<br>○ **Reasoning:** To assess glucose metabolism more comprehensively, particularly if diabetes diagnosis is uncertain or to evaluate the extent of glucose intolerance.<br>5. **Quantitative Sensory Testing (QST)**<br>○ **Reasoning:** To assess the extent and severity of sensory nerve damage and to differentiate between small fiber and large fiber neuropathy.<br>6. **Skin Biopsy**<br>○ **Reasoning:** To evaluate intraepidermal nerve fiber density, which can provide evidence for small fiber neuropathy often missed in nerve conduction studies.<br>7. **Lumbar Puncture and Cerebrospinal Fluid (CSF) Analysis**<br>○ **Reasoning:** To rule out inflammatory or infectious causes of neuropathy, especially if there's suspicion of autoimmune neuropathy.<br>8. **Electromyography (EMG)**<br>○ **Reasoning:** To further characterize the neuropathy by evaluating muscle response and identifying any additional neurogenic changes.<br>9. **Autoimmune Panel (Expanded)**<br>○ **Reasoning:** To assess for other autoimmune conditions that might be contributing to the neuropathy, including additional specific antibodies (e.g., anti-Ro, anti-La, anti-dsDNA).<br>10. **Serum Protein Electrophoresis (SPEP) and Immunofixation**<br>○ **Reasoning:** To rule out monoclonal gammopathy, which can be associated with neuropathies like POEMS syndrome or multiple myeloma.<br>**Most Crucial Test**<br>**Serum Methylmalonic Acid and Homocysteine Levels**<br>**Reasoning:**<br>● These tests are crucial for confirming the functional deficiency of vitamin B12, which is directly implicated in neuropathy. While the initial lab results showed vitamin B12 deficiency, the confirmation through methylmalonic acid and homocysteine levels will establish the deficiency's severity and direct connection to the neuropathy.<br>● Proper identification and treatment of vitamin B12 deficiency can significantly impact the patient's neuropathy prognosis, especially given the subacute onset and severe symptoms.<br>● Additionally, addressing vitamin B12 deficiency may provide immediate symptomatic relief and prevent further neurological damage, making it an essential step in the patient's management.<br>By systematically evaluating these aspects, we ensure a thorough approach to diagnosing and managing the patient's peripheral neuropathy.
64	A 74-year-old male patient presents with a 1-year history of sensory-motor, symmetrical, length dependent polyneuropathy with subacute onset and slow-progressive course. The patient presents with paresthesia and neuropathic pain distally in the lower limbs and sensory ataxia. Clinical examination reveals the presence of mild hand tremor and weakness of toe dorsiflexion. There are no foot deformities, cranial nerve or autonomic involvement. Past medical history is positive for cardiac insufficiency, diabetes and IgM monoclonal gammopathy. Nerve conduction studies show a demyelinating, sensory-motor, symmetric polyneuropathy. Screening lab tests reveal the presence of IgM	Anti-MAG polyneuropathy	**Step-by-Step Analysis**<br>**Additional Examinations Needed**<br>1. **Serum Immunofixation Electrophoresis (IFE) and Immunoglobulin Levels**<br>○ **Reasoning**: While the presence of IgM paraprotein suggests a monoclonal gammopathy, it is crucial to characterize the monoclonal protein further and quantify immunoglobulin levels. This helps in identifying the subtype of MGUS or other possible plasma cell disorders.<br>2. **Anti-MAG Antibodies**<br>○ **Reasoning**: Myelin-associated glycoprotein (MAG) antibodies are often present in patients with IgM MGUS-related neuropathy. Testing for these antibodies can confirm the involvement of anti-MAG neuropathy, which is commonly associated with a demyelinating pattern.<br>3. **Lumbar Puncture with Cerebrospinal Fluid (CSF) Analysis**<br>○ **Reasoning**: CSF analysis can help differentiate between CIDP and MGUS-related neuropathy. Elevated CSF protein without pleocytosis is characteristic of CIDP. In MGUS-related neuropathy, CSF protein might be elevated, but the findings are not as specific as in CIDP.<br>4. **Skin Biopsy for Intraepidermal Nerve Fiber Density**<br>○ **Reasoning**: A skin biopsy can assess small fiber neuropathy, which might not be apparent on nerve conduction studies. This can help in assessing the extent of neuropathy and confirming the sensory involvement.<br>5. **MRI of the Spine with Contrast**<br>○ **Reasoning**: MRI can help identify any structural abnormalities or inflammation in the spinal cord and nerve roots, which might be contributing to the neuropathy. It is particularly useful in ruling out CIDP-related root hypertrophy or enhancement.<br>6. **Quantitative Sensory Testing (QST)**<br>○ **Reasoning**: QST evaluates the sensory thresholds and can provide quantitative data on sensory deficits. This is useful in understanding the extent and distribution of sensory involvement.

	paraprotein and mildly elevated HbA1c levels. Remaining lab tests including vitamin B12, folate, vitamin B6, vitamin E, HCV, HBV, ANA, ANCA, ENA, CRP, ESR, glycemia, copper, and cryoglobulins are negative. There is no family history of polyneuropathy.		7. **Electromyography (EMG) and Repeat Nerve Conduction Studies (NCS)**<br>○ **Reasoning**: Repeat NCS and EMG can help in monitoring the progression of the neuropathy and further characterizing the demyelinating versus axonal components. It provides dynamic information about the nerve function over time.<br>8. **Autoimmune Panel for Paraneoplastic Syndromes**<br>○ **Reasoning**: Since monoclonal gammopathies can be associated with paraneoplastic syndromes, an autoimmune panel can help identify antibodies related to paraneoplastic neurological syndromes (e.g., anti-Hu, anti-Ri antibodies).<br>9. **PET/CT Scan**<br>○ **Reasoning**: To rule out underlying malignancies, especially lymphoproliferative disorders, which might be associated with monoclonal gammopathy and can present with neuropathy. This imaging can help in staging and identifying any occult neoplasms.<br>10. **Genetic Testing**<br>○ **Reasoning**: Although there is no family history of polyneuropathy, genetic testing for hereditary neuropathies (e.g., Charcot-Marie-Tooth disease) can be considered to rule out late-onset genetic causes of demyelinating neuropathy.<br>**Most Crucial Test**<br>**Anti-MAG Antibodies**<br>● This test is crucial because the presence of anti-MAG antibodies would strongly support a diagnosis of MGUS-related neuropathy, specifically anti-MAG neuropathy. This condition is associated with IgM monoclonal gammopathy and presents with a demyelinating pattern on nerve conduction studies, fitting the patient's clinical presentation. Identifying anti-MAG antibodies would provide a definitive diagnostic marker and guide specific therapeutic interventions.
65	An 83-year-old female patient presents with a 6-week history of pure sensory, symmetrical, length dependent polyneuropathy with subacute onset. The patient presents with distal paresthesia, hypoesthesia, and neuropathic pain in the lower limbs with associated mild sensory ataxia. Clinical examination does not show the presence of weakness, tremor, foot deformities, cranial nerve or autonomic involvement. Romberg test resulted positive with inability to maintain postural station, feeling of instability, and inability to walk with closed eyes. Past medical history is positive for leukopenia, undifferentiated connective tissue disease, and type 2 diabetes. Nerve conduction studies show a pure sensory, symmetric, length dependent, axonal polyneuropathy. Screening lab tests show high glycemia and raised HbA1c, positivity for ANA antibodies, and evidence of vitamin B12 deficiency. Remaining exams including folate, vitamin B6, vitamin E, HCV, HBV, ANCA, CRP, ESR, copper, cryoglobulins, and serum electrophoresis are all negative. There is no family history of polyneuropathy.	Vit B12 deficiency neuropathy	**Step-by-Step Analysis and Additional Examinations**<br>1. **Review of Clinical Data:**<br>1. **Patient Profile:** 83-year-old female with 6-week history of pure sensory, symmetrical, length-dependent polyneuropathy.<br>2. **Symptoms:** Distal paresthesia, hypoesthesia, neuropathic pain, mild sensory ataxia, positive Romberg test.<br>3. **History:** Leukopenia, undifferentiated connective tissue disease, type 2 diabetes.<br>4. **Investigations:** High glycemia, raised HbA1c, positive ANA antibodies, vitamin B12 deficiency. Negative tests include folate, vitamin B6, vitamin E, HCV, HBV, ANCA, CRP, ESR, copper, cryoglobulins, serum electrophoresis.<br>2. **Proposed Additional Examinations:**<br>**A. Laboratory Tests**<br>1. **Anti-GAD Antibodies:**<br>■ **Reasoning:** Anti-glutamic acid decarboxylase (GAD) antibodies are associated with autoimmune diabetes and can be seen in some cases of diabetic neuropathy. Given the patient's type 2 diabetes and autoimmune background, checking for these antibodies can help determine if there is an autoimmune component to the neuropathy.<br>2. **Anti-Hu, Anti-Ri, Anti-Yo Antibodies:**<br>■ **Reasoning:** These paraneoplastic antibodies are associated with paraneoplastic syndromes that can present with neuropathy. Considering the patient's age and recent onset of symptoms, it's prudent to rule out a paraneoplastic etiology.<br>3. **Serum Protein Electrophoresis with Immunofixation:**<br>■ **Reasoning:** Although initial serum electrophoresis was negative, immunofixation can detect monoclonal gammopathies more sensitively, which can be associated with neuropathies (e.g., MGUS, multiple myeloma).<br>4. **HbA1c Repeat Test:**<br>■ **Reasoning:** Confirming the level of glycemic control is crucial, especially since the patient has type 2 diabetes. It helps correlate neuropathy severity with glycemic control.<br>5. **Serum MMA and Homocysteine Levels:**<br>■ **Reasoning:** These markers can help confirm the severity and functional impact of vitamin B12 deficiency.<br>6. **TSH and Free T4:**<br>■ **Reasoning:** Hypothyroidism can cause polyneuropathy. Screening for thyroid function ensures another potential cause is ruled out.<br>3. **B. Instrumental Tests**<br>1. **MRI of the Spine:**<br>■ **Reasoning:** To rule out compressive myelopathy or other spinal pathologies that might contribute to sensory symptoms and ataxia. MRI can visualize spinal cord and root involvement.<br>2. **Lumbar Puncture with CSF Analysis:**<br>■ **Reasoning:** CSF analysis can help rule out inflammatory, infectious, or paraneoplastic processes. Elevated protein levels can indicate chronic inflammatory demyelinating polyneuropathy (CIDP) or other neuropathies.<br>3. **Skin Biopsy:**<br>■ **Reasoning:** To assess small fiber neuropathy, which might not be detected in nerve conduction studies. It can provide information about the density of intraepidermal nerve fibers.<br>4. **Most Crucial Test:**<br>**Serum Protein Electrophoresis with Immunofixation**<br>**Reasoning:** This test is highly sensitive for detecting monoclonal gammopathies that are often associated with neuropathies. Given the patient's age and the subacute onset of symptoms, it is important to rule out a paraneoplastic or monoclonal gammopathy of undetermined significance (MGUS) as a potential cause. Identifying or excluding a monoclonal protein can significantly narrow down the differential diagnoses and guide further management.<br>**Conclusion**<br>While several tests are necessary to comprehensively evaluate the potential causes of the patient's polyneuropathy, serum protein electrophoresis with immunofixation stands out as the most crucial. This test is vital in identifying or ruling out monoclonal gammopathies, which are a treatable cause of neuropathy and important to consider in elderly patients with new-onset neuropathy.
66	A 71-year-old female patient presents with a 4-month history of pure sensory, symmetrical, length dependent polyneuropathy with subacute onset. The	Chemotherapy-induced neuropathy	**Step-by-Step Analysis and Additional Examinations**<br>**1. Comprehensive Clinical Data Recap**<br>● **Patient Demographics**: 71-year-old female<br>● **Symptoms**: 4-month history of pure sensory, symmetrical, length-dependent polyneuropathy with subacute onset. Neuropathic pain and paresthesia in the feet, hyperalgesia, and allodynia.

	patient presents with neuropathic pain and paresthesia in the feet. She does not report weakness, ataxia, tremor, cranial nerve or autonomic involvement. Clinical examination shows hyperalgesia and allodynia in the feet. There are no foot deformities, sensory ataxia or weakness. Past medical history is positive for cervical cancer, for which the patient started chemotherapy 6 months ago. Nerve conduction studies show a sensory-motor, symmetric, length dependent polyneuropathy. Screening lab tests show raised CRP and ESR, and positivity for ANA antibodies. Remaining exams including vitamin B12, folate, vitamin B6, vitamin E, HBV, HCV, ANCA, ENA, glycemia, HbA1c, copper, cryoglobulins, and serum electrophoresis are negative. There is no family history of polyneuropathy.		● **Past Medical History**: Cervical cancer, chemotherapy started 6 months ago.<br>● **Physical Examination**: No weakness, ataxia, tremor, cranial nerve, or autonomic involvement.<br>● **Nerve Conduction Studies**: Sensory-motor, symmetric, length-dependent polyneuropathy.<br>● **Lab Results**: Raised CRP and ESR, positive ANA antibodies. Negative for vitamin B12, folate, vitamin B6, vitamin E, HBV, HCV, ANCA, ENA, glycemia, HbA1c, copper, cryoglobulins, and serum electrophoresis.<br>● **Family History**: No family history of polyneuropathy.<br>**2. Additional Examinations**<br>1. **Serum Immunofixation Electrophoresis (IFE)**<br>○ **Reasoning**: To rule out monoclonal gammopathies such as multiple myeloma or MGUS, which can cause neuropathy.<br>○ **Relevance**: Despite negative serum electrophoresis, IFE is more sensitive and can detect smaller amounts of monoclonal protein.<br>2. **Anti-Hu and Anti-Yo Antibodies (Paraneoplastic Panel)**<br>○ **Reasoning**: To evaluate for paraneoplastic neuropathy, given the history of cancer.<br>○ **Relevance**: Identifying paraneoplastic antibodies could confirm an immune-mediated neuropathy related to the malignancy.<br>3. **Lumbar Puncture with CSF Analysis**<br>○ **Reasoning**: To examine cerebrospinal fluid (CSF) for elevated protein levels, which can indicate inflammatory or demyelinating neuropathy.<br>○ **Relevance**: Helpful in distinguishing between different types of neuropathy, particularly if there is suspicion of Guillain-Barré syndrome or chronic inflammatory demyelinating polyneuropathy (CIDP).<br>4. **MRI of the Spine with Gadolinium**<br>○ **Reasoning**: To assess for any structural lesions, including metastases, that might be causing neuropathy.<br>○ **Relevance**: Given the patient's history of cervical cancer, this can rule out spinal involvement or compressive neuropathy.<br>5. **Skin Biopsy for Intraepidermal Nerve Fiber Density (IENFD)**<br>○ **Reasoning**: To assess small fiber neuropathy directly.<br>○ **Relevance**: Particularly useful if large fiber studies (NCS/EMG) are normal or inconclusive.<br>6. **Quantitative Sensory Testing (QST)**<br>○ **Reasoning**: To objectively measure sensory thresholds and quantify sensory loss.<br>○ **Relevance**: Can provide detailed information on the extent of sensory neuropathy and correlate with clinical symptoms.<br>7. **Additional Autoimmune Screening (e.g., anti-Ro/SSA, anti-La/SSB)**<br>○ **Reasoning**: To explore other autoimmune conditions such as Sjögren's syndrome.<br>○ **Relevance**: Positive ANA suggests possible autoimmune etiology, and further testing can identify specific conditions.<br>8. **Glucose Tolerance Test**<br>○ **Reasoning**: To rule out impaired glucose tolerance or prediabetes, which can cause neuropathy even when HbA1c is normal.<br>○ **Relevance**: Diabetes and prediabetes are common causes of neuropathy, and an oral glucose tolerance test can uncover hidden glucose intolerance.<br>9. **Re-evaluation of Current Chemotherapy Regimen**<br>○ **Reasoning**: To assess the neurotoxic potential of the chemotherapy agents being used.<br>○ **Relevance**: Identifying specific agents responsible for neurotoxicity can guide treatment adjustments.<br>**3. Determination of the Most Crucial Test**<br>**Most Crucial Test**: **Serum Immunofixation Electrophoresis (IFE)**<br>● **Reasoning**: While other tests are important, IFE is the most crucial as it can detect monoclonal gammopathies that might have been missed by standard electrophoresis. Monoclonal gammopathies are a significant cause of neuropathy in elderly patients, and identifying them can significantly alter the management and prognosis. Given the patient's age and presentation, ruling out such conditions is paramount.<br>**Conclusion**<br>A thorough analysis should start with Serum Immunofixation Electrophoresis to rule out monoclonal gammopathies. Concurrently, testing for paraneoplastic antibodies, performing a lumbar puncture, and evaluating the chemotherapy regimen are also essential steps in narrowing down the differential diagnoses.
67	An 83-year-old male patient presents with a 1-year history of sensory-motor, symmetrical, length dependent polyneuropathy with insidious onset and progressive course. The patient presents with distal leg paresthesia, hypoesthesia, and sensory ataxia. Clinical examination confirms the presence of sensory ataxia with positive Romberg test and inability to walk with closed eyes. There is also postural hand tremor, which appeared concurrently with the other symptoms. Clinical examination does not show the presence of foot deformities, cranial nerve or autonomic involvement. Past medical history is positive for prostatic cancer, for which the patient underwent chemotherapy four years ago. Nerve conduction studies show a demyelinating, sensory-motor, length dependent polyneuropathy. Screening lab tests reveal the presence of IgM paraprotein and positivity for ANA antibodies. Remaining	Anti-MAG polyneuropathy	**Step-by-Step Analysis**<br>**Comprehensive Data Recap:**<br>● **Patient Details:** 83-year-old male<br>● **History:** Prostatic cancer treated with chemotherapy 4 years ago<br>● **Symptoms:** 1-year history of sensory-motor, symmetrical, length-dependent polyneuropathy with distal leg paresthesia, hypoesthesia, sensory ataxia, positive Romberg test, postural hand tremor<br>● **Clinical Examination:** Sensory ataxia, positive Romberg test, no foot deformities, no cranial nerve or autonomic involvement<br>● **Nerve Conduction Studies:** Demyelinating, sensory-motor, length-dependent polyneuropathy<br>● **Lab Results:** Presence of IgM paraprotein and positive ANA antibodies; negative for vitamin B12, folate, vitamin B6, vitamin E, HCV, HBV, ANCA, ENA, CRP, ESR, glycemia, HbA1c, copper, and cryoglobulins<br>● **Family History:** No family history of polyneuropathy<br>**Additional Examinations**<br>1. **Serum and Urine Protein Electrophoresis with Immunofixation**<br>○ **Reasoning:** To further characterize the IgM paraprotein and identify any monoclonal gammopathy. This test will help determine if the paraprotein is related to MGUS, multiple myeloma, or another plasma cell dyscrasia, which could be contributing to the neuropathy.<br>2. **Lumbar Puncture with Cerebrospinal Fluid (CSF) Analysis**<br>○ **Reasoning:** To assess for elevated CSF protein levels without pleocytosis, which is typical in CIDP. It can also help rule out infectious or other inflammatory causes of neuropathy.<br>3. **Anti-MAG Antibodies Testing**<br>○ **Reasoning:** The presence of IgM paraprotein raises the possibility of an IgM MGUS with anti-myelin-associated glycoprotein (MAG) antibodies, which is associated with a demyelinating neuropathy.<br>4. **MRI of the Spine with Contrast**<br>○ **Reasoning:** To evaluate for any structural abnormalities, inflammation, or enhancement of the nerve roots, which can support the diagnosis of CIDP.<br>5. **Repeat ANA Profile and Extended Autoimmune Panel**<br>○ **Reasoning:** To identify any additional autoantibodies that may indicate an autoimmune connective tissue disease or other autoimmune process that could contribute to the neuropathy.<br>6. **PET/CT Scan**<br>○ **Reasoning:** Given the history of prostatic cancer, this imaging can help rule out any current malignancy or metastasis, which could be contributing to a paraneoplastic syndrome.

	exams including vitamin B12, folate, vitamin B6, vitamin E, HCV, HBV, ANCA, ENA, CRP, ESR, glycemia, HbA1c, copper ,and cryoglobulins are negative. There is no family history of polyneuropathy.		7. **Quantitative Sensory Testing (QST)**<br>○ **Reasoning:** To objectively measure sensory nerve function and quantify the degree of sensory loss, which can help in monitoring disease progression and response to treatment.<br>8. **Nerve Biopsy**<br>○ **Reasoning:** Although invasive and typically reserved for unclear cases, a nerve biopsy can provide direct evidence of demyelination, axonal loss, inflammation, or vasculitis, aiding in definitive diagnosis.<br>**Most Crucial Test**<br>**Serum and Urine Protein Electrophoresis with Immunofixation**<br>**Reasoning:** This test is crucial because it will provide detailed information about the IgM paraprotein, which is a key finding in this patient. Understanding whether this paraprotein is monoclonal or polyclonal, and if it is associated with conditions like MGUS, Waldenström's macroglobulinemia, or multiple myeloma, is essential. This test will help narrow down the differential diagnoses significantly by either confirming or ruling out a monoclonal gammopathy as the underlying cause of the neuropathy.
68	A 46-year-old male patient presents with a 3-year history of sensory-motor, symmetrical, non-length dependent polyneuropathy with insidious onset. The patient presents with leg paresthesia, sensory ataxia, proximal and distal weakness in the four limbs, and cramps. There are no cranial nerve or autonomic involvement, tremor, neuropathic pain or foot deformities. Past medical history shows IgG MGUS and chronic renal insufficiency. Nerve conduction studies show demyelinating, sensory-motor polyneuropathy in the four limbs. Screening lab tests including vitamin B12, folate, vitamin B6, vitamin E, HCV, HBV, ANA, ANCA, ENA, CRP, ESR, glycemia, HbA1c, copper and cryoglobulins are all negative. Serum electrophoresis confirms the presence of IgG monoclonal gammopathy. There is no family history of polyneuropathy.	CIDP	**Step-by-Step Analysis of Additional Examinations**<br>To narrow down the differential diagnoses for this patient, additional laboratory and instrumental examinations are necessary. Here is a step-by-step approach, listing each test with detailed reasoning:<br>**Step 1: Laboratory Tests**<br>1. **Serum Protein Electrophoresis with Immunofixation:**<br>○ **Reasoning:** While serum electrophoresis has confirmed IgG monoclonal gammopathy, immunofixation provides more precise identification and quantification of monoclonal protein. This helps in assessing the extent and nature of the MGUS and ruling out multiple myeloma or other plasma cell dyscrasias.<br>2. **Urine Protein Electrophoresis:**<br>○ **Reasoning:** To detect Bence Jones proteins, which are free light chains in the urine. This is important in ruling out multiple myeloma or light chain deposition disease, which could be contributing to the neuropathy.<br>3. **Anti-MAG (Myelin-Associated Glycoprotein) Antibodies:**<br>○ **Reasoning:** High titers of anti-MAG antibodies are associated with a specific type of demyelinating neuropathy seen in patients with IgM MGUS. Although this patient has IgG MGUS, it is prudent to rule out anti-MAG-associated neuropathy due to overlapping clinical features.<br>4. **Serum Free Light Chain Assay:**<br>○ **Reasoning:** To evaluate the ratio of kappa to lambda light chains, which can provide additional information about monoclonal gammopathy and potential progression to more severe plasma cell disorders.<br>5. **Paraneoplastic Panel (including anti-Hu, anti-Ri, anti-Yo antibodies):**<br>○ **Reasoning:** Paraneoplastic syndromes can cause neuropathy and may be associated with monoclonal gammopathies. Checking for these antibodies helps in ruling out malignancy-associated neuropathies.<br>6. **Repeat Renal Function Tests and Electrolyte Panel:**<br>○ **Reasoning:** Chronic renal insufficiency can fluctuate and further decline may exacerbate neuropathic symptoms. Electrolyte imbalances can also contribute to or mimic neuropathy.<br>**Step 2: Instrumental Examinations**<br>1. **Lumbar Puncture with Cerebrospinal Fluid (CSF) Analysis:**<br>○ **Reasoning:** To evaluate for elevated protein levels without pleocytosis, which is characteristic of CIDP. This helps in differentiating CIDP from other forms of demyelinating neuropathy.<br>2. **Nerve Biopsy:**<br>○ **Reasoning:** Particularly useful in diagnosing atypical demyelinating neuropathies or amyloidosis. It provides direct histopathological evidence of nerve pathology.<br>3. **MRI of the Spine and Brain:**<br>○ **Reasoning:** To rule out central nervous system involvement and structural causes of neuropathy. It can also help visualize nerve root and plexus involvement typical of CIDP.<br>4. **Electromyography (EMG):**<br>○ **Reasoning:** To complement nerve conduction studies and provide further detail on the type and extent of neuromuscular involvement.<br>5. **Bone Marrow Biopsy:**<br>○ **Reasoning:** Indicated if there is suspicion of progression from MGUS to multiple myeloma or other hematological malignancies based on the results of serum and urine electrophoresis.<br>**Conclusion: Most Crucial Test**<br>**Serum Protein Electrophoresis with Immunofixation** is the most crucial test from this list. This test is essential to provide a precise identification and quantification of the monoclonal protein present. It will help determine if the patient's MGUS has progressed to a more serious condition such as multiple myeloma, which would significantly alter the management and prognosis of the patient's neuropathy.
69	A 78-year-old female patient presents with a 2-year history of sensory-motor, symmetrical, length dependent polyneuropathy with subacute onset. Clinical examination reveals presence of numbness, tingling, and neuropathic pain distally in the upper and lower limbs, weakness of ankle dorsiflexion, sensory ataxia, hand tremor and dysphonia, without autonomic involvement or foot deformities. Past medical history reveals diabetes and IgM kappa monoclonal gammopathy. Nerve conduction studies show a length dependent, symmetric, sensory-motor, demyelinating polyneuropathy. Screening lab tests including vitamin B12, folate, vitamin B6, vitamin E, HCV, HBV, ANA, ANCA, ENA, CRP, ESR, glycemia, copper and cryoglobulins are all negative, with positivity for IgM kappa paraprotein and raised HbA1c. There is no family history of polyneuropathy.	Anti-MAG polyneuropathy	**Step-by-Step Analysis and Additional Examinations**<br>**Clinical Summary Recap:**<br>● **Patient:** 78-year-old female<br>● **History:** 2-year sensory-motor, symmetrical, length-dependent polyneuropathy with subacute onset<br>● **Examination Findings:** Numbness, tingling, neuropathic pain, ankle dorsiflexion weakness, sensory ataxia, hand tremor, dysphonia, no autonomic involvement or foot deformities<br>● **Medical History:** Diabetes, IgM kappa monoclonal gammopathy<br>● **Nerve Conduction Studies:** Symmetric, sensory-motor, demyelinating polyneuropathy<br>● **Screening Lab Tests:** All negative except for IgM kappa paraprotein and raised HbA1c<br>● **Family History:** No polyneuropathy<br>**Additional Examinations and Reasoning**<br>1. **Serum Immunofixation Electrophoresis (IFE)**<br>○ **Reasoning:** To further characterize the monoclonal gammopathy. This test can confirm the presence of monoclonal IgM kappa and help differentiate between MGUS and more serious conditions like multiple myeloma or Waldenström's macroglobulinemia.<br>○ **Relevance:** Essential for determining the significance of the IgM kappa paraprotein.<br>2. **Lumbar Puncture with Cerebrospinal Fluid (CSF) Analysis**<br>○ **Reasoning:** To check for elevated protein levels without pleocytosis, which is indicative of CIDP. This can also rule out infectious or inflammatory causes.<br>○ **Relevance:** Helps confirm or exclude CIDP.<br>3. **Skin Biopsy with Intraepidermal Nerve Fiber Density (IENFD)**<br>○ **Reasoning:** To evaluate small fiber neuropathy, which may not be detected by nerve conduction studies.<br>○ **Relevance:** Can provide additional diagnostic information about the nature of the neuropathy.<br>4. **Quantitative Sensory Testing (QST)**<br>○ **Reasoning:** To assess the function of small and large nerve fibers through evaluation of thermal and vibratory sensation thresholds.<br>○ **Relevance:** Complements nerve conduction studies by providing functional data on sensory fibers.

			5. **Autonomic Testing (e.g., Tilt Table Test, Heart Rate Variability)**<br>○ **Reasoning:** Although the patient shows no autonomic symptoms, subtle autonomic dysfunction might be present and can help differentiate between neuropathy types.<br>○ **Relevance:** Confirms or excludes autonomic involvement.<br>6. **MRI of the Brain and Spine**<br>○ **Reasoning:** To rule out central nervous system causes of dysphonia and other neurologic symptoms.<br>○ **Relevance:** Ensures that symptoms are not due to central lesions or compressive neuropathies.<br>7. **Repeat HbA1c and Oral Glucose Tolerance Test (OGTT)**<br>○ **Reasoning:** To reassess and confirm the control of diabetes, as poor glycemic control can exacerbate neuropathy.<br>○ **Relevance:** Ensures comprehensive management of diabetic neuropathy if present.<br>8. **Serum Free Light Chain Assay**<br>○ **Reasoning:** To detect light chain deposition disease or amyloidosis, which can present with neuropathy.<br>○ **Relevance:** Helps assess the monoclonal gammopathy's impact on neuropathy.<br>**Most Crucial Test**<br>**Serum Immunofixation Electrophoresis (IFE)**<br>● **Reasoning:** This test is crucial because it will help to confirm and further characterize the monoclonal IgM kappa protein. Understanding the nature of the monoclonal gammopathy is essential to differentiate MGUS from more serious hematologic conditions, such as multiple myeloma or Waldenström's macroglobulinemia, which require different management strategies and have different prognoses.<br>● **Impact:** Confirming MGUS would focus the treatment on managing the neuropathy, whereas finding a more malignant process would significantly change the therapeutic approach.<br>**Conclusion**<br>Performing Serum Immunofixation Electrophoresis (IFE) is the most crucial test in this clinical scenario. It will provide vital information about the monoclonal gammopathy, helping to narrow down the differential diagnosis and guiding further management.
70	A 51-year-old female patient presents with a five-month history of sensory-motor, asymmetrical, non-length-dependent polyneuropathy with subacute onset and progressive course. The patient reports numbness and tingling in a stocking and glove distribution, dysphagia, asymmetric proximal and distal weakness in all four limbs, and mild sensory ataxia. Clinical examination confirms the presence of weakness and sensory deficits in all four limbs, with no evidence of autonomic involvement, tremor, or foot deformities. Past medical history is unremarkable. Nerve conduction studies show a demyelinating, non-length-dependent, sensory-motor polyneuropathy. Screening lab tests including vitamin B12, folate, vitamin B6, vitamin E, HBV, HCV, ANA, ANCA, ENA, CRP, ESR, glycemia, HbA1c, copper, cryoglobulins, and serum electrophoresis are all negative. There is no family history of polyneuropathy.	CIDP	**Step-by-Step Analysis and Additional Examinations**<br>1. **Review Clinical History and Initial Presentation**<br>○ 52-year-old male<br>○ 6-month history of progressive numbness, tingling, burning in feet, extending to hands<br>○ Muscle weakness in lower extremities<br>○ Medical history: Type 2 diabetes mellitus, hypertension, hyperlipidemia<br>○ Medications: Metformin, lisinopril, atorvastatin<br>○ No smoking, occasional alcohol use<br>○ Physical exam: Decreased sensation in stocking-glove distribution, diminished ankle reflexes, mild distal muscle weakness<br>○ Laboratory tests: Elevated fasting blood glucose, HbA1c of 8.5%, normal renal function, no vitamin deficiencies<br>○ Nerve conduction studies: Length-dependent axonal polyneuropathy<br>2. **Additional Examinations**<br>**a. Serum Electrophoresis and Immunofixation Electrophoresis (IFE)**<br>○ **Reasoning**: To rule out monoclonal gammopathies such as multiple myeloma or amyloidosis, which can cause peripheral neuropathy. The presence of monoclonal proteins can be indicative of these conditions.<br>3. **b. Autoantibody Panel (ANA, ANCA, Anti-Ro, Anti-La, Anti-dsDNA)**<br>○ **Reasoning**: To evaluate for autoimmune diseases such as lupus, Sjögren's syndrome, and vasculitis, which can present with peripheral neuropathy.<br>4. **c. Thyroid Function Tests (TSH, Free T4)**<br>○ **Reasoning**: Hypothyroidism can present with peripheral neuropathy. Evaluating thyroid function is essential to rule out this potential cause.<br>5. **d. Vitamin B12 and Folate Levels (with MMA and Homocysteine if B12 is low-normal)**<br>○ **Reasoning**: Vitamin B12 deficiency is a common cause of peripheral neuropathy. Despite initial tests showing no deficiencies, confirmatory tests with MMA and homocysteine levels are necessary if B12 levels are borderline.<br>6. **e. Lumbar Puncture (CSF Analysis)**<br>○ **Reasoning**: To check for elevated protein levels or presence of inflammatory cells, which can indicate CIDP or other inflammatory neuropathies.<br>7. **f. Electromyography (EMG)**<br>○ **Reasoning**: To assess the electrical activity of muscles and further characterize the type and extent of neuropathy, distinguishing between axonal and demyelinating processes.<br>8. **g. Liver Function Tests (LFTs) and Hepatitis Panel**<br>○ **Reasoning**: Chronic liver disease and hepatitis (especially hepatitis C) can cause peripheral neuropathy. Screening for liver dysfunction and viral hepatitis is warranted.<br>9. **h. Heavy Metal Screening (Lead, Arsenic, Mercury)**<br>○ **Reasoning**: Exposure to heavy metals can lead to peripheral neuropathy. This test is important if there's any suspicion of environmental or occupational exposure.<br>10. **i. Genetic Testing**<br>○ **Reasoning**: If there is a family history of neuropathy, genetic testing can be considered for inherited neuropathies like Charcot-Marie-Tooth disease.<br>11. **Most Crucial Test**<br>**Serum Electrophoresis and Immunofixation Electrophoresis (IFE)**<br>○ **Reasoning**: This test is critical because it helps to identify monoclonal gammopathies, which are serious and treatable causes of peripheral neuropathy. Given the age of the patient and the subacute progression of symptoms, ruling out conditions like multiple myeloma or amyloidosis is essential. These conditions require early diagnosis and specific treatment strategies to prevent further complications.<br>By conducting these additional examinations, we can systematically exclude other potential causes of peripheral neuropathy and further substantiate the diagnosis of Diabetic Peripheral Neuropathy if no other underlying condition is identified.
71	A 69-year-old male patient presents with a 7-month history of sensory-motor, asymmetrical, non-length dependent polyneuropathy with subacute onset and progressive course. The patient presents	CIDP	**Step-by-Step Approach to Additional Examinations**<br>**Step 1: Confirmatory and Supporting Laboratory Tests**<br>1. **HbA1c and Fasting Blood Glucose**<br>○ **Reasoning**: To further confirm the level of glycemic control, as the patient has type 2 diabetes with high glycemia and HbA1c levels. Poor glycemic control can exacerbate diabetic neuropathy.<br>2. **Oral Glucose Tolerance Test (OGTT)**

	with paresthesia and neuropathic pain in the four limbs, weakness of ankle dorsiflexion, and mild urinary retention. Clinical examination does not show the presence of tremor, ataxia, foot deformities or cranial nerve involvement. Past medical history is positive for type 2 diabetes. Nerve conduction studies show a demyelinating, sensory-motor, symmetric, polyneuropathy. Screening lab tests including vitamin B12, folate, vitamin B6, vitamin E, HBV, HCV, ANA, ANCA, ENA, CRP, ESR, copper, cryoglobulins and serum electrophoresis are all negative, while glycemia and HbA1c levels are high. There is no family history of polyneuropathy.		○ **Reasoning**: To assess for any postprandial spikes in blood sugar that may not be captured by fasting glucose levels. This can provide a more comprehensive picture of glucose metabolism.<br>3. **Autoimmune Panel (if not already performed)**<br>○ **Reasoning**: To rule out autoimmune causes of neuropathy, such as CIDP. Testing for specific antibodies (e.g., anti-GM1, anti-MAG) can help in this differentiation.<br>4. **CSF Analysis**<br>○ **Reasoning**: To check for elevated protein levels without pleocytosis, which is characteristic of CIDP. This can help distinguish CIDP from other neuropathies.<br>**Step 2: Imaging and Electrophysiological Studies**<br>1. **MRI of the Spine and Brain**<br>○ **Reasoning**: To rule out structural causes of neuropathy, such as spinal cord compression or lesions, which can mimic polyneuropathy symptoms. This can also identify any central nervous system involvement.<br>2. **Repeat Nerve Conduction Studies and Electromyography (EMG)**<br>○ **Reasoning**: To evaluate the progression of the neuropathy and further characterize its nature (demyelinating vs. axonal). EMG can provide additional information on muscle involvement.<br>**Step 3: Specific Tests for Differential Diagnoses**<br>1. **Serum and Urine Immunofixation Electrophoresis**<br>○ **Reasoning**: To rule out monoclonal gammopathies (e.g., multiple myeloma, MGUS) which can be associated with neuropathies. Paraproteins can cause both demyelinating and axonal neuropathies.<br>2. **PET-CT Scan**<br>○ **Reasoning**: To rule out underlying malignancies that may present with paraneoplastic neuropathy. This is especially important given the patient's age and increased cancer risk.<br>3. **Autonomic Function Tests**<br>○ **Reasoning**: To assess the extent of autonomic involvement, which can be seen in both diabetic neuropathy and paraneoplastic syndromes. These tests can include heart rate variability, sweat tests, and blood pressure response to positional changes.<br>**Step 4: Genetic Testing (if family history is revised)**<br>1. **Genetic Testing for Hereditary Neuropathies**<br>○ **Reasoning**: If there is any revision in the family history or new suspicion arises, genetic tests can help identify hereditary neuropathies (e.g., Charcot-Marie-Tooth disease).<br>**Most Crucial Test**<br>The **most crucial test** from this list is the **CSF Analysis**. This test is key in distinguishing between CIDP and other neuropathies, such as diabetic neuropathy or paraneoplastic neuropathy. Elevated protein levels without a significant increase in white blood cells in the CSF are highly suggestive of CIDP. Given the patient's clinical presentation and nerve conduction study findings, confirming or ruling out CIDP is essential for guiding appropriate treatment.<br>**Conclusion**<br>By systematically conducting these additional examinations, we can further narrow down the differential diagnoses and identify the underlying cause of the patient's polyneuropathy. The CSF analysis stands out as the most crucial test to perform next, as it provides pivotal information to differentiate between CIDP and other potential causes.
72	A 58-year old male patient presents with a 1-year history of sensory-motor, symmetrical, non-length dependent polyneuropathy with subacute onset. The patient presents with paresthesia and weakness in the distal areas of the four limbs. Clinical examination does not show the presence of sensory ataxia, neuropathic pain, tremor, foot deformities, cranial nerve or autonomic involvement. Clinical examination shows cutaneous hemangiomas and hyperpigmentation. Past medical history is positive for IgA lambda monoclonal gammopathy. Nerve conduction studies show mixed demyelinating-axonal, sensory-motor, symmetric polyneuropathy. Screening lab tests including vitamin B12, folate, vitamin B6, vitamin E, HCV, HBV, ANA, ANCA, ENA, CRP, ESR, glycemia, HbA1c, copper and cryoglobulins are all negative, with positivity for IgA lambda paraprotein. There is no family history of polyneuropathy.	POEMS	**Step-by-Step Analysis and Additional Examinations**<br>1. **Clinical Assessment for POEMS Syndrome:**<br>○ **Reasoning:** Given the high suspicion of POEMS syndrome, further evaluation of its associated features is crucial.<br>○ **Tests:**<br>■ **VEGF (Vascular Endothelial Growth Factor) Levels:** Elevated in POEMS syndrome, VEGF is a biomarker for the disease.<br>■ **Full Body CT or MRI:** To identify organomegaly (spleen, liver, lymph nodes) and sclerotic bone lesions, both of which are common in POEMS syndrome.<br>■ **Endocrine Panel:** To evaluate for endocrinopathy which can include hypothyroidism, diabetes, hypogonadism, and adrenal insufficiency.<br>2. **Further Evaluation for CIDP:**<br>○ **Reasoning:** CIDP remains a strong differential diagnosis and warrants further specific testing.<br>○ **Tests:**<br>■ **Lumbar Puncture with CSF Analysis:** To check for elevated protein levels without pleocytosis, a characteristic finding in CIDP.<br>■ **Repetitive Nerve Stimulation or Single Fiber EMG:** To differentiate between demyelinating and axonal processes more clearly.<br>3. **Assessment for Monoclonal Gammopathy-Associated Neuropathy:**<br>○ **Reasoning:** Given the presence of monoclonal gammopathy, further tests to evaluate the extent and impact of this gammopathy on the neuropathy are necessary.<br>○ **Tests:**<br>■ **Bone Marrow Biopsy:** To assess the extent of plasma cell infiltration, which can help differentiate MGUS from more severe plasma cell disorders like multiple myeloma.<br>■ **Serum Free Light Chain Assay and Immunofixation Electrophoresis:** To quantify the monoclonal protein levels and evaluate the presence of light chain disease.<br>4. **General Neuropathy Assessment:**<br>○ **Reasoning:** Ensuring no other underlying or contributing causes for the neuropathy have been overlooked.<br>○ **Tests:**<br>■ **Autoantibody Panel (Anti-MAG, Anti-GM1, etc.):** To rule out other autoimmune neuropathies that may present similarly.<br>■ **Skin Biopsy for Nerve Fiber Density:** To evaluate small fiber neuropathy, which may not be detected on standard nerve conduction studies.<br>**Most Crucial Test:**<br>**VEGF (Vascular Endothelial Growth Factor) Levels** is the most crucial test from the list.<br>**Reasoning:**<br>● **High Suspicion of POEMS:** The combination of IgA lambda monoclonal gammopathy, skin changes (hemangiomas, hyperpigmentation), and mixed demyelinating-axonal neuropathy is highly suggestive of POEMS syndrome.<br>● **Diagnostic and Prognostic Value:** Elevated VEGF levels are not only diagnostic of POEMS syndrome but also correlate with disease activity and severity.<br>● **Specificity:** Elevated VEGF is more specific for POEMS syndrome compared to the other tests, which can be indicative of several other conditions.<br>Thus, measuring VEGF levels will significantly narrow down the differential diagnosis and guide further diagnostic and therapeutic steps.
73	An 87-year-old male patient presents with a seven-month history of sensory-motor, symmetrical, length-dependent polyneuropathy with subacute onset and progressive course. The patient reports	CIDP	**Step-by-Step Analysis and Additional Examinations**<br>**Step 1: Review Current Findings**<br>● **Clinical Findings:** Subacute onset and progressive course of sensory-motor, symmetrical, length-dependent polyneuropathy with sensory ataxia and mild weakness in ankle dorsiflexion.<br>● **Past Medical History:** IgG kappa monoclonal gammopathy, prostate cancer.<br>● **Nerve Conduction Studies:** Demyelinating, sensory-motor, symmetric polyneuropathy.

	paresthesia and hypoesthesia in the lower limbs, associated with sensory ataxia and mild weakness in ankle dorsiflexion. Clinical examination does not reveal tremor, foot deformities, cranial nerve involvement, or autonomic dysfunction. Past medical history is positive for IgG kappa monoclonal gammopathy and prostate cancer. Nerve conduction studies show a demyelinating, sensory-motor, symmetric polyneuropathy. Screening lab tests including vitamin B12, folate, vitamin B6, vitamin E, HBV, HCV, ANA, ANCA, ENA, CRP, ESR, glycemia, HbA1c, copper, and cryoglobulins are all negative, with positivity for IgG kappa paraprotein. There is no family history of polyneuropathy.		● **Laboratory Tests:** Extensive negative screening (including vitamins, infections, autoimmune markers, metabolic markers) except for positive IgG kappa paraprotein.<br>**Step 2: Identify Gaps in Current Information**<br>● The primary gap is distinguishing between MGUS-related neuropathy, CIDP, and paraneoplastic neuropathy. Additional examinations are needed to further narrow the differential diagnoses.<br>**Step 3: Propose Additional Examinations**<br>1. **Serum and Urine Immunofixation Electrophoresis**<br>○ **Reasoning:**<br>■ To further characterize the monoclonal protein (IgG kappa) and quantify the level of monoclonal protein.<br>■ Helps to confirm MGUS and evaluate for progression to multiple myeloma or other plasma cell dyscrasias.<br>2. **Lumbar Puncture with Cerebrospinal Fluid (CSF) Analysis**<br>○ **Reasoning:**<br>■ In CIDP, CSF analysis often shows elevated protein without pleocytosis (albumin-cytological dissociation).<br>■ Helps to differentiate CIDP from other inflammatory and infectious causes of neuropathy.<br>■ Can rule out malignant cells, indicating paraneoplastic syndrome if cancer cells are present.<br>3. **MRI of the Spine**<br>○ **Reasoning:**<br>■ To evaluate for possible spinal cord involvement or compressive neuropathies.<br>■ Helps to identify nerve root enhancement that can be seen in CIDP.<br>4. **Paraneoplastic Antibody Panel**<br>○ **Reasoning:**<br>■ To detect antibodies associated with paraneoplastic syndromes, which could suggest a link between the neuropathy and the patient's prostate cancer.<br>■ Relevant antibodies include anti-Hu, anti-Yo, anti-Ri, and others.<br>5. **Repeat and Advanced Nerve Conduction Studies and Electromyography (EMG)**<br>○ **Reasoning:**<br>■ To provide a more detailed assessment of the extent and nature of the neuropathy.<br>■ Helps to confirm the demyelinating nature and evaluate for possible axonal involvement.<br>6. **Bone Marrow Biopsy**<br>○ **Reasoning:**<br>■ To assess the bone marrow for plasma cell dyscrasia, which could indicate multiple myeloma or a related condition.<br>■ Important for staging if progression from MGUS to a more severe condition is suspected.<br>**Step 4: Determine the Most Crucial Test**<br>**The most crucial test from the list is the Lumbar Puncture with Cerebrospinal Fluid (CSF) Analysis.**<br>**Reasoning:**<br>● **Key Diagnostic Information:** CSF analysis can provide definitive clues about the inflammatory nature of the neuropathy (e.g., elevated protein without pleocytosis in CIDP).<br>● **Differentiating Factor:** It helps to differentiate between CIDP and other causes of neuropathy that might not show the same CSF profile.<br>● **Immediate Impact:** Results can guide immediate treatment decisions, such as immunotherapy for CIDP.<br>● **Rule Out Other Conditions:** Helps rule out infectious or malignant causes, which are critical given the patient's cancer history.<br>This test is particularly crucial because it has the potential to confirm CIDP, a treatable condition, and significantly impact the patient's management and prognosis.
74	A 51-year old female patient presents with a 35-year history of chronic, sensory-motor, symmetrical, length dependent polyneuropathy with insidious onset and slow progression. The patient presents with paresthesia, tremor, hypoesthesia up to the calves, bilateral steppage, and hammertoes. Clinical examination does not reveal cranial nerve or autonomic involvement. Past medical history shows chronic renal insufficiency. Nerve conduction studies show an axonal, symmetric, sensory-motor polyneuropathy. Screening lab tests including vitamin B12, folate, vitamin B6, vitamin E, HBV, HCV, glycemia, HbA1c, ANA, ANCA, ENA, CRP, ESR, copper, cryoglobulins, and serum electrophoresis are all negative. Her father and two uncles have walking difficulties.	CMT2	**Step-by-Step Analysis and Additional Examinations**<br>**Step 1: Clinical Summary Review**<br>● **Patient:** 51-year-old female<br>● **History:** 35-year history of chronic, sensory-motor, symmetrical, length-dependent polyneuropathy<br>● **Symptoms:** Paresthesia, tremor, hypoesthesia up to the calves, bilateral steppage, hammertoes<br>● **Clinical Examination:** No cranial nerve or autonomic involvement<br>● **Past Medical History:** Chronic renal insufficiency<br>● **Family History:** Father and two uncles with walking difficulties<br>● **Nerve Conduction Studies:** Axonal, symmetric, sensory-motor polyneuropathy<br>● **Screening Lab Tests:** All negative (vitamin B12, folate, vitamin B6, vitamin E, HBV, HCV, glycemia, HbA1c, ANA, ANCA, ENA, CRP, ESR, copper, cryoglobulins, and serum electrophoresis)<br>**Step 2: Identification of Additional Examinations**<br>**1. Genetic Testing for Hereditary Neuropathy**<br>● **Reasoning:** Given the strong family history of similar symptoms and the clinical features suggestive of a hereditary neuropathy like Charcot-Marie-Tooth (CMT) disease, genetic testing is crucial. Identifying specific mutations associated with CMT (e.g., PMP22, MPZ, GJB1) can confirm the diagnosis and subtype.<br>**2. Renal Function Tests and Evaluation**<br>● **Reasoning:** The patient has a history of chronic renal insufficiency. A comprehensive renal function panel (including serum creatinine, BUN, eGFR) will help assess the current status of renal function and determine if renal insufficiency could be contributing to the neuropathy.<br>**3. Skin Biopsy for Intraepidermal Nerve Fiber Density (IENFD)**<br>● **Reasoning:** Skin biopsy can help assess small fiber neuropathy, which may not be detected by standard nerve conduction studies. Reduced IENFD can indicate a small fiber component in neuropathies.<br>**4. Electromyography (EMG)**<br>● **Reasoning:** EMG can help differentiate between neuropathic and myopathic conditions, further characterizing the type and extent of nerve damage and confirming the axonal nature of the neuropathy.<br>**5. Comprehensive Metabolic Panel (CMP)**<br>● **Reasoning:** Evaluating electrolytes, liver function, and glucose levels can help identify metabolic causes or contributing factors for the neuropathy.<br>**6. Lumbar Puncture (CSF Analysis)**<br>● **Reasoning:** CSF analysis can rule out inflammatory or infectious causes of neuropathy, such as chronic inflammatory demyelinating polyneuropathy (CIDP), which can occasionally present with axonal features.<br>**7. Immunofixation Electrophoresis (IFE) and Serum Free Light Chains**

			● **Reasoning:** To exclude the possibility of a monoclonal gammopathy, which can be associated with neuropathy. Conditions like amyloidosis or multiple myeloma may not be detected by standard serum electrophoresis.<br>**Step 3: Determination of the Most Crucial Test**<br>**Most Crucial Test: Genetic Testing for Hereditary Neuropathy**<br>● **Reasoning:** The most compelling evidence points towards a hereditary neuropathy, given the patient's chronic, slowly progressive symptoms, typical clinical features, and strong family history. Genetic testing can definitively diagnose and subtype Charcot-Marie-Tooth disease or other hereditary neuropathies. This will not only confirm the diagnosis but also provide important information for family counseling and future management.<br>**Conclusion**<br>The additional examinations listed above are essential to narrow down the differential diagnoses and identify the exact cause of the polyneuropathy. Genetic testing for hereditary neuropathy stands out as the most crucial test due to the significant family history and clinical features indicative of a genetic cause. This will provide a definitive diagnosis and guide further management.
75	An 81-year-old male patient presents with a 13-month history of sensory-motor, symmetrical, non-length dependent polyneuropathy with subacute onset and progressive course. The patient presents with lumbar pain associated with paresthesia, neuropathic pain, and weakness that are distributed both proximally and distally in the four limbs. Clinical examination also reveals the presence of mild sensory ataxia. There are no tremor, cramps, foot deformities, cranial nerve or autonomic involvement. Past medical history is positive for type 2 diabetes and Hashimoto thyroiditis. Nerve conduction studies show a mixed demyelinating-axonal, sensory-motor polyneuropathy. Screening lab tests show the presence of IgG lambda monoclonal gammopathy and raised HbA1c levels. Remaining exams including vitamin B12, folate, vitamin B6, vitamin E, HCV, HBV, ANA, ANCA, ENA, CRP, ESR, copper and cryoglobulins are all negative. Lumbosacral spine x-ray shows the presence of a single osteosclerotic bone lesion. There is no family history of polyneuropathy.	POEMS	**Step-by-Step Analysis and Additional Examinations**<br>**Step 1: Review of Clinical Data**<br>Based on the clinical scenario provided, the patient presents with a complex sensory-motor, symmetrical, non-length dependent polyneuropathy with a subacute onset and progressive course. Key findings include:<br>● IgG lambda monoclonal gammopathy<br>● Raised HbA1c levels<br>● Single osteosclerotic bone lesion<br>● Mixed demyelinating-axonal, sensory-motor polyneuropathy on nerve conduction studies<br>**Step 2: Identification of Potential Diagnoses**<br>The leading differential diagnoses include:<br>1. Monoclonal Gammopathy of Undetermined Significance (MGUS)<br>2. Diabetic Polyneuropathy<br>3. POEMS Syndrome<br>**Step 3: Additional Examinations**<br>To narrow down the differential diagnoses, the following additional examinations are recommended:<br>1. **Serum and Urine Immunofixation Electrophoresis (IFE)**<br>○ **Reasoning:** To further characterize the monoclonal gammopathy and check for the presence of other monoclonal proteins. This helps differentiate MGUS from more serious plasma cell disorders like multiple myeloma or POEMS syndrome.<br>2. **Serum Free Light Chain Assay**<br>○ **Reasoning:** To assess for abnormal free light chain ratios, which can indicate conditions such as POEMS syndrome or multiple myeloma.<br>3. **Bone Marrow Biopsy**<br>○ **Reasoning:** To evaluate the bone marrow for plasma cell disorders, which is crucial in diagnosing POEMS syndrome, multiple myeloma, and other hematological conditions.<br>4. **PET-CT Scan**<br>○ **Reasoning:** To identify additional osteosclerotic lesions, organomegaly, or other features suggestive of POEMS syndrome or malignancy.<br>5. **VEGF (Vascular Endothelial Growth Factor) Level**<br>○ **Reasoning:** Elevated VEGF is a key diagnostic marker for POEMS syndrome.<br>6. **MRI of the Spine**<br>○ **Reasoning:** To assess for any other skeletal abnormalities or spinal involvement that may be contributing to the patient's symptoms.<br>7. **Nerve Biopsy**<br>○ **Reasoning:** To obtain a detailed pathological analysis of the peripheral nerves, which can help differentiate between various types of neuropathy.<br>8. **Electromyography (EMG)**<br>○ **Reasoning:** To further characterize the neuropathy and assess the extent of nerve and muscle involvement.<br>9. **Comprehensive Endocrine Panel**<br>○ **Reasoning:** To check for other endocrine abnormalities that could be associated with POEMS syndrome or related disorders.<br>10. **Chest and Abdominal Imaging (CT or Ultrasound)**<br>○ **Reasoning:** To look for organomegaly or lymphadenopathy, which are features of POEMS syndrome.<br>**Step 4: Most Crucial Test**<br>**Bone Marrow Biopsy**<br>● **Reasoning:** This test is the most crucial as it can provide definitive information regarding the presence of plasma cell dyscrasias, which are central to diagnosing POEMS syndrome, multiple myeloma, or confirming MGUS. A bone marrow biopsy can reveal the percentage of plasma cells, the presence of clonal plasma cells, and other hematological abnormalities, guiding the diagnosis and subsequent management.<br>**Conclusion**<br>The bone marrow biopsy is the most crucial test from the list as it provides comprehensive information about the underlying hematological condition, which is pivotal in diagnosing POEMS syndrome and distinguishing it from other causes of monoclonal gammopathy and polyneuropathy.
76	A 51-year-old female patient presents with a 6-week history of sensory, symmetrical, length dependent polyneuropathy with acute-subacute onset. The patient presents with paresthesia and hypoesthesia with a stocking-glove distribution and sensory ataxia. Clinical examination reveals presence of severe ataxia with inability to stand without support. There is no weakness or neuropathic pain, tremor, foot deformities, cranial nerve or autonomic involvement. Past medical history is positive for type 1 diabetes since	Copper deficiency neuropathy	**Step-by-Step Analysis and Additional Examinations**<br>**Patient Summary Recap:**<br>● **Age:** 51 years old<br>● **Gender:** Female<br>● **Symptoms:** Sensory, symmetrical, length-dependent polyneuropathy; acute-subacute onset; paresthesia and hypoesthesia in a stocking-glove distribution; sensory ataxia; severe ataxia with inability to stand without support.<br>● **Clinical Examination:** Severe ataxia; no weakness, neuropathic pain, tremor, foot deformities, cranial nerve, or autonomic involvement.<br>● **Medical History:** Type 1 diabetes since age 23.<br>● **Nerve Conduction Studies:** Pure sensory, length-dependent, axonal polyneuropathy.<br>● **Laboratory Findings:** Hyperglycemia, elevated HbA1c, severe copper deficiency. Negative for vitamin B12, folate, vitamin B6, vitamin E, HBV, HCV, ANA, ANCA, ENA, CRP, ESR, cryoglobulins, and serum electrophoresis.<br>● **Family History:** No family history of polyneuropathy.<br>**Additional Examinations**<br>**1. Serum Ceruloplasmin and Copper Levels**<br>● **Reasoning:**

	the age of 23. Nerve conduction studies show presence of a pure sensory, length dependent, axonal polyneuropathy. Screening lab tests reveal hyperglycemia, elevated HbA1c levels and severe copper deficiency. Remaining exams including vitamin B12, folate, vitamin B6, vitamin E, HBV, HCV, ANA, ANCA, ENA, CRP, ESR, cryoglobulins, and serum electrophoresis are all negative. There is no family history of polyneuropathy.		○ Copper deficiency is already noted, but further investigation into ceruloplasmin levels is necessary to understand the underlying cause of copper deficiency. Ceruloplasmin is a copper-carrying protein in the blood, and its deficiency can indicate Wilson's disease or other disorders of copper metabolism.<br>**2. Genetic Testing for Wilson's Disease**<br>● **Reasoning:**<br>○ Given the severe copper deficiency, Wilson's disease (a genetic disorder of copper metabolism) should be ruled out. Genetic testing can confirm or exclude this diagnosis.<br>**3. Detailed Gastrointestinal Evaluation**<br>● **Reasoning:**<br>○ Copper is absorbed in the stomach and proximal small intestine. Malabsorption syndromes, celiac disease, or previous gastrointestinal surgeries could contribute to copper deficiency. Endoscopy, biopsy, and specific tests for celiac disease (tissue transglutaminase antibodies) can help identify gastrointestinal causes.<br>**4. Skin Biopsy for Intraepidermal Nerve Fiber Density (IENFD)**<br>● **Reasoning:**<br>○ A skin biopsy can provide additional information about small fiber neuropathy, which might not be evident on standard nerve conduction studies. It helps assess the density of small nerve fibers and can be useful in diagnosing causes of sensory neuropathies.<br>**5. MRI of the Brain and Spinal Cord**<br>● **Reasoning:**<br>○ MRI can help identify any structural abnormalities in the central nervous system that might contribute to the ataxia and sensory deficits. It can also detect changes suggestive of demyelination, lesions, or other pathologies affecting the dorsal columns or peripheral nerves.<br>**6. Serum and Urine Heavy Metals Screen**<br>● **Reasoning:**<br>○ Exposure to heavy metals (like lead or arsenic) can cause polyneuropathy. Screening for heavy metals in blood and urine can help identify toxic causes of neuropathy.<br>**7. Electromyography (EMG)**<br>● **Reasoning:**<br>○ Although the nerve conduction studies indicated a sensory neuropathy, an EMG can provide further insight into the extent of the neuropathy and help rule out any motor involvement that might have been initially overlooked.<br>**8. Lumbar Puncture with Cerebrospinal Fluid (CSF) Analysis**<br>● **Reasoning:**<br>○ CSF analysis can help rule out inflammatory or infectious causes of neuropathy. Elevated protein levels in CSF can indicate Guillain-Barré syndrome or chronic inflammatory demyelinating polyneuropathy (CIDP).<br>**Most Crucial Test**<br>**Serum Ceruloplasmin and Copper Levels**<br>This test is most crucial because the patient already has severe copper deficiency, and understanding the underlying cause of this deficiency is essential. Determining ceruloplasmin levels will help diagnose or exclude Wilson's disease and other disorders of copper metabolism, which is pivotal for targeted treatment and management.<br>**Conclusion**<br>Conducting a serum ceruloplasmin and copper level test is the most immediate and crucial step. This will provide a clearer understanding of the underlying cause of the severe copper deficiency, guiding further investigations and appropriate management. The additional examinations will complement this primary test and help narrow down the differential diagnoses effectively.
77	A 70-year-old female patient presents with a 1-month history of sensory-motor, asymmetrical, non-length dependent, step-wise multineuropathy with acute onset. The patient presents with paresthesia, hypoesthesia, neuropathic pain and weakness in a multifocal distribution. Clinical examination does not reveal presence of ataxia, tremor, foot deformities, cranial nerve or autonomic involvement. Past medical history reveals the presence of intermittent fever and malaise. Nerve conduction studies show a mixed sensory-motor, axonal multineuropathy and unilateral carpal tunnel syndrome. Screening lab tests reveal high levels of CRP and ESR. Remaining lab exams including vitamin B12, folate, vitamin B6, vitamin E, HCV, HBV, ANA, ENA, glycemia, HbA1c, copper, cryoglobulins and serum electrophoresis are normal. There is no family history of polyneuropathy.	Vasculitic neuropathy	**Step-by-Step Analysis and Additional Examinations**<br>**1. Comprehensive Clinical Evaluation:**<br>Given the initial findings, further evaluation is necessary to narrow down the differential diagnoses. The following steps outline additional examinations that would be valuable.<br>**2. Additional Laboratory Tests:**<br>1. **Antineutrophil Cytoplasmic Antibodies (ANCA)**<br>○ **Reasoning:** To evaluate for ANCA-associated vasculitis, such as granulomatosis with polyangiitis (GPA) or microscopic polyangiitis (MPA), which can present with systemic inflammatory signs and neuropathy.<br>2. **Anti-Myeloperoxidase (MPO) and Anti-Proteinase 3 (PR3) Antibodies**<br>○ **Reasoning:** Specific subtypes of ANCA that help differentiate between types of vasculitis.<br>3. **Lyme Serology (ELISA and Western Blot)**<br>○ **Reasoning:** To rule out Lyme disease, especially if the patient resides in or has traveled to an endemic area, given the potential for infectious neuropathy.<br>4. **HIV and Syphilis Serologies**<br>○ **Reasoning:** To exclude these infections, which can cause neuropathy and systemic symptoms.<br>5. **Complement Levels (C3, C4, CH50)**<br>○ **Reasoning:** Decreased complement levels may indicate an underlying immune complex-mediated vasculitis.<br>6. **Serum Protein Electrophoresis (SPEP) and Immunofixation Electrophoresis (IFE)**<br>○ **Reasoning:** To evaluate for monoclonal gammopathies, which can be associated with neuropathy.<br>7. **Urinalysis with Microscopy**<br>○ **Reasoning:** To check for hematuria or proteinuria, which can indicate renal involvement, often seen in systemic vasculitis.<br>8. **Rheumatoid Factor (RF) and Anti-Cyclic Citrullinated Peptide (Anti-CCP)**<br>○ **Reasoning:** To evaluate for rheumatoid arthritis, which can be associated with vasculitis and neuropathy.<br>9. **Angiotensin-Converting Enzyme (ACE) Level**<br>○ **Reasoning:** Elevated ACE levels can be seen in sarcoidosis, which can cause neuropathy.<br>**3. Instrumental Examinations:**<br>1. **Nerve Biopsy**<br>○ **Reasoning:** To directly assess for vasculitic changes, inflammation, and other pathological findings in the peripheral nerves.<br>2. **Skin Biopsy with Immunofluorescence**<br>○ **Reasoning:** To evaluate for small-vessel vasculitis if systemic vasculitis is suspected.<br>3. **MRI with Contrast of Affected Limbs**<br>○ **Reasoning:** To assess for structural abnormalities, inflammation, and to provide a detailed evaluation of the affected nerves and surrounding tissues.

			4. **Positron Emission Tomography (PET) Scan**<br>○ **Reasoning:** To identify potential malignancies or systemic inflammatory diseases that may not be evident on other imaging modalities.<br>5. **Electromyography (EMG)**<br>○ **Reasoning:** To further characterize the extent and pattern of neuropathy and to differentiate between axonal and demyelinating processes.<br>**4. Most Crucial Test:**<br>Among the additional examinations, the most crucial test to narrow down the diagnosis is the **nerve biopsy**.<br>**Reasoning:**<br>● A nerve biopsy provides direct histopathological evidence of vasculitis, including the presence of inflammatory cells, fibrinoid necrosis, and other specific features of vasculitic neuropathy.<br>● This test can confirm the diagnosis of vasculitic neuropathy, differentiate it from other types of neuropathies, and help guide appropriate treatment.<br>● Given the patient's acute, multifocal neuropathy with elevated inflammatory markers and systemic symptoms, a nerve biopsy can provide definitive diagnostic information that other tests may not.<br>**Conclusion**<br>To narrow down the differential diagnoses, the following additional tests should be conducted:<br>● Laboratory tests: ANCA, MPO/PR3, Lyme serology, HIV/syphilis serologies, complement levels, SPEP/IFE, urinalysis, RF/Anti-CCP, ACE level.<br>● Instrumental examinations: nerve biopsy, skin biopsy, MRI with contrast, PET scan, EMG.<br>The most crucial test from this list is the **nerve biopsy**, as it offers direct evidence of vasculitis, which is the most likely diagnosis based on the clinical scenario.
78	A 48-year-old male patient presents with a 4-month history of sensory-motor, asymmetric, length dependent polyneuropathy with subacute onset and rapidly progressive course. The patient presents with paresthesia, hypoesthesia, and weakness in the distal segments of the four limbs in an asymmetric distribution. Clinical evaluation reveals peripheral edema in the lower limbs. The patient reports recent appearance of several skin growths that were diagnosed as cutaneous angiomas by the dermatologist. He also reports losing approximately 10 kg over the past 12 months. There are no tremor, ataxia, neuropathic pain, foot deformities, cranial nerve or autonomic involvement. Past medical history is positive for fibromyalgia and liver steatosis. Nerve conduction studies show a mixed demyelinating-axonal, sensory-motor, symmetric polyneuropathy. Screening lab tests including folate, vitamin B6, vitamin E, HCV, HBV, ANA, ANCA, ENA, CRP, ESR, glycemia, HbA1c, copper and cryoglobulins are negative. Serum electrophoresis shows positivity for IgA lambda paraprotein, and there is evidence of vitamin B12 deficiency. There is no family history of polyneuropathy.	POEMS	**Step-by-Step Analysis of Additional Examinations**<br>**Step 1: Assess Comprehensive Data**<br>The patient presents with a 4-month history of sensory-motor, asymmetric, length-dependent polyneuropathy with subacute onset and rapidly progressive course. Symptoms include paresthesia, hypoesthesia, and weakness in the distal segments of the four limbs, with peripheral edema in the lower limbs. There are no tremor, ataxia, neuropathic pain, foot deformities, cranial nerve, or autonomic involvement. The patient has a history of fibromyalgia and liver steatosis, with recent weight loss of 10 kg and cutaneous angiomas. Nerve conduction studies indicate a mixed demyelinating-axonal, sensory-motor, symmetric polyneuropathy. Laboratory tests are negative except for IgA lambda paraprotein and vitamin B12 deficiency.<br>**Step 2: List Additional Examinations**<br>1. **Full Body PET-CT Scan**<br>○ **Reasoning:** To identify any occult malignancies that could be causing paraneoplastic polyneuropathy. Given the patient's rapid symptom progression, weight loss, and the presence of cutaneous angiomas, a PET-CT scan is crucial for detecting tumors or metastases that might not be apparent through other imaging techniques.<br>2. **Bone Marrow Biopsy**<br>○ **Reasoning:** To investigate the presence of hematologic malignancies such as multiple myeloma or other plasma cell dyscrasias associated with the IgA lambda paraprotein detected in the serum electrophoresis. This test is essential for confirming or ruling out malignancies that could cause paraneoplastic syndromes.<br>3. **SPEP (Serum Protein Electrophoresis) and UPEP (Urine Protein Electrophoresis) with Immunofixation**<br>○ **Reasoning:** To further characterize the monoclonal gammopathy and to check for Bence Jones proteins in urine, which can be indicative of multiple myeloma. Detailed protein electrophoresis helps in identifying the type and extent of monoclonal proteins.<br>4. **CT Scan of Chest, Abdomen, and Pelvis**<br>○ **Reasoning:** To detect any tumors or abnormal growths in organs that could be causing paraneoplastic syndromes. This imaging helps identify primary or secondary malignancies contributing to the patient's symptoms.<br>5. **Skin Biopsy of Cutaneous Angiomas**<br>○ **Reasoning:** To histologically examine the angiomas and identify any specific markers or characteristics that might indicate an underlying malignancy or a paraneoplastic syndrome. This can provide clues about the systemic condition leading to neuropathy.<br>6. **Lumbar Puncture with CSF Analysis**<br>○ **Reasoning:** To analyze cerebrospinal fluid for any inflammatory markers, abnormal cells, or infectious agents that might contribute to neuropathy. This is particularly useful in ruling out central nervous system involvement in paraneoplastic syndromes.<br>7. **Additional Laboratory Tests:**<br>○ **Reasoning:** To rule out other potential causes of neuropathy, such as:<br>■ **Heavy Metal Screening:** Exposure to heavy metals like lead or arsenic can cause neuropathy.<br>■ **Thyroid Function Tests:** Hypothyroidism can cause peripheral neuropathy.<br>■ **HIV Testing:** HIV infection can lead to neuropathy.<br>**Step 3: Determine the Most Crucial Test**<br>**Full Body PET-CT Scan:**<br>● **Reasoning:** This test is the most crucial because it can provide comprehensive imaging of the entire body to identify any occult malignancies. Given the patient's clinical presentation with rapid progression, asymmetric neuropathy, significant weight loss, and cutaneous angiomas, detecting an underlying malignancy is critical. A PET-CT scan offers a detailed overview that can guide further diagnostic and therapeutic steps.<br>**Conclusion**<br>The most crucial test to conduct is the full body PET-CT scan. This examination will help in identifying any hidden malignancies that could explain the patient's symptoms and guide the next steps in diagnosis and treatment.
79	A 55-year-old male patient presents with a 1-year history of a pure sensory, length dependent polyneuropathy with insidious onset and slow progressive course. The patient presents with paresthesia and neuropathic pain in feet. He also reports cramps. Clinical examination does not reveal ataxia, tremor, foot deformities, cranial nerve or autonomic involvement. Past medical history is positive for IgG MGUS and Hashimoto's thyroiditis. Nerve conduction studies show an axonal,	MGUS-associated neuropathy	**Step-by-Step Analysis and Additional Examinations**<br>To narrow down the differential diagnoses, additional laboratory and instrumental examinations are required. Here is a step-by-step approach to the necessary tests and their rationales:<br>**1. Serum Protein Electrophoresis (SPEP) and Immunofixation Electrophoresis (IFE)**<br>**Reasoning:**<br>● The patient has IgG MGUS, and further characterization of the monoclonal protein is essential. SPEP and IFE can help quantify the monoclonal protein and detect any additional paraproteins.<br>● These tests will assist in confirming the diagnosis of MGUS and rule out progression to multiple myeloma or other plasma cell disorders.<br>**2. Serum Free Light Chain Assay**<br>**Reasoning:**<br>● To evaluate the presence and levels of free light chains, which can be elevated in MGUS and related disorders.<br>● This test helps in assessing the risk of progression from MGUS to malignant conditions like multiple myeloma.<br>**3. Lumbar Puncture with Cerebrospinal Fluid (CSF) Analysis**<br>**Reasoning:**

	sensory-motor, symmetric, length dependent polyneuropathy. Screening lab tests including vitamin B12, folate, vitamin B6, vitamin E, HBV, HCV, glycemia, HbA1c, ANA, ANCA, ENA, CRP, ESR, and copper are all negative, with positivity for IgG monoclonal gammopathy. There is no family history of polyneuropathy.		● This can help rule out chronic inflammatory demyelinating polyneuropathy (CIDP) by checking for elevated protein levels and the presence of oligoclonal bands.<br>● CSF analysis is crucial for identifying inflammatory or infectious causes of neuropathy.<br>**4. Quantitative Sensory Testing (QST)**<br>**Reasoning:**<br>● QST can help quantify the sensory deficits and differentiate between small and large fiber involvement.<br>● This test will provide objective data on the sensory disturbances reported by the patient.<br>**5. Skin Biopsy with Intraepidermal Nerve Fiber Density (IENFD) Measurement**<br>**Reasoning:**<br>● To assess small fiber neuropathy, which might not be detected by routine nerve conduction studies.<br>● This test can be particularly useful in cases with a predominant sensory phenotype.<br>**6. Autonomic Testing (e.g., QSART, Tilt Table Test)**<br>**Reasoning:**<br>● Despite the lack of autonomic symptoms, autonomic testing can uncover subclinical autonomic dysfunction, which may support specific neuropathy diagnoses like amyloidosis.<br>● These tests help in ruling out autonomic neuropathy which could be associated with certain systemic conditions.<br>**7. Whole-body Imaging (e.g., MRI or PET-CT)**<br>**Reasoning:**<br>● To rule out malignancies, lymphoproliferative disorders, or other systemic conditions that could present with neuropathy.<br>● Imaging can also detect skeletal abnormalities or compression syndromes contributing to the neuropathy.<br>**8. Thyroid Function Tests (TFTs) including Anti-TPO and Anti-thyroglobulin Antibodies**<br>**Reasoning:**<br>● Given the patient's history of Hashimoto’s thyroiditis, it is essential to assess thyroid function as hypothyroidism can cause or exacerbate neuropathy.<br>● Anti-TPO and anti-thyroglobulin antibodies can help gauge the autoimmune activity.<br>**9. Heavy Metal Screening (e.g., Lead, Mercury, Arsenic)**<br>**Reasoning:**<br>● To exclude heavy metal toxicity as a cause of neuropathy, especially if the patient has a history of exposure or occupational risk.<br>● This test is pertinent due to the nonspecific nature of symptoms that could overlap with toxic neuropathies.<br>**Conclusion: Most Crucial Test**<br>The **Serum Protein Electrophoresis (SPEP) and Immunofixation Electrophoresis (IFE)** is the most crucial test in this scenario. It will provide detailed information about the monoclonal protein, quantify it, and help determine if there has been any progression from MGUS to a more serious hematologic condition. This test is pivotal given the patient's known IgG MGUS and its strong association with neuropathy.
80	A 70-year-old male patient presents with a 2-year history of pure sensory, symmetrical, length dependent polyneuropathy with insidious onset. The patient presents with neuropathic pain in the lower limbs in a distal distribution. Clinical examination reveals hyperalgesia to pinprick and absence of tactile hypoesthesia. There are no foot deformities, ataxia, tremor, or cranial nerve involvement. The patient reports reduced sweating. Past medical history is negative. Nerve conduction studies show a pure sensory, symmetric, axonal polyneuropathy. Screening lab tests show raised glycemia, elevated HbA1c, and reduced vitamin B12. Remaining exams including folate, vitamin B6, vitamin E, HBV, HCV, ANA, ANCA, ENA, CRP, ESR, copper, cryoglobulins, and serum electrophoresis are all negative. There is no family history of polyneuropathy.	Diabetic neuropathy	**Step-by-Step Analysis of Additional Examinations**<br>**Step 1: Review Comprehensive Data from the Clinical Scenario**<br>● **Patient Profile:** 70-year-old male<br>● **Symptoms:**<br>○ 2-year history of pure sensory, symmetrical, length-dependent polyneuropathy<br>○ Insidious onset<br>○ Neuropathic pain in lower limbs (distal distribution)<br>○ Hyperalgesia to pinprick<br>○ Absence of tactile hypoesthesia<br>○ Reduced sweating<br>● **Clinical Findings:**<br>○ No foot deformities, ataxia, tremor, or cranial nerve involvement<br>● **Past Medical History:** Negative<br>● **Nerve Conduction Studies:** Pure sensory, symmetric, axonal polyneuropathy<br>● **Lab Results:**<br>○ Raised glycemia<br>○ Elevated HbA1c<br>○ Reduced vitamin B12<br>○ Normal levels of folate, vitamin B6, vitamin E, HBV, HCV, ANA, ANCA, ENA, CRP, ESR, copper, cryoglobulins, serum electrophoresis<br>● **Family History:** No polyneuropathy<br>**Step 2: List Necessary Additional Examinations**<br>1. **Glucose Tolerance Test (GTT)**<br>2. **Methylmalonic Acid and Homocysteine Levels**<br>3. **Quantitative Sensory Testing (QST)**<br>4. **Autonomic Function Testing**<br>5. **Skin Biopsy for Intraepidermal Nerve Fiber Density**<br>6. **Electromyography (EMG)**<br>7. **MRI of the Spine**<br>**Step 3: Detailed Reasoning for Each Proposed Test**<br>1. **Glucose Tolerance Test (GTT):**<br>○ **Reasoning:** To confirm the diagnosis of diabetes mellitus, especially since the patient has raised glycemia and elevated HbA1c. This test can help assess glucose metabolism over time.<br>○ **Utility:** It will provide a more comprehensive picture of glucose handling and can confirm or exclude diabetes as a contributing factor to the neuropathy.<br>2. **Methylmalonic Acid and Homocysteine Levels:**<br>○ **Reasoning:** To further evaluate the extent of vitamin B12 deficiency. Elevated levels of these metabolites can indicate functional B12 deficiency even when serum B12 levels are borderline or slightly low.

			○ **Utility:** These tests can help confirm if the neuropathy is related to B12 deficiency by revealing underlying metabolic disturbances.<br>3. **Quantitative Sensory Testing (QST):**<br>○ **Reasoning:** To objectively assess the sensory nerve function and quantify the severity and pattern of sensory loss.<br>○ **Utility:** This can help in differentiating between different types of sensory neuropathies and provide a baseline for monitoring progression or response to treatment.<br>4. **Autonomic Function Testing:**<br>○ **Reasoning:** Given the patient's reduced sweating, this test can assess the involvement of the autonomic nervous system.<br>○ **Utility:** It helps in diagnosing autonomic neuropathy which is common in diabetic patients and can provide insights into the extent of autonomic dysfunction.<br>5. **Skin Biopsy for Intraepidermal Nerve Fiber Density:**<br>○ **Reasoning:** To directly evaluate small nerve fibers, which might not be adequately assessed by routine nerve conduction studies.<br>○ **Utility:** It can confirm small fiber neuropathy, which is often associated with diabetic neuropathy and other systemic conditions.<br>6. **Electromyography (EMG):**<br>○ **Reasoning:** To further evaluate the extent and type of neuropathy. While nerve conduction studies have been done, EMG can provide additional information on muscle response and motor neuron involvement.<br>○ **Utility:** This test can help rule out other neuromuscular disorders and provide a complete picture of the neuropathy.<br>7. **MRI of the Spine:**<br>○ **Reasoning:** To rule out any structural causes of neuropathy such as spinal stenosis or other compressive pathologies that might be contributing to the symptoms.<br>○ **Utility:** MRI can reveal structural abnormalities that might explain some of the patient's symptoms.<br>**Step 4: Determine the Most Crucial Test**<br>**Most Crucial Test: Glucose Tolerance Test (GTT)**<br>● **Reasoning:** While the patient already has elevated glycemia and HbA1c indicating diabetes, the GTT can definitively confirm the diagnosis and assess the severity of glucose intolerance. Given the strong likelihood of diabetic neuropathy as the primary diagnosis, this test is crucial for confirming diabetes, which is necessary for appropriate management and treatment of the neuropathy.<br>**Conclusion**<br>The most crucial additional examination for this patient is the **Glucose Tolerance Test (GTT)**. This test will confirm the diagnosis of diabetes mellitus, which is strongly suspected based on the clinical presentation and laboratory findings, thereby facilitating targeted management of the underlying cause of the polyneuropathy.
81	A 67-year-old male patient presents with a 1-week history of pure motor, symmetrical, non-length dependent polyneuropathy with hyperacute onset. The patient presents with facial diplegia, diplopia, and weakness of the intrinsic muscles of the hands. Clinical examination reveals the presence of ophthalmoparesis affecting the right retrolateral muscle and the left superior rectus, facial diplegia, weakness of the intrinsic muscles of both hands, and mild sensory ataxia. There are no neuropathic pain, tremor, foot deformities or autonomic involvement. Past medical history is positive for type 1 diabetes, IgG MGUS, and arterial hypertension. Nerve conduction studies show normal result. Screening lab tests show hyperglycaemia and raised HbA1c levels, IgG monoclonal gammopathy, and mild increase of ESR and CRP levels. Remaining exams including vitamin B12, folate, vitamin B6, vitamin E, HBV, HCV, ANA, ENA, ANCA, copper, and cryoglobulins are normal. There is no family history of polyneuropathy.	GBS-facial diplegia variant	**Step-by-Step Analysis of Additional Examinations**<br>1. **Cerebrospinal Fluid (CSF) Analysis**<br>○ **Reasoning:**<br>■ CSF analysis is crucial in diagnosing Guillain-Barré Syndrome (GBS). Elevated protein levels with a normal white cell count (albuminocytologic dissociation) are typical in GBS, including its Miller Fisher Syndrome (MFS) variant.<br>■ CSF analysis helps exclude other infectious or inflammatory causes of polyneuropathy.<br>■ Given the hyperacute onset and cranial nerve involvement, CSF analysis is necessary to confirm the diagnosis and rule out other central nervous system pathologies.<br>2. **Anti-GQ1b Antibodies**<br>○ **Reasoning:**<br>■ Anti-GQ1b antibodies are highly specific for Miller Fisher Syndrome (MFS), a variant of GBS characterized by ophthalmoparesis, ataxia, and areflexia.<br>■ Testing for these antibodies would support the diagnosis of MFS, given the patient's presentation with ophthalmoparesis and facial diplegia.<br>3. **Magnetic Resonance Imaging (MRI) of the Brain and Spinal Cord**<br>○ **Reasoning:**<br>■ MRI can help exclude structural lesions or demyelinating diseases such as multiple sclerosis that might present with cranial nerve involvement and motor weakness.<br>■ An MRI can also rule out other potential causes of acute motor neuropathy, including stroke or space-occupying lesions.<br>4. **Electromyography (EMG)**<br>○ **Reasoning:**<br>■ EMG can provide additional information about the nature of the neuropathy. It can help differentiate between axonal and demyelinating processes, which is important in the context of motor neuropathy.<br>■ Although nerve conduction studies were normal, EMG might show acute denervation, supporting a diagnosis of GBS/MFS.<br>5. **Paraneoplastic Panel**<br>○ **Reasoning:**<br>■ Given the patient's history of IgG monoclonal gammopathy (MGUS), it is essential to rule out a paraneoplastic syndrome that might present with polyneuropathy.<br>■ Testing for paraneoplastic antibodies can help identify any underlying malignancy-associated neuropathy.<br>6. **Autonomic Function Testing**<br>○ **Reasoning:**<br>■ Although there is no reported autonomic involvement, comprehensive autonomic function testing can help identify subtle autonomic dysfunction, which can occur in GBS.<br>■ This testing can provide a more complete understanding of the patient's neuropathy.<br>**Most Crucial Test**<br>**Cerebrospinal Fluid (CSF) Analysis**<br>**Reasoning:**<br>● CSF analysis is the most crucial test because it provides direct evidence for diagnosing Guillain-Barré Syndrome (GBS). The presence of albuminocytologic dissociation (elevated protein with normal cell count) is a hallmark of GBS.<br>● Given the patient's rapid onset of symptoms and the cranial nerve involvement, ruling out central nervous system infections or inflammations is essential.<br>● Confirming or excluding GBS early is critical for timely management, including considering intravenous immunoglobulin (IVIG) or plasmapheresis treatment if GBS is confirmed.<br>In conclusion, while all proposed tests have their importance, CSF analysis stands out as the most crucial for confirming the diagnosis of Guillain-Barré Syndrome, particularly the Miller Fisher Syndrome variant, and guiding further management.
82	An 87-year-old male patient presents with a 6-month history of pure sensory, symmetrical, length dependent	B12-deficiency	**Step-by-Step Analysis for Additional Examinations**<br>**Step 1: Review Current Data**<br>**Patient Profile:**

	polyneuropathy with subacute onset. The patient presents with leg paresthesia, tremor and sensory ataxia without neuropathic pain, foot deformities, cranial nerve or autonomic involvement. Past medical history is silent. Nerve conduction studies show pure a symmetric, sensory, axonal polyneuropathy and unilateral carpal tunnel syndrome. Screening lab tests including folate, vitamin B6, vitamin E, HCV, HBV, ANA, ANCA, ENA, CRP, ESR, glycemia, HbA1c, copper, cryoglobulins and serum electrophoresis are all negative, with evidence of vitamin B12 deficiency. There is no family history of polyneuropathy.	polyneuropathy	● 87-year-old male<br>● 6-month history of pure sensory, symmetrical, length-dependent polyneuropathy<br>● Symptoms: leg paresthesia, tremor, sensory ataxia<br>● Diagnostic findings: Pure sensory, symmetric, axonal polyneuropathy, unilateral carpal tunnel syndrome<br>● Laboratory results: Negative for multiple systemic and autoimmune markers, but positive for vitamin B12 deficiency<br>● No family history of polyneuropathy<br>**Step 2: List Additional Examinations**<br>1. **Methylmalonic Acid (MMA) and Homocysteine Levels**<br>○ **Reasoning:**<br>■ Elevated MMA and homocysteine levels are sensitive indicators of vitamin B12 deficiency.<br>■ Helps confirm the functional deficiency of vitamin B12 even if serum B12 levels are borderline.<br>○ **Expected Outcome:**<br>■ Elevated levels would confirm the diagnosis of vitamin B12 deficiency neuropathy.<br>2. **Serum Protein Electrophoresis (SPEP) with Immunofixation and Urine Protein Electrophoresis (UPEP)**<br>○ **Reasoning:**<br>■ To rule out monoclonal gammopathies, such as multiple myeloma or monoclonal gammopathy of undetermined significance (MGUS), which can cause polyneuropathy.<br>■ Given the patient's age, these conditions are more prevalent and can present with sensory neuropathy.<br>○ **Expected Outcome:**<br>■ Absence of monoclonal protein would rule out these causes.<br>3. **Anti-Ganglioside Antibodies (e.g., anti-GM1, anti-GQ1b)**<br>○ **Reasoning:**<br>■ These antibodies are associated with certain types of immune-mediated neuropathies, such as multifocal motor neuropathy (MMN) or other variants.<br>■ Considering the chronic nature and sensory ataxia, ruling out an immune-mediated etiology is prudent.<br>○ **Expected Outcome:**<br>■ Negative results would help rule out immune-mediated neuropathies.<br>4. **MRI of the Cervical and Thoracic Spine**<br>○ **Reasoning:**<br>■ To rule out structural causes of myelopathy, such as cervical spondylotic myelopathy, which can present with sensory ataxia and paresthesia.<br>■ Helps evaluate for spinal cord compression or other abnormalities.<br>○ **Expected Outcome:**<br>■ Normal MRI would rule out structural spinal causes.<br>5. **Lumbar Puncture for Cerebrospinal Fluid (CSF) Analysis**<br>○ **Reasoning:**<br>■ To rule out central nervous system (CNS) involvement or infectious causes.<br>■ Helps identify elevated protein levels which are common in chronic inflammatory demyelinating polyneuropathy (CIDP).<br>○ **Expected Outcome:**<br>■ Normal or non-specific findings would help rule out CNS or inflammatory causes.<br>**Step 3: Determine the Most Crucial Test**<br>**Most Crucial Test: Methylmalonic Acid (MMA) and Homocysteine Levels**<br>**Reasoning:**<br>● **Primary Focus:** Given the confirmed vitamin B12 deficiency, it is crucial to determine the functional impact of this deficiency. Elevated MMA and homocysteine levels are direct indicators of vitamin B12 deficiency at the cellular level.<br>● **Diagnostic Value:** This test will provide definitive evidence supporting the diagnosis of vitamin B12 deficiency neuropathy.<br>● **Clinical Impact:** Confirming the diagnosis will guide appropriate treatment (e.g., vitamin B12 supplementation) and likely lead to symptom improvement, making it the most actionable test.<br>**Conclusion**<br>The most crucial test from the list is measuring methylmalonic acid (MMA) and homocysteine levels. This test will confirm the functional deficiency of vitamin B12, solidifying the diagnosis and guiding appropriate therapeutic interventions.
83	A 72-year-old male patient presents with a 1-year history of sensory-motor, symmetrical, non-length dependent polyneuropathy with subacute onset. The patient presents with hand weakness, paresthesia and hypoesthesia in the lower limbs, sensory ataxia, dysphagia, and pes cavus. There are no neuropathic pain or autonomic involvement. Past medical history is silent. Nerve conduction studies show a demyelinating, sensory-motor, asymmetric polyneuropathy. Screening lab tests including vitamin B12, folate, vitamin B6, vitamin E, HCV, HBV, ANA, ANCA, ENA, CRP, ESR, glycemia, HbA1c, copper, cryoglobulins and serum electrophoresis	CIDP	**Step-by-Step Analysis of Additional Examinations**<br>**Step 1: Comprehensive Analysis of Clinical Scenario**<br>The patient presents with:<br>● 1-year history of sensory-motor, symmetrical, non-length dependent polyneuropathy<br>● Subacute onset with hand weakness, paresthesia, hypoesthesia in lower limbs, sensory ataxia, dysphagia, and pes cavus<br>● Demyelinating, sensory-motor, asymmetric polyneuropathy on nerve conduction studies<br>● Negative past medical history and family history<br>● Negative initial screening lab tests<br>**Step 2: List of Necessary Additional Examinations**<br>**1. Lumbar Puncture for Cerebrospinal Fluid (CSF) Analysis**<br>● **Reasoning:**<br>○ CSF analysis is crucial in diagnosing CIDP.<br>○ Elevated CSF protein with a normal cell count (albuminocytologic dissociation) is typical in CIDP.<br>○ Helps exclude other inflammatory or infectious causes of polyneuropathy.<br>**2. Genetic Testing for PMP22 Gene Deletion/Duplication**<br>● **Reasoning:**<br>○ Hereditary Neuropathy with Liability to Pressure Palsies (HNPP) is associated with a deletion of the PMP22 gene.

	are all negative. There is no family history of polyneuropathy.		○ Genetic testing can confirm or exclude HNPP and other hereditary neuropathies like Charcot-Marie-Tooth disease.<br>○ This is particularly relevant given the patient's pes cavus and sensory ataxia.<br>**3. Serum Immunofixation Electrophoresis**<br>● **Reasoning:**<br>○ To detect monoclonal gammopathies which can be associated with neuropathies.<br>○ Conditions like POEMS syndrome or multiple myeloma can present with similar neuropathic features.<br>○ Even if initial serum electrophoresis was negative, immunofixation is more sensitive for detecting small monoclonal proteins.<br>**4. Anti-GM1 Antibodies**<br>● **Reasoning:**<br>○ Anti-GM1 antibodies are often present in patients with Multifocal Motor Neuropathy (MMN).<br>○ Testing for these antibodies helps in diagnosing MMN, especially given the asymmetrical findings on nerve conduction studies.<br>**5. Magnetic Resonance Imaging (MRI) of the Spine and Brachial Plexus**<br>● **Reasoning:**<br>○ MRI can detect nerve root or plexus enlargement, which is common in CIDP.<br>○ Helps in ruling out compressive or structural causes of neuropathy.<br>○ Useful in evaluating the extent of demyelination and inflammatory changes.<br>**6. Autoantibody Panel Including Anti-MAG and Anti-Hu Antibodies**<br>● **Reasoning:**<br>○ Anti-MAG antibodies are associated with demyelinating neuropathies, especially in older adults.<br>○ Paraneoplastic syndromes can present with neuropathies; anti-Hu antibodies can help detect these.<br>○ This panel helps identify autoimmune and paraneoplastic causes.<br>**7. Skin Biopsy for Intraepidermal Nerve Fiber Density**<br>● **Reasoning:**<br>○ Helps assess small fiber neuropathy which may not be detected on nerve conduction studies.<br>○ Useful if there are clinical signs of small fiber involvement despite normal screening tests.<br>**Step 3: Determine the Most Crucial Test**<br>**Most Crucial Test: Lumbar Puncture for Cerebrospinal Fluid (CSF) Analysis**<br>**Reasoning:**<br>● **Direct Relevance:** CSF analysis is directly related to confirming the most probable diagnosis, CIDP.<br>● **Diagnostic Value:** Elevated CSF protein with normal cell count is a hallmark of CIDP.<br>● **Exclusivity:** Helps exclude other differential diagnoses, such as infectious or inflammatory causes, that might not be apparent on initial blood tests.<br>● **Therapeutic Implications:** A positive result would not only confirm CIDP but also guide specific immunotherapy treatments.
84	A 60-year-old male patient presents with a 1-month history of pure sensory, symmetrical, length dependent polyneuropathy with subacute onset. The patient presents with frequent cramps, paresthesia, and neuropathic pain in the feet. There are no sensory ataxia, tremor, foot deformities, cranial nerve or autonomic involvement. Past medical history is positive for multiple myeloma, for which the patient has undergone chemotherapy. Symptoms started after the first cycle of chemotherapy. Nerve conduction studies show a pure sensory, symmetric, axonal polyneuropathy. Screening lab tests including vitamin B12, folate, vitamin B6, vitamin E, HBV, HCV, glycemia, HbA1c, ANA, ANCA, ENA, CRP, ESR, copper, and cryoglobulins are all negative, with positivity for IgG kappa paraprotein. There is no family history of polyneuropathy.	Chemotherapy-induced neuropathy	**Step-by-Step Analysis**<br>**1. Review of Clinical Information:**<br>● **Patient Profile:** 60-year-old male with subacute onset of pure sensory, symmetrical, length-dependent polyneuropathy after chemotherapy for multiple myeloma.<br>● **Key Symptoms:** Frequent cramps, paresthesia, neuropathic pain in the feet.<br>● **Exclusion of Symptoms:** No sensory ataxia, tremor, foot deformities, cranial nerve, or autonomic involvement.<br>● **Nerve Conduction Studies:** Pure sensory, symmetric, axonal polyneuropathy.<br>● **Lab Results:** Extensive screening negative, but positive for IgG kappa paraprotein.<br>● **Family History:** No history of polyneuropathy.<br>**2. Differential Diagnoses:**<br>● **Chemotherapy-Induced Peripheral Neuropathy (CIPN)**<br>● **Paraneoplastic Neuropathy**<br>● **MGUS-Related Neuropathy**<br>**Additional Examinations:**<br>**Laboratory Tests**<br>1. **Serum Protein Electrophoresis (SPEP) and Immunofixation Electrophoresis (IFE)**<br>○ **Reasoning:** To further characterize the IgG kappa paraprotein, which could help distinguish between MGUS and active multiple myeloma.<br>○ **Importance:** Determines the concentration and type of paraprotein, aiding in the diagnosis of MGUS or other monoclonal gammopathies.<br>2. **CSF Analysis (Cerebrospinal Fluid Analysis)**<br>○ **Reasoning:** To evaluate for paraneoplastic syndromes and infectious or inflammatory causes of neuropathy.<br>○ **Importance:** CSF analysis can reveal elevated protein levels, oligoclonal bands, or malignant cells, which are indicative of paraneoplastic processes or other underlying conditions.<br>3. **Anti-Hu, Anti-Ri, and Anti-Yo Antibodies**<br>○ **Reasoning:** These antibodies are often present in paraneoplastic neuropathies.<br>○ **Importance:** Their presence would strongly support a diagnosis of paraneoplastic neuropathy associated with the patient's multiple myeloma.<br>4. **Serum Free Light Chains (FLC) Assay**<br>○ **Reasoning:** To quantify free light chains, which are often elevated in multiple myeloma and can help assess disease activity.<br>○ **Importance:** Helps in distinguishing between MGUS and active myeloma, and can indicate the extent of disease burden.<br>**Instrumental Tests**<br>1. **MRI of the Spine**<br>○ **Reasoning:** To evaluate for spinal lesions or compressions that might contribute to neuropathic symptoms, particularly given the patient's history of multiple myeloma.<br>○ **Importance:** Detects structural abnormalities or metastases that might cause or exacerbate neuropathy.<br>2. **Skin Biopsy for Intraepidermal Nerve Fiber Density (IENFD)**

			○ **Reasoning:** Provides a direct measure of small nerve fiber density, useful in diagnosing small fiber neuropathy.<br>○ **Importance:** Confirms neuropathy and helps to characterize the type and extent of nerve fiber damage.<br>3. **Autonomic Function Testing**<br>○ **Reasoning:** Though the patient does not report autonomic symptoms, subclinical autonomic involvement can sometimes accompany sensory neuropathies.<br>○ **Importance:** Provides a comprehensive view of the extent of neuropathy.<br>**Most Crucial Test**<br>**Serum Protein Electrophoresis (SPEP) and Immunofixation Electrophoresis (IFE)**<br>● **Reasoning:** This test is critical to characterize the IgG kappa paraprotein found in the initial screening. It will help distinguish between MGUS, multiple myeloma, and other monoclonal gammopathies, providing crucial information for narrowing down the differential diagnosis.<br>● **Importance:** Identifying and quantifying the paraprotein is central to understanding the underlying pathology, particularly given the patient's history of multiple myeloma and the presence of an IgG kappa paraprotein. This will guide further management and additional testing if needed.<br>**Conclusion**<br>The most crucial test in this scenario is **Serum Protein Electrophoresis (SPEP) and Immunofixation Electrophoresis (IFE)**. This will provide essential information regarding the nature of the IgG kappa paraprotein, helping to confirm or rule out diagnoses such as MGUS, multiple myeloma, or related conditions.
85	A 63-year-old male patient presents with a 4-year history of sensory-motor, symmetrical, length dependent polyneuropathy with insidious onset and fast progression. The patient presents with severe distal motor weakness and hypoesthesia in the four limbs, dysphagia, dysphonia, and autonomic involvement with no foot deformities or tremor. Past medical history shows lung cancer (20 years ago) but he did not undergo chemotherapy. Nerve conduction studies show an axonal, sensory-motor polyneuropathy with bilateral carpal tunnel syndrome. Screening lab tests including vitamin B12, folate, vitamin B6, vitamin E, HBV, HCV, ANA, ANCA, ENA, glycaemia, HbA1c, CRP, ESR, copper, cryoglobulins, and serum electrophoresis are all negative. There is no family history of polyneuropathy.	TTR-Amyloidosis	**Step-by-Step Analysis of Additional Examinations:**<br>1. **Paraneoplastic Antibody Panel**:<br>○ **Reasoning**: Given the patient's history of lung cancer and the suspicion of paraneoplastic neuropathy, testing for paraneoplastic antibodies (e.g., anti-Hu, anti-Yo, anti-Ri, and others) is essential. These antibodies can help confirm the diagnosis of a paraneoplastic syndrome associated with neuropathy.<br>○ **Impact**: Identifying specific paraneoplastic antibodies would strongly support the diagnosis and help guide further management.<br>2. **Imaging Studies**:<br>○ **Chest CT Scan**:<br>■ **Reasoning**: To check for recurrence of lung cancer or presence of a new primary tumor that might be associated with paraneoplastic syndromes.<br>■ **Impact**: Detection of a new or recurrent malignancy would significantly support a diagnosis of paraneoplastic neuropathy and indicate the need for oncologic intervention.<br>○ **PET Scan**:<br>■ **Reasoning**: To detect any occult malignancies that might not be visible on a CT scan.<br>■ **Impact**: A PET scan can reveal metabolically active tumors, aiding in identifying the source of paraneoplastic syndrome.<br>3. **Cerebrospinal Fluid (CSF) Analysis**:<br>○ **Reasoning**: To check for elevated protein levels, presence of inflammatory cells, or oligoclonal bands which can indicate chronic inflammatory demyelinating polyradiculoneuropathy (CIDP) or other inflammatory neuropathies.<br>○ **Impact**: Abnormal CSF findings would support the diagnosis of CIDP or other inflammatory neuropathies, guiding treatment towards immunomodulatory therapies.<br>4. **Nerve Biopsy**:<br>○ **Reasoning**: To obtain a direct tissue diagnosis which can reveal amyloid deposits, vasculitis, or other specific pathological changes.<br>○ **Impact**: A positive finding for amyloid would confirm amyloidosis, while evidence of inflammation could suggest CIDP or vasculitic neuropathy.<br>5. **Fat Pad Aspirate or Rectal Biopsy**:<br>○ **Reasoning**: To check for amyloid deposits which could indicate amyloidosis.<br>○ **Impact**: Detecting amyloid deposits would confirm systemic amyloidosis, aiding in the diagnosis and management plan.<br>6. **Autonomic Function Testing**:<br>○ **Reasoning**: To assess the extent and specific nature of autonomic involvement, which can be seen in paraneoplastic syndromes, amyloidosis, and CIDP.<br>○ **Impact**: Detailed autonomic testing can help in evaluating the severity and guiding symptomatic treatment for autonomic dysfunction.<br>**Most Crucial Test:**<br>**Paraneoplastic Antibody Panel**<br>**Reasoning**: Given the patient's history of lung cancer and the clinical presentation suggestive of paraneoplastic syndrome, the paraneoplastic antibody panel is the most critical test. This panel can provide a definitive link between the patient's neuropathy and a potential paraneoplastic etiology, guiding both diagnostic confirmation and subsequent management. Detecting specific antibodies would significantly narrow down the differential diagnosis and direct further oncologic and neurologic care.
86	A 39-year-old female patient presents with a 34-year history of sensory-motor, symmetrical, length-dependent polyneuropathy with insidious onset and slowly progressive course. The patient reports paresthesia and neuropathic pain in the hands and feet, sensory ataxia, cramps, distal weakness in all four limbs, moderate calf atrophy, and pes cavus. Clinical examination does not reveal cranial nerve or autonomic involvement. Past medical history is unremarkable. Nerve conduction studies show an axonal, symmetric, length-dependent sensory-motor polyneuropathy. Screening lab tests including vitamin B12, folate, vitamin B6, vitamin E, HBV, HCV, glycemia, HbA1c, ANA, ANCA, ENA, CRP, ESR, copper,	CMT2	**Step-by-Step Analysis for Additional Examinations**<br>**1. Genetic Testing**<br>**Reasoning**:<br>● **Purpose**: To identify specific genetic mutations associated with hereditary neuropathies like Charcot-Marie-Tooth disease (CMT) and Hereditary Sensory and Autonomic Neuropathy (HSAN).<br>● **Specifics**: Testing for common mutations in CMT genes such as PMP22, MPZ, GJB1, and MFN2. Given the strong family history, genetic testing can confirm the diagnosis of CMT or other hereditary neuropathies.<br>● **Outcome**: Confirmation of a genetic mutation would definitively diagnose a hereditary neuropathy, particularly CMT.<br>**2. Nerve Biopsy**<br>**Reasoning**:<br>● **Purpose**: To examine the structure of peripheral nerves and identify characteristic changes associated with specific neuropathies, such as amyloid deposits in hereditary amyloidosis.<br>● **Specifics**: Sural nerve biopsy can provide histopathological evidence of amyloidosis or other nerve pathologies.<br>● **Outcome**: The presence of amyloid deposits would support a diagnosis of hereditary amyloidosis, while characteristic changes in myelin or axons could support CMT.<br>**3. Skin Biopsy for Intraepidermal Nerve Fiber Density (IENFD)**<br>**Reasoning**:<br>● **Purpose**: To assess small fiber neuropathy which might be overlooked in standard nerve conduction studies.<br>● **Specifics**: Quantifying nerve fiber density in a skin biopsy can help diagnose small fiber neuropathies that might be part of HSAN or amyloidosis.<br>● **Outcome**: Reduced nerve fiber density would suggest a small fiber neuropathy component, which is seen in HSAN and hereditary amyloidosis.<br>**4. Serum Protein Electrophoresis and Immunofixation Electrophoresis**<br>**Reasoning**:

	cryoglobulins, and serum electrophoresis are all negative. Her father is wheelchair-bound, and her brother and sister have walking difficulties.		• **Purpose**: To detect monoclonal gammopathy, which can be associated with neuropathies and amyloidosis.<br>• **Specifics**: These tests can identify abnormal proteins that might indicate a plasma cell dyscrasia or amyloidosis.<br>• **Outcome**: Identification of monoclonal proteins would suggest the need to consider amyloidosis or related conditions.<br>**5. Autonomic Function Tests**<br>**Reasoning**:<br>• **Purpose**: To detect any subtle autonomic involvement that might support a diagnosis of HSAN or amyloidosis.<br>• **Specifics**: Tests like heart rate variability, blood pressure response to tilt, and sudomotor testing.<br>• **Outcome**: Any abnormalities would indicate autonomic involvement, which is more typical of HSAN or amyloidosis than CMT.<br>**6. MRI of the Spine**<br>**Reasoning**:<br>• **Purpose**: To rule out any structural causes of neuropathy and to look for evidence of nerve root or plexus involvement.<br>• **Specifics**: Detailed imaging of the spine and peripheral nerves.<br>• **Outcome**: Excludes compressive or structural lesions that could mimic polyneuropathy.<br>**Most Crucial Test**<br>**Genetic Testing** is the most crucial test in this scenario. Given the strong family history and clinical presentation consistent with a hereditary neuropathy, identifying a specific genetic mutation can provide a definitive diagnosis. Genetic testing will directly address the most likely diagnosis (CMT) and differentiate it from other hereditary neuropathies like HSAN and hereditary amyloidosis. This will guide targeted management and genetic counseling for the patient and her family.<br>**Conclusion**<br>While a range of tests can help narrow down the differential diagnoses, genetic testing stands out as the most critical examination due to its ability to provide a definitive diagnosis in hereditary neuropathies, which aligns closely with the patient's clinical and family history.
87	A 50-year-old male patient presents with a 1-month history of pure motor multineuropathy with acute onset while hospitalized in ICU for sepsis. Clinical examination reveals the presence of dysphagia, dysphonia, hand tremor, and upper limb weakness in a multifocal distribution. There are no sensory deficits, nor neuropathic pain, tremor or foot deformities. Past medical history is positive for testicular cancer, for which the patient underwent chemotherapy three years ago. Nerve conduction studies show pure motor, axonal multineuropathy with one motor conduction block. Screening lab tests including vitamin B12, folate, vitamin B6, vitamin E, HCV, HBV, ANA, ANCA, ENA, CRP, ESR, glycemia, HbA1c, copper, cryoglobulins and serum electrophoresis are all negative. There is no family history of polyneuropathy.	GBS	**Step-by-Step Analysis of Additional Examinations**<br>1. **Repeat Nerve Conduction Studies (NCS) and Electromyography (EMG)**<br>○ **Reasoning:**<br>■ To confirm the initial findings of pure motor, axonal multineuropathy and to assess the progression or any changes in the pattern of neuropathy.<br>■ EMG can provide additional information on the presence of active denervation and reinnervation, which helps to understand the severity and chronicity of the neuropathy.<br>2. **Lumbar Puncture (CSF Analysis)**<br>○ **Reasoning:**<br>■ To check for elevated protein levels without pleocytosis, which can be indicative of an inflammatory neuropathy such as Guillain-Barré syndrome (GBS) or its variants, such as acute motor axonal neuropathy (AMAN).<br>■ This can help rule out infections or paraneoplastic syndromes that may present with similar symptoms.<br>3. **Paraneoplastic Panel**<br>○ **Reasoning:**<br>■ Given the history of testicular cancer, it is essential to check for paraneoplastic antibodies (e.g., anti-Hu, anti-Ri, anti-Yo, anti-Ma2) that can cause paraneoplastic neurological syndromes.<br>■ These antibodies can sometimes provide a direct link between the cancer and the neuropathy.<br>4. **Onconeural Antibodies Test**<br>○ **Reasoning:**<br>■ To further investigate the possibility of a paraneoplastic syndrome. Onconeural antibodies can help identify a hidden or recurrent malignancy that might not be detected through standard imaging.<br>5. **Serum and Urine Immunofixation Electrophoresis**<br>○ **Reasoning:**<br>■ To rule out monoclonal gammopathy, which can be associated with paraproteinemic neuropathy.<br>■ Important in ruling out conditions like POEMS syndrome (Polyneuropathy, Organomegaly, Endocrinopathy, Monoclonal gammopathy, Skin changes).<br>6. **Imaging Studies: CT or PET Scan**<br>○ **Reasoning:**<br>■ To search for any occult malignancy given the history of testicular cancer.<br>■ CT of the chest, abdomen, and pelvis, or a PET scan can help detect small tumors that might be missed on routine examination.<br>7. **Autonomic Function Tests**<br>○ **Reasoning:**<br>■ Though the patient presents with pure motor symptoms, autonomic testing can help identify subclinical autonomic involvement, which might support a diagnosis of paraneoplastic or other systemic neuropathies.<br>8. **Anti-GM1 Antibodies Test**<br>○ **Reasoning:**<br>■ Anti-GM1 antibodies are often found in multifocal motor neuropathy (MMN) and other motor neuropathies.<br>■ Their presence can help differentiate MMN from other causes of motor neuropathy.<br>9. **Creatine Kinase (CK) Levels**<br>○ **Reasoning:**<br>■ To rule out muscle disease contributing to weakness.<br>■ Elevated CK levels can indicate muscle breakdown or a myopathic process.<br>10. **Thyroid Function Tests (TFTs)**<br>○ **Reasoning:**<br>■ To exclude thyroid dysfunction as a contributing factor, as thyroid diseases can sometimes present with neuropathic symptoms.<br>**Conclusion: Most Crucial Test**<br>**Lumbar Puncture (CSF Analysis):**<br>• **Reasoning:**

			○ This test is crucial as it provides direct insight into any inflammatory processes that may be affecting the peripheral nerves. Elevated protein levels without pleocytosis in the CSF would support a diagnosis of Guillain-Barré syndrome (GBS) or its variants, which is highly relevant given the patient's acute onset of motor symptoms in the context of recent sepsis.<br>○ Additionally, CSF analysis can help rule out infectious causes and contribute to the diagnosis of paraneoplastic syndromes if cancer cells or specific paraneoplastic antibodies are present in the CSF.<br>Therefore, the lumbar puncture (CSF analysis) is the most crucial test to conduct next, as it can significantly narrow down the differential diagnoses and provide critical information regarding the underlying pathology of the patient's neuropathy.
88	A 58-year-old male patient presents with a 15-month history of sensory-motor, symmetrical, length-dependent polyneuropathy with insidious onset and progressive course. The patient reports distal paresthesia, numbness, neuropathic pain in the lower limbs in a symmetric distribution, and sensory ataxia. Clinical examination confirms the presence of sensory ataxia, postural hand tremor, and mild weakness in ankle dorsiflexion. There are no foot deformities, cranial nerve involvement, or autonomic dysfunction. Past medical history is positive for IgM kappa MGUS and type 2 diabetes. Nerve conduction studies show a demyelinating, length-dependent, symmetric, sensory-motor polyneuropathy. Screening lab tests including vitamin B12, folate, vitamin B6, vitamin E, HCV, HBV, ANA, ANCA, ENA, CRP, ESR, glycemia, copper, and cryoglobulins are all negative, with evidence of IgM kappa paraprotein and elevated HbA1c levels. There is no family history of polyneuropathy.	Anti-MAG polyneuropathy	**Step-by-Step Analysis and Additional Examinations**<br>Given the detailed clinical scenario, the following additional examinations are necessary to narrow down the differential diagnoses of polyneuropathy:<br>1. **Serum and Urine Protein Electrophoresis and Immunofixation**<br>○ **Reasoning**:<br>■ This test is essential to further characterize the IgM kappa paraprotein detected in the patient. It will help determine the extent and type of monoclonal gammopathy and rule out multiple myeloma or Waldenström's macroglobulinemia, which could present with similar neuropathic symptoms.<br>2. **Lumbar Puncture (CSF Analysis)**<br>○ **Reasoning**:<br>■ Analyzing cerebrospinal fluid (CSF) can help detect elevated protein levels without pleocytosis, which is suggestive of CIDP.<br>■ CSF analysis can also help rule out infections and other inflammatory conditions that might present with similar neuropathic symptoms.<br>3. **Skin Biopsy for Intraepidermal Nerve Fiber Density (IENFD)**<br>○ **Reasoning**:<br>■ This test is useful in diagnosing small fiber neuropathy, which might not be evident on nerve conduction studies. Given the patient's symptoms of distal paresthesia and neuropathic pain, it's essential to assess the integrity of small nerve fibers.<br>4. **Anti-GM1 and Anti-MAG Antibodies**<br>○ **Reasoning**:<br>■ These antibodies are often associated with demyelinating neuropathies and can help diagnose specific subtypes of neuropathy linked to monoclonal gammopathies.<br>■ Testing for these antibodies can further clarify whether the patient's neuropathy is related to MGUS.<br>5. **Nerve Biopsy**<br>○ **Reasoning**:<br>■ A nerve biopsy can provide histological evidence of demyelination, axonal degeneration, and inflammatory infiltrates, which are crucial for diagnosing conditions like CIDP or vasculitic neuropathy.<br>■ It can also help identify amyloid deposits if amyloidosis is suspected.<br>6. **MRI of the Lumbosacral Plexus**<br>○ **Reasoning**:<br>■ MRI can help visualize nerve root and plexus involvement, which can be indicative of CIDP or other inflammatory neuropathies.<br>■ It can also rule out structural causes of neuropathy.<br>7. **Glucose Tolerance Test**<br>○ **Reasoning**:<br>■ Given the history of type 2 diabetes and elevated HbA1c, assessing the glucose tolerance status can provide more information about the severity of diabetes and its contribution to neuropathy.<br>8. **Serum Free Light Chains**<br>○ **Reasoning**:<br>■ This test helps in detecting light chain multiple myeloma or amyloidosis, which can present with polyneuropathy.<br>■ It can also quantify the burden of monoclonal protein production.<br>9. **Quantitative Sensory Testing (QST)**<br>○ **Reasoning**:<br>■ QST evaluates the function of small and large nerve fibers, which can help in differentiating between various types of neuropathies, especially when nerve conduction studies are inconclusive for small fiber involvement.<br>10. **Retinal Examination**<br>○ **Reasoning**:<br>■ Given the history of diabetes, checking for diabetic retinopathy can provide insights into the extent of microvascular complications, which might correlate with the severity of diabetic neuropathy.<br>**Most Crucial Test**<br>**Serum and Urine Protein Electrophoresis and Immunofixation**<br>● **Reasoning**:<br>○ This test is pivotal because it will help to characterize the monoclonal protein more precisely. The presence of IgM kappa paraprotein needs to be further investigated to rule out conditions like multiple myeloma or Waldenström's macroglobulinemia, which can present with neuropathy. Confirming or ruling out these diagnoses is crucial, as it will significantly influence the management and treatment approach for the patient's polyneuropathy.<br>In summary, while all the listed tests are important, the most crucial test for this patient is serum and urine protein electrophoresis and immunofixation. This will provide critical information about the nature of the monoclonal gammopathy and its potential link to the patient's neuropathy.
89	A 73-year-old male patient presents with a 2-year history of sensory-motor, symmetrical, non-length dependent polyneuropathy with subacute onset. The patient presents with paresthesia and neuropathic pain in a stocking and glove distribution, proximal and distal weakness in the lower limbs, mild sensory ataxia and cramps. There are no foot deformities, tremor, cranial nerve or autonomic	POEMS	**Step-by-Step Analysis and Additional Examinations**<br>**Step 1: Review of Current Data**<br>● Symmetrical, sensory-motor, non-length dependent polyneuropathy with subacute onset<br>● Mixed demyelinating-axonal features on nerve conduction studies<br>● Presence of IgG lambda monoclonal gammopathy<br>● Negative screening lab tests (vitamins, infections, autoimmune markers)<br>● Clinical symptoms (paresthesia, neuropathic pain, proximal and distal weakness, mild sensory ataxia, cramps)<br>● Physical exam finding of cutaneous hyperpigmentation<br>**Step 2: Identify Additional Examinations**<br>1. **Serum Immunofixation Electrophoresis (IFE) and Free Light Chain Assay:**

	involvement. Clinical examination reveals cutaneous hyperpigmentation. Past medical history is positive for IgG lambda monoclonal gammopathy. Nerve conduction studies show a mixed demyelinating-axonal, sensory-motor, symmetric polyneuropathy. Screening lab tests including vitamin B12, folate, vitamin B6, vitamin E, HCV, HBV, ANA, ANCA, ENA, CRP, ESR, glycemia, HbA1c, copper and cryoglobulins are all negative, with positivity for IgG lambda paraprotein. There is no family history of polyneuropathy.		○ **Reasoning:** To further characterize the monoclonal protein and quantify the levels of free light chains, which can help distinguish between MGUS, multiple myeloma, or other plasma cell dyscrasias.<br>○ **Utility:** Confirms the type and burden of monoclonal protein, critical for diagnosing MGUS or other related conditions.<br>2. **Bone Marrow Biopsy:**<br>○ **Reasoning:** To evaluate the bone marrow for plasma cell infiltration, which can help differentiate MGUS from multiple myeloma or POEMS syndrome.<br>○ **Utility:** Identifies clonal plasma cell population and helps in staging and diagnosis of plasma cell disorders.<br>3. **Electromyography (EMG):**<br>○ **Reasoning:** To assess the electrical activity of muscles and further delineate the extent and pattern of neuropathy.<br>○ **Utility:** Provides additional information on muscle involvement, which is useful in the diagnosis of CIDP or other neuromuscular disorders.<br>4. **Lumbar Puncture with Cerebrospinal Fluid (CSF) Analysis:**<br>○ **Reasoning:** To look for elevated protein levels and exclude infectious or inflammatory causes of polyneuropathy.<br>○ **Utility:** Elevated CSF protein without pleocytosis supports the diagnosis of CIDP.<br>5. **CT or MRI of Chest, Abdomen, and Pelvis:**<br>○ **Reasoning:** To check for organomegaly, lymphadenopathy, or other malignancies that could be part of POEMS syndrome.<br>○ **Utility:** Identifies systemic involvement typical of POEMS syndrome.<br>6. **Skin Biopsy:**<br>○ **Reasoning:** To investigate the cause of cutaneous hyperpigmentation and check for amyloid deposits.<br>○ **Utility:** Helps to diagnose systemic amyloidosis, which can be associated with monoclonal gammopathies.<br>7. **Testing for Anti-MAG Antibodies:**<br>○ **Reasoning:** Anti-myelin-associated glycoprotein (MAG) antibodies are associated with certain types of paraproteinemic neuropathies.<br>○ **Utility:** Helps identify specific autoimmune neuropathies related to monoclonal gammopathies.<br>8. **Endocrine Workup:**<br>○ **Reasoning:** To screen for endocrinopathies (thyroid function tests, adrenal function tests, etc.), which can be part of POEMS syndrome.<br>○ **Utility:** Identifies hormonal abnormalities that can contribute to neuropathy.<br>**Step 3: Determine the Most Crucial Test**<br>Based on the case specifics, the **most crucial test** would be **Bone Marrow Biopsy**.<br>**Reasoning:**<br>● A bone marrow biopsy will provide definitive information on the presence and extent of plasma cell infiltration, which is crucial for distinguishing MGUS from multiple myeloma and POEMS syndrome.<br>● It offers direct insight into the hematological condition underlying the patient's symptoms, especially in the context of a known monoclonal gammopathy.<br>● It will help in the identification of POEMS syndrome if there is significant plasma cell infiltration and the presence of other features like angiogenesis.<br>**Conclusion:**<br>While all additional tests are important for a comprehensive assessment, a bone marrow biopsy stands out as the key diagnostic tool to narrow down the differential diagnoses and guide further management in this patient with polyneuropathy and monoclonal gammopathy.
90	A 64-year-old male patient presents with a predominantly motor, asymmetrical, non-length dependent polyneuropathy with acute onset that has progressed in the past three months. The patient also reports the presence of neuropathic pain and postural hand tremor. Clinical examination does not reveal ataxia, foot deformities, cranial nerve or autonomic involvement. Past medical history is positive for diabetes. Nerve conduction studies show demyelinating, pure motor polyneuropathy with conduction blocks. Screening lab tests including vitamin B12, folate, vitamin B6, vitamin E, HBV, HCV, ANA, ANCA, ENA, CRP, ESR, glycemia, HbA1c, copper, and cryoglobulins are all negative. Serum electrophoresis reveals the presence of IgM monoclonal gammopathy. There is no family history of polyneuropathy.	Acute onset CIDP	**Step-by-Step Analysis and Additional Examinations**<br>Given the comprehensive data from the clinical scenario, the next step involves identifying additional examinations to further narrow down the differential diagnoses.<br>**Additional Examinations and Their Reasoning**<br>1. **Serum Anti-GM1 Antibodies**<br>○ **Reasoning**: Anti-GM1 antibodies are commonly associated with Multifocal Motor Neuropathy (MMN). Testing for these antibodies can provide strong supportive evidence for MMN if present.<br>2. **Cerebrospinal Fluid (CSF) Analysis**<br>○ **Reasoning**: CSF analysis can help distinguish between different types of polyneuropathies. Elevated protein levels without pleocytosis (albuminocytologic dissociation) can be seen in CIDP. Paraneoplastic syndromes may show different CSF characteristics.<br>3. **MRI of the Brachial Plexus and Spine**<br>○ **Reasoning**: MRI can identify structural causes of nerve compression or inflammation. In MMN, MRI might show enhancement of the brachial plexus. It can also rule out other causes such as tumors or significant inflammatory processes.<br>4. **PET-CT Scan**<br>○ **Reasoning**: This imaging can help in identifying any underlying malignancies that could be causing a paraneoplastic neuropathy. The presence of an IgM monoclonal gammopathy warrants a thorough search for potential malignancies.<br>5. **Electromyography (EMG)**<br>○ **Reasoning**: EMG can provide detailed information on muscle activity and help distinguish between neuropathic and myopathic processes. It can also help in confirming the diagnosis of MMN by showing motor neuron involvement.<br>6. **Bone Marrow Biopsy**<br>○ **Reasoning**: This test can help determine if there is a hematologic malignancy or disorder associated with the monoclonal gammopathy, such as Waldenström's macroglobulinemia or multiple myeloma.<br>7. **Paraneoplastic Antibody Panel**<br>○ **Reasoning**: Testing for paraneoplastic antibodies can help identify a paraneoplastic syndrome. This can be critical if there is suspicion of an occult malignancy associated with the neuropathy.<br>8. **Repeat Serum Immunofixation Electrophoresis**<br>○ **Reasoning**: To further characterize the IgM monoclonal gammopathy and monitor for changes over time, which can provide clues towards a diagnosis of a plasma cell dyscrasia.<br>9. **Skin Biopsy**<br>○ **Reasoning**: In some cases, a skin biopsy can provide information about small fiber neuropathies, though it's less relevant in predominantly motor neuropathies.<br>10. **Genetic Testing for Hereditary Neuropathies**<br>○ **Reasoning**: Despite no family history, genetic testing can rule out hereditary causes of neuropathy, which sometimes present atypically in older age.<br>**Determining the Most Crucial Test**<br>Among the listed tests, the most crucial initial test to conduct would be **Serum Anti-GM1 Antibodies**.<br>**Reasoning**:<br>● **Specificity for MMN**: Anti-GM1 antibodies are highly specific for MMN, a condition strongly suspected given the patient's predominantly motor symptoms, asymmetrical involvement, and nerve conduction study findings.

			● **Non-Invasiveness**: This is a relatively non-invasive blood test compared to procedures like a bone marrow biopsy or lumbar puncture.<br>● **Immediate Diagnostic Clarity**: A positive result would provide immediate strong supportive evidence for MMN, guiding further management and treatment.<br>This test will significantly help in confirming the most likely diagnosis of MMN or necessitating the need for further testing if the results are negative.
91	A 78-year-old male patient presents with a 1-year history of a sensory-motor, symmetrical, length dependent polyneuropathy with insidious onset and progressive course. The patient presents with sensory ataxia, hand tremor, weakness of ankle dorsiflexion, and distal leg paraesthesia. He also reports mild neuropathic pain in the feet. Clinical examination does not reveal foot deformities, autonomic or cranial nerve involvement. Past medical history is positive for type 2 diabetes, IgM kappa monoclonal gammopathy, and Waldenström macroglobulinemia. Nerve conduction studies show a demyelinating, sensory-motor, length dependent polyneuropathy. Screening lab tests shows hyperglycaemia, elevated HbA1c, and IgM monoclonal gammopathy. Remaining exams including vitamin B12, folate, vitamin B6, vitamin E, HCV, ANA, ENA, ANCA, ENA, CRP, copper, and cryoglobulins are negative. There is no family history of polyneuropathy.	Anti-MAG neuropathy	**Step-by-Step Analysis and Additional Examinations**<br>**Step 1: Analyze Comprehensive Data**<br>● **Patient Profile:** 78-year-old male<br>● **Symptoms:** Sensory-motor, symmetrical, length-dependent polyneuropathy (sensory ataxia, hand tremor, weakness of ankle dorsiflexion, distal leg paraesthesia, mild neuropathic pain in the feet)<br>● **Clinical Examination:** No foot deformities, autonomic or cranial nerve involvement<br>● **Past Medical History:** Type 2 diabetes, IgM kappa monoclonal gammopathy, Waldenström macroglobulinemia<br>● **Nerve Conduction Studies:** Demyelinating, sensory-motor, length-dependent polyneuropathy<br>● **Lab Results:** Hyperglycaemia, elevated HbA1c, IgM monoclonal gammopathy; negative for vitamin B12, folate, vitamin B6, vitamin E, HCV, ANA, ENA, ANCA, CRP, copper, and cryoglobulins<br>● **Family History:** No polyneuropathy<br>**Step 2: Identify Necessary Additional Examinations**<br>1. **Serum Protein Electrophoresis (SPEP) and Immunofixation Electrophoresis (IFE)**<br>○ **Reasoning:**<br>■ To further characterize the monoclonal gammopathy and quantify the monoclonal protein.<br>■ Differentiates between monoclonal gammopathy of undetermined significance (MGUS) and more severe conditions like Waldenström macroglobulinemia.<br>■ Important in assessing the extent of the monoclonal protein's impact on neuropathy.<br>2. **Quantitative Immunoglobulins (IgG, IgA, IgM)**<br>○ **Reasoning:**<br>■ Helps in understanding the overall immune profile and burden of monoclonal protein.<br>■ Determines if the patient has immunodeficiency or hypergammaglobulinemia which can be associated with polyneuropathy.<br>3. **Anti-MAG Antibodies (Myelin-Associated Glycoprotein)**<br>○ **Reasoning:**<br>■ High prevalence in patients with IgM monoclonal gammopathies.<br>■ Presence of anti-MAG antibodies would support the diagnosis of neuropathy associated with monoclonal gammopathy.<br>■ Important in distinguishing between different causes of demyelinating polyneuropathies.<br>4. **Lumbar Puncture and Cerebrospinal Fluid (CSF) Analysis**<br>○ **Reasoning:**<br>■ To evaluate for albuminocytologic dissociation which is suggestive of CIDP.<br>■ Helps to rule out other inflammatory or infectious causes of neuropathy.<br>■ An increased protein level without a significant increase in white blood cells supports CIDP.<br>5. **Skin Biopsy**<br>○ **Reasoning:**<br>■ To assess small fiber neuropathy which might not be detected in nerve conduction studies.<br>■ Useful in confirming the presence of length-dependent polyneuropathy.<br>6. **Repeat Nerve Conduction Studies with EMG (Electromyography)**<br>○ **Reasoning:**<br>■ To confirm the findings of demyelinating neuropathy and further characterize the severity and distribution.<br>■ Helps to track the progression of neuropathy and evaluate the involvement of additional nerves.<br>7. **Glucose Tolerance Test (GTT)**<br>○ **Reasoning:**<br>■ Provides a more detailed assessment of glucose metabolism and insulin sensitivity.<br>■ Useful in confirming the impact of diabetes on neuropathy.<br>8. **Serum Free Light Chains (sFLC) Assay**<br>○ **Reasoning:**<br>■ To evaluate the presence and levels of free light chains in the blood.<br>■ Helps in diagnosing and monitoring conditions like light chain amyloidosis which can also present with neuropathy.<br>9. **Whole Body Imaging (CT or PET-CT Scan)**<br>○ **Reasoning:**<br>■ To assess for systemic involvement or progression of Waldenström macroglobulinemia or other malignancies.<br>■ Important in staging and determining the extent of the disease.<br>**Step 3: Determine the Most Crucial Test**<br>**Anti-MAG Antibodies**<br>**Reasoning:**<br>● Given the patient's history of IgM monoclonal gammopathy and Waldenström macroglobulinemia, the presence of anti-MAG antibodies would strongly indicate that the neuropathy is associated with the monoclonal gammopathy.<br>● This test directly addresses the differential diagnosis between diabetic neuropathy and neuropathy associated with monoclonal gammopathy, potentially narrowing down the most likely cause of the patient's symptoms.<br>**Conclusion**<br>Conducting an anti-MAG antibodies test is the most crucial additional examination as it will provide significant insight into whether the patient's neuropathy is primarily due to the monoclonal gammopathy, which is essential for accurate diagnosis and management.
92	A 58-year-old female patient presents with a 3-month history of sensory-motor,	Acute onset CIDP	**Step-by-Step Analysis of Comprehensive Data**<br>**Clinical Summary Recap:**

	symmetrical, non-length dependent polyneuropathy with acute onset and progressive course. Two weeks after an infectious event, the patient reports the appearance of paraesthesia and weakness in the hands and lower limbs. At nadir she was unable to walk. The symptoms were interpreted as Guillain Barré syndrome and the patient received a course of immunoglobulin therapy with clear benefit. After the therapy she started walking again with some difficulty. About three months after the onset of symptoms, she noticed the reappearance of the paresthesia and a worsening of her strength, so she started using a crutch. Neurological examination shows the presence of weakness, distributed proximally and distally in the lower limbs and distally in the upper limbs, and sensory ataxia. Past medical history is silent. Nerve conduction studies show a demyelinating, sensory-motor polyneuropathy. Screening lab tests including vitamin B12, folate, vitamin B6, vitamin E, HBV, HCV, ANA, ANCA, ENA, CRP, ESR, glycemia, HbA1c, copper, cryoglobulins, and serum electrophoresis are negative. There is no family history of polyneuropathy.		● 58-year-old female with 3-month history of sensory-motor, symmetrical, non-length dependent polyneuropathy<br>● Acute onset after infectious event, progressive course<br>● Initial symptoms: paraesthesia and weakness in hands and lower limbs<br>● Improvement with immunoglobulin therapy for presumed Guillain Barré syndrome<br>● Recurrent symptoms after three months, requiring crutch for ambulation<br>● Neurological exam: weakness (proximal and distal in lower limbs, distal in upper limbs), sensory ataxia<br>● Nerve conduction studies: demyelinating, sensory-motor polyneuropathy<br>● Negative screening labs (vitamins B12, B6, E, HBV, HCV, ANA, ANCA, ENA, CRP, ESR, glycemia, HbA1c, copper, cryoglobulins, serum electrophoresis)<br>● No family history of polyneuropathy<br>**Additional Examinations**<br>**1. Cerebrospinal Fluid (CSF) Analysis**<br>● **Reasoning:**<br>○ CSF analysis is critical in distinguishing between various neuropathies. CIDP often shows elevated CSF protein with normal cell count (albuminocytologic dissociation), which is not typically seen in other neuropathies.<br>○ **Specific Tests:**<br>■ CSF protein<br>■ CSF cell count<br>■ Oligoclonal bands<br>**2. Repeat Nerve Conduction Studies and Electromyography (EMG)**<br>● **Reasoning:**<br>○ Detailed electrophysiological studies can help in confirming the diagnosis of CIDP by demonstrating demyelination patterns such as prolonged distal latencies, conduction blocks, and slowed nerve conduction velocities.<br>○ **Specific Tests:**<br>■ Detailed motor and sensory nerve conduction studies<br>■ EMG to assess for axonal involvement<br>**3. Magnetic Resonance Imaging (MRI) of the Spine**<br>● **Reasoning:**<br>○ MRI with gadolinium contrast can detect nerve root or plexus inflammation or hypertrophy, which is suggestive of CIDP.<br>○ **Specific Tests:**<br>■ MRI of the lumbar spine<br>■ MRI of the cervical spine<br>**4. Anti-GM1 and Anti-GQ1b Antibodies**<br>● **Reasoning:**<br>○ These antibodies can help in diagnosing autoimmune neuropathies like multifocal motor neuropathy (anti-GM1) and Miller Fisher syndrome (anti-GQ1b), which can present with similar clinical features.<br>○ **Specific Tests:**<br>■ Anti-GM1 antibody<br>■ Anti-GQ1b antibody<br>**5. Autoimmune and Paraneoplastic Panel**<br>● **Reasoning:**<br>○ Although initial autoimmune screenings were negative, comprehensive panels for less common antibodies (e.g., anti-MAG, anti-Hu, anti-Yo, anti-Ri) can help detect paraneoplastic syndromes or rare autoimmune neuropathies.<br>○ **Specific Tests:**<br>■ Anti-MAG antibody<br>■ Paraneoplastic antibody panel (anti-Hu, anti-Yo, anti-Ri, etc.)<br>**6. Infectious Disease Panel**<br>● **Reasoning:**<br>○ Given the onset after an infectious event, it is essential to rule out infections that can cause neuropathy such as HIV, Lyme disease, and syphilis.<br>○ **Specific Tests:**<br>■ HIV antibody and viral load<br>■ Lyme serology<br>■ VDRL/RPR for syphilis<br>**Most Crucial Test**<br>**Cerebrospinal Fluid (CSF) Analysis**<br>● **Reasoning:**<br>○ CSF analysis is the most critical test because it can provide direct evidence supporting the diagnosis of CIDP (elevated protein with normal cell count). This finding, known as albuminocytologic dissociation, is highly characteristic of CIDP and helps distinguish it from other causes of polyneuropathy.<br>○ Additionally, CSF analysis can help rule out other infectious or inflammatory causes of neuropathy, providing further diagnostic clarity.<br>Thus, while several tests are valuable in this diagnostic workup, CSF analysis is paramount for confirming CIDP and excluding other differential diagnoses.
93	A 42-year-old female patient presents with a 6-month history of chronic, sensory-motor, symmetrical, non-length dependent polyneuropathy with subacute onset. The patient presents with paresthesia and severe neuropathic pain in the four limbs in a stocking and glove distribution and	Chemotherapy-induced neuropathy	**Step-by-Step Analysis and Additional Examinations**<br>1. **Comprehensive Neurological Examination**<br>○ **Reasoning**:<br>■ To thoroughly assess the extent and distribution of the neuropathy.<br>■ To document the progression and severity of sensory and motor deficits.<br>■ To evaluate for any subtle signs of cranial nerve or autonomic involvement that may have been missed initially.<br>○ **Specifics**: Detailed sensory examination (pinprick, vibration, proprioception), motor strength testing, reflexes, and coordination tests.

	sensory ataxia. Clinical examination reveals the presence of postural hand tremor, which the patient reports being present for years. Thereare no foot deformities, cranial nerve or autonomic involvement. Past medical history is positive for rectal cancer, for which the patient has undergone chemotherapy for three years. Neuropathy symptoms started during chemotherapy. Nerve conduction studies show sensory-motor, symmetric, axonal polyneuropathy. Screening lab tests including vitamin B6, vitamin E, HBV, HCV, ANA, ANCA, ENA, CRP, ESR, glycemia, HbA1c, copper, cryoglobulins and serum electrophoresis are all negative, with evidence of vitamin B12 deficiency. There is no family history of polyneuropathy.		2. **Electromyography (EMG) and Repeat Nerve Conduction Studies (NCS)**<br>○ **Reasoning**:<br>■ To further characterize the type and distribution of the neuropathy.<br>■ To differentiate between axonal and demyelinating processes more precisely.<br>■ To assess for any signs of muscle denervation which might suggest a more severe or different type of neuropathy.<br>○ **Specifics**: EMG to detect muscle activity and NCS to measure the speed and strength of electrical signals in nerves.<br>3. **Lumbar Puncture and Cerebrospinal Fluid (CSF) Analysis**<br>○ **Reasoning**:<br>■ To rule out inflammatory, infectious, or paraneoplastic causes of neuropathy.<br>■ To check for elevated protein levels which may indicate Guillain-Barré syndrome or chronic inflammatory demyelinating polyneuropathy (CIDP).<br>■ To identify oligoclonal bands that may suggest an autoimmune etiology.<br>○ **Specifics**: CSF analysis for cell count, protein, glucose, and oligoclonal bands.<br>4. **Repeat Blood Tests and Specific Serologies**<br>○ **Reasoning**:<br>■ To rule out other metabolic, infectious, or autoimmune causes not covered in initial screening.<br>■ To check for antibodies associated with paraneoplastic syndromes or autoimmune neuropathies.<br>○ **Specifics**:<br>■ Repeat vitamin B12, homocysteine, and methylmalonic acid levels.<br>■ Antibodies against neuronal antigens (e.g., anti-Hu, anti-Yo, anti-Ri).<br>■ Further viral serologies (e.g., HIV, Lyme disease).<br>5. **Magnetic Resonance Imaging (MRI) of the Brain and Spinal Cord**<br>○ **Reasoning**:<br>■ To assess for any central nervous system involvement which might suggest a different or concurrent neurological condition.<br>■ To rule out structural lesions, inflammation, or demyelinating diseases affecting the central nervous system.<br>○ **Specifics**: MRI with and without contrast of the brain and entire spine.<br>6. **Skin Biopsy**<br>○ **Reasoning**:<br>■ To evaluate small fiber neuropathy, which might not be detected on standard nerve conduction studies.<br>■ To assess for loss of intraepidermal nerve fibers which can be indicative of certain types of neuropathy, such as those caused by diabetes or chemotherapy.<br>○ **Specifics**: Punch biopsy of the skin to examine intraepidermal nerve fiber density.<br>**Most Crucial Test**<br>Given the patient's clinical scenario, **Electromyography (EMG) and Repeat Nerve Conduction Studies (NCS)** are the most crucial tests.<br>**Reasoning**:<br>● These tests will provide detailed information about the nature (axonal vs. demyelinating), distribution, and severity of the neuropathy.<br>● They will help differentiate between potential causes (e.g., chemotherapy-induced neuropathy vs. paraneoplastic vs. vitamin deficiency-related).<br>● Repeating these tests can track the progression of the neuropathy and evaluate the effectiveness of any interventions (e.g., supplementation for vitamin B12 deficiency).<br>● They offer the most direct insight into the function of the peripheral nerves and can guide further diagnostic and therapeutic steps.
94	A 73-year-old female patient presents with a 1-year history of pure sensory, symmetrical, length dependent polyneuropathy with insidious onset. The patient presents with paresthesia, neuropathic pain and sensory ataxia without weakness, cranial nerve or autonomic involvement. There are also no foot deformities or tremor. Past medical history is positive for sarcoidosis. Nerve conduction studies show pure a pure sensory, axonal polyneuropathy. Screening lab tests including vitamin B12, folate, vitamin B6, vitamin E, HCV, HBV, ANCA, ENA, CRP, ESR, glycemia, HbA1c, copper, cryoglobulins and serum electrophoresis are all negative, with positivity for ANA autoantibodies. There is no family history of polyneuropathy.	Sarcoidosis associated polyneuropathy	**Step-by-Step Analysis and Additional Examinations**<br>**1. Comprehensive Data Review**<br>● **Patient Information:** 73-year-old female<br>● **Chief Complaint:** 1-year history of pure sensory, symmetrical, length-dependent polyneuropathy<br>● **Symptoms:** Paresthesia, neuropathic pain, sensory ataxia, no weakness, cranial nerve involvement, or autonomic involvement<br>● **Physical Exam:** No foot deformities or tremor<br>● **Medical History:** Sarcoidosis<br>● **Diagnostic Findings:**<br>○ Nerve conduction studies: Pure sensory, axonal polyneuropathy<br>○ Screening lab tests: Negative for vitamin deficiencies, viral infections, inflammatory markers, glycemia, and serum electrophoresis, positive for ANA autoantibodies<br>○ No family history of polyneuropathy<br>**2. Additional Examinations**<br>**A. Laboratory Tests**<br>1. **Anti-Ro/SSA and Anti-La/SSB Antibodies**<br>○ **Reasoning:** The presence of ANA autoantibodies suggests a possible autoimmune etiology. Anti-Ro and Anti-La antibodies are associated with Sjögren's syndrome, which can cause sensory neuropathy.<br>2. **Anti-neuronal Antibodies (e.g., Anti-Hu, Anti-Yo, Anti-Ri)**<br>○ **Reasoning:** To investigate a possible paraneoplastic syndrome. These antibodies are linked with various cancers and associated neuropathies.<br>3. **Angiotensin-Converting Enzyme (ACE) Levels**<br>○ **Reasoning:** Elevated ACE levels can support the diagnosis of active sarcoidosis, which may have neurological manifestations.<br>4. **Serum Immunofixation Electrophoresis**<br>○ **Reasoning:** To detect monoclonal proteins which could indicate multiple myeloma or other plasma cell dyscrasias, conditions that can cause sensory polyneuropathy.<br>**B. Instrumental Tests**<br>5. **High-Resolution Chest CT Scan**<br>○ **Reasoning:** Sarcoidosis commonly affects the lungs. A chest CT can reveal hilar lymphadenopathy or pulmonary nodules suggestive of sarcoidosis, supporting the diagnosis if there are no current respiratory symptoms.<br>6. **Whole-body PET/CT Scan**<br>○ **Reasoning:** This can help identify occult malignancies that could be causing a paraneoplastic syndrome and provide a comprehensive overview of potential sarcoid involvement.

<table>
<tr><td></td><td></td><td></td><td>
7. Nerve Biopsy
○ Reasoning: To directly assess for granulomatous inflammation in the peripheral nerves, which would confirm sarcoidosis-related neuropathy.
8. Lumbar Puncture with CSF Analysis
○ Reasoning: To evaluate for inflammatory or infectious causes of neuropathy, including elevated protein levels that may suggest a paraneoplastic or autoimmune process.
C. Other Specialized Tests
9. Skin Biopsy for Intraepidermal Nerve Fiber Density
○ Reasoning: To evaluate small fiber neuropathy, which might not be detected on standard nerve conduction studies but can present with similar symptoms.
3. Determination of the Most Crucial Test
Nerve Biopsy
Reasoning:
● Direct Evidence: A nerve biopsy provides direct evidence of the underlying pathology within the nerve tissue. In this case, it can reveal granulomatous inflammation, which would confirm sarcoidosis-related neuropathy.
● Diagnostic Clarity: It can help distinguish between different types of neuropathy (e.g., vasculitic, amyloid, granulomatous).
● High Specificity: While other tests (like ACE levels and imaging) can suggest sarcoidosis, a nerve biopsy can definitively diagnose it in the context of neuropathy.
Conclusion
Nerve Biopsy is the most crucial test to pursue in this clinical scenario. It offers definitive diagnostic information that can confirm or rule out sarcoidosis-related neuropathy by directly examining the affected tissue. This will guide the subsequent management and treatment plan for the patient effectively.
</td></tr>
<tr><td>95</td><td>A 62-year-old male patient presents with a 5-month history of sensory-motor, symmetrical, length dependent polyneuropathy with subacute onset. The patient presents with distal paresthesia and neuropathic pain in the lower limbs, associated with weakness of toe dorsiflexion. Clinical examination also reveals the presence of orthostatic hypotension. There are no sensory ataxia, tremor, foot deformities or cranial nerve involvement. Past medical history is positive for diabetes since the age of 50, and plasmacytoma, for which the patient has undergone chemotherapy. Symptoms started during treatment with chemotherapy. Nerve conduction studies show a mixed sensory-motor, symmetric, axonal polyneuropathy. Screening lab tests including vitamin B12, folate, vitamin B6, vitamin E, HBV, HCV, ANA, ANCA, ENA, CRP, ESR, copper, cryoglobulins and serum electrophoresis are all negative, with evidence of hyperglycaemia and elevated HbA1c. There is no family history of polyneuropathy.</td><td>Chemotherapy-induced neuropathy</td><td>
Step-by-Step Analysis and Additional Examinations:
1. Detailed Clinical Examination:
○ Reasoning: To confirm the presence of sensory, motor, and autonomic involvement and to assess the progression of neuropathy.
○ Specifics: A thorough neurological examination including reflex testing, muscle strength assessment, detailed sensory testing, and autonomic function tests (e.g., tilt-table test for orthostatic hypotension).
2. Electromyography (EMG) and Repeated Nerve Conduction Studies (NCS):
○ Reasoning: To evaluate the type and extent of neuropathy, to differentiate between axonal and demyelinating neuropathies, and to track disease progression or response to treatment.
○ Specifics: EMG to assess muscle electrical activity, and NCS to measure nerve signal conduction velocities and amplitudes, focusing on any changes since the initial study.
3. Serum Protein Electrophoresis (SPEP) and Immunofixation Electrophoresis (IFE):
○ Reasoning: To detect monoclonal proteins that might indicate an underlying plasma cell disorder such as amyloidosis or multiple myeloma.
○ Specifics: SPEP to screen for monoclonal gammopathies, and IFE for a more sensitive and specific identification of monoclonal proteins.
4. Urine Protein Electrophoresis (UPEP) and Immunofixation Electrophoresis:
○ Reasoning: To detect Bence Jones proteins, which are light chains indicative of plasma cell dyscrasias such as amyloidosis.
○ Specifics: UPEP to screen for abnormal proteins in the urine, and immunofixation to identify specific types of light chains.
5. Skin Biopsy for Intraepidermal Nerve Fiber Density (IENFD):
○ Reasoning: To assess small fiber neuropathy, which might be present even if large fibers appear normal in NCS.
○ Specifics: A small skin biopsy to evaluate nerve fiber density and morphology under the microscope.
6. Quantitative Sensory Testing (QST):
○ Reasoning: To quantify sensory deficits and pain perception, which can help differentiate between types of neuropathies.
○ Specifics: Tests to measure thresholds for vibration, thermal, and pain stimuli.
7. Autonomic Function Tests:
○ Reasoning: To evaluate the extent of autonomic involvement, particularly important given the patient's orthostatic hypotension.
○ Specifics: Tests such as heart rate variability, sweat testing, and tilt-table testing.
8. Blood Tests:
○ Reasoning: To rule out or confirm other potential causes of neuropathy.
○ Specifics:
■ Serum Free Light Chains: To check for evidence of light chain deposition disease.
■ Heavy Metal Screening: To rule out toxic causes of neuropathy (e.g., lead, arsenic).
■ Hemoglobin A1c: To confirm and monitor diabetes control.
9. Lumbar Puncture with Cerebrospinal Fluid (CSF) Analysis:
○ Reasoning: To rule out inflammatory or infectious causes of neuropathy.
○ Specifics: CSF analysis for cell count, protein, glucose, and oligoclonal bands.
Most Crucial Test:
Serum Protein Electrophoresis (SPEP) and Immunofixation Electrophoresis (IFE): This test is crucial as it directly assesses for the presence of monoclonal proteins that could indicate a paraneoplastic process or amyloidosis, both of which are associated with the patient's history of plasmacytoma. Given the context and the need to rule out secondary causes of neuropathy related to the patient's cancer history, SPEP and IFE are essential to either confirm or exclude these serious underlying conditions.
</td></tr>
<tr><td>96</td><td>An 86-year-old male patient presents with a 1-year history of sensory-motor, symmetrical, non-length dependent polyneuropathy with subacute onset and progressive course. The patient presents with distal paresthesia in the upper limbs and sensory ataxia with weakness of ankle dorsiflexion. Clinical examination reveals mild hand tremor. There are no neuropathic pain, foot deformities, cranial nerve or autonomic involvement. Past medical history shows IgG kappa</td><td>CIDP</td><td>
Step-by-Step Analysis and Additional Examinations
Step 1: Review Initial Data
● Patient Information: 86-year-old male with 1-year history of sensory-motor, symmetrical, non-length dependent polyneuropathy.
● Clinical Findings: Distal paresthesia in upper limbs, sensory ataxia, ankle dorsiflexion weakness, mild hand tremor.
● Past Medical History: IgG kappa monoclonal gammopathy, basal cell skin cancer.
● Nerve Conduction Studies: Demyelinating, sensory-motor polyneuropathy.
● Lab Tests: Negative for multiple metabolic and infectious causes, positive for chronic HBV infection and IgG kappa paraprotein.
● Family History: No polyneuropathy.
Step 2: Identify Potential Differential Diagnoses
● Monoclonal Gammopathy of Undetermined Significance (MGUS)-related Polyneuropathy.
● Chronic Inflammatory Demyelinating Polyneuropathy (CIDP).
● Paraneoplastic Neuropathy.
</td></tr>
</table>

	monoclonal gammopathy and basal cell skin cancer. Nerve conduction studies show a demyelinating, sensory-motor polyneuropathy. Screening lab tests including vitamin B12, folate, vitamin B6, vitamin E, HCV, ANA, ANCA, ENA, CRP, ESR, glycemia, HbA1c, copper and cryoglobulins are all negative, with evidence of chronic HBV infection and positivity for IgG kappa paraprotein. There is no family history of polyneuropathy.		**Step 3: List Additional Examinations**<br>1. **Serum Immunofixation Electrophoresis (IFE) and Free Light Chain (FLC) Assay**:<br>○ **Reasoning**: To further characterize the monoclonal protein and assess for light chain involvement which can be seen in conditions like MGUS and multiple myeloma. This helps in confirming the presence and type of monoclonal gammopathy.<br>2. **Cerebrospinal Fluid (CSF) Analysis**:<br>○ **Reasoning**: To look for albuminocytologic dissociation (elevated protein without pleocytosis) which is indicative of CIDP. Also, to rule out infectious or malignant causes.<br>3. **MRI of the Spine with Contrast**:<br>○ **Reasoning**: To assess for nerve root enhancement which can be seen in CIDP and to exclude other structural causes of polyneuropathy.<br>4. **Anti-MAG (Myelin-Associated Glycoprotein) Antibodies**:<br>○ **Reasoning**: Anti-MAG neuropathy is associated with IgM paraproteins, but in some cases, it can be associated with IgG. Testing for these antibodies can help in differentiating the type of paraprotein-related neuropathy.<br>5. **Paraneoplastic Panel**:<br>○ **Reasoning**: To check for antibodies associated with paraneoplastic syndromes which can cause polyneuropathy, especially in the context of a history of cancer.<br>6. **Repeat Electrophysiological Studies**:<br>○ **Reasoning**: To monitor the progression and pattern of the neuropathy, which can help in differentiating between CIDP and MGUS-related neuropathy.<br>7. **Quantitative Sensory Testing (QST)**:<br>○ **Reasoning**: To objectively measure sensory deficits and differentiate between small and large fiber neuropathy.<br>8. **Skin Biopsy with Intraepidermal Nerve Fiber Density (IENFD) Measurement**:<br>○ **Reasoning**: To evaluate for small fiber neuropathy which might not be detected on standard nerve conduction studies.<br>9. **Hepatitis B Viral Load and Liver Function Tests**:<br>○ **Reasoning**: To assess the extent and activity of HBV infection which might have systemic implications, including neuropathy.<br>**Step 4: Determine the Most Crucial Test**<br>● **Most Crucial Test: Serum Immunofixation Electrophoresis (IFE) and Free Light Chain (FLC) Assay**<br>○ **Reasoning**: Confirming the type and presence of the monoclonal protein is crucial for diagnosing MGUS-related neuropathy. This test provides detailed information on the paraprotein, which is essential for differentiating between MGUS, multiple myeloma, and other paraproteinemias. Accurate characterization of the monoclonal protein is key in guiding further management and establishing the primary cause of the polyneuropathy.<br>By conducting these additional examinations, particularly focusing on the characterization of the monoclonal gammopathy, we can better narrow down the differential diagnoses and tailor the treatment approach for the patient.
97	A 57-year-old male patient presents with a 1-year history of sensory-motor, asymmetric, non-length dependent polyneuropathy with subacute onset and progressive course. The patient presents with weakness and paresthesia in the proximal and distal areas of the upper limbs in an asymmetric distribution. Clinical examination excludes the presence of ataxia, tremor, neuropathic pain, foot deformities, cranial nerve or autonomic involvement. Past medical history is silent. Nerve conduction studies show a demyelinating, sensory-motor multineuropathy. Screening lab tests reveal the presence of folate deficiency. Remaining exams including vitamin B12, vitamin B6, vitamin E, HBV, HCV, ANA, ANCA, ENA, CRP, ESR, glycemia, HbA1c, copper, cryoglobulins and serum electrophoresis are all negative. There is no family history of polyneuropathy.	CIDP	**Step-by-Step Analysis of Comprehensive Data**<br>1. **Review of Presenting Symptoms and Clinical Findings**<br>○ **Symptoms**: Asymmetric sensory-motor polyneuropathy, weakness, and paresthesia in upper limbs.<br>○ **Clinical Findings**: Demyelinating sensory-motor multineuropathy on nerve conduction studies, no ataxia, tremor, neuropathic pain, foot deformities, cranial nerve, or autonomic involvement.<br>○ **Laboratory Results**: Folate deficiency, negative for other common neuropathy causes.<br>○ **Past Medical History**: Silent.<br>○ **Family History**: No family history of polyneuropathy.<br>2. **Differential Diagnoses**: CIDP, MMN, Paraneoplastic Neuropathy.<br>**Necessary Additional Examinations**<br>**1. Lumbar Puncture for Cerebrospinal Fluid (CSF) Analysis**<br>● **Reasoning**: CSF analysis can help identify elevated protein levels without pleocytosis, which is characteristic of CIDP.<br>● **Expected Findings**: Elevated CSF protein (albuminocytologic dissociation) supports CIDP. Normal CSF findings would be less suggestive of CIDP.<br>**2. Magnetic Resonance Imaging (MRI) of the Brachial Plexus and Spinal Cord**<br>● **Reasoning**: MRI can detect nerve root hypertrophy or gadolinium enhancement, which are indicative of CIDP. It also helps rule out structural lesions that could cause asymmetric neuropathy.<br>● **Expected Findings**: Hypertrophy and/or enhancement of the nerve roots would support CIDP. Absence of these findings might suggest other etiologies.<br>**3. Anti-GM1 Antibody Testing**<br>● **Reasoning**: Anti-GM1 antibodies are often elevated in patients with MMN. Testing for these antibodies can help differentiate between CIDP and MMN.<br>● **Expected Findings**: Positive anti-GM1 antibodies would support a diagnosis of MMN.<br>**4. Paraneoplastic Panel**<br>● **Reasoning**: Paraneoplastic syndromes can cause neuropathies, and detecting specific onconeural antibodies can aid in diagnosing paraneoplastic neuropathy.<br>● **Expected Findings**: Positive paraneoplastic antibodies would suggest an underlying malignancy as the cause of neuropathy.<br>**5. Positron Emission Tomography (PET) Scan or Computed Tomography (CT) Scan**<br>● **Reasoning**: To screen for occult malignancies that might be causing a paraneoplastic syndrome, especially given the patient's age.<br>● **Expected Findings**: Detection of an occult malignancy would support the diagnosis of paraneoplastic neuropathy.<br>**Conclusion: Most Crucial Test**<br>The **most crucial test** from the list is the **Lumbar Puncture for Cerebrospinal Fluid (CSF) Analysis**.<br>● **Reasoning**:<br>○ The CSF analysis is critical for distinguishing CIDP from other neuropathies because it directly assesses the typical pathophysiological feature of CIDP (elevated protein without pleocytosis).<br>○ Given that CIDP is a leading differential diagnosis based on the clinical presentation and nerve conduction study findings, confirming or ruling out CIDP will significantly impact management and further diagnostic steps.<br>○ CSF analysis results will guide the necessity for other tests (e.g., if CIDP is confirmed, anti-GM1 antibody testing and extensive malignancy screening might be deprioritized).<br>Thus, obtaining CSF analysis through lumbar puncture is a pivotal step in narrowing down the diagnosis and determining the appropriate treatment pathway.
98	A 74-year-old male patient presents with a 1-month of sensory-motor, multifocal neuropathy with subacute onset and stepwise progression. The patient presents with paresthesia, neuropathic pain, and	Vasculitic neuropathy	**Additional Examinations and Reasoning**<br>**Step-by-Step Approach to Additional Examinations**<br>1. **Detailed Laboratory Tests:**<br>○ **Complete Blood Count (CBC) with Differential:**<br>■ **Reasoning:** To identify any signs of infection, anemia, or hematologic malignancies that could contribute to neuropathy.

	weakness with a multifocal distribution in the four limbs. He also reports arthralgia and headache, which began in the last two weeks. Clinical examination excludes the presence of sensory ataxia, tremor, foot deformities, cranial nerve or autonomic involvement. Past medical history is positive for arterial hypertension, Hashimoto's thyroiditis, chronic HBV infection, and type 2 diabetes mellitus. Nerve conduction studies show a multifocal, axonal, sensory-motor neuropathy. Screening lab tests reveal elevated glycemia and HbA1c leves, vitamin B12 deficiency, and raised ESR and CRP. Remaining exams including folate, vitamin B6, vitamin E, HCV, ANA, ANCA, ENA, copper, cryoglobulins, and serum electrophoresis are normal. There is no family history of polyneuropathy.		○ **Serum Immunofixation Electrophoresis:**<br>■ **Reasoning:** To check for monoclonal gammopathies (e.g., multiple myeloma, MGUS) that can cause neuropathy.<br>○ **Anti-Hepatitis B Core Antigen (Anti-HBc) and Hepatitis B Virus DNA (HBV DNA):**<br>■ **Reasoning:** To assess the activity of chronic HBV infection, which can contribute to vasculitis or neuropathy.<br>○ **Rheumatoid Factor (RF) and Anti-Cyclic Citrullinated Peptide (Anti-CCP):**<br>■ **Reasoning:** To evaluate for rheumatoid arthritis, which can be associated with vasculitis.<br>○ **Complement Levels (C3, C4, CH50):**<br>■ **Reasoning:** To assess for complement system activation, which can be involved in vasculitic processes.<br>○ **Antiphospholipid Antibodies (APA):**<br>■ **Reasoning:** To evaluate for antiphospholipid syndrome, which can cause vasculitis and neuropathy.<br>○ **Serum ACE and Chest X-ray:**<br>■ **Reasoning:** To rule out sarcoidosis, which can present with neuropathy and systemic inflammatory signs.<br>○ **Cerebrospinal Fluid (CSF) Analysis:**<br>■ **Reasoning:** To look for any signs of infection, inflammation, or malignancy affecting the nervous system.<br>■ **Specific tests in CSF:** Protein levels, cell count, glucose, oligoclonal bands.<br>2. **Instrumental Examinations:**<br>○ **Nerve Biopsy:**<br>■ **Reasoning:** To obtain a definitive diagnosis of vasculitis by examining the structure of the nerve and the presence of inflammatory infiltrates, vessel wall destruction, and other characteristic features.<br>○ **Skin Biopsy:**<br>■ **Reasoning:** To look for evidence of small fiber neuropathy and potential vasculitic changes in small vessels.<br>○ **MRI of the Brain and Spinal Cord:**<br>■ **Reasoning:** To evaluate for central nervous system involvement and rule out other potential causes of neuropathy such as multiple sclerosis or structural abnormalities.<br>○ **Ultrasound/Doppler of Peripheral Nerves:**<br>■ **Reasoning:** To assess for any structural changes in the peripheral nerves that might suggest entrapment or vasculitic processes.<br>**Most Crucial Test**<br>**Nerve Biopsy:**<br>● **Reasoning:** Given the high suspicion of vasculitic neuropathy, a nerve biopsy is the most crucial test. It can provide direct evidence of vasculitis by demonstrating characteristic features such as inflammation, vessel wall destruction, and fibrinoid necrosis within the nerve. This test can confirm the diagnosis and guide specific treatment strategies for vasculitic neuropathy.<br>**Conclusion**<br>To narrow down the differential diagnoses for the patient with multifocal sensory-motor neuropathy, additional laboratory tests and instrumental examinations should be conducted. Among these, a **nerve biopsy** is the most crucial test due to its potential to provide definitive evidence of vasculitic neuropathy, thereby confirming the diagnosis and guiding appropriate treatment.
99	A 71-year-old male patient presents with a 2-year history of sensory-motor, symmetrical, length dependent polyneuropathy with insidious onset and fast disease progression. The patient presents with moderate distal weakness and hypoesthesia in the four limbs, sensory ataxia and autonomic involvement with no foot deformities or neuropathic pain. Past medical history is positive for cardiac insufficiency. Nerve conduction studies show an axonal, sensory-motor polyneuropathy with bilateral carpal tunnel syndrome. Screening lab tests including vitamin B12, folate, vitamin B6, vitamin E, HBV, HCV, ANA, ANCA, ENA, glycaemia, HbA1c, CRP, ESR, copper, cryoglobulins, and serum electrophoresis are all negative. His brother is wheelchair bound.	TTR-Amyloidosis	**Step-by-Step Analysis and Additional Examinations:**<br>**1. Genetic Testing for Transthyretin (TTR) Mutation:**<br>● **Reasoning:** Given the strong suspicion of hereditary transthyretin amyloidosis (hATTR) due to the combination of progressive sensory-motor polyneuropathy, autonomic involvement, cardiac insufficiency, and family history, genetic testing for TTR mutations is critical. Identifying a mutation in the TTR gene would confirm the diagnosis of hATTR.<br>● **Specifics:** TTR gene sequencing.<br>**2. Nerve Biopsy:**<br>● **Reasoning:** A nerve biopsy can provide direct evidence of amyloid deposits in the peripheral nerves. Congo red staining of the biopsy specimen can detect amyloid deposits, and immunohistochemistry can help differentiate between AL and ATTR amyloidosis.<br>● **Specifics:** Sural nerve biopsy with Congo red staining and immunohistochemistry.<br>**3. Echocardiogram and Cardiac MRI:**<br>● **Reasoning:** Given the patient's cardiac insufficiency, it is essential to assess for amyloid infiltration in the heart. An echocardiogram can show thickened ventricular walls with a "sparkling" appearance, and a cardiac MRI can provide more detailed imaging to assess the extent of amyloid deposits.<br>● **Specifics:** Transthoracic echocardiogram and cardiac MRI.<br>**4. Fat Pad Biopsy:**<br>● **Reasoning:** A less invasive procedure compared to nerve biopsy, a fat pad biopsy can also detect amyloid deposits. This test can be useful if a nerve biopsy is not feasible.<br>● **Specifics:** Abdominal fat pad biopsy with Congo red staining.<br>**5. Quantitative Sensory Testing (QST):**<br>● **Reasoning:** QST can provide objective measures of sensory deficits, helping to quantify the degree of sensory involvement and monitor disease progression or response to treatment.<br>● **Specifics:** Quantitative sensory testing for thermal and vibration thresholds.<br>**6. Autonomic Testing:**<br>● **Reasoning:** Since the patient has autonomic involvement, autonomic function tests can help assess the extent and severity of autonomic dysfunction. Tests may include heart rate variability, tilt table test, and sudomotor function tests.<br>● **Specifics:** Heart rate variability, tilt table test, sudomotor function tests.<br>**7. Serum and Urine Immunofixation Electrophoresis and Serum Free Light Chains:**<br>● **Reasoning:** Although initial serum electrophoresis was negative, immunofixation electrophoresis and serum free light chains testing are more sensitive for detecting monoclonal proteins that can indicate AL amyloidosis.<br>● **Specifics:** Serum and urine immunofixation electrophoresis, serum free light chains.<br>**Conclusion: Most Crucial Test**<br>**Genetic Testing for Transthyretin (TTR) Mutation:**<br>● **Reasoning:** Given the high clinical suspicion of hATTR amyloidosis based on the patient's symptoms and family history, genetic testing for TTR mutations is the most crucial test. It can definitively diagnose hATTR, guiding appropriate treatment and management. Identifying a TTR mutation directly confirms the hereditary form of amyloidosis and is critical for both the patient and potentially affected family members.
100	A 76-year-old female patient presents with a 6-year history of chronic, pure sensory,	Small fibre neuropathy	**Step-by-Step Analysis of Additional Examinations:**

<table>
<tr>
<td></td>
<td>symmetrical, length dependent polyneuropathy with insidious onset and slow progressive course. The main symptom is severe burning and tingling pain in the feet. Clinical examination reveals hyperalgesia upon pinprick testing, along with redness and warmth in the feet. There are no weakness or tactile hypoesthesia, sensory ataxia, tremor, foot deformities, or cranial nerve involvement. Past medical history is silent. Nerve conduction studies are normal with no signs of polyneuropathy. Screening lab tests including vitamin B12, folate, vitamin B6, vitamin E, HBV, HCV, ANA, ANCA, ENA, CRP, ESR, glycemia, HbA1c, copper, cryoglobulins, and serum electrophoresis are negative. There is family history of polyneuropathy.</td>
<td></td>
<td>Given the clinical scenario, the patient's presentation strongly suggests a small fiber neuropathy (SFN). To confirm this and further narrow down the differential diagnoses, a combination of laboratory and instrumental tests should be conducted. Here is a detailed reasoning for each proposed test:
Additional Examinations:

Skin Biopsy:

Reasoning: Skin biopsy is a gold standard for diagnosing small fiber neuropathy. It involves taking a small sample of skin to assess the density of small nerve fibers. Reduced intraepidermal nerve fiber density is diagnostic of SFN.
Utility: Confirms the diagnosis of SFN and can help differentiate it from large fiber neuropathies.

Quantitative Sensory Testing (QST):

Reasoning: QST evaluates the functional status of small nerve fibers by measuring responses to thermal and mechanical stimuli. It is useful in assessing the extent and functional impact of SFN.
Utility: Provides objective data on sensory function, supporting the clinical diagnosis.

Autonomic Testing (e.g., Tilt Table Test, Heart Rate Variability):

Reasoning: Autonomic dysfunction often accompanies SFN. Tests like the tilt table test and heart rate variability can assess autonomic involvement, which is common in small fiber neuropathy.
Utility: Helps determine the presence and extent of autonomic involvement, providing a more comprehensive understanding of the neuropathy.

Genetic Testing:

Reasoning: Given the family history of polyneuropathy, genetic testing for mutations known to cause hereditary sensory and autonomic neuropathies (e.g., SCN9A, SCN10A) should be considered.
Utility: Identifies hereditary causes of SFN and helps in genetic counseling and family planning.

Advanced Laboratory Tests:

Reasoning: Re-evaluation for less common causes of neuropathy can be helpful. This includes:

Anti-Hu antibodies: To rule out paraneoplastic syndromes.
Thyroid function tests (TFTs): Hypothyroidism can sometimes cause neuropathy.
Serum protein electrophoresis and immunofixation electrophoresis: To rule out monoclonal gammopathy, which can be associated with neuropathy.

Utility: Ensures no secondary causes of neuropathy are missed, providing a thorough differential workup.

Corneal Confocal Microscopy (CCM):

Reasoning: CCM is a non-invasive imaging technique that can assess small nerve fibers in the cornea. It correlates well with skin biopsy results in diagnosing SFN.
Utility: Offers a non-invasive alternative to skin biopsy with quick results, confirming small fiber involvement.

Most Crucial Test:
The skin biopsy is the most crucial test from the list. It directly measures intraepidermal nerve fiber density, which is a definitive diagnostic tool for small fiber neuropathy. This test would confirm the diagnosis of SFN and provide a clear path for further management and potential genetic testing if a hereditary cause is suspected.
Conclusion:
The comprehensive approach includes both laboratory and instrumental tests to confirm the diagnosis of SFN and to explore potential hereditary and secondary causes. Among these, the skin biopsy stands out as the most essential test for confirming the presence of small fiber neuropathy, thereby guiding further diagnostic and therapeutic steps.</td>
</tr>
</table>

<u>Supplementary Table 5.</u> Comparison of performance metrics across the first and last half of the presented cases

LEGEND: SD, Standard deviation

**Metrics** (mean ± SD)	**GPT-4o** (first 50 cases)	**GPT-4o** (last 50 cases)	**p-value**	**Specialists** (first 50 cases)	**Specialists** (last 50 cases)	**p-value**	**Non-Specialists** (first 50 cases)	**Non-Specialists** (last 50 cases)	**p-value**
**Accuracy**									
Leading diagnosis	0.700 ± (0.451)	0.610 ± (0.456)	0.323	0.788 ± (0.206)	0.776 ± (0.196)	0.766	0.640 ± (0.290)	0.618 ± (0.294)	0.705
Differentials	0.8200 ± (0.375)	0.820 ± (0.361)	1.000	0.894 ± (0.123)	0.910 ± (0.122)	0.516	0.789 ± (0.283)	0.762 ± (0.273)	0.623
**Confirmatory test**	0.630 ± (0.414)	0.730 ± (0.381)	0.212	0.694 ± (0.212)	0.717 ± (0.195)	0.566	0.540 ± (0.282)	0.520 ± (0.228)	0.703
